# Supplementary material for: Notos - a galaxy tool to analyze CpN observed expected ratios for inferring DNA methylation types
Source: BMC Bioinformatics. 2018 Mar 27;19:105. doi: 10.1186/s12859-018-2115-4 (PMC5870242; doi:10.1186/s12859-018-2115-4)
Supplement: Supplementary file 1 — CpG o/e ratios from dbEST analyzed by Notos: data preparation output - graphics. This file shows the figure produced by the data cleaning step. (PDF 1850 kb) [file 12859_2018_2115_MOESM1_ESM.pdf]

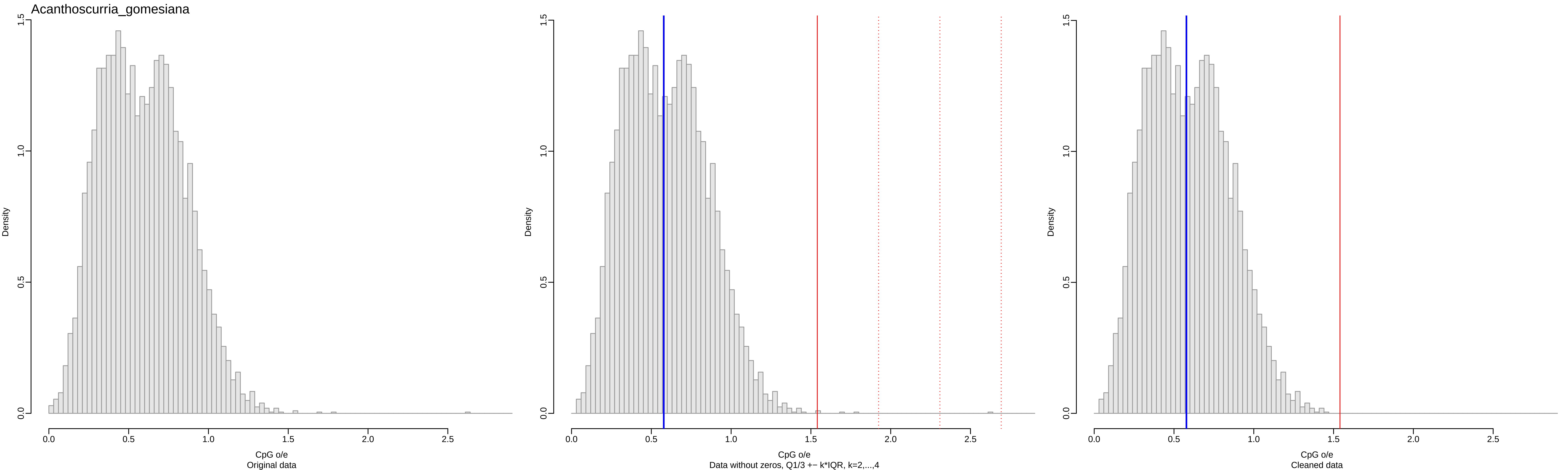

Acanthoscurria_gomesiana
Density
CpG o/e
Original data
0.0
0.5
1.0
1.5
2.0
2.5
Data without zeros, Q1/3 +− k*IQR, k=2,...,4
Cleaned data

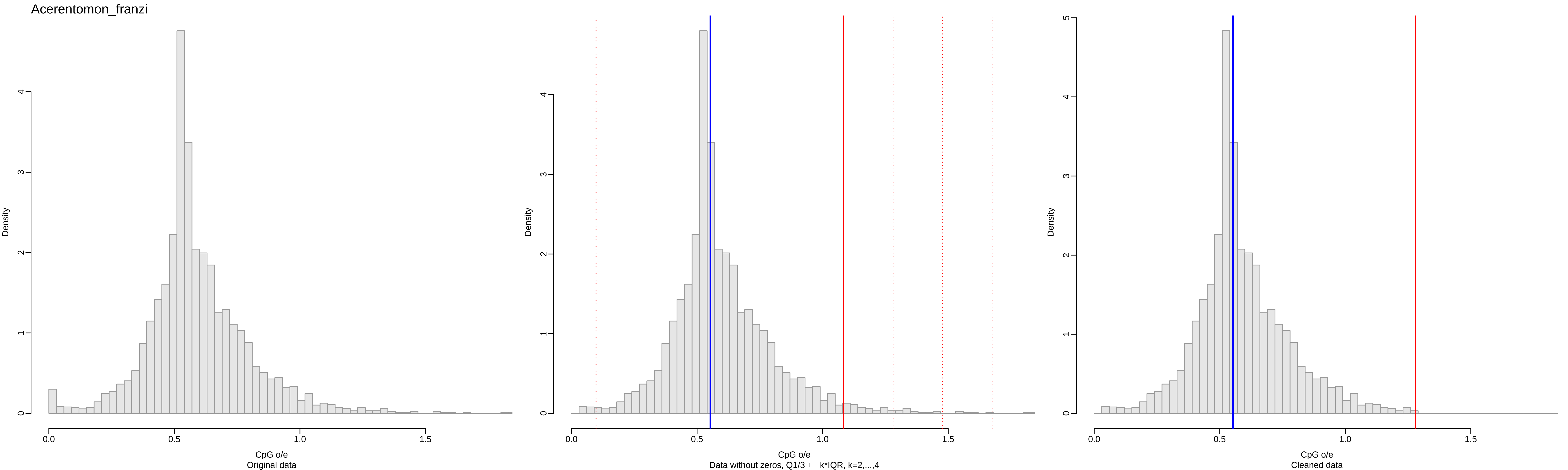
Acerentomon_franzi
Density
CpG o/e
Original data
Density
CpG o/e
Data without zeros, Q1/3 +− k*IQR, k=2,...,4
Density
CpG o/e
Cleaned data

Acetabularia_acetabulum

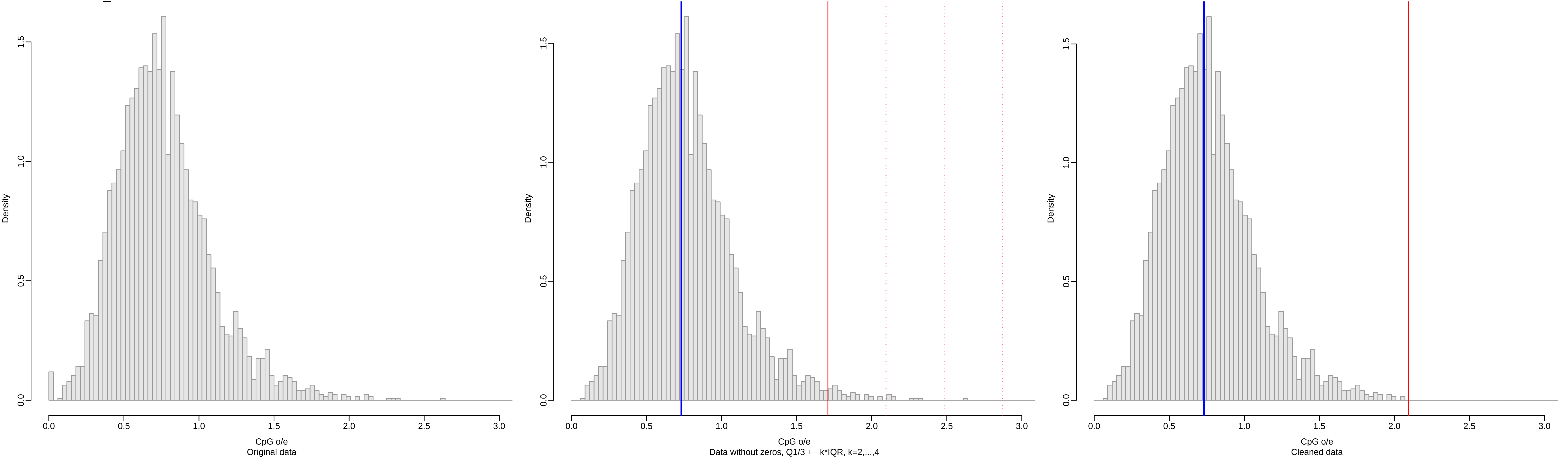

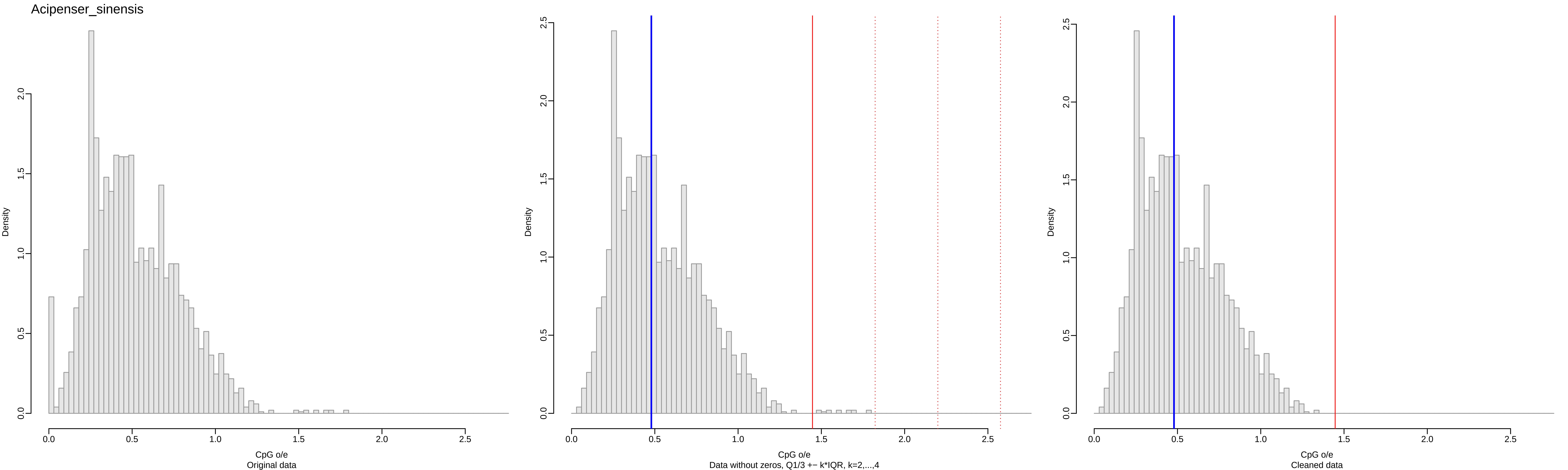

Acipenser_sinensis
Density
CpG o/e
Original data
Data without zeros, Q1/3 +− k*IQR, k=2,...,4
Cleaned data
0.0
0.5
1.0
1.5
2.0
2.5

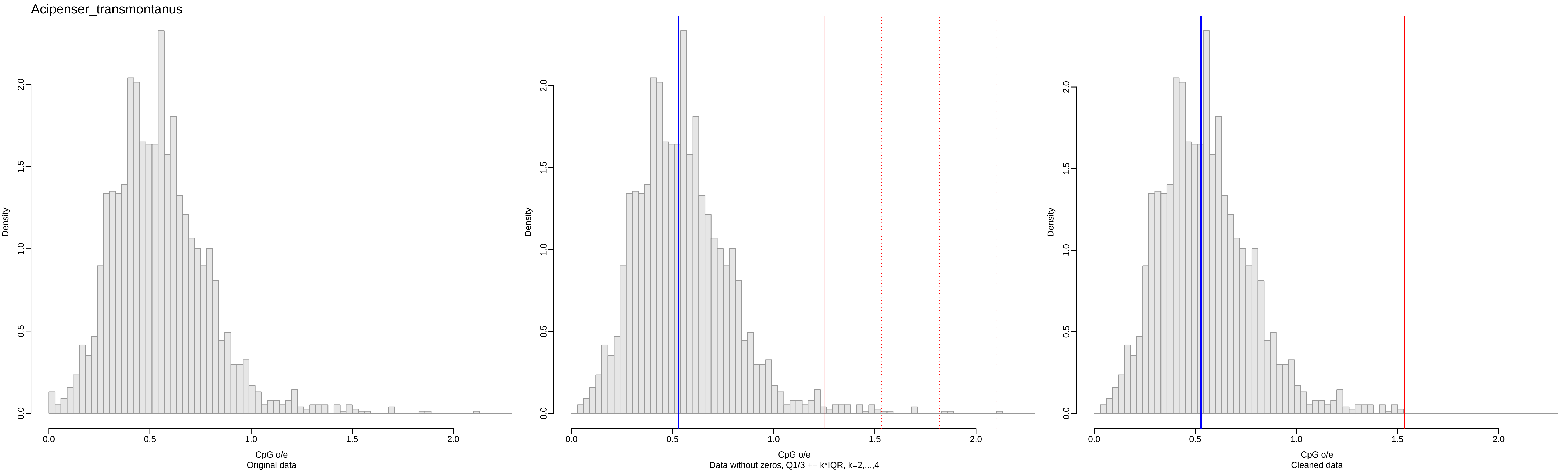
Acipenser_transmontanus
Density
CpG o/e
Original data
Data without zeros, Q1/3 +− k*IQR, k=2,...,4
Cleaned data
0.0
0.5
1.0
1.5
2.0

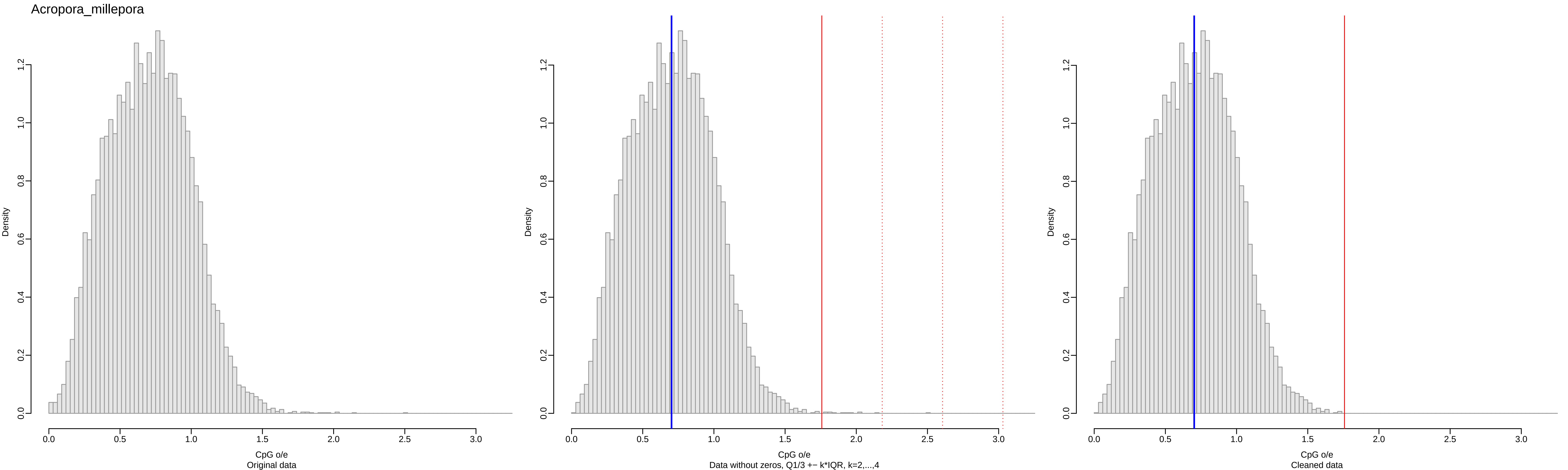

Acropora_millepora
Density
CpG o/e
Original data
Data without zeros, Q1/3 +− k*IQR, k=2,...,4
Cleaned data

Acropora_palmata

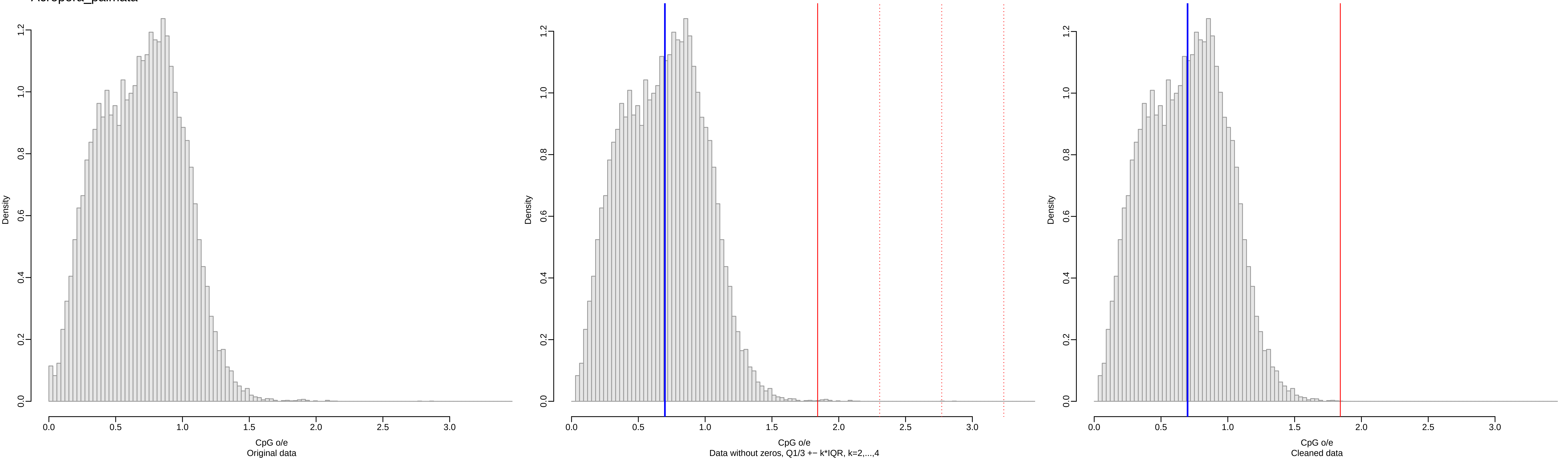

Actinidia_arguta

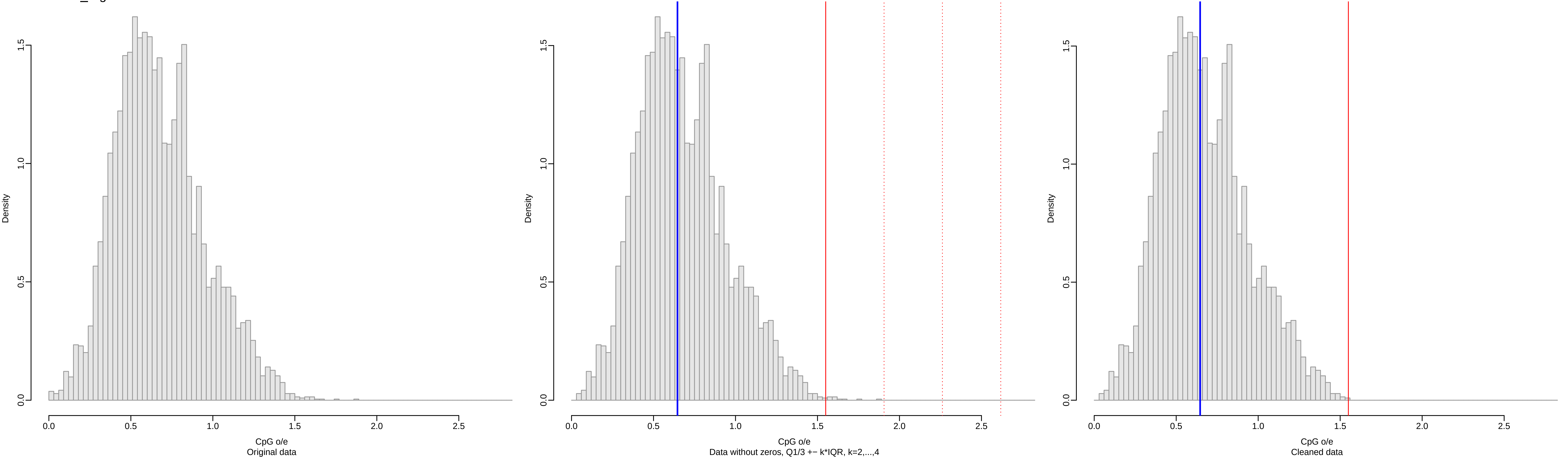

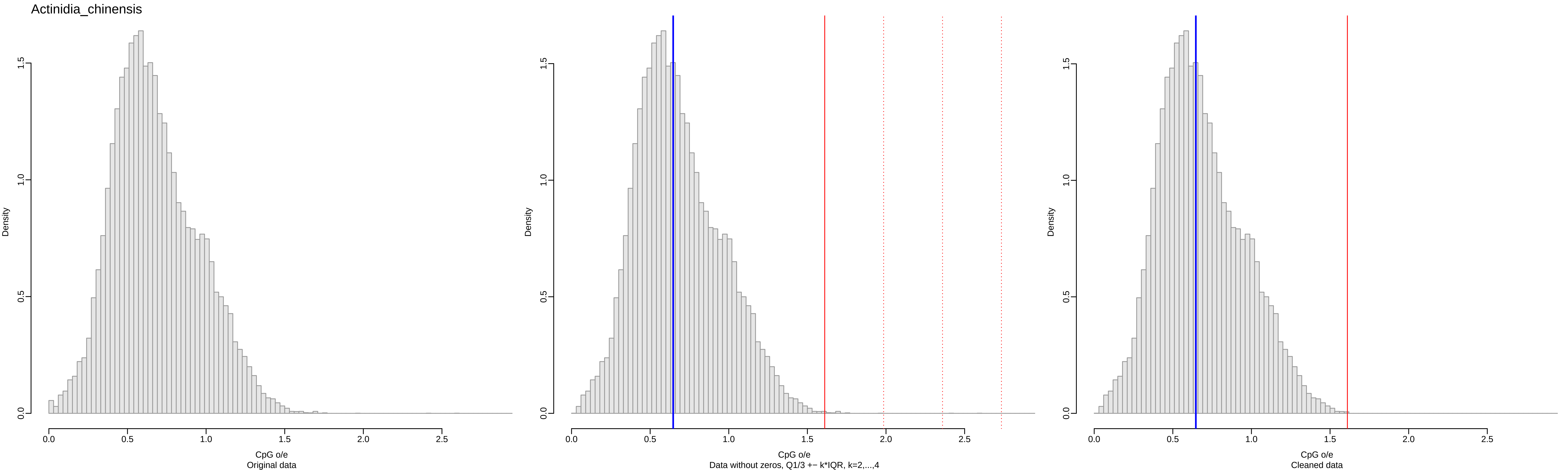
Actinidia_chinensis
Density
CpG o/e
Original data
CpG o/e
Data without zeros, Q1/3 +− k*IQR, k=2,...,4
CpG o/e
Cleaned data
0.0
0.5
1.0
1.5
2.0
2.5

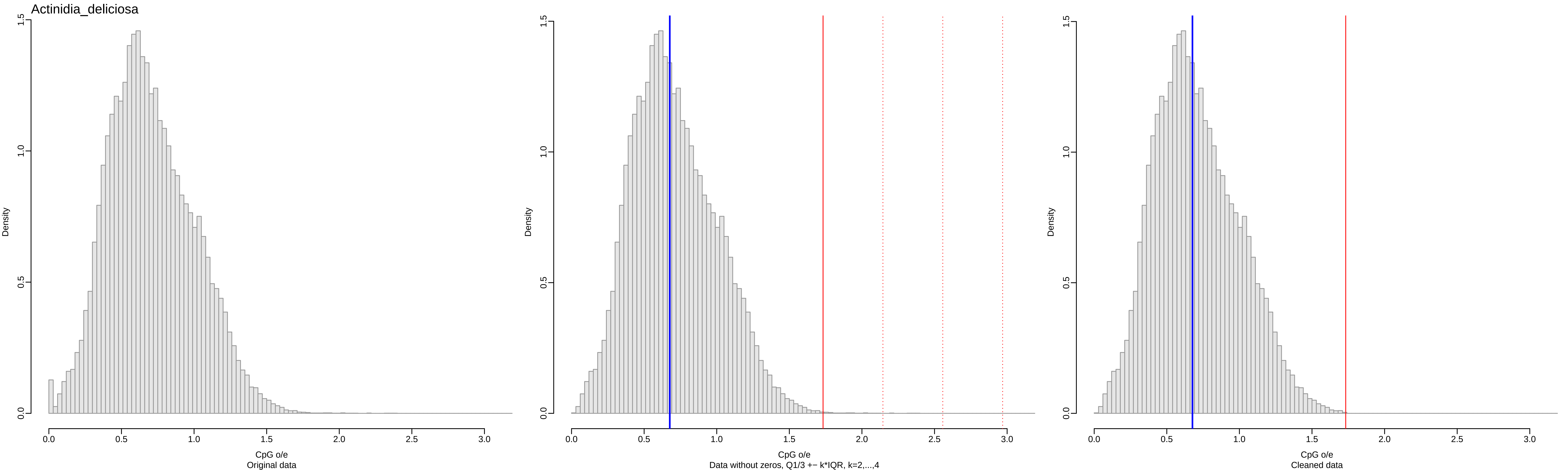
Actinidia_deliciosa
Density
CpG o/e
Original data
Data without zeros, Q1/3 +− k*IQR, k=2,...,4
Cleaned data

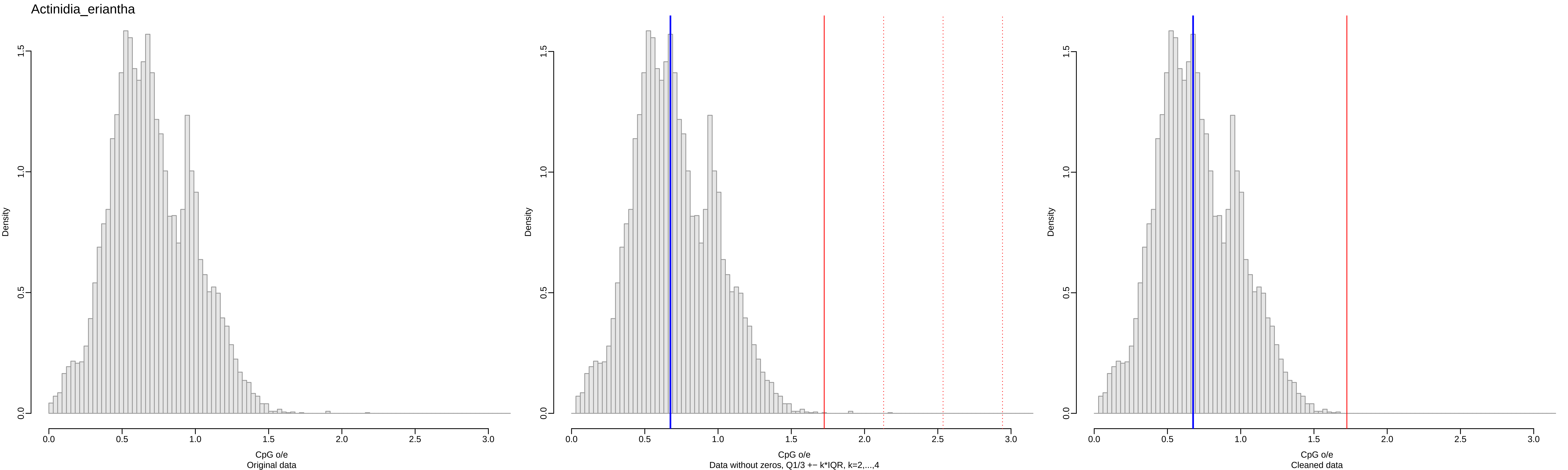

Actinidia_eriantha
Density
CpG o/e
Original data
Data without zeros, Q1/3 +− k*IQR, k=2,...,4
Cleaned data

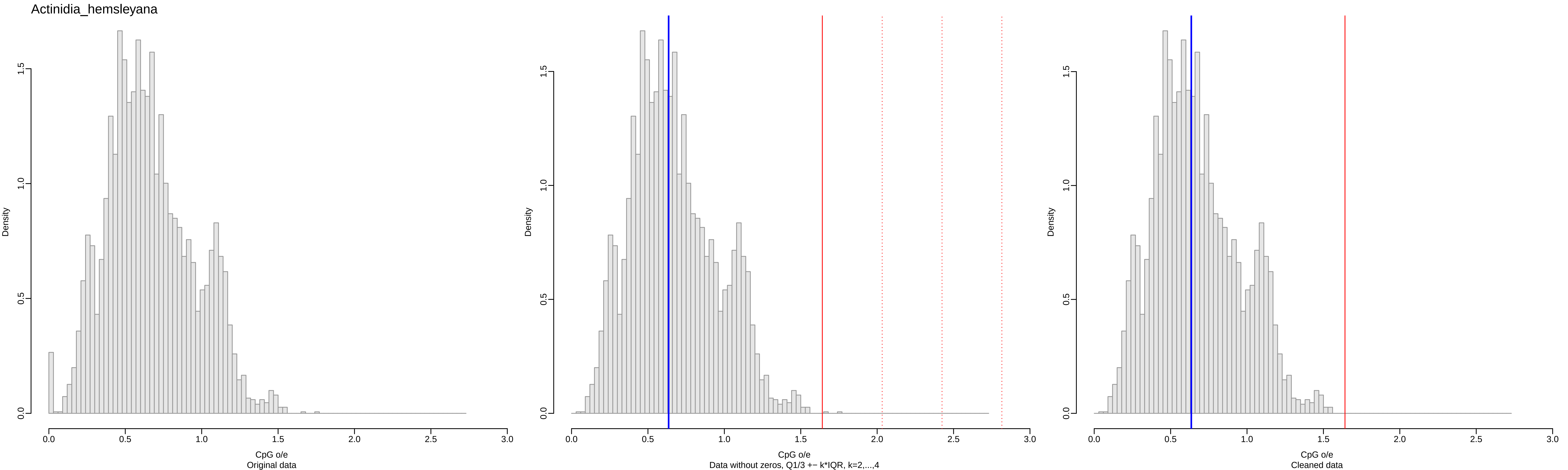

Actinidia_hemsleyana
Density
CpG o/e
Original data
Data without zeros, Q1/3 +− k*IQR, k=2,...,4
Cleaned data
0.0
0.5
1.0
1.5
2.0
2.5
3.0

Actinidia_polygama

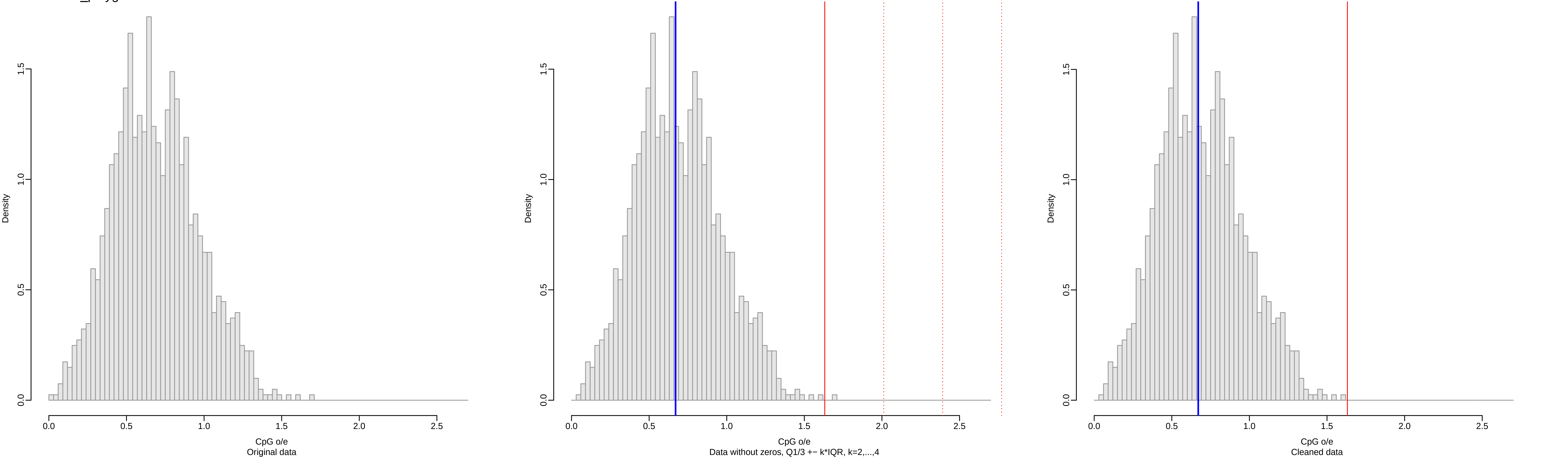

Actinidia_setosa

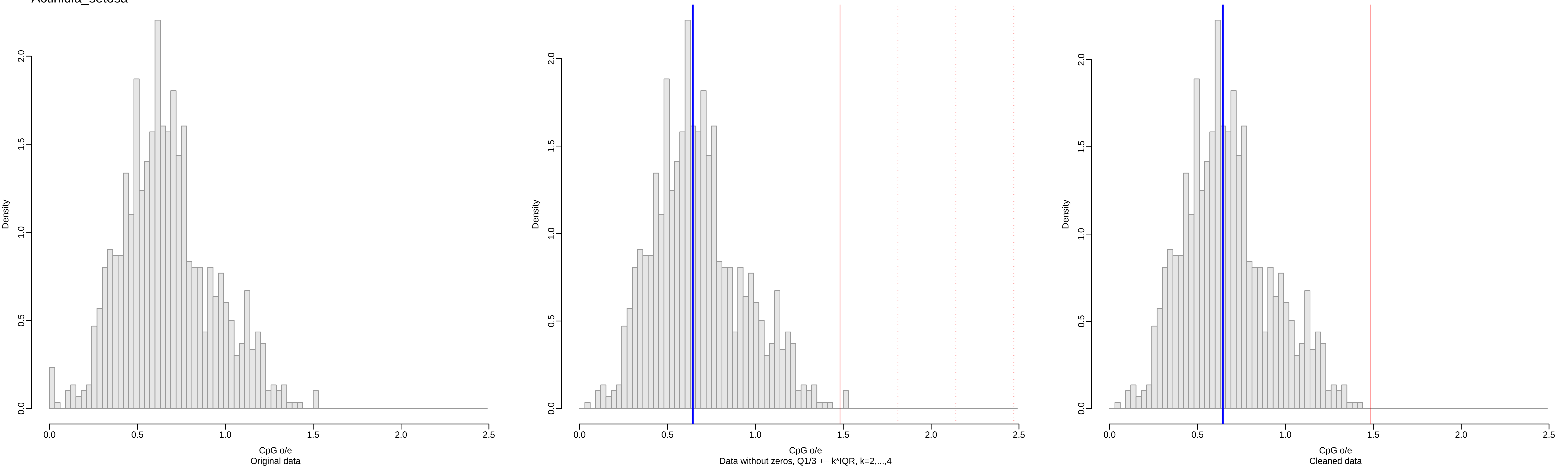

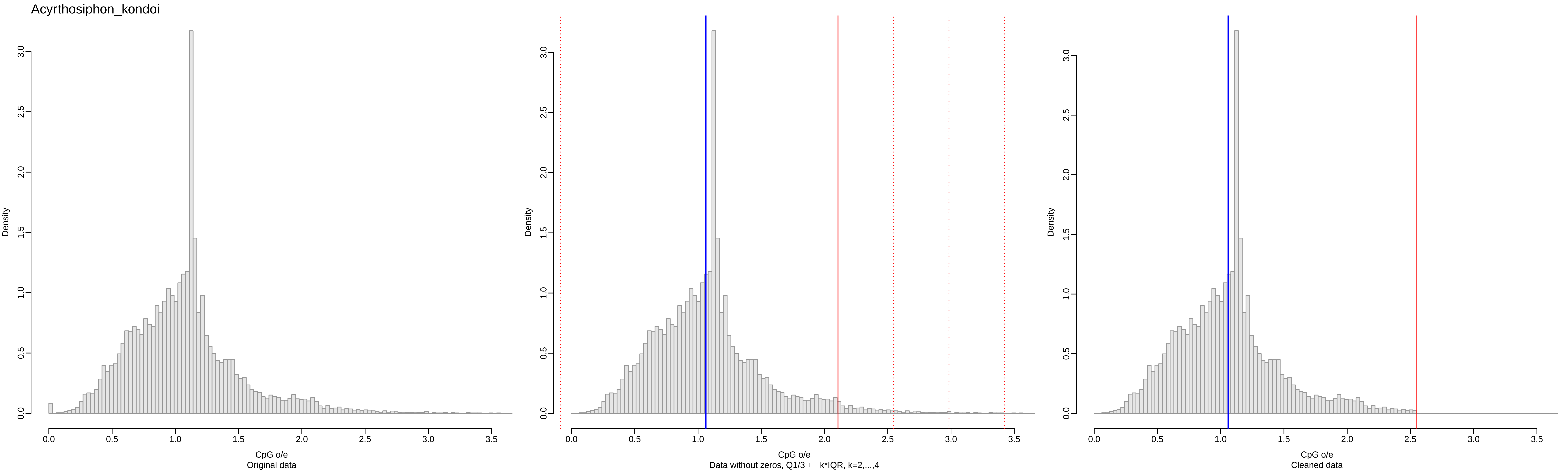

Acyrthosiphon_kondoi
Density
CpG o/e
Original data
Density
CpG o/e
Data without zeros, Q1/3 +− k*IQR, k=2,...,4
Density
CpG o/e
Cleaned data

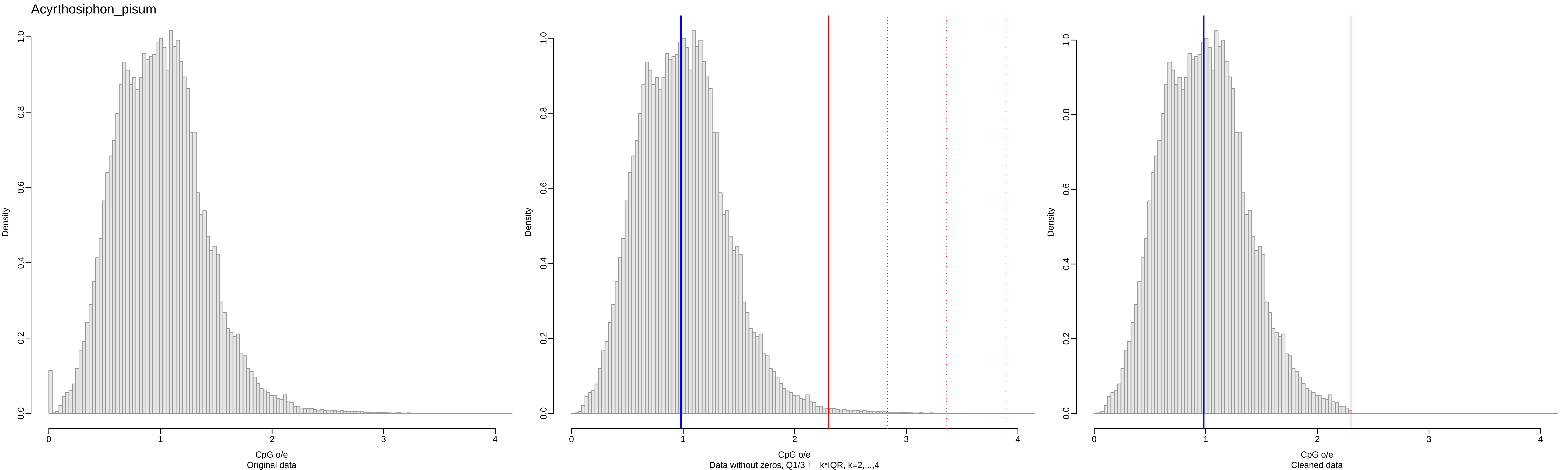

Acyrthosiphon_pisum
Density
CpG o/e
Original data
CpG o/e
Data without zeros, Q1/3 +− k*IQR, k=2,...,4
CpG o/e
Cleaned data

Adelphocoris_lineolatus

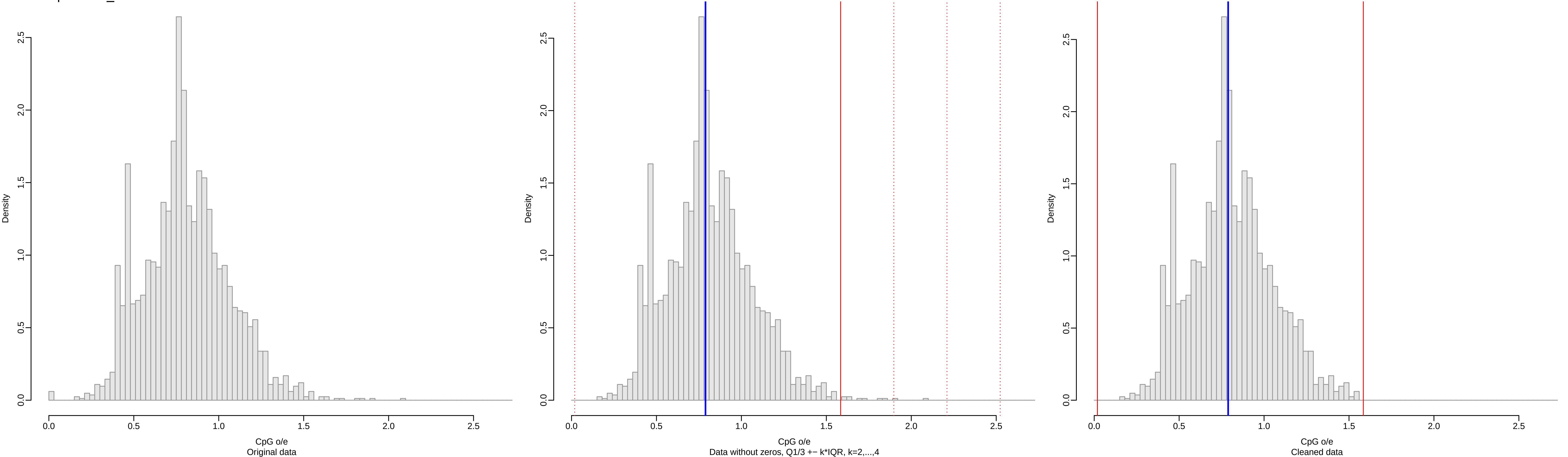

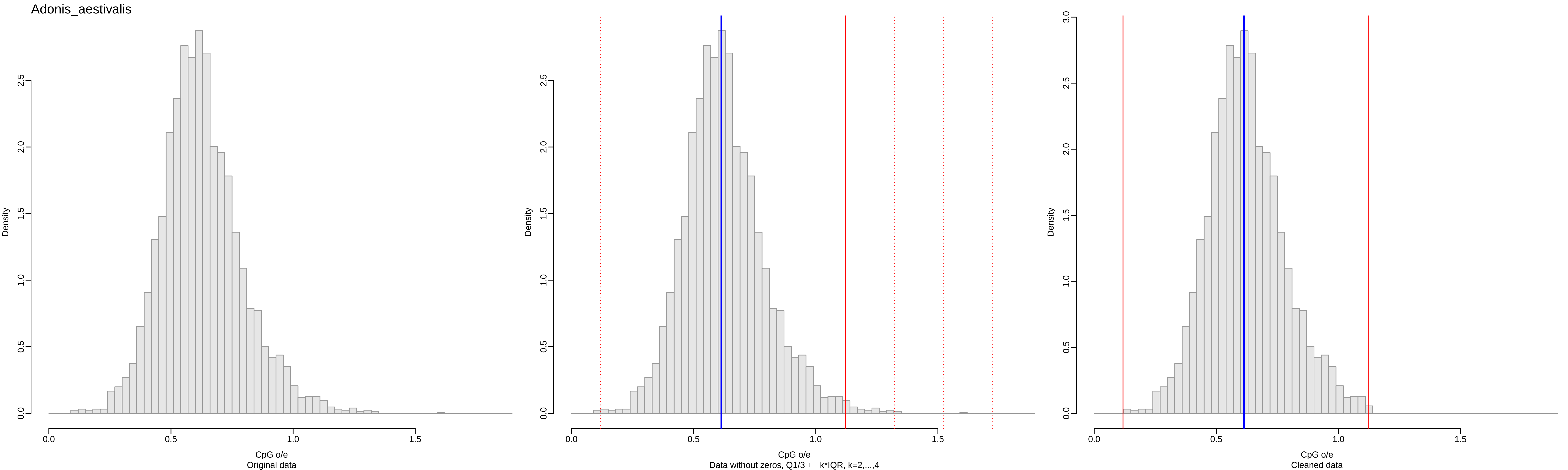

Adonis_aestivalis
Density
CpG o/e
Original data
CpG o/e
Data without zeros, Q1/3 +− k*IQR, k=2,...,4
CpG o/e
Cleaned data

Aegilops_speltoides

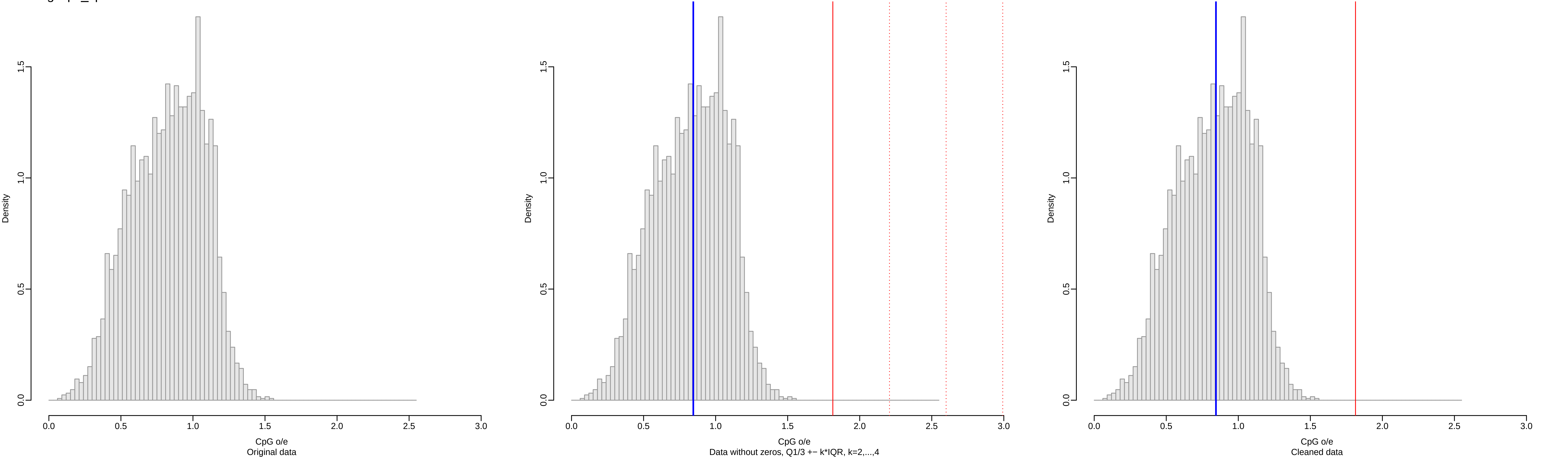

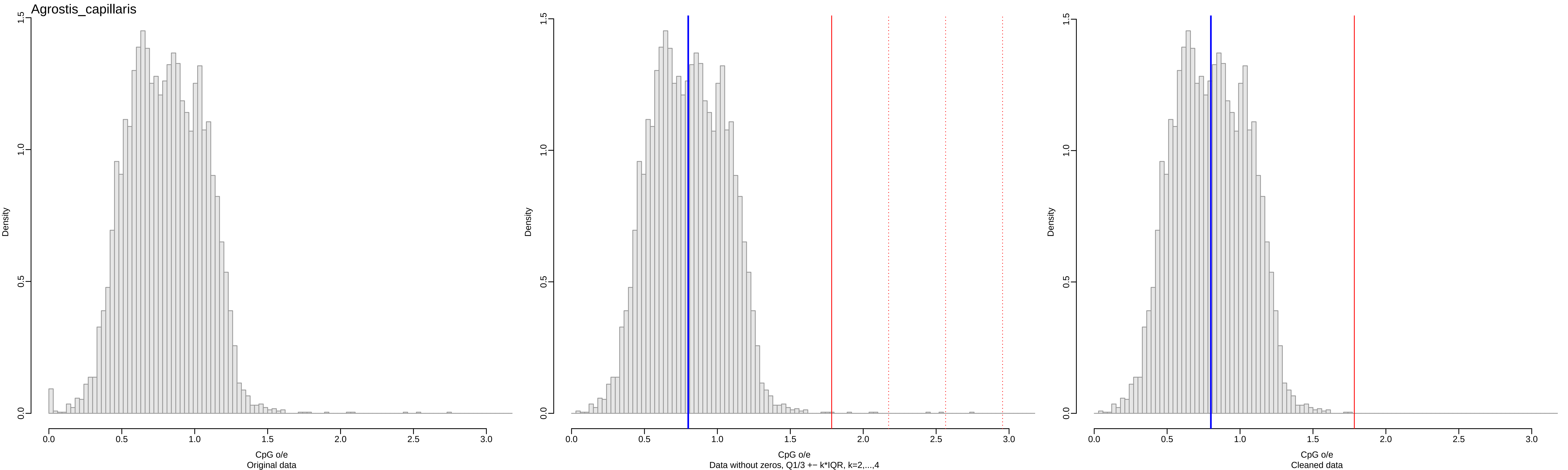

Agrostis_capillaris
Density
CpG o/e
Original data
Data without zeros, Q1/3 +− k*IQR, k=2,...,4
Cleaned data

Agrostis_stolonifera

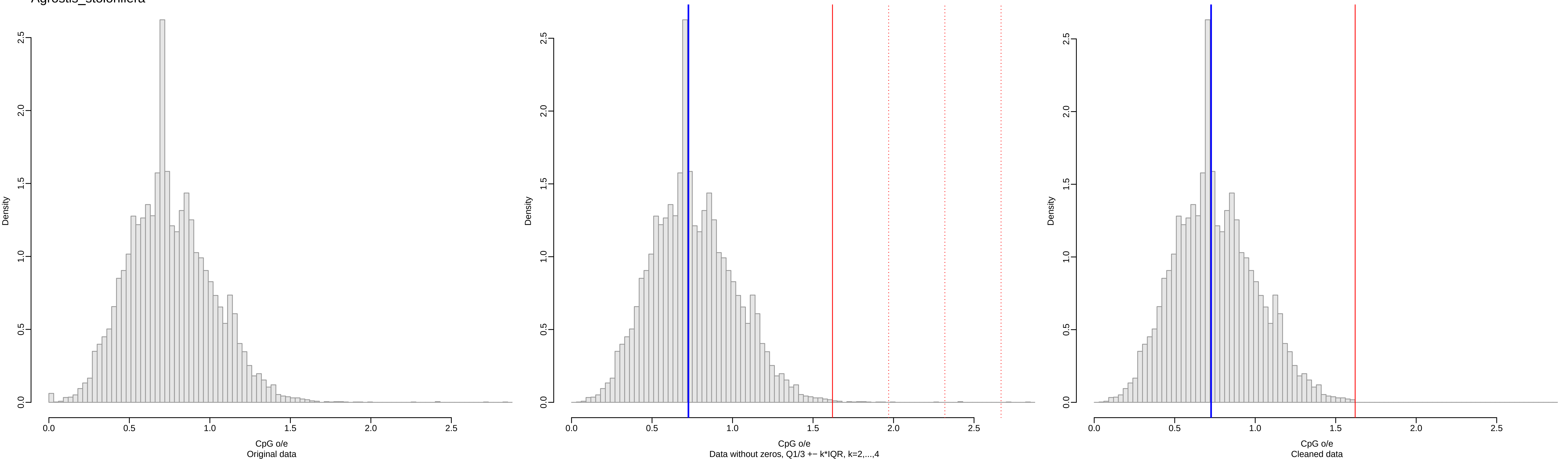

Aiptasia_pallida

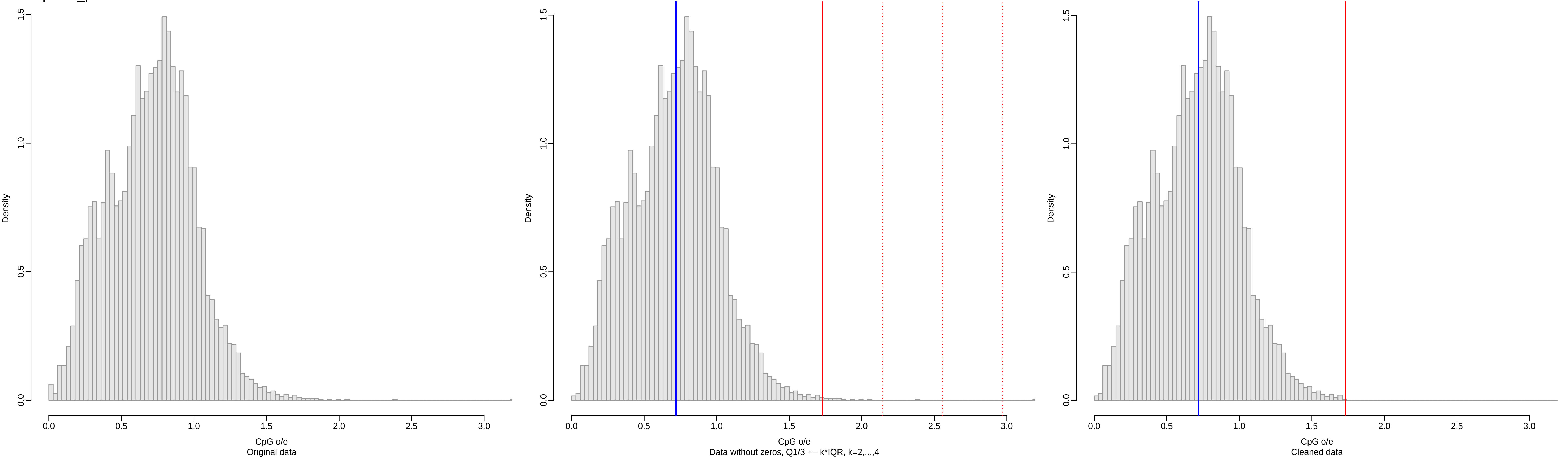

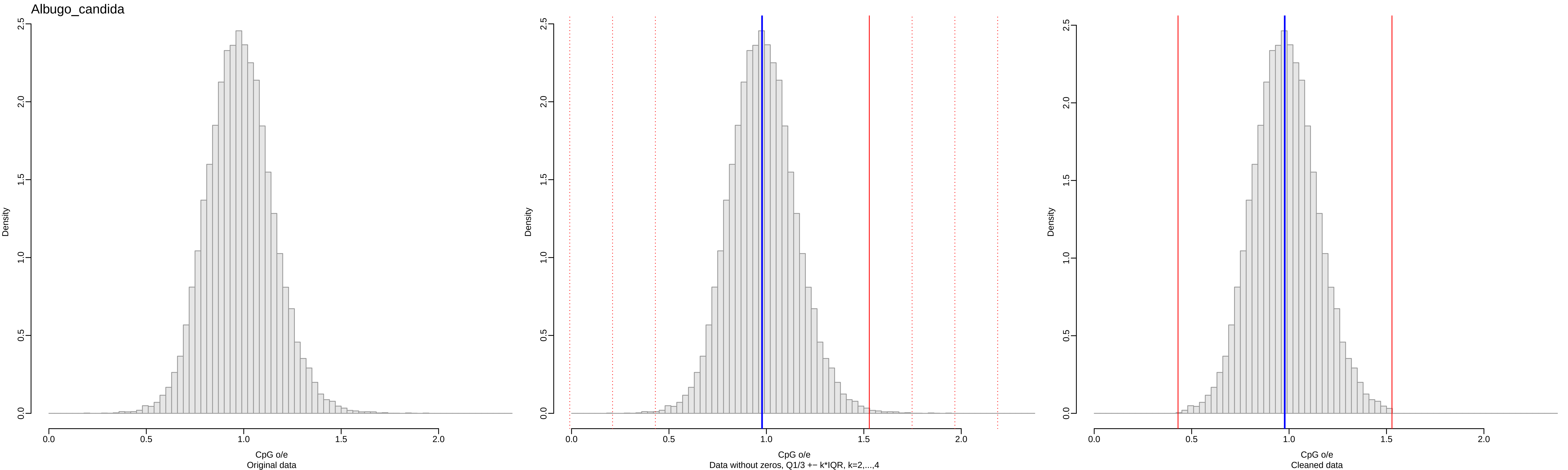
Albugo_candida
Density
CpG o/e
Original data
Data without zeros, Q1/3 +− k*IQR, k=2,...,4
Cleaned data
0.0
0.5
1.0
1.5
2.0
2.5

Alexandrium_catenella

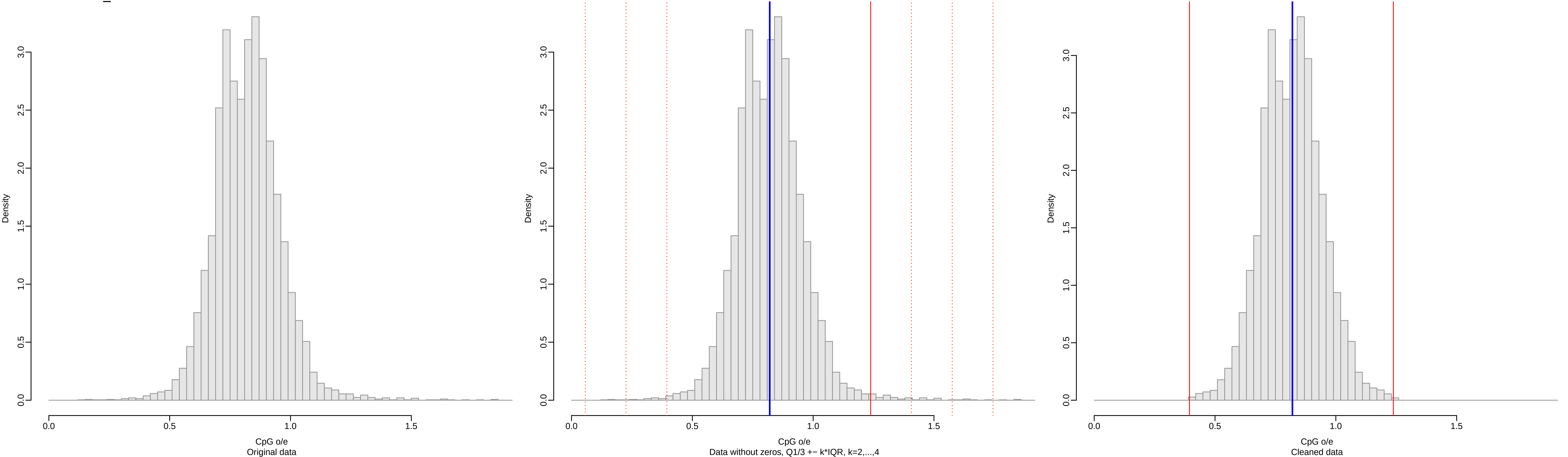

Alexandrium_minutum

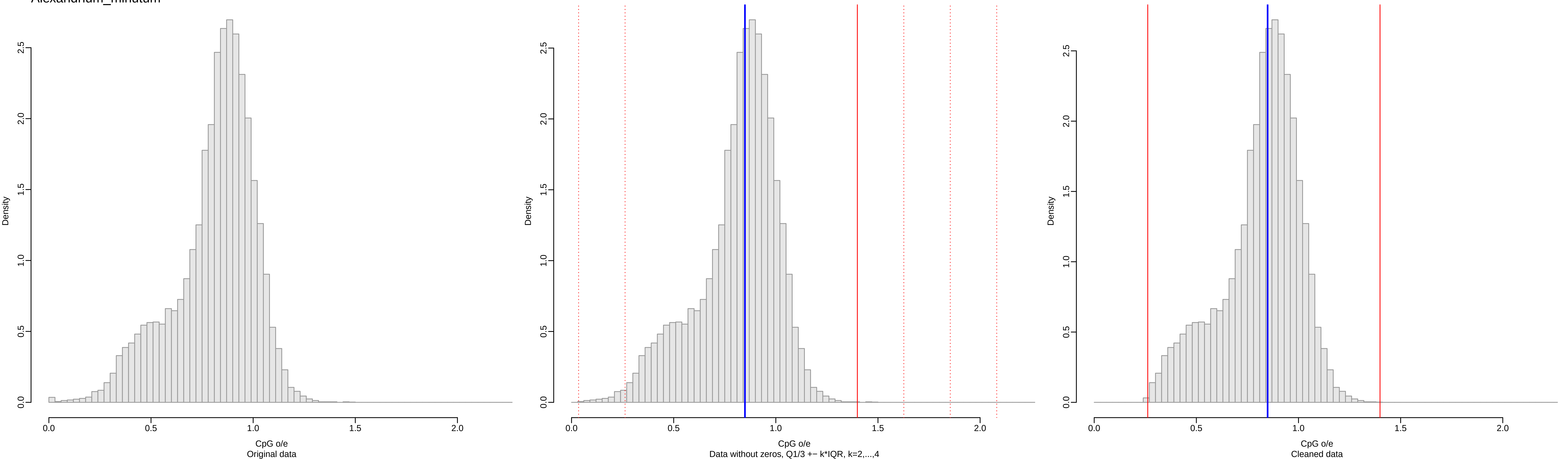

Alexandrium_ostenfeldii

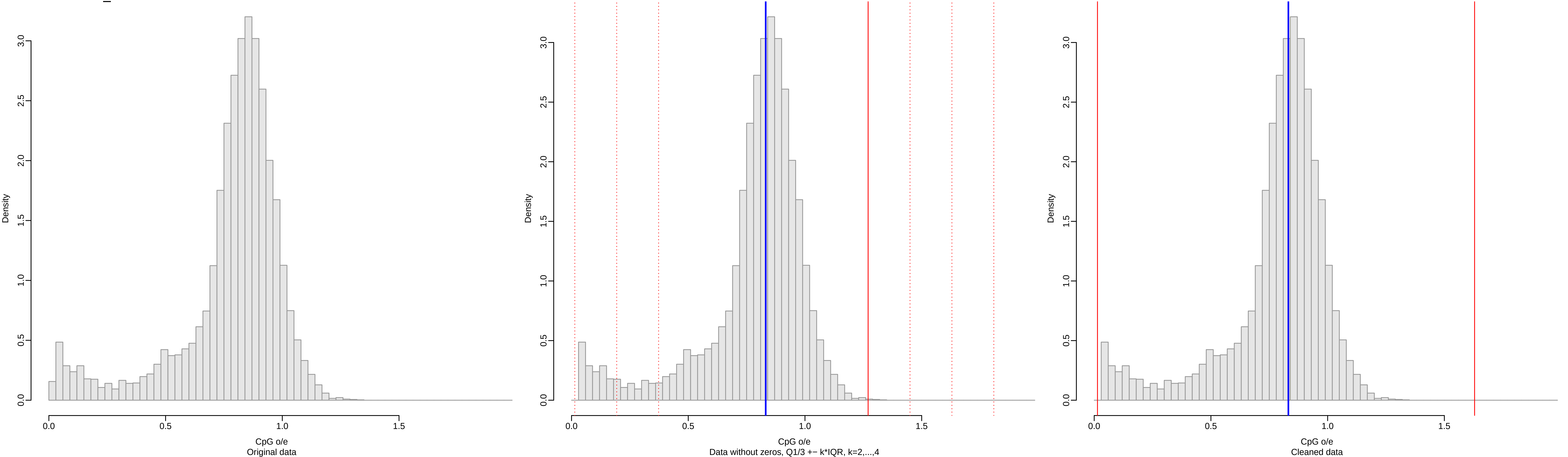

Alexandrium_tamarense

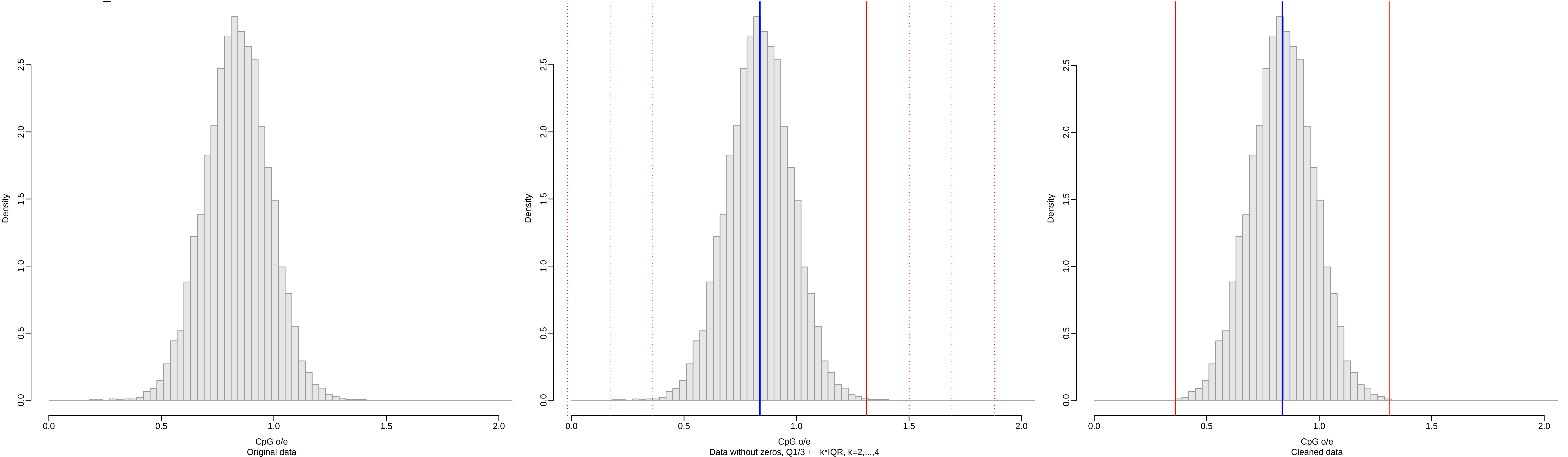

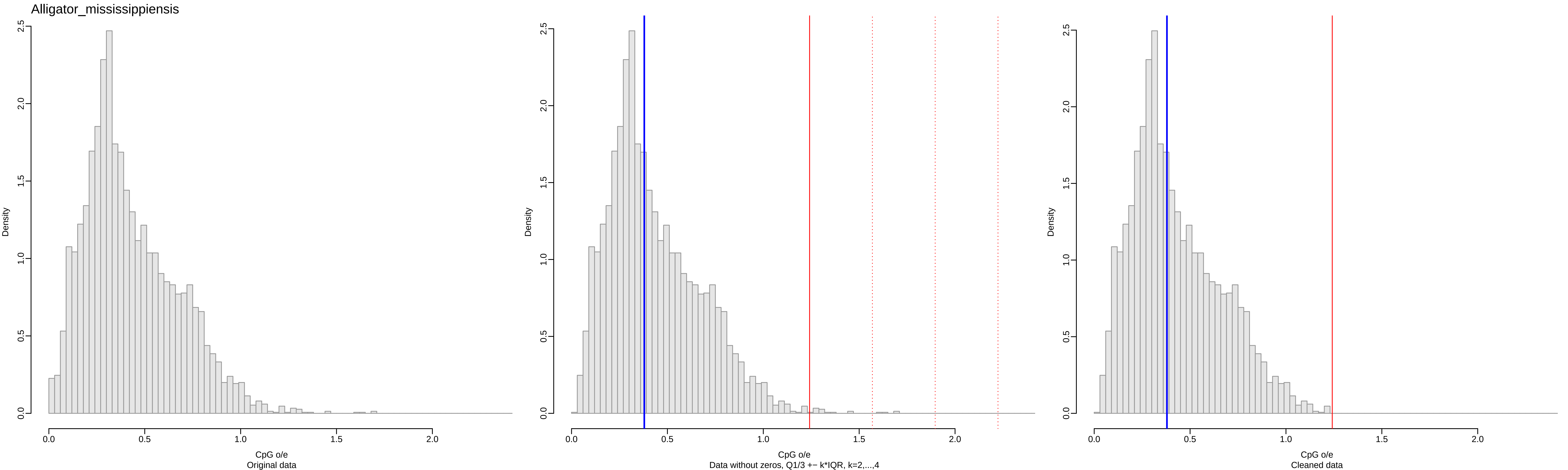
Alligator_mississippiensis
Density
CpG o/e
Original data
CpG o/e
Data without zeros, Q1/3 +− k*IQR, k=2,...,4
CpG o/e
Cleaned data

Allium_cepa

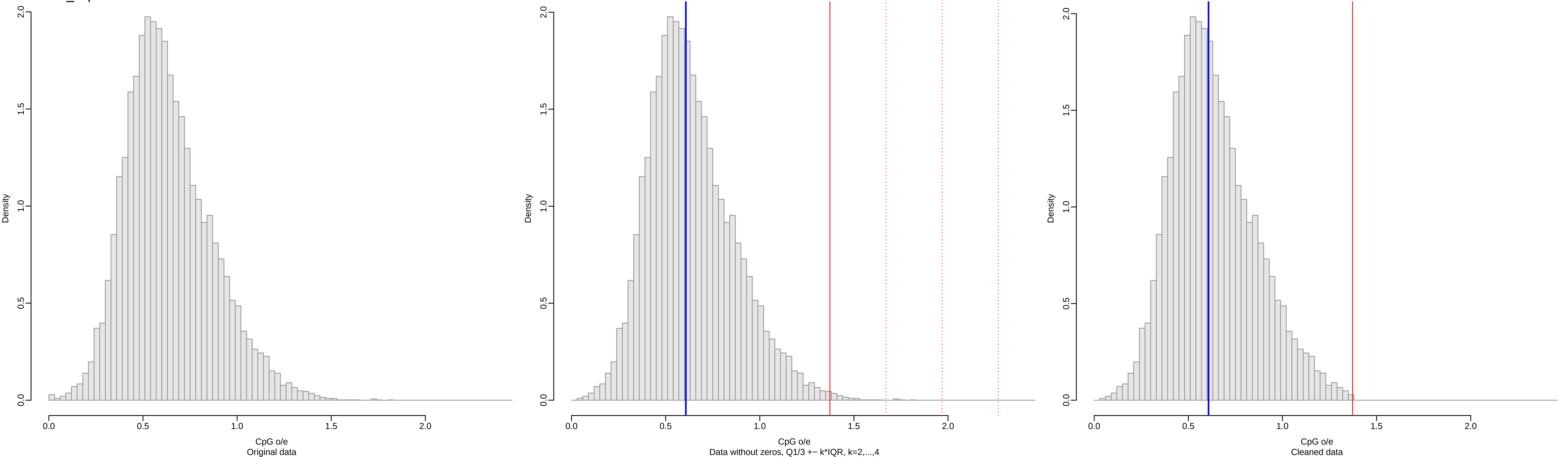
Density
CpG o/e
Original data
CpG o/e
Data without zeros, Q1/3 +− k*IQR, k=2,...,4
CpG o/e
Cleaned data

Allium_sativum

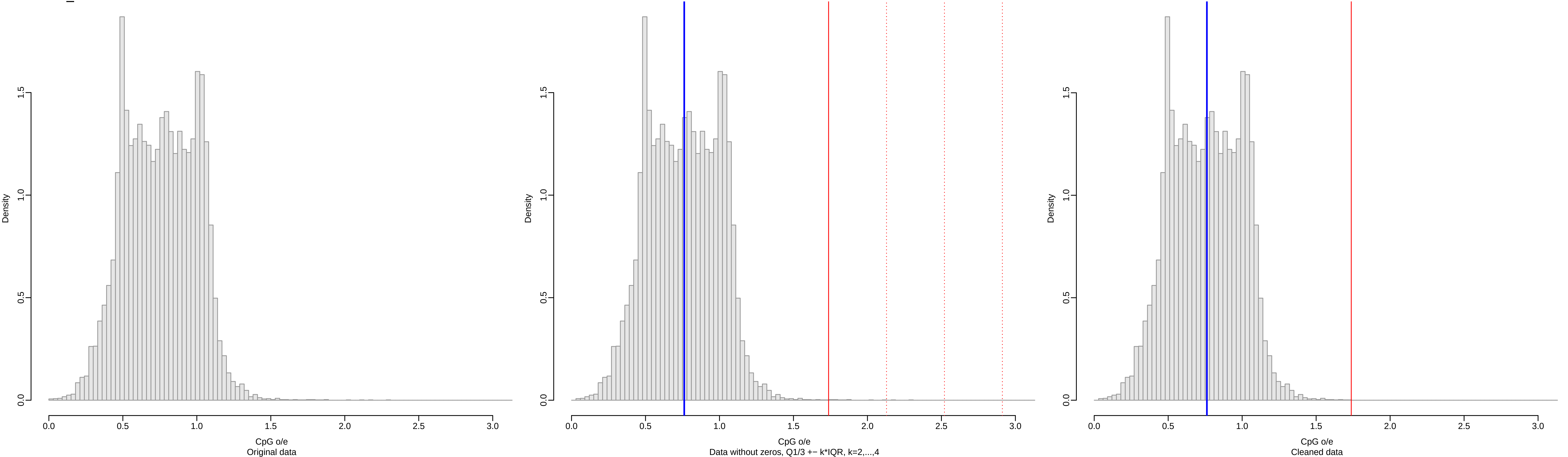

Allomyces_macrogynus

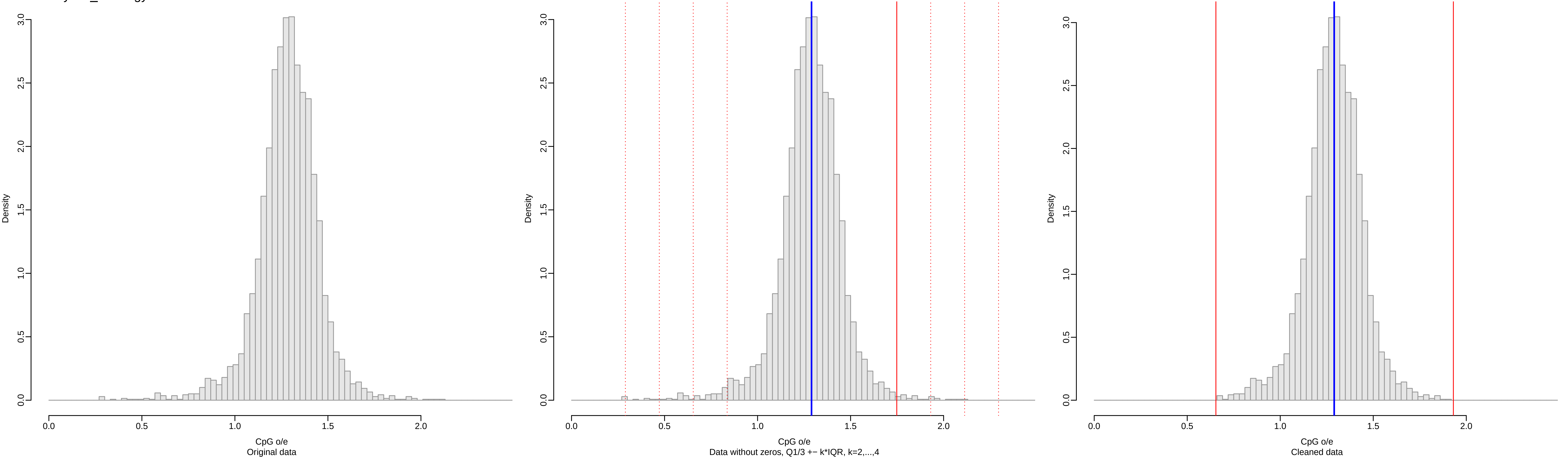

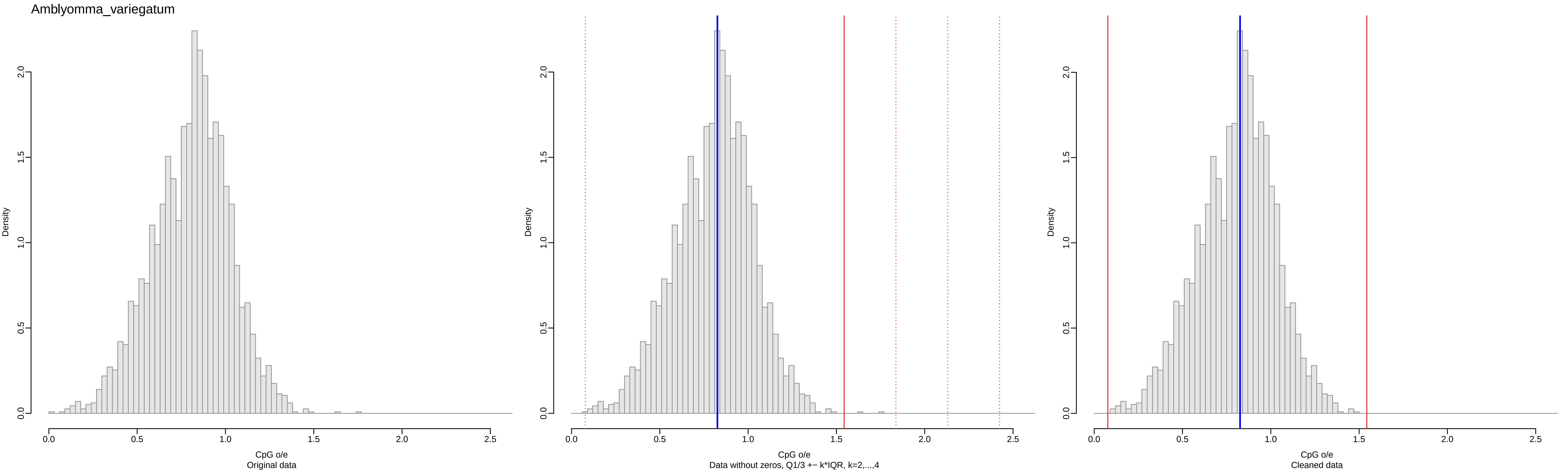
Amblyomma_variegatum
Density
CpG o/e
Original data
Density
CpG o/e
Data without zeros, Q1/3 +− k*IQR, k=2,...,4
Density
CpG o/e
Cleaned data

Ambystoma_mexicanum

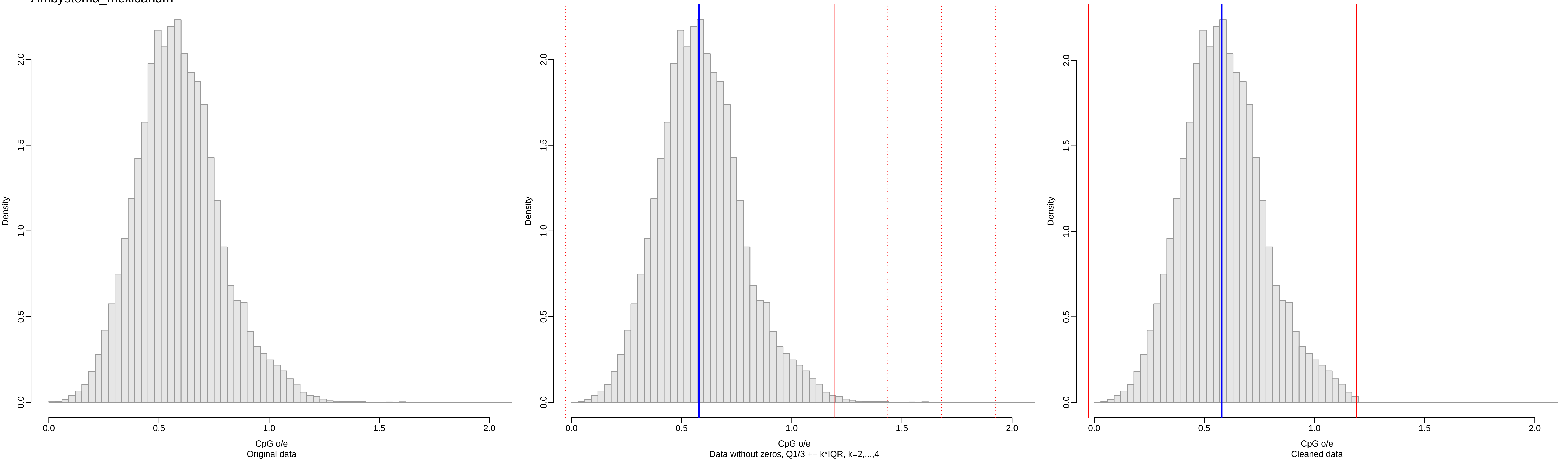

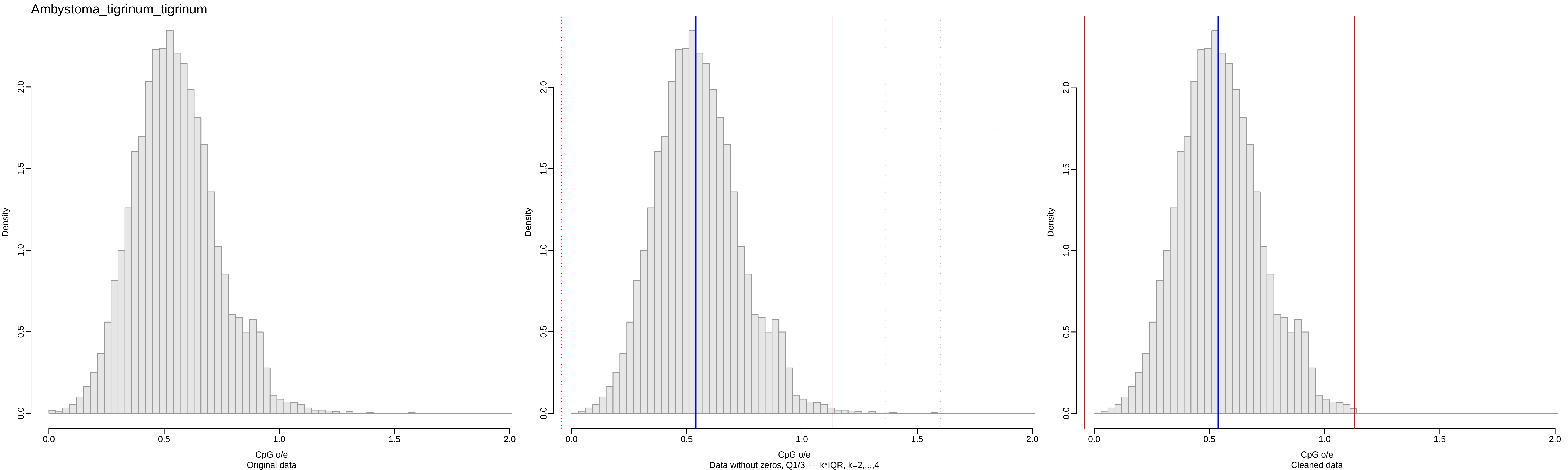
Ambystoma_tigrinum_tigrinum
Density
CpG o/e
Original data
Density
CpG o/e
Data without zeros, Q1/3 +− k*IQR, k=2,...,4
Density
CpG o/e
Cleaned data

Ampelisca_abdita

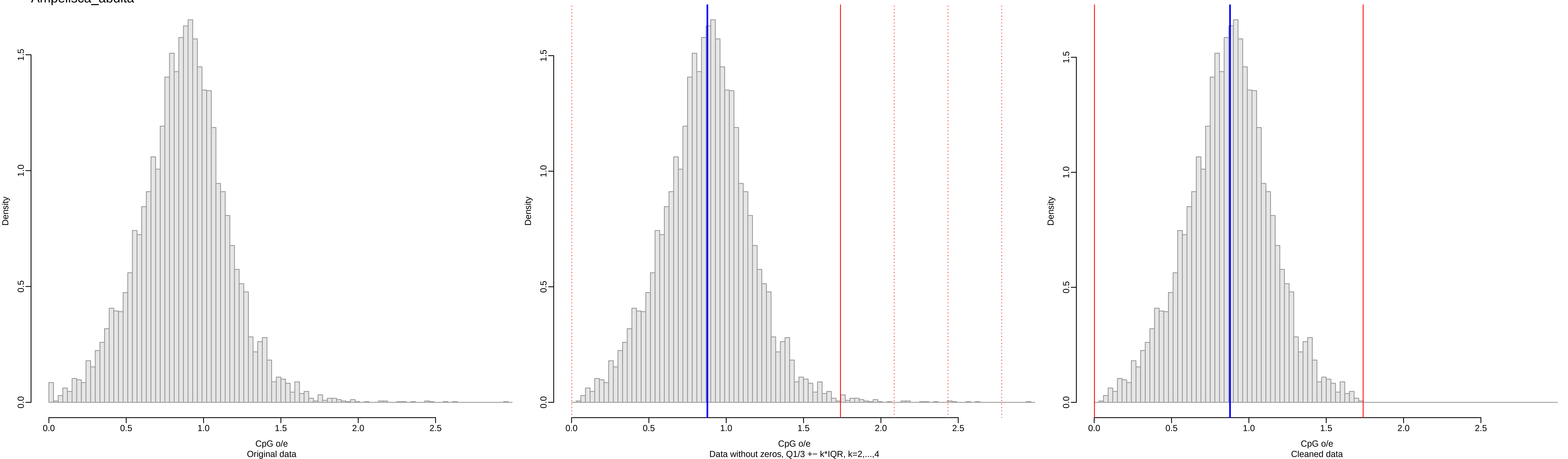

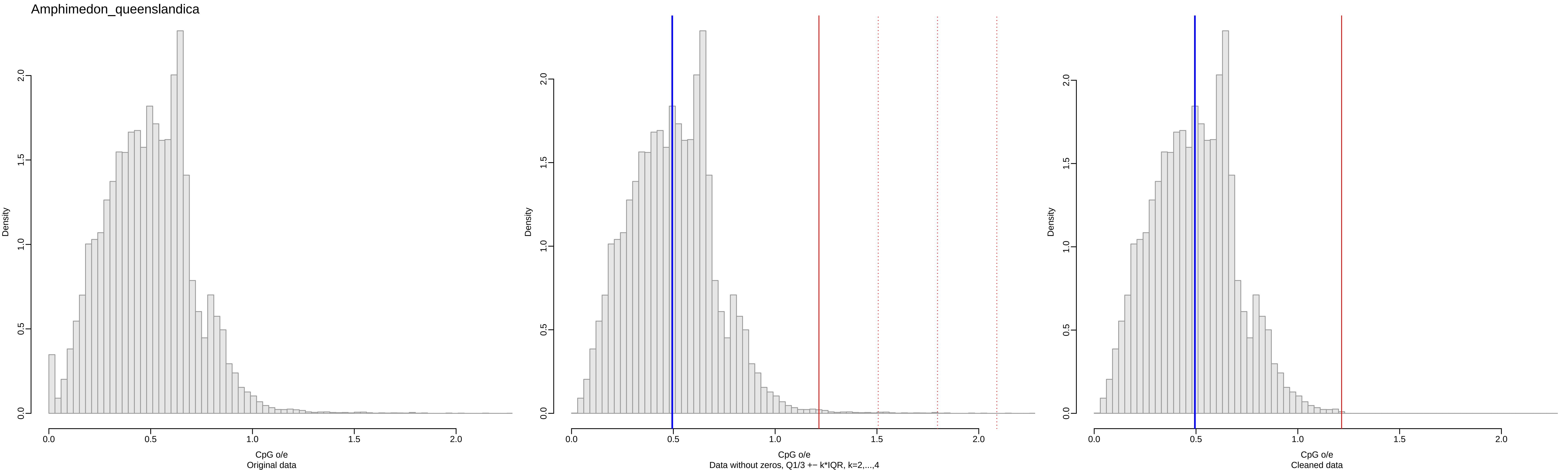
Amphimedon_queenslandica
Density
CpG o/e
Original data
Data without zeros, Q1/3 +− k*IQR, k=2,...,4
Cleaned data
0.0
0.5
1.0
1.5
2.0

Amphiura_filiformis

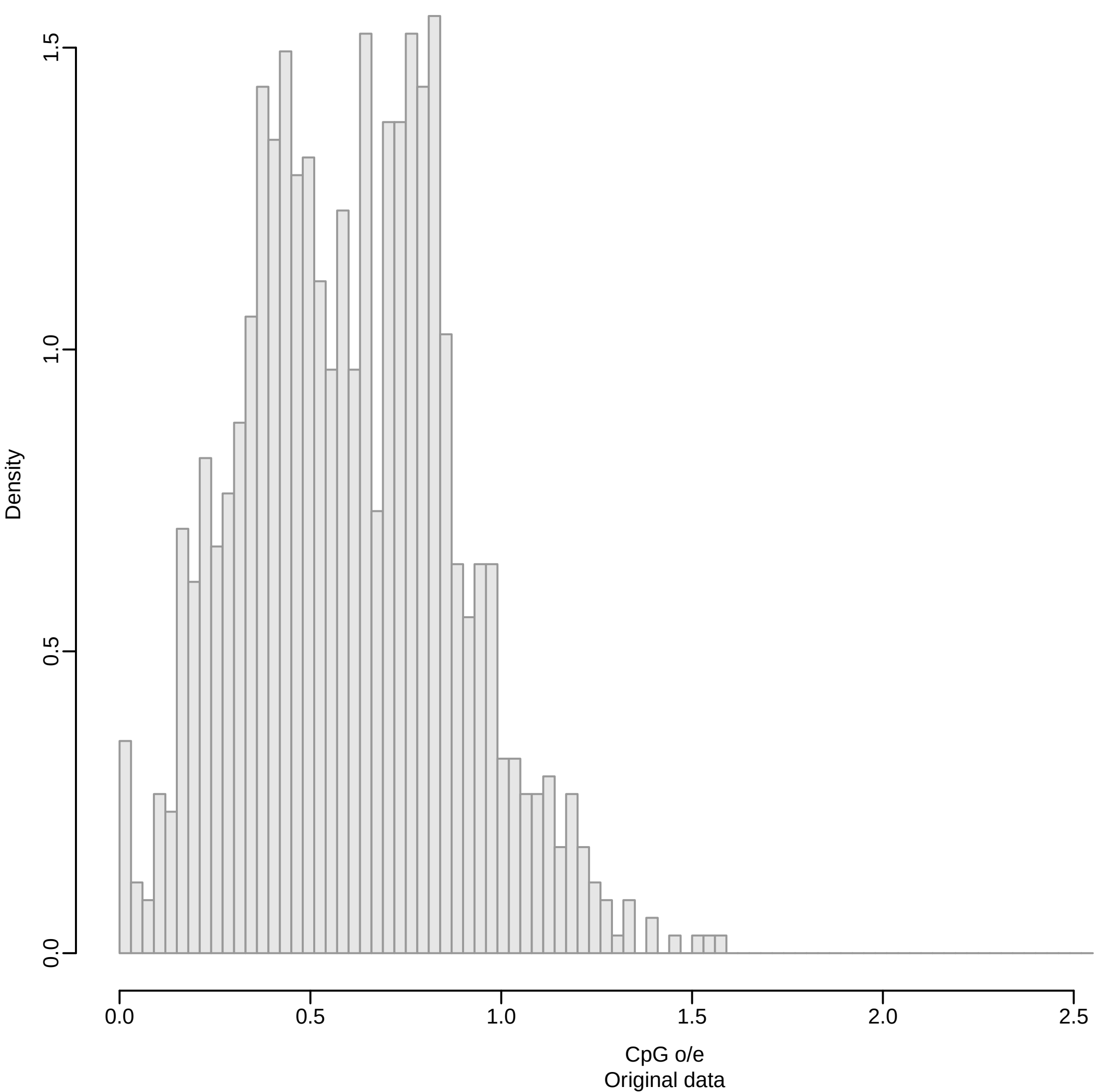

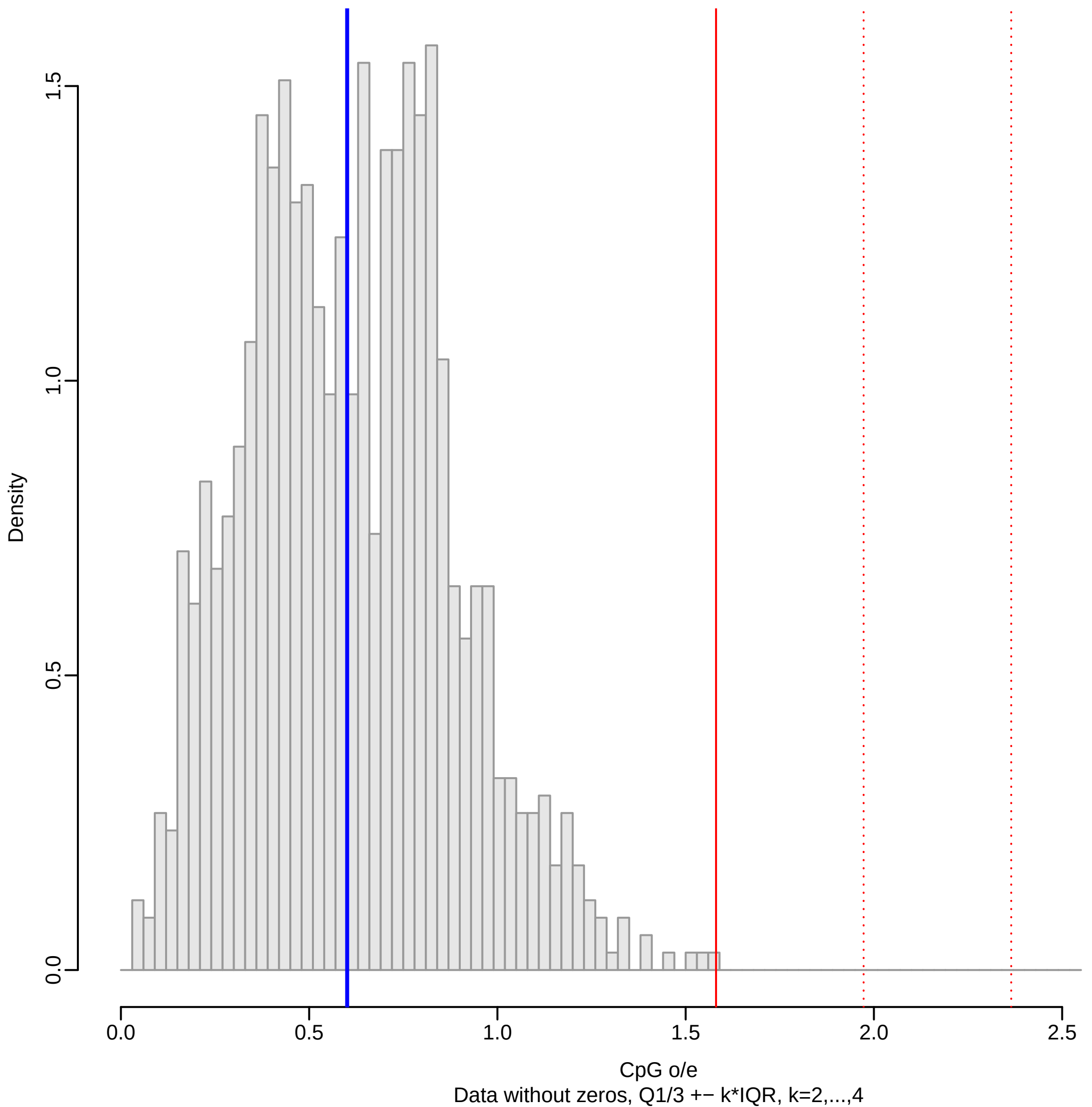

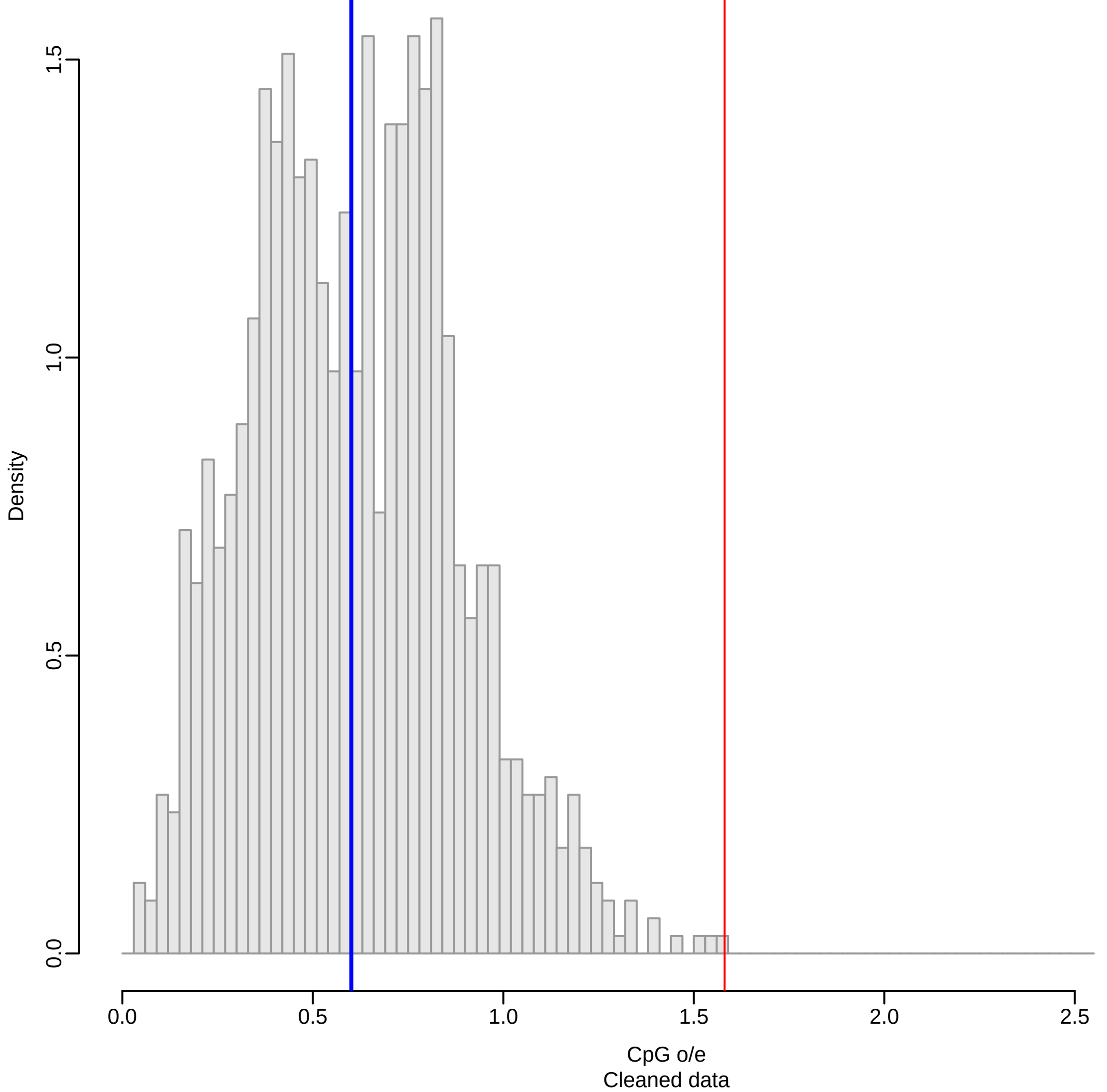

Anas_platyrhynchos

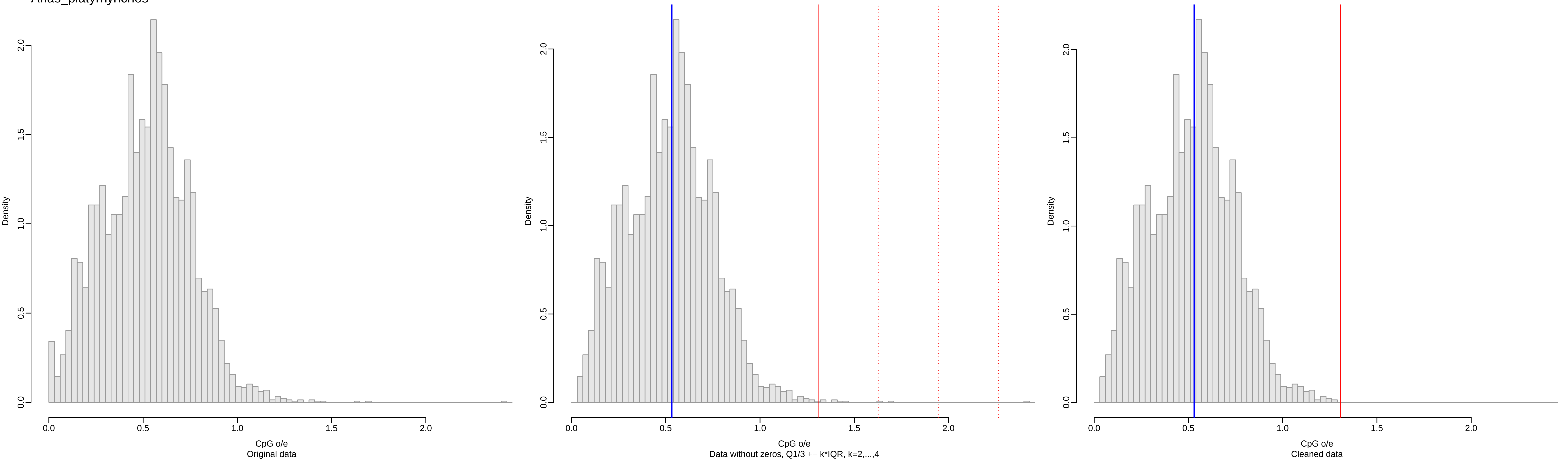

Ancylostoma_caninum

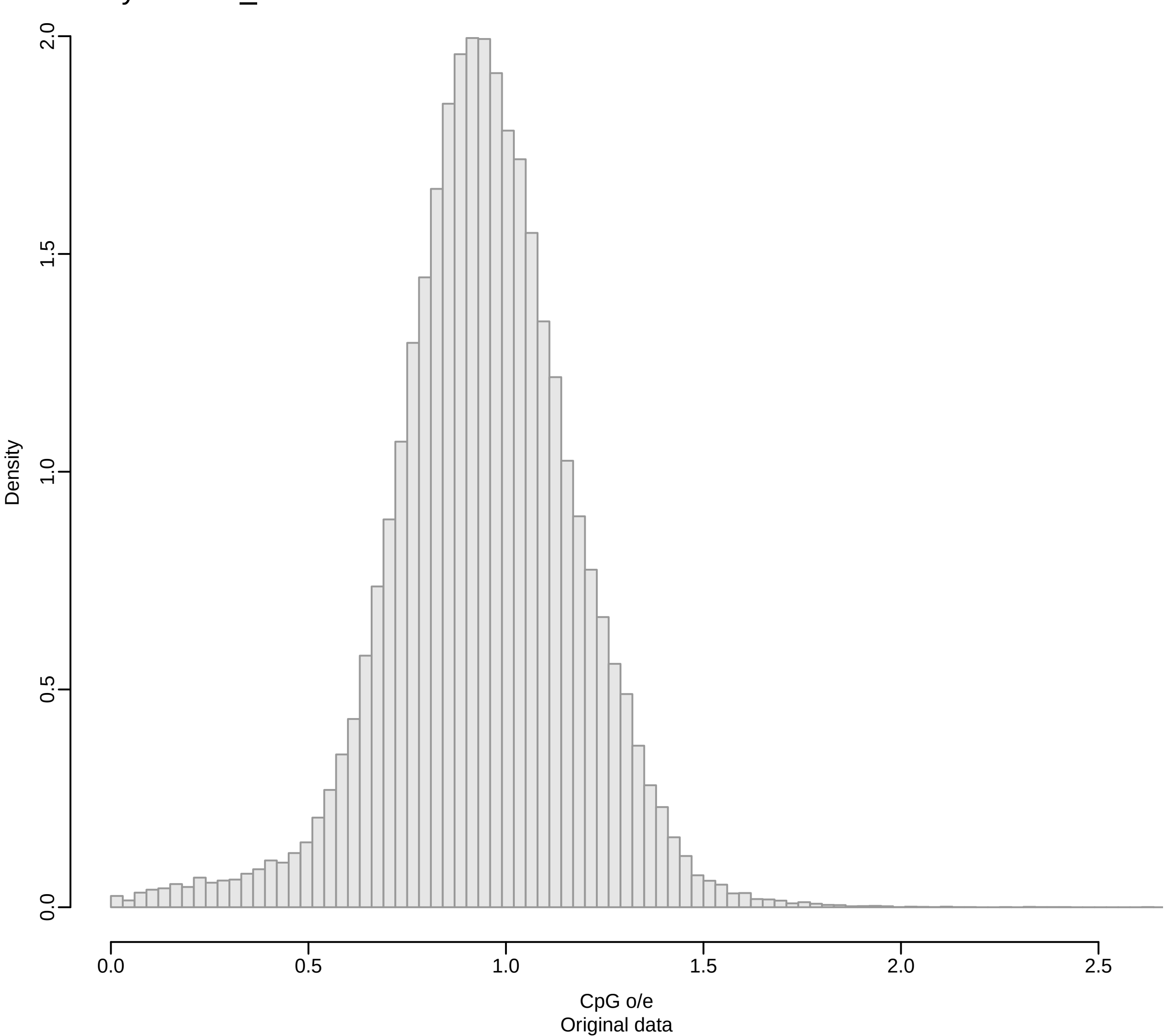

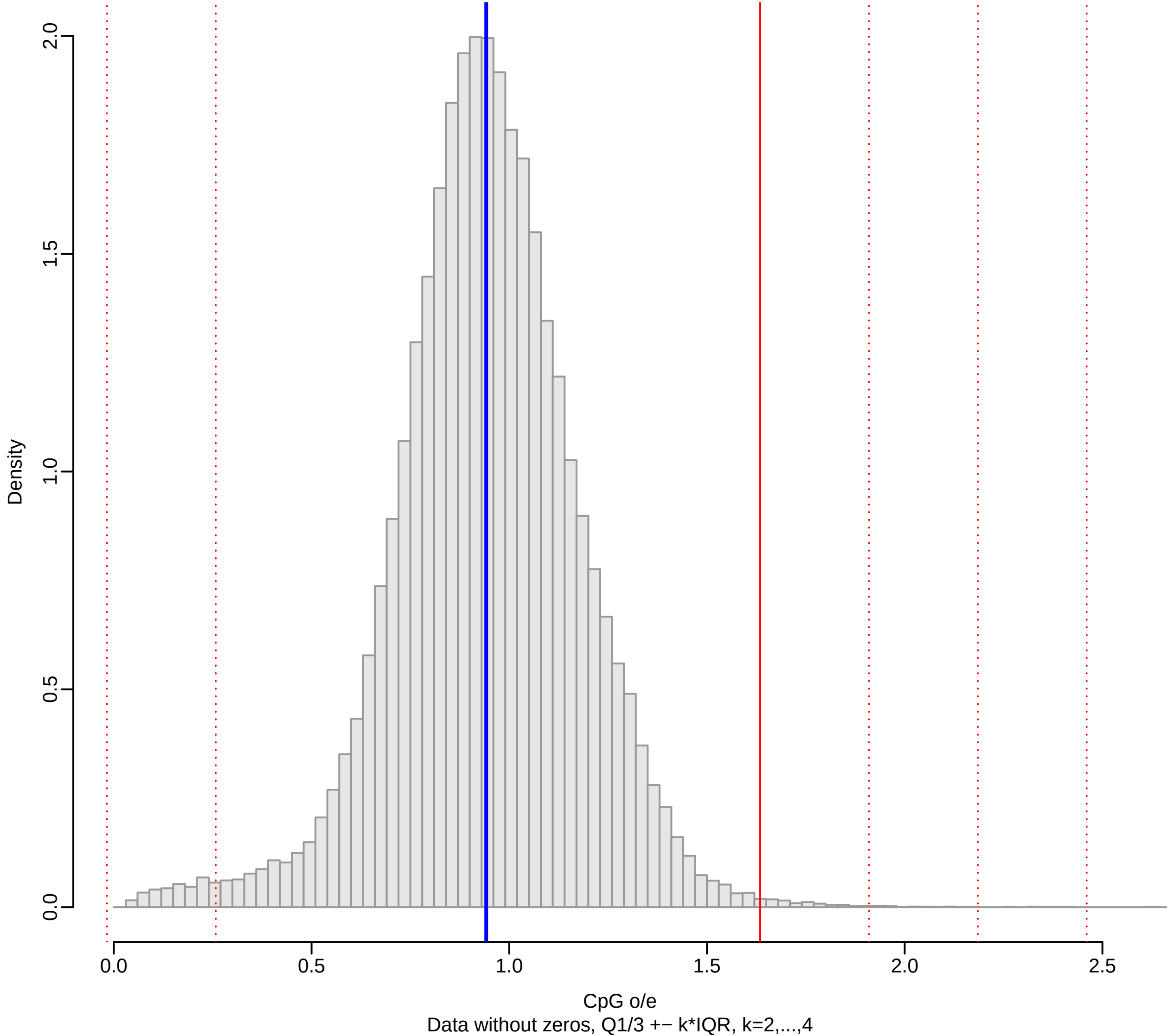

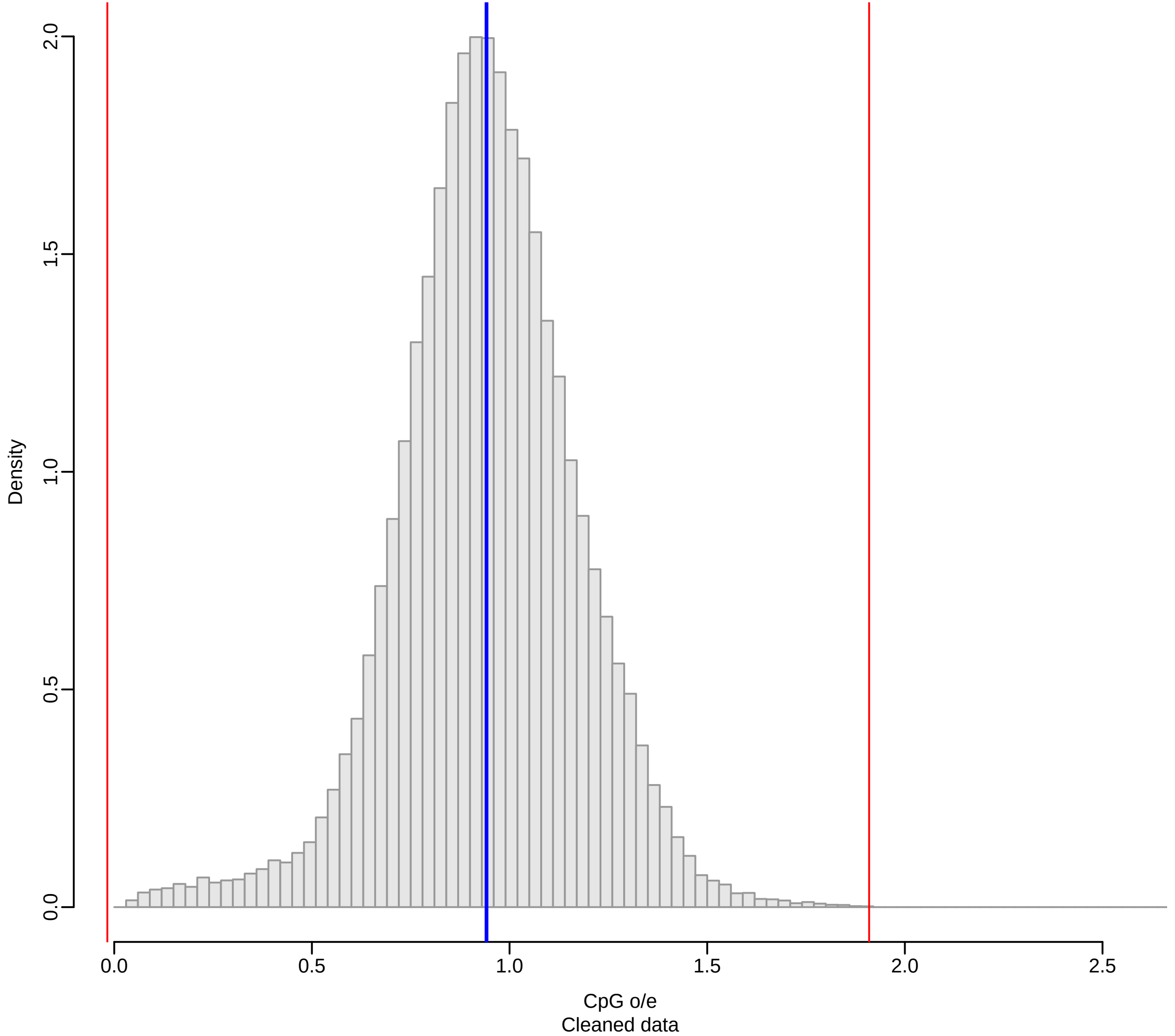

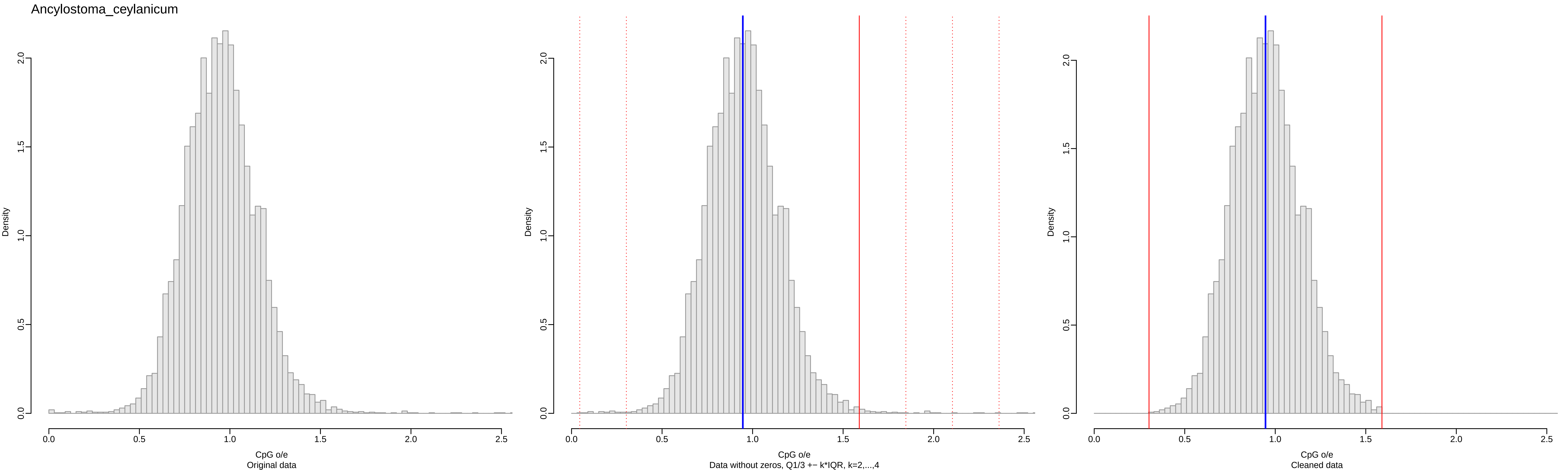

Ancylostoma_ceylanicum
Density
CpG o/e
Original data
Data without zeros, Q1/3 +− k*IQR, k=2,...,4
Cleaned data
0.0
0.5
1.0
1.5
2.0
2.5

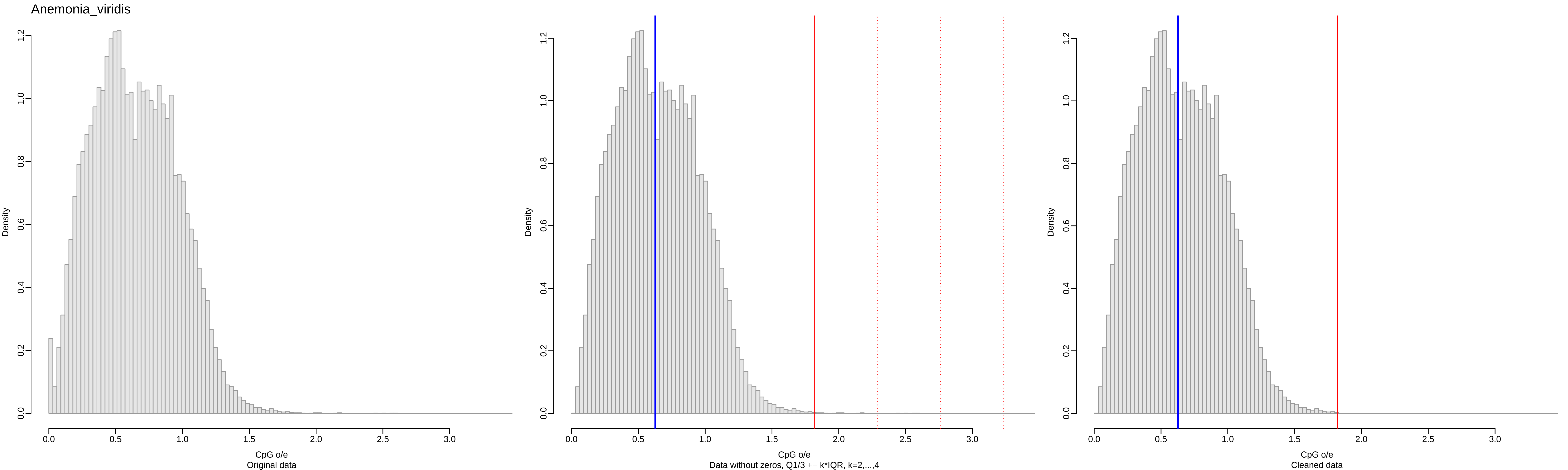

Anemonia_viridis
Density
CpG o/e
Original data
Data without zeros, Q1/3 +− k*IQR, k=2,...,4
Cleaned data

Anolis_carolinensis

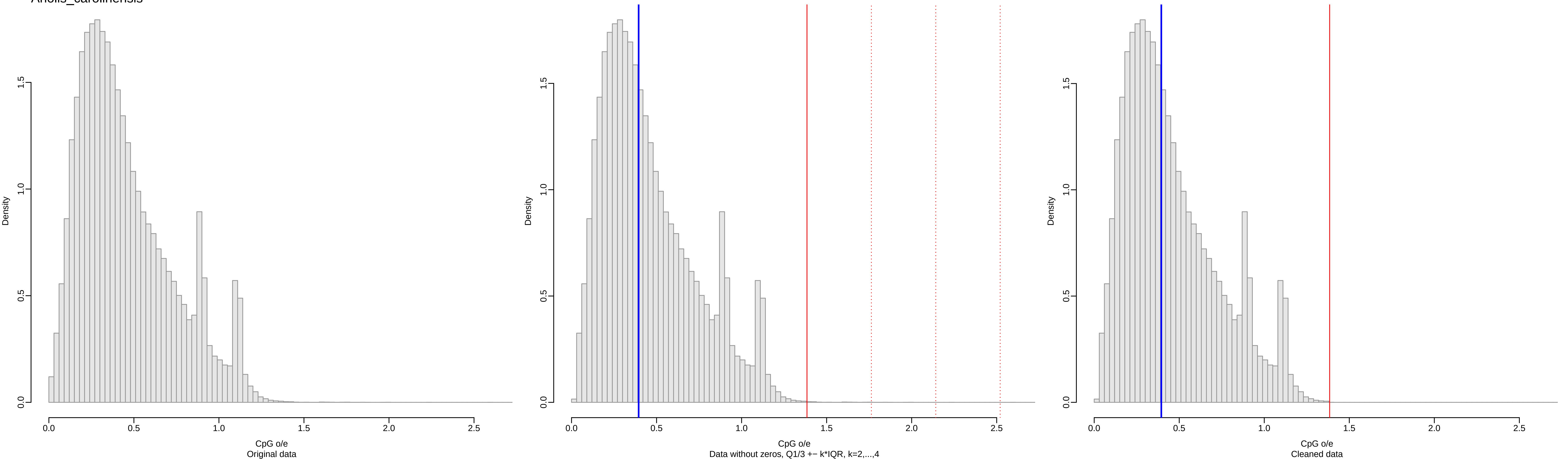

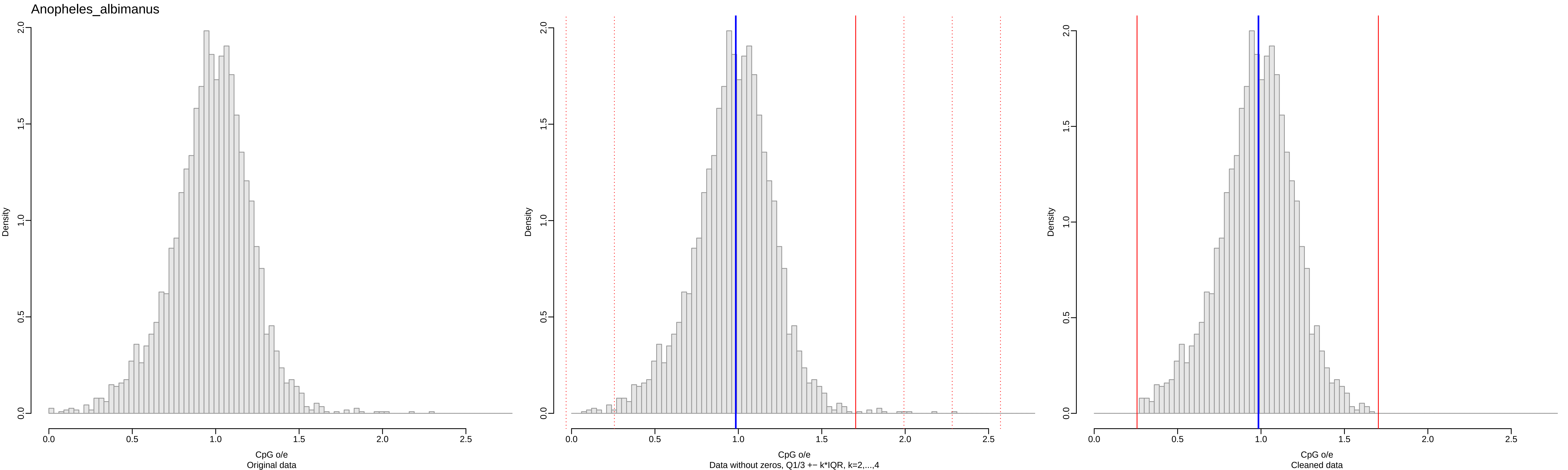

Anopheles_albimanus
Density
CpG o/e
Original data
Data without zeros, Q1/3 +− k*IQR, k=2,...,4
Cleaned data

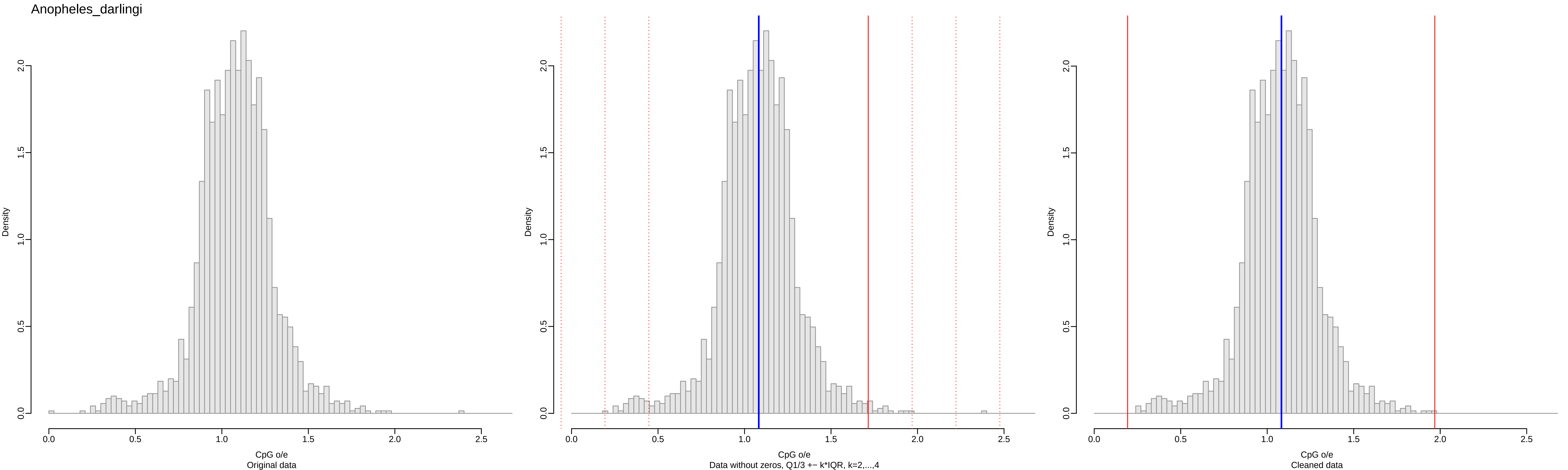

Anopheles_darlingi
Density
0.0
0.5
1.0
1.5
2.0
0.0
0.5
1.0
1.5
2.0
2.5
CpG o/e
Original data
Data without zeros, Q1/3 +− k*IQR, k=2,...,4
Cleaned data

Anopheles_gambiae

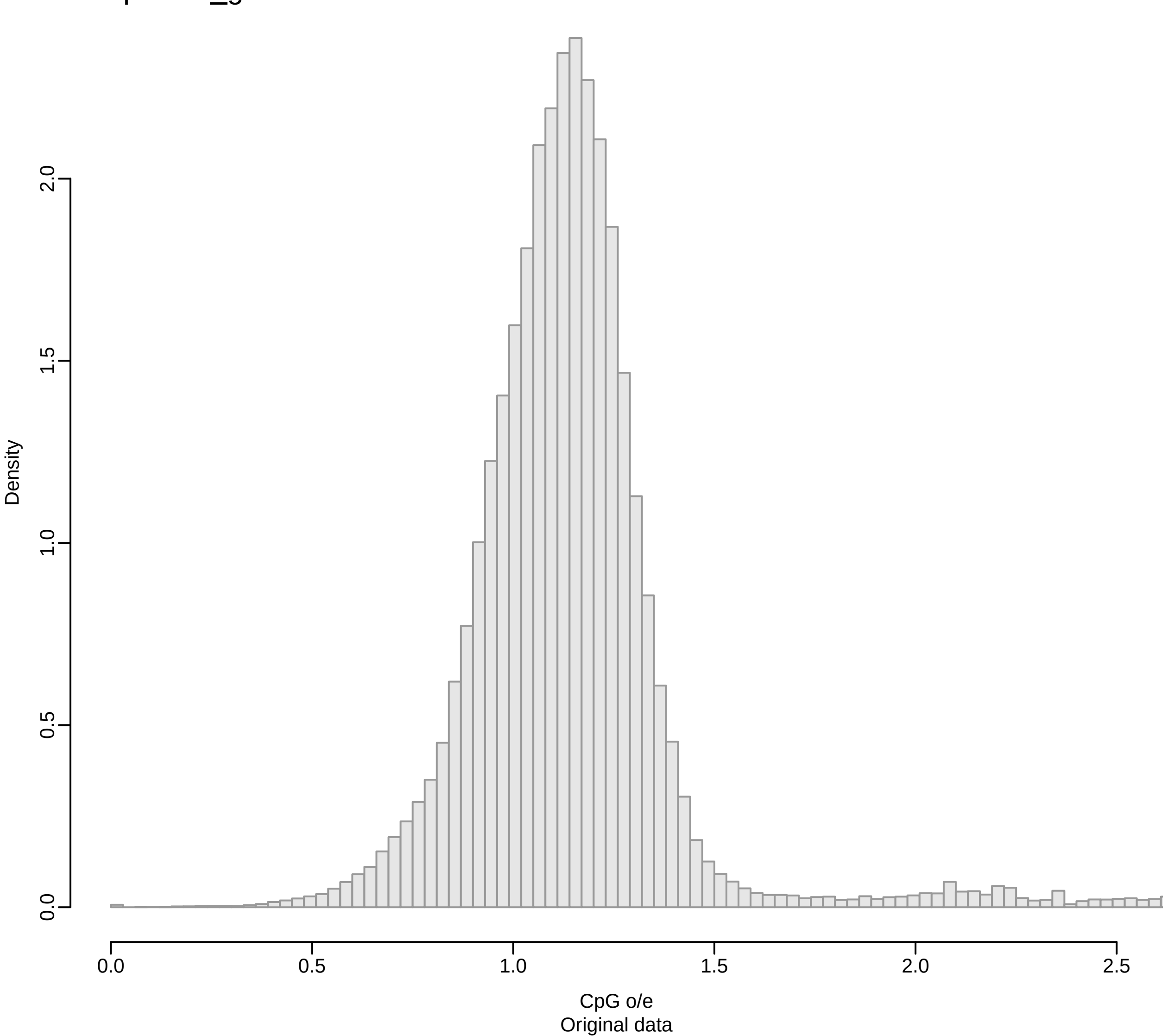

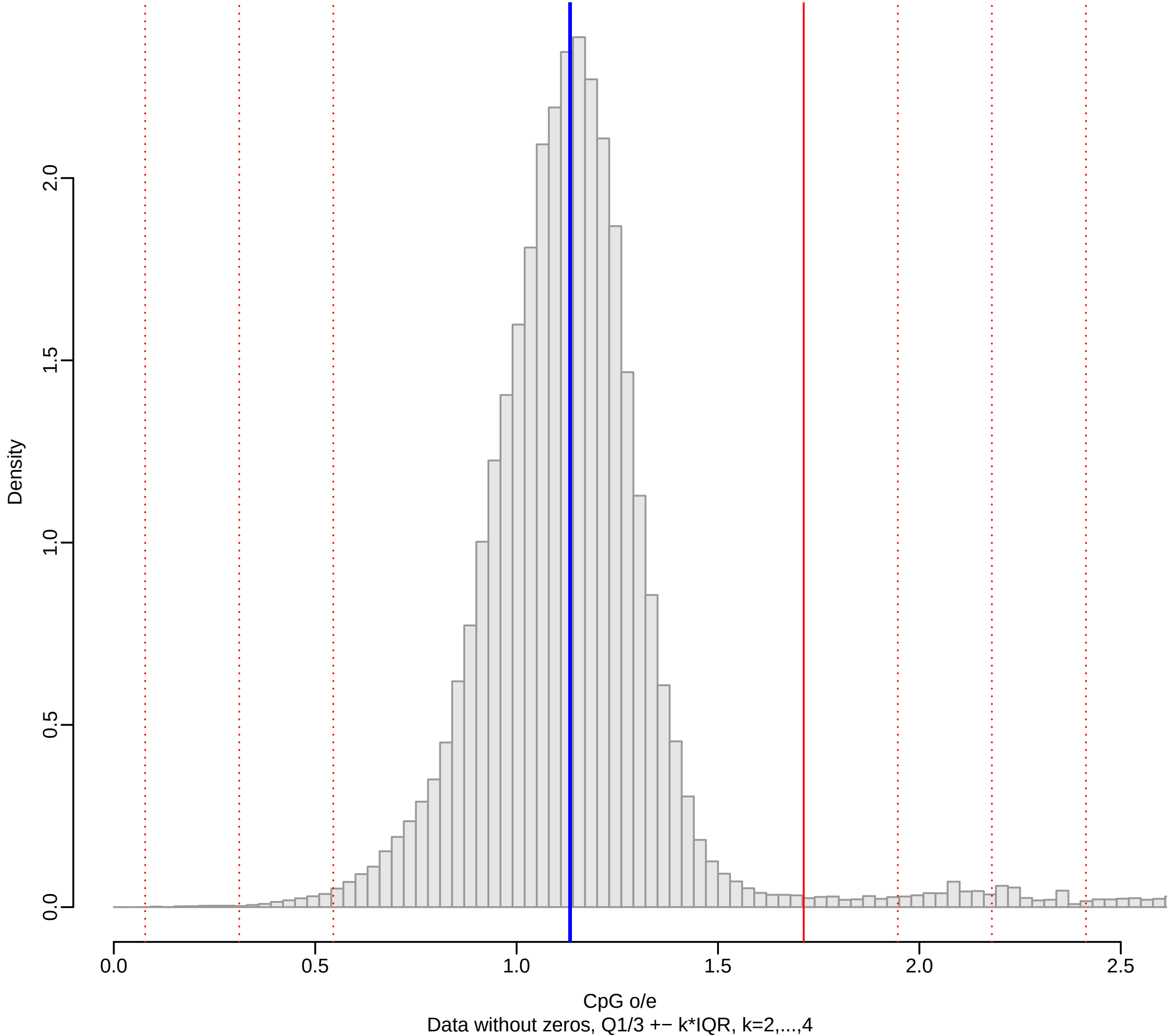

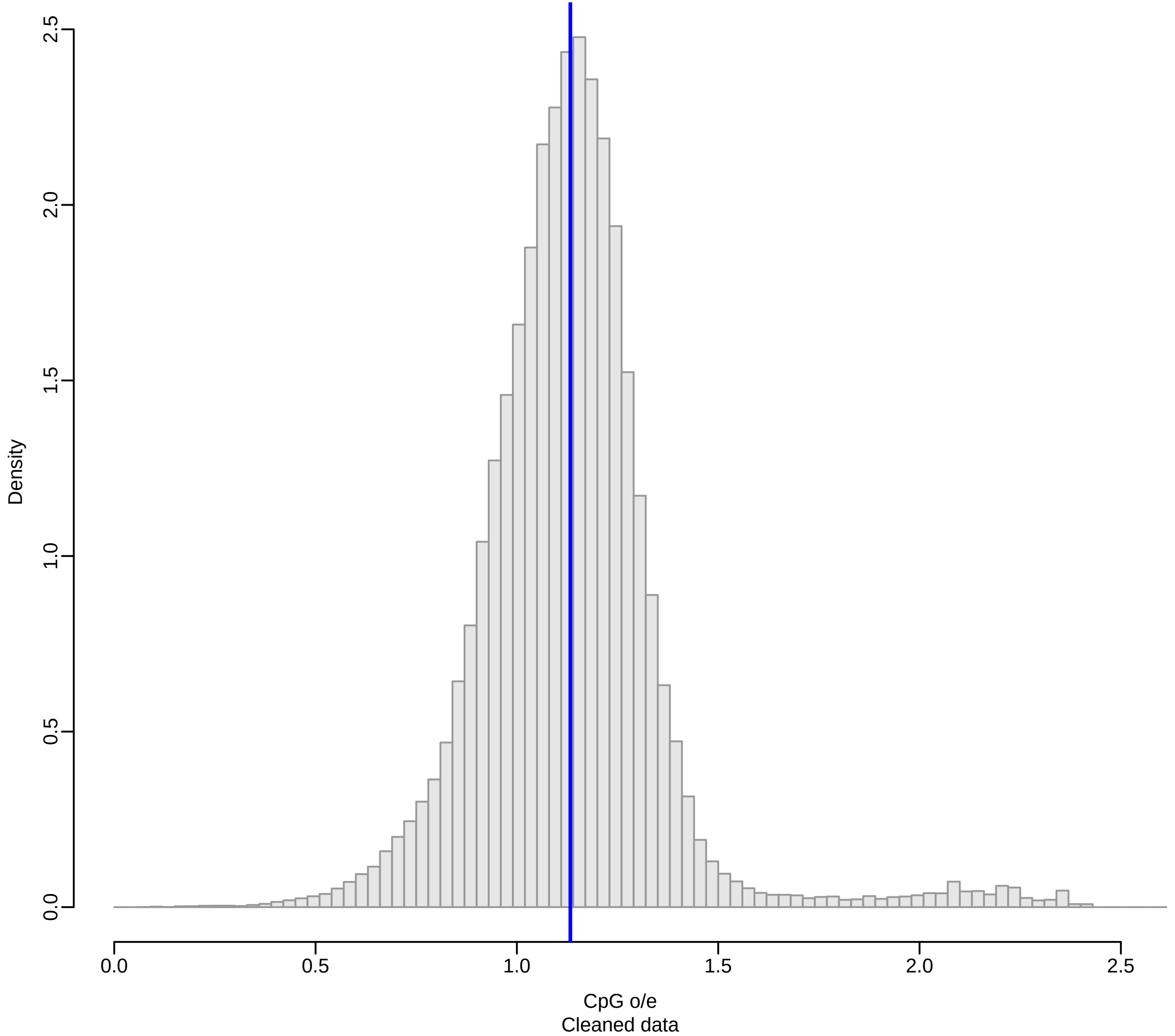

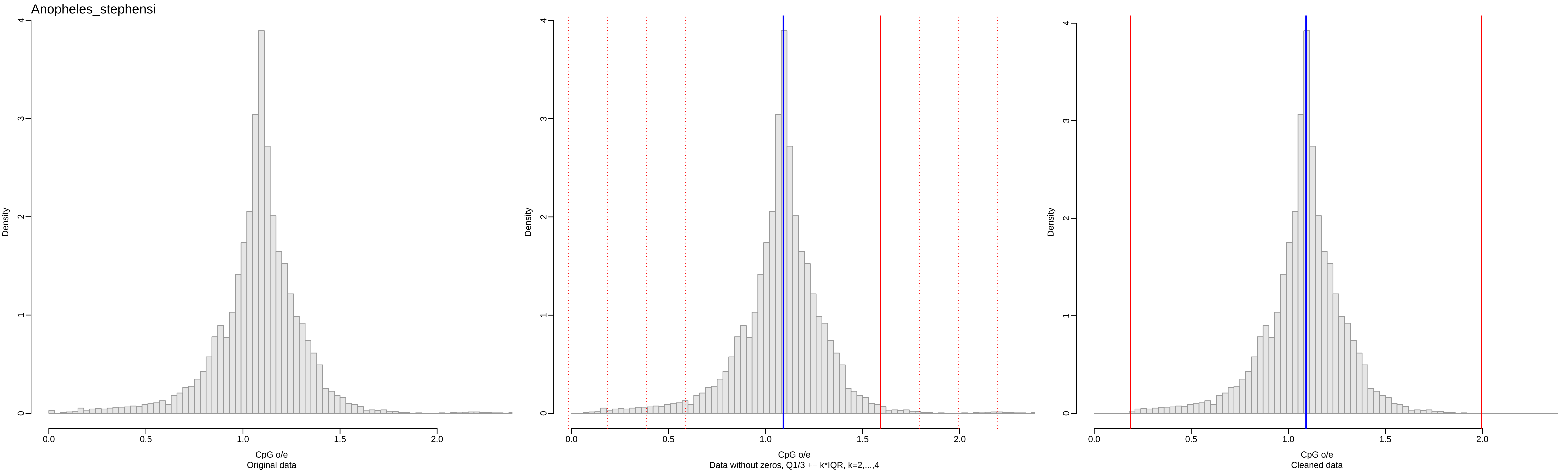
Anopheles_stephensi
Density
CpG o/e
Original data
Data without zeros, Q1/3 +− k*IQR, k=2,...,4
Cleaned data
0
1
2
3
4
0.0
0.5
1.0
1.5
2.0

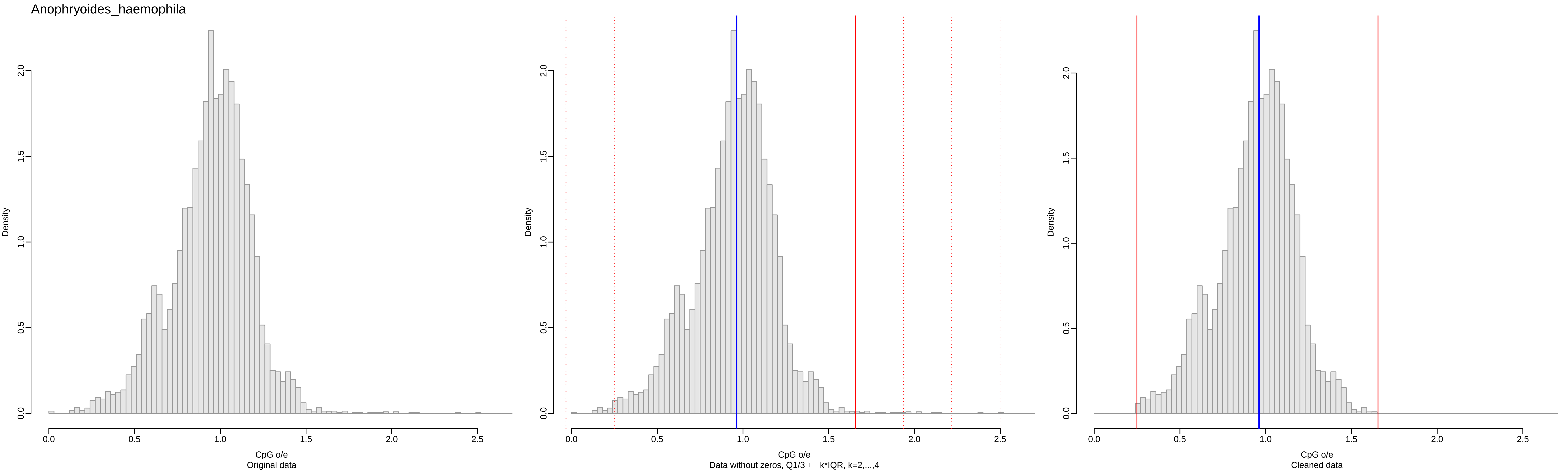
Anophryoides_haemophila
Density
CpG o/e
Original data
Data without zeros, Q1/3 +− k*IQR, k=2,...,4
Cleaned data
0.0
0.5
1.0
1.5
2.0
2.5

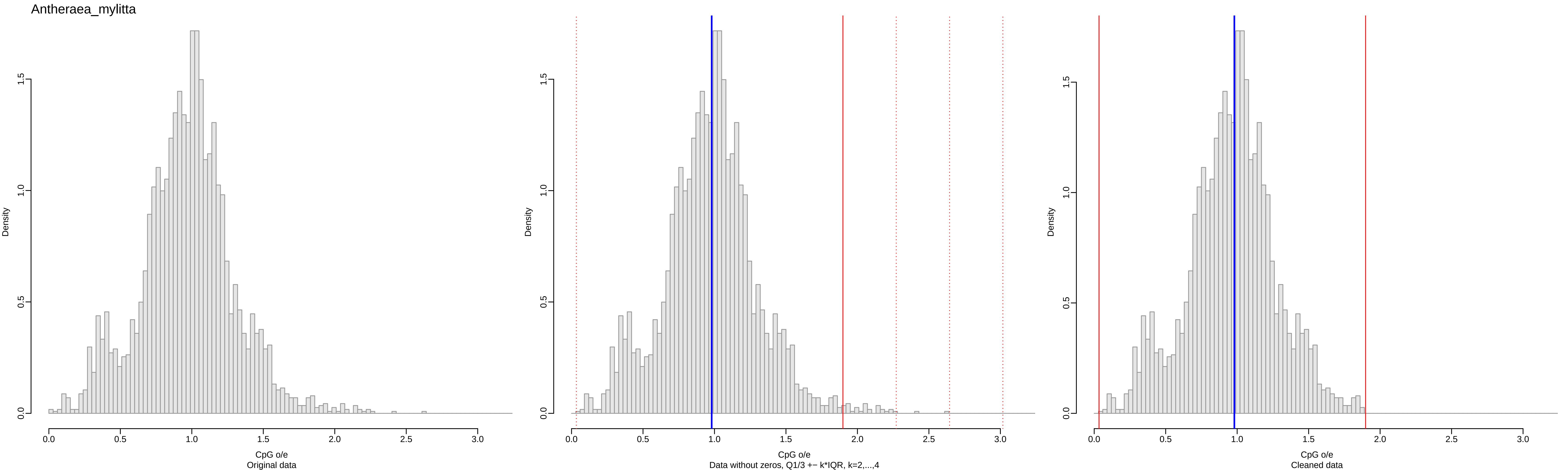
Antheraea_mylitta
Density
CpG o/e
Original data
Data without zeros, Q1/3 +− k*IQR, k=2,...,4
Cleaned data

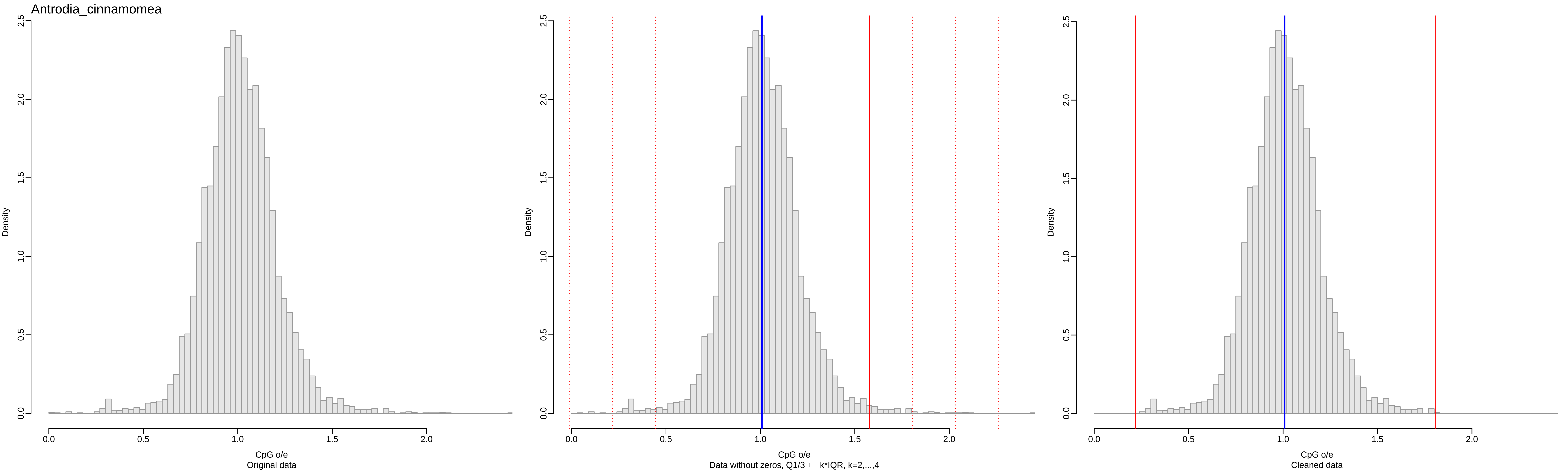

Antrodia_cinnamomea
Density
CpG o/e
Original data
Data without zeros, Q1/3 +− k*IQR, k=2,...,4
Cleaned data

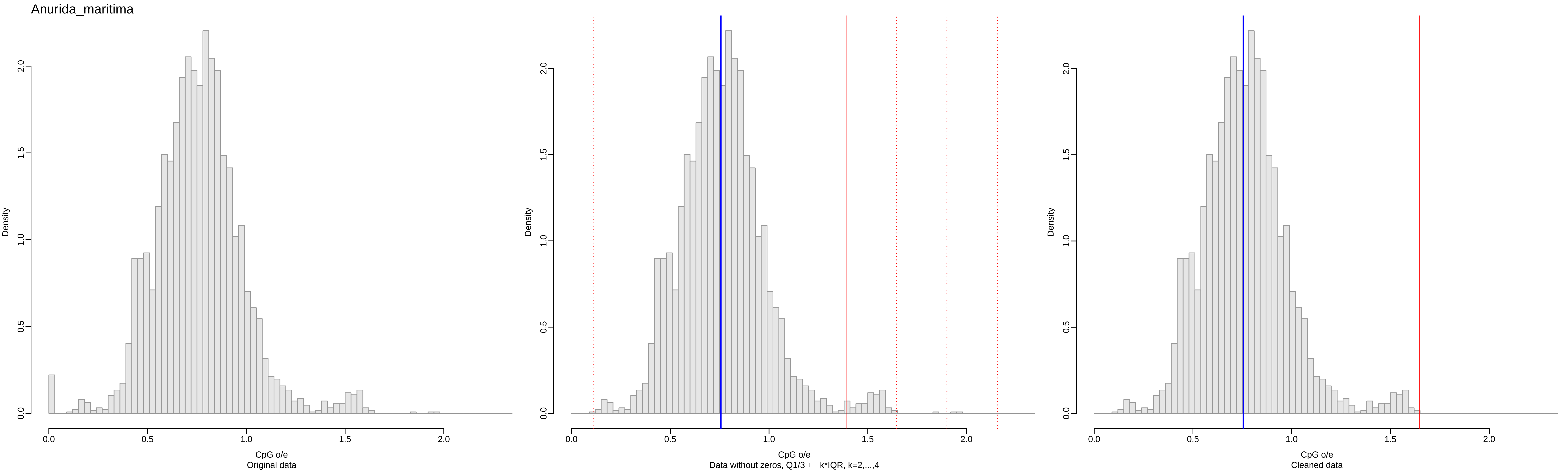
Anurida_maritima
Density
CpG o/e
Original data
Data without zeros, Q1/3 +− k*IQR, k=2,...,4
Cleaned data
0.0
0.5
1.0
1.5
2.0

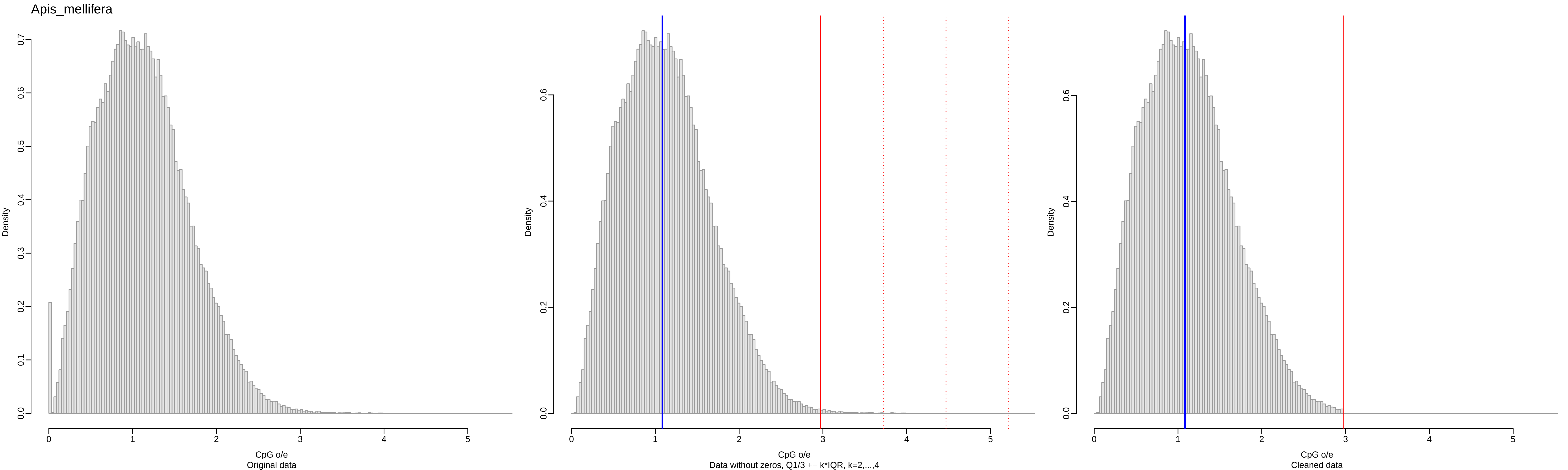

Apis_mellifera
Density
CpG o/e
Original data
Data without zeros, Q1/3 +− k*IQR, k=2,...,4
Cleaned data

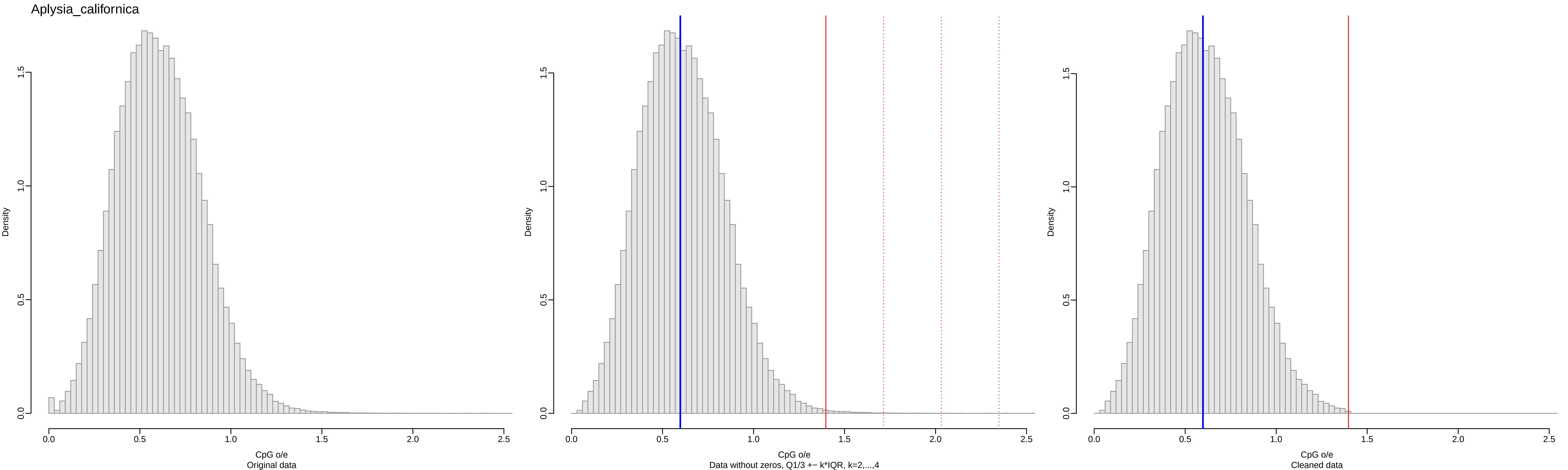

Aplysia_californica
Density
CpG o/e
Original data
Data without zeros, Q1/3 +− k*IQR, k=2,...,4
Cleaned data

Aplysia_kurodai

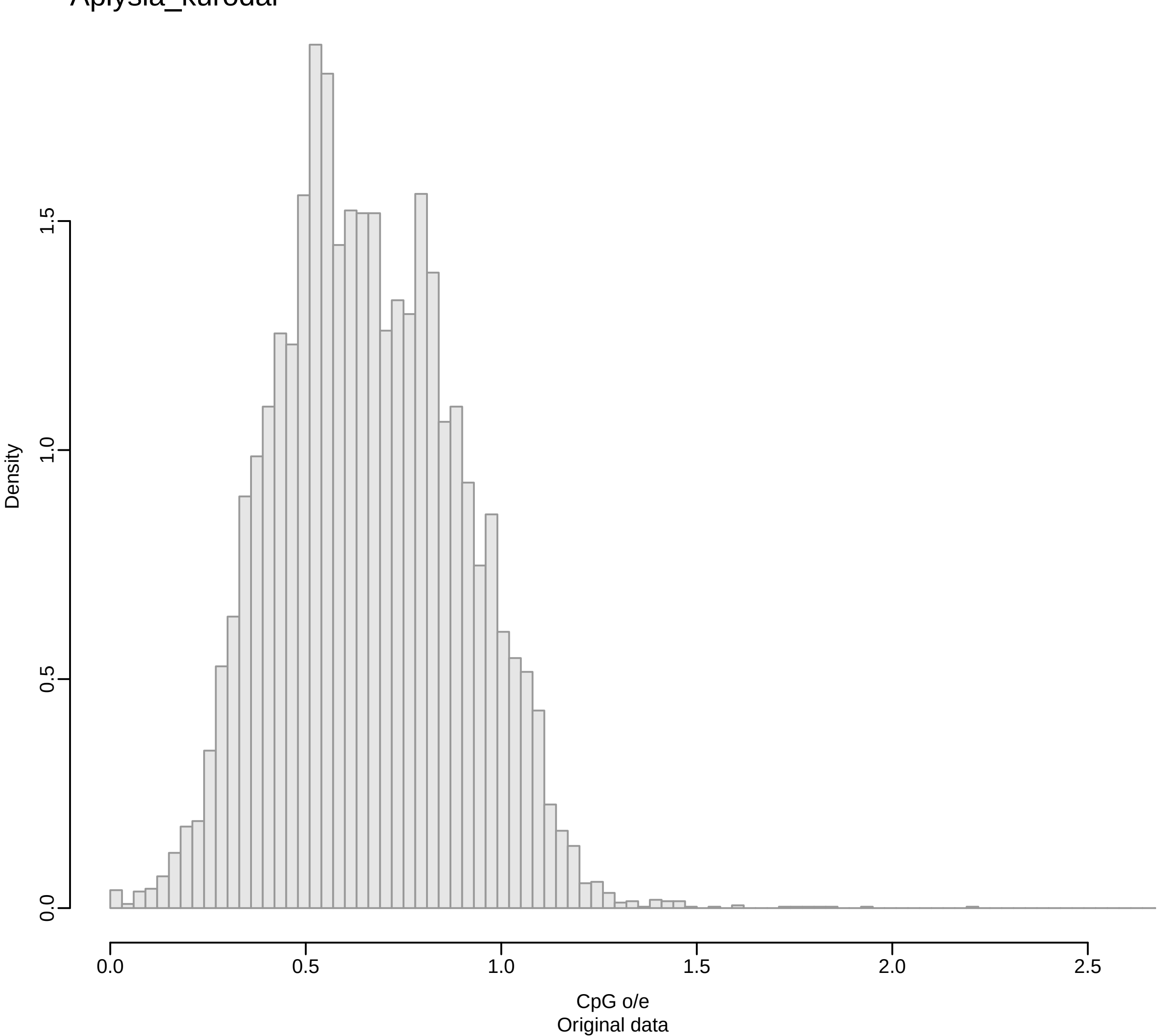

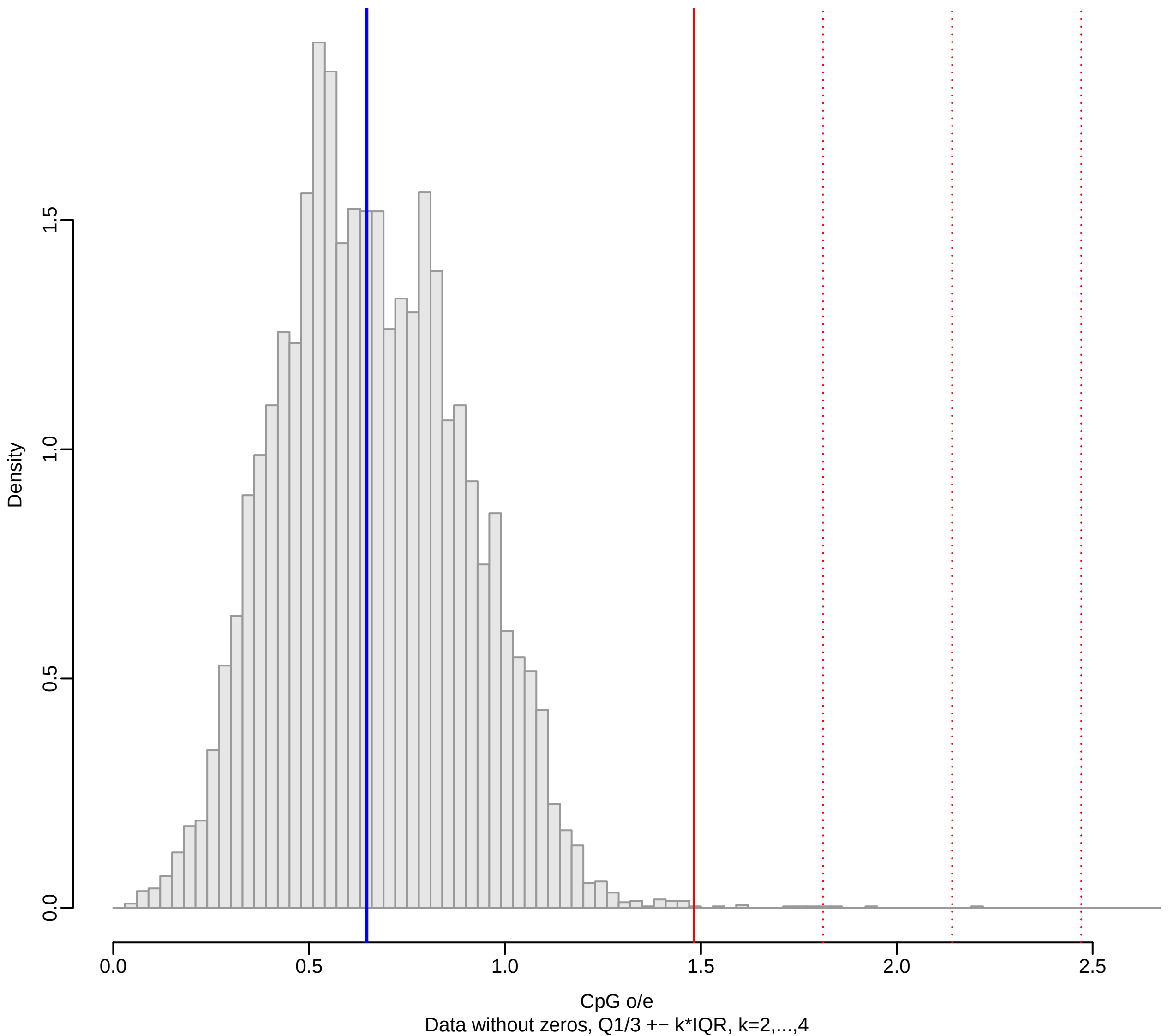

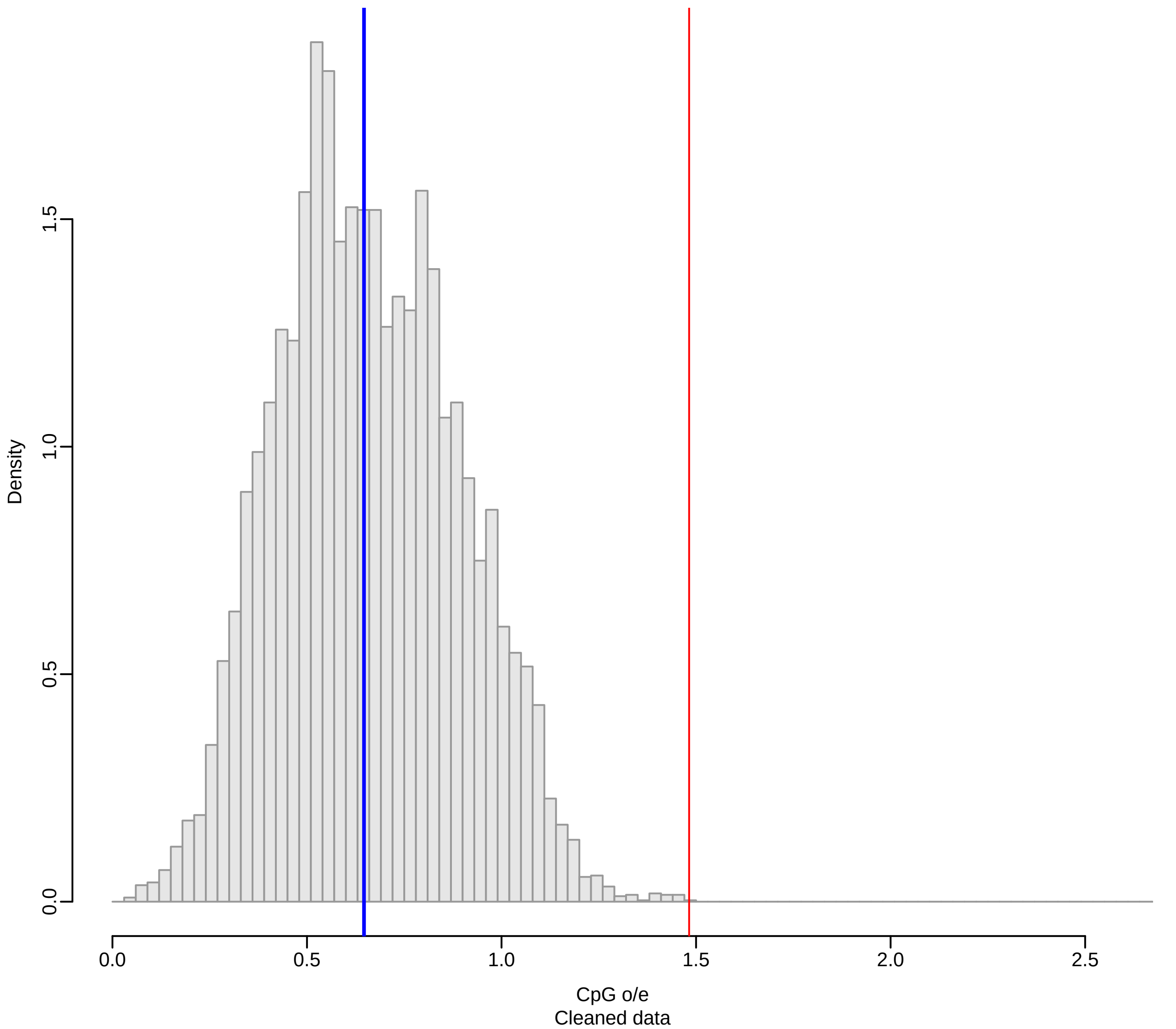

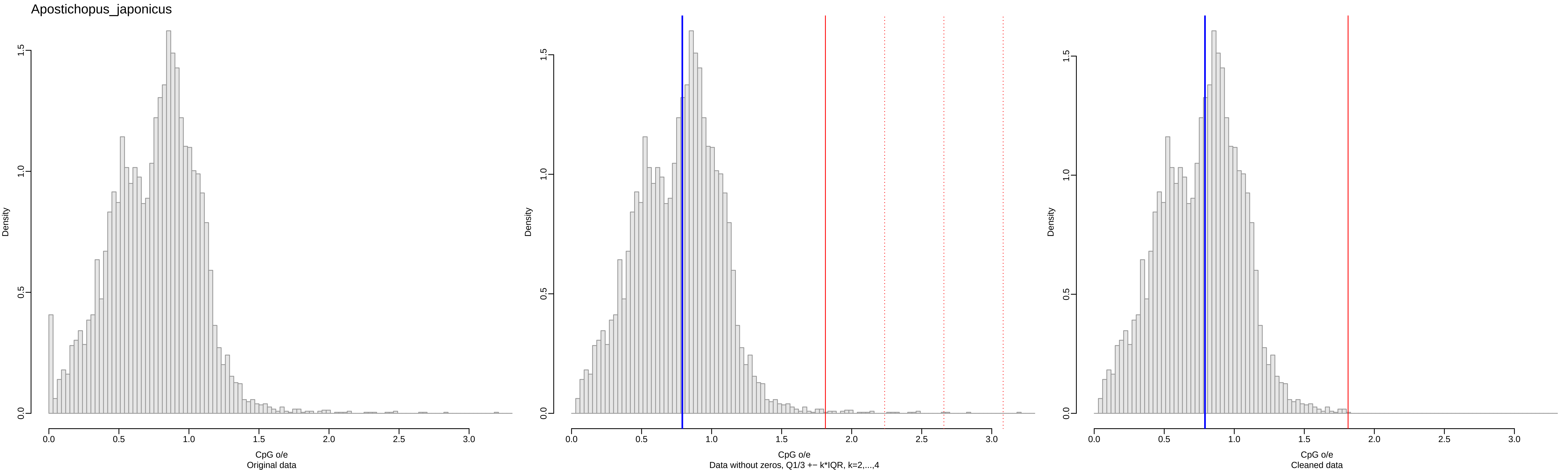

Apostichopus_japonicus
Density
CpG o/e
Original data
CpG o/e
Data without zeros, Q1/3 +− k*IQR, k=2,...,4
CpG o/e
Cleaned data

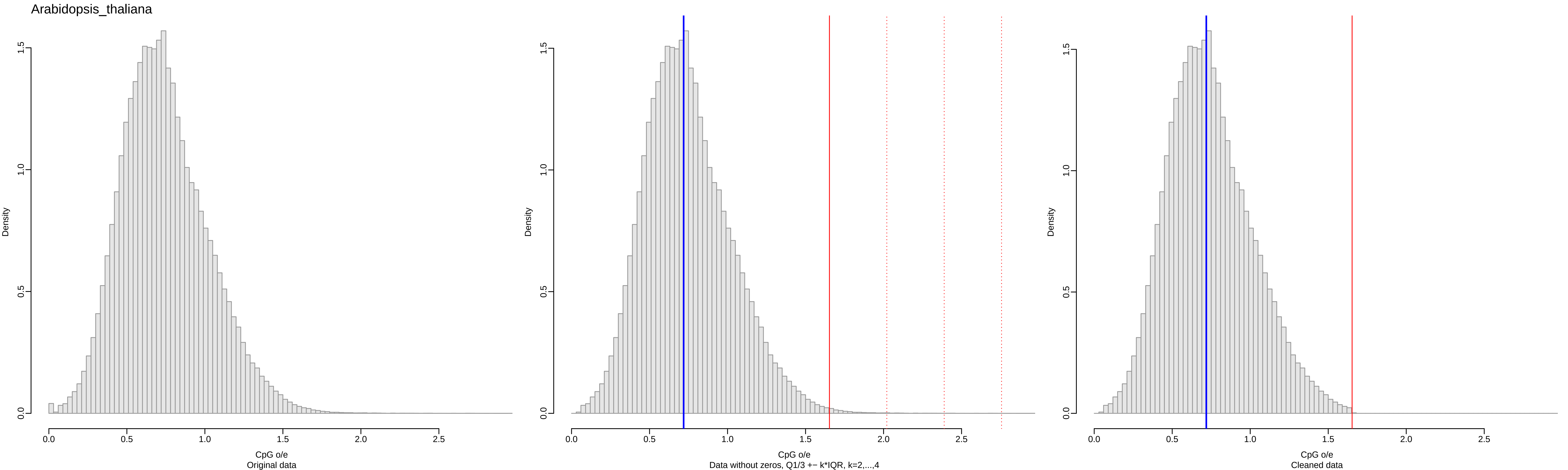

Arabidopsis_thaliana
Density
CpG o/e
Original data
Data without zeros, Q1/3 +− k*IQR, k=2,...,4
Cleaned data
0.0
0.5
1.0
1.5
2.0
2.5

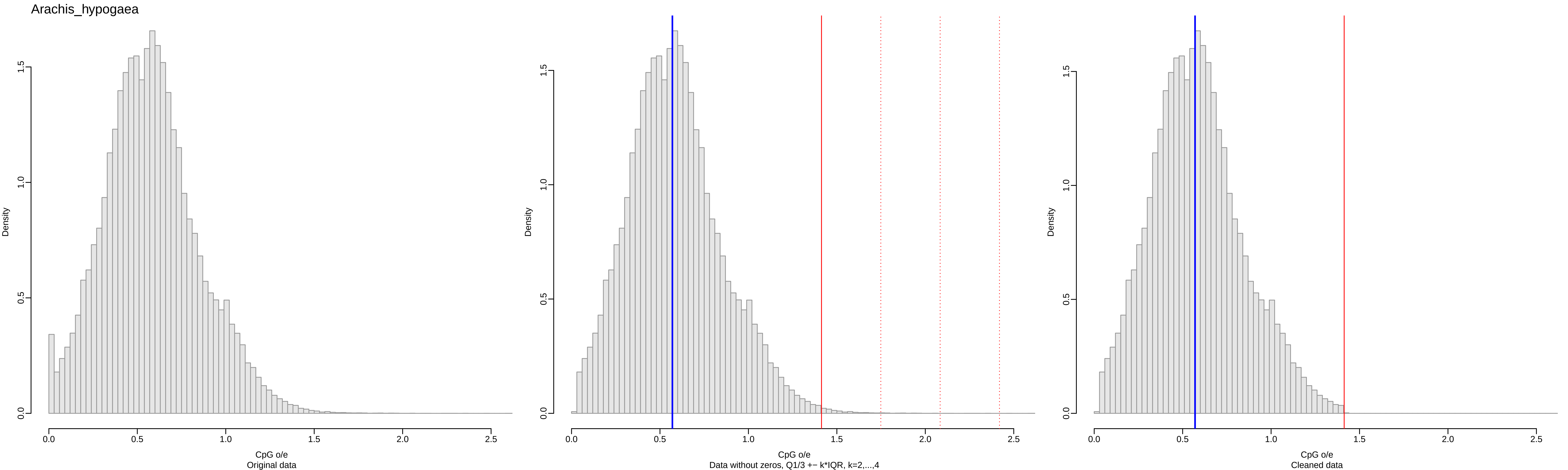

Arachis_hypogaea
Density
CpG o/e
Original data
CpG o/e
Data without zeros, Q1/3 +− k*IQR, k=2,...,4
CpG o/e
Cleaned data

Archispirostreptus_gigas

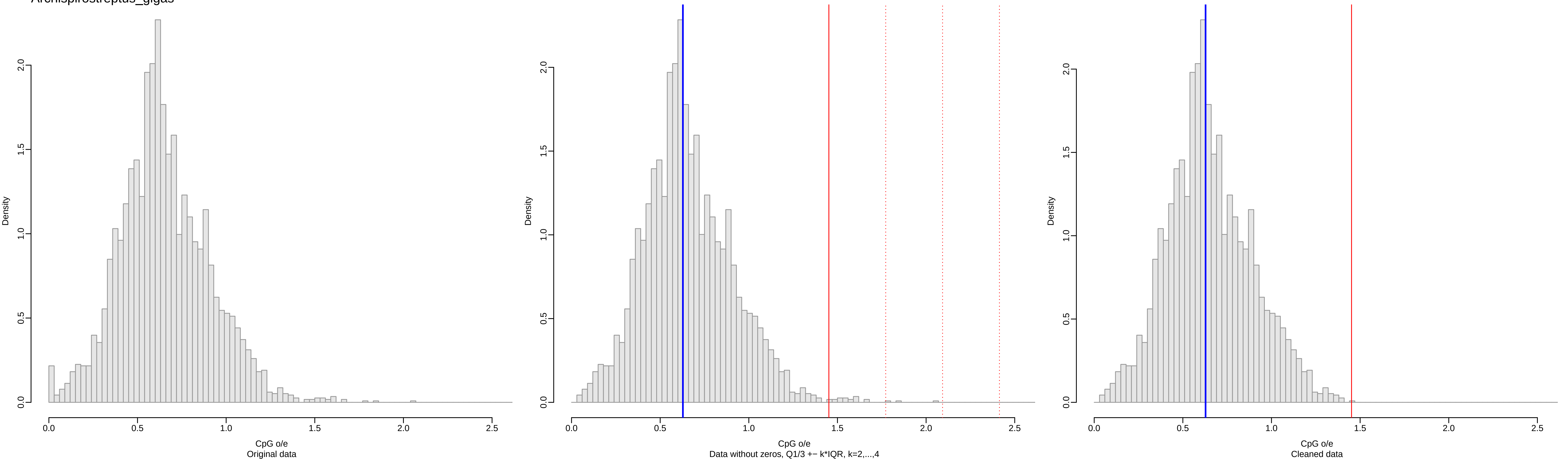

Arenicola_marina

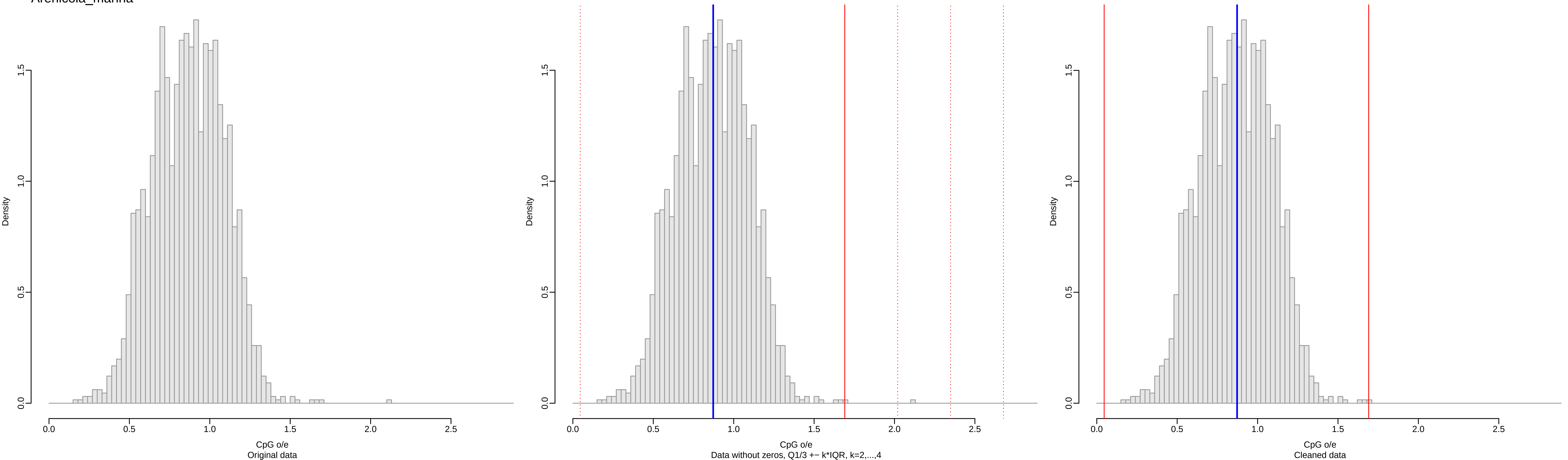

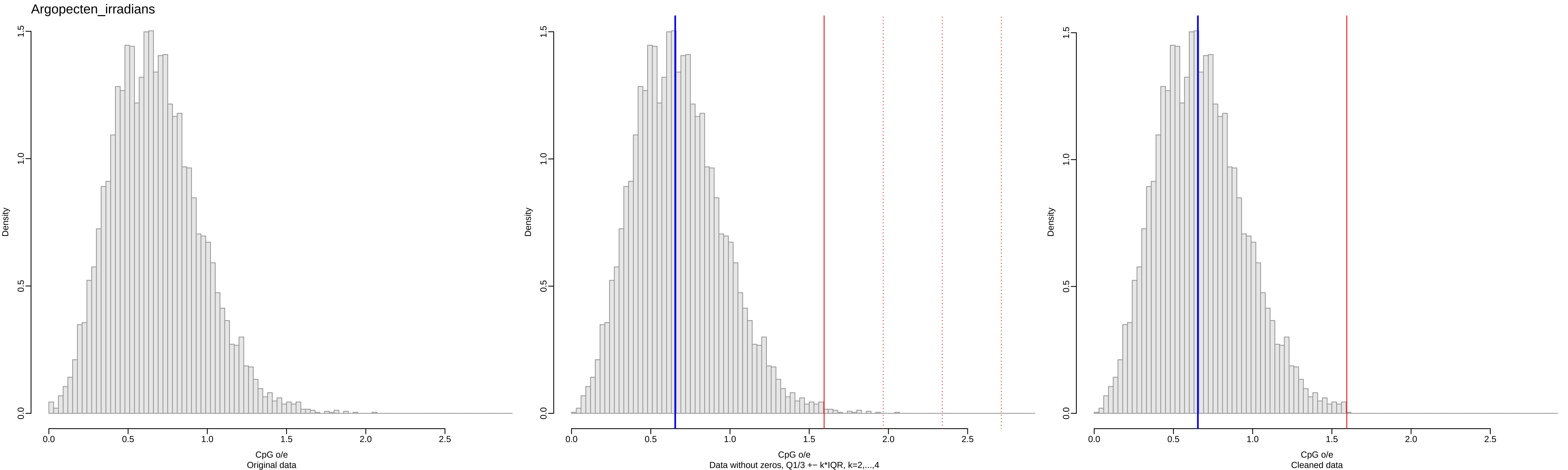

Argopecten_irradians
Density
CpG o/e
Original data
CpG o/e
Data without zeros, Q1/3 +− k*IQR, k=2,...,4
CpG o/e
Cleaned data

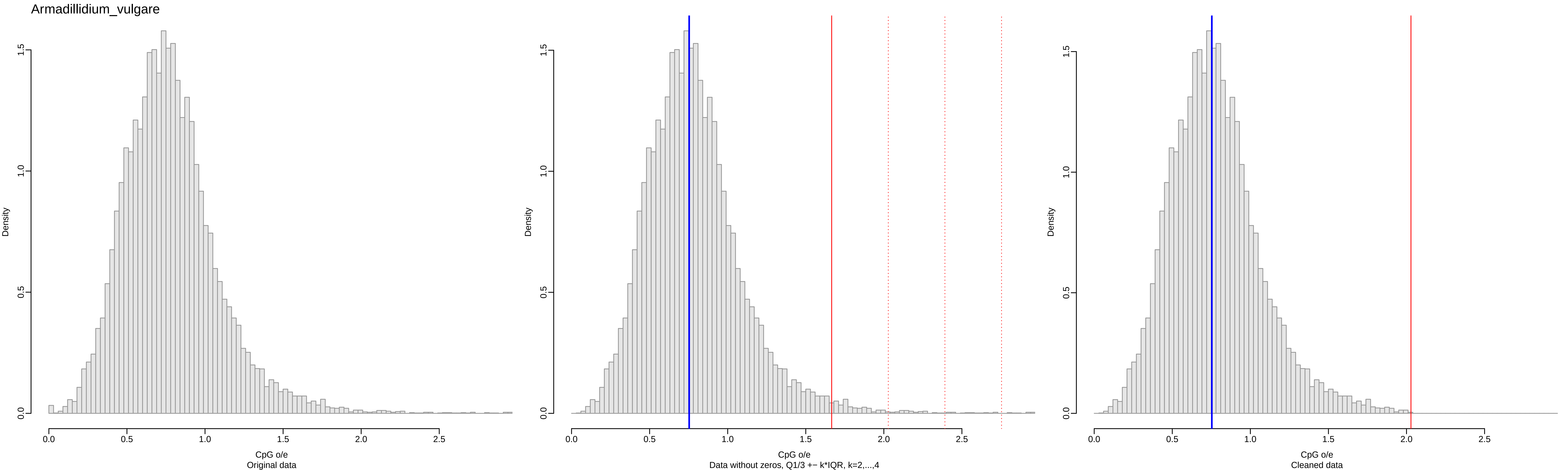

Armadillidium_vulgare
Density
CpG o/e
Original data
Data without zeros, Q1/3 +− k*IQR, k=2,...,4
Cleaned data

Artemia_franciscana

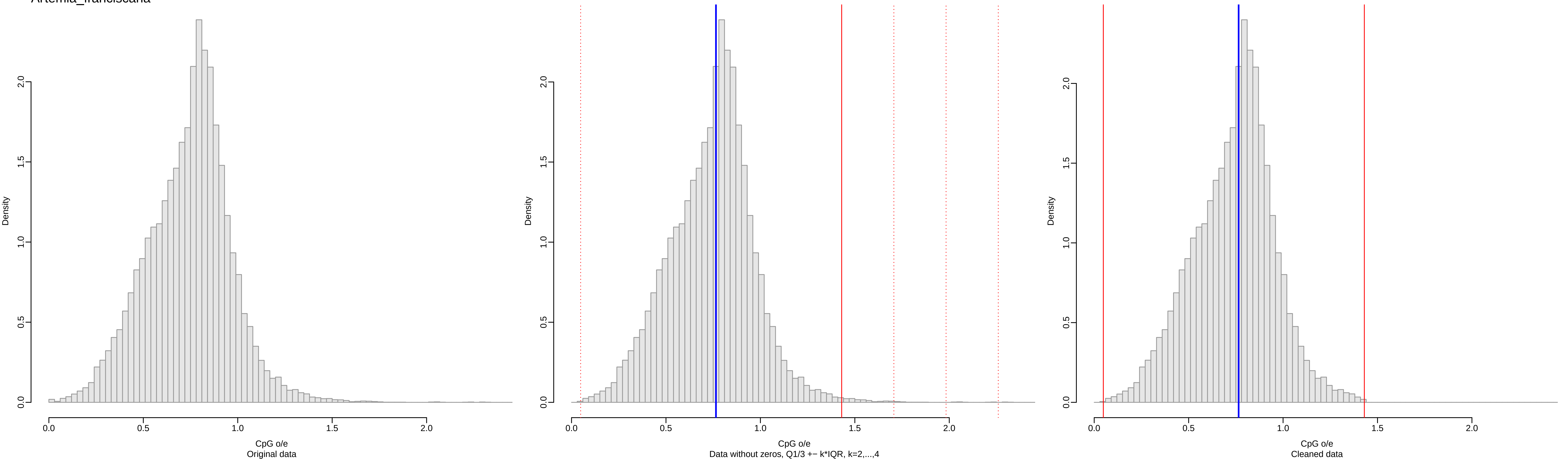

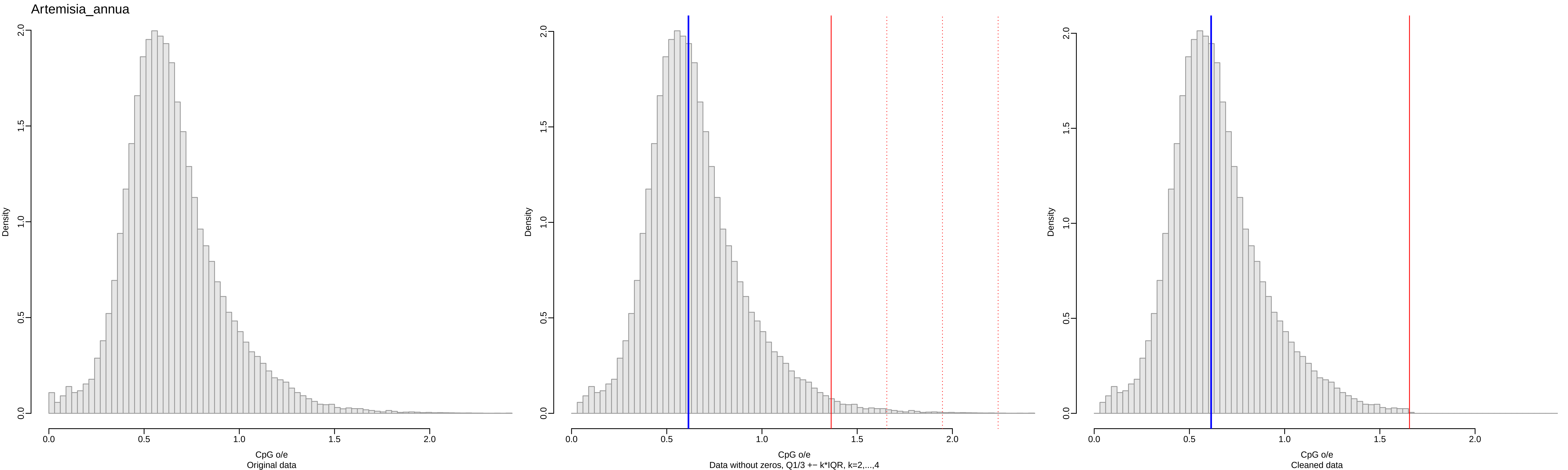

Artemisia_annua
Density
CpG o/e
Original data
Density
CpG o/e
Data without zeros, Q1/3 +− k*IQR, k=2,...,4
Density
CpG o/e
Cleaned data

Asparagus_officinalis

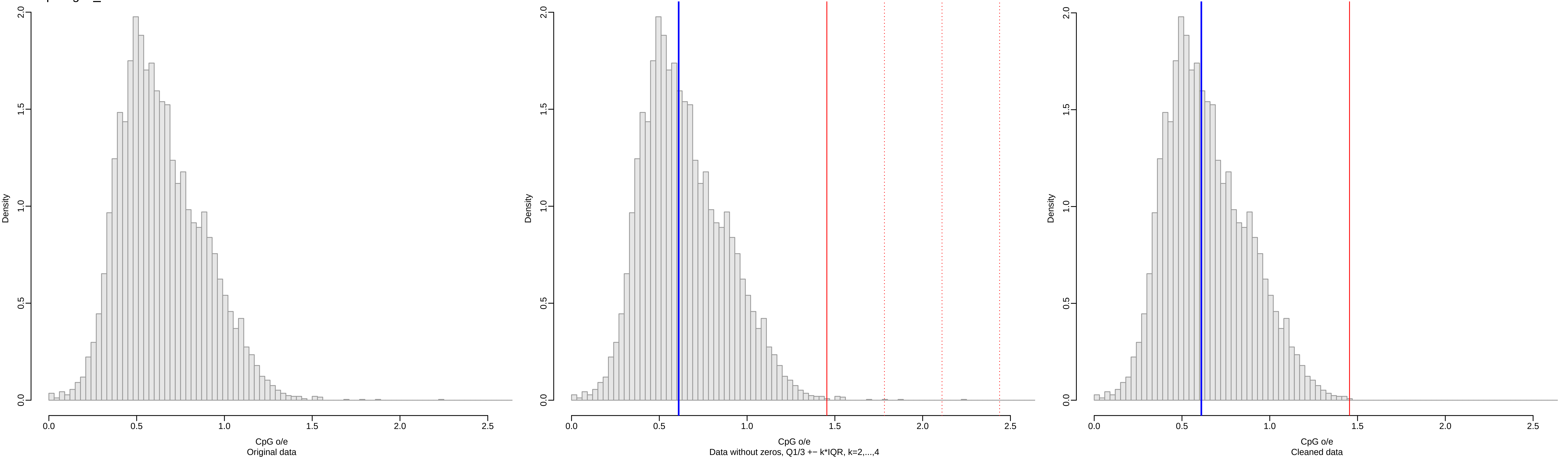

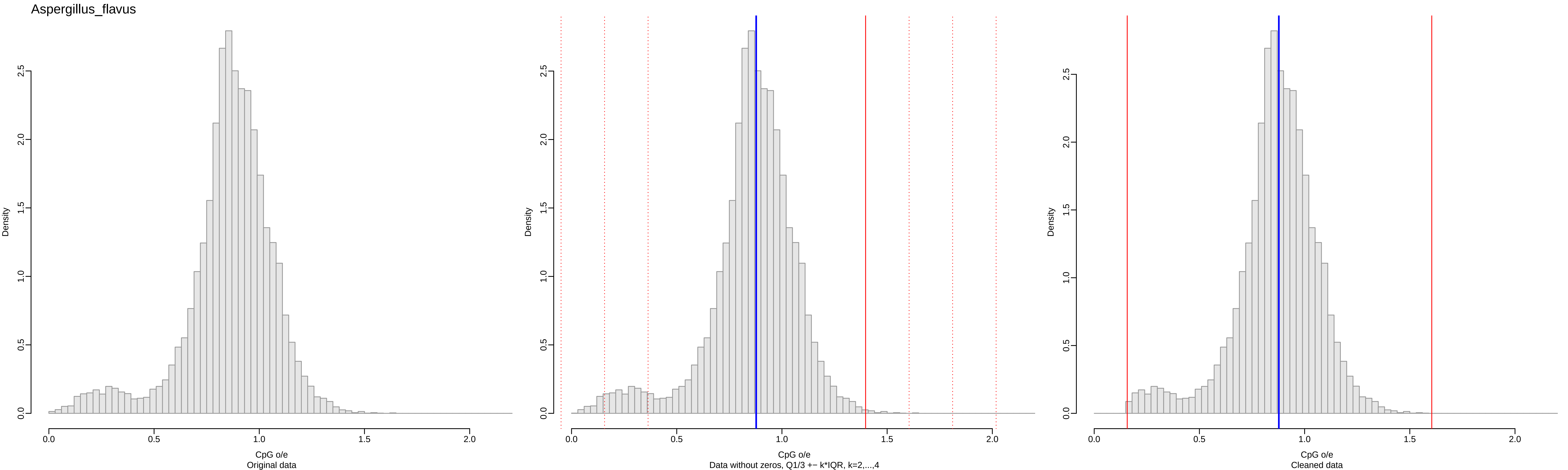

Aspergillus_flavus
Density
CpG o/e
Original data
Data without zeros, Q1/3 +− k*IQR, k=2,...,4
Cleaned data

Aspergillus_niger

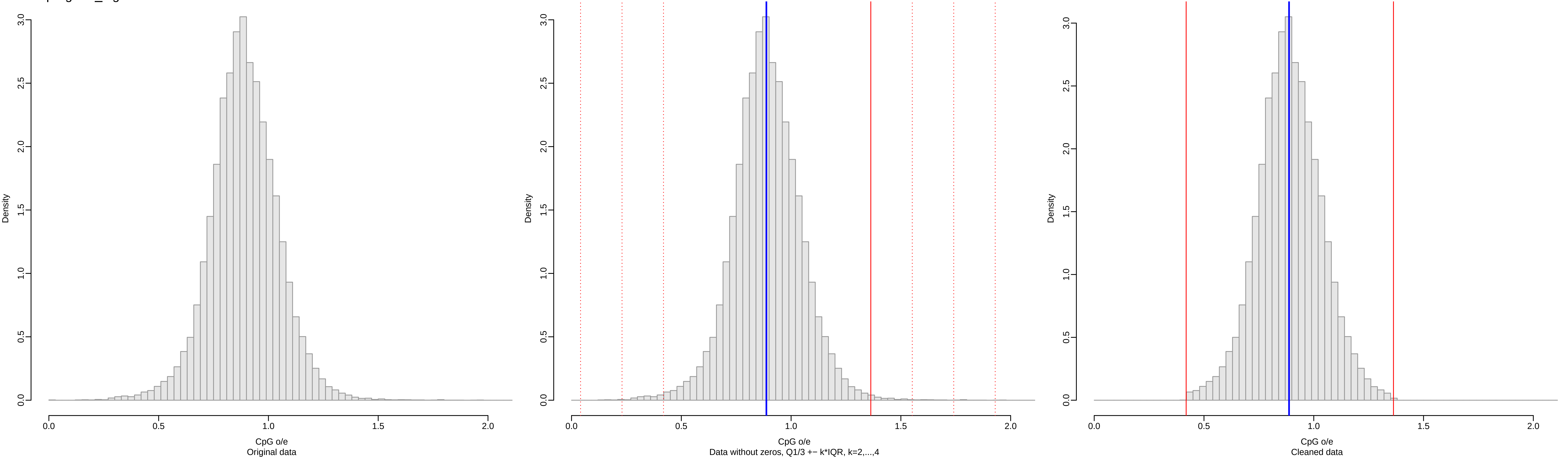

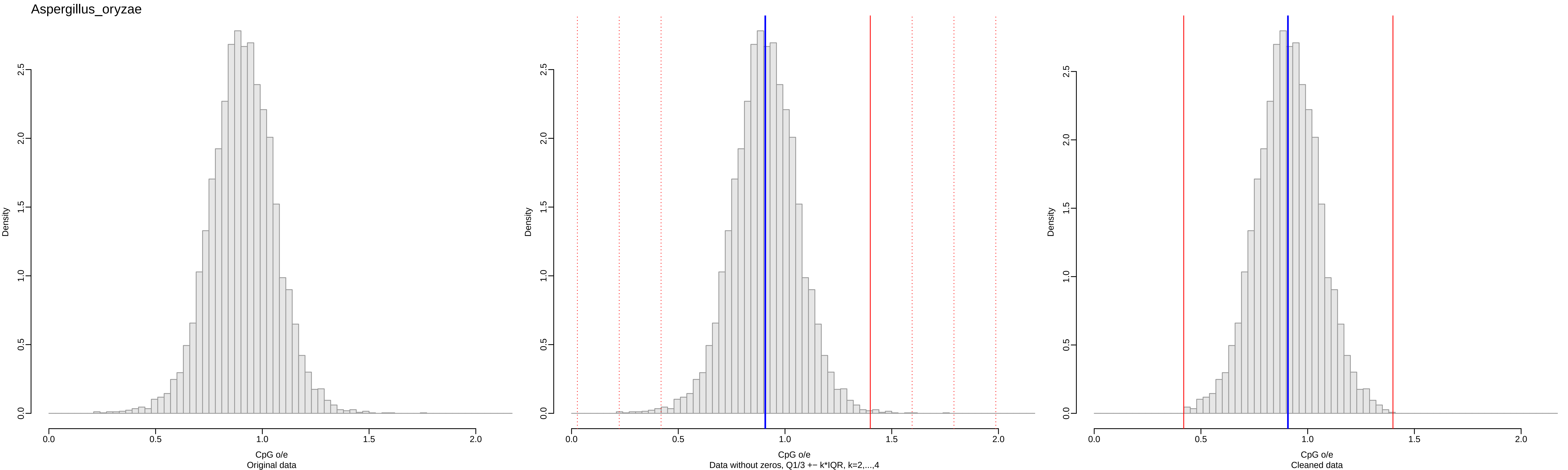

Aspergillus_oryzae
Density
CpG o/e
Original data
Density
CpG o/e
Data without zeros, Q1/3 +− k*IQR, k=2,...,4
Density
CpG o/e
Cleaned data

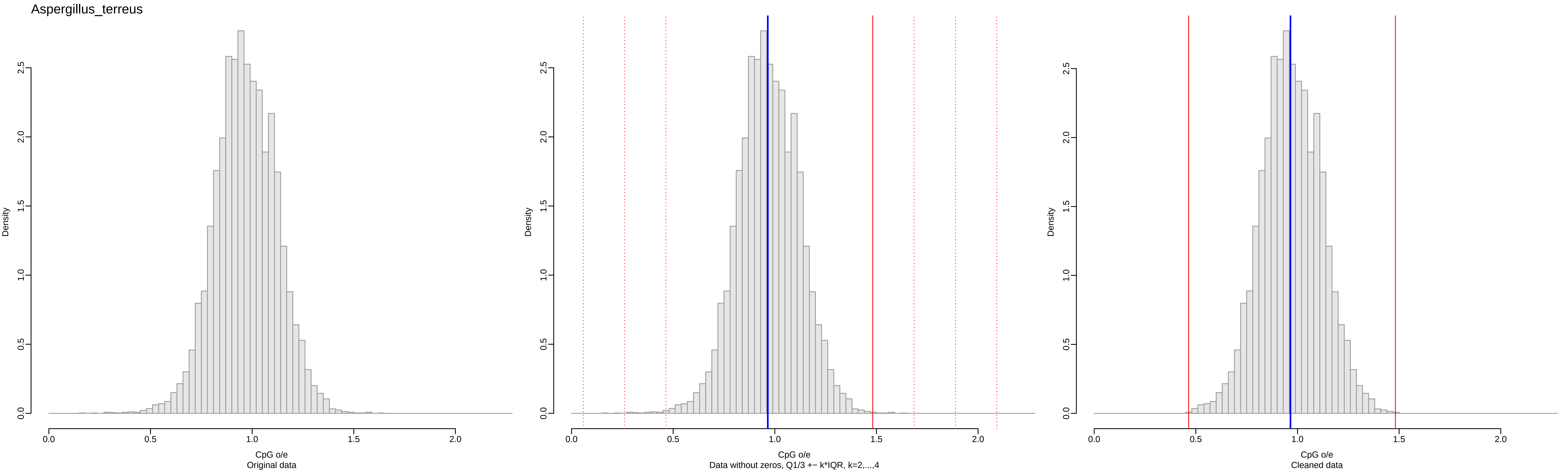
Aspergillus_terreus
Density
CpG o/e
Original data
Data without zeros, Q1/3 +− k*IQR, k=2,...,4
Cleaned data
0.0
0.5
1.0
1.5
2.0
2.5

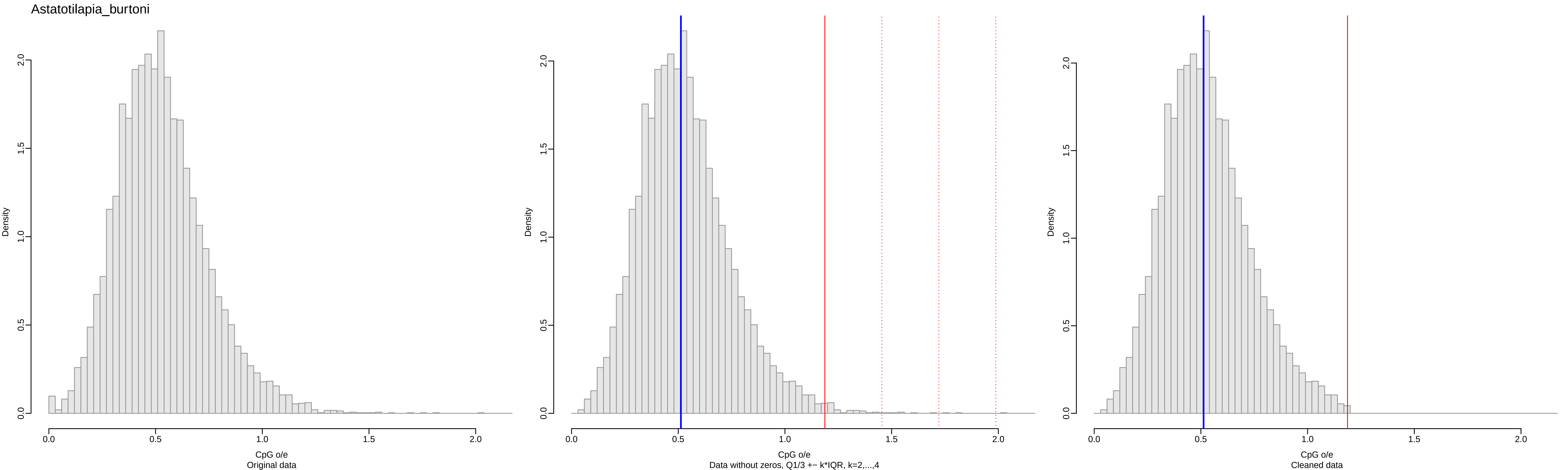
Astatotilapia_burtoni
Density
CpG o/e
Original data
Data without zeros, Q1/3 +− k*IQR, k=2,...,4
Cleaned data
0.0
0.5
1.0
1.5
2.0

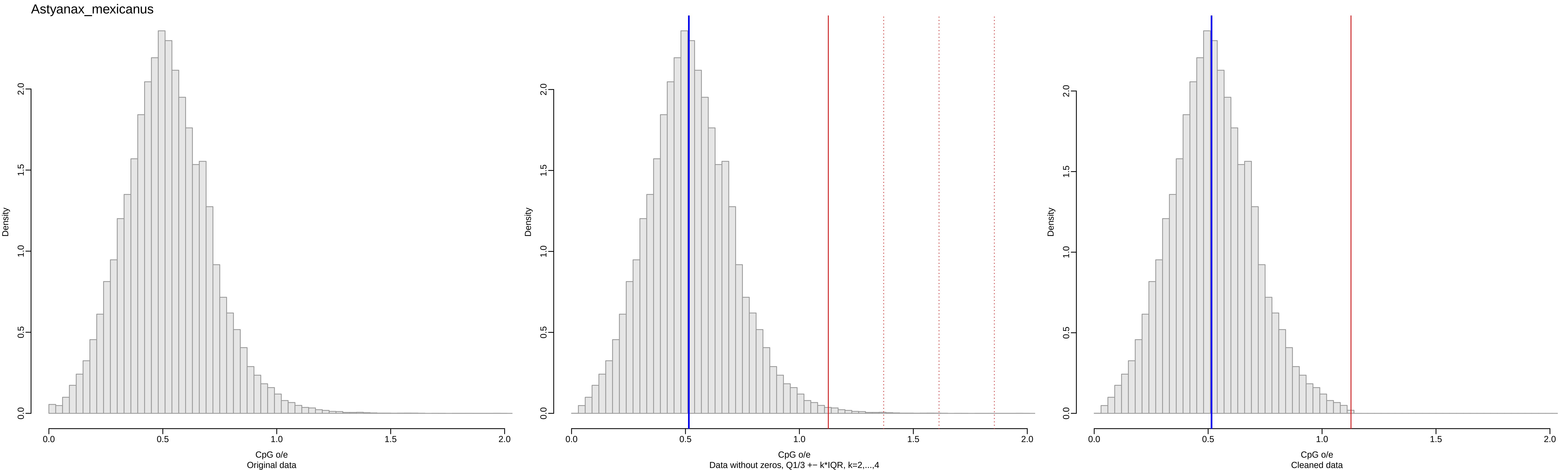

Astyanax_mexicanus
Density
0.0
0.5
1.0
1.5
2.0
CpG o/e
Original data
Data without zeros, Q1/3 +− k*IQR, k=2,...,4
Cleaned data

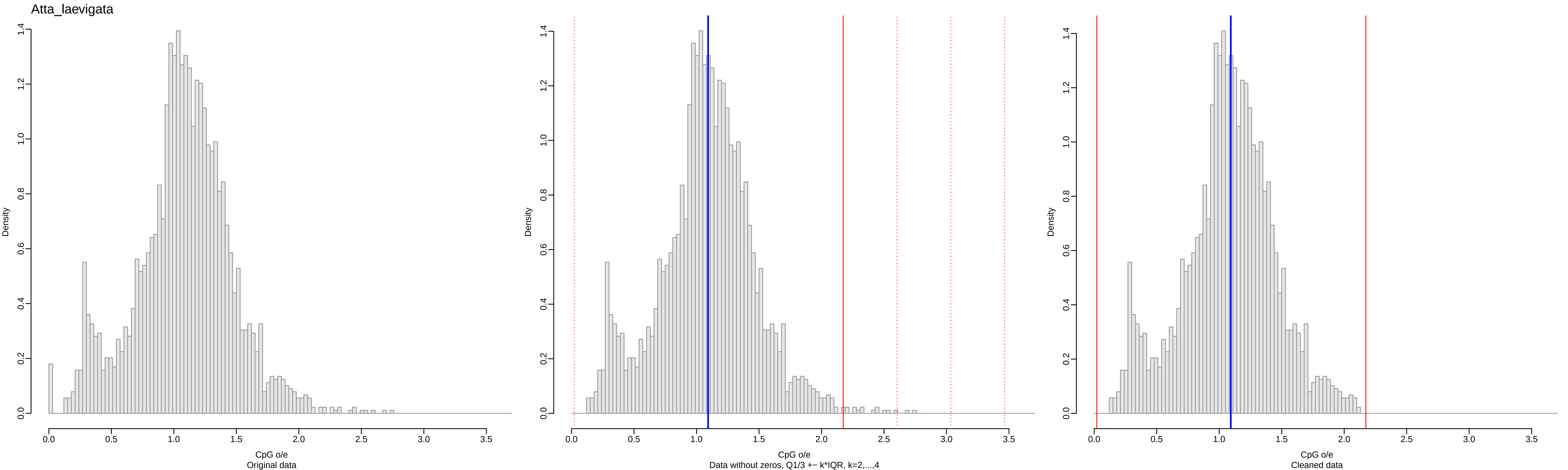
Atta_laevigata
Density
CpG o/e
Original data
Data without zeros, Q1/3 +− k*IQR, k=2,...,4
Cleaned data

Aureobasidium_pullulans

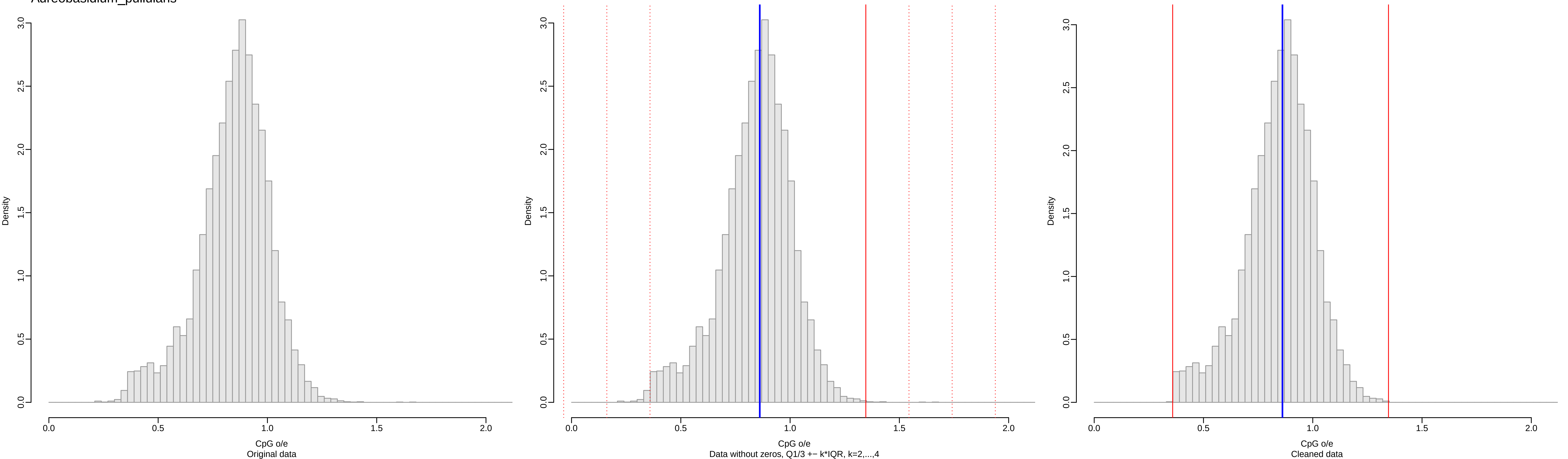

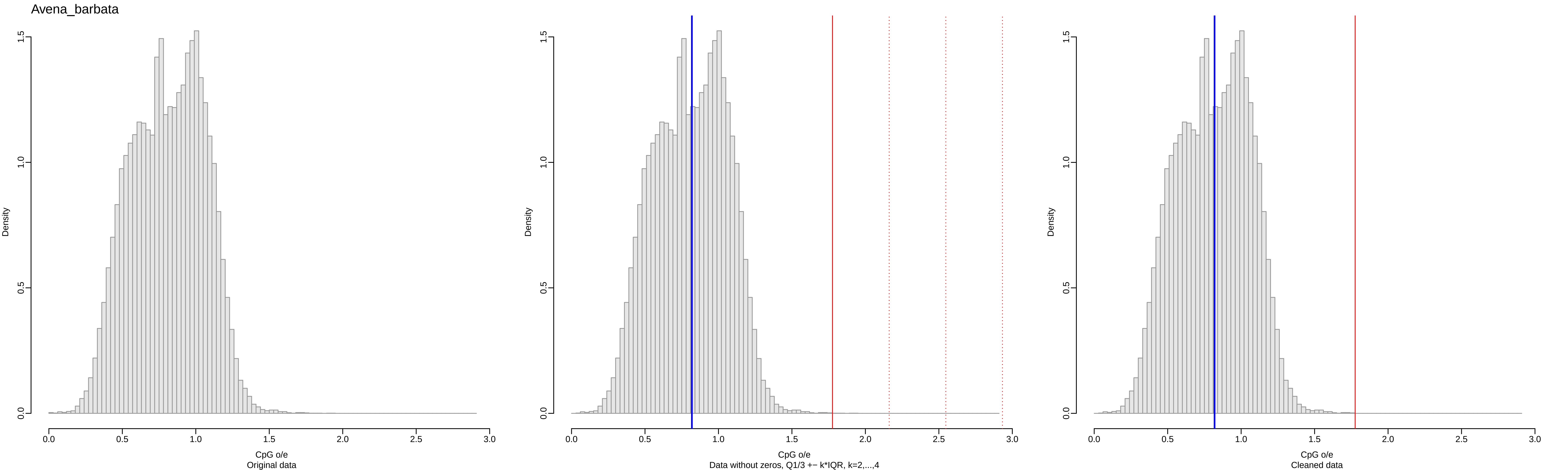
Avena_barbata
Density
CpG o/e
Original data
Data without zeros, Q1/3 +− k*IQR, k=2,...,4
Cleaned data
0.0
0.5
1.0
1.5
2.0
2.5
3.0

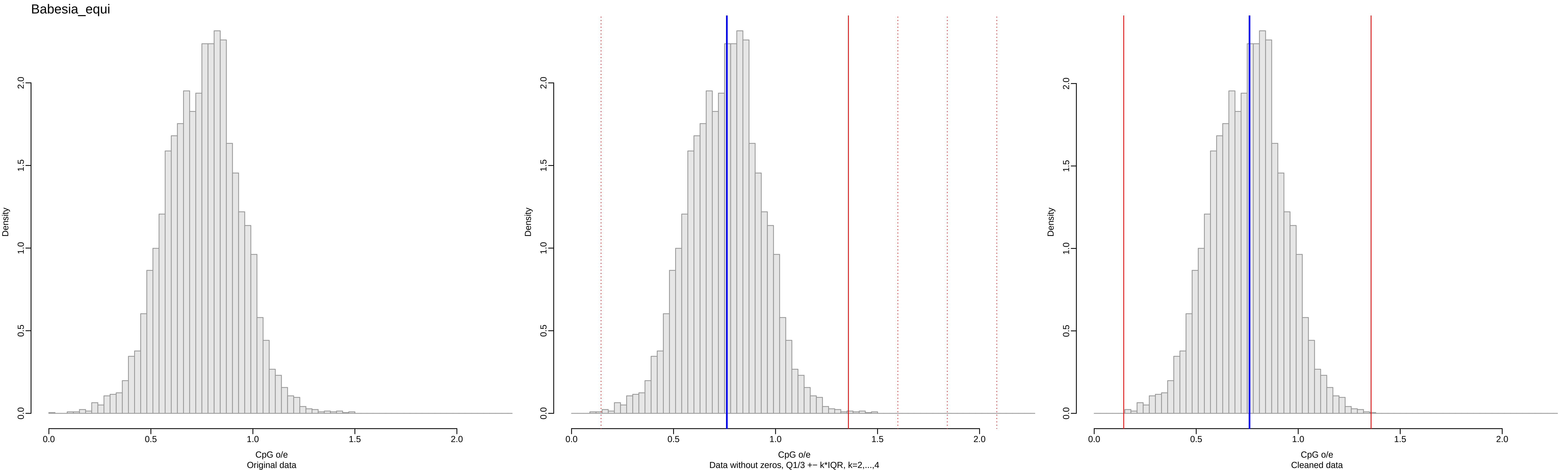
Babesia_equi
Density
CpG o/e
Original data
Data without zeros, Q1/3 +− k*IQR, k=2,...,4
Cleaned data
0.0
0.5
1.0
1.5
2.0

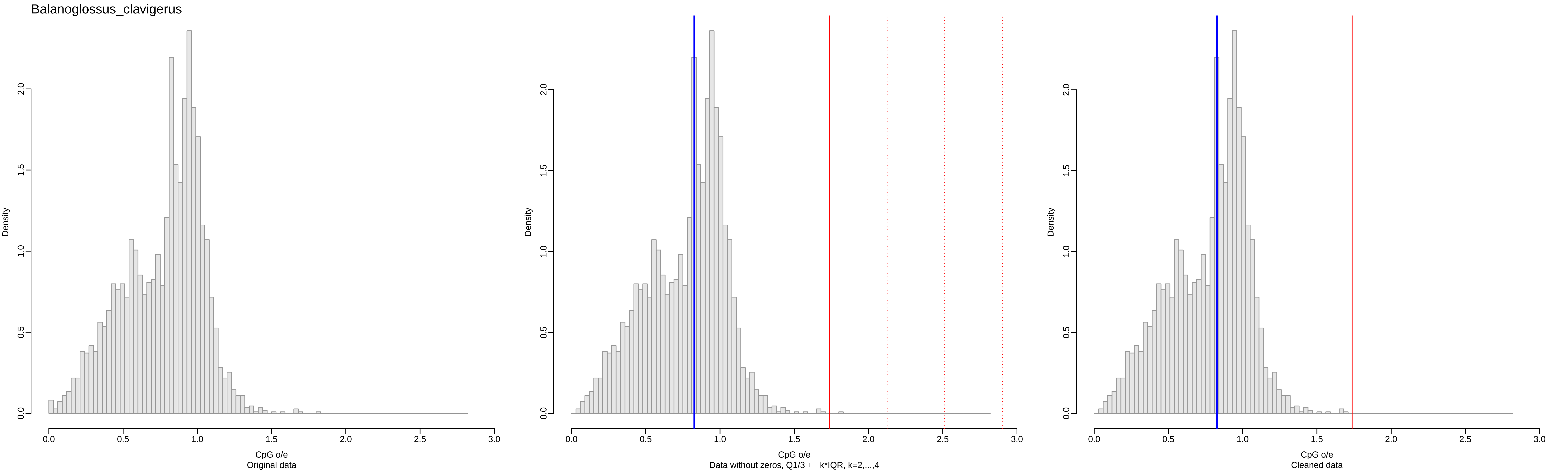

Balanoglossus_clavigerus
Density
CpG o/e
Original data
Density
CpG o/e
Data without zeros, Q1/3 +− k*IQR, k=2,...,4
Density
CpG o/e
Cleaned data

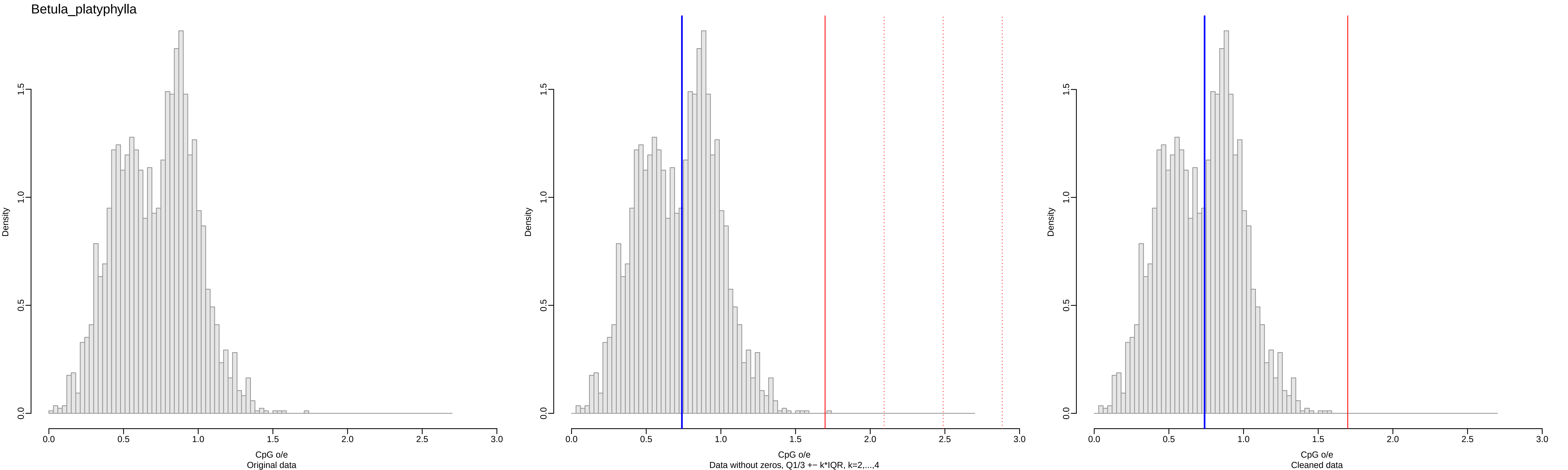
Betula_platyphylla
Density
CpG o/e
Original data
Data without zeros, Q1/3 +− k*IQR, k=2,...,4
Cleaned data
0.0
0.5
1.0
1.5
2.0
2.5
3.0

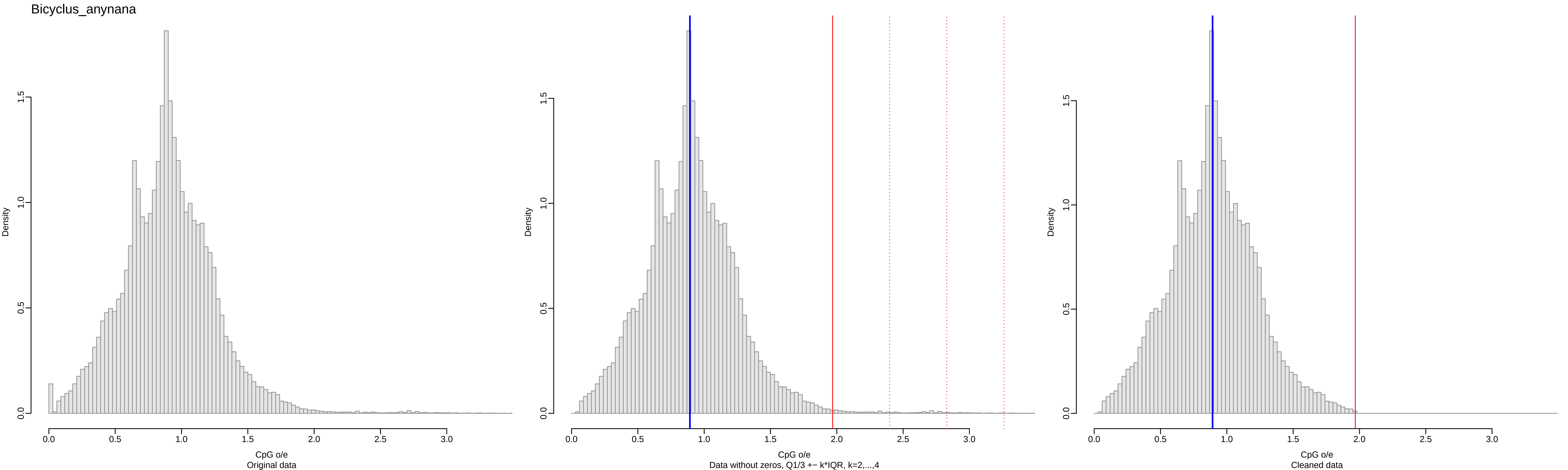

Bicyclus_anynana
Density
CpG o/e
Original data
Data without zeros, Q1/3 +− k*IQR, k=2,...,4
Cleaned data
0.0
0.5
1.0
1.5
2.0
2.5
3.0

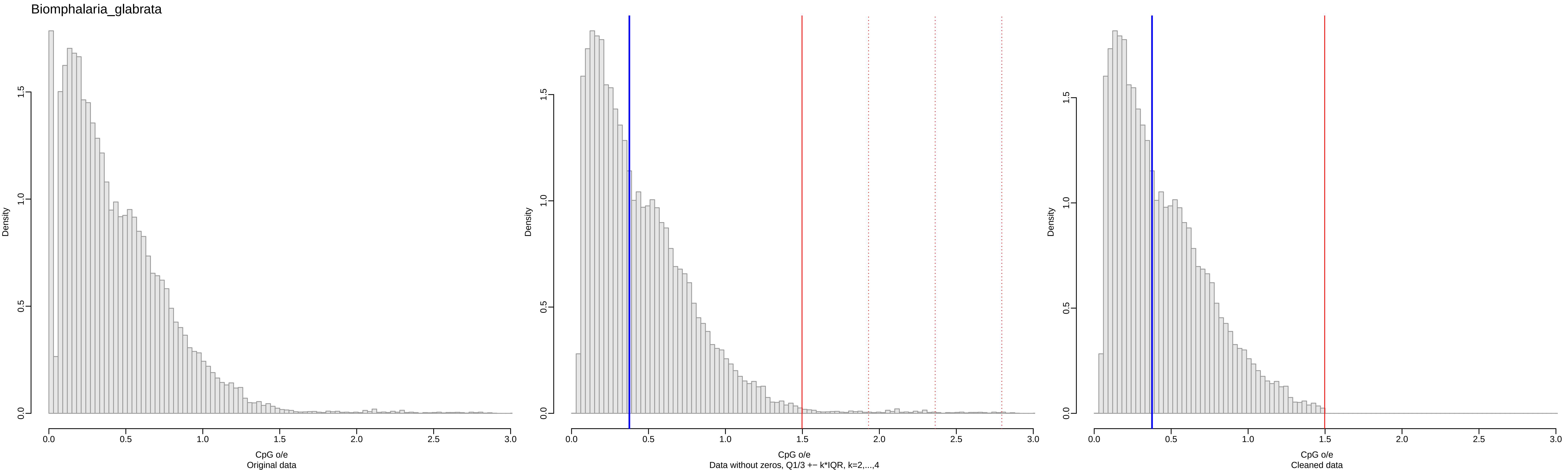
Biomphalaria_glabrata
Density
CpG o/e
Original data
CpG o/e
Data without zeros, Q1/3 +− k*IQR, k=2,...,4
CpG o/e
Cleaned data

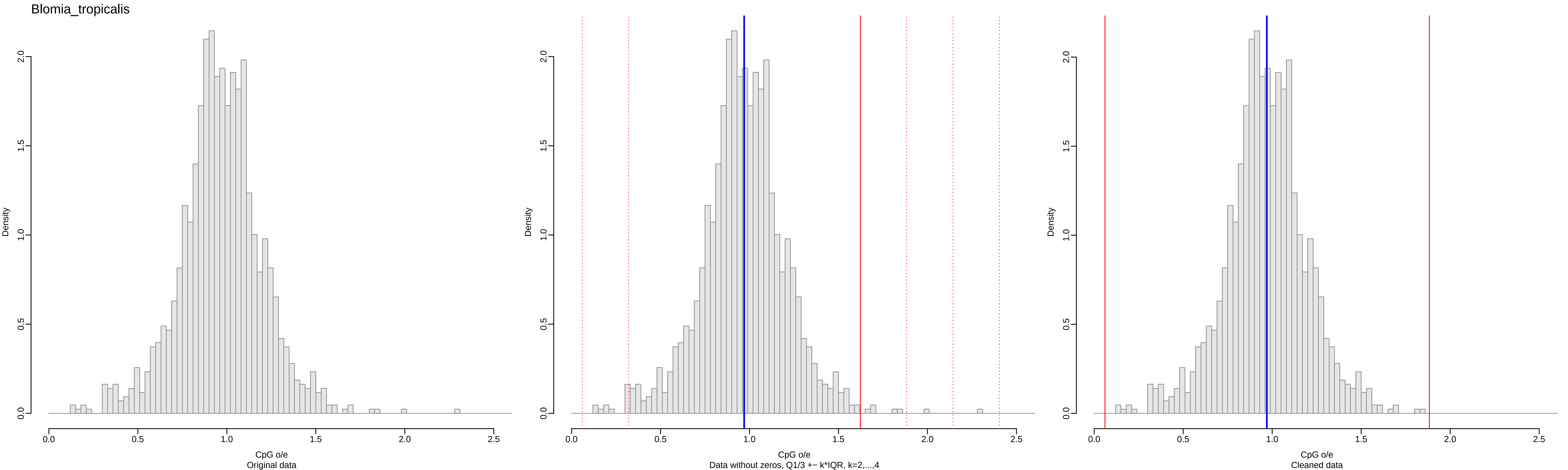
Blomia_tropicalis
Density
CpG o/e
Original data
Data without zeros, Q1/3 +− k*IQR, k=2,...,4
Cleaned data
0.0
0.5
1.0
1.5
2.0
2.5

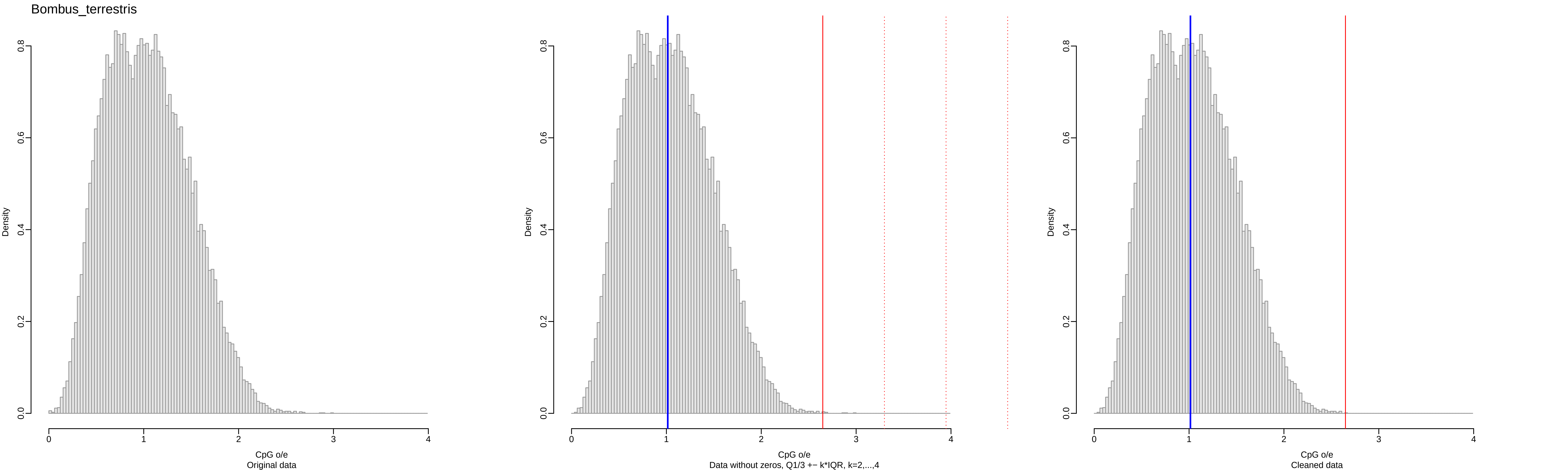
Bombus_terrestris
Density
CpG o/e
Original data
Density
CpG o/e
Data without zeros, Q1/3 +− k*IQR, k=2,...,4
Density
CpG o/e
Cleaned data

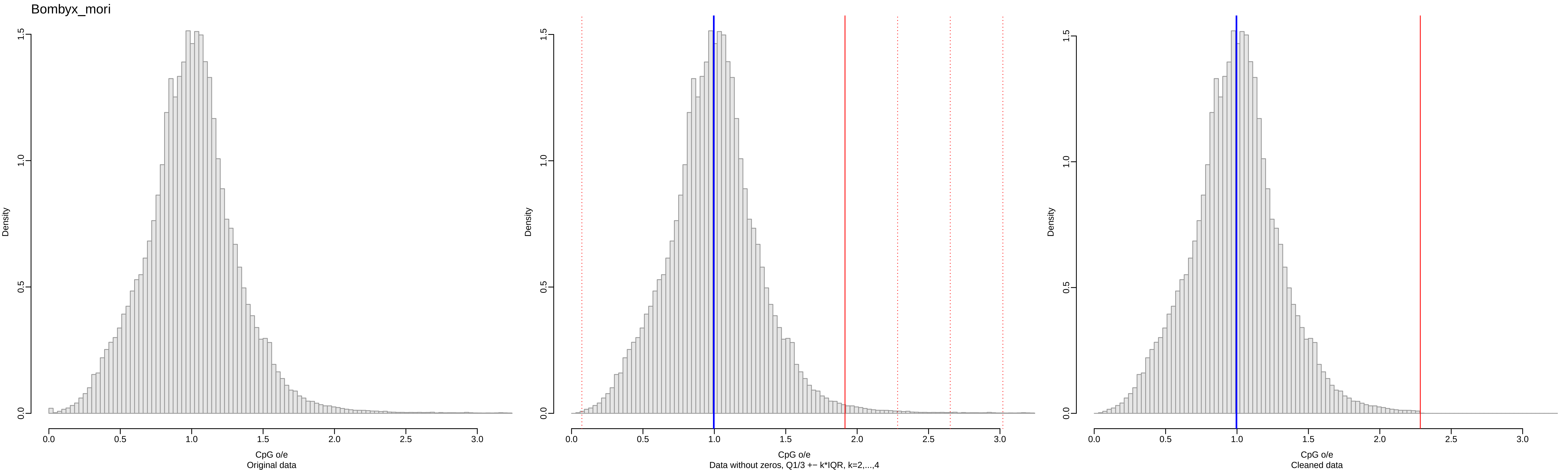

Bombyx_mori
Density
CpG o/e
Original data
Data without zeros, Q1/3 +− k*IQR, k=2,...,4
Cleaned data
0.0
0.5
1.0
1.5
2.0
2.5
3.0

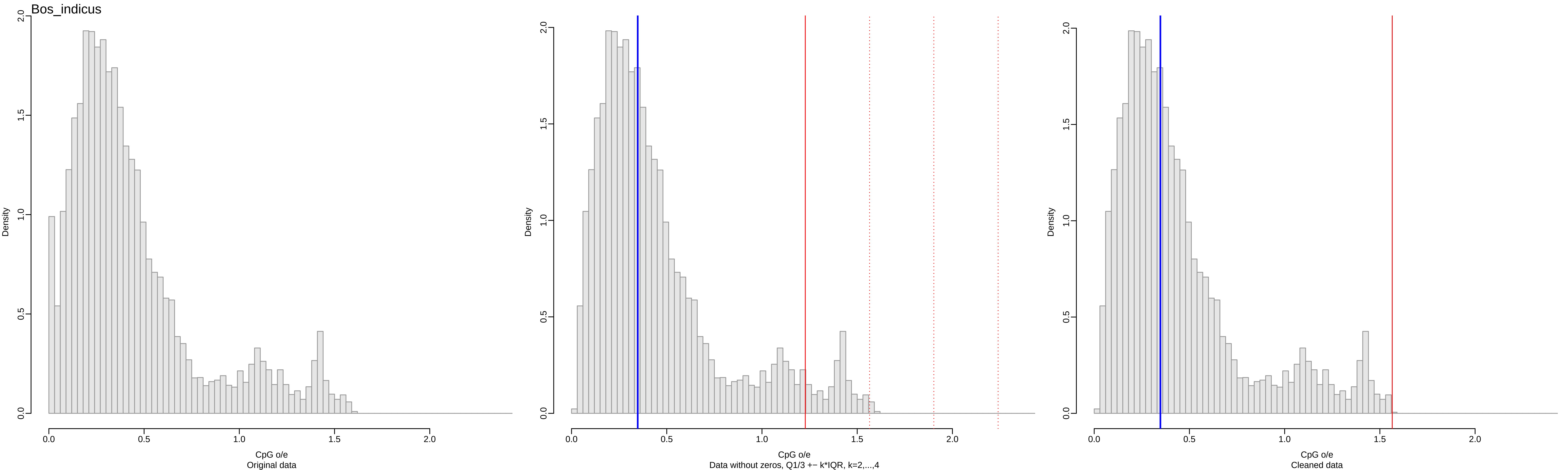
Bos_indicus
Density
CpG o/e
Original data
CpG o/e
Data without zeros, Q1/3 +− k*IQR, k=2,...,4
CpG o/e
Cleaned data
0.0
0.5
1.0
1.5
2.0

Bos_taurus

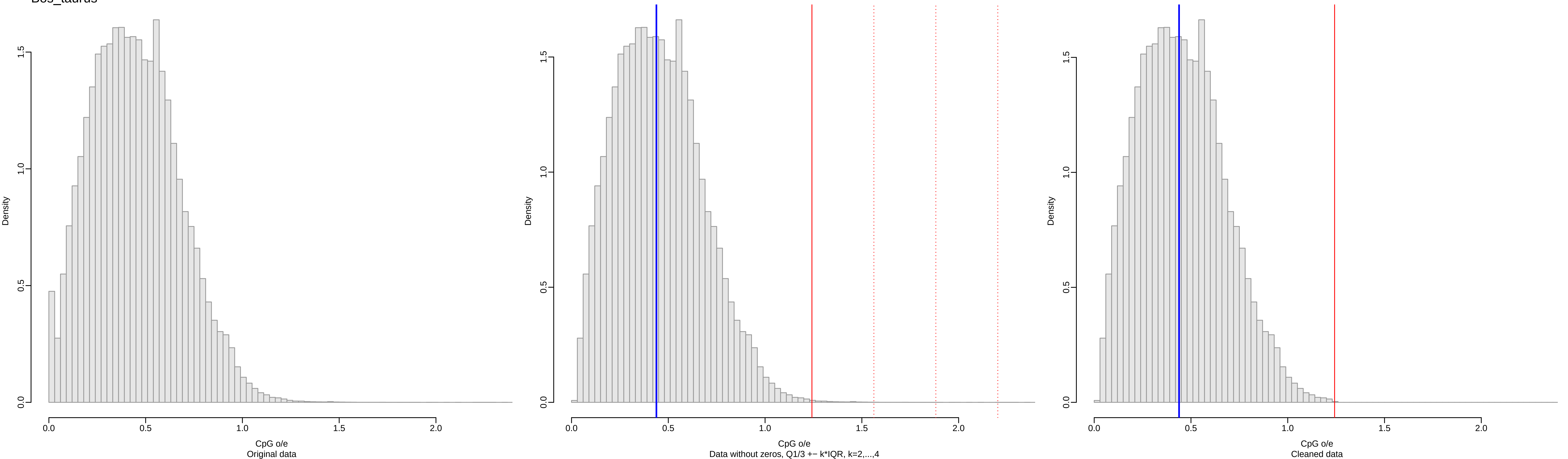

Density
0.0
0.5
1.0
1.5
0.0
0.5
1.0
1.5
2.0
CpG o/e
Original data
Density
CpG o/e
Data without zeros, Q1/3 +− k*IQR, k=2,...,4
Density
CpG o/e
Cleaned data

Bothrops_alternatus

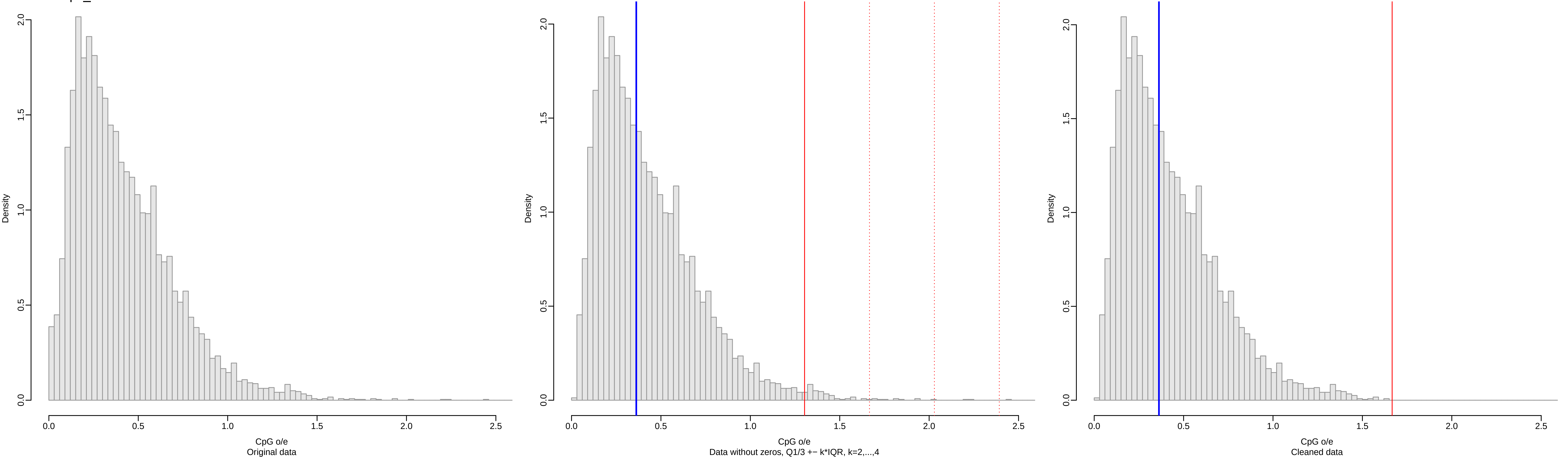

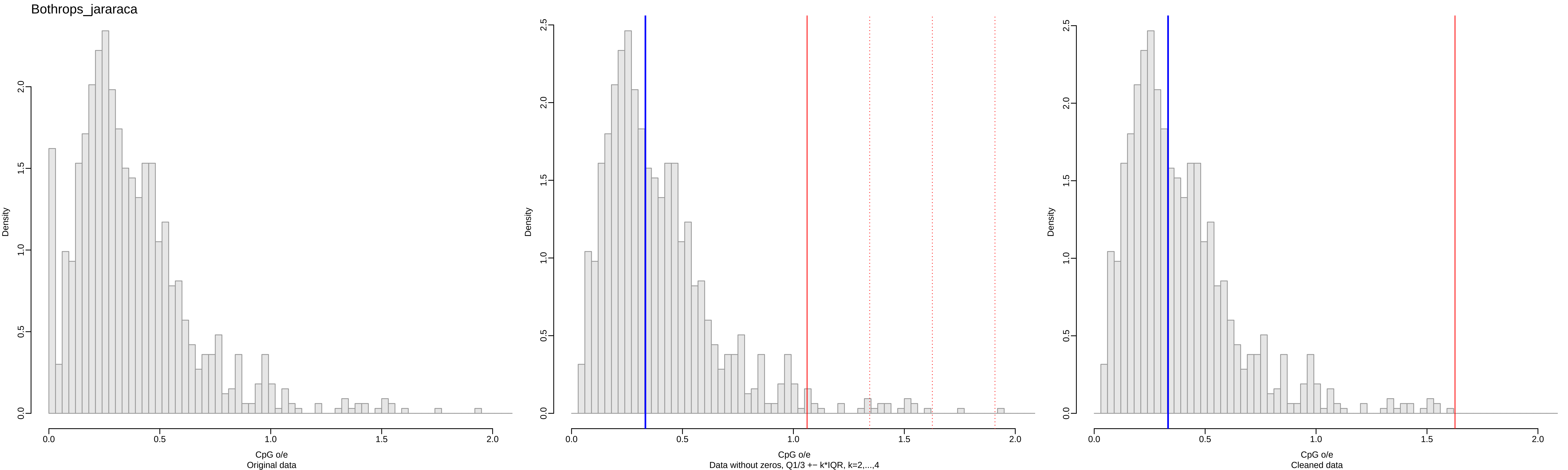

Bothrops_jararaca
Density
CpG o/e
Original data
Data without zeros, Q1/3 +− k*IQR, k=2,...,4
Cleaned data

Botryllus_schlosseri

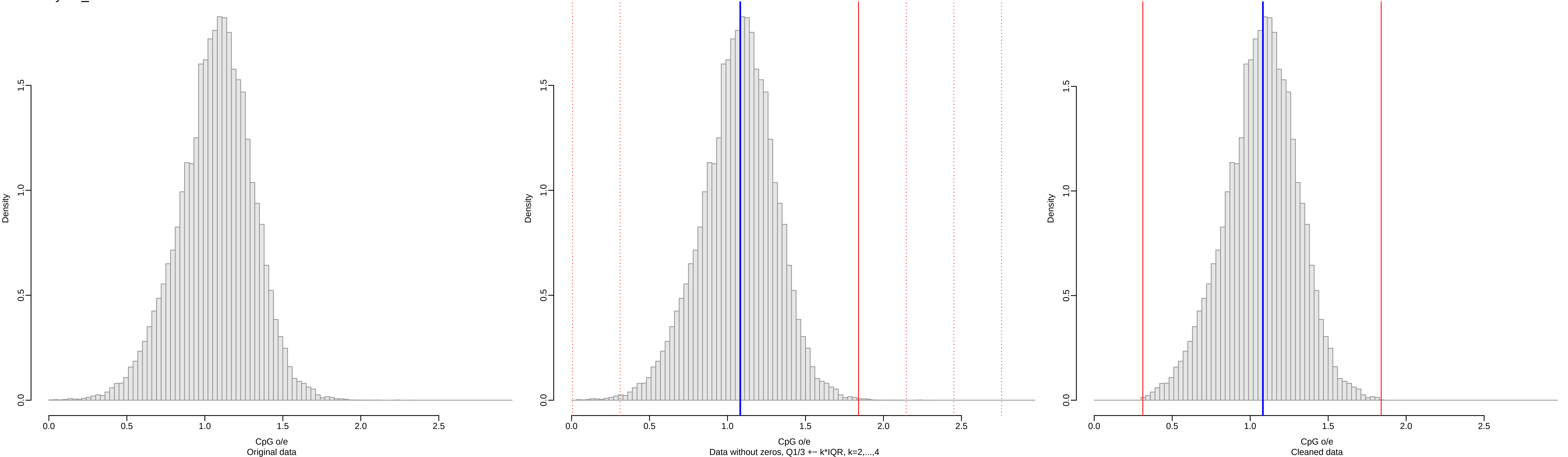

Botryococcus_braunii

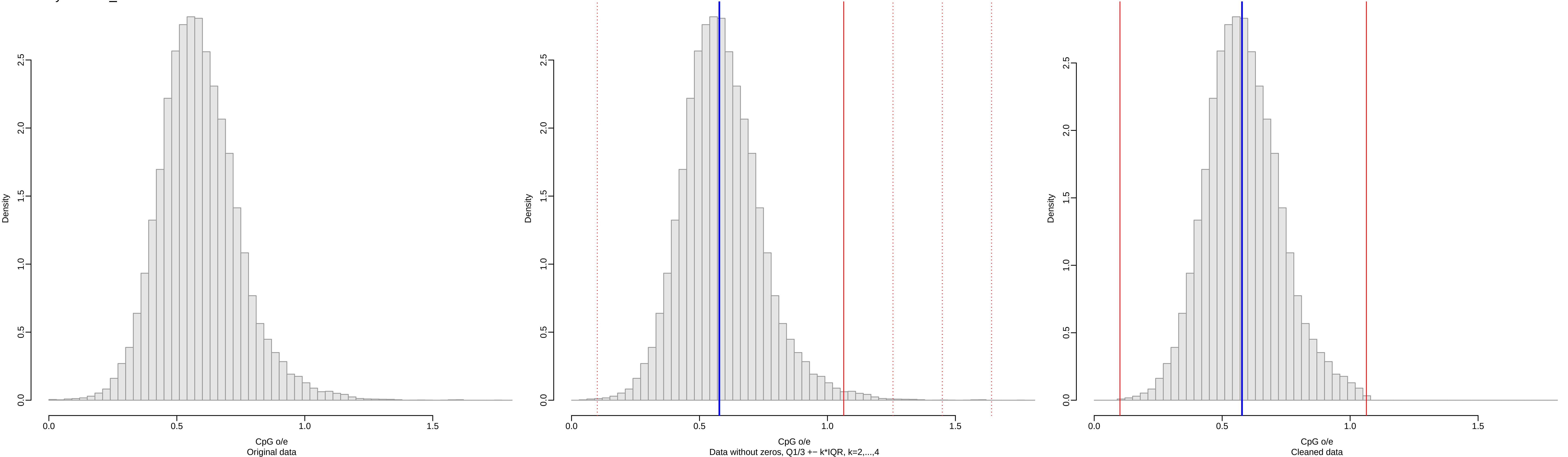

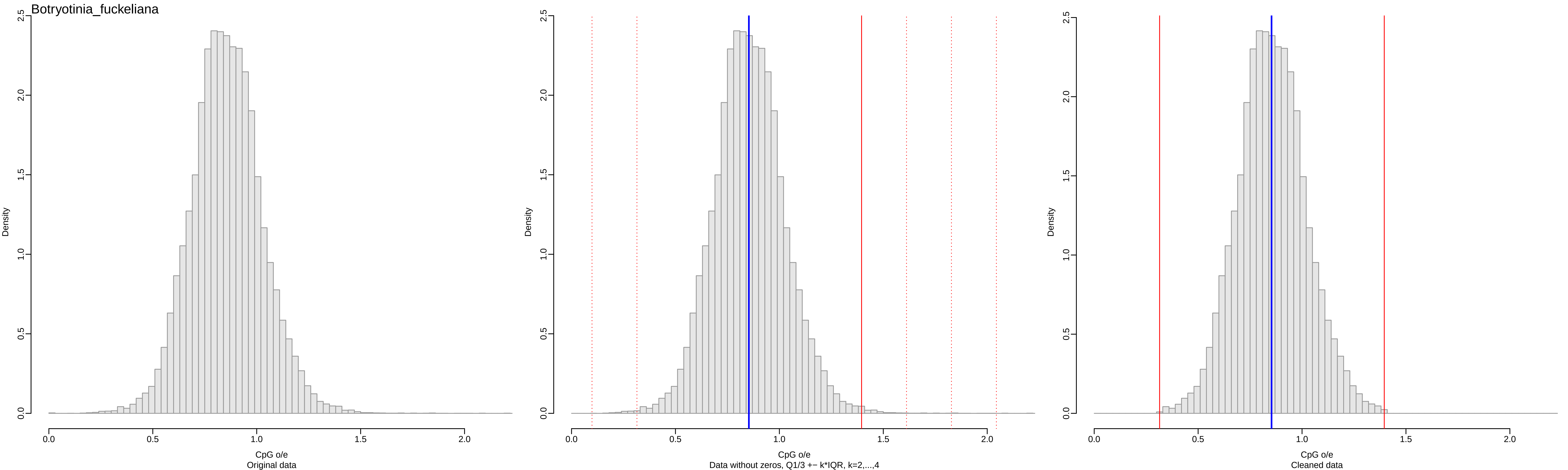

Botryotinia_fuckeliana
Density
CpG o/e
Original data
CpG o/e
Data without zeros, Q1/3 +− k*IQR, k=2,...,4
CpG o/e
Cleaned data

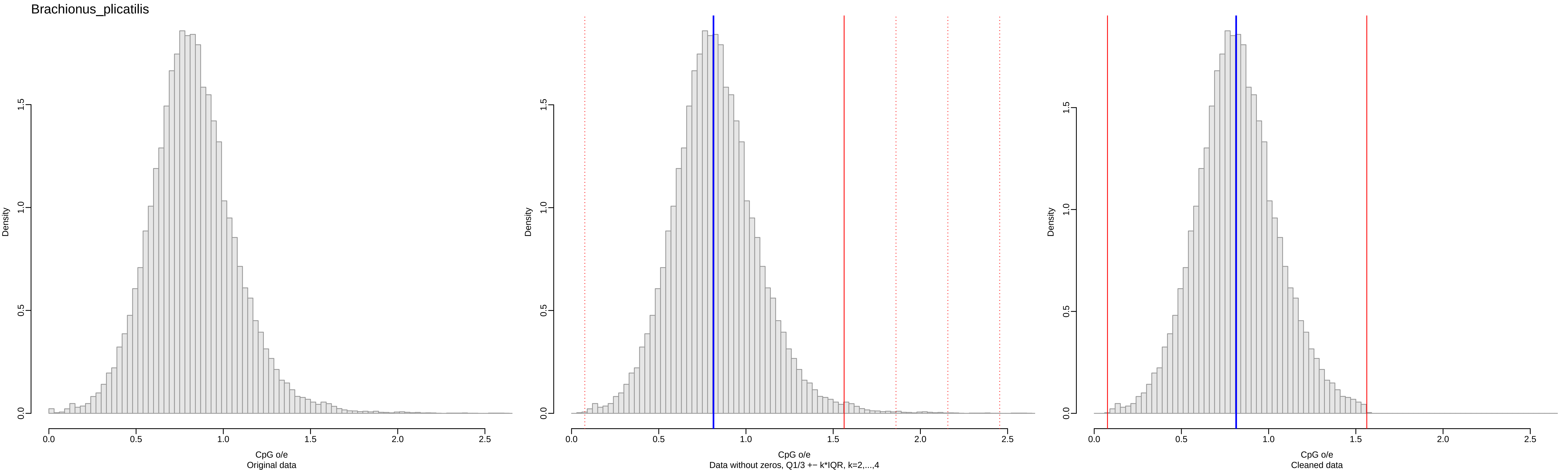

Brachionus_plicatilis
Density
0.0
0.5
1.0
1.5
2.0
2.5
CpG o/e
Original data
Data without zeros, Q1/3 +− k*IQR, k=2,...,4
Cleaned data

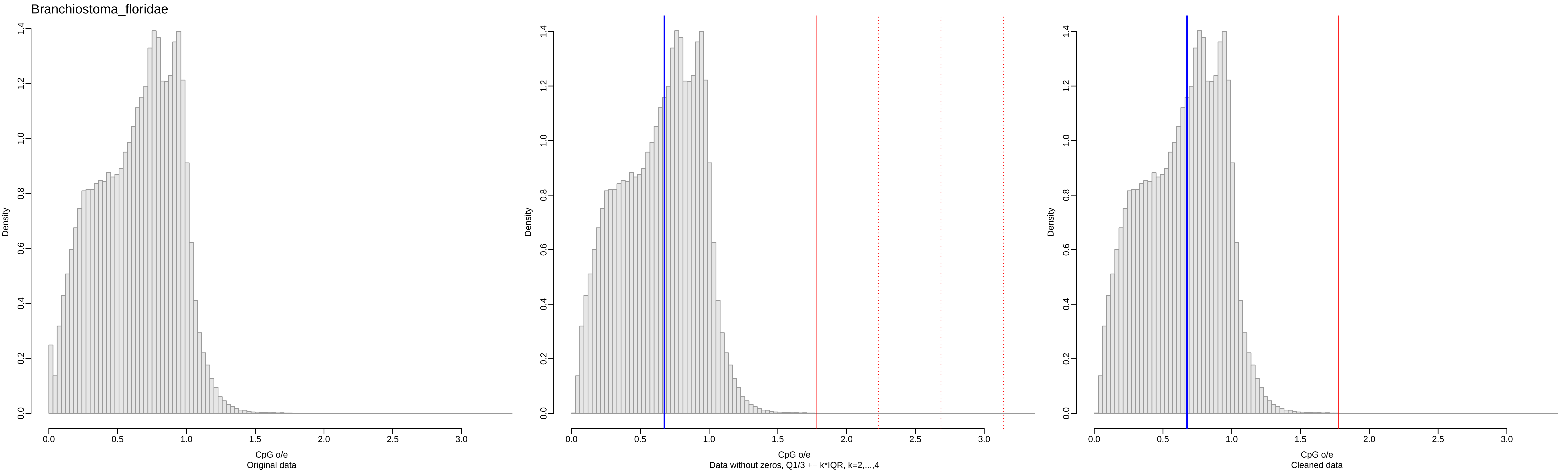

Branchiostoma_floridae
Density
0.0
0.2
0.4
0.6
0.8
1.0
1.2
1.4
0.0
0.5
1.0
1.5
2.0
2.5
3.0
CpG o/e
Original data
CpG o/e
Data without zeros, Q1/3 +− k*IQR, k=2,...,4
CpG o/e
Cleaned data

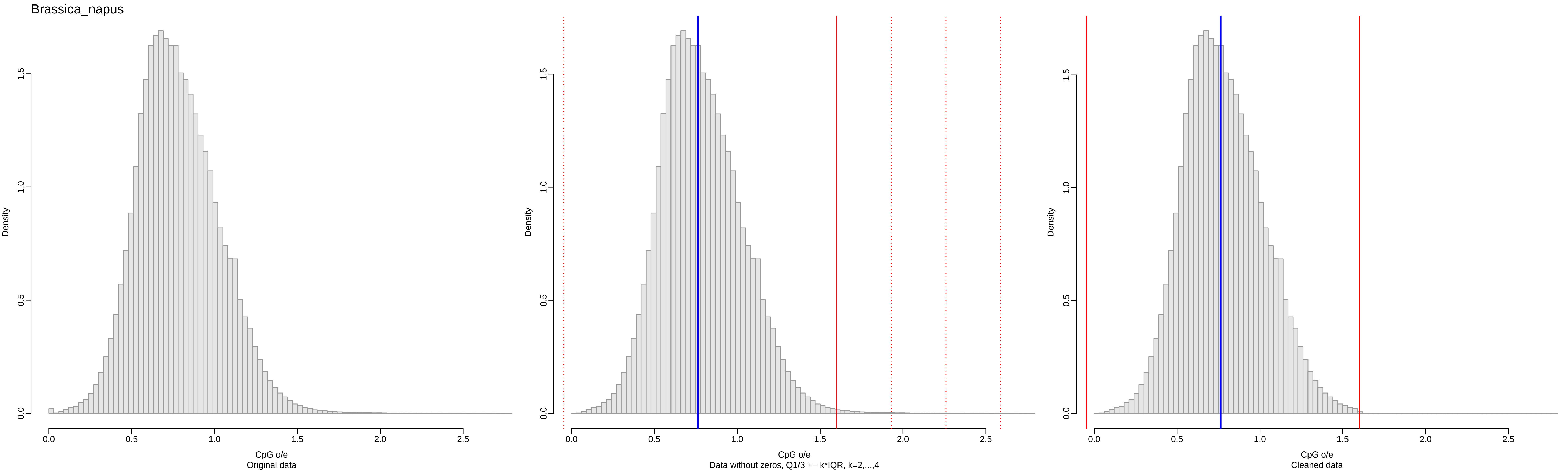
Brassica_napus
Density
CpG o/e
Original data
Density
CpG o/e
Data without zeros, Q1/3 +− k*IQR, k=2,...,4
Density
CpG o/e
Cleaned data

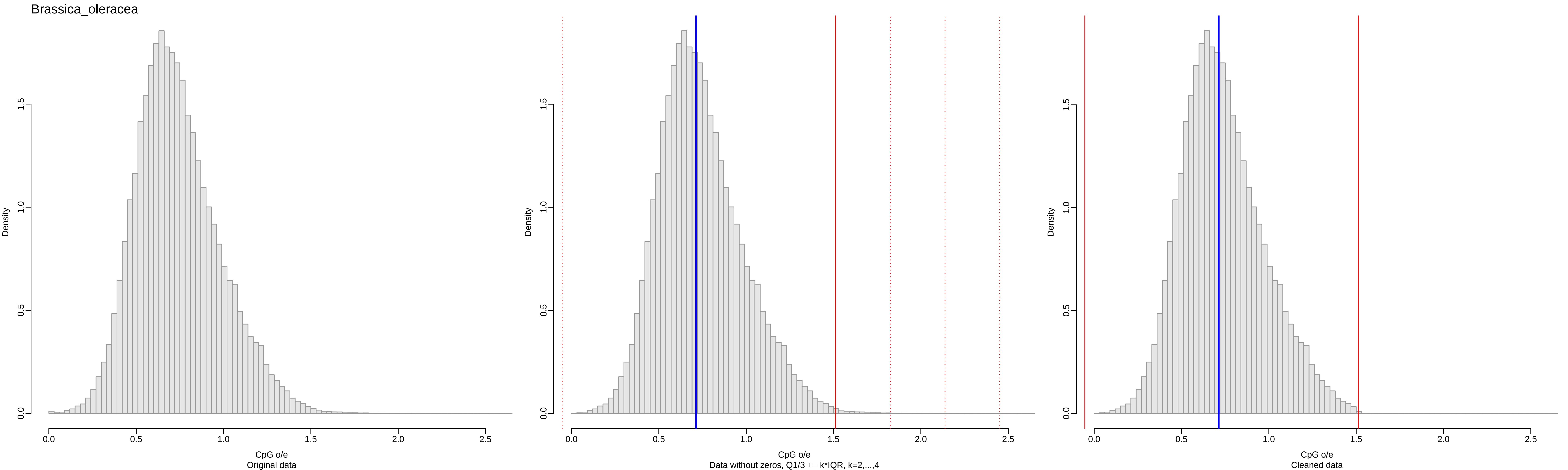

Brassica_oleracea
Density
0.0
0.5
1.0
1.5
2.0
2.5
CpG o/e
Original data
Data without zeros, Q1/3 +− k*IQR, k=2,...,4
Cleaned data

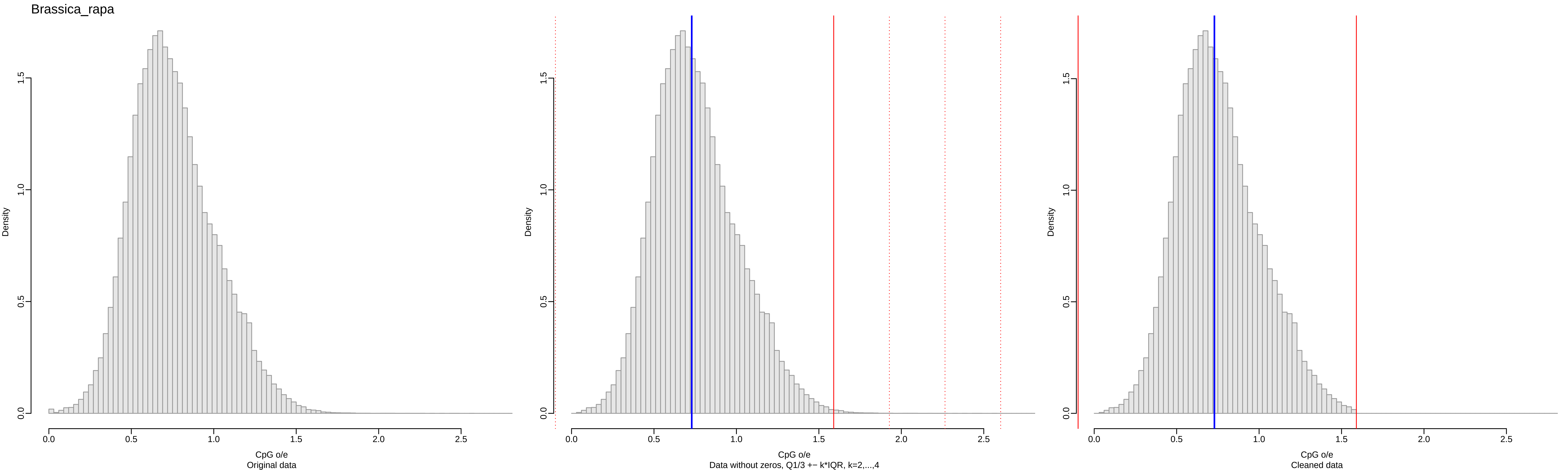
Brassica_rapa
Density
0.0
0.5
1.0
1.5
2.0
2.5
CpG o/e
Original data
Data without zeros, Q1/3 +− k*IQR, k=2,...,4
Cleaned data

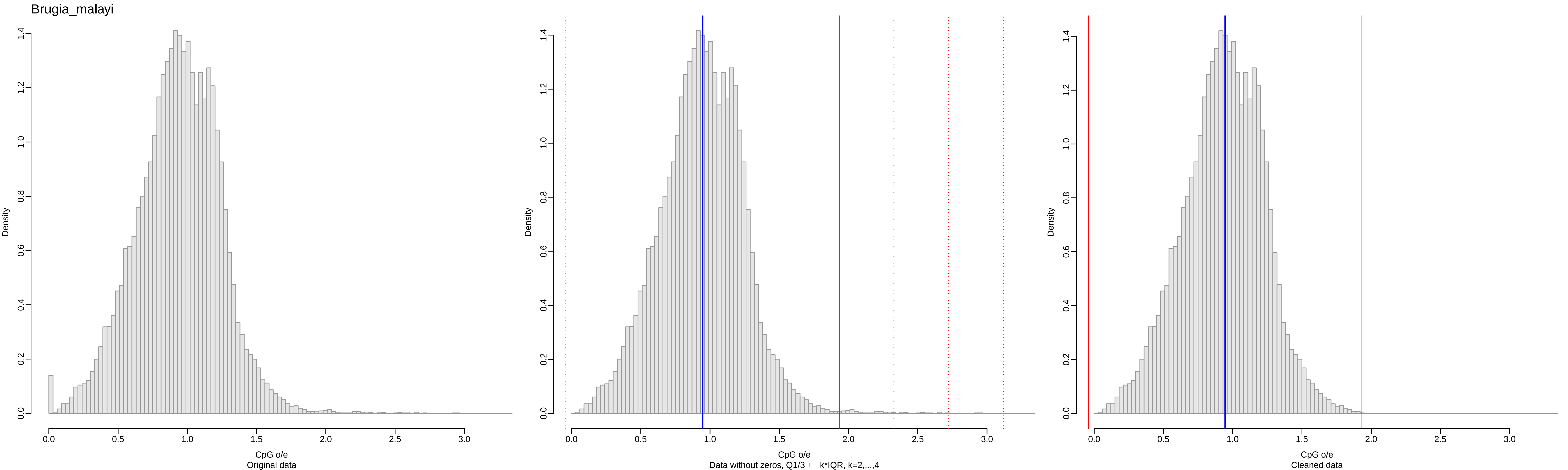
Brugia_malayi
Density
CpG o/e
Original data
Data without zeros, Q1/3 +− k*IQR, k=2,...,4
Cleaned data
0.0
0.2
0.4
0.6
0.8
1.0
1.2
1.4
0.5
1.5
2.0
2.5
3.0

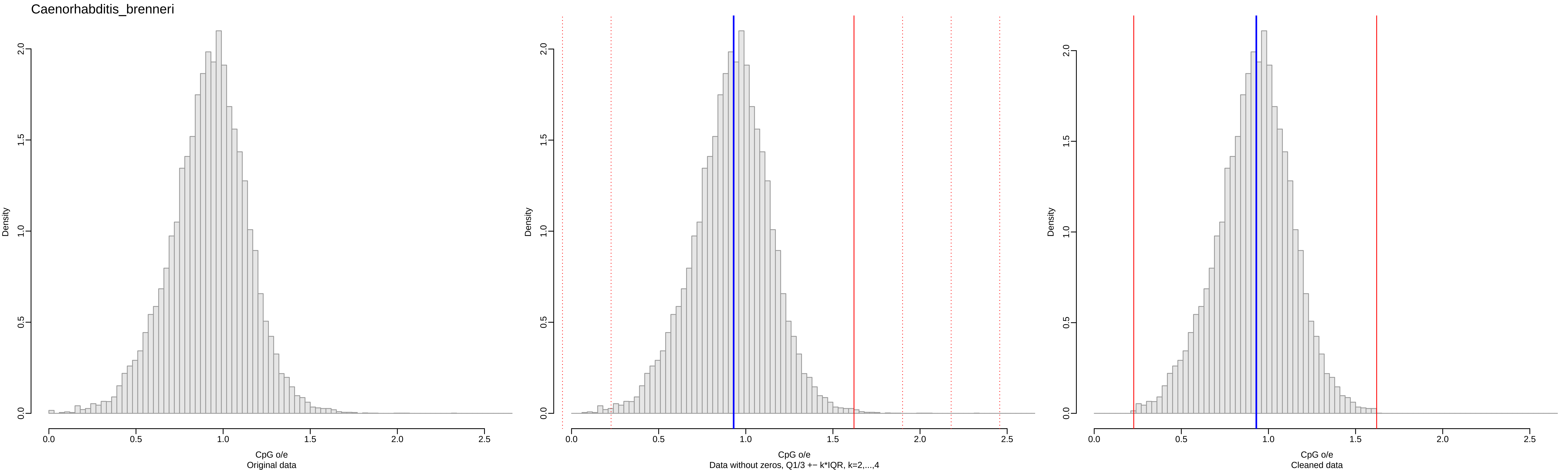
Caenorhabditis_brenneri
Density
CpG o/e
Original data
Data without zeros, Q1/3 +− k*IQR, k=2,...,4
Cleaned data
0.0
0.5
1.0
1.5
2.0
2.5

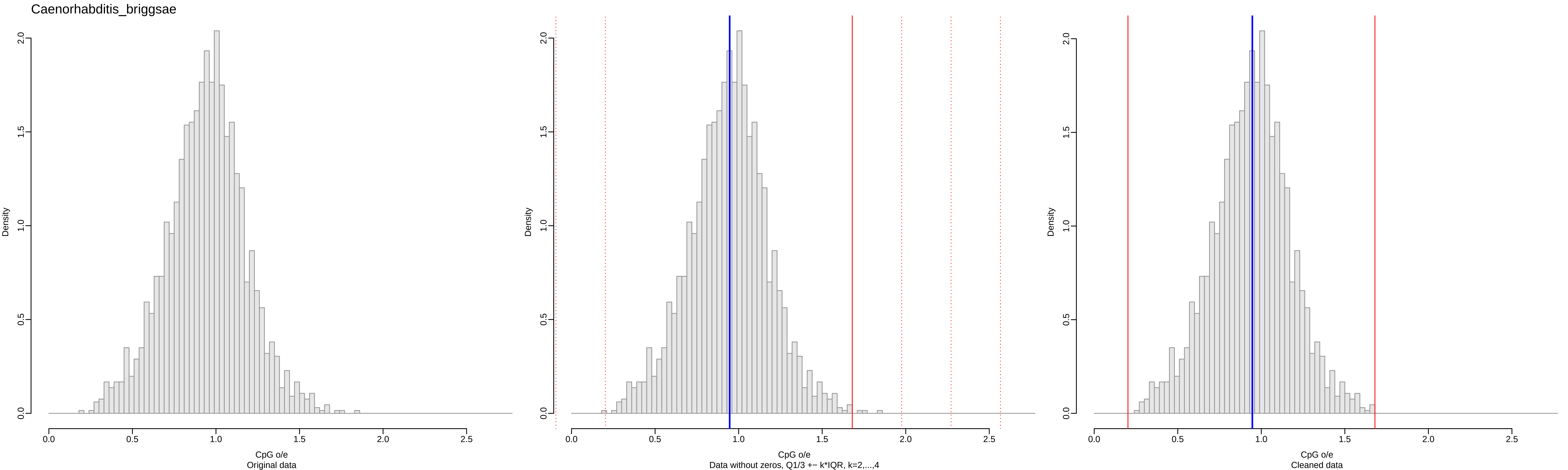

Caenorhabditis_briggsae
Density
CpG o/e
Original data
Data without zeros, Q1/3 +− k*IQR, k=2,...,4
Cleaned data

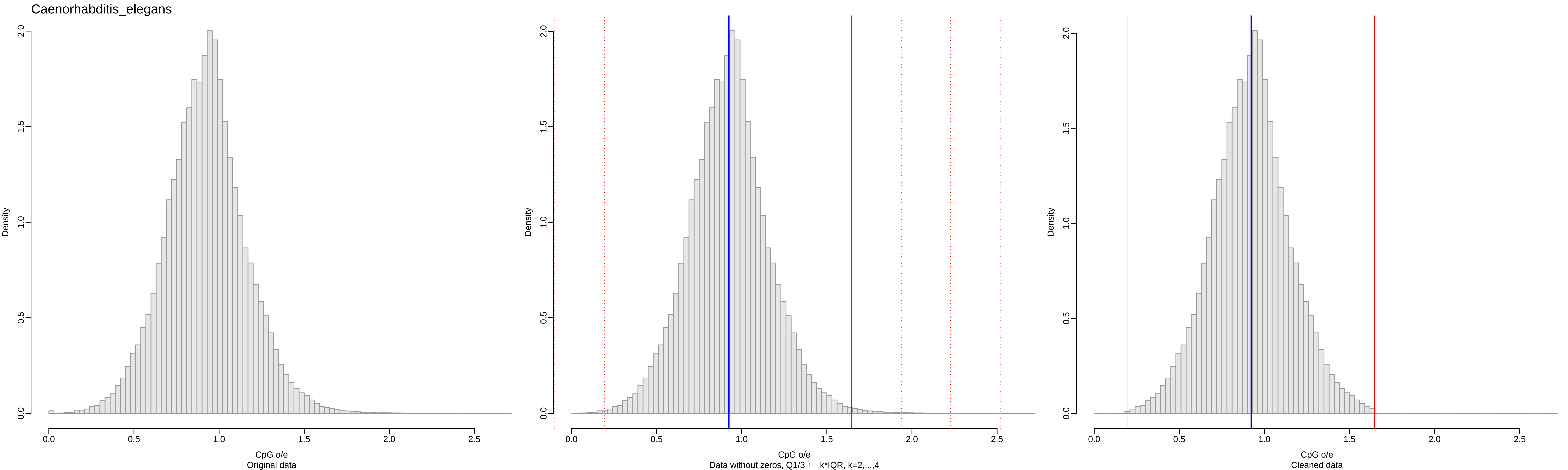
Caenorhabditis_elegans
Density
0.0
0.5
1.0
1.5
2.0
2.5
CpG o/e
Original data
CpG o/e
Data without zeros, Q1/3 +− k*IQR, k=2,...,4
CpG o/e
Cleaned data

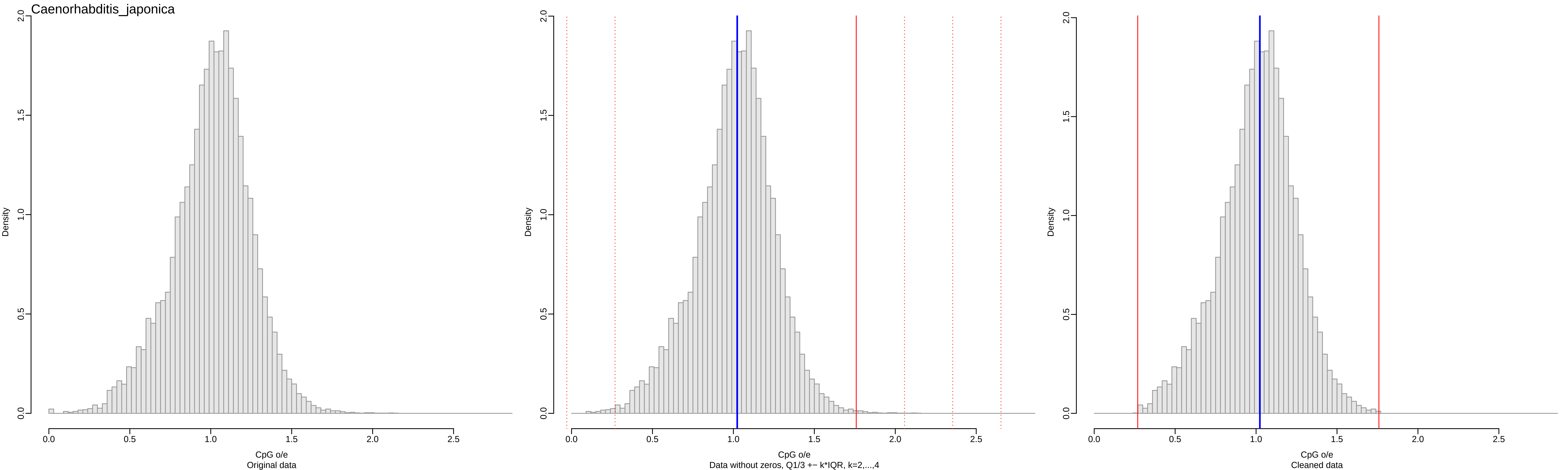

Caenorhabditis_japonica
Density
CpG o/e
Original data
Data without zeros, Q1/3 +− k*IQR, k=2,...,4
Cleaned data
0.0
0.5
1.0
1.5
2.0
2.5

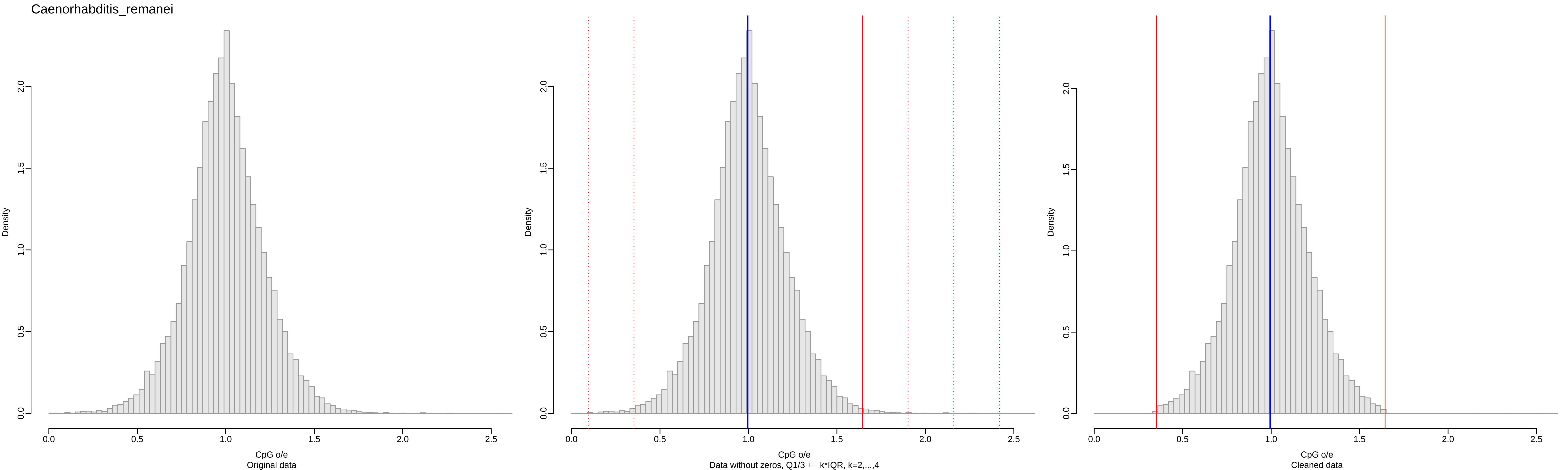
Caenorhabditis_remanei
Density
CpG o/e
Original data
Density
CpG o/e
Data without zeros, Q1/3 +− k*IQR, k=2,...,4
Density
CpG o/e
Cleaned data
0.0
0.5
1.0
1.5
2.0
2.5

Callinectes_sapidus

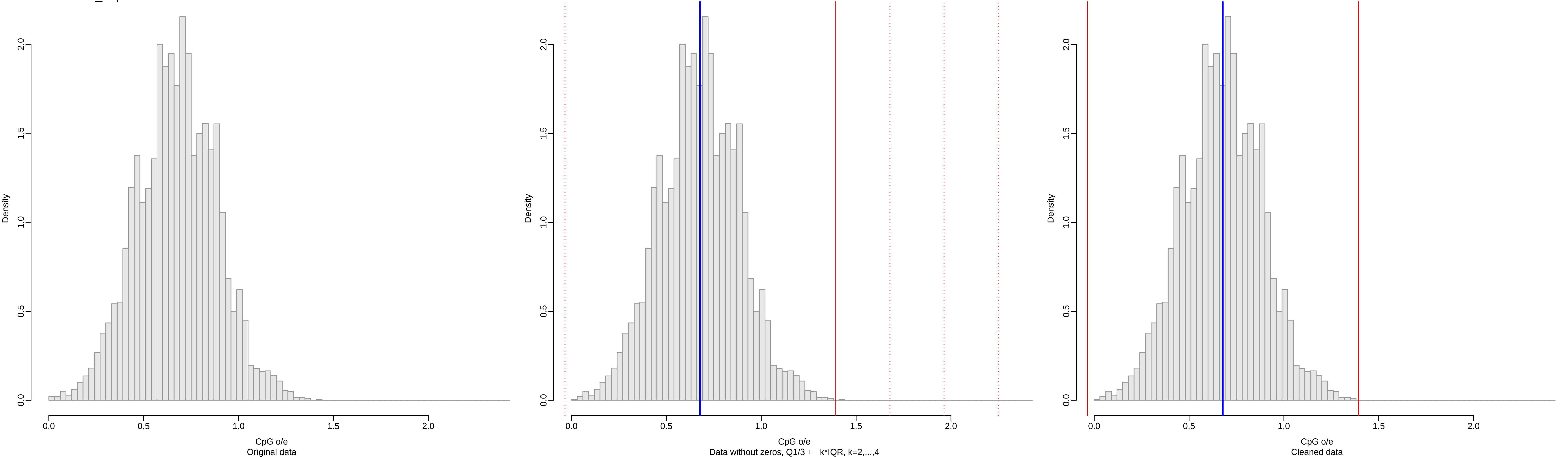

Callorhinchus_milii

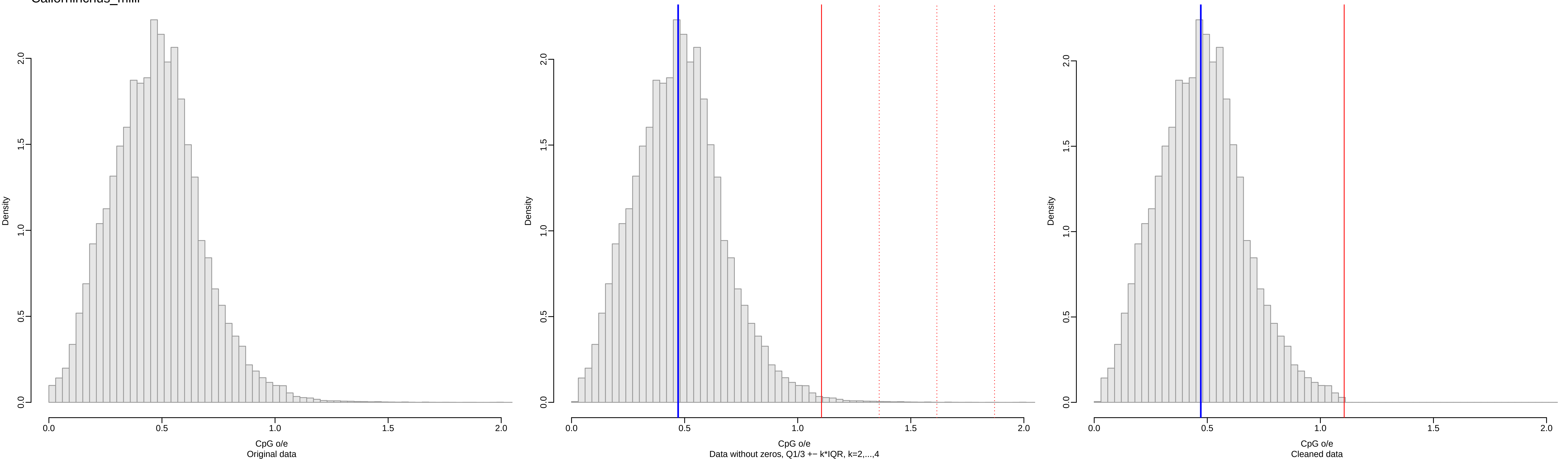

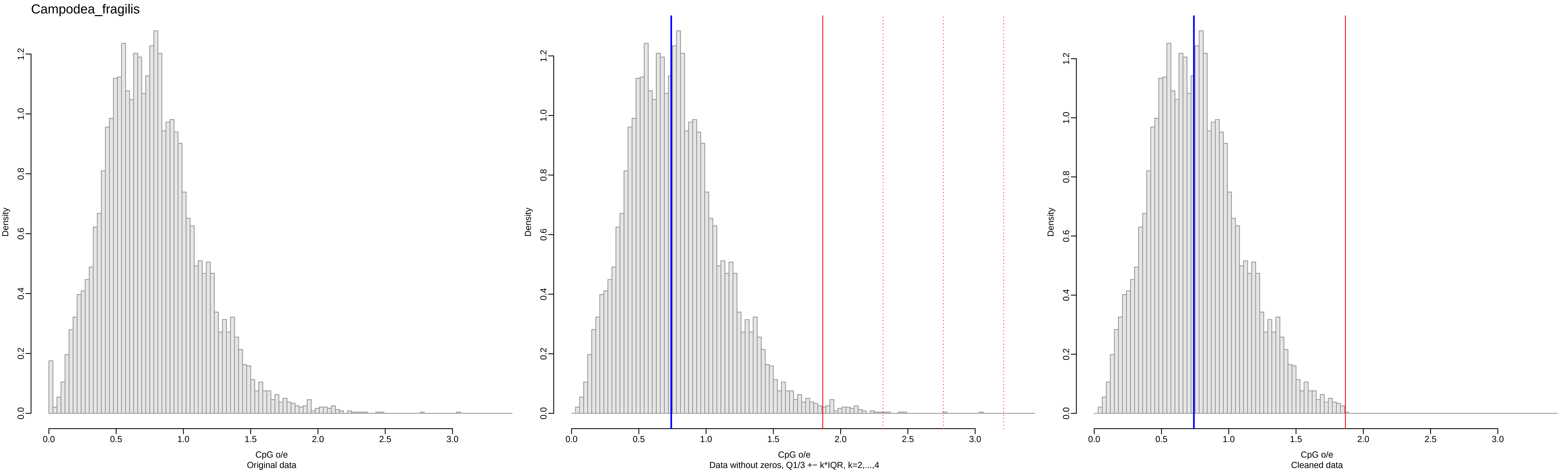
Campodea_fragilis
Density
CpG o/e
Original data
Data without zeros, Q1/3 +− k*IQR, k=2,...,4
Cleaned data
0.0
0.2
0.4
0.6
0.8
1.0
1.2
0.5
1.5
2.0
2.5
3.0

Cancer_magister

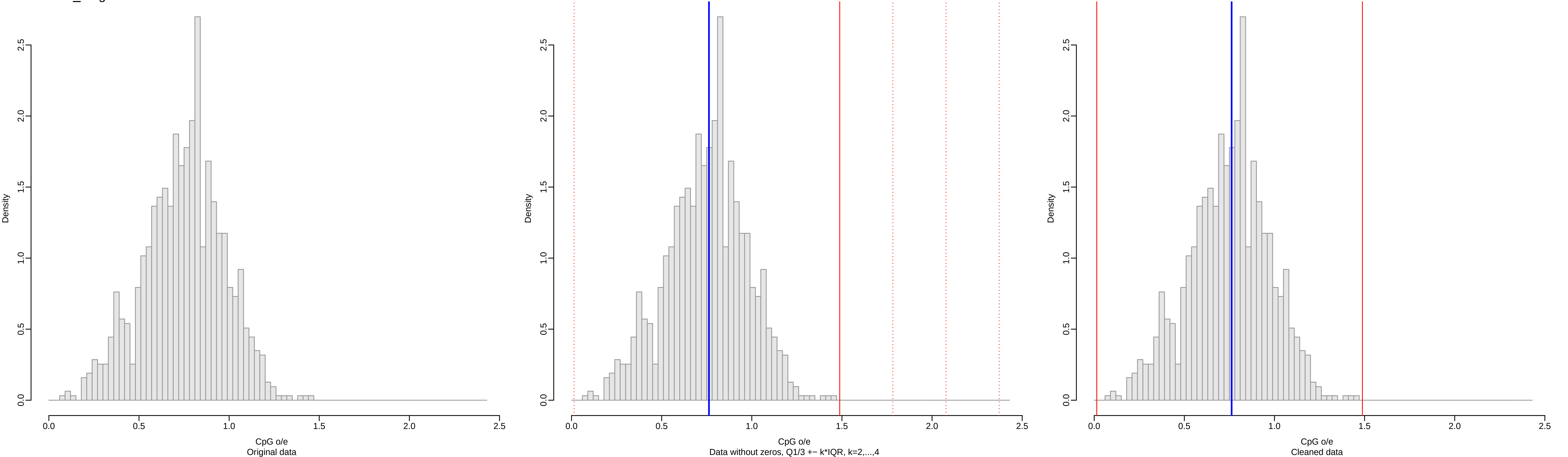

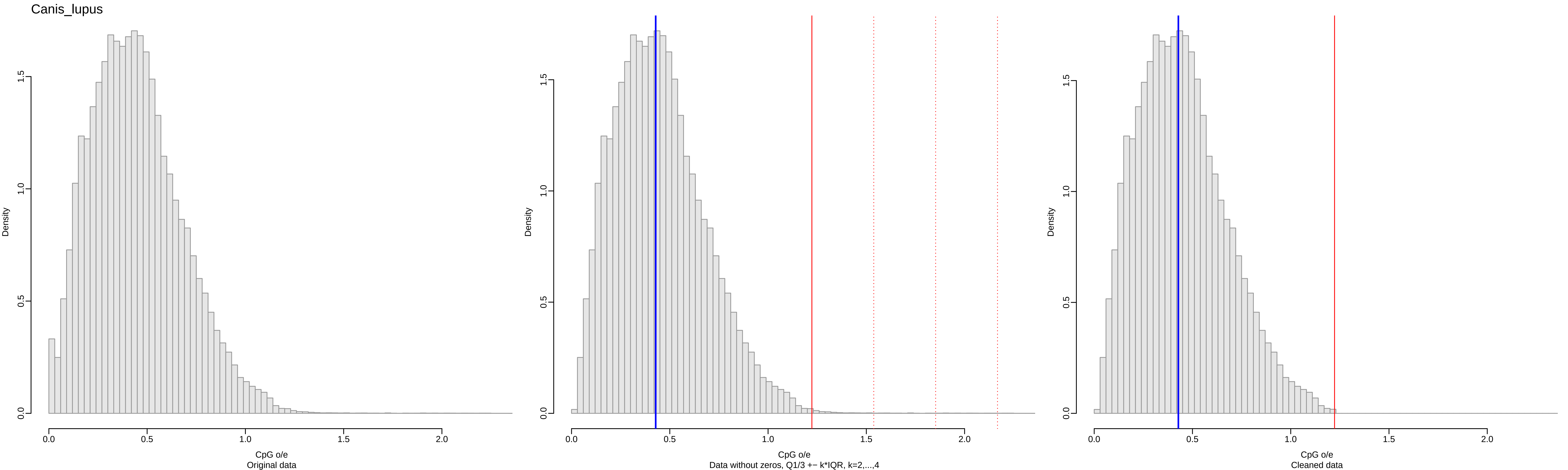
Canis_lupus
Density
CpG o/e
Original data
Data without zeros, Q1/3 +− k*IQR, k=2,...,4
Cleaned data
0.0
0.5
1.0
1.5
2.0

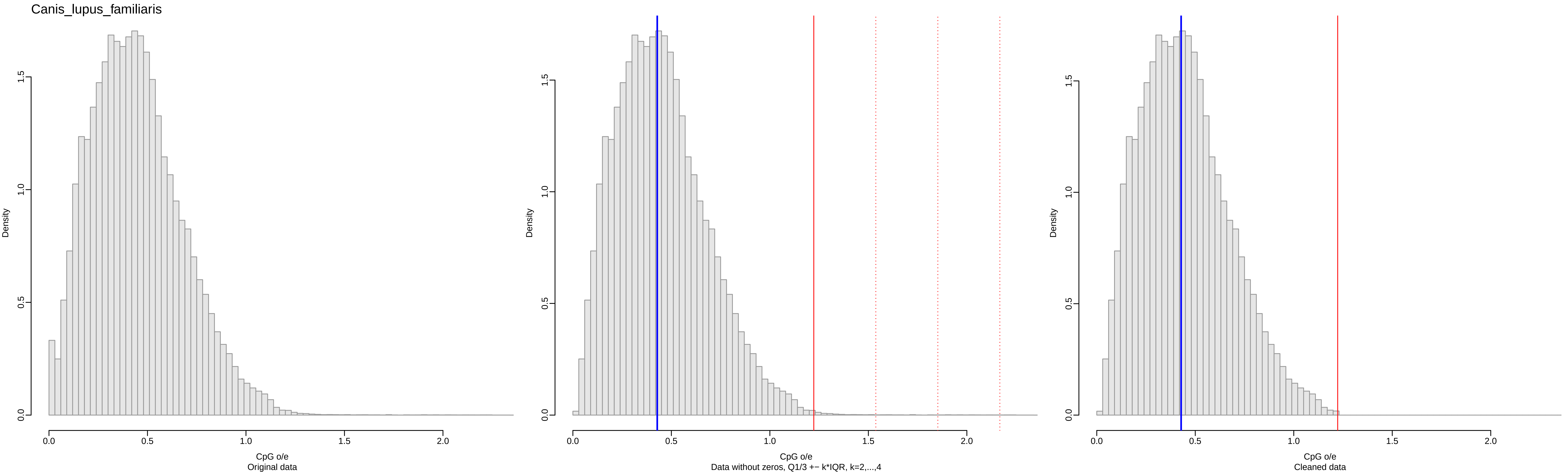

Canis_lupus_familiaris
Density
CpG o/e
Original data
Data without zeros, Q1/3 +− k*IQR, k=2,...,4
Cleaned data

Cannabis_sativa

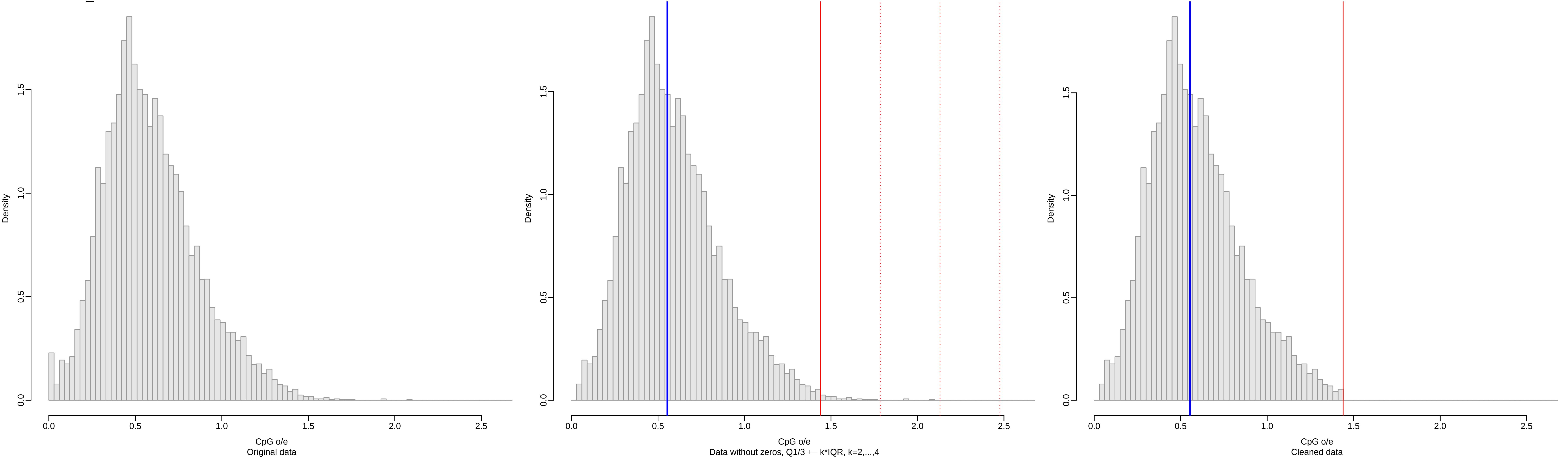

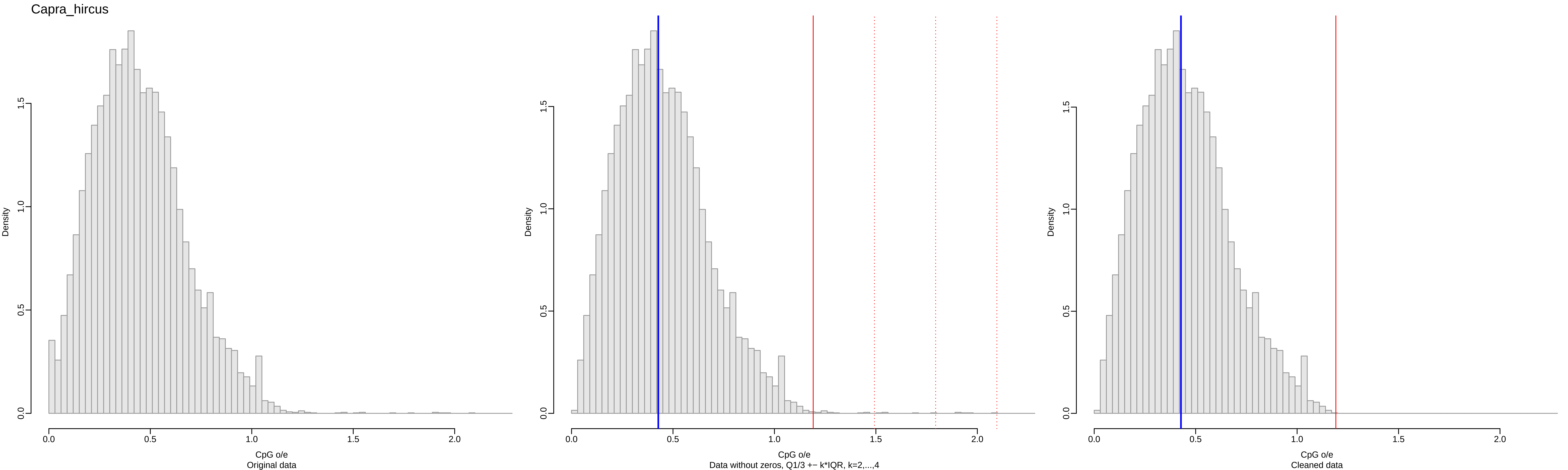
Capra_hircus
Density
CpG o/e
Original data
Density
CpG o/e
Data without zeros, Q1/3 +− k*IQR, k=2,...,4
Density
CpG o/e
Cleaned data
0.0
0.5
1.0
1.5
2.0

Capsicum_annuum

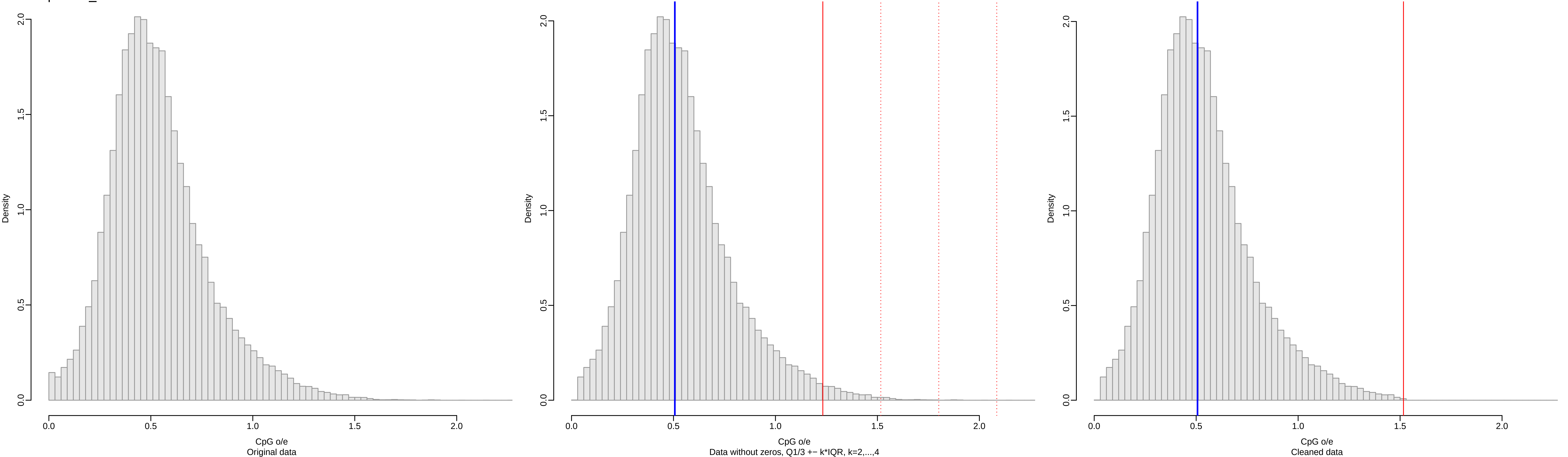

Carassius_auratus

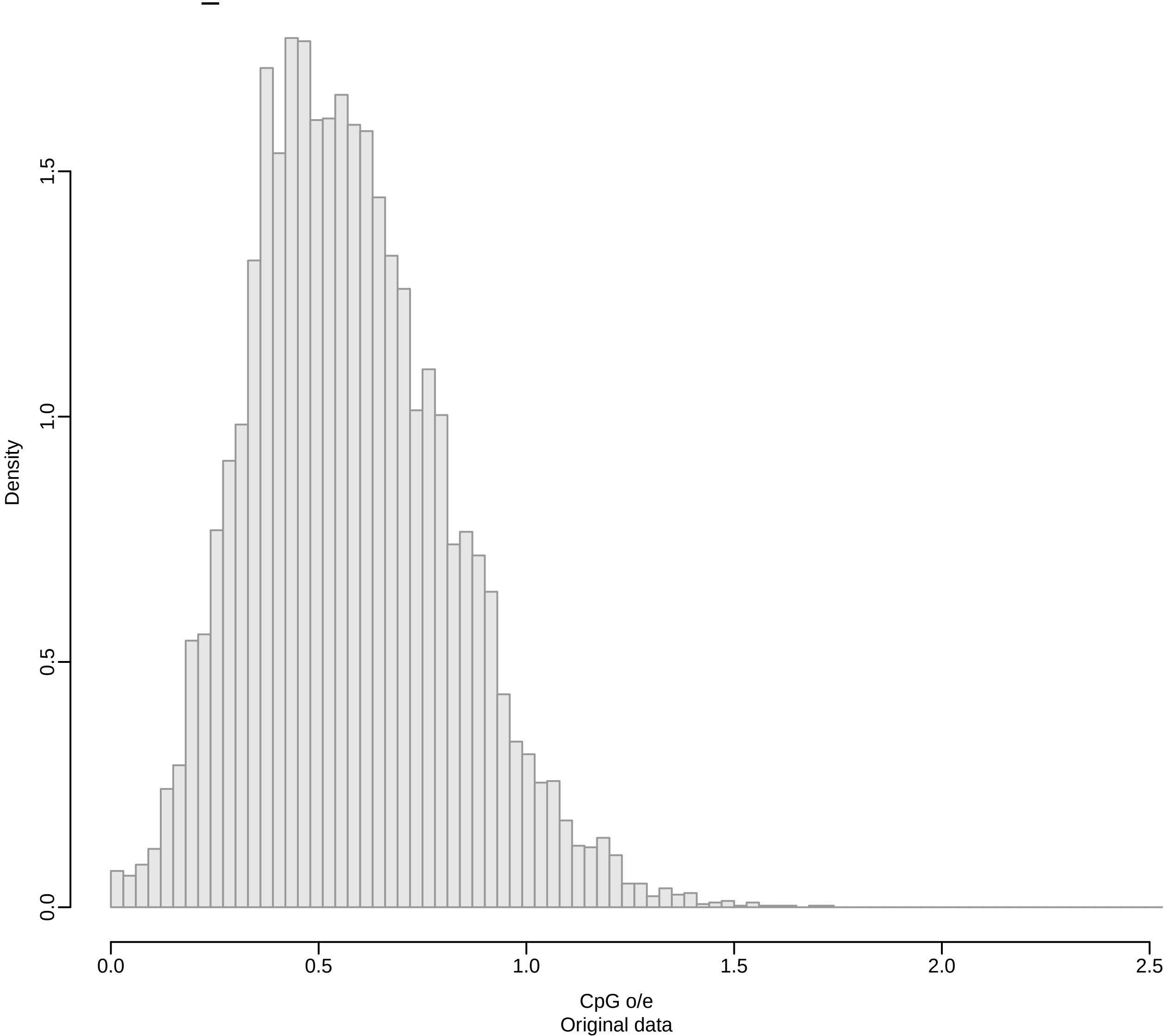

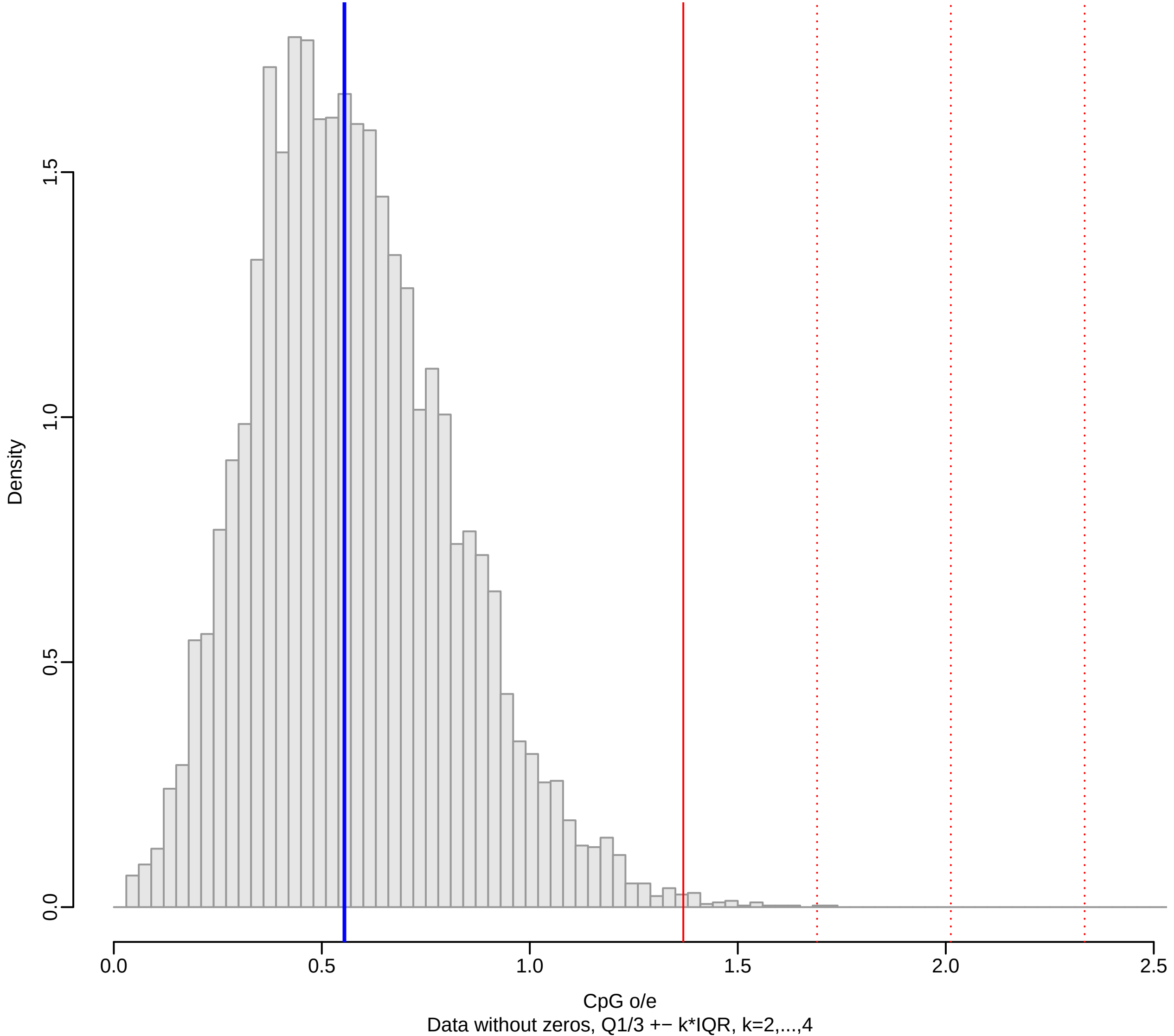

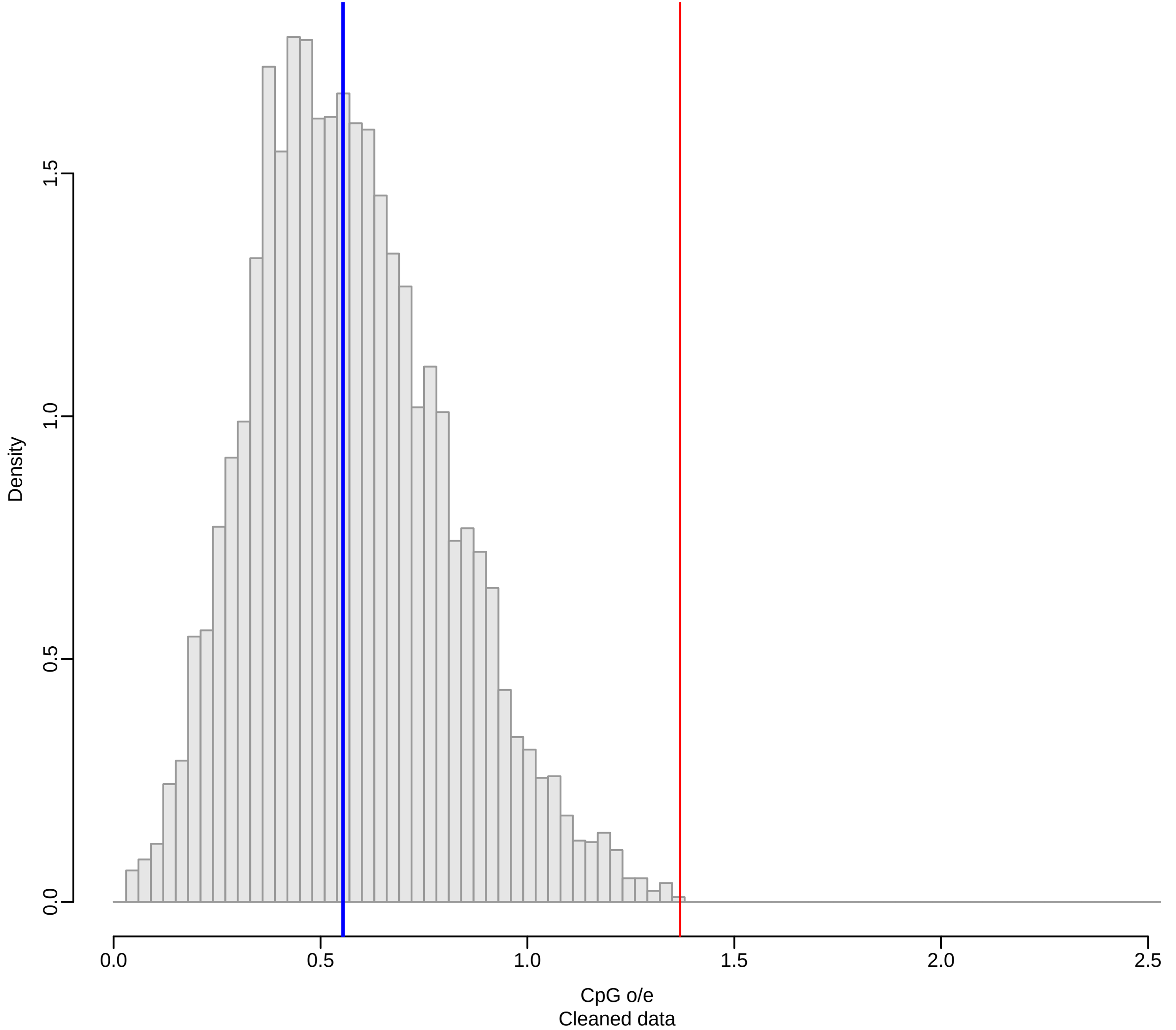

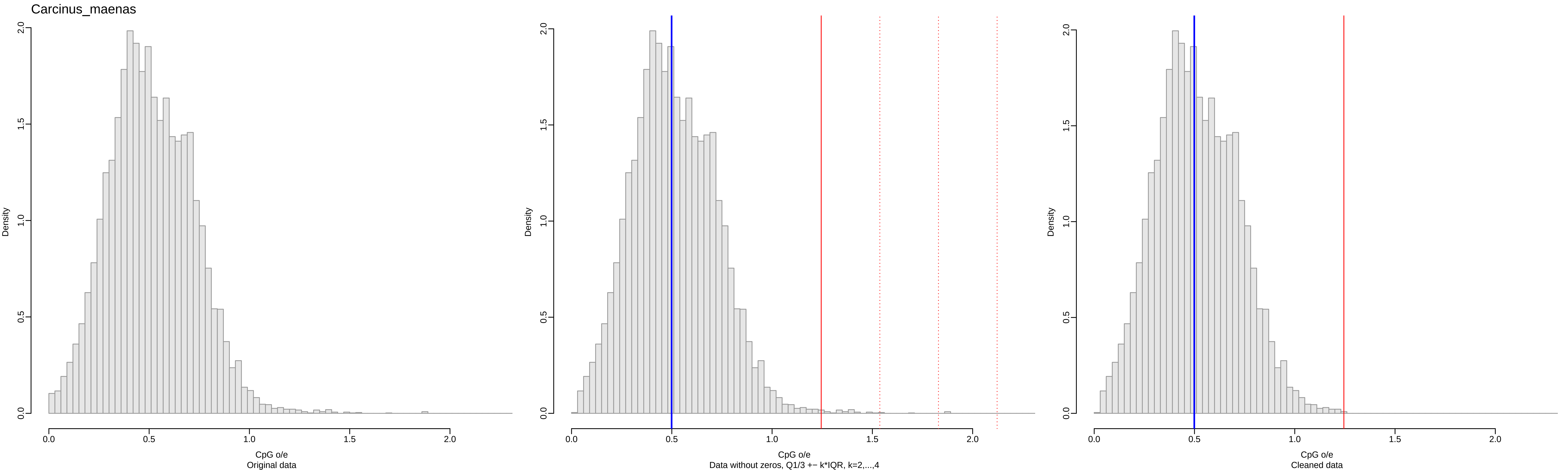
Carcinus_maenas
Density
CpG o/e
Original data
Data without zeros, Q1/3 +− k*IQR, k=2,...,4
Cleaned data
0.0
0.5
1.0
1.5
2.0

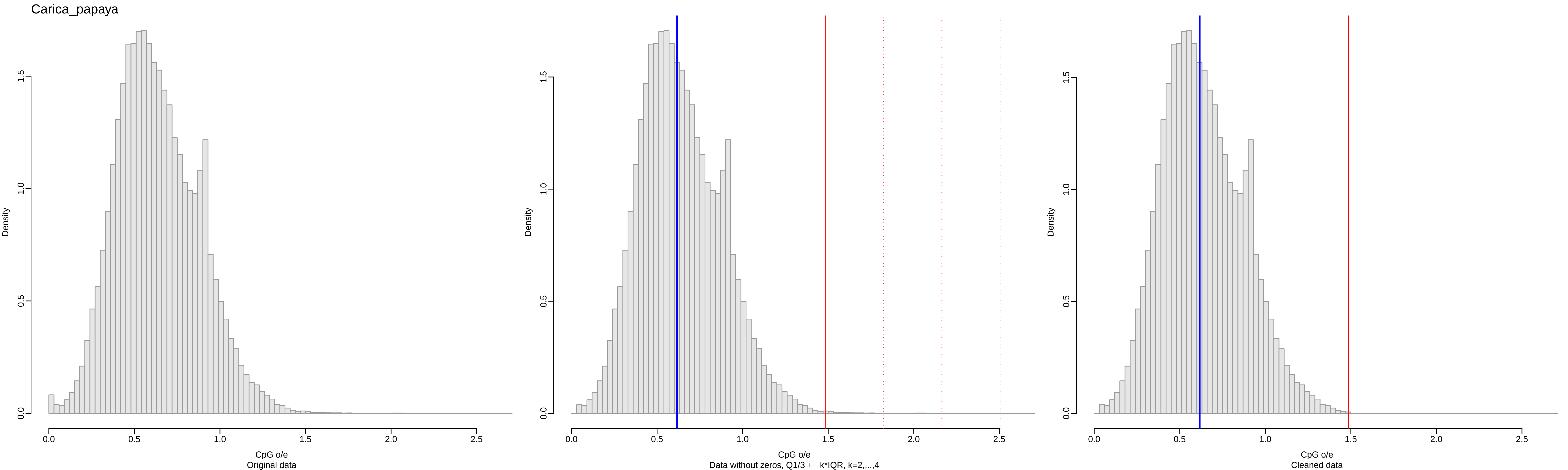
Carica_papaya
Density
CpG o/e
Original data
Data without zeros, Q1/3 +− k*IQR, k=2,...,4
Cleaned data
0.0
0.5
1.0
1.5
2.0
2.5

Carteriospongia_foliascens

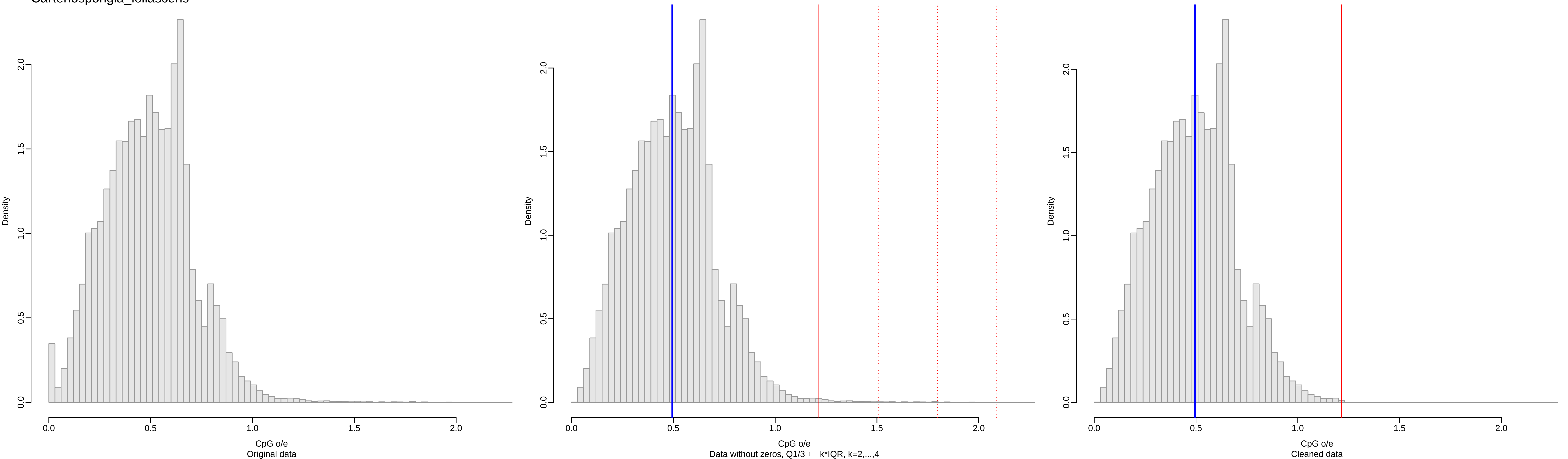

Carthamus_tinctorius

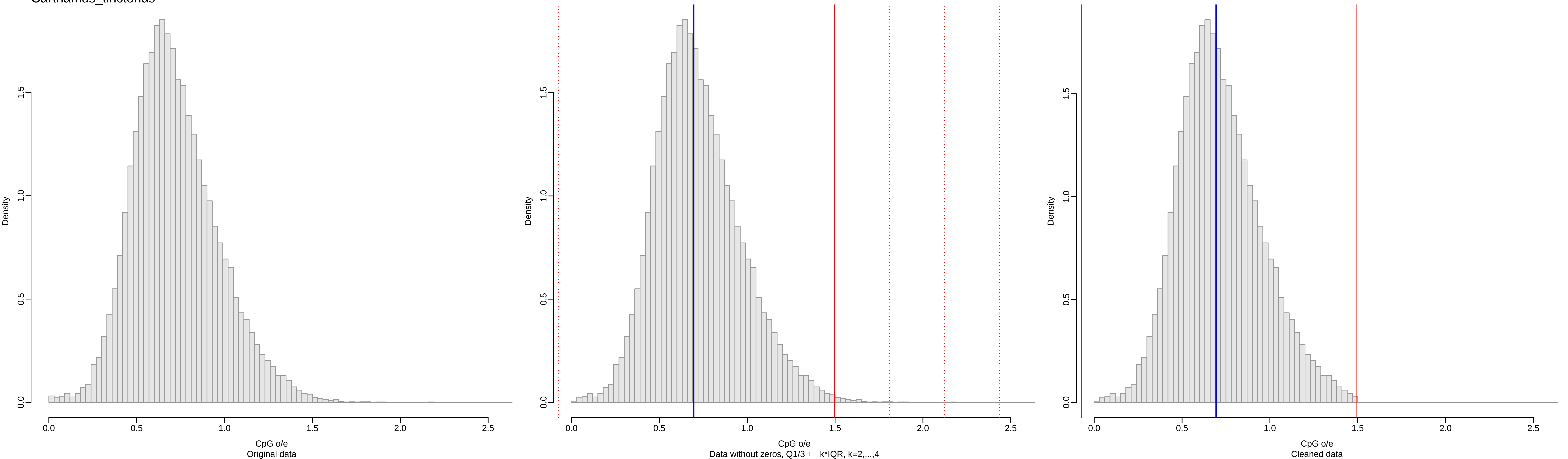

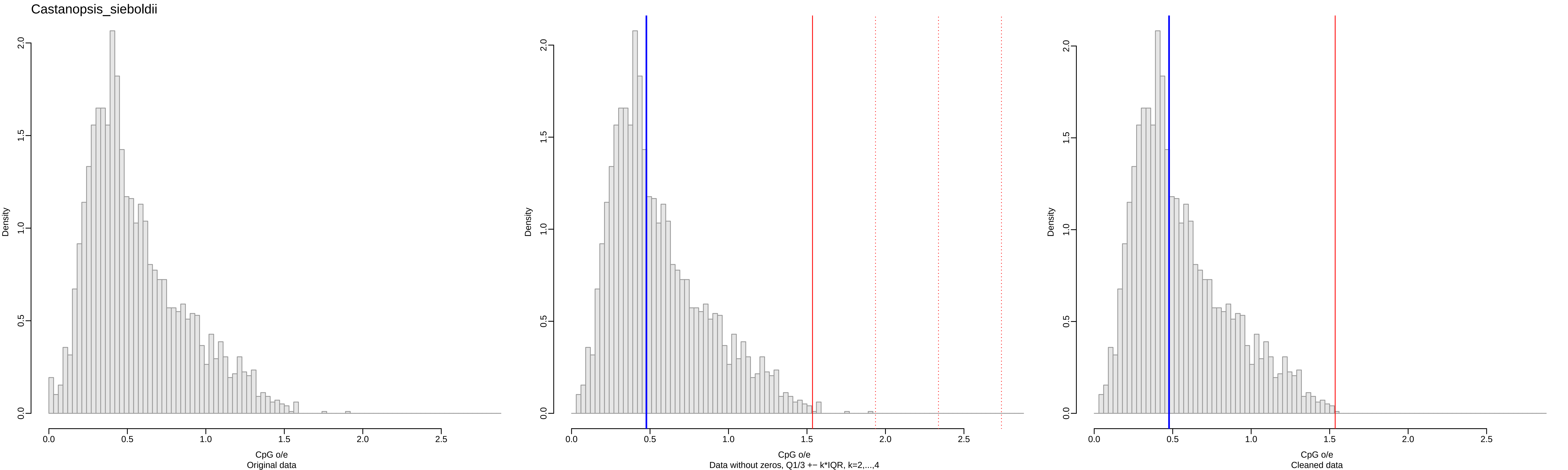
Castanopsis_sieboldii
Density
CpG o/e
Original data
CpG o/e
Data without zeros, Q1/3 +− k*IQR, k=2,...,4
CpG o/e
Cleaned data

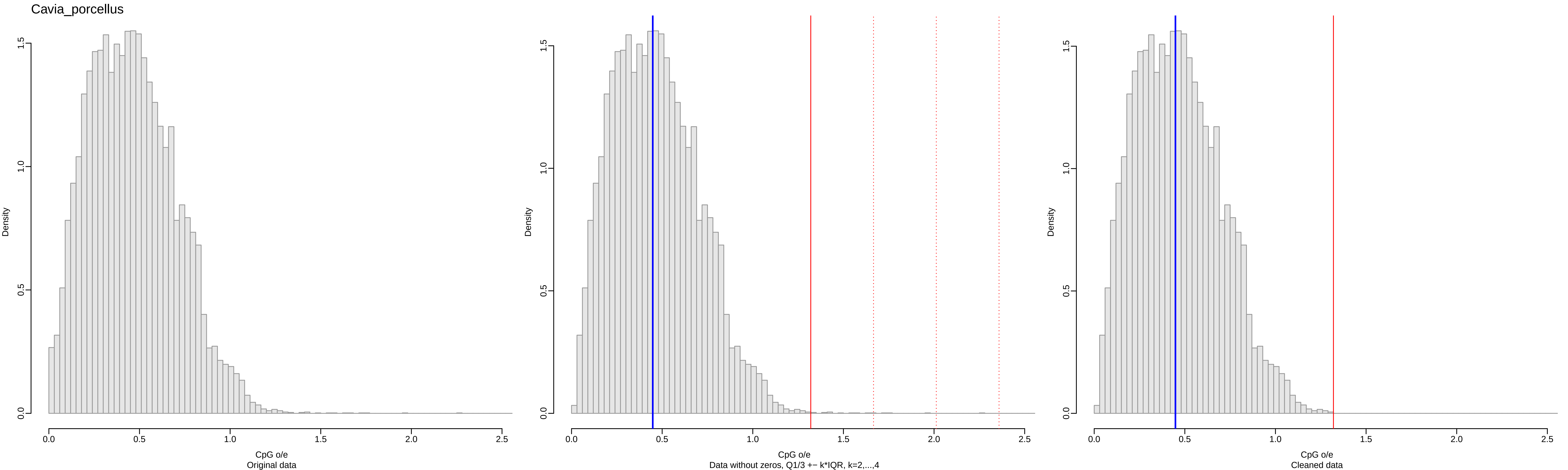
Cavia_porcellus
Density
CpG o/e
Original data
Data without zeros, Q1/3 +− k*IQR, k=2,...,4
Cleaned data
0.0
0.5
1.0
1.5
2.0
2.5

Celuca_pugilator

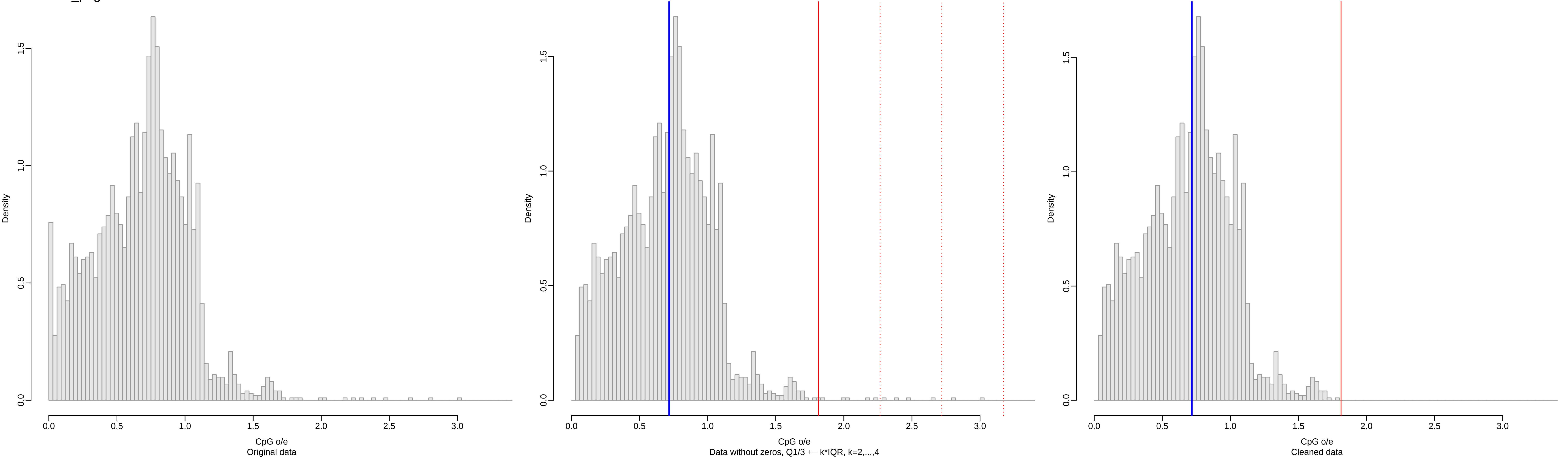

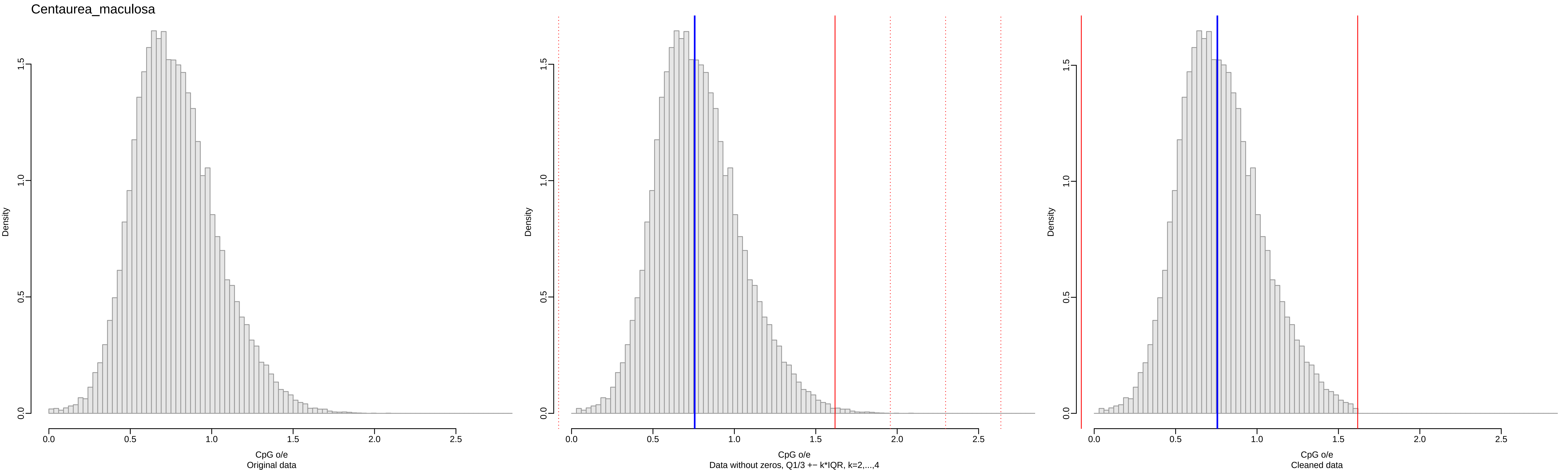
Centaurea_maculosa
Density
CpG o/e
Original data
Data without zeros, Q1/3 +− k*IQR, k=2,...,4
Cleaned data
0.0
0.5
1.0
1.5
2.0
2.5

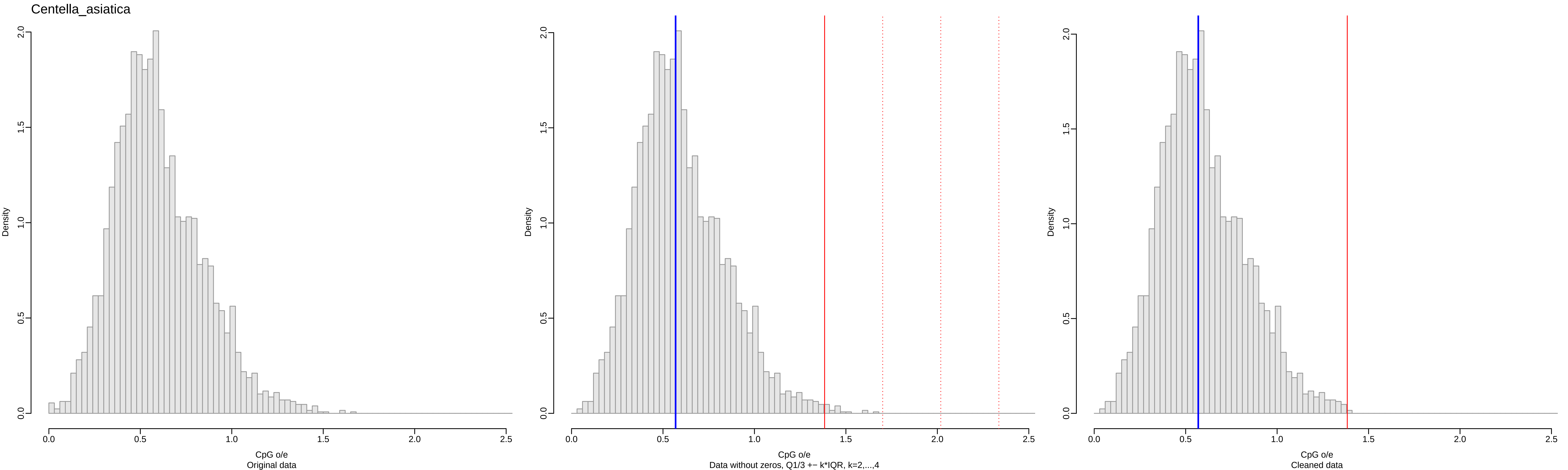

Centella_asiatica
Density
CpG o/e
Original data
Density
CpG o/e
Data without zeros, Q1/3 +− k*IQR, k=2,...,4
Density
CpG o/e
Cleaned data

Ceratopteris_richardii

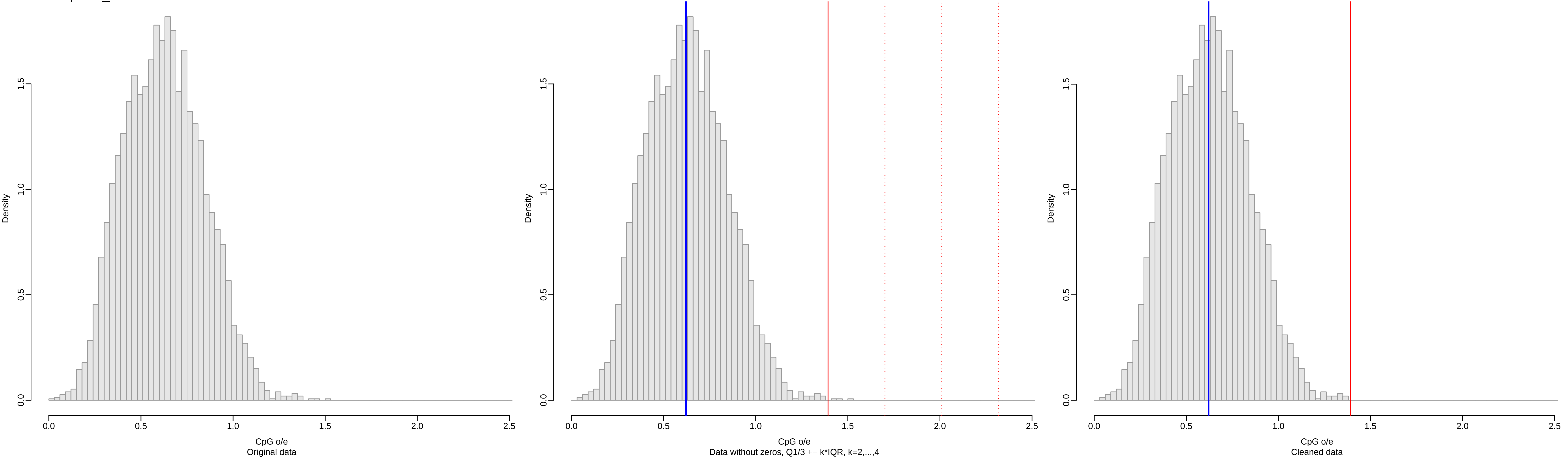

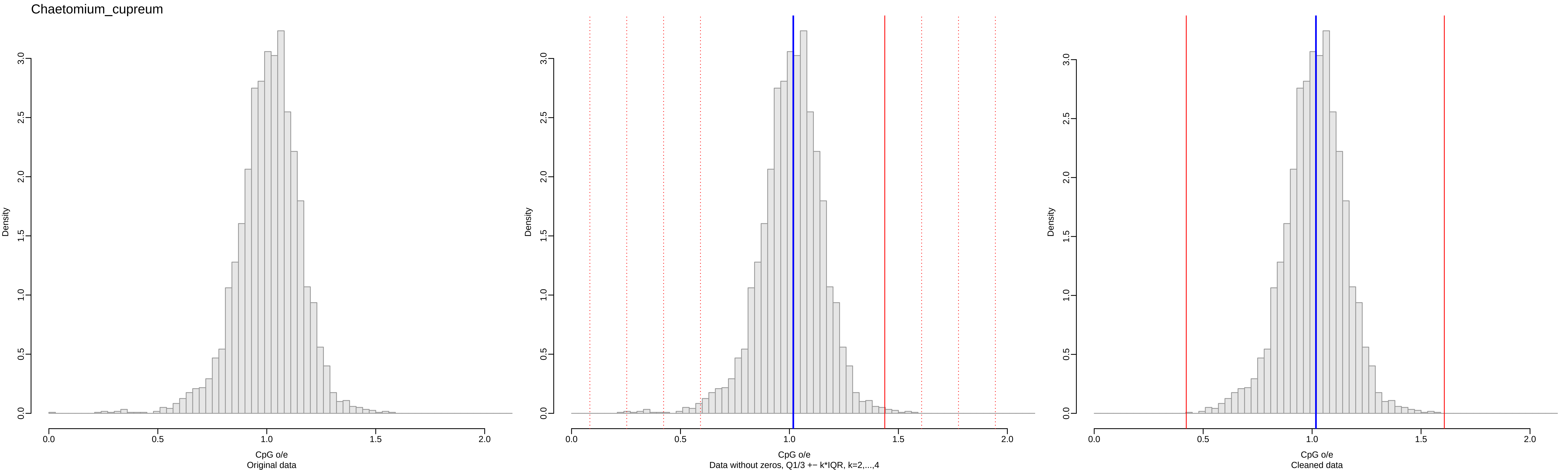
Chaetomium_cupreum
Density
CpG o/e
Original data
Density
CpG o/e
Data without zeros, Q1/3 +− k*IQR, k=2,...,4
Density
CpG o/e
Cleaned data

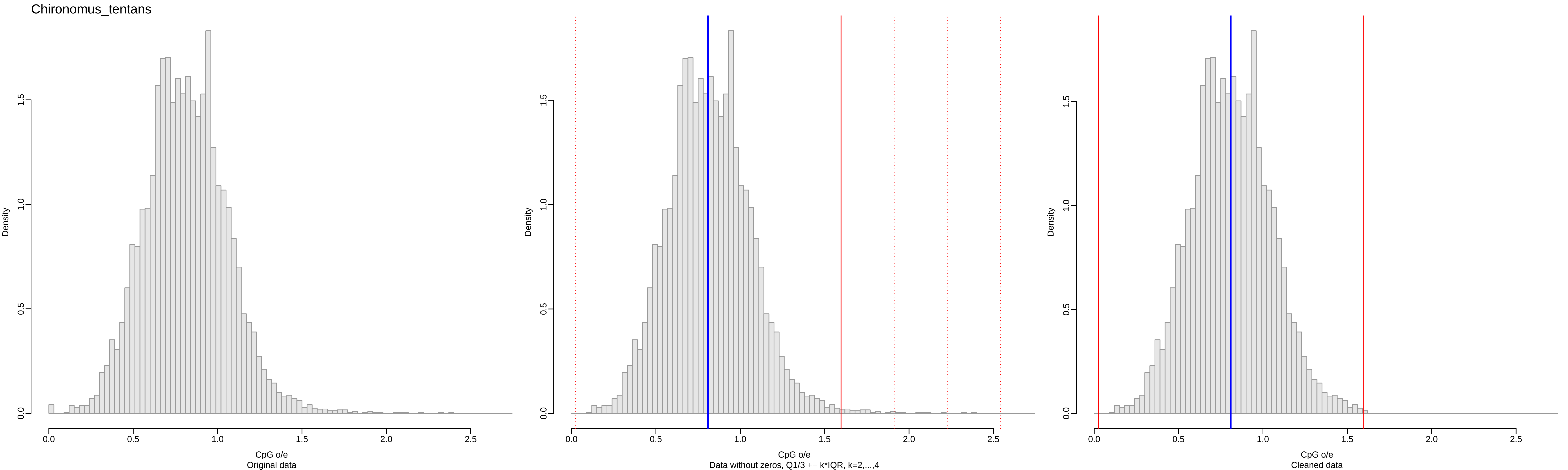
Chironomus_tentans
Density
CpG o/e
Original data
CpG o/e
Data without zeros, Q1/3 +− k*IQR, k=2,...,4
CpG o/e
Cleaned data

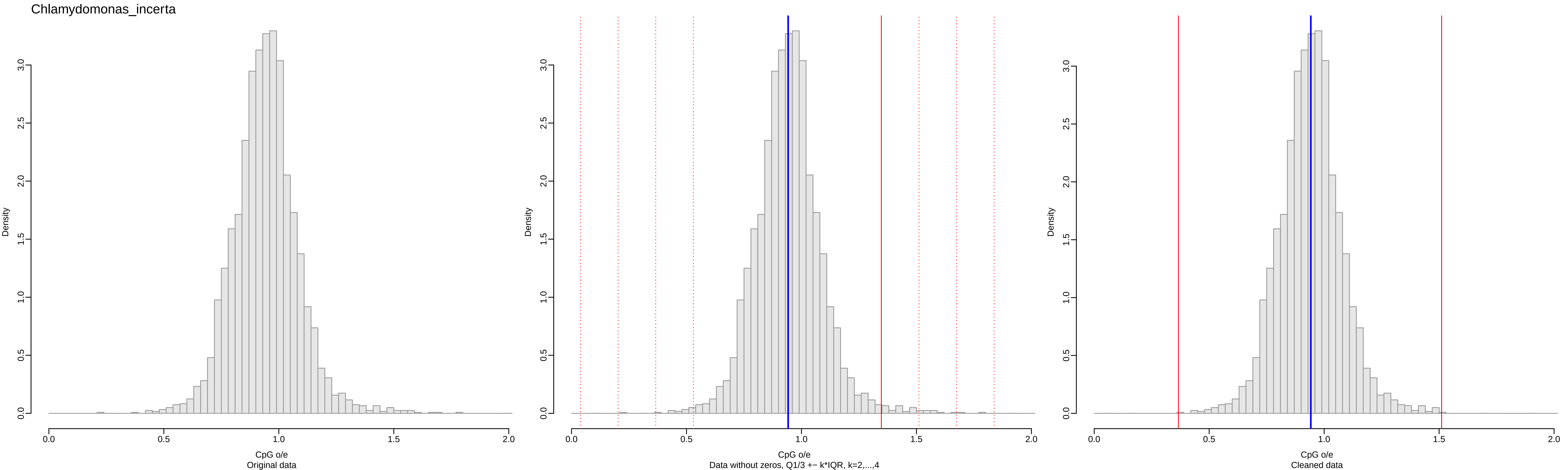
Chlamydomonas_incerta
Density
0.0
0.5
1.0
1.5
2.0
2.5
3.0
CpG o/e
Original data
Data without zeros, Q1/3 +− k*IQR, k=2,...,4
Cleaned data

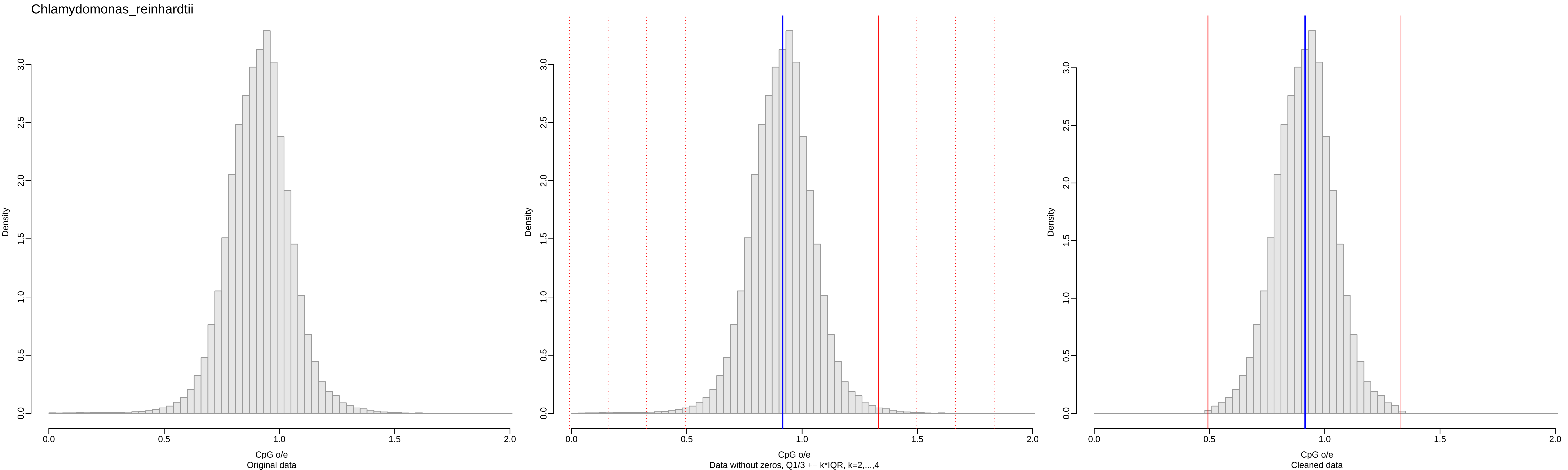

Chlamydomonas_reinhardtii
Density
CpG o/e
Original data
Density
CpG o/e
Data without zeros, Q1/3 +− k*IQR, k=2,...,4
Density
CpG o/e
Cleaned data

Chlamys_farreri

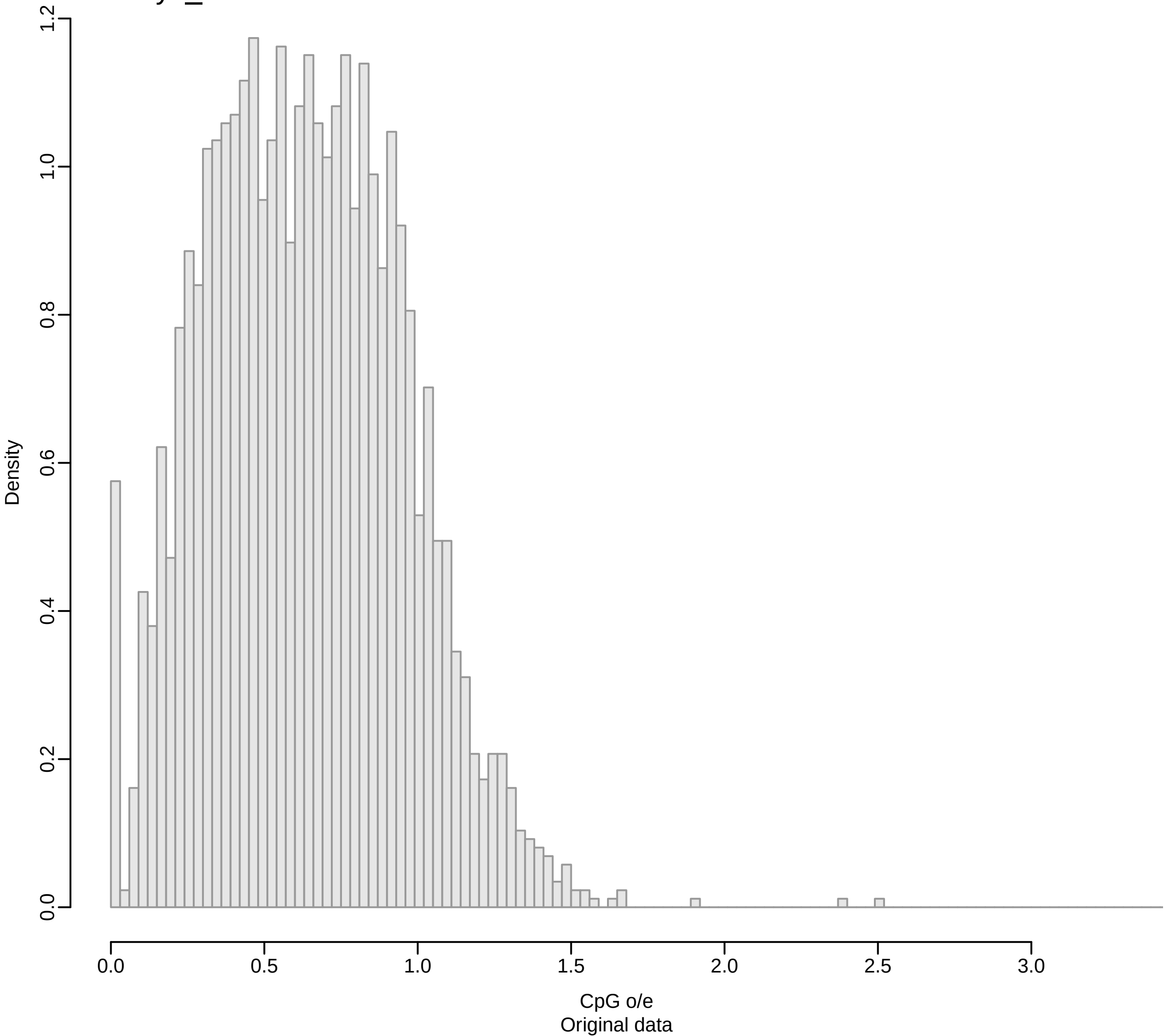

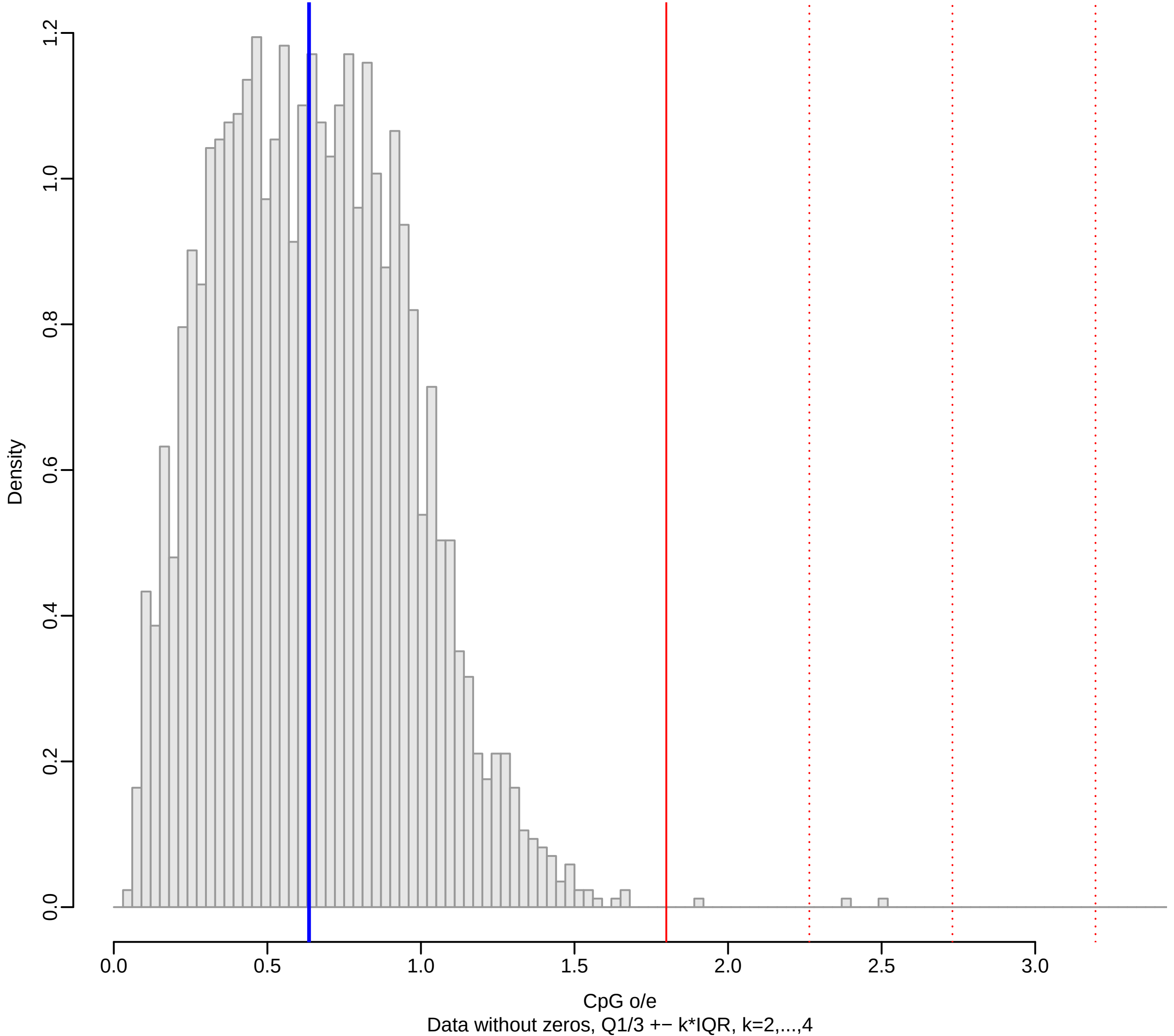

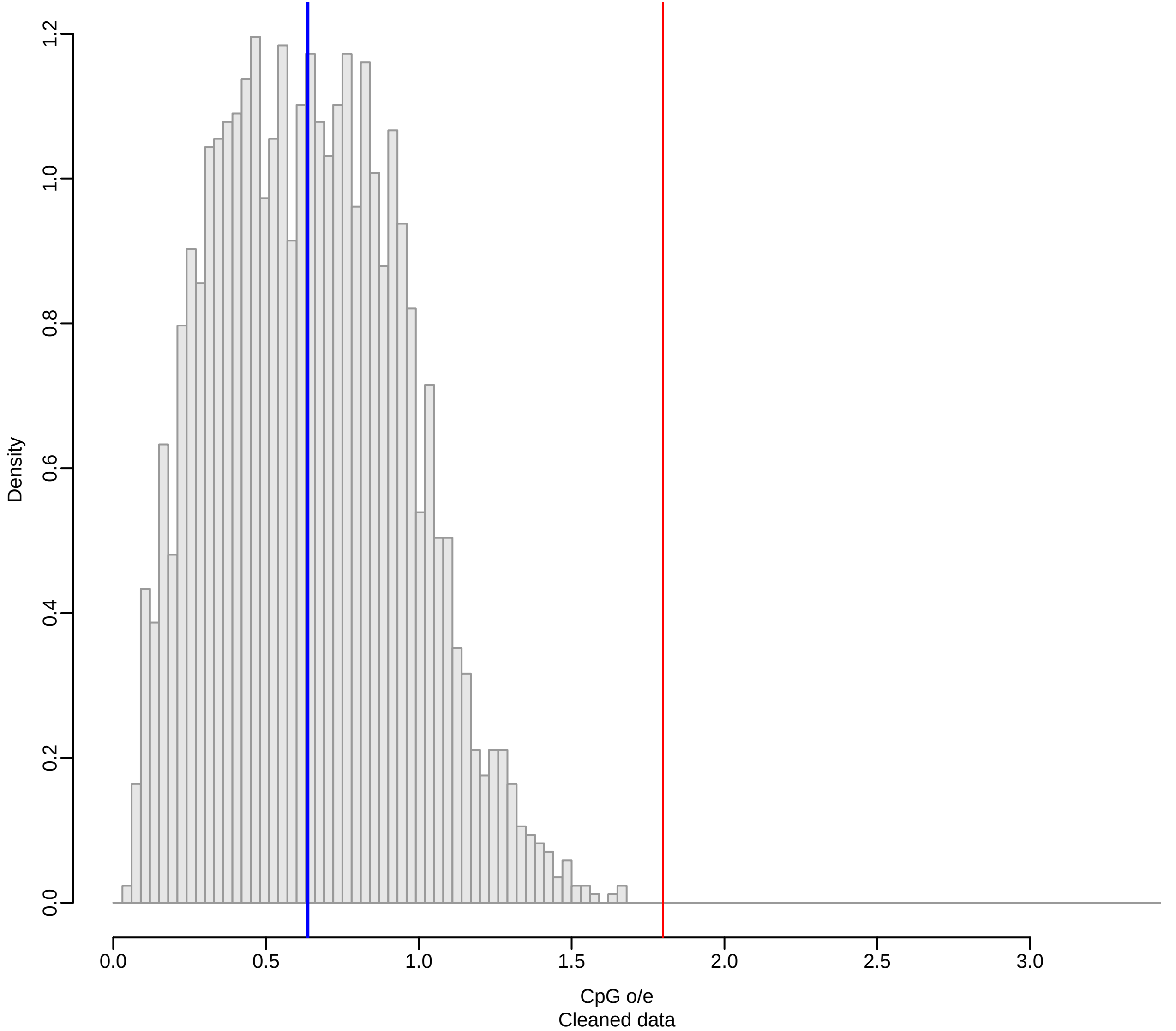

Chlorocebus_sabaeus

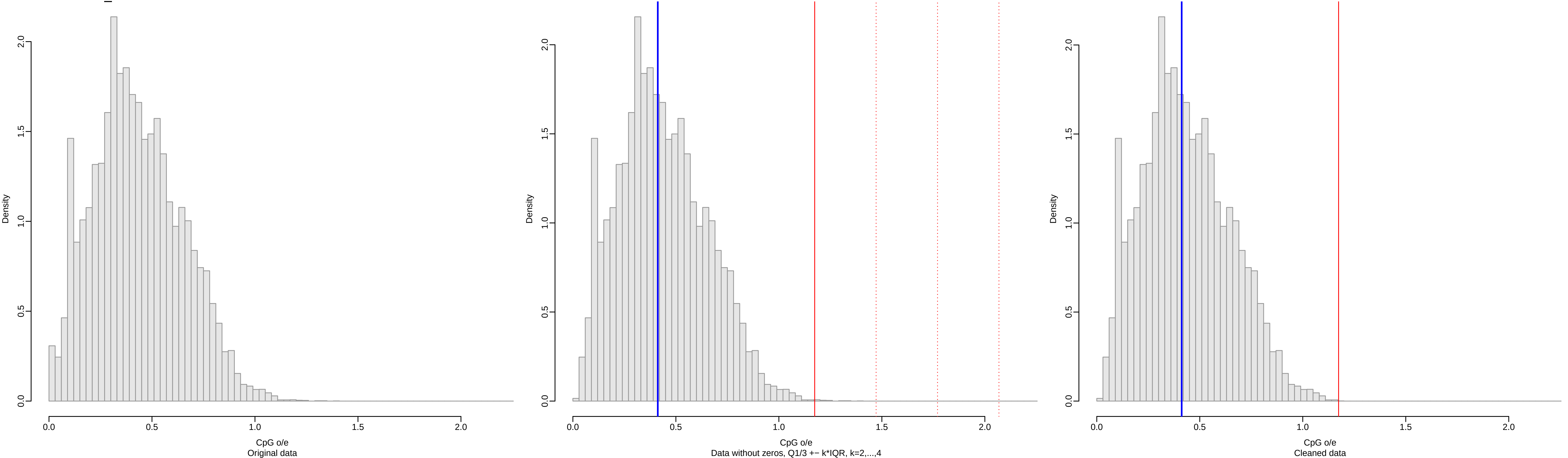

Chlorokybus_atmophyticus

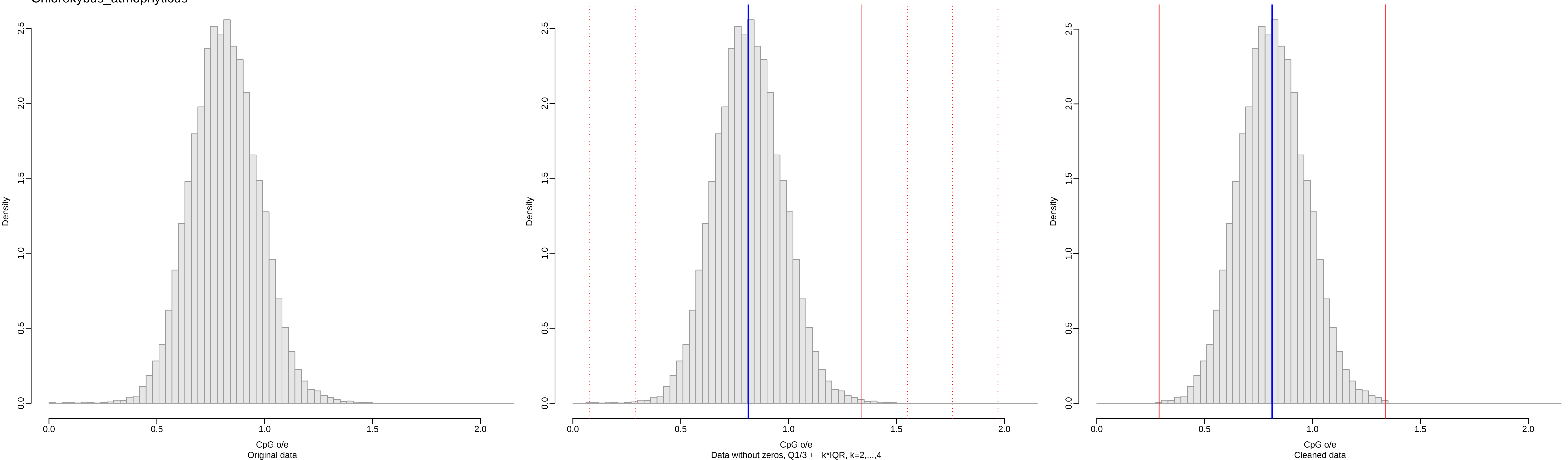

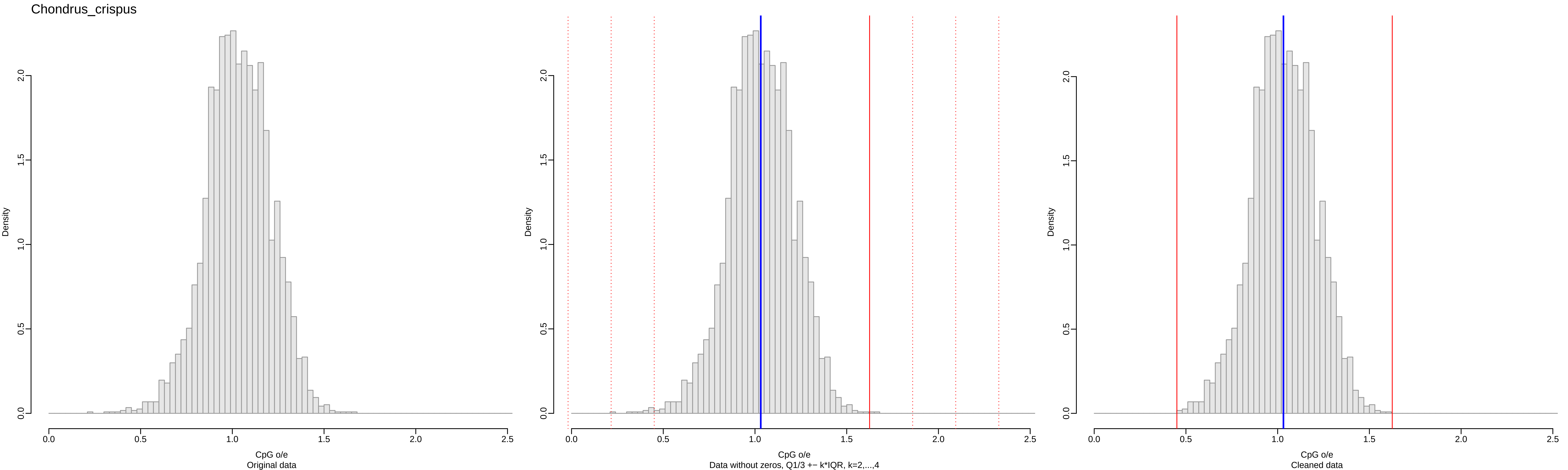

Chondrus_crispus
Density
CpG o/e
Original data
Density
CpG o/e
Data without zeros, Q1/3 +− k*IQR, k=2,...,4
Density
CpG o/e
Cleaned data

Choristoneura_fumiferana

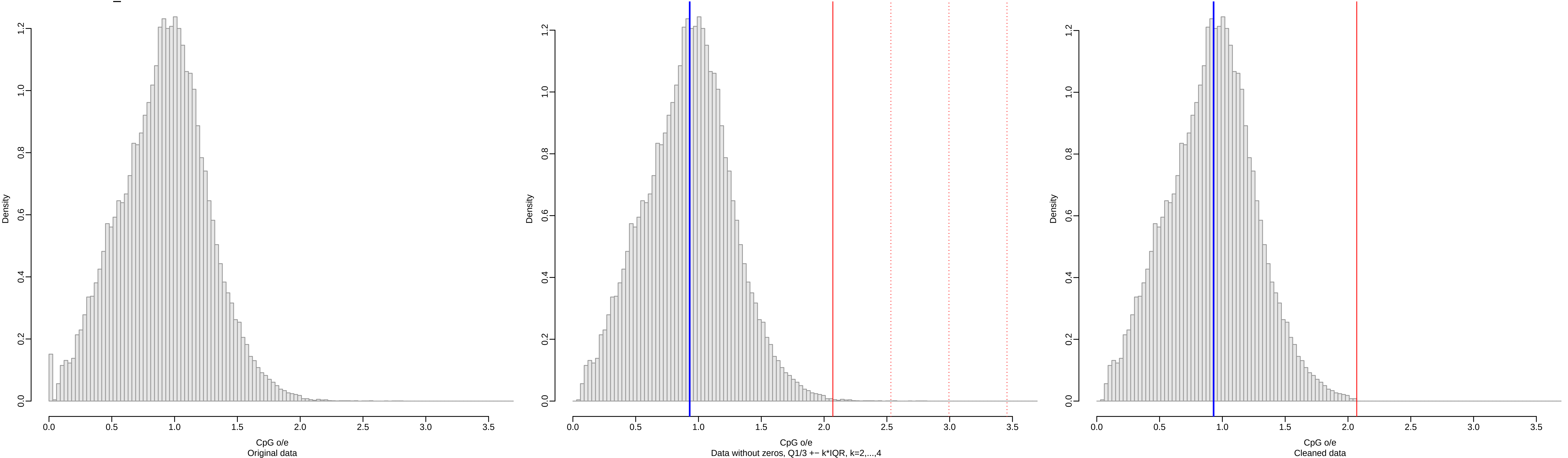

Cicer_arietinum

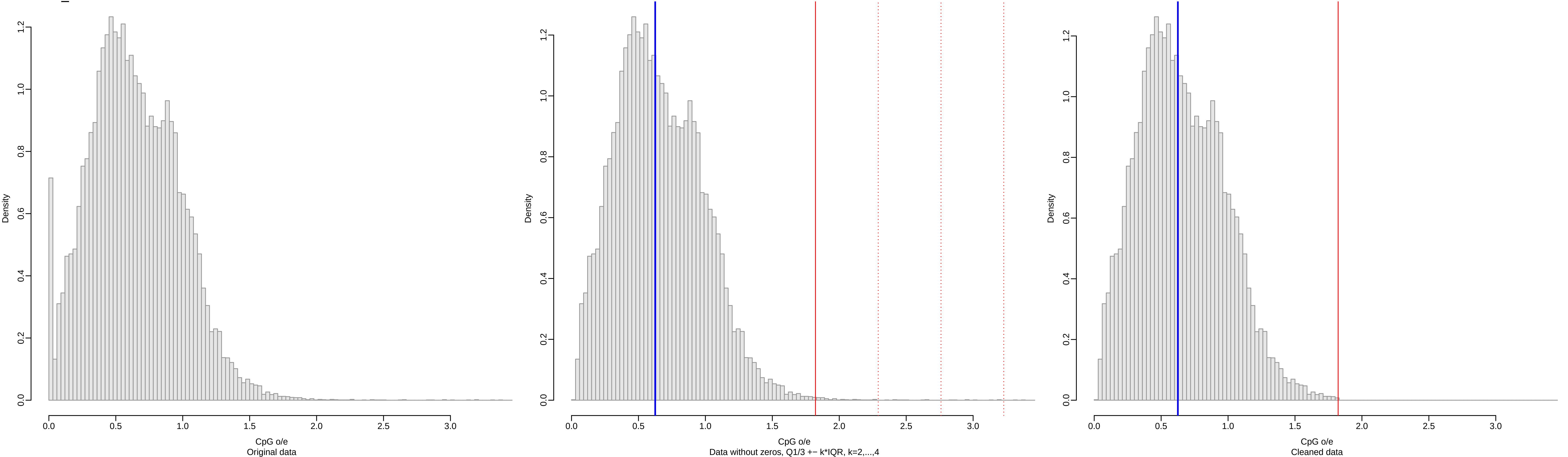

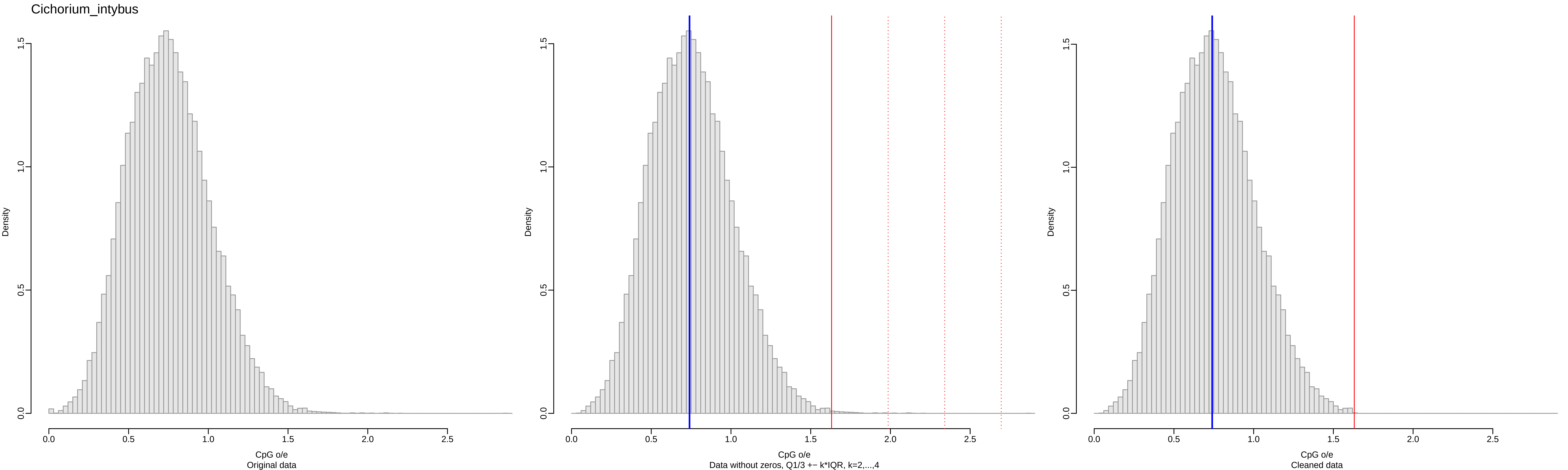

Cichorium_intybus
Density
CpG o/e
Original data
Data without zeros, Q1/3 +− k*IQR, k=2,...,4
Cleaned data
0.0
0.5
1.0
1.5
2.0
2.5

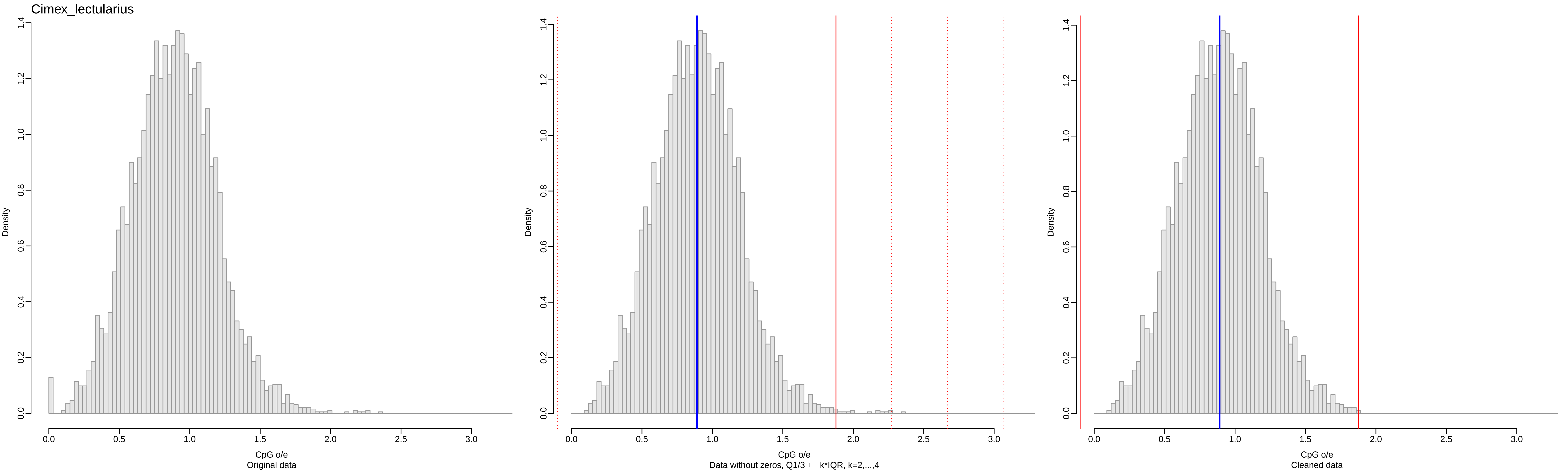

Cimex_lectularius
Density
CpG o/e
Original data
CpG o/e
Data without zeros, Q1/3 +− k*IQR, k=2,...,4
CpG o/e
Cleaned data

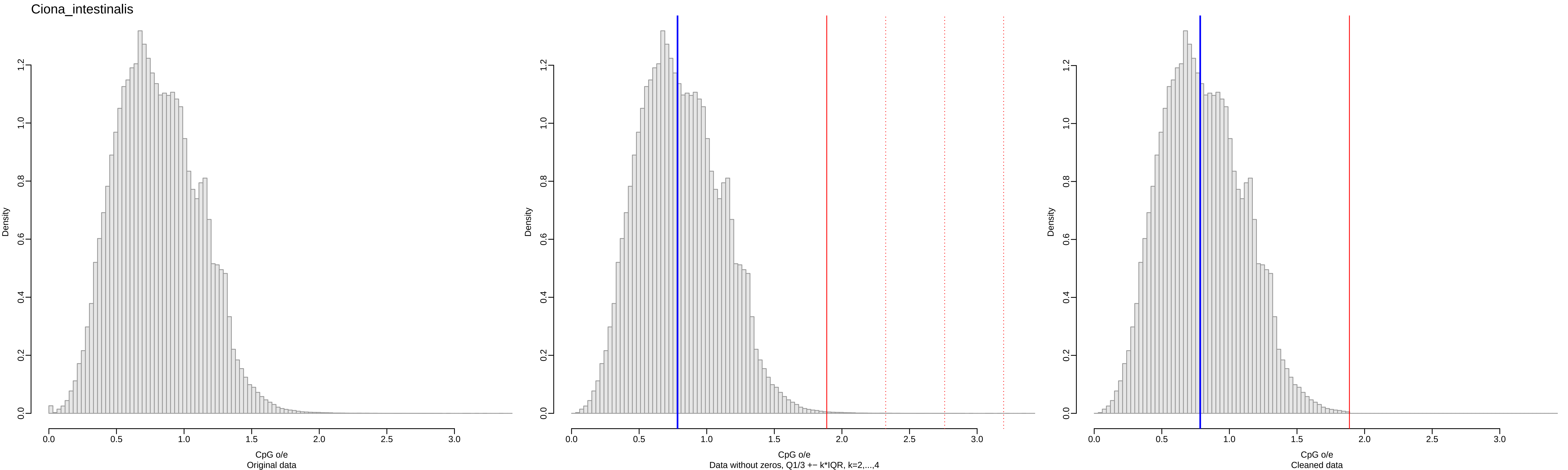

Ciona_intestinalis
Density
CpG o/e
Original data
Data without zeros, Q1/3 +− k*IQR, k=2,...,4
Cleaned data
0.0
0.2
0.4
0.6
0.8
1.0
1.2
0.5
1.5
2.0
2.5
3.0

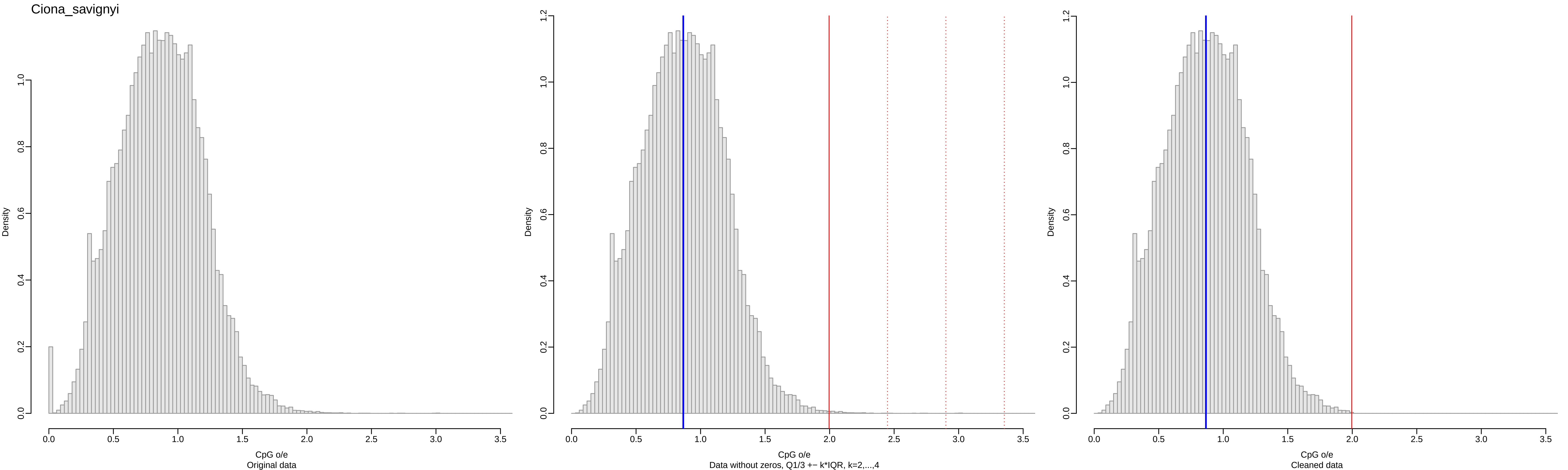

Ciona_savignyi
Density
CpG o/e
Original data
Density
CpG o/e
Data without zeros, Q1/3 +− k*IQR, k=2,...,4
Density
CpG o/e
Cleaned data

Citrus_aurantiifolia

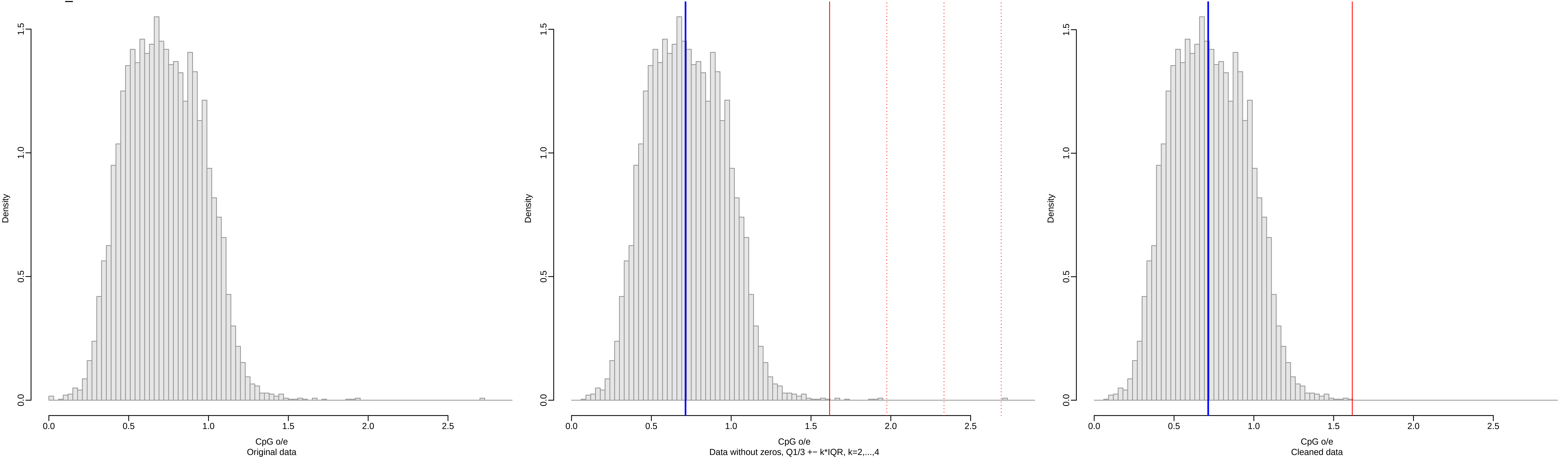

Citrus_aurantium

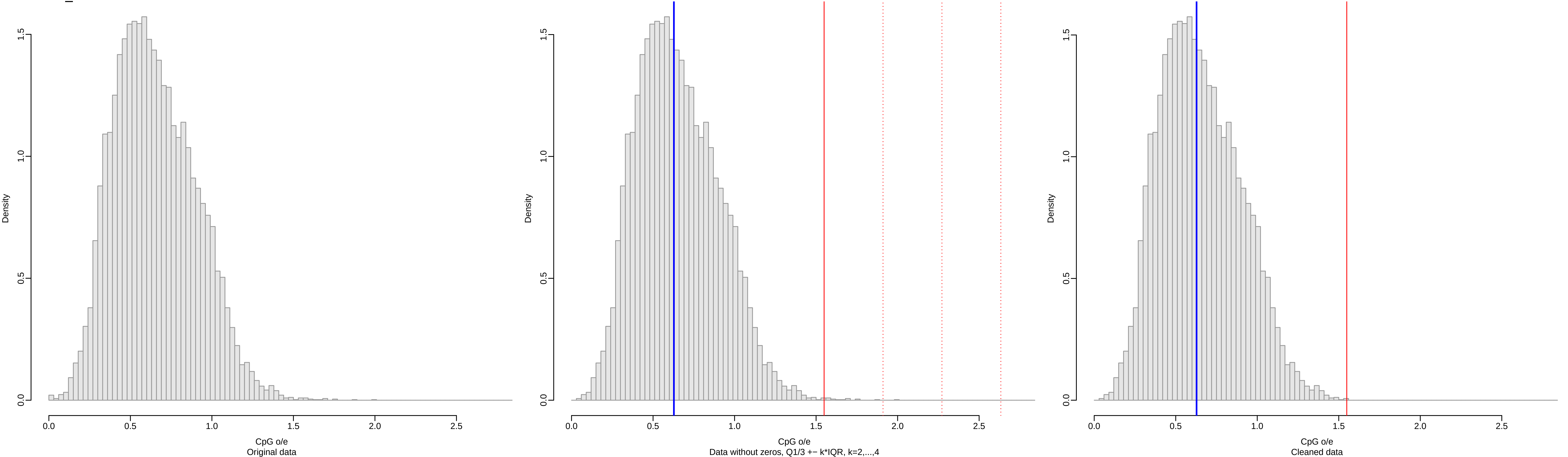

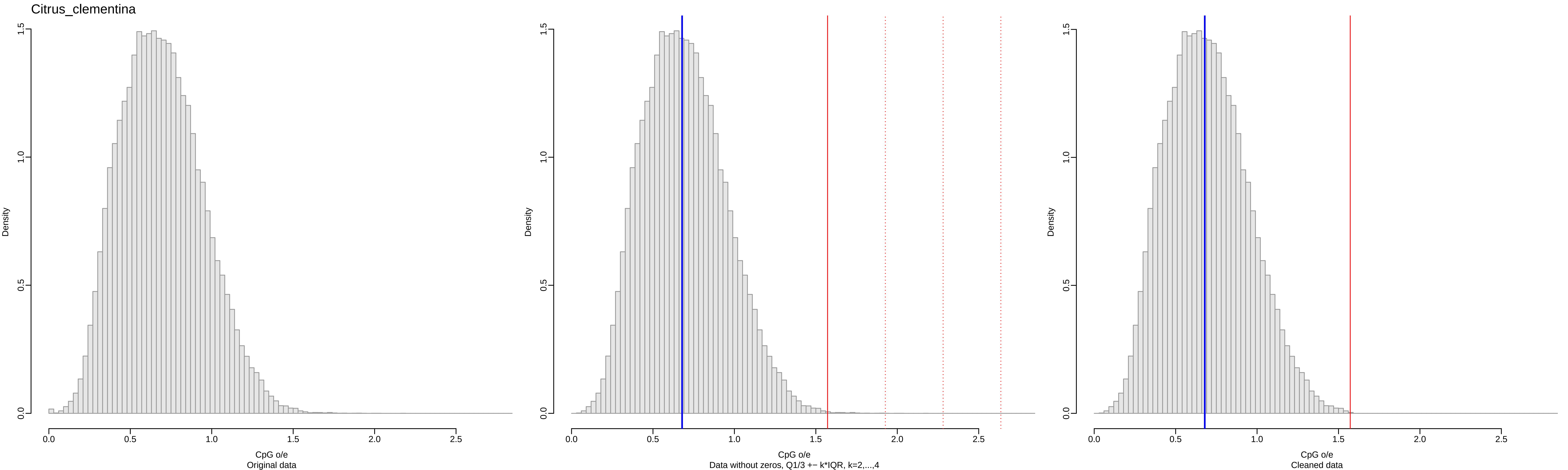
Citrus_clementina
Density
CpG o/e
Original data
Data without zeros, Q1/3 +− k*IQR, k=2,...,4
Cleaned data
0.0
0.5
1.0
1.5
2.0
2.5

Citrus_jambhiri

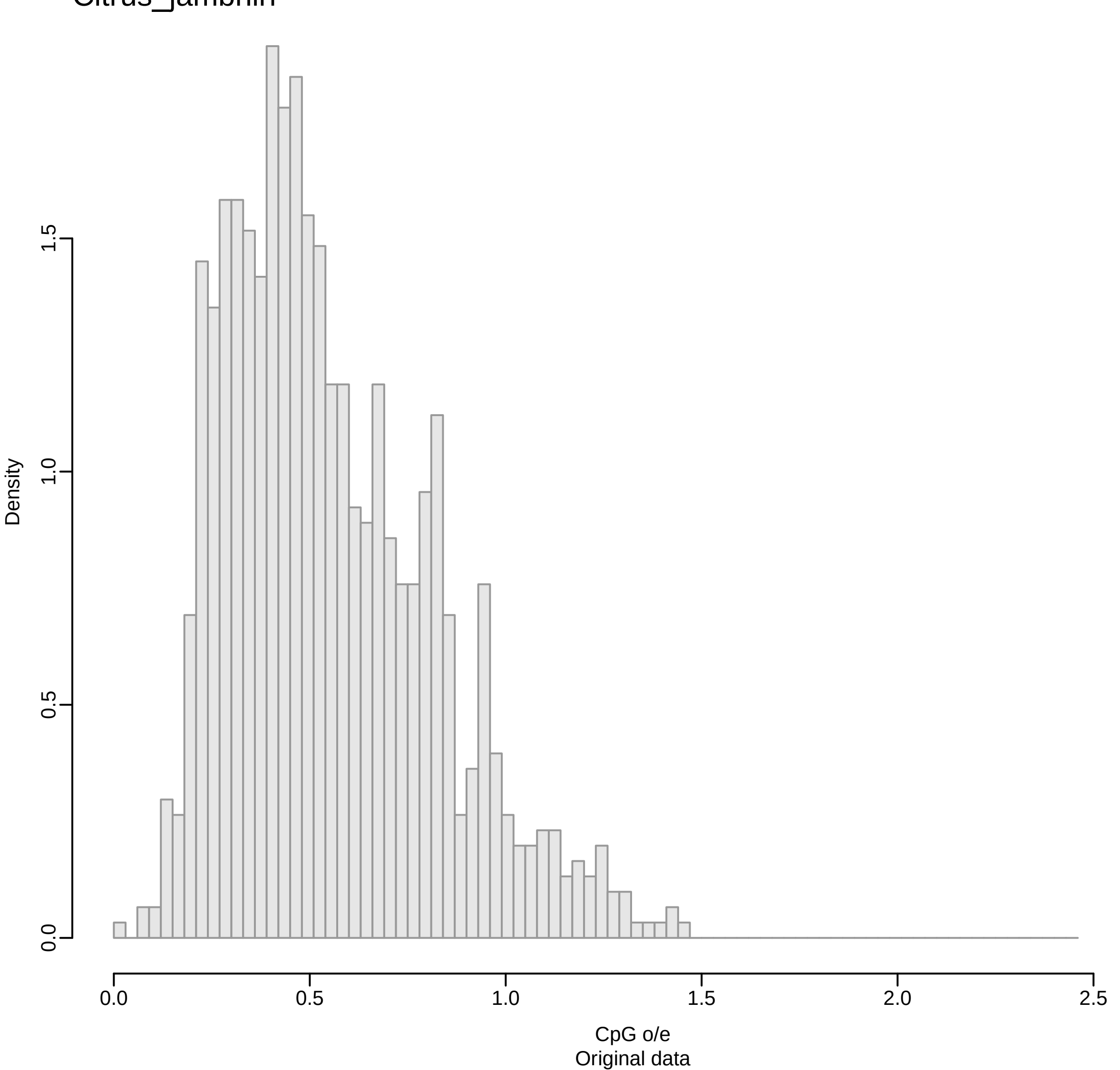

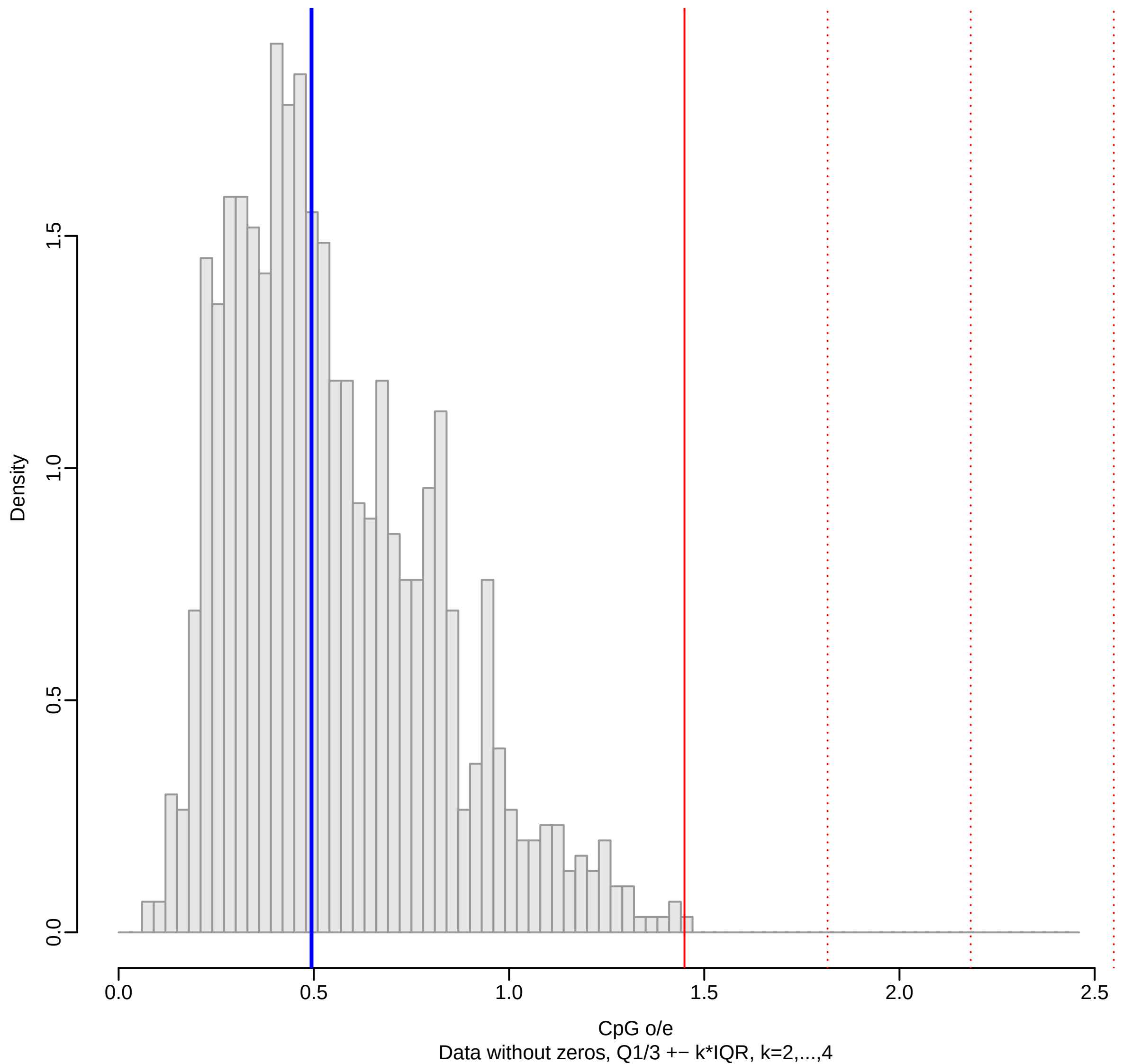

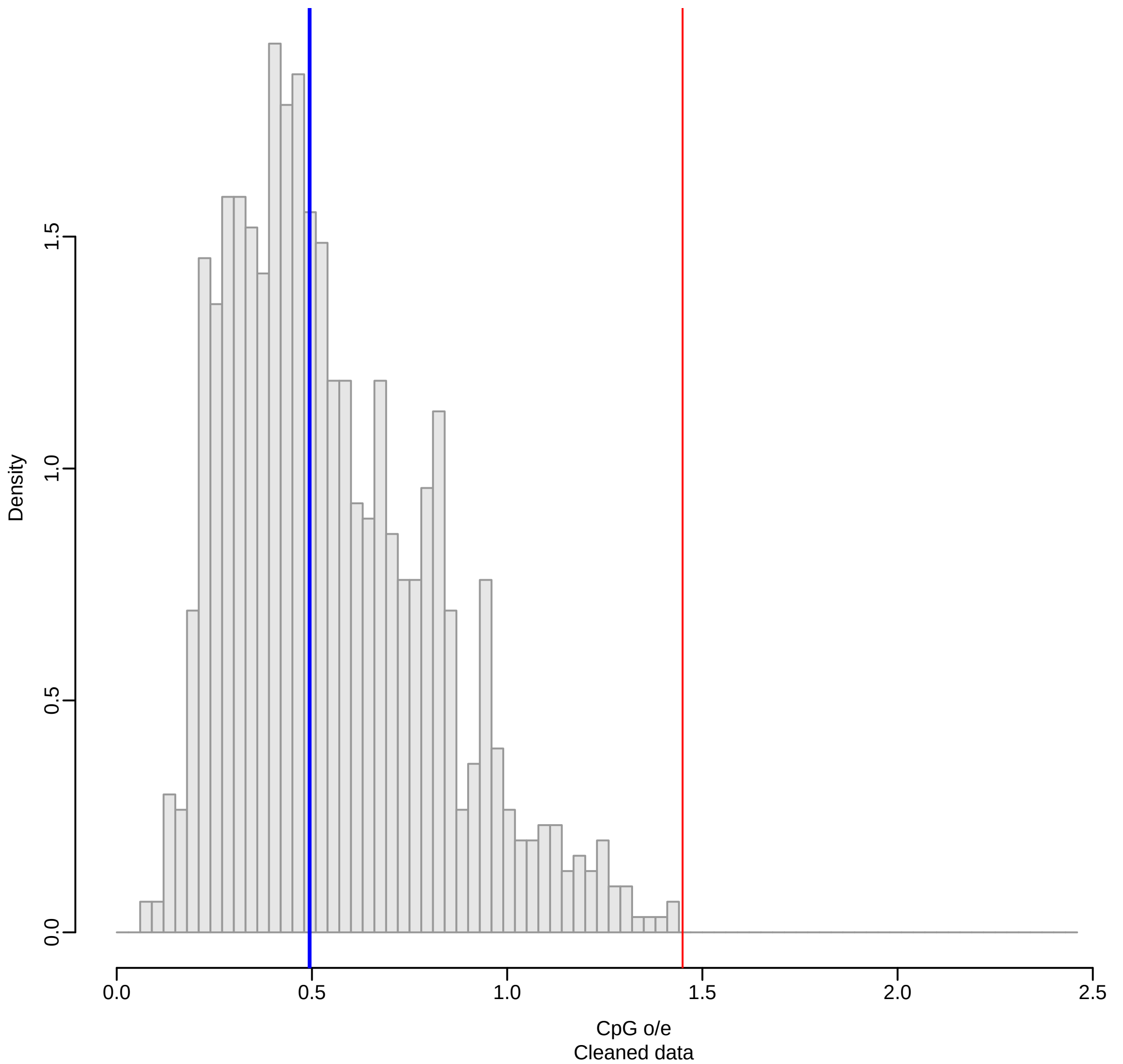

Citrus_latifolia

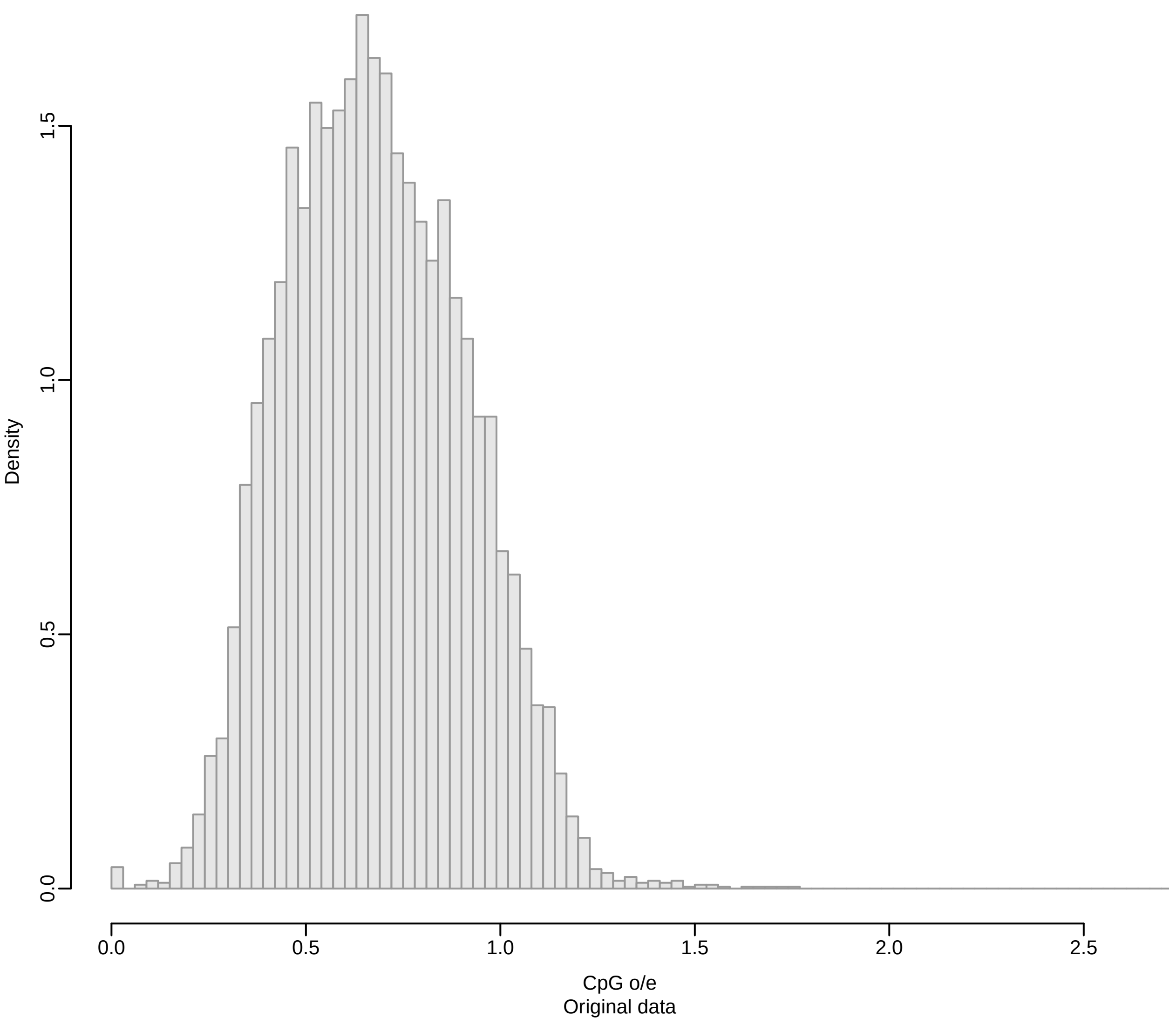

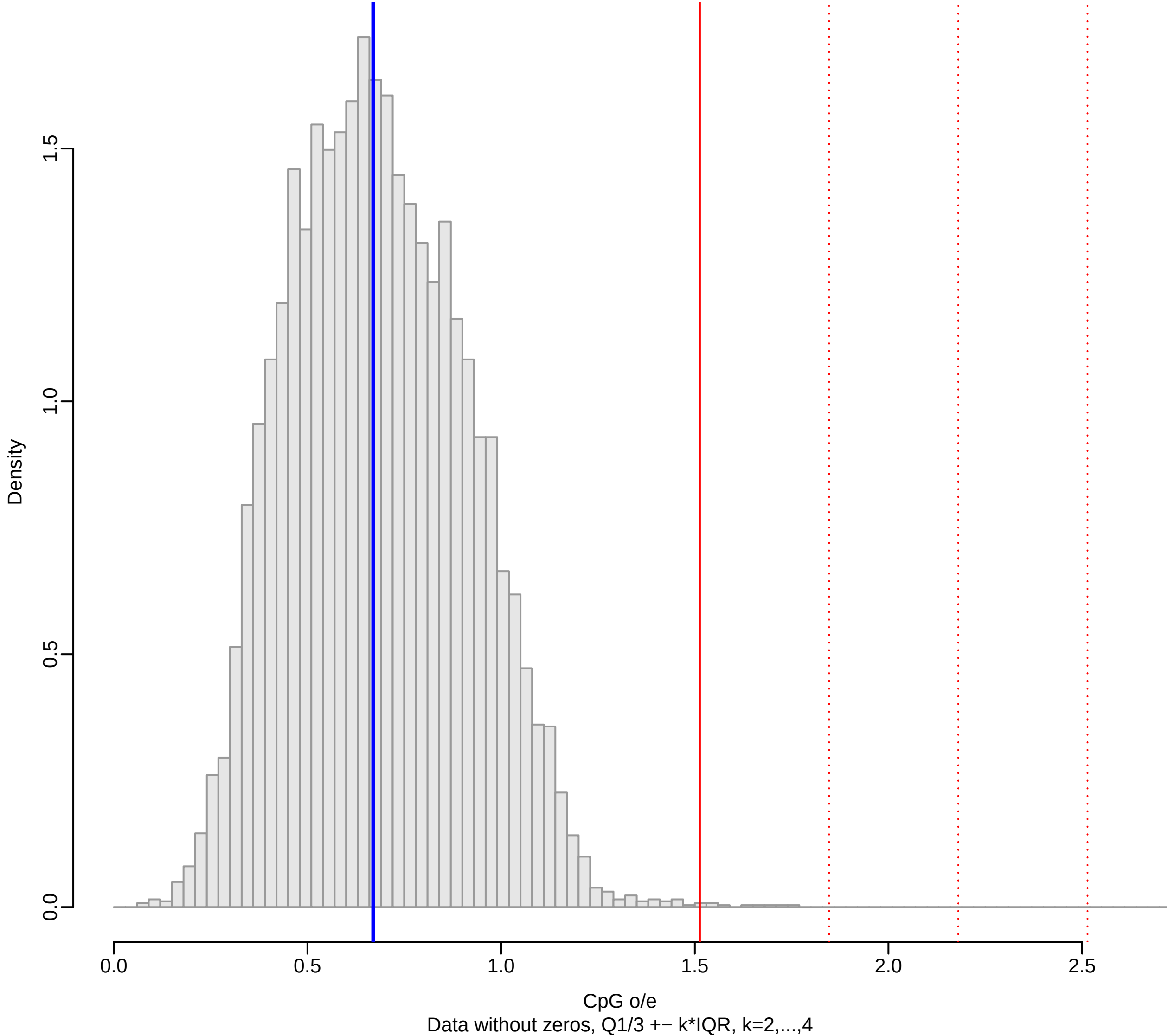

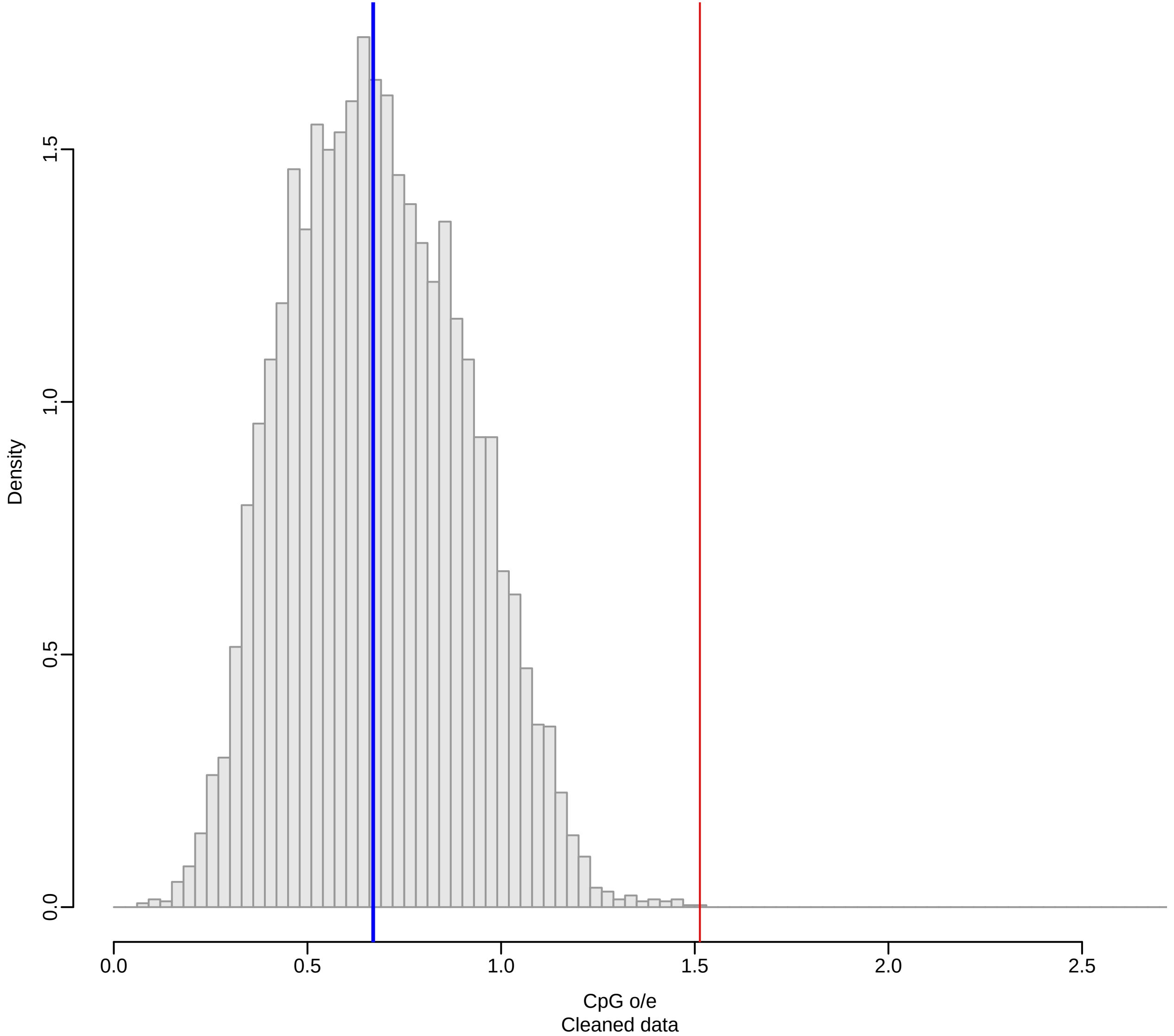

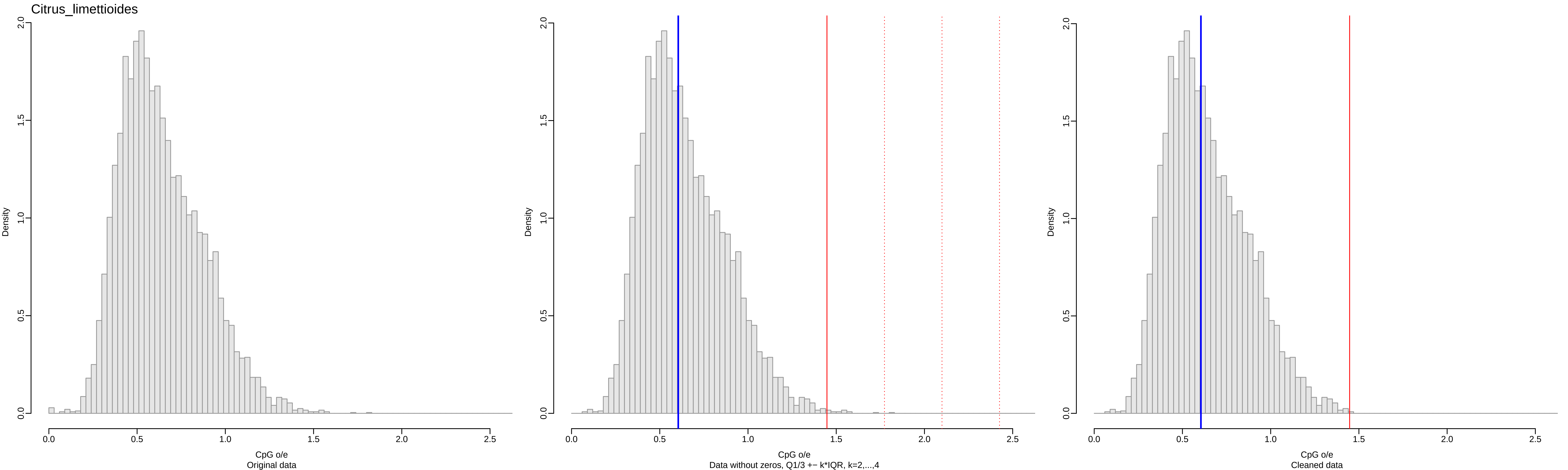
Citrus_limettioides
Density
CpG o/e
Original data
Data without zeros, Q1/3 +− k*IQR, k=2,...,4
Cleaned data
0.0
0.5
1.0
1.5
2.0
2.5

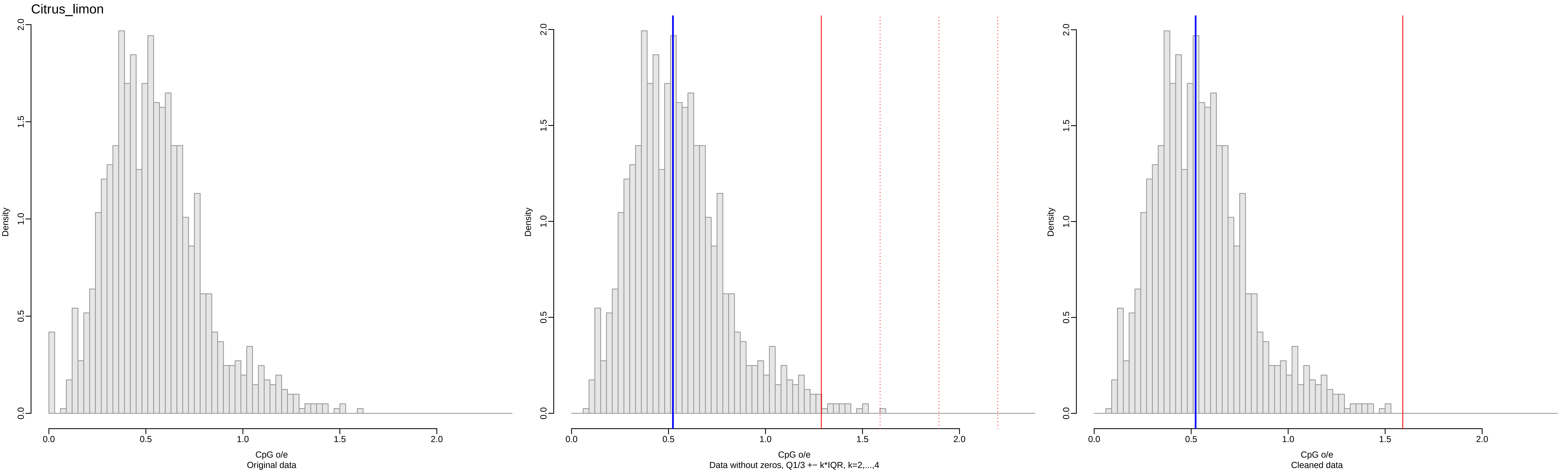

Citrus_limon
Density
CpG o/e
Original data
Density
CpG o/e
Data without zeros, Q1/3 +− k*IQR, k=2,...,4
Density
CpG o/e
Cleaned data
0.0
0.5
1.0
1.5
2.0

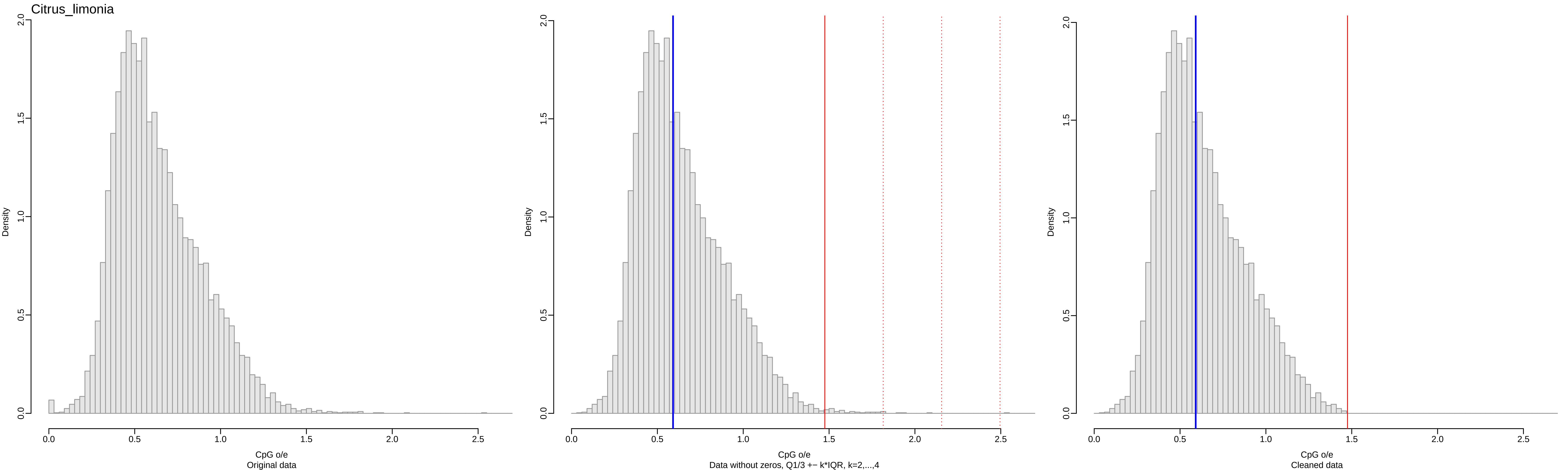

Citrus_limonia
Density
CpG o/e
Original data
Data without zeros, Q1/3 +− k*IQR, k=2,...,4
Cleaned data
0.0
0.5
1.0
1.5
2.0
2.5

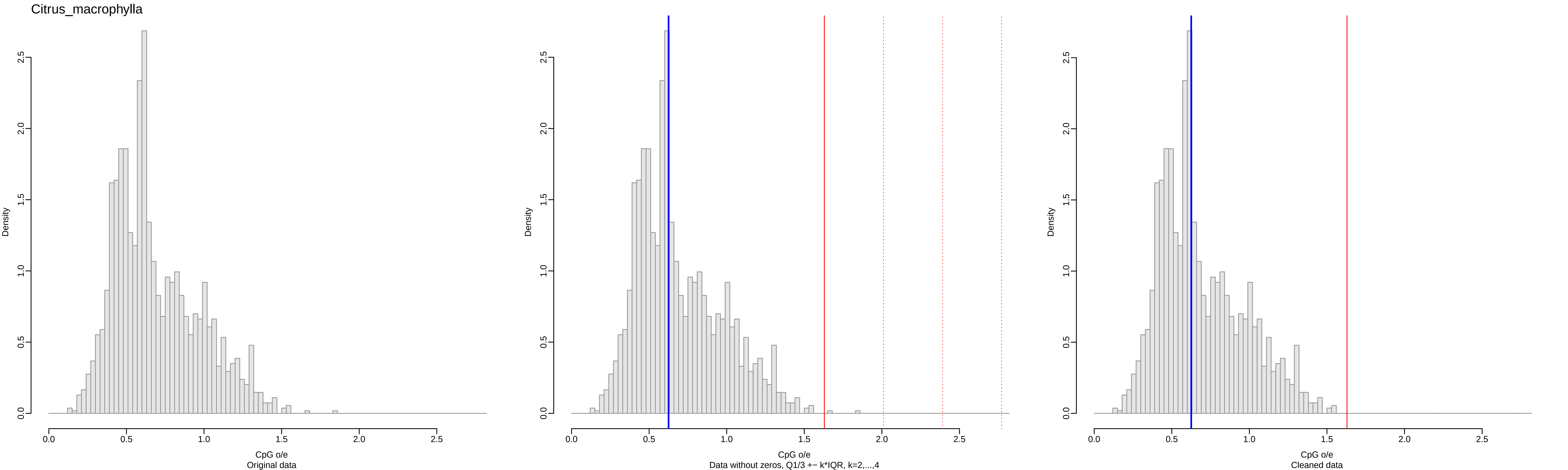

Citrus_macrophylla
Density
CpG o/e
Original data
Data without zeros, Q1/3 +− k*IQR, k=2,...,4
Cleaned data

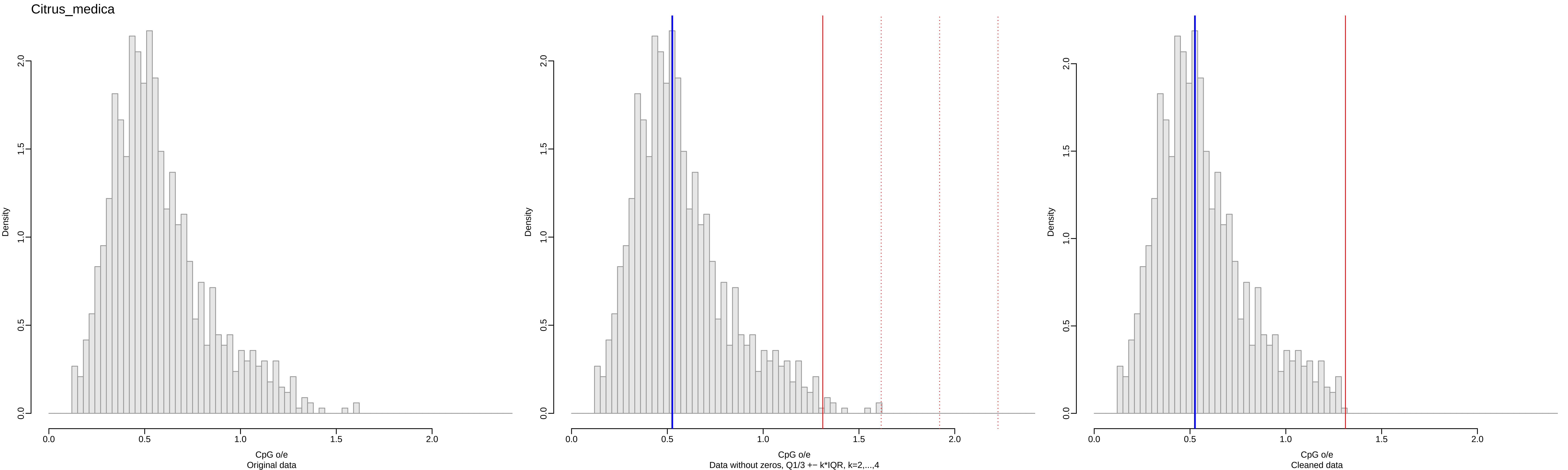

Citrus_medica
Density
CpG o/e
Original data
Data without zeros, Q1/3 +− k*IQR, k=2,...,4
Cleaned data

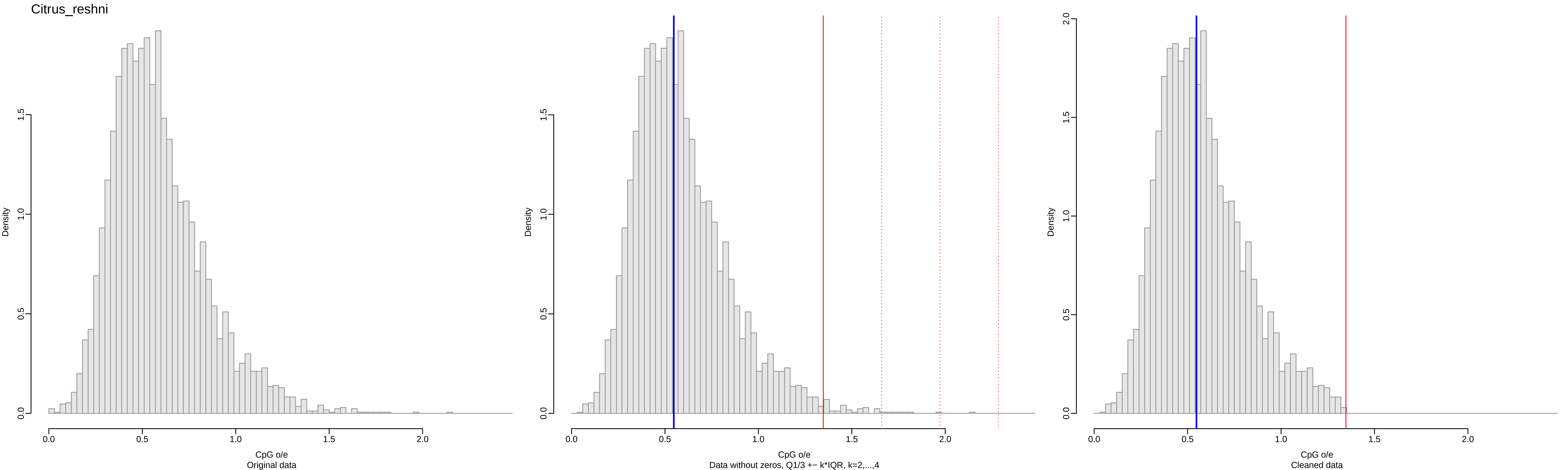

Citrus_reshni
Density
CpG o/e
Original data
Data without zeros, Q1/3 +− k*IQR, k=2,...,4
Cleaned data
0.0
0.5
1.0
1.5
2.0

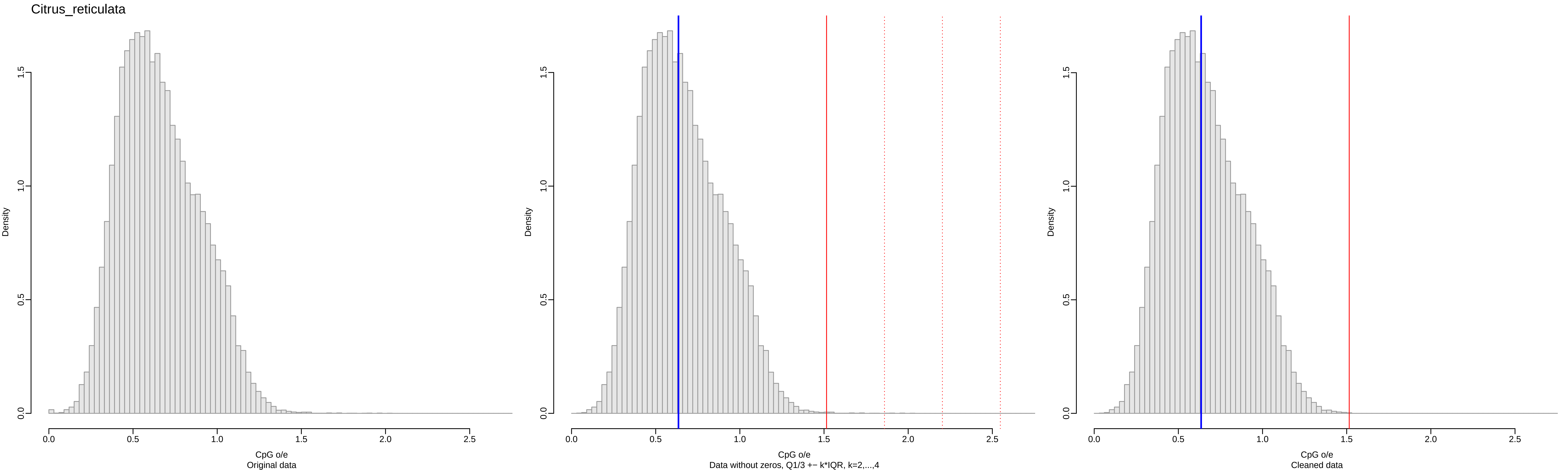
Citrus_reticulata
Density
CpG o/e
Original data
Data without zeros, Q1/3 +− k*IQR, k=2,...,4
Cleaned data
0.0
0.5
1.0
1.5
2.0
2.5

Citrus_sinensis

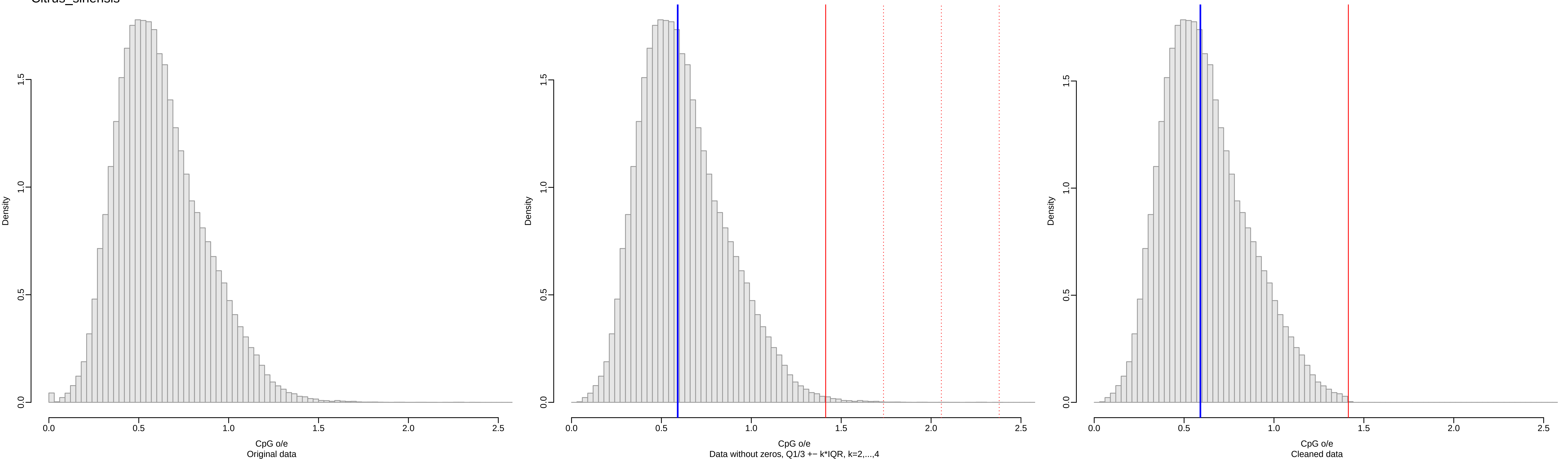

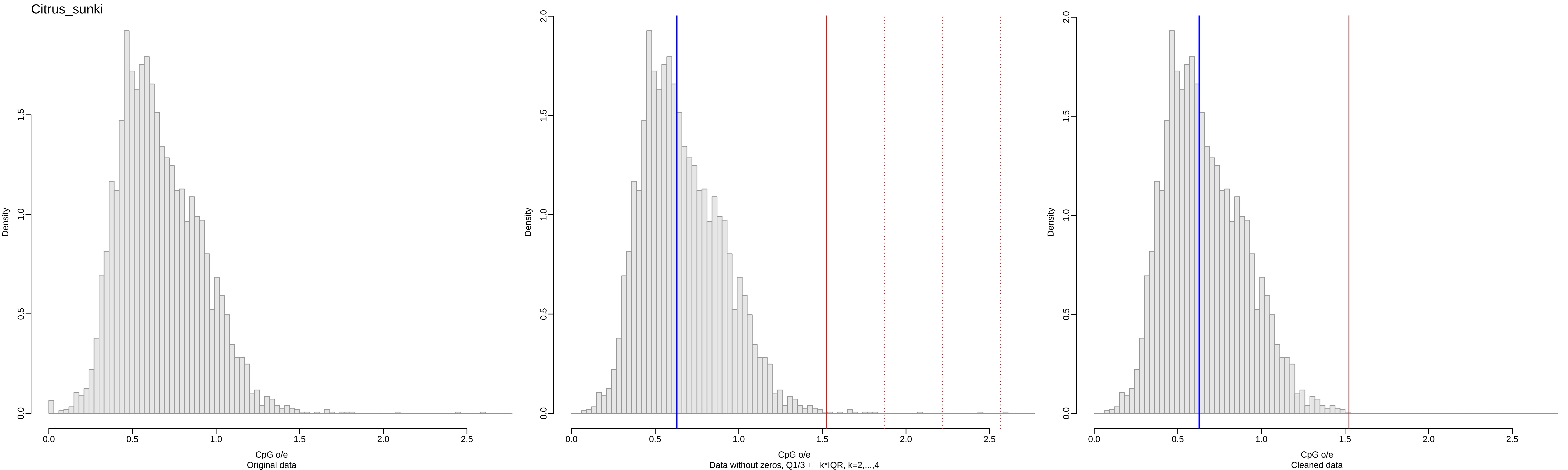

Citrus_sunki
Density
CpG o/e
Original data
CpG o/e
Data without zeros, Q1/3 +− k*IQR, k=2,...,4
CpG o/e
Cleaned data

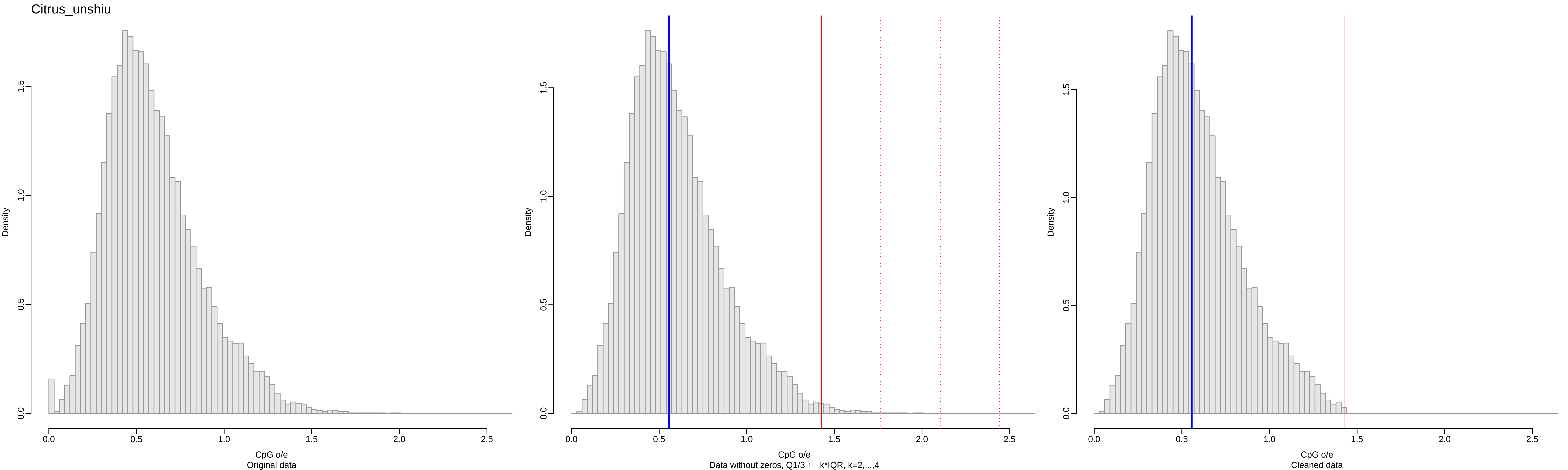

Citrus_unshiu
Density
CpG o/e
Original data
Data without zeros, Q1/3 +− k*IQR, k=2,...,4
Cleaned data

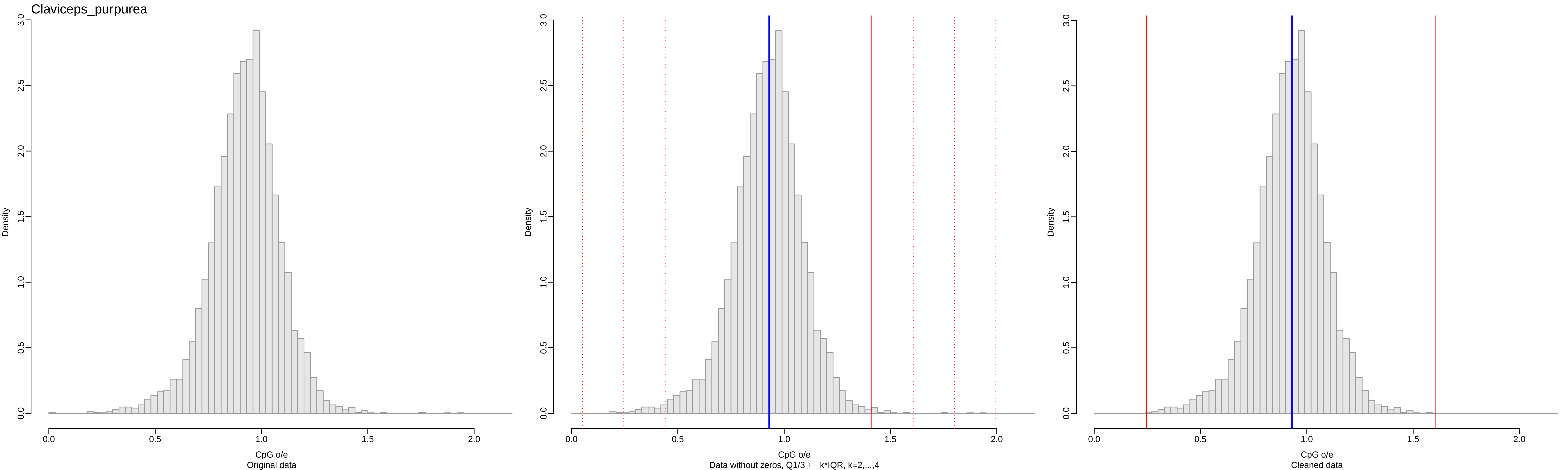

Claviceps_purpurea
Density
CpG o/e
Original data
Data without zeros, Q1/3 +− k*IQR, k=2,...,4
Cleaned data

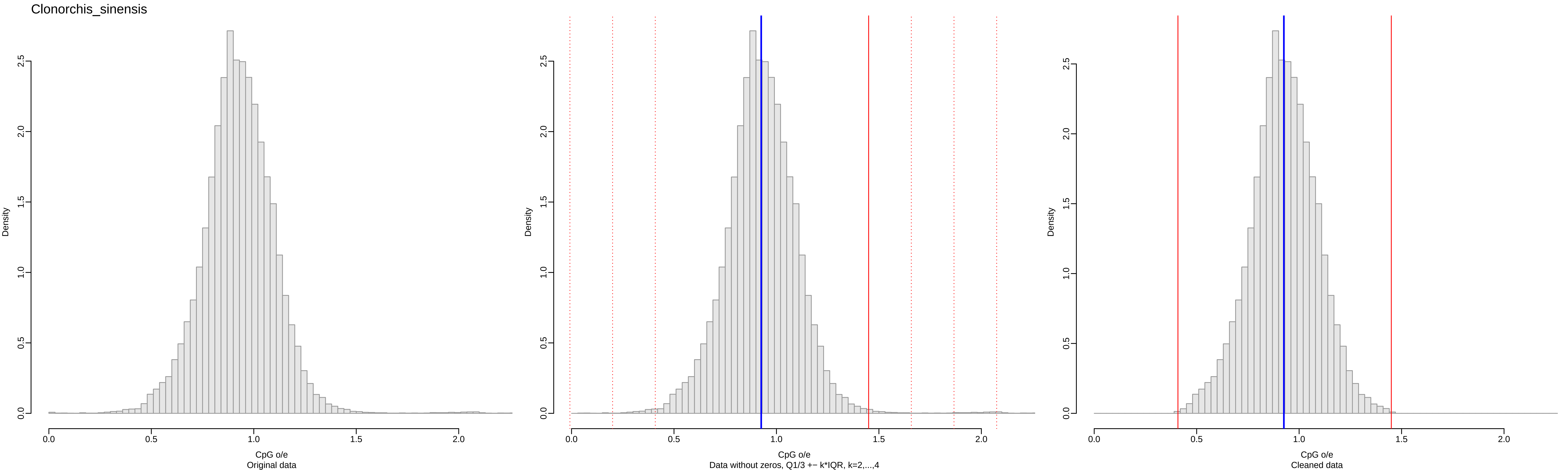
Clonorchis_sinensis
Density
CpG o/e
Original data
Data without zeros, Q1/3 +− k*IQR, k=2,...,4
Cleaned data
0.0
0.5
1.0
1.5
2.0
2.5

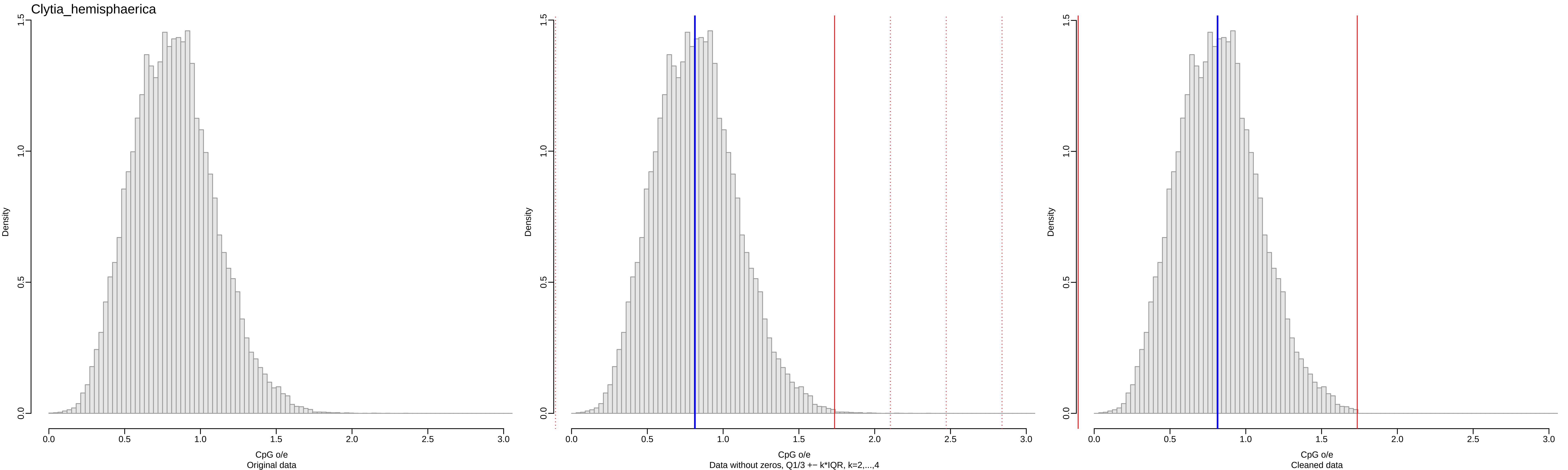
Clytia_hemisphaerica
Density
CpG o/e
Original data
Data without zeros, Q1/3 +− k*IQR, k=2,...,4
Cleaned data
0.0
0.5
1.0
1.5
2.0
2.5
3.0

Coccidioides_posadasii

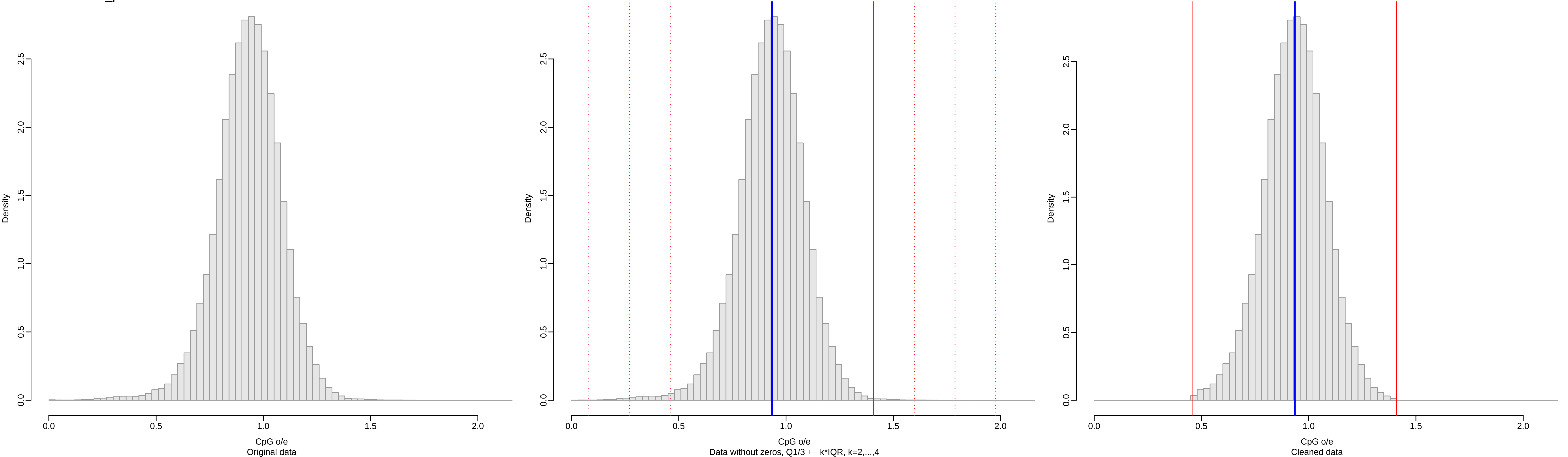

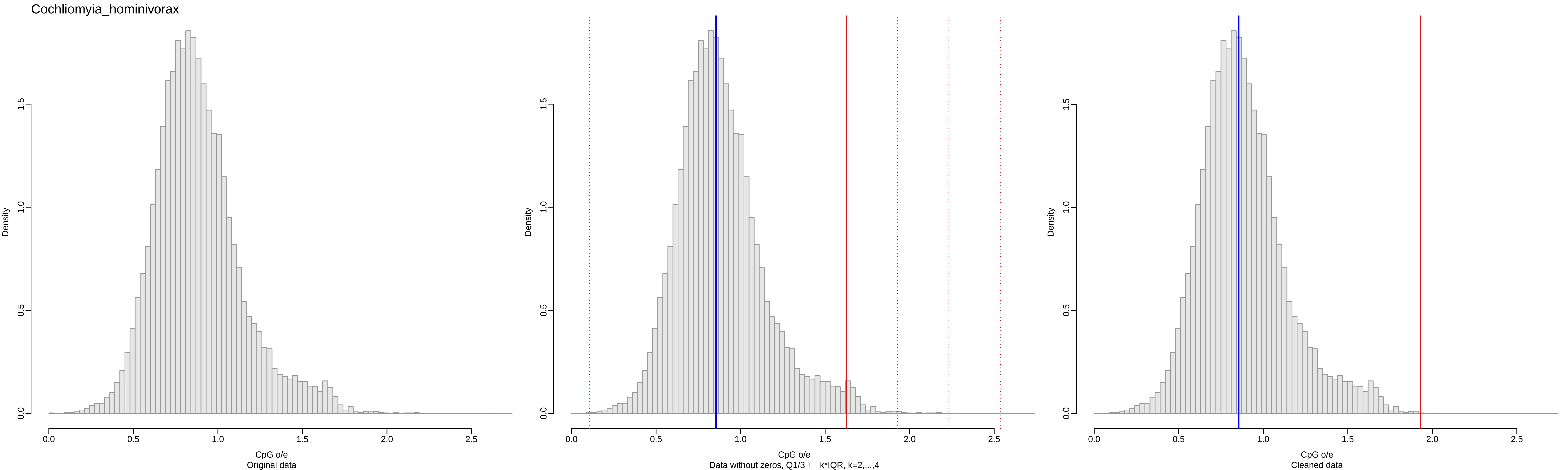
Cochliomyia_hominivorax
Density
0.0
0.5
1.0
1.5
0.0
0.5
1.0
1.5
2.0
2.5
CpG o/e
Original data
Data without zeros, Q1/3 +− k*IQR, k=2,...,4
Cleaned data

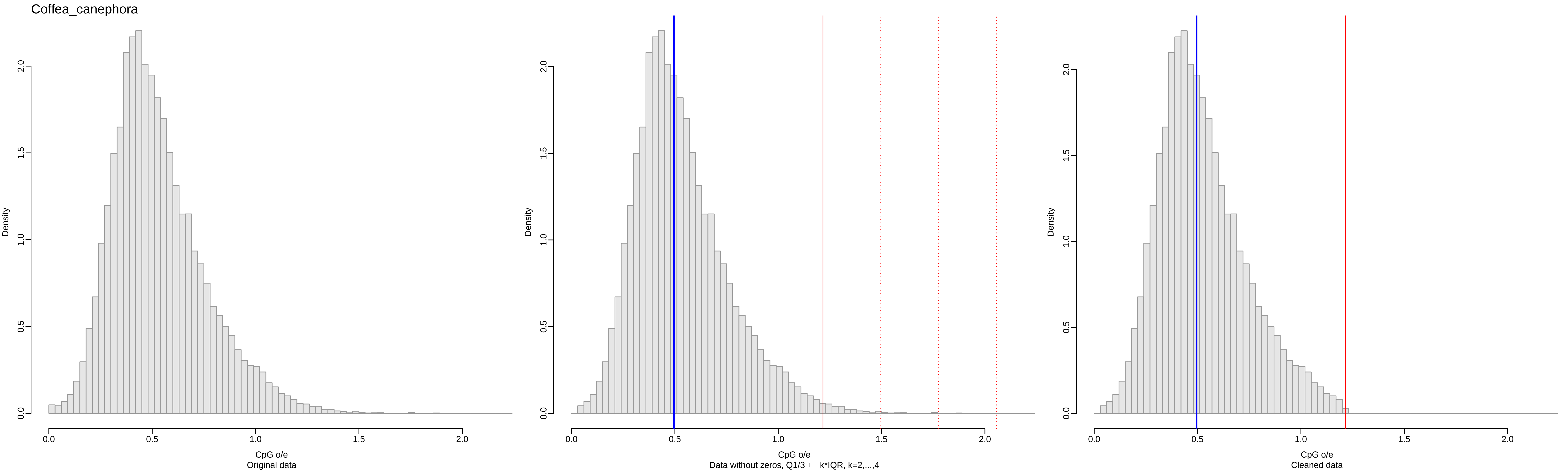

Coffea_canephora
Density
CpG o/e
Original data
Data without zeros, Q1/3 +− k*IQR, k=2,...,4
Cleaned data

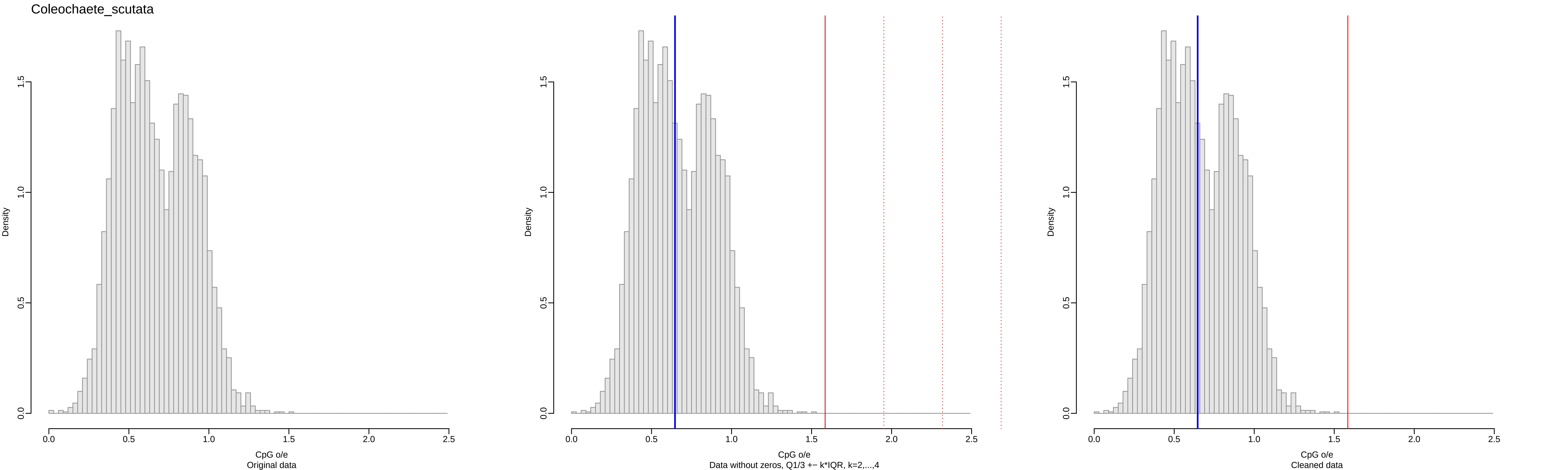

Coleochaete_scutata
Density
CpG o/e
Original data
Density
CpG o/e
Data without zeros, Q1/3 +− k*IQR, k=2,...,4
Density
CpG o/e
Cleaned data

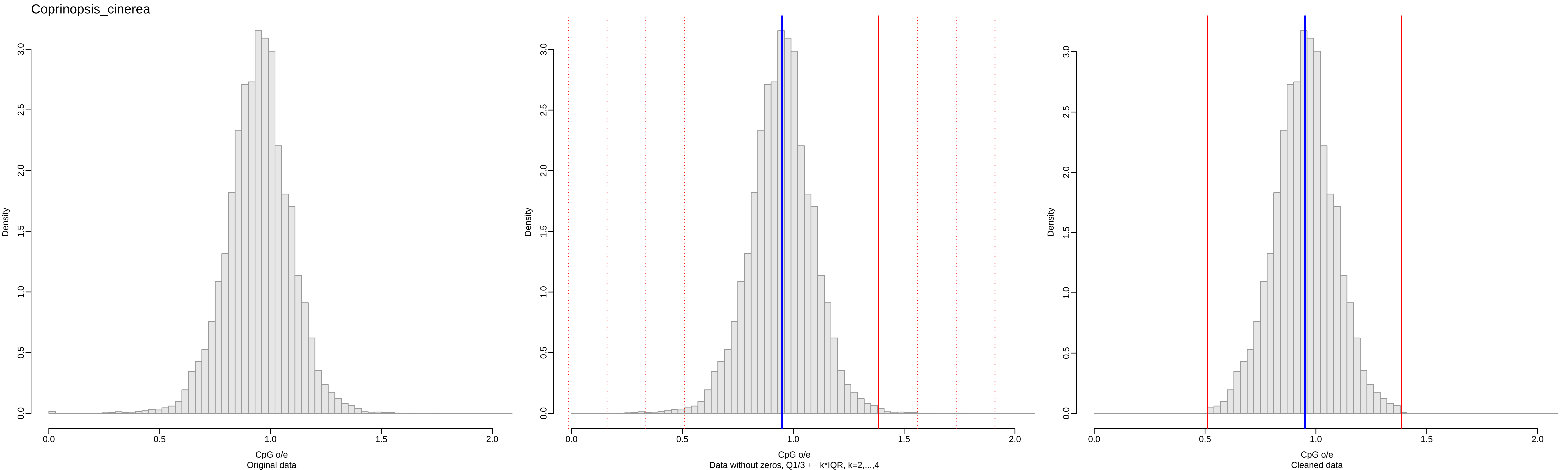

Coprinopsis_cinerea
Density
CpG o/e
Original data
Density
CpG o/e
Data without zeros, Q1/3 +− k*IQR, k=2,...,4
Density
CpG o/e
Cleaned data

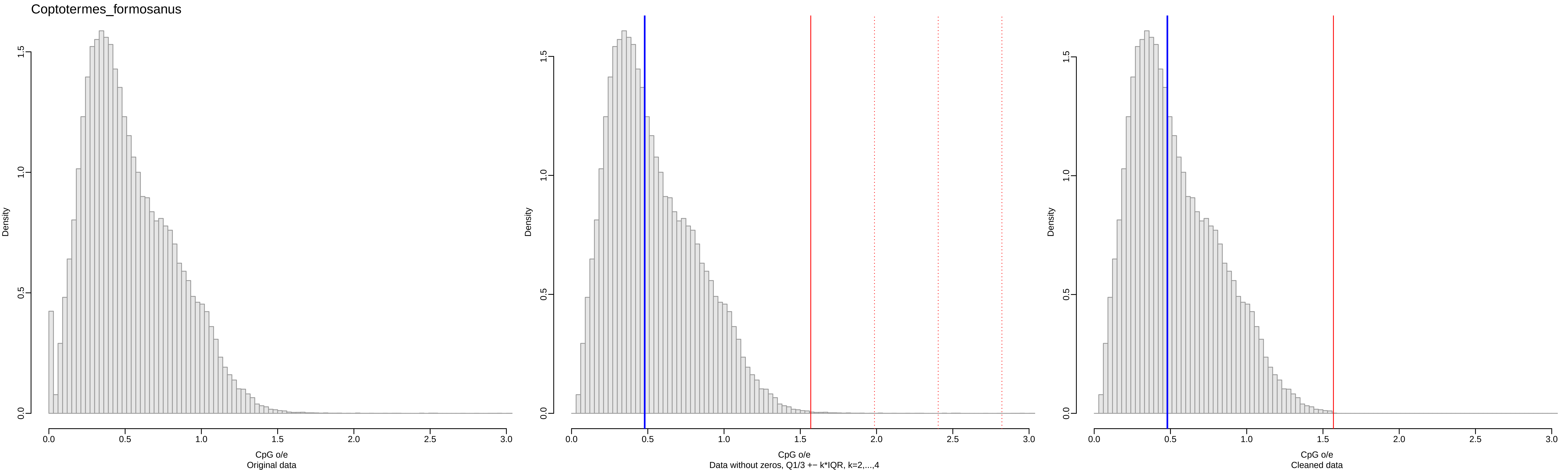

Coptotermes_formosanus
Density
CpG o/e
Original data
Data without zeros, Q1/3 +− k*IQR, k=2,...,4
Cleaned data
0.0
0.5
1.0
1.5
2.0
2.5
3.0

Cordyceps_militaris

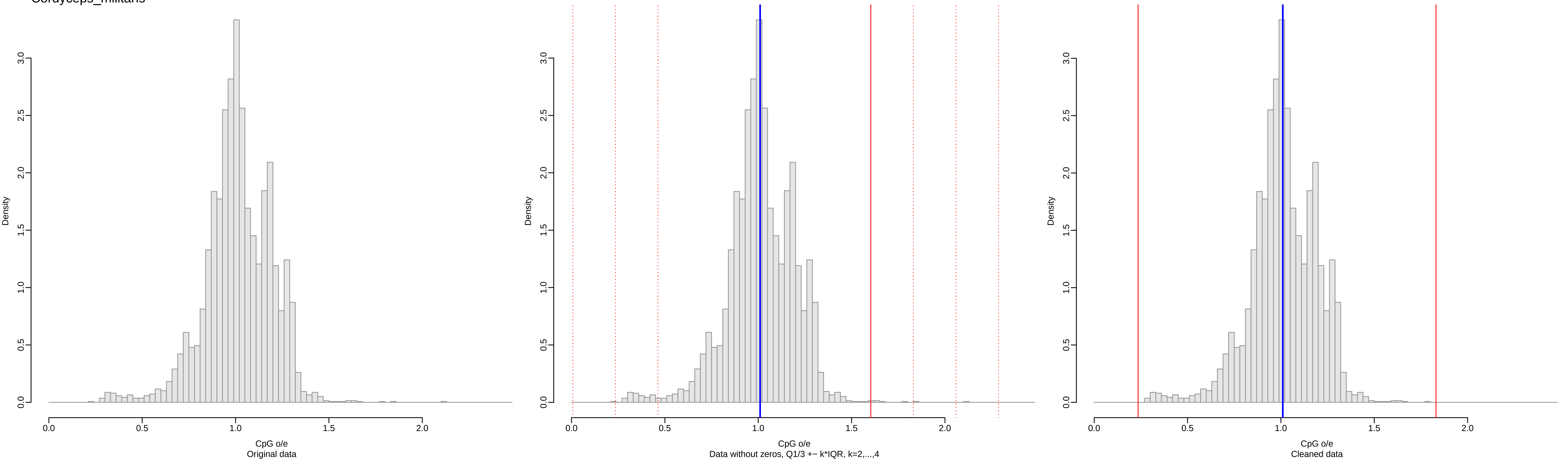

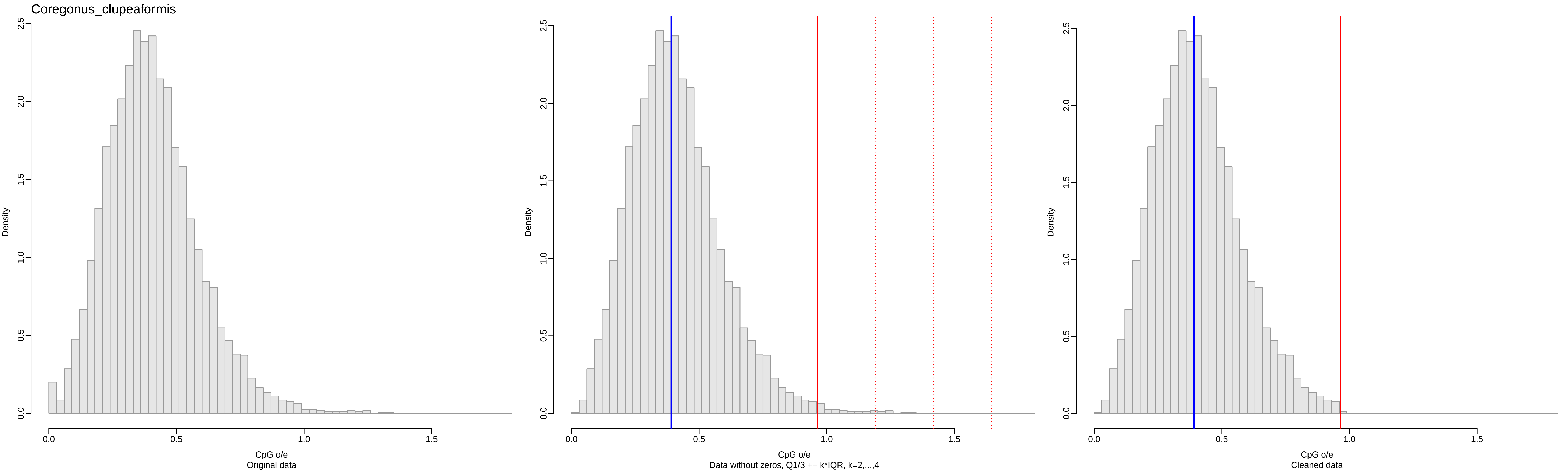
Coregonus_clupeaformis
Density
0.0
0.5
1.0
1.5
2.0
2.5
CpG o/e
Original data
Density
CpG o/e
Data without zeros, Q1/3 +− k*IQR, k=2,...,4
Density
CpG o/e
Cleaned data

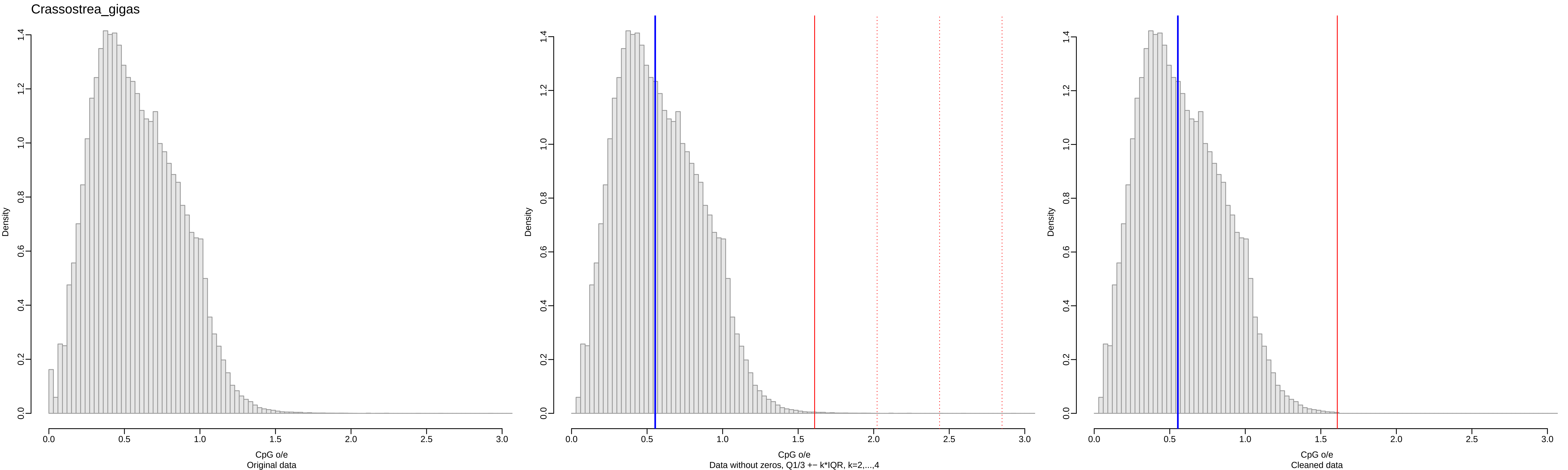
Crassostrea_gigas
Density
CpG o/e
Original data
Data without zeros, Q1/3 +− k*IQR, k=2,...,4
Cleaned data

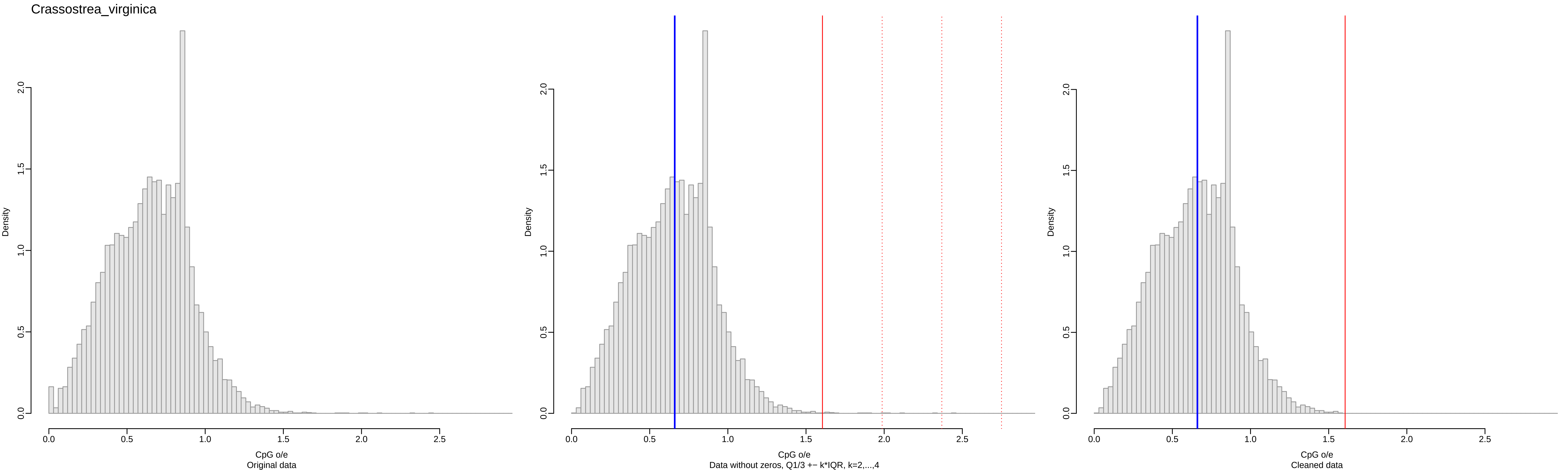
Crassostrea_virginica
Density
CpG o/e
Original data
Data without zeros, Q1/3 +− k*IQR, k=2,...,4
Cleaned data

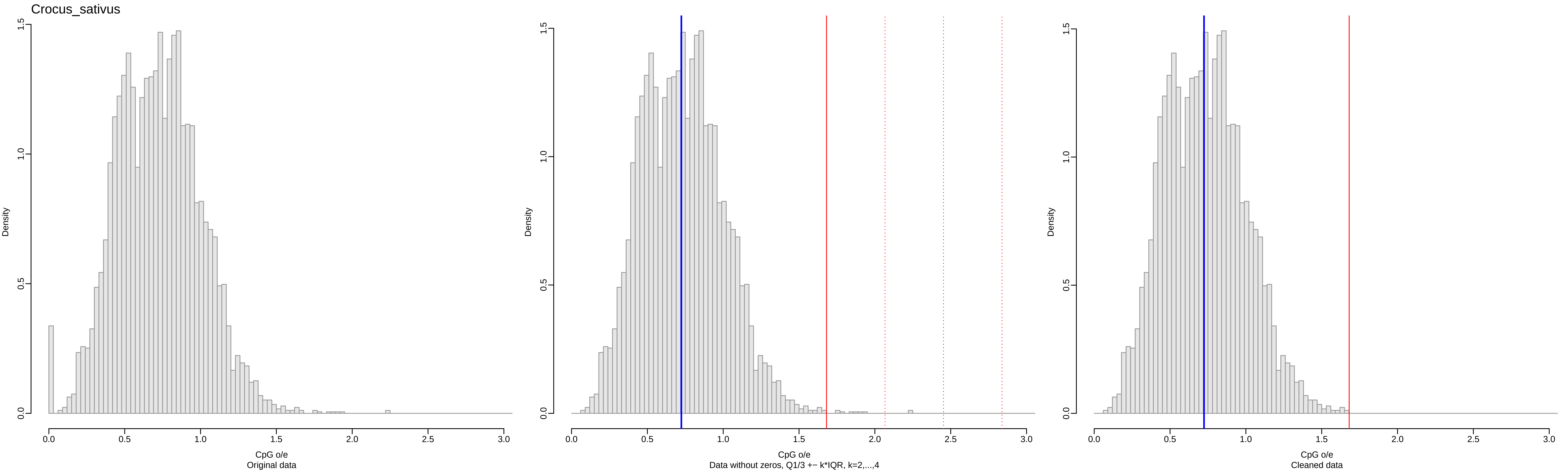

Crocus_sativus
Density
CpG o/e
Original data
Data without zeros, Q1/3 +− k*IQR, k=2,...,4
Cleaned data

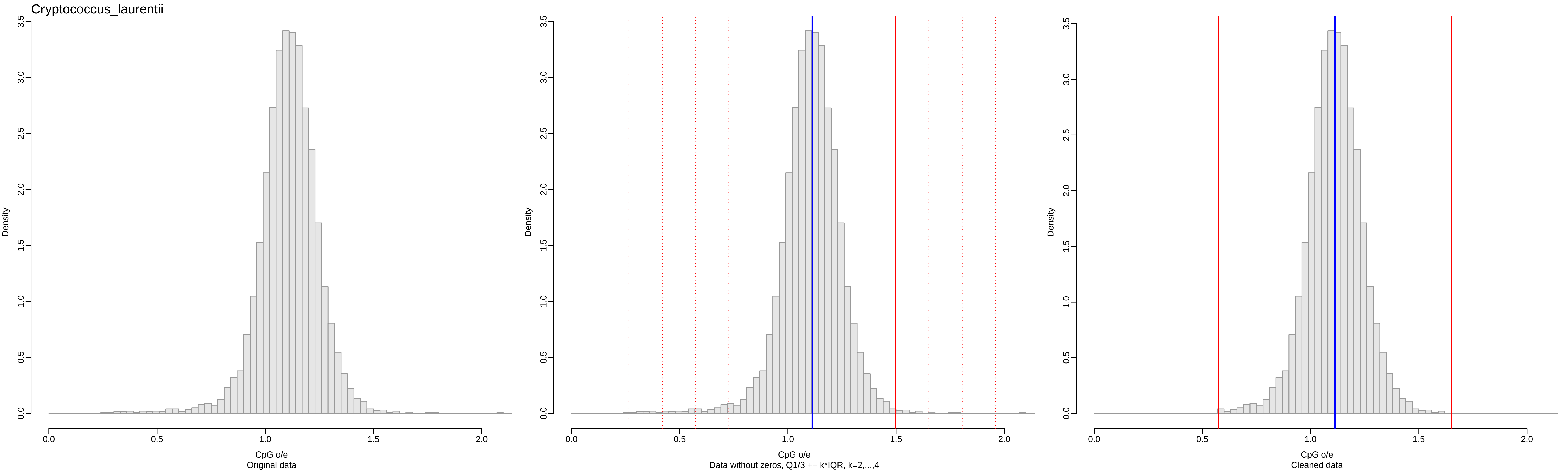
Cryptococcus_laurentii
Density
CpG o/e
Original data
Data without zeros, Q1/3 +− k*IQR, k=2,...,4
Cleaned data
0.0
0.5
1.0
1.5
2.0
2.5
3.0
3.5

Cryptopygus_antarcticus

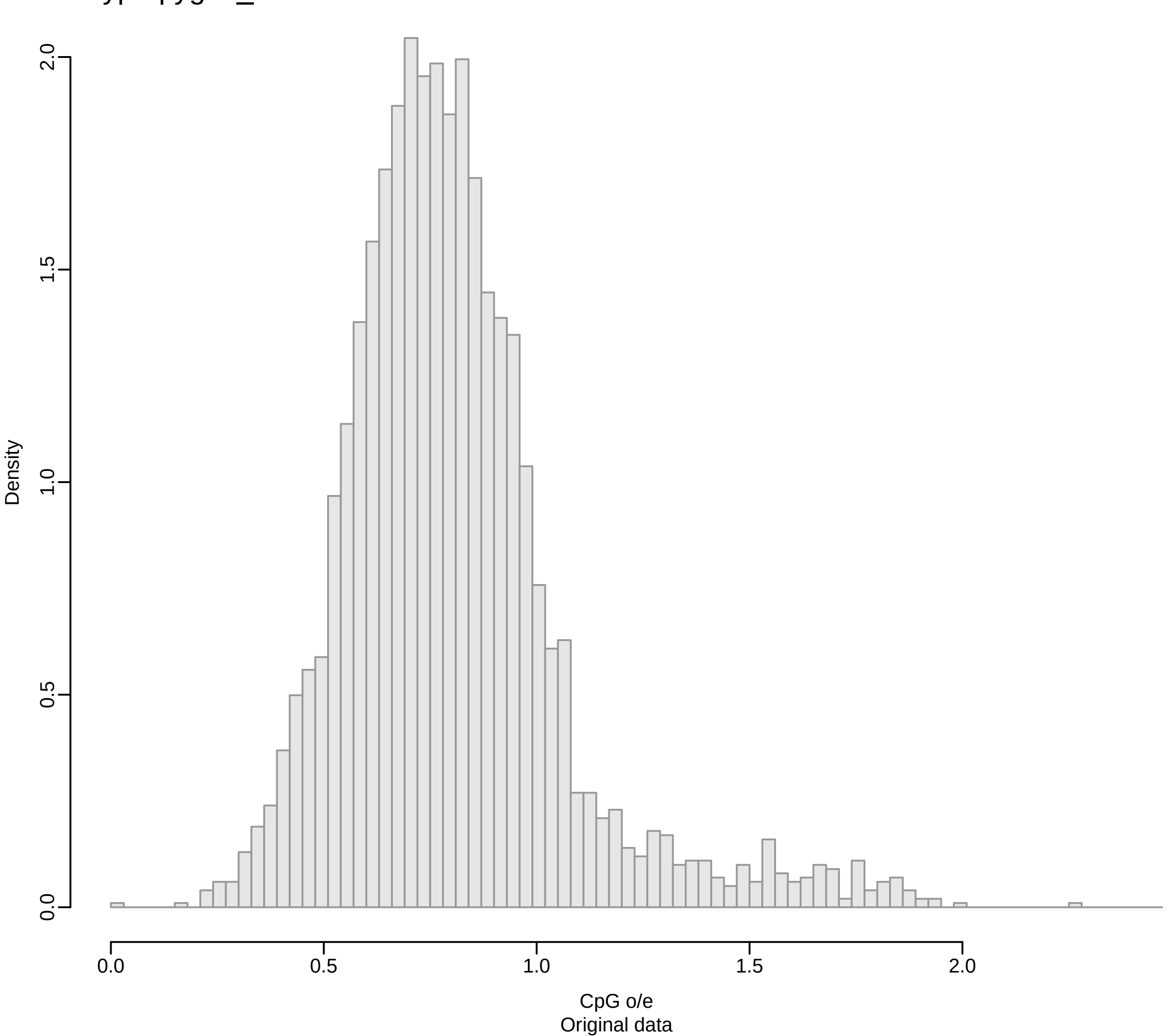

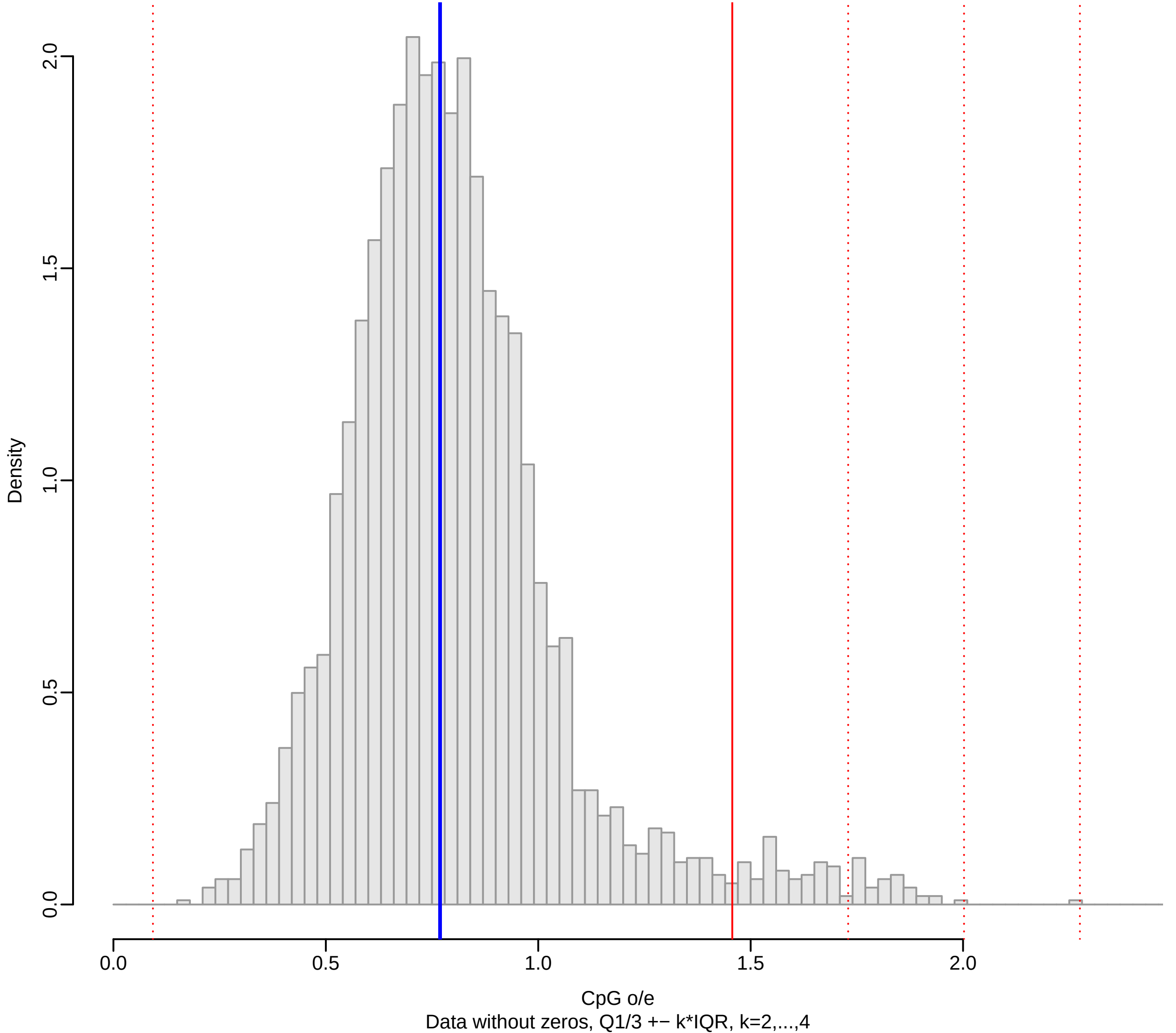

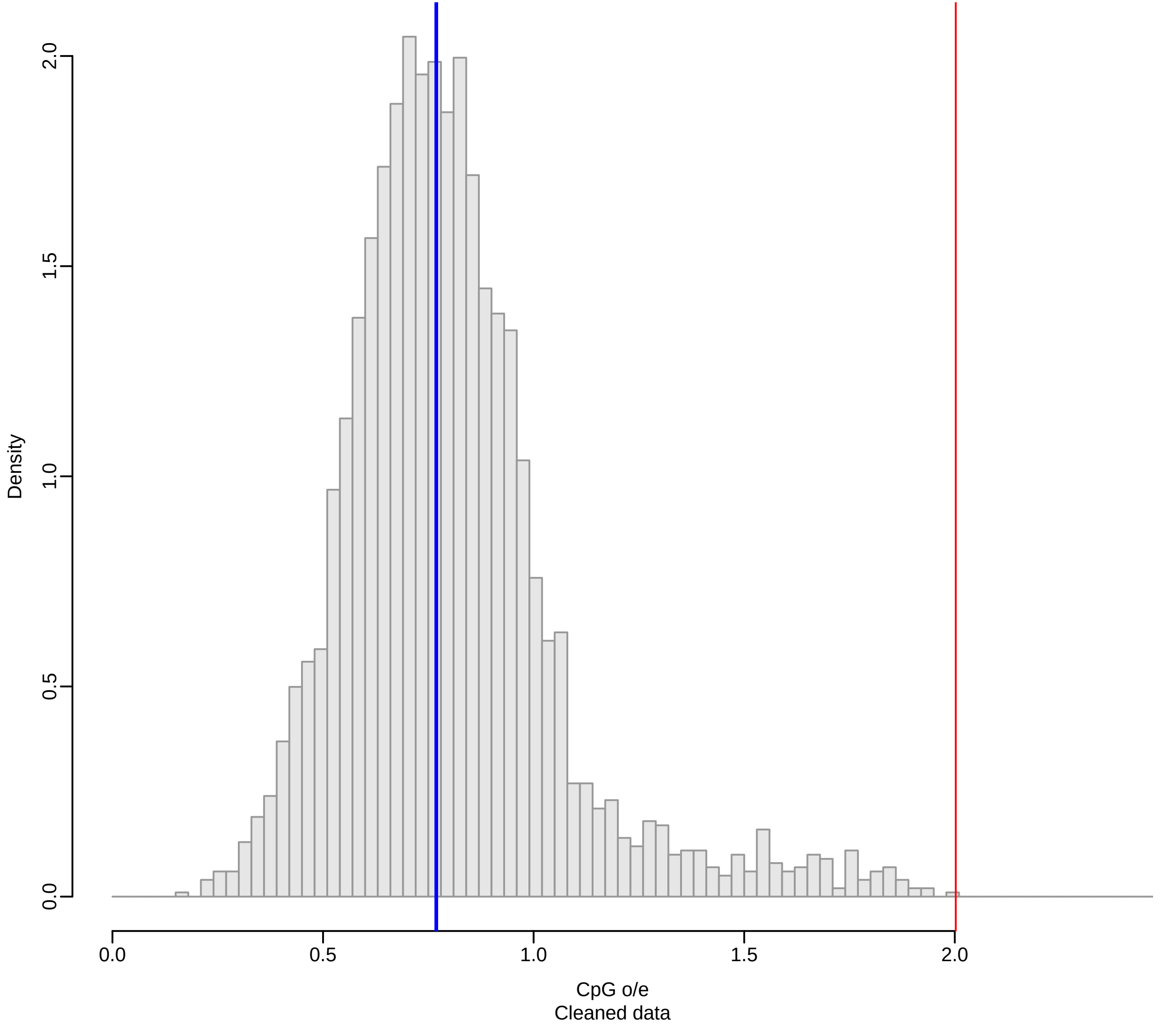

Cryptosporidium_parvum

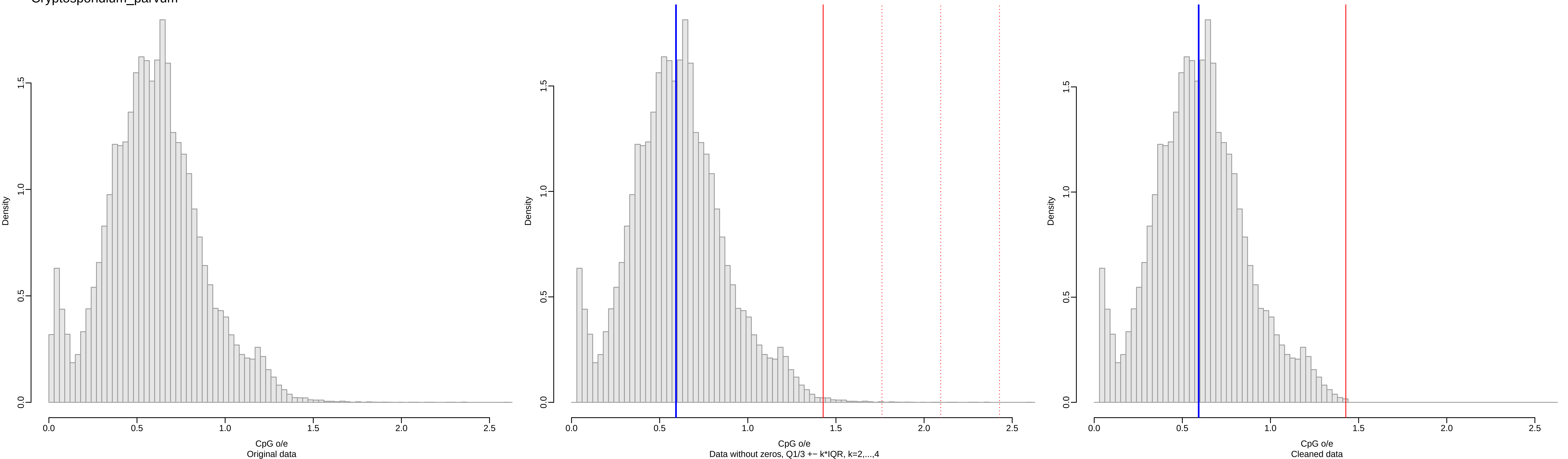

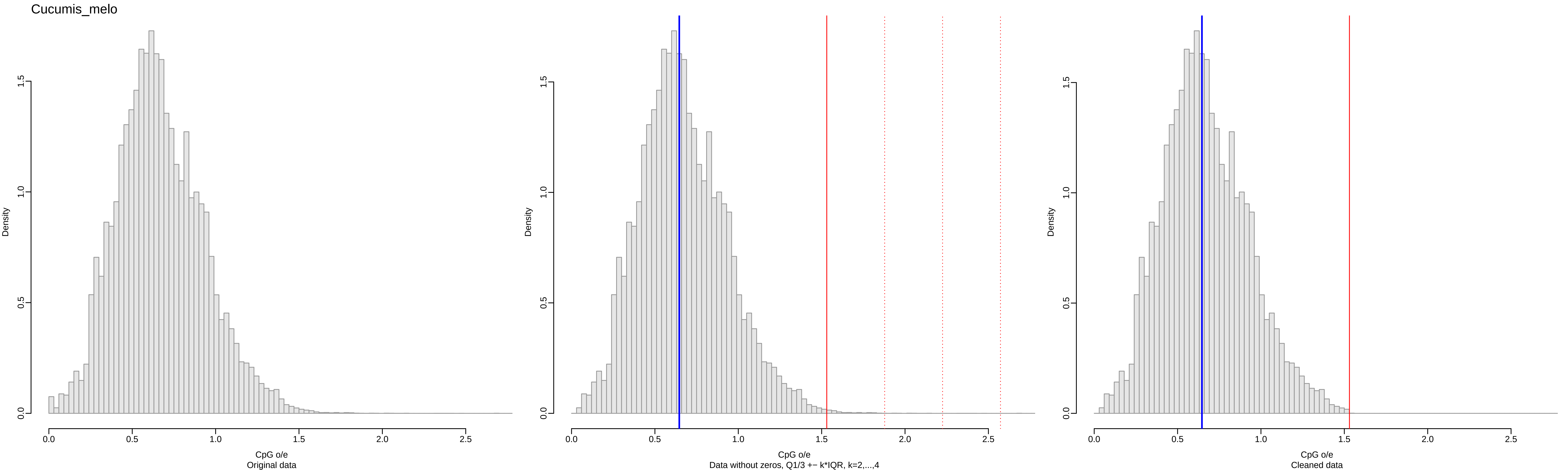
Cucumis_melo
Density
CpG o/e
Original data
Density
CpG o/e
Data without zeros, Q1/3 +− k*IQR, k=2,...,4
Density
CpG o/e
Cleaned data

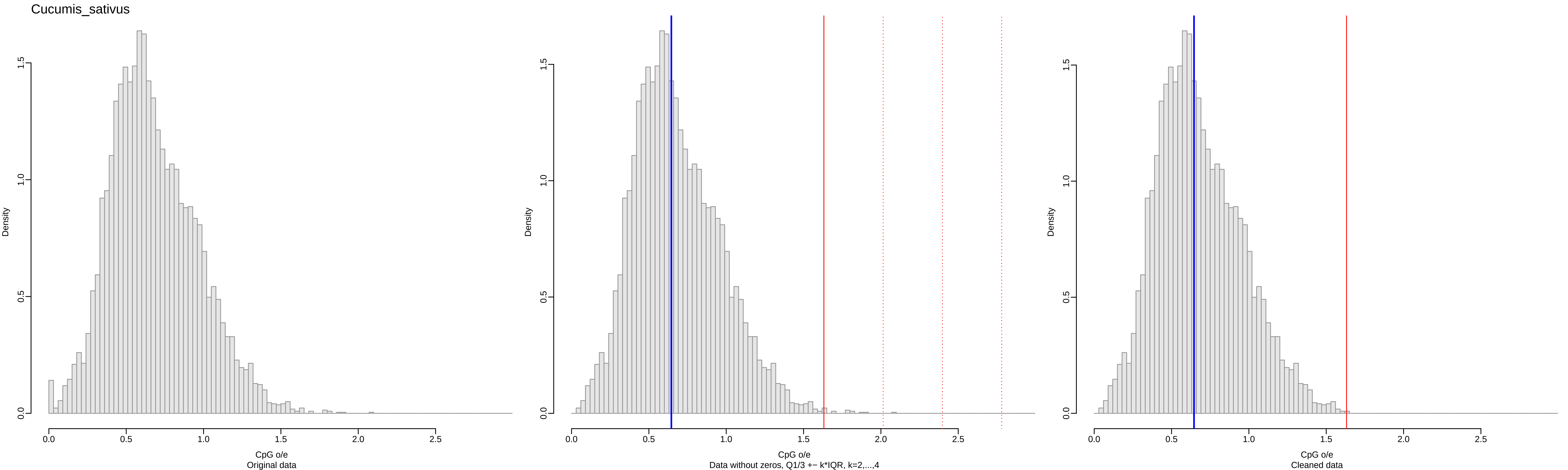

Cucumis_sativus
Density
0.0
0.5
1.0
1.5
2.0
2.5
CpG o/e
Original data
Data without zeros, Q1/3 +− k*IQR, k=2,...,4
Cleaned data

Culex_quinquefasciatus

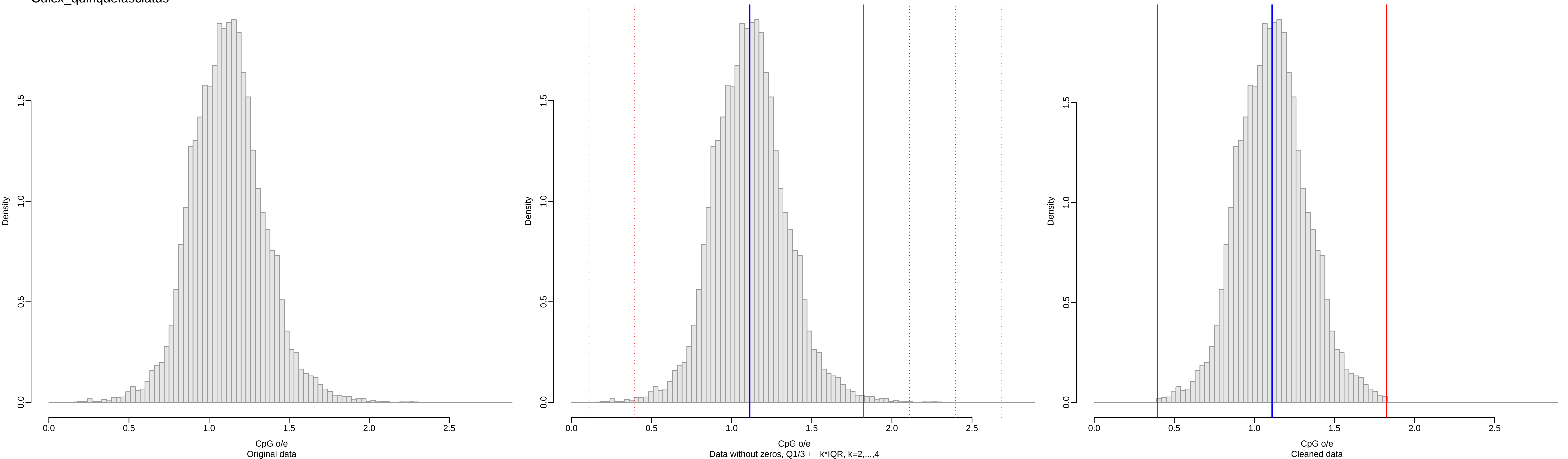

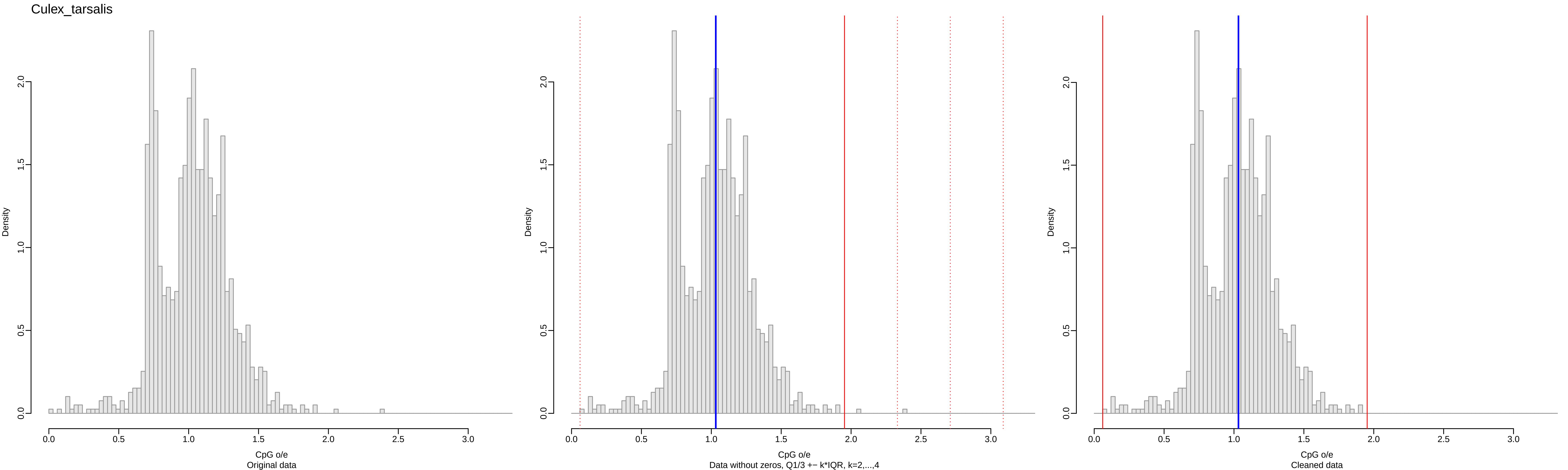
Culex_tarsalis
Density
CpG o/e
Original data
CpG o/e
Data without zeros, Q1/3 +− k*IQR, k=2,...,4
CpG o/e
Cleaned data

Curcuma_longa

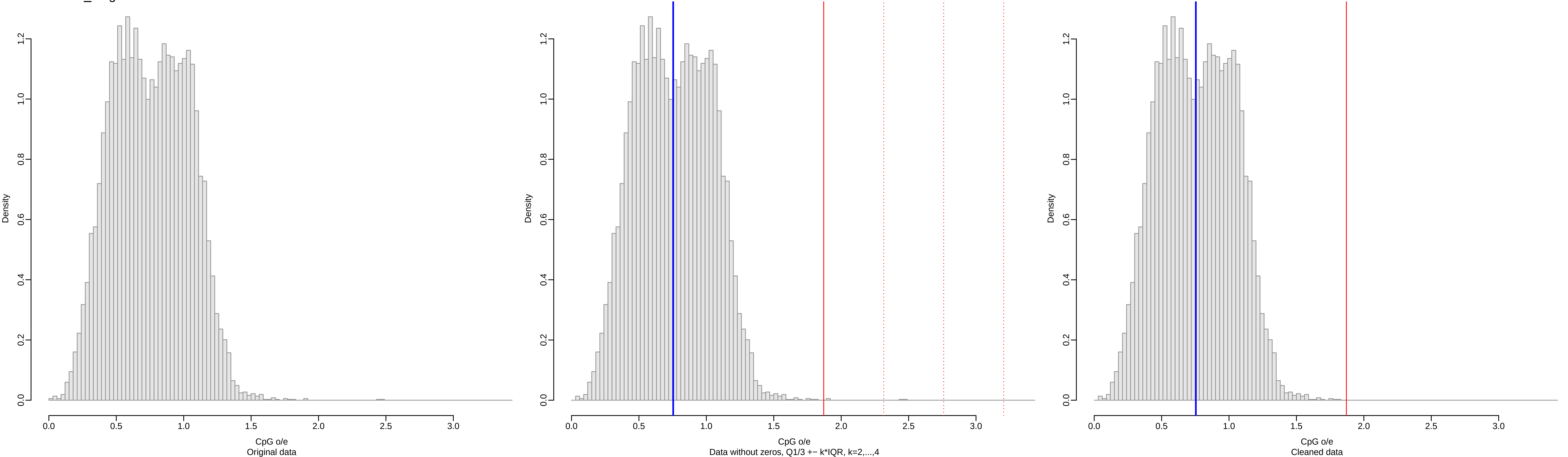

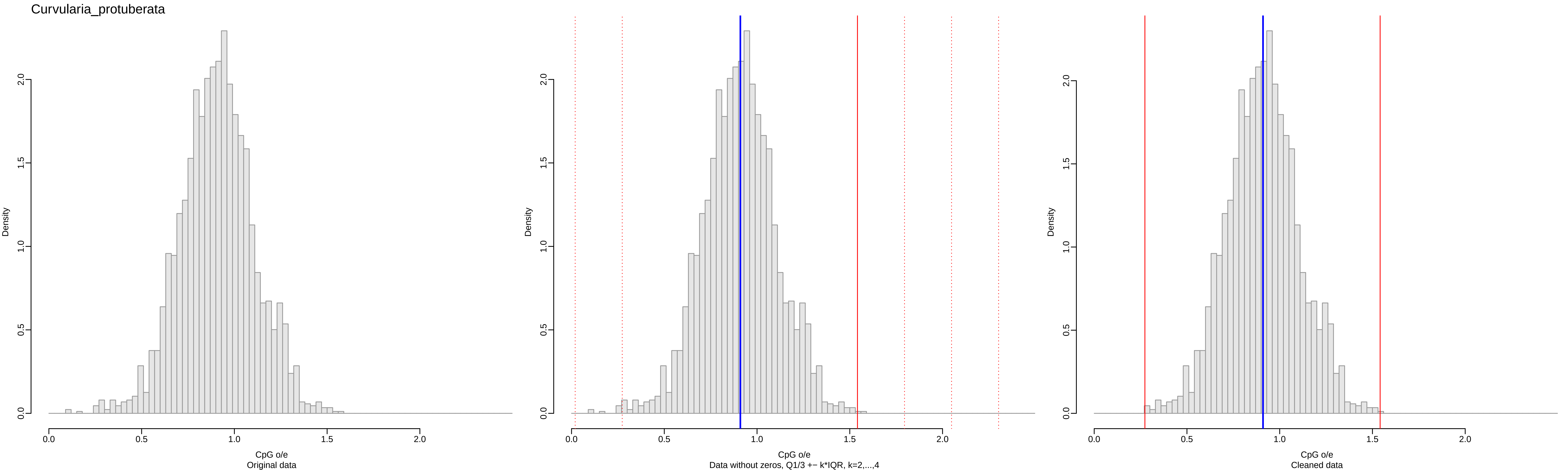

Curvularia_protuberata
Density
CpG o/e
Original data
CpG o/e
Data without zeros, Q1/3 +− k*IQR, k=2,...,4
CpG o/e
Cleaned data
0.0
0.5
1.0
1.5
2.0

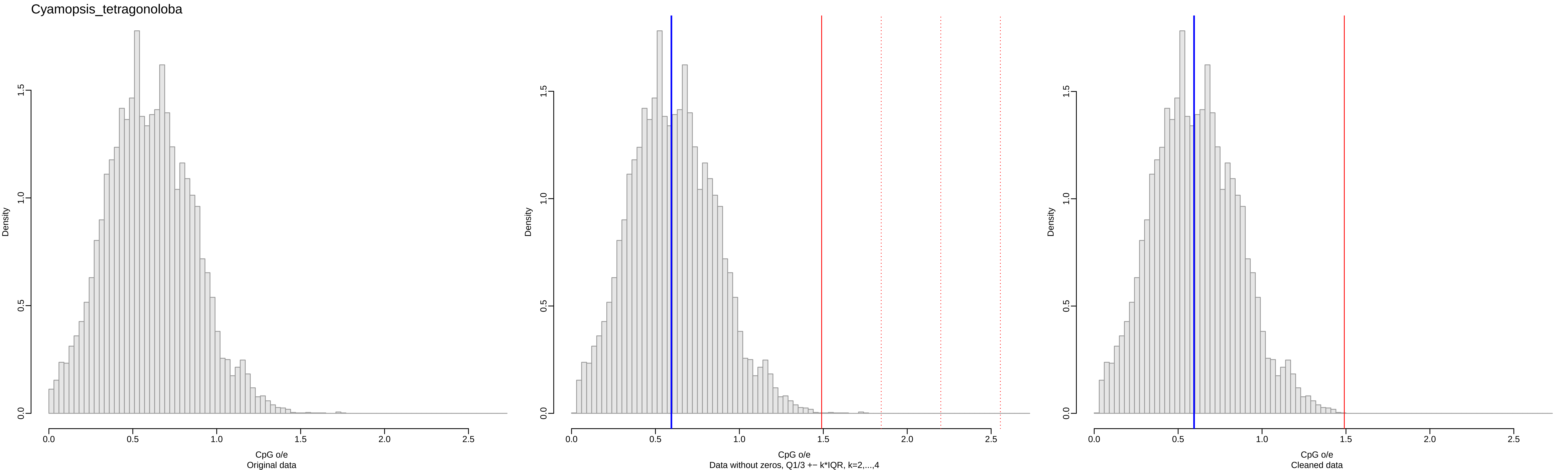
Cyamopsis_tetragonoloba
Density
CpG o/e
Original data
Data without zeros, Q1/3 +− k*IQR, k=2,...,4
Cleaned data
0.0
0.5
1.0
1.5
2.0
2.5

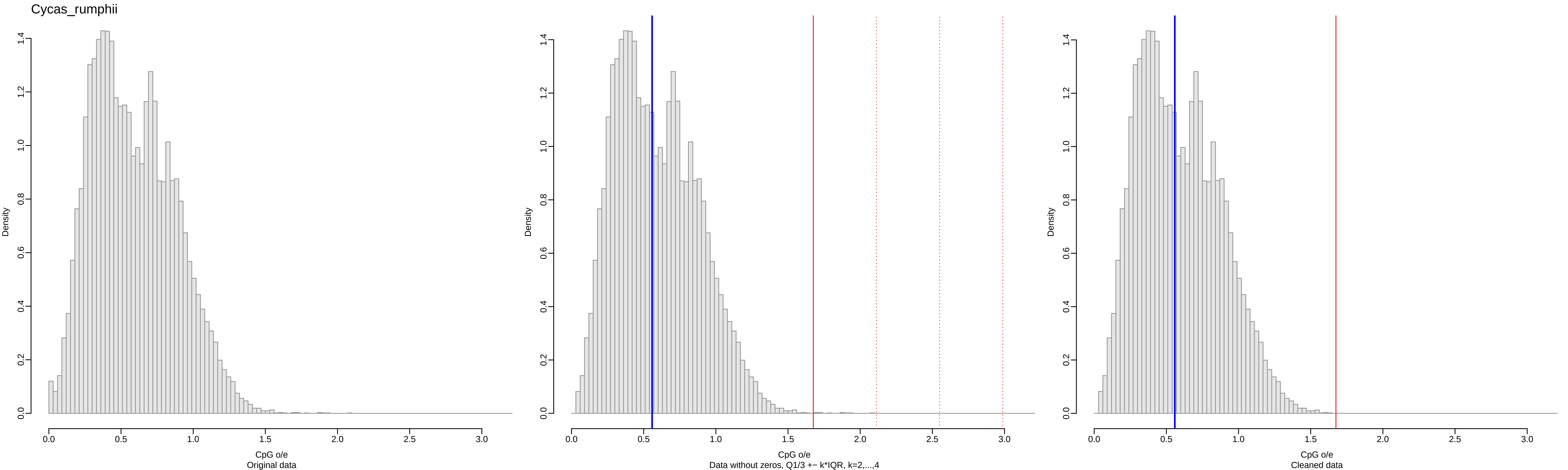

Cycas_rumphii
Density
CpG o/e
Original data
Data without zeros, Q1/3 +− k*IQR, k=2,...,4
Cleaned data
0.0
0.2
0.4
0.6
0.8
1.0
1.2
1.4
0.5
1.5
2.0
2.5
3.0

Cynodon_dactylon

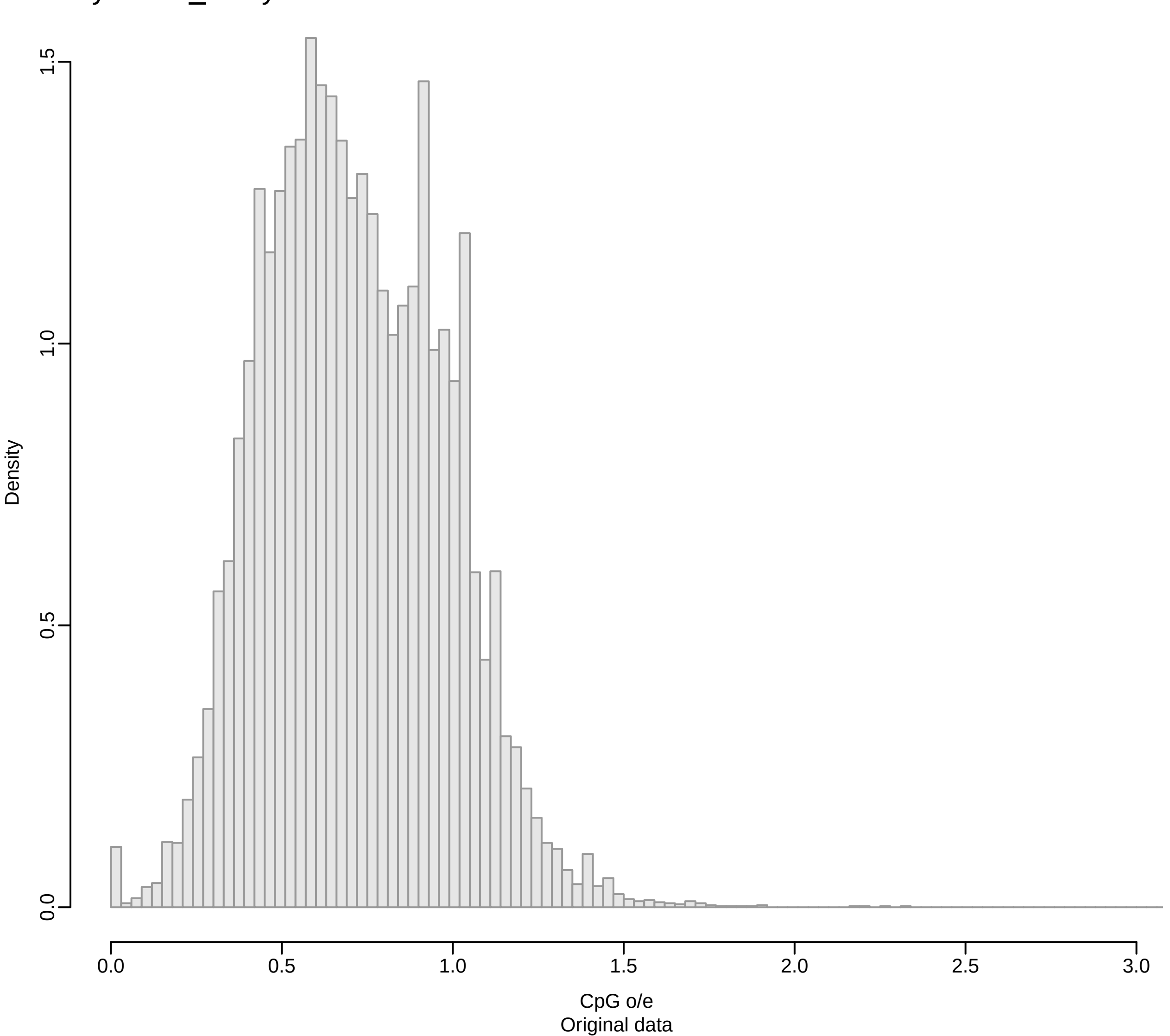

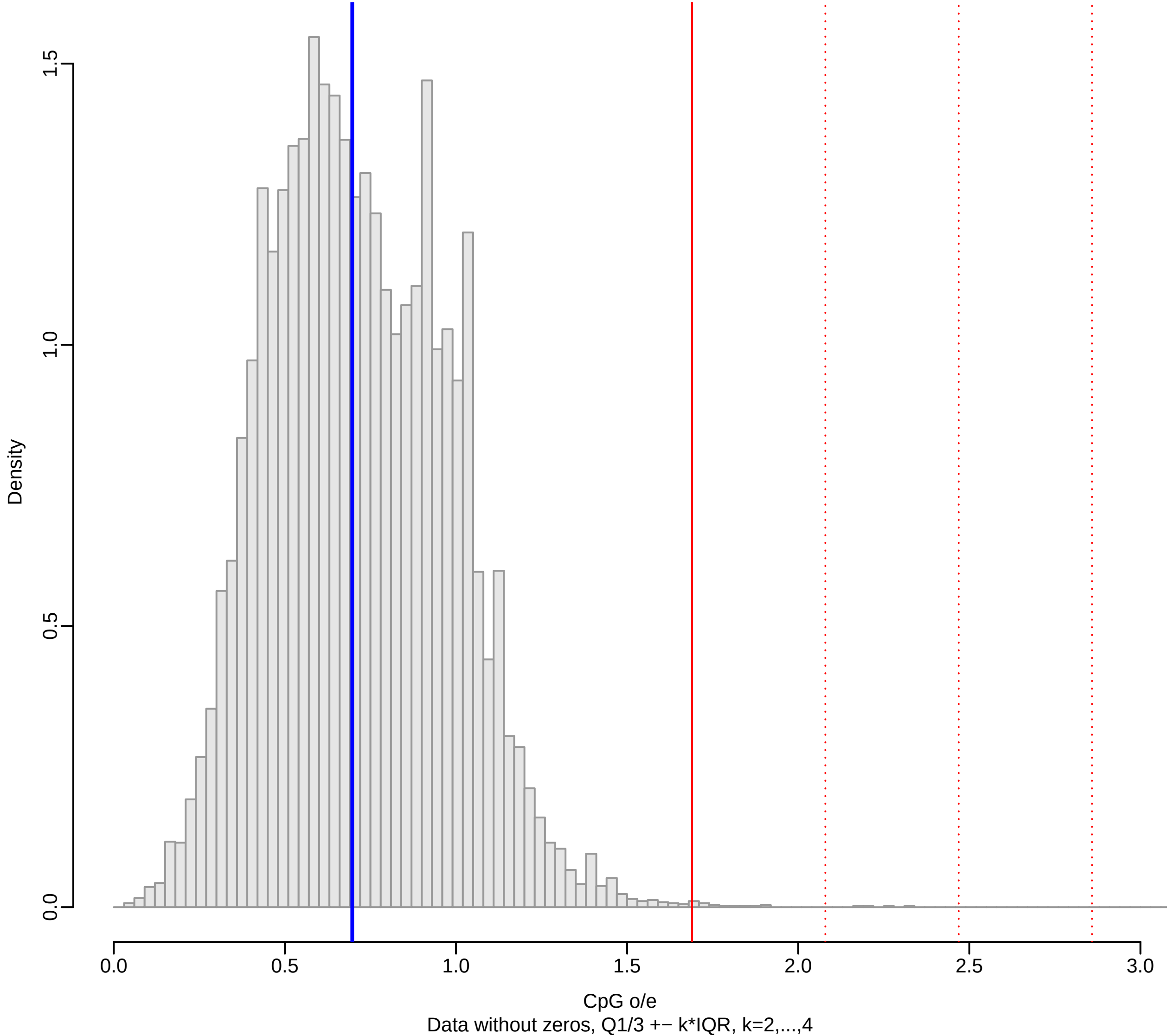

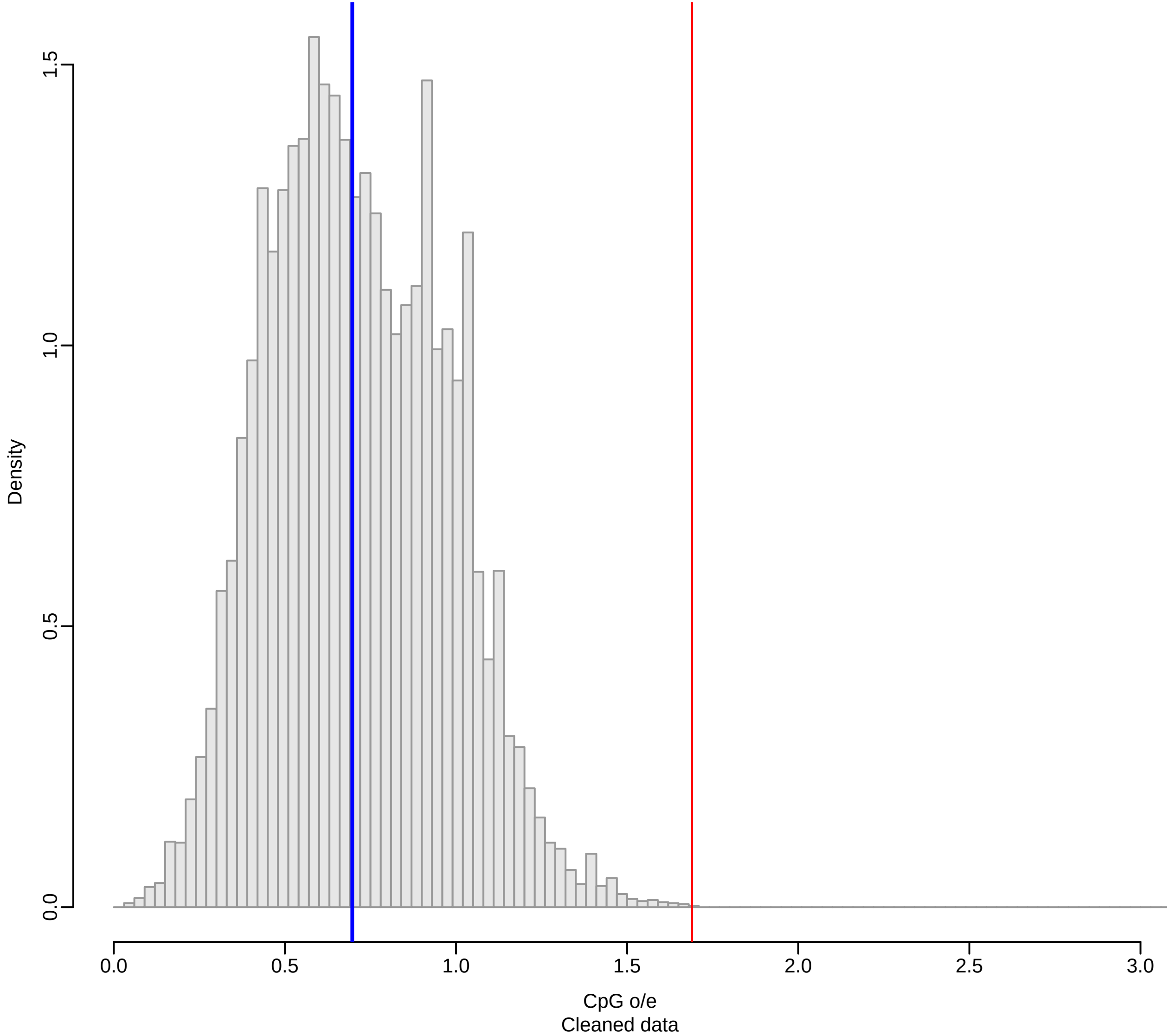

Cyprinodon_variegatus

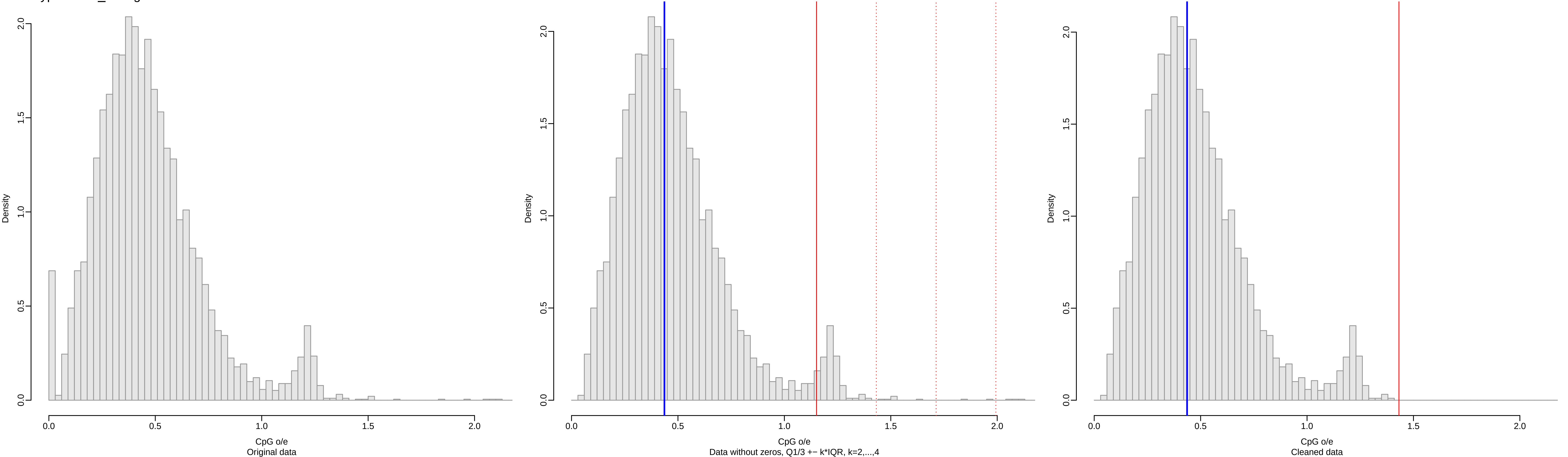

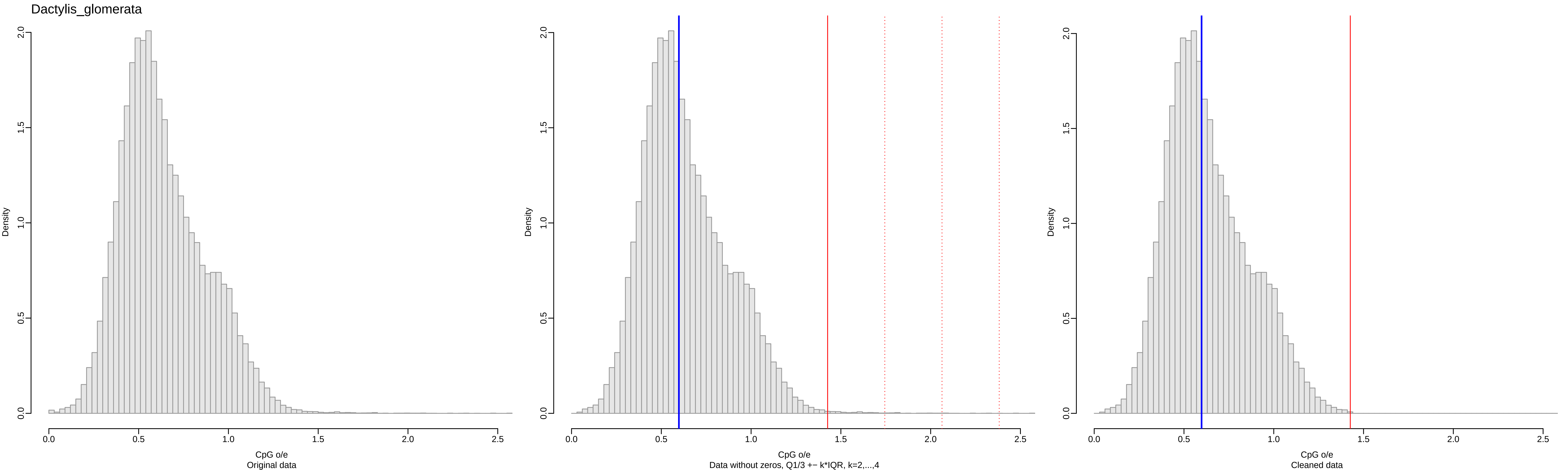

Dactylis_glomerata
Density
CpG o/e
Original data
Data without zeros, Q1/3 +− k*IQR, k=2,...,4
Cleaned data
0.0
0.5
1.0
1.5
2.0
2.5

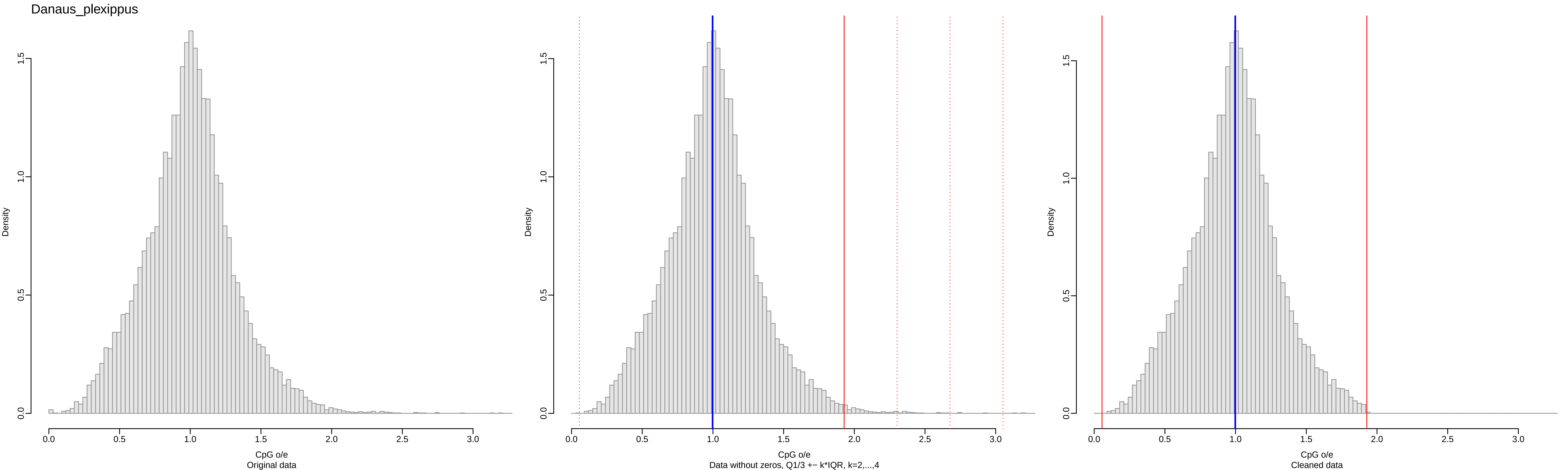
Danaus_plexippus
Density
0.0
0.5
1.0
1.5
0.0
0.5
1.0
1.5
2.0
2.5
3.0
CpG o/e
Original data
Data without zeros, Q1/3 +− k*IQR, k=2,...,4
Cleaned data

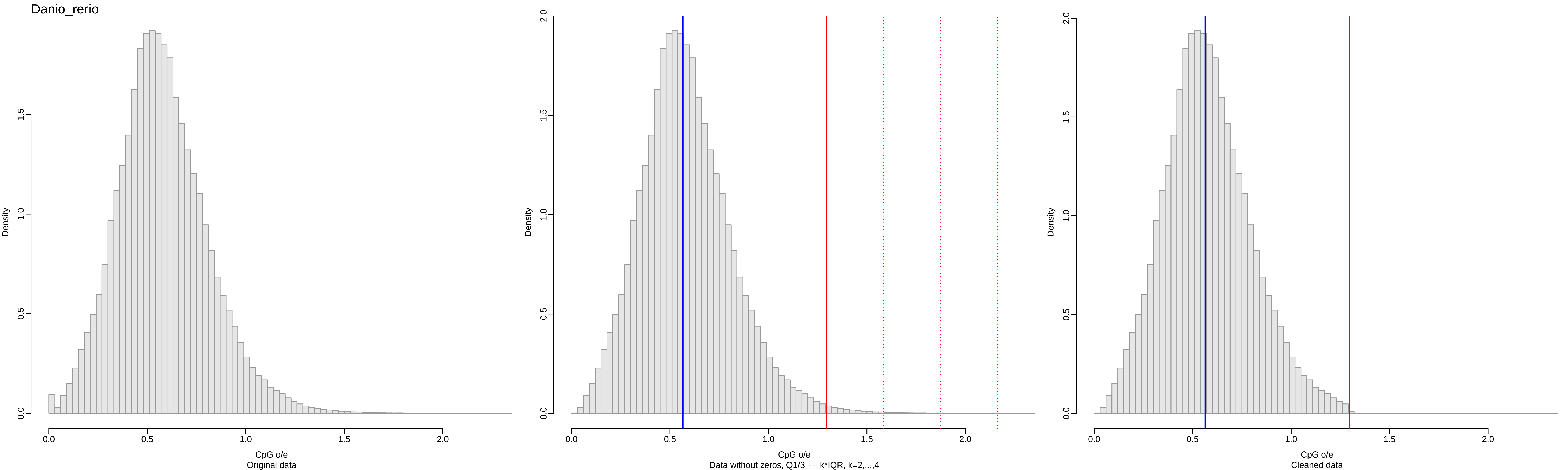
Danio_rerio
Density
CpG o/e
Original data
CpG o/e
Data without zeros, Q1/3 +− k*IQR, k=2,...,4
CpG o/e
Cleaned data

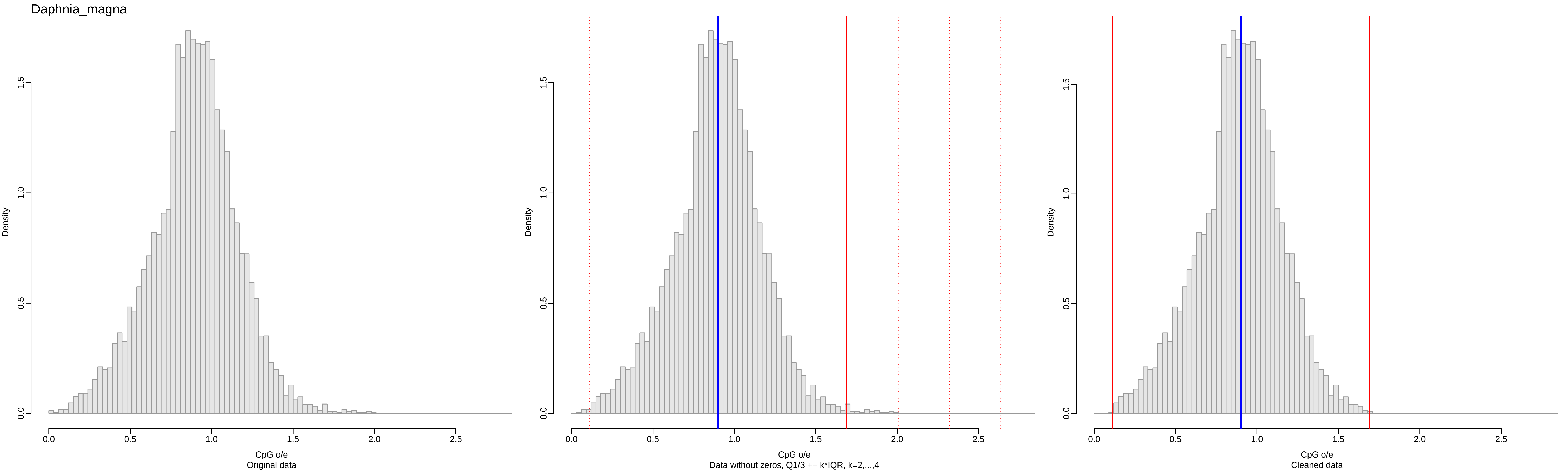
Daphnia_magna
Density
CpG o/e
Original data
Data without zeros, Q1/3 +− k*IQR, k=2,...,4
Cleaned data
0.0
0.5
1.0
1.5
2.0
2.5

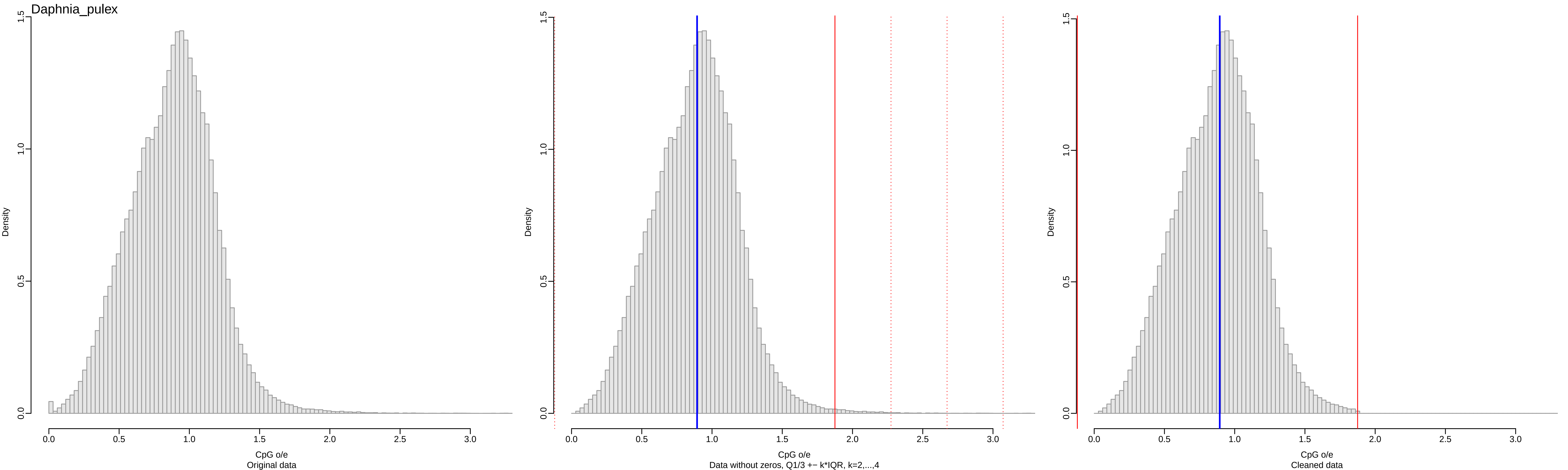

Daphnia_pulex
Density
CpG o/e
Original data
Density
CpG o/e
Data without zeros, Q1/3 +− k*IQR, k=2,...,4
Density
CpG o/e
Cleaned data

Deinagkistrodon_acutus

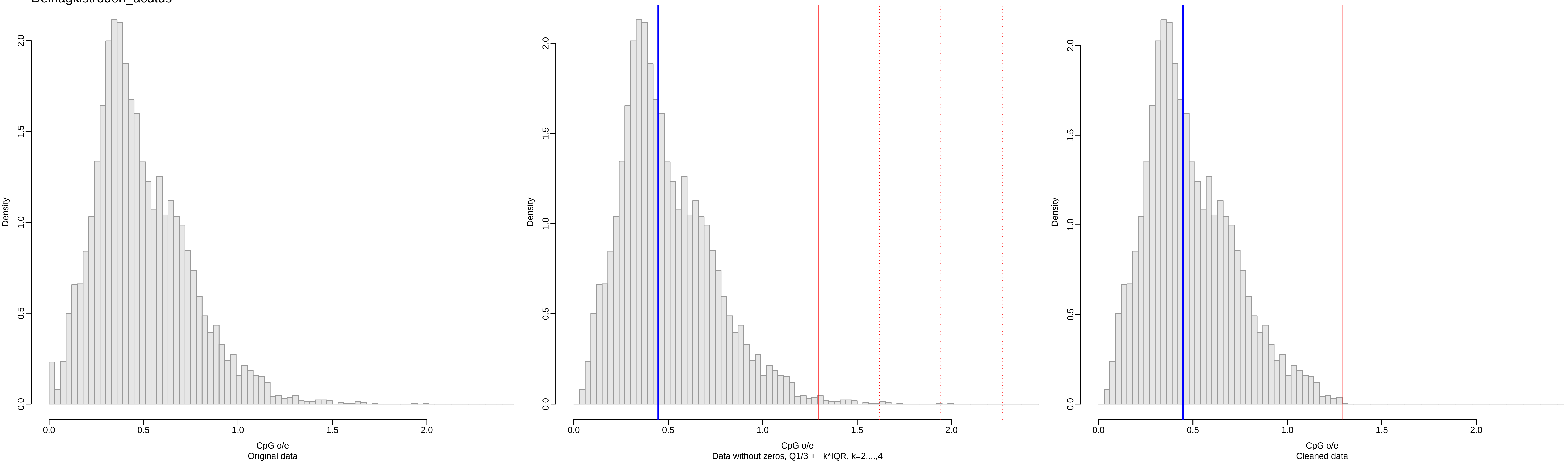

Dendroctonus_ponderosae

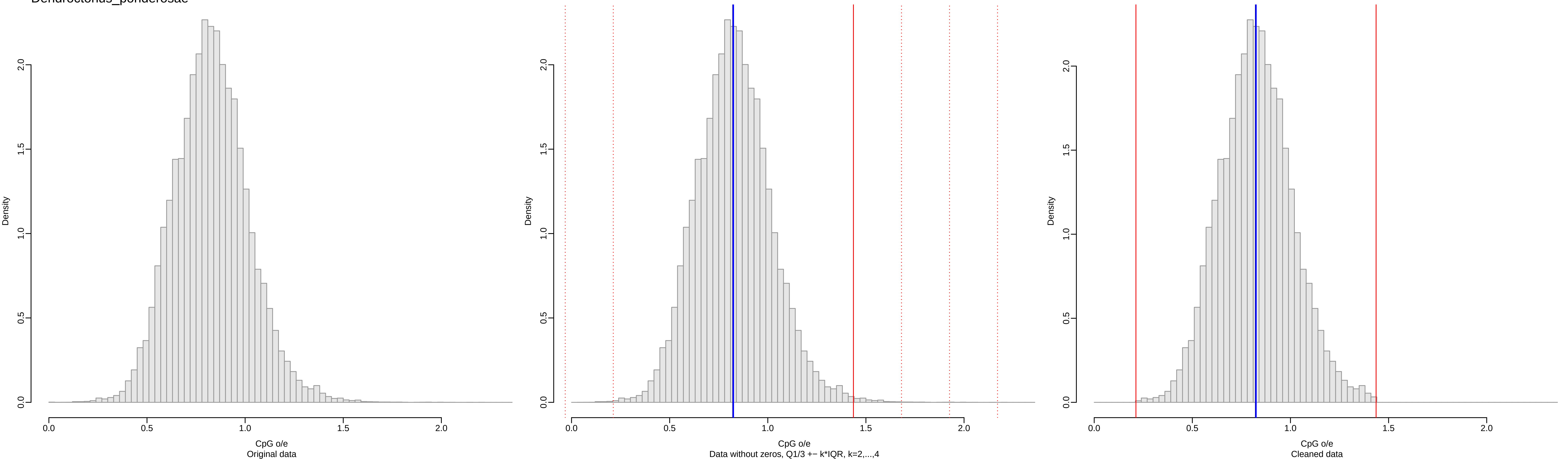

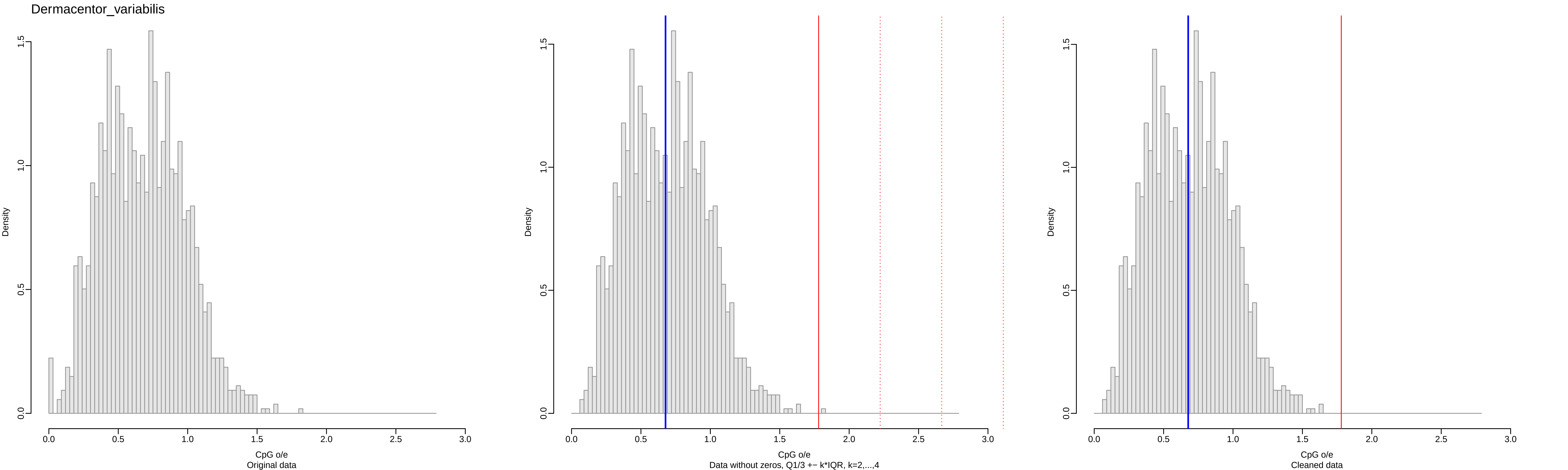
Dermacentor_variabilis
Density
CpG o/e
Original data
Data without zeros, Q1/3 +− k*IQR, k=2,...,4
Cleaned data
0.0
0.5
1.0
1.5
2.0
2.5
3.0

Diaphorina_citri

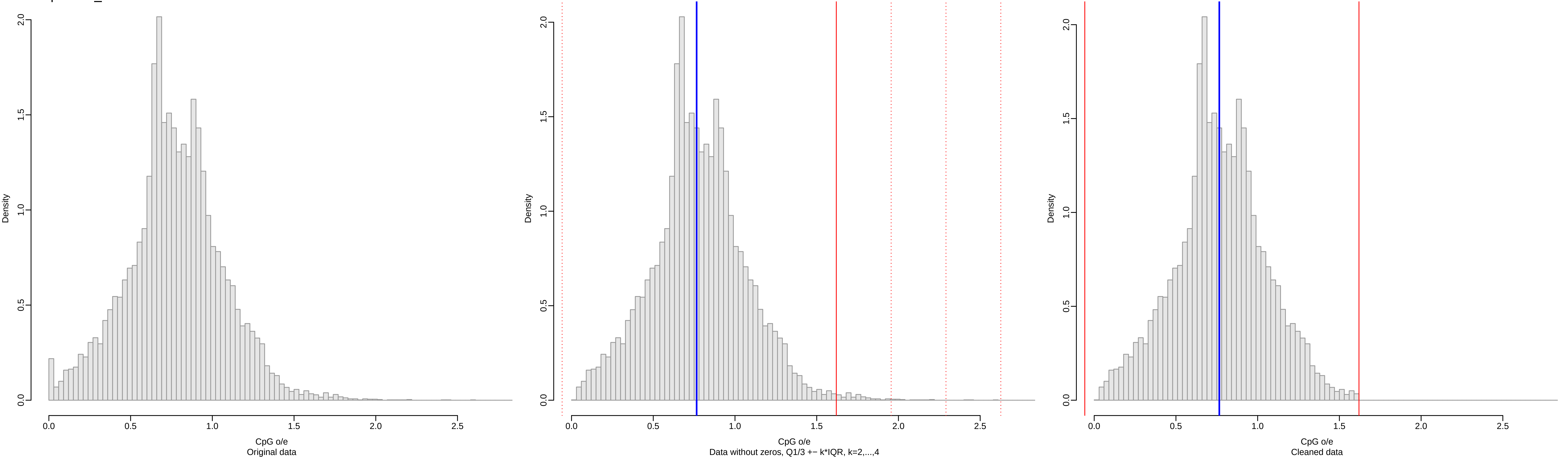

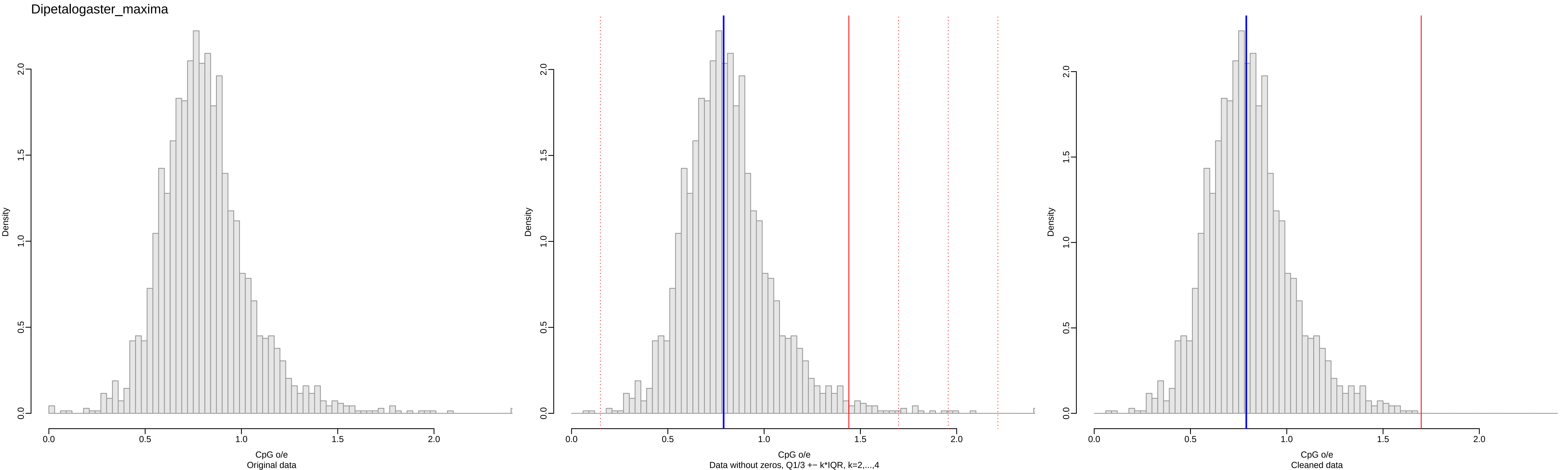

Dipetalogaster_maxima
Density
CpG o/e
Original data
Data without zeros, Q1/3 +− k*IQR, k=2,...,4
Cleaned data
0.0
0.5
1.0
1.5
2.0

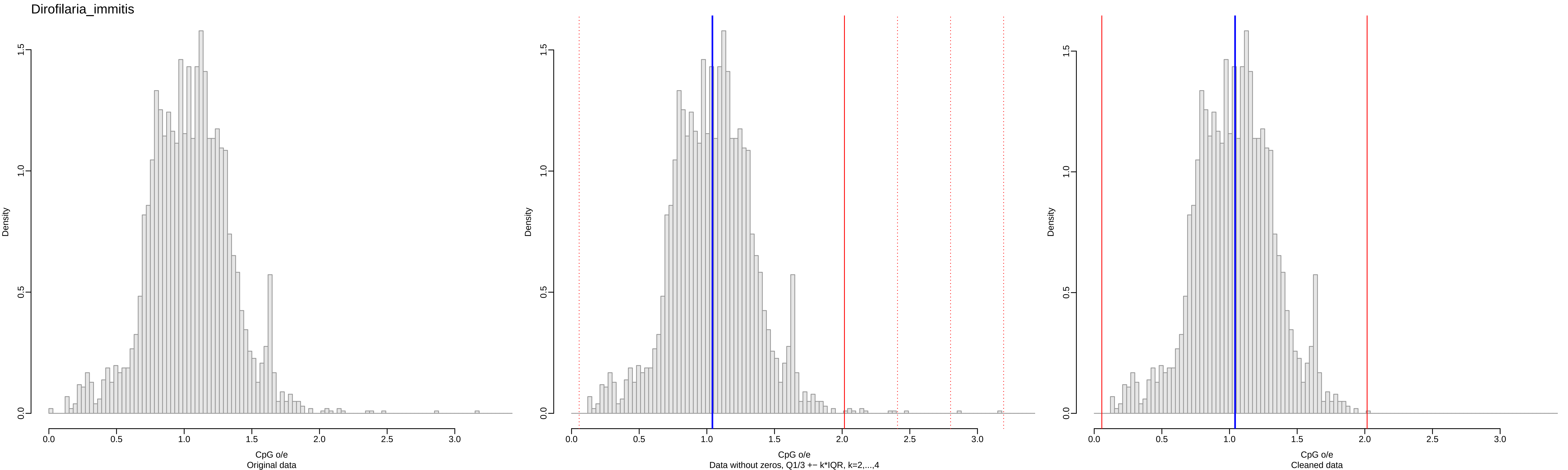
Dirofilaria_immitis
Density
CpG o/e
Original data
CpG o/e
Data without zeros, Q1/3 +− k*IQR, k=2,...,4
CpG o/e
Cleaned data
0.0
0.5
1.0
1.5
2.0
2.5
3.0

Dissostichus_mawsoni

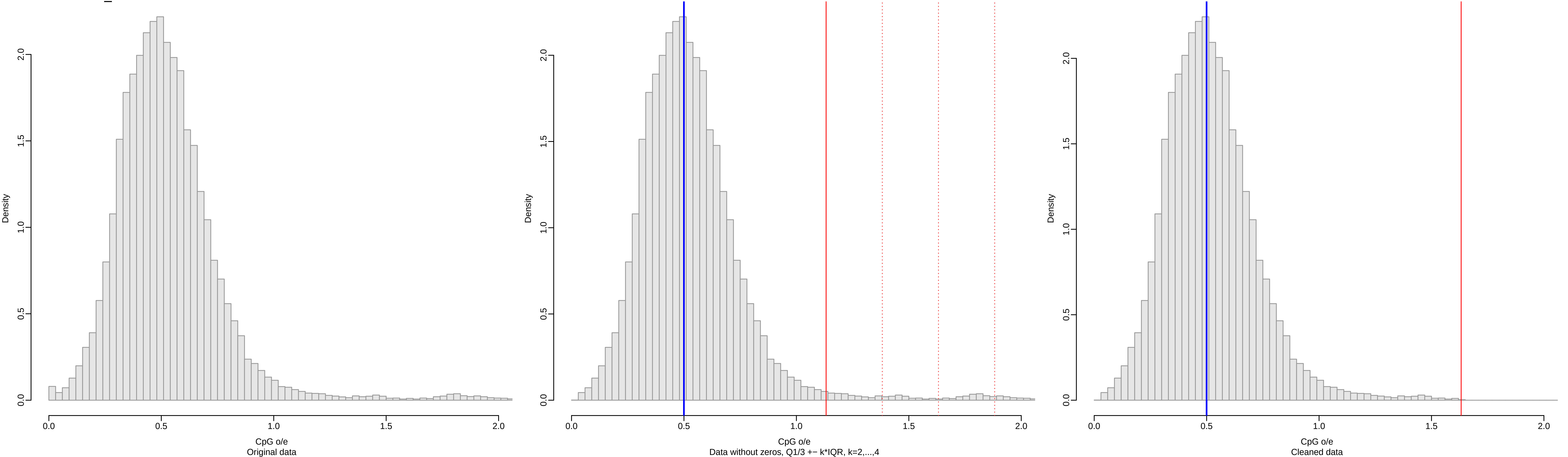

Ditylenchus_africanus

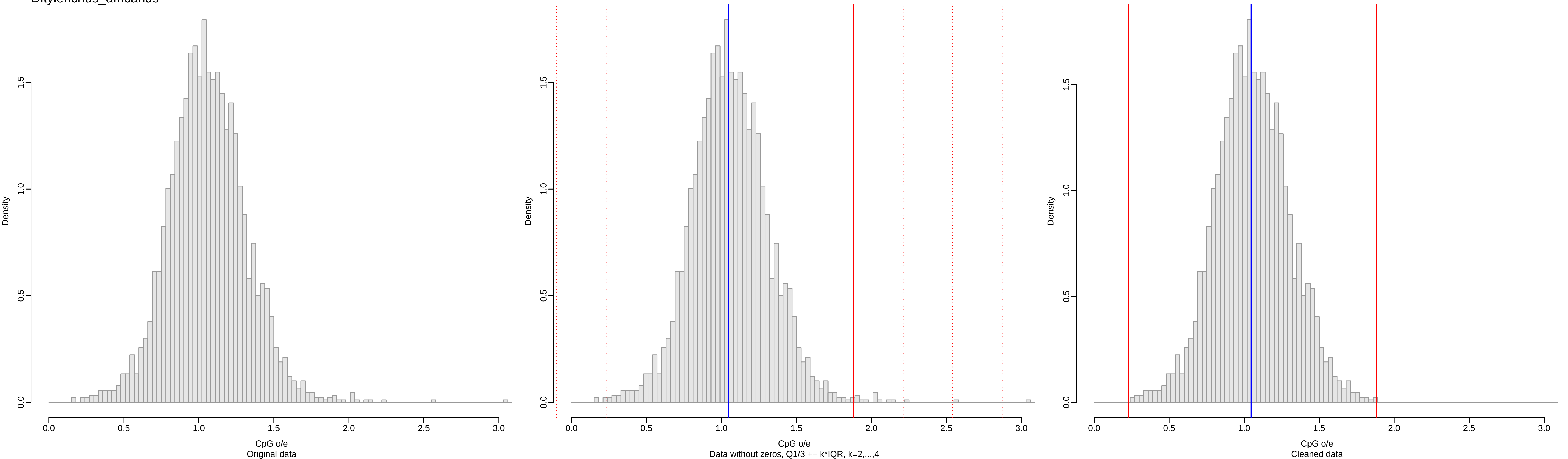

Drosophila_ananassae

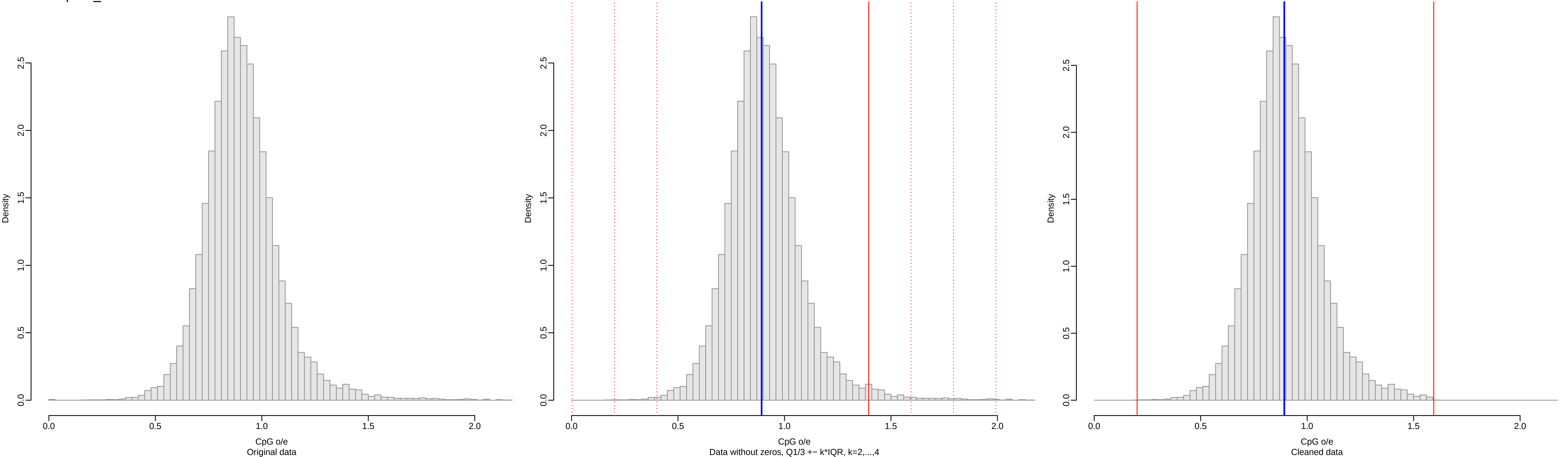

Drosophila_auraria

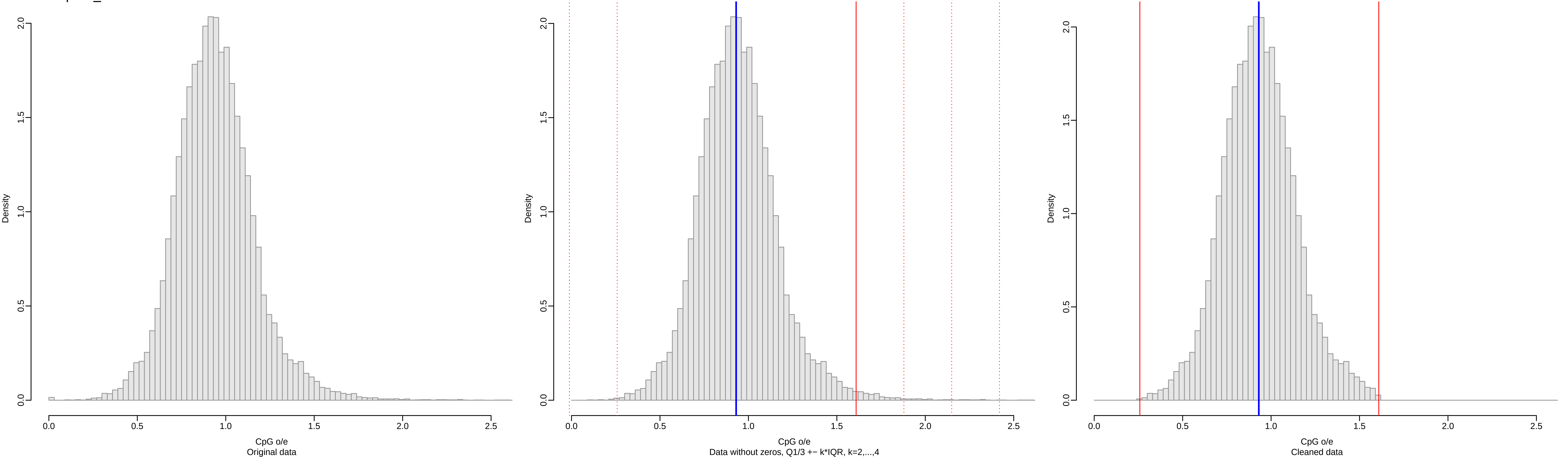

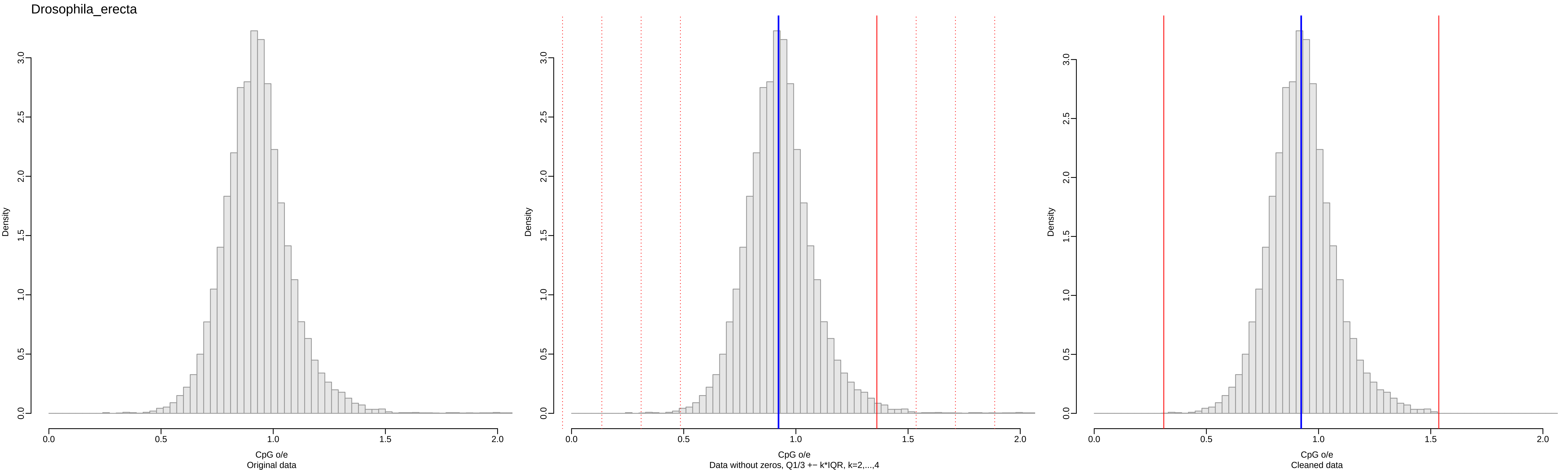

Drosophila_erecta
Density
CpG o/e
Original data
Density
CpG o/e
Data without zeros, Q1/3 +− k*IQR, k=2,...,4
Density
CpG o/e
Cleaned data

Drosophila_grimshawi

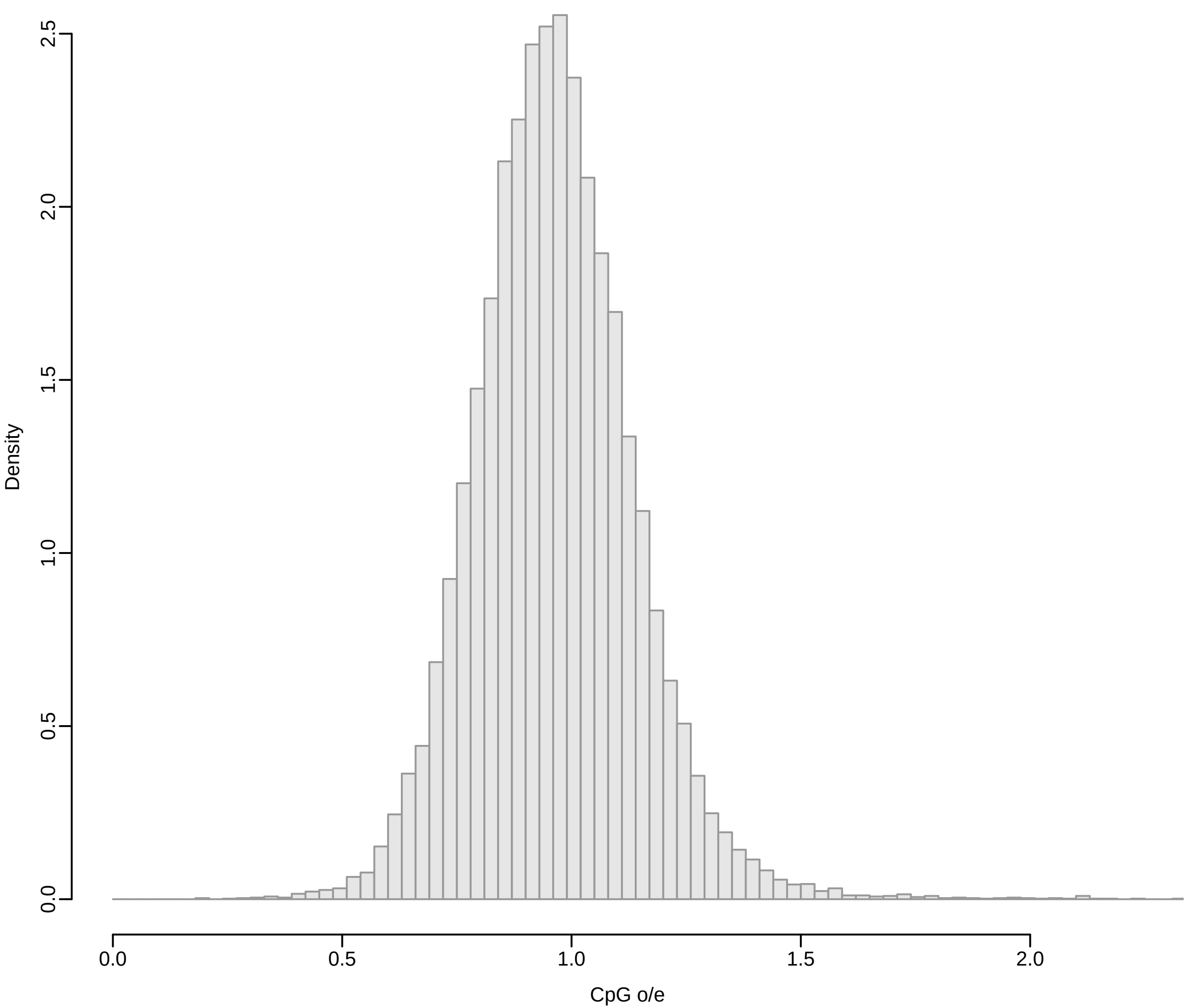

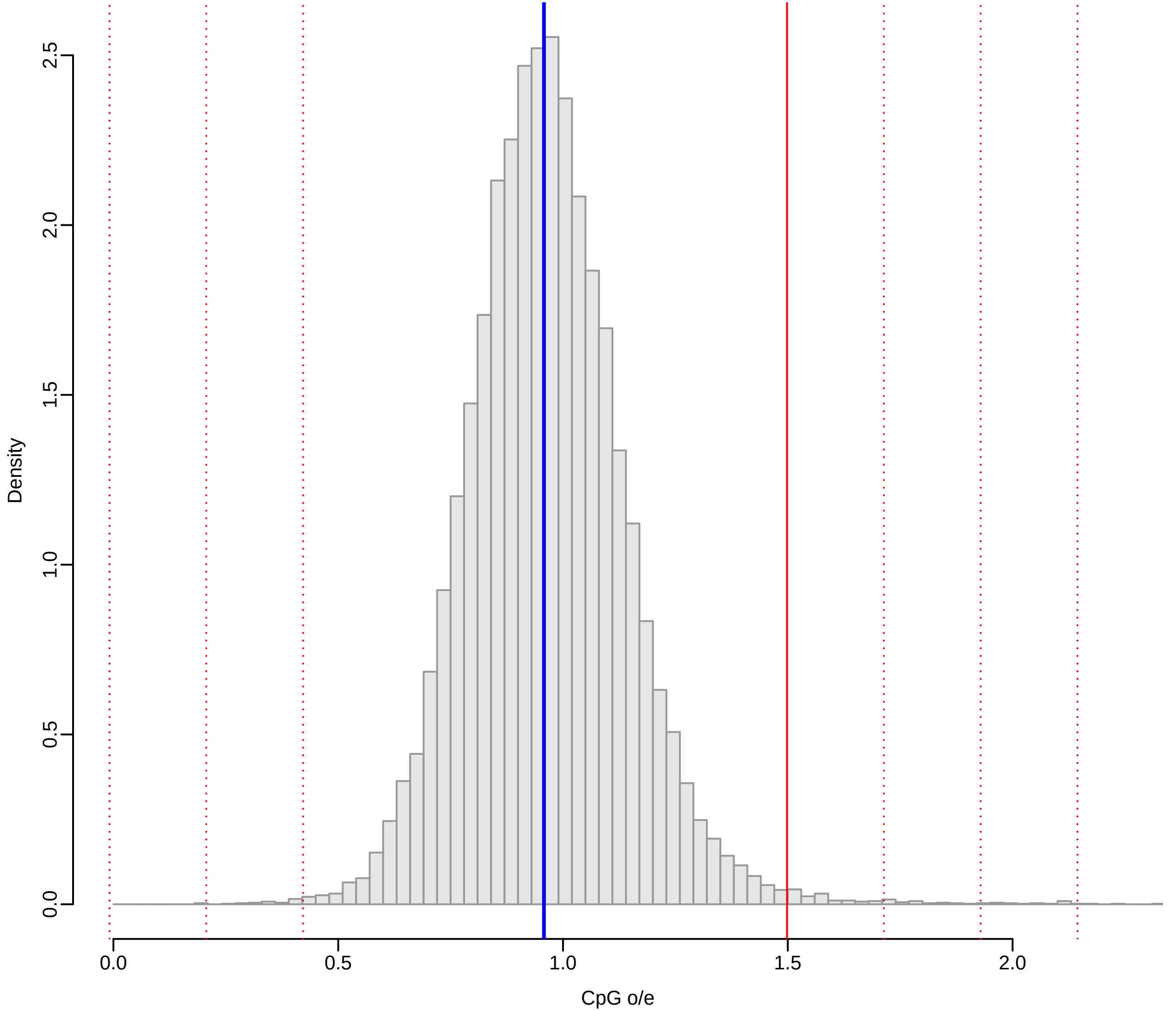

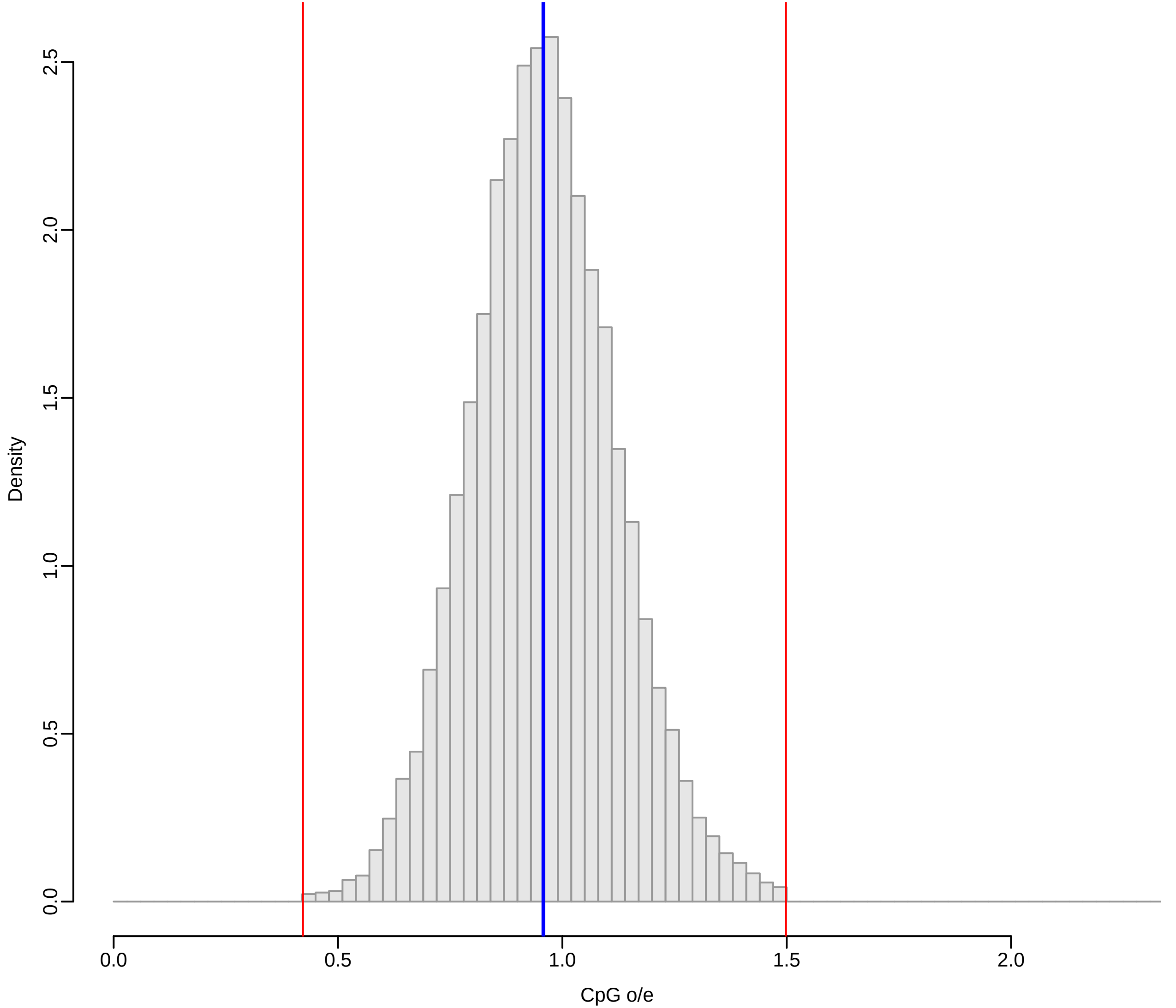

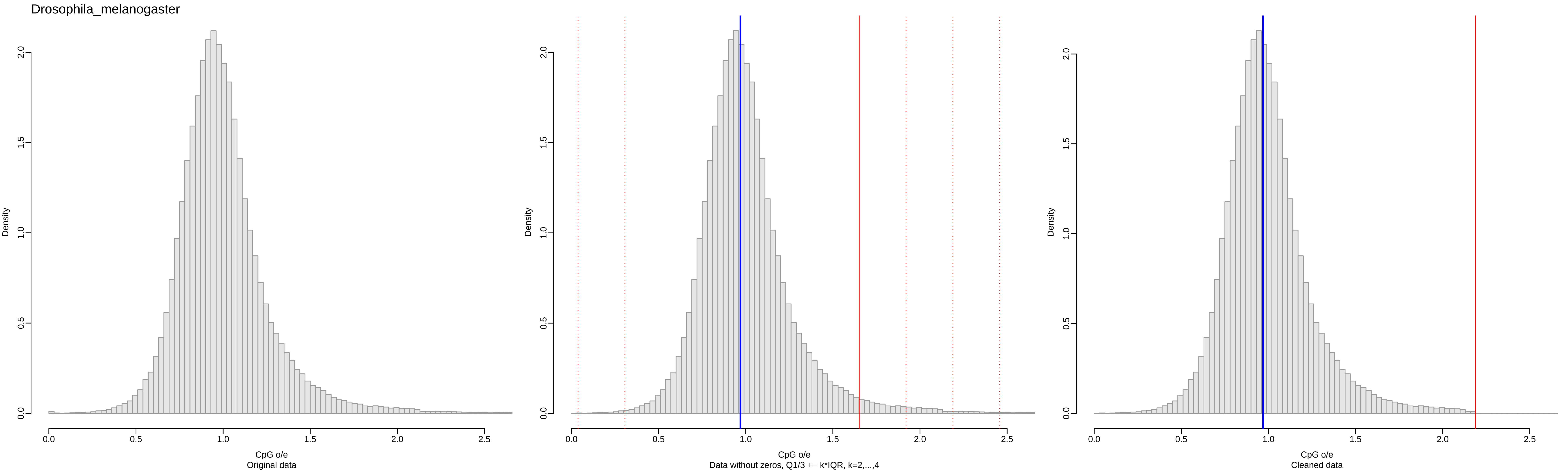
Drosophila_melanogaster
Density
CpG o/e
Original data
Data without zeros, Q1/3 +− k*IQR, k=2,...,4
Cleaned data
0.0
0.5
1.0
1.5
2.0
2.5

Drosophila_mojavensis

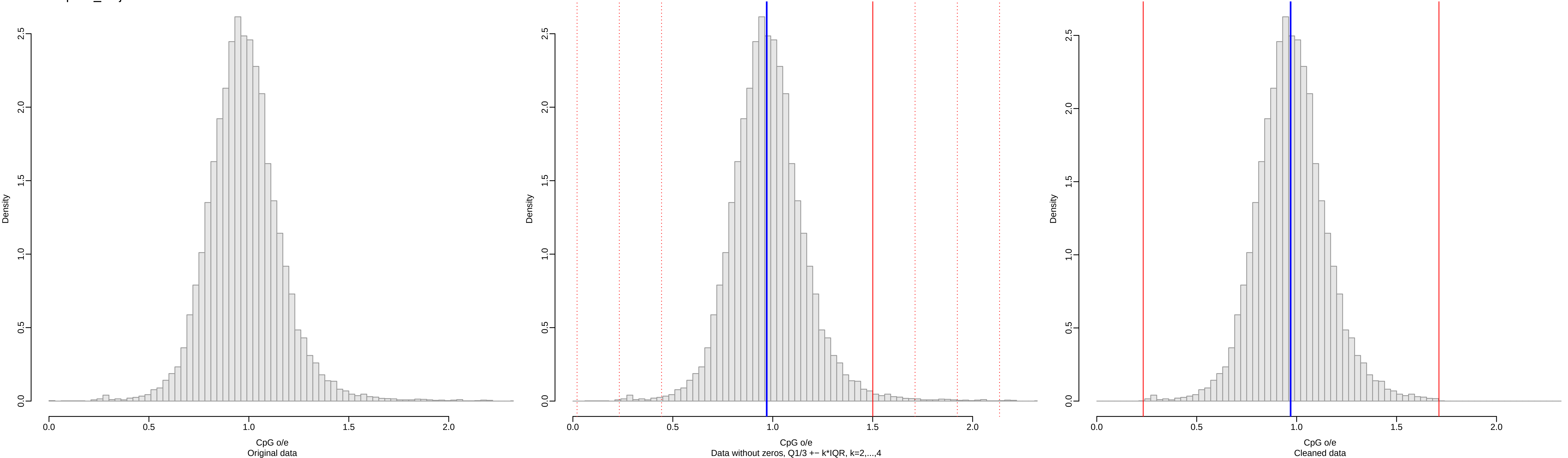

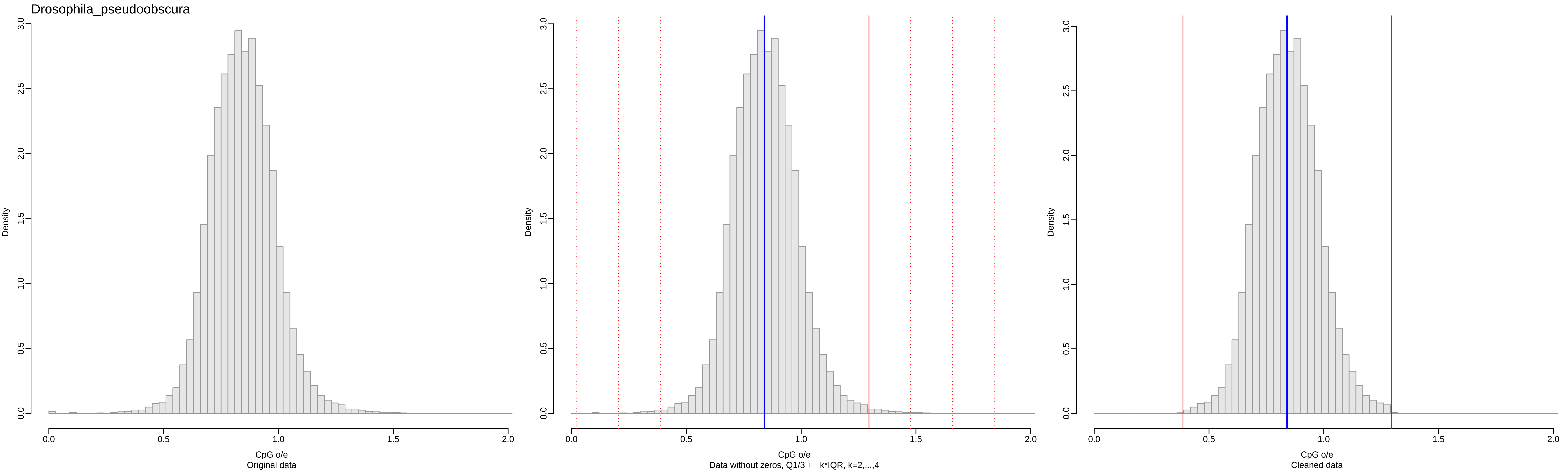
Drosophila_pseudoobscura
Density
CpG o/e
Original data
Data without zeros, Q1/3 +− k*IQR, k=2,...,4
Cleaned data
0.0
0.5
1.0
1.5
2.0
2.5
3.0

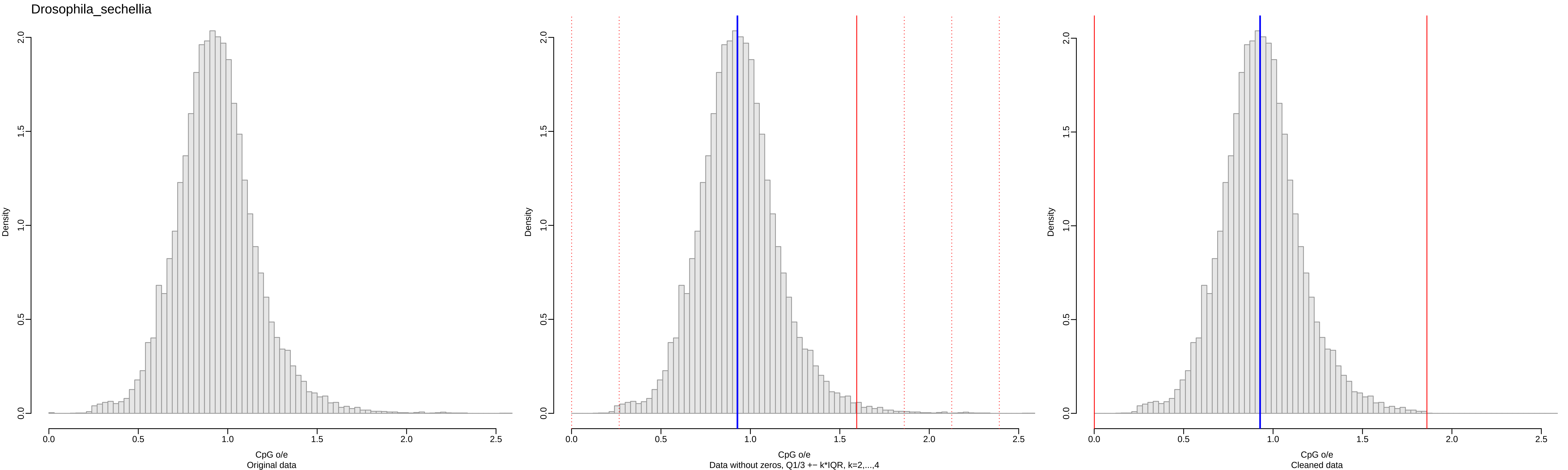

Drosophila_sechellia
Density
0.0
0.5
1.0
1.5
2.0
2.5
CpG o/e
Original data
Data without zeros, Q1/3 +− k*IQR, k=2,...,4
Cleaned data

Drosophila_serrata

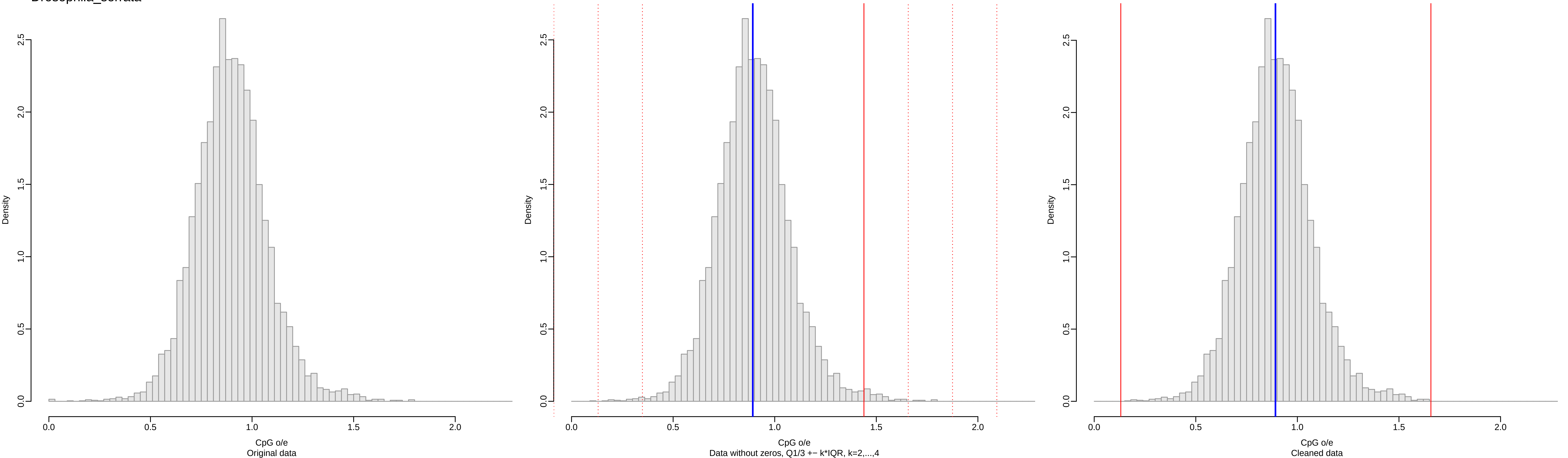

Drosophila_simulans

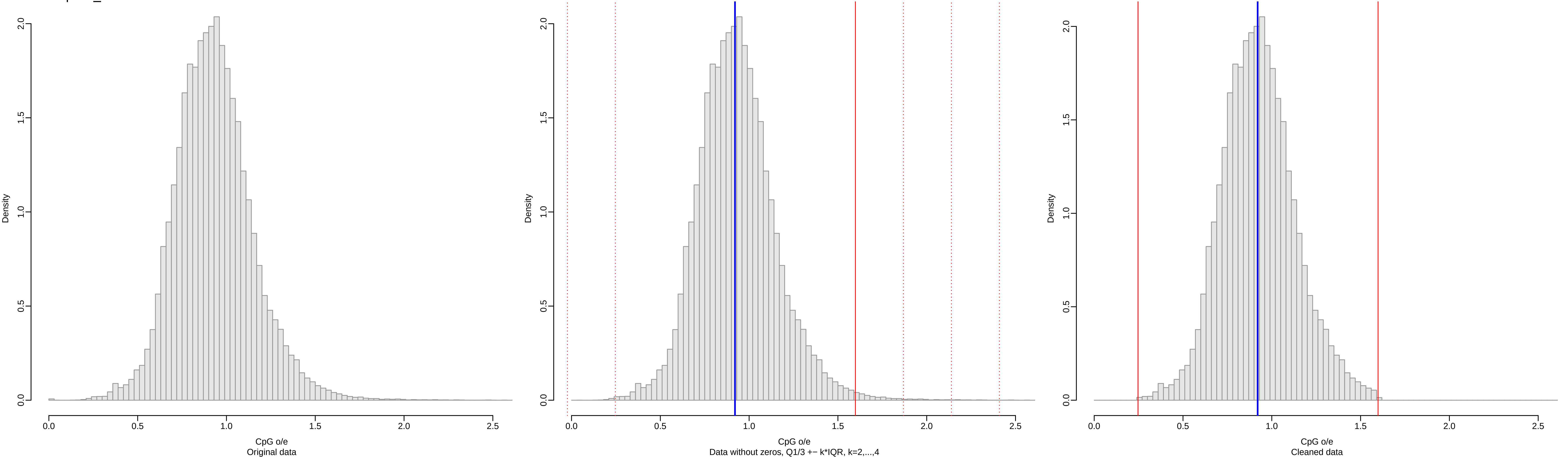

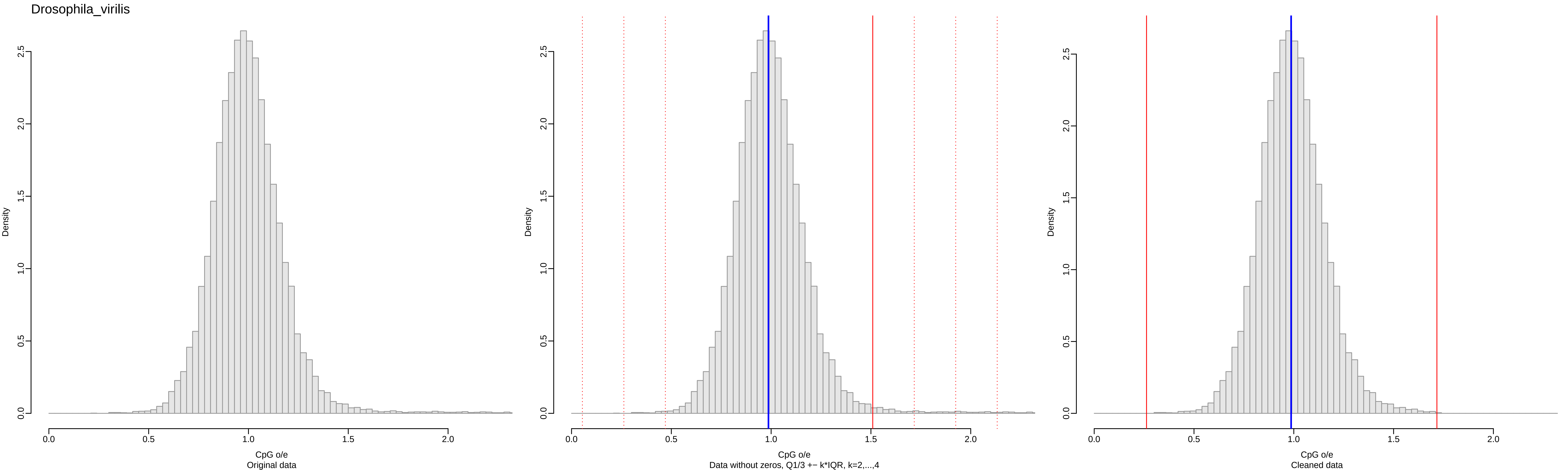
Drosophila_virilis
Density
CpG o/e
Original data
Data without zeros, Q1/3 +− k*IQR, k=2,...,4
Cleaned data
0.0
0.5
1.0
1.5
2.0
2.5

Drosophila_willistoni

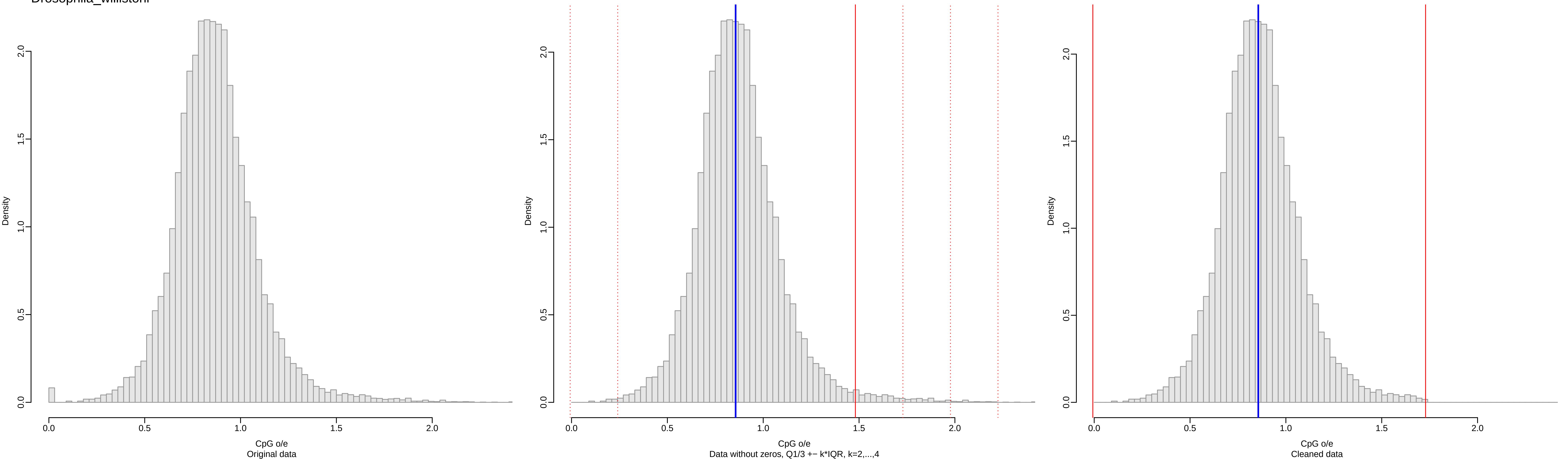

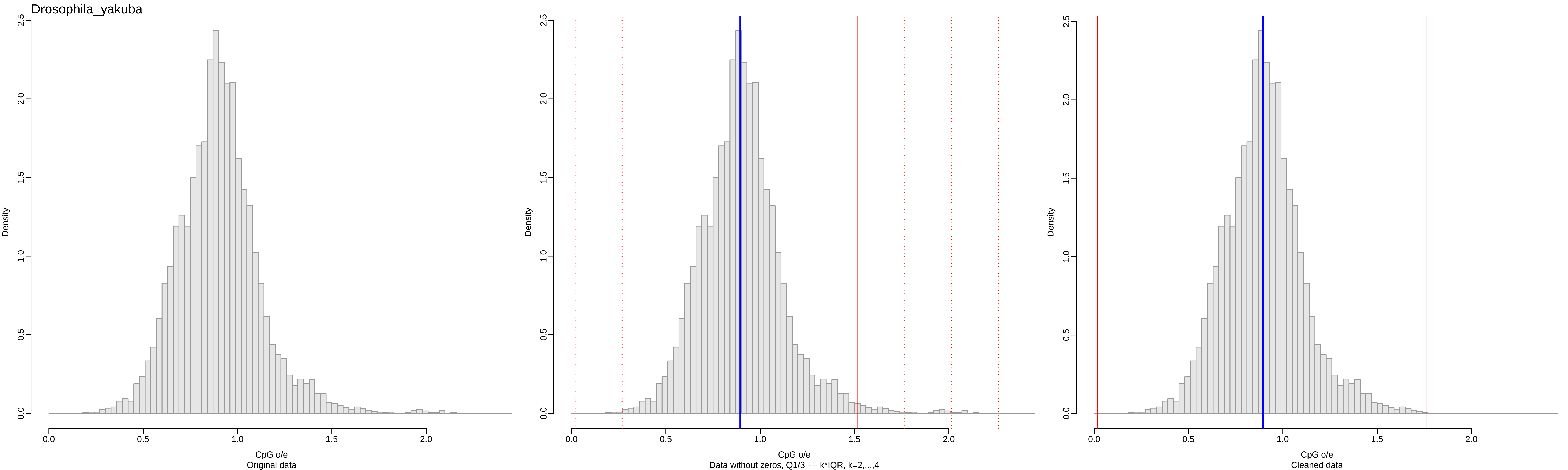

Drosophila_yakuba
Density
CpG o/e
Original data
Data without zeros, Q1/3 +− k*IQR, k=2,...,4
Cleaned data
0.0
0.5
1.0
1.5
2.0
2.5

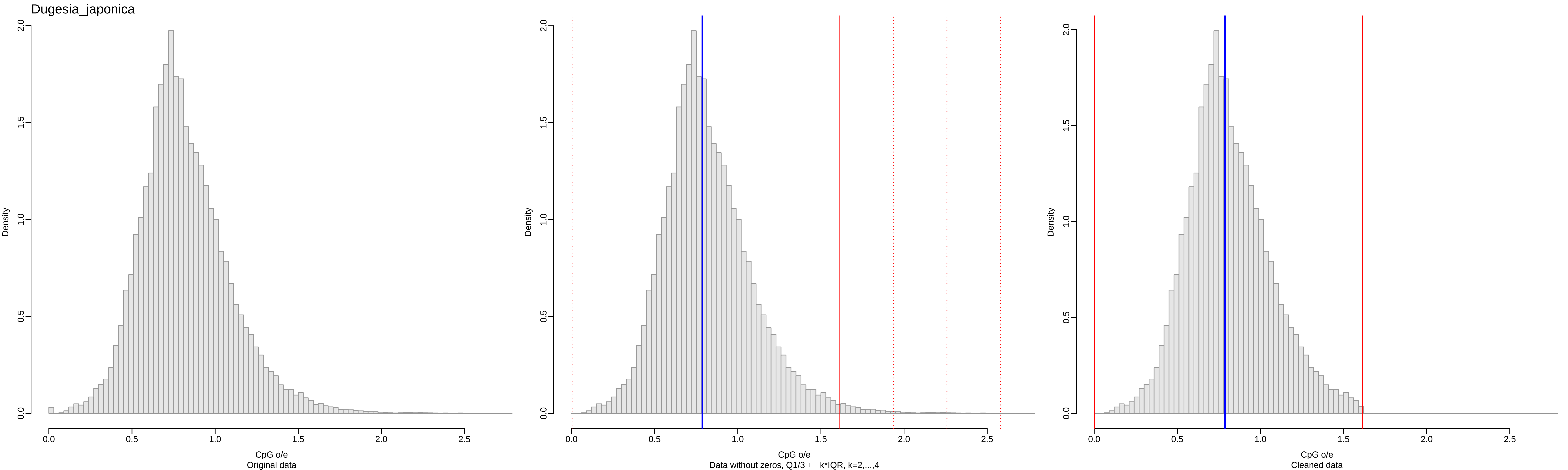
Dugesia_japonica
Density
CpG o/e
Original data
Density
CpG o/e
Data without zeros, Q1/3 +− k*IQR, k=2,...,4
Density
CpG o/e
Cleaned data

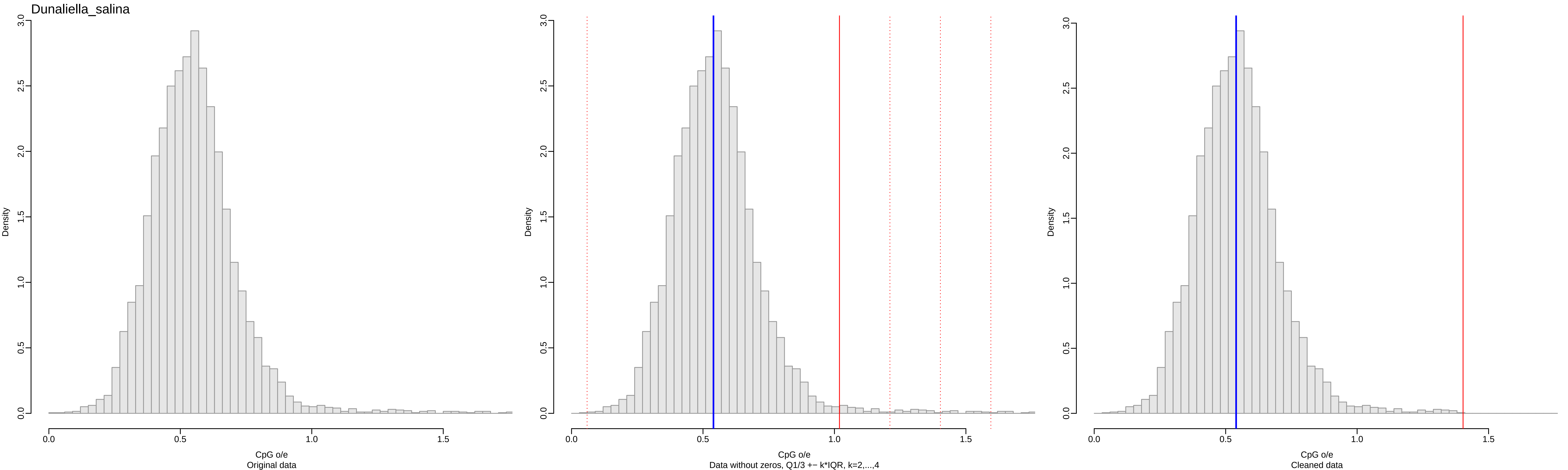

Dunaliella_salina
Density
CpG o/e
Original data
Density
CpG o/e
Data without zeros, Q1/3 +− k*IQR, k=2,...,4
Density
CpG o/e
Cleaned data

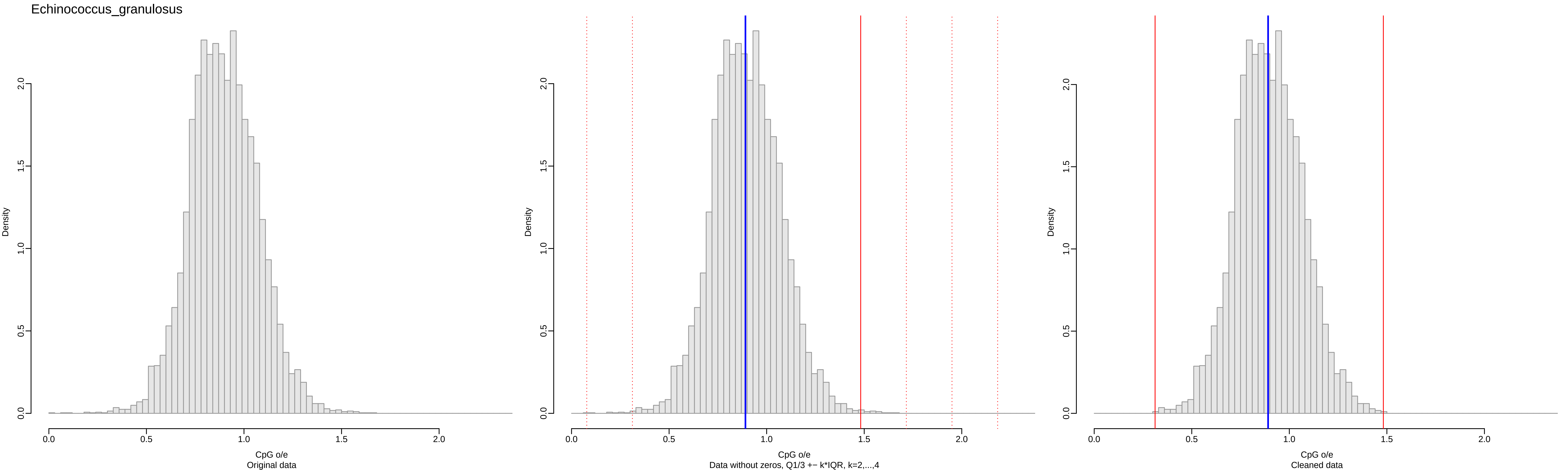
Echinococcus_granulosus
Density
0.0
0.5
1.0
1.5
2.0
CpG o/e
Original data
Data without zeros, Q1/3 +− k*IQR, k=2,...,4
Cleaned data

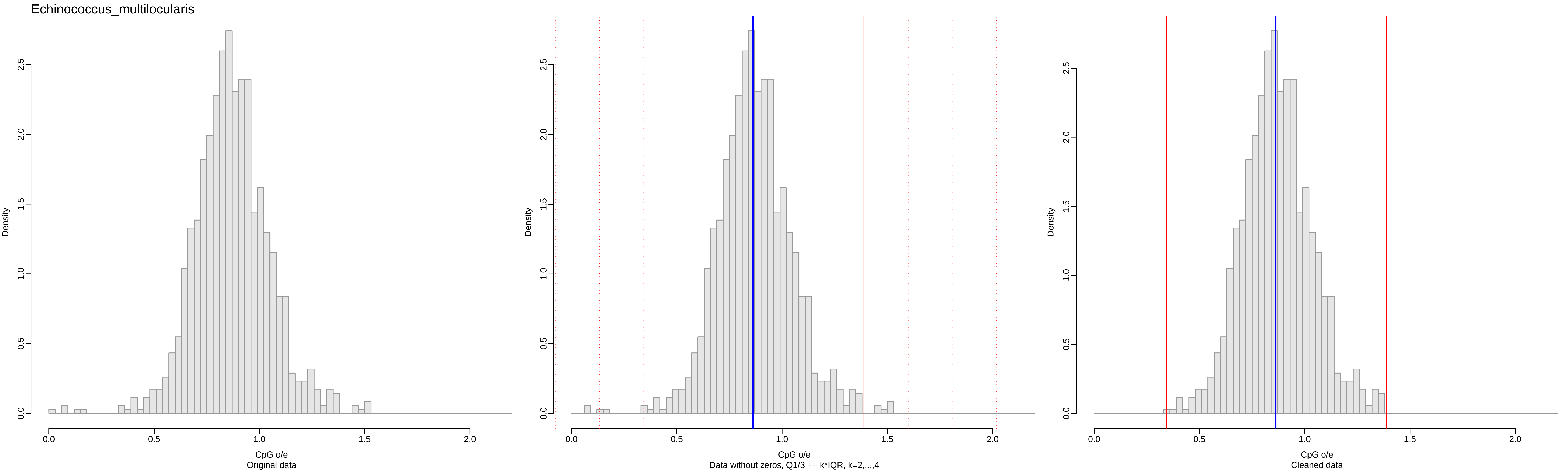

Echinococcus_multilocularis
Density
CpG o/e
Original data
Data without zeros, Q1/3 +− k*IQR, k=2,...,4
Cleaned data
0.0
0.5
1.0
1.5
2.0
2.5

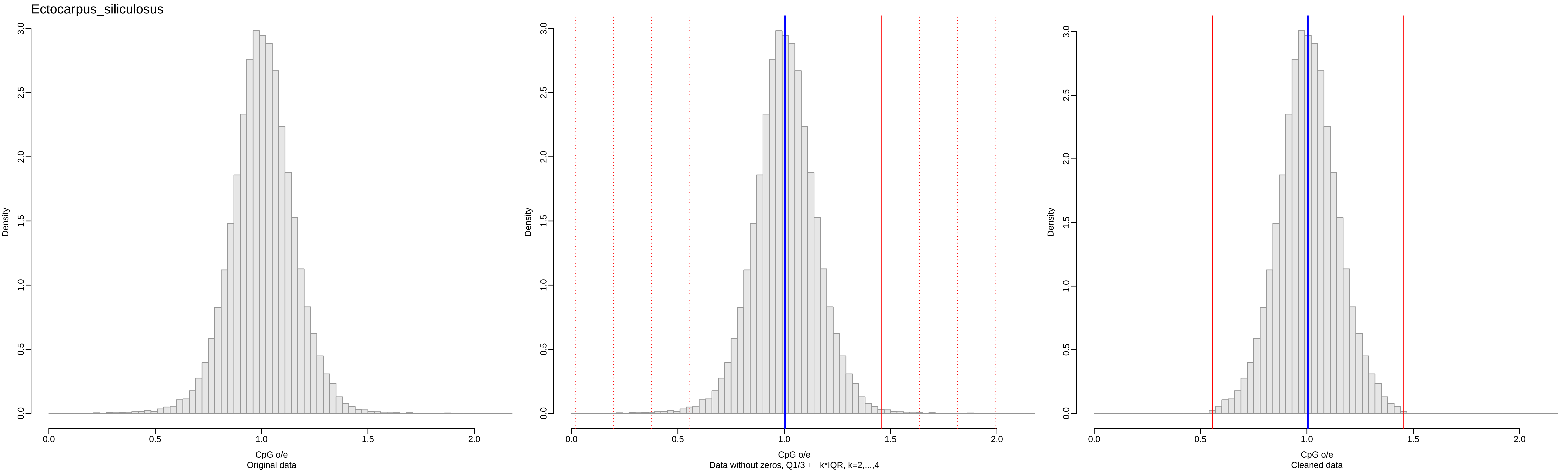

Ectocarpus_siliculosus
Density
CpG o/e
Original data
Data without zeros, Q1/3 +− k*IQR, k=2,...,4
Cleaned data
0.0
0.5
1.0
1.5
2.0
2.5
3.0

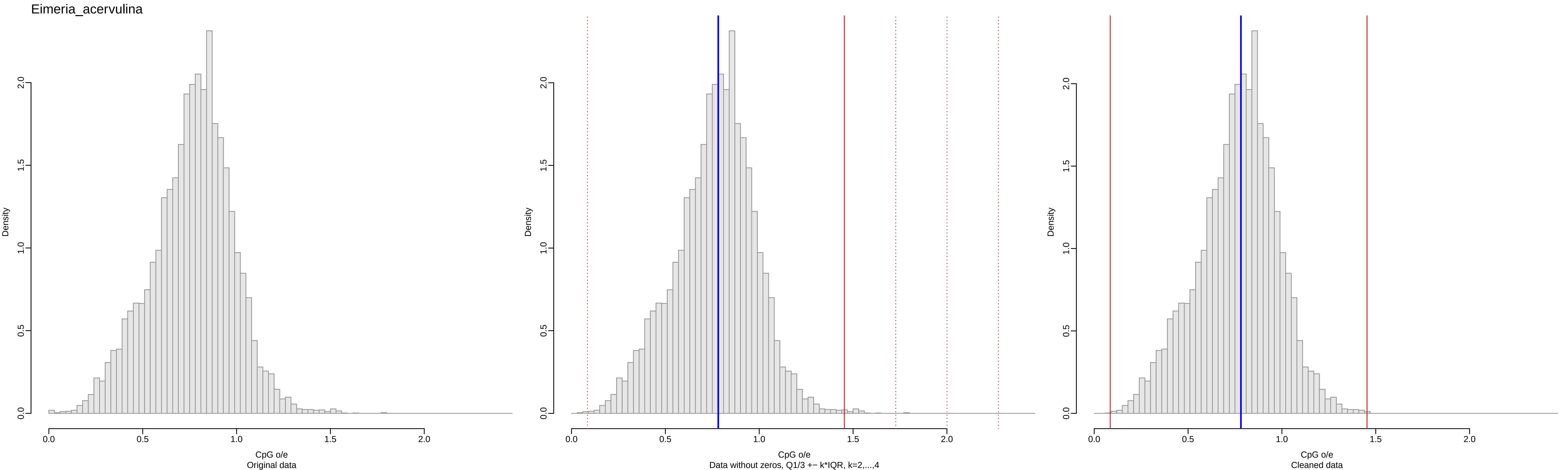

Eimeria_acervulina
Density
CpG o/e
Original data
Data without zeros, Q1/3 +− k*IQR, k=2,...,4
Cleaned data
0.0
0.5
1.0
1.5
2.0

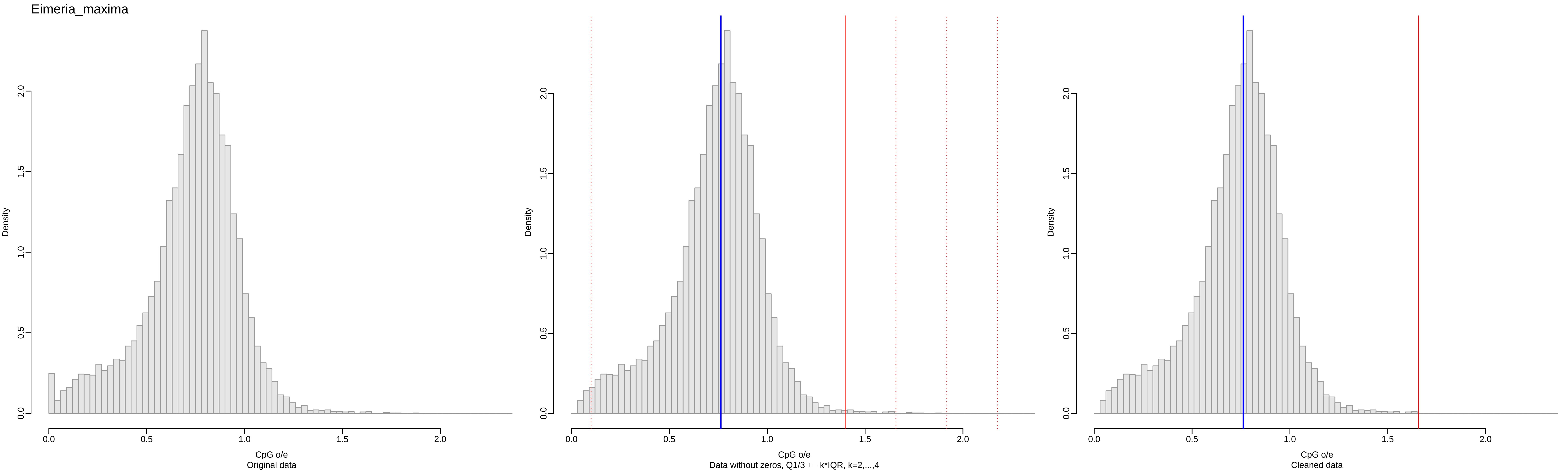
Eimeria_maxima
Density
CpG o/e
Original data
Density
CpG o/e
Data without zeros, Q1/3 +− k*IQR, k=2,...,4
Density
CpG o/e
Cleaned data

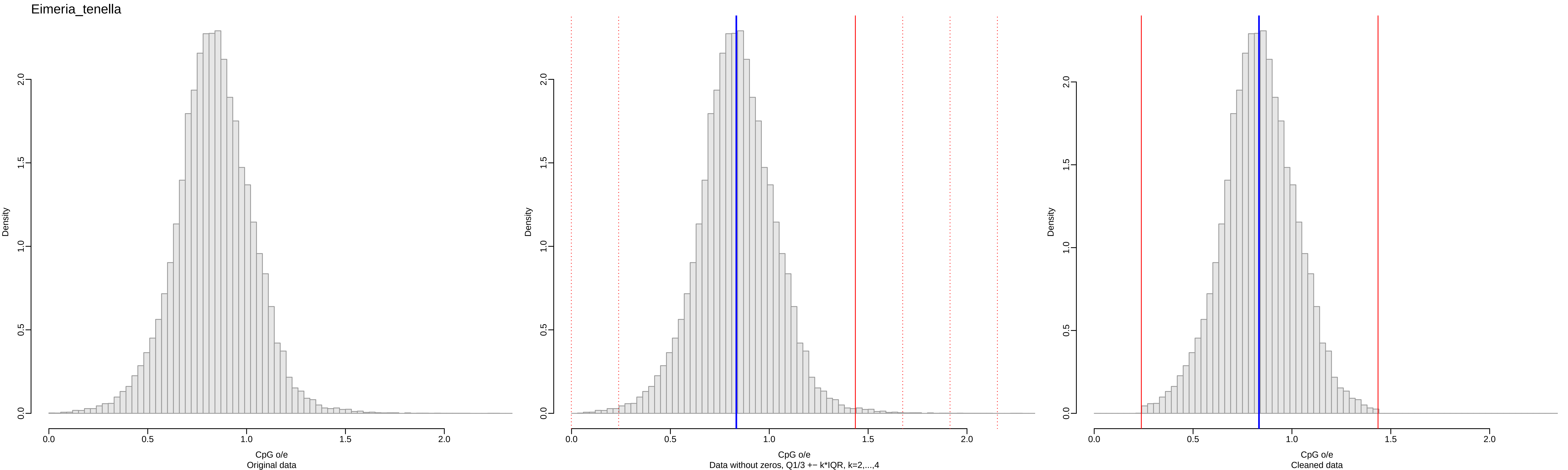

Eimeria_tenella
Density
0.0
0.5
1.0
1.5
2.0
CpG o/e
Original data
Data without zeros, Q1/3 +− k*IQR, k=2,...,4
Cleaned data

Elaeis_guineensis

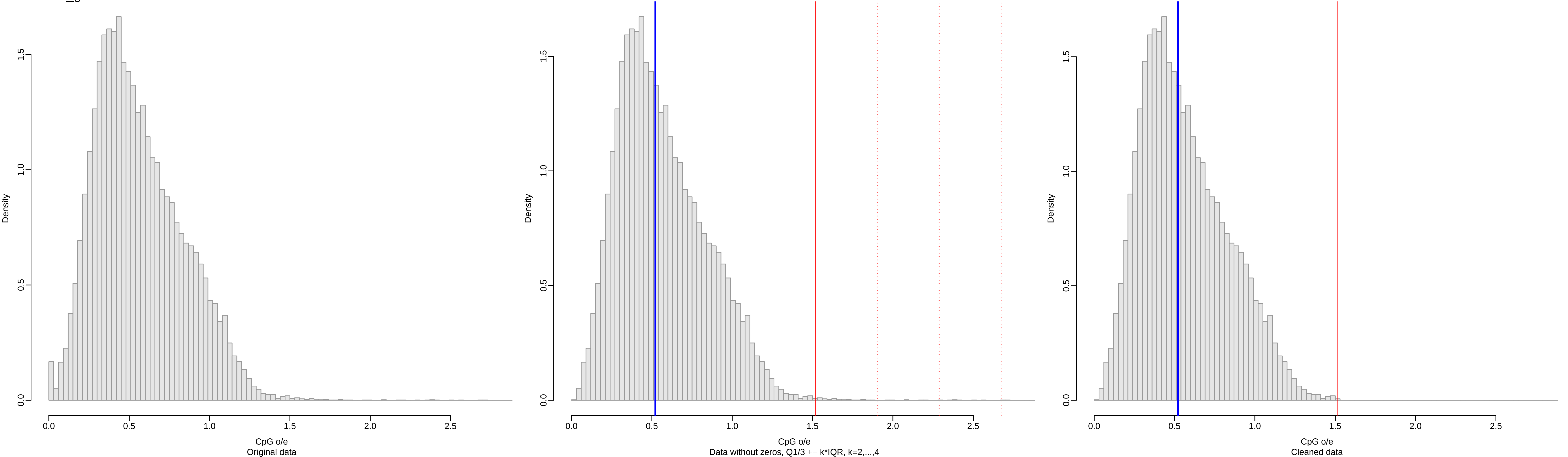

Elaphe_quadrivirgata

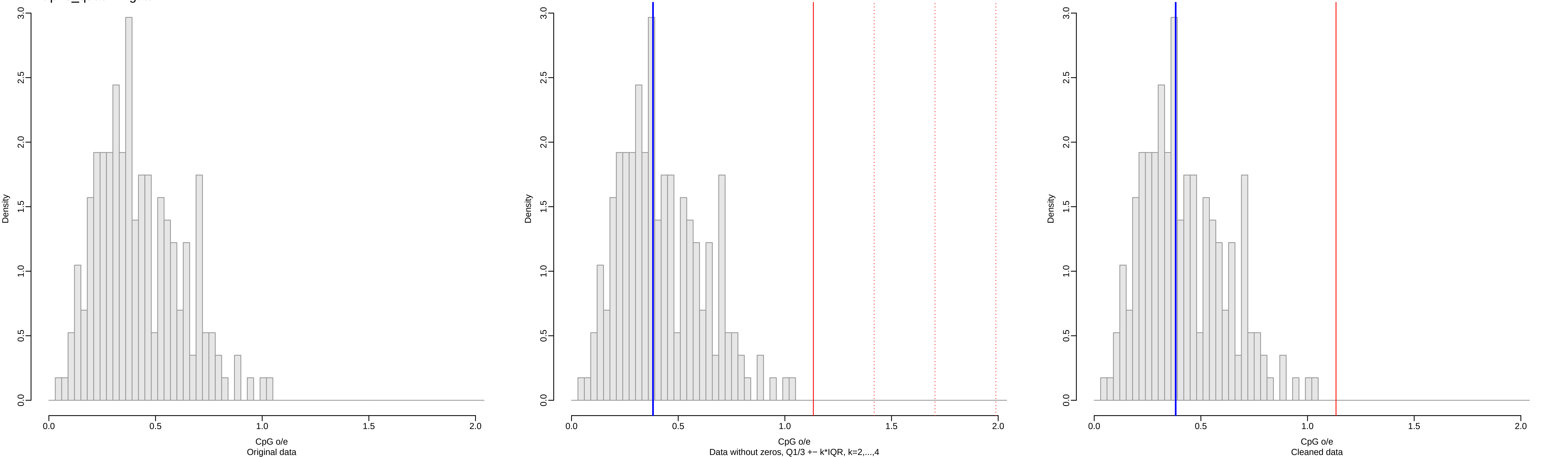

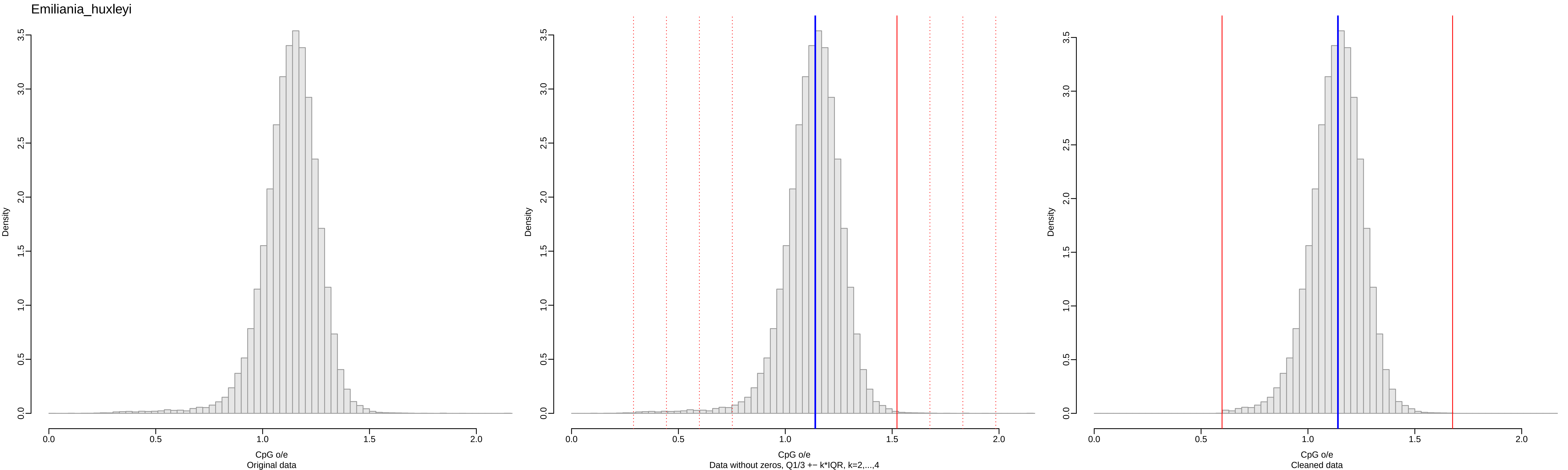

Emiliania_huxleyi
Density
CpG o/e
Original data
Data without zeros, Q1/3 +− k*IQR, k=2,...,4
Cleaned data
0.0
0.5
1.0
1.5
2.0
2.5
3.0
3.5

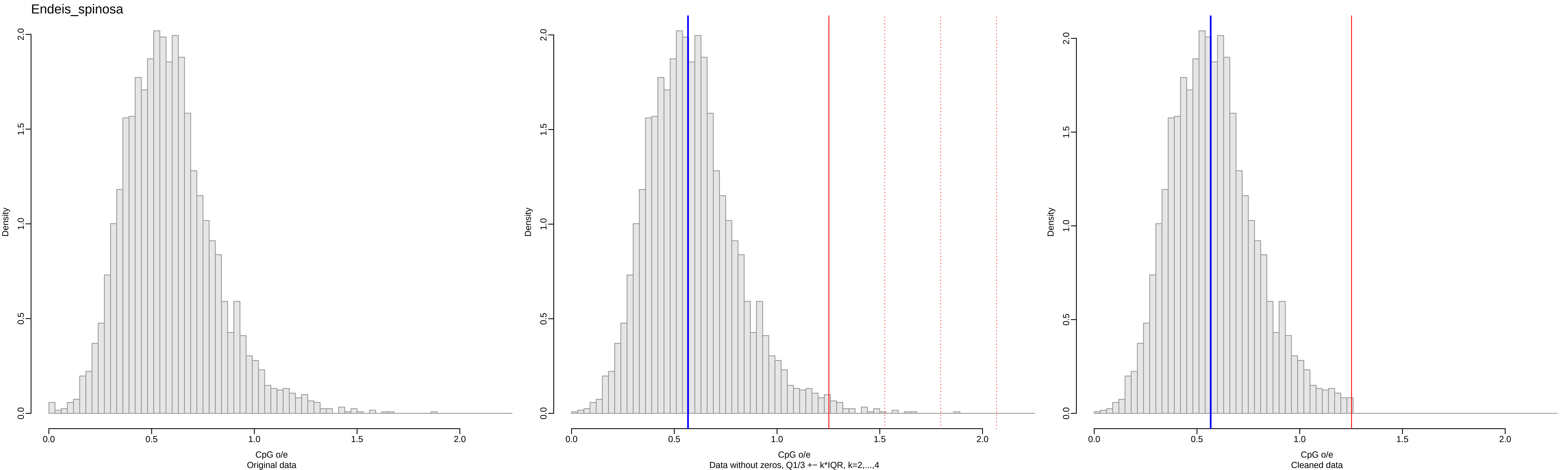
Endeis_spinosa
Density
CpG o/e
Original data
Data without zeros, Q1/3 +− k*IQR, k=2,...,4
Cleaned data
0.0
0.5
1.0
1.5
2.0

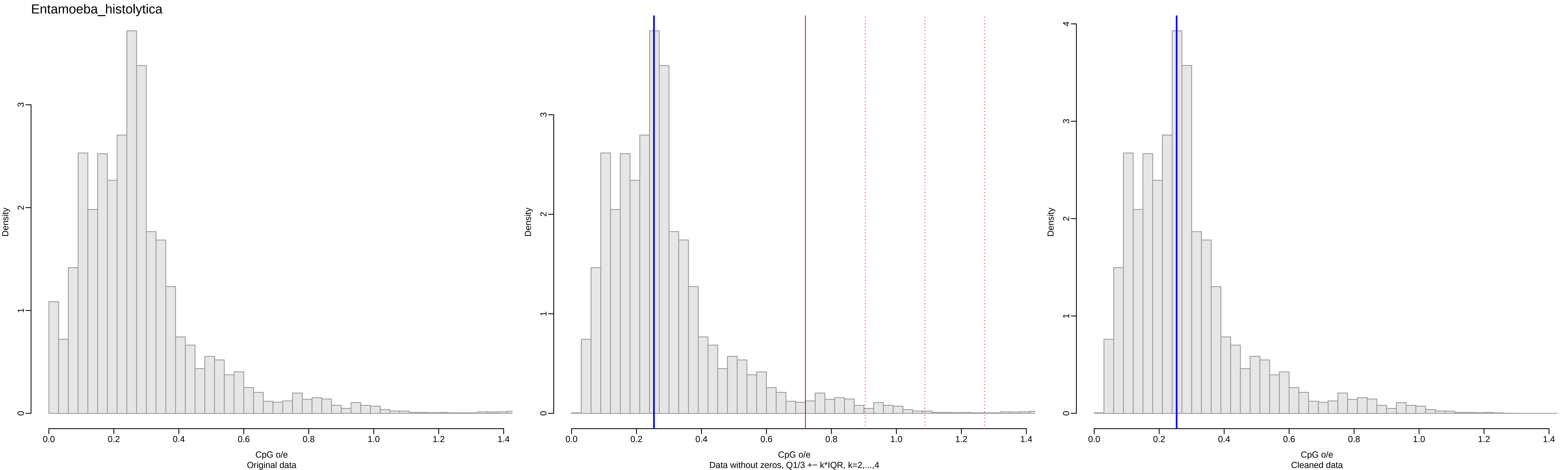
Entamoeba_histolytica
Density
CpG o/e
Original data
Density
CpG o/e
Data without zeros, Q1/3 +− k*IQR, k=2,...,4
Density
CpG o/e
Cleaned data
0.0
0.2
0.4
0.6
0.8
1.0
1.2
1.4
0
1
2
3
4

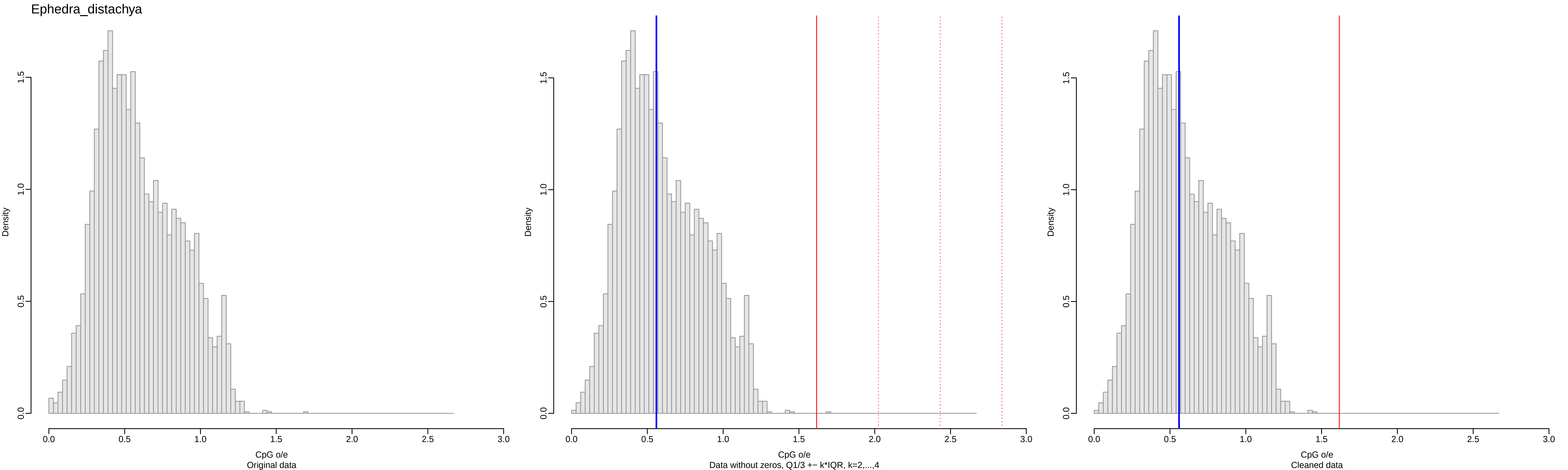

Ephedra_distachya
Density
CpG o/e
Original data
Data without zeros, Q1/3 +− k*IQR, k=2,...,4
Cleaned data
0.0
0.5
1.0
1.5
2.0
2.5
3.0

Epichloe_festucae

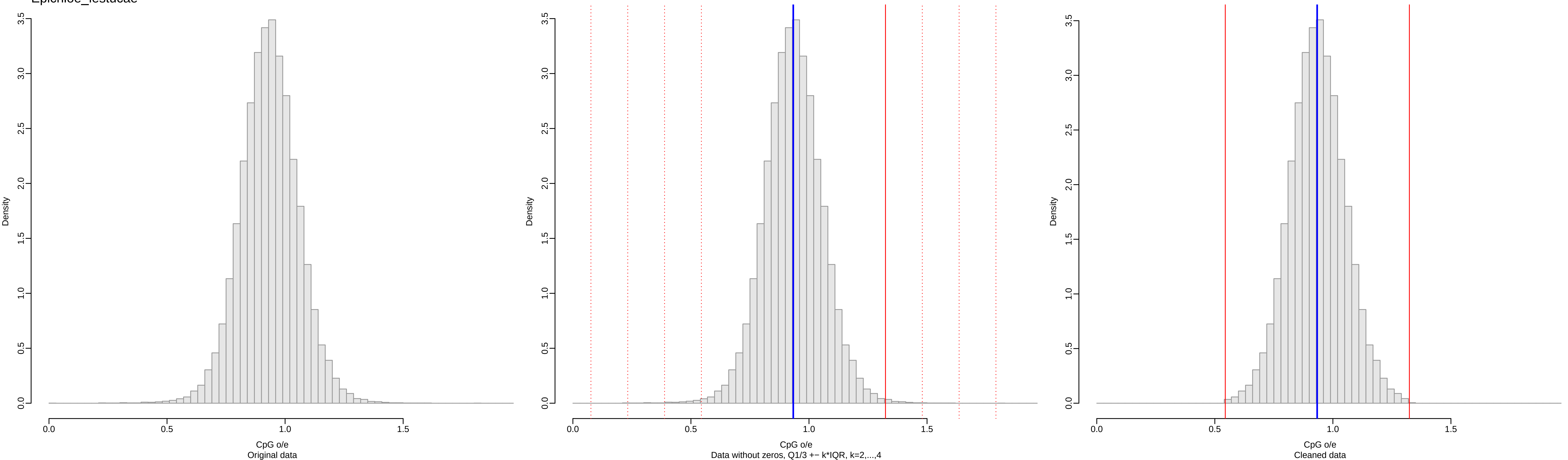

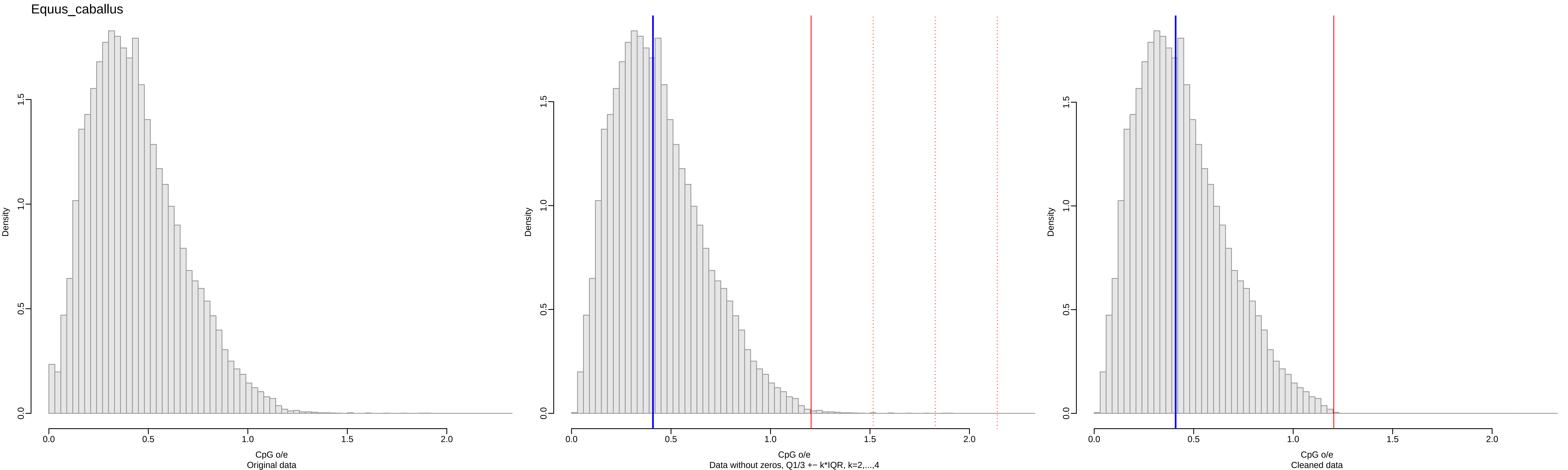

Equus_caballus
Density
CpG o/e
Original data
Data without zeros, Q1/3 +− k*IQR, k=2,...,4
Cleaned data

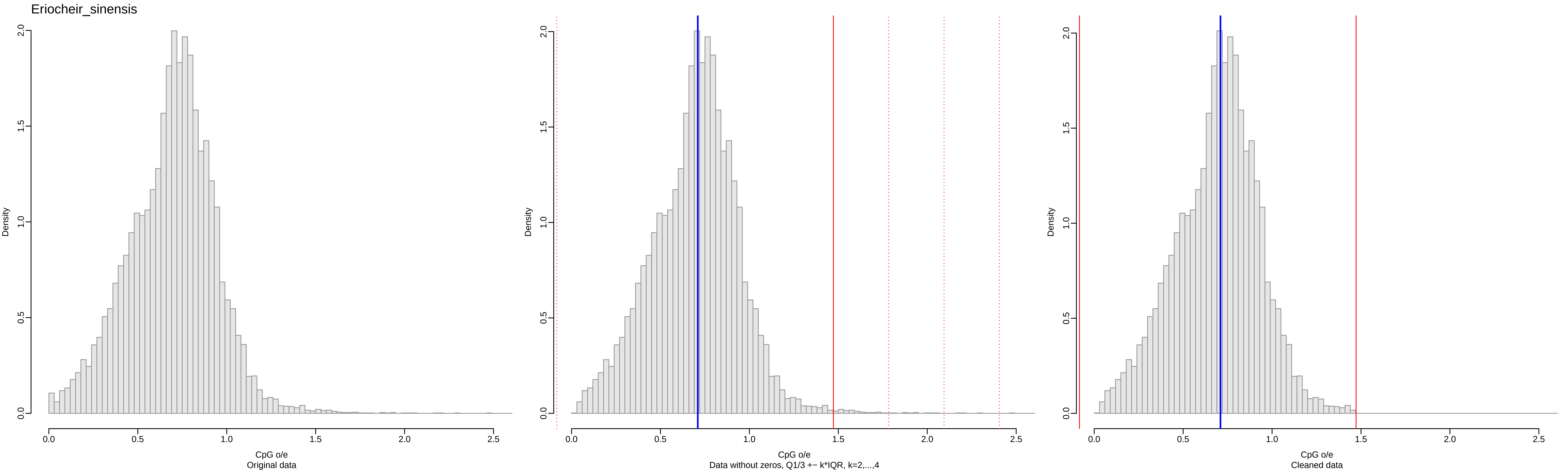

Eriocheir_sinensis
Density
CpG o/e
Original data
Density
CpG o/e
Data without zeros, Q1/3 +− k*IQR, k=2,...,4
Density
CpG o/e
Cleaned data

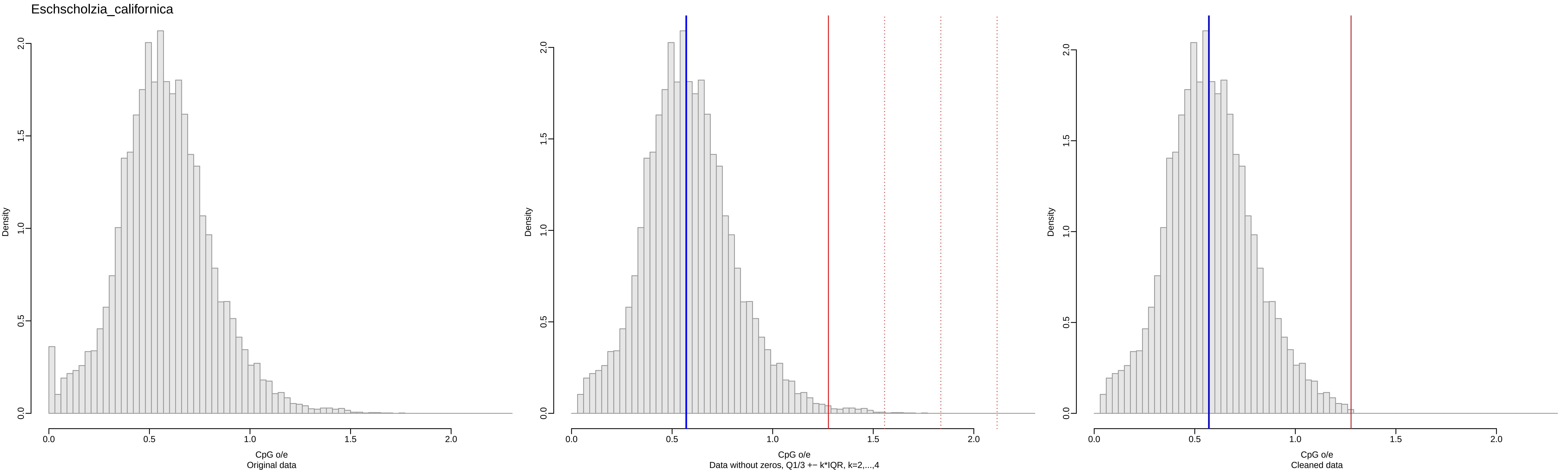
Eschscholzia_californica
Density
CpG o/e
Original data
CpG o/e
Data without zeros, Q1/3 +− k*IQR, k=2,...,4
CpG o/e
Cleaned data
0.0
0.5
1.0
1.5
2.0

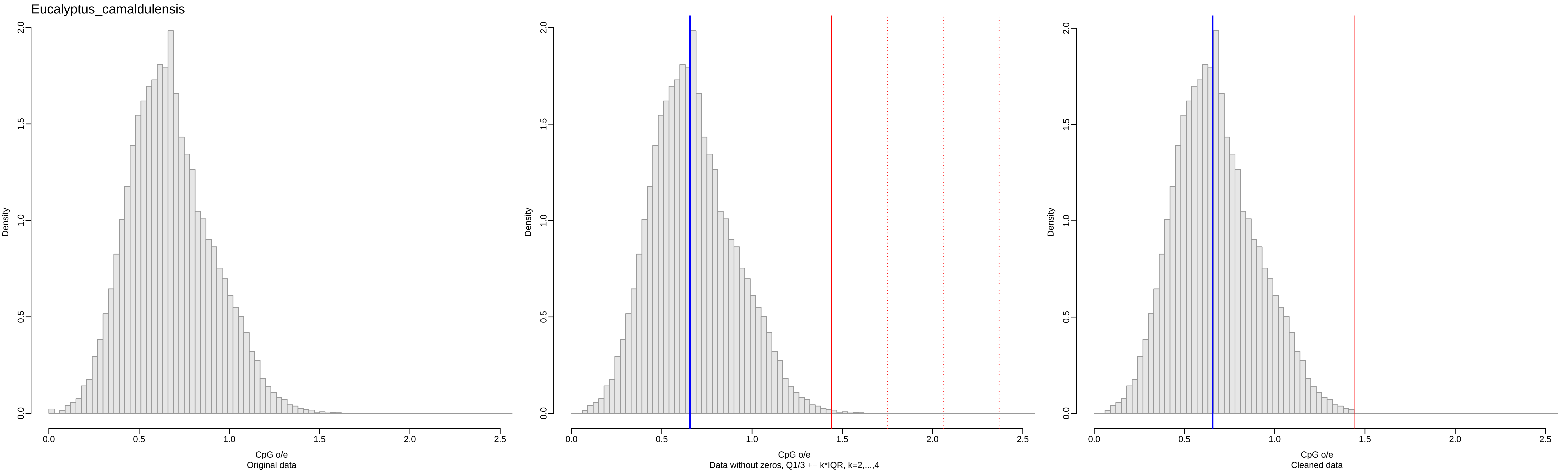
Eucalyptus_camaldulensis
Density
CpG o/e
Original data
Data without zeros, Q1/3 +− k*IQR, k=2,...,4
Cleaned data
0.0
0.5
1.0
1.5
2.0
2.5

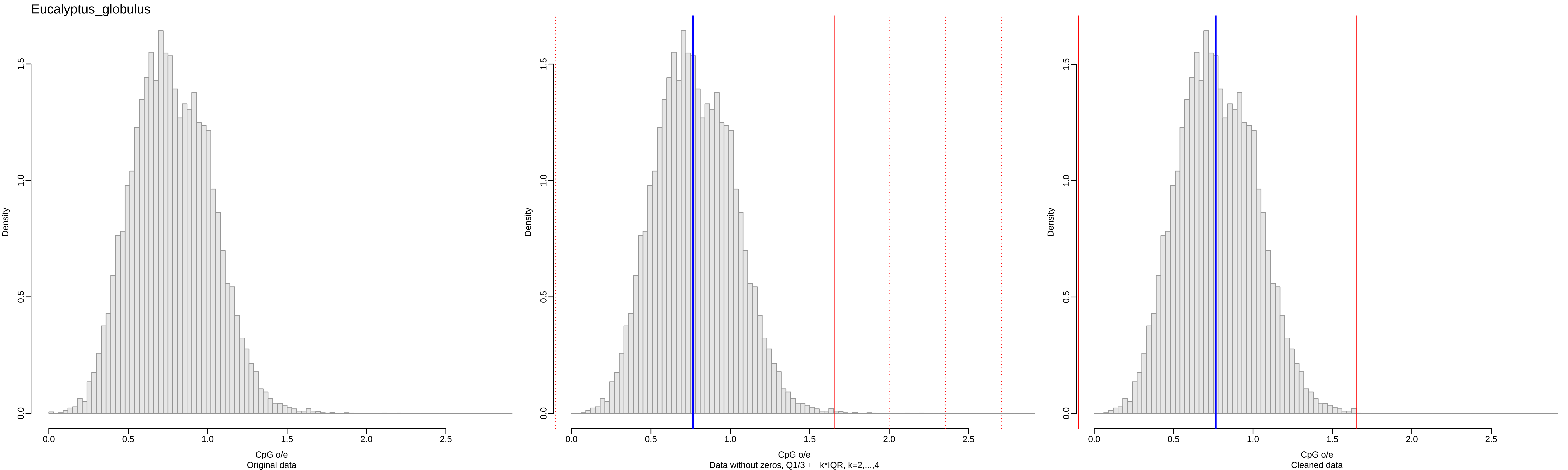

Eucalyptus_globulus
Density
0.0
0.5
1.0
1.5
2.0
2.5
CpG o/e
Original data
Data without zeros, Q1/3 +− k*IQR, k=2,...,4
Cleaned data

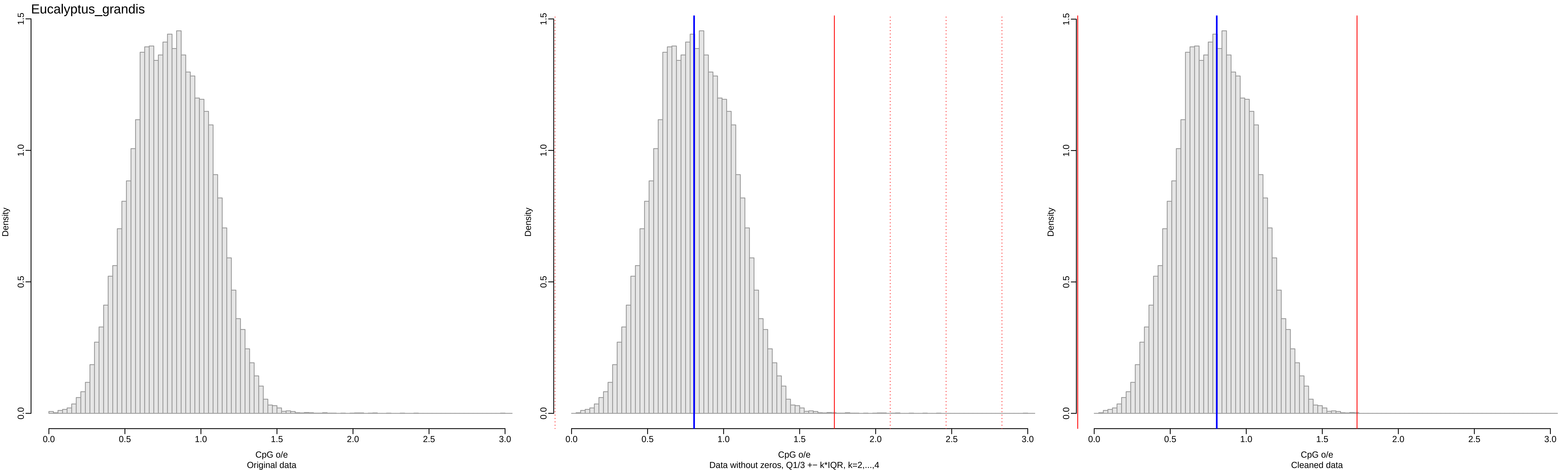
Eucalyptus_grandis
Density
CpG o/e
Original data
Density
CpG o/e
Data without zeros, Q1/3 +− k*IQR, k=2,...,4
Density
CpG o/e
Cleaned data

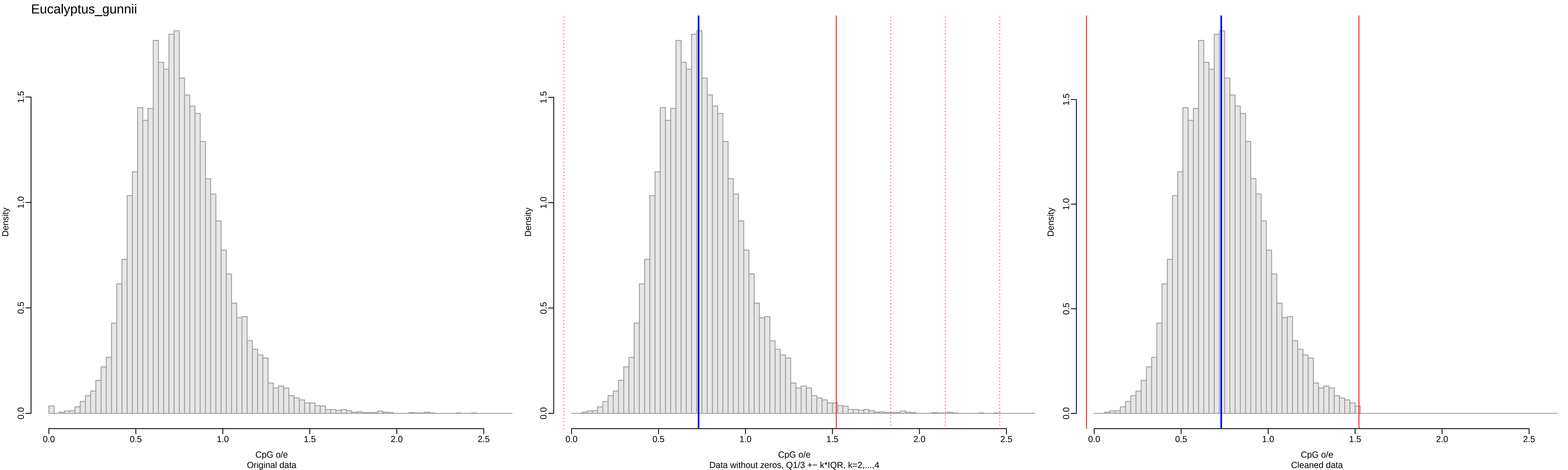

Eucalyptus_gunnii
Density
CpG o/e
Original data
Data without zeros, Q1/3 +− k*IQR, k=2,...,4
Cleaned data
0.0
0.5
1.0
1.5
2.0
2.5

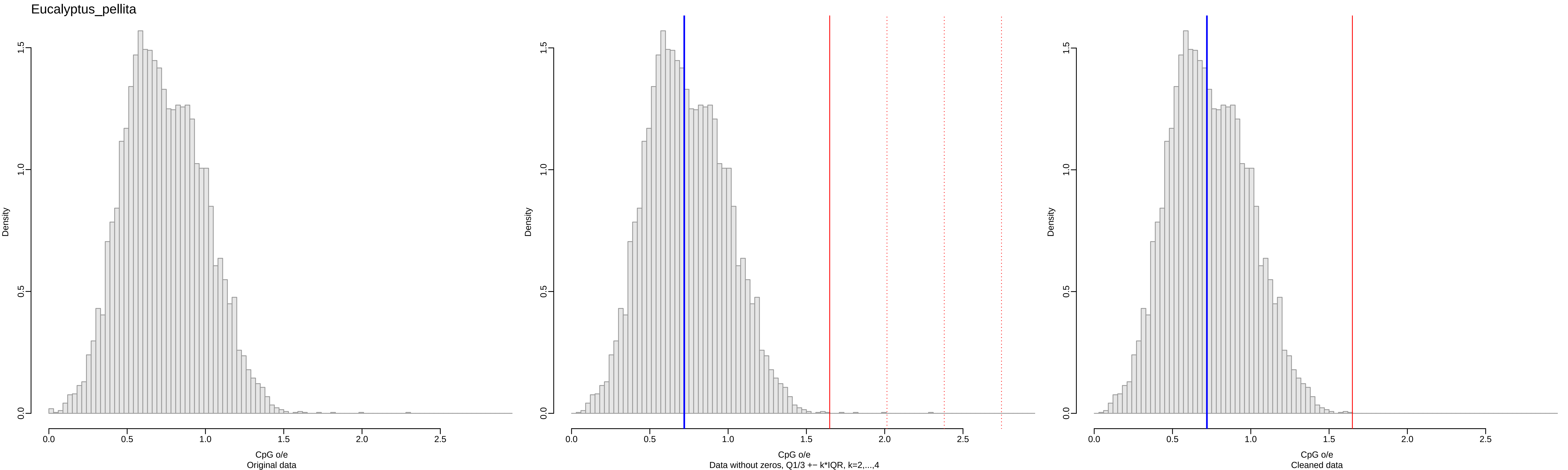
Eucalyptus_pellita
Density
CpG o/e
Original data
Data without zeros, Q1/3 +− k*IQR, k=2,...,4
Cleaned data
0.0
0.5
1.0
1.5
2.0
2.5

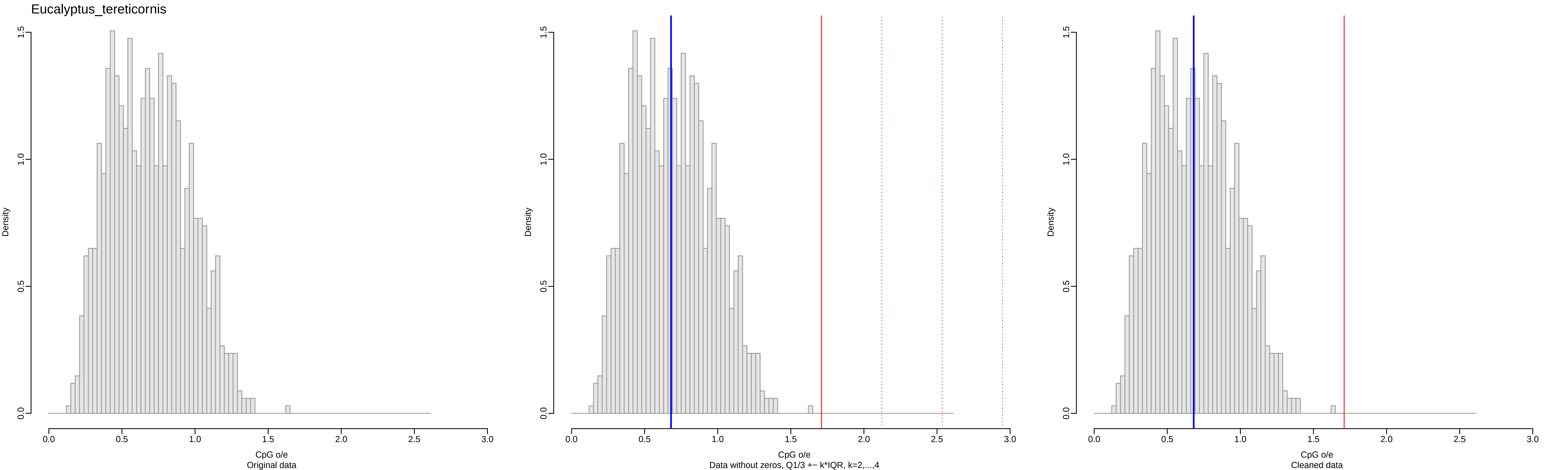

Eucalyptus_tereticornis
Density
CpG o/e
Original data
Density
CpG o/e
Data without zeros, Q1/3 +− k*IQR, k=2,...,4
Density
CpG o/e
Cleaned data

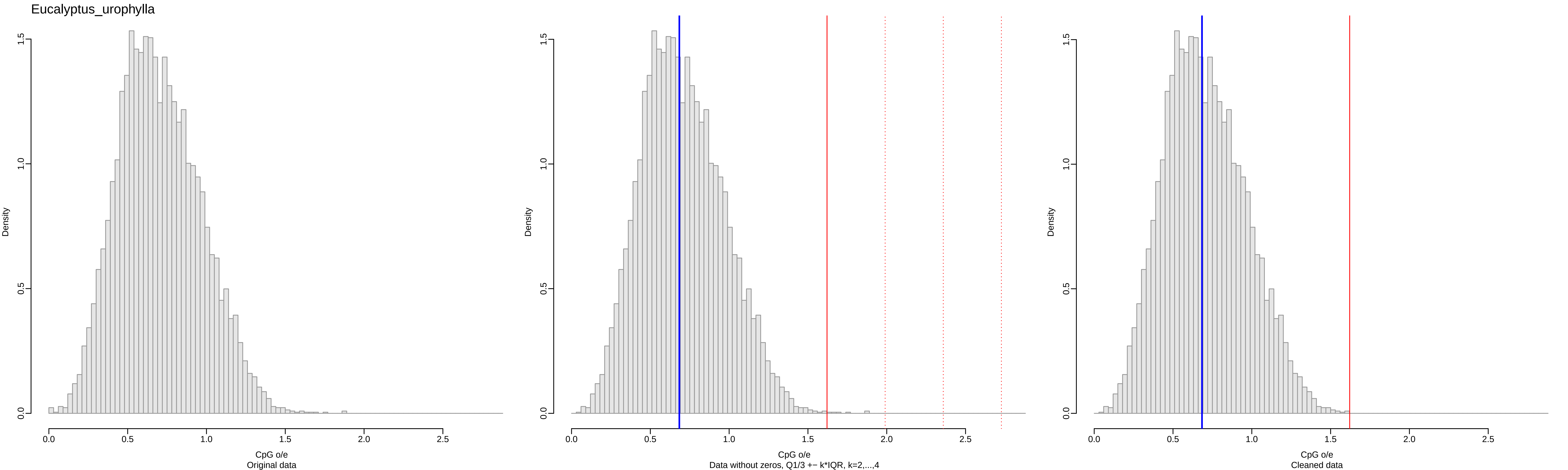

Eucalyptus_urophylla
Density
0.0
0.5
1.0
1.5
2.0
2.5
CpG o/e
Original data
Data without zeros, Q1/3 +− k*IQR, k=2,...,4
Cleaned data

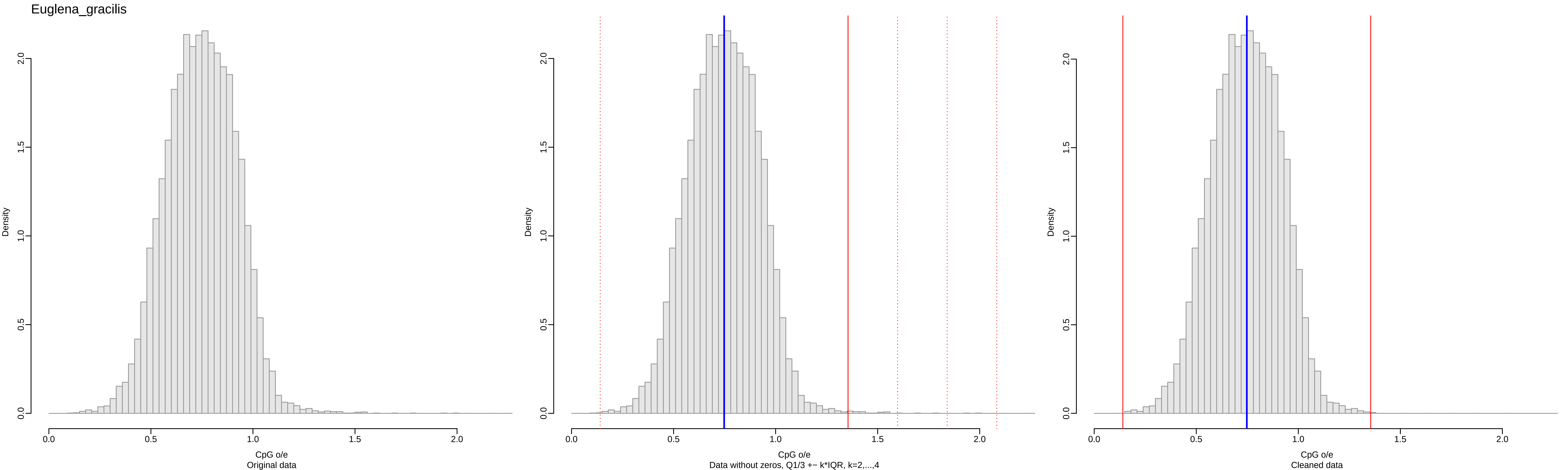
Euglena_gracilis
Density
CpG o/e
Original data
CpG o/e
Data without zeros, Q1/3 +− k*IQR, k=2,...,4
CpG o/e
Cleaned data

Euprymna_scolopes

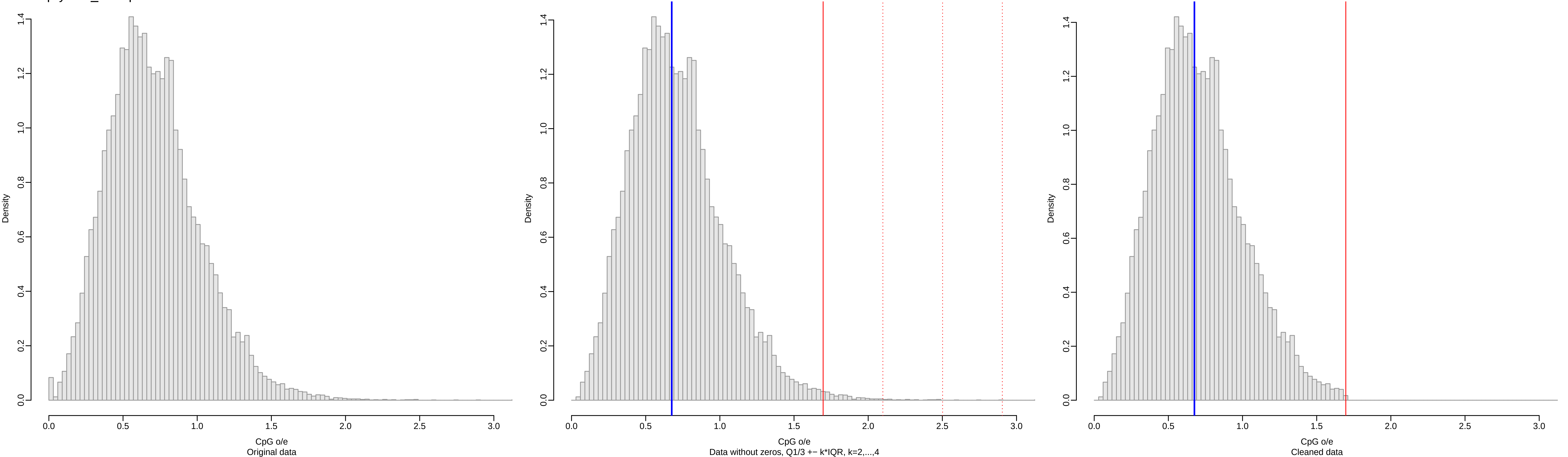

Fasciola_gigantica

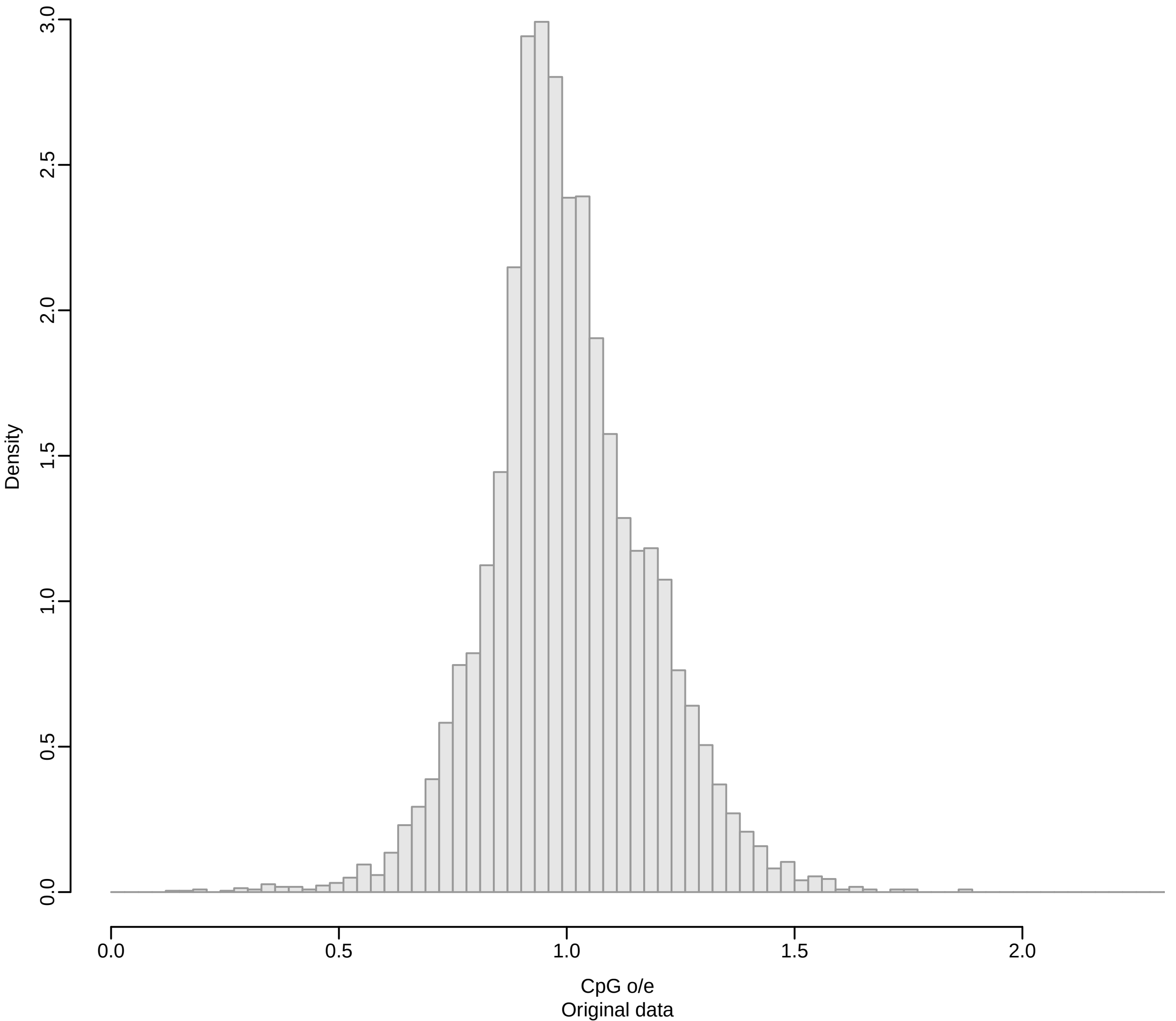

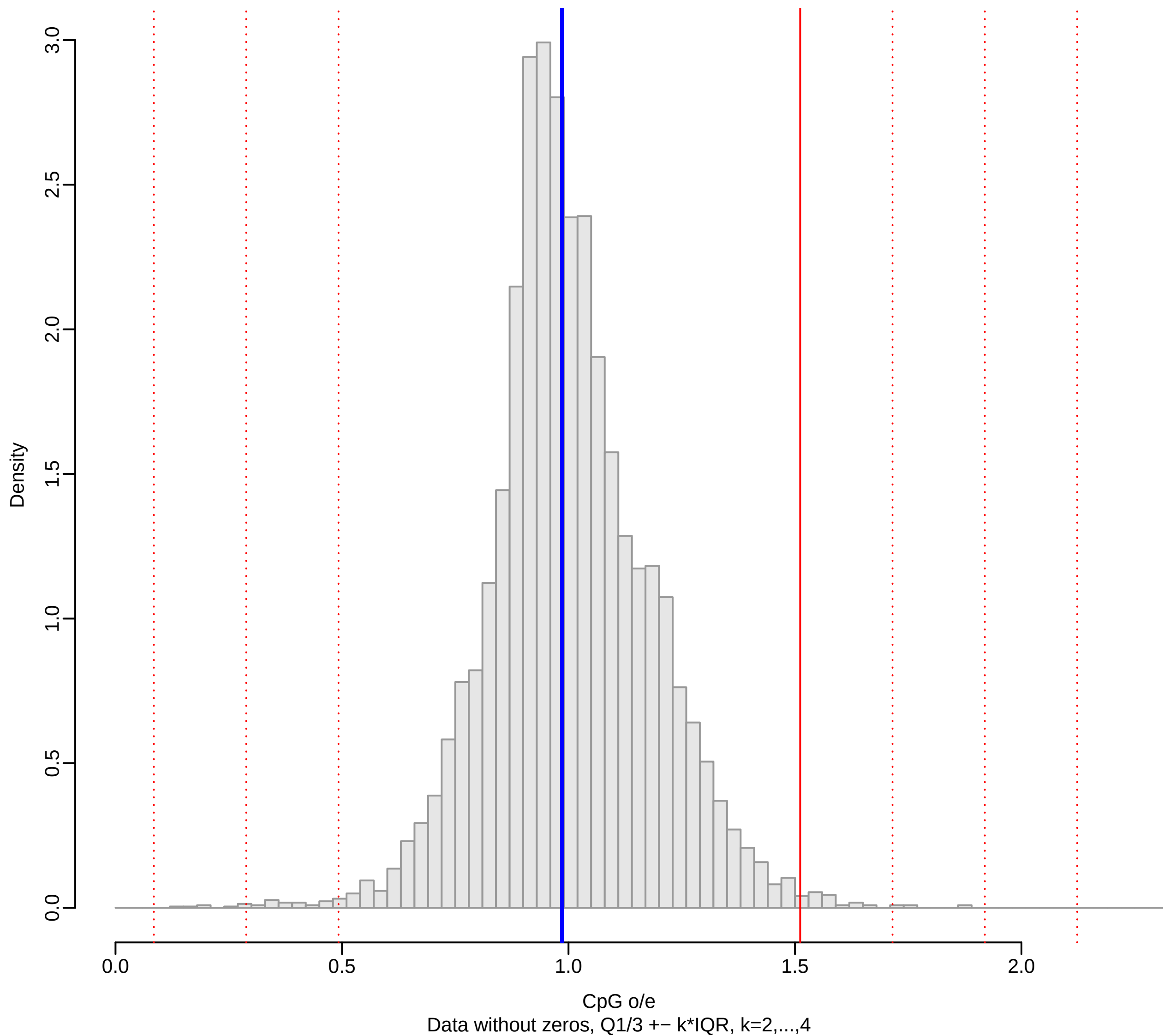

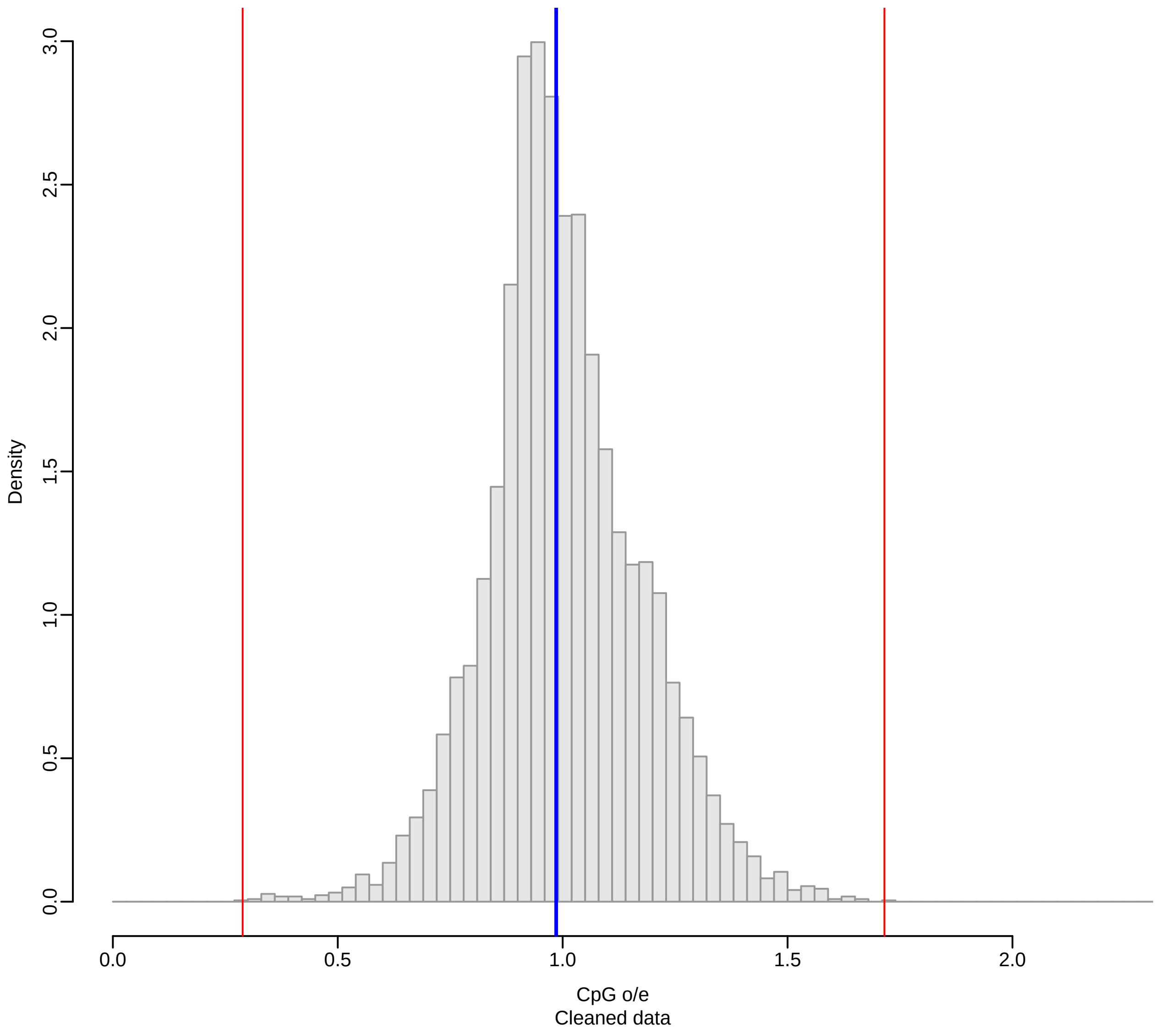

Fasciola_hepatica

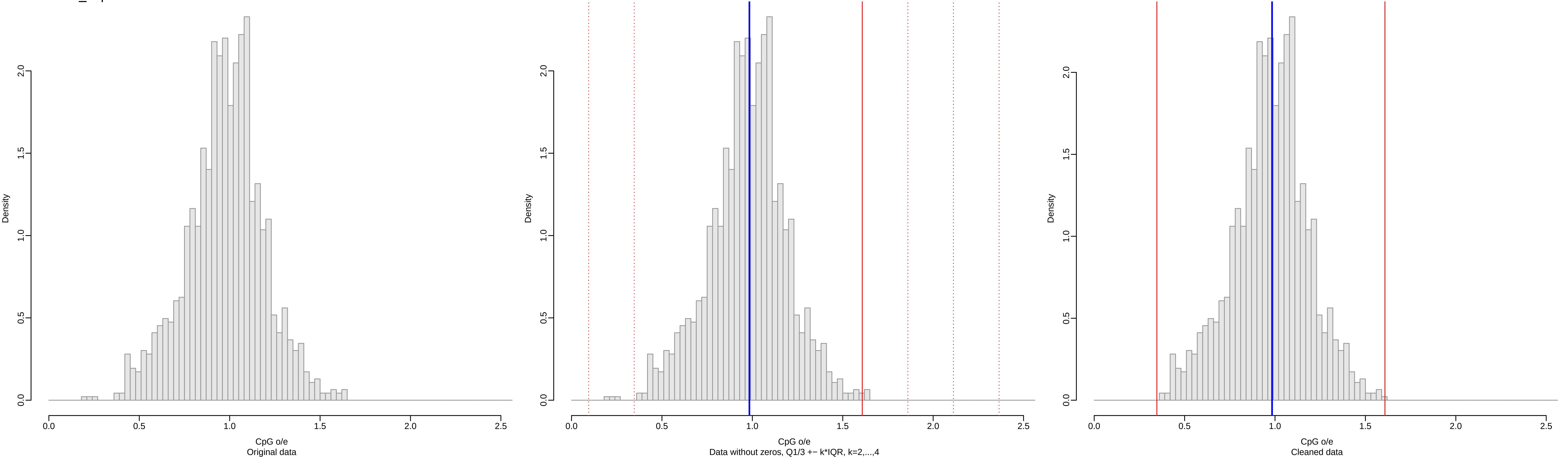

Fenneropenaeus_chinensis

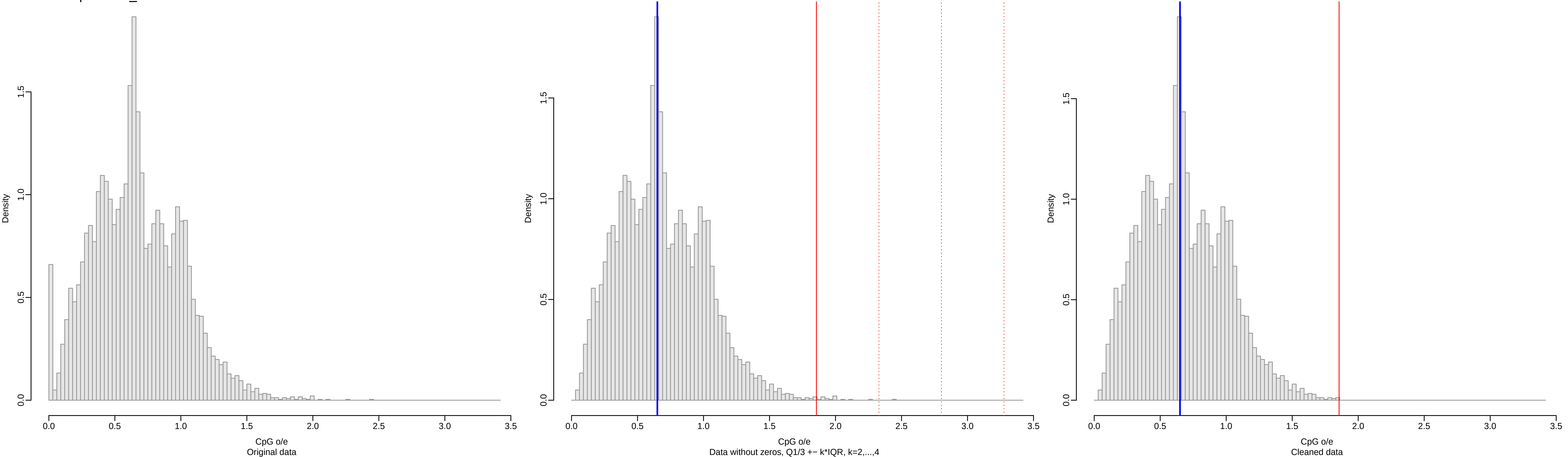

Festuca_pratensis

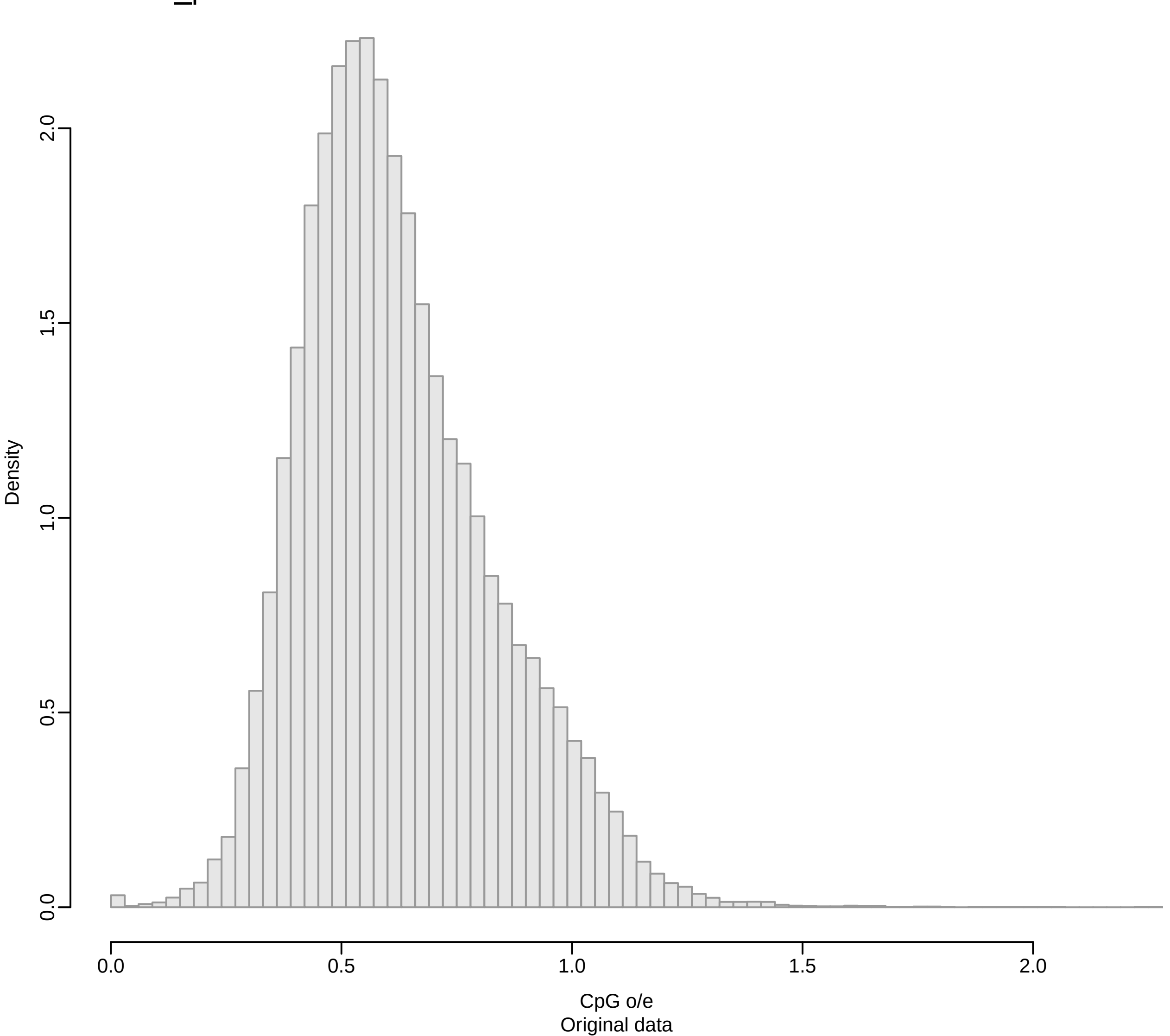

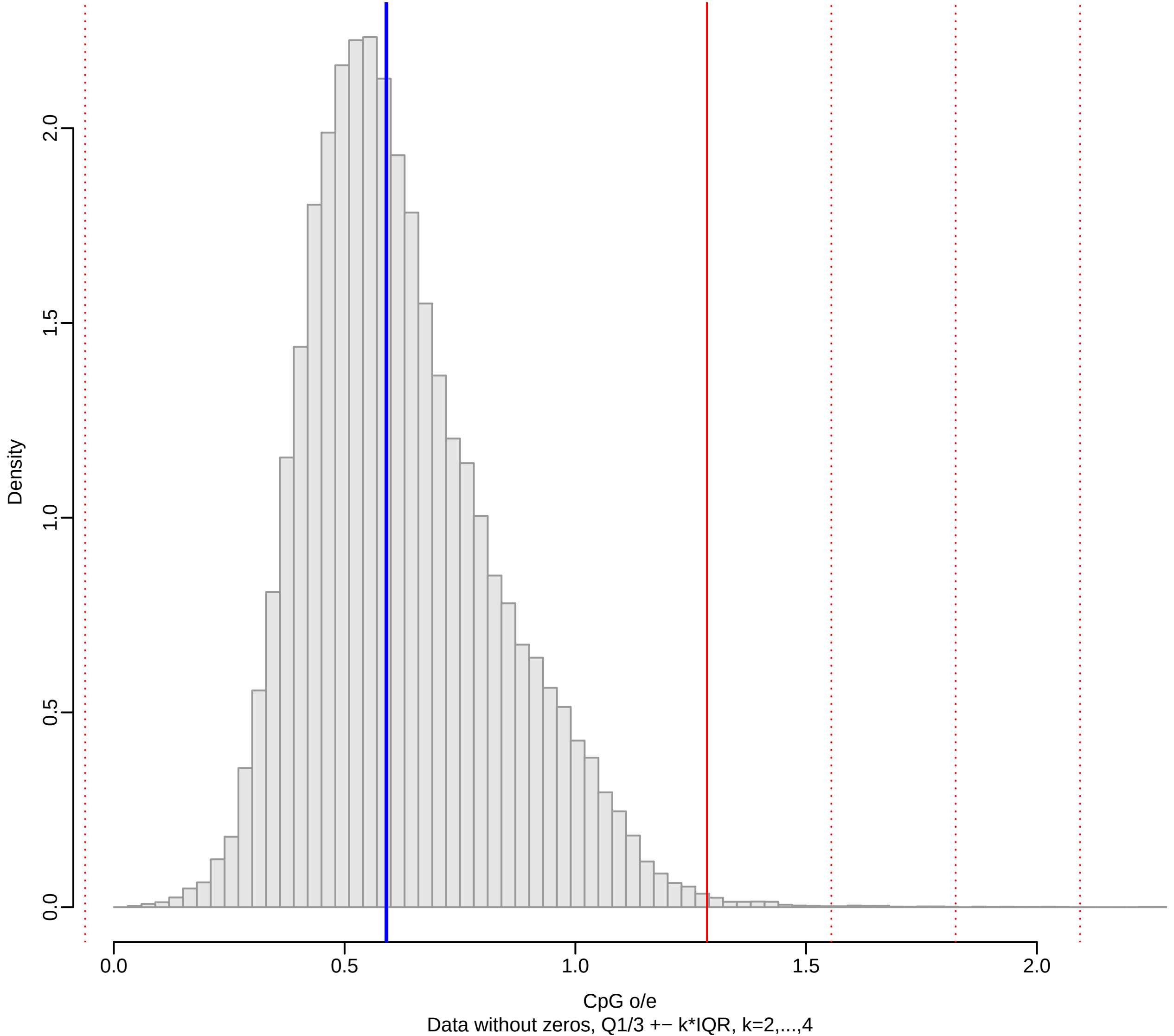

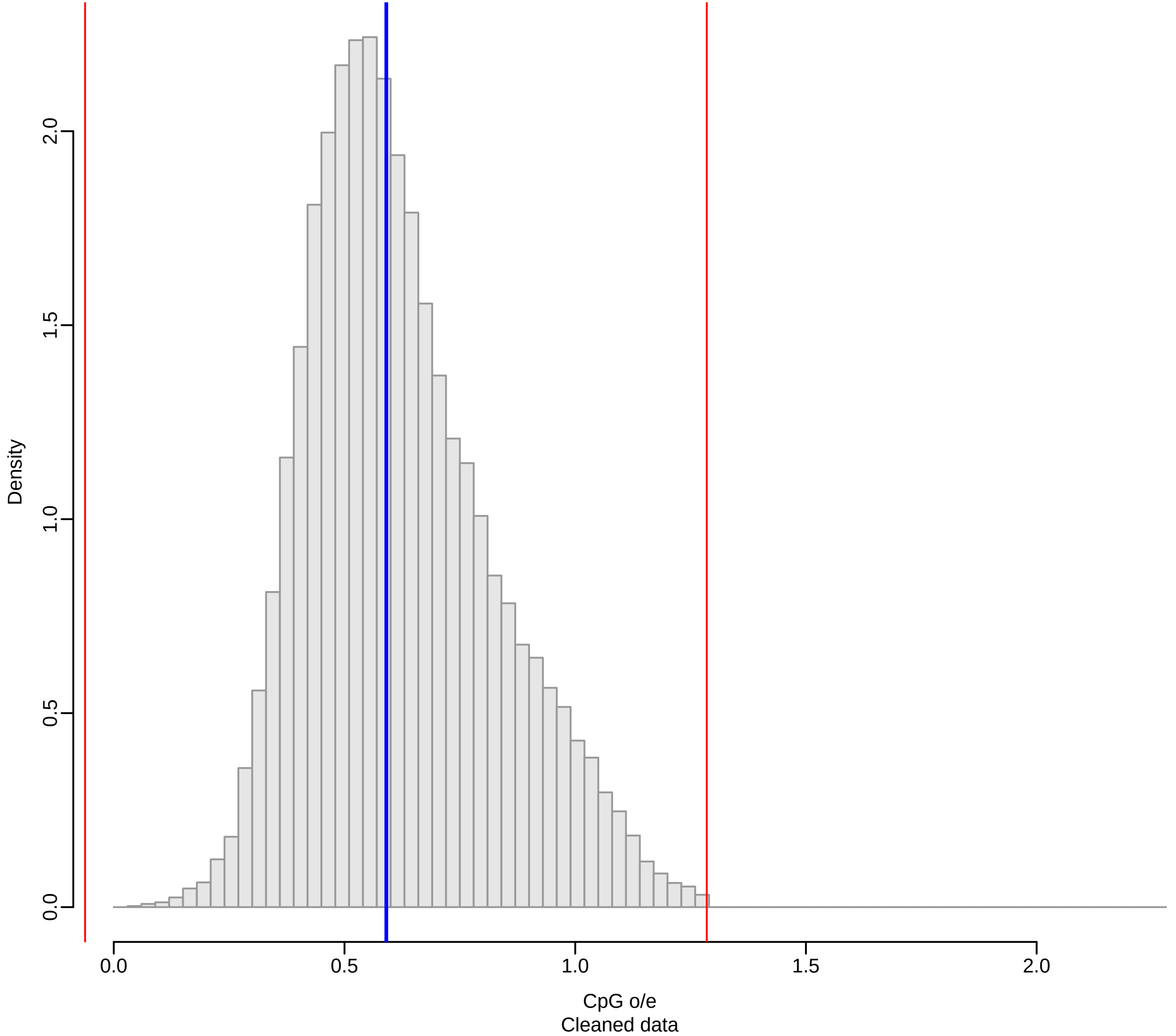

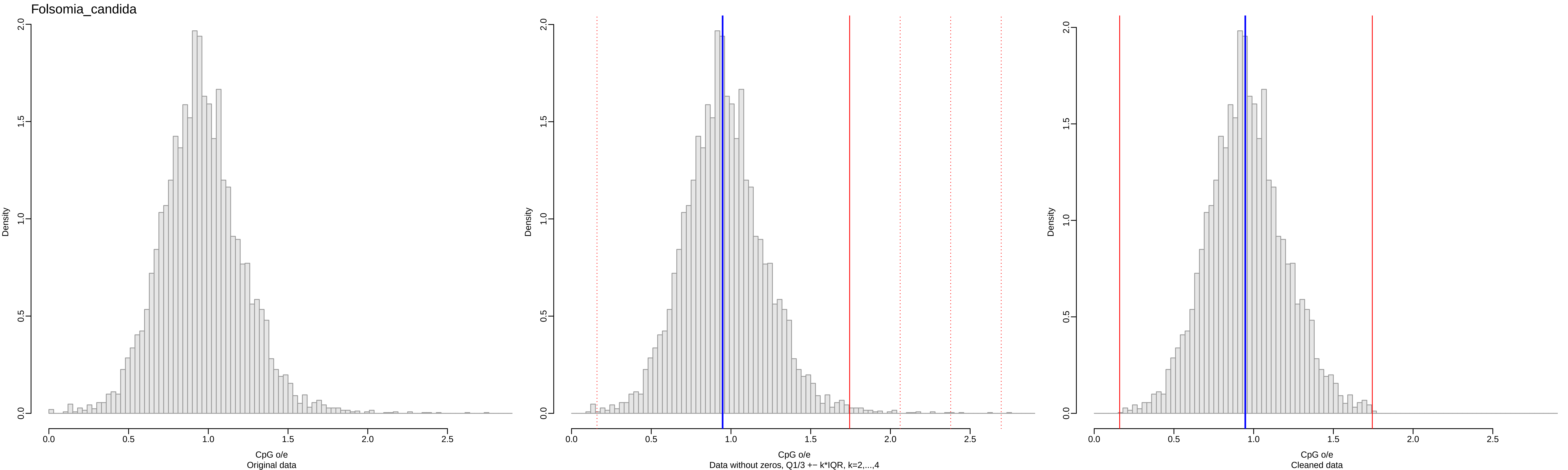

Folsomia_candida
Density
CpG o/e
Original data
Data without zeros, Q1/3 +− k*IQR, k=2,...,4
Cleaned data
0.0
0.5
1.0
1.5
2.0
2.5

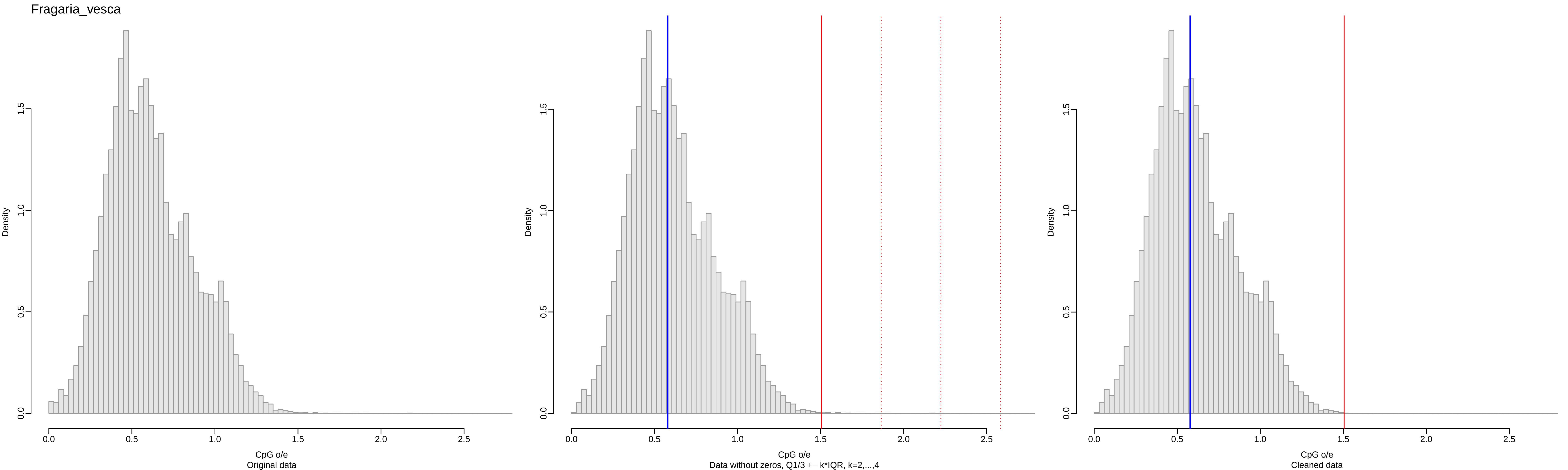
Fragaria_vesca
Density
CpG o/e
Original data
Data without zeros, Q1/3 +− k*IQR, k=2,...,4
Cleaned data
0.0
0.5
1.0
1.5
2.0
2.5

Fucus_serratus

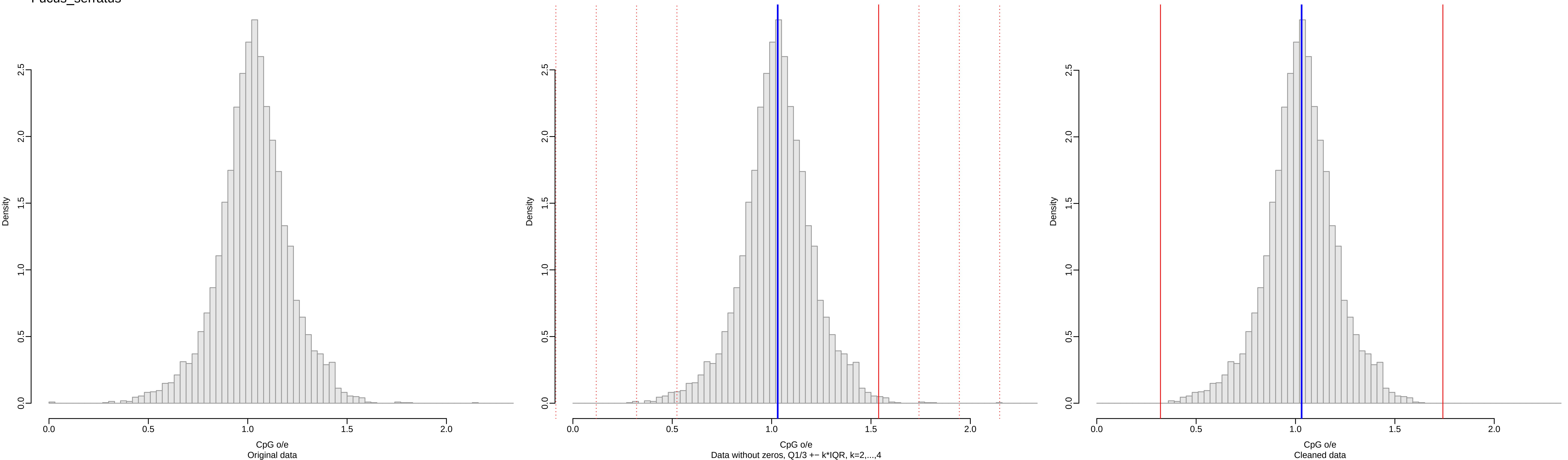

Fucus_vesiculosus

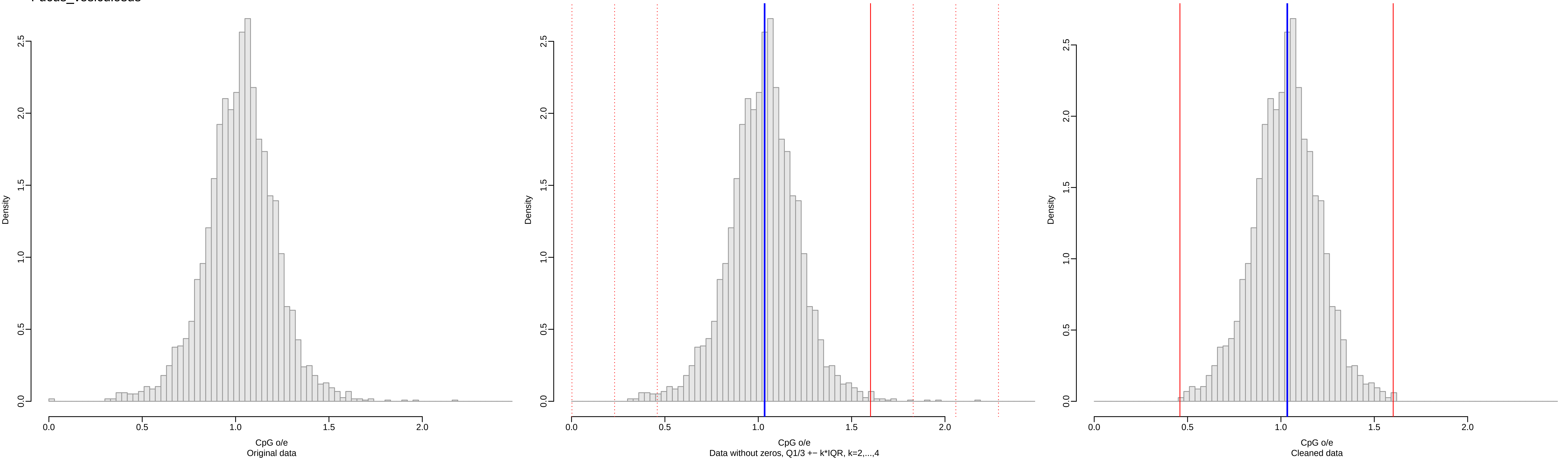

Fumaria_officinalis

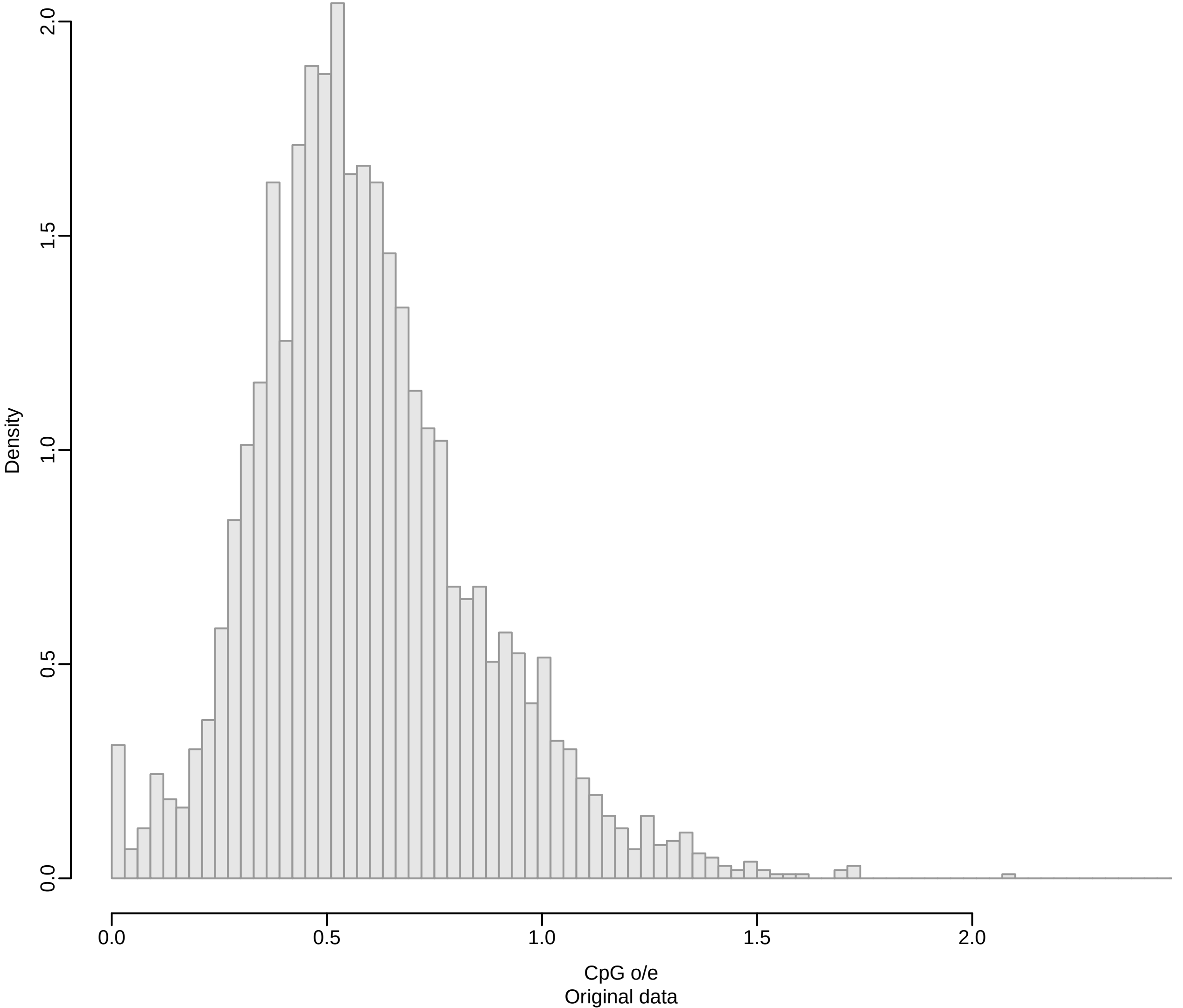

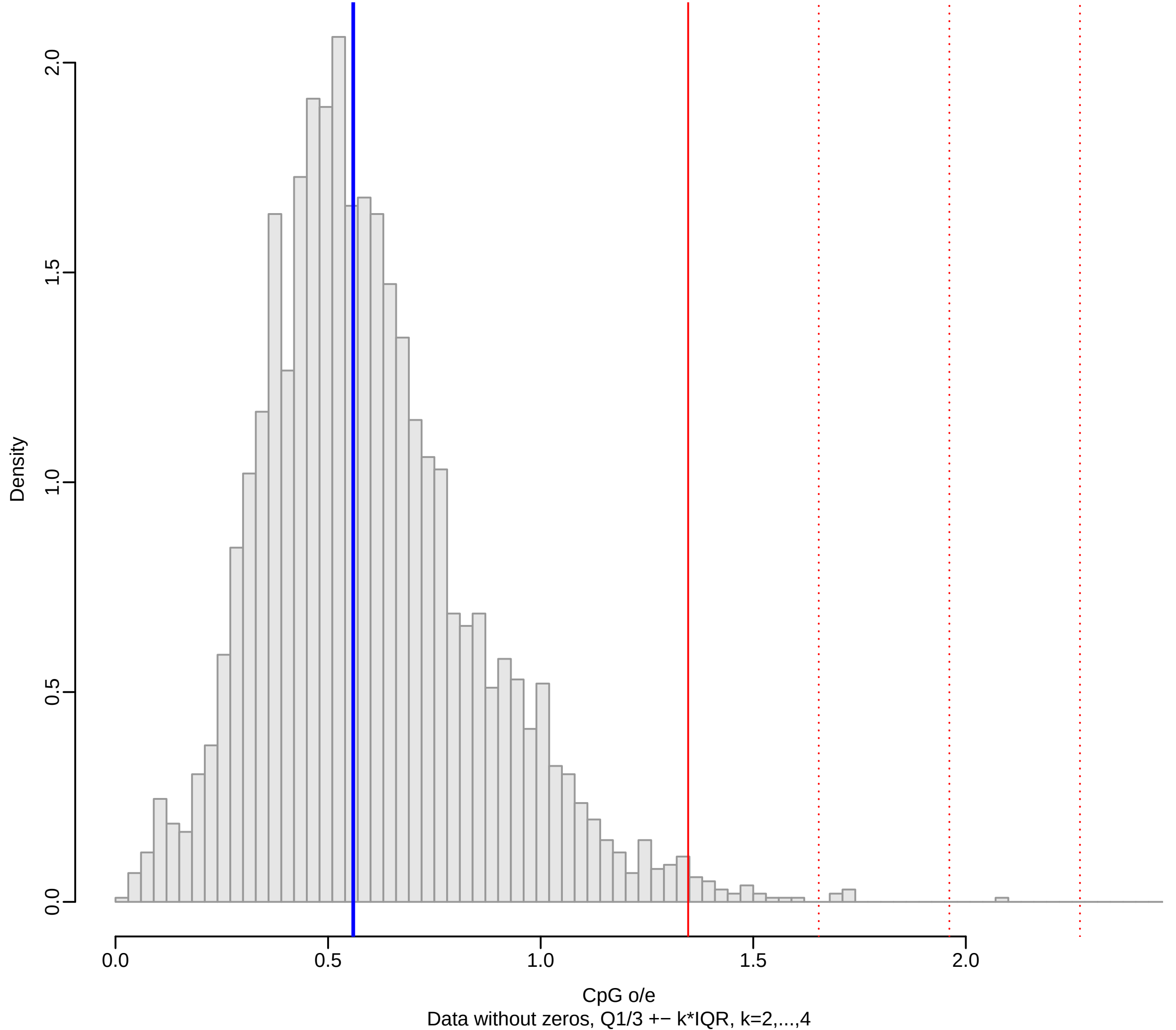

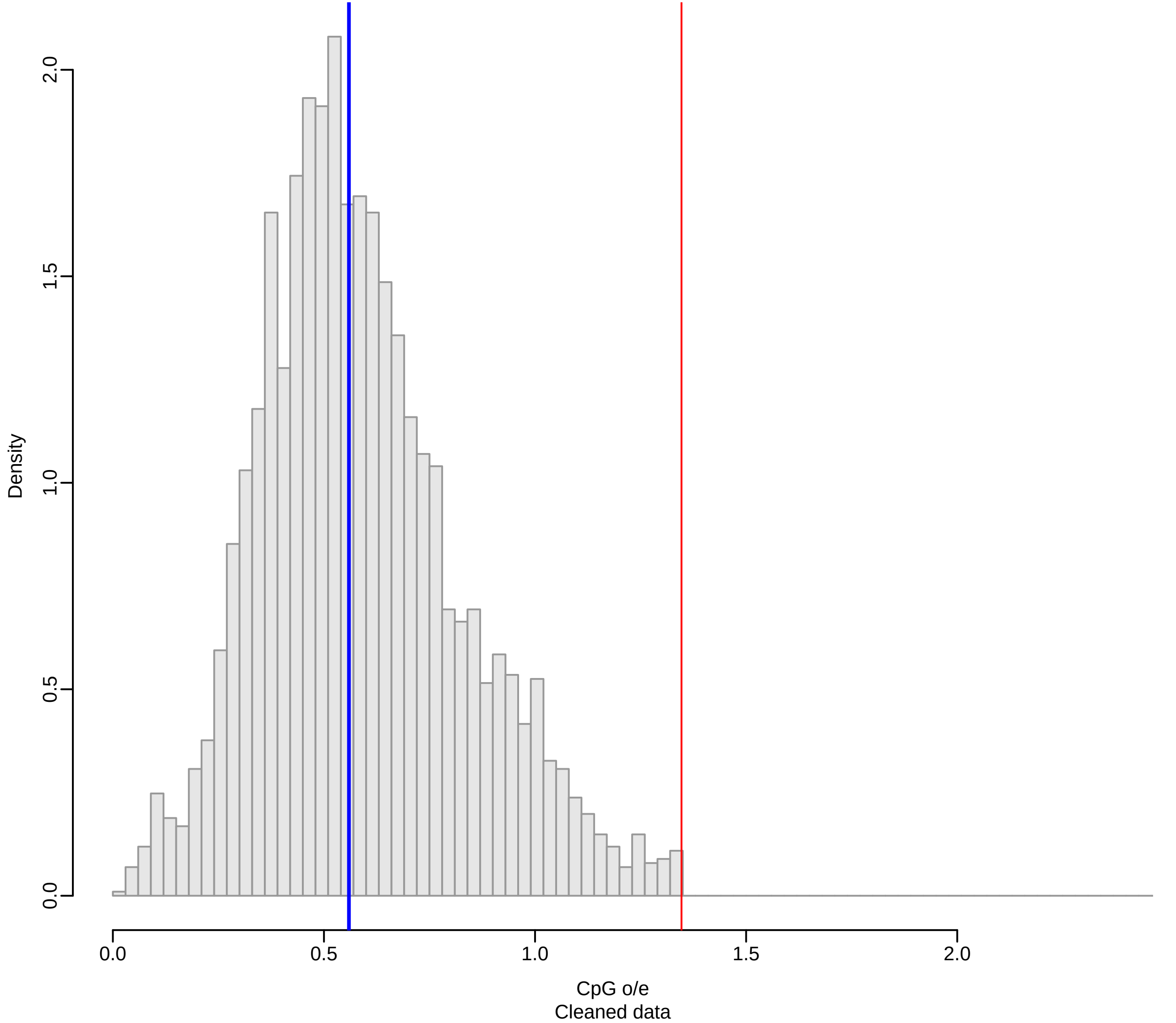

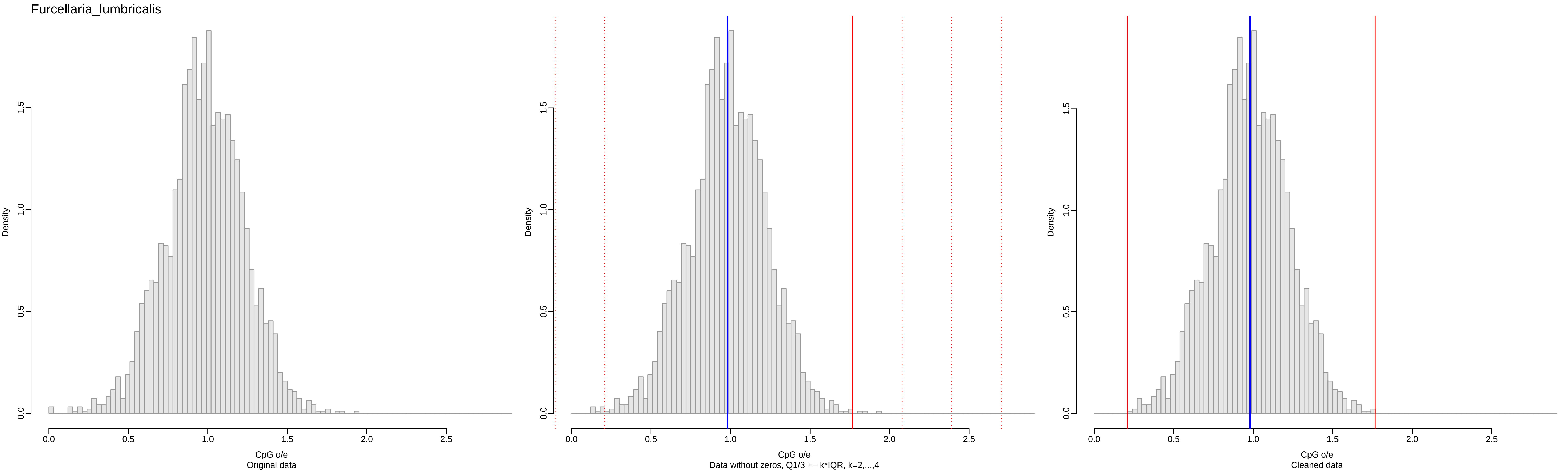

Furcellaria_lumbricalis
Density
CpG o/e
Original data
Data without zeros, Q1/3 +− k*IQR, k=2,...,4
Cleaned data
0.0
0.5
1.0
1.5
2.0
2.5

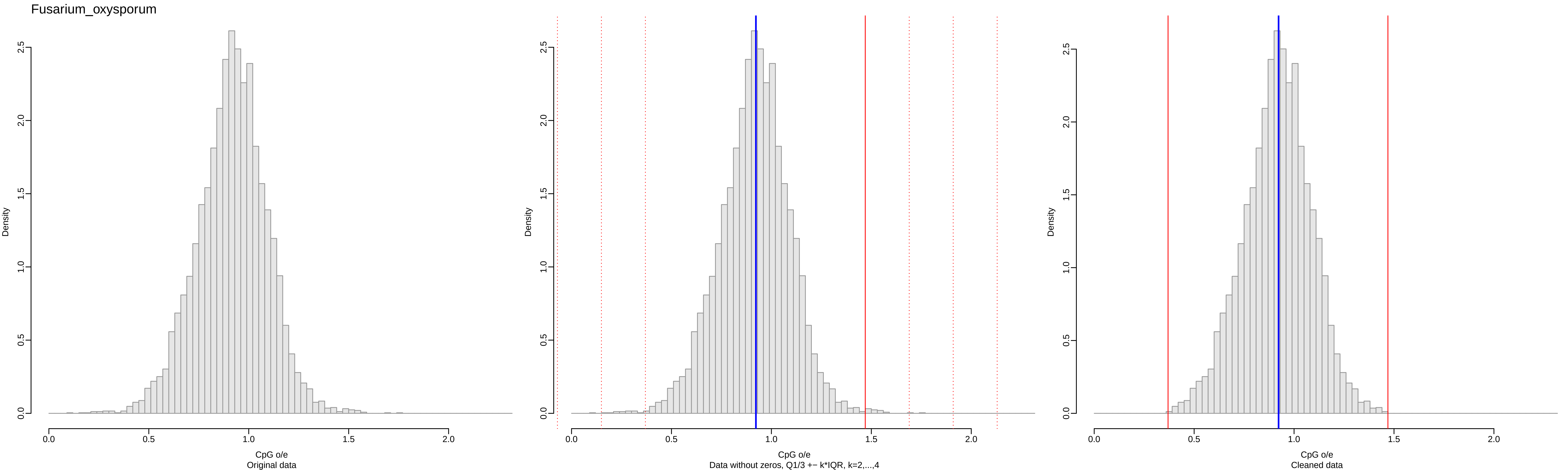

Fusarium_oxysporum
Density
CpG o/e
Original data
Data without zeros, Q1/3 +− k*IQR, k=2,...,4
Cleaned data
0.0
0.5
1.0
1.5
2.0
2.5

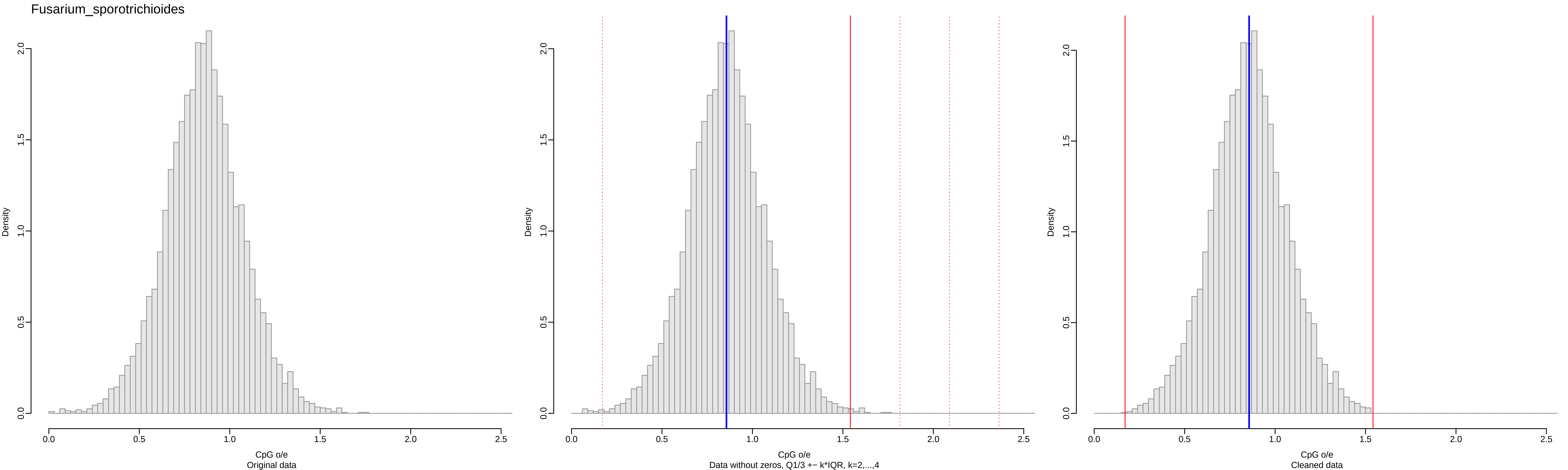

Fusarium_sporotrichioides
Density
CpG o/e
Original data
Data without zeros, Q1/3 +− k*IQR, k=2,...,4
Cleaned data
0.0
0.5
1.0
1.5
2.0
2.5

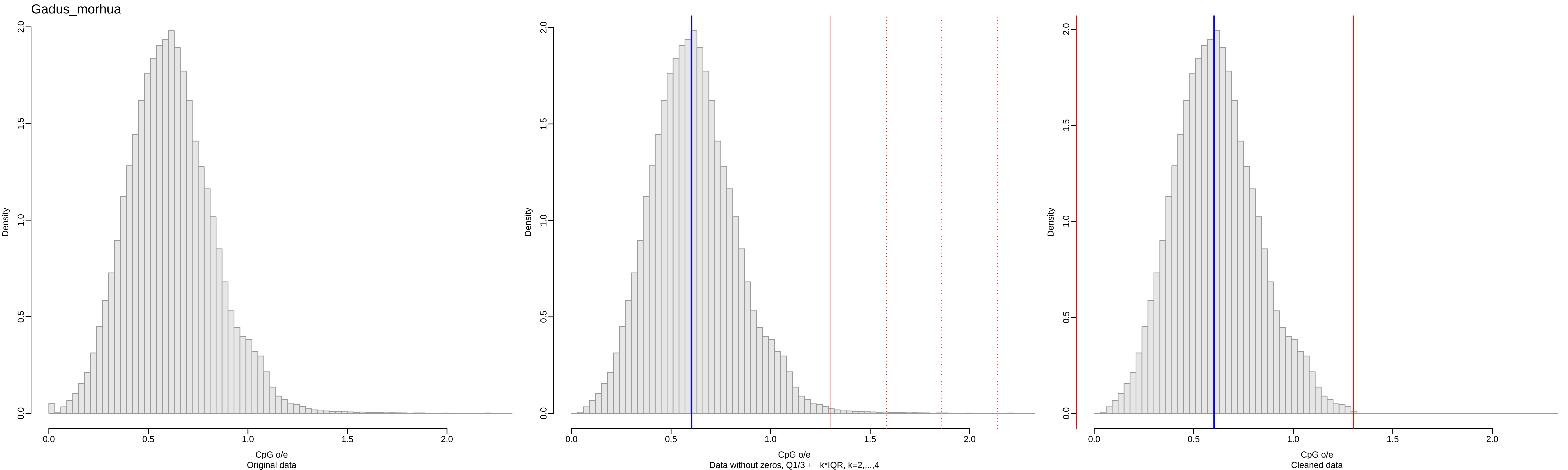

Gadus_morhua
Density
CpG o/e
Original data
Data without zeros, Q1/3 +− k*IQR, k=2,...,4
Cleaned data
0.0
0.5
1.0
1.5
2.0

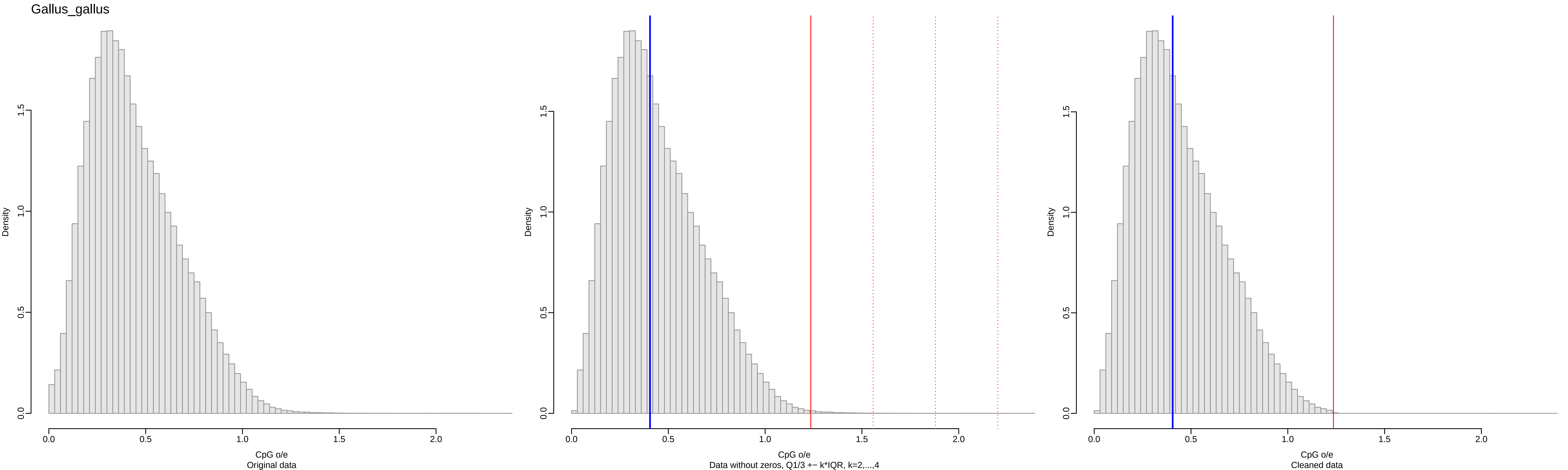

Gallus_gallus
Density
0.0
0.5
1.0
1.5
2.0
CpG o/e
Original data
Data without zeros, Q1/3 +− k*IQR, k=2,...,4
Cleaned data

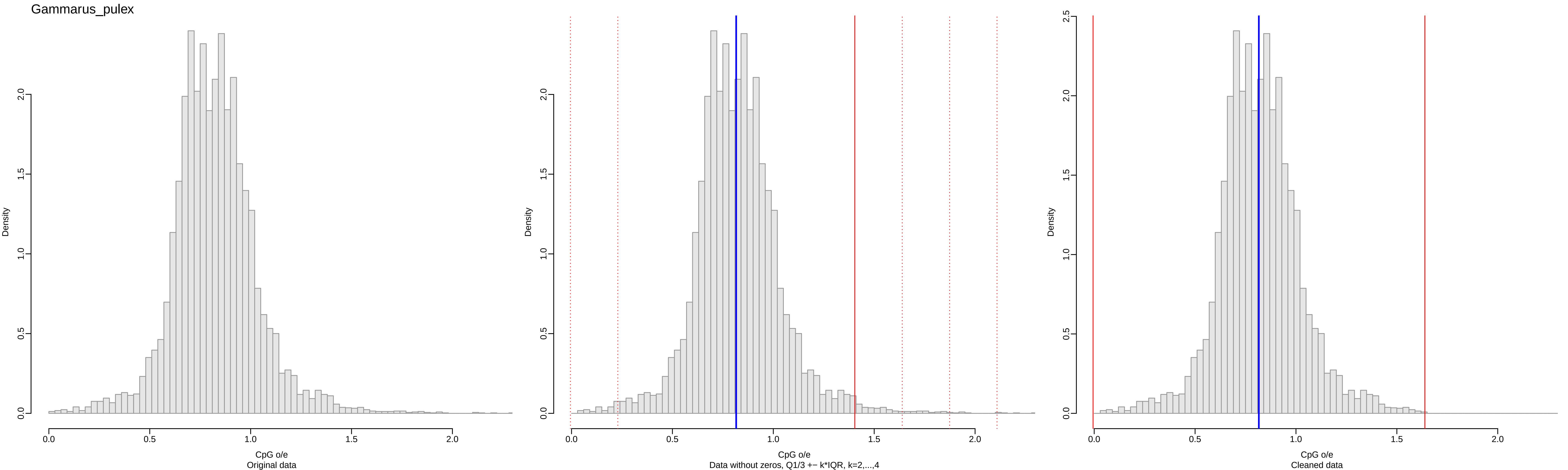

Gammarus_pulex
Density
CpG o/e
Original data
CpG o/e
Data without zeros, Q1/3 +− k*IQR, k=2,...,4
CpG o/e
Cleaned data

Gasterosteus_aculeatus

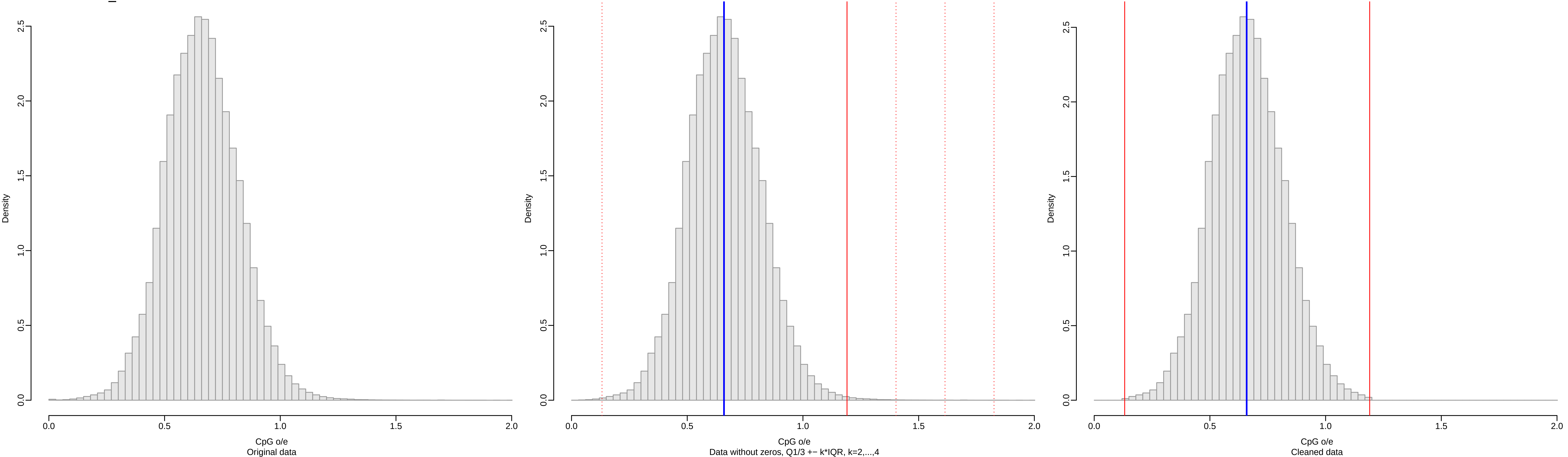

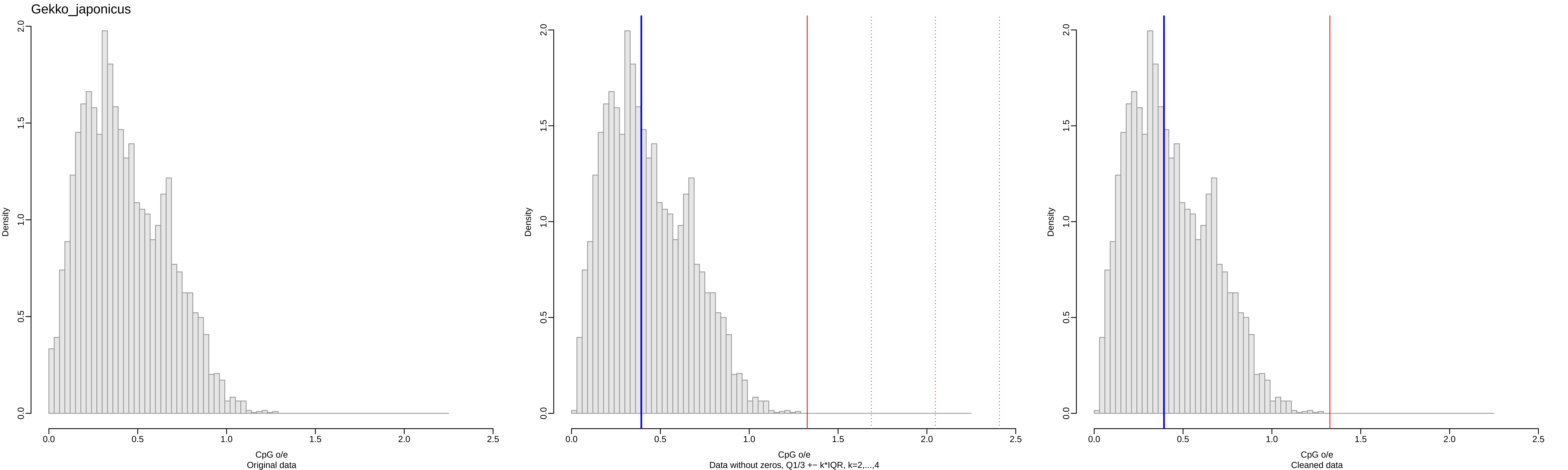

Gekko_japonicus
Density
CpG o/e
Original data
Data without zeros, Q1/3 +− k*IQR, k=2,...,4
Cleaned data
0.0
0.5
1.0
1.5
2.0
2.5

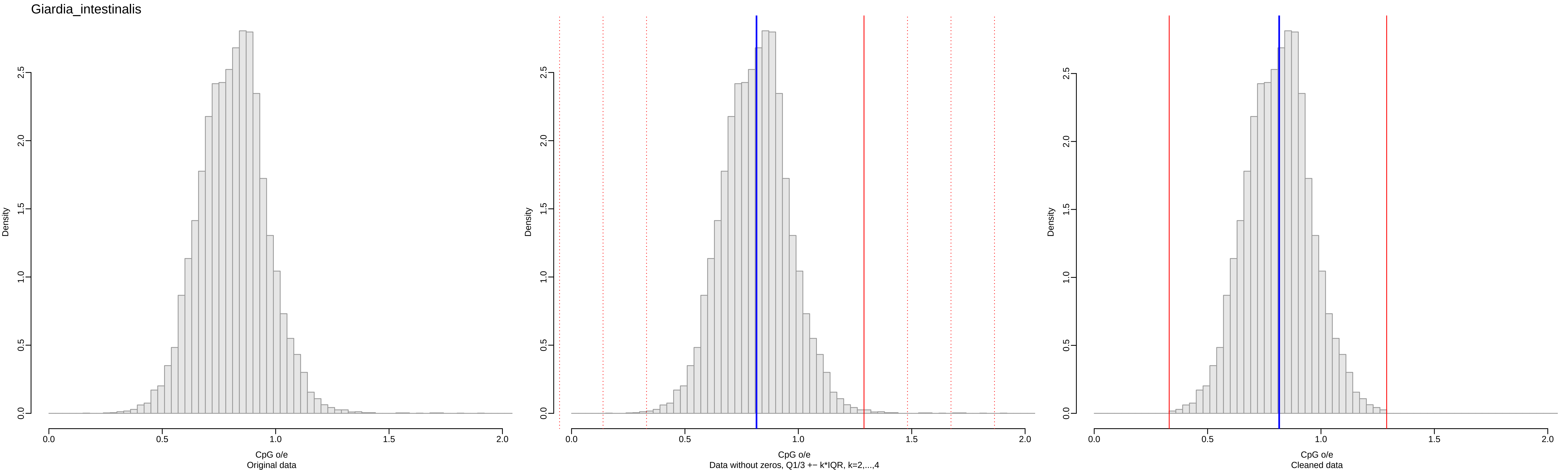
Giardia_intestinalis
Density
CpG o/e
Original data
CpG o/e
Data without zeros, Q1/3 +− k*IQR, k=2,...,4
CpG o/e
Cleaned data

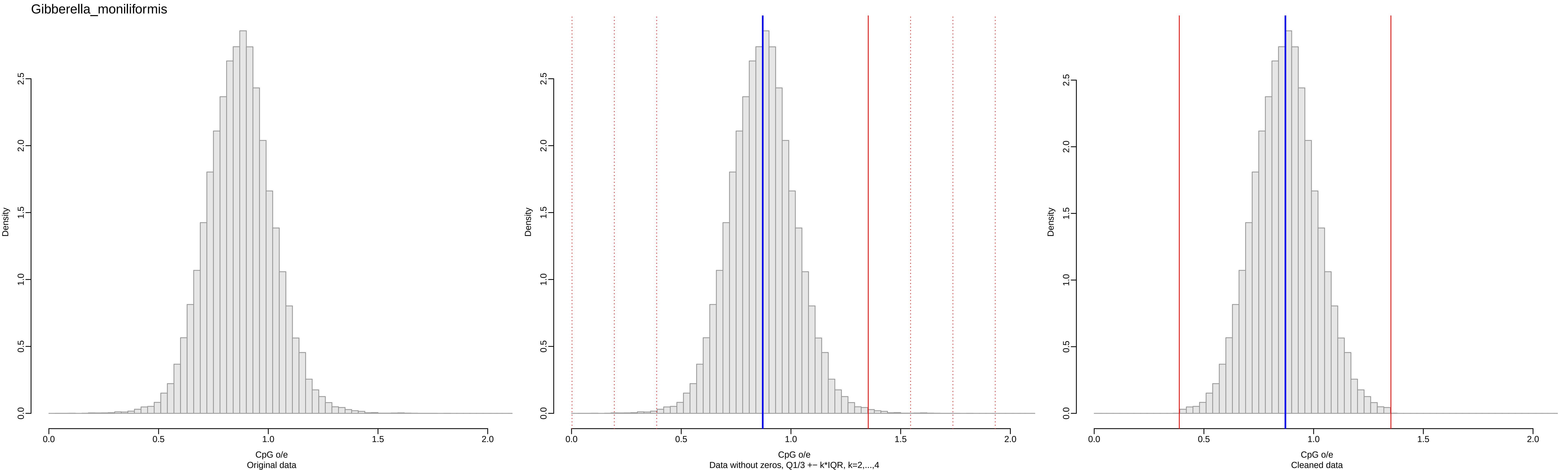
Gibberella_moniliformis
Density
CpG o/e
Original data
Data without zeros, Q1/3 +− k*IQR, k=2,...,4
Cleaned data
0.0
0.5
1.0
1.5
2.0
2.5

Gibberella_zeae

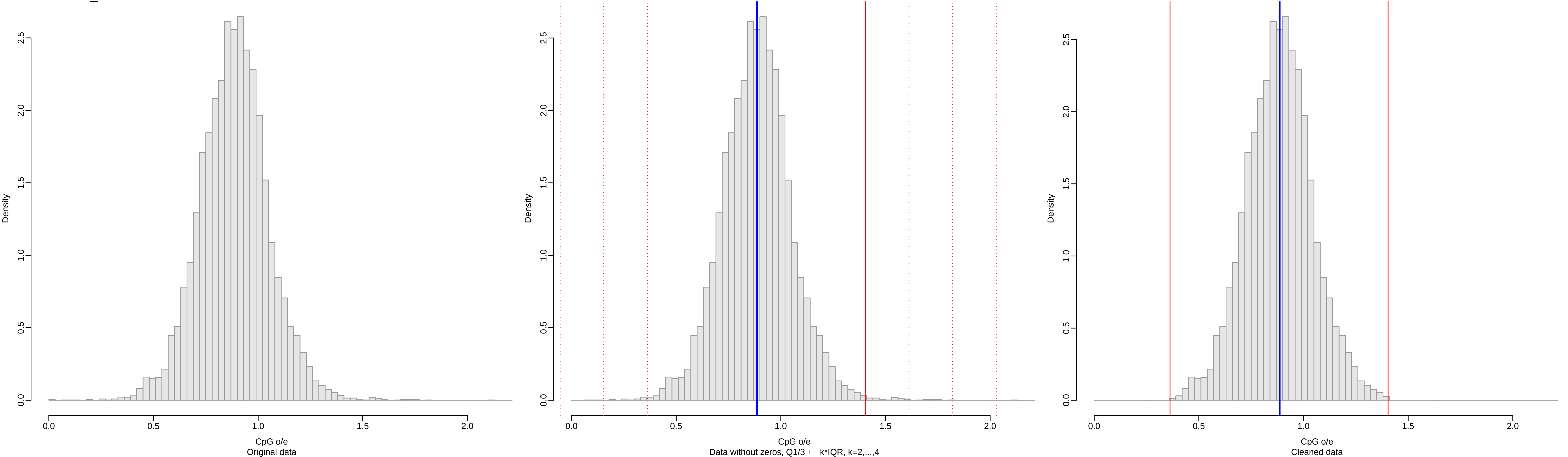

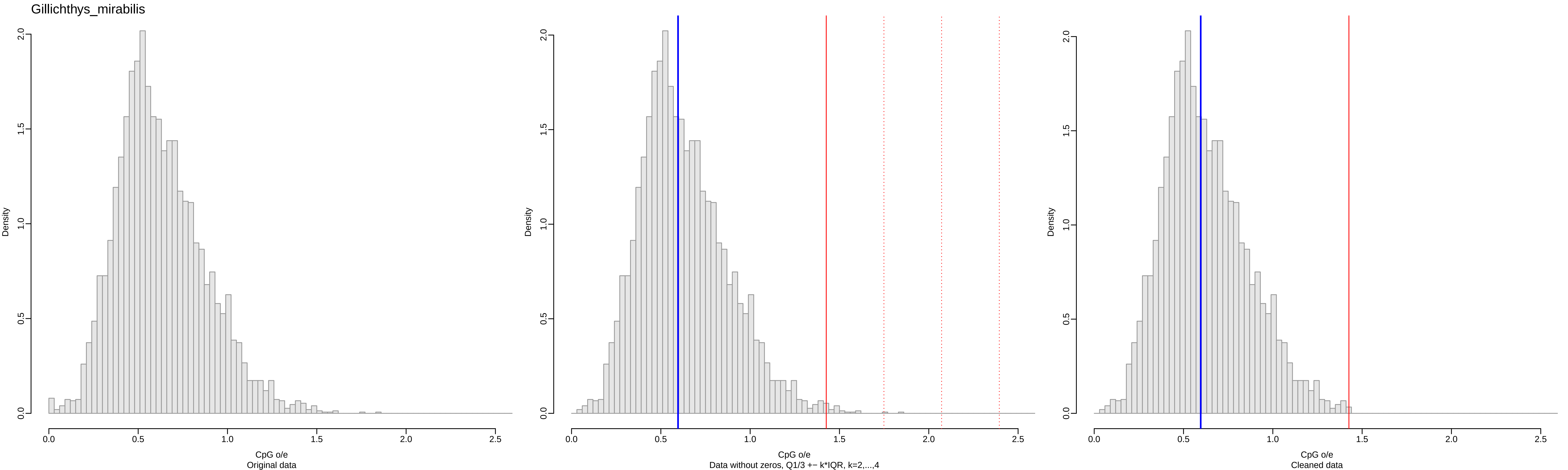

Gillichthys_mirabilis
Density
CpG o/e
Original data
Data without zeros, Q1/3 +− k*IQR, k=2,...,4
Cleaned data

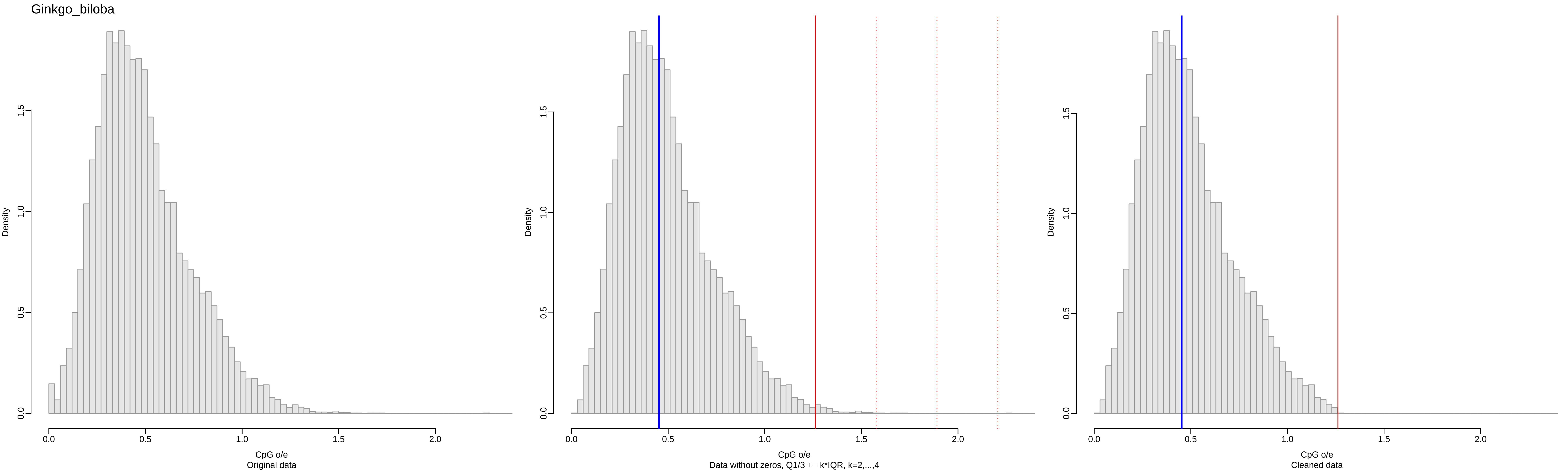

Ginkgo_biloba
Density
CpG o/e
Original data
Density
CpG o/e
Data without zeros, Q1/3 +− k*IQR, k=2,...,4
Density
CpG o/e
Cleaned data

Glaucocystis_nostochinearum

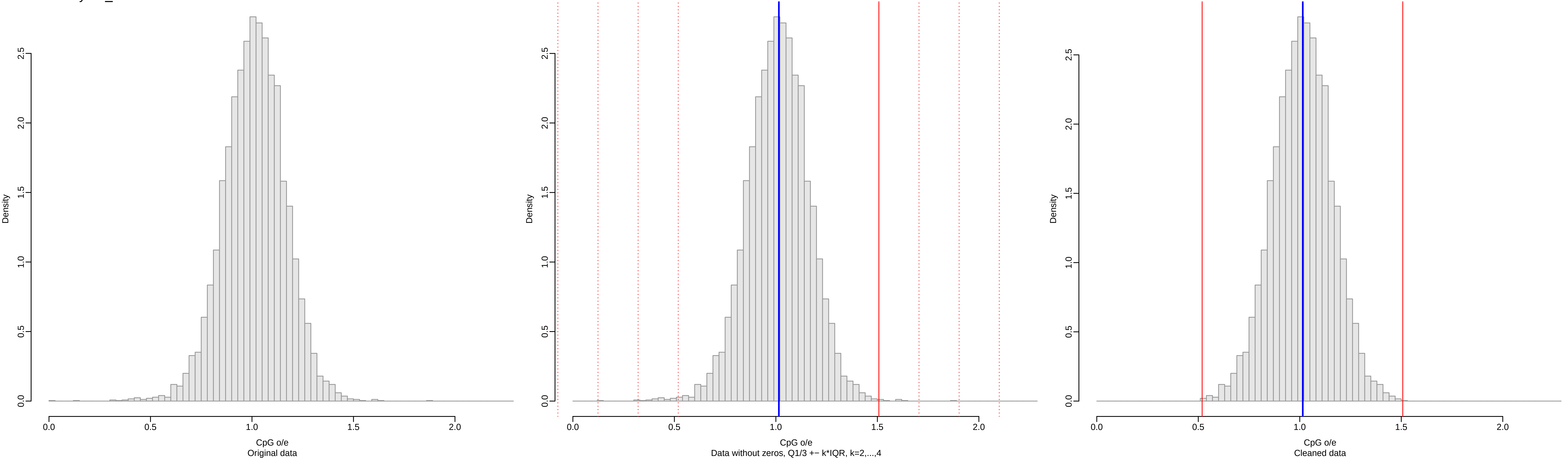

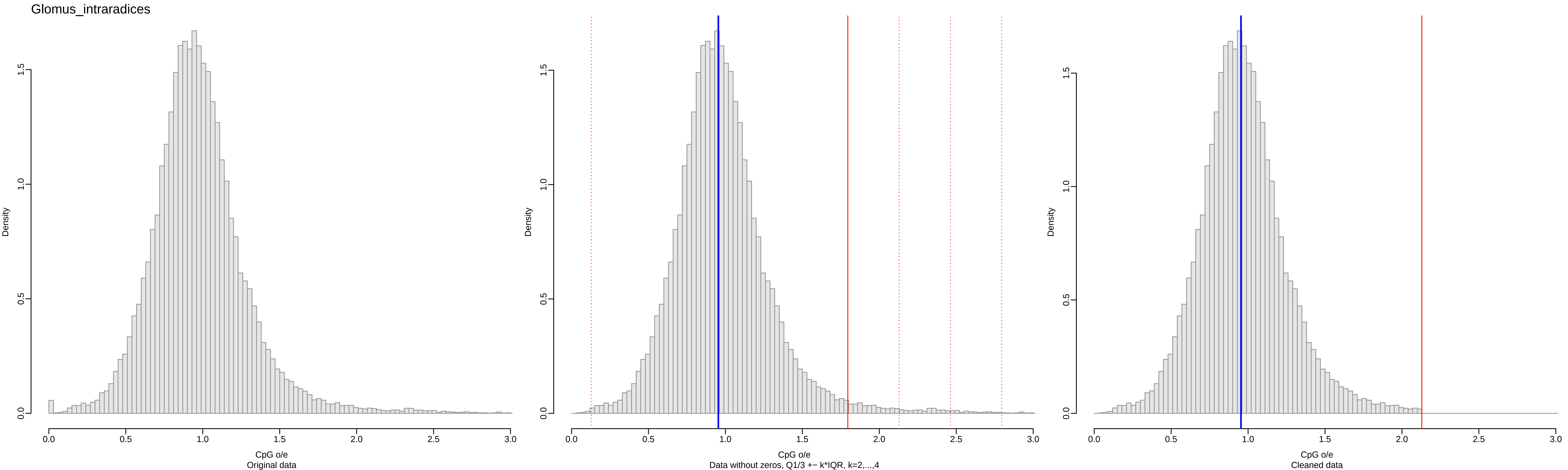
Glomus_intraradices
Density
CpG o/e
Original data
Density
CpG o/e
Data without zeros, Q1/3 +− k*IQR, k=2,...,4
Density
CpG o/e
Cleaned data
0.0
0.5
1.0
1.5
2.0
2.5
3.0

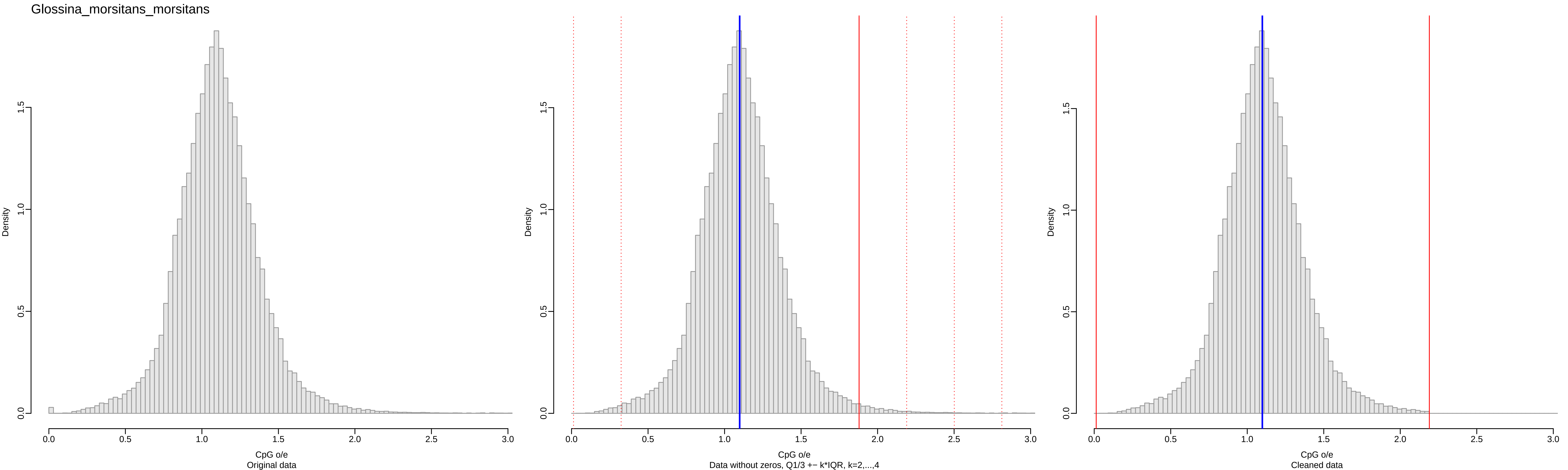
Glossina_morsitans_morsitans
Density
0.0
0.5
1.0
1.5
2.0
2.5
3.0
CpG o/e
Original data
CpG o/e
Data without zeros, Q1/3 +− k*IQR, k=2,...,4
CpG o/e
Cleaned data

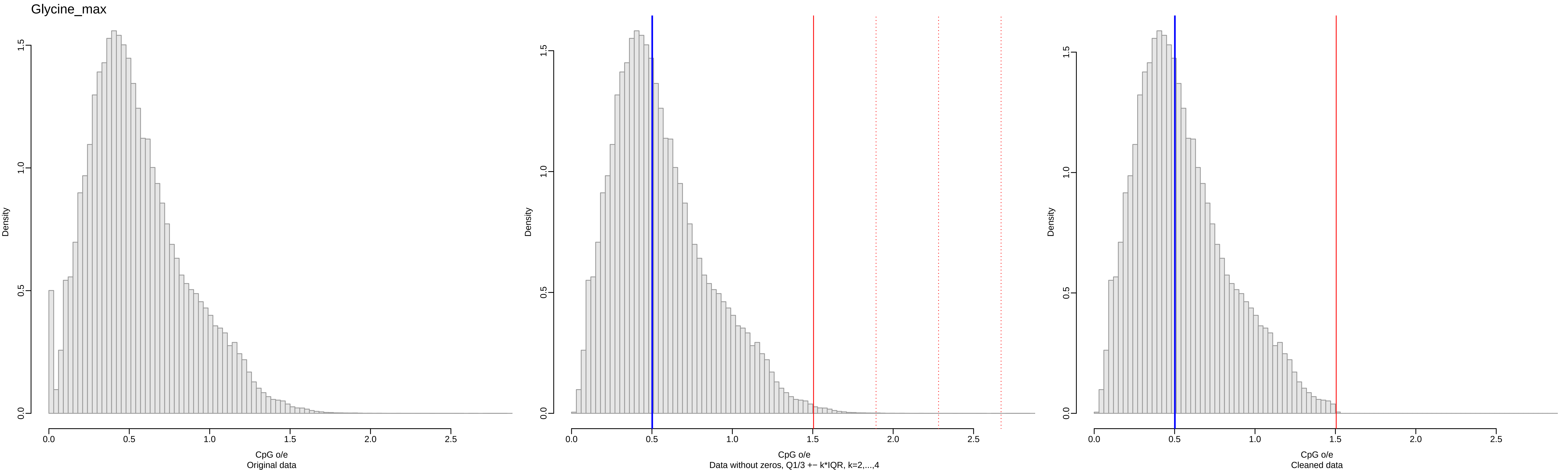

Glycine_max
Density
CpG o/e
Original data
Data without zeros, Q1/3 +− k*IQR, k=2,...,4
Cleaned data
0.0
0.5
1.0
1.5
2.0
2.5

Gnetum_gnemon

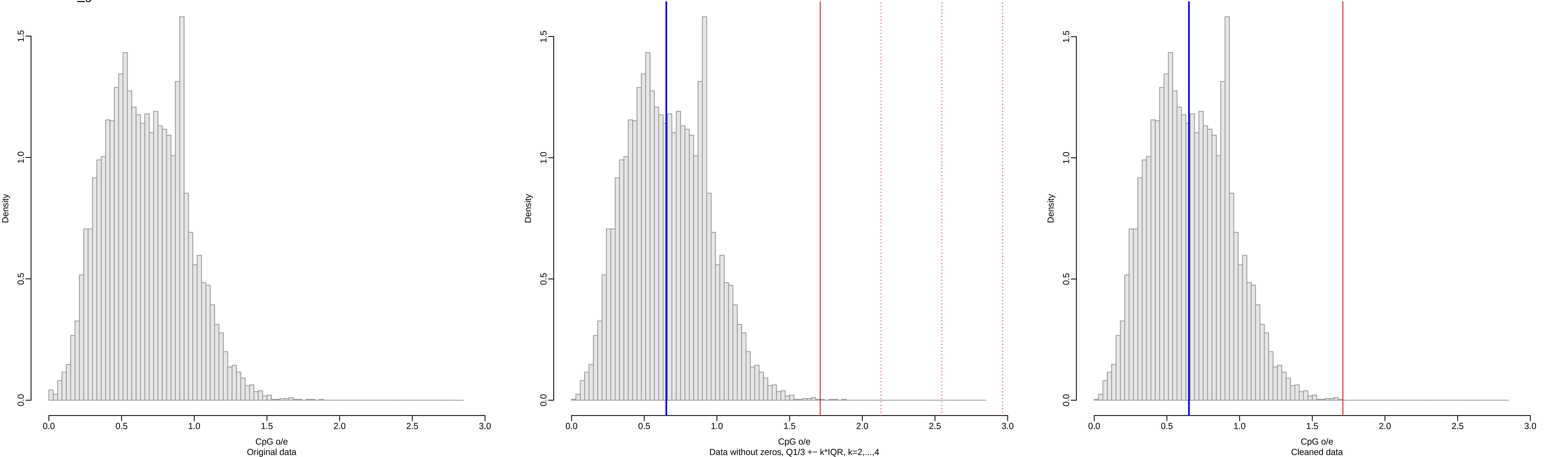

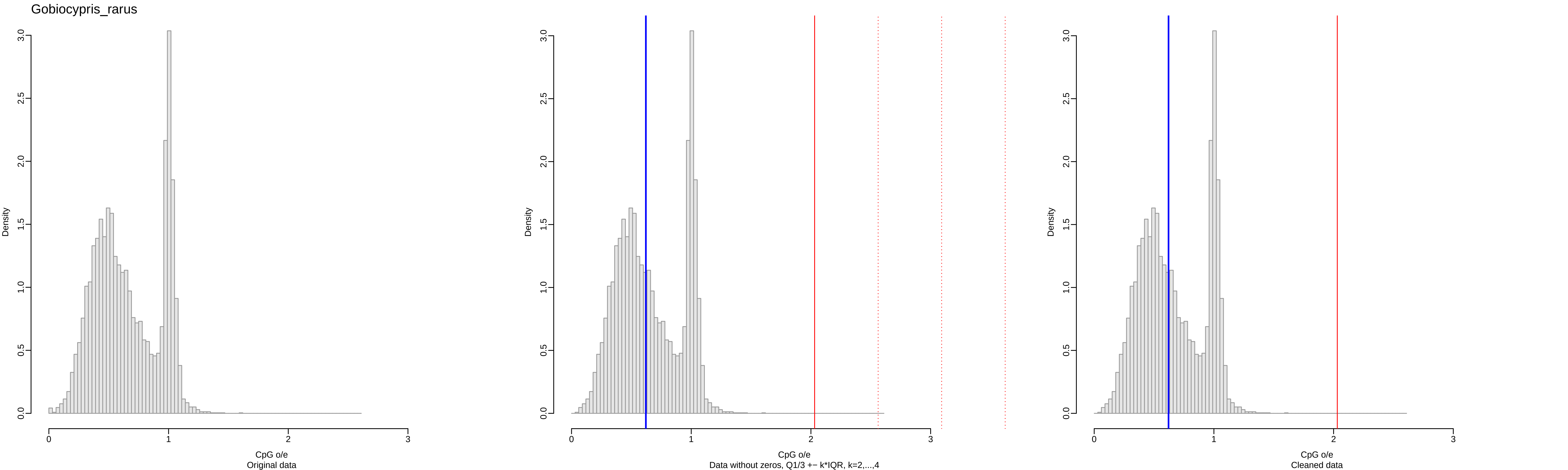
Gobiocypris_rarus
Density
CpG o/e
Original data
Data without zeros, Q1/3 +− k*IQR, k=2,...,4
Cleaned data
0.0
0.5
1.0
1.5
2.0
2.5
3.0
0
1
2
3

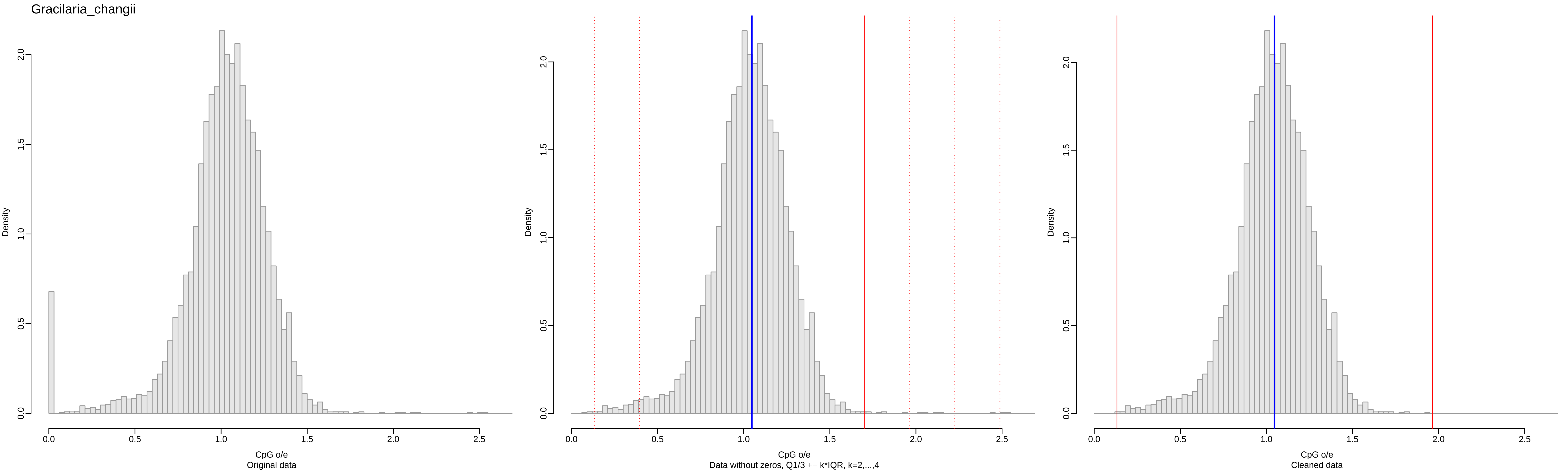

Gracilaria_changii
Density
CpG o/e
Original data
Density
CpG o/e
Data without zeros, Q1/3 +− k*IQR, k=2,...,4
Density
CpG o/e
Cleaned data

Haematobia_irritans_irritans

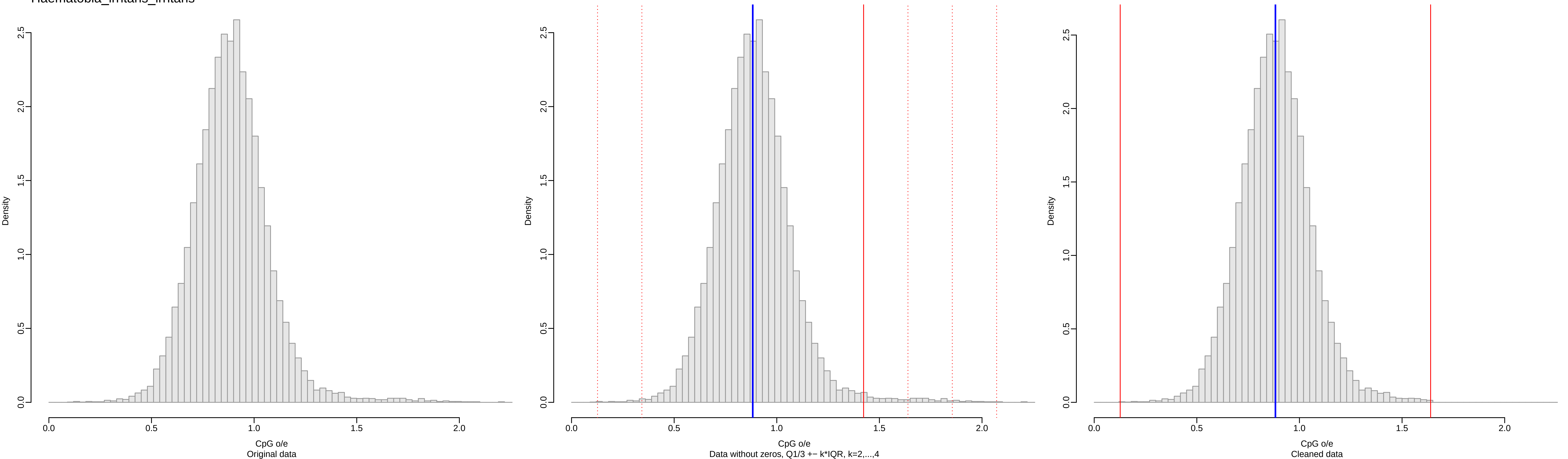

Haemonchus_contortus

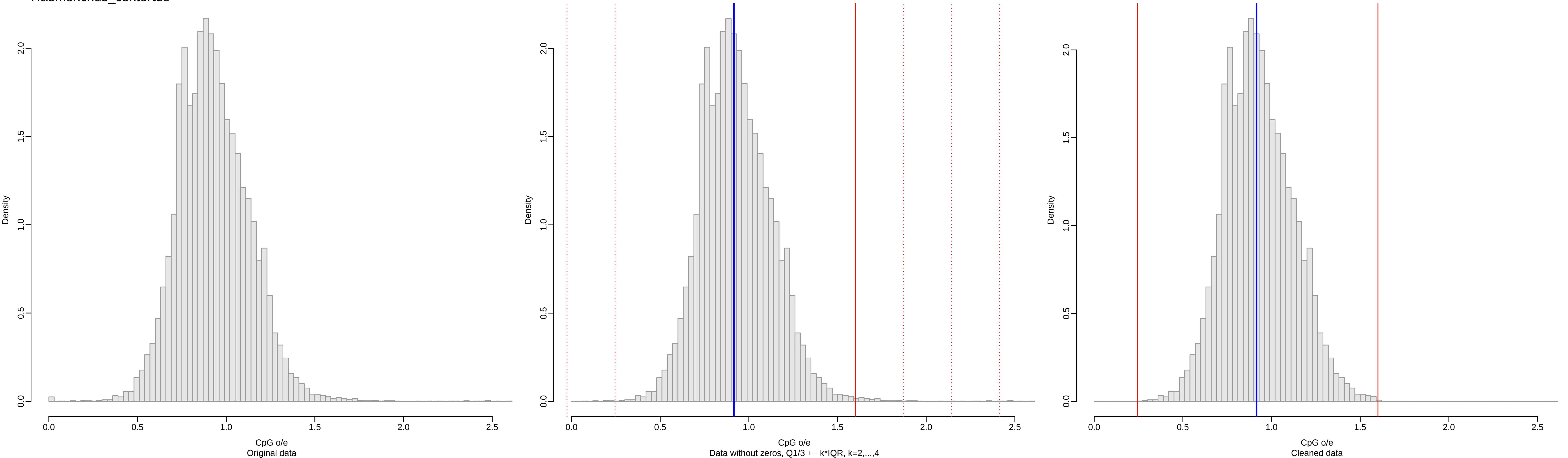

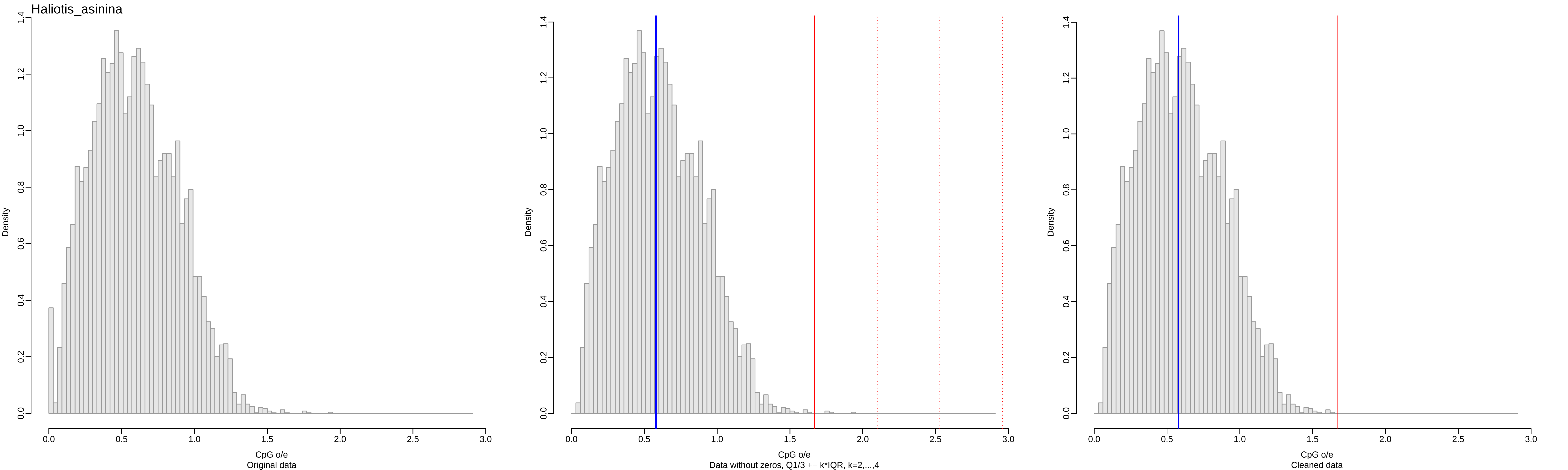
Haliotis_asinina
Density
CpG o/e
Original data
Data without zeros, Q1/3 +− k*IQR, k=2,...,4
Cleaned data
0.0
0.2
0.4
0.6
0.8
1.0
1.2
1.4
0.5
1.5
2.0
2.5
3.0

Haliotis_discus

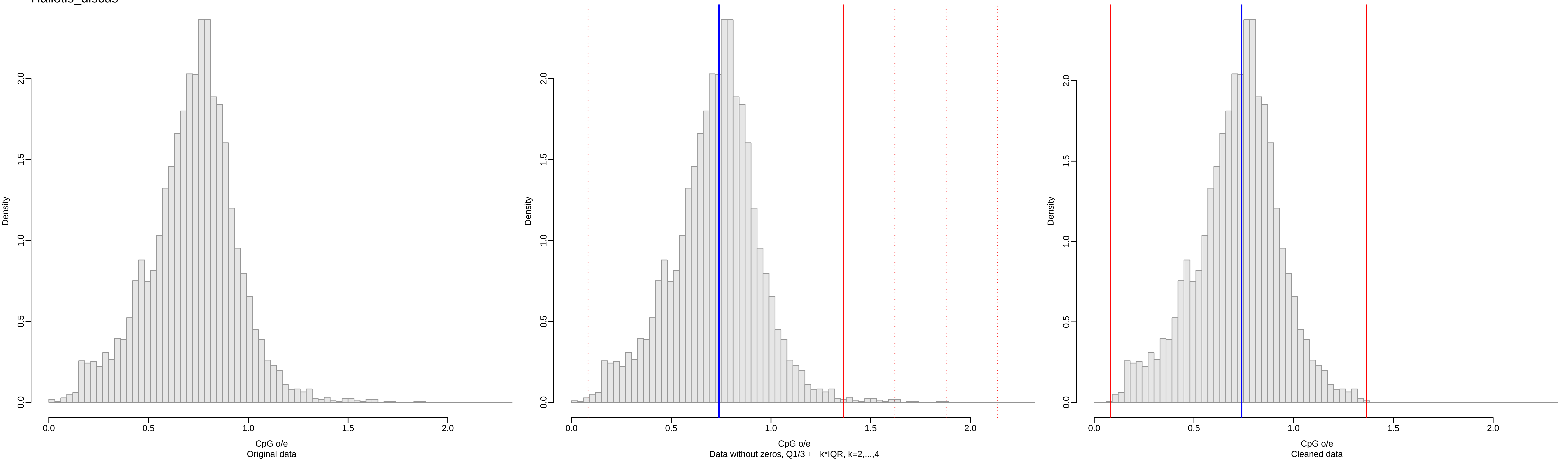

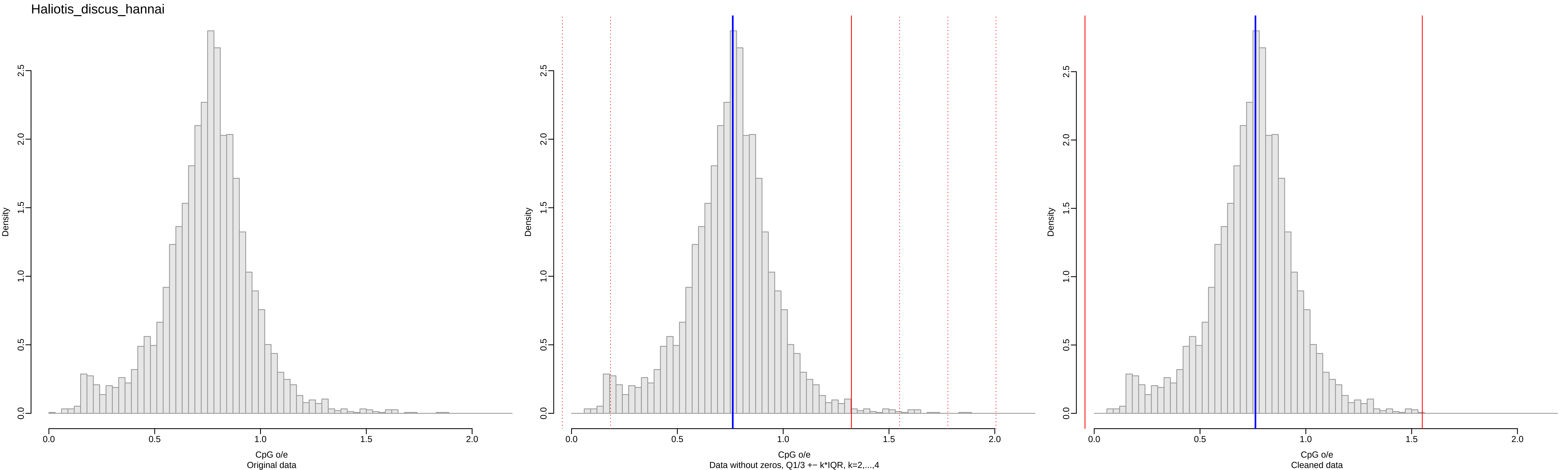

Haliotis_discus_hannai
Density
CpG o/e
Original data
CpG o/e
Data without zeros, Q1/3 +− k*IQR, k=2,...,4
CpG o/e
Cleaned data
0.0
0.5
1.0
1.5
2.0
2.5

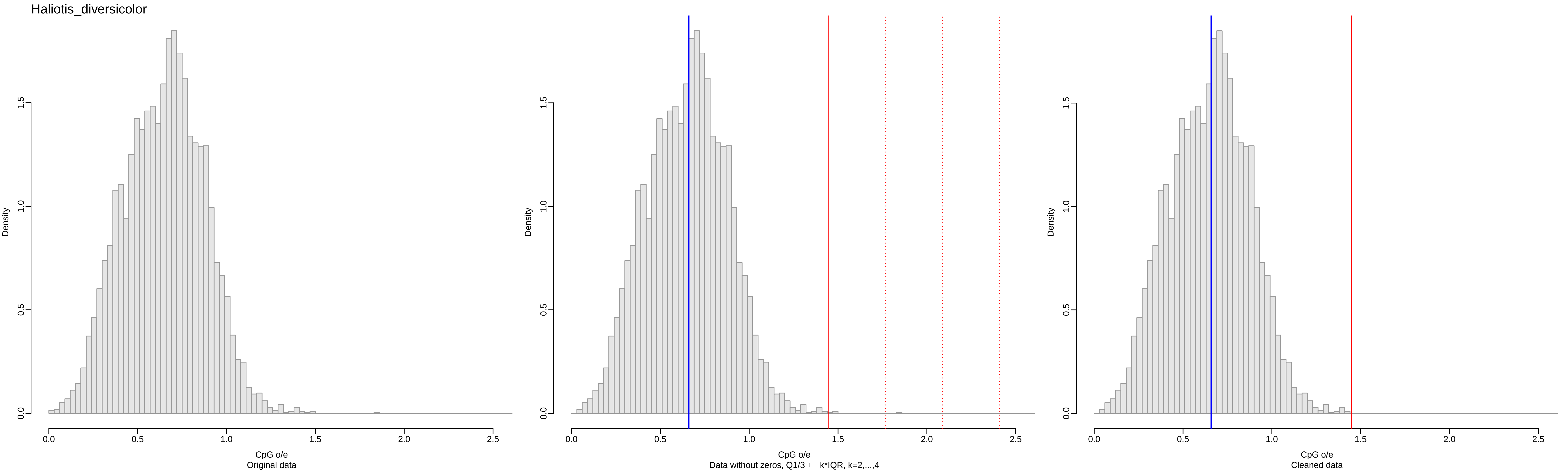
Haliotis_diversicolor
Density
CpG o/e
Original data
Data without zeros, Q1/3 +− k*IQR, k=2,...,4
Cleaned data
0.0
0.5
1.0
1.5
2.0
2.5

Halocynthia_roretzi

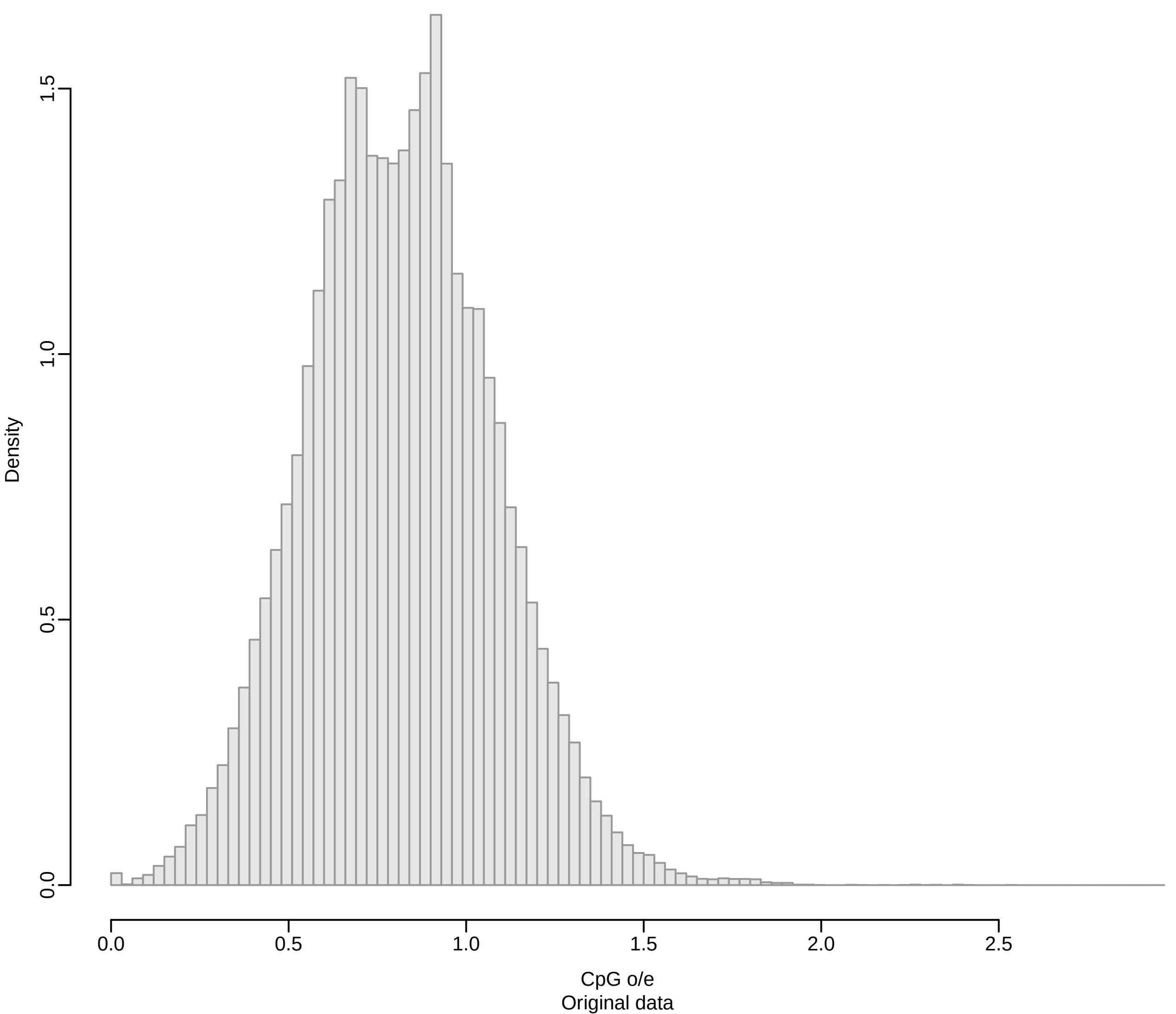

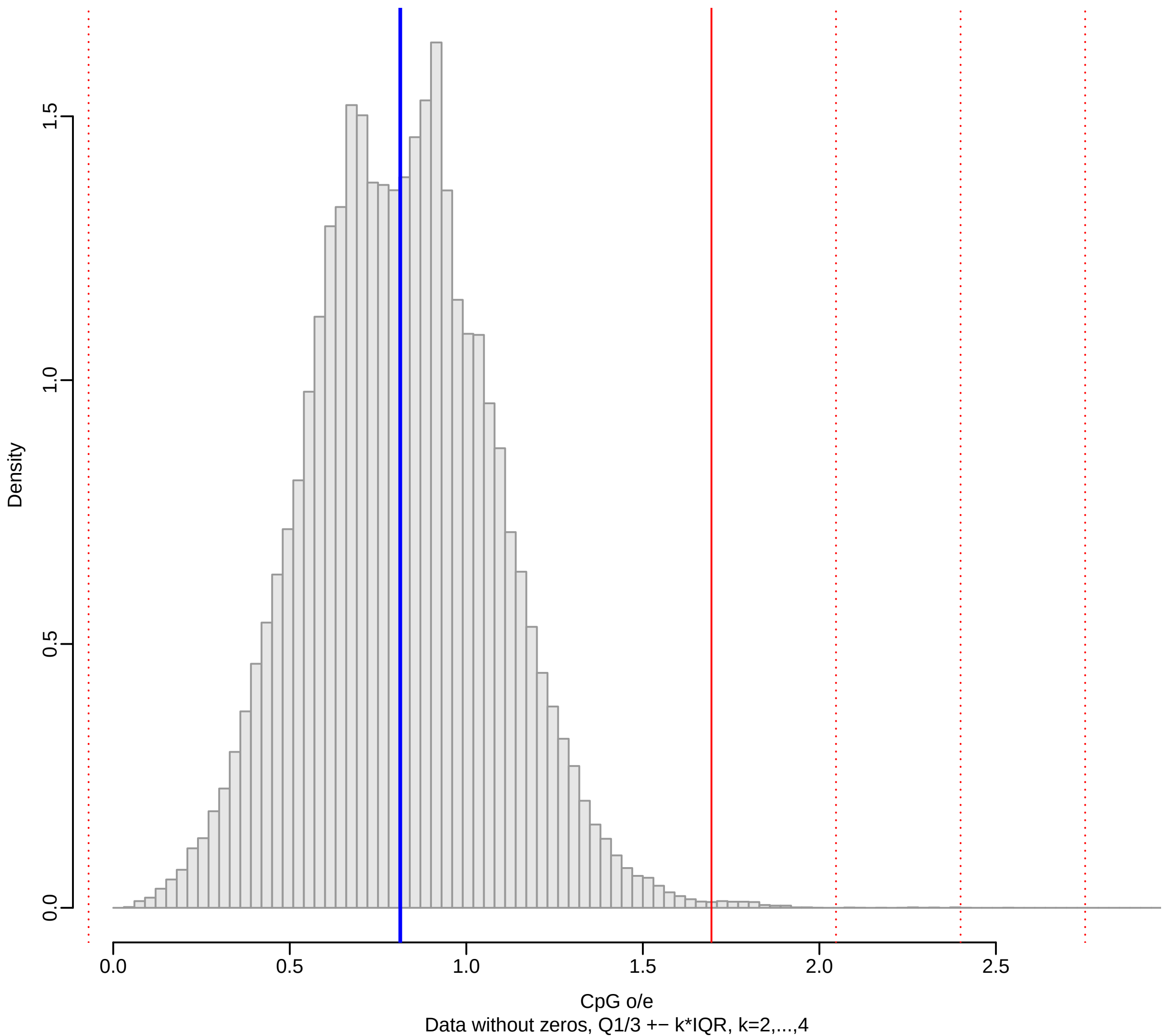

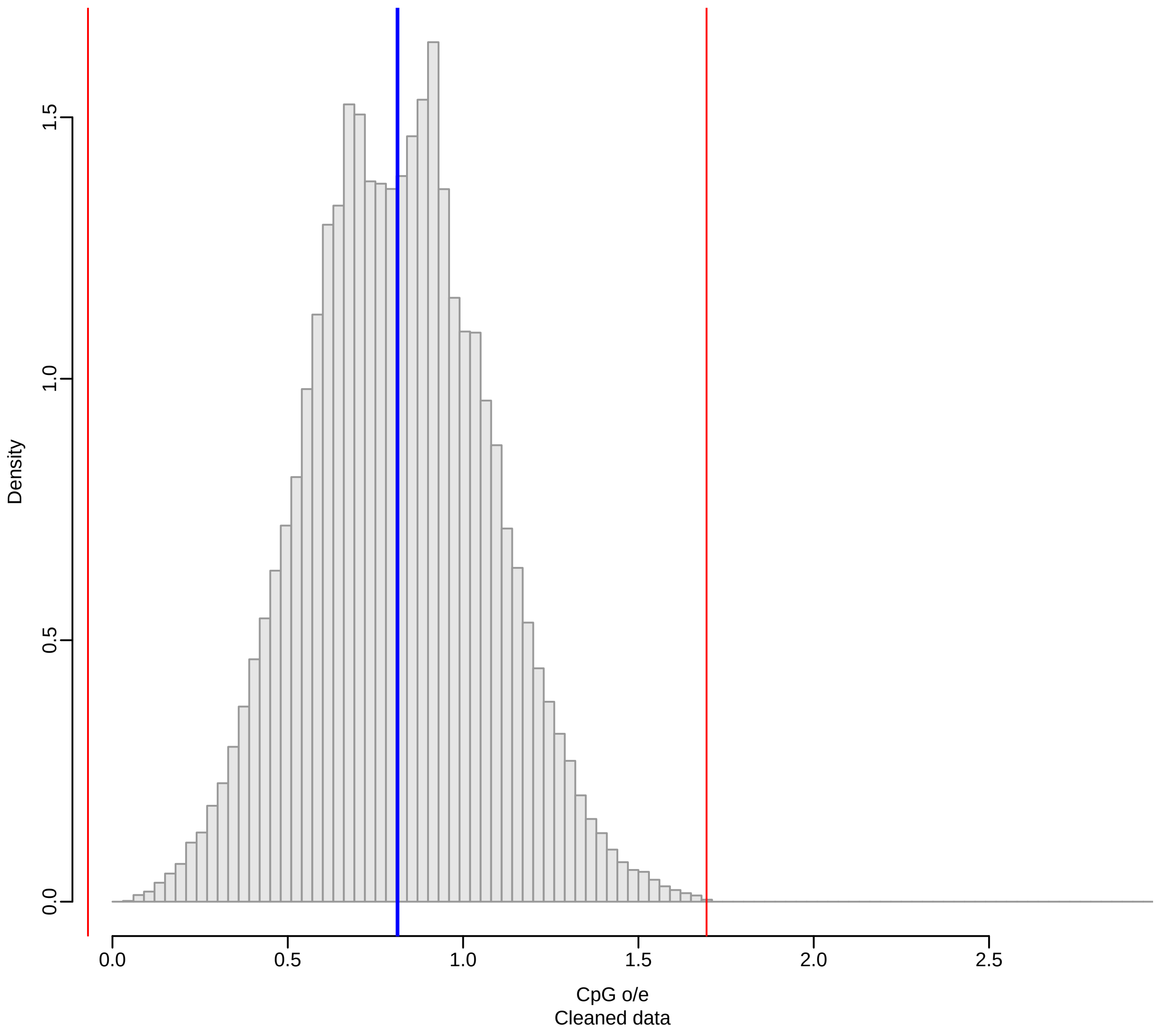

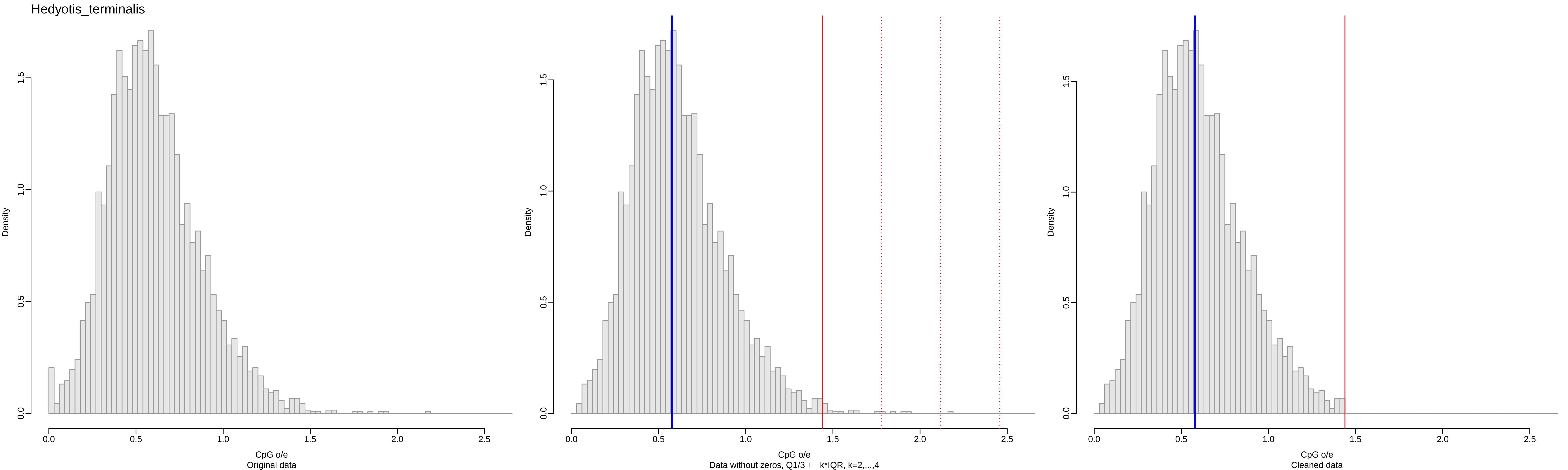

Hedyotis_terminalis
Density
CpG o/e
Original data
Data without zeros, Q1/3 +− k*IQR, k=2,...,4
Cleaned data
0.0
0.5
1.0
1.5
2.0
2.5

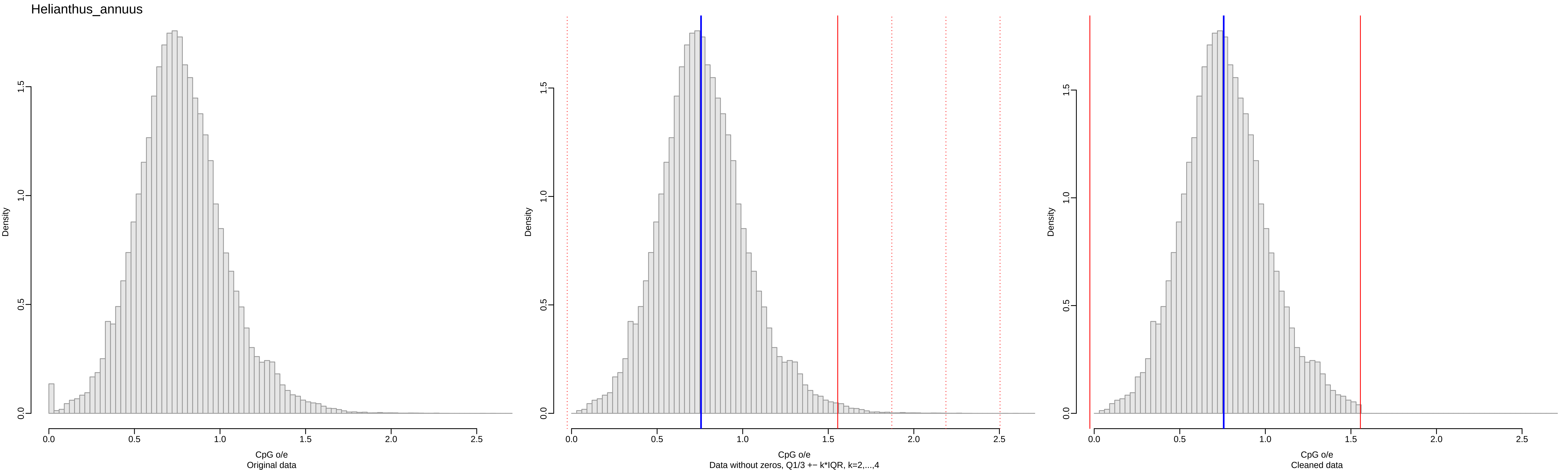

Helianthus_annuus
Density
0.0
0.5
1.0
1.5
2.0
2.5
CpG o/e
Original data
Data without zeros, Q1/3 +− k*IQR, k=2,...,4
Cleaned data

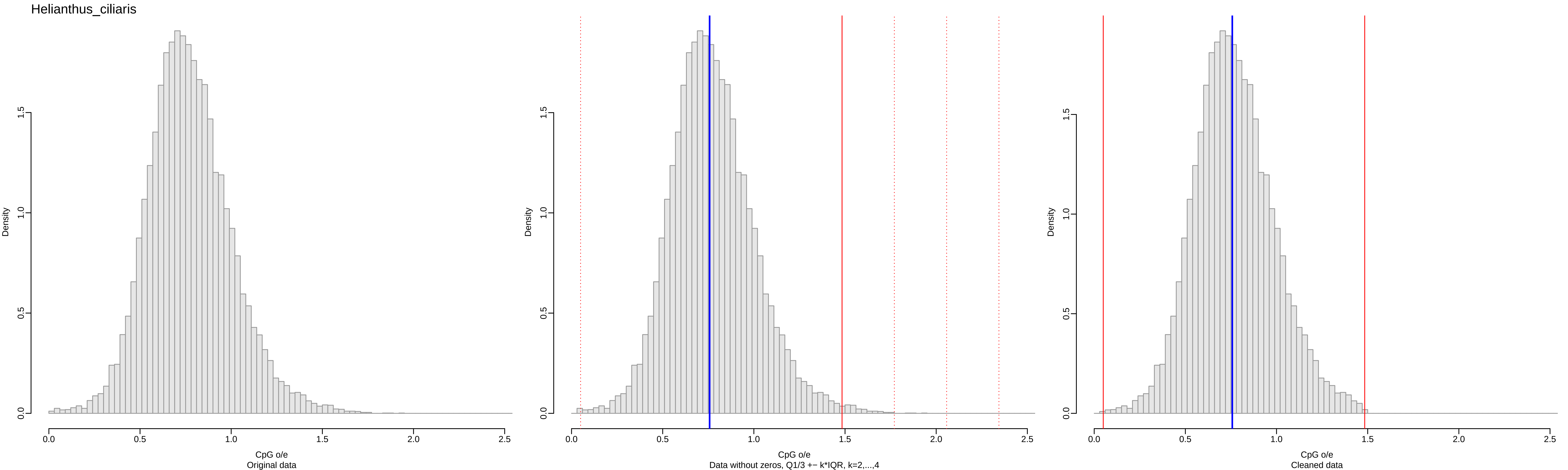
Helianthus_ciliaris
Density
CpG o/e
Original data
Data without zeros, Q1/3 +− k*IQR, k=2,...,4
Cleaned data
0.0
0.5
1.0
1.5
2.0
2.5

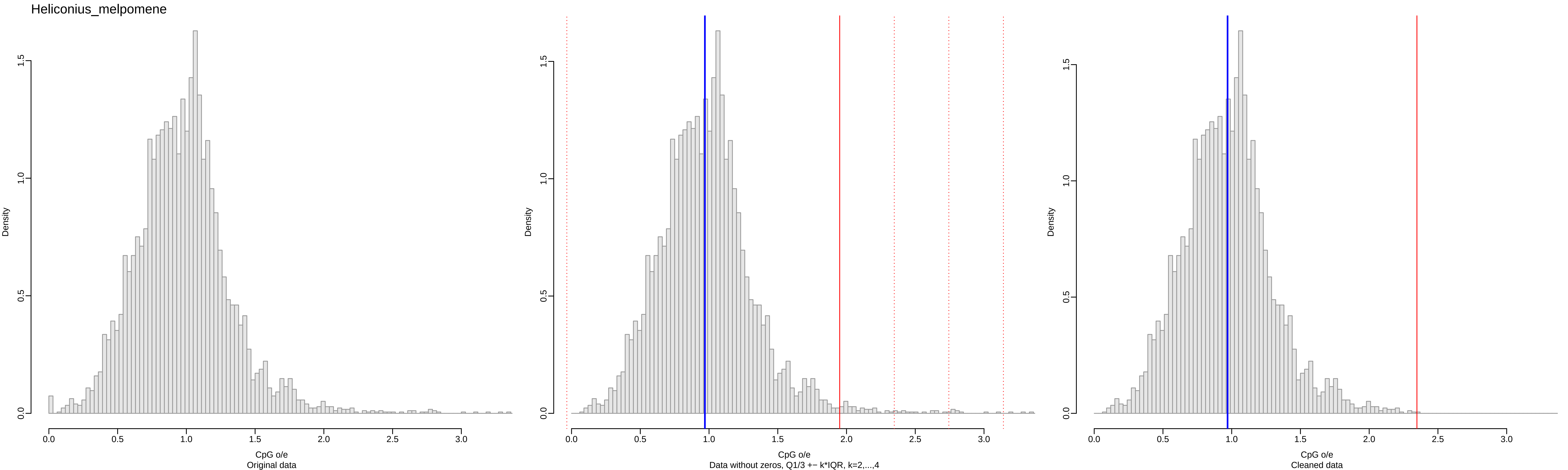
Heliconius_melpomene
Density
CpG o/e
Original data
CpG o/e
Data without zeros, Q1/3 +− k*IQR, k=2,...,4
CpG o/e
Cleaned data

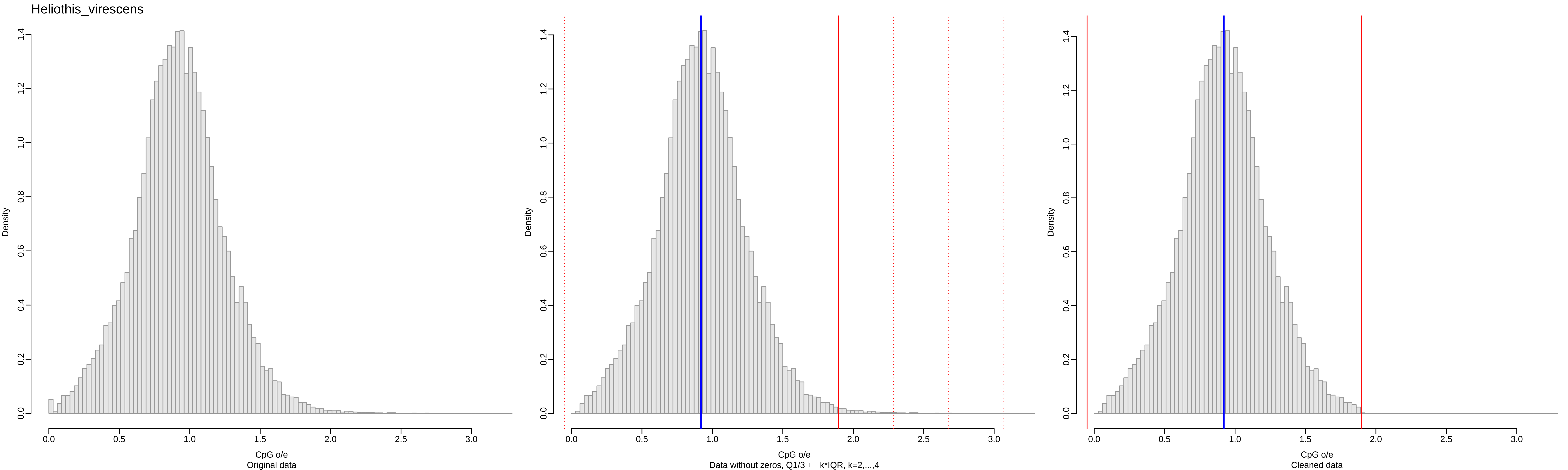

Heliothis_virescens
Density
CpG o/e
Original data
Density
CpG o/e
Data without zeros, Q1/3 +− k*IQR, k=2,...,4
Density
CpG o/e
Cleaned data

Helobdella_robusta

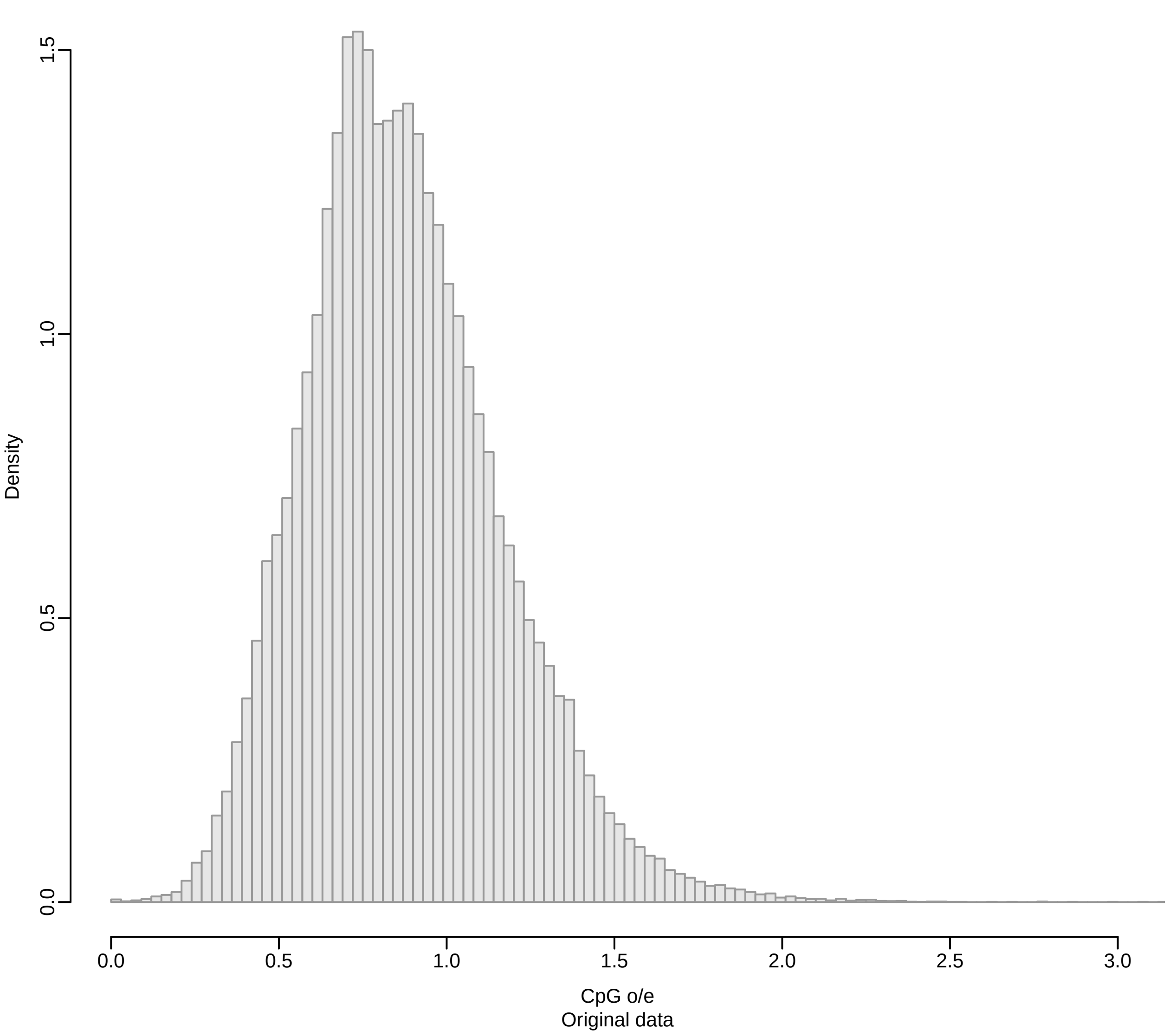

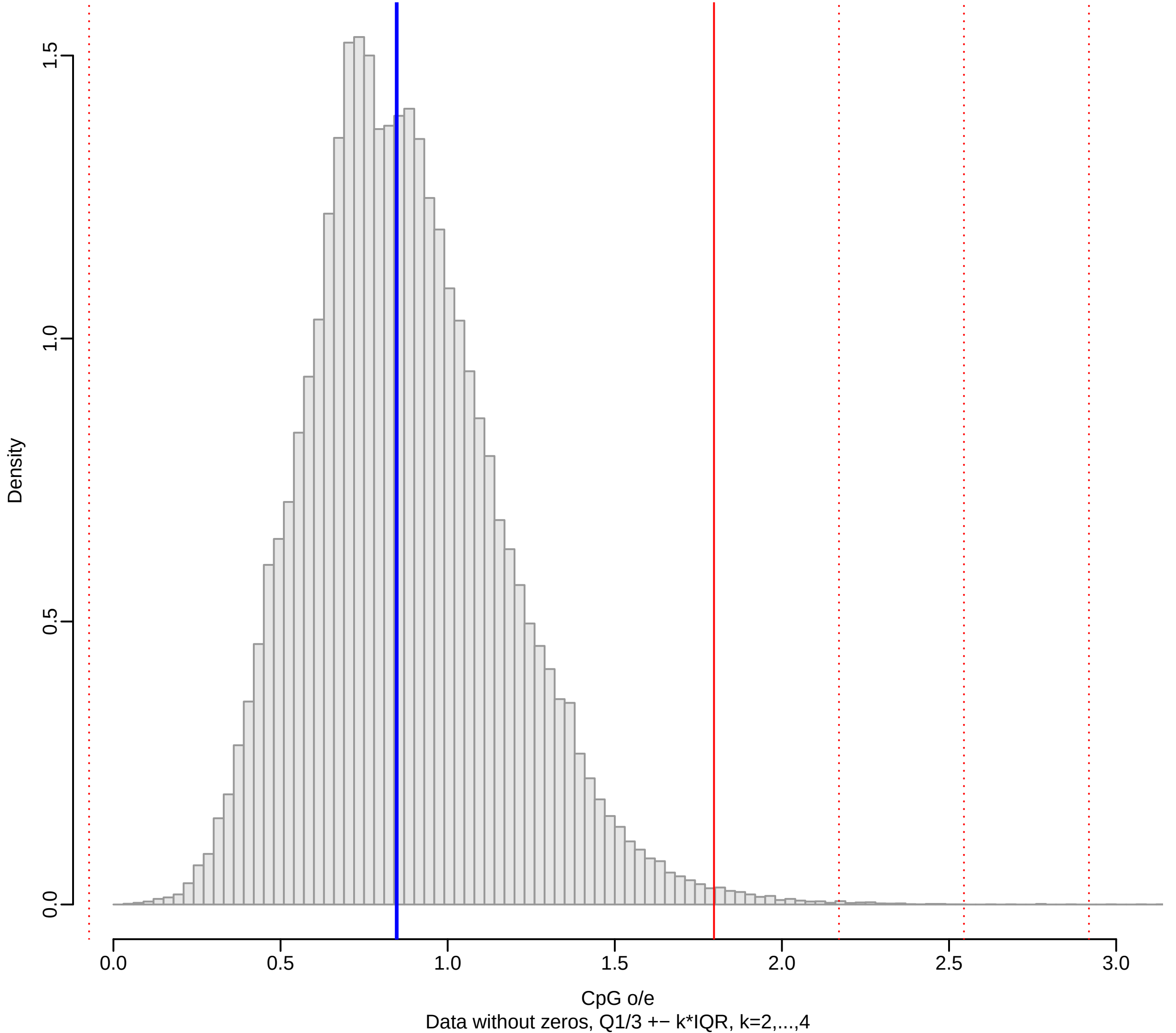

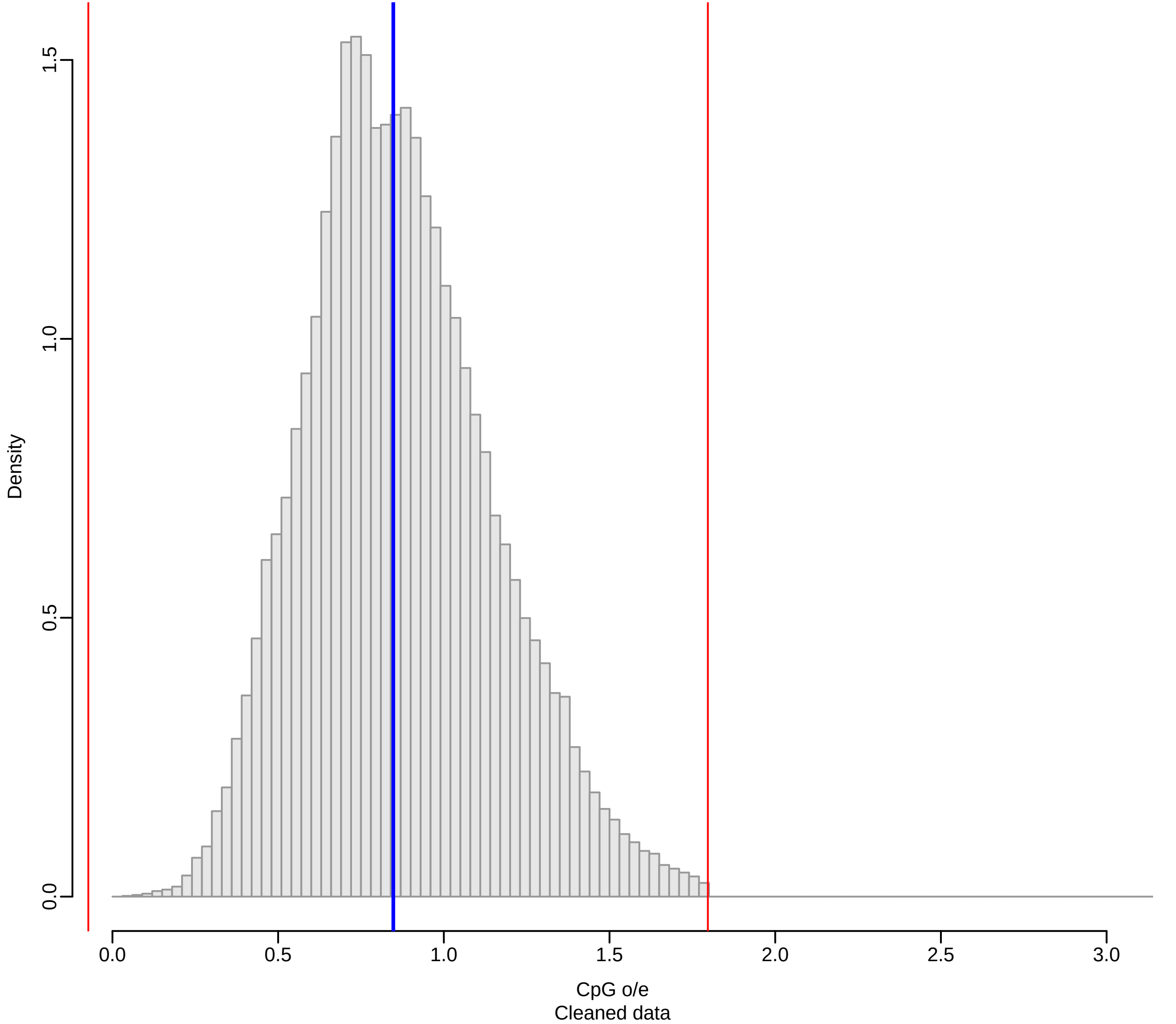

Hemicentrotus_pulcherrimus

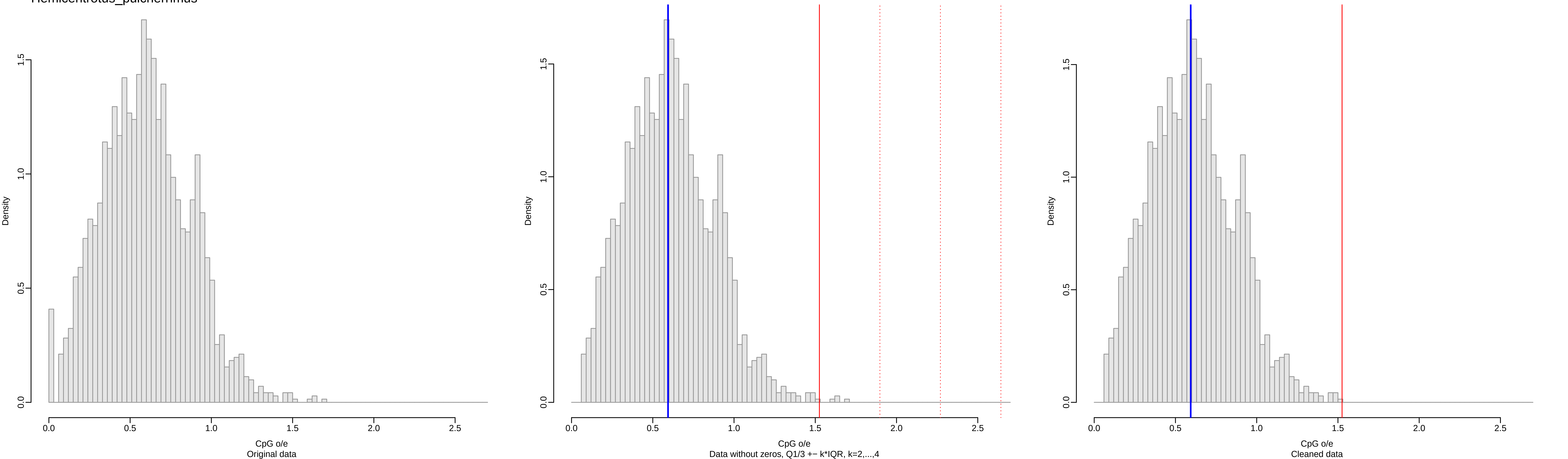

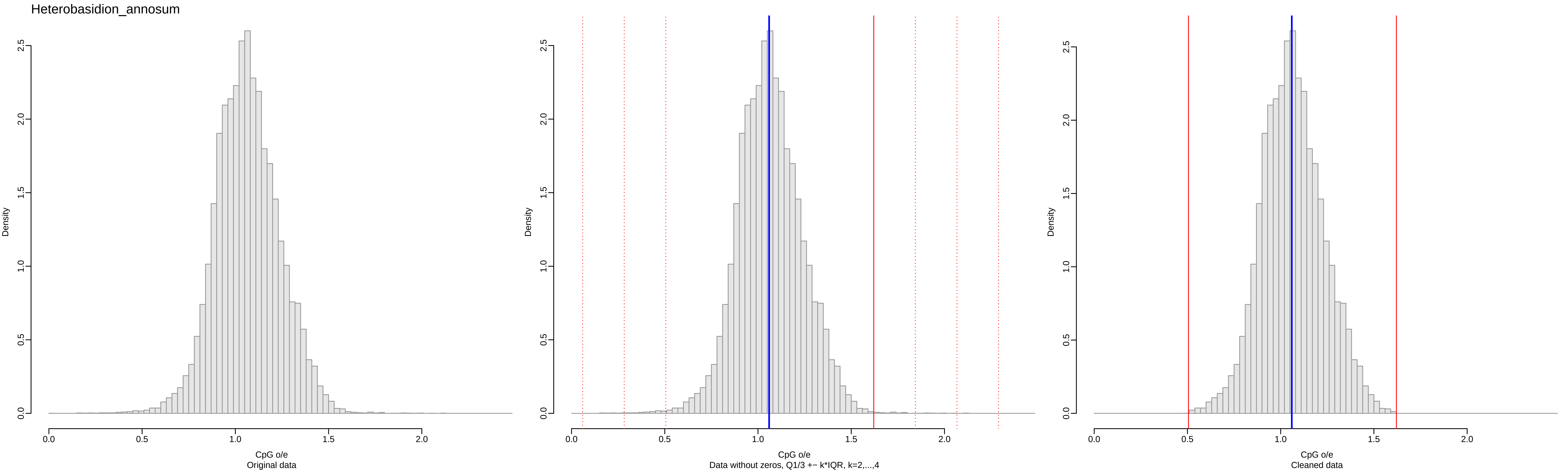
Heterobasidion_annosum
Density
CpG o/e
Original data
Density
CpG o/e
Data without zeros, Q1/3 +− k*IQR, k=2,...,4
Density
CpG o/e
Cleaned data
0.0
0.5
1.0
1.5
2.0
2.5

Heterocapsa_triquetra

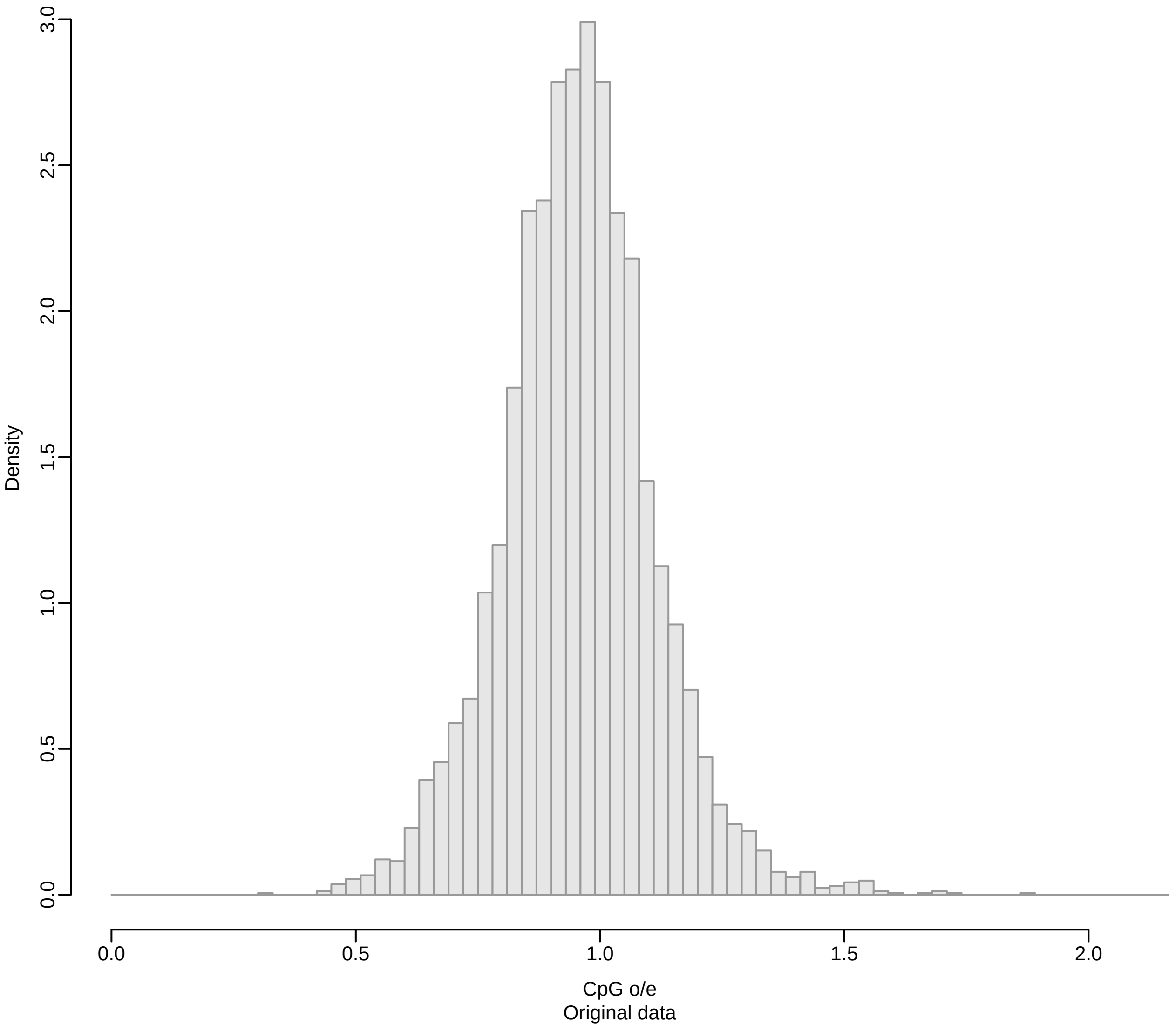

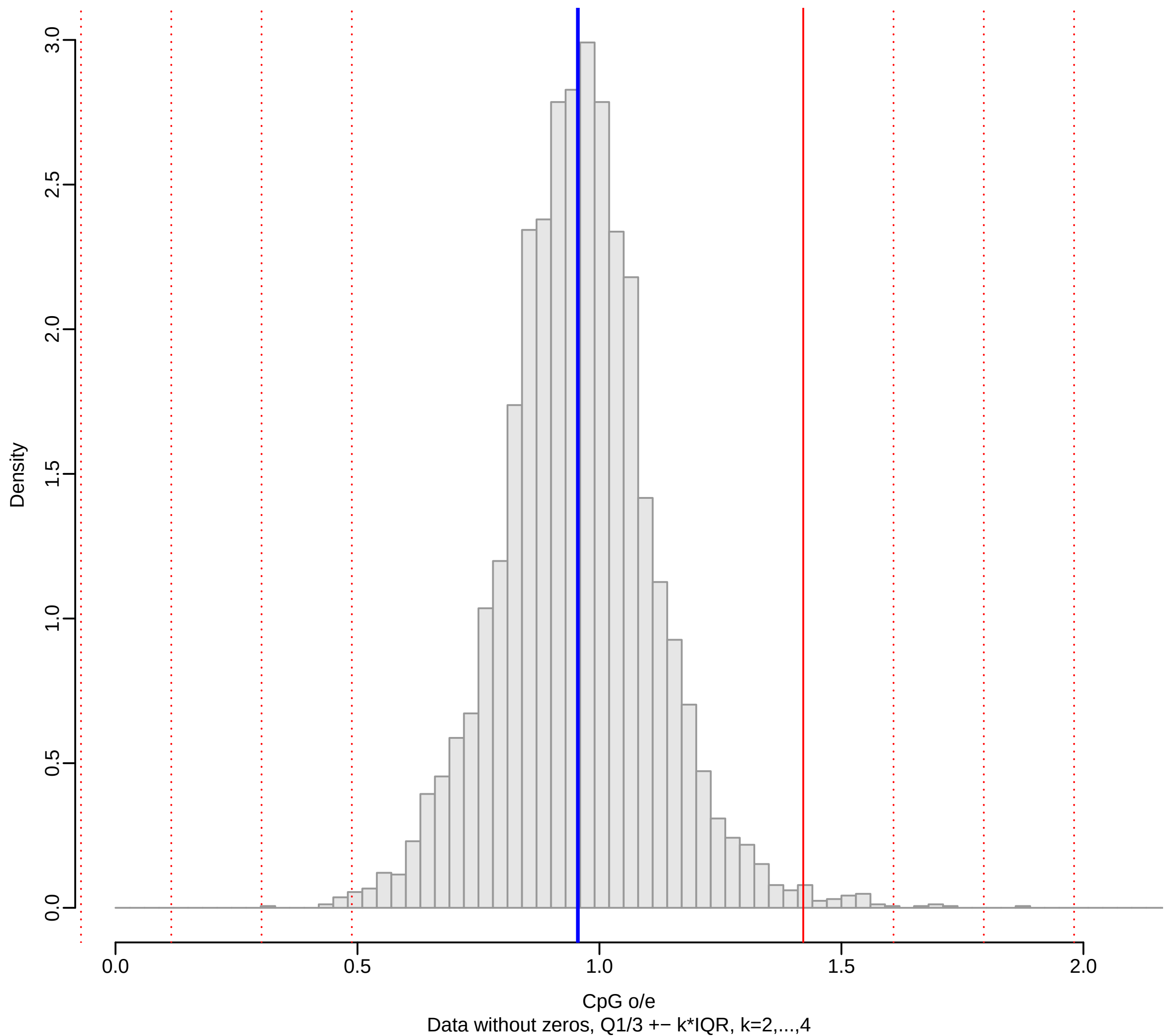

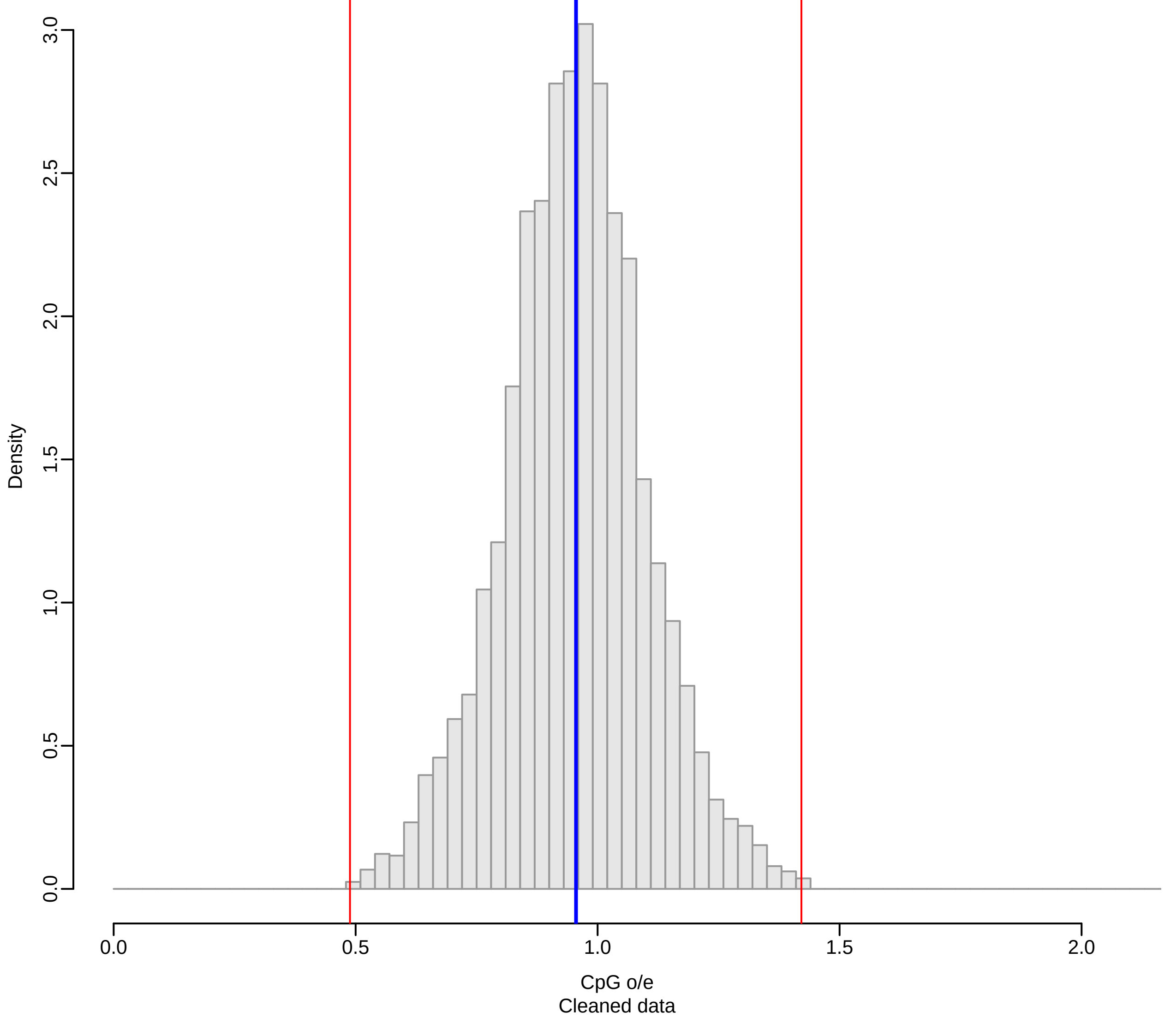

Heterochone_calyx

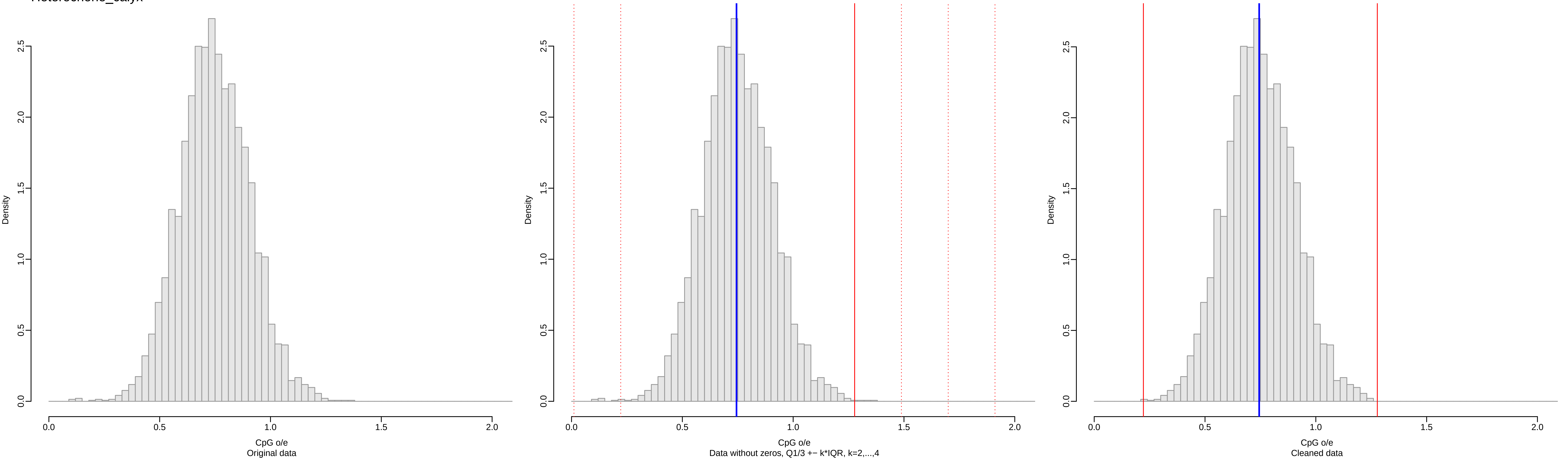

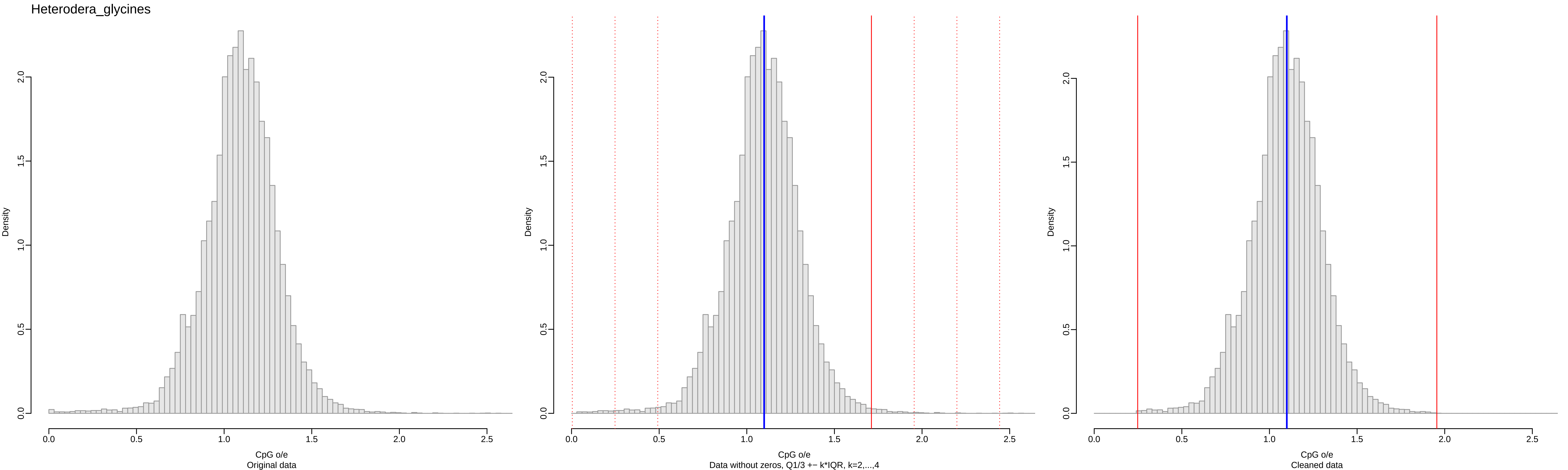

Heterodera_glycines
Density
CpG o/e
Original data
Data without zeros, Q1/3 +− k*IQR, k=2,...,4
Cleaned data
0.0
0.5
1.0
1.5
2.0
2.5

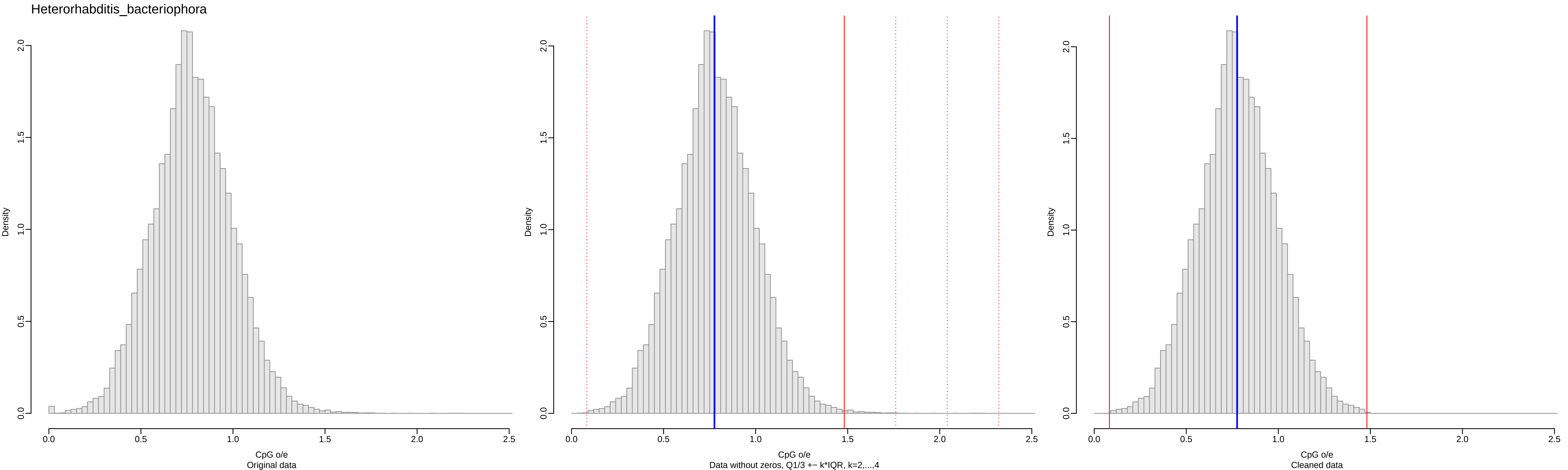

Heterorhabditis_bacteriophora
Density
CpG o/e
Original data
Data without zeros, Q1/3 +− k*IQR, k=2,...,4
Cleaned data
0.0
0.5
1.0
1.5
2.0
2.5

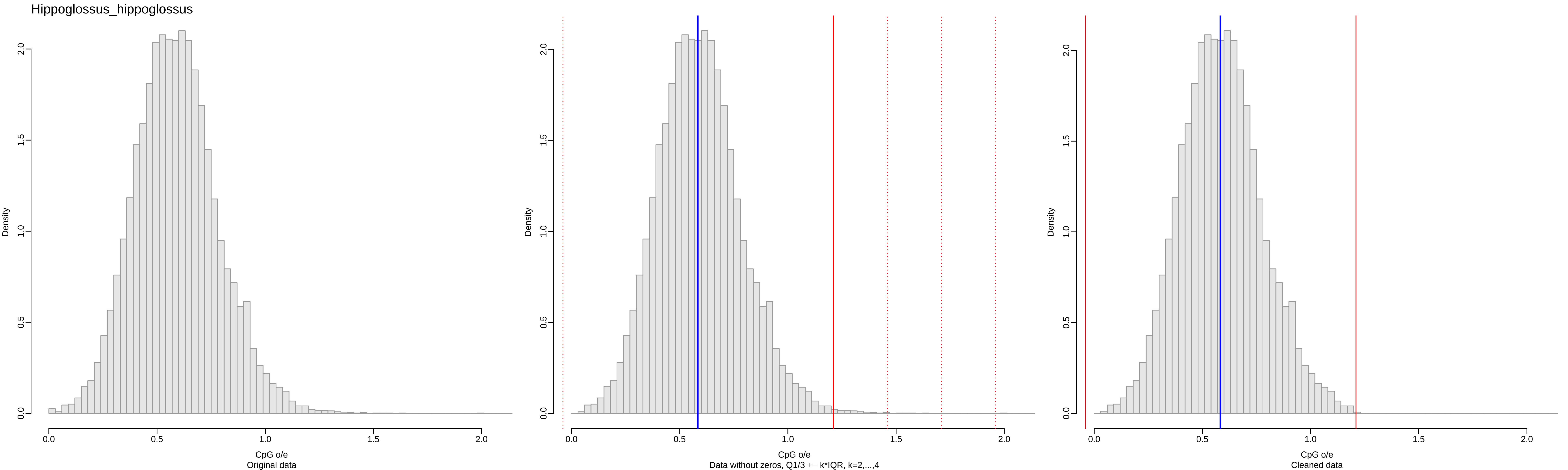

Hippoglossus_hippoglossus
Density
0.0
0.5
1.0
1.5
2.0
CpG o/e
Original data
Data without zeros, Q1/3 +− k*IQR, k=2,...,4
Cleaned data

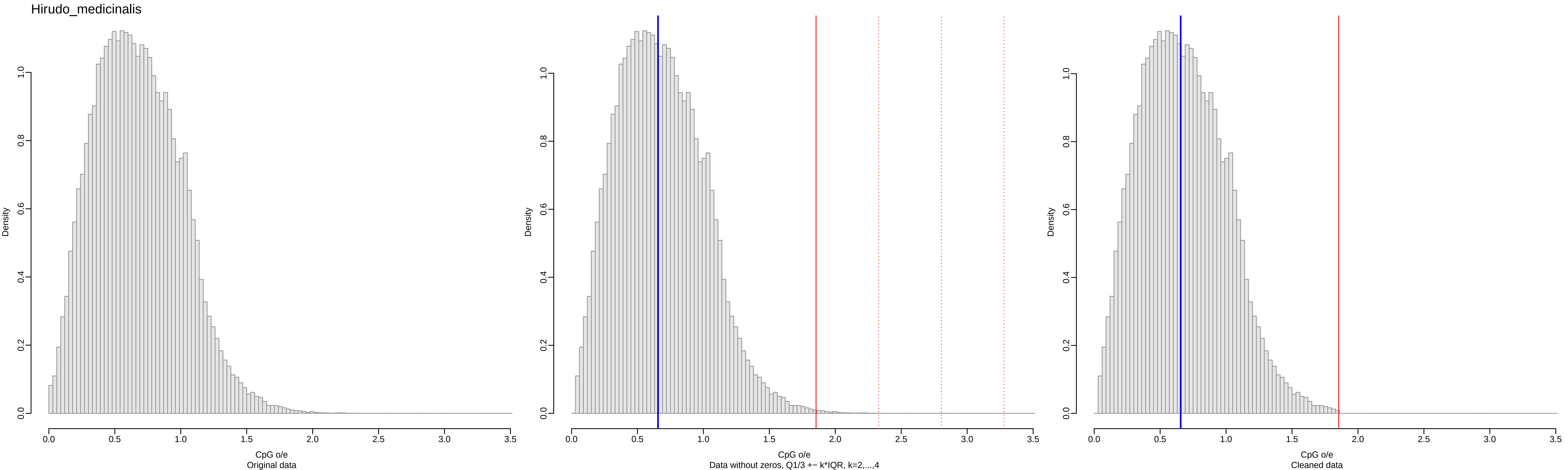

Hirudo_medicinalis
Density
CpG o/e
Original data
Data without zeros, Q1/3 +− k*IQR, k=2,...,4
Cleaned data
0.0
0.2
0.4
0.6
0.8
1.0
0.5
1.5
2.0
2.5
3.0
3.5

Homalodisca_coagulata

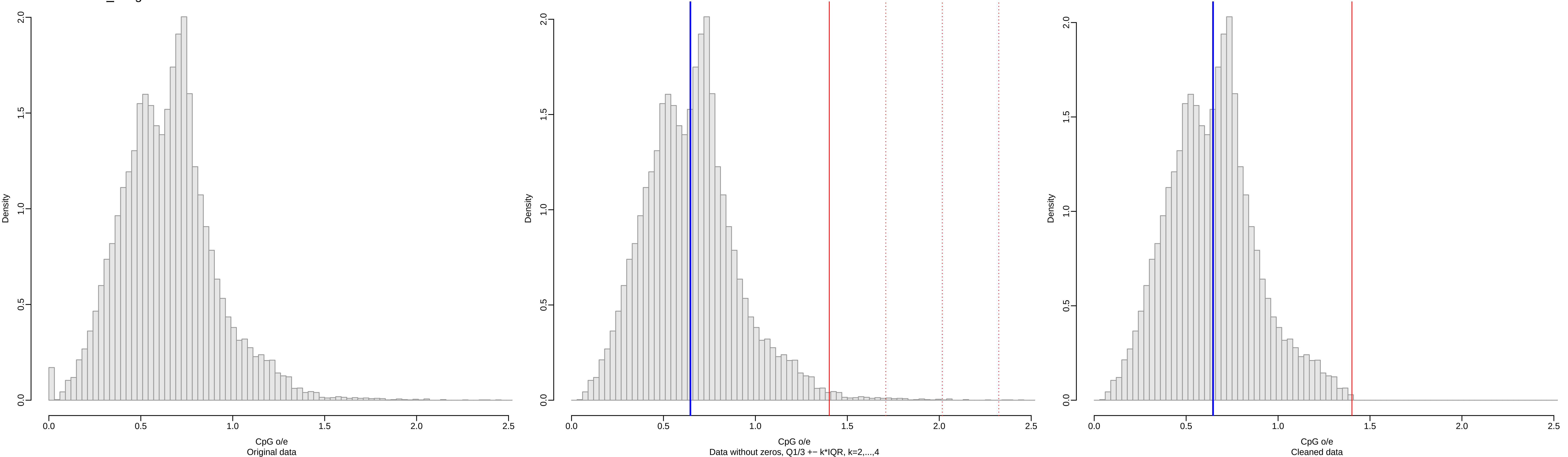

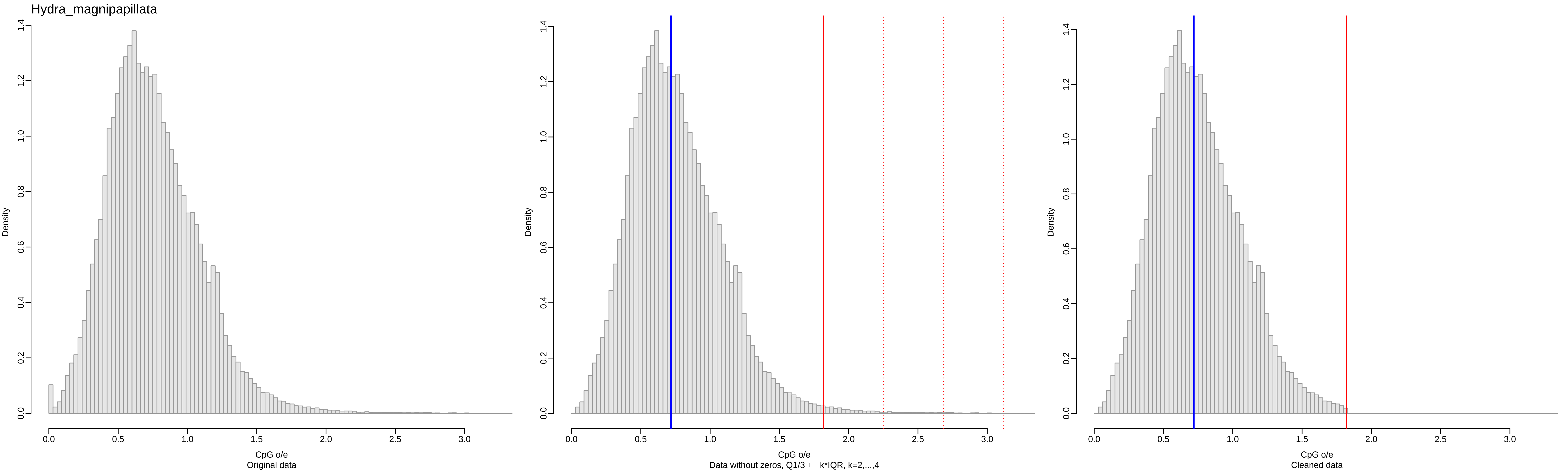
Hydra_magnipapillata
Density
CpG o/e
Original data
Data without zeros, Q1/3 +− k*IQR, k=2,...,4
Cleaned data

Hydra_oligactis

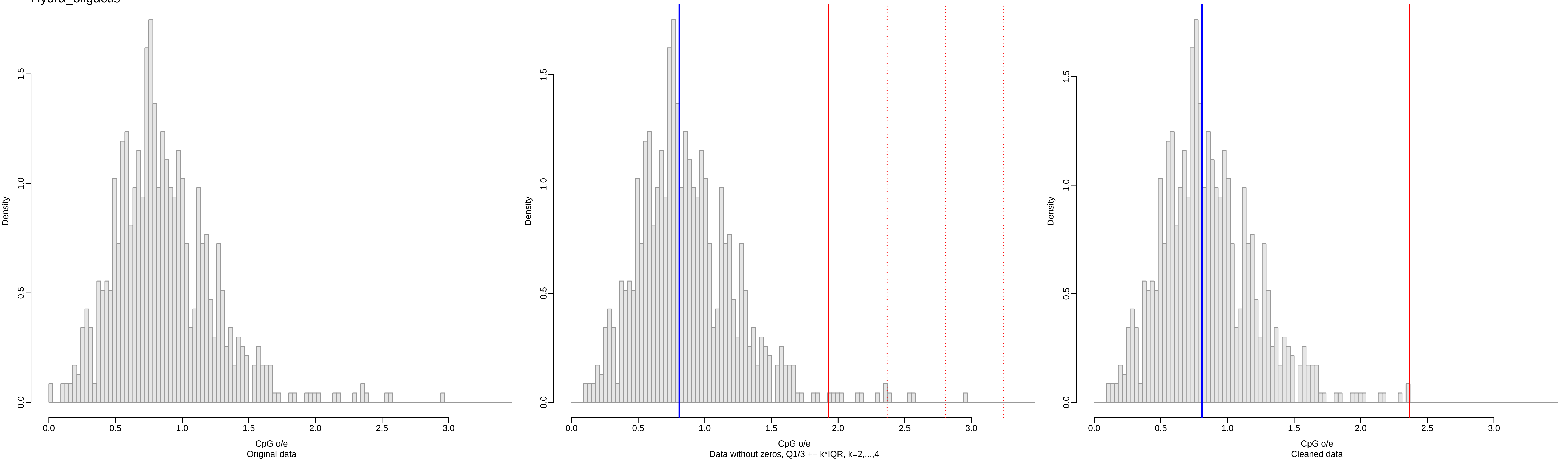

Hydra_vulgaris

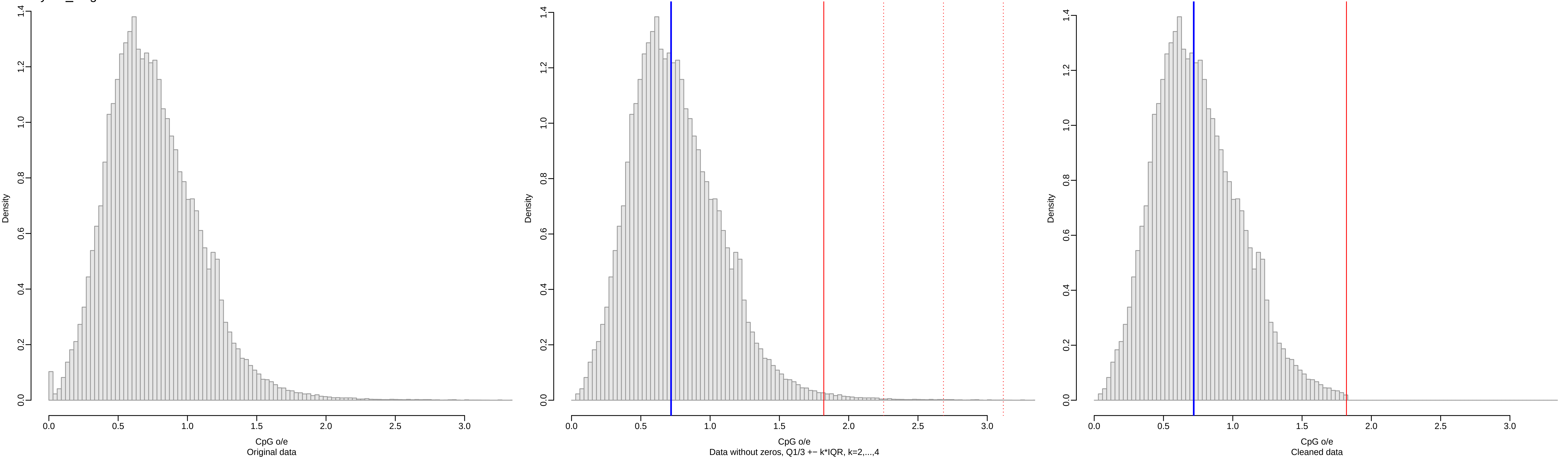

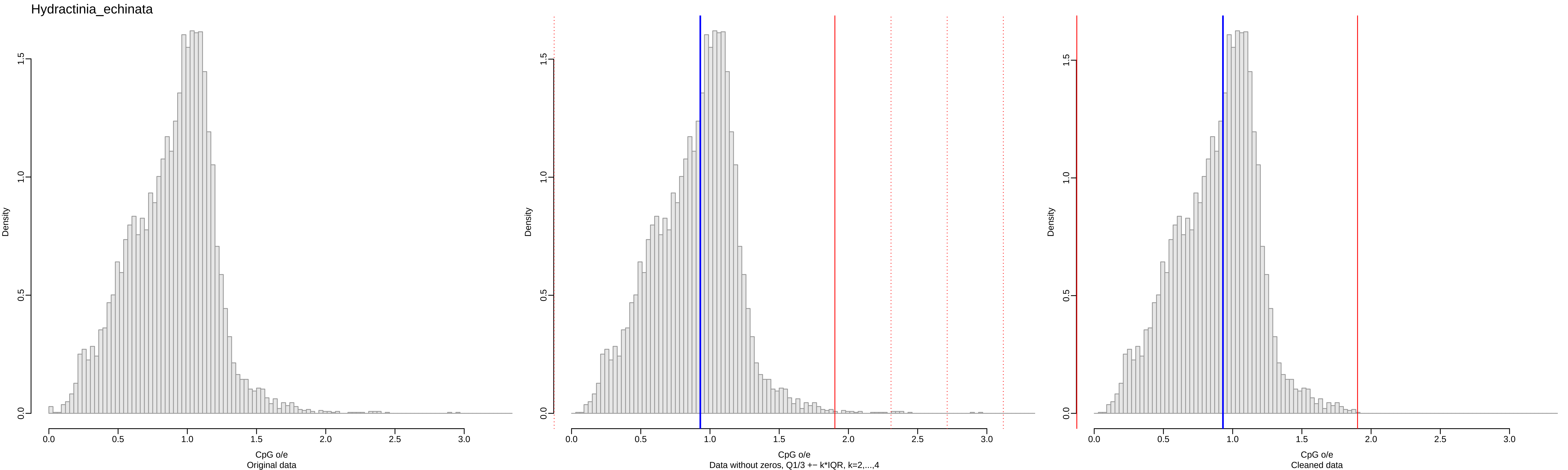
Hydractinia_echinata
Density
CpG o/e
Original data
Data without zeros, Q1/3 +− k*IQR, k=2,...,4
Cleaned data
0.0
0.5
1.0
1.5
2.0
2.5
3.0

Hyoscyamus_niger

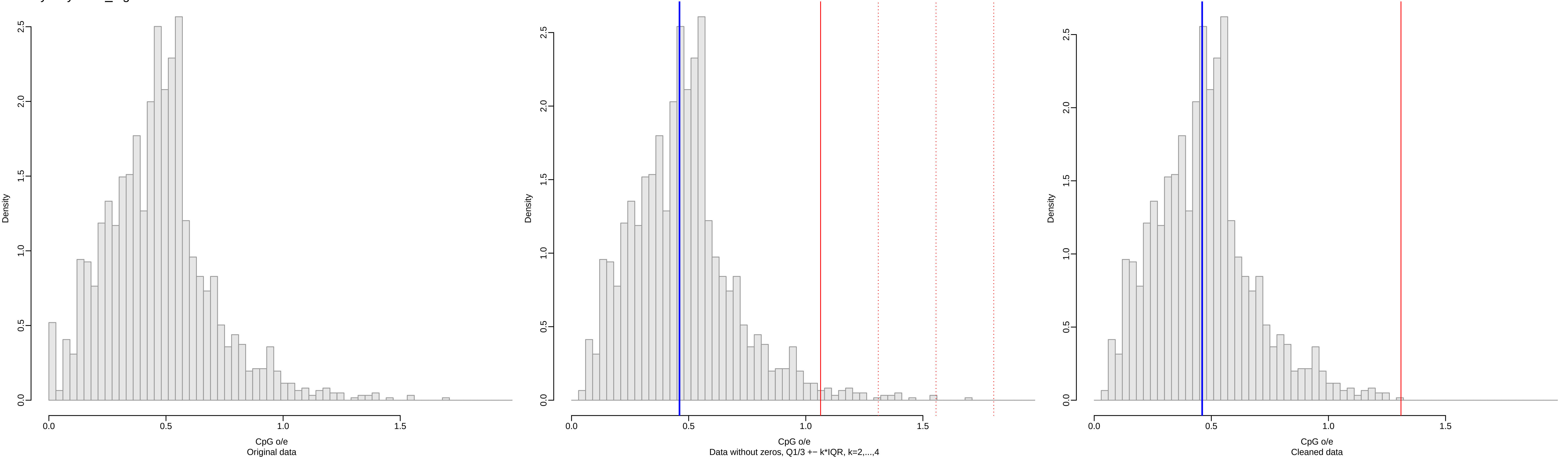

Hypocrea_lixii

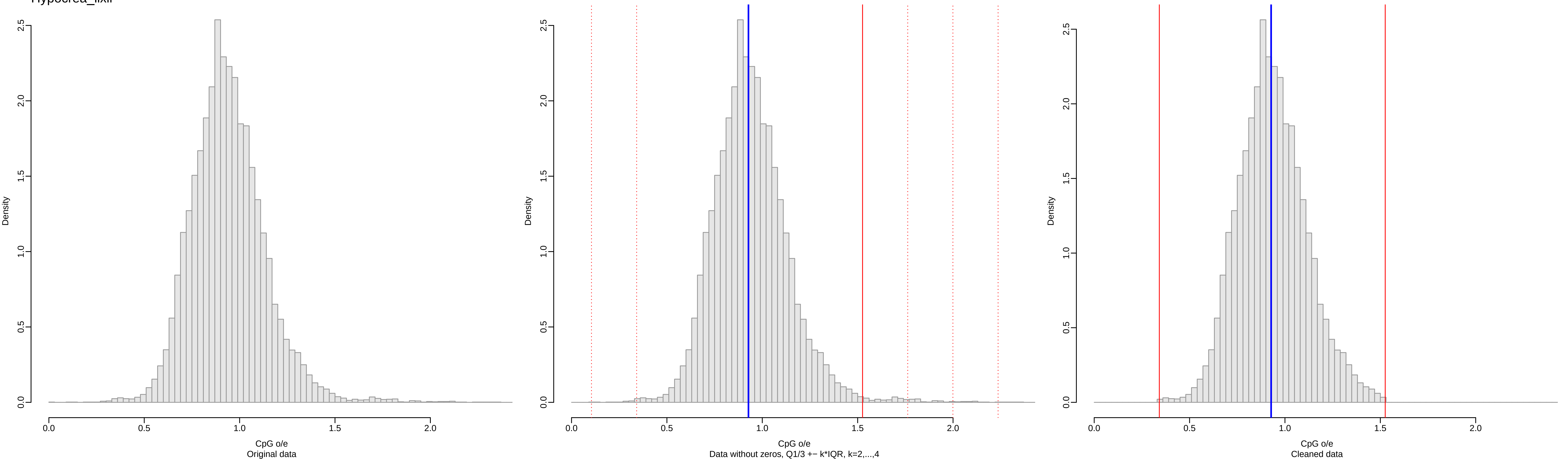

Hypocrea_virens

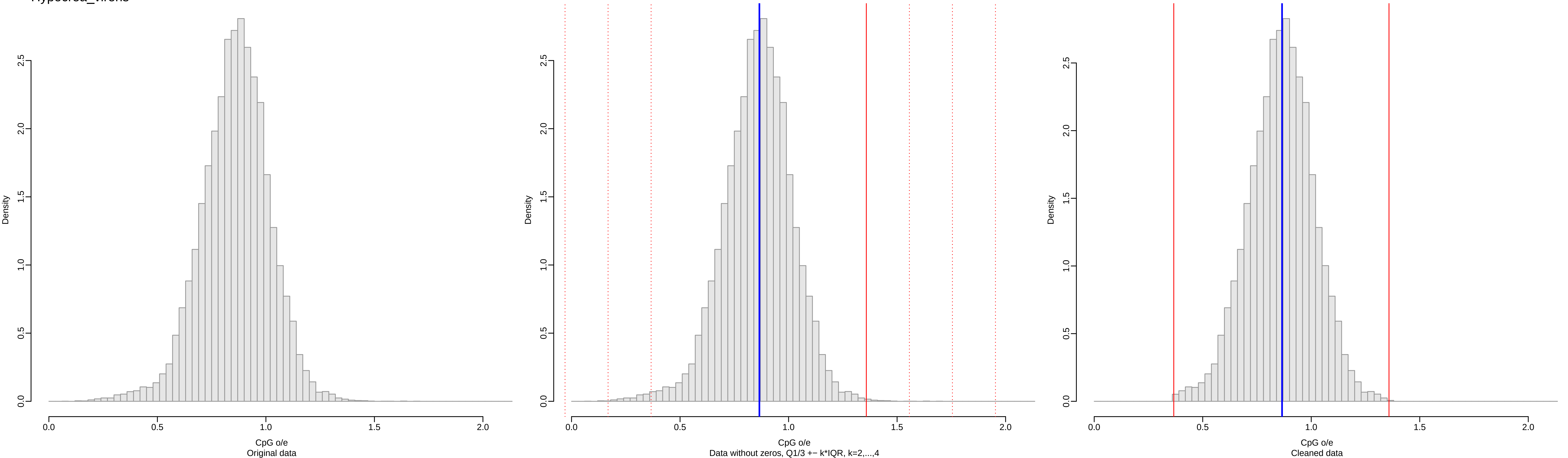

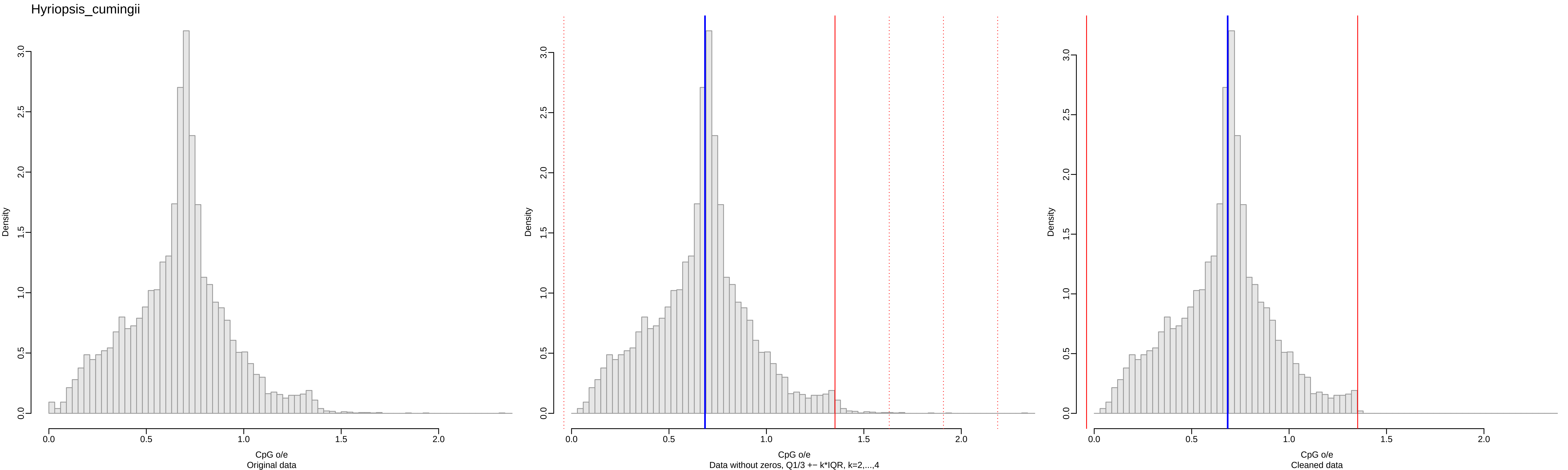
Hyriopsis_cumingii
Density
CpG o/e
Original data
Density
CpG o/e
Data without zeros, Q1/3 +− k*IQR, k=2,...,4
Density
CpG o/e
Cleaned data

Ictalurus_furcatus

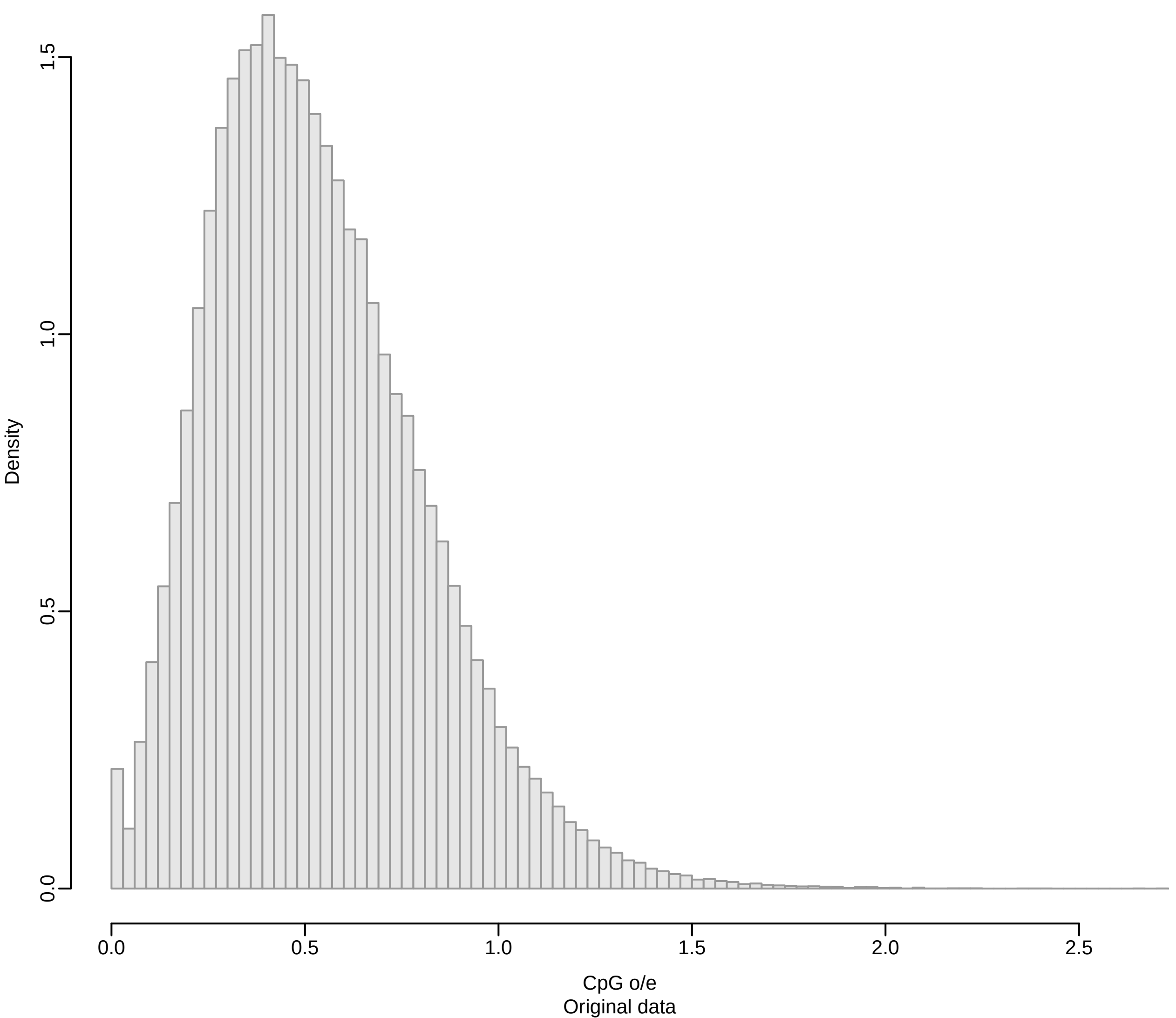

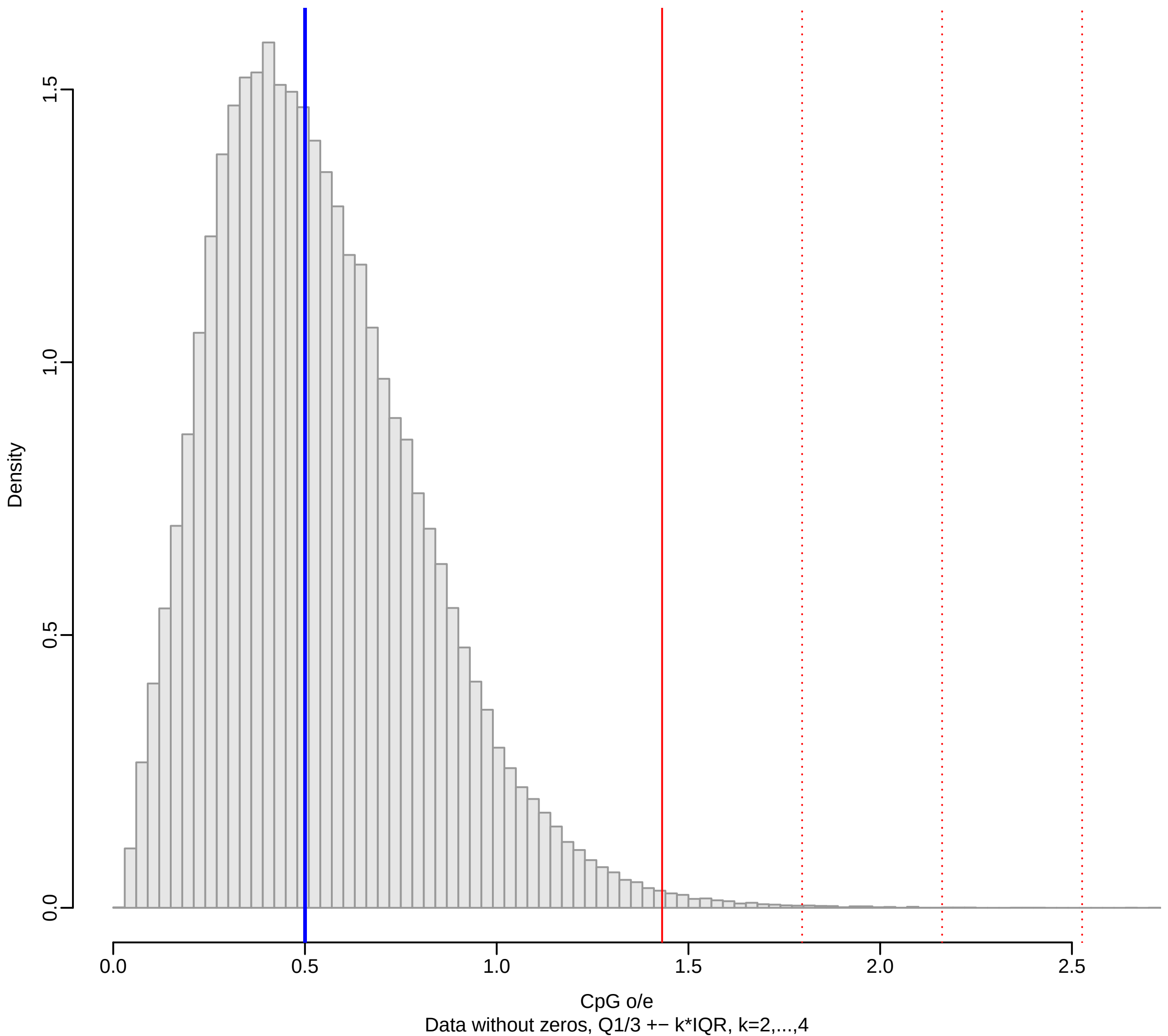

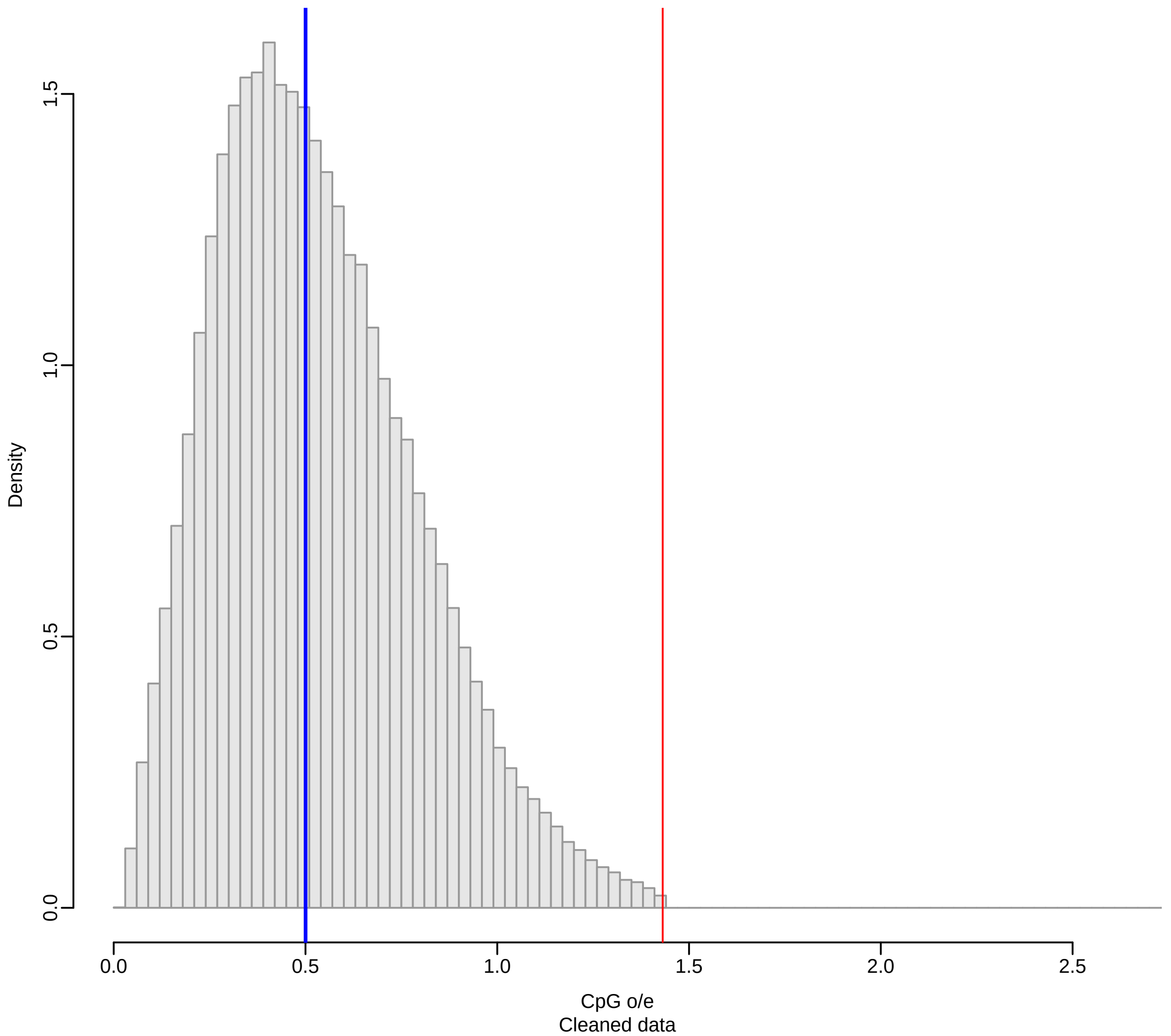

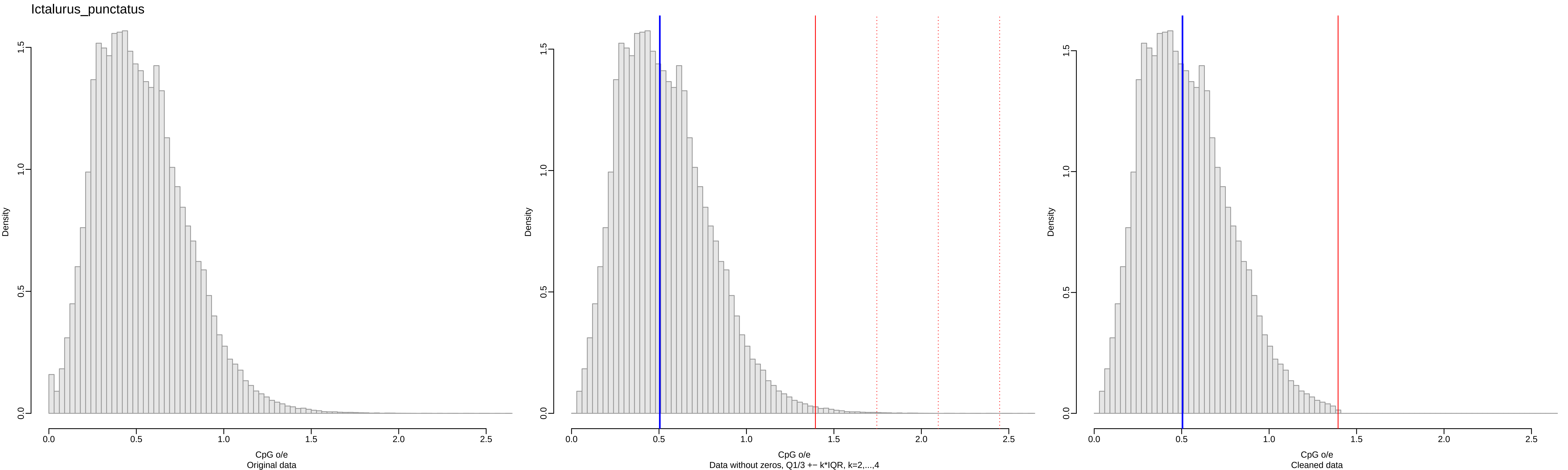
Ictalurus_punctatus
Density
CpG o/e
Original data
Data without zeros, Q1/3 +− k*IQR, k=2,...,4
Cleaned data
0.0
0.5
1.0
1.5
2.0
2.5

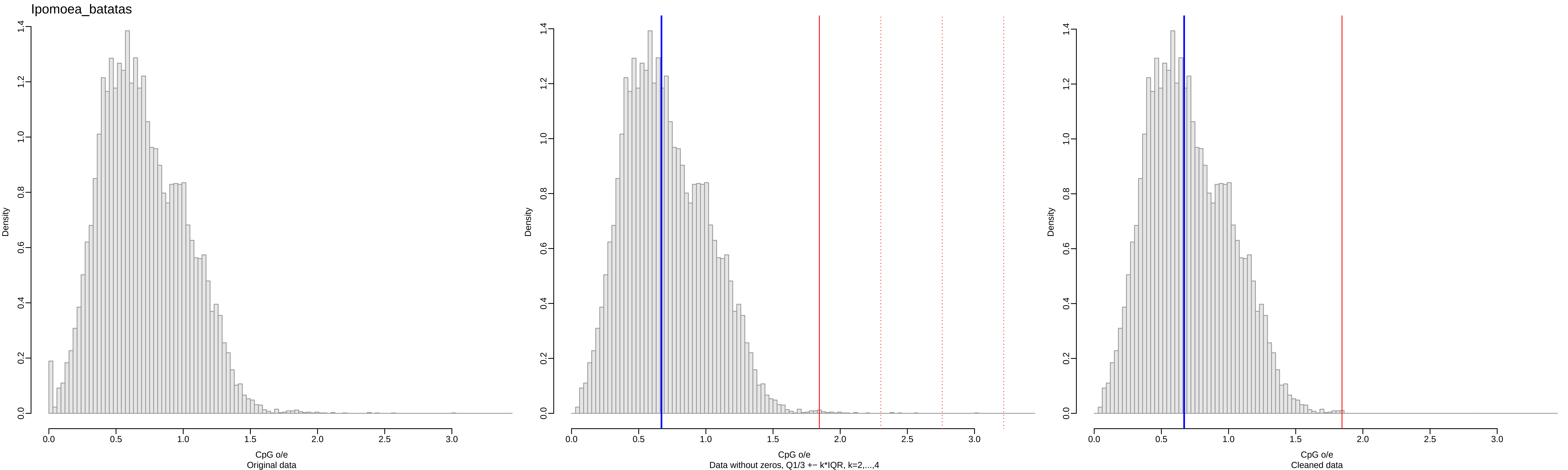

Ipomoea_batatas
Density
CpG o/e
Original data
Data without zeros, Q1/3 +− k*IQR, k=2,...,4
Cleaned data
0.0
0.2
0.4
0.6
0.8
1.0
1.2
1.4
0.5
1.5
2.0
2.5
3.0

Ischnura_elegans

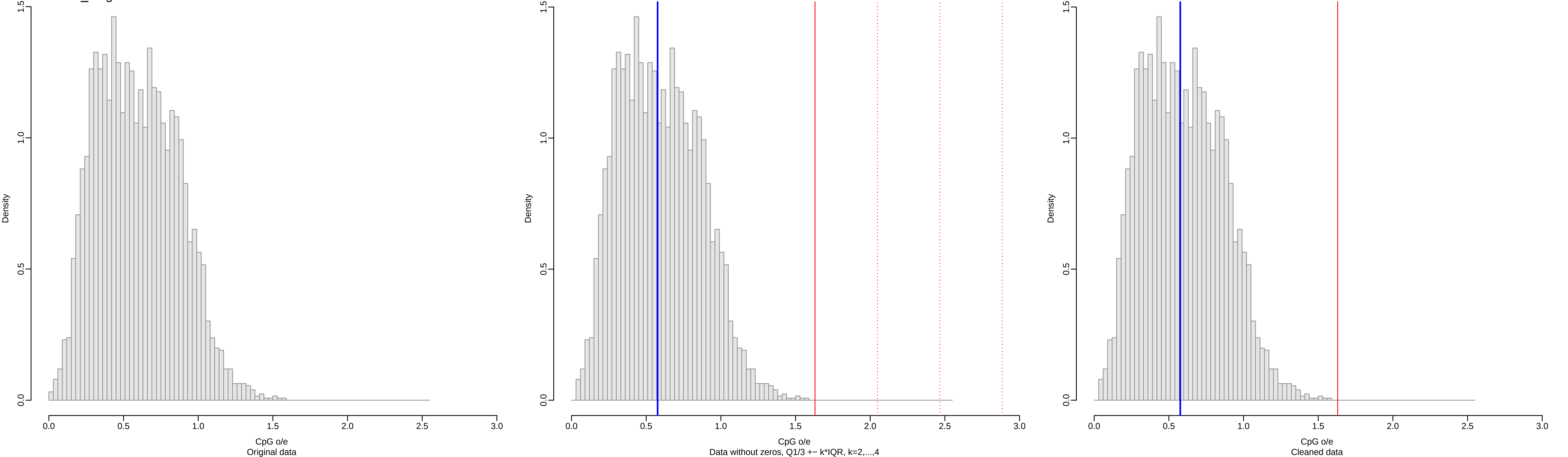

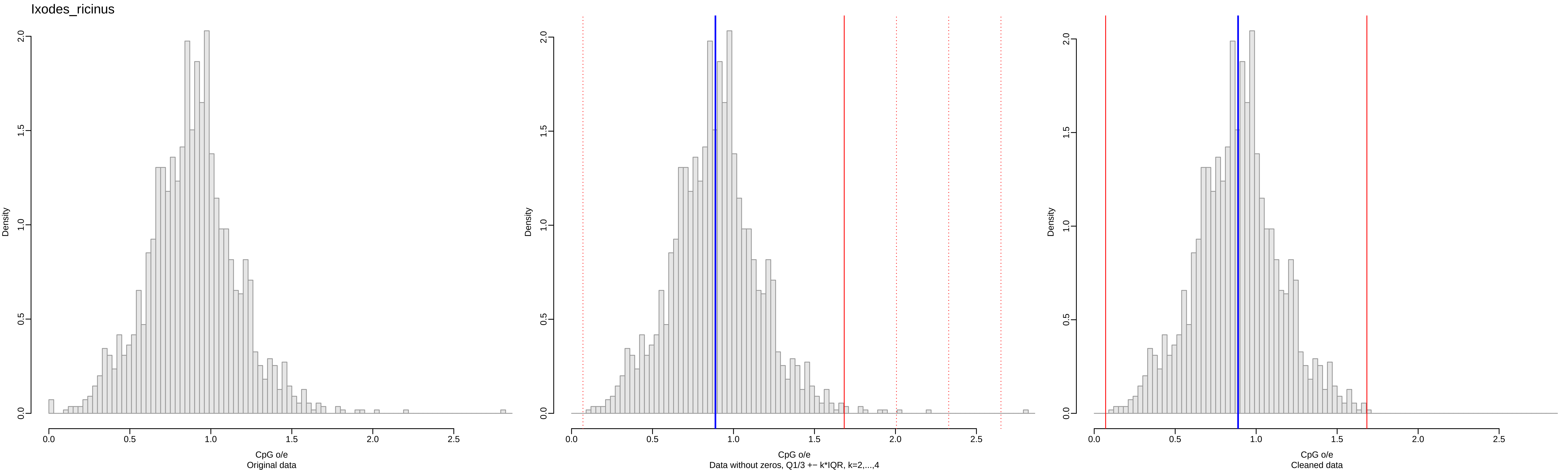

Ixodes_ricinus
Density
CpG o/e
Original data
Data without zeros, Q1/3 +− k*IQR, k=2,...,4
Cleaned data
0.0
0.5
1.0
1.5
2.0
2.5

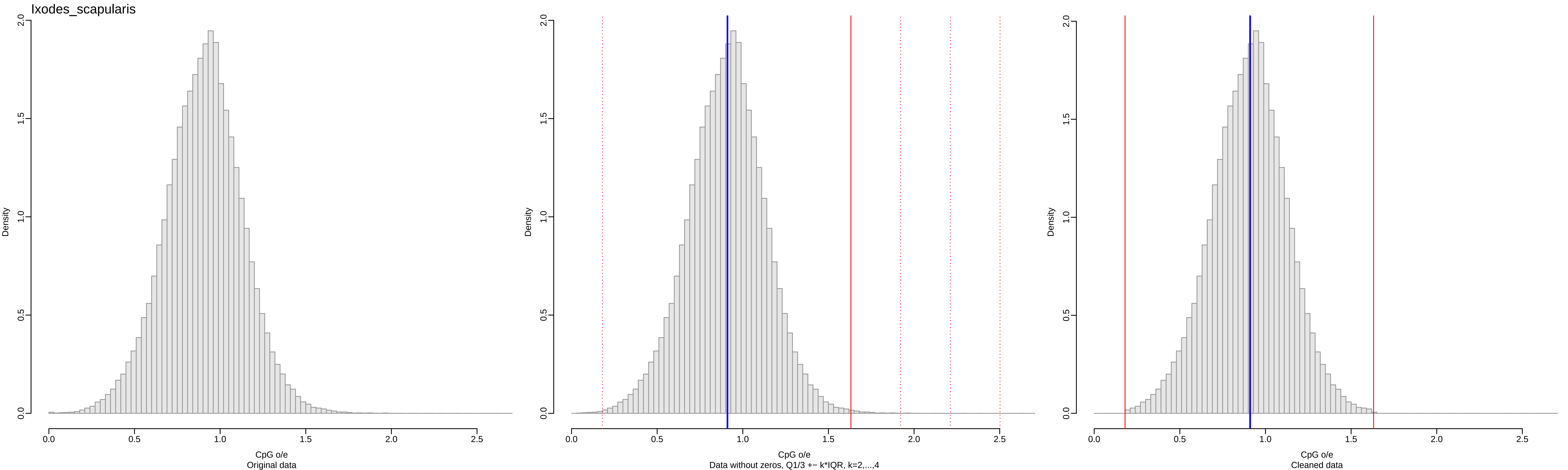

Ixodes_scapularis
Density
CpG o/e
Original data
Data without zeros, Q1/3 +− k*IQR, k=2,...,4
Cleaned data
0.0
0.5
1.0
1.5
2.0
2.5

Jakoba_bahamiensis

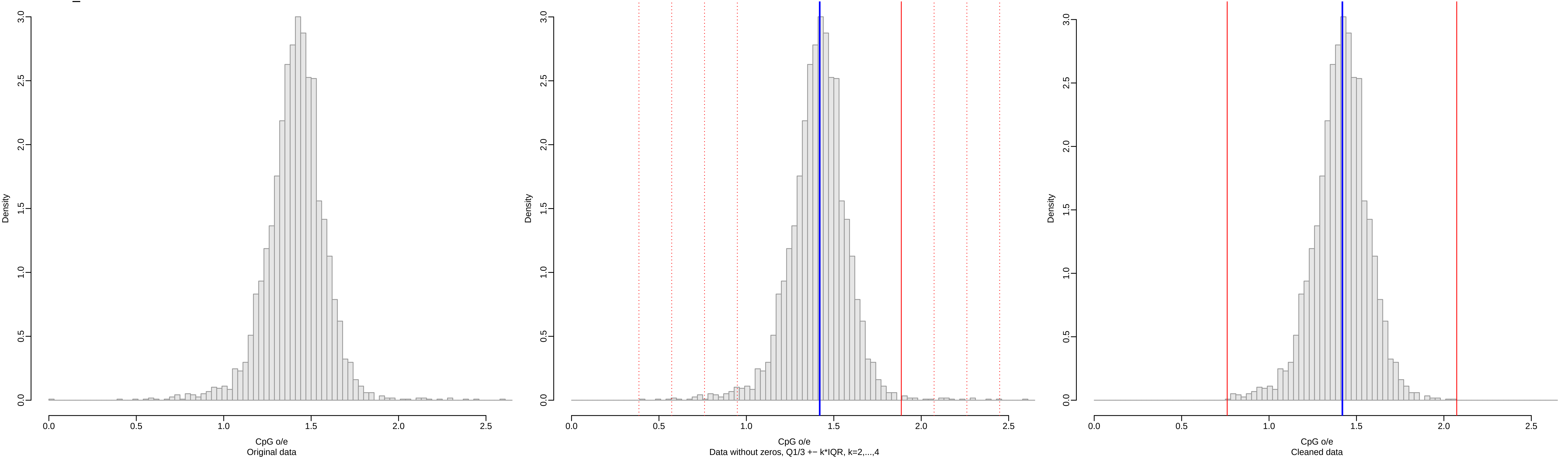

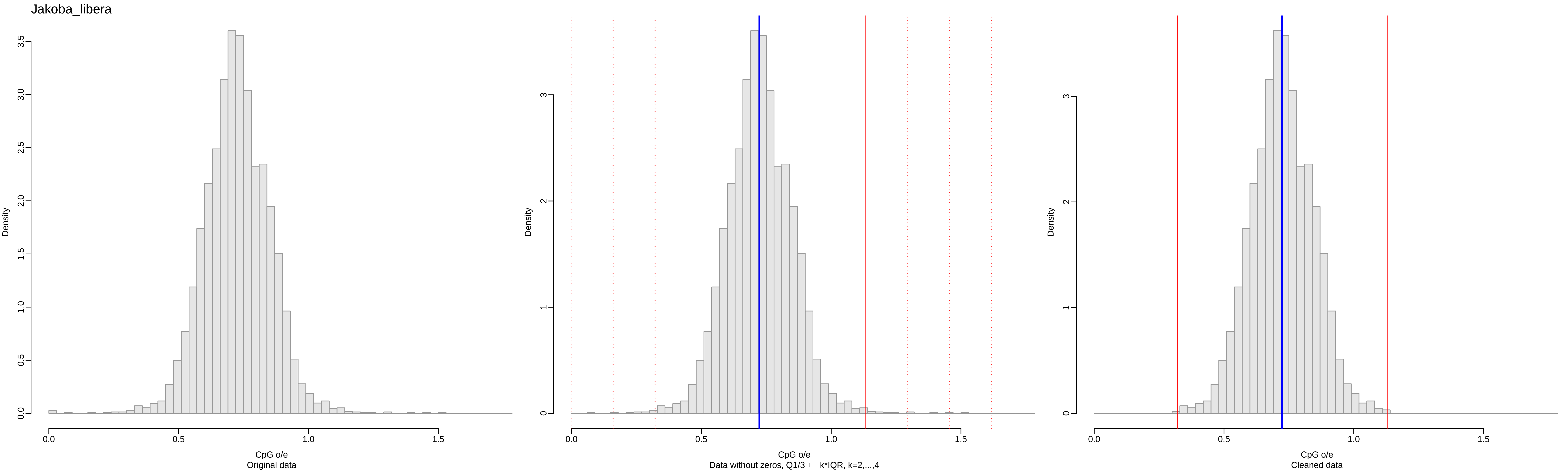

Jakoba_libera
Density
CpG o/e
Original data
Density
CpG o/e
Data without zeros, Q1/3 +− k*IQR, k=2,...,4
Density
CpG o/e
Cleaned data

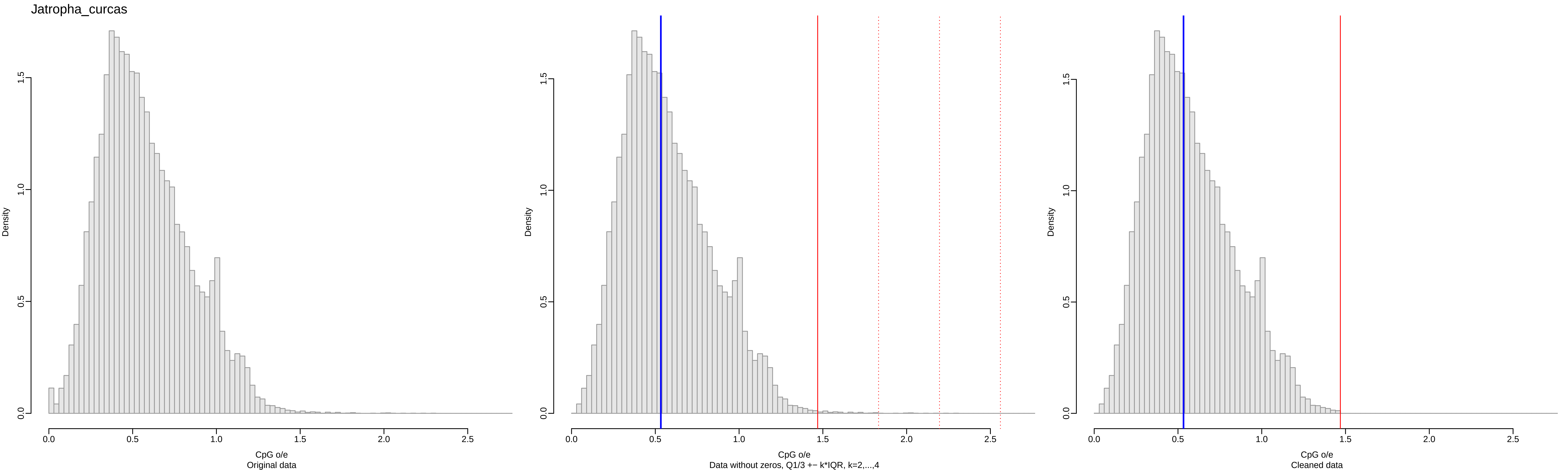

Jatropha_curcas
Density
CpG o/e
Original data
Data without zeros, Q1/3 +− k*IQR, k=2,...,4
Cleaned data
0.0
0.5
1.0
1.5
2.0
2.5

Labeo_rohita

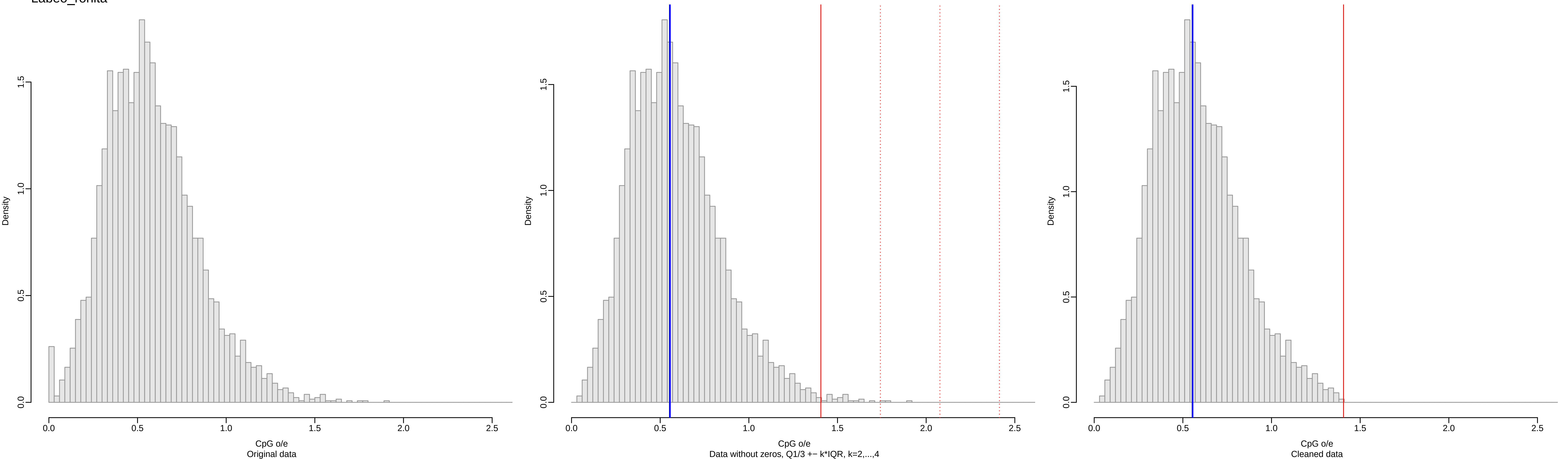

Lagopus_lagopus_scotica

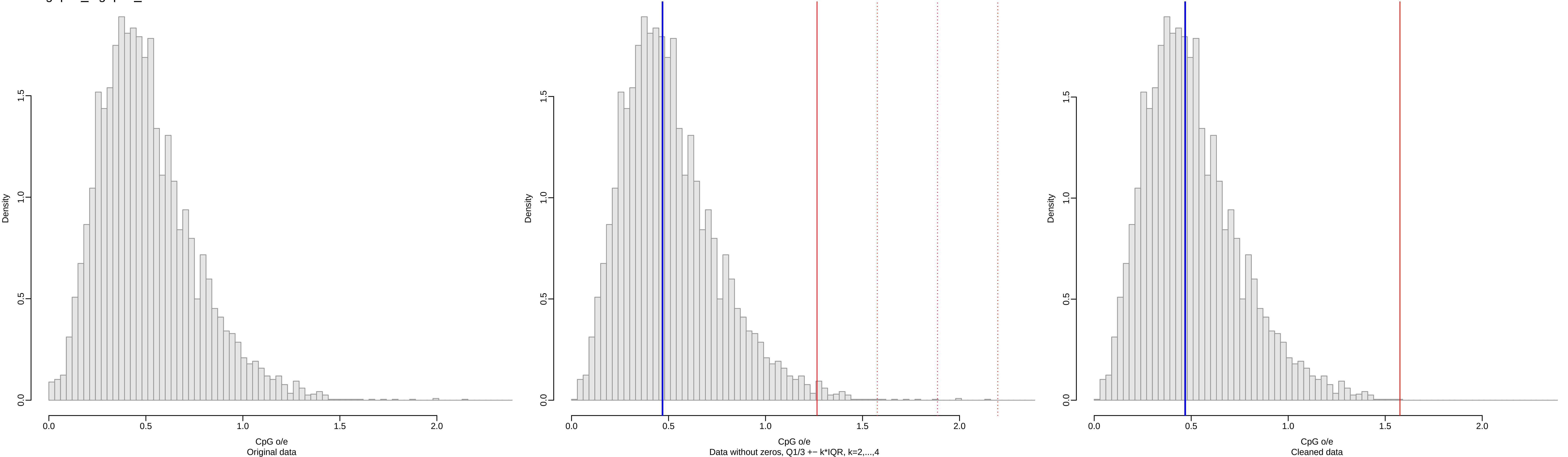

Lama_pacos

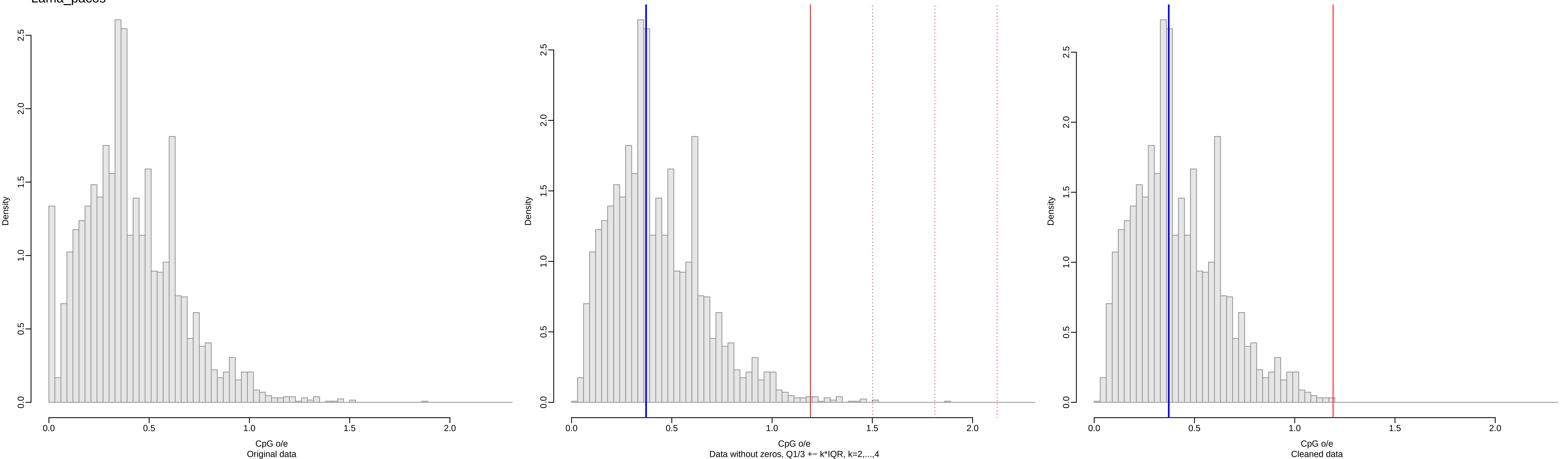

Density
0.0
0.5
1.0
1.5
2.0
2.5
0.0
0.5
1.0
1.5
2.0
CpG o/e
Original data
Density
CpG o/e
Data without zeros, Q1/3 +− k*IQR, k=2,...,4
Density
CpG o/e
Cleaned data

Laminaria_digitata

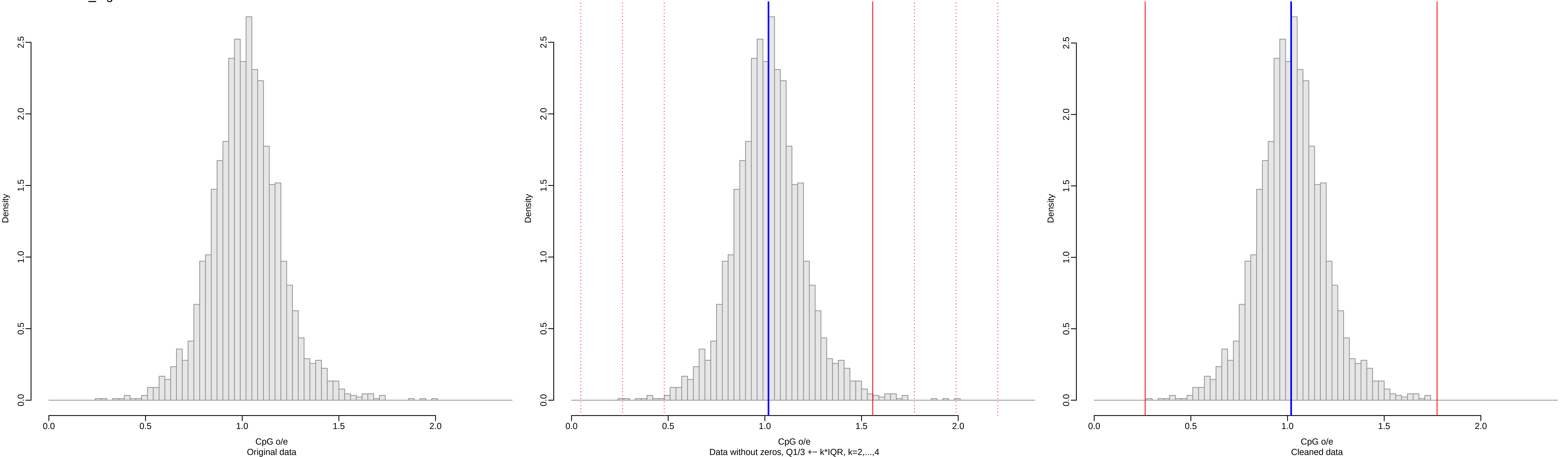

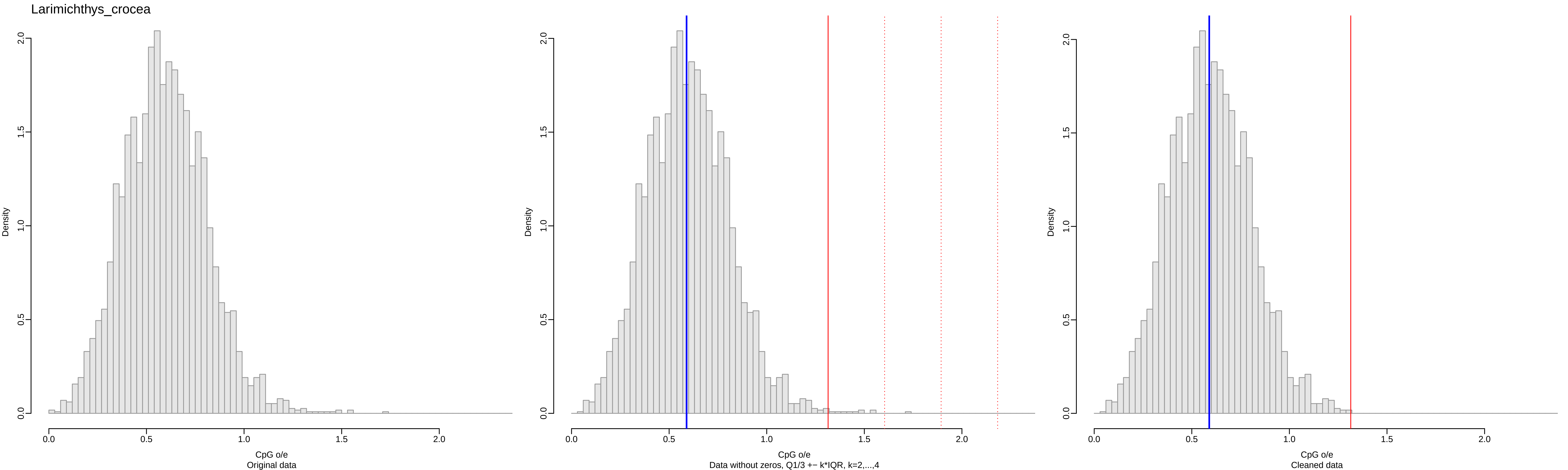

Larimichthys_crocea
Density
CpG o/e
Original data
Data without zeros, Q1/3 +− k*IQR, k=2,...,4
Cleaned data
0.0
0.5
1.0
1.5
2.0

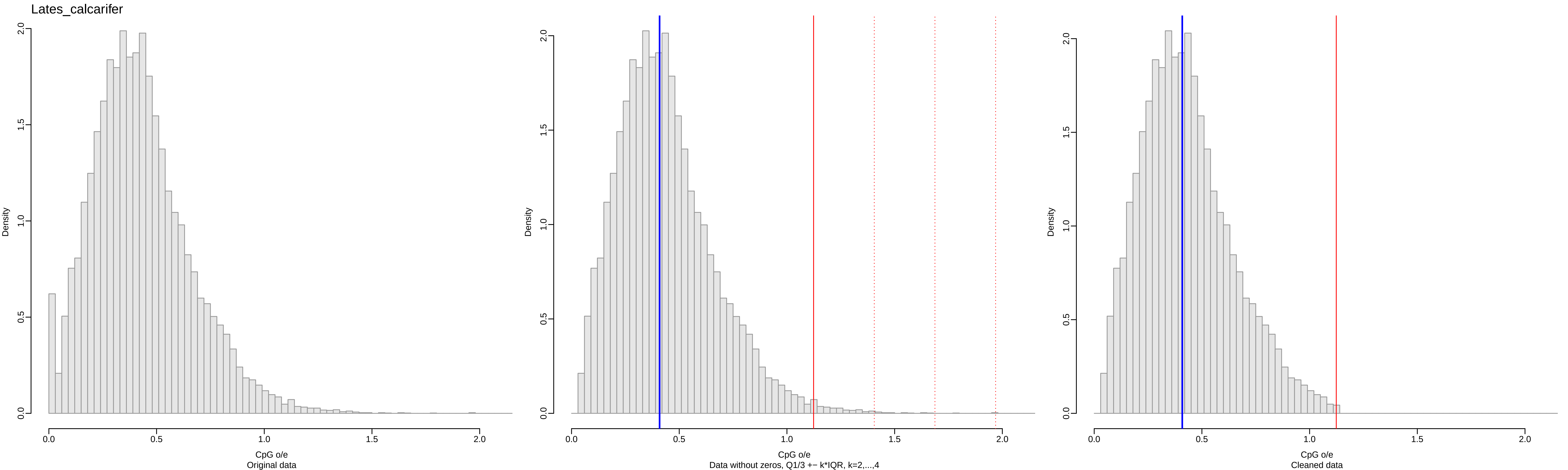
Lates_calcarifer
Density
CpG o/e
Original data
Data without zeros, Q1/3 +− k*IQR, k=2,...,4
Cleaned data
0.0
0.5
1.0
1.5
2.0

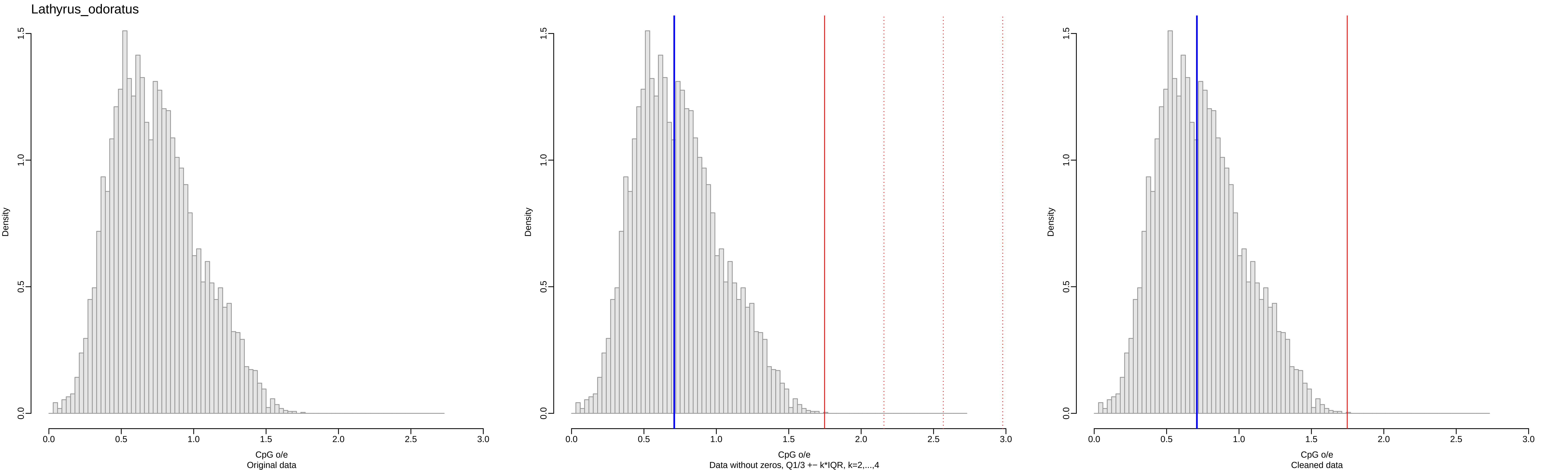

Lathyrus_odoratus
Density
CpG o/e
Original data
Data without zeros, Q1/3 +− k*IQR, k=2,...,4
Cleaned data
0.0
0.5
1.0
1.5
2.0
2.5
3.0

Laupala_kohalensis

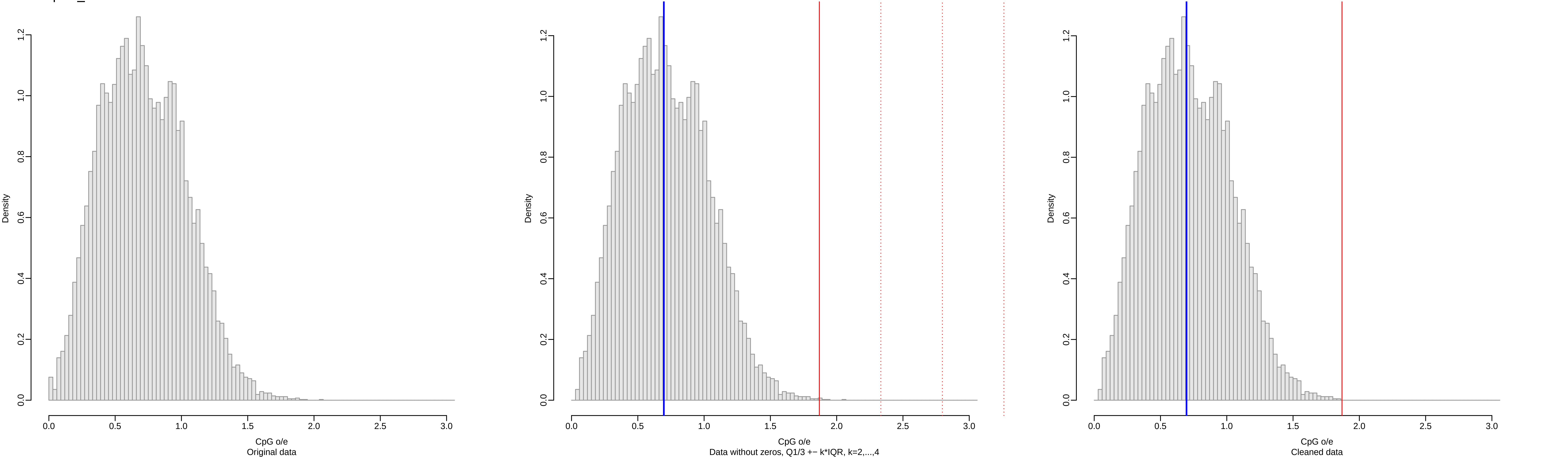

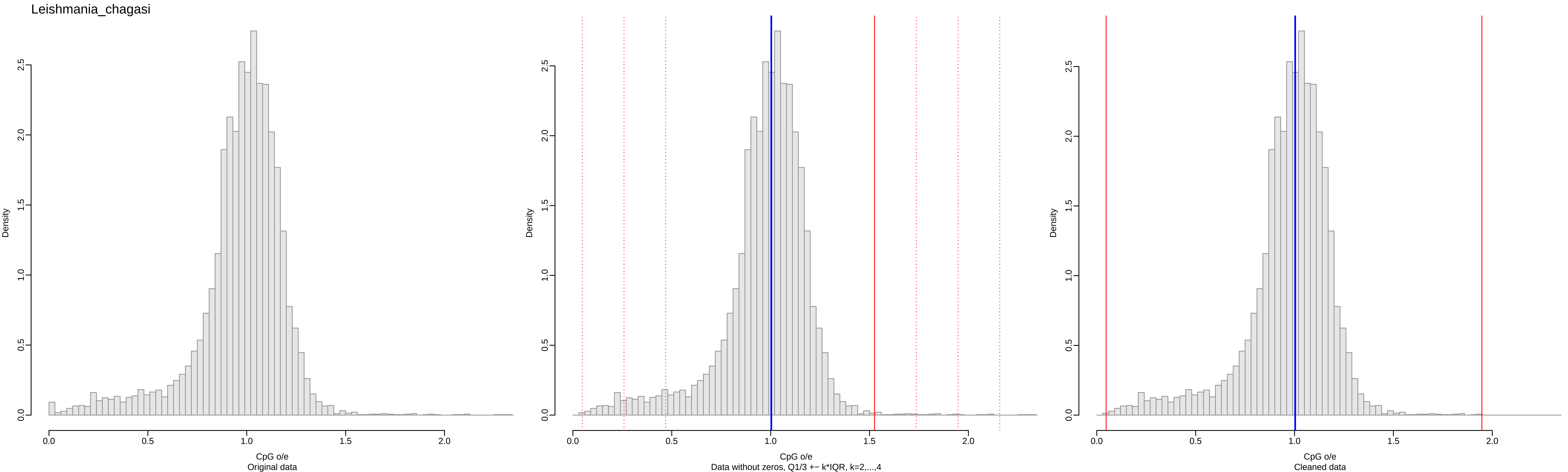

Leishmania_chagasi
Density
CpG o/e
Original data
Data without zeros, Q1/3 +− k*IQR, k=2,...,4
Cleaned data
0.0
0.5
1.0
1.5
2.0
2.5

Leishmania_major

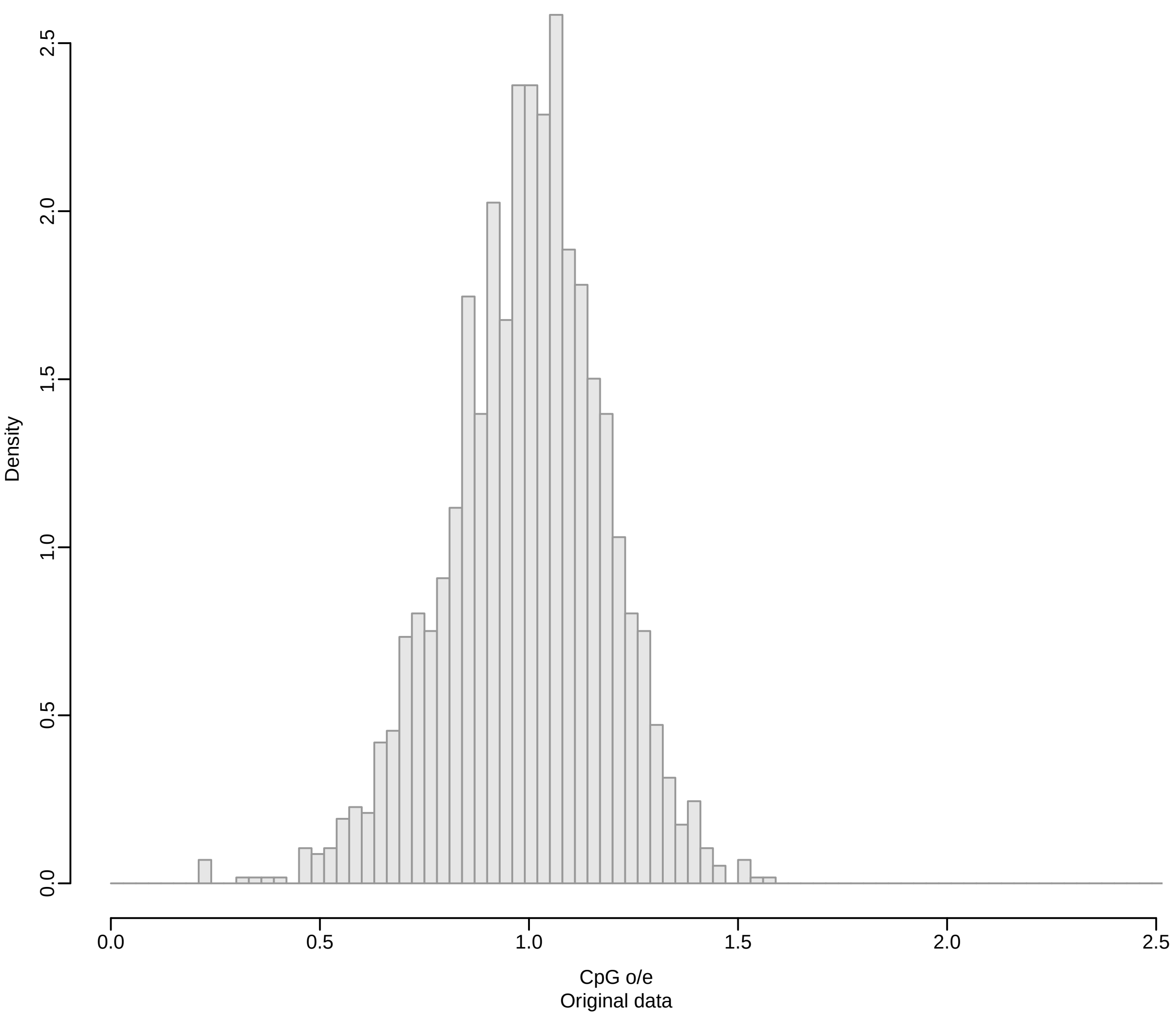

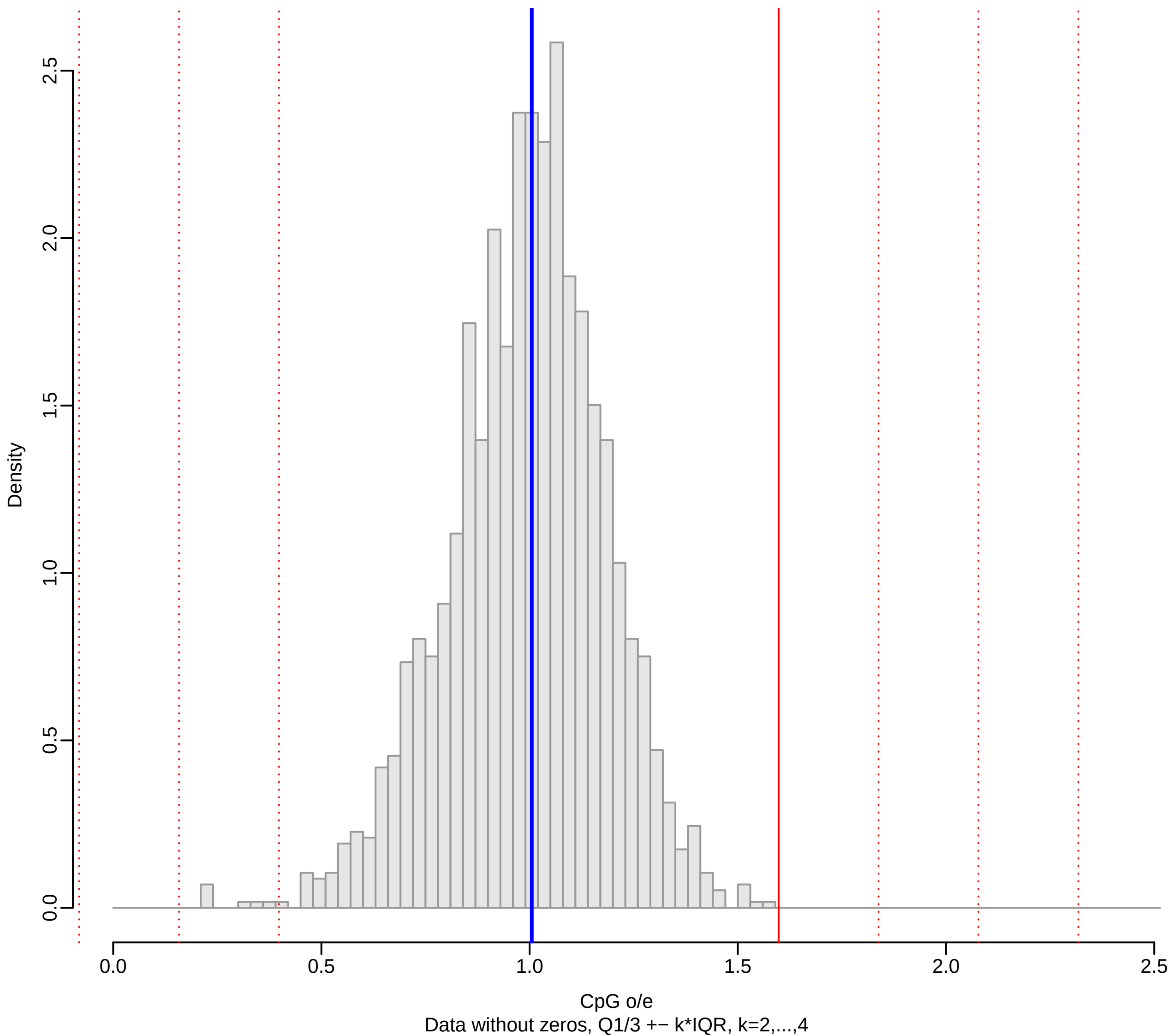

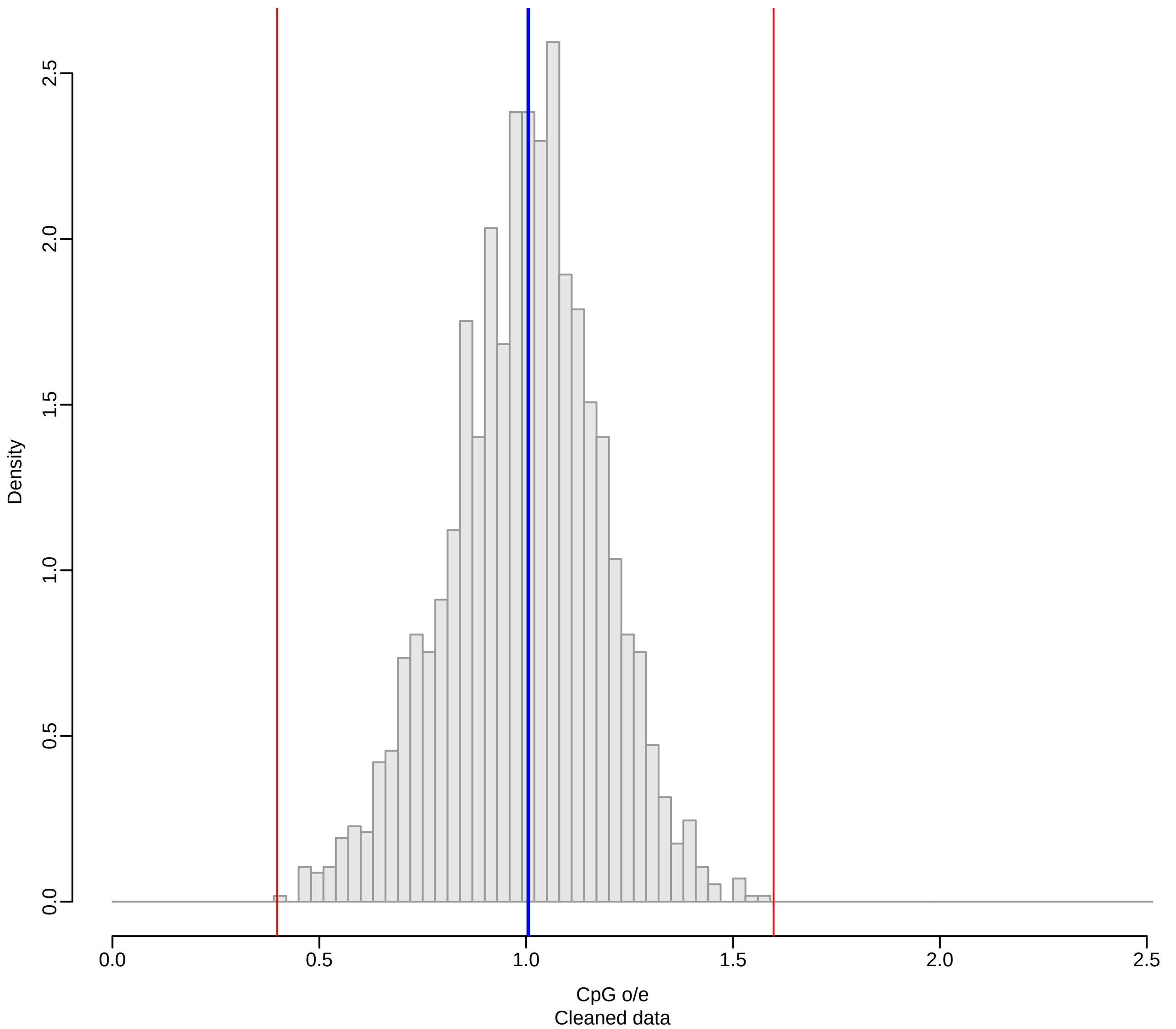

Lepeophtheirus_salmonis

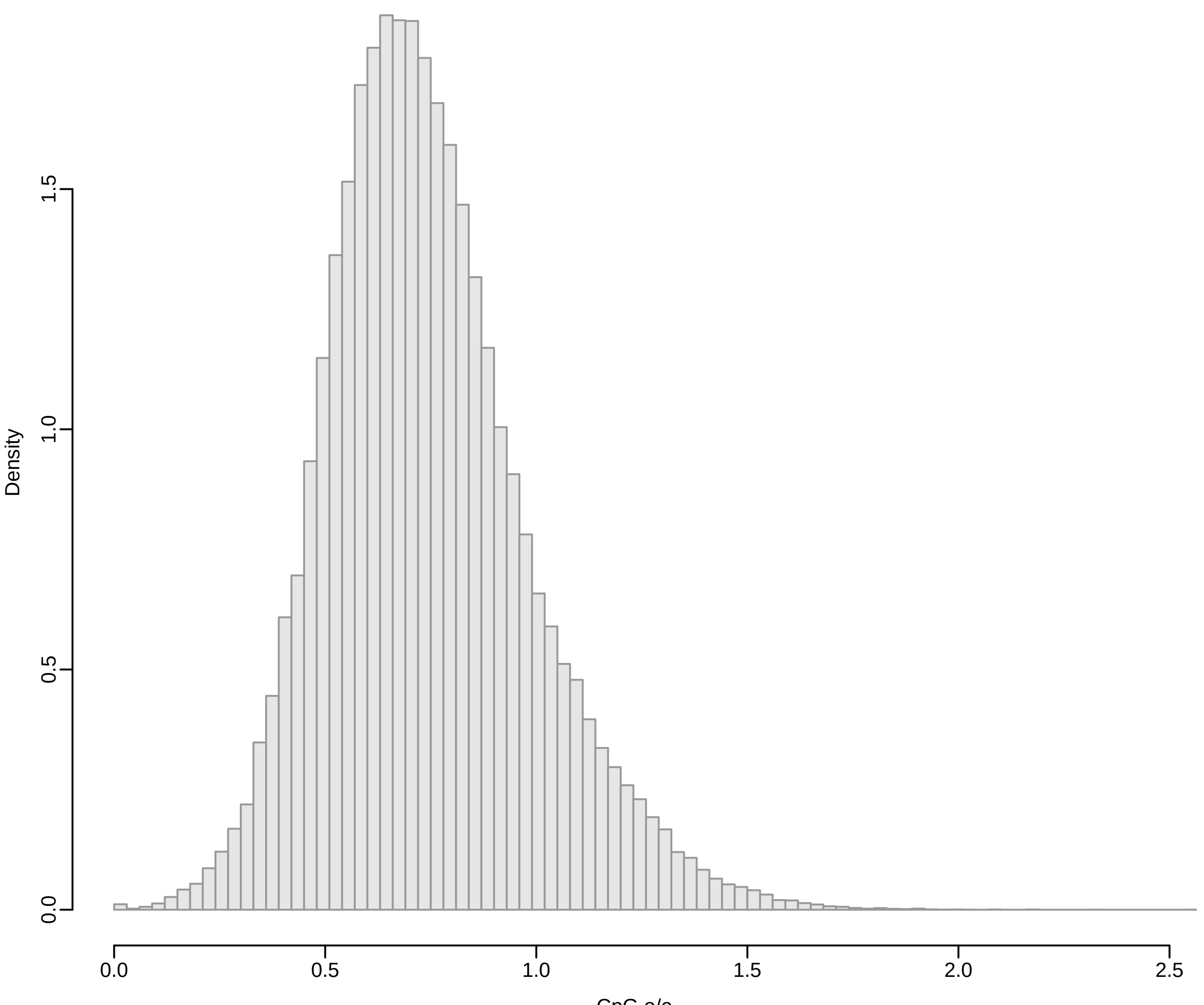

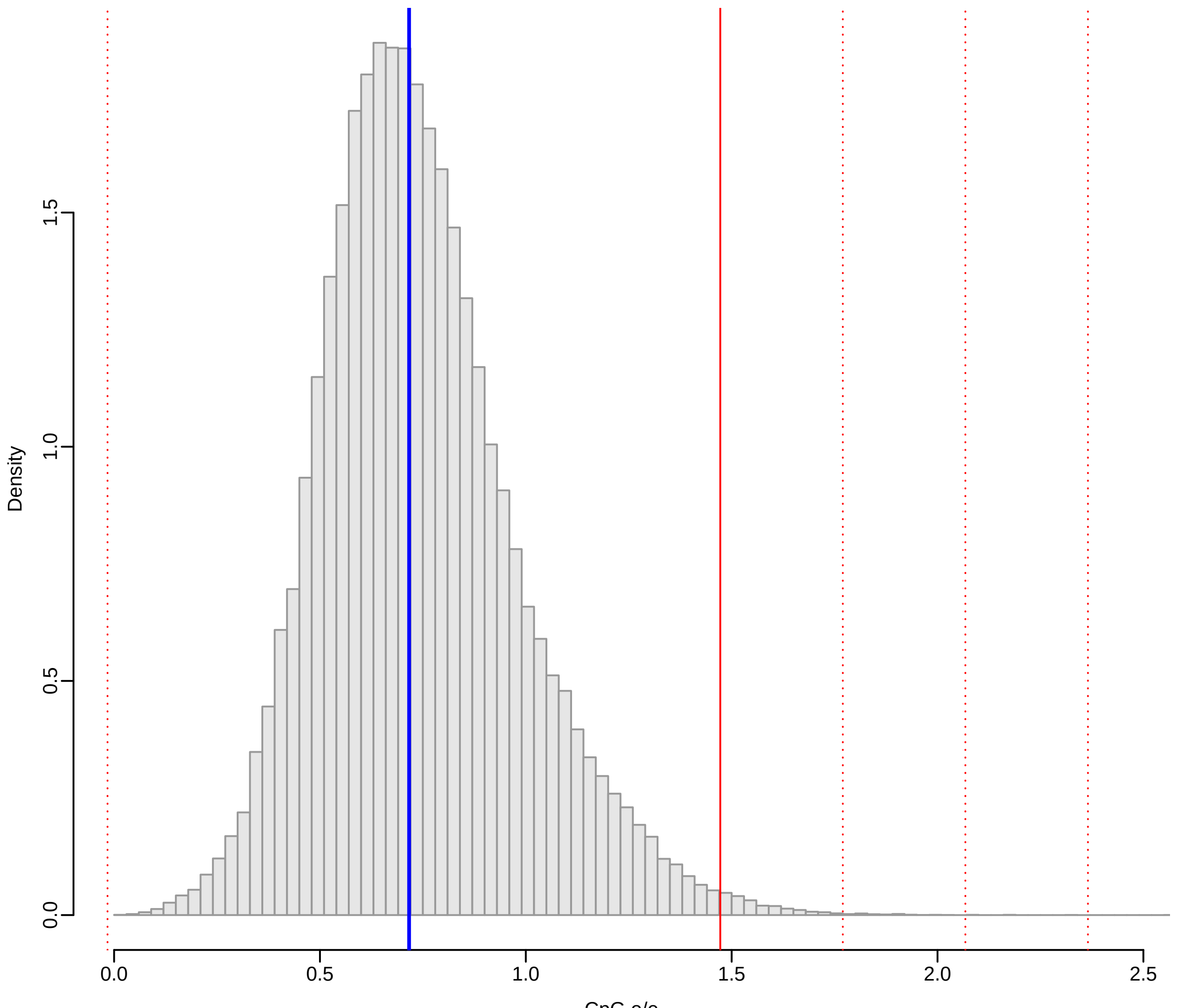

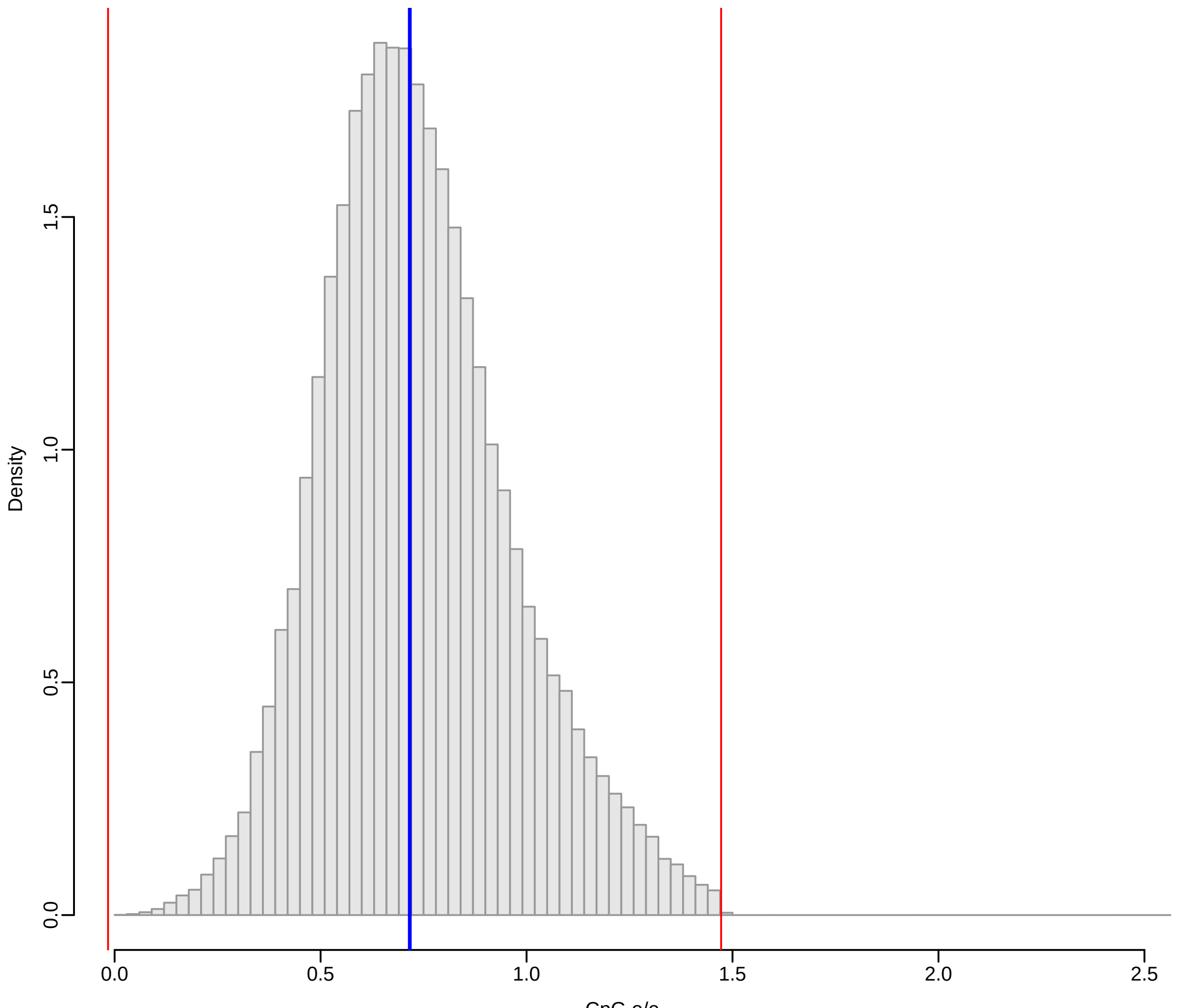

Lepismachilis_y−signata

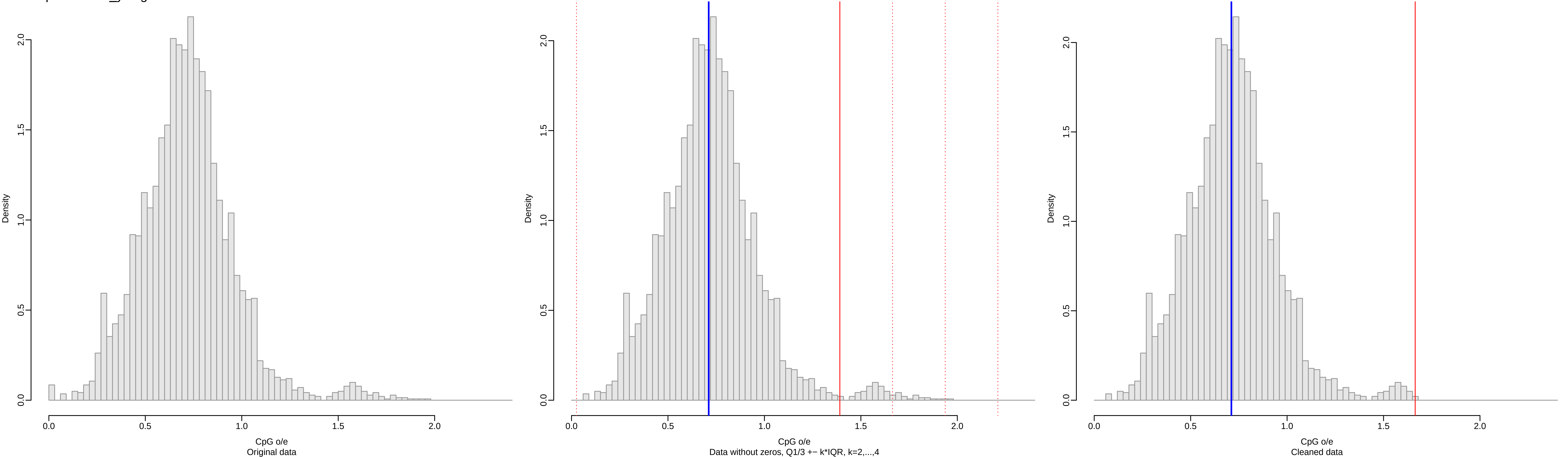

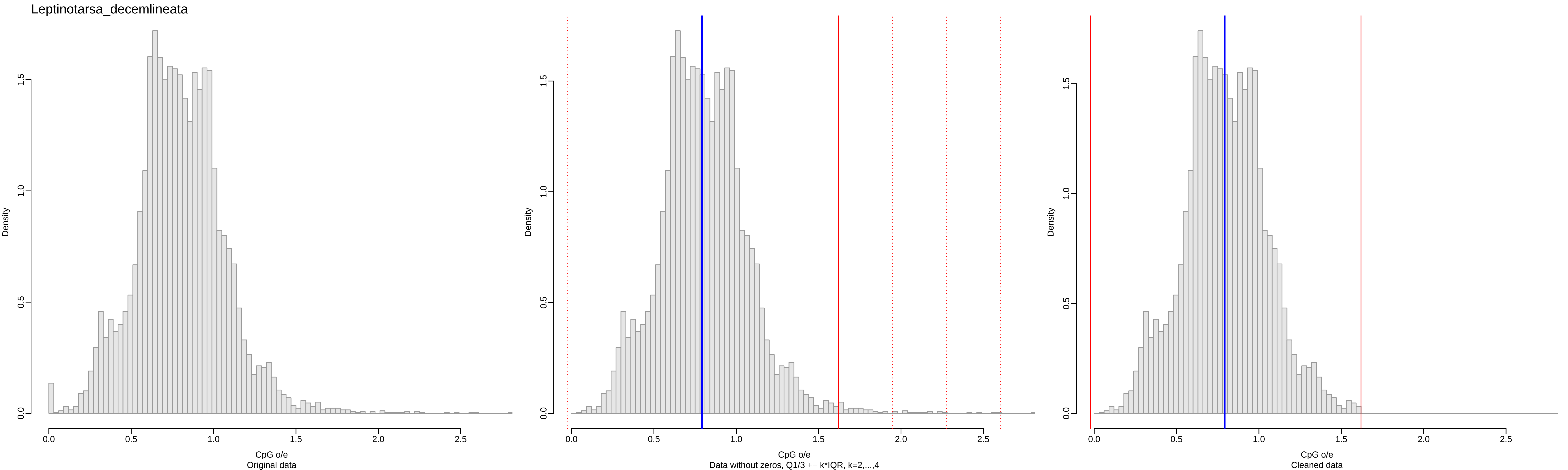
Leptinotarsa_decemlineata
Density
CpG o/e
Original data
Data without zeros, Q1/3 +− k*IQR, k=2,...,4
Cleaned data
0.0
0.5
1.0
1.5
2.0
2.5

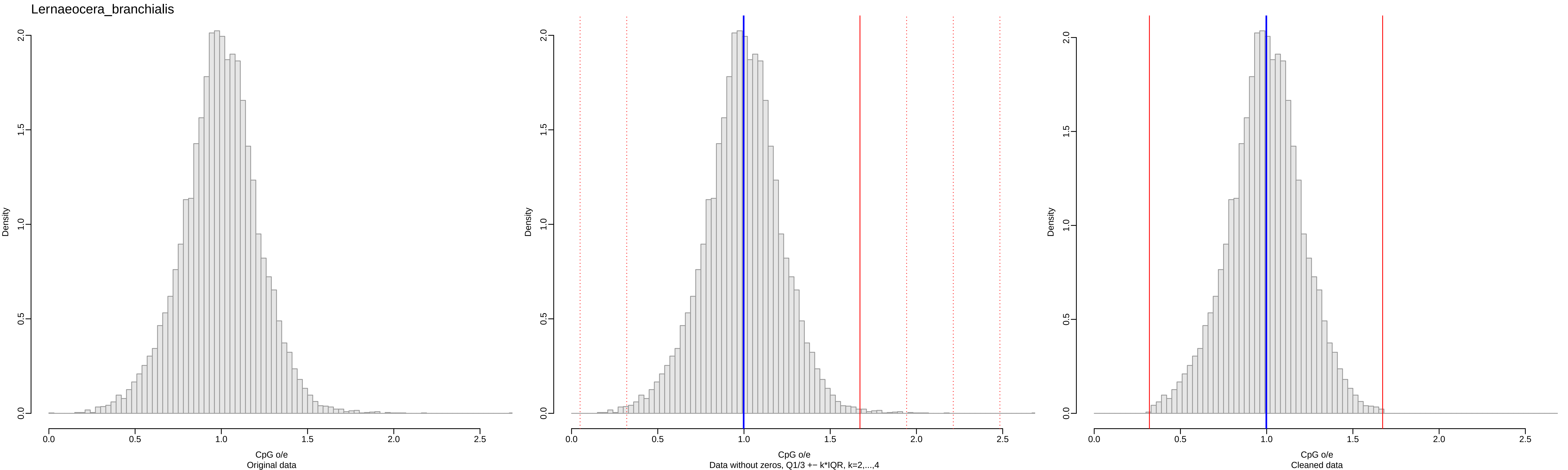
Lernaeocera_branchialis
Density
CpG o/e
Original data
Data without zeros, Q1/3 +− k*IQR, k=2,...,4
Cleaned data

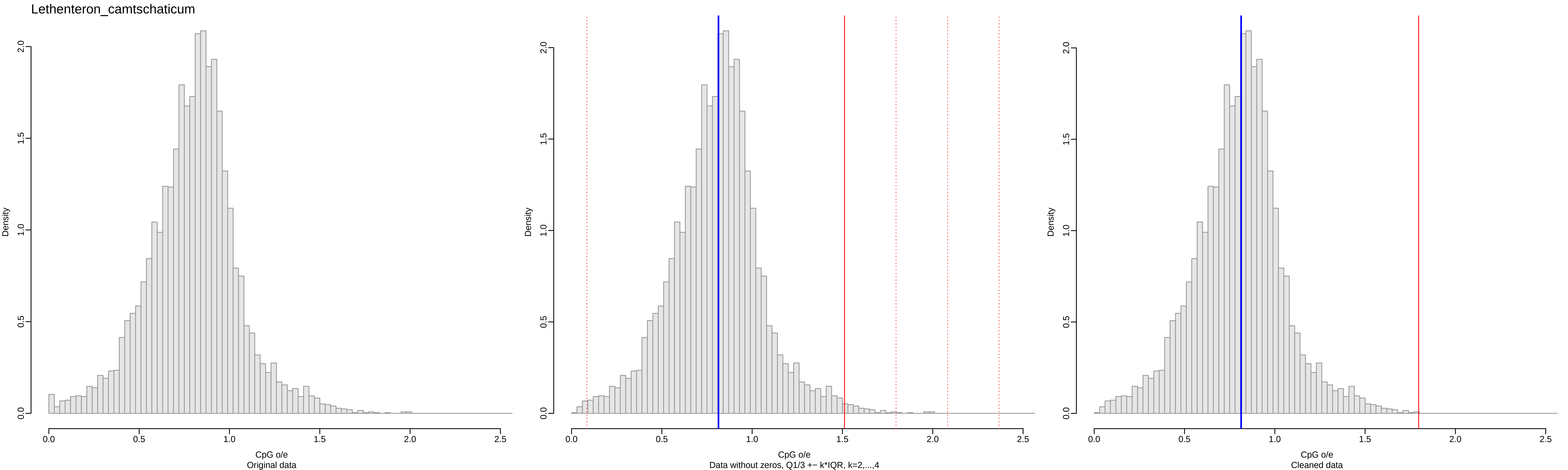
Lethenteron_camtschaticum
Density
CpG o/e
Original data
Data without zeros, Q1/3 +− k*IQR, k=2,...,4
Cleaned data
0.0
0.5
1.0
1.5
2.0
2.5

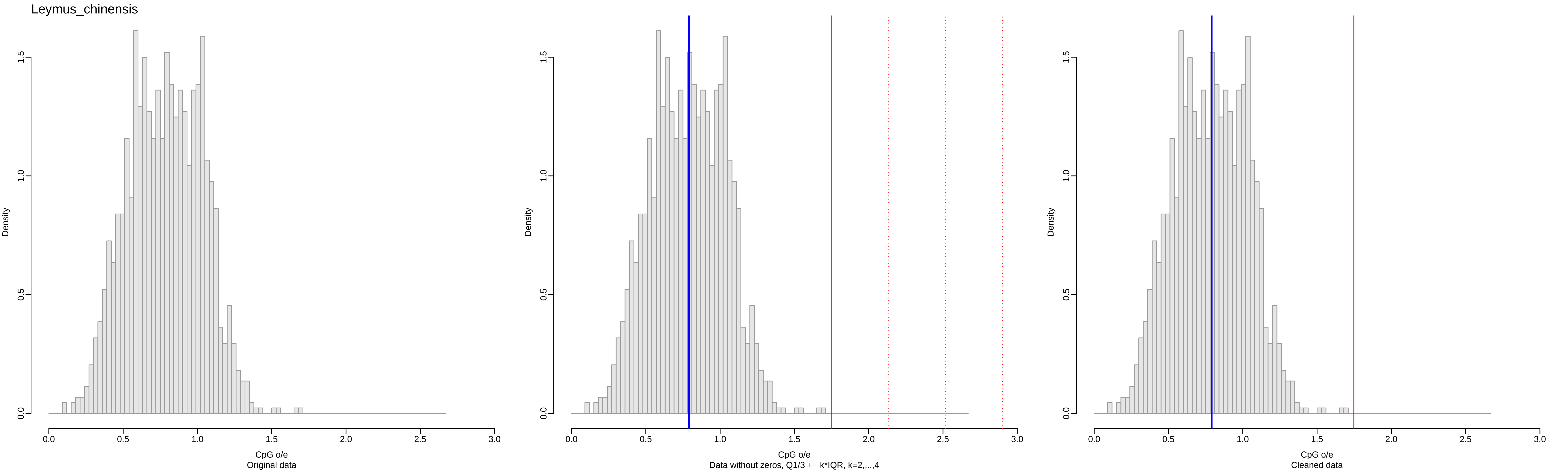

Leymus_chinensis
Density
CpG o/e
Original data
Data without zeros, Q1/3 +− k*IQR, k=2,...,4
Cleaned data
0.0
0.5
1.0
1.5
2.0
2.5
3.0

Limnanthes_alba

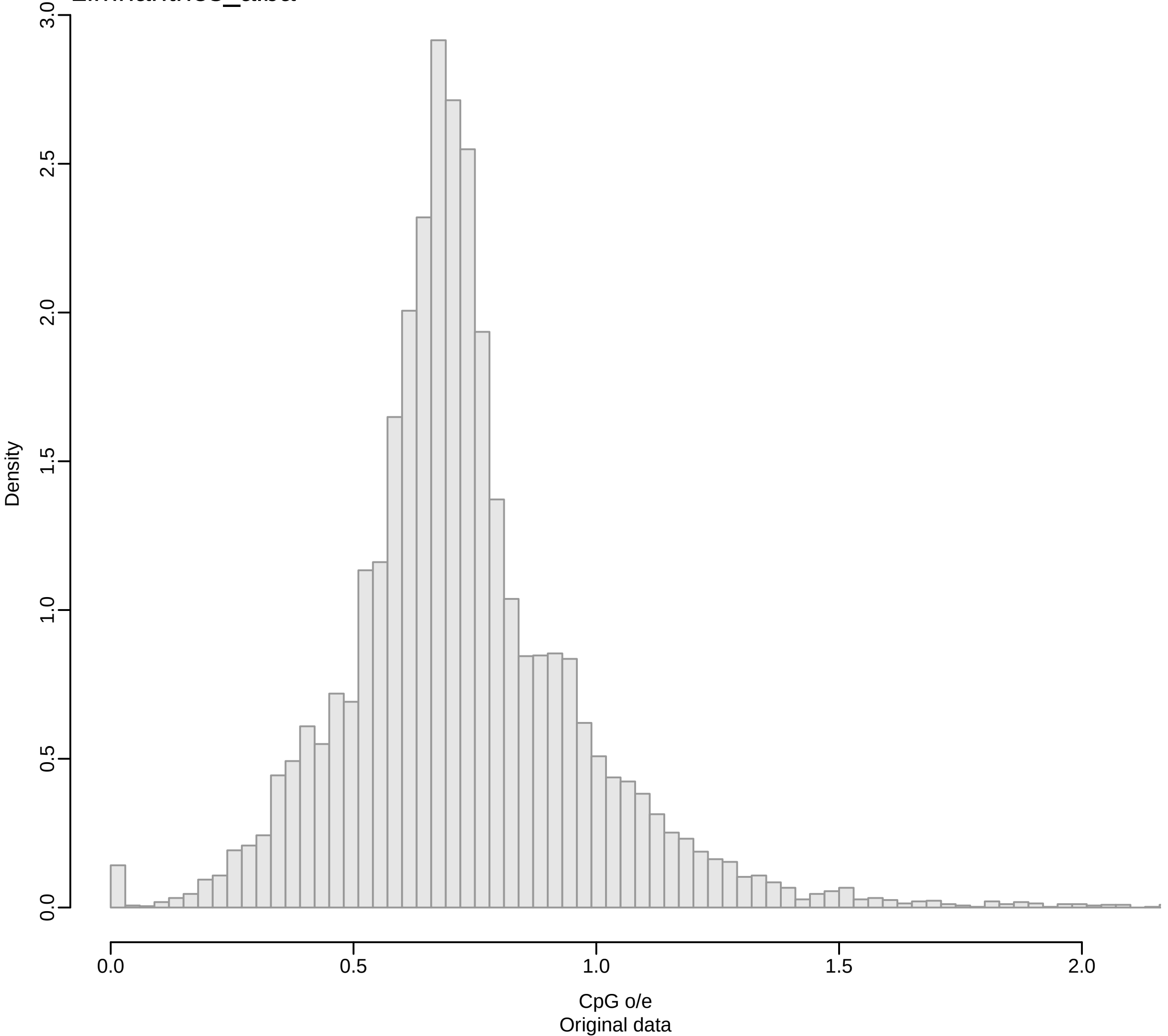

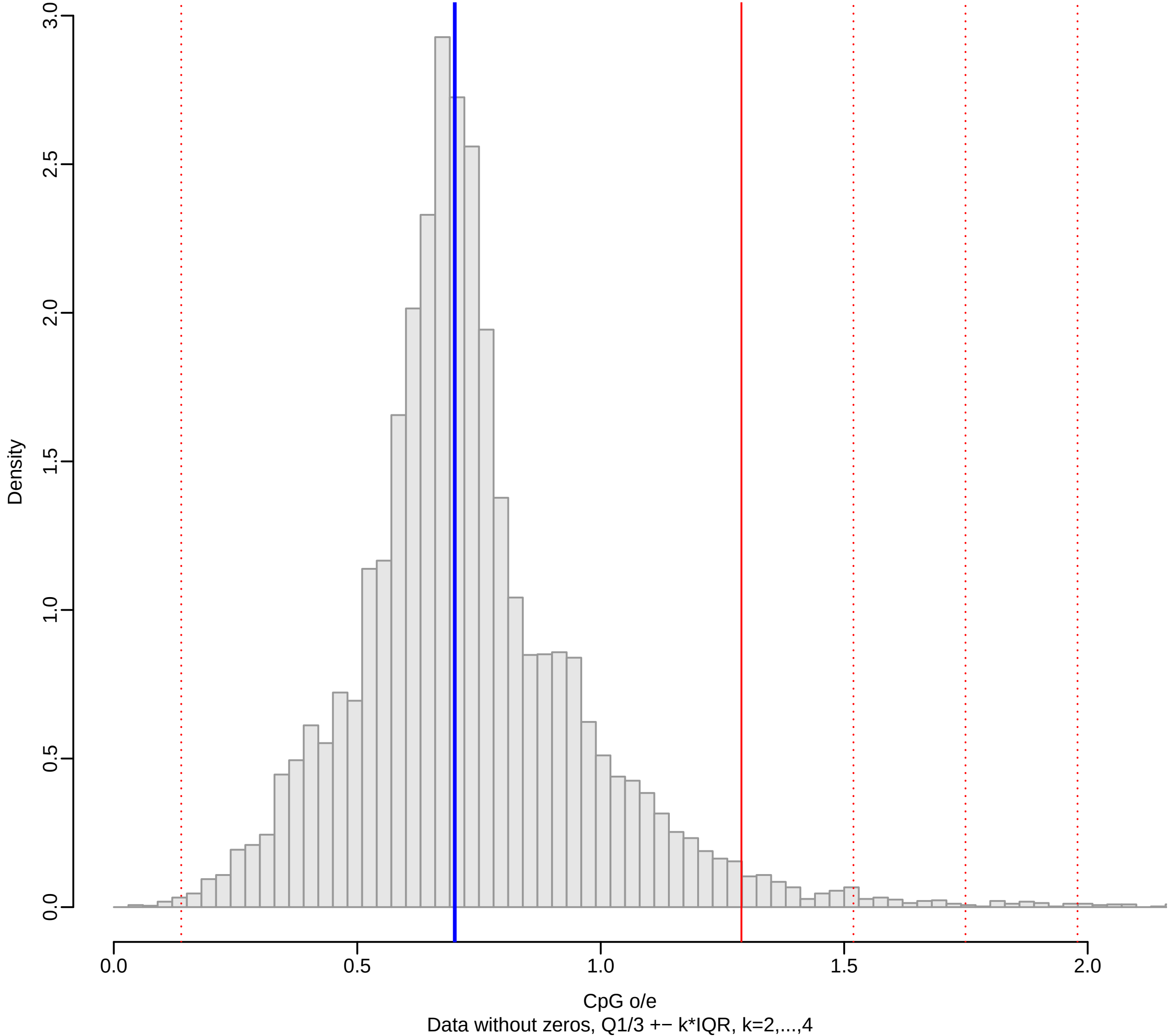

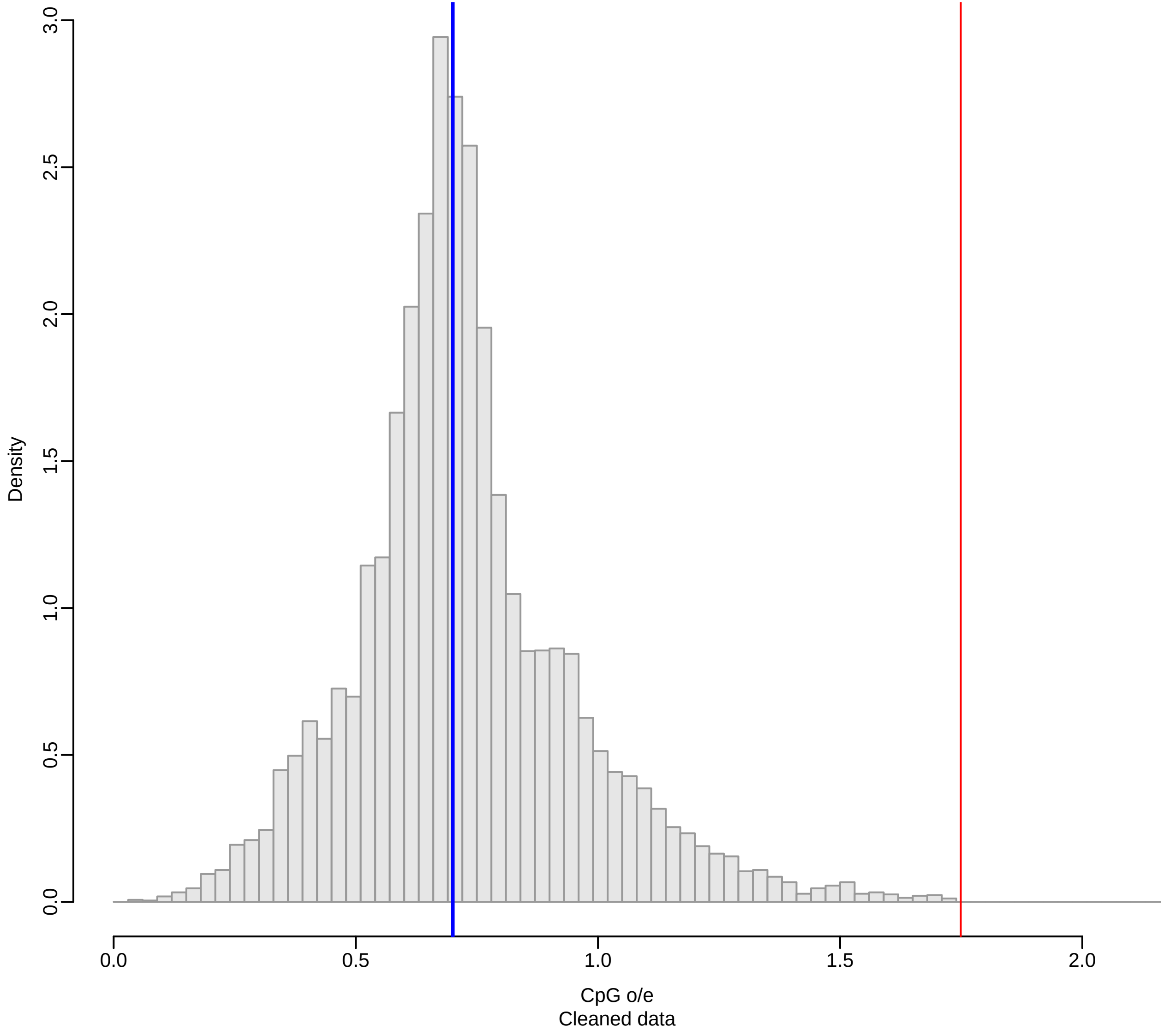

Limonium_bicolor

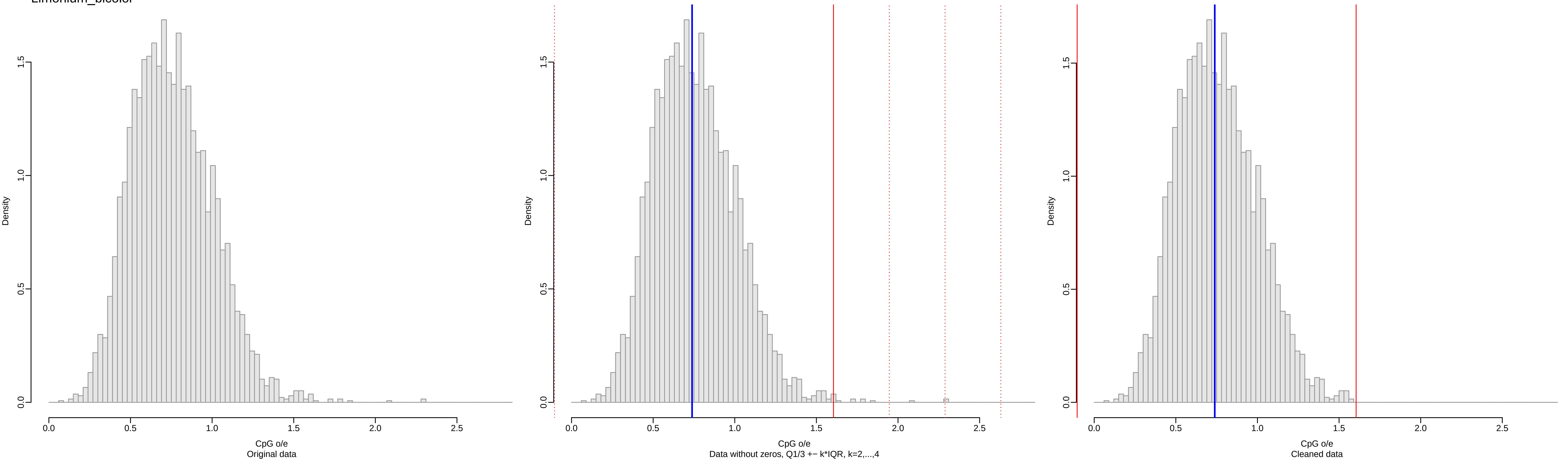

Limulus_polyphemus

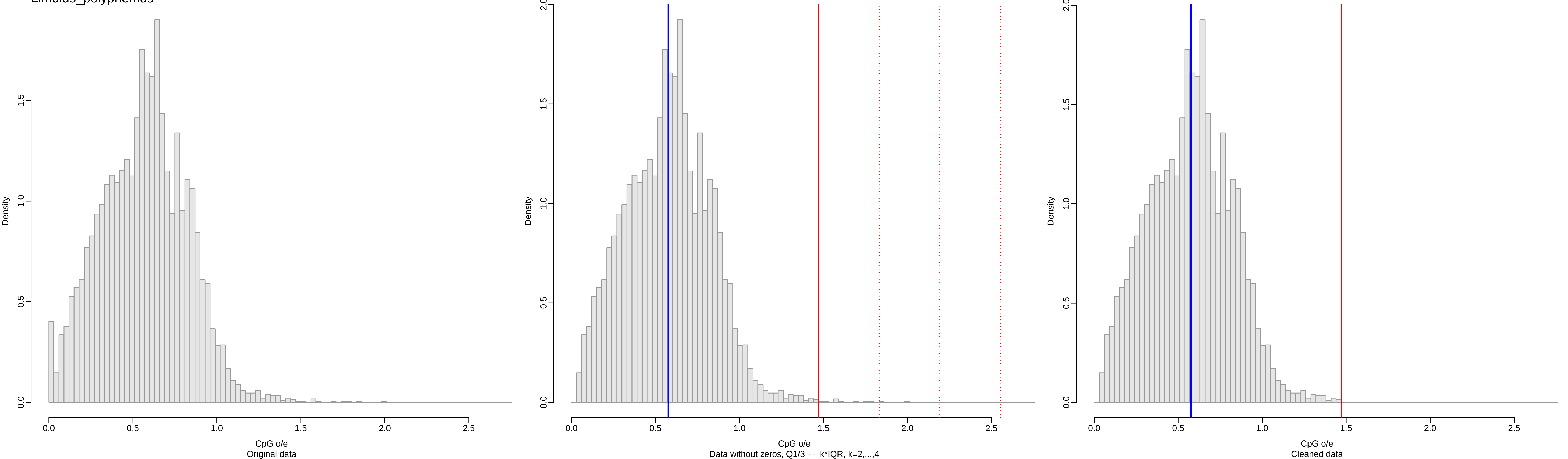

Lithognathus_mormyrus

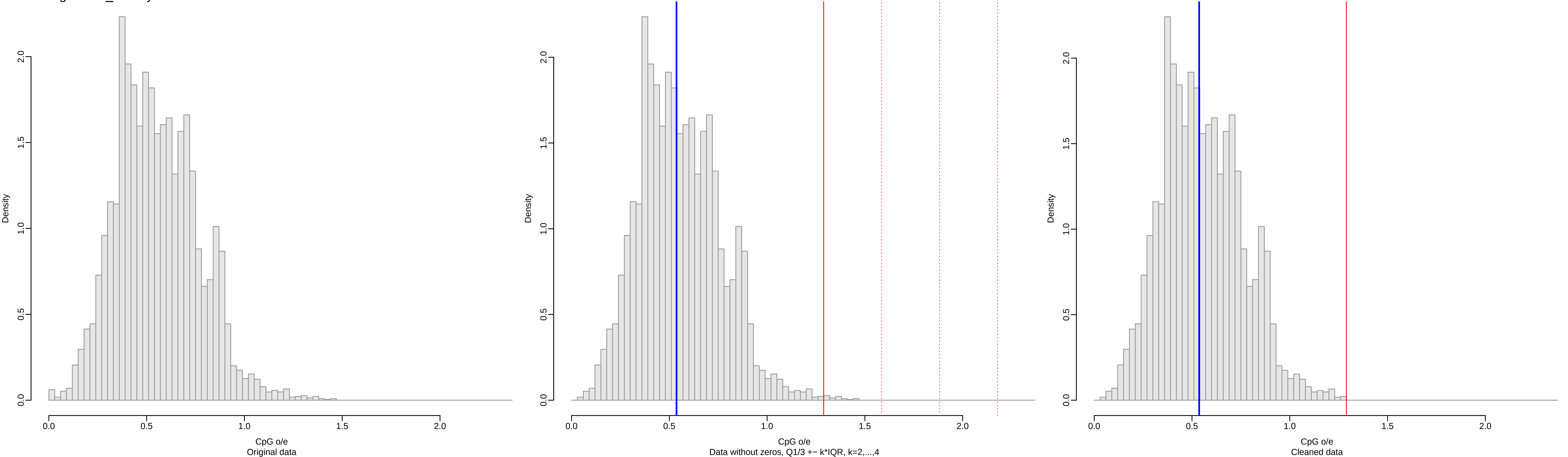

Litopenaeus_vannamei

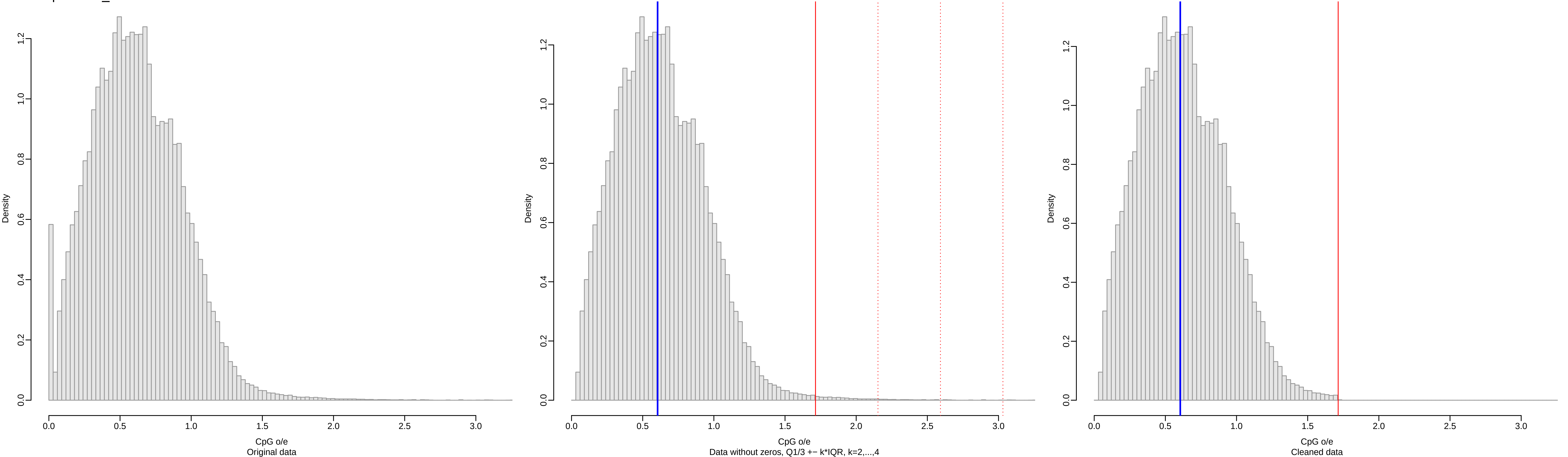

Littorina_saxatilis

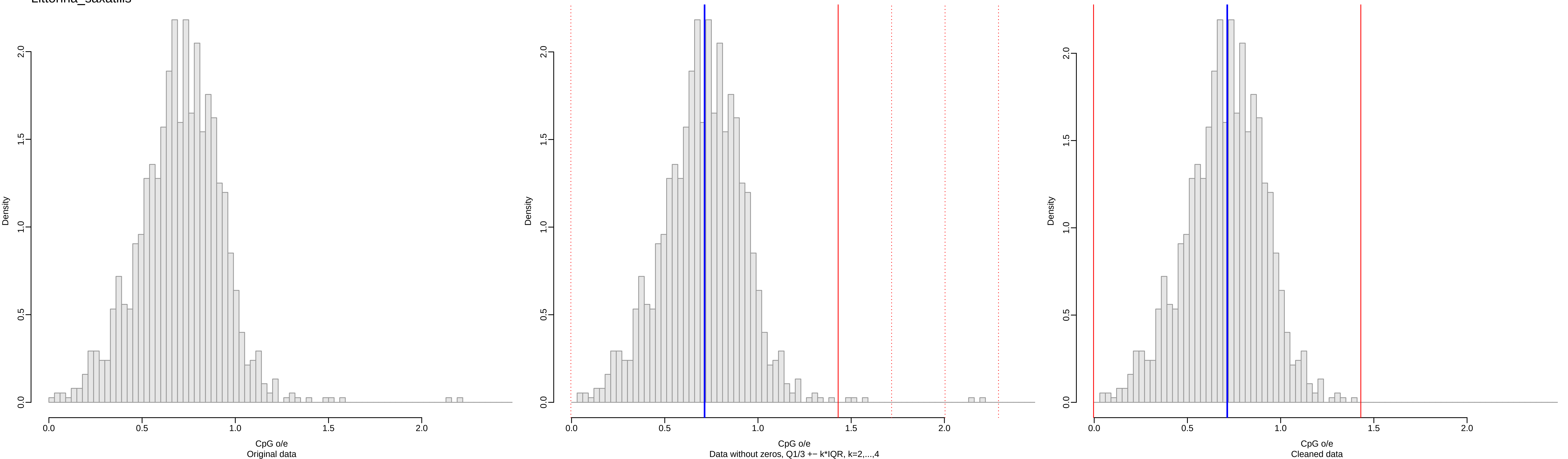

Loa_loa

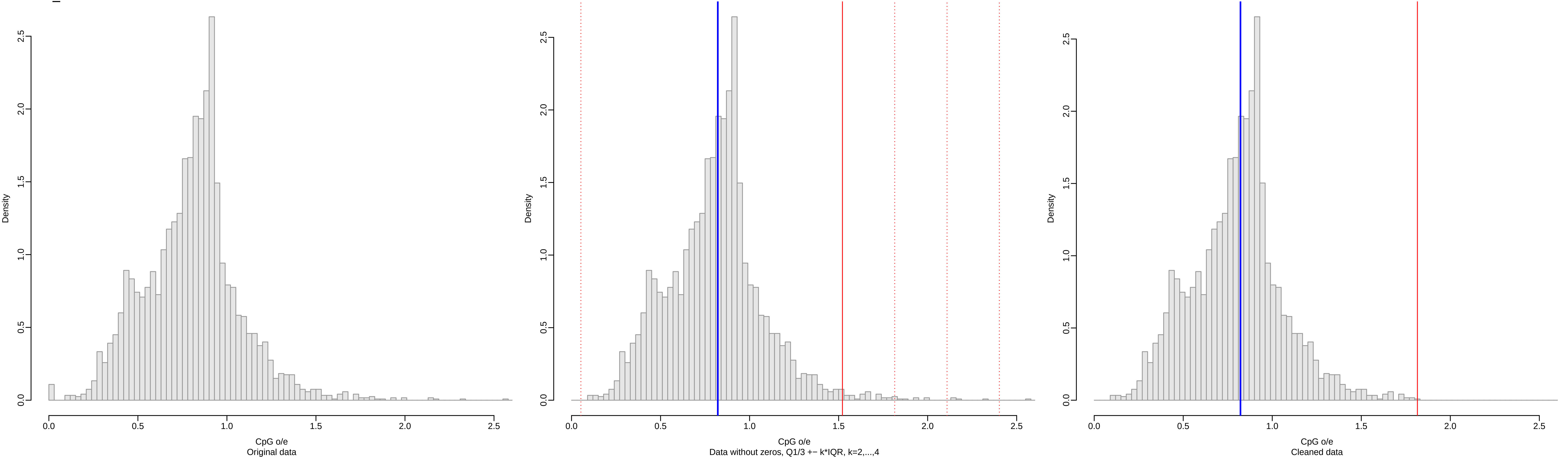
Density
CpG o/e
Original data
Density
CpG o/e
Data without zeros, Q1/3 +− k*IQR, k=2,...,4
Density
CpG o/e
Cleaned data

Locusta_migratoria

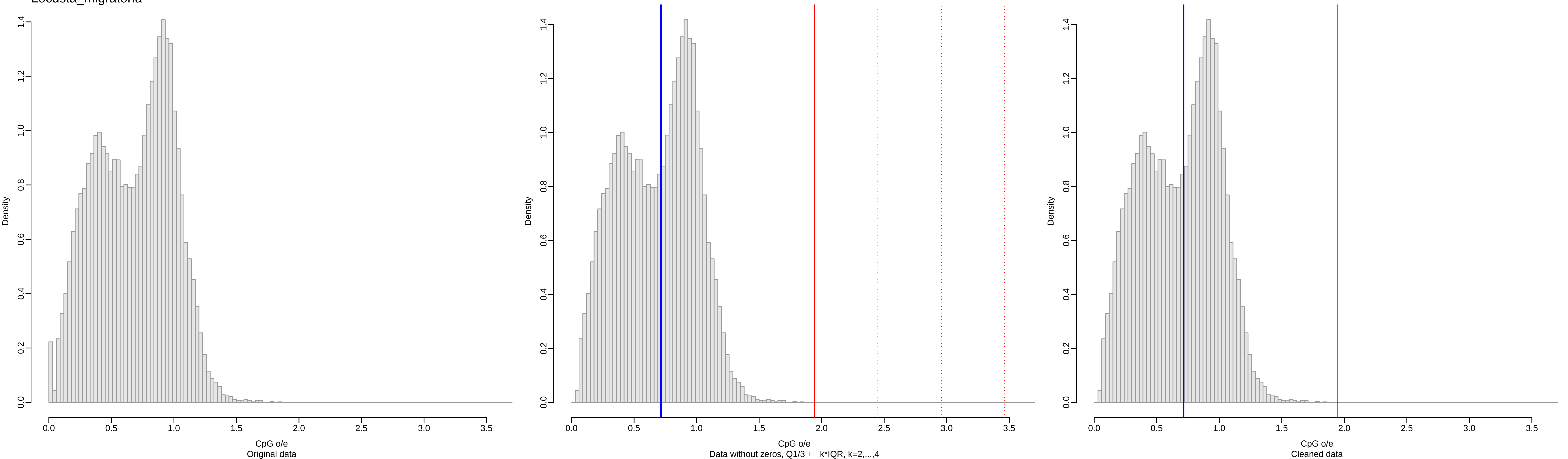

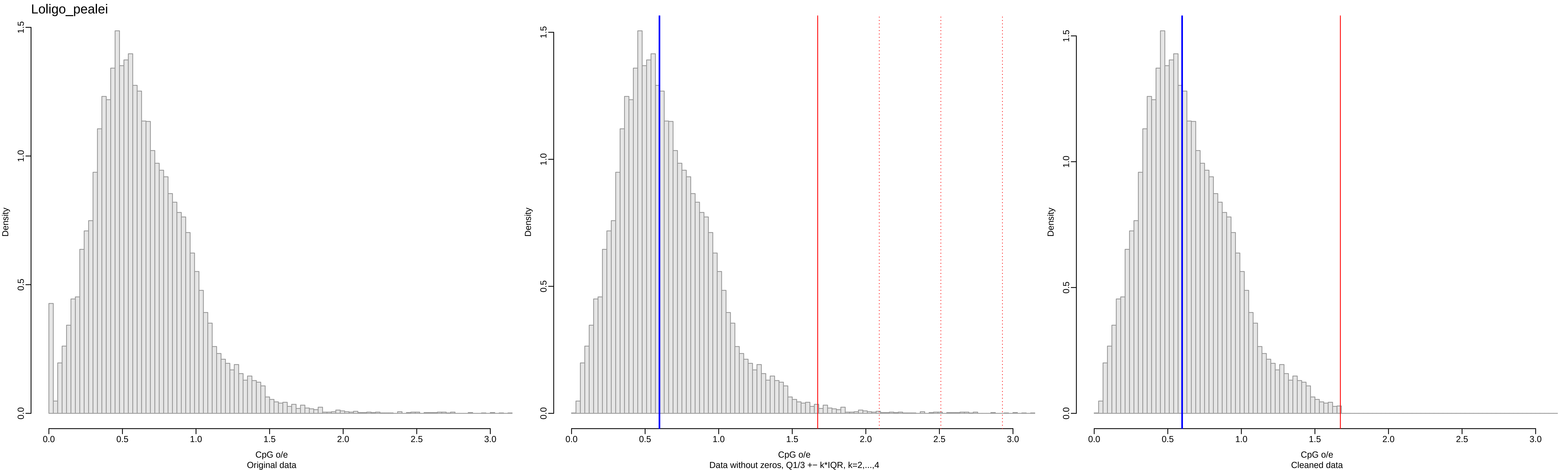

Loligo_pealei
Density
CpG o/e
Original data
Data without zeros, Q1/3 +− k*IQR, k=2,...,4
Cleaned data
0.0
0.5
1.0
1.5
2.0
2.5
3.0

Lolium_perenne

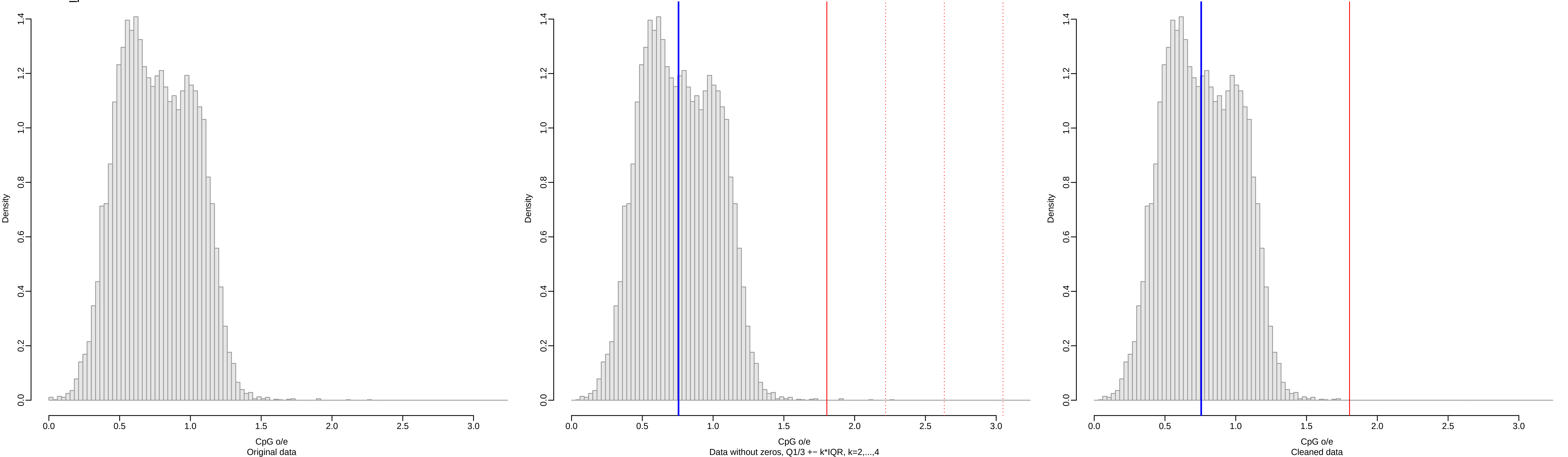

Lottia_gigantea

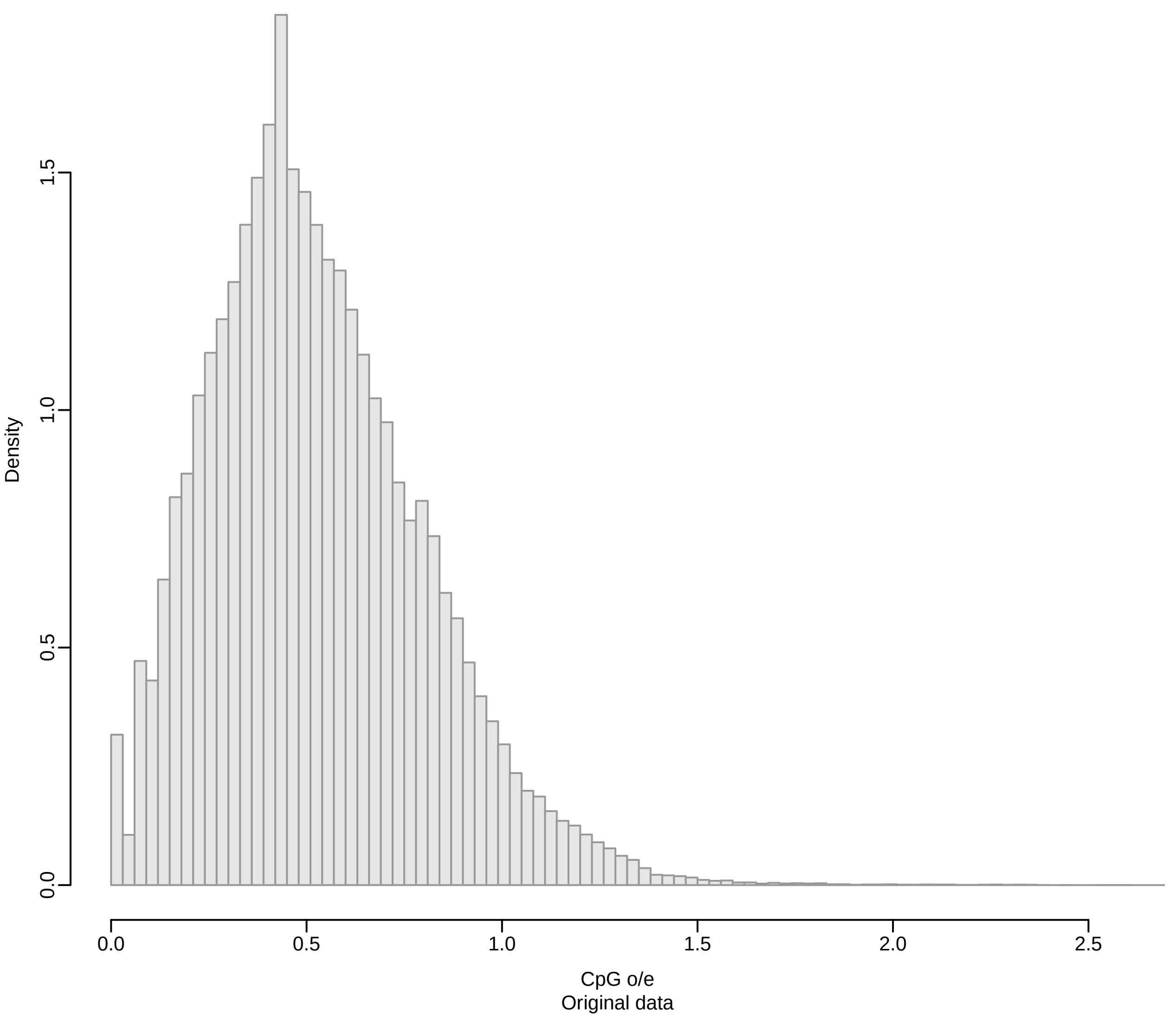

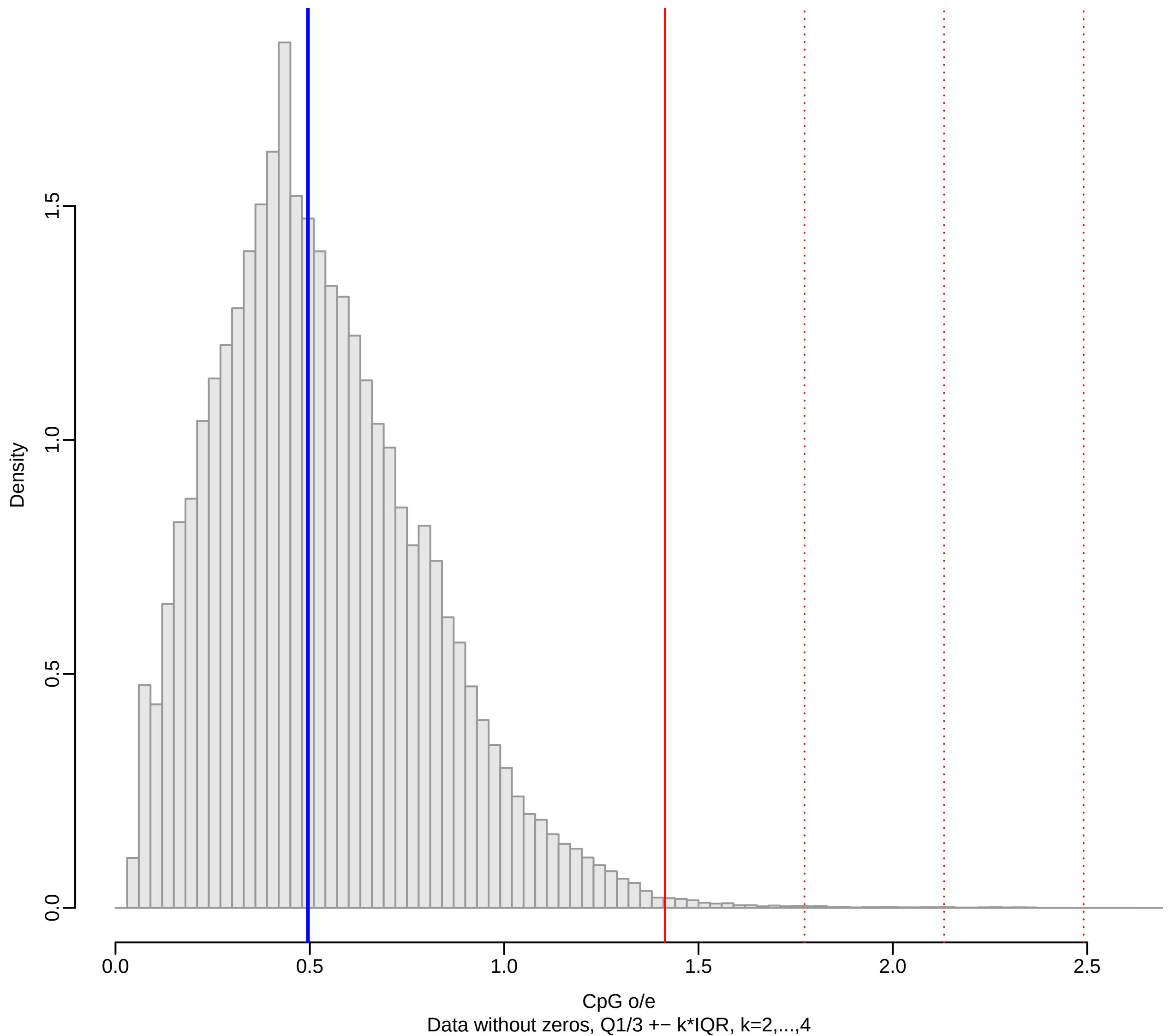

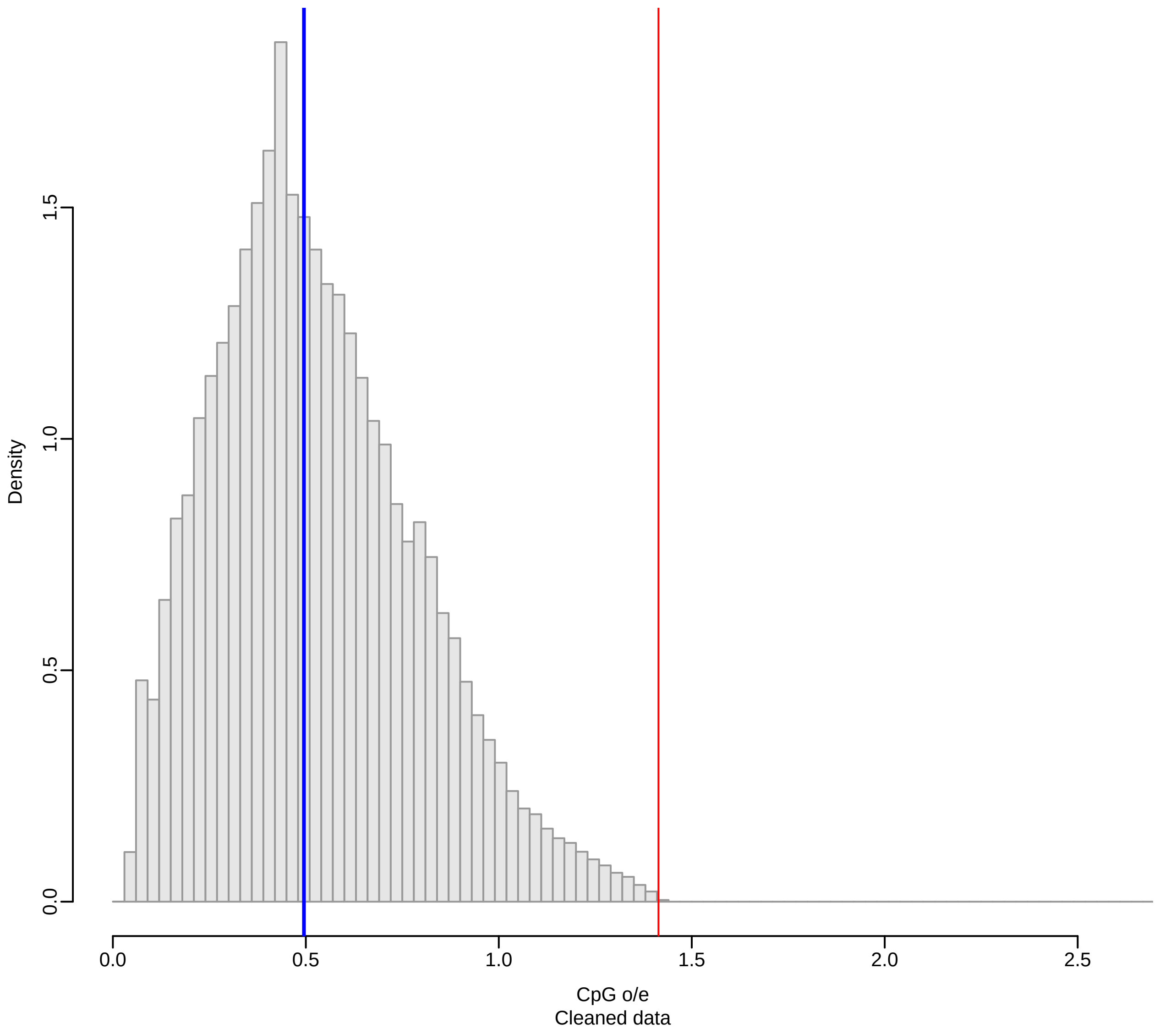

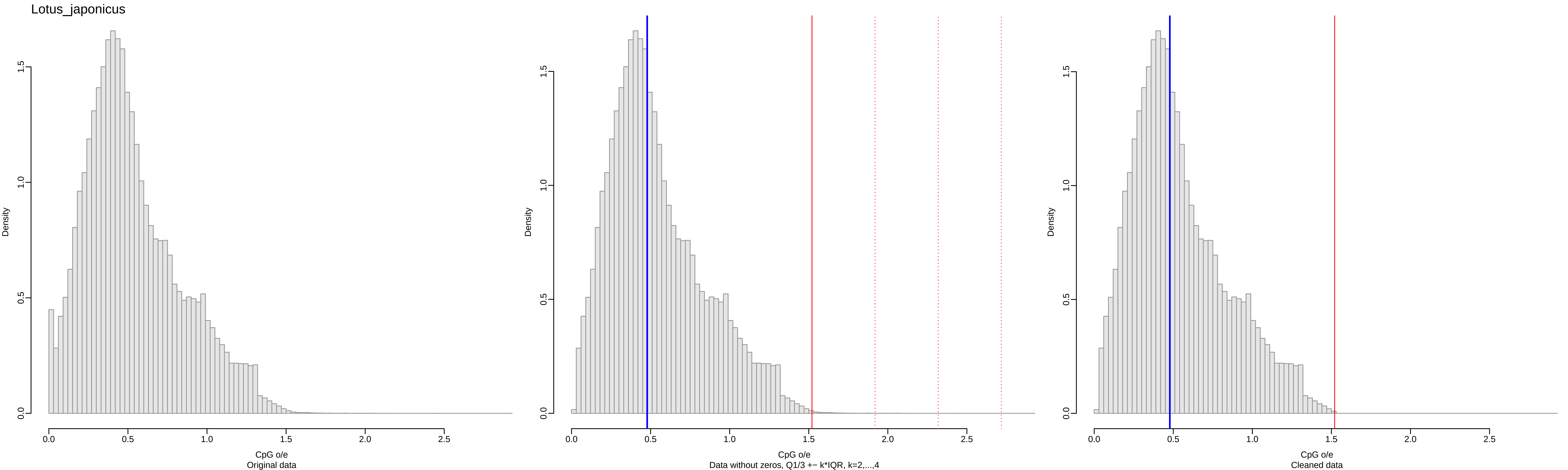

Lotus_japonicus
Density
0.0
0.5
1.0
1.5
2.0
2.5
CpG o/e
Original data
Data without zeros, Q1/3 +− k*IQR, k=2,...,4
Cleaned data

Lumbricus_rubellus

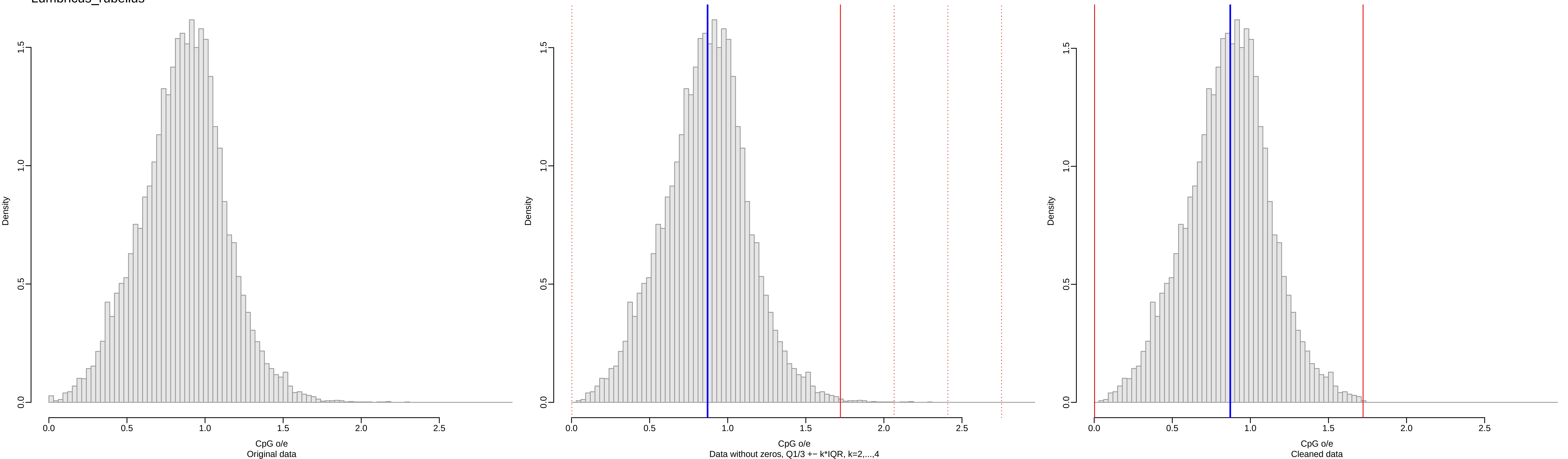

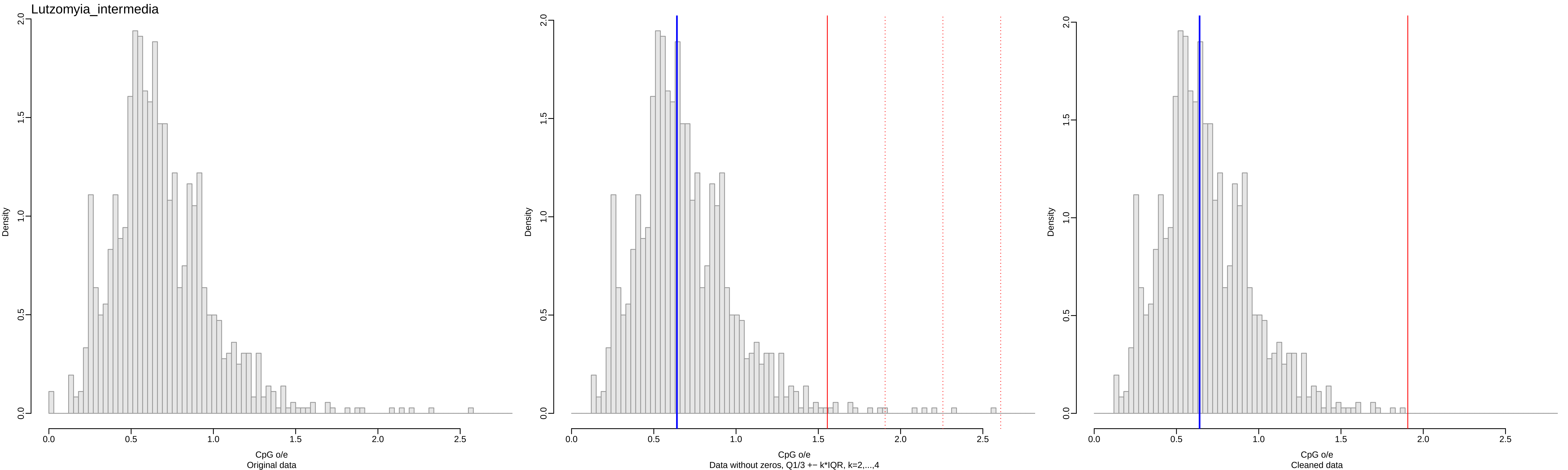
Lutzomyia_intermedia
Density
CpG o/e
Original data
Data without zeros, Q1/3 +− k*IQR, k=2,...,4
Cleaned data
0.0
0.5
1.0
1.5
2.0
2.5

Lutzomyia_longipalpis

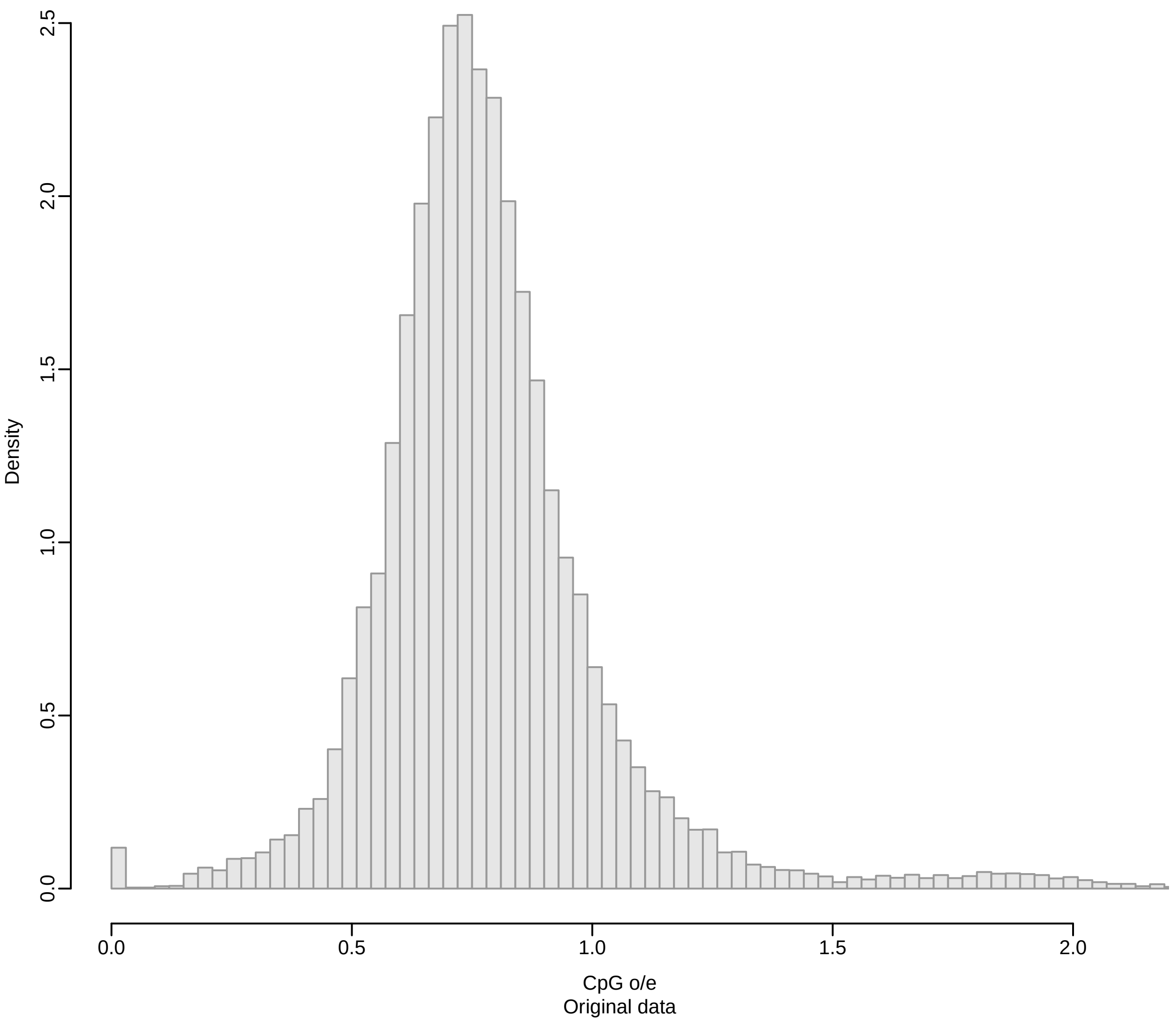

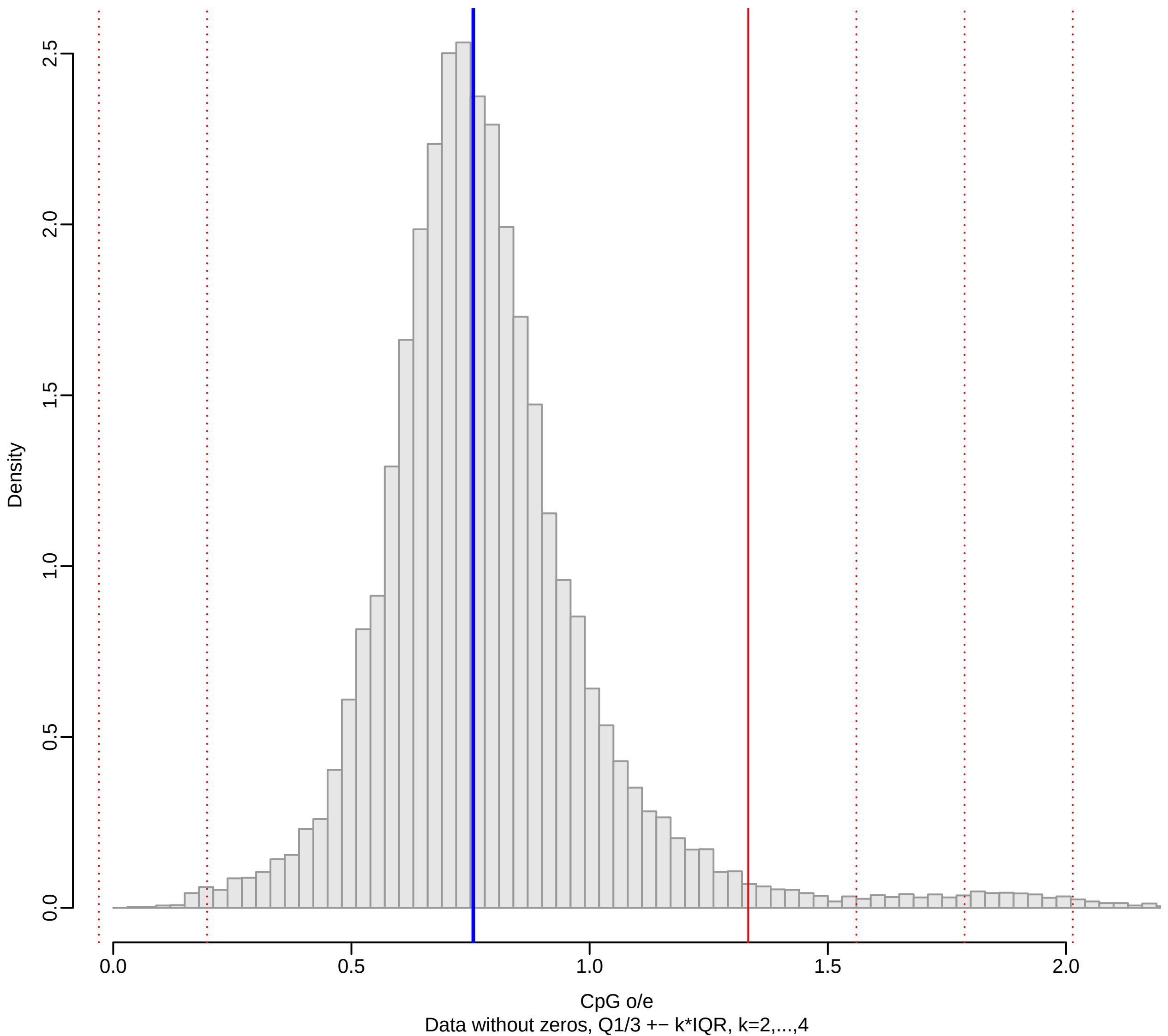

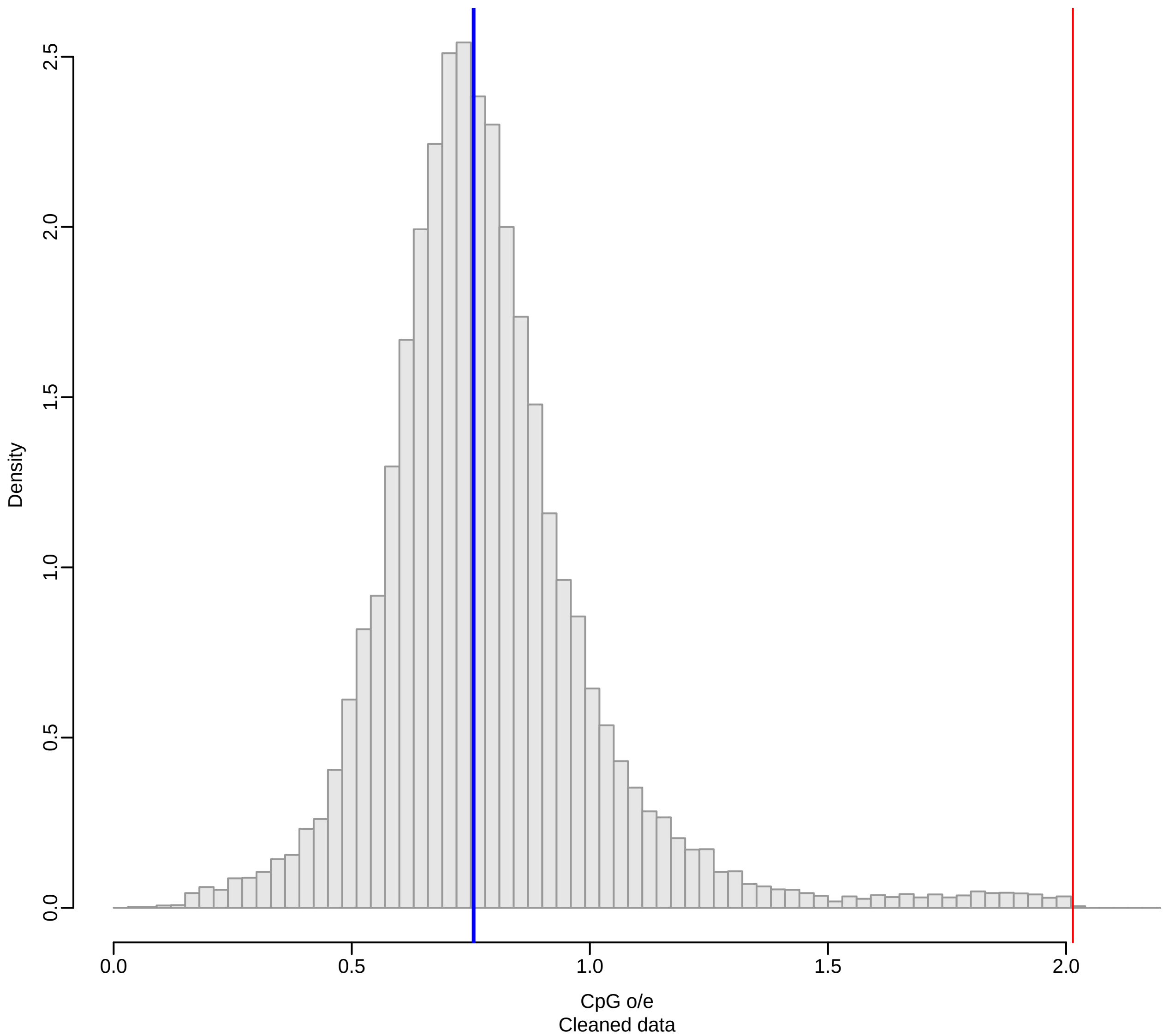

Lutzomyia_umbratilis

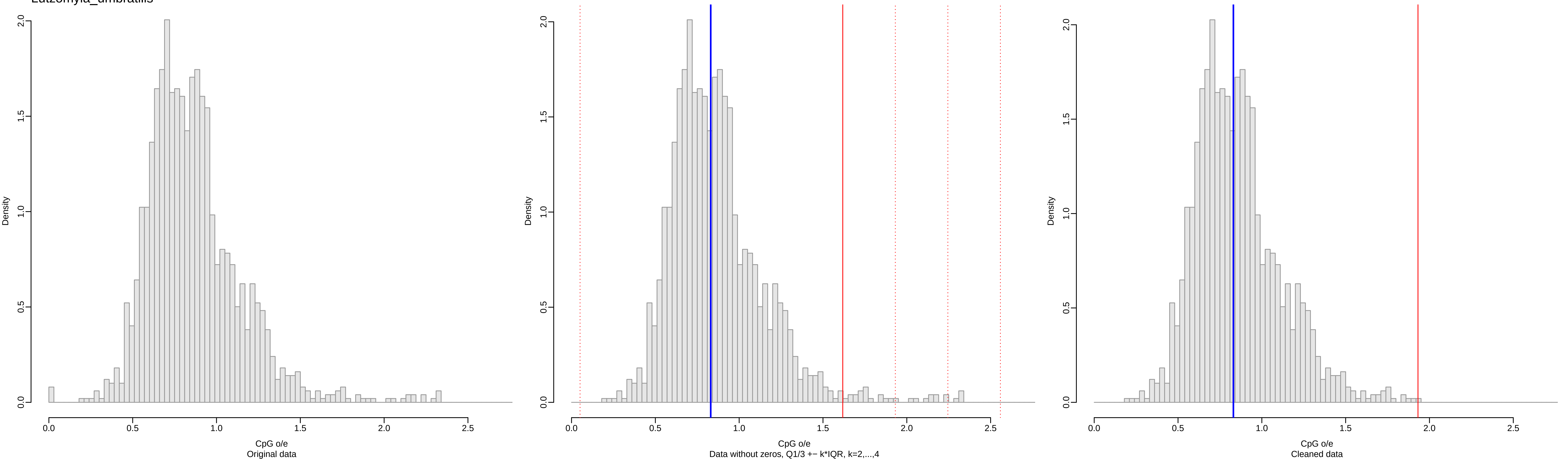

Lymnaea_stagnalis

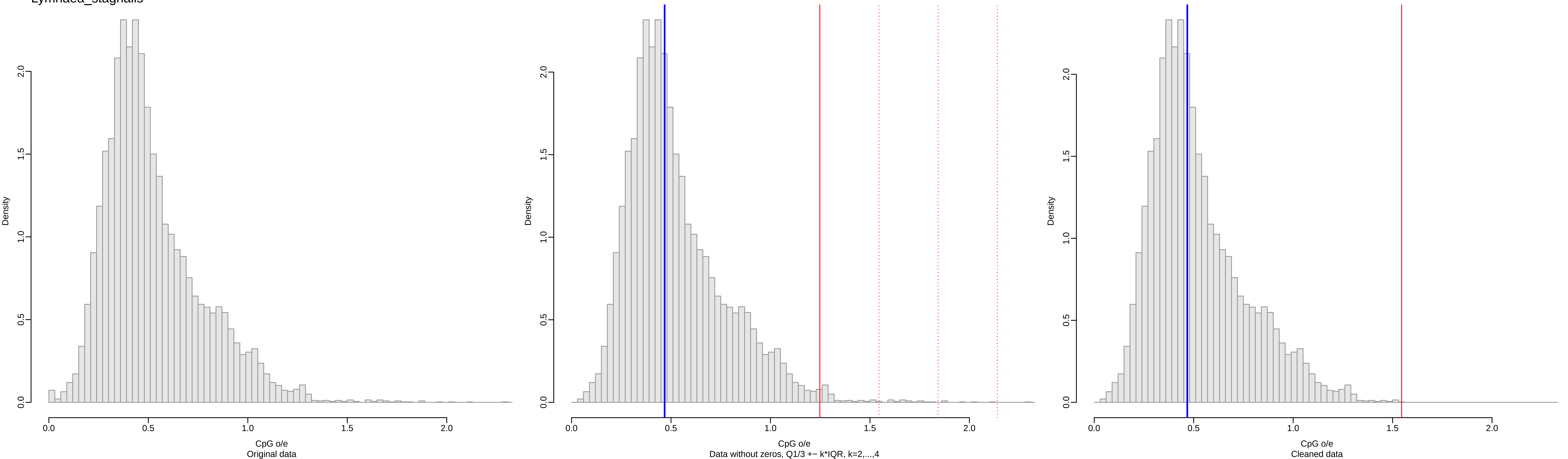

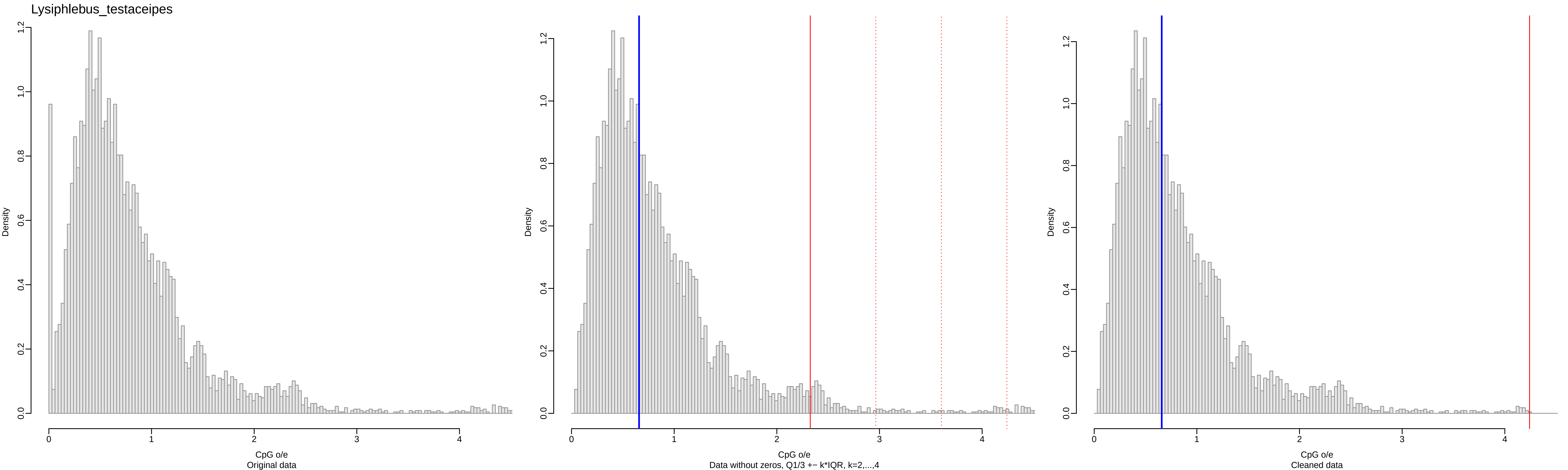
Lysiphlebus_testaceipes
Density
CpG o/e
Original data
Data without zeros, Q1/3 +− k*IQR, k=2,...,4
Cleaned data
0.0
0.2
0.4
0.6
0.8
1.0
1.2
0
1
2
3
4

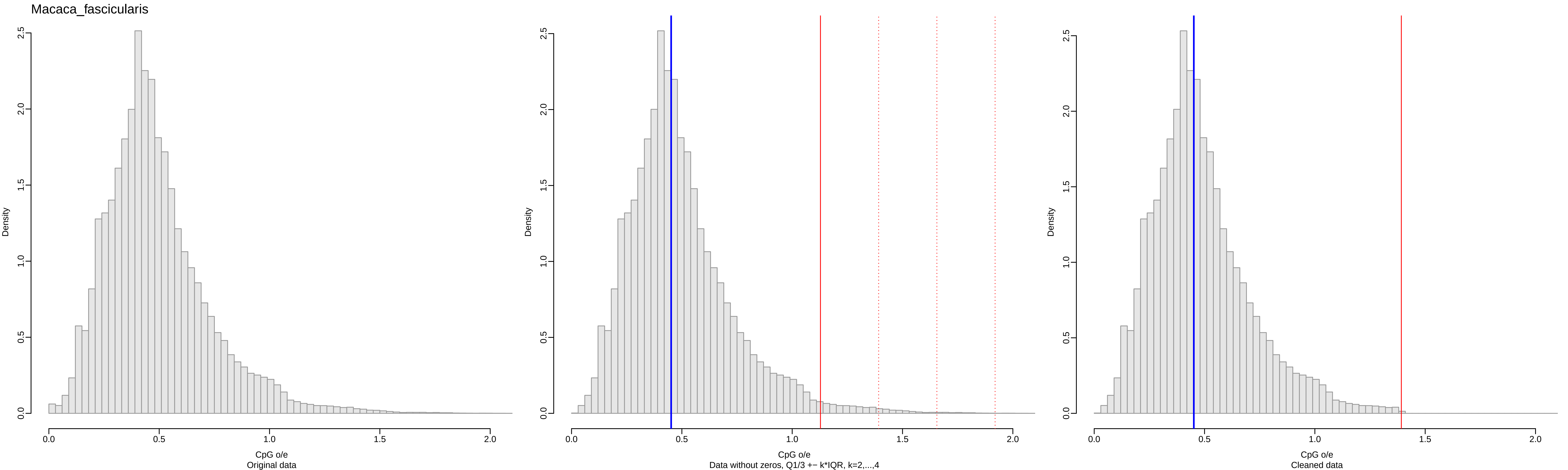

Macaca_fascicularis
Density
CpG o/e
Original data
Data without zeros, Q1/3 +− k*IQR, k=2,...,4
Cleaned data
0.0
0.5
1.0
1.5
2.0
2.5

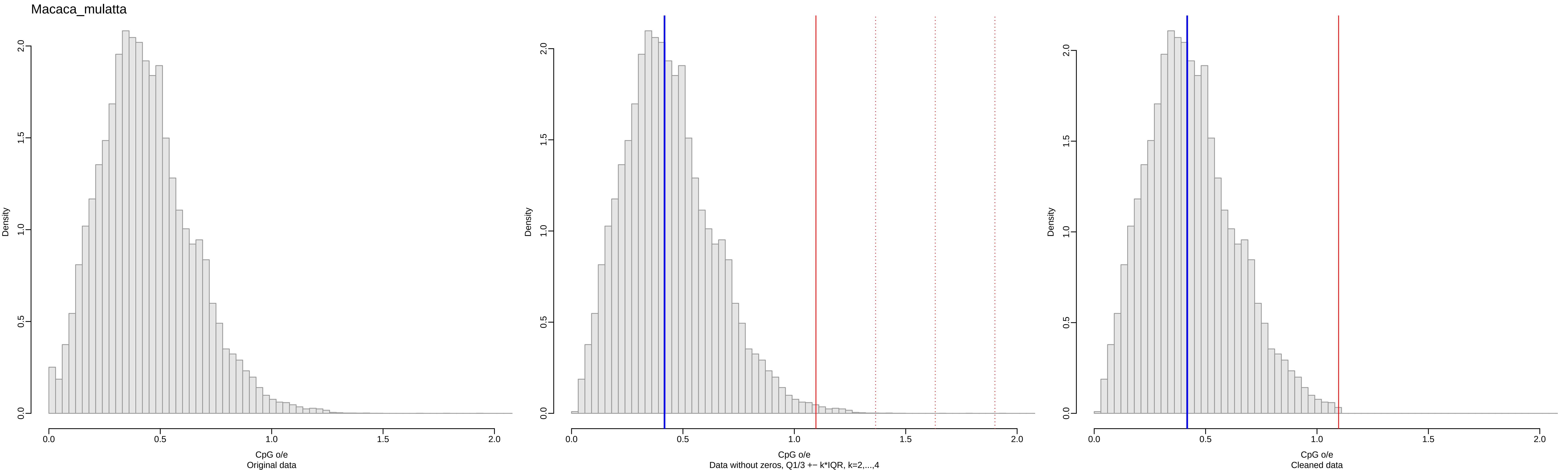
Macaca_mulatta
Density
CpG o/e
Original data
CpG o/e
Data without zeros, Q1/3 +− k*IQR, k=2,...,4
CpG o/e
Cleaned data
0.0
0.5
1.0
1.5
2.0

Macaca_nemestrina

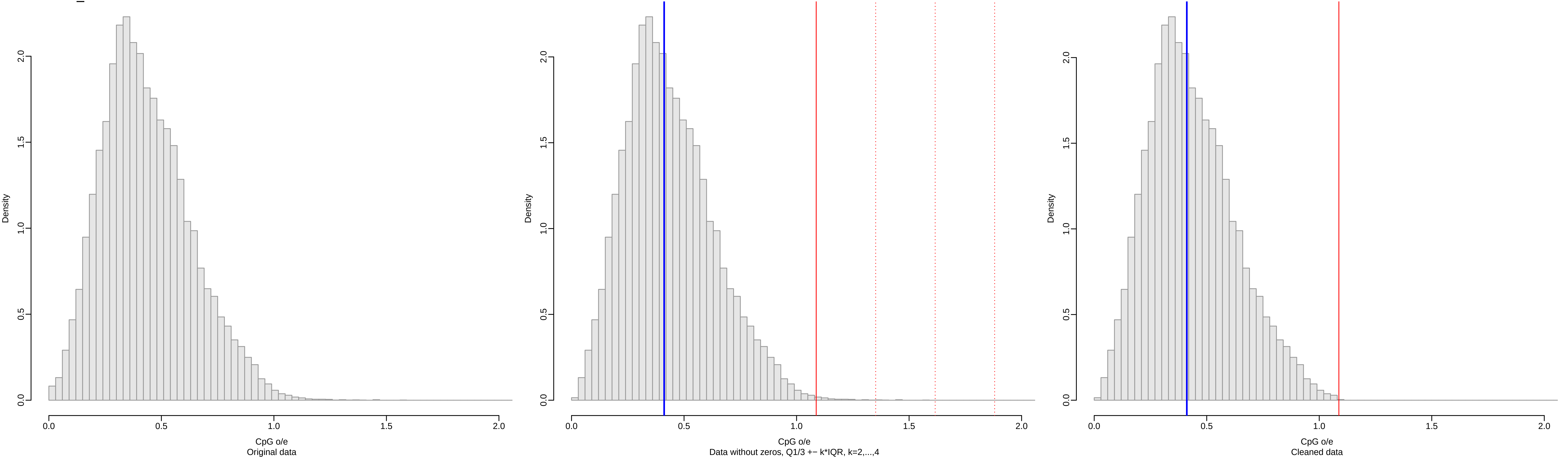

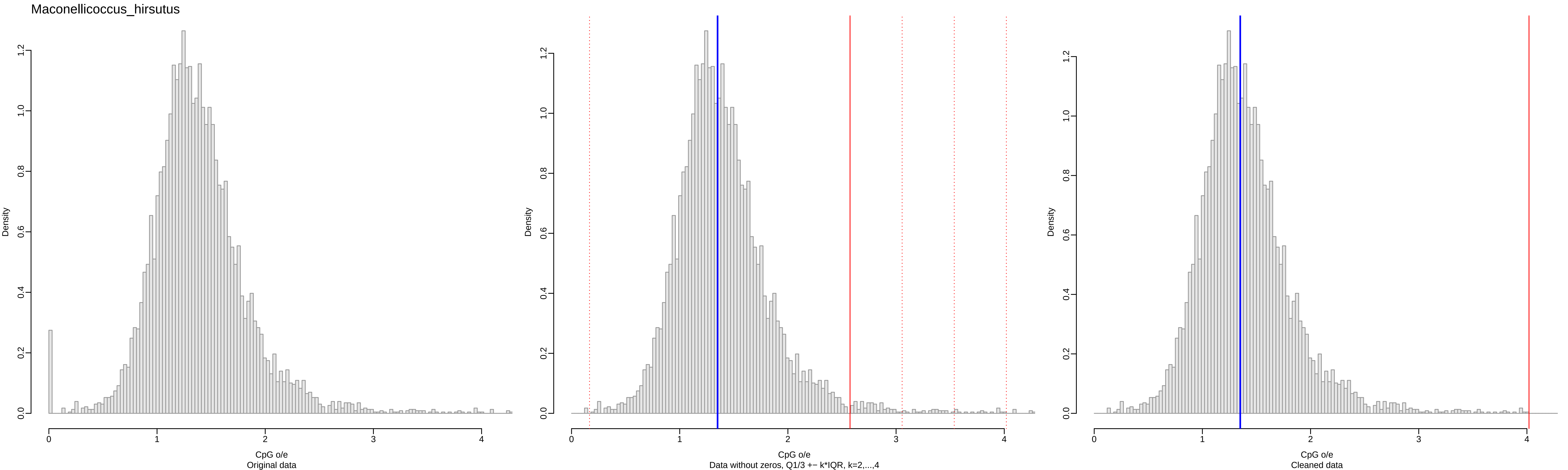
Maconellicoccus_hirsutus
Density
CpG o/e
Original data
Data without zeros, Q1/3 +− k*IQR, k=2,...,4
Cleaned data
0.0
0.2
0.4
0.6
0.8
1.0
1.2
0
1
2
3
4

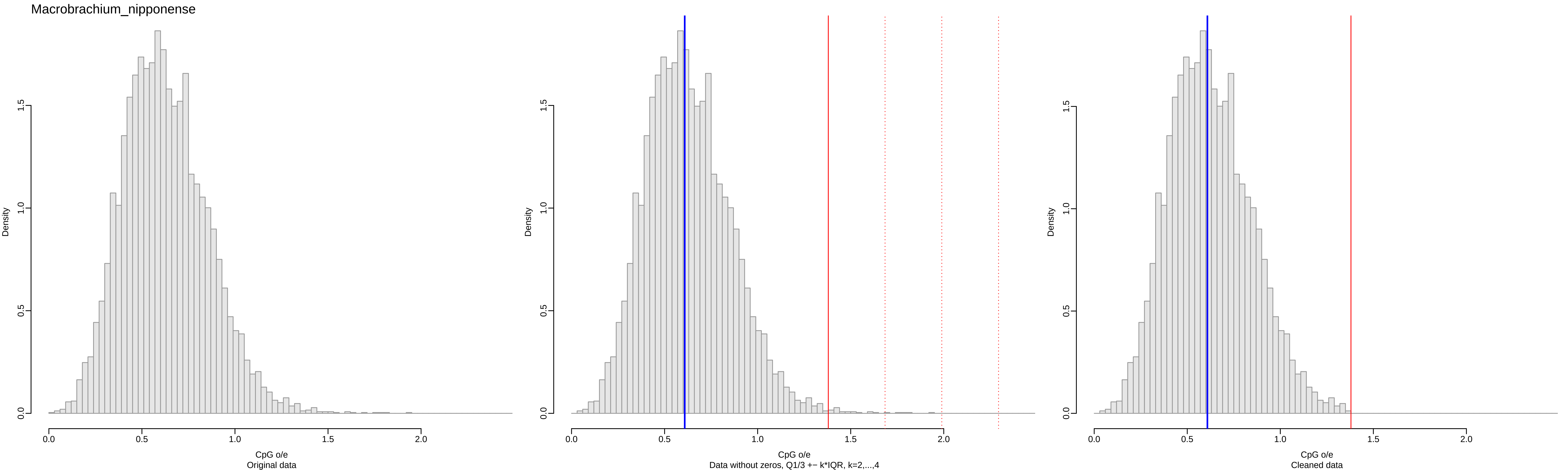

Macrobrachium_nipponense
Density
CpG o/e
Original data
Data without zeros, Q1/3 +− k*IQR, k=2,...,4
Cleaned data
0.0
0.5
1.0
1.5
2.0

Macrobrachium_rosenbergii

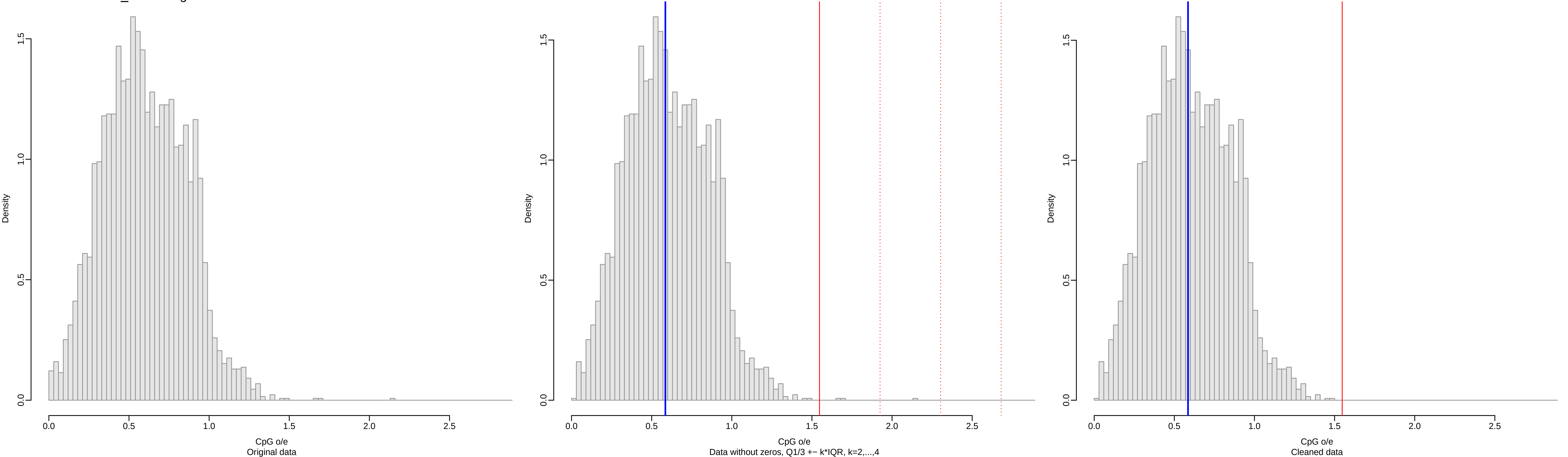

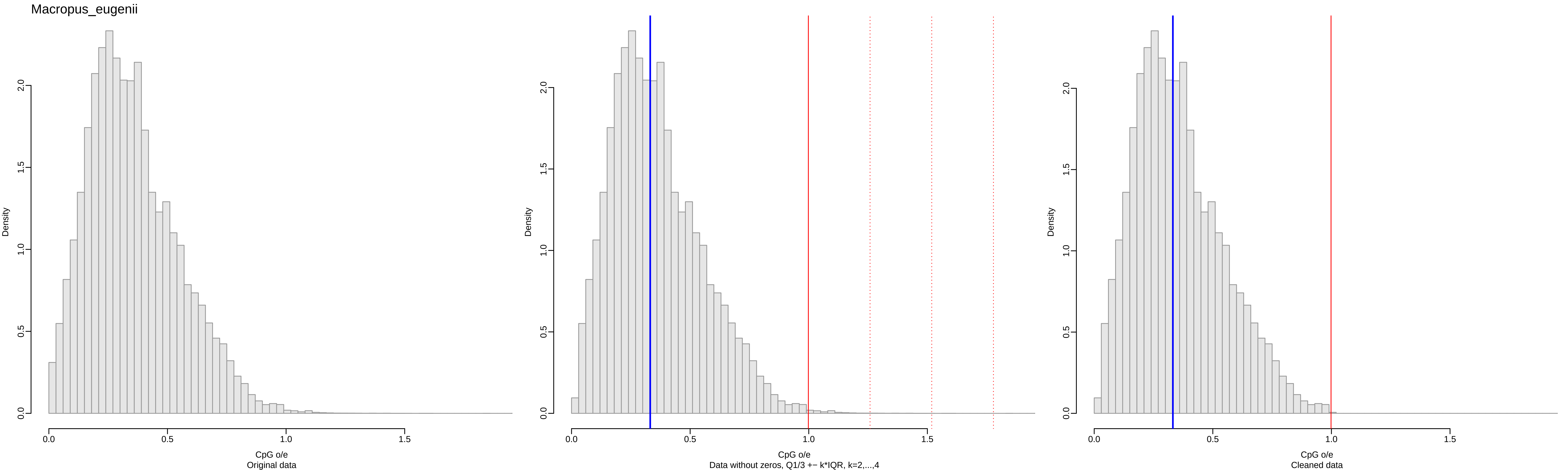

Macropus_eugenii
Density
CpG o/e
Original data
Data without zeros, Q1/3 +− k*IQR, k=2,...,4
Cleaned data

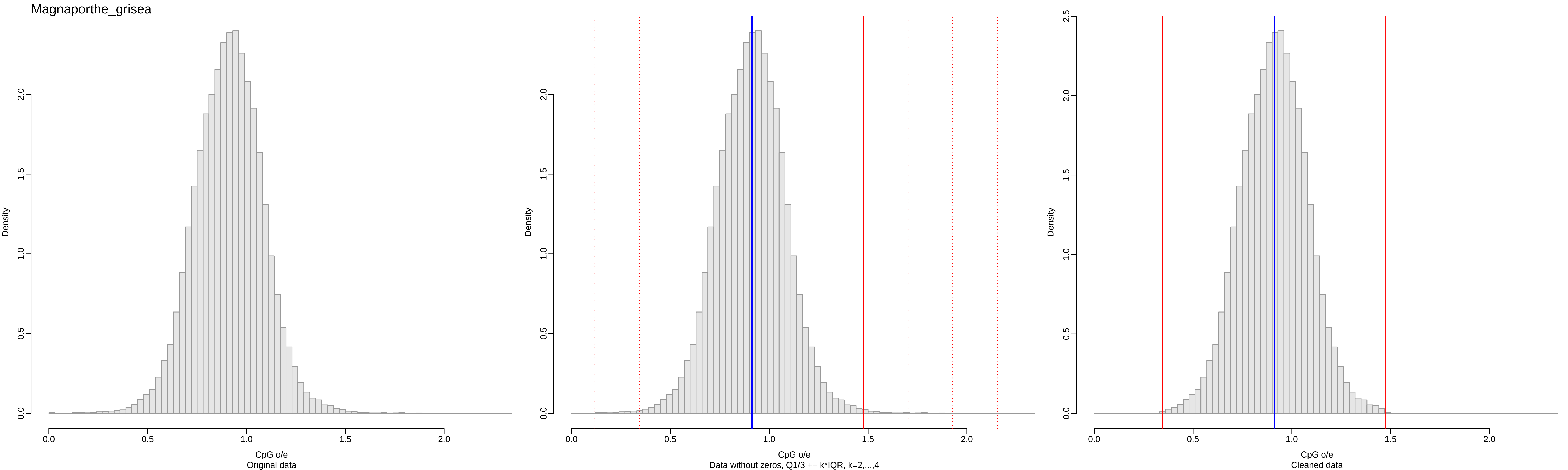
Magnaporthe_grisea
Density
CpG o/e
Original data
Data without zeros, Q1/3 +− k*IQR, k=2,...,4
Cleaned data
0.0
0.5
1.0
1.5
2.0
2.5

Magnaporthe_oryzae

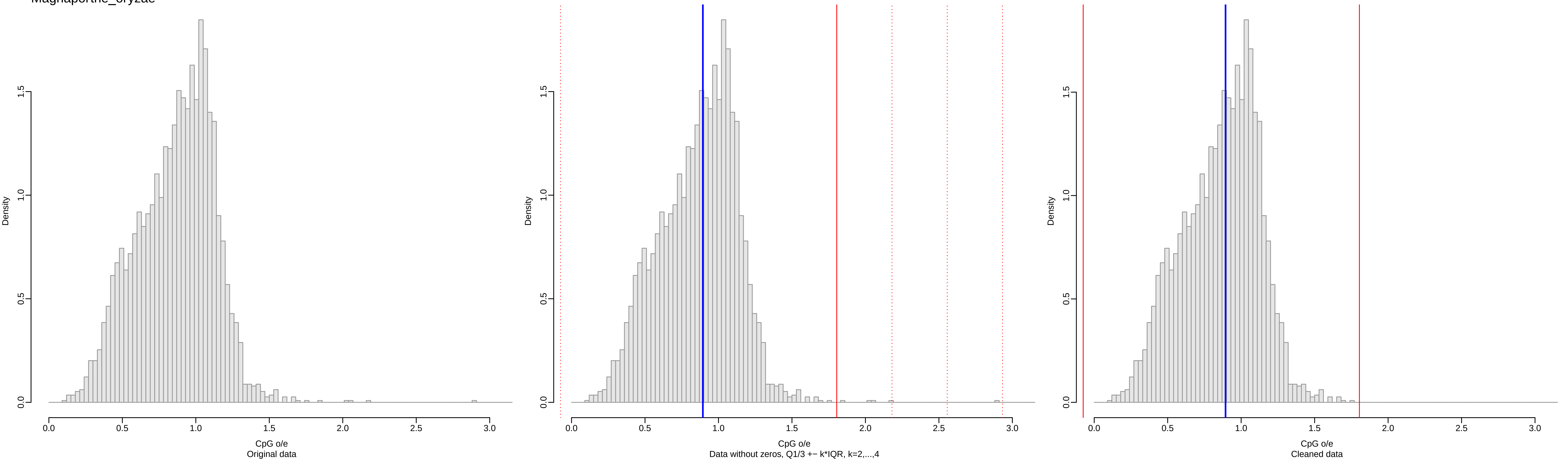

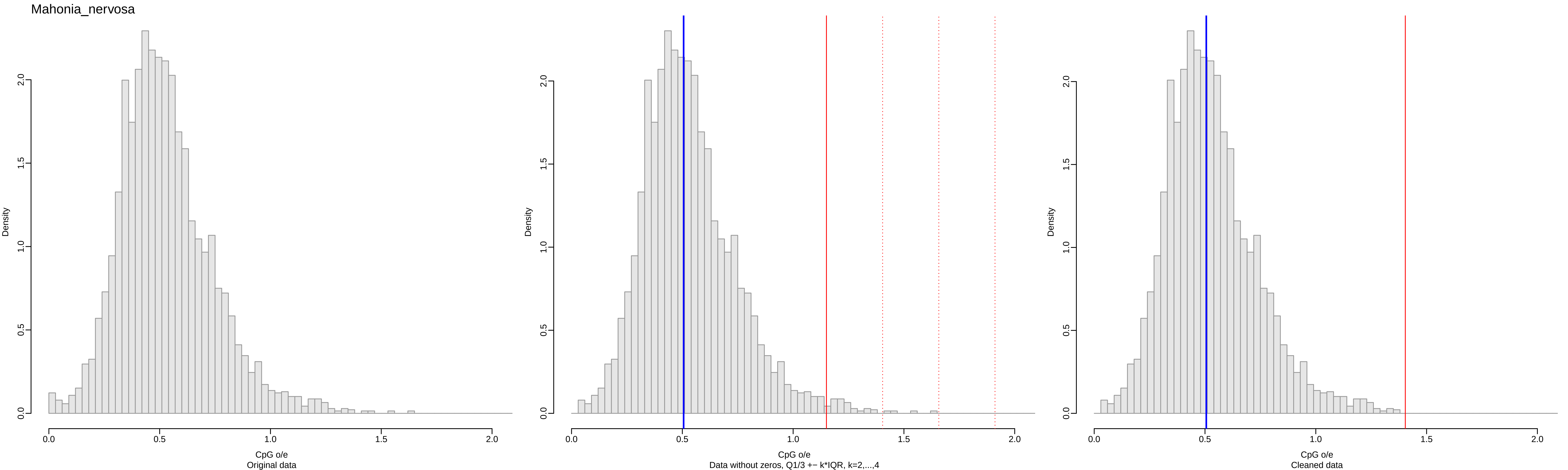

Mahonia_nervosa
Density
CpG o/e
Original data
Data without zeros, Q1/3 +− k*IQR, k=2,...,4
Cleaned data
0.0
0.5
1.0
1.5
2.0

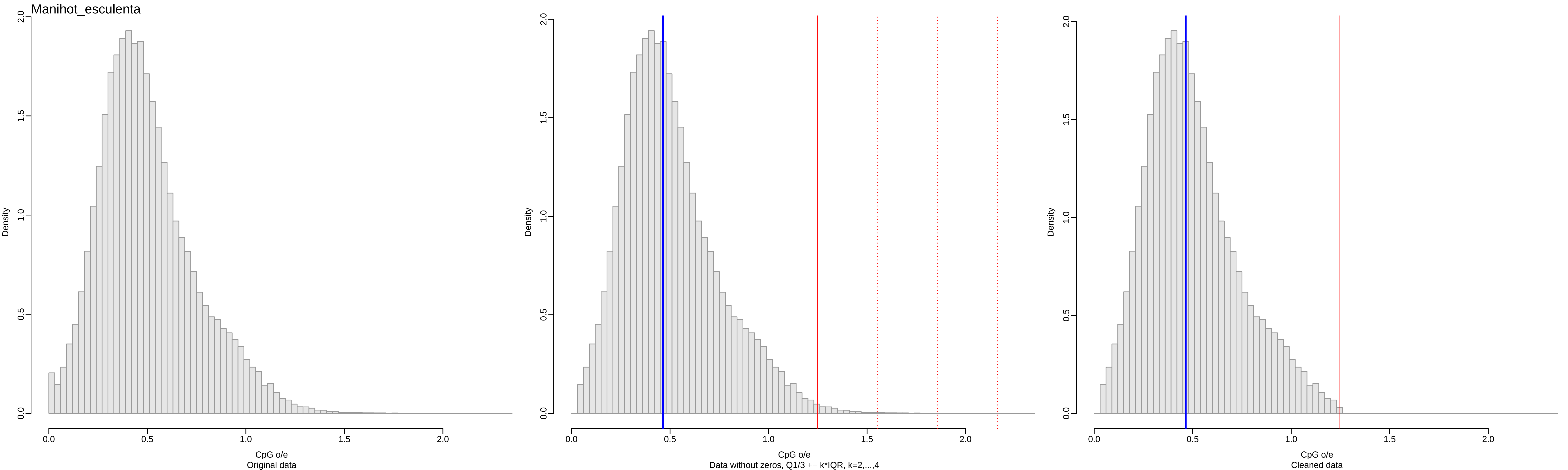
Manihot_esculenta
Density
CpG o/e
Original data
Data without zeros, Q1/3 +− k*IQR, k=2,...,4
Cleaned data
0.0
0.5
1.0
1.5
2.0

Marsupenaeus_japonicus

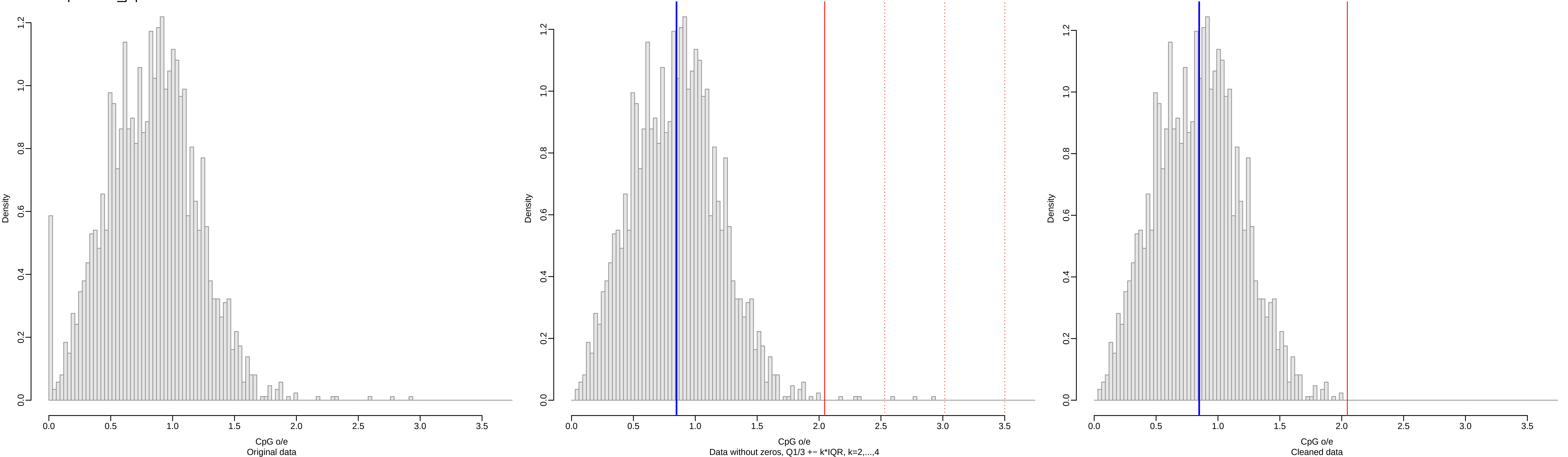

Mastigamoeba_balamuthi

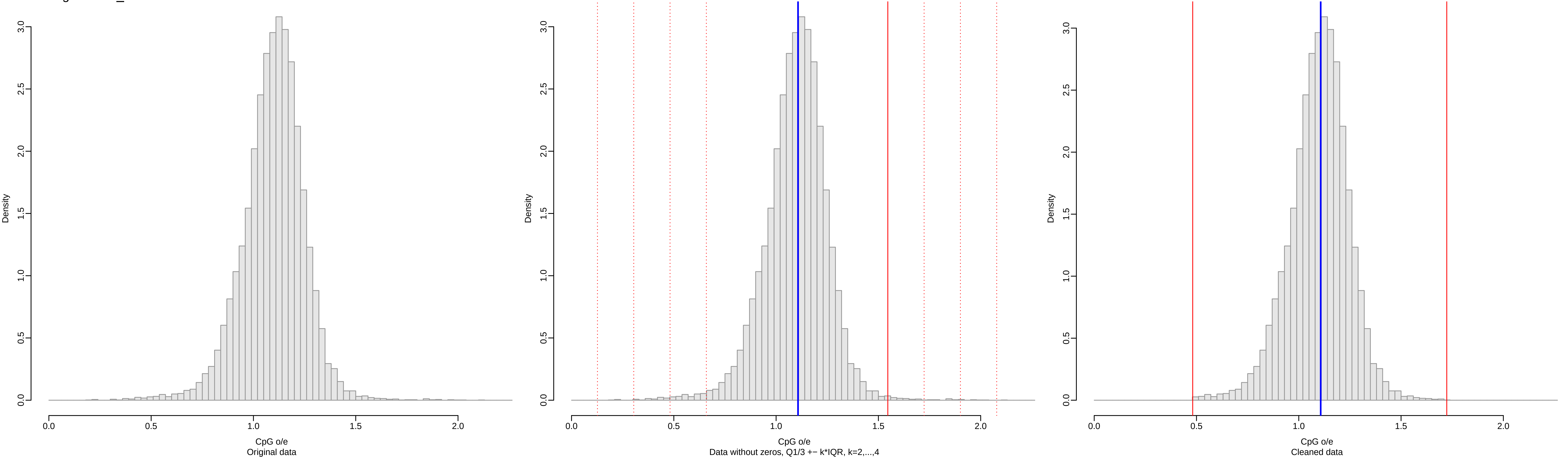

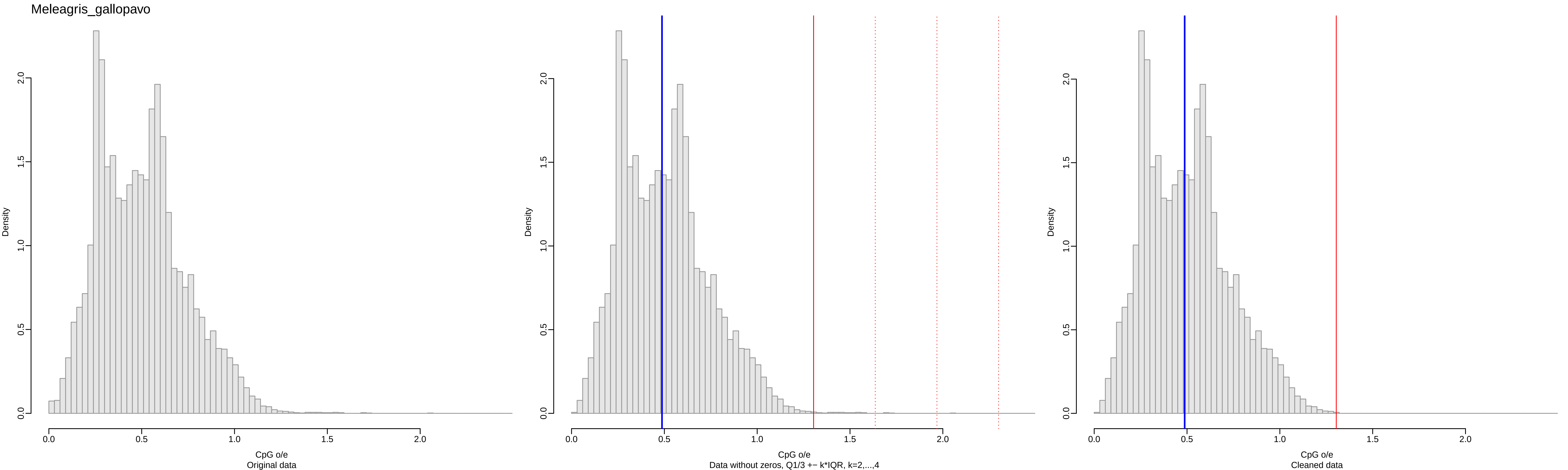
Meleagris_gallopavo
Density
CpG o/e
Original data
CpG o/e
Data without zeros, Q1/3 +− k*IQR, k=2,...,4
CpG o/e
Cleaned data

Meloidogyne_arenaria

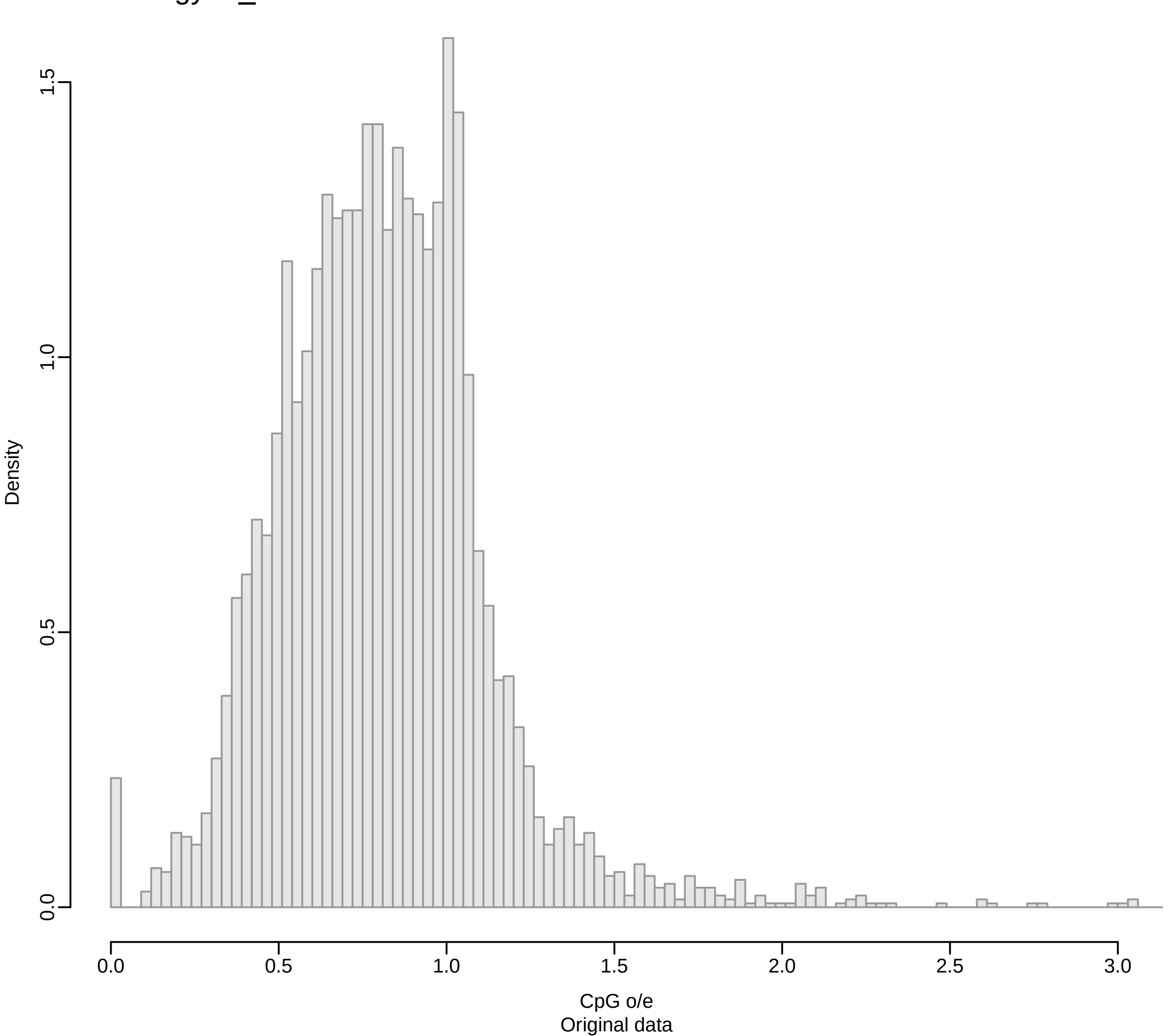

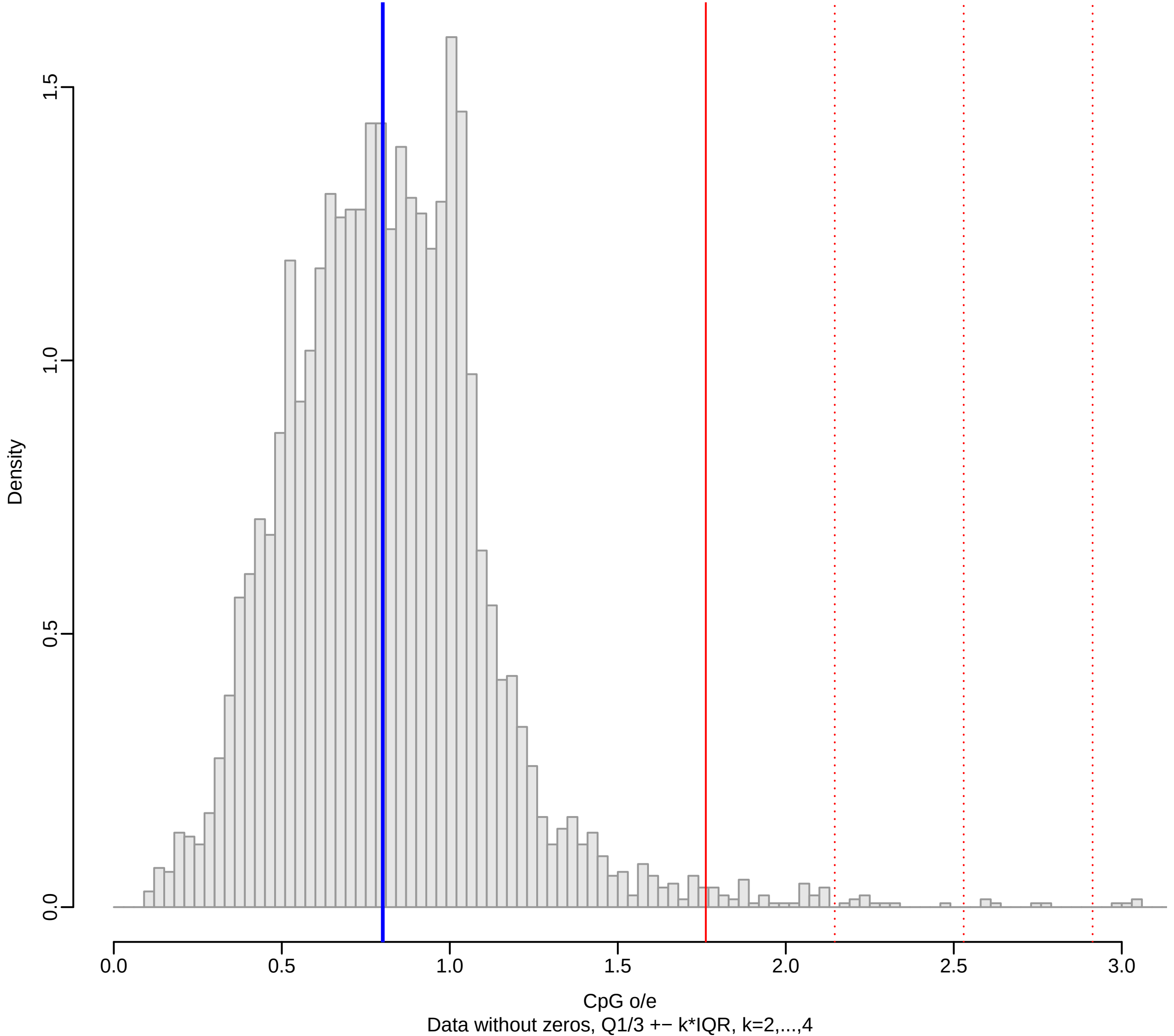

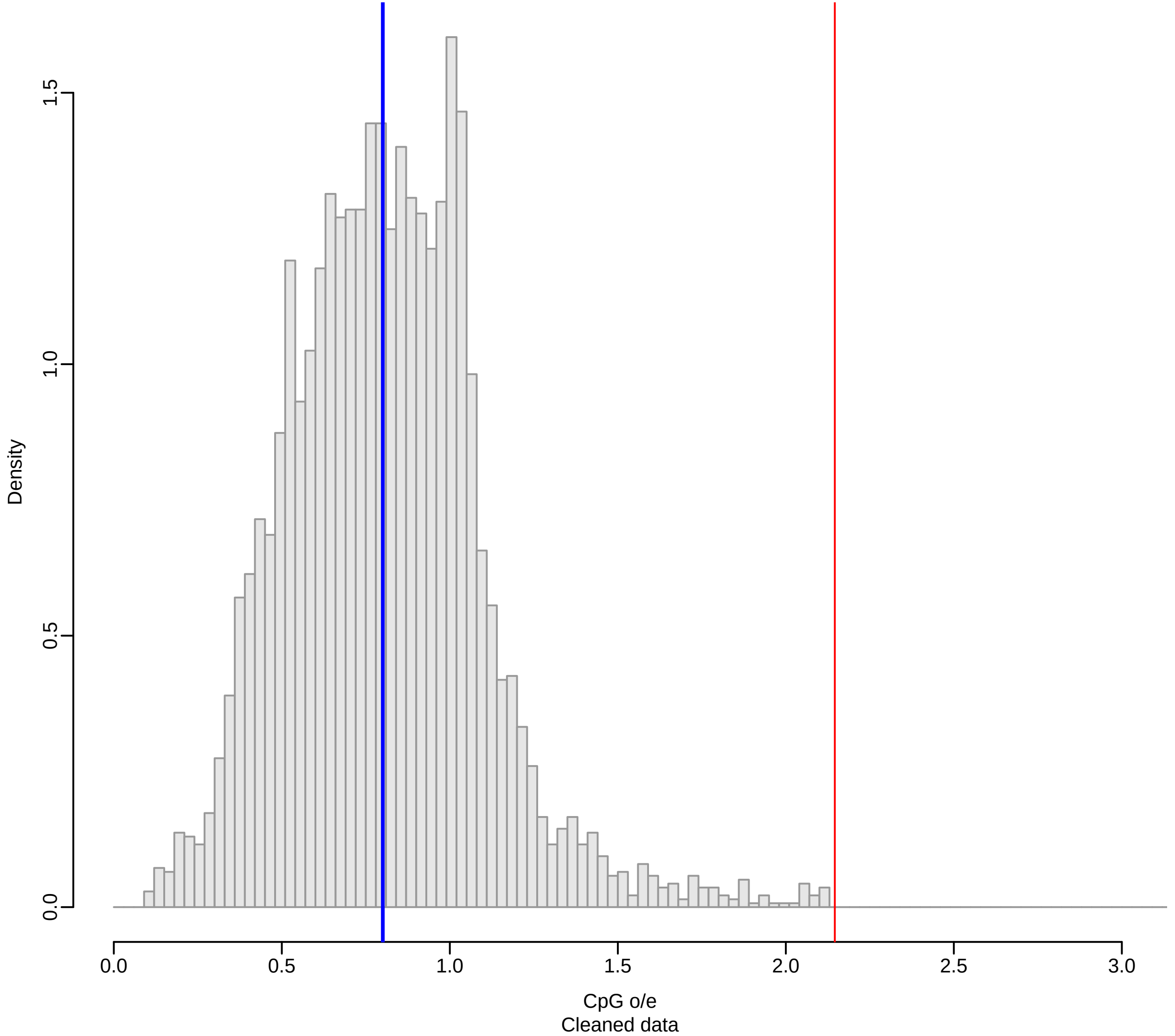

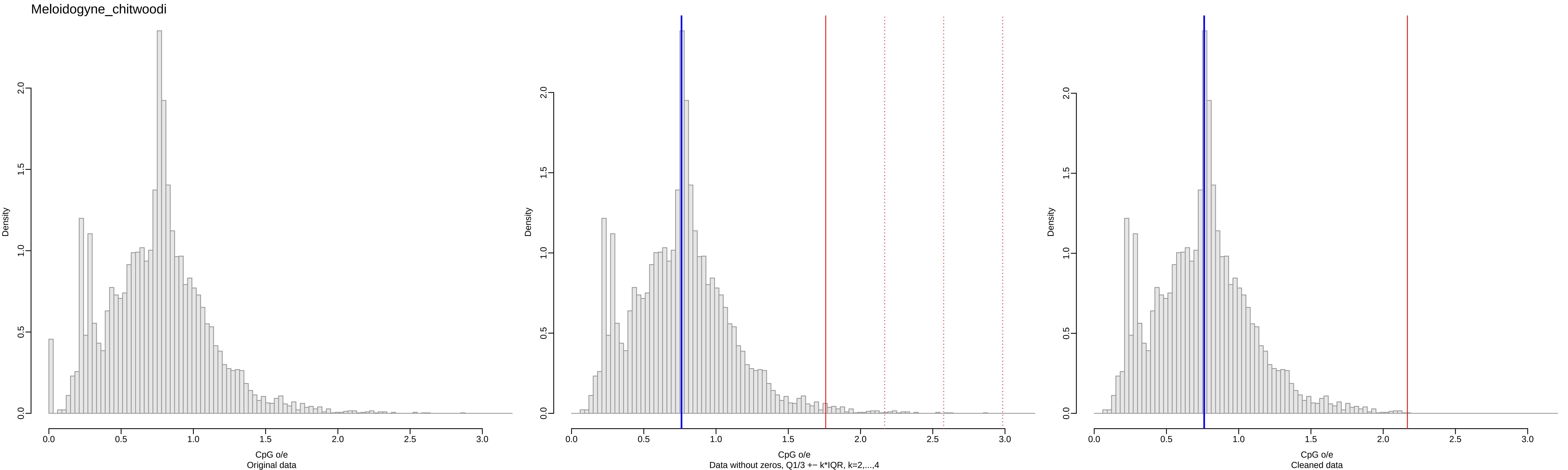

Meloidogyne_chitwoodi
Density
CpG o/e
Original data
Data without zeros, Q1/3 +− k*IQR, k=2,...,4
Cleaned data
0.0
0.5
1.0
1.5
2.0
2.5
3.0

Meloidogyne_hapla

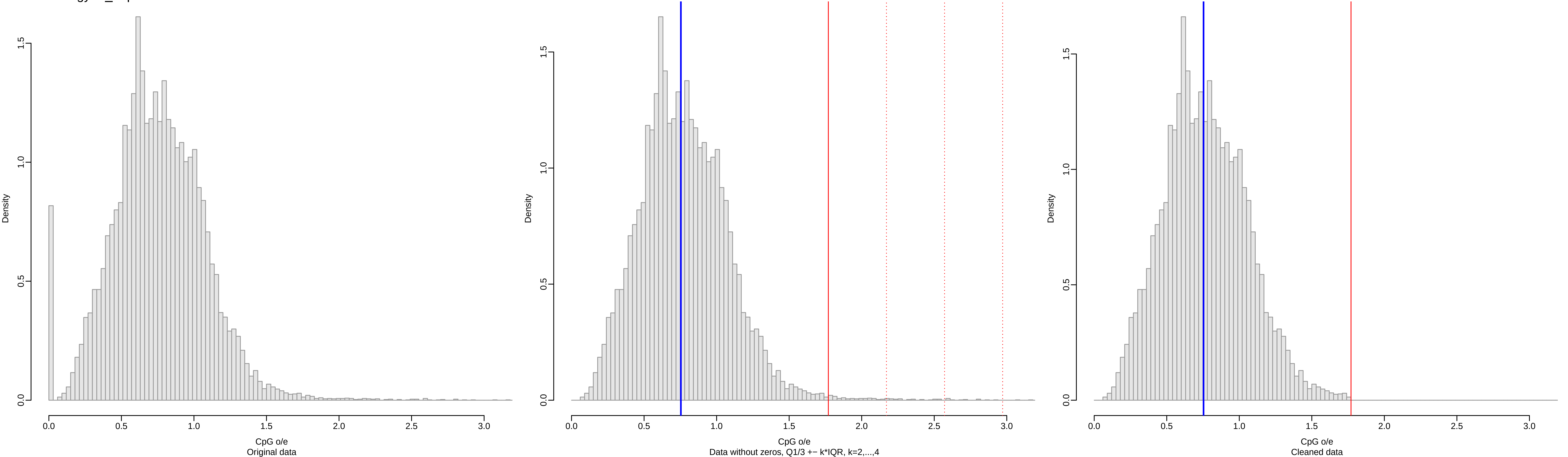

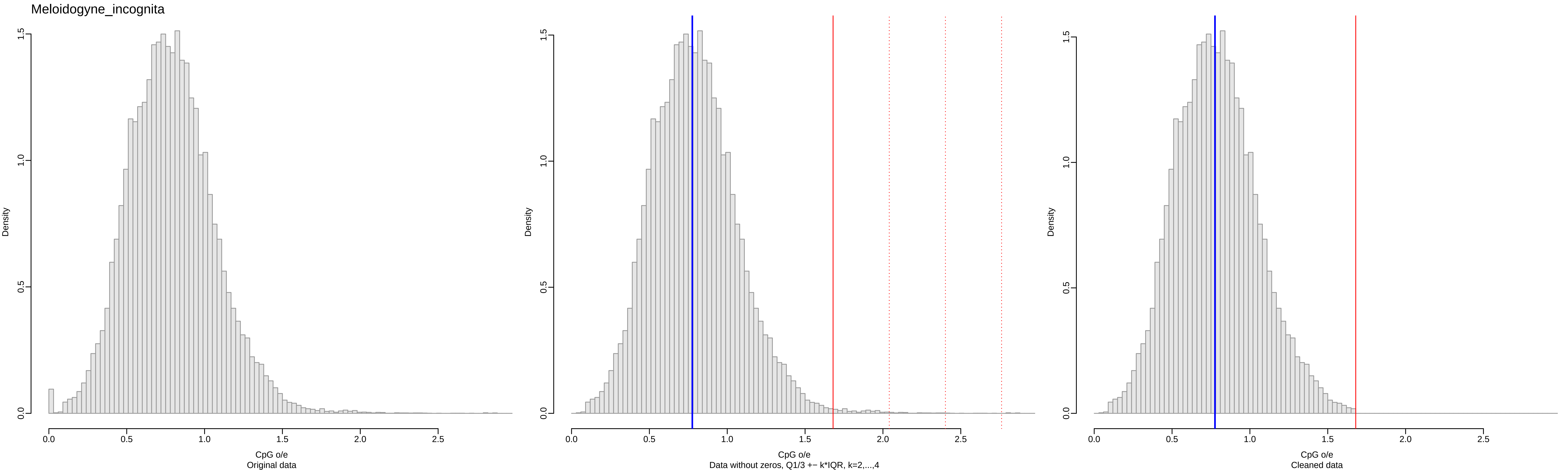

Meloidogyne_incognita
Density
0.0
0.5
1.0
1.5
2.0
2.5
CpG o/e
Original data
Data without zeros, Q1/3 +− k*IQR, k=2,...,4
Cleaned data

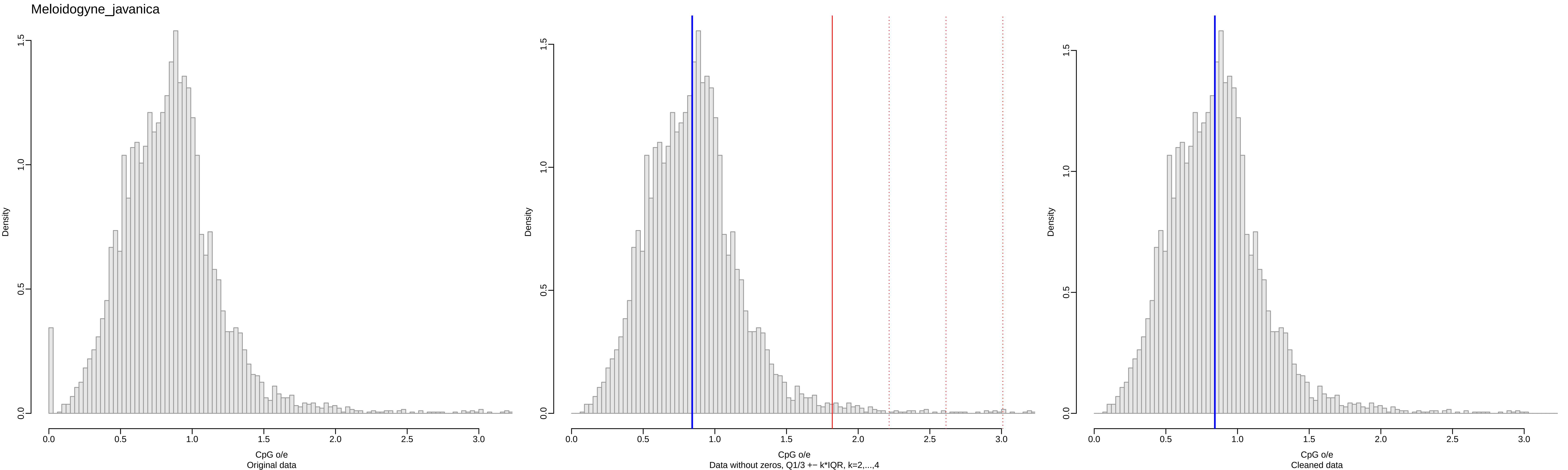

Meloidogyne_javanica
Density
CpG o/e
Original data
Data without zeros, Q1/3 +− k*IQR, k=2,...,4
Cleaned data
0.0
0.5
1.0
1.5
2.0
2.5
3.0

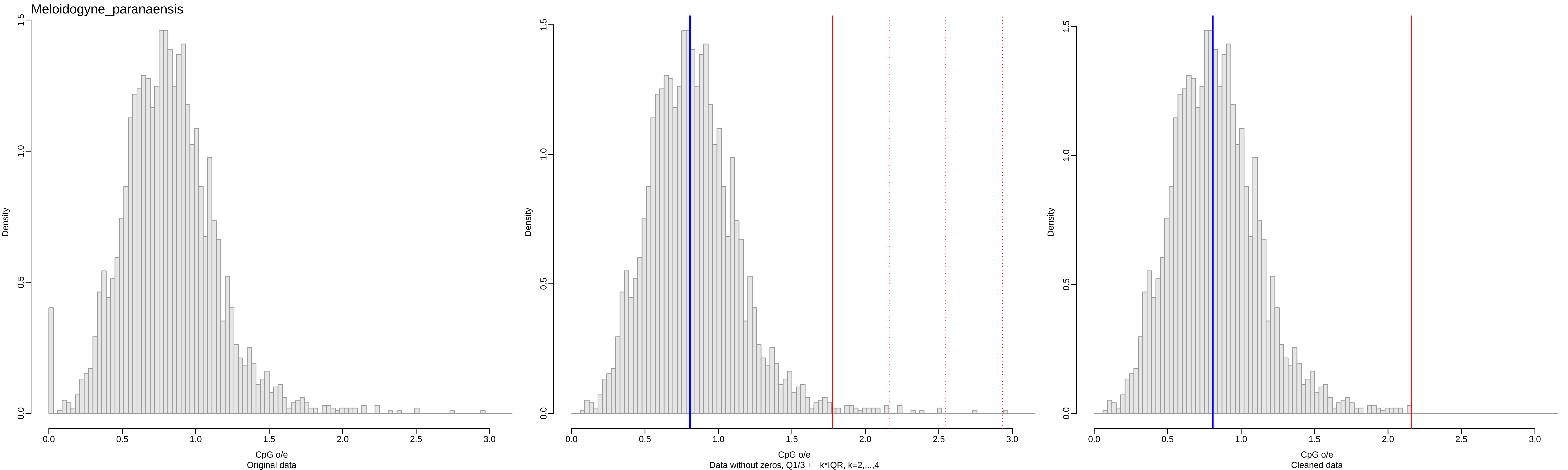

Meloidogyne_paranaensis
Density
CpG o/e
Original data
Data without zeros, Q1/3 +− k*IQR, k=2,...,4
Cleaned data
0.0
0.5
1.0
1.5
2.0
2.5
3.0

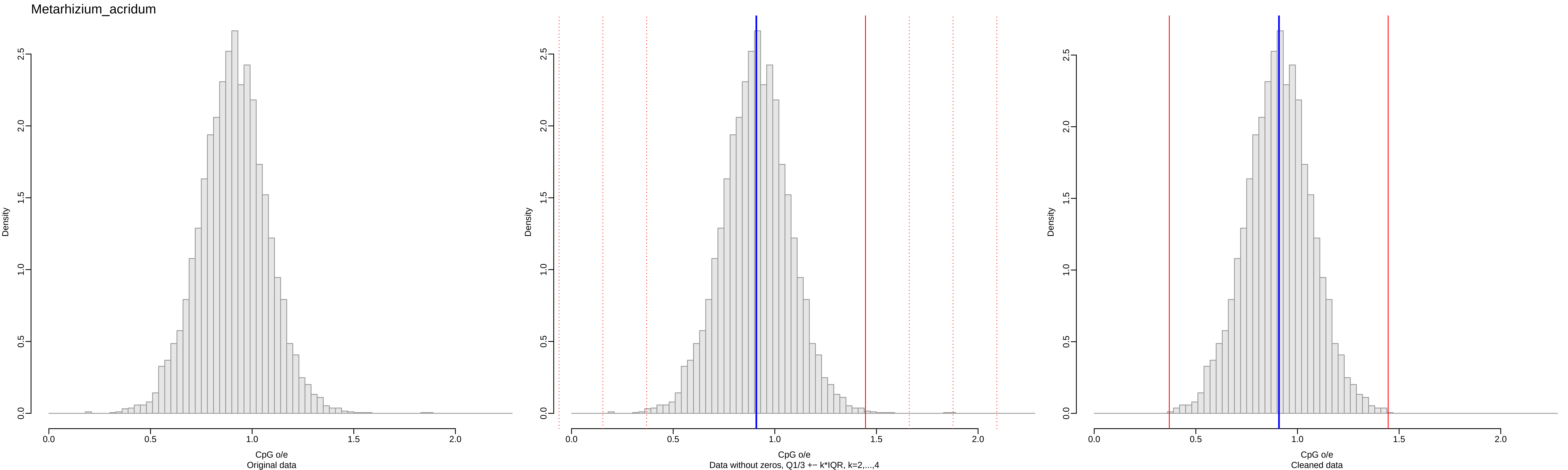
Metarhizium_acridum
Density
0.0
0.5
1.0
1.5
2.0
2.5
CpG o/e
Original data
Data without zeros, Q1/3 +− k*IQR, k=2,...,4
Cleaned data

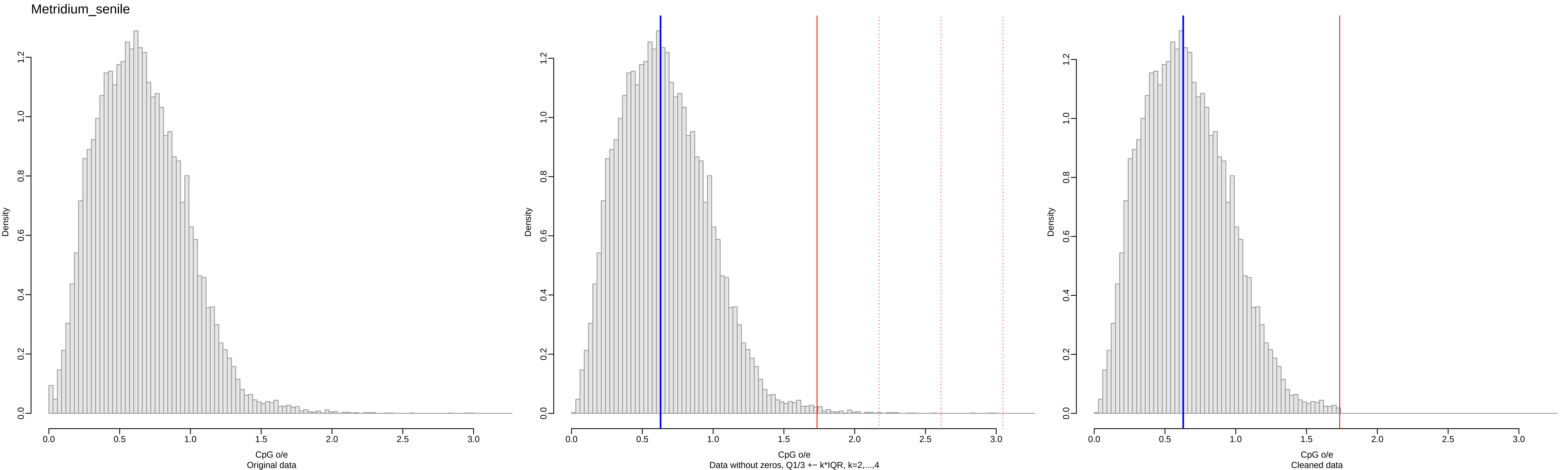
Metridium_senile
Density
CpG o/e
Original data
Density
CpG o/e
Data without zeros, Q1/3 +− k*IQR, k=2,...,4
Density
CpG o/e
Cleaned data

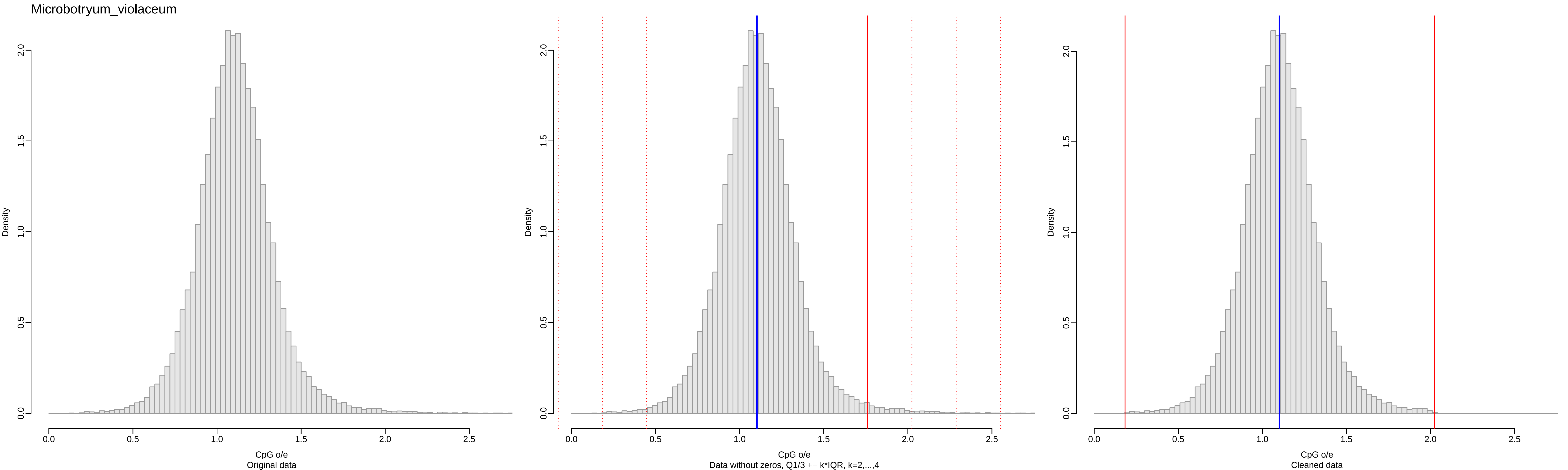

Microbotryum_violaceum
Density
0.0
0.5
1.0
1.5
2.0
2.5
CpG o/e
Original data
Data without zeros, Q1/3 +− k*IQR, k=2,...,4
Cleaned data

Milnesium_tardigradum

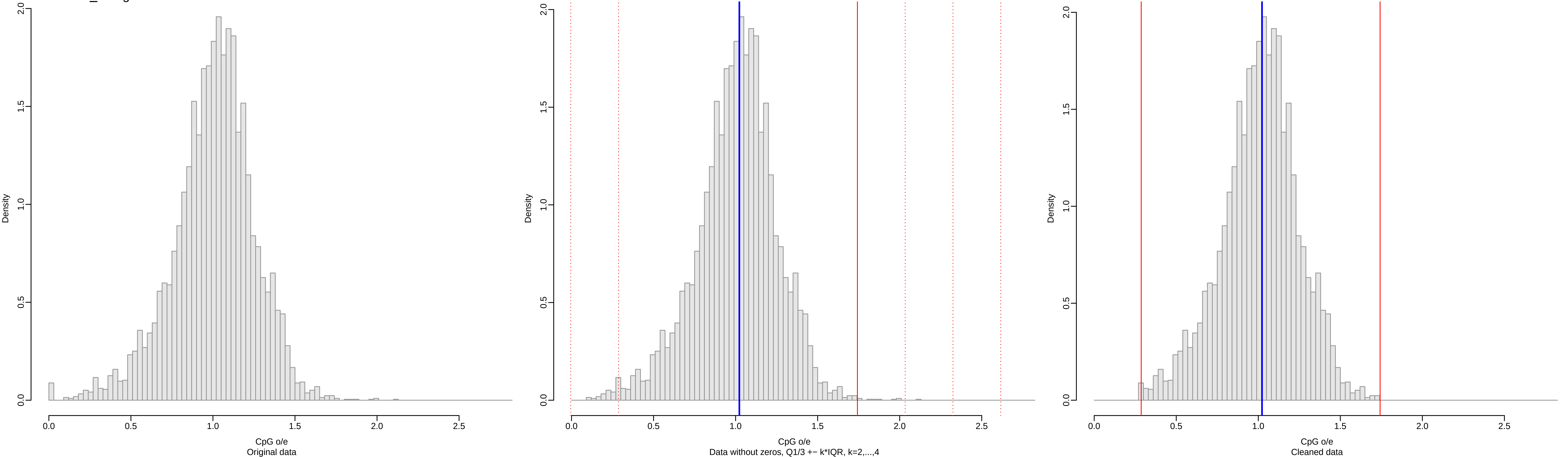

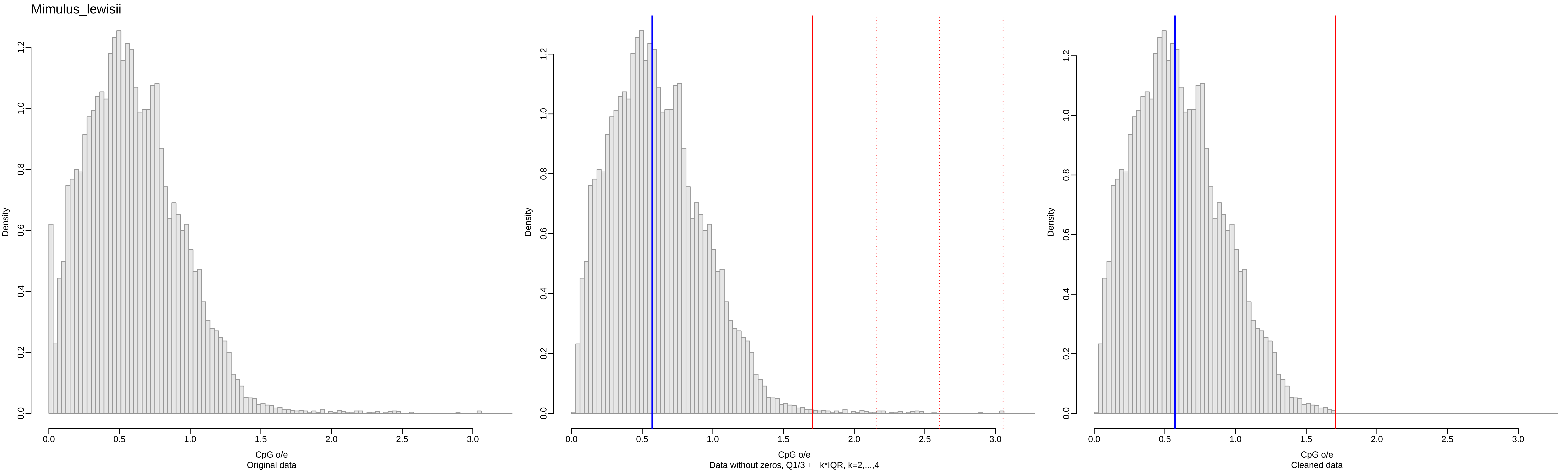

Mimulus_lewisii
Density
CpG o/e
Original data
CpG o/e
Data without zeros, Q1/3 +− k*IQR, k=2,...,4
CpG o/e
Cleaned data

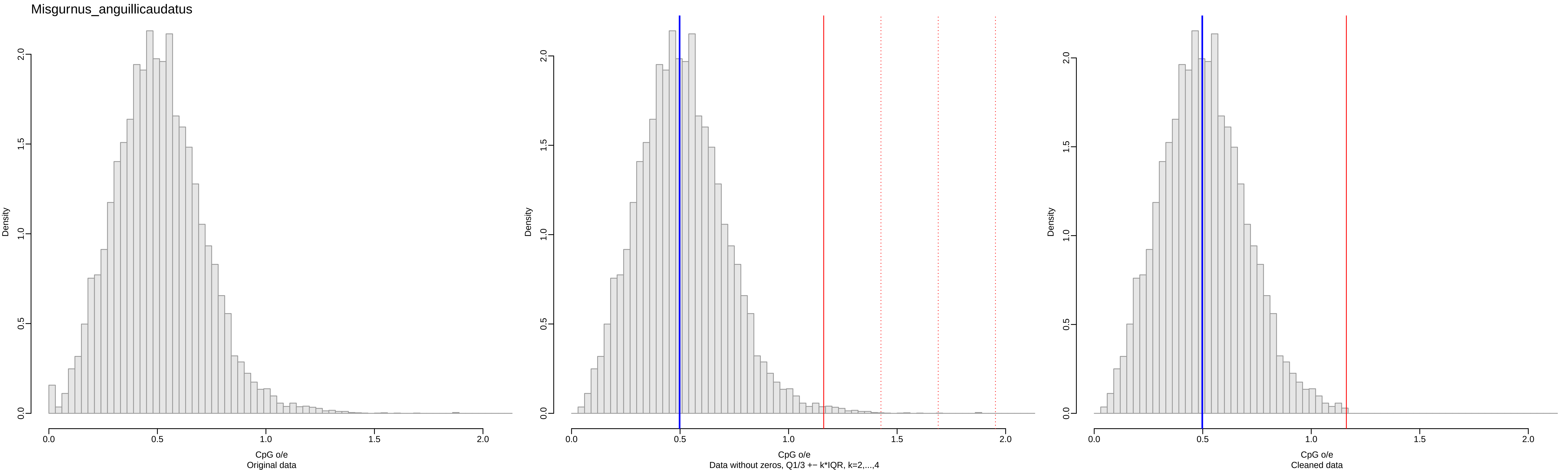

Misgurnus_anguillicaudatus
Density
CpG o/e
Original data
CpG o/e
Data without zeros, Q1/3 +− k*IQR, k=2,...,4
CpG o/e
Cleaned data
0.0
0.5
1.0
1.5
2.0

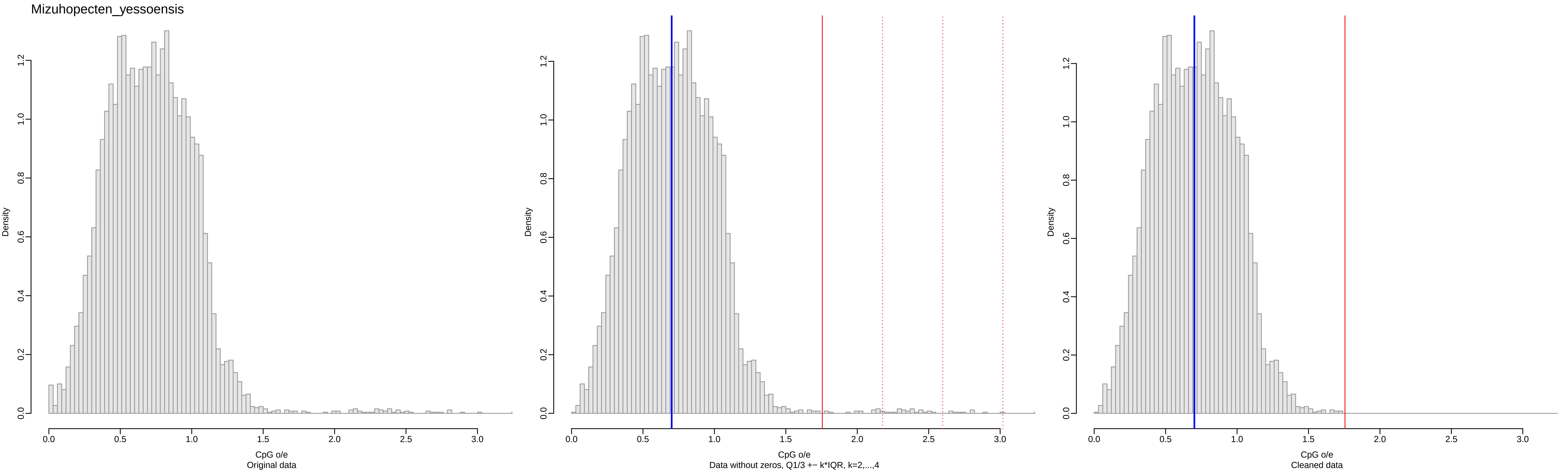
Mizuhopecten_yessoensis
Density
CpG o/e
Original data
Density
CpG o/e
Data without zeros, Q1/3 +− k*IQR, k=2,...,4
Density
CpG o/e
Cleaned data

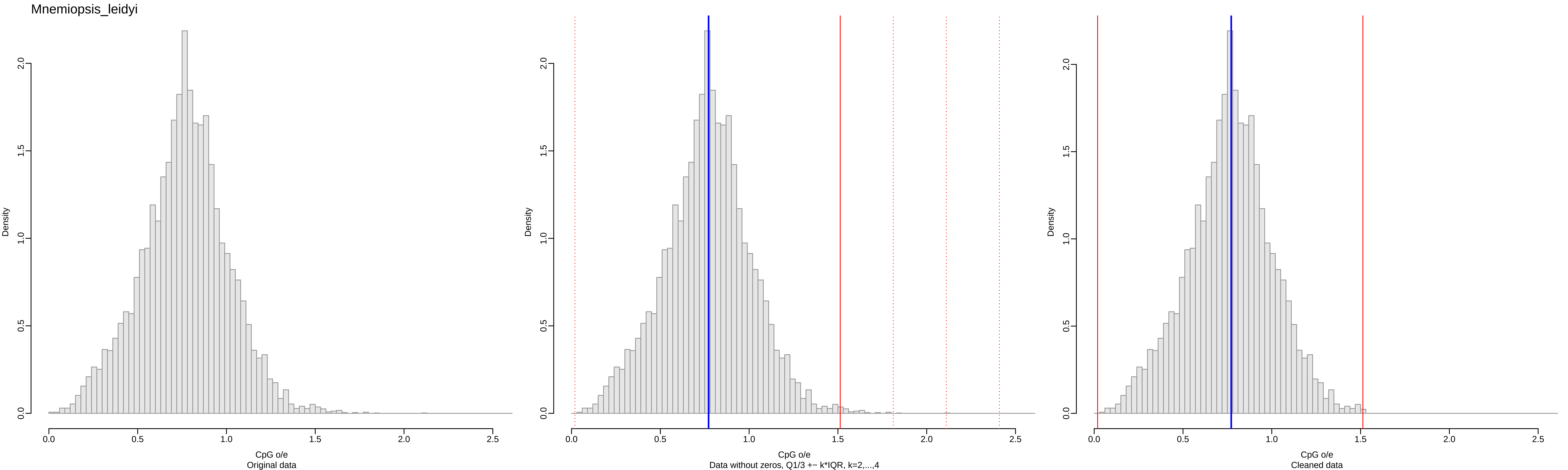
Mnemiopsis_leidyi
Density
CpG o/e
Original data
Data without zeros, Q1/3 +− k*IQR, k=2,...,4
Cleaned data
0.0
0.5
1.0
1.5
2.0
2.5

Molgula_tectiformis

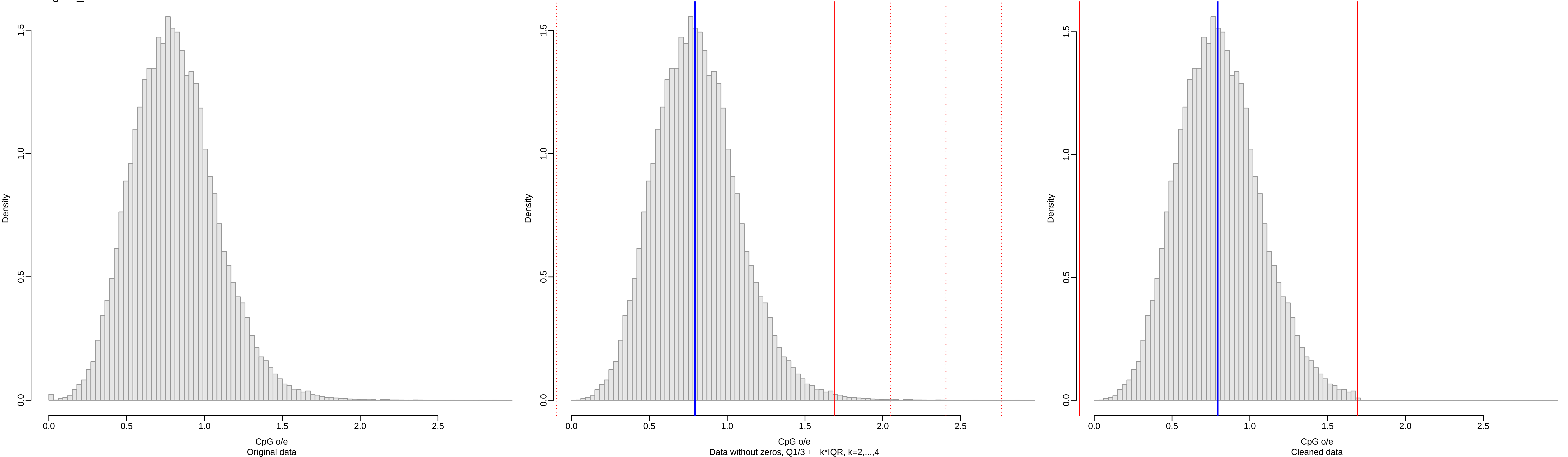

Moniliophthora_perniciosa

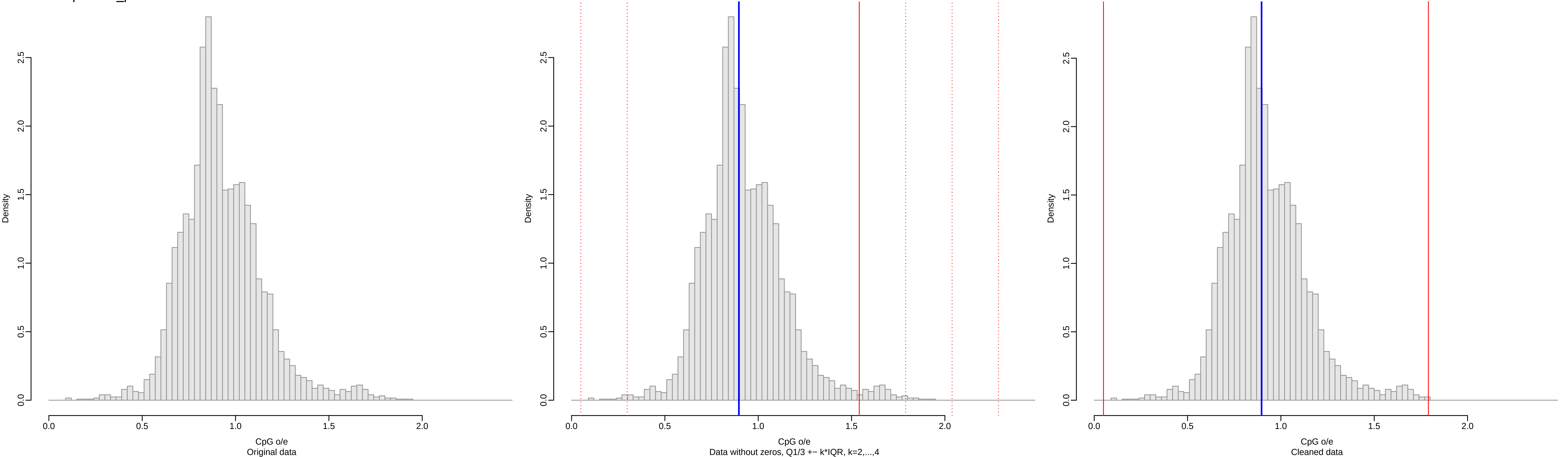

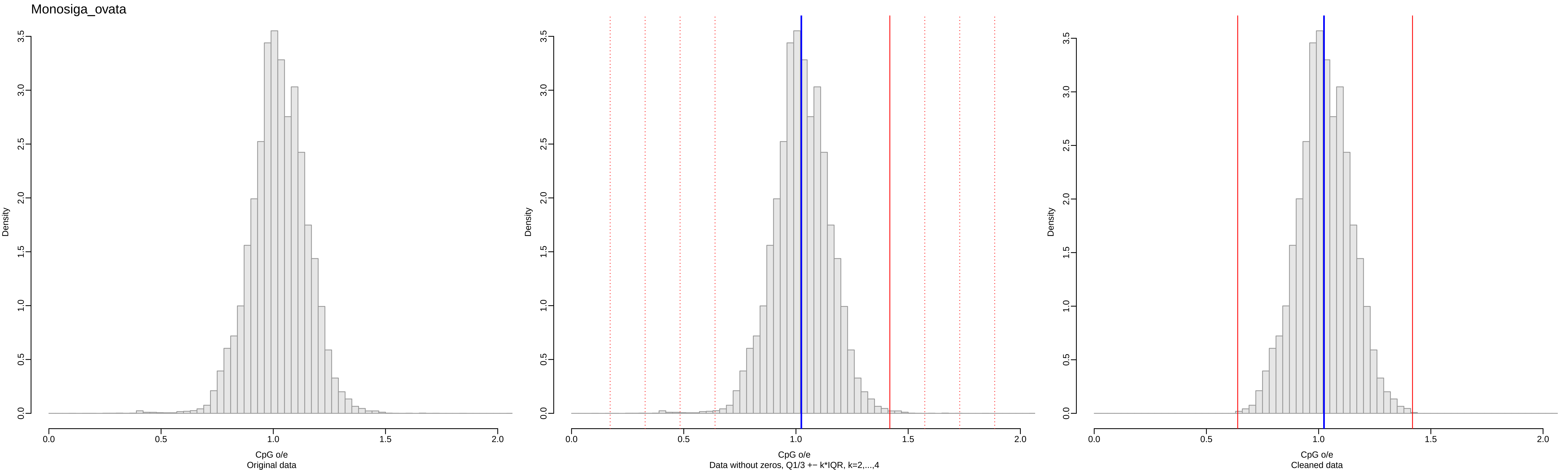

Monosiga_ovata
Density
CpG o/e
Original data
Data without zeros, Q1/3 +− k*IQR, k=2,...,4
Cleaned data
0.0
0.5
1.0
1.5
2.0
2.5
3.0
3.5

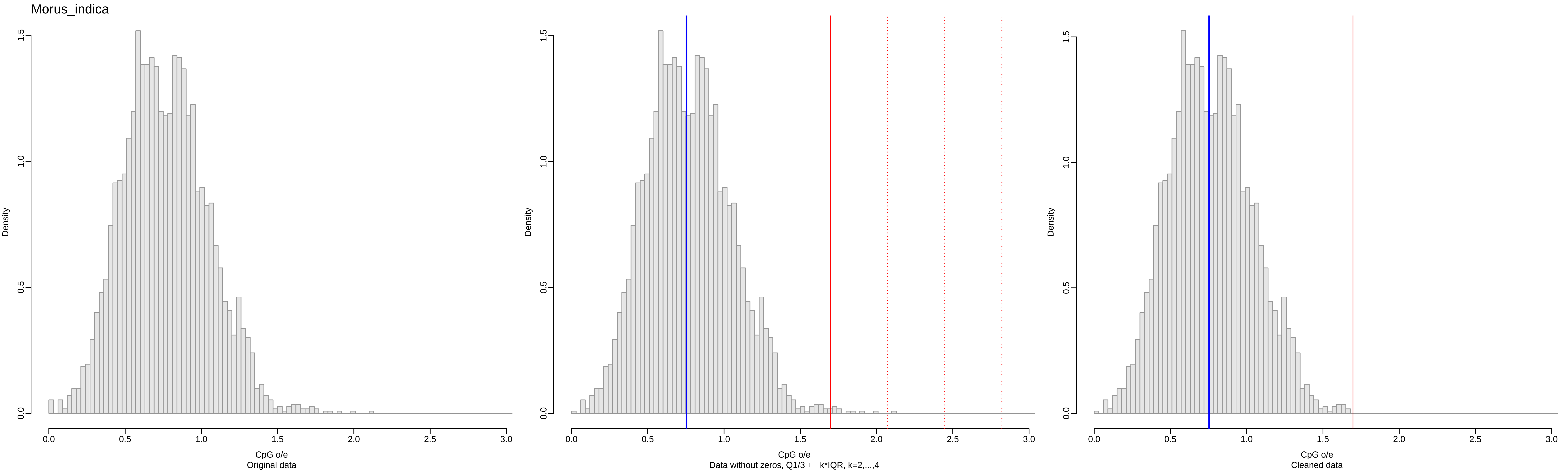

Morus_indica
Density
CpG o/e
Original data
Data without zeros, Q1/3 +− k*IQR, k=2,...,4
Cleaned data
0.0
0.5
1.0
1.5
2.0
2.5
3.0

Mycosphaerella_fijiensis

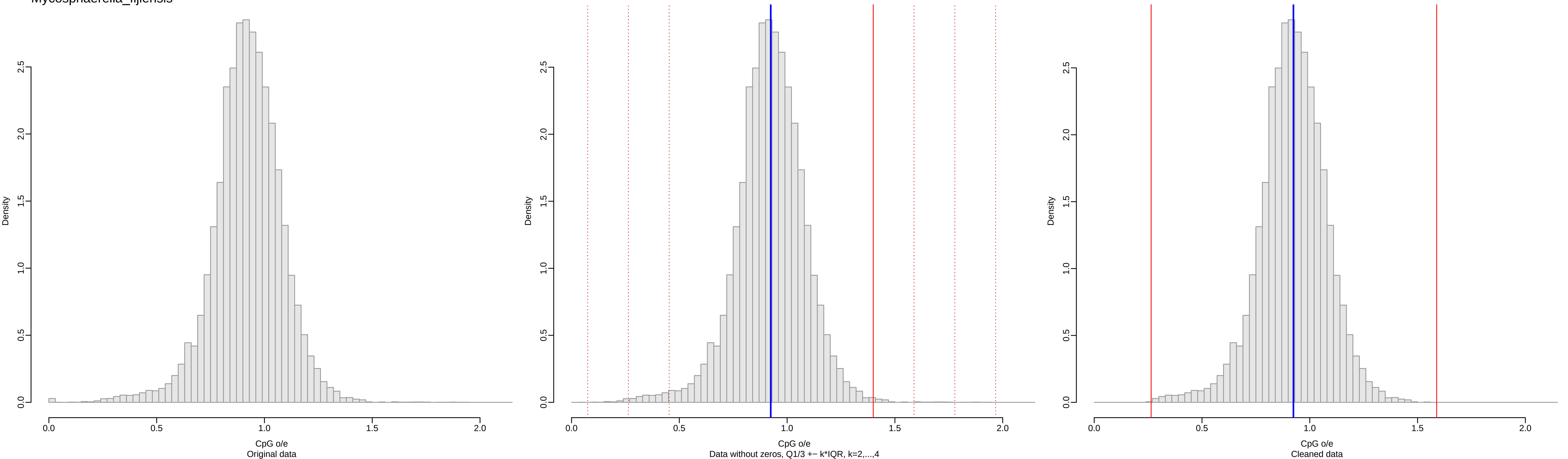

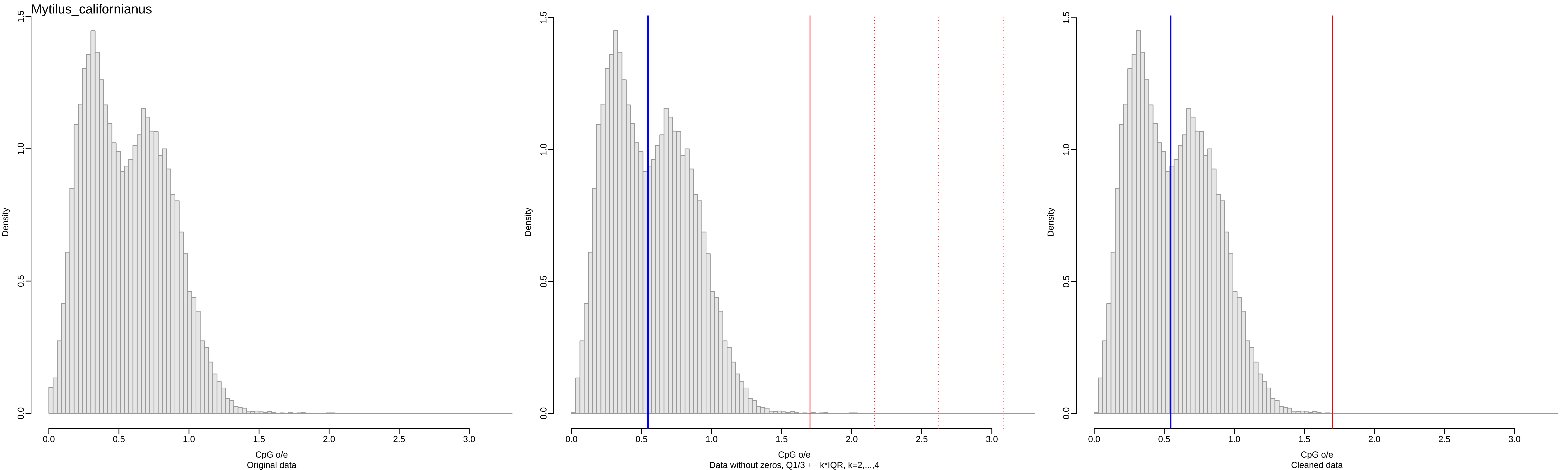

Mytilus_californianus
Density
CpG o/e
Original data
Data without zeros, Q1/3 +− k*IQR, k=2,...,4
Cleaned data

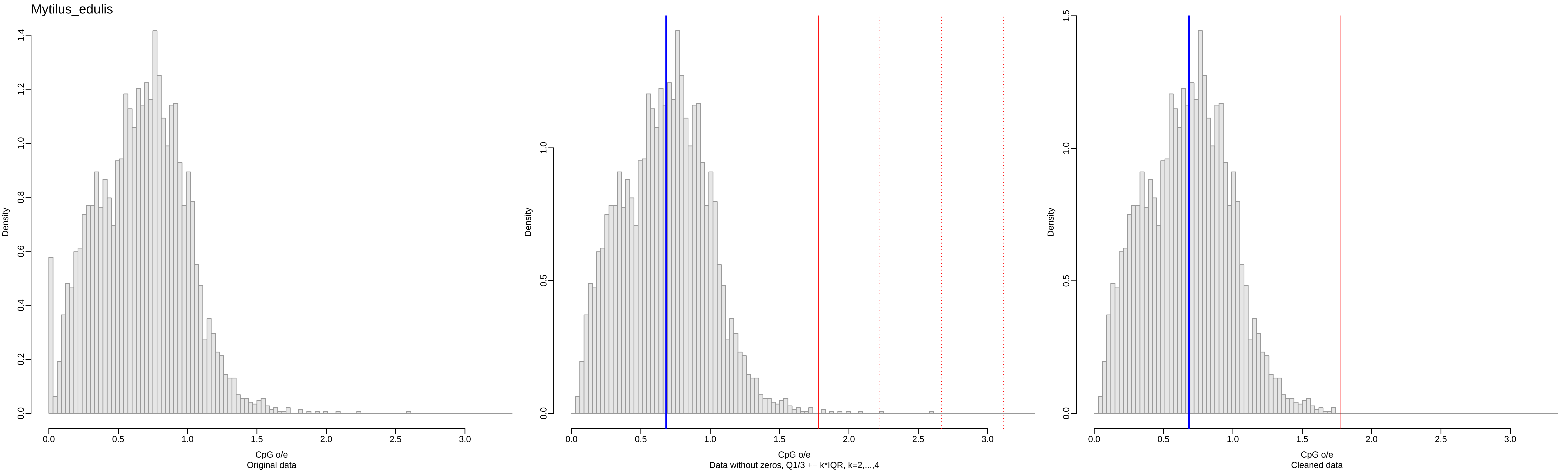
Mytilus_edulis
Density
CpG o/e
Original data
Density
CpG o/e
Data without zeros, Q1/3 +− k*IQR, k=2,...,4
Density
CpG o/e
Cleaned data

Mytilus_galloprovincialis

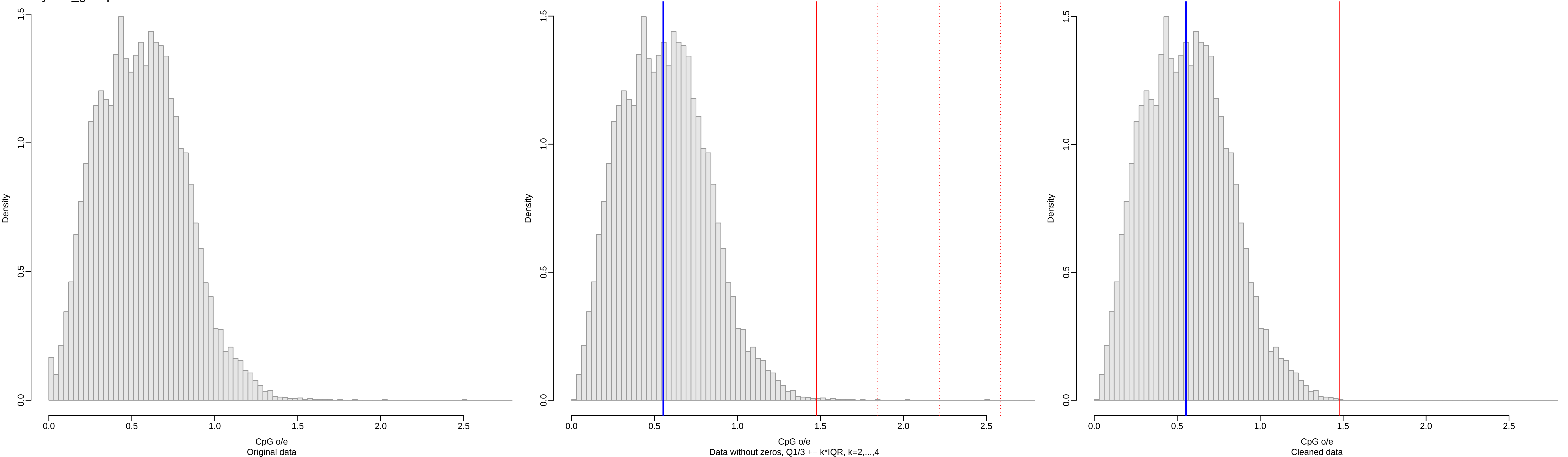

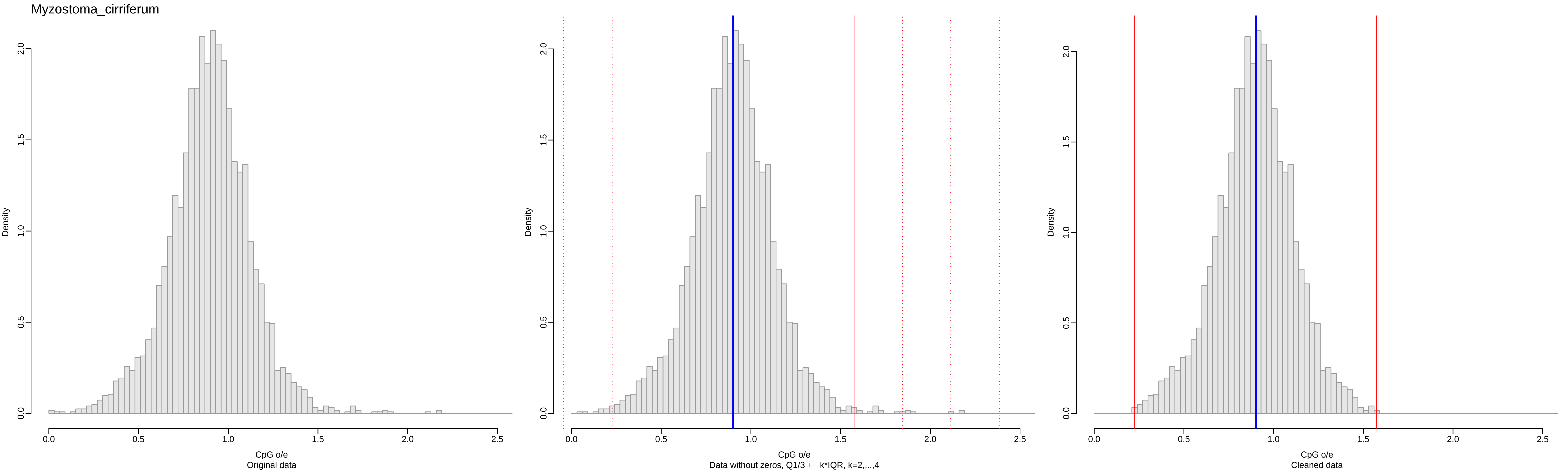

Myzostoma_cirriferum
Density
CpG o/e
Original data
Data without zeros, Q1/3 +− k*IQR, k=2,...,4
Cleaned data
0.0
0.5
1.0
1.5
2.0
2.5

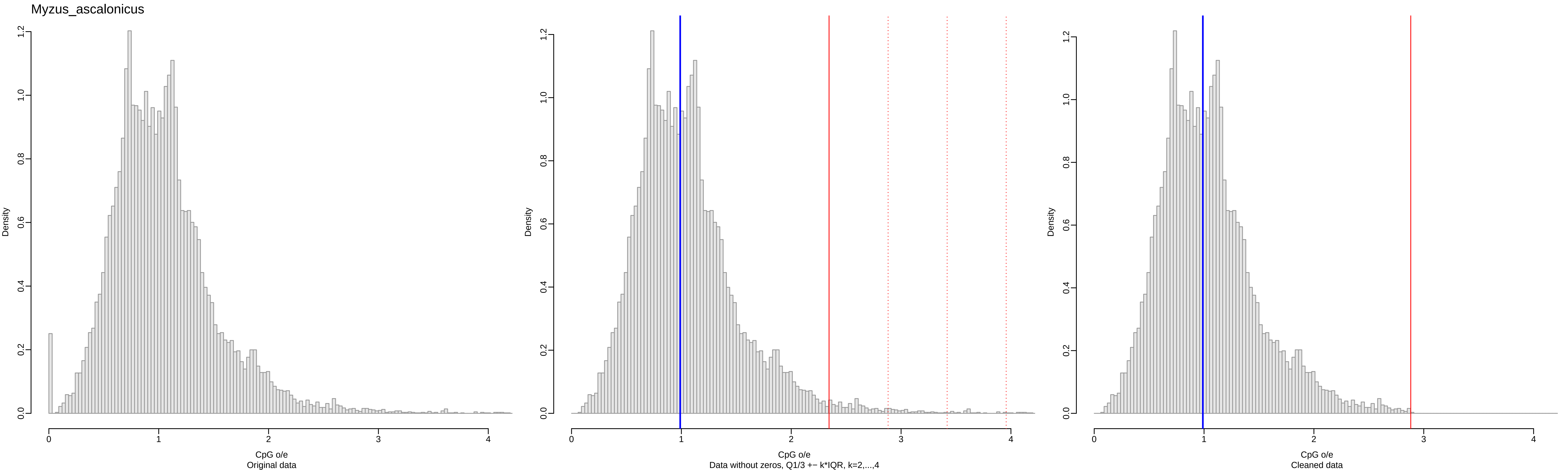
Myzus_ascalonicus
Density
CpG o/e
Original data
CpG o/e
Data without zeros, Q1/3 +− k*IQR, k=2,...,4
CpG o/e
Cleaned data

Myzus_persicae

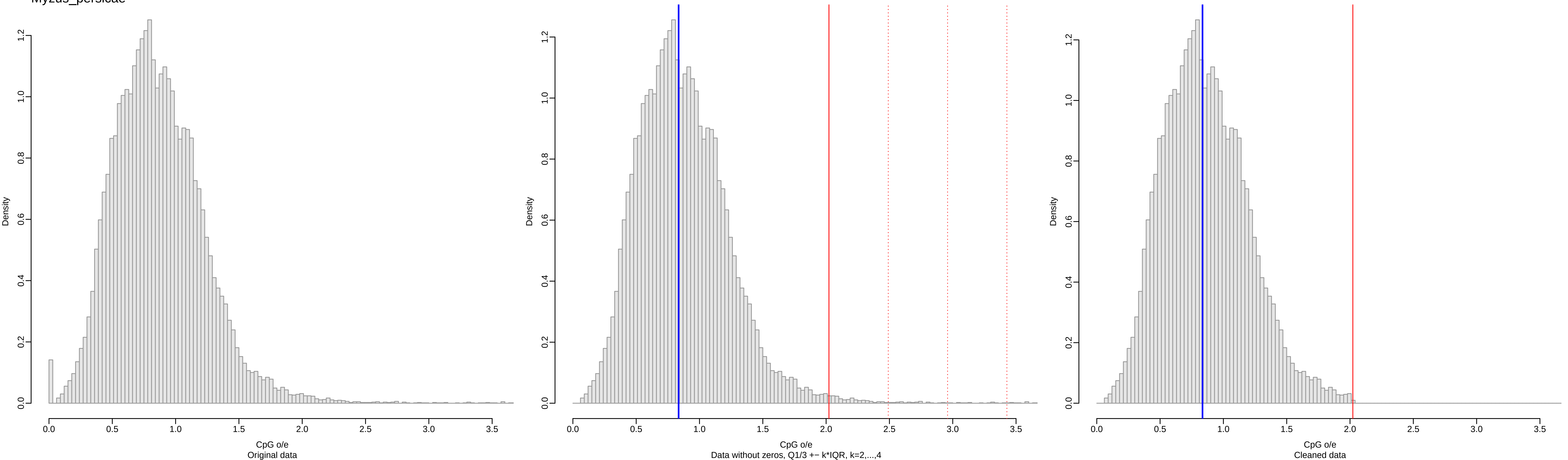

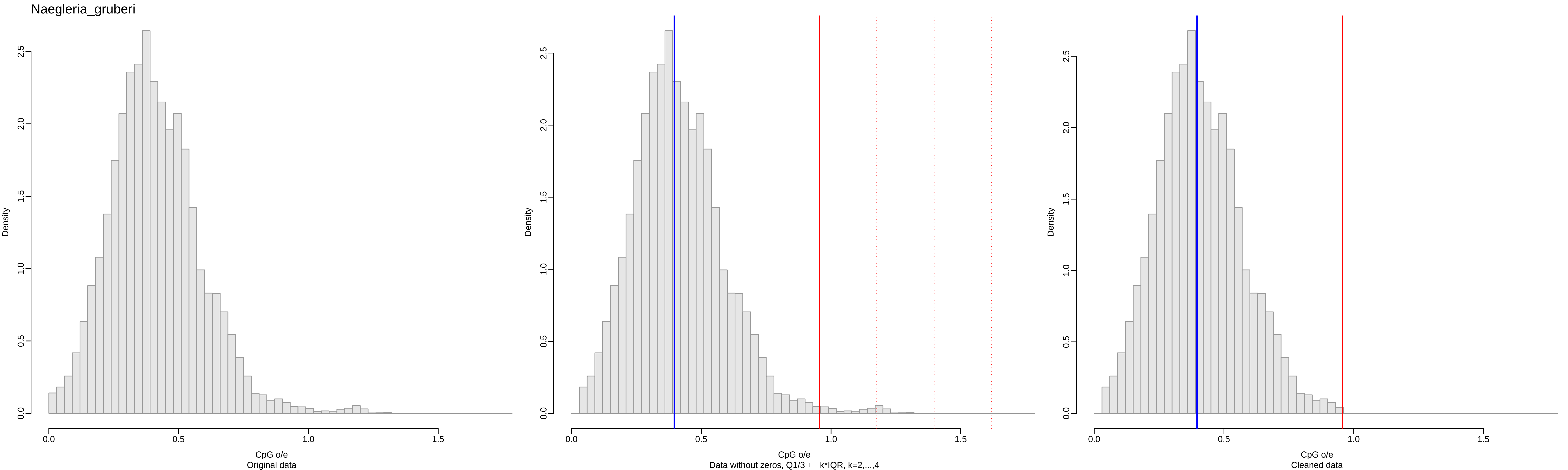
Naegleria_gruberi
Density
CpG o/e
Original data
Density
CpG o/e
Data without zeros, Q1/3 +− k*IQR, k=2,...,4
Density
CpG o/e
Cleaned data
0.0
0.5
1.0
1.5
2.0
2.5

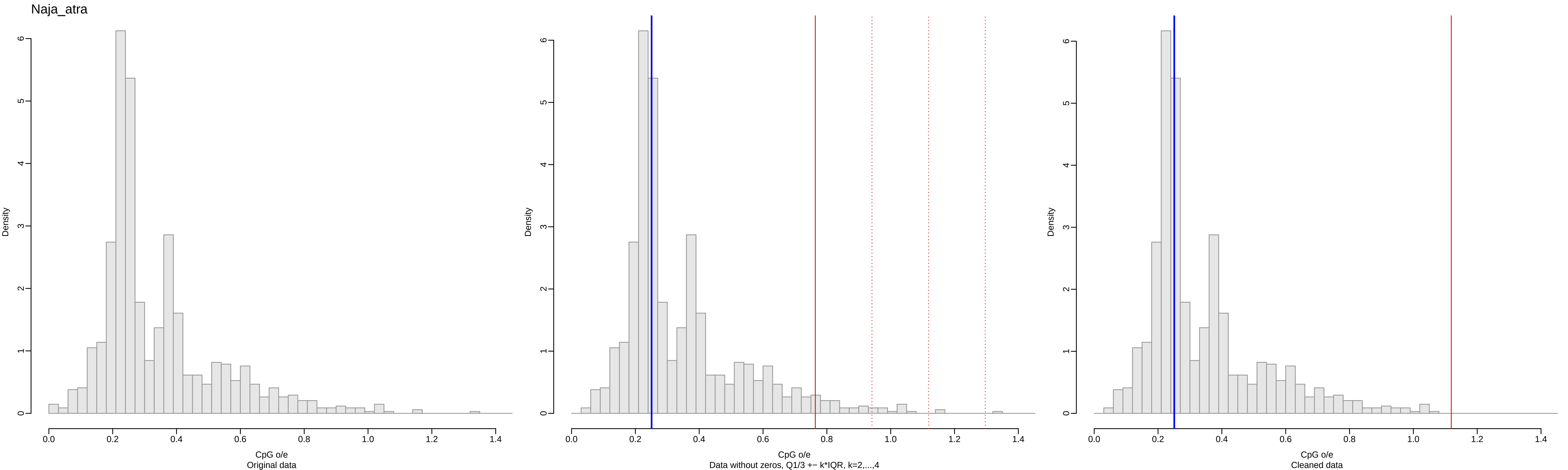

Naja_atra
Density
CpG o/e
Original data
Density
CpG o/e
Data without zeros, Q1/3 +− k*IQR, k=2,...,4
Density
CpG o/e
Cleaned data

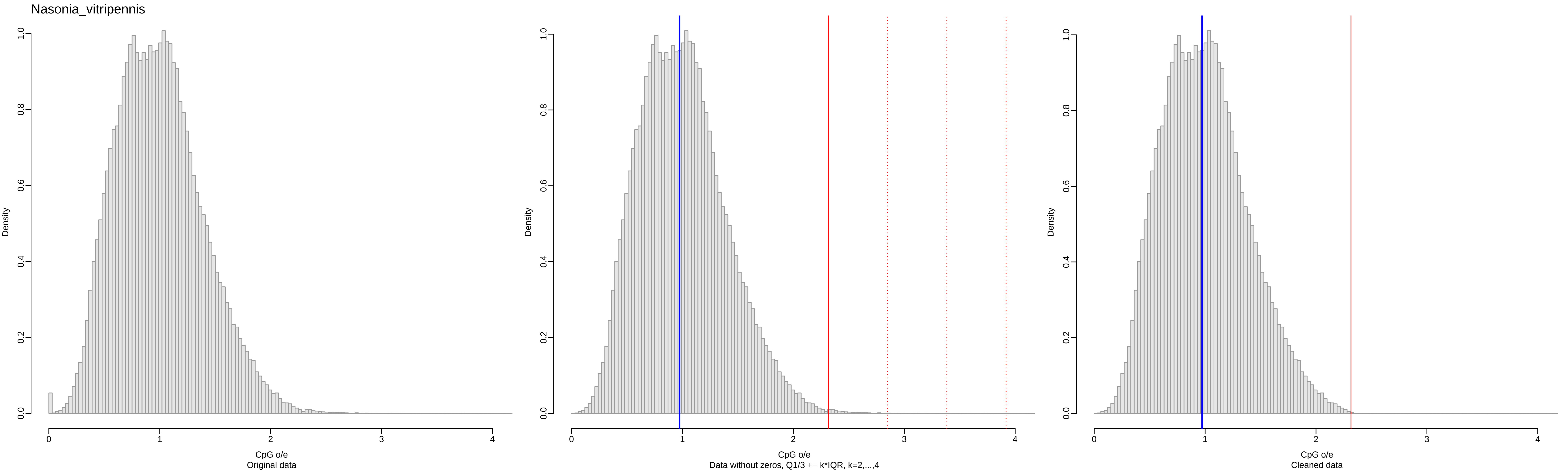
Nasonia_vitripennis
Density
CpG o/e
Original data
CpG o/e
Data without zeros, Q1/3 +− k*IQR, k=2,...,4
CpG o/e
Cleaned data
0.0
0.2
0.4
0.6
0.8
1.0
0
1
2
3
4

Necator_americanus

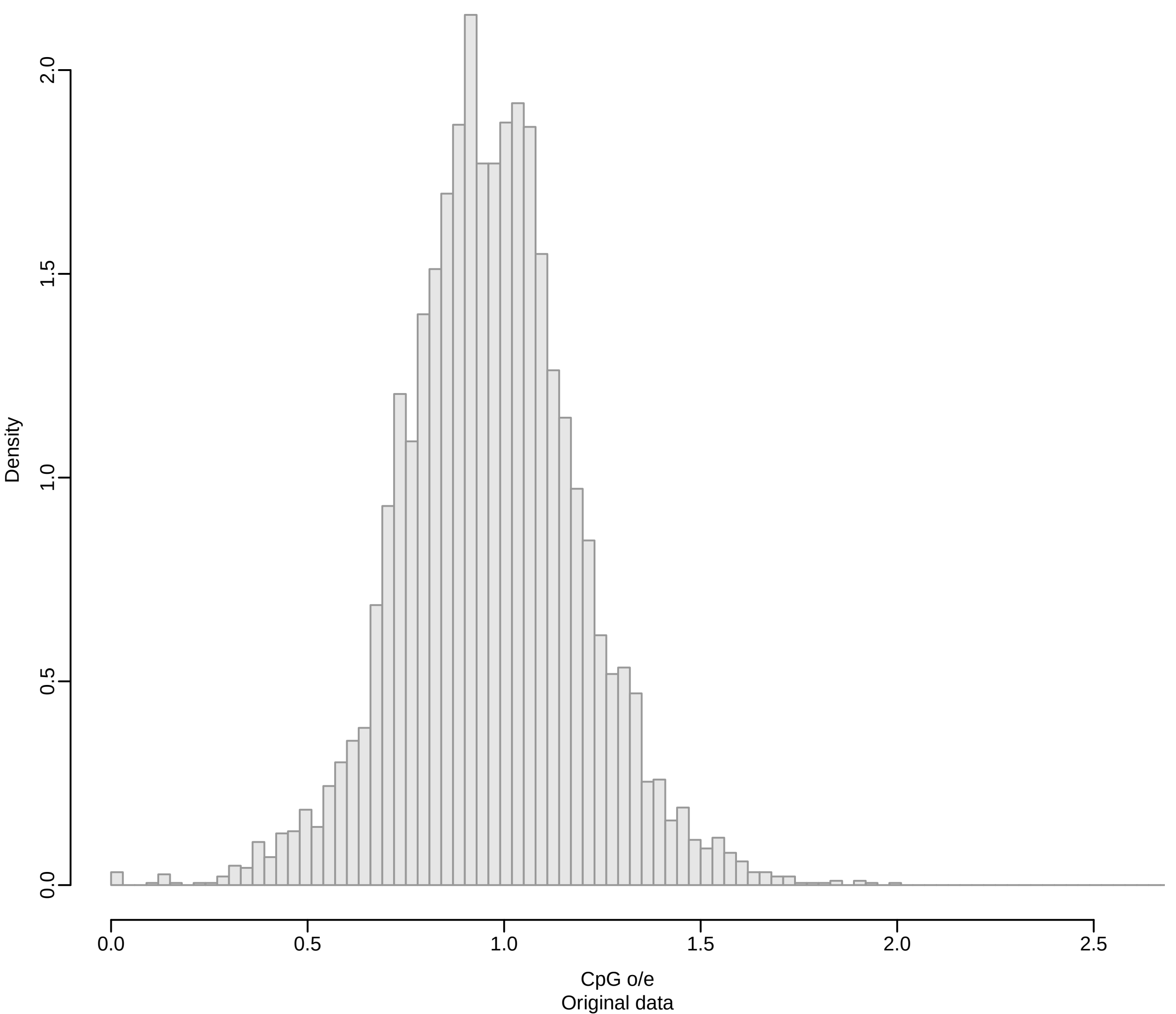

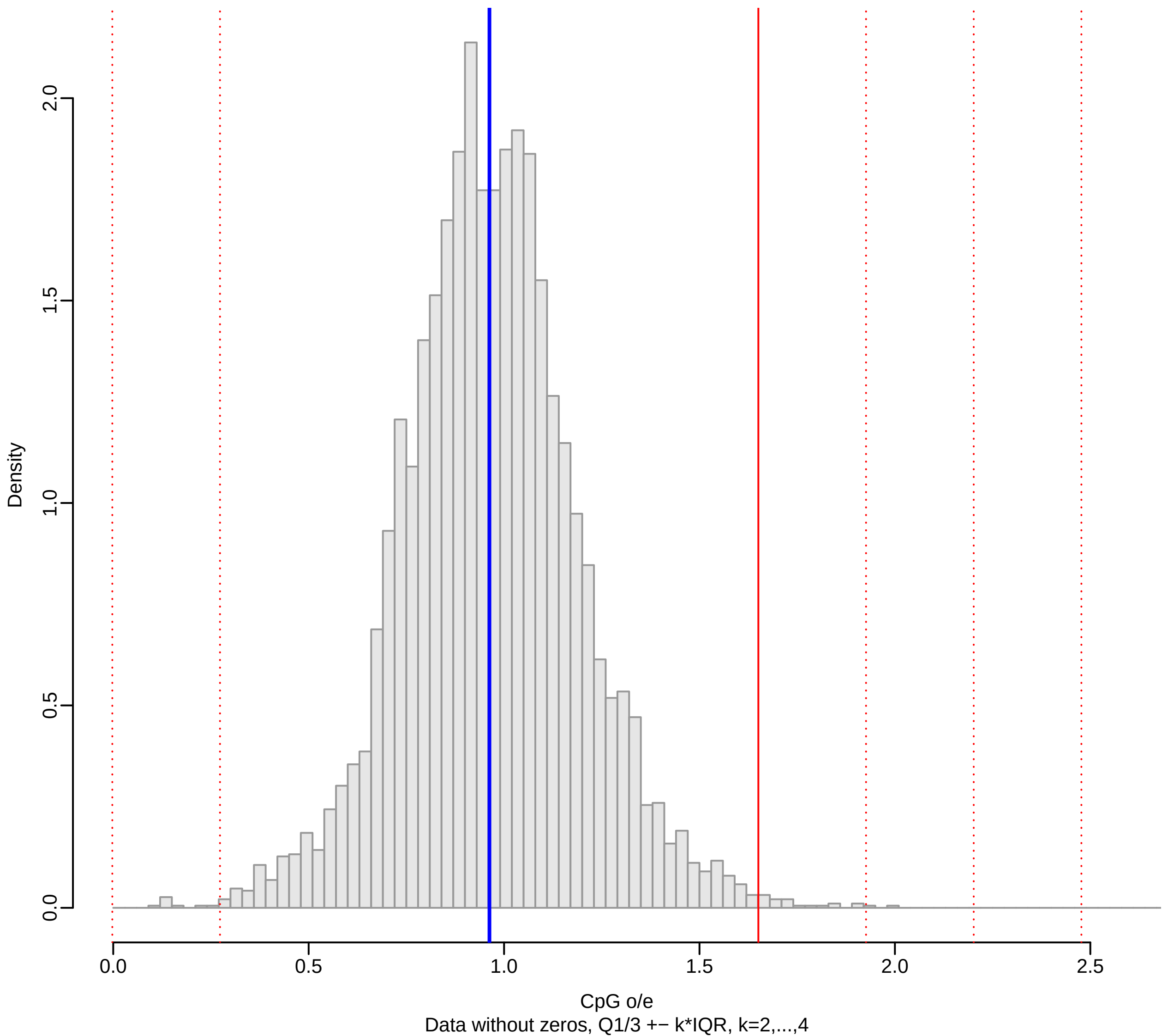

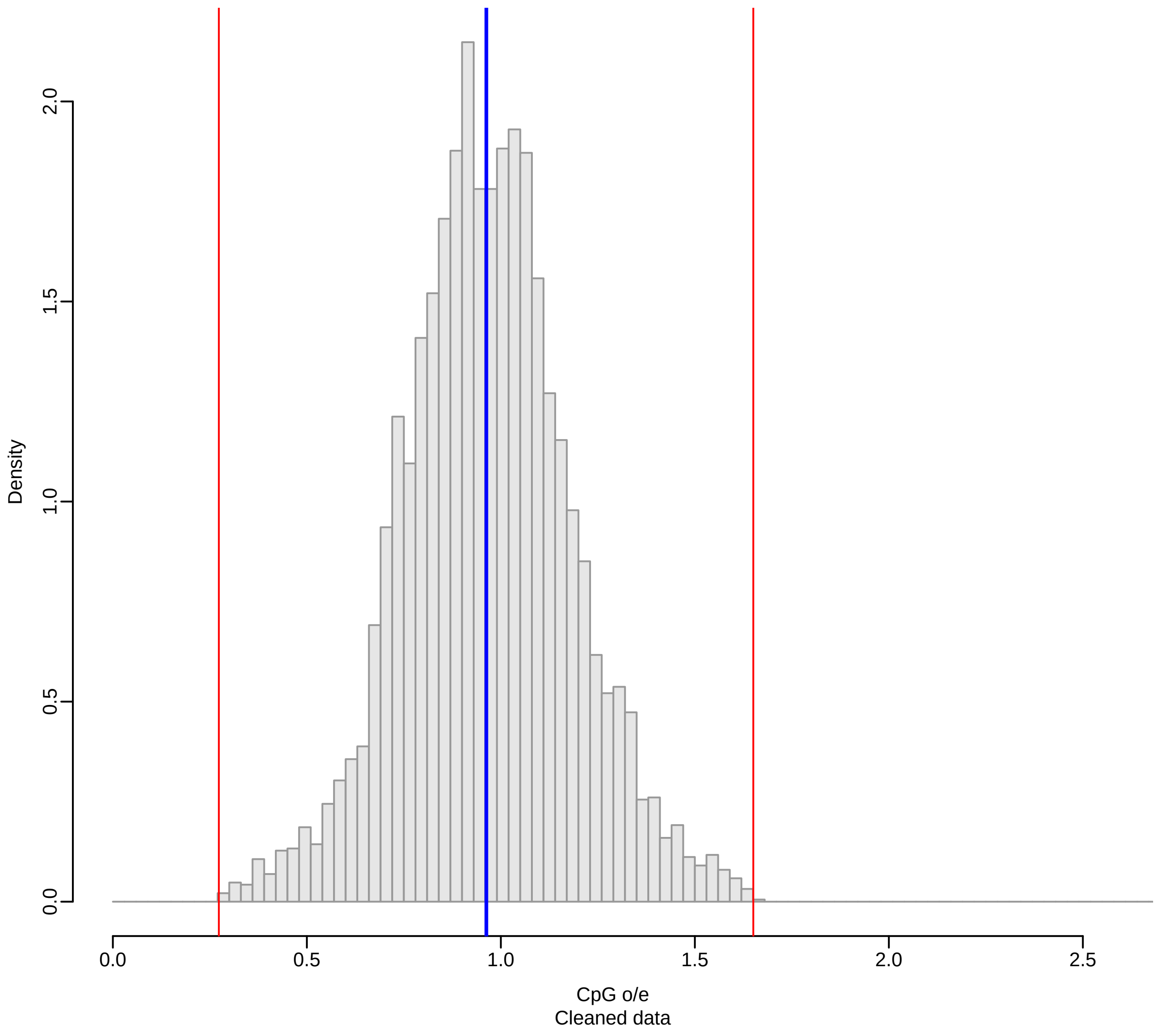

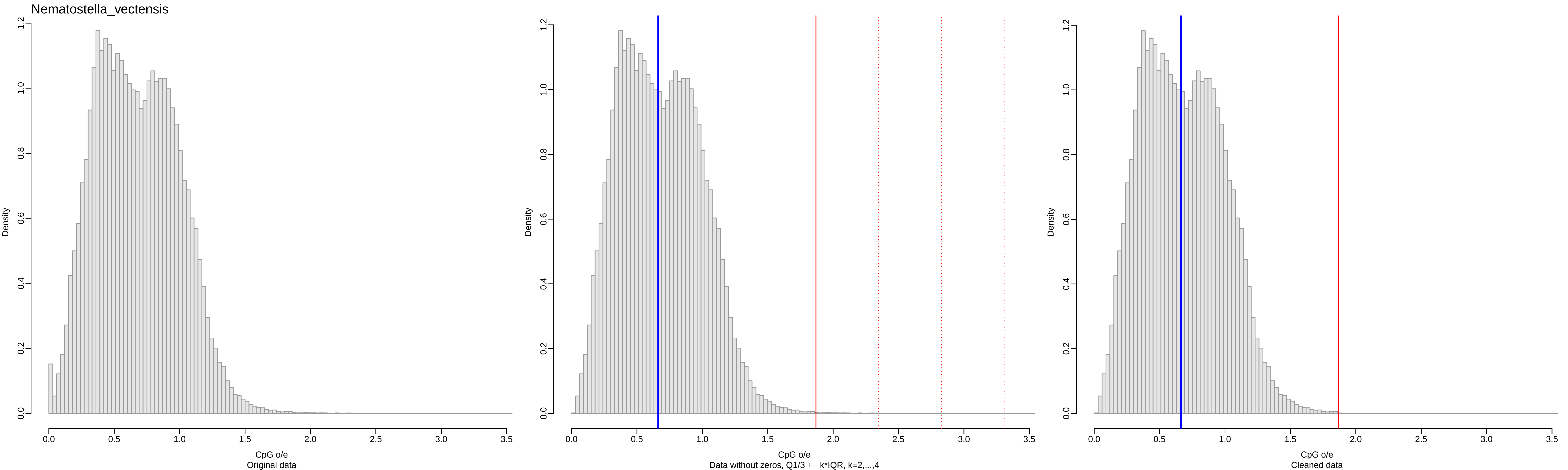

Nematostella_vectensis
Density
CpG o/e
Original data
Data without zeros, Q1/3 +− k*IQR, k=2,...,4
Cleaned data
0.0
0.2
0.4
0.6
0.8
1.0
1.2
0.5
1.5
2.0
2.5
3.0
3.5

Neotyphodium_lolii

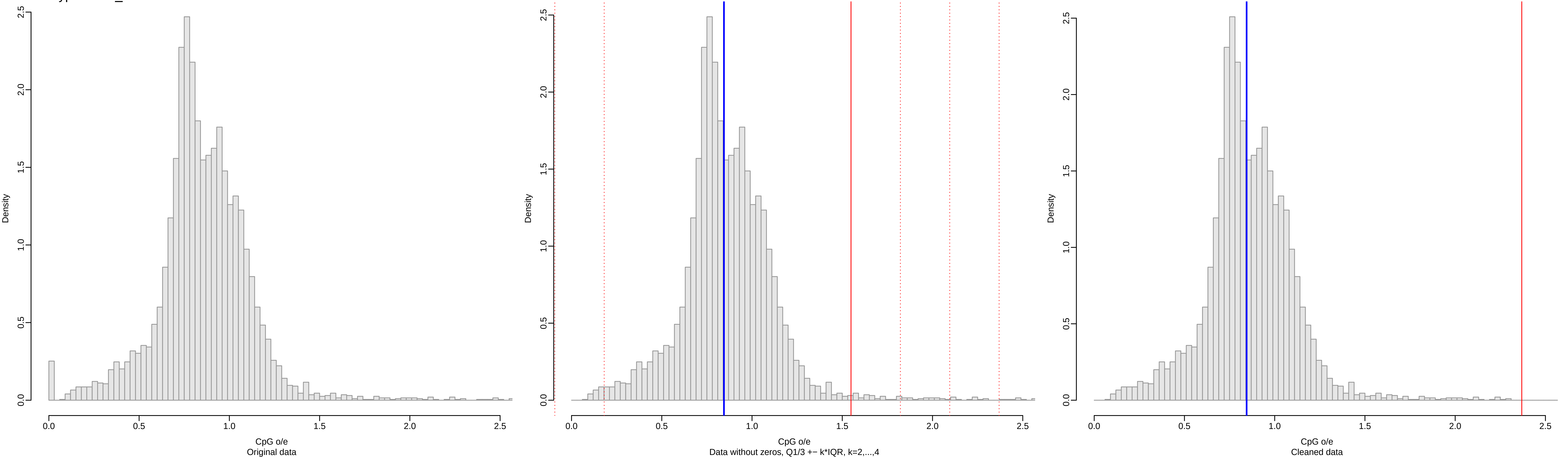

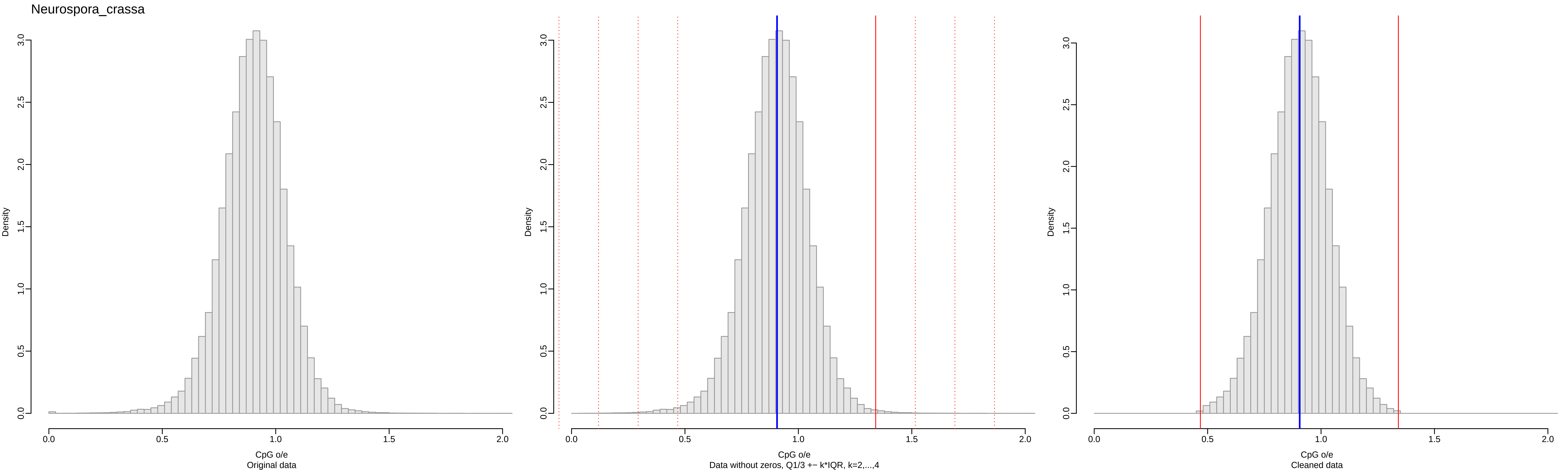

Neurospora_crassa
Density
CpG o/e
Original data
Data without zeros, Q1/3 +− k*IQR, k=2,...,4
Cleaned data

Nicotiana_sylvestris

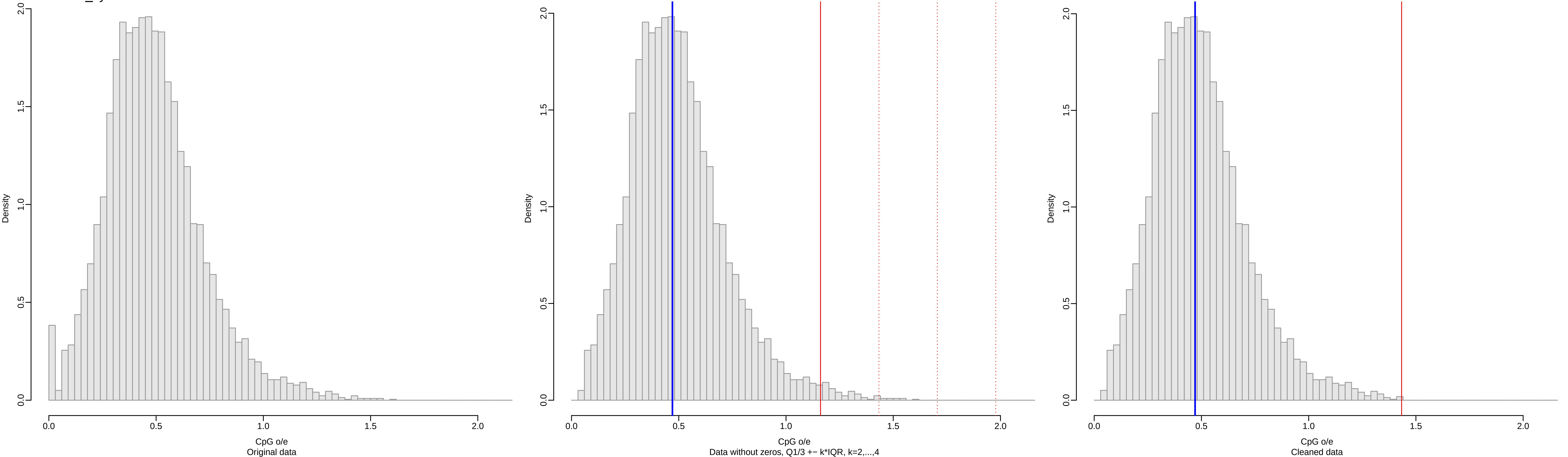

Nicotiana_tabacum

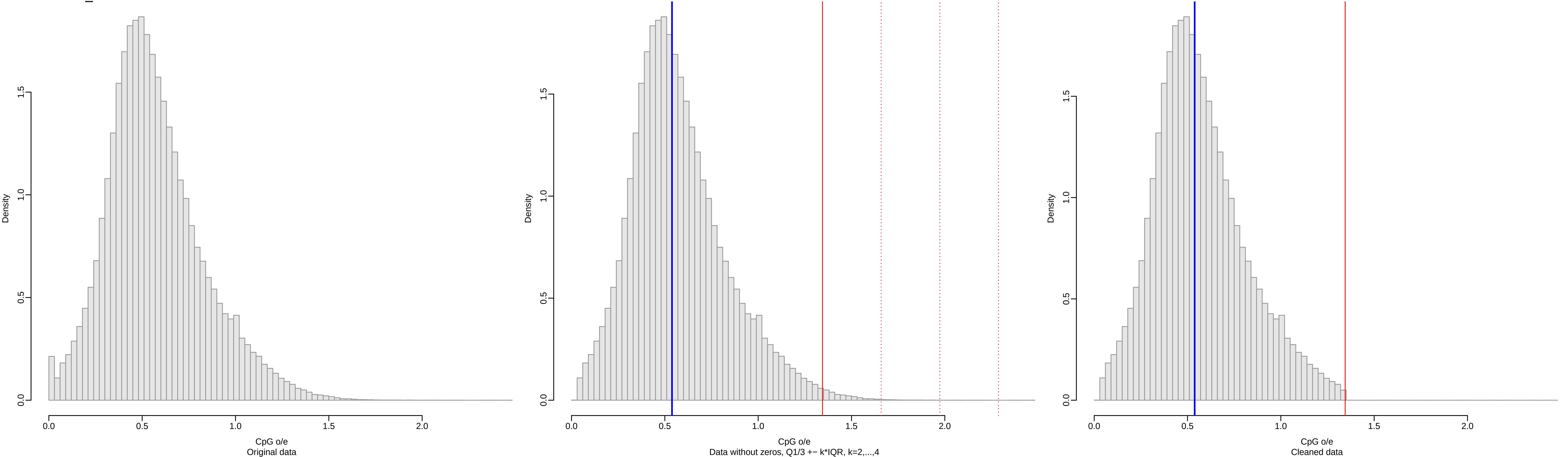

Nilaparvata_lugens

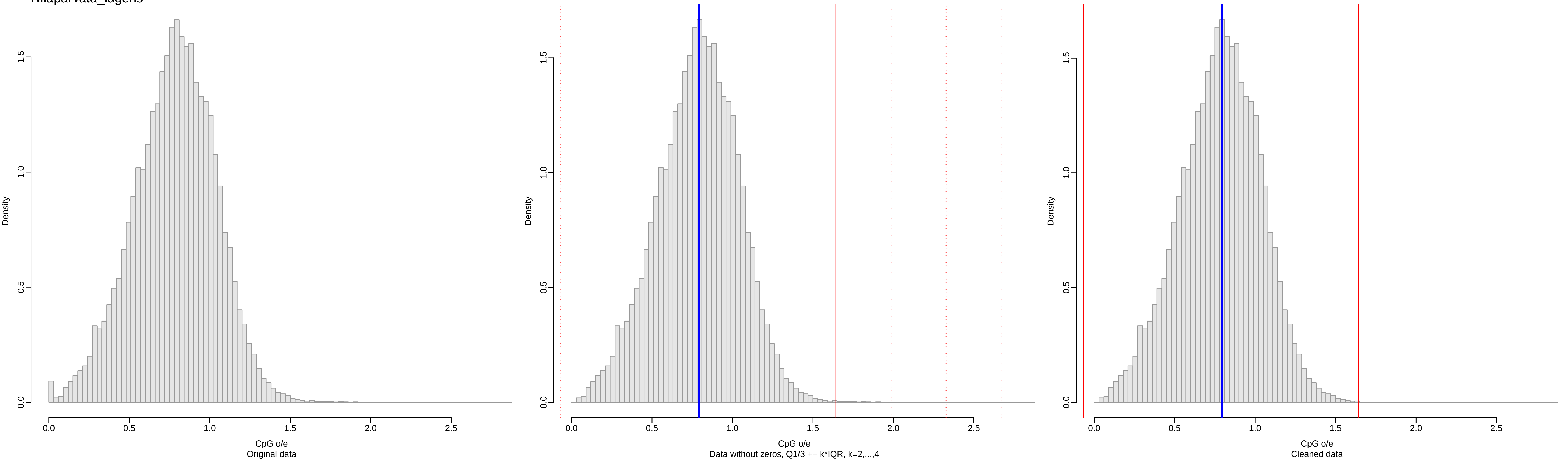

Nippostrongylus_brasiliensis

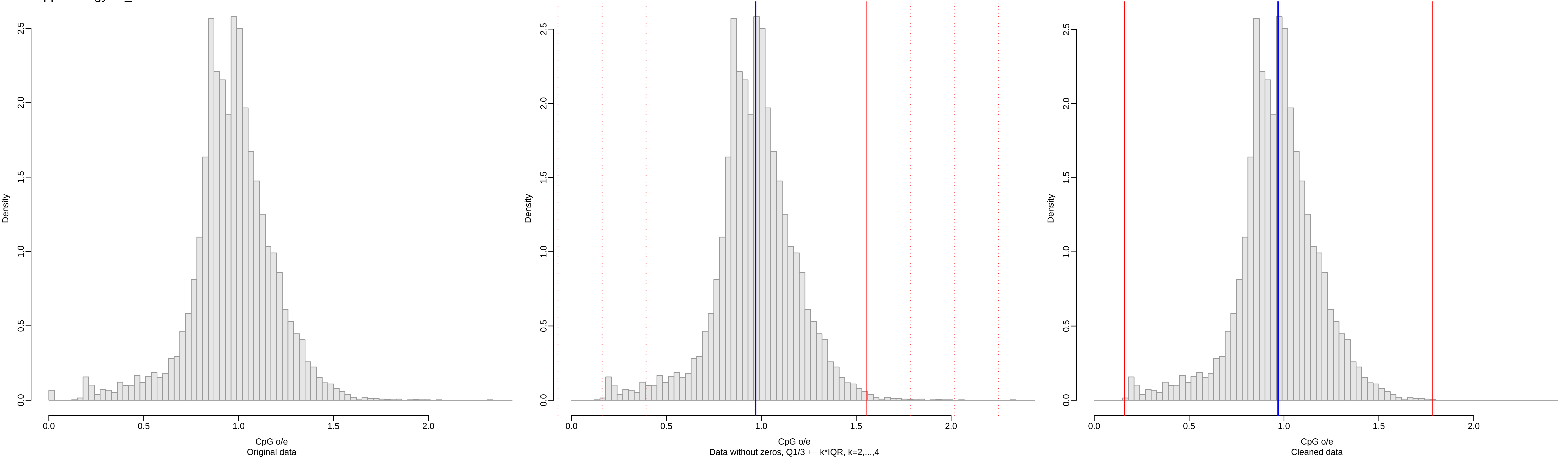

Nitella_hyalina

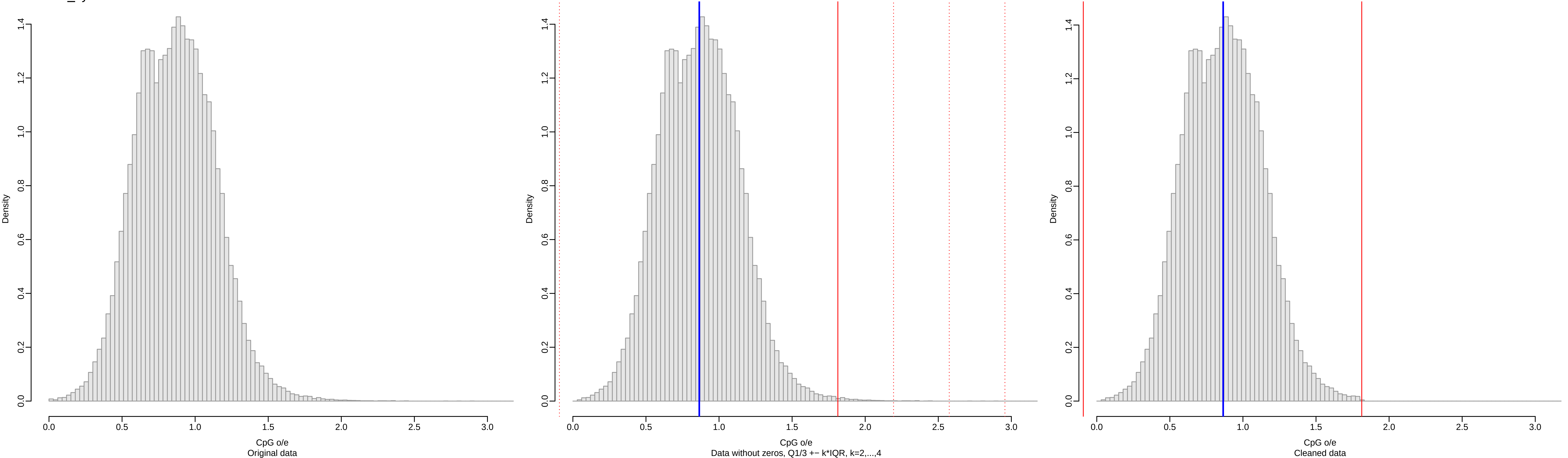

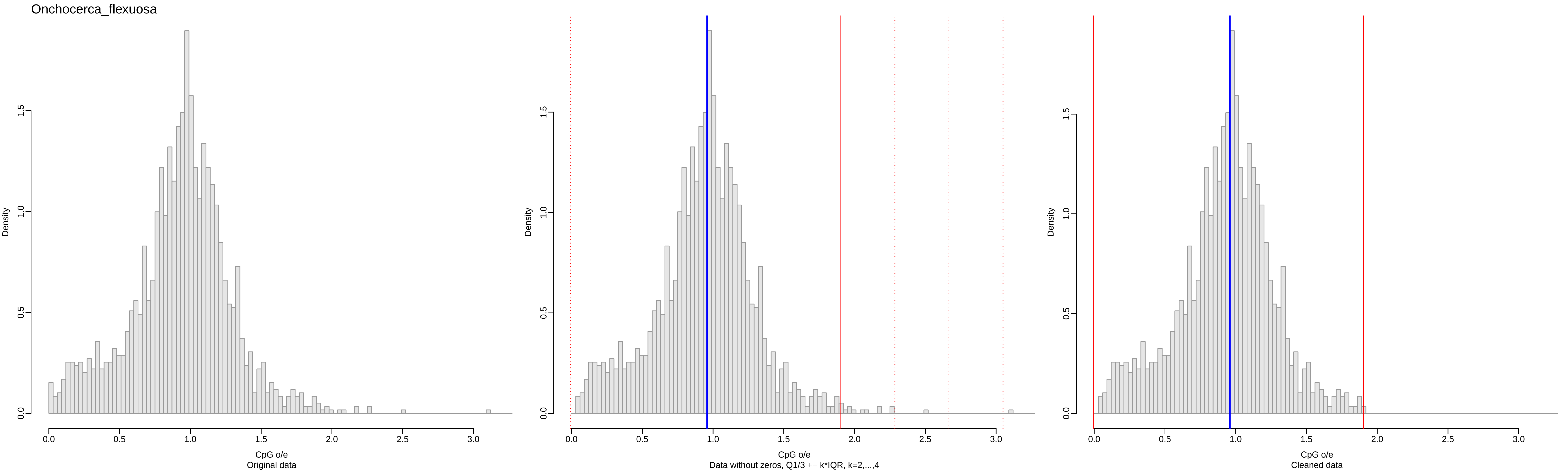

Onchocerca_flexuosa
Density
CpG o/e
Original data
Data without zeros, Q1/3 +− k*IQR, k=2,...,4
Cleaned data
0.0
0.5
1.0
1.5
2.0
2.5
3.0

Onchocerca_volvulus

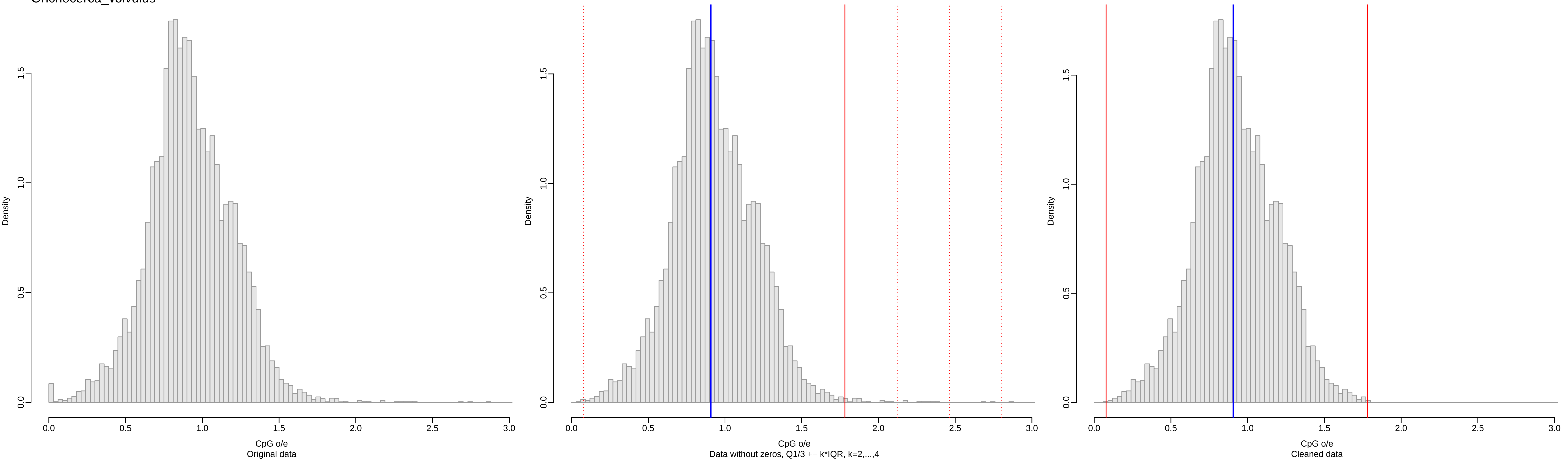

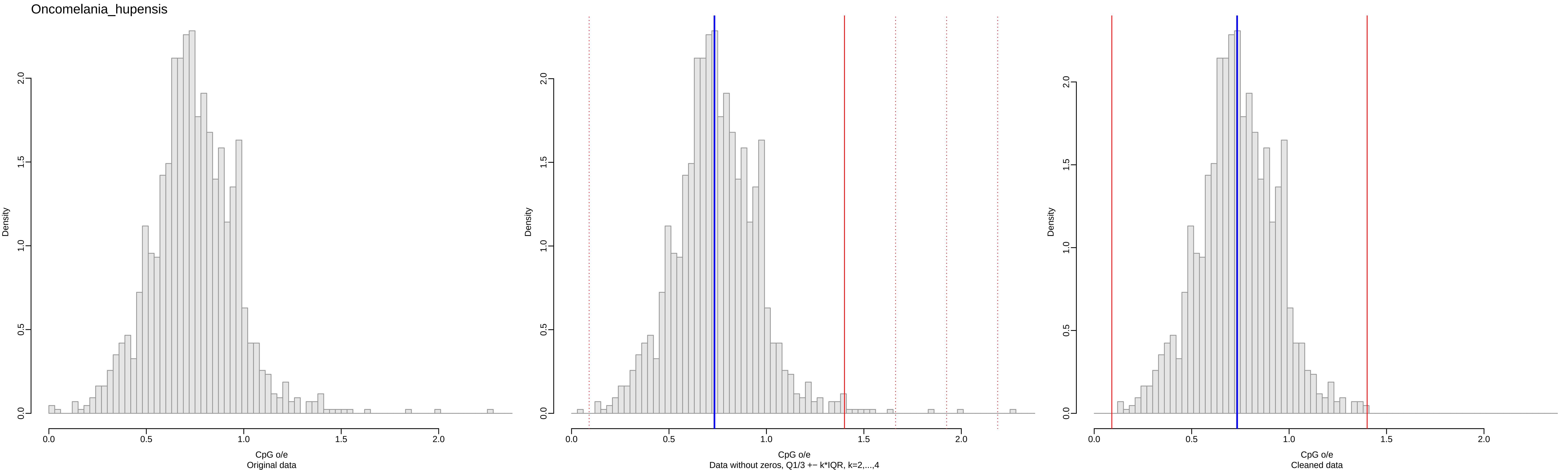

Oncomelania_hupensis
Density
CpG o/e
Original data
Data without zeros, Q1/3 +− k*IQR, k=2,...,4
Cleaned data
0.0
0.5
1.0
1.5
2.0

Oncorhynchus_kisutch

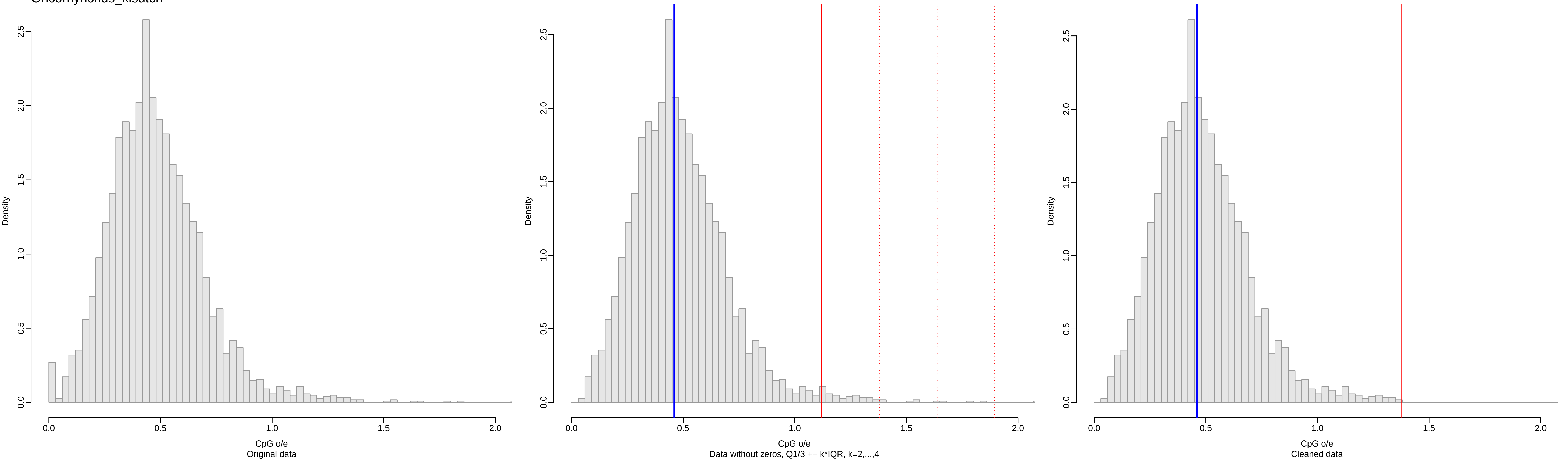

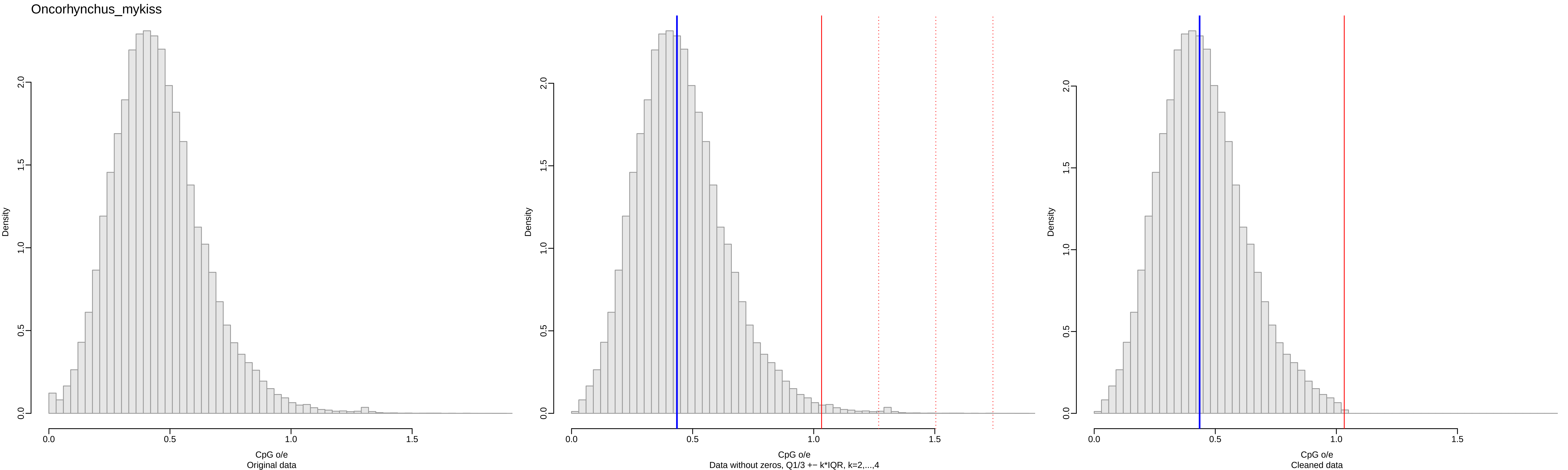

Oncorhynchus_mykiss
Density
CpG o/e
Original data
Data without zeros, Q1/3 +− k*IQR, k=2,...,4
Cleaned data
0.0
0.5
1.0
1.5
2.0

Oncorhynchus_nerka

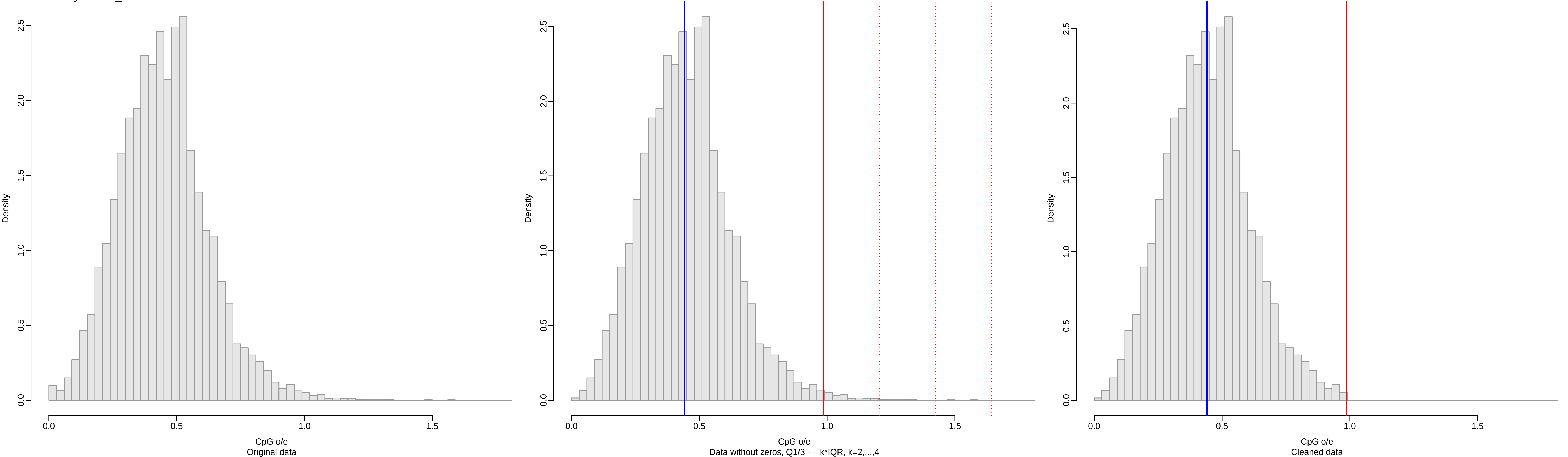

Oncorhynchus_tshawytscha

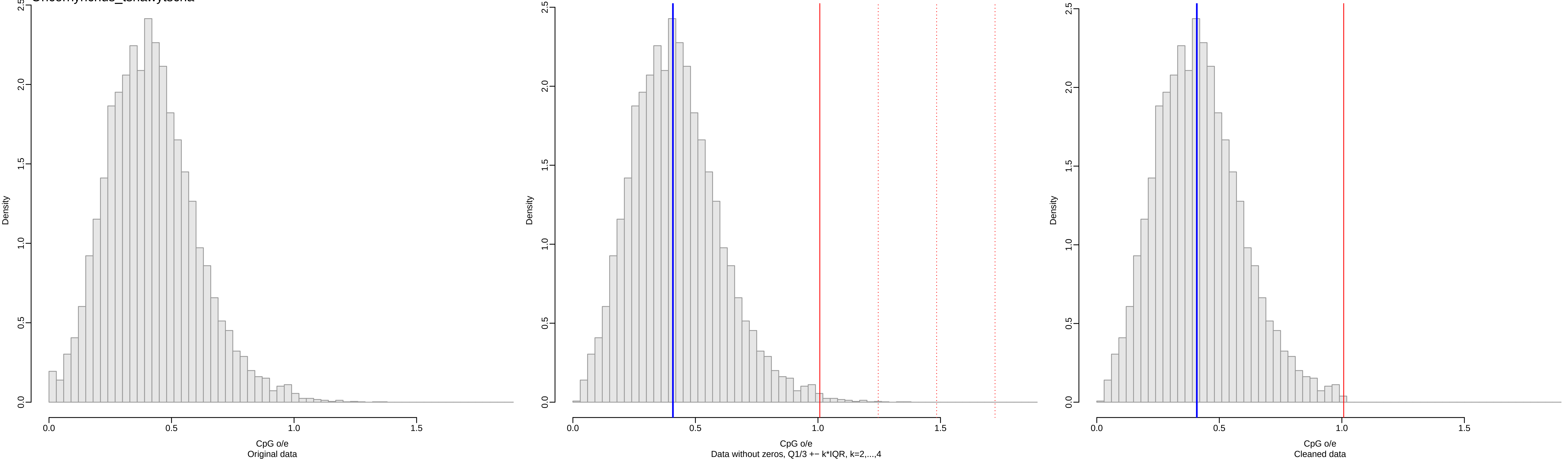

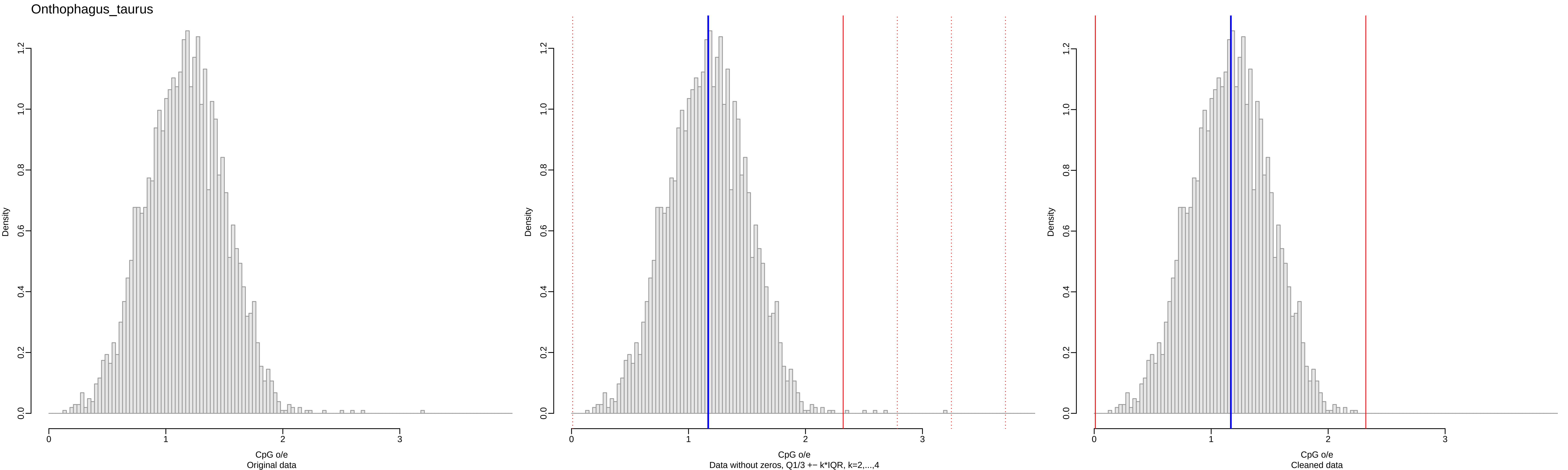

Onthophagus_taurus
Density
CpG o/e
Original data
CpG o/e
Data without zeros, Q1/3 +− k*IQR, k=2,...,4
CpG o/e
Cleaned data

Onychiurus_arcticus

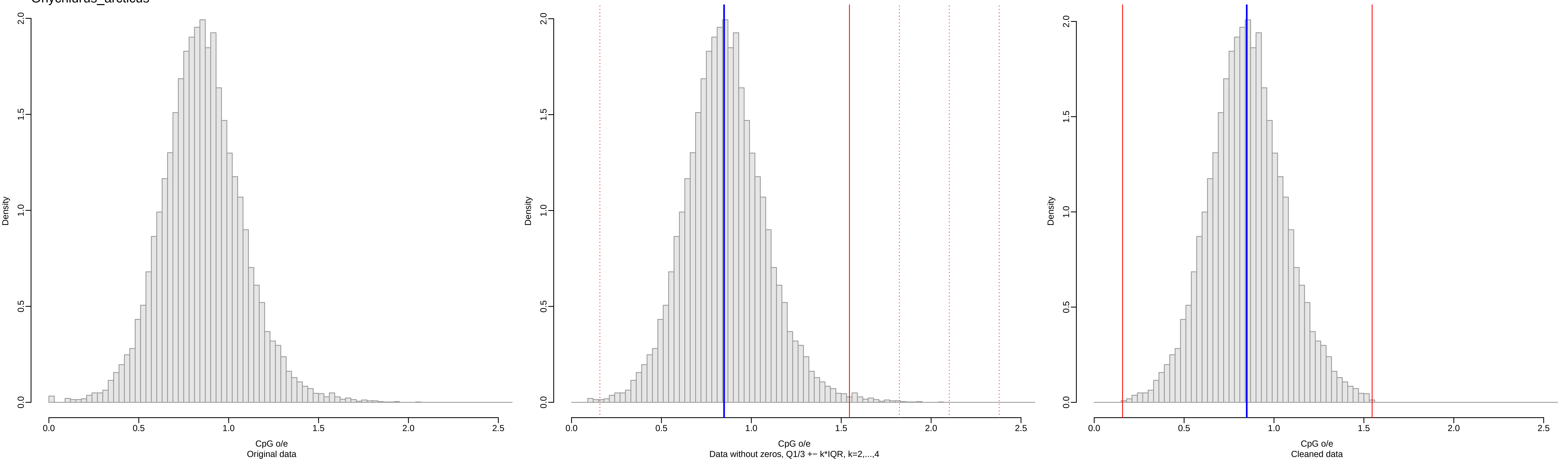

Ophiostoma_novo−ulmi

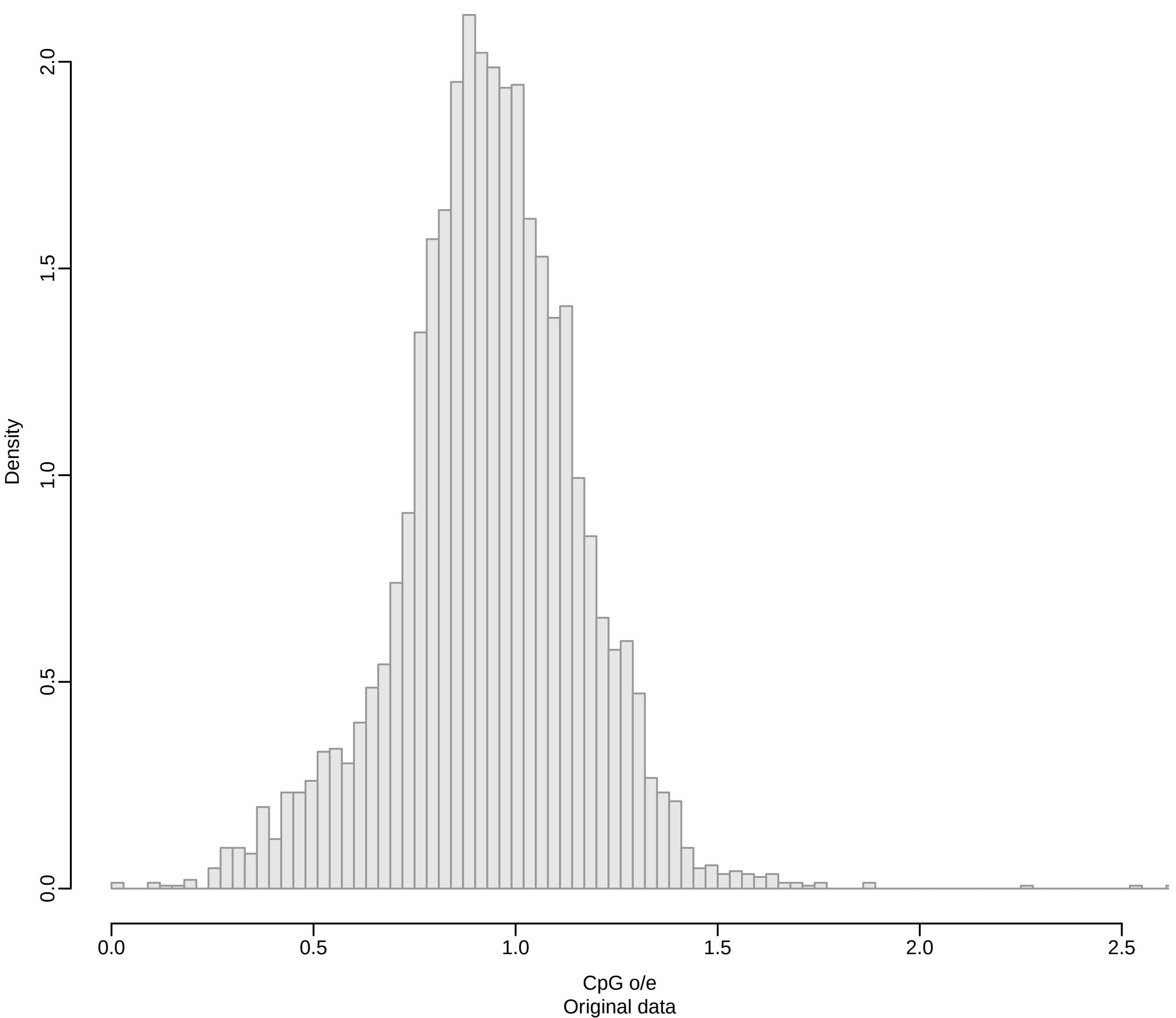

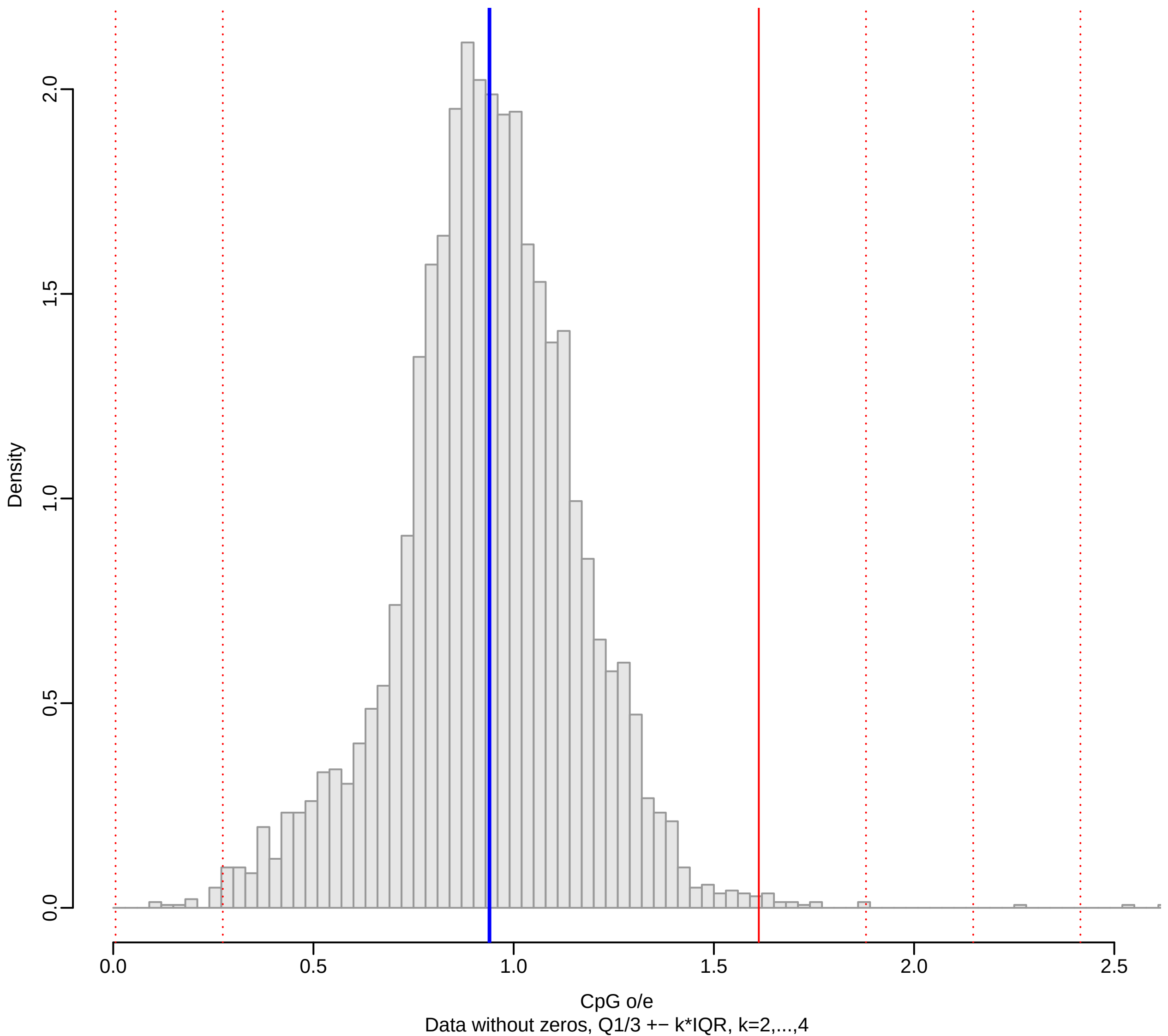

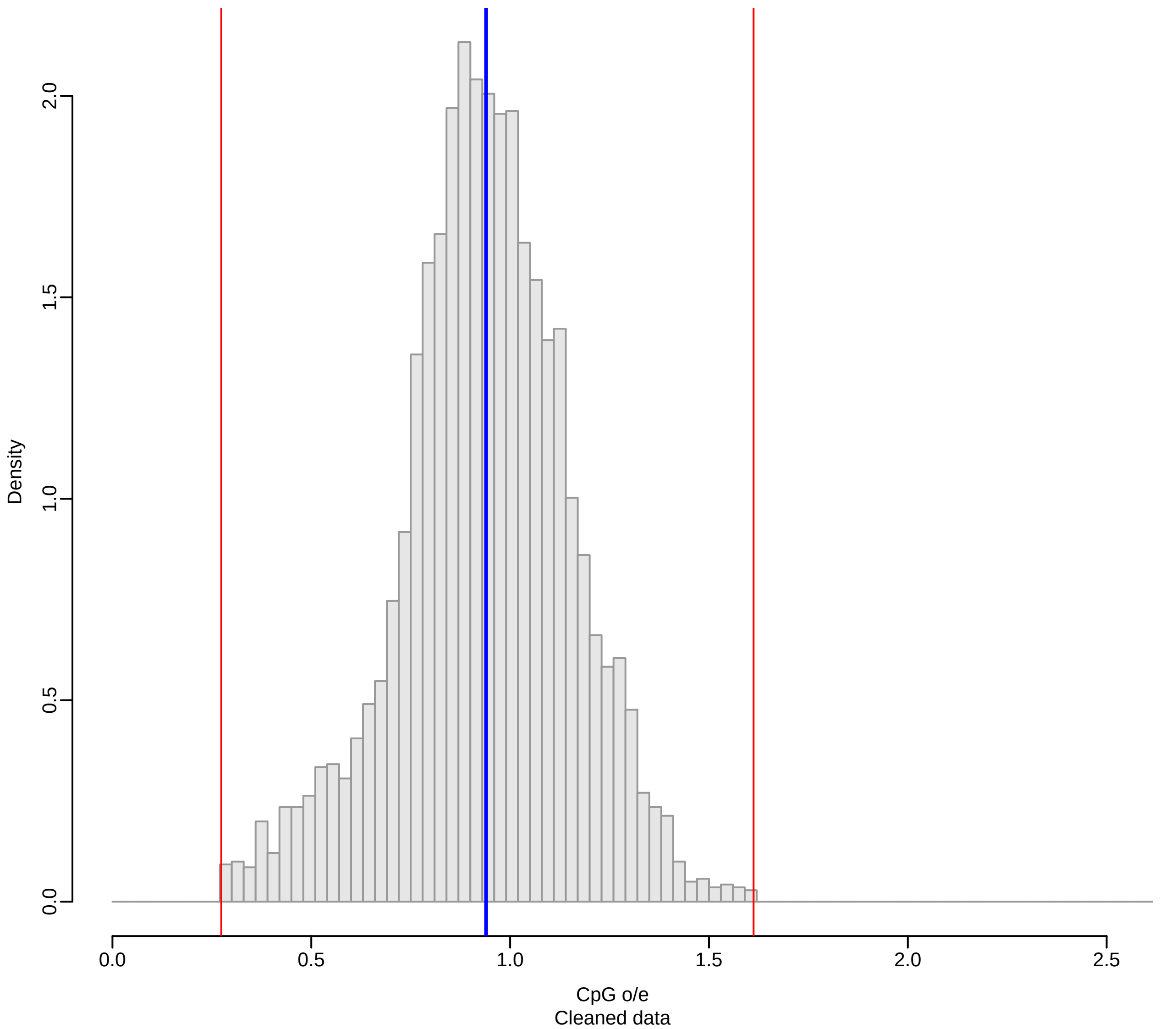

Ophryotrocha_puerilis

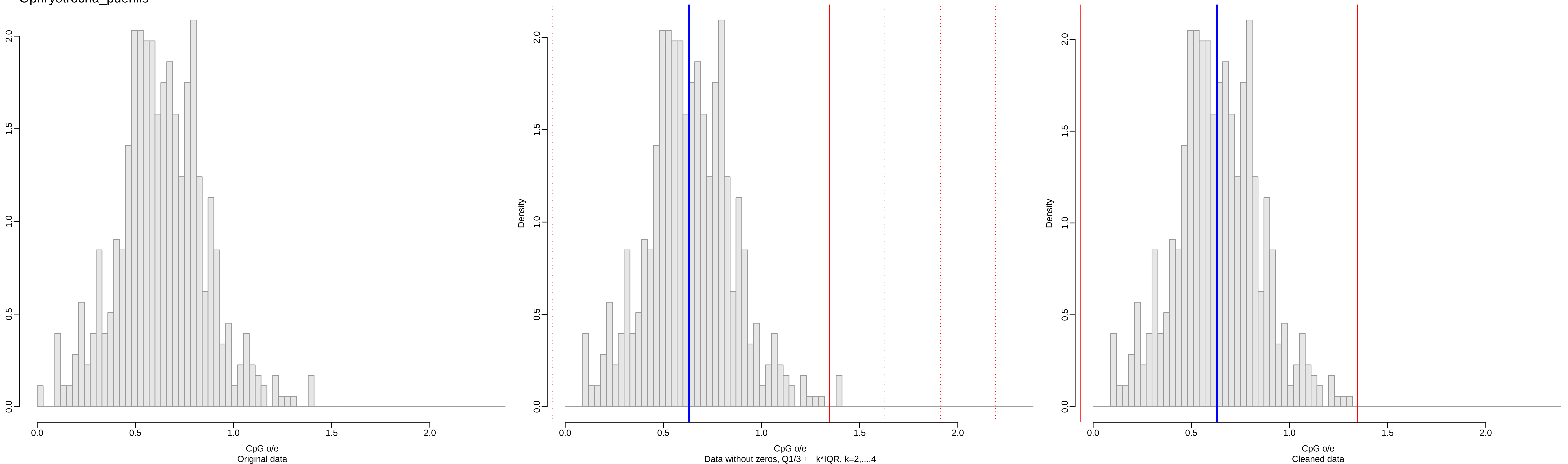

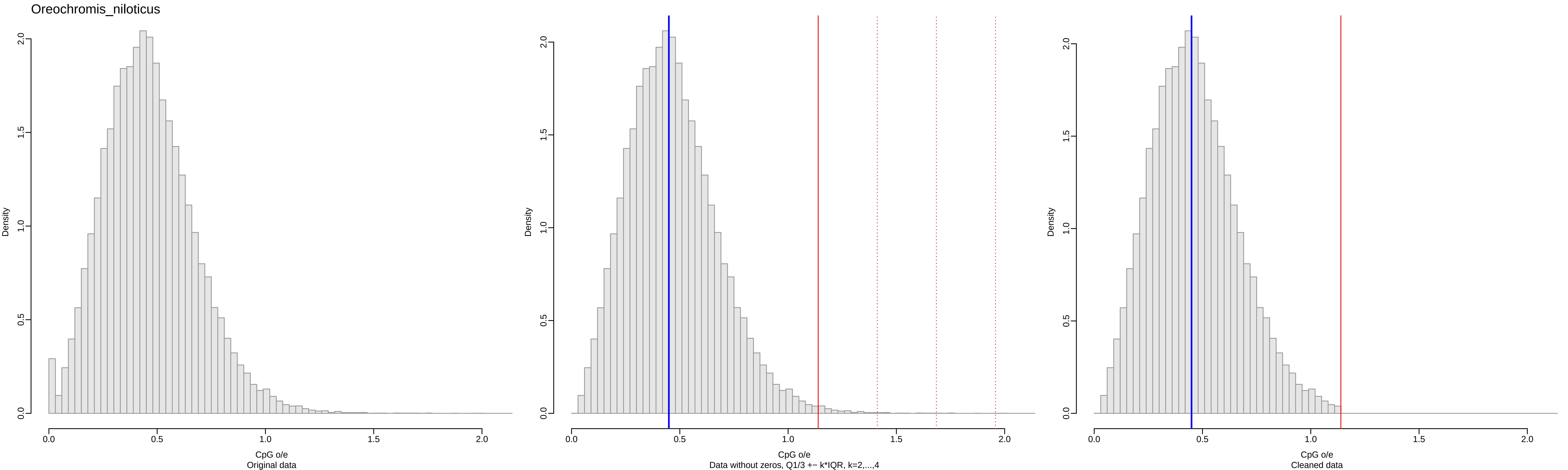
Oreochromis_niloticus
Density
CpG o/e
Original data
Density
CpG o/e
Data without zeros, Q1/3 +− k*IQR, k=2,...,4
Density
CpG o/e
Cleaned data

Ornithorhynchus_anatinus

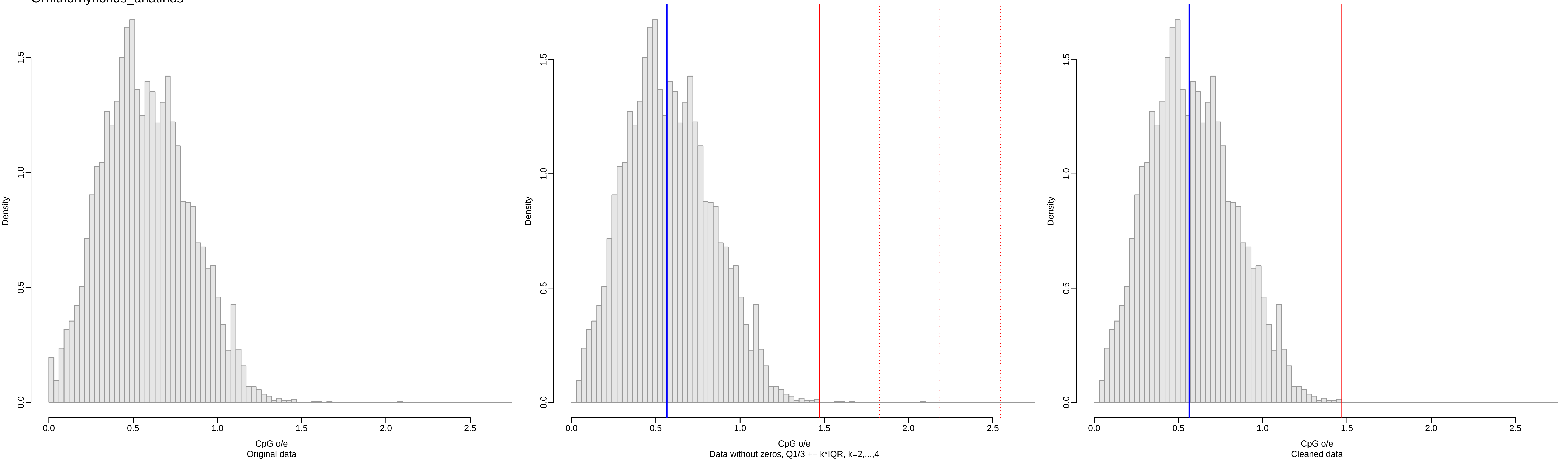

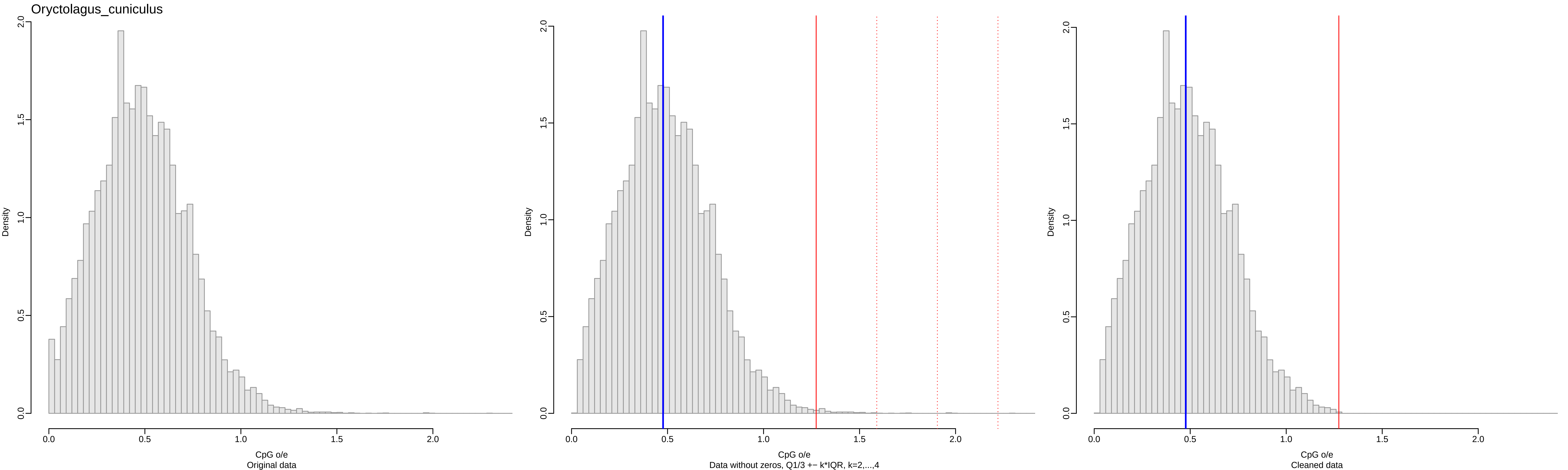

Oryctolagus_cuniculus
Density
0.0
0.5
1.0
1.5
2.0
CpG o/e
Original data
Data without zeros, Q1/3 +− k*IQR, k=2,...,4
Cleaned data

Oryza_longistaminata

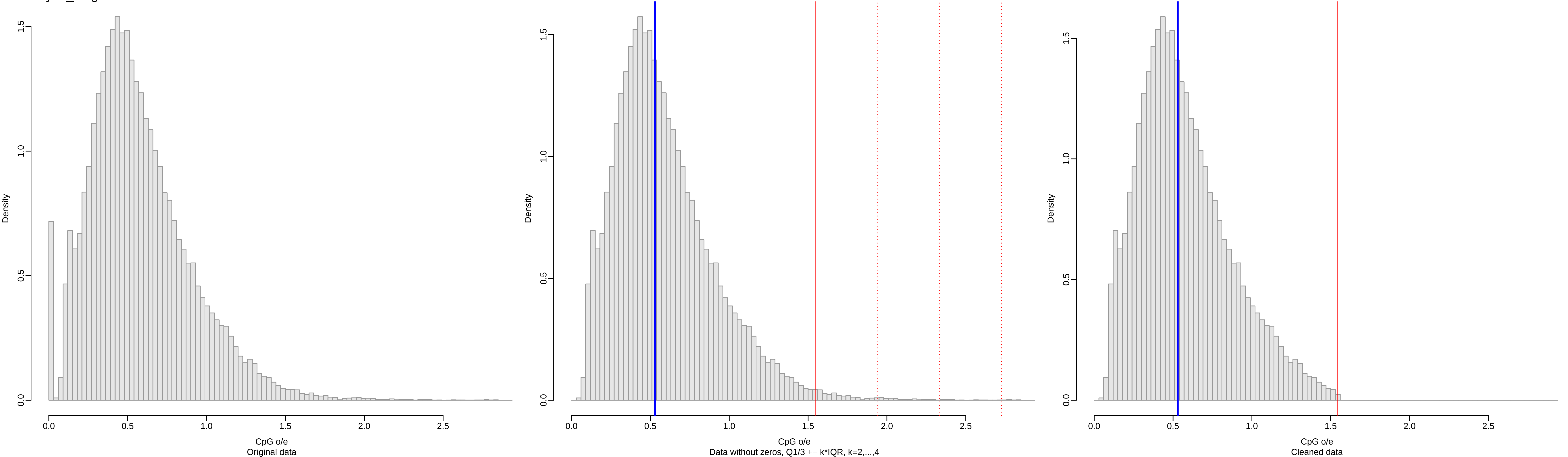

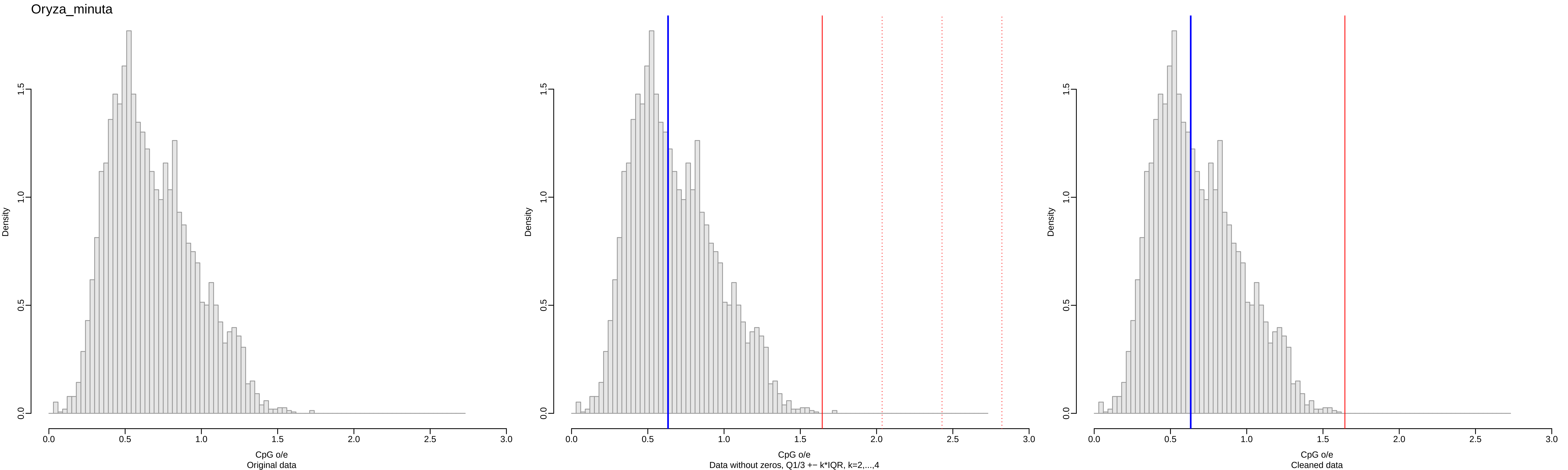
Oryza_minuta
Density
0.0
0.5
1.0
1.5
0.0
0.5
1.0
1.5
2.0
2.5
3.0
CpG o/e
Original data
CpG o/e
Data without zeros, Q1/3 +− k*IQR, k=2,...,4
CpG o/e
Cleaned data

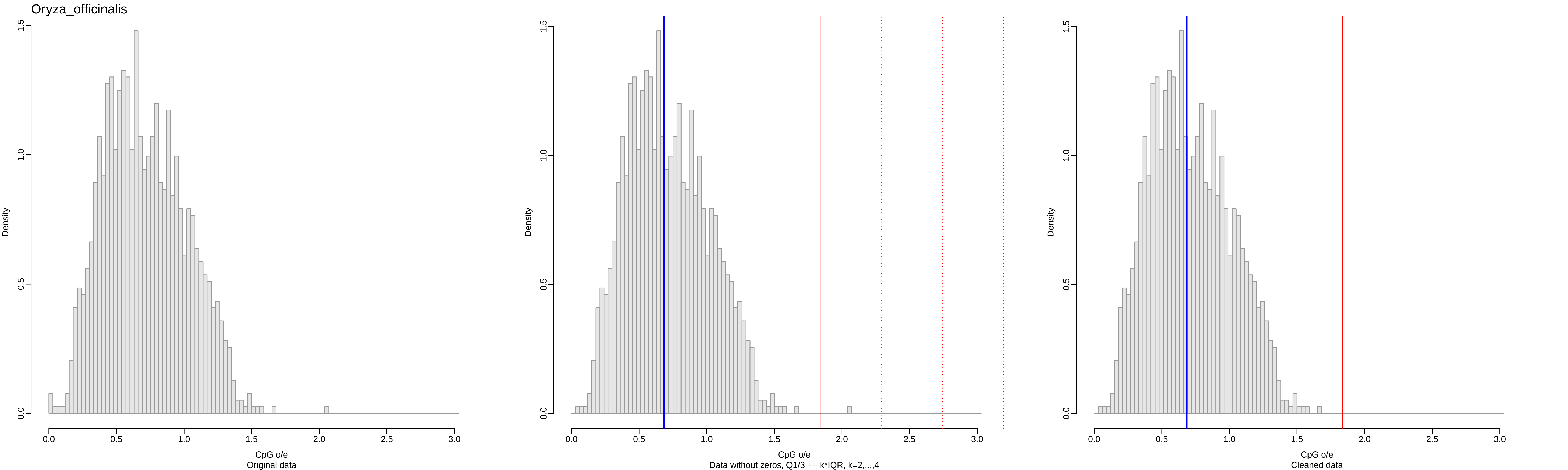

Oryza_officinalis
Density
CpG o/e
Original data
Data without zeros, Q1/3 +− k*IQR, k=2,...,4
Cleaned data
0.0
0.5
1.0
1.5
2.0
2.5
3.0

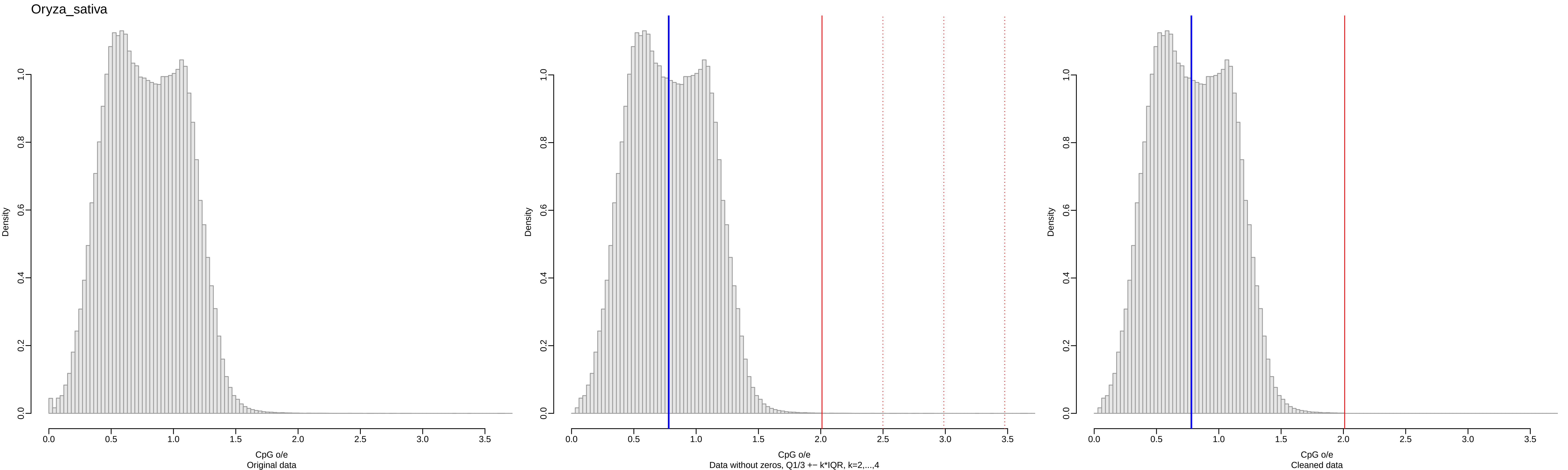
Oryza_sativa
Density
CpG o/e
Original data
Density
CpG o/e
Data without zeros, Q1/3 +− k*IQR, k=2,...,4
Density
CpG o/e
Cleaned data

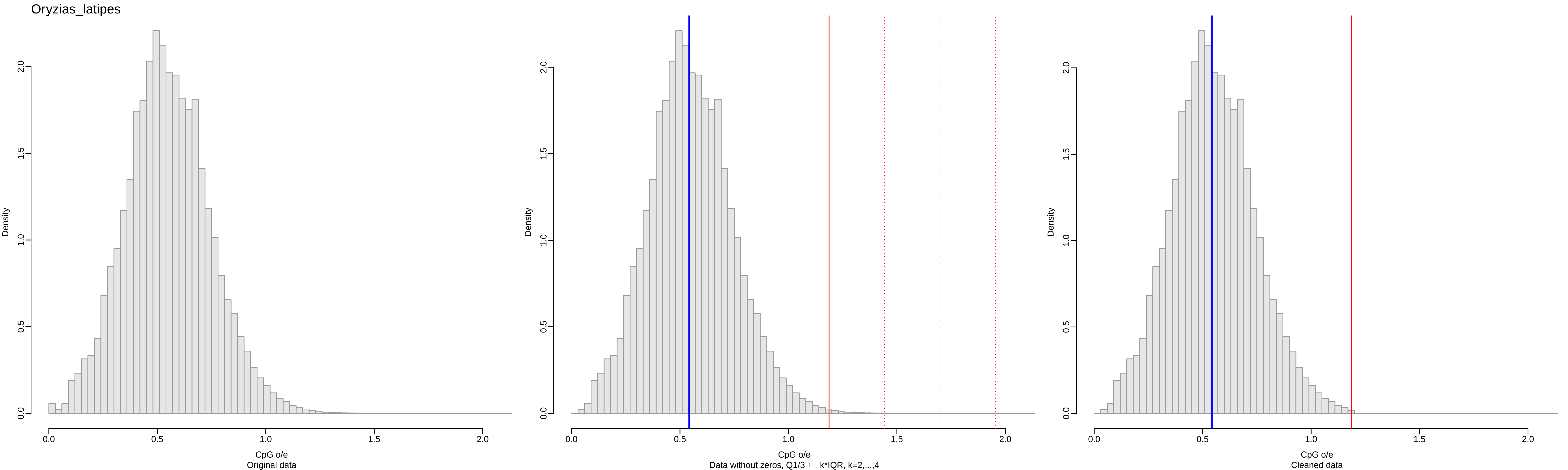
Oryzias_latipes
Density
0.0
0.5
1.0
1.5
2.0
CpG o/e
Original data
Data without zeros, Q1/3 +− k*IQR, k=2,...,4
Cleaned data

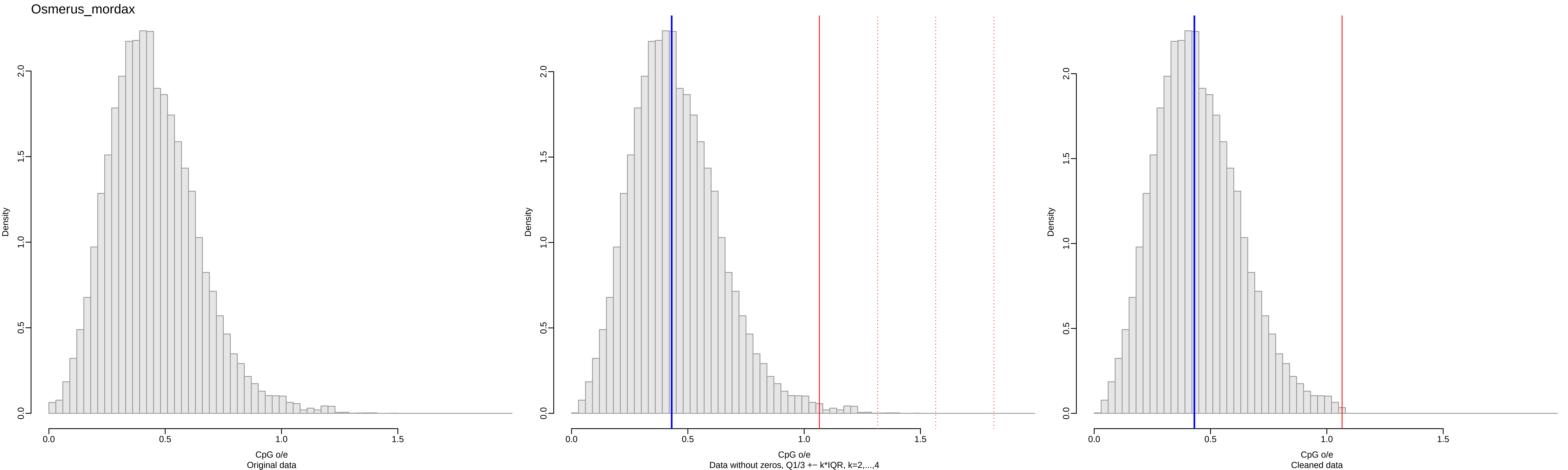
Osmerus_mordax
Density
CpG o/e
Original data
Data without zeros, Q1/3 +− k*IQR, k=2,...,4
Cleaned data
0.0
0.5
1.0
1.5
2.0

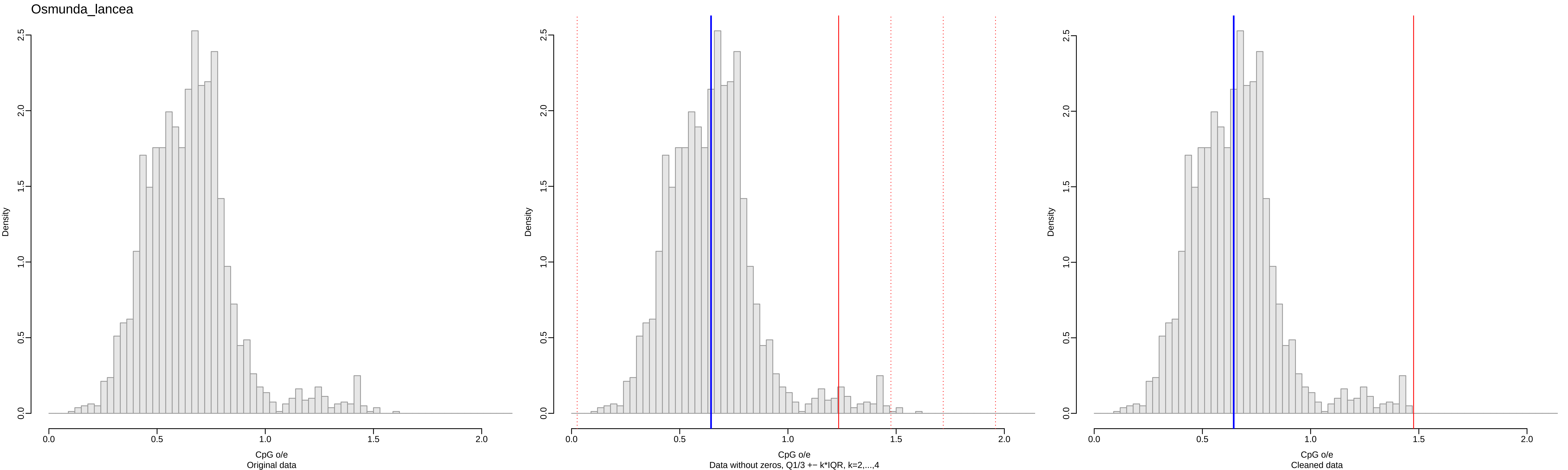
Osmunda_lancea
Density
CpG o/e
Original data
Data without zeros, Q1/3 +− k*IQR, k=2,...,4
Cleaned data
0.0
0.5
1.0
1.5
2.0
2.5

Ostertagia_ostertagi

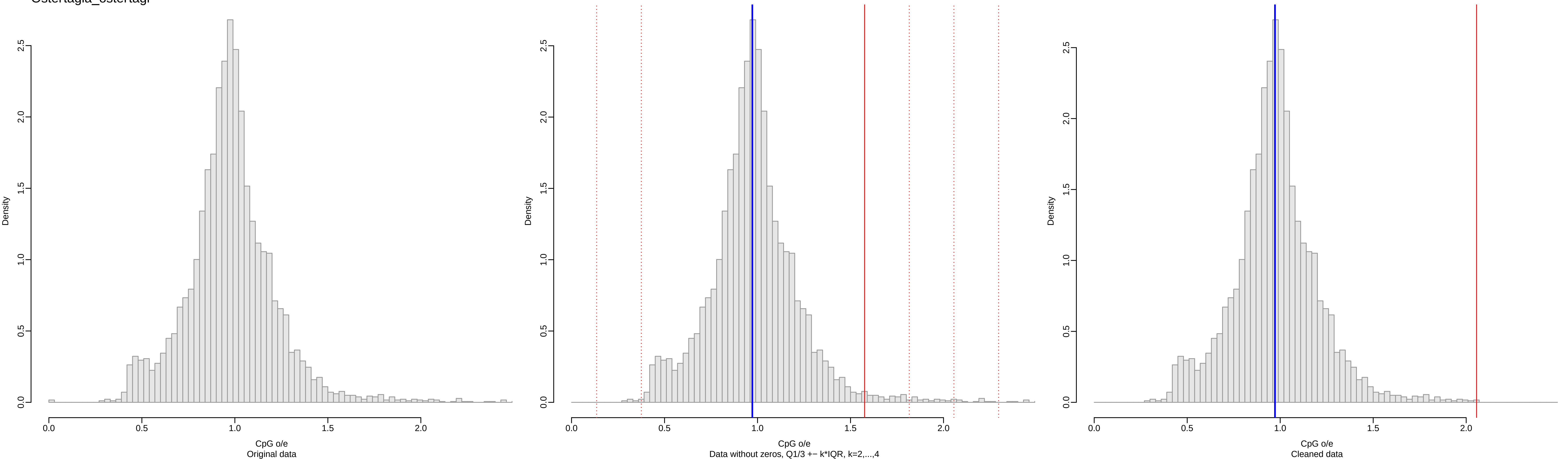

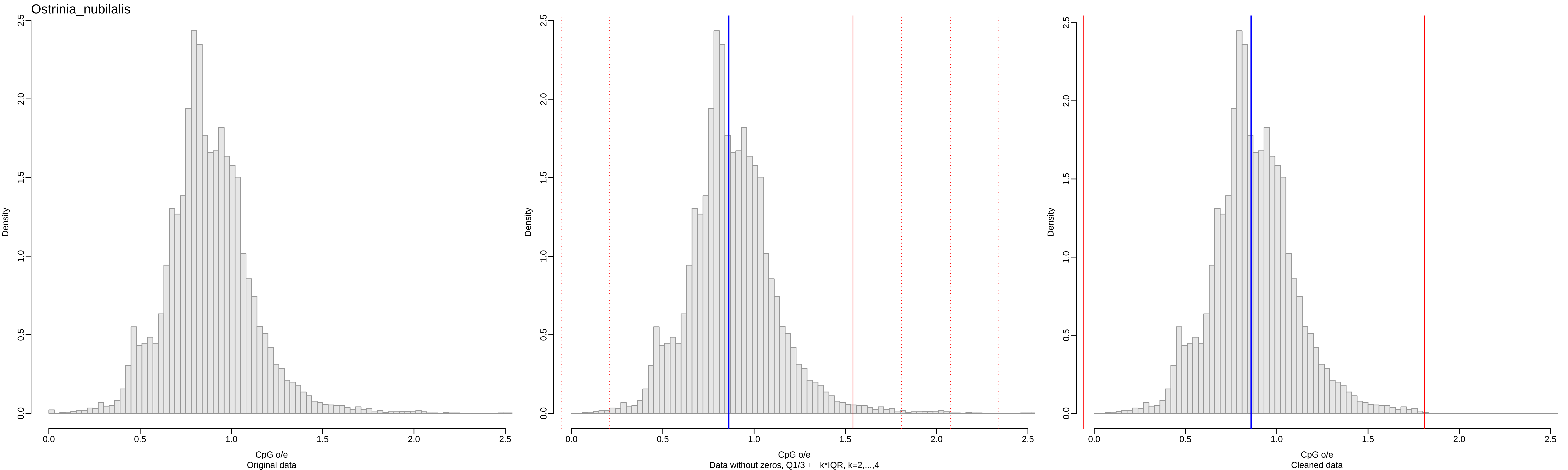
Ostrinia_nubilalis
Density
CpG o/e
Original data
Data without zeros, Q1/3 +− k*IQR, k=2,...,4
Cleaned data
0.0
0.5
1.0
1.5
2.0
2.5

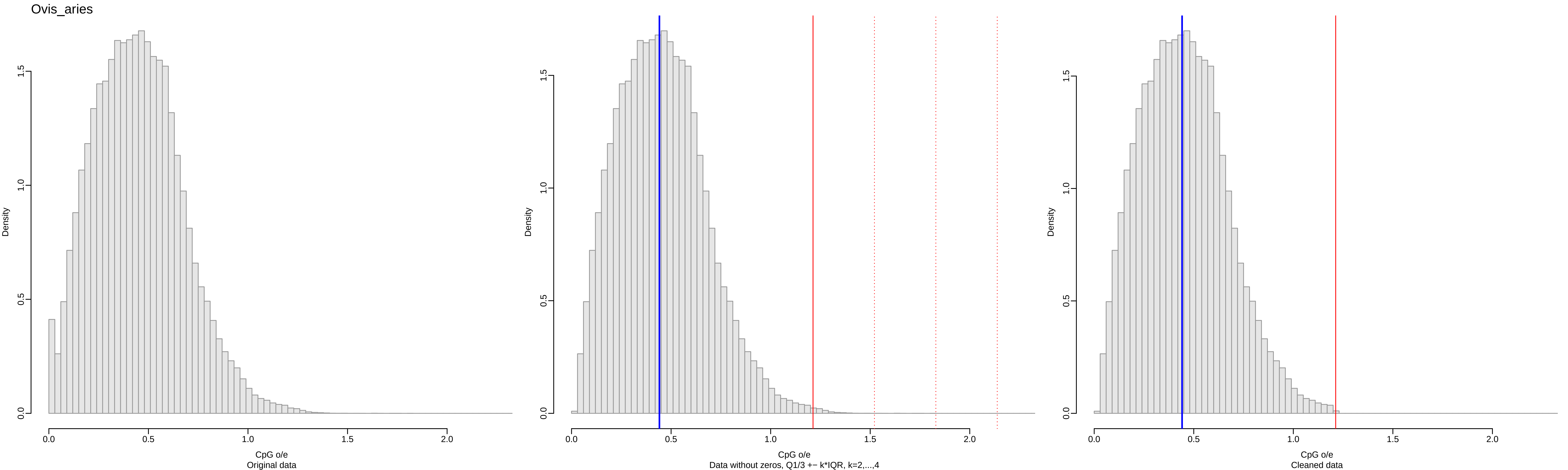
Ovis_aries
Density
CpG o/e
Original data
Data without zeros, Q1/3 +− k*IQR, k=2,...,4
Cleaned data
0.0
0.5
1.0
1.5
2.0

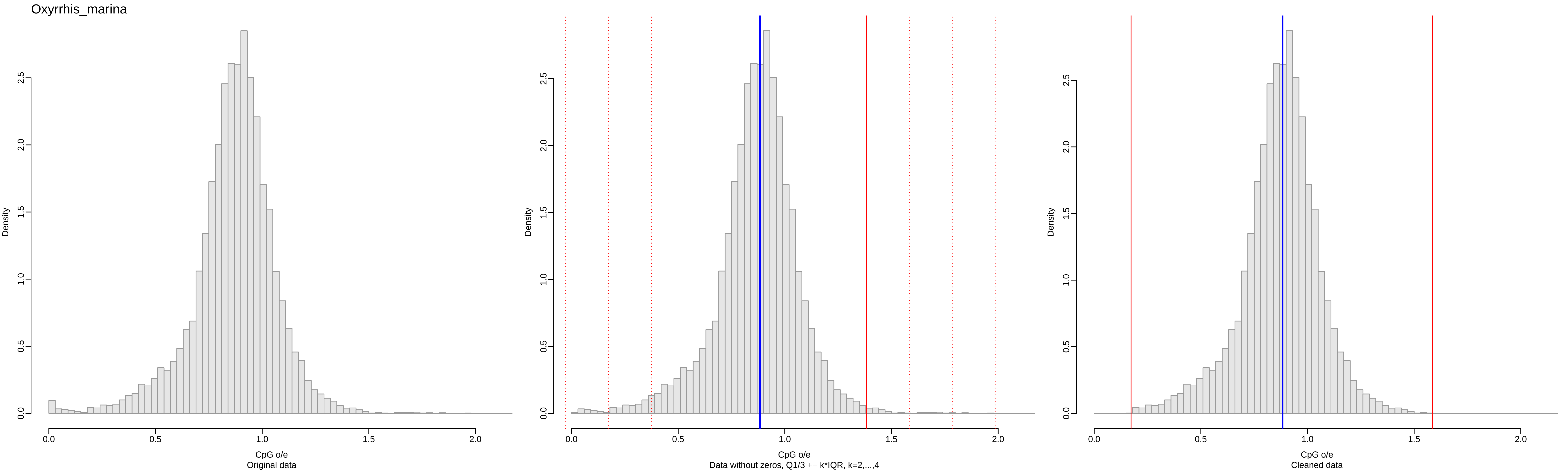

Oxyrrhis_marina
Density
CpG o/e
Original data
Density
CpG o/e
Data without zeros, Q1/3 +− k*IQR, k=2,...,4
Density
CpG o/e
Cleaned data

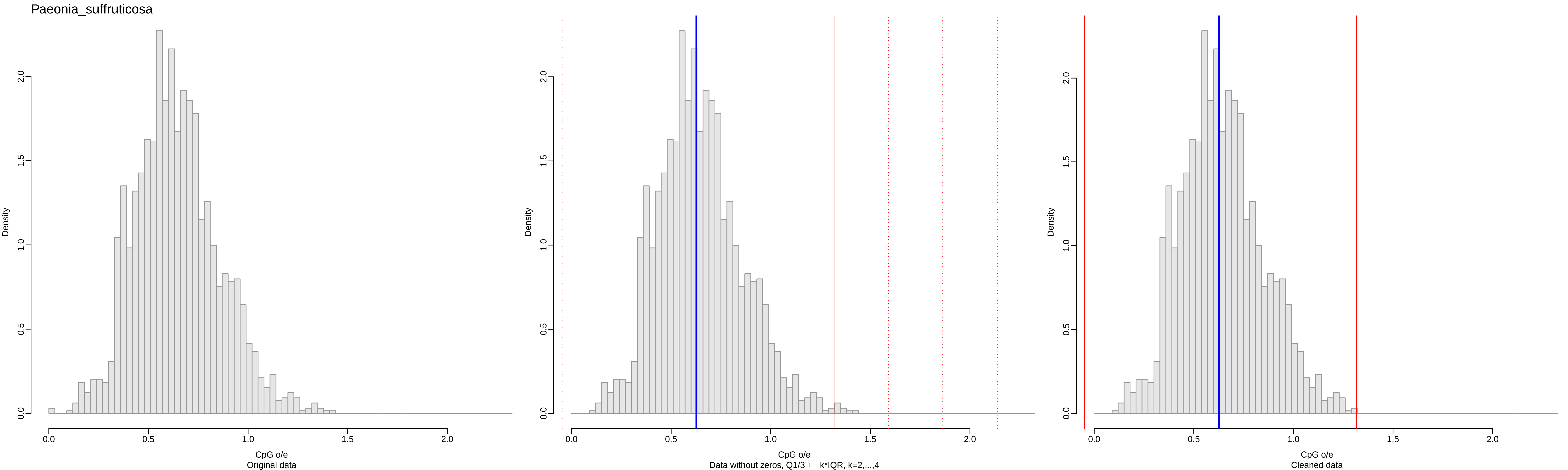

Paeonia_suffruticosa
Density
CpG o/e
Original data
CpG o/e
Data without zeros, Q1/3 +− k*IQR, k=2,...,4
CpG o/e
Cleaned data

Pagrus_major

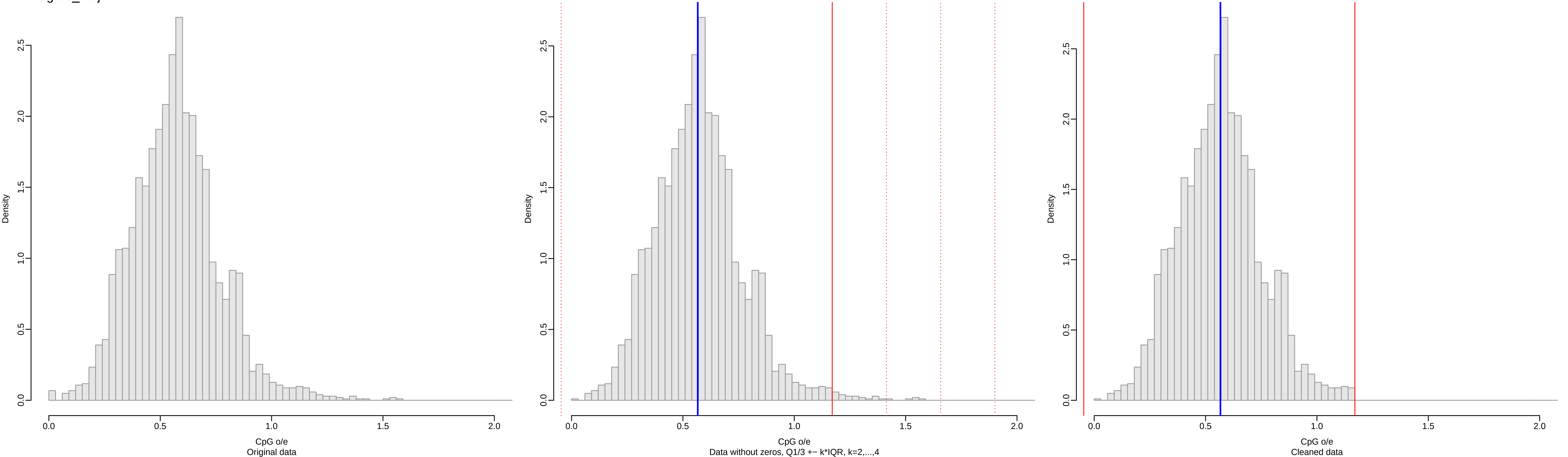

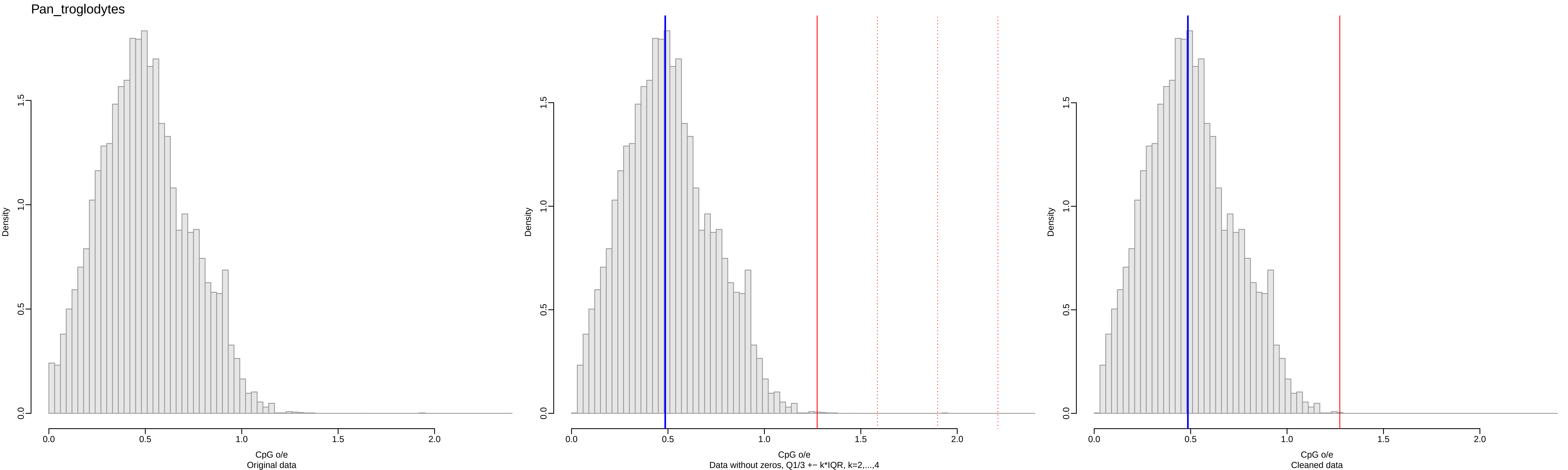
Pan_troglodytes
Density
CpG o/e
Original data
Data without zeros, Q1/3 +− k*IQR, k=2,...,4
Cleaned data
0.0
0.5
1.0
1.5
2.0

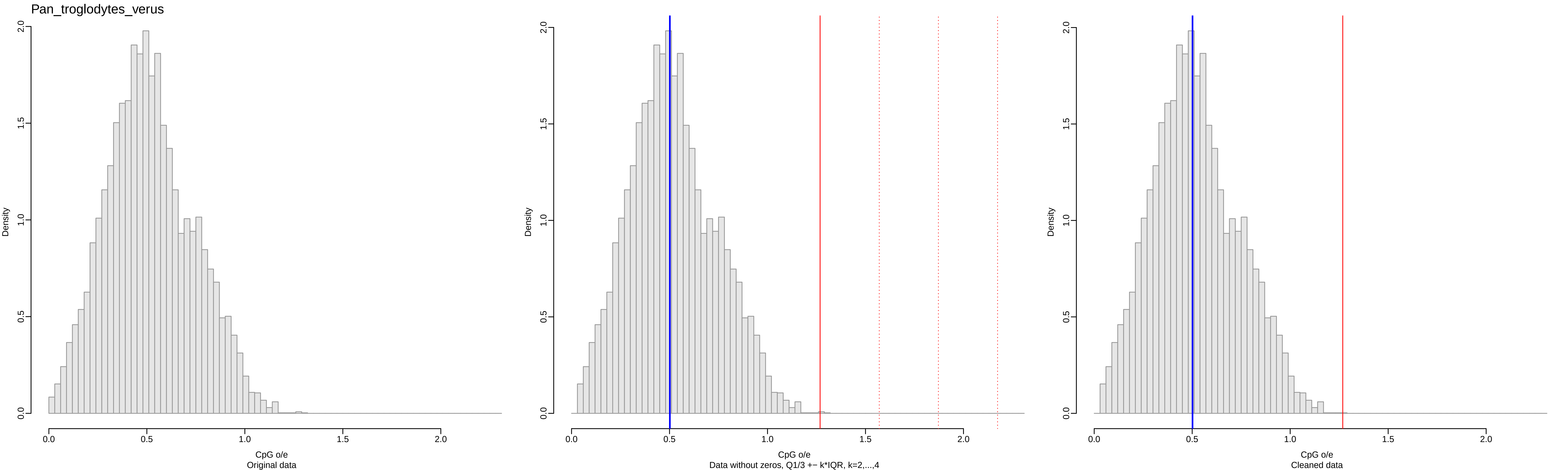
Pan_troglodytes_verus
Density
CpG o/e
Original data
Data without zeros, Q1/3 +− k*IQR, k=2,...,4
Cleaned data
0.0
0.5
1.0
1.5
2.0

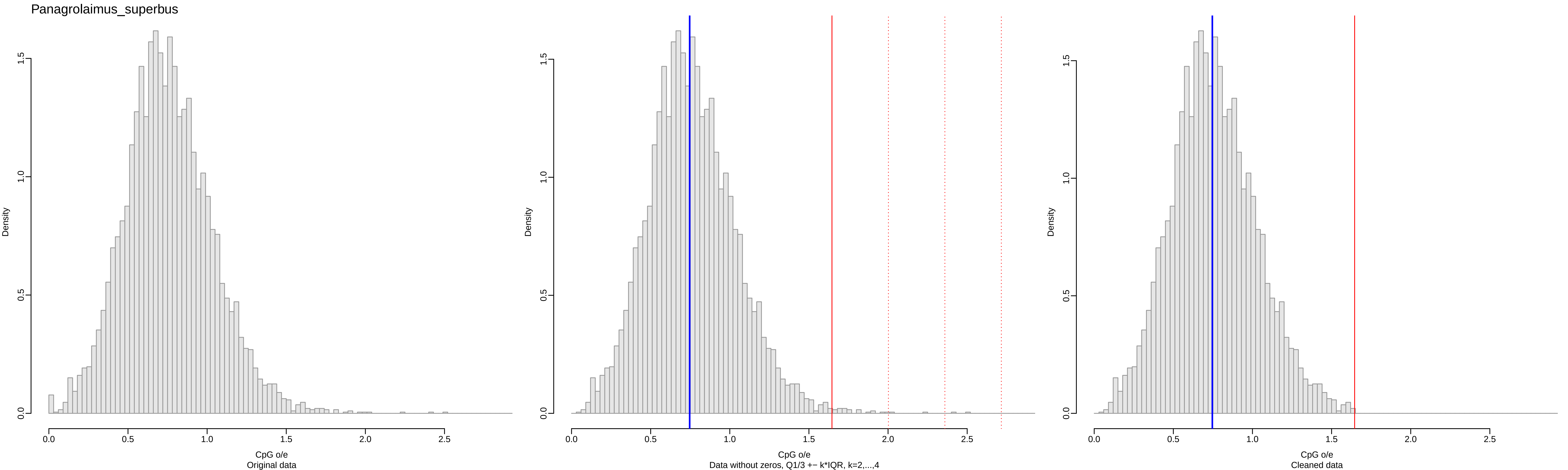

Panagrolaimus_superbus
Density
0.0
0.5
1.0
1.5
0.0
0.5
1.0
1.5
2.0
2.5
CpG o/e
Original data
Density
CpG o/e
Data without zeros, Q1/3 +− k*IQR, k=2,...,4
Density
CpG o/e
Cleaned data

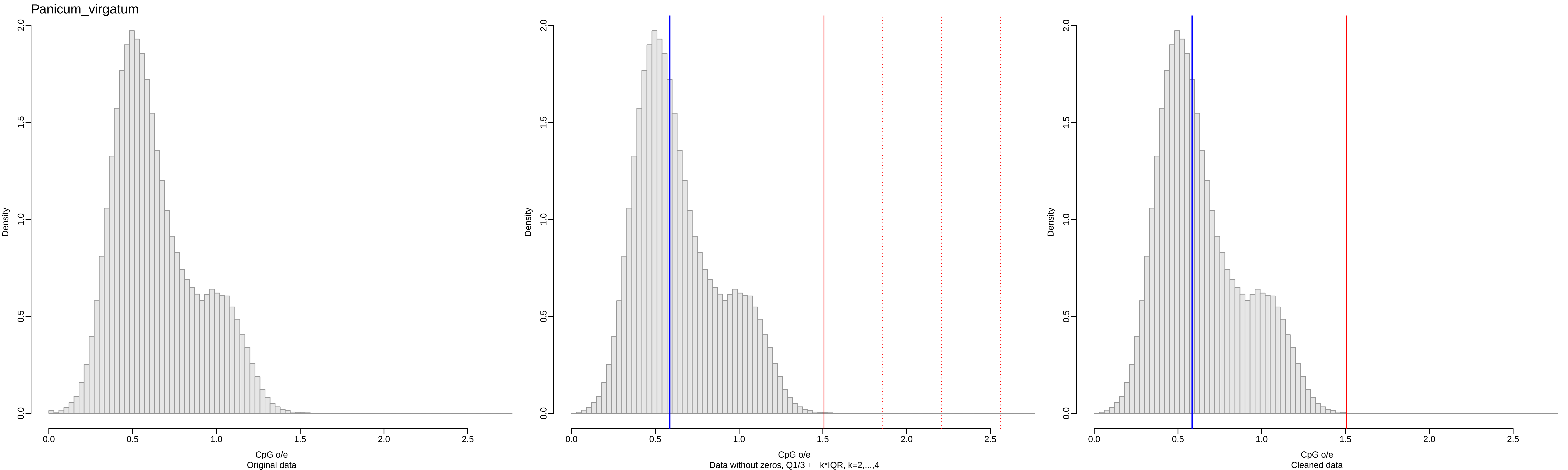
Panicum_virgatum
Density
CpG o/e
Original data
Density
CpG o/e
Data without zeros, Q1/3 +− k*IQR, k=2,...,4
Density
CpG o/e
Cleaned data
0.0
0.5
1.0
1.5
2.0
2.5

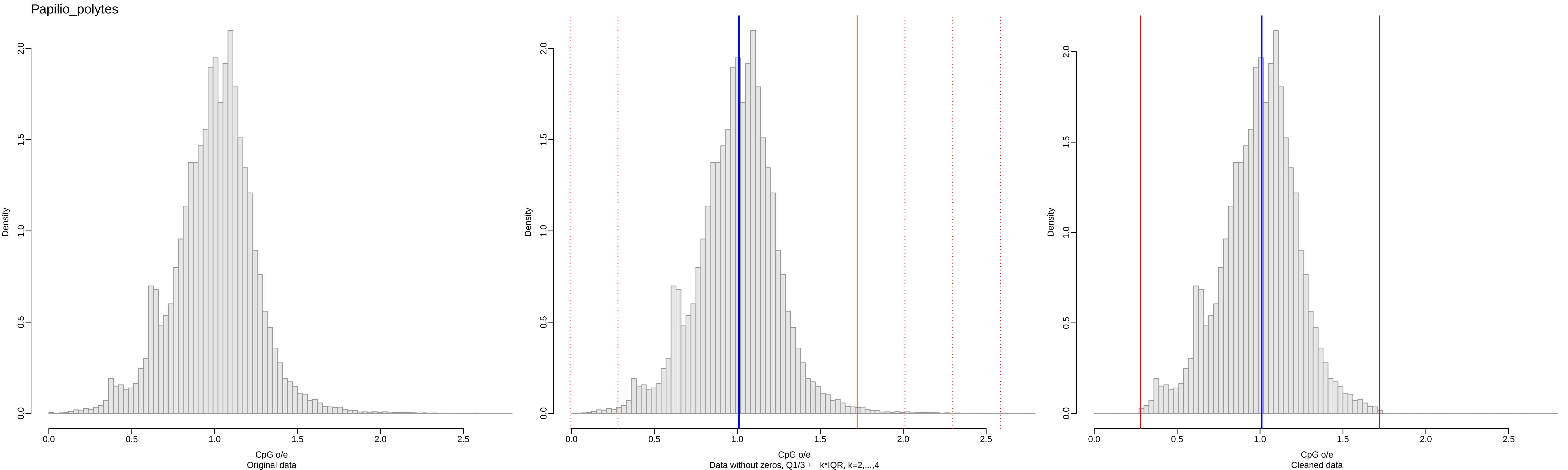

Papilio_polytes
Density
CpG o/e
Original data
Density
CpG o/e
Data without zeros, Q1/3 +− k*IQR, k=2,...,4
Density
CpG o/e
Cleaned data

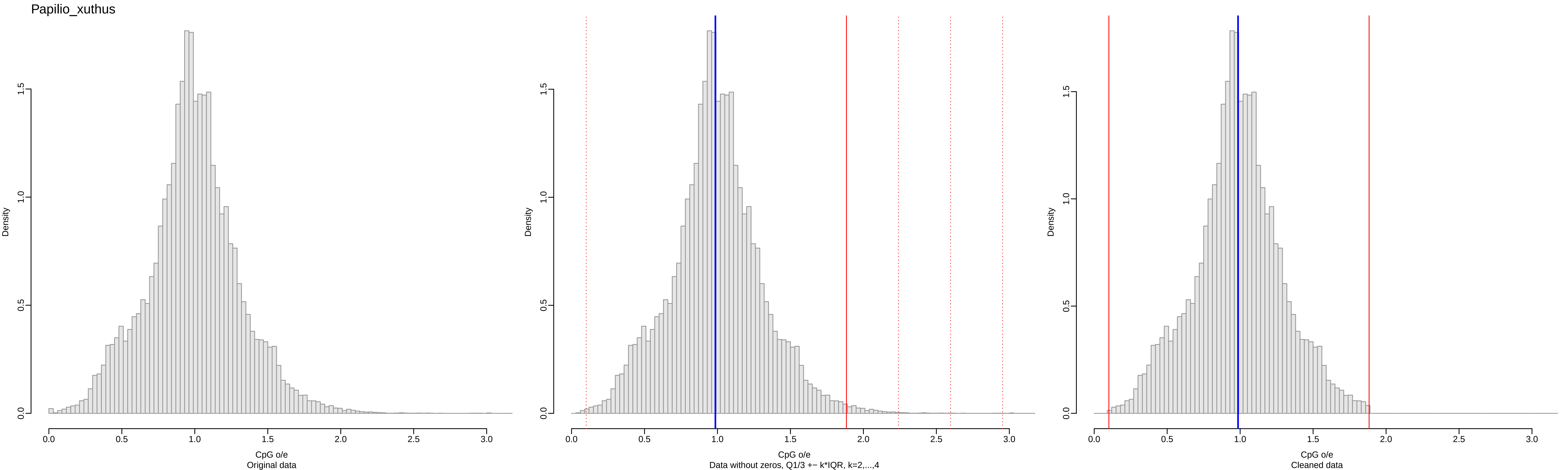

Papilio_xuthus
Density
CpG o/e
Original data
Data without zeros, Q1/3 +− k*IQR, k=2,...,4
Cleaned data
0.0
0.5
1.0
1.5
2.0
2.5
3.0

Papio_anubis

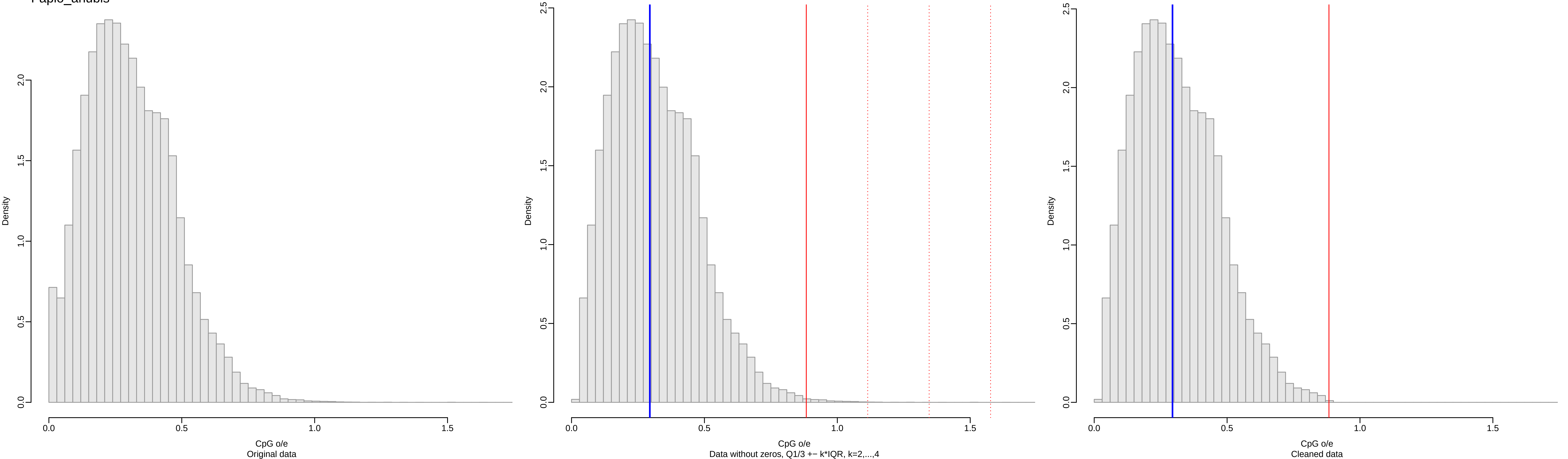

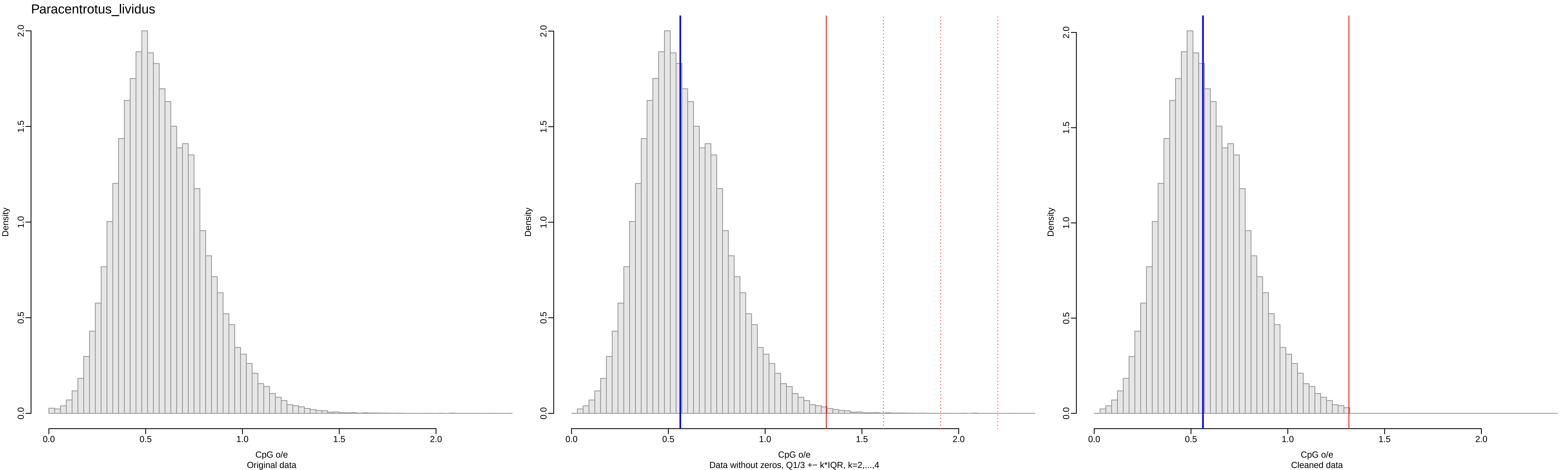
Paracentrotus_lividus
Density
CpG o/e
Original data
Density
CpG o/e
Data without zeros, Q1/3 +− k*IQR, k=2,...,4
Density
CpG o/e
Cleaned data
0.0
0.5
1.0
1.5
2.0

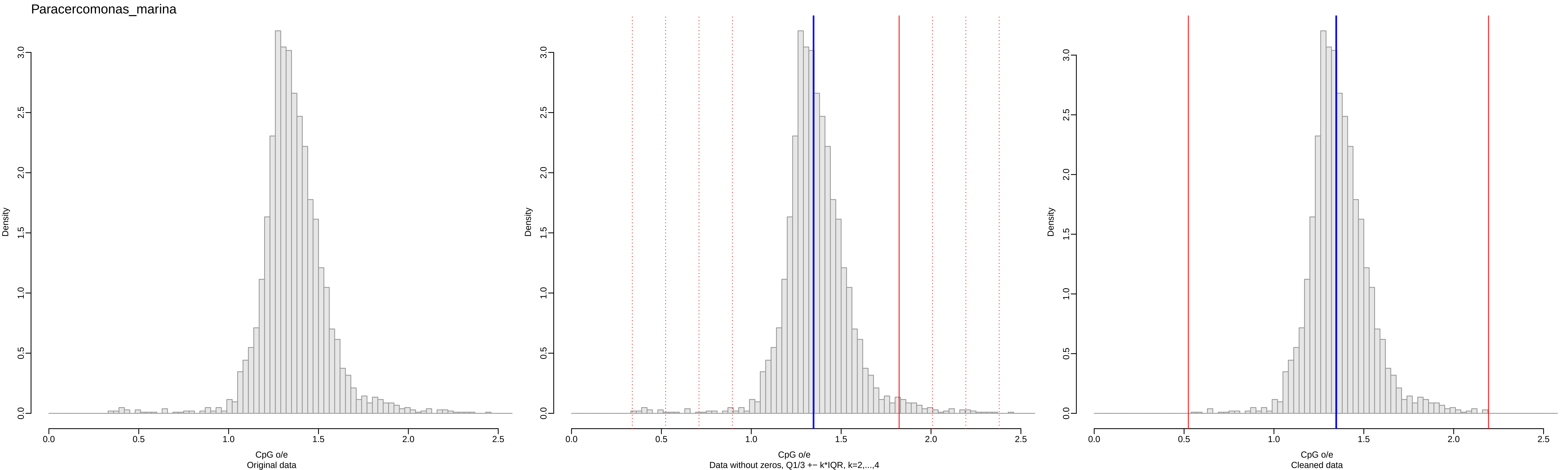

Paracercomonas_marina
Density
CpG o/e
Original data
Density
CpG o/e
Data without zeros, Q1/3 +− k*IQR, k=2,...,4
Density
CpG o/e
Cleaned data

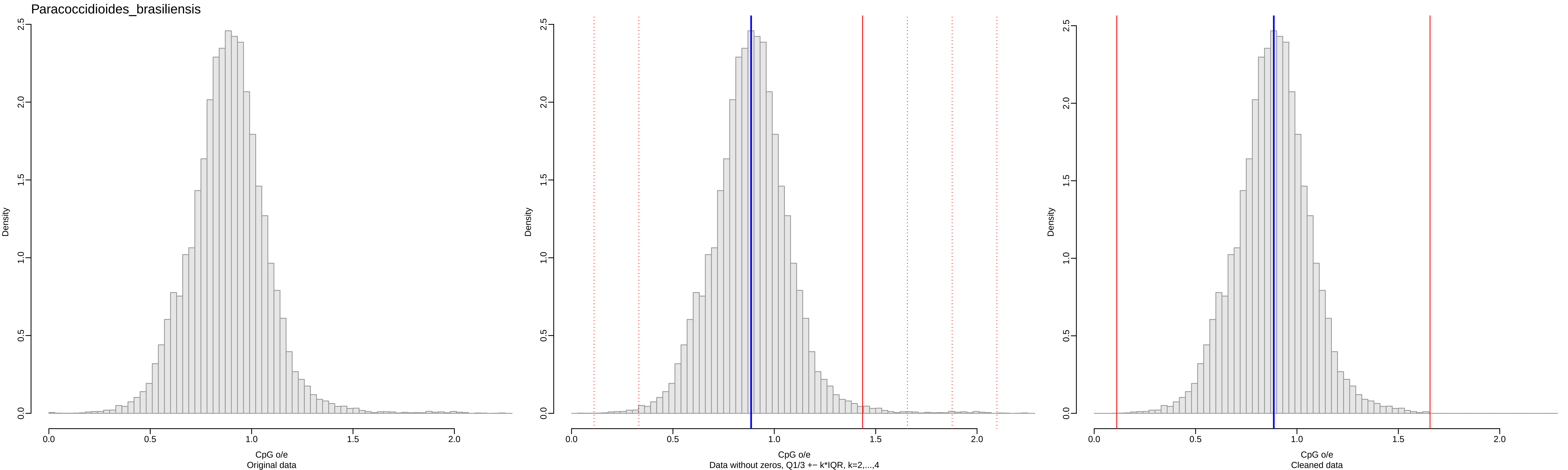

Paracoccidioides_brasiliensis
Density
CpG o/e
Original data
Data without zeros, Q1/3 +− k*IQR, k=2,...,4
Cleaned data
0.0
0.5
1.0
1.5
2.0
2.5

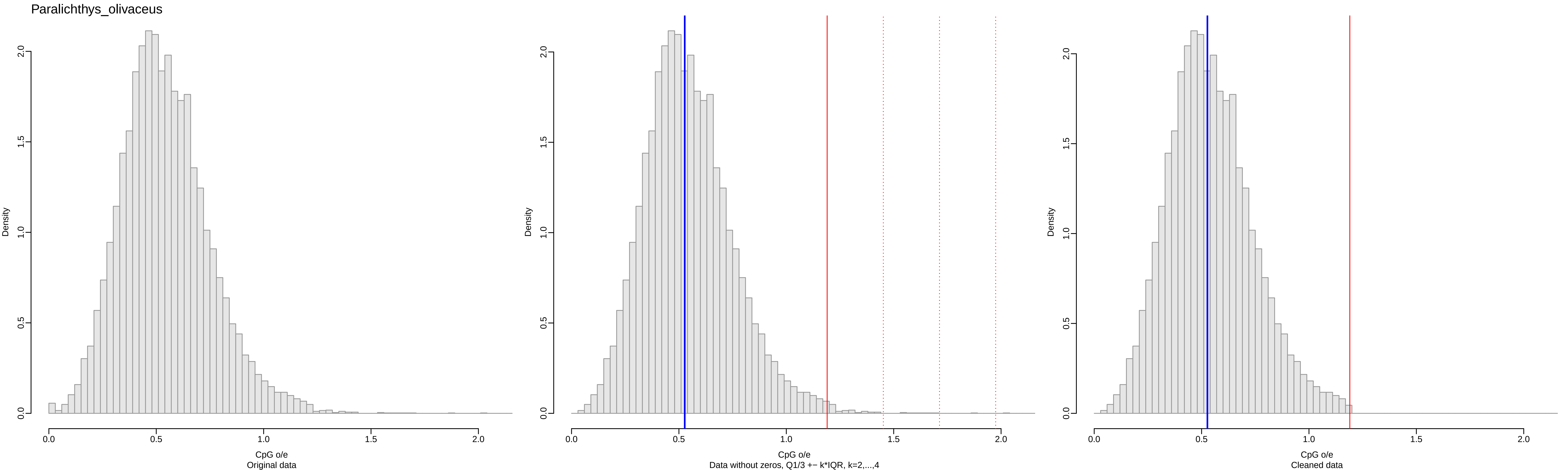

Paralichthys_olivaceus
Density
CpG o/e
Original data
Data without zeros, Q1/3 +− k*IQR, k=2,...,4
Cleaned data
0.0
0.5
1.0
1.5
2.0

Paramecium_tetraurelia

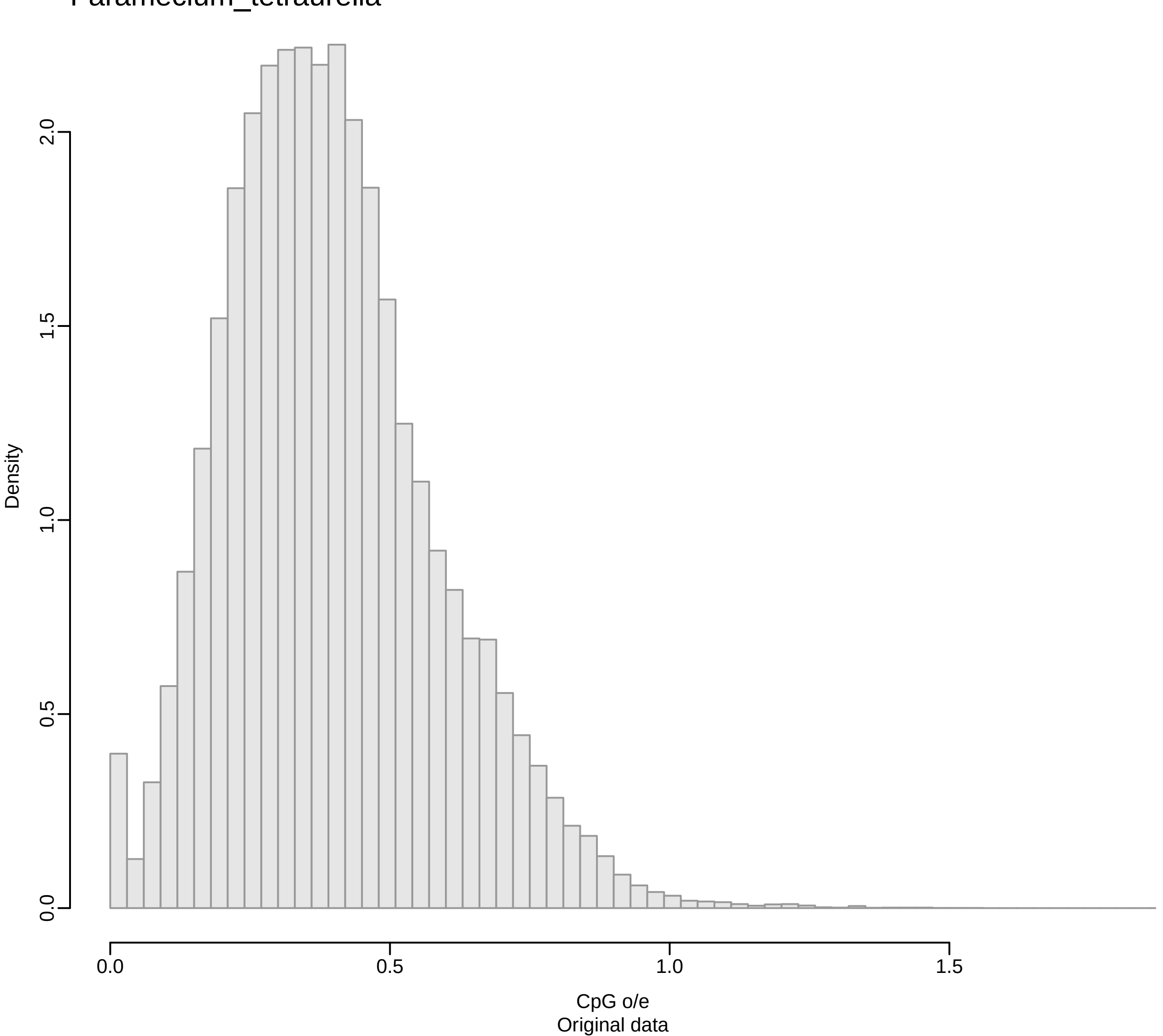

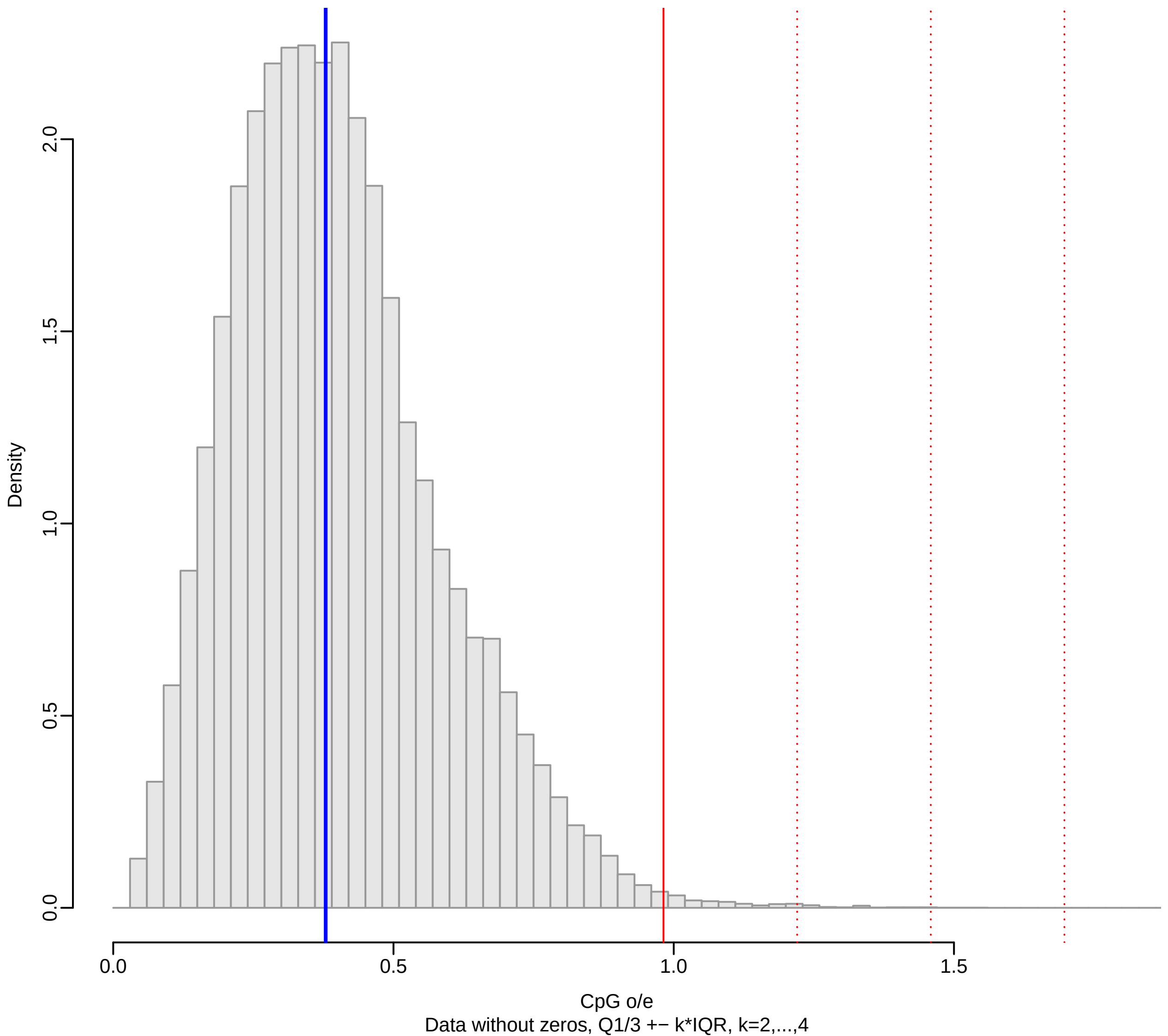

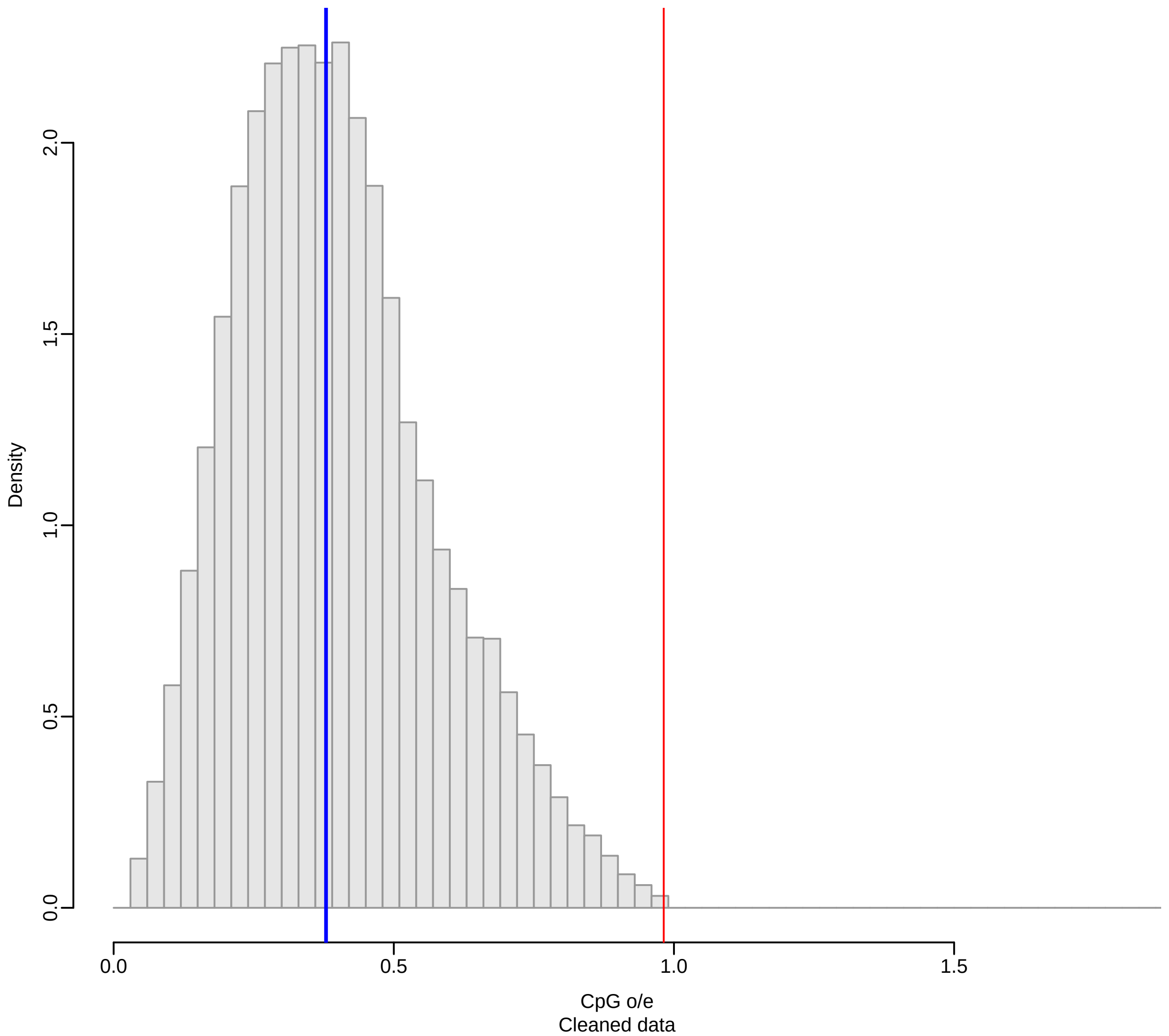

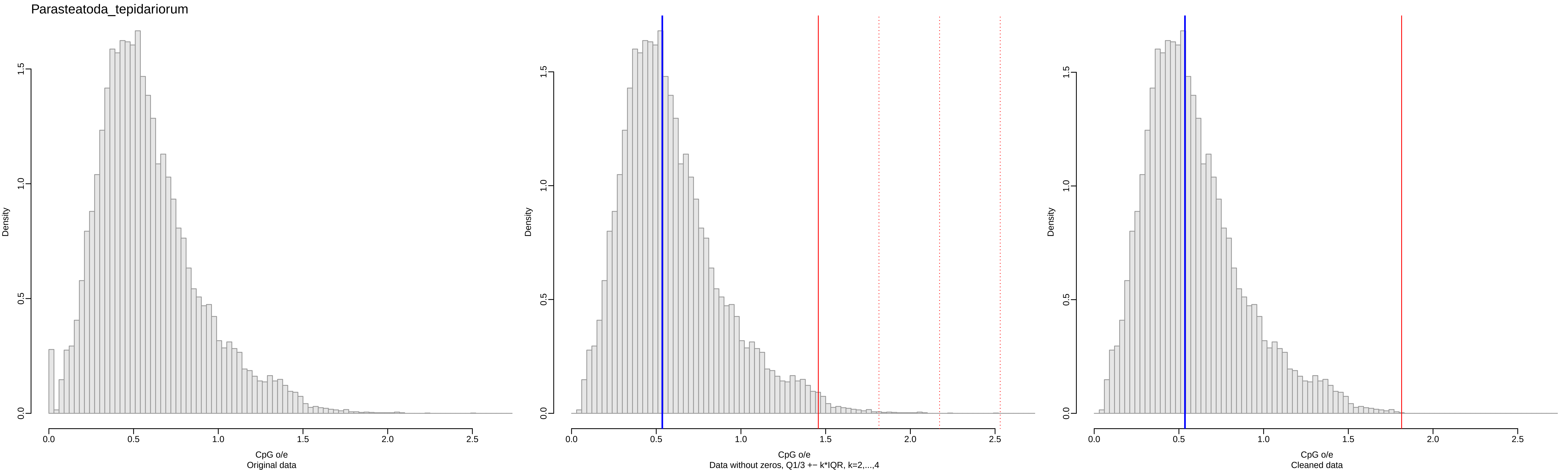
Parasteatoda_tepidariorum
Density
CpG o/e
Original data
Data without zeros, Q1/3 +− k*IQR, k=2,...,4
Cleaned data
0.0
0.5
1.0
1.5
2.0
2.5

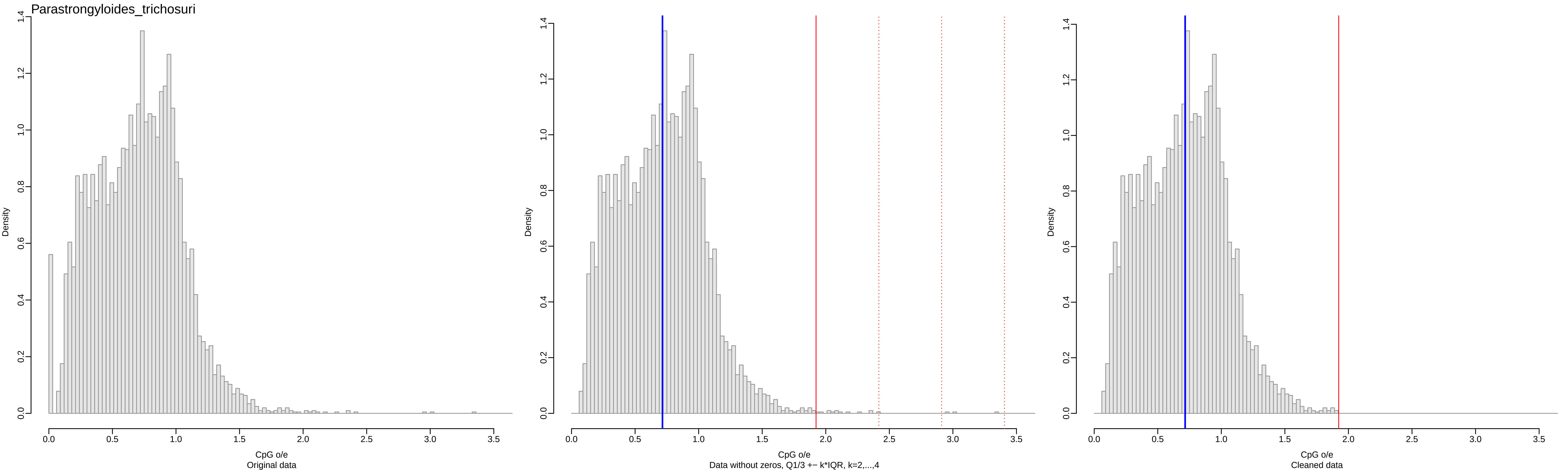
Parastrongyloides_trichosuri
Density
CpG o/e
Original data
Data without zeros, Q1/3 +− k*IQR, k=2,...,4
Cleaned data
0.0
0.2
0.4
0.6
0.8
1.0
1.2
1.4
0.5
1.5
2.0
2.5
3.0
3.5

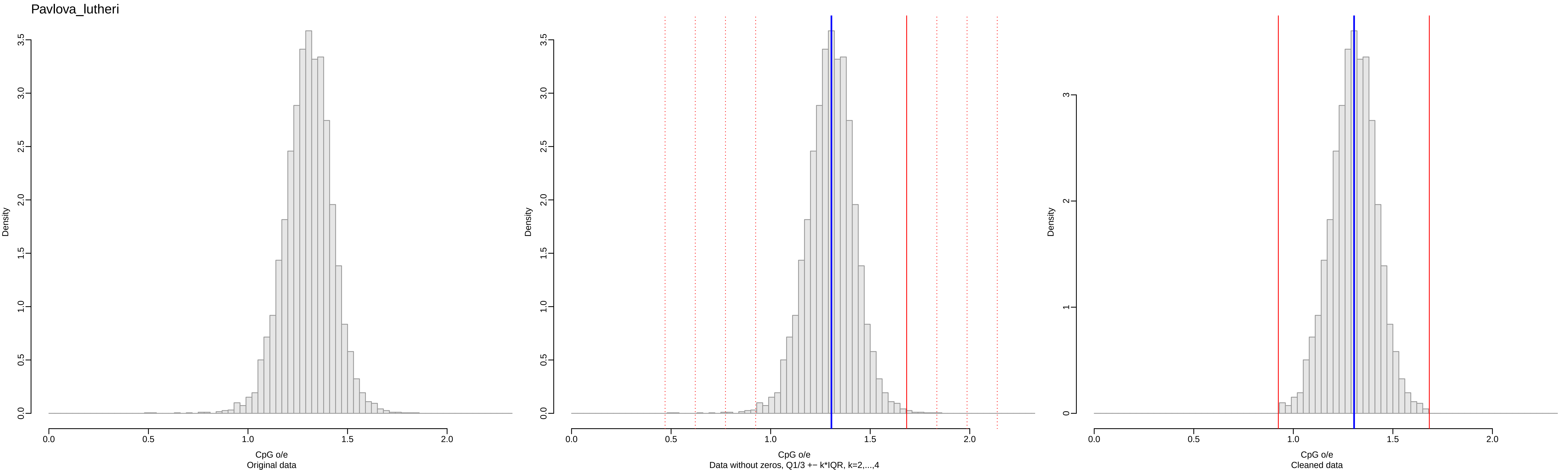

Pavlova_lutheri
Density
CpG o/e
Original data
Density
CpG o/e
Data without zeros, Q1/3 +− k*IQR, k=2,...,4
Density
CpG o/e
Cleaned data

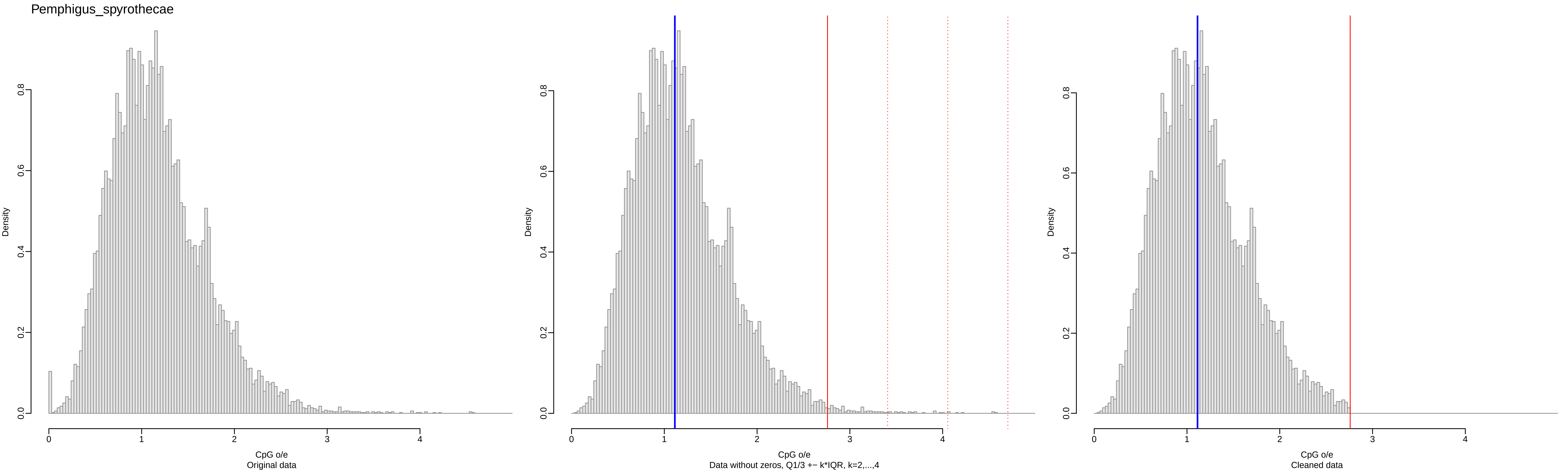
Pemphigus_spyrothecae
Density
CpG o/e
Original data
Density
CpG o/e
Data without zeros, Q1/3 +− k*IQR, k=2,...,4
Density
CpG o/e
Cleaned data

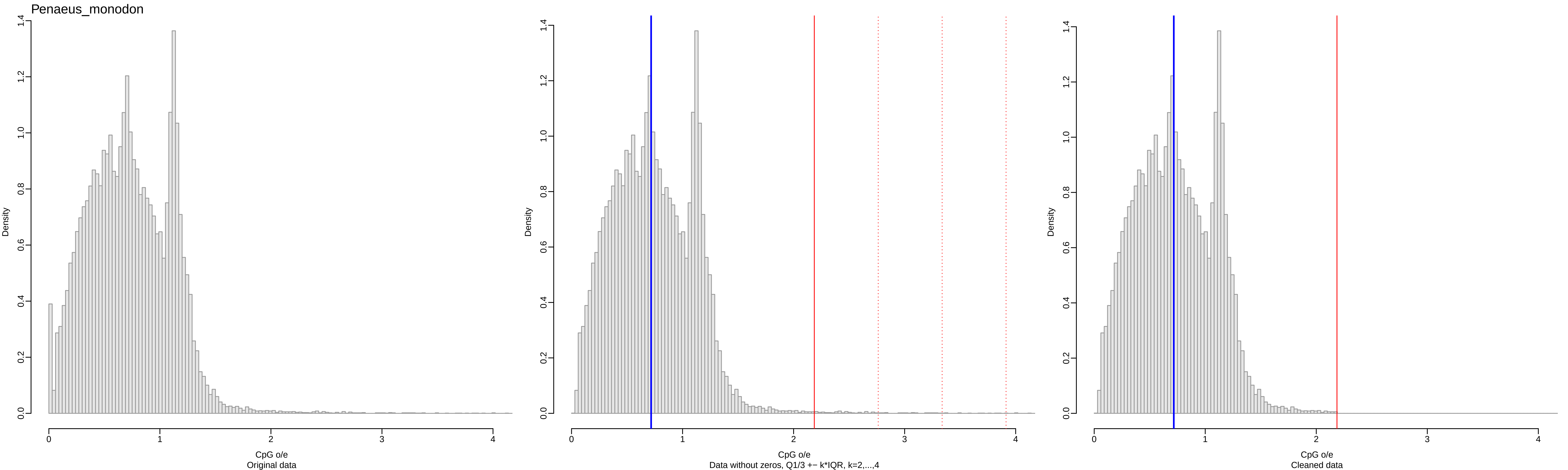

Penaeus_monodon
Density
CpG o/e
Original data
CpG o/e
Data without zeros, Q1/3 +− k*IQR, k=2,...,4
CpG o/e
Cleaned data

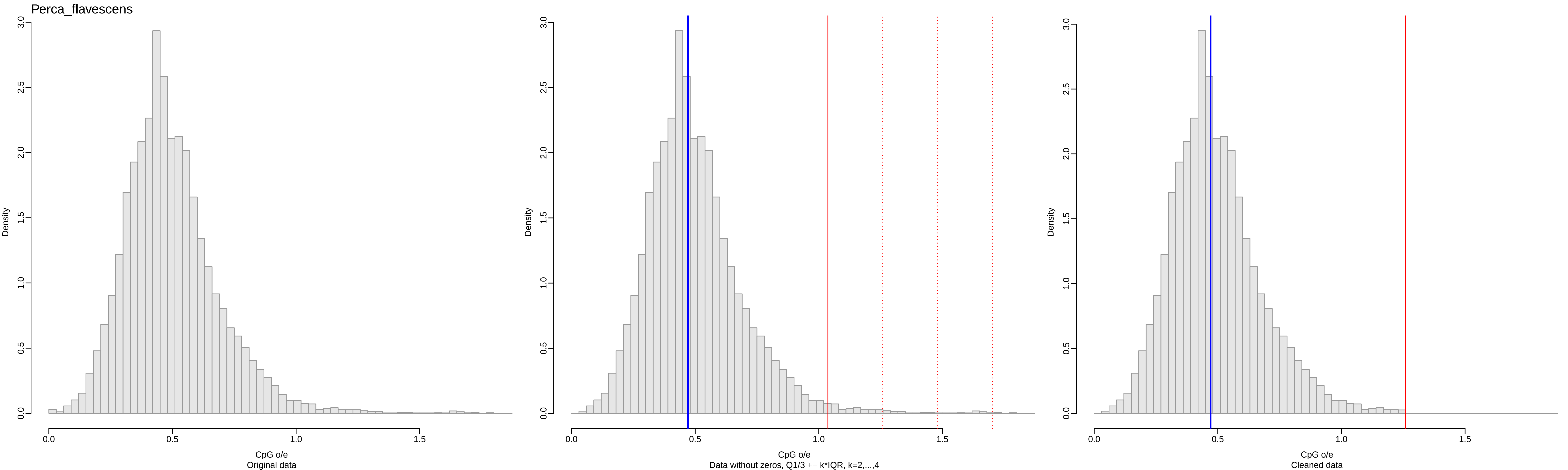

Perca_flavescens
Density
CpG o/e
Original data
Data without zeros, Q1/3 +− k*IQR, k=2,...,4
Cleaned data
0.0
0.5
1.0
1.5
2.0
2.5
3.0

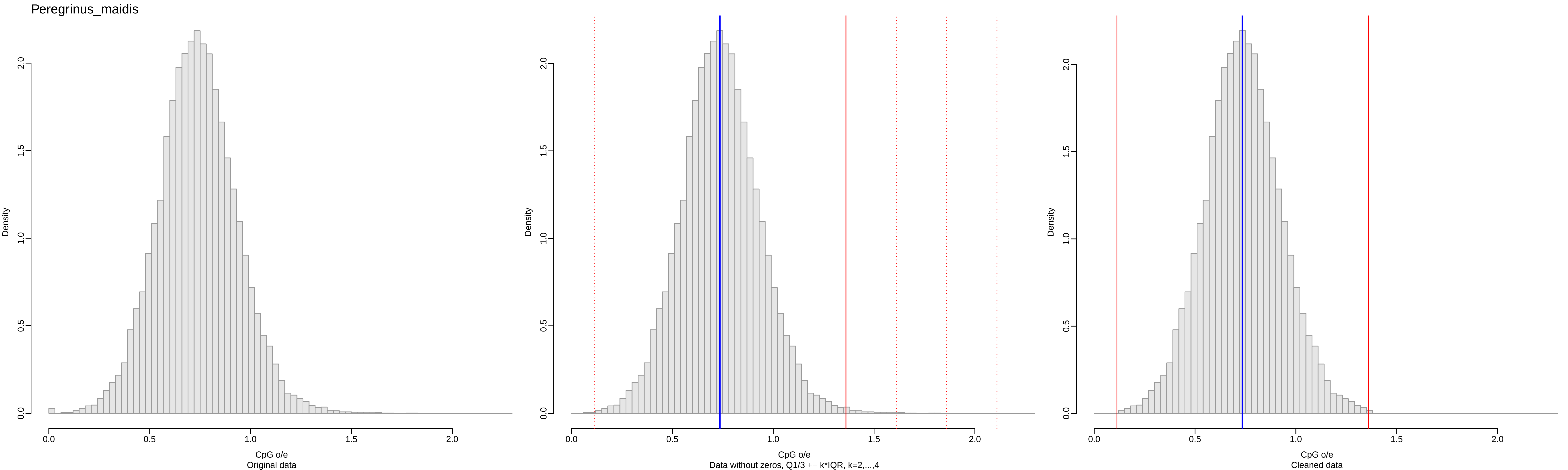
Peregrinus_maidis
Density
CpG o/e
Original data
Data without zeros, Q1/3 +− k*IQR, k=2,...,4
Cleaned data
0.0
0.5
1.0
1.5
2.0

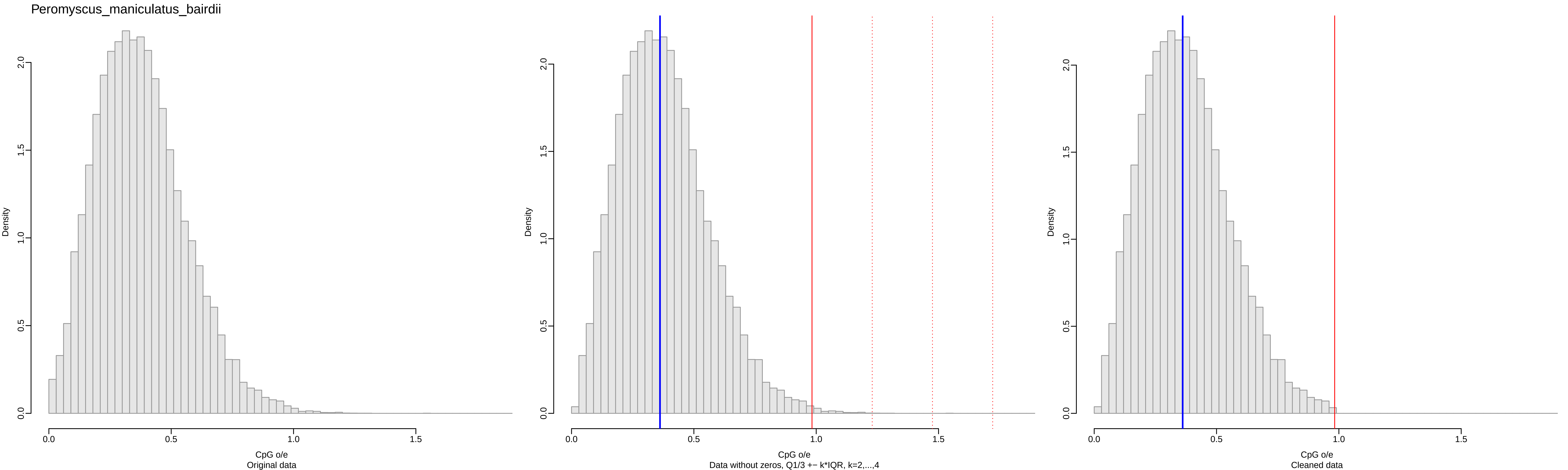
Peromyscus_maniculatus_bairdii
Density
CpG o/e
Original data
Density
CpG o/e
Data without zeros, Q1/3 +− k*IQR, k=2,...,4
Density
CpG o/e
Cleaned data

Peromyscus_polionotus_subgriseus

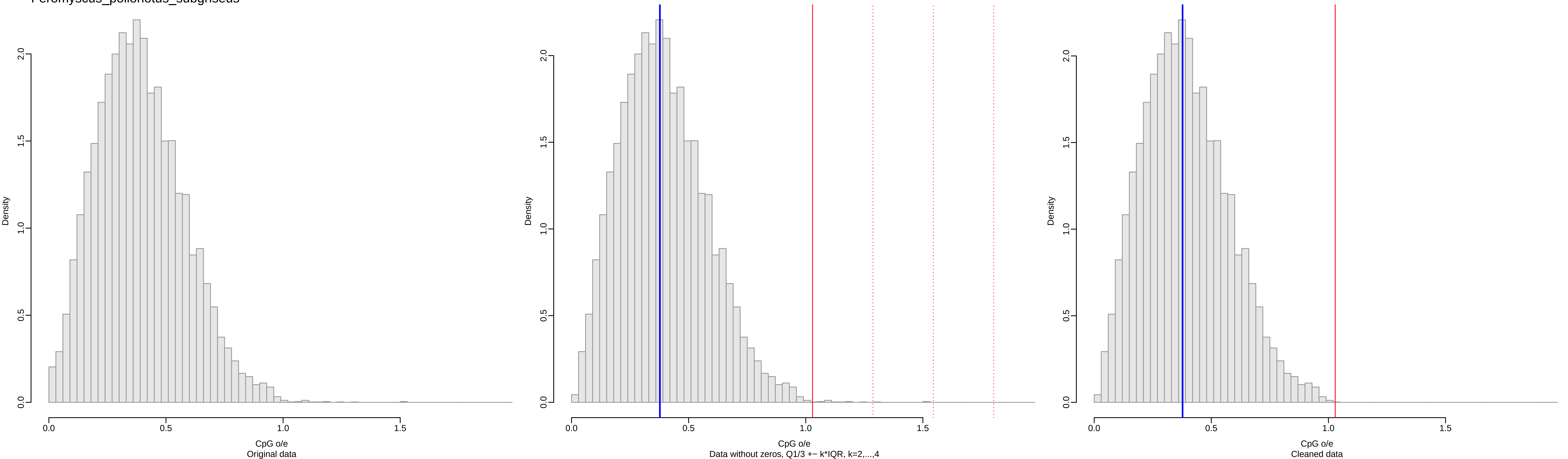

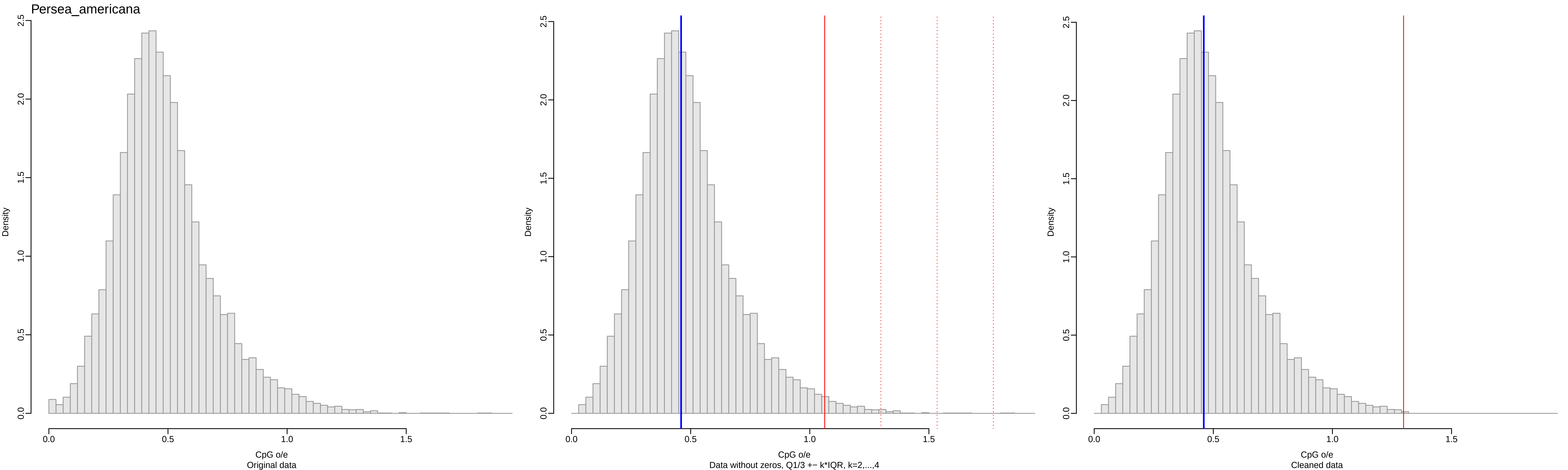
Persea_americana
Density
CpG o/e
Original data
Data without zeros, Q1/3 +− k*IQR, k=2,...,4
Cleaned data
0.0
0.5
1.0
1.5
2.0
2.5

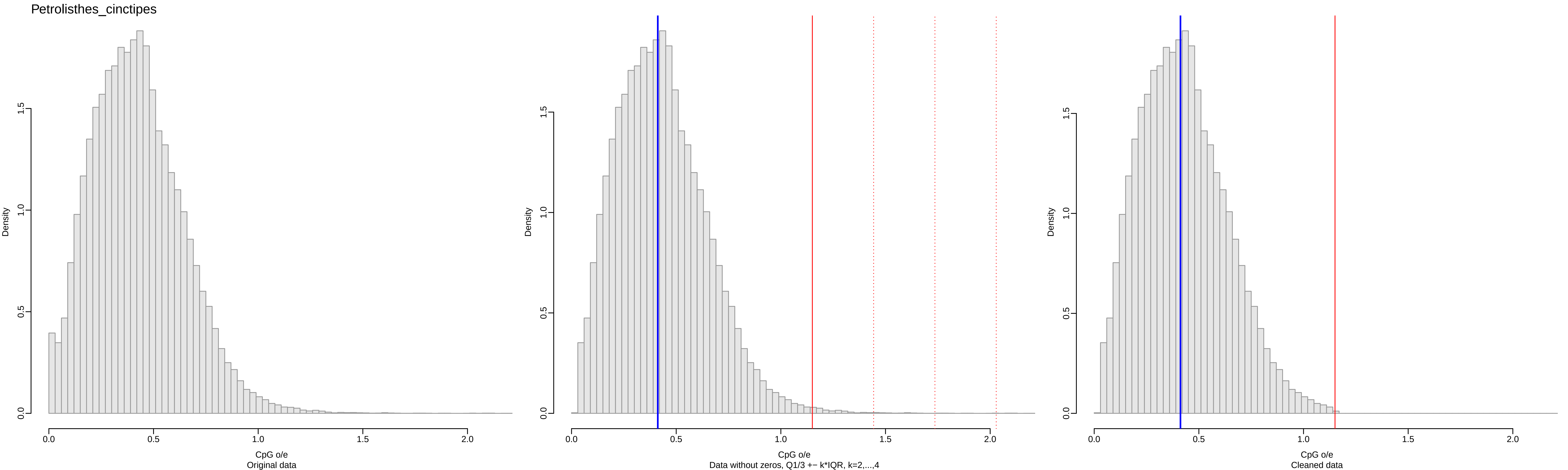

Petrolisthes_cinctipes
Density
CpG o/e
Original data
Data without zeros, Q1/3 +− k*IQR, k=2,...,4
Cleaned data
0.0
0.5
1.0
1.5
2.0

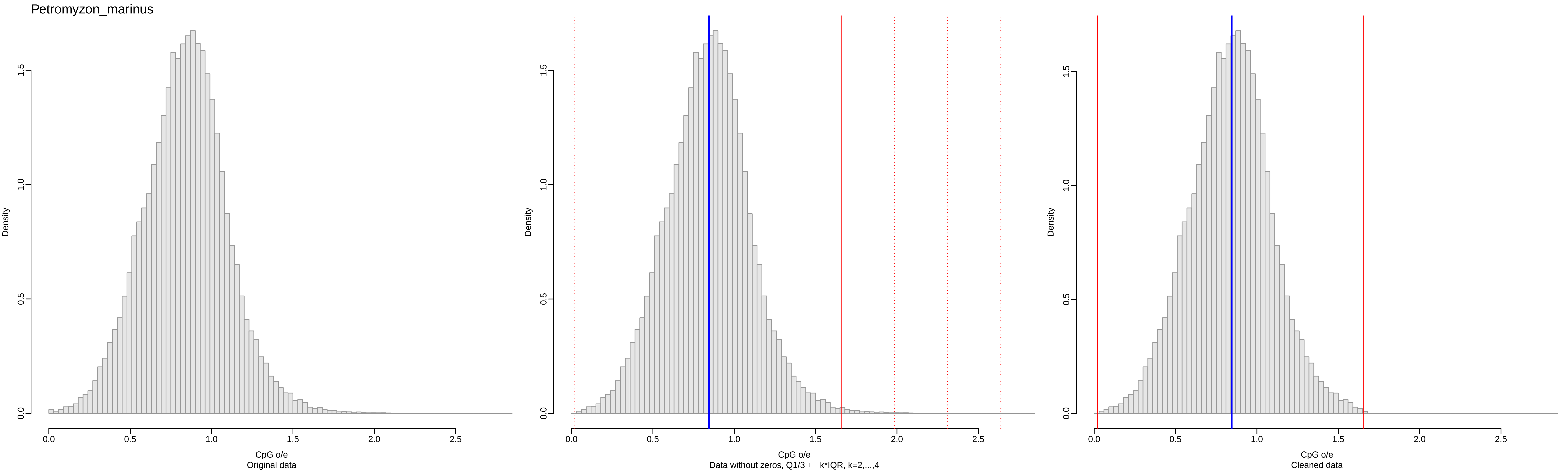
Petromyzon_marinus
Density
0.0
0.5
1.0
1.5
2.0
2.5
CpG o/e
Original data
Data without zeros, Q1/3 +− k*IQR, k=2,...,4
Cleaned data

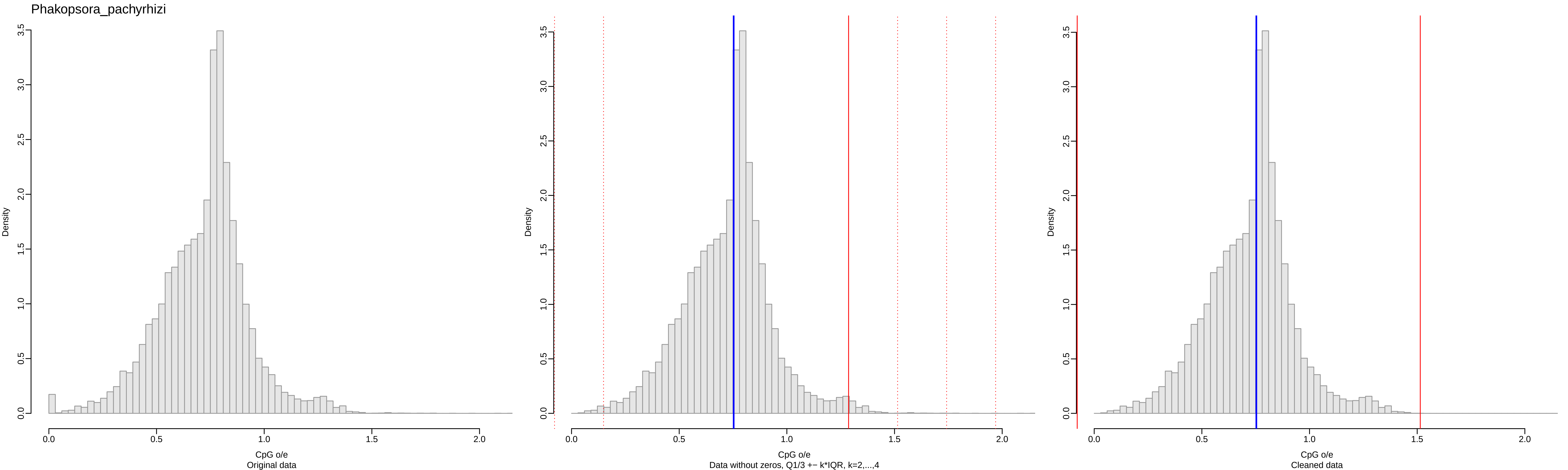

Phakopsora_pachyrhizi
Density
CpG o/e
Original data
Density
CpG o/e
Data without zeros, Q1/3 +− k*IQR, k=2,...,4
Density
CpG o/e
Cleaned data

Phanerochaete_carnosa

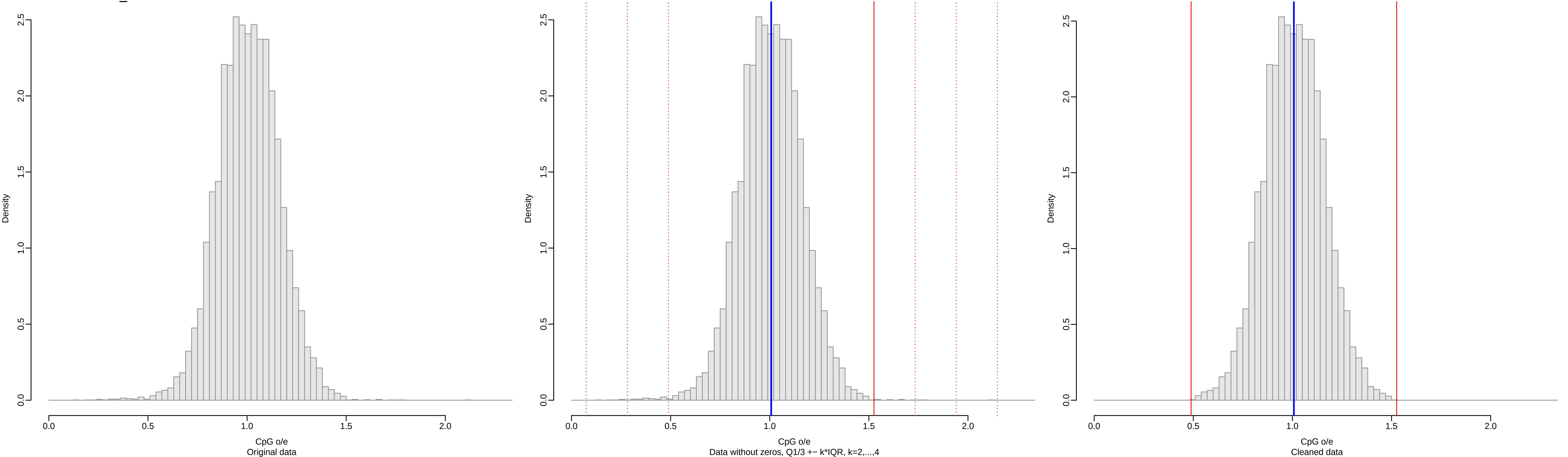

Phanerochaete_chrysosporium

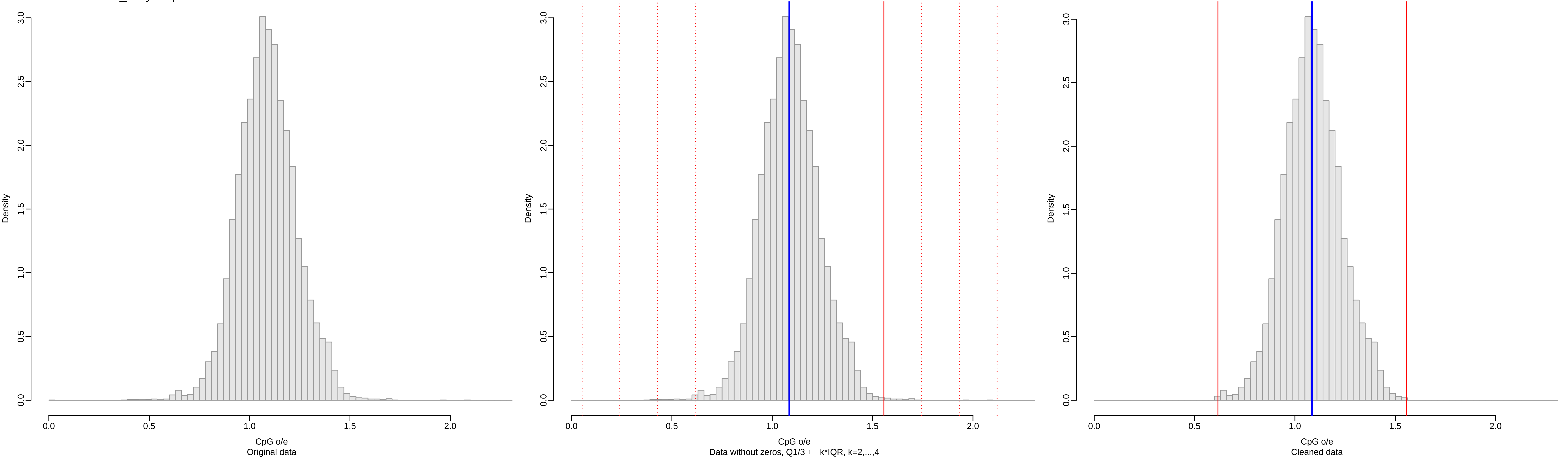

Phaseolus_coccineus

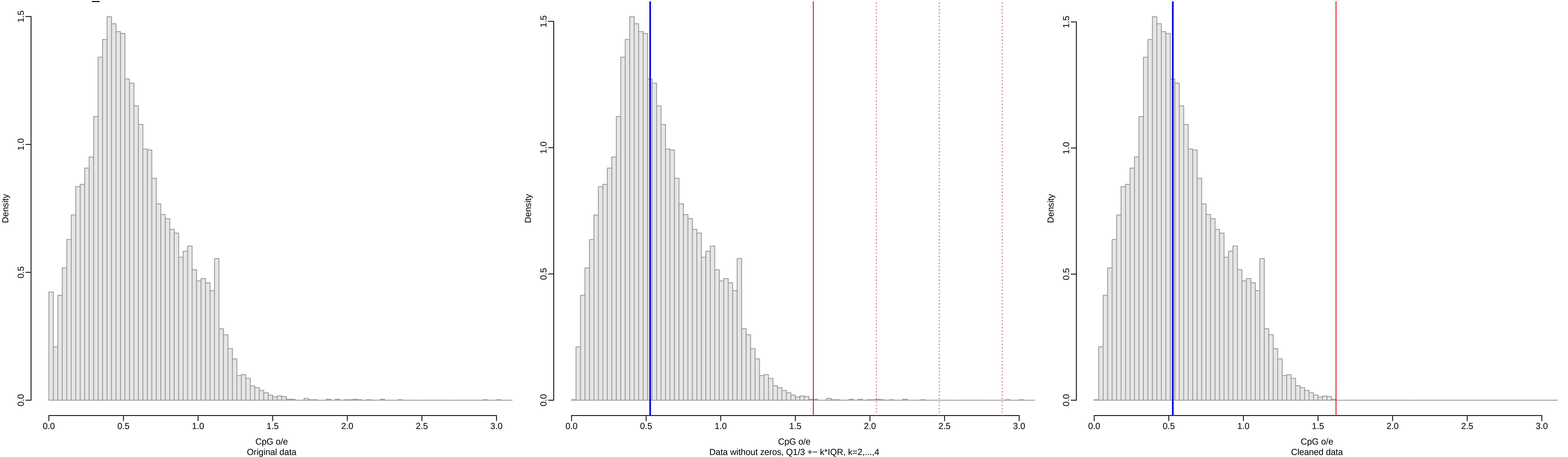

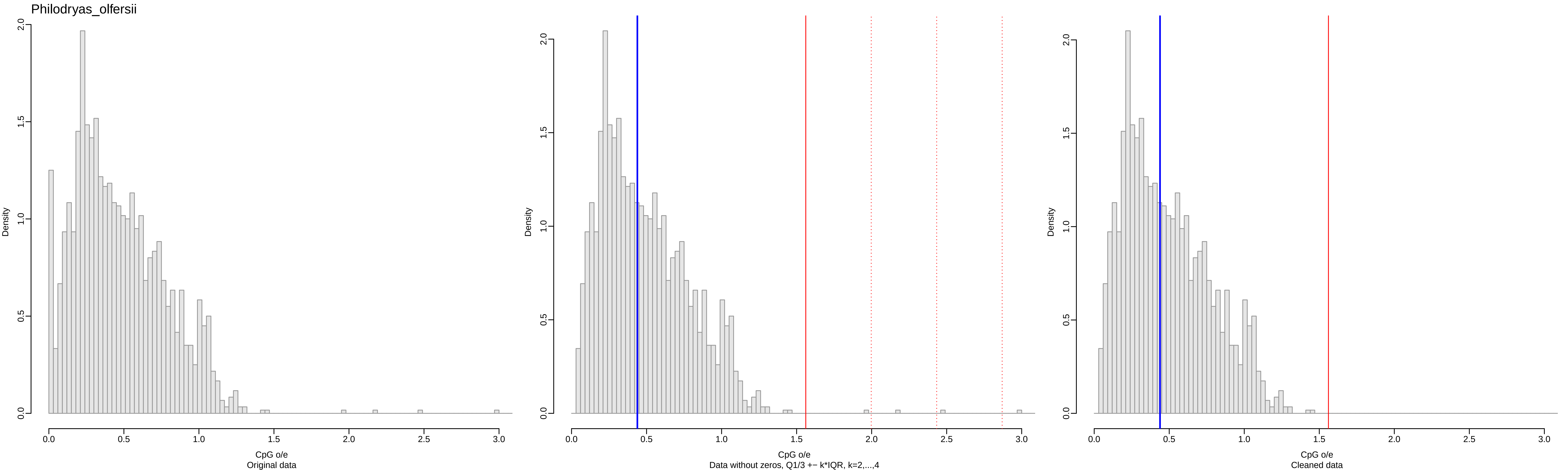

Philodryas_olfersii
Density
CpG o/e
Original data
Data without zeros, Q1/3 +− k*IQR, k=2,...,4
Cleaned data
0.0
0.5
1.0
1.5
2.0
2.5
3.0

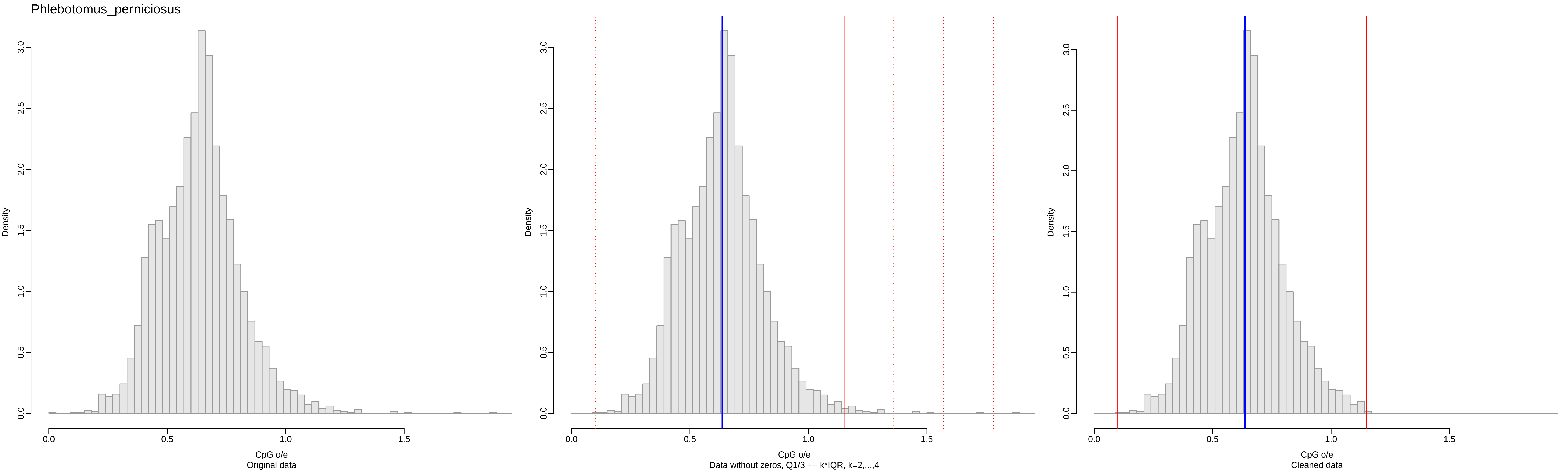

Phlebotomus_perniciosus
Density
CpG o/e
Original data
Data without zeros, Q1/3 +− k*IQR, k=2,...,4
Cleaned data

Phycomyces_blakesleeanus

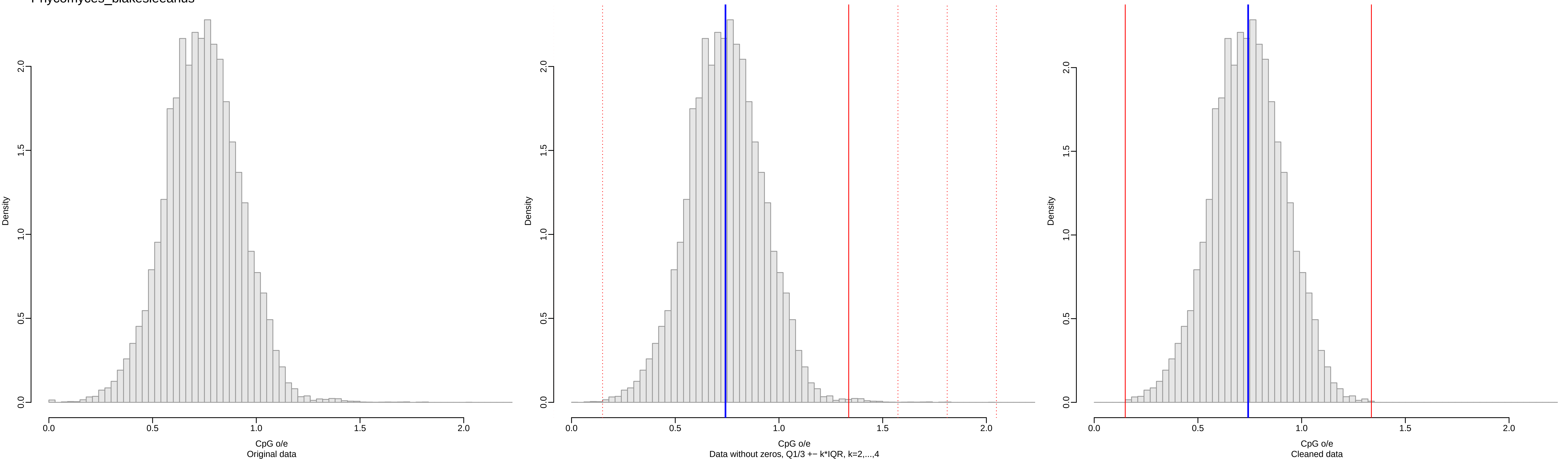

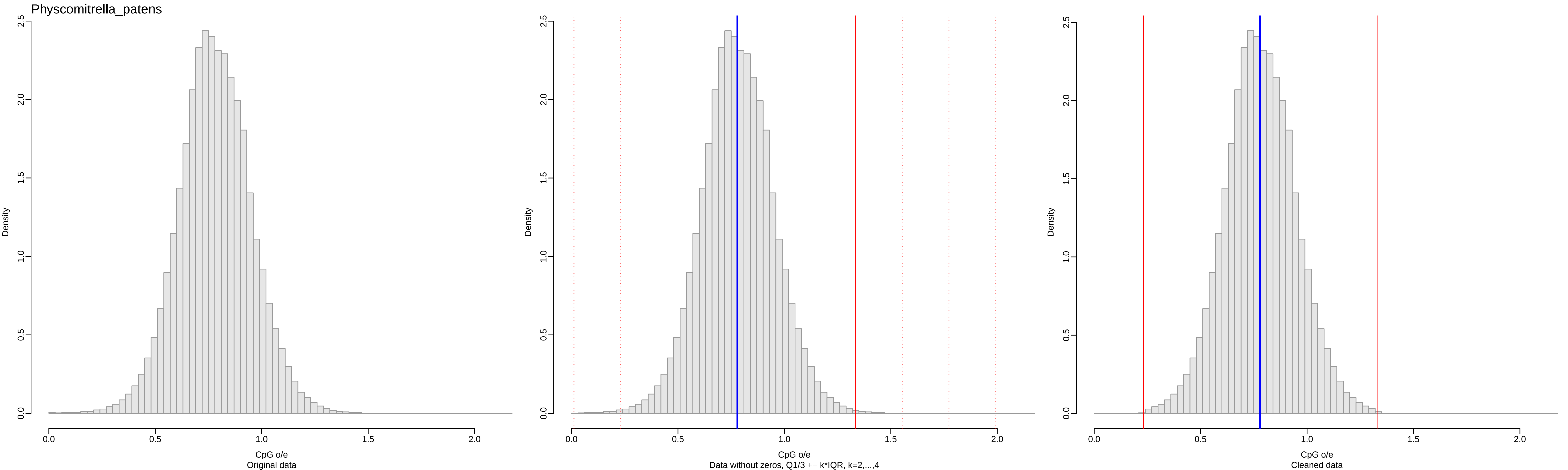
Physcomitrella_patens
Density
CpG o/e
Original data
Data without zeros, Q1/3 +− k*IQR, k=2,...,4
Cleaned data
0.0
0.5
1.0
1.5
2.0
2.5

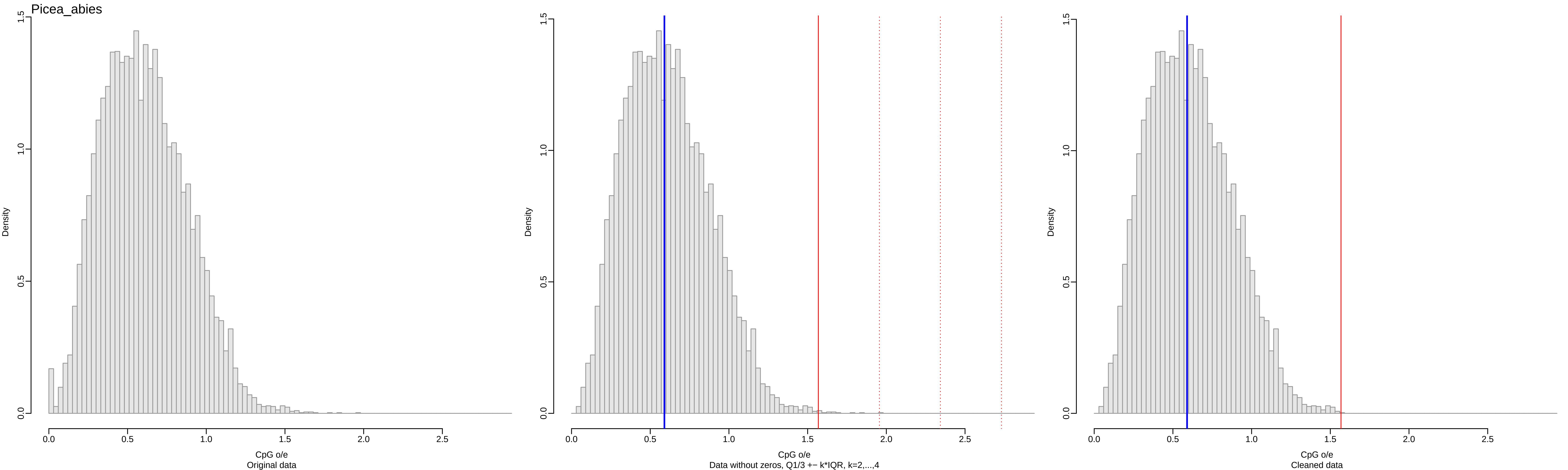
Picea_abies
Density
CpG o/e
Original data
Data without zeros, Q1/3 +− k*IQR, k=2,...,4
Cleaned data
0.0
0.5
1.0
1.5
2.0
2.5

Picea_glauca

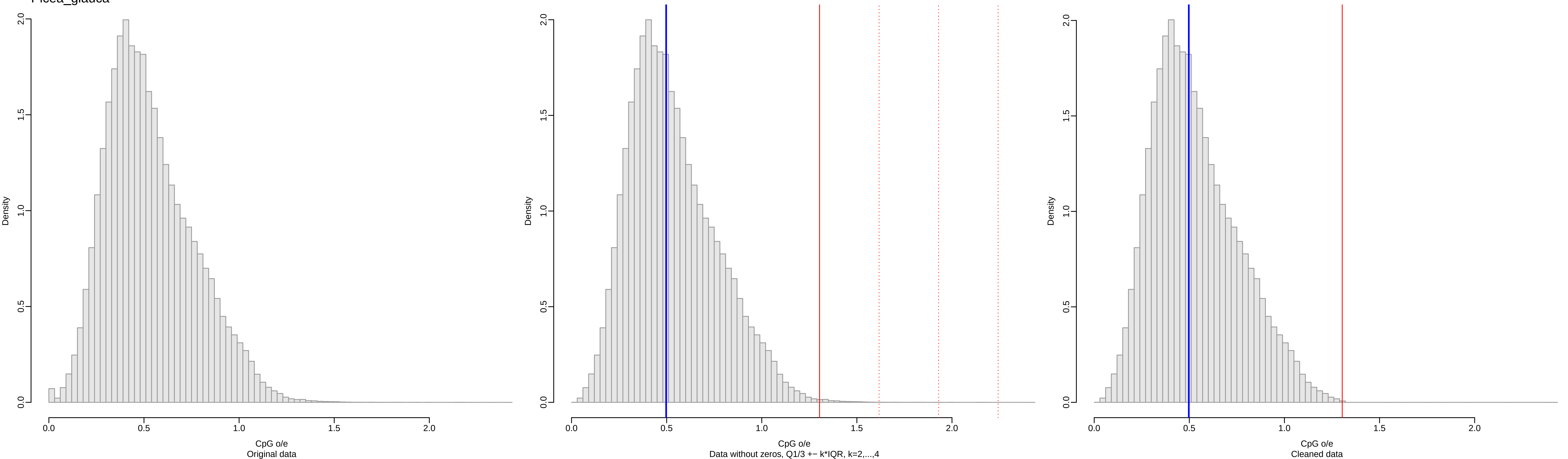

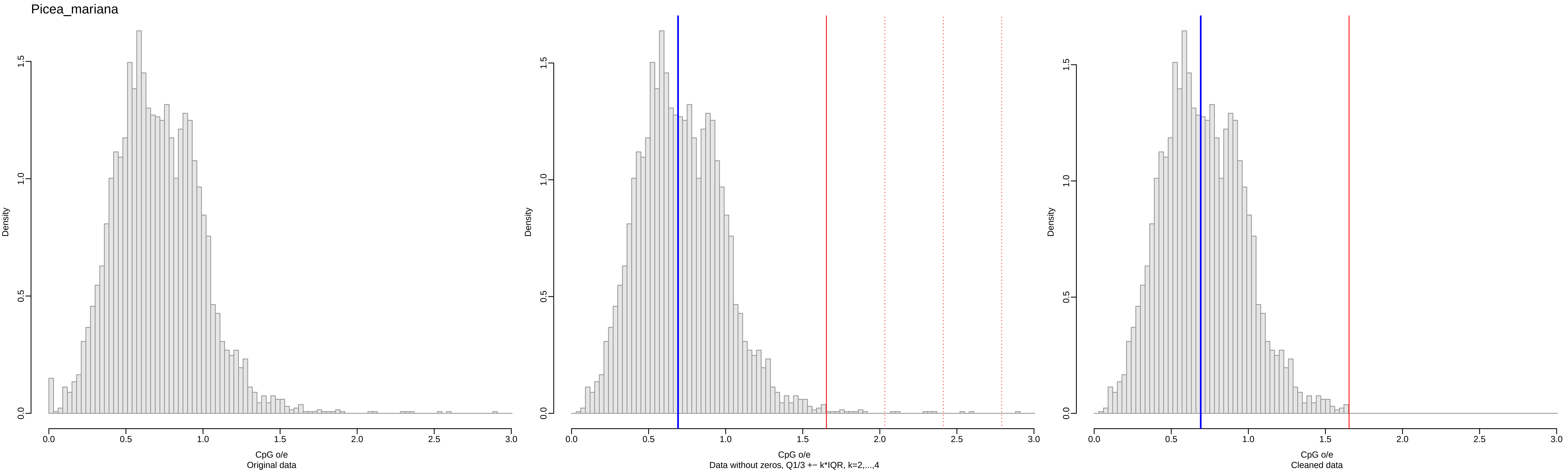

Picea_mariana
Density
CpG o/e
Original data
Data without zeros, Q1/3 +− k*IQR, k=2,...,4
Cleaned data
0.0
0.5
1.0
1.5
2.0
2.5
3.0

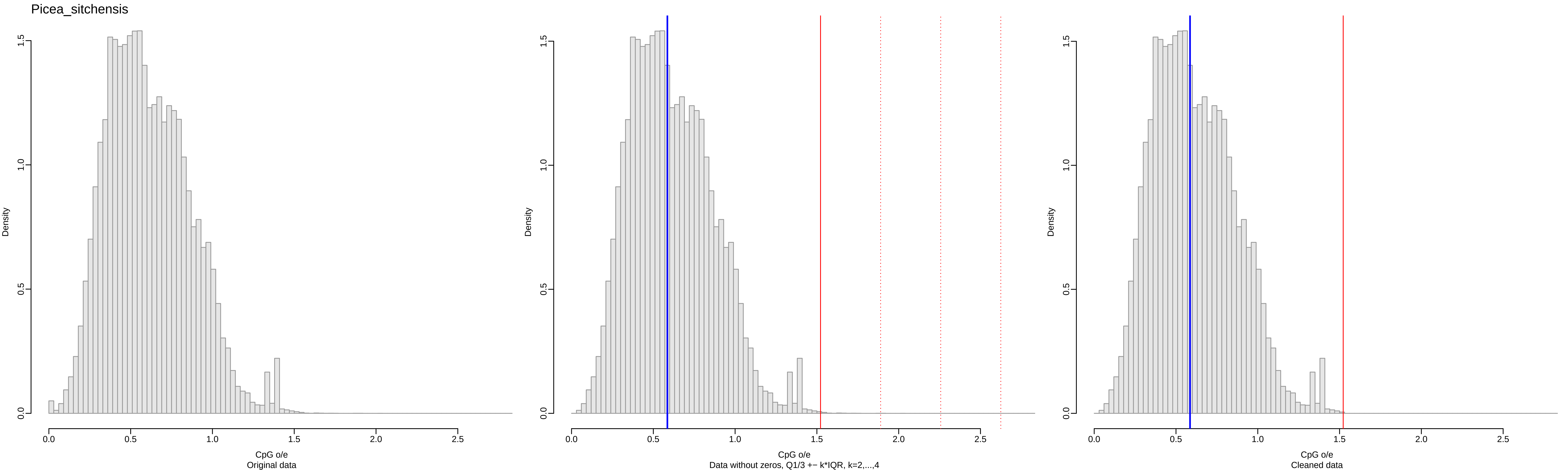
Picea_sitchensis
Density
CpG o/e
Original data
Data without zeros, Q1/3 +− k*IQR, k=2,...,4
Cleaned data
0.0
0.5
1.0
1.5
2.0
2.5

Pichia_stipitis

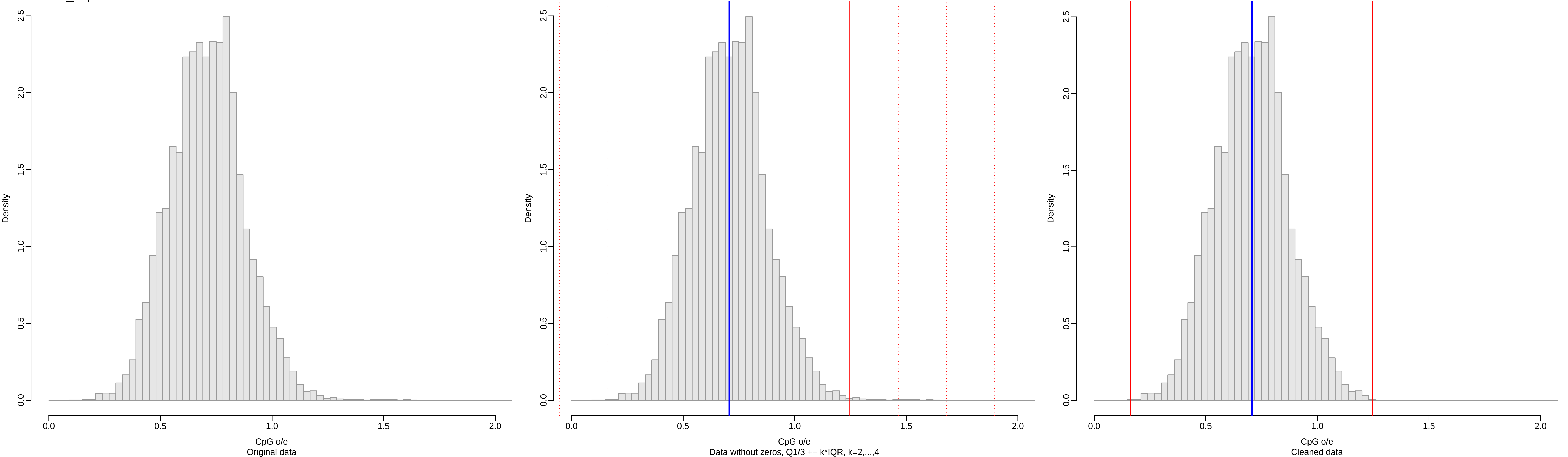

Pimephales_promelas

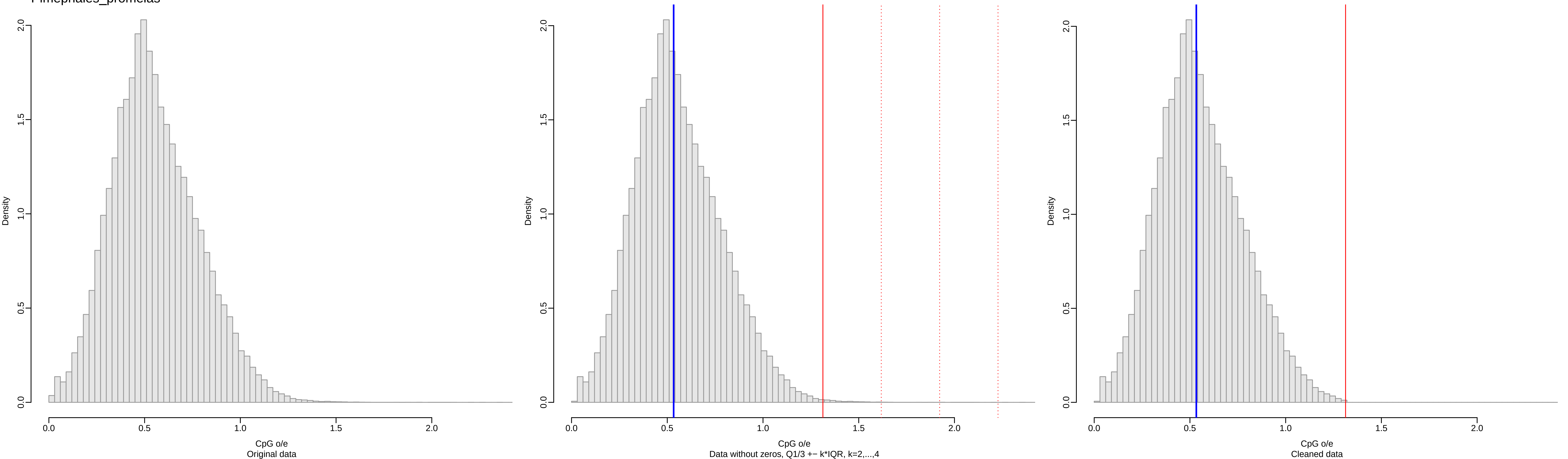

Pinctada_fucata

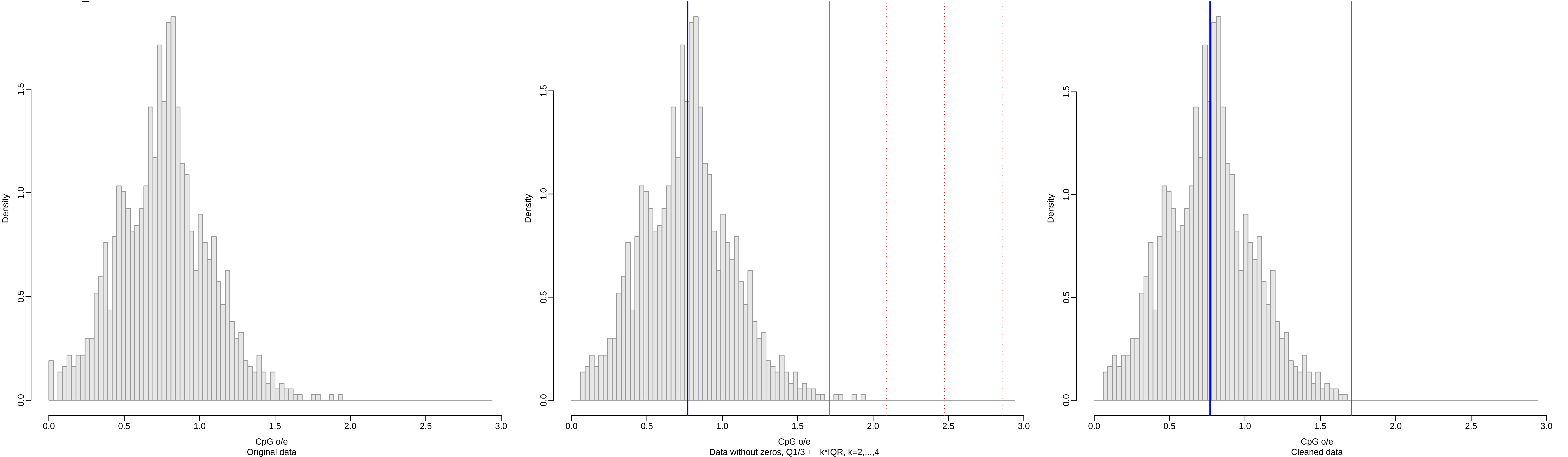

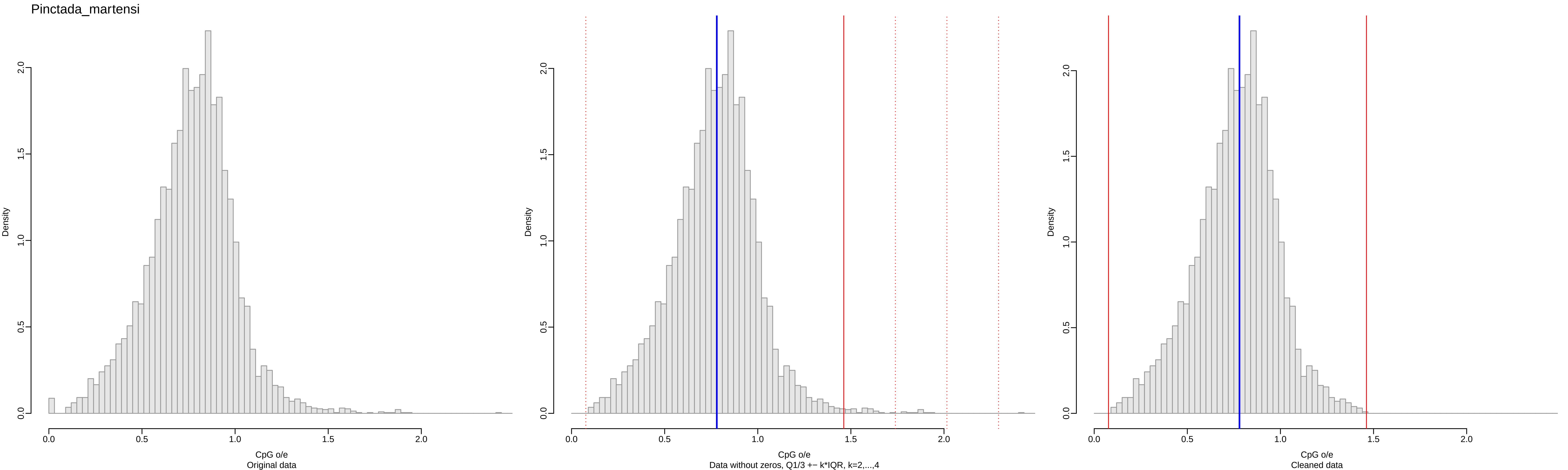

Pinctada_martensi
Density
0.0
0.5
1.0
1.5
2.0
CpG o/e
Original data
Data without zeros, Q1/3 +− k*IQR, k=2,...,4
Cleaned data

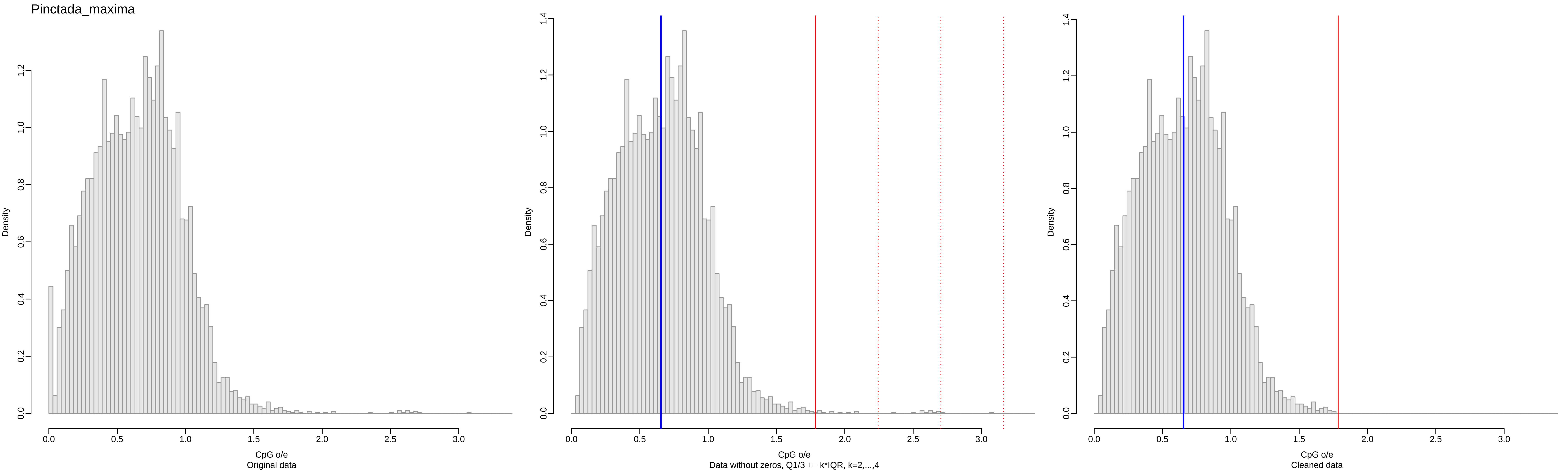
Pinctada_maxima
Density
CpG o/e
Original data
CpG o/e
Data without zeros, Q1/3 +− k*IQR, k=2,...,4
CpG o/e
Cleaned data
0.0
0.2
0.4
0.6
0.8
1.0
1.2
1.4
0.5
1.5
2.0
2.5
3.0

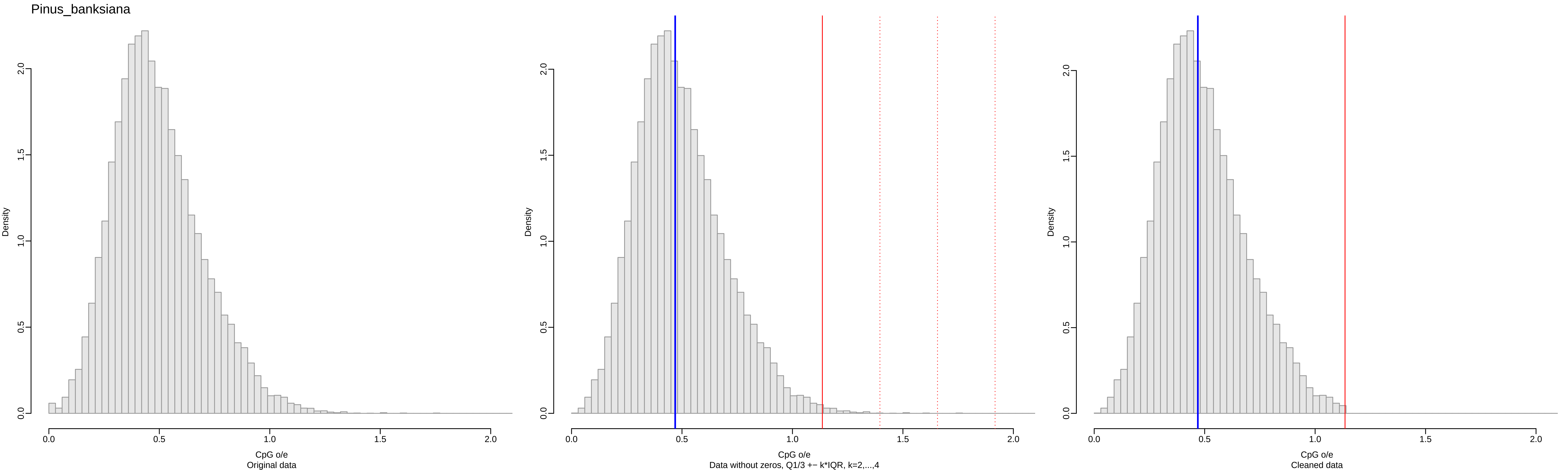

Pinus_banksiana
Density
CpG o/e
Original data
Data without zeros, Q1/3 +− k*IQR, k=2,...,4
Cleaned data

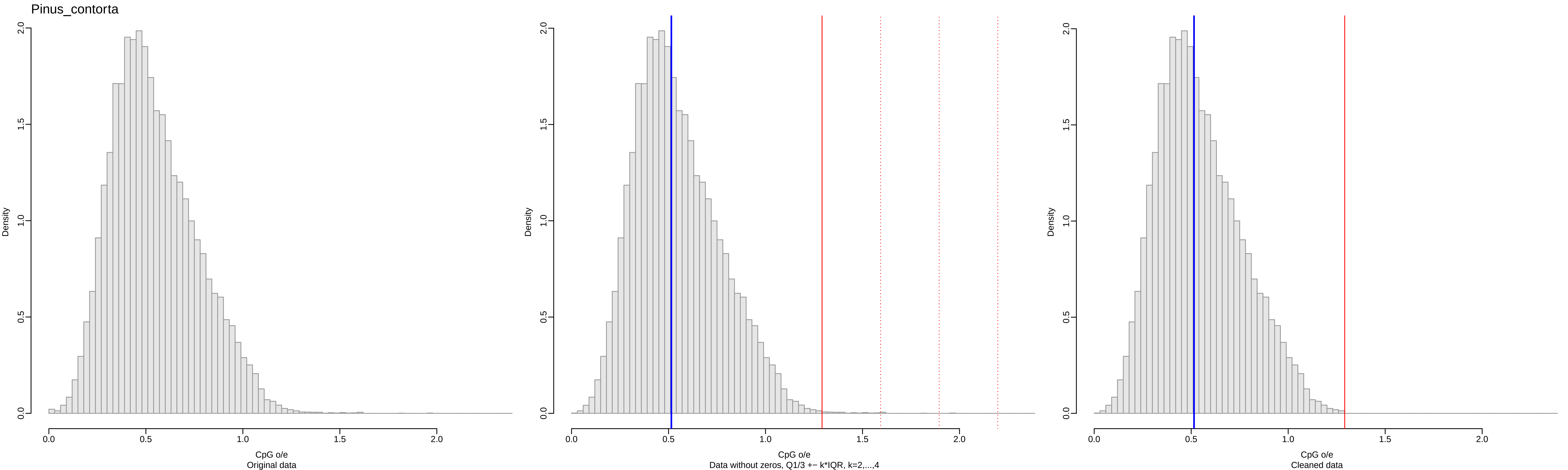

Pinus_contorta
Density
0.0
0.5
1.0
1.5
2.0
CpG o/e
Original data
Data without zeros, Q1/3 +− k*IQR, k=2,...,4
Cleaned data

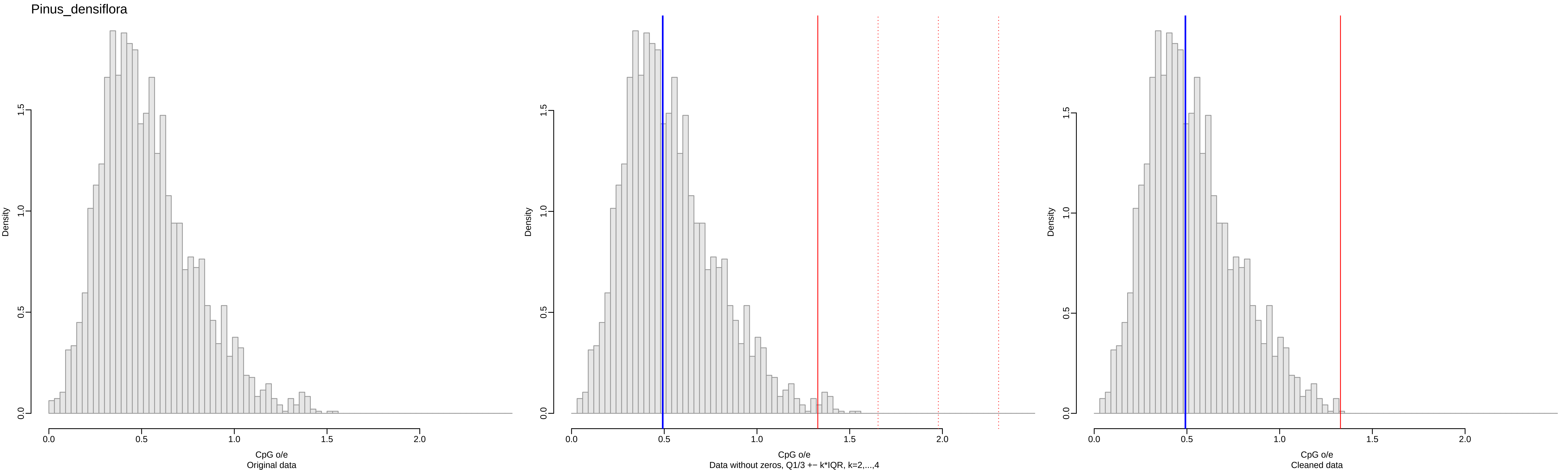
Pinus_densiflora
Density
0.0
0.5
1.0
1.5
2.0
CpG o/e
Original data
Data without zeros, Q1/3 +− k*IQR, k=2,...,4
Cleaned data

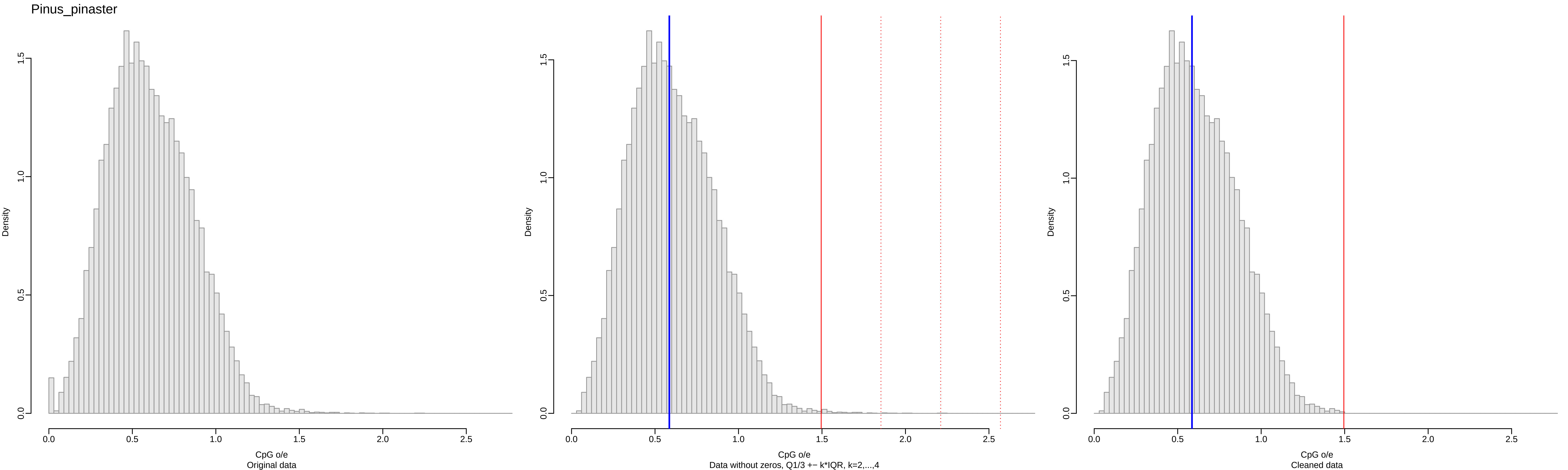

Pinus_pinaster
Density
0.0
0.5
1.0
1.5
2.0
2.5
CpG o/e
Original data
Data without zeros, Q1/3 +− k*IQR, k=2,...,4
Cleaned data

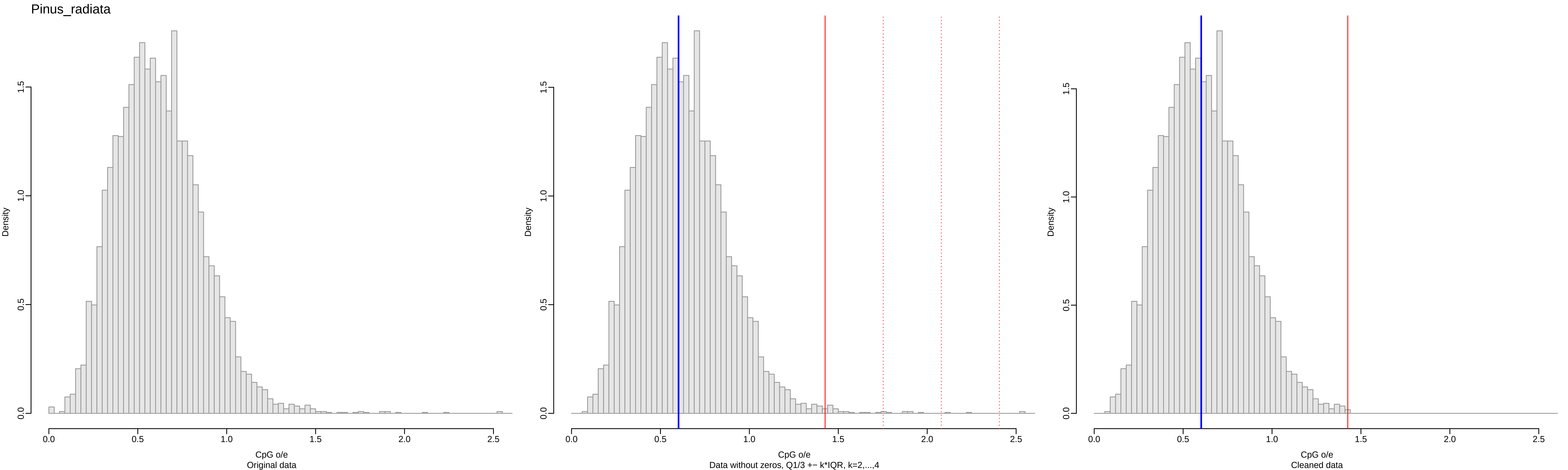
Pinus_radiata
Density
CpG o/e
Original data
CpG o/e
Data without zeros, Q1/3 +− k*IQR, k=2,...,4
CpG o/e
Cleaned data
0.0
0.5
1.0
1.5
2.0
2.5

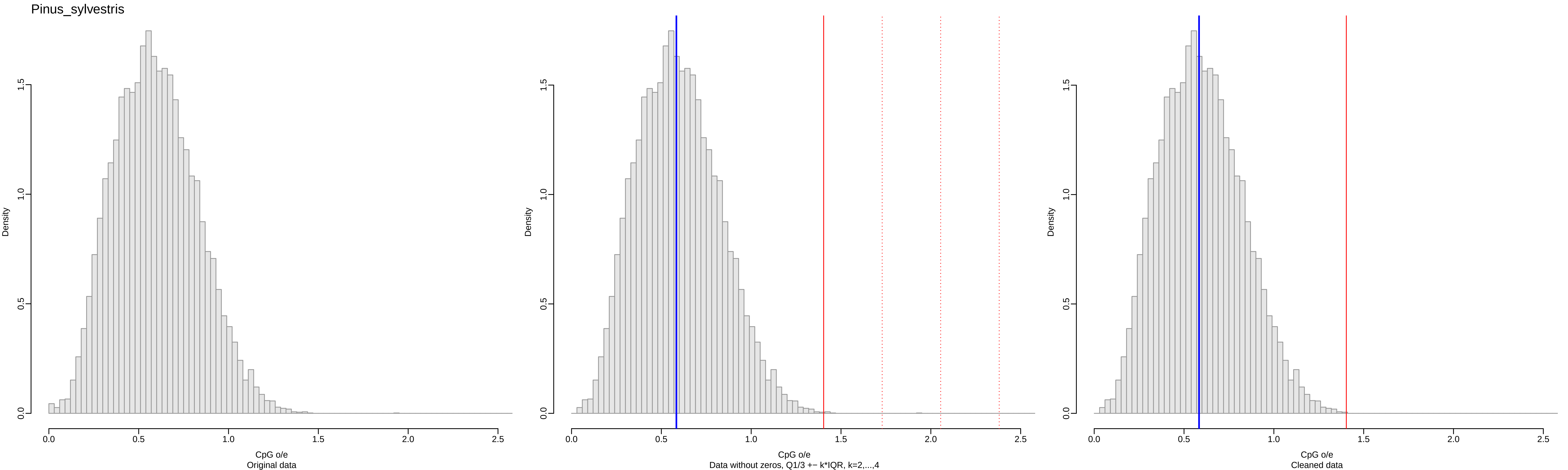
Pinus_sylvestris
Density
0.0
0.5
1.0
1.5
2.0
2.5
CpG o/e
Original data
Data without zeros, Q1/3 +− k*IQR, k=2,...,4
Cleaned data

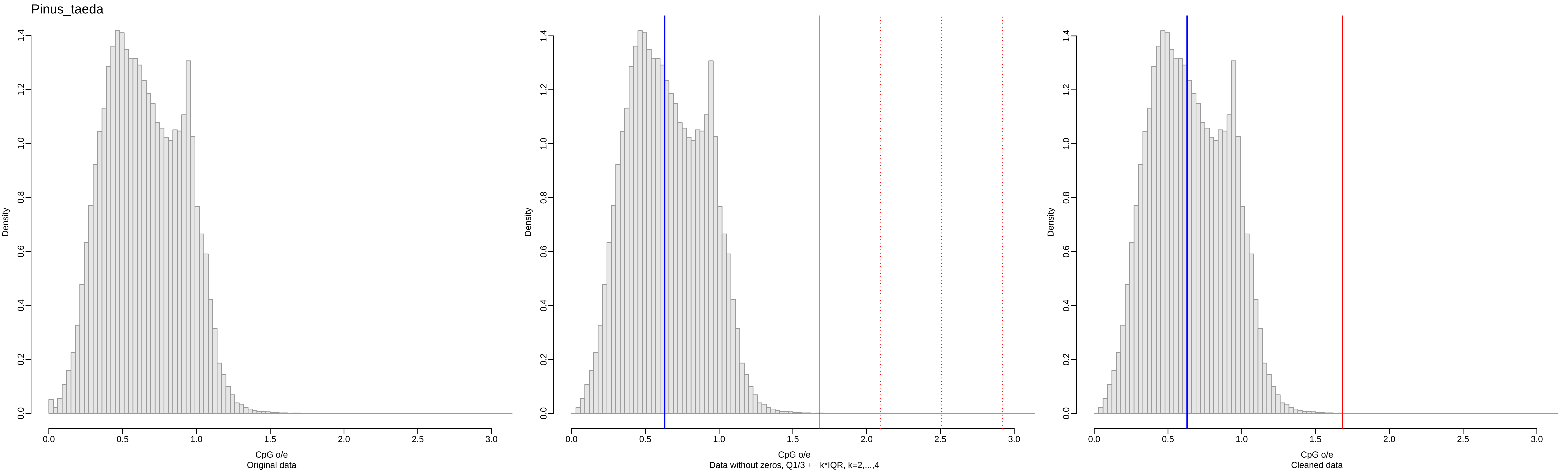

Pinus_taeda
Density
0.0
0.2
0.4
0.6
0.8
1.0
1.2
1.4
0.0
0.5
1.0
1.5
2.0
2.5
3.0
CpG o/e
Original data
CpG o/e
Data without zeros, Q1/3 +− k*IQR, k=2,...,4
CpG o/e
Cleaned data

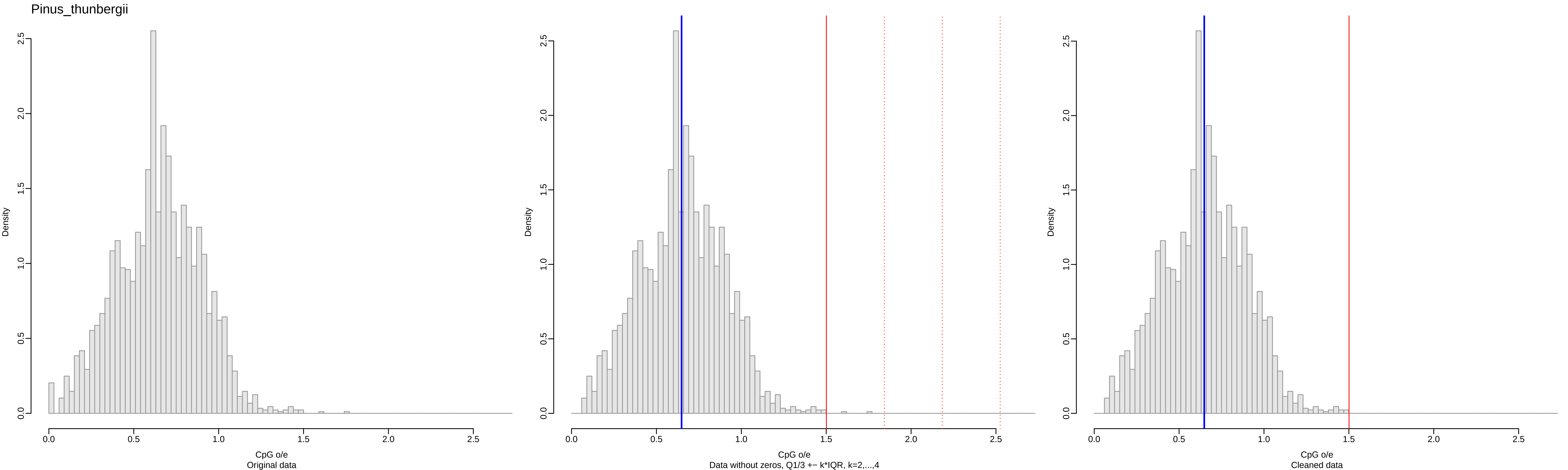

Pinus_thunbergii
Density
CpG o/e
Original data
CpG o/e
Data without zeros, Q1/3 +− k*IQR, k=2,...,4
CpG o/e
Cleaned data

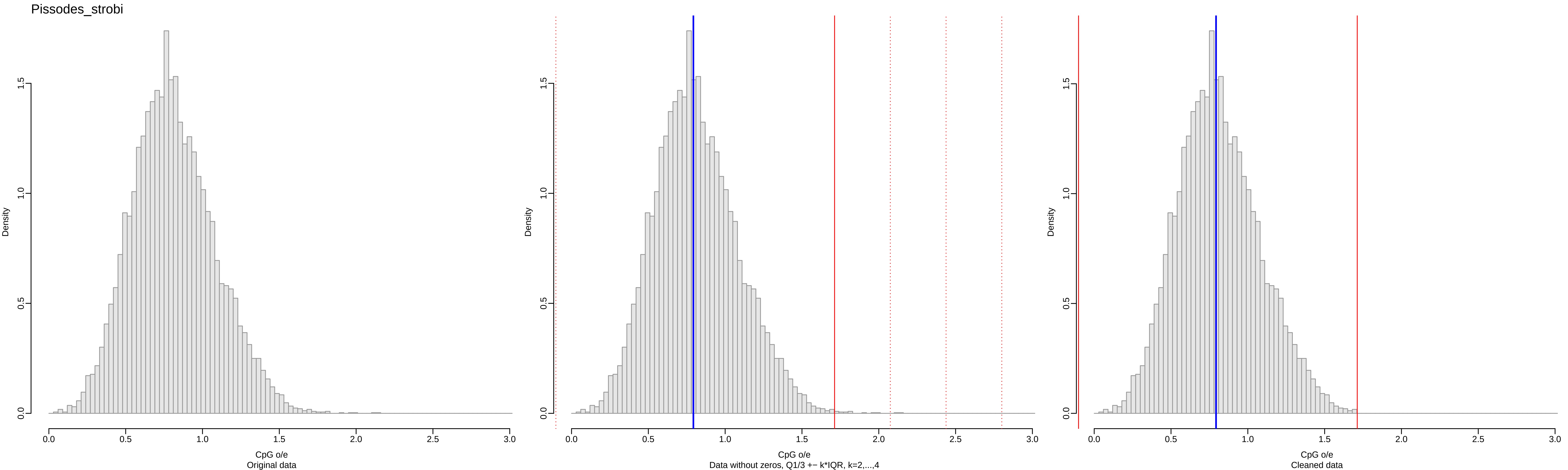

Pissodes_strobi
Density
CpG o/e
Original data
Data without zeros, Q1/3 +− k*IQR, k=2,...,4
Cleaned data
0.0
0.5
1.0
1.5
2.0
2.5
3.0

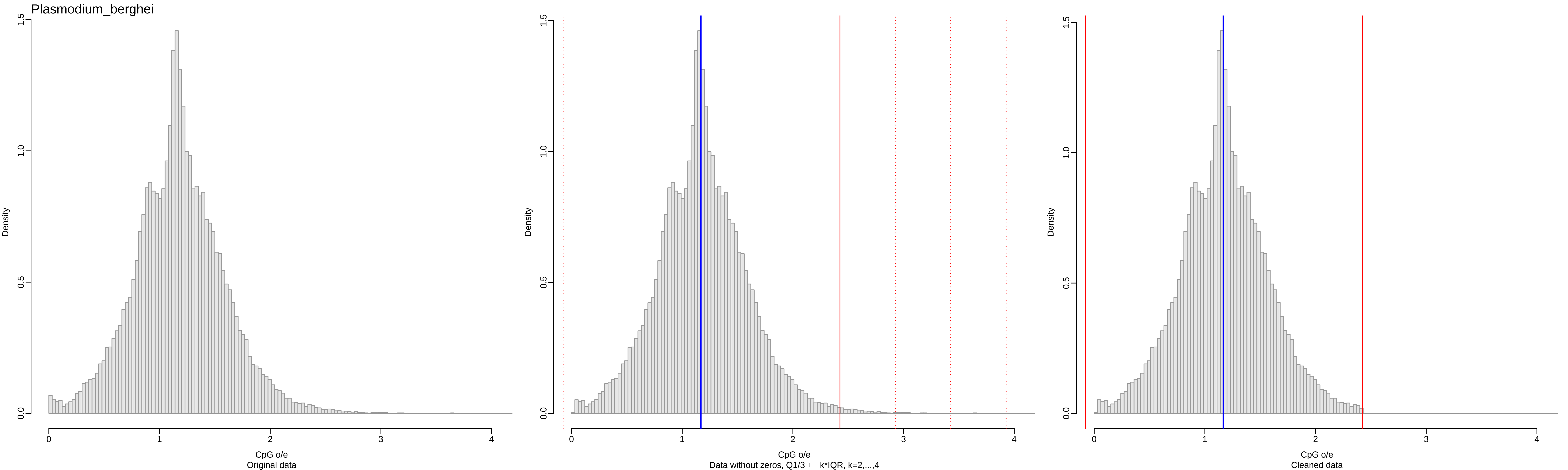

Plasmodium_berghei
Density
0.0
0.5
1.0
1.5
0
1
2
3
4
CpG o/e
Original data
Data without zeros, Q1/3 +− k*IQR, k=2,...,4
Cleaned data

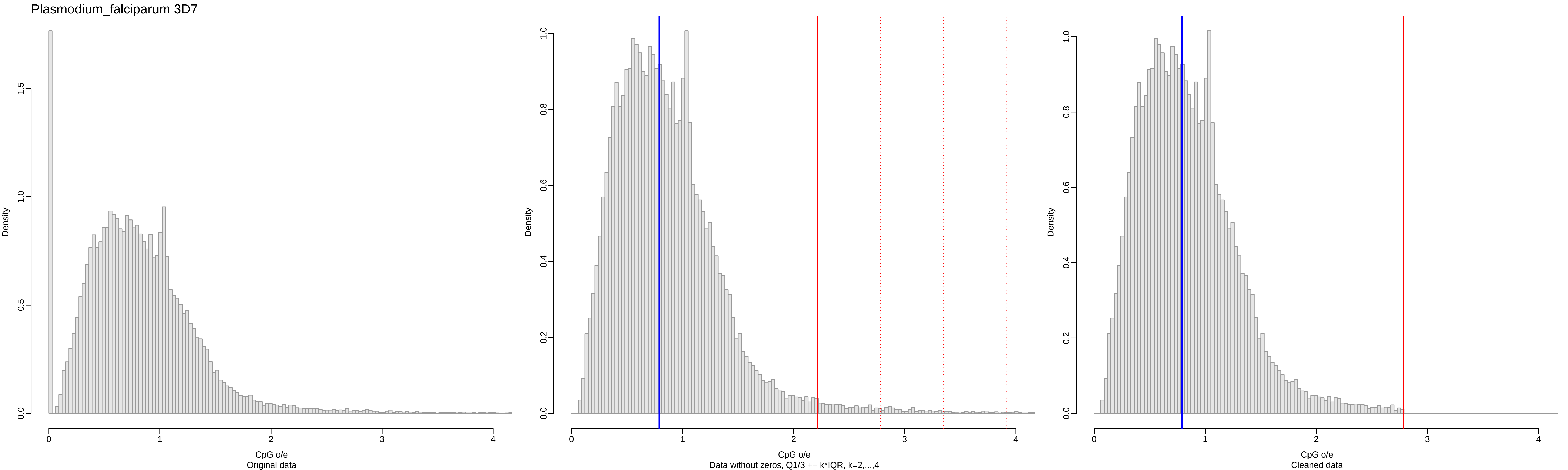

Plasmodium_falciparum 3D7
Density
CpG o/e
Original data
Density
CpG o/e
Data without zeros, Q1/3 +− k*IQR, k=2,...,4
Density
CpG o/e
Cleaned data

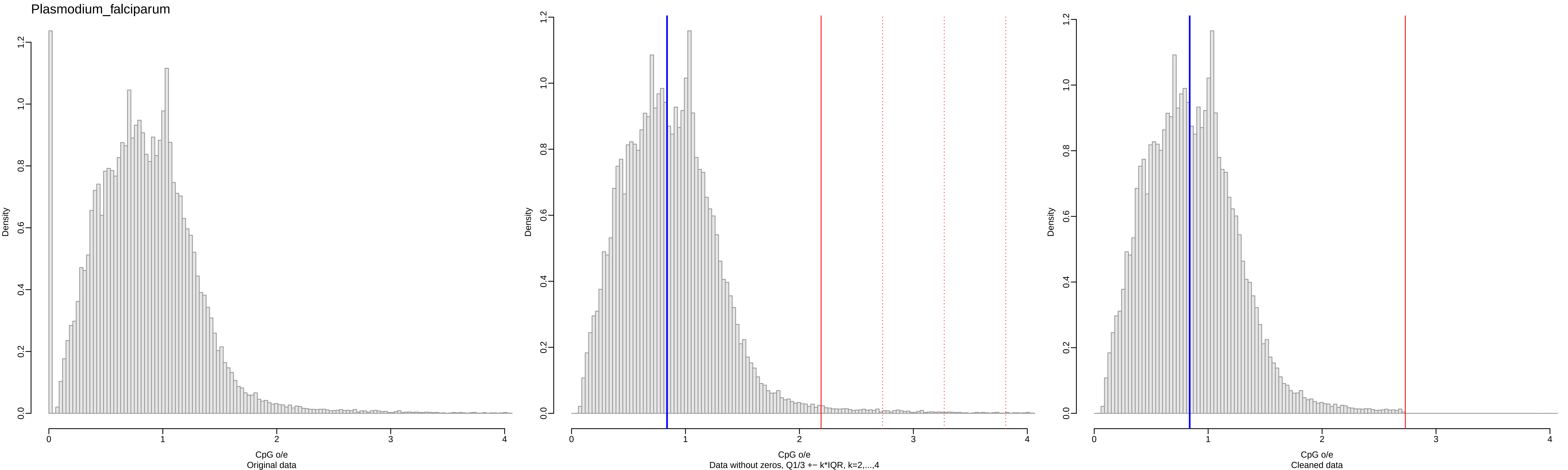
Plasmodium_falciparum
Density
CpG o/e
Original data
Data without zeros, Q1/3 +− k*IQR, k=2,...,4
Cleaned data
0.0
0.2
0.4
0.6
0.8
1.0
1.2
0
1
2
3
4

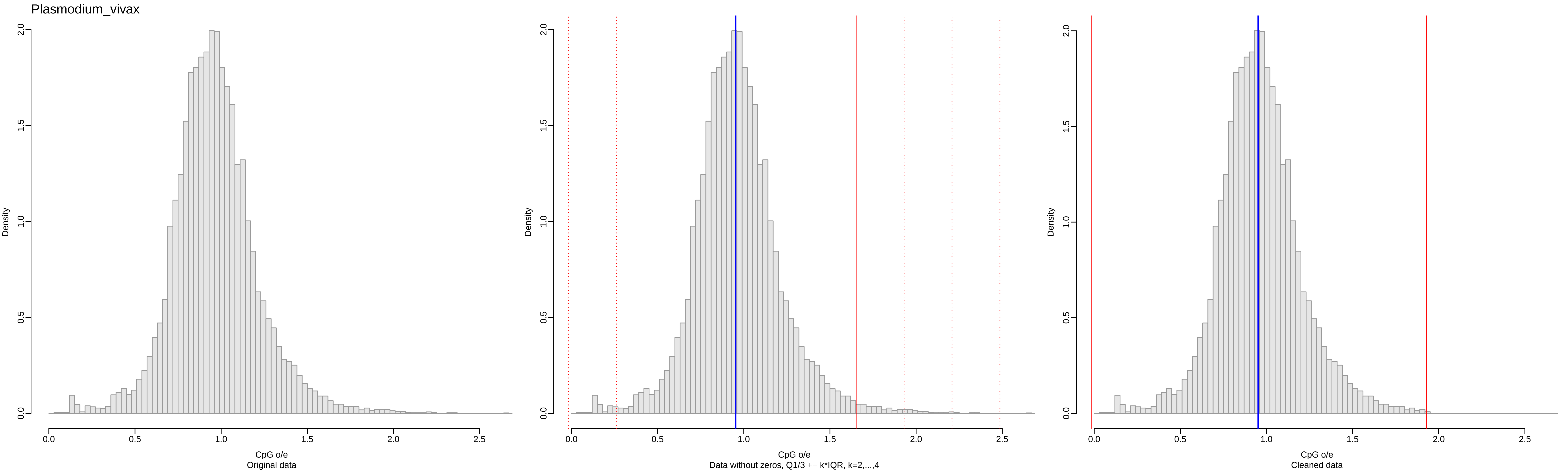

Plasmodium_vivax
Density
CpG o/e
Original data
Density
CpG o/e
Data without zeros, Q1/3 +− k*IQR, k=2,...,4
Density
CpG o/e
Cleaned data

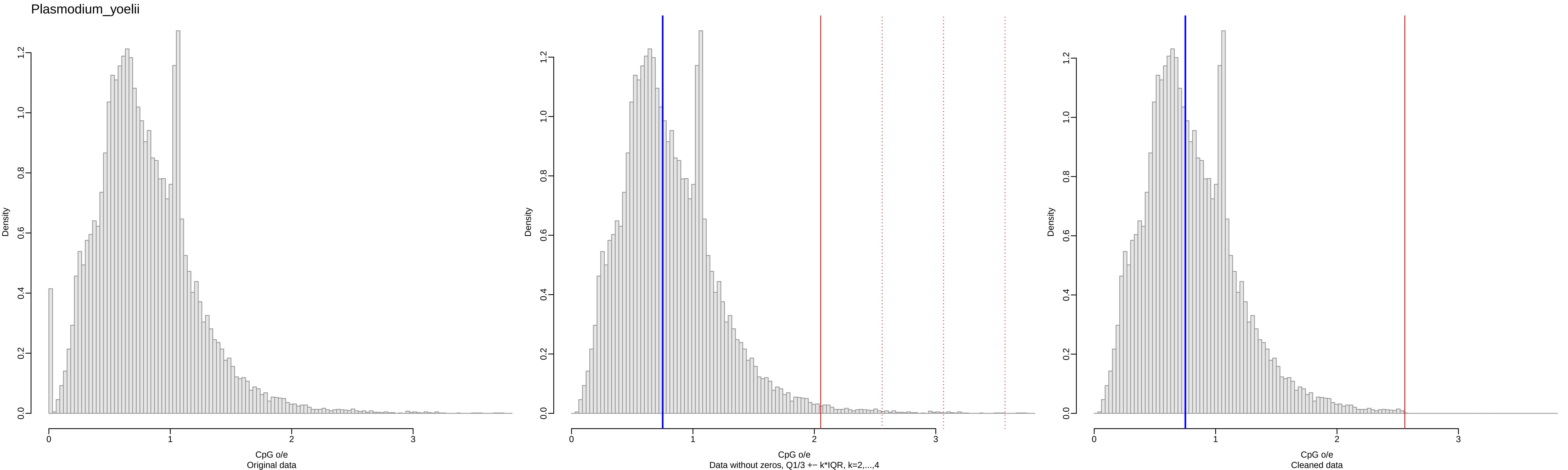
Plasmodium_yoelii
Density
CpG o/e
Original data
Density
CpG o/e
Data without zeros, Q1/3 +− k*IQR, k=2,...,4
Density
CpG o/e
Cleaned data

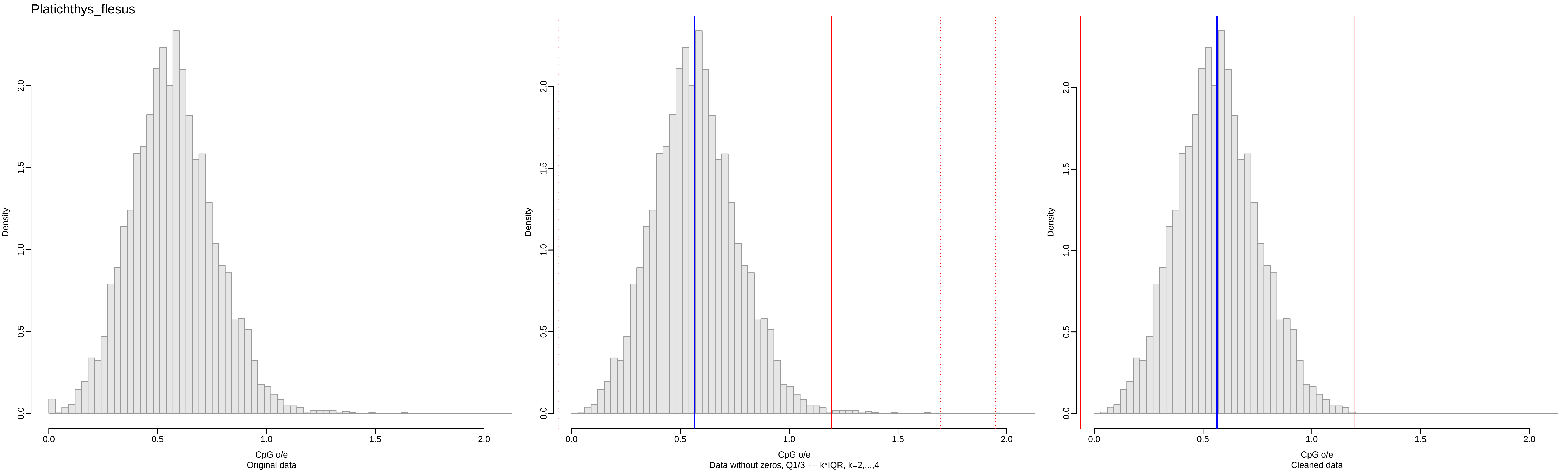

Platichthys_flesus
Density
CpG o/e
Original data
Data without zeros, Q1/3 +− k*IQR, k=2,...,4
Cleaned data
0.0
0.5
1.0
1.5
2.0

Pleurobrachia_pileus

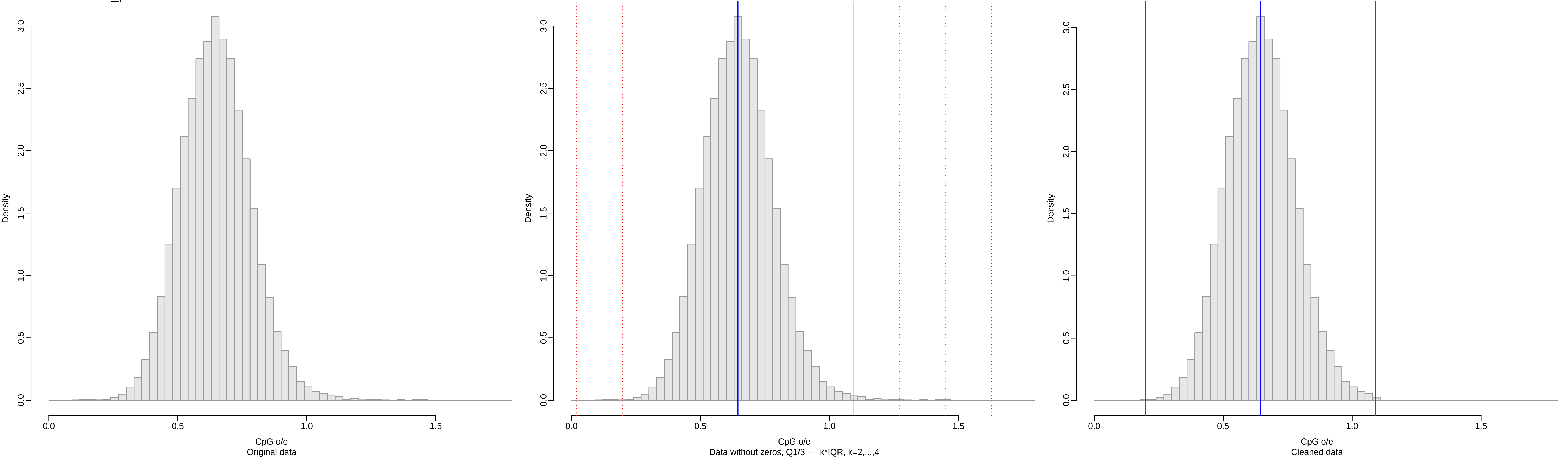

Pleuronectes_platessa

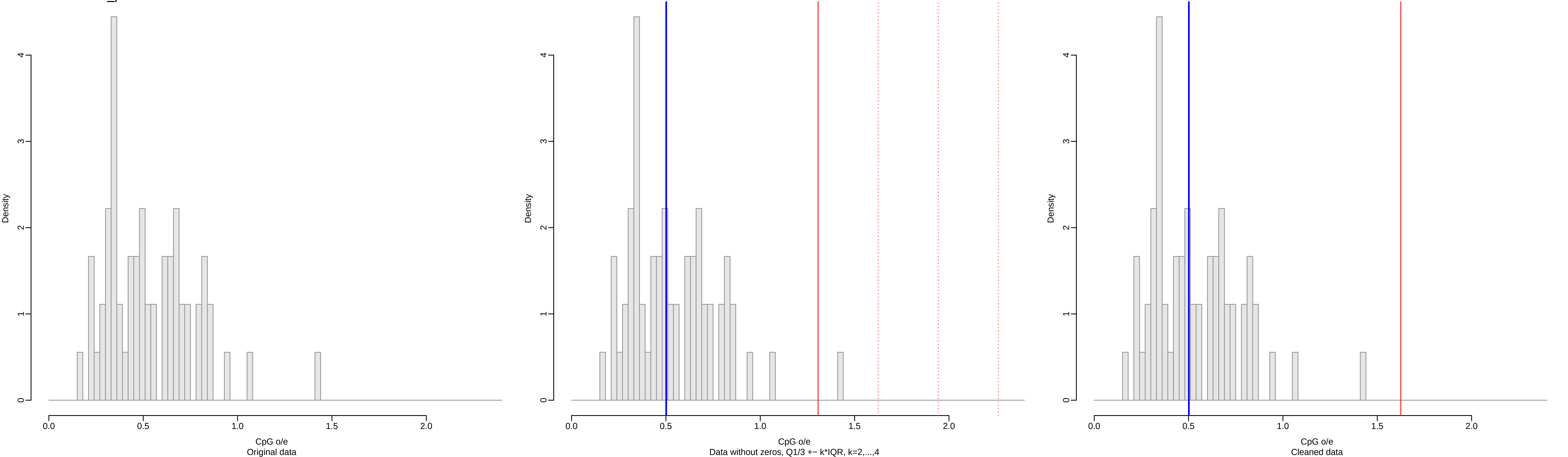

Plutella_xylostella

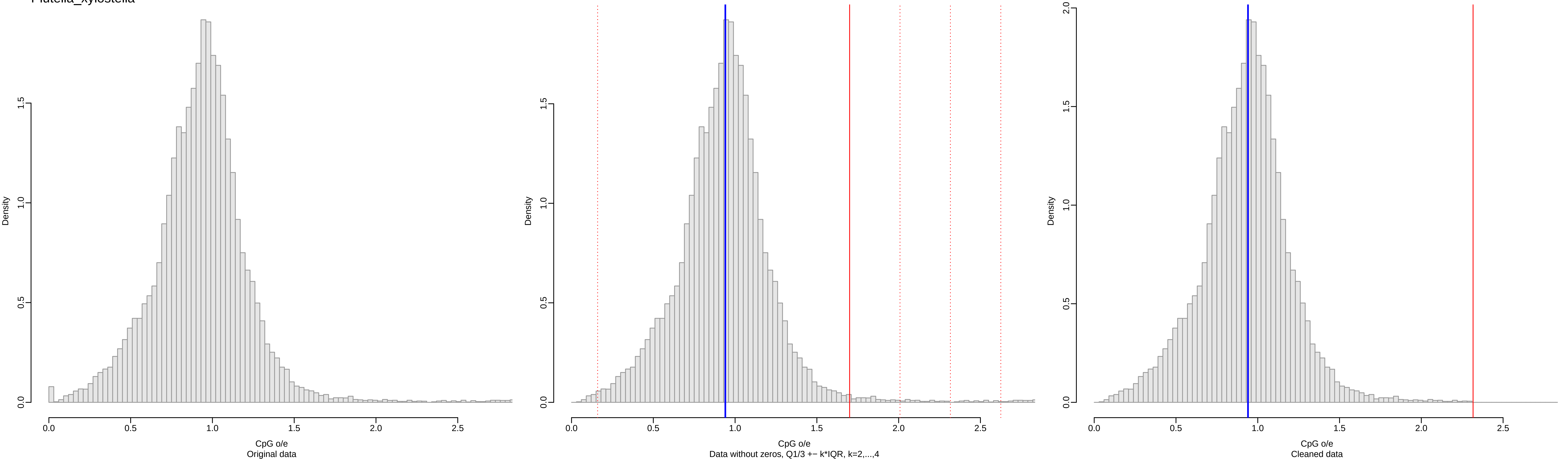

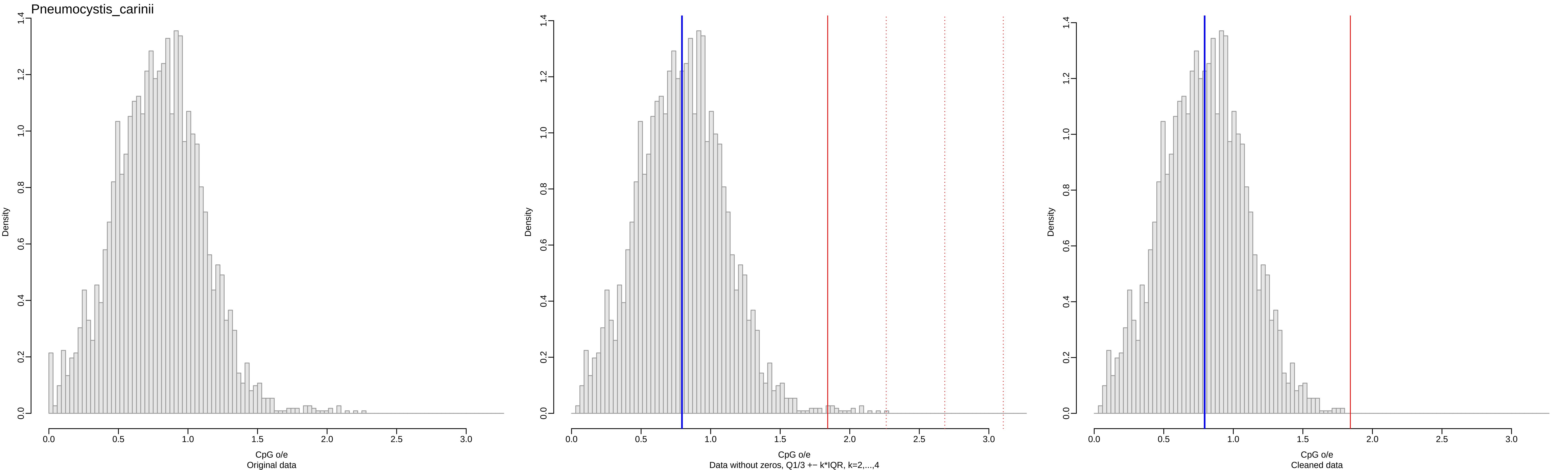

Pneumocystis_carinii
Density
CpG o/e
Original data
Data without zeros, Q1/3 +− k*IQR, k=2,...,4
Cleaned data

Podocoryne_carnea

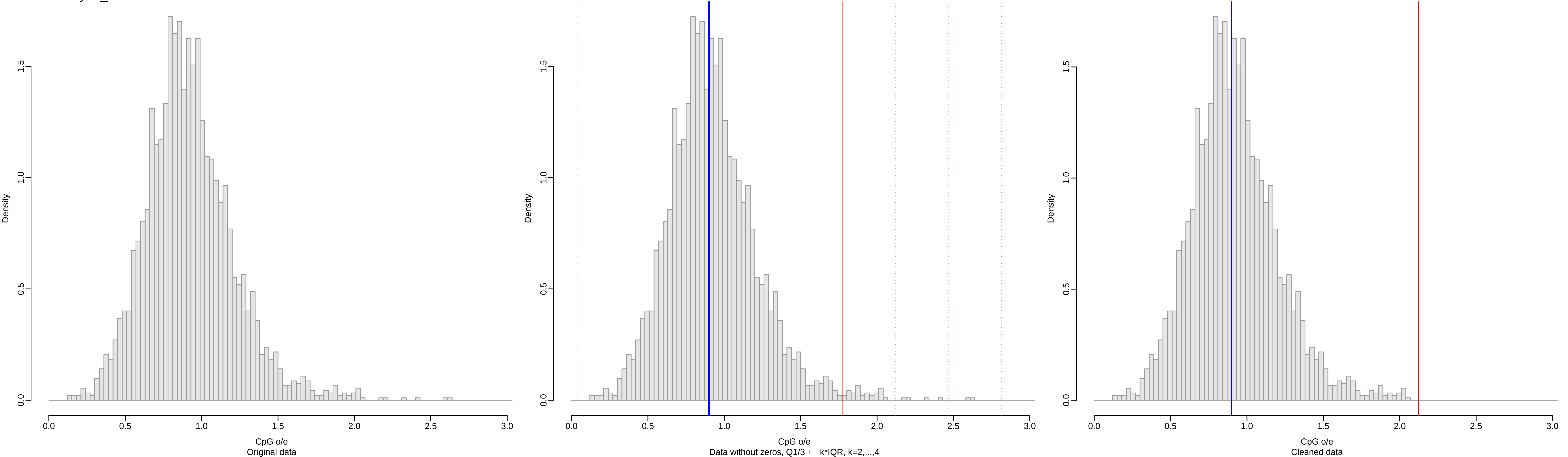

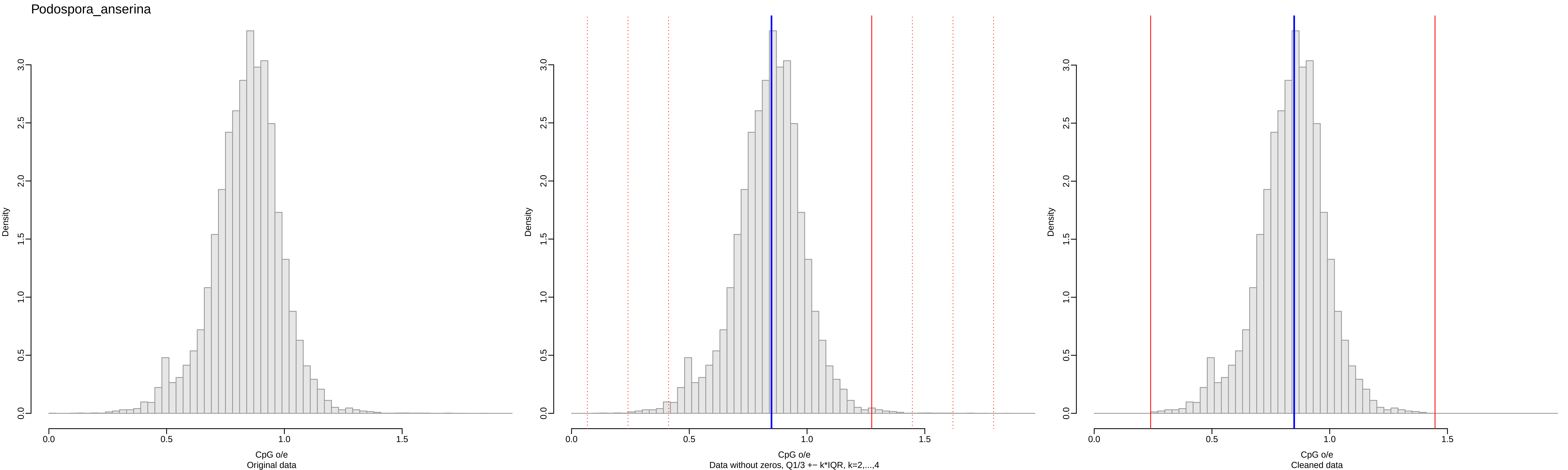
Podospora_anserina
Density
CpG o/e
Original data
Data without zeros, Q1/3 +− k*IQR, k=2,...,4
Cleaned data

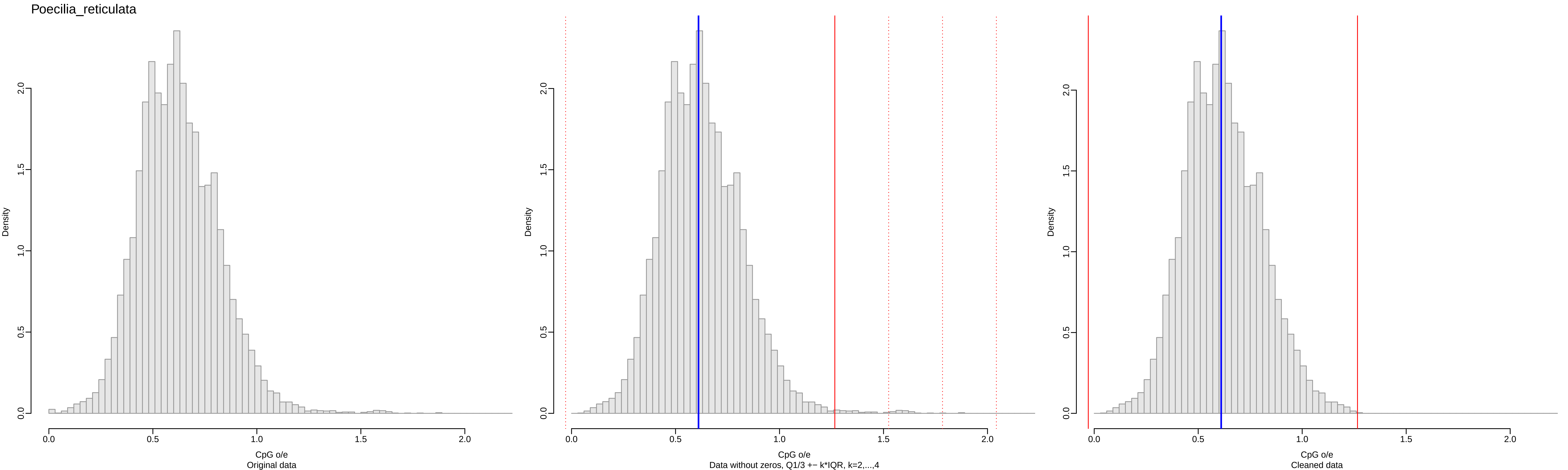

Poecilia_reticulata
Density
CpG o/e
Original data
Data without zeros, Q1/3 +− k*IQR, k=2,...,4
Cleaned data
0.0
0.5
1.0
1.5
2.0

Pollicipes_pollicipes

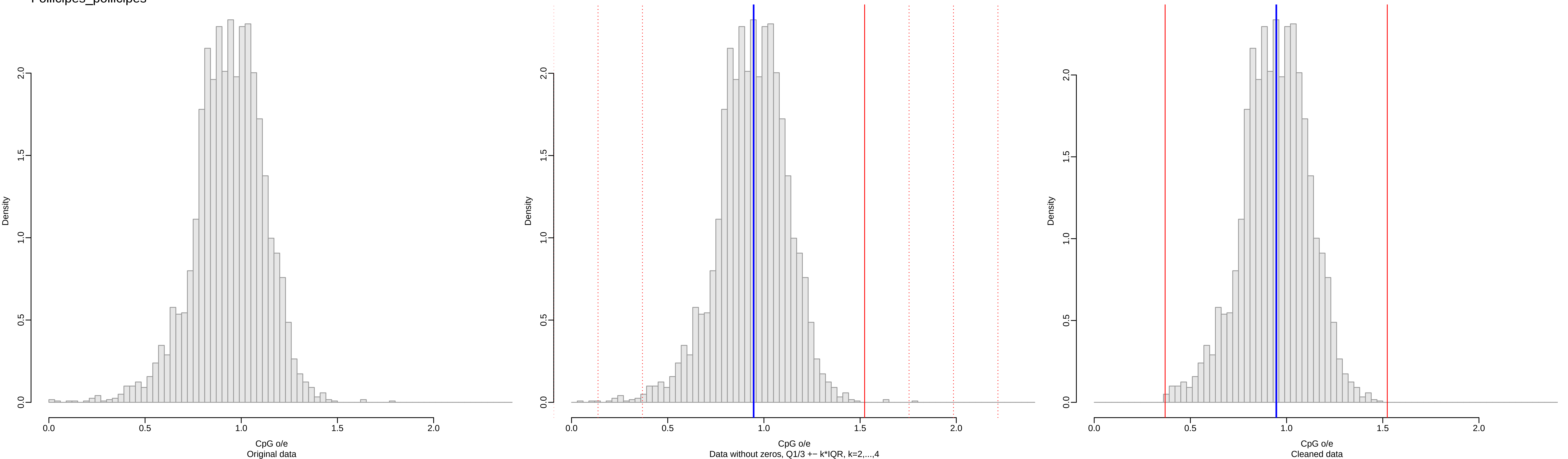

Polygonum_minus

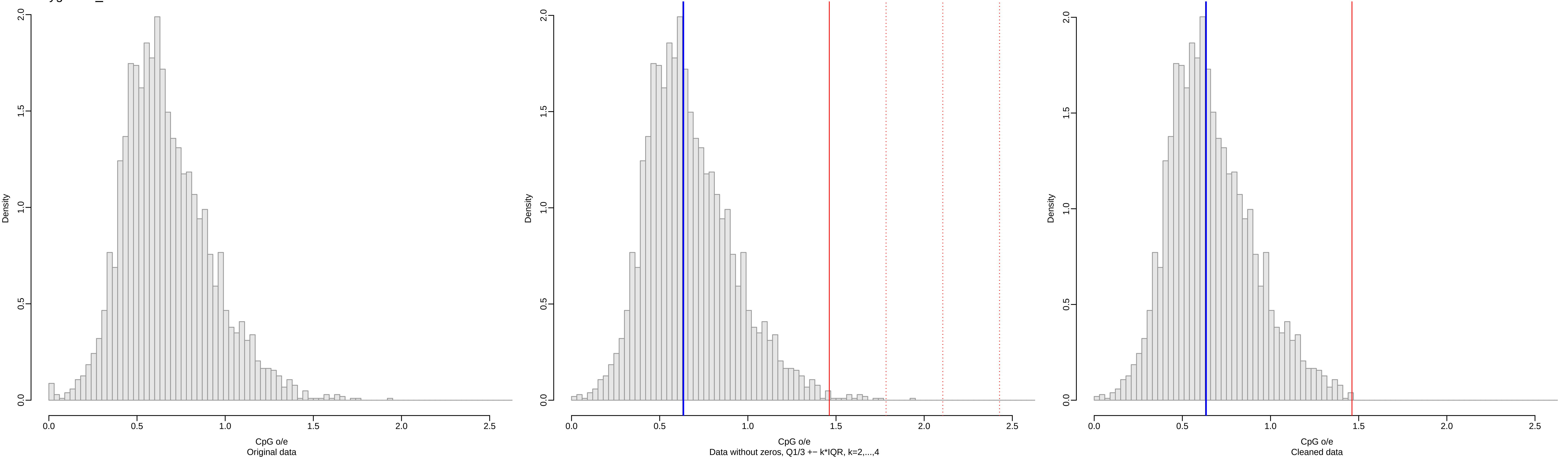

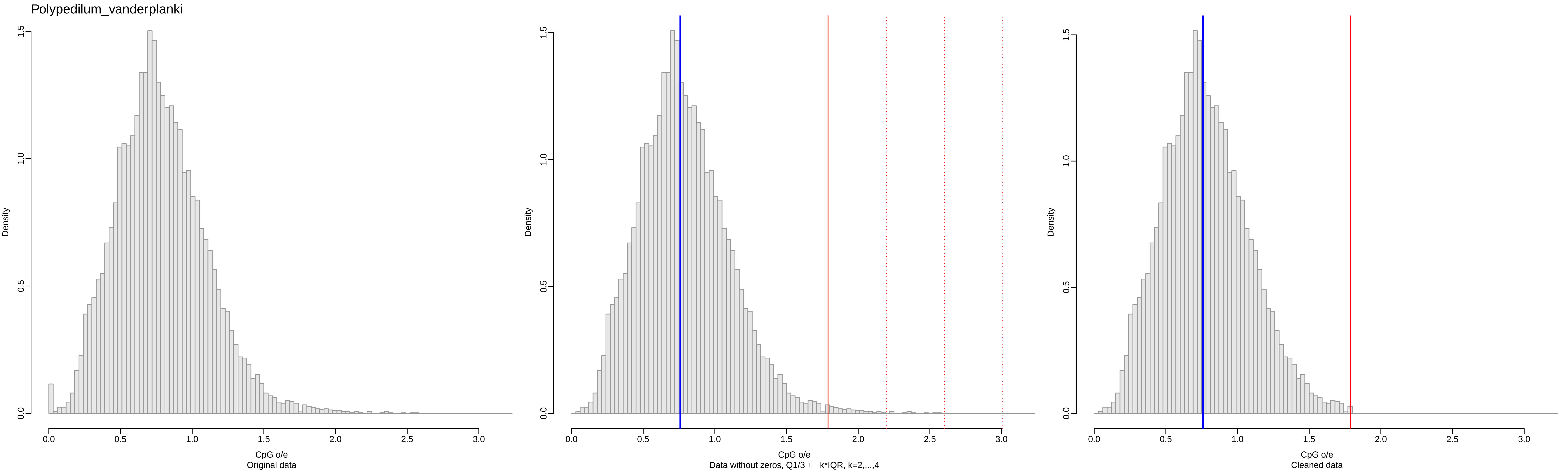
Polypedilum_vanderplanki
Density
CpG o/e
Original data
Data without zeros, Q1/3 +− k*IQR, k=2,...,4
Cleaned data
0.0
0.5
1.0
1.5
2.0
2.5
3.0

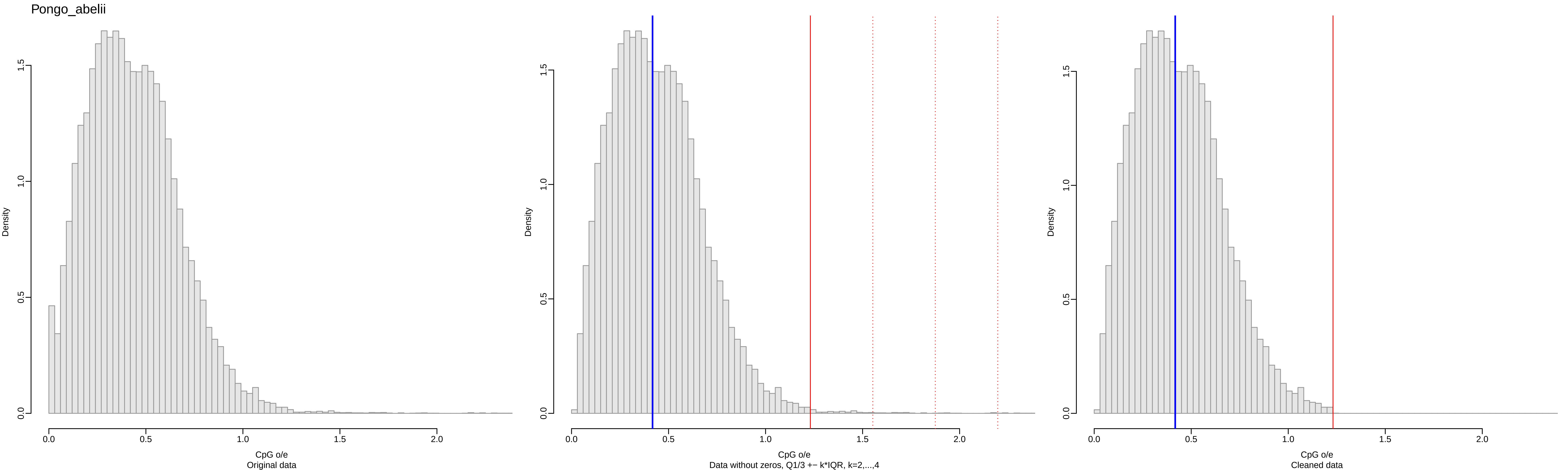
Pongo_abelii
Density
0.0
0.5
1.0
1.5
2.0
CpG o/e
Original data
Data without zeros, Q1/3 +− k*IQR, k=2,...,4
Cleaned data

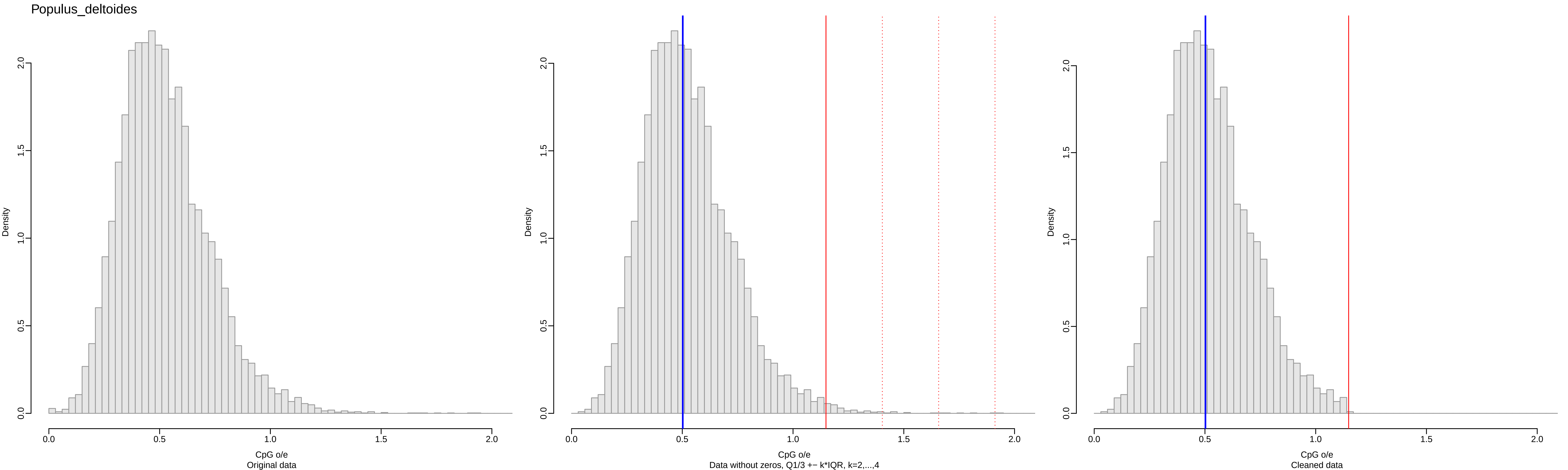

Populus_deltoides
Density
CpG o/e
Original data
Data without zeros, Q1/3 +− k*IQR, k=2,...,4
Cleaned data
0.0
0.5
1.0
1.5
2.0

Populus_euphratica

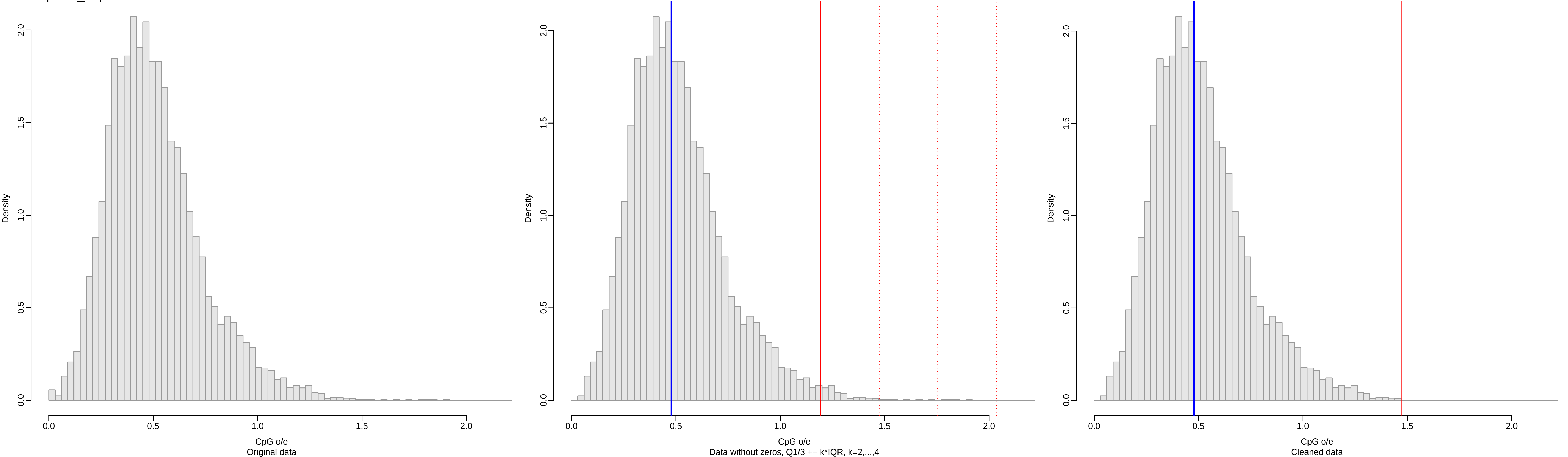

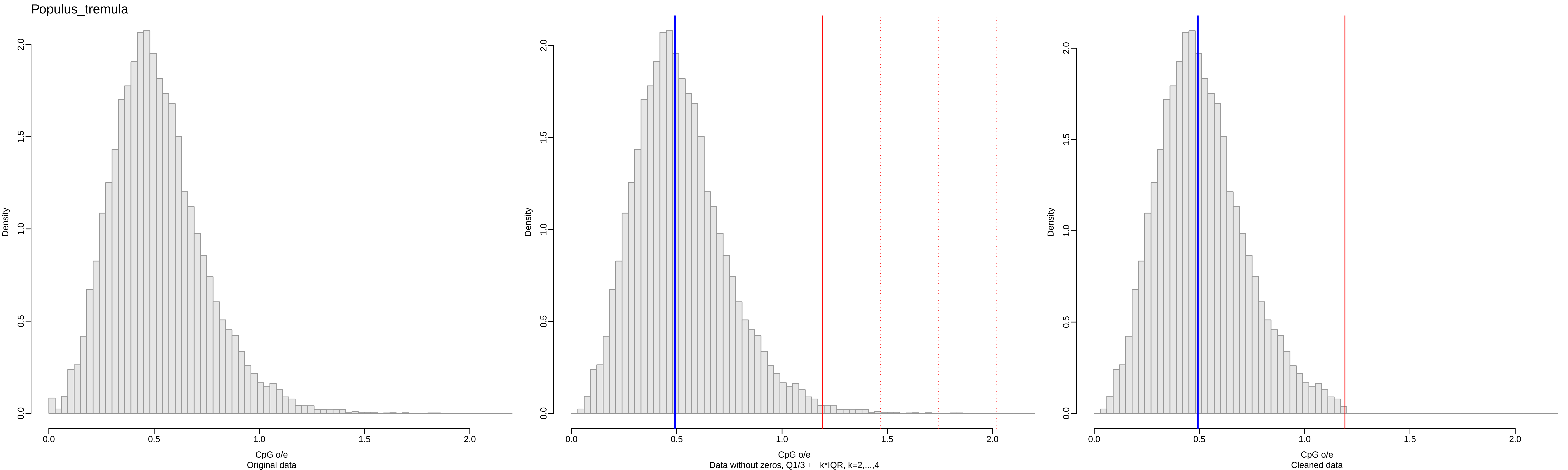

Populus_tremula
Density
CpG o/e
Original data
Density
CpG o/e
Data without zeros, Q1/3 +− k*IQR, k=2,...,4
Density
CpG o/e
Cleaned data

Populus_tremuloides

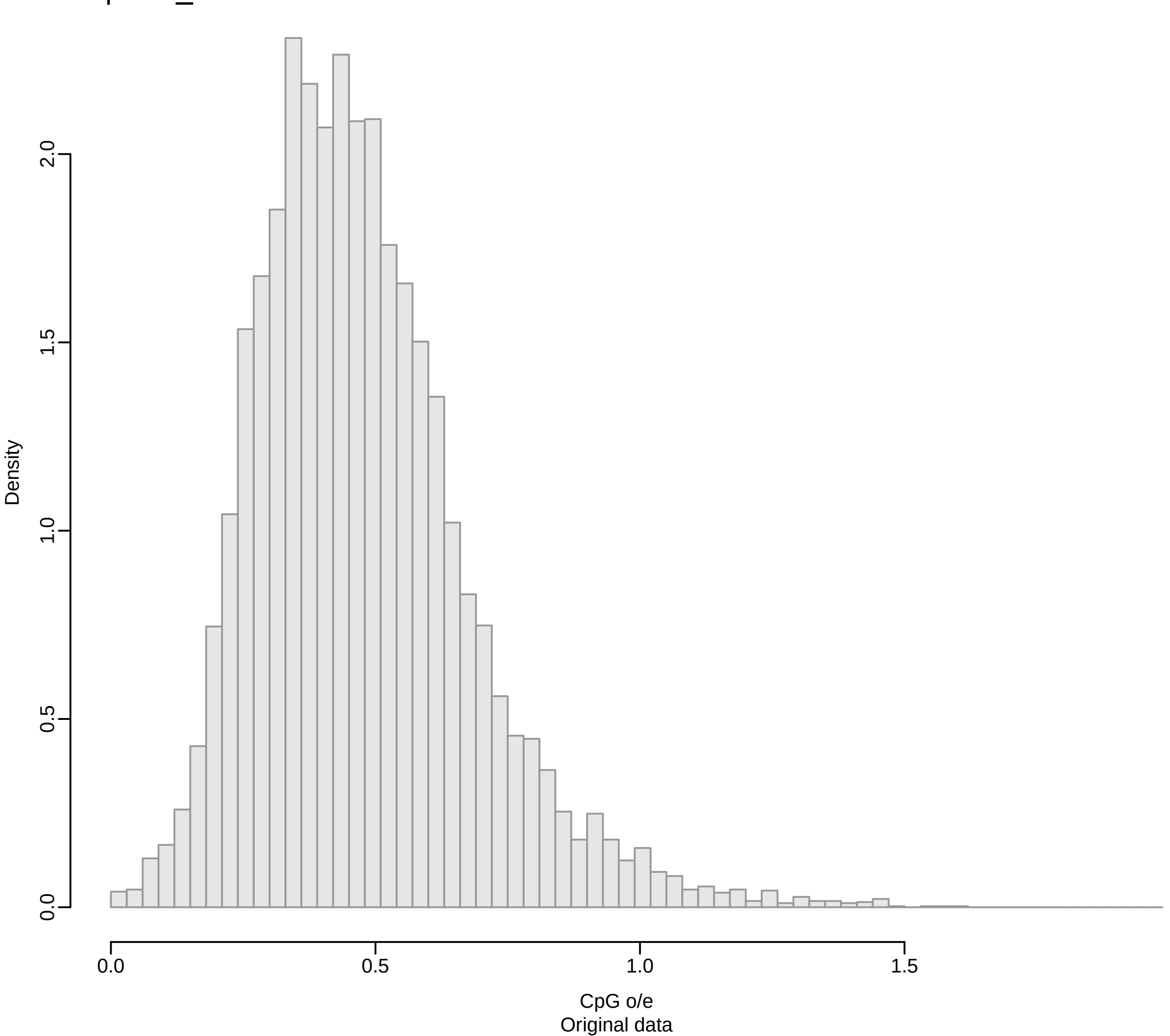

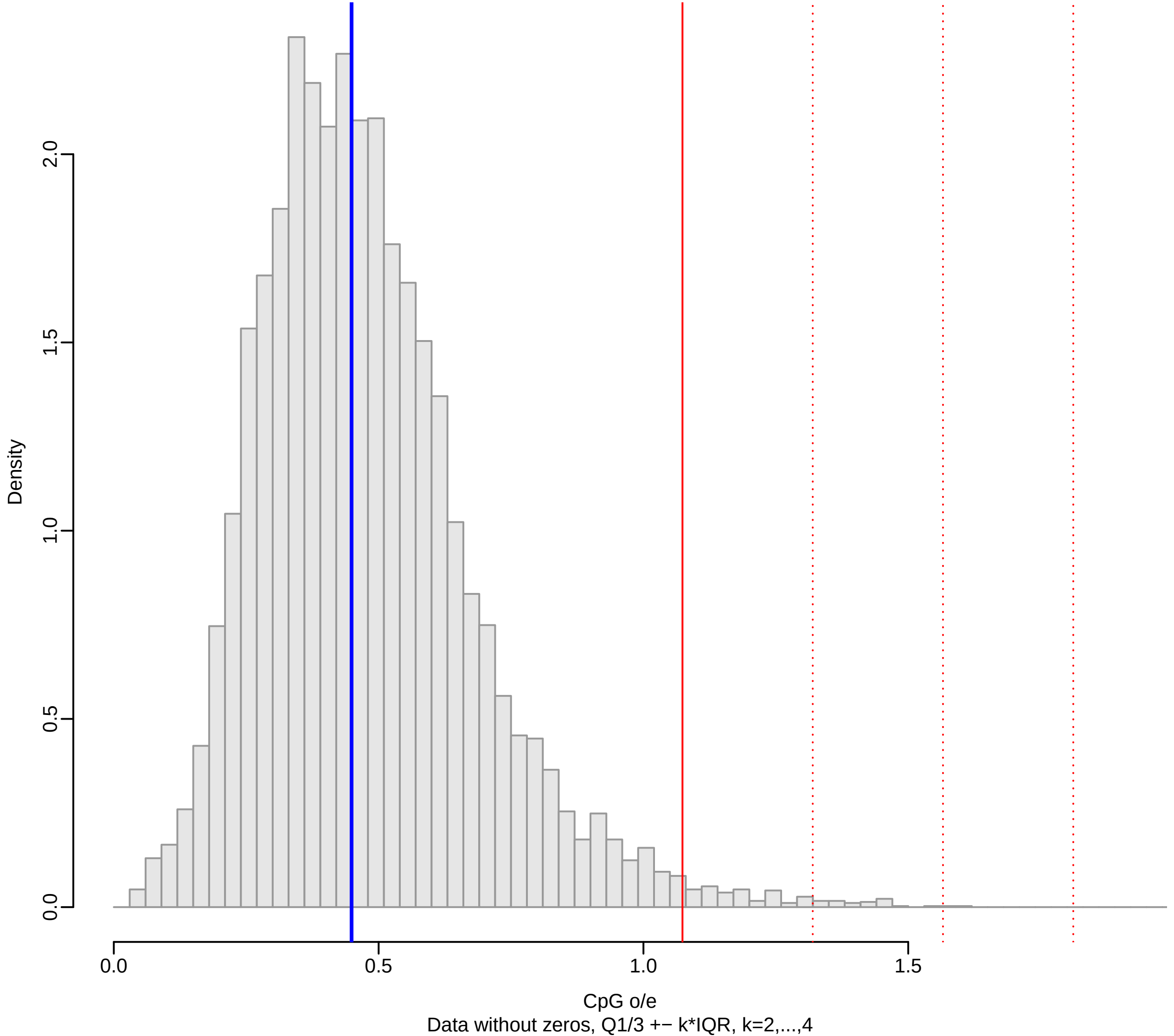

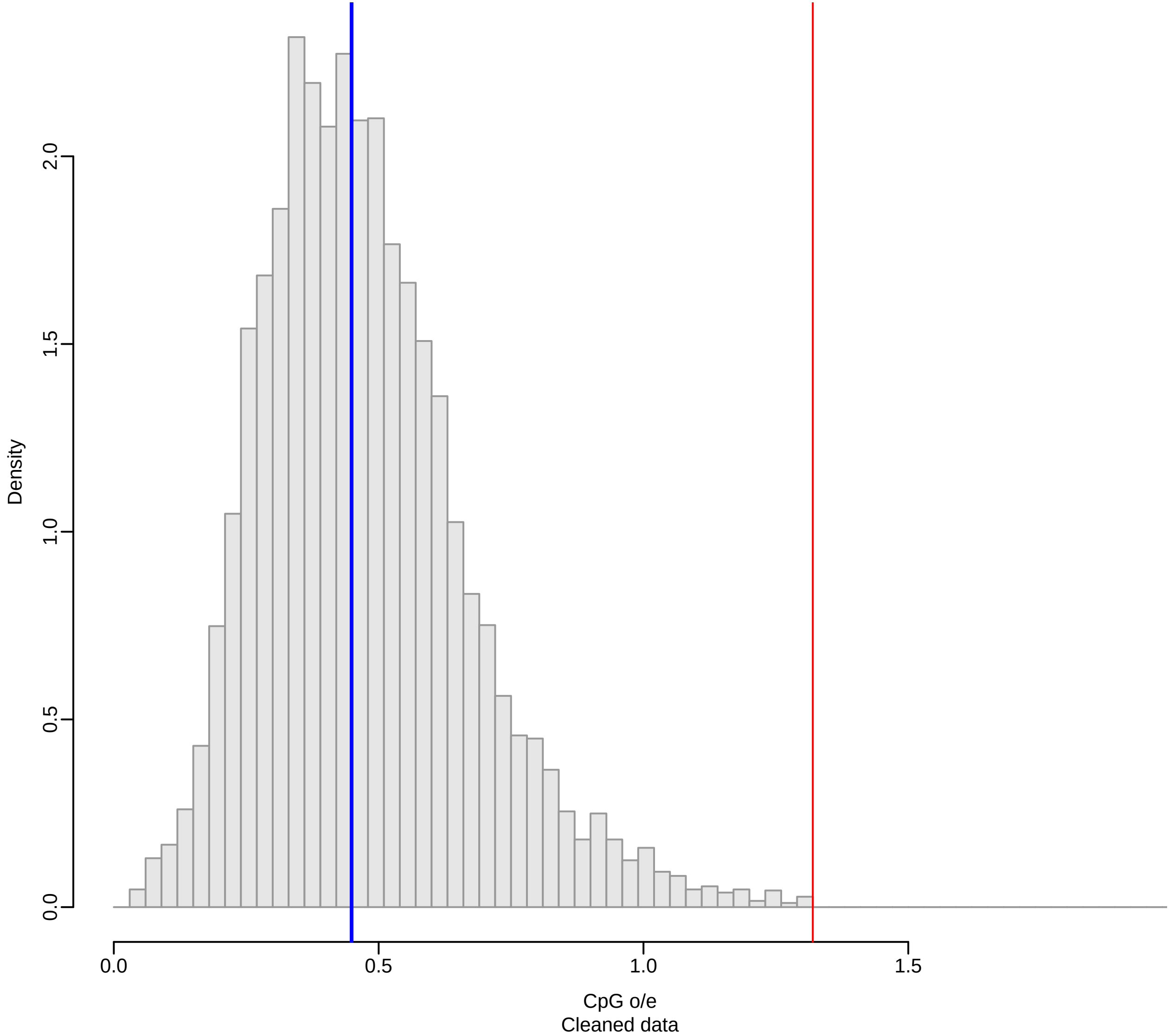

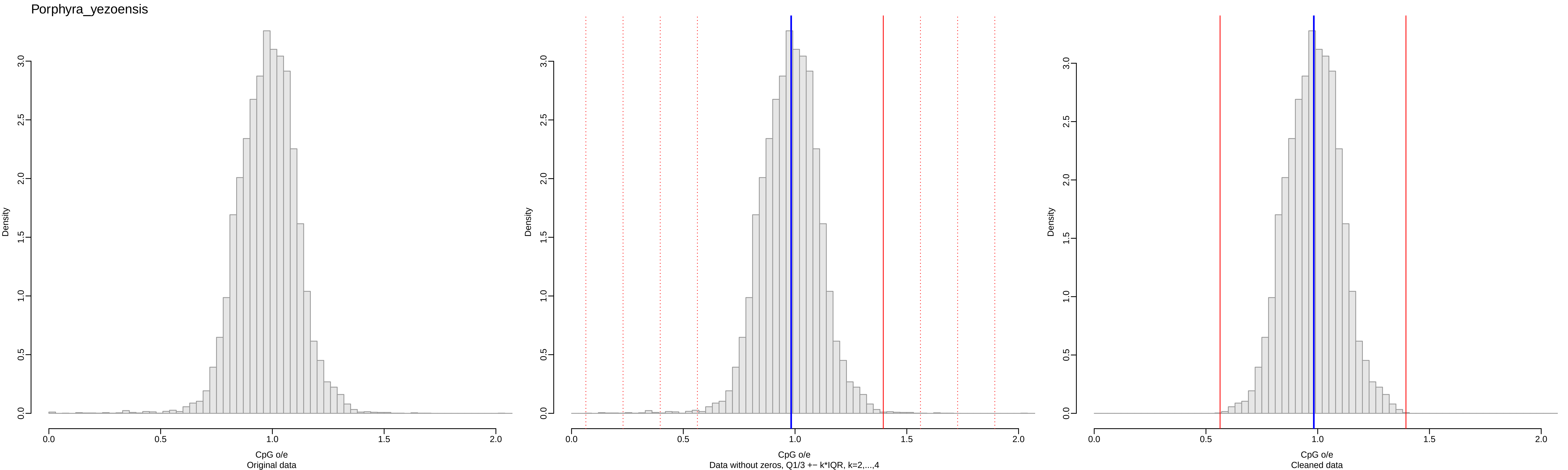

Porphyra_yezoensis
Density
CpG o/e
Original data
Data without zeros, Q1/3 +− k*IQR, k=2,...,4
Cleaned data

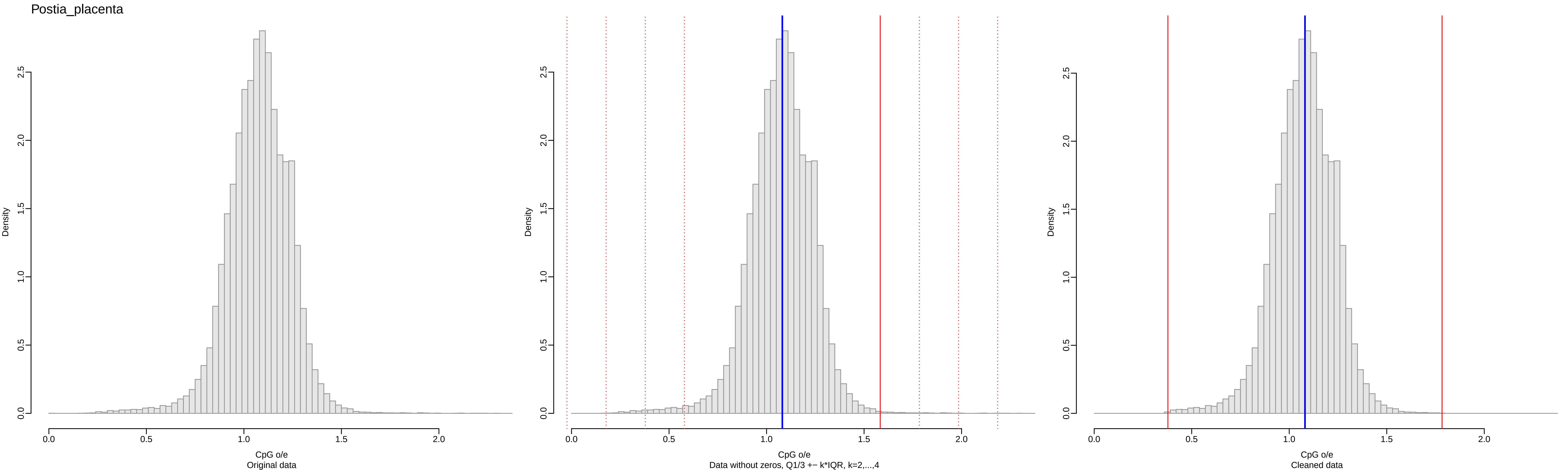

Postia_placenta
Density
CpG o/e
Original data
Data without zeros, Q1/3 +− k*IQR, k=2,...,4
Cleaned data
0.0
0.5
1.0
1.5
2.0
2.5

Pristionchus_pacificus

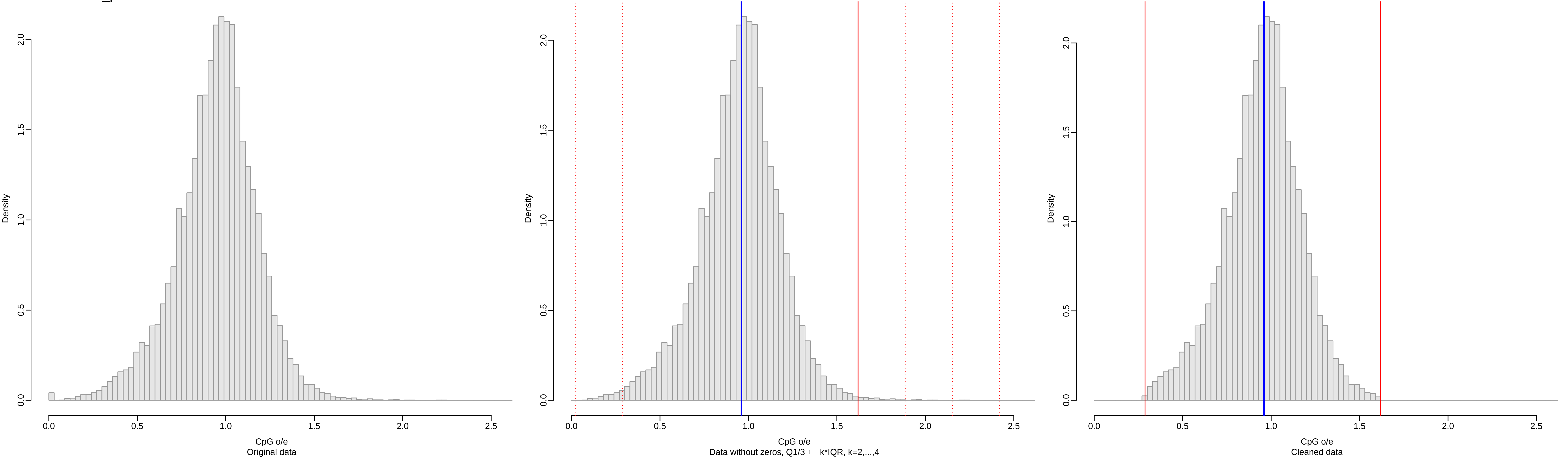

Prunus_armeniaca

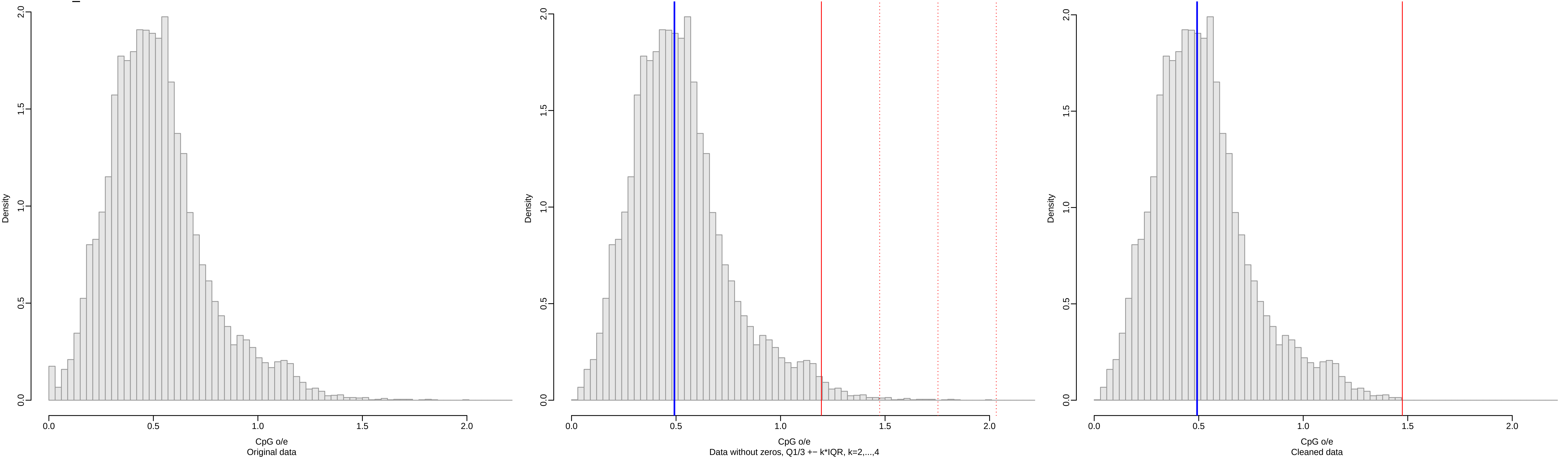

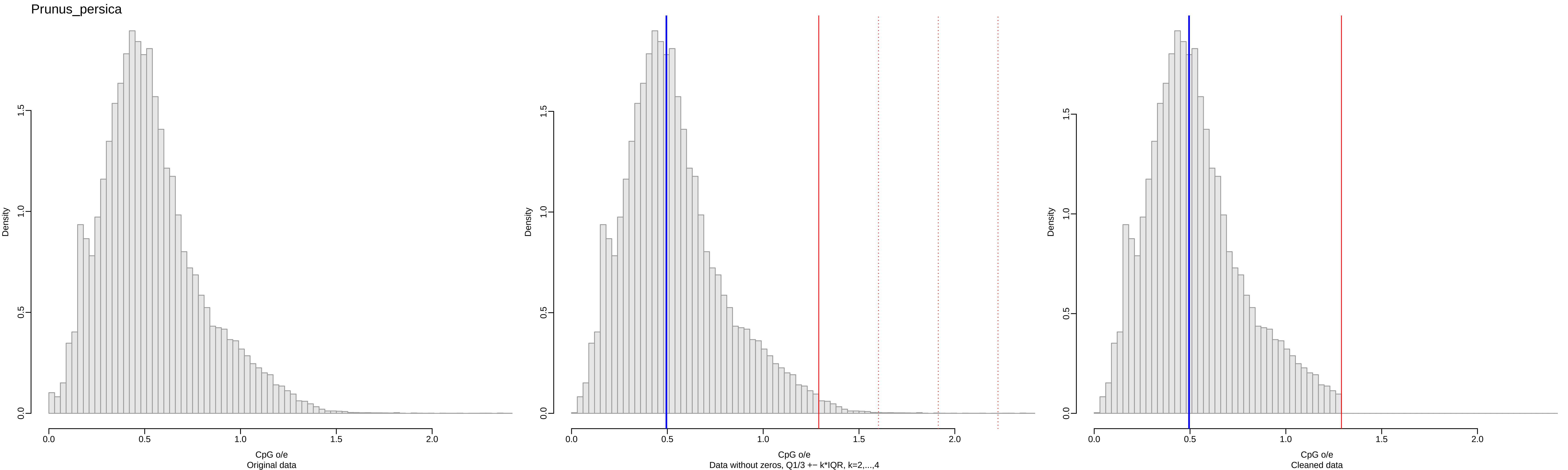

Prunus_persica
Density
0.0
0.5
1.0
1.5
2.0
CpG o/e
Original data
Data without zeros, Q1/3 +− k*IQR, k=2,...,4
Cleaned data

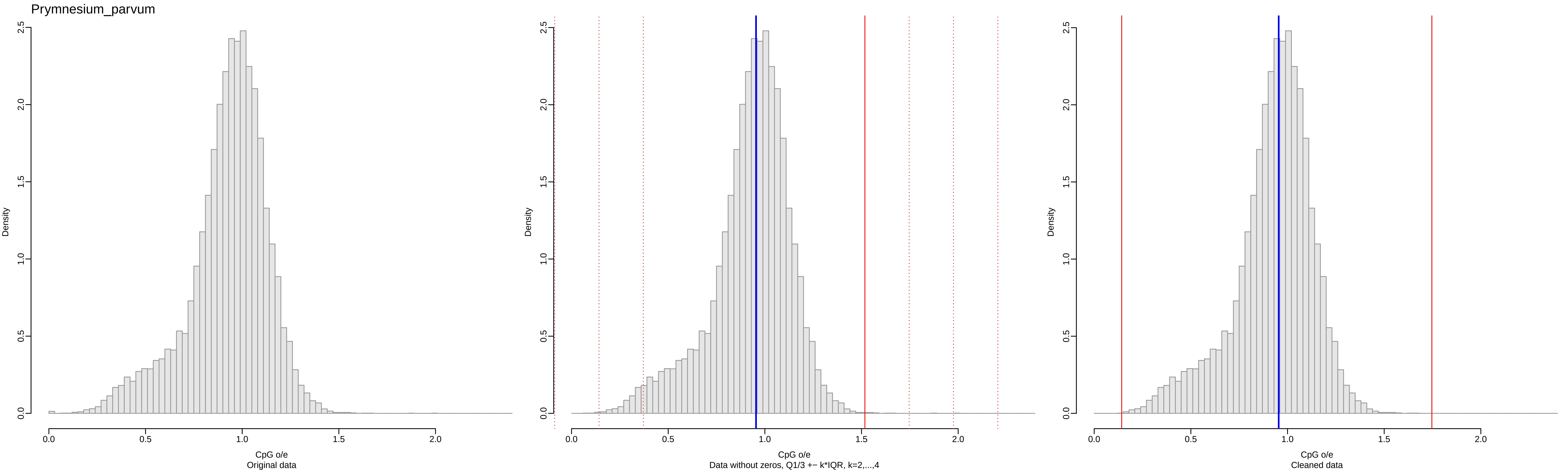
Prymnesium_parvum
Density
CpG o/e
Original data
Data without zeros, Q1/3 +− k*IQR, k=2,...,4
Cleaned data
0.0
0.5
1.0
1.5
2.0
2.5

Psetta_maxima

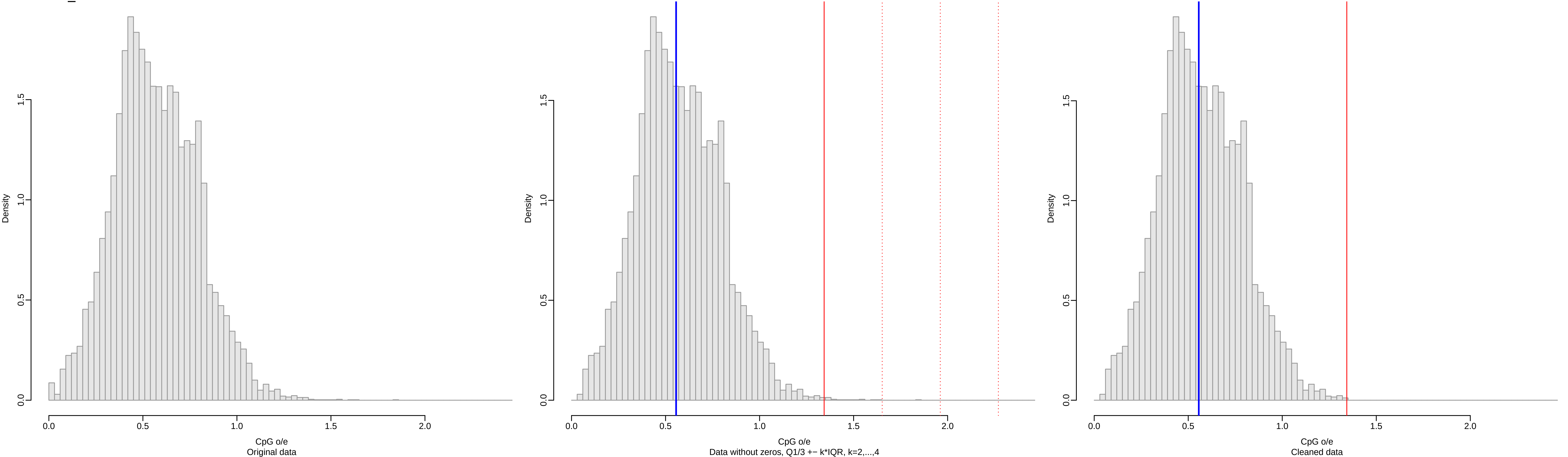

Pteridium_aquilinum

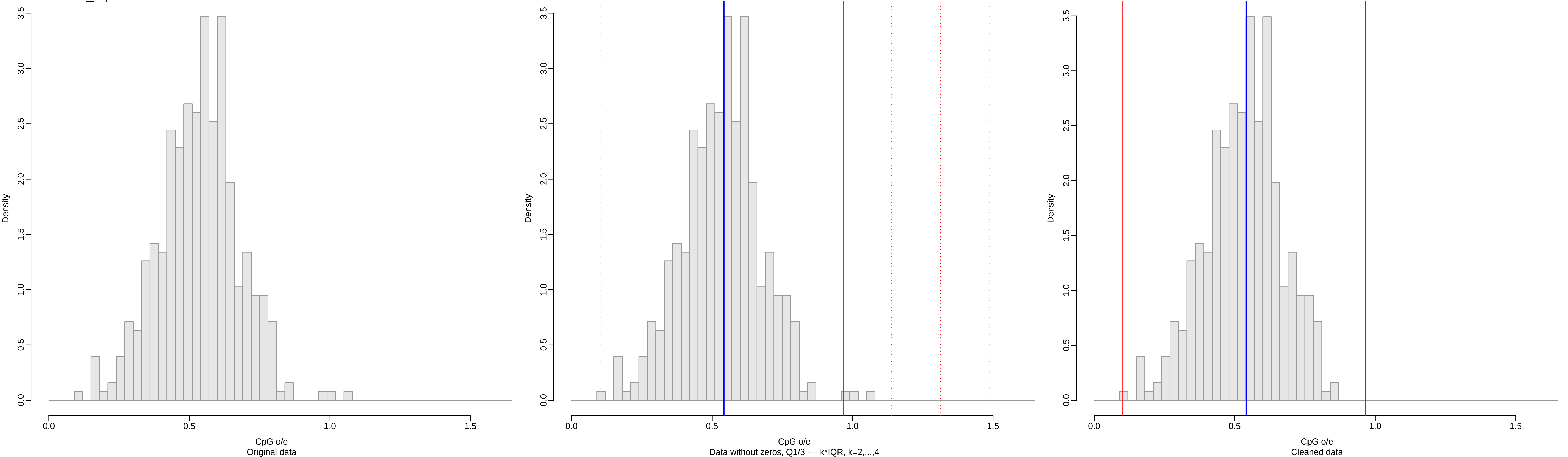

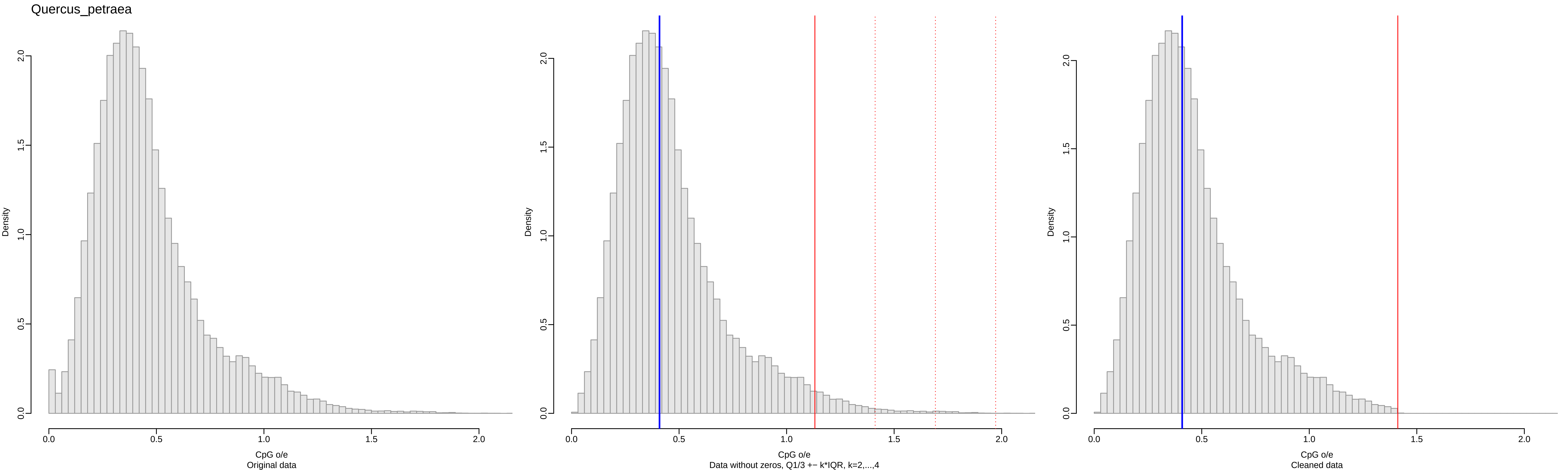

Quercus_petraea
Density
CpG o/e
Original data
Data without zeros, Q1/3 +− k*IQR, k=2,...,4
Cleaned data
0.0
0.5
1.0
1.5
2.0

Quercus_robur

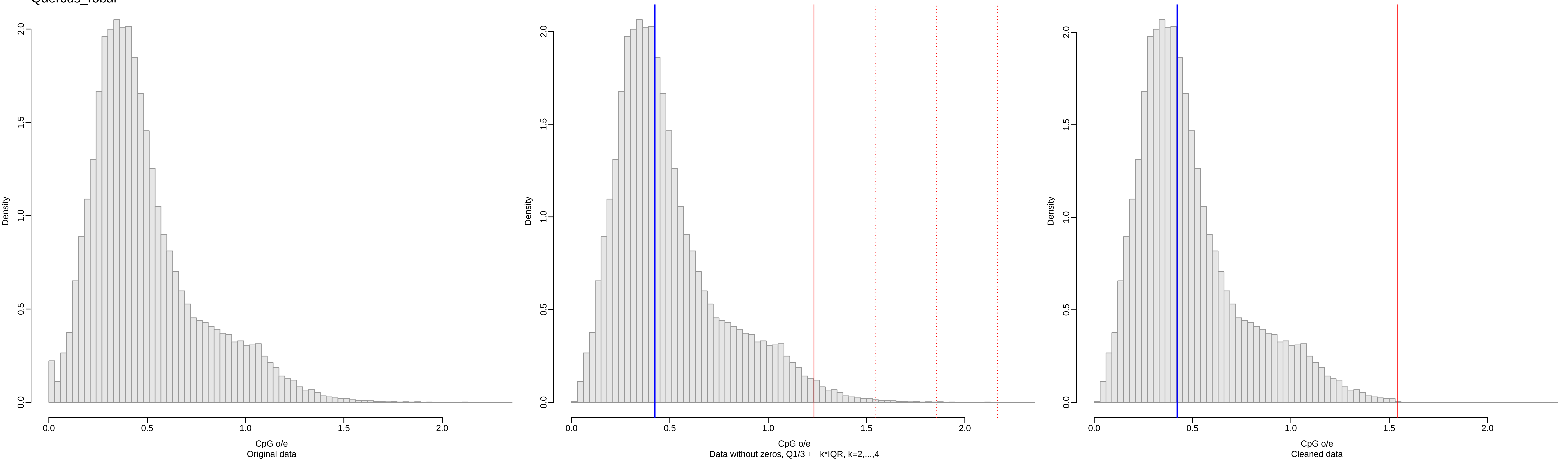

Quercus_suber

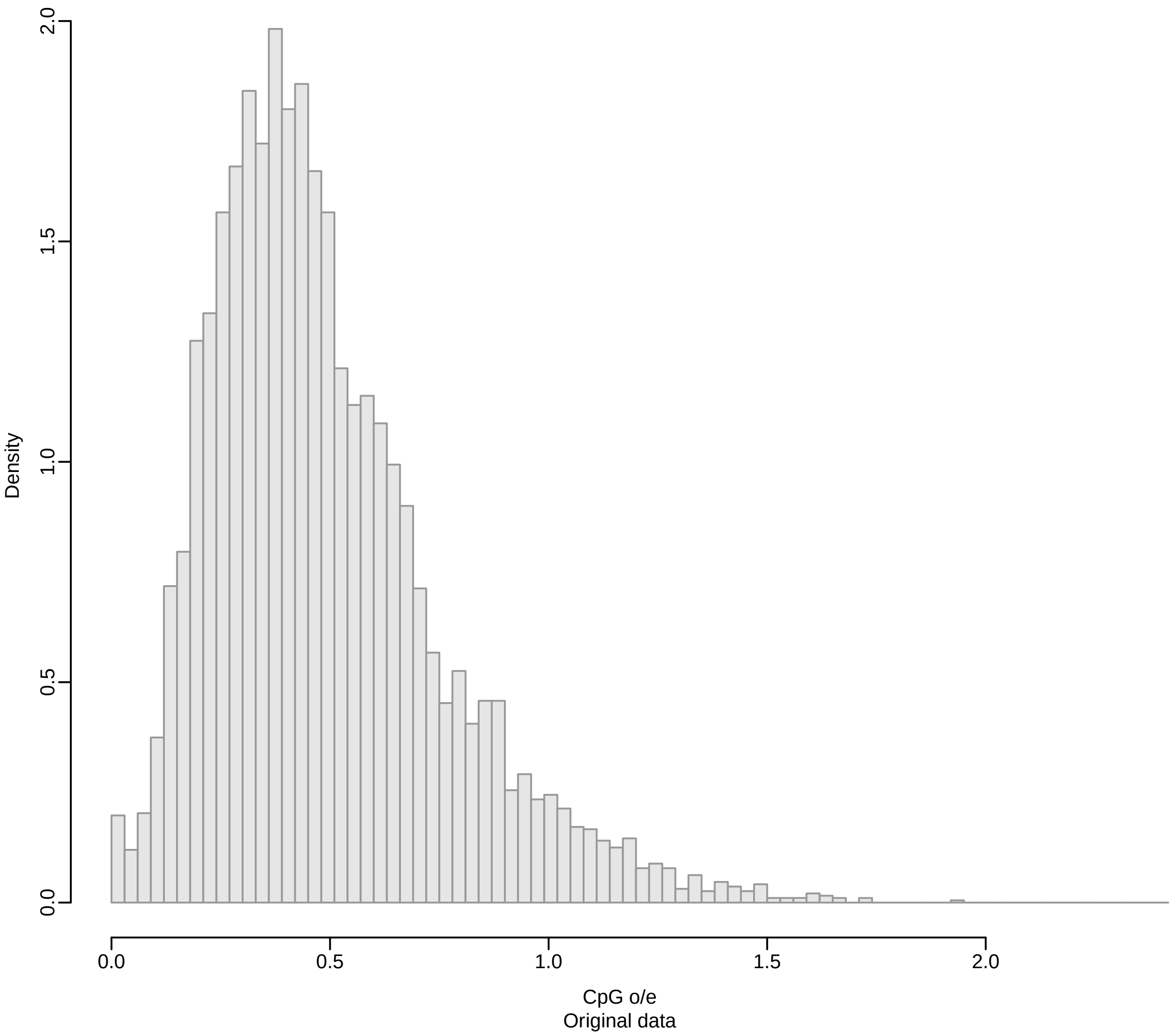

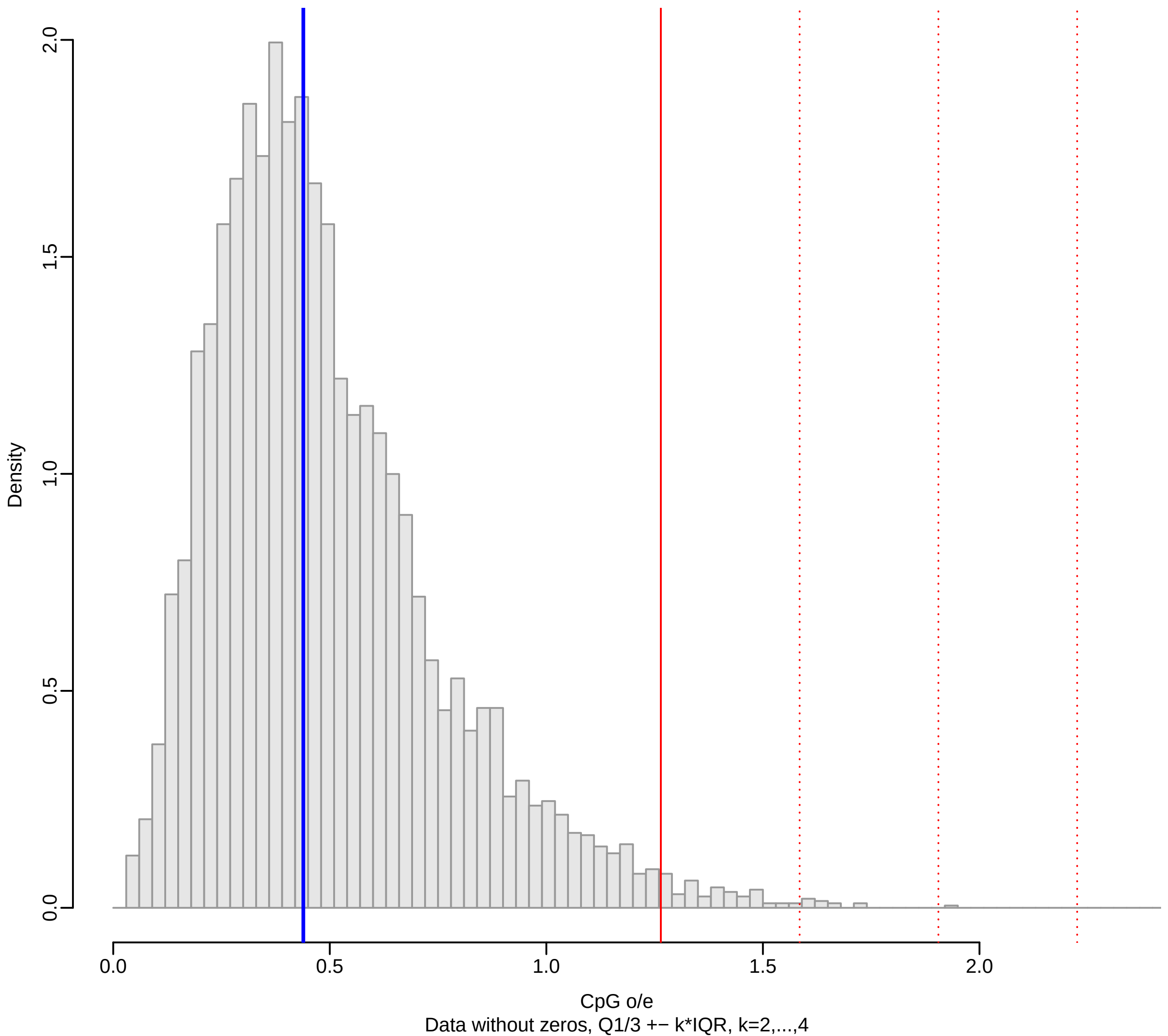

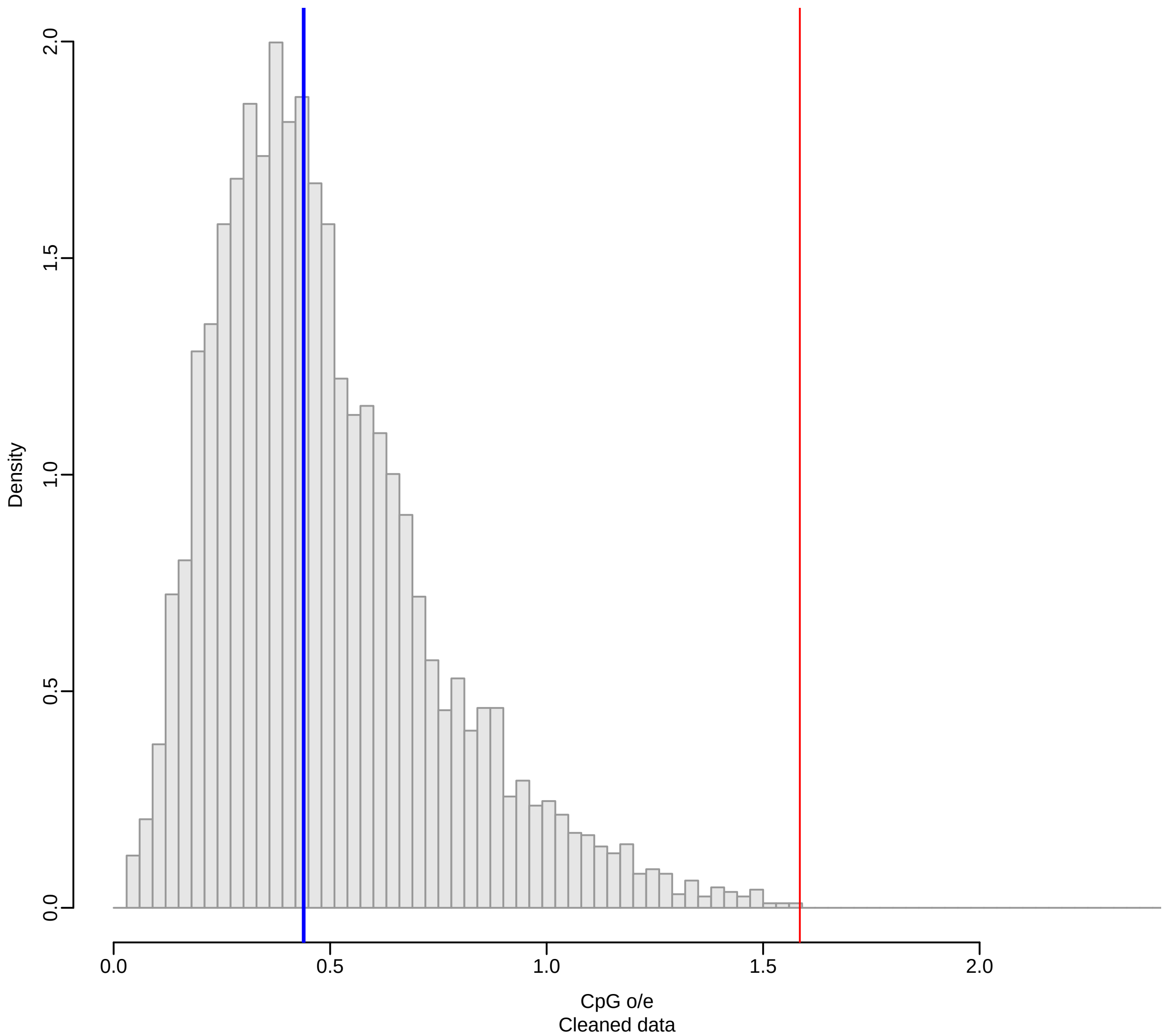

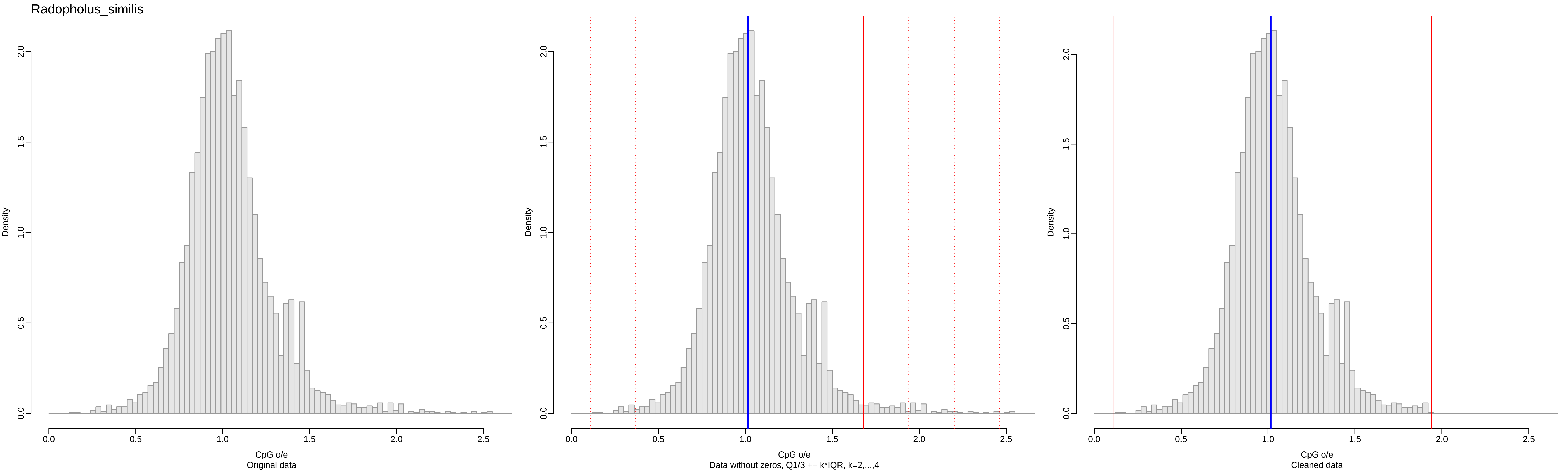
Radopholus_similis
Density
CpG o/e
Original data
Density
CpG o/e
Data without zeros, Q1/3 +− k*IQR, k=2,...,4
Density
CpG o/e
Cleaned data

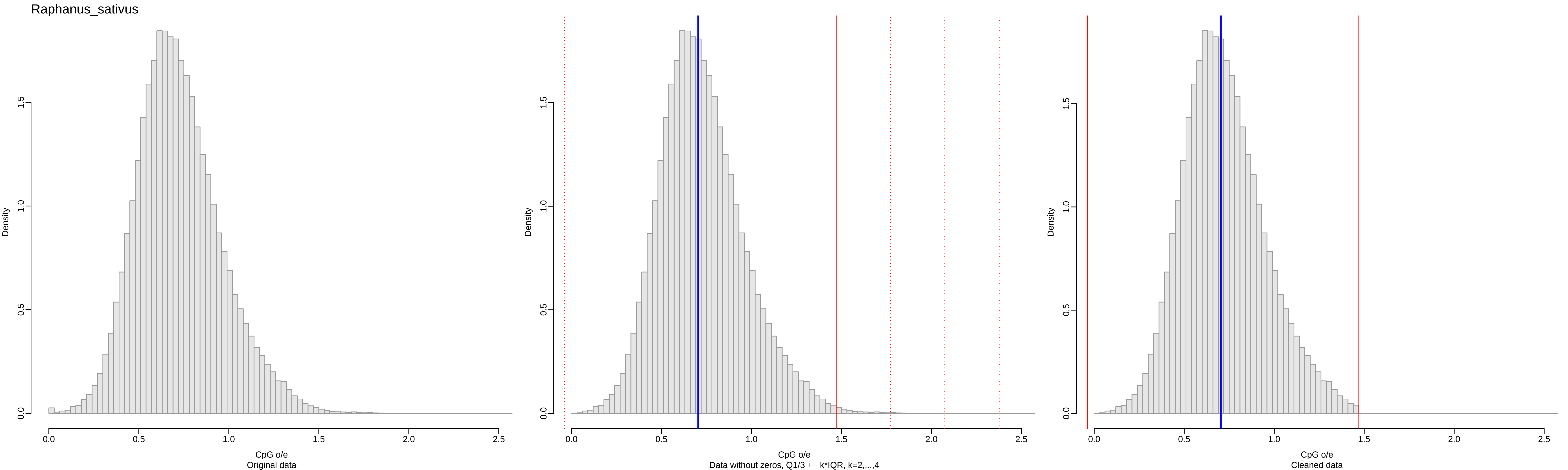

Raphanus_sativus
Density
0.0
0.5
1.0
1.5
0.0
0.5
1.0
1.5
2.0
2.5
CpG o/e
Original data
Data without zeros, Q1/3 +− k*IQR, k=2,...,4
Cleaned data

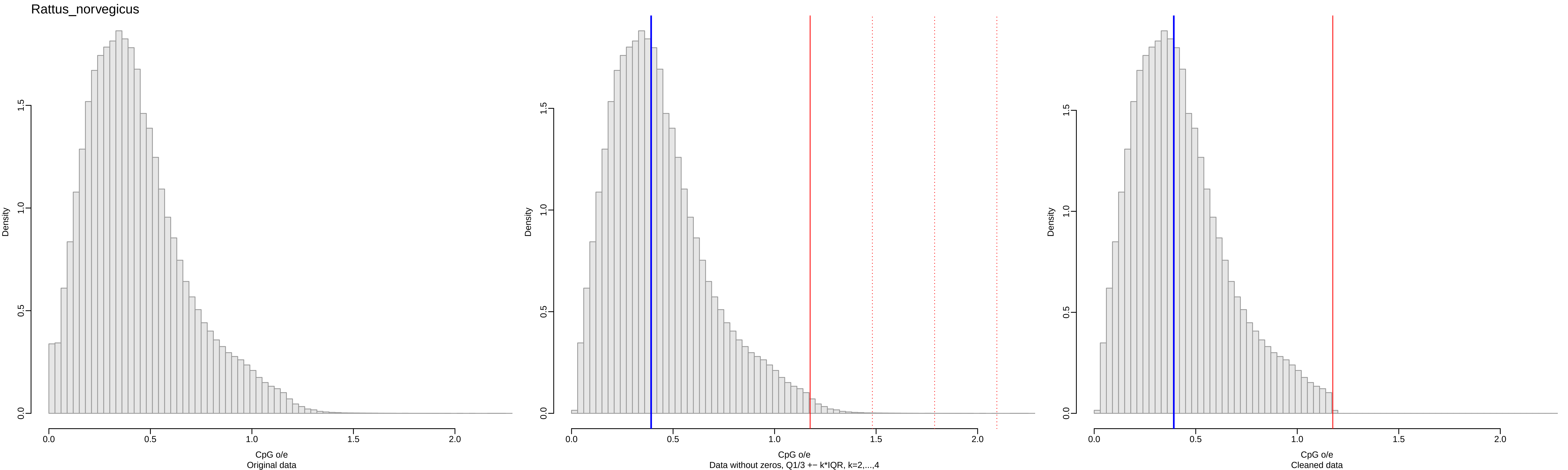

Rattus_norvegicus
Density
CpG o/e
Original data
Data without zeros, Q1/3 +− k*IQR, k=2,...,4
Cleaned data
0.0
0.5
1.0
1.5
2.0

Reclinomonas_americana

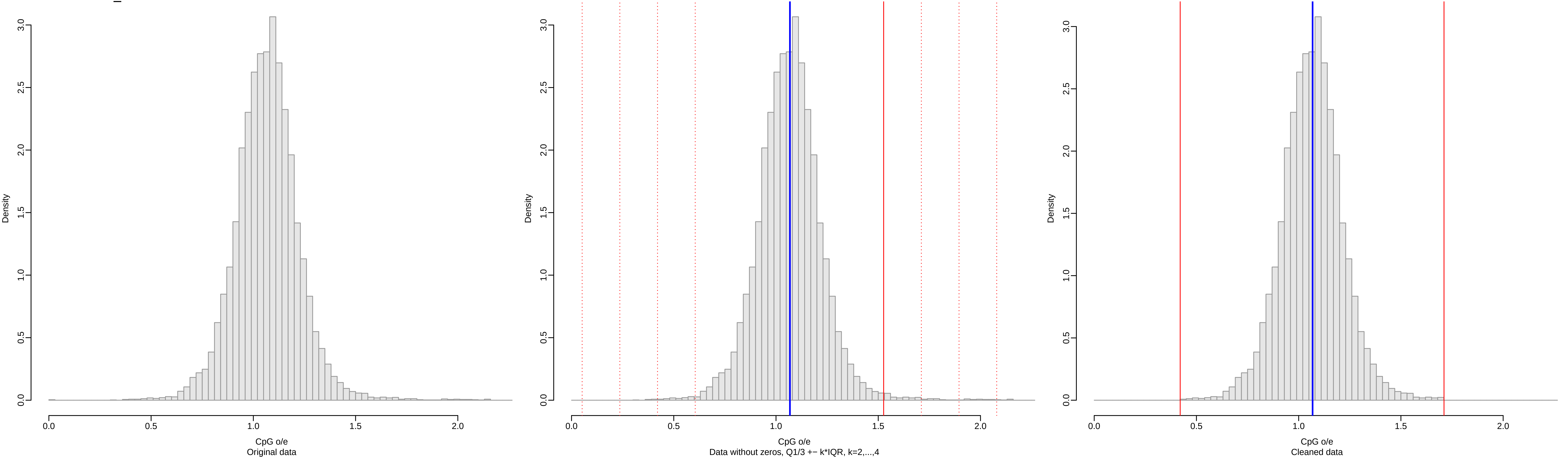

Reticulitermes_flavipes

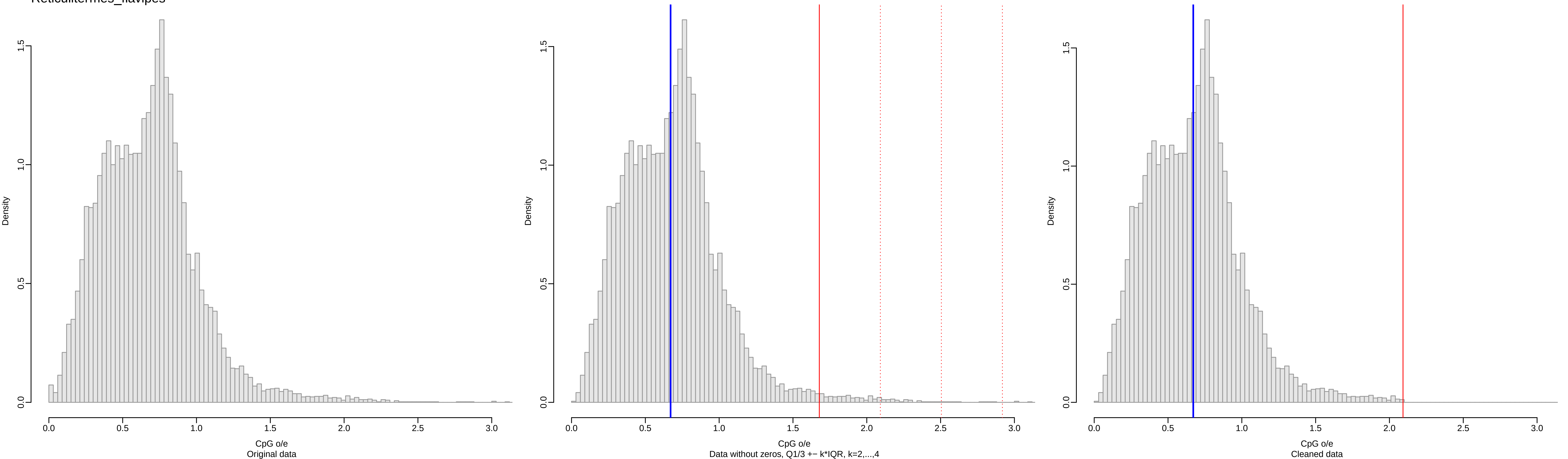

Rhipicephalus_appendiculatus

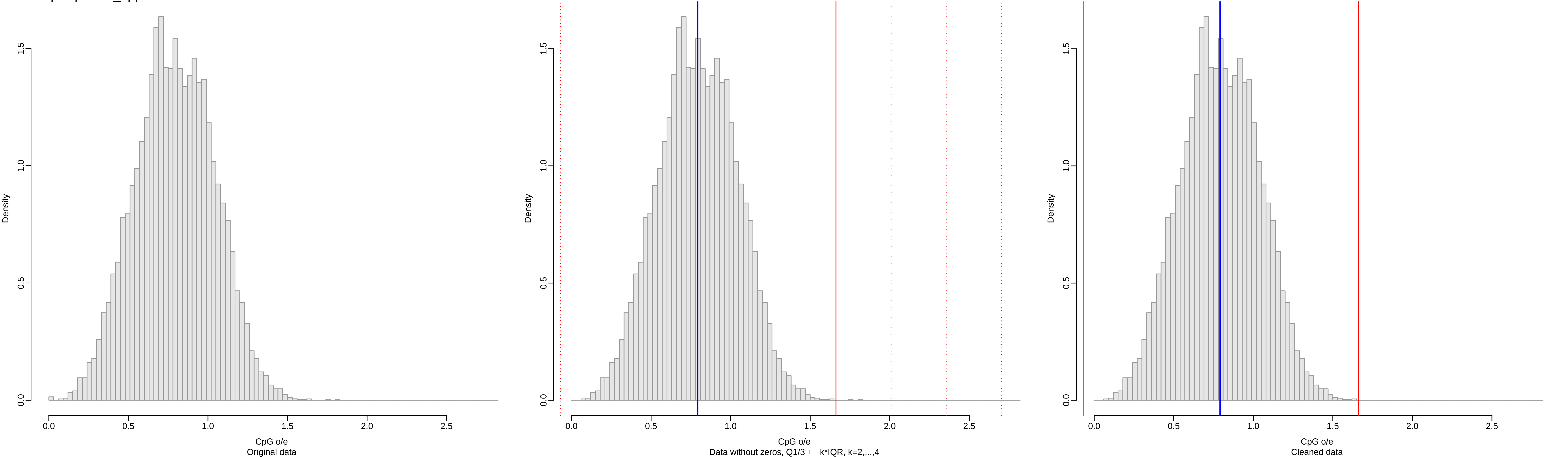

Rhipicephalus_microplus

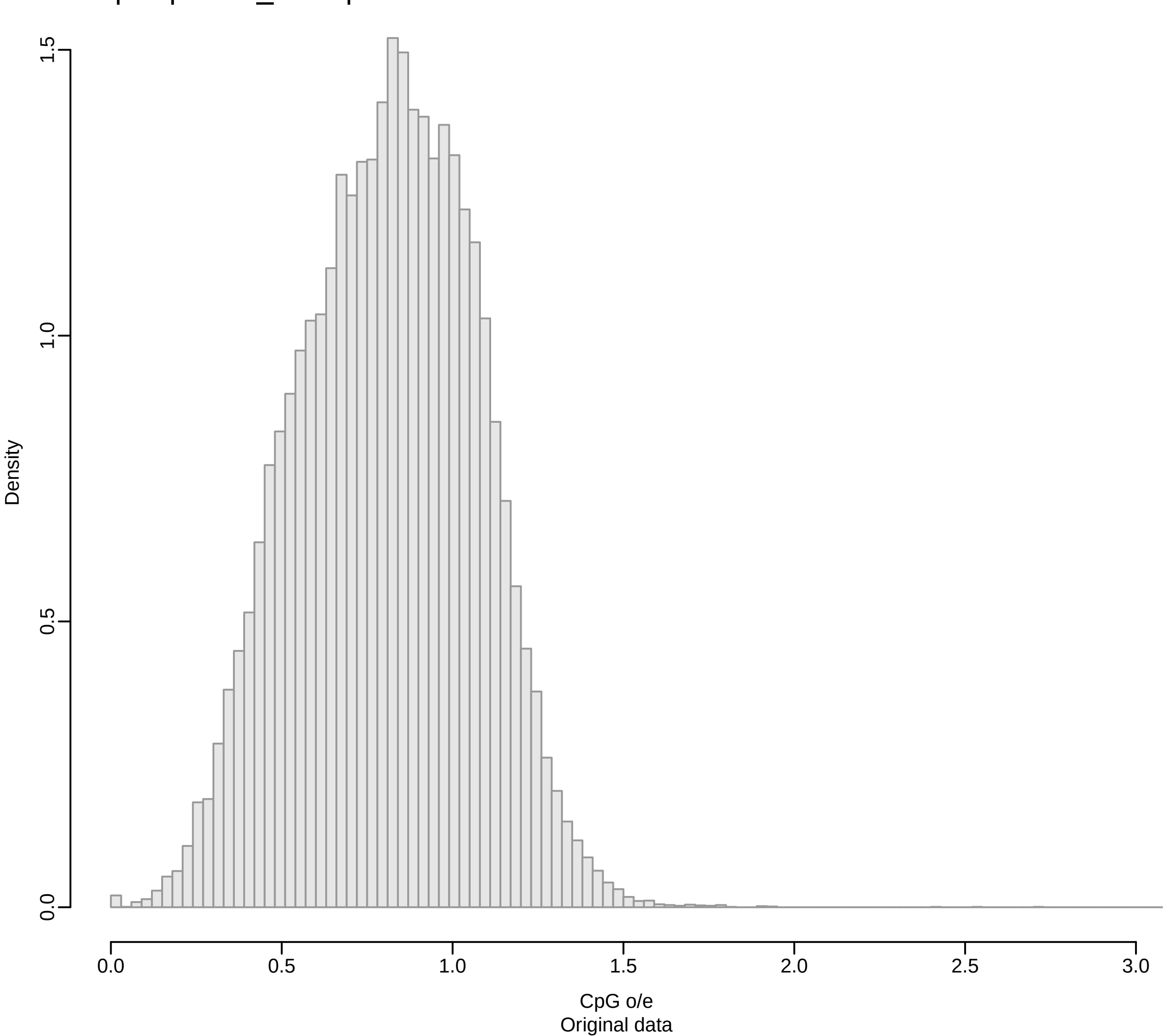

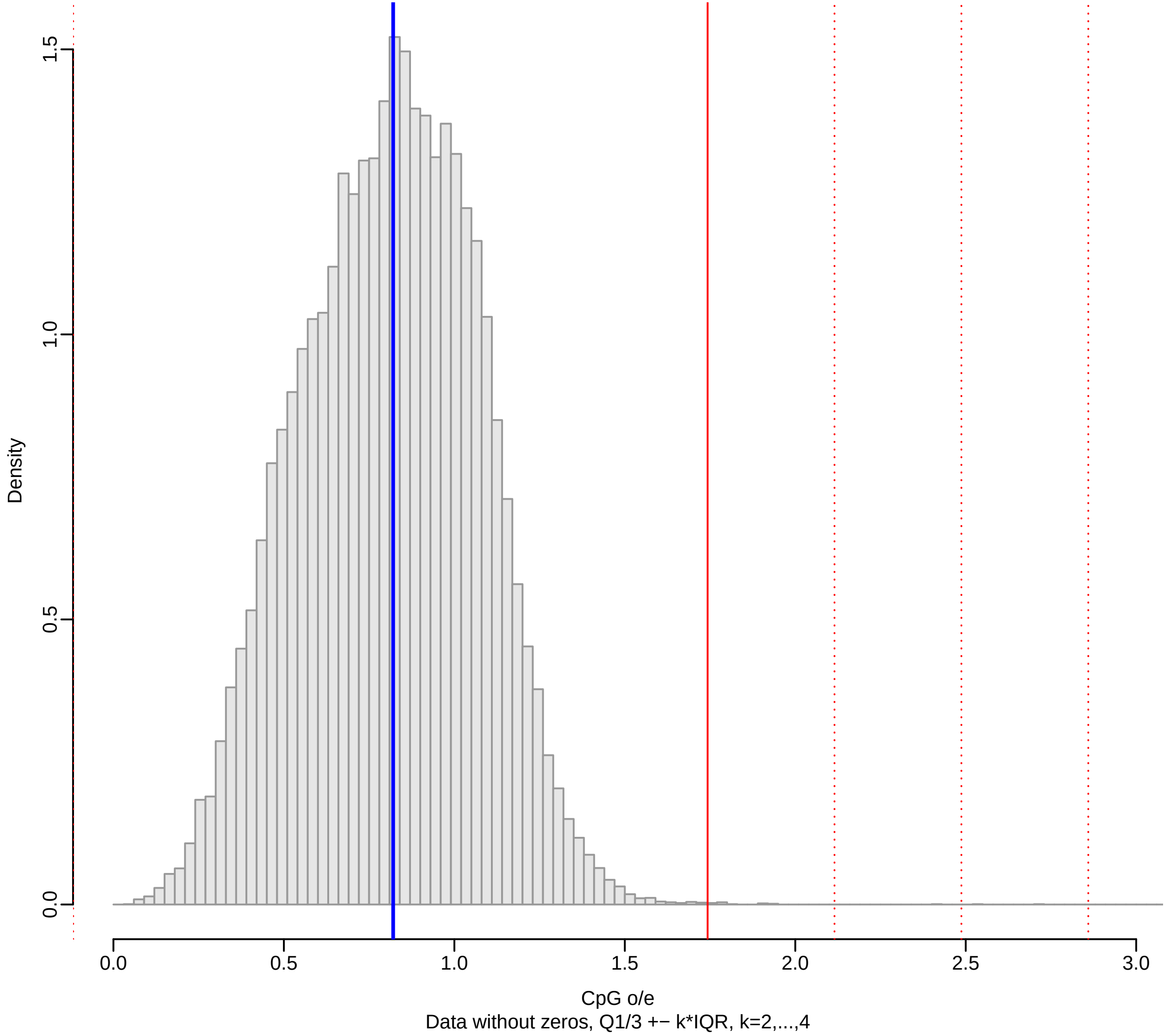

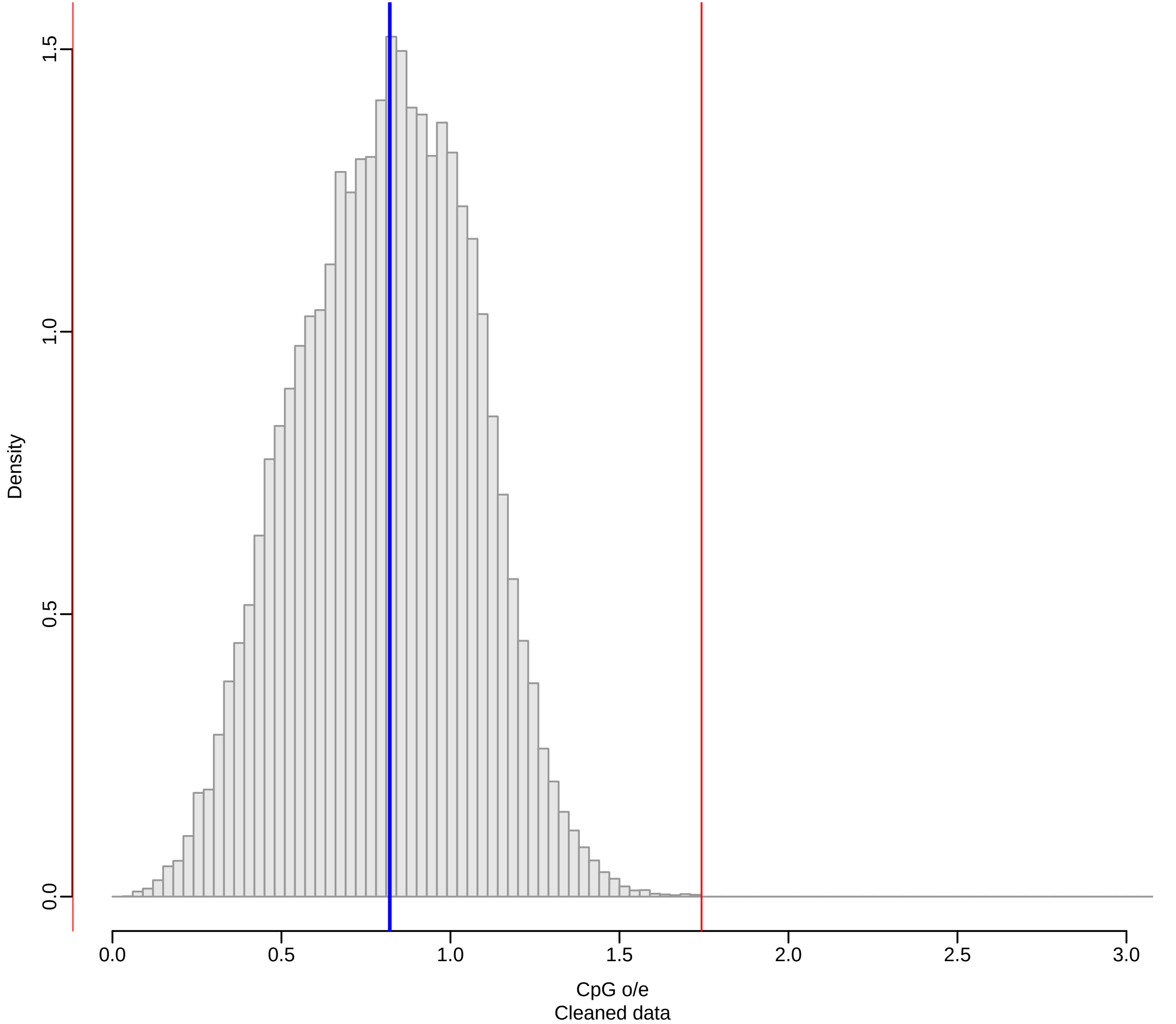

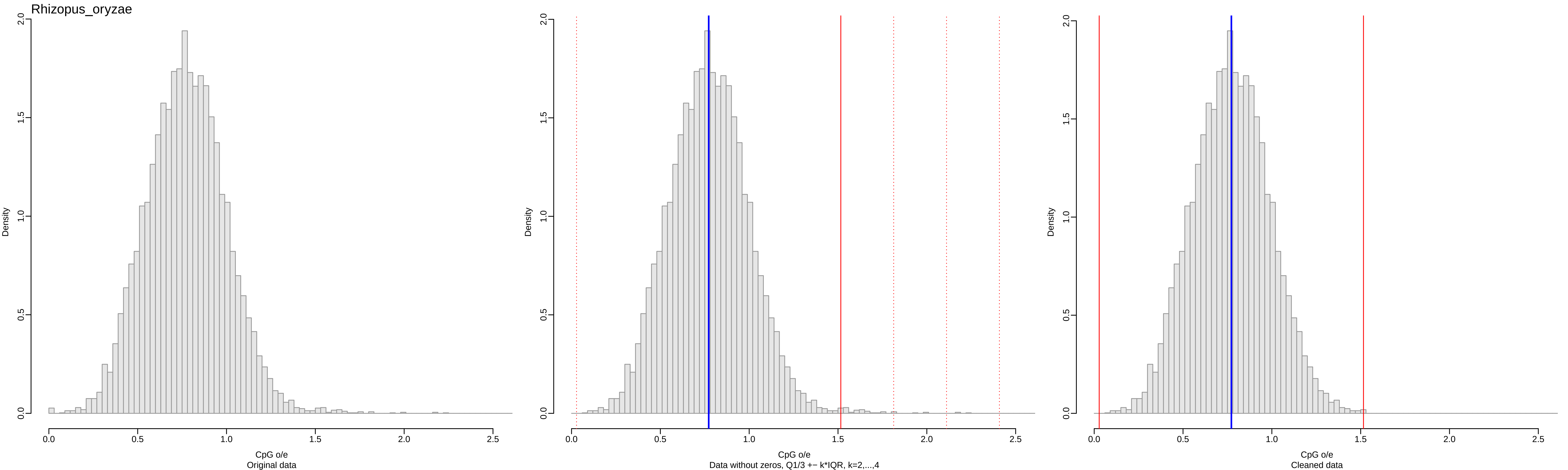
Rhizopus_oryzae
Density
CpG o/e
Original data
Data without zeros, Q1/3 +− k*IQR, k=2,...,4
Cleaned data
0.0
0.5
1.0
1.5
2.0
2.5

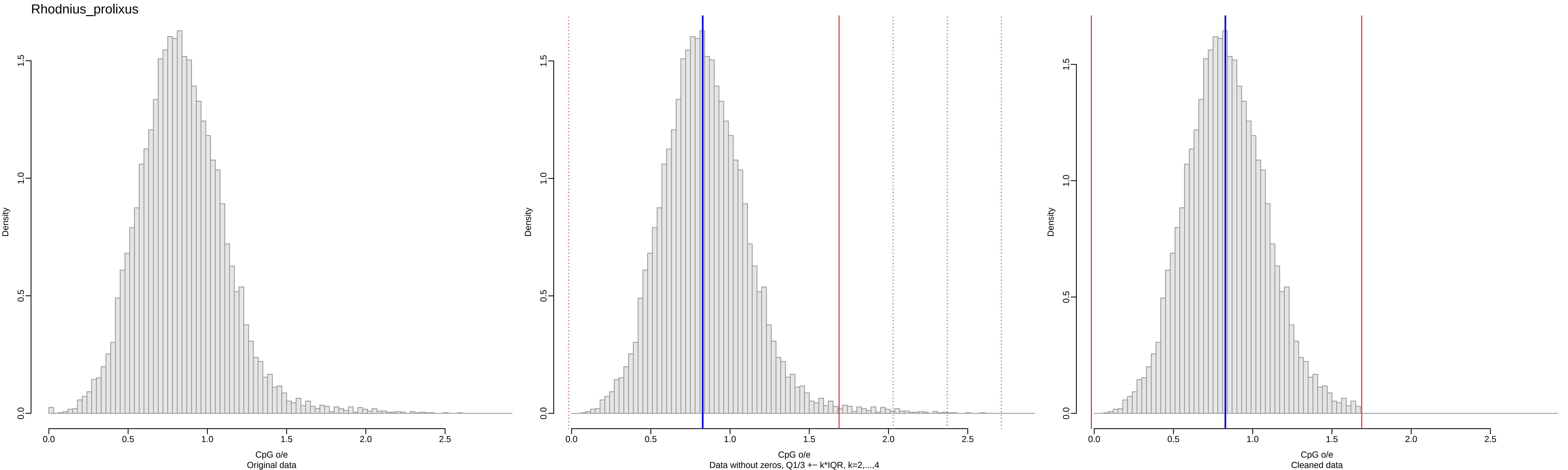

Rhodnius_prolixus
Density
CpG o/e
Original data
Data without zeros, Q1/3 +− k*IQR, k=2,...,4
Cleaned data
0.0
0.5
1.0
1.5
2.0
2.5

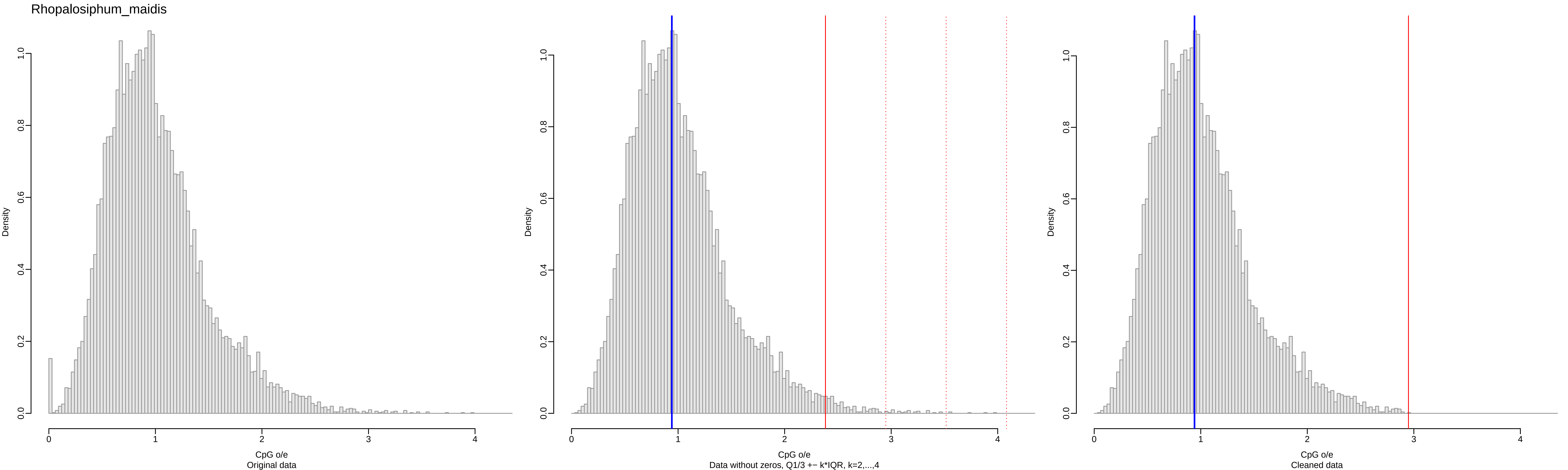
Rhopalosiphum_maidis
Density
CpG o/e
Original data
CpG o/e
Data without zeros, Q1/3 +− k*IQR, k=2,...,4
CpG o/e
Cleaned data

Rhopalosiphum_padi

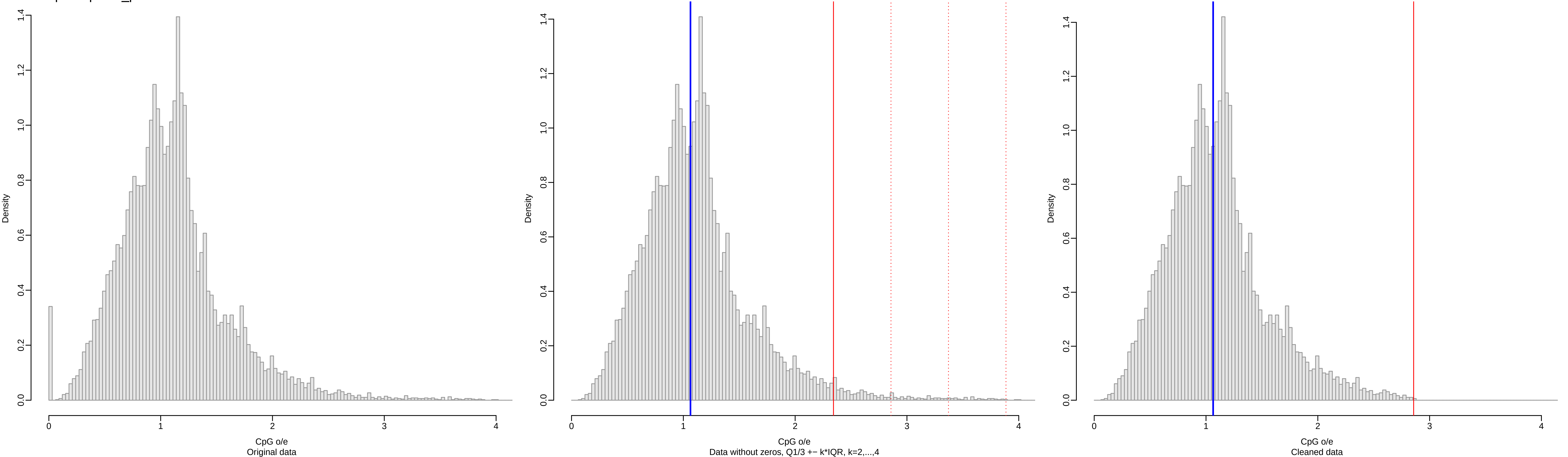

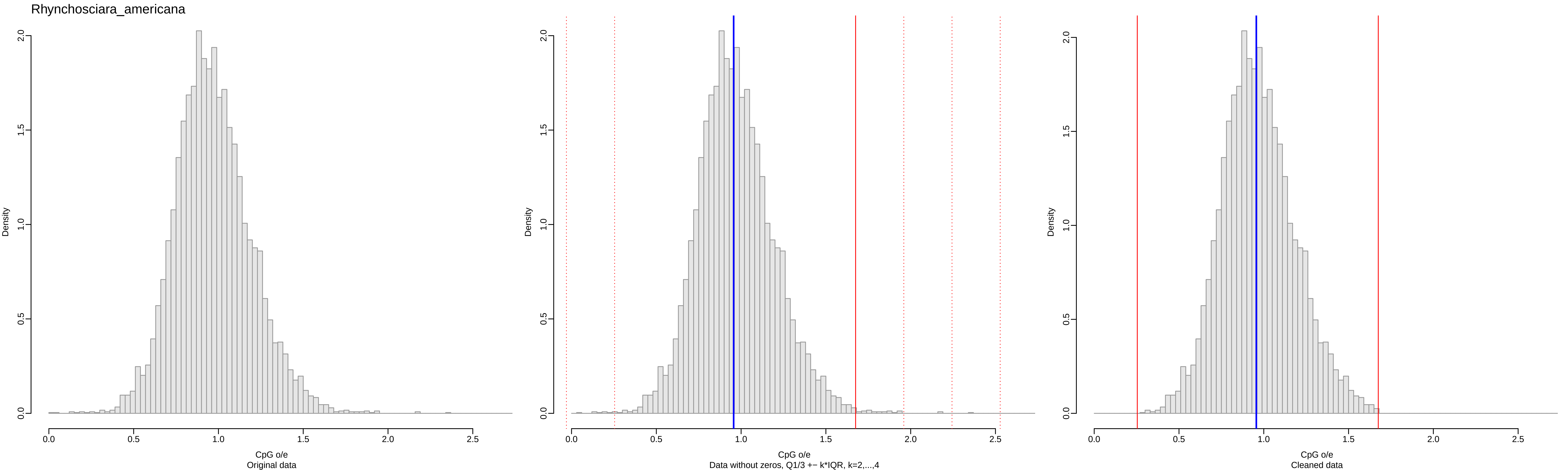

Rhynchosciara_americana
Density
CpG o/e
Original data
Data without zeros, Q1/3 +− k*IQR, k=2,...,4
Cleaned data
0.0
0.5
1.0
1.5
2.0
2.5

Rutilus_rutilus

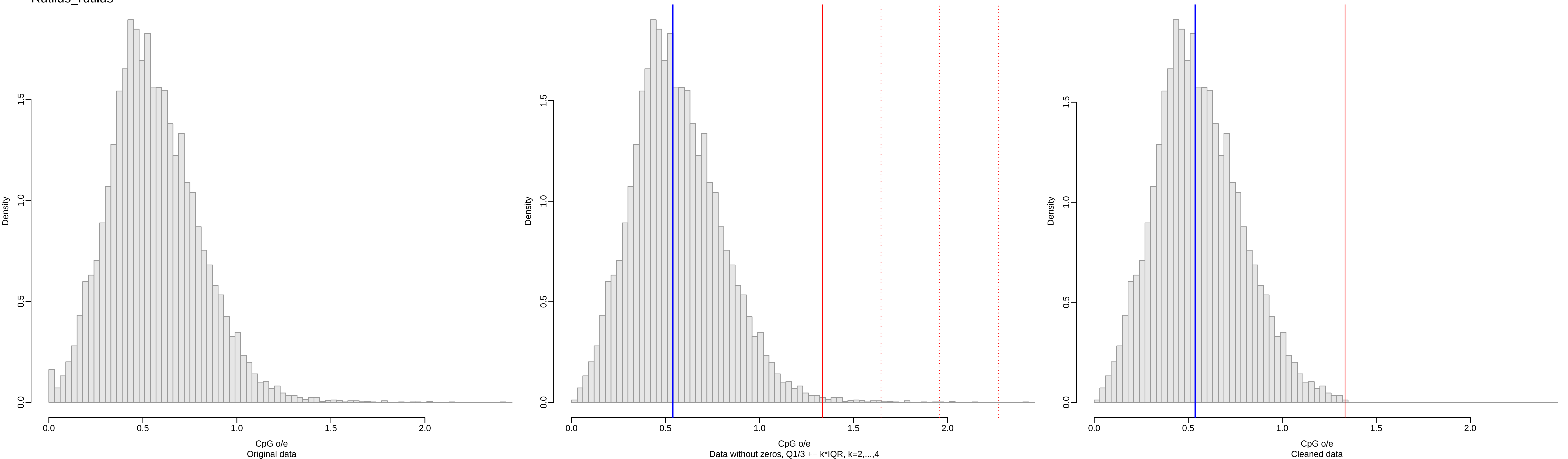

Saccharomyces_cerevisiae

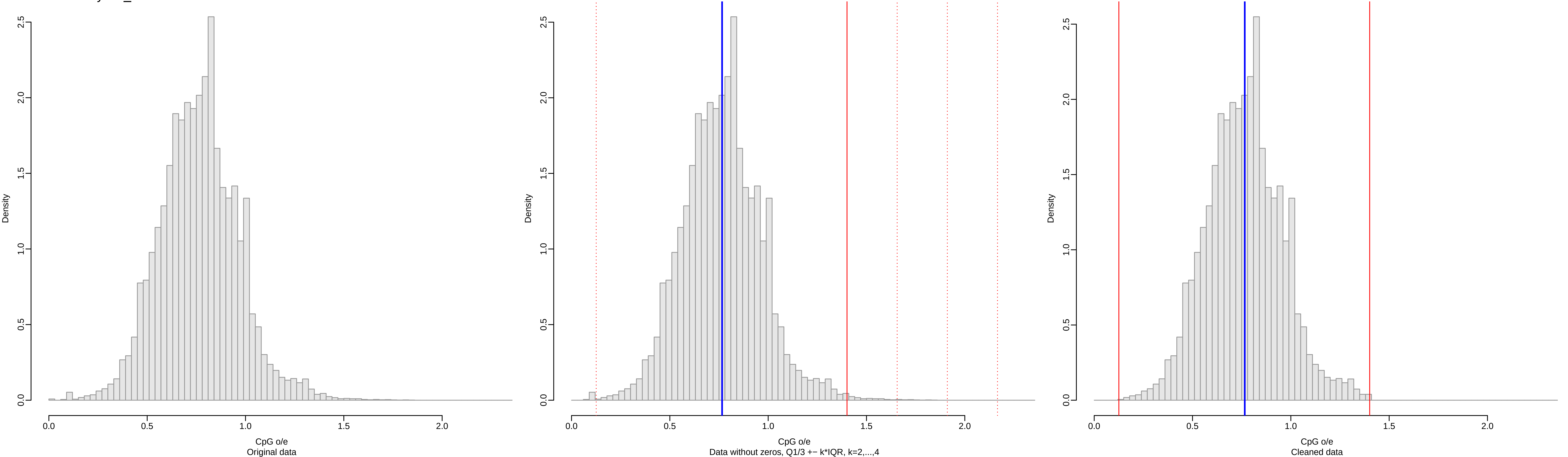

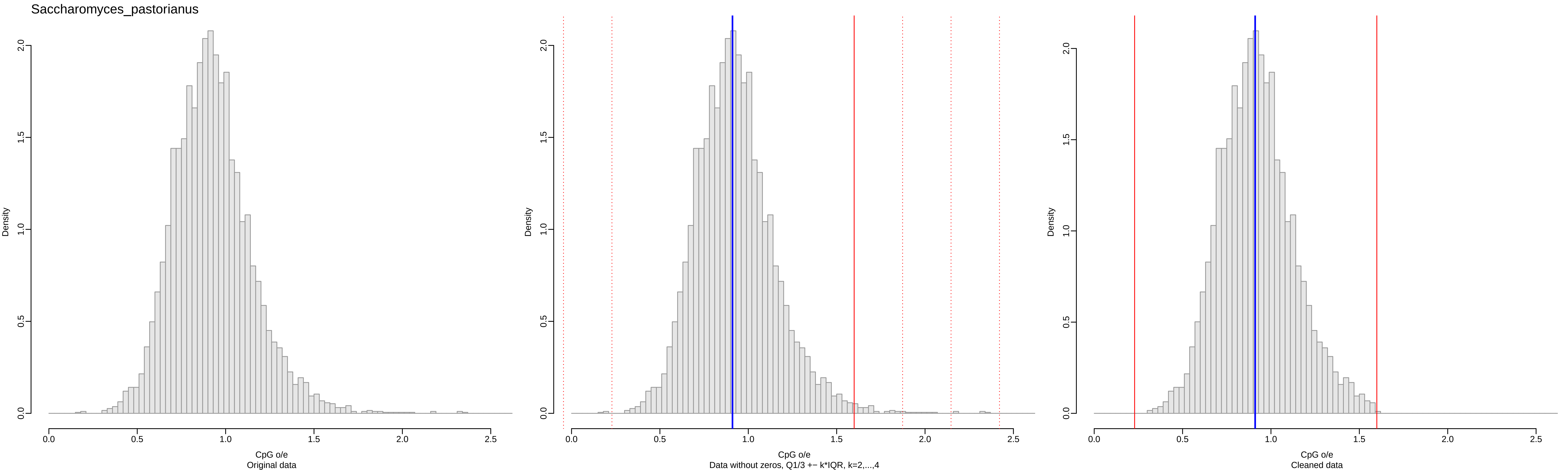

Saccharomyces_pastorianus
Density
CpG o/e
Original data
CpG o/e
Data without zeros, Q1/3 +− k*IQR, k=2,...,4
CpG o/e
Cleaned data

Saccoglossus_kowalevskii

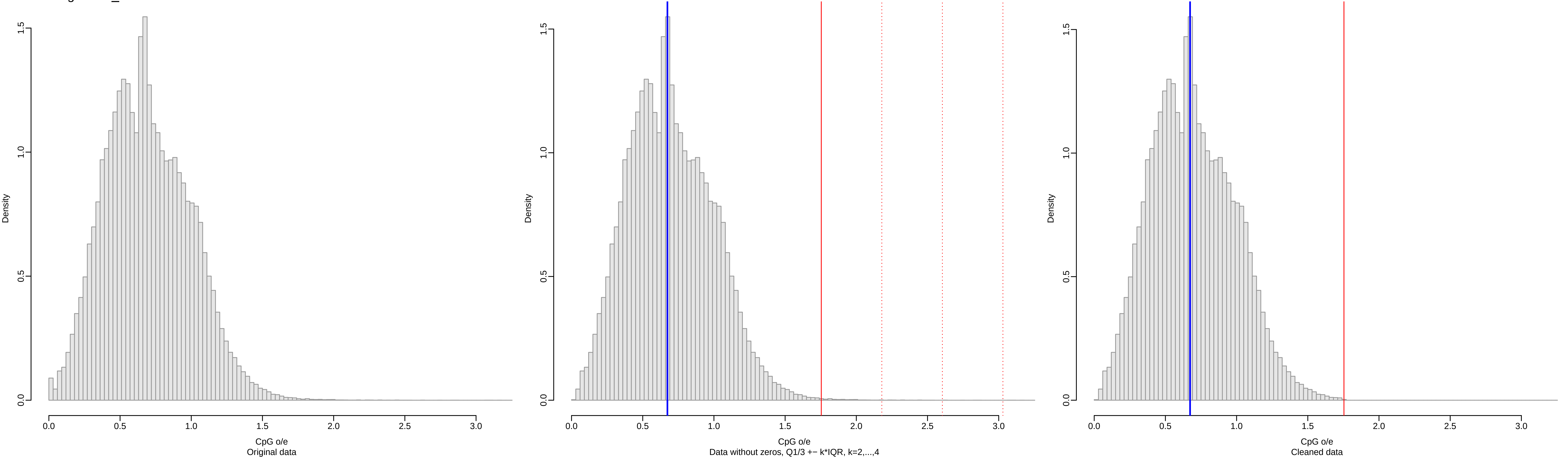

Salmo_salar

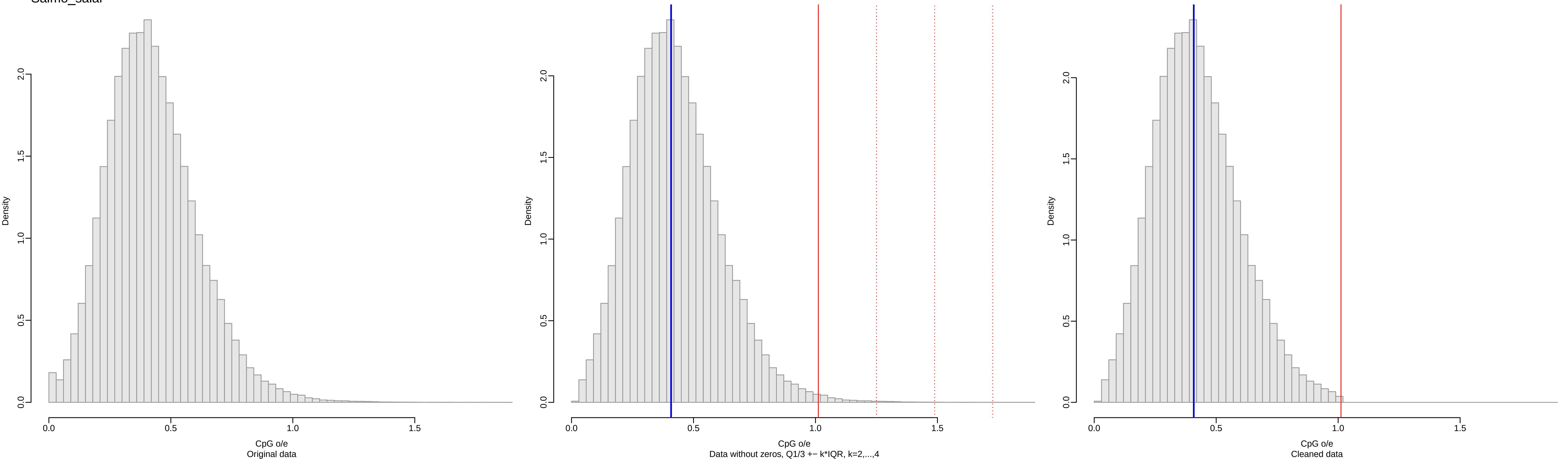

Density
CpG o/e
Original data
Density
CpG o/e
Data without zeros, Q1/3 +− k*IQR, k=2,...,4
Density
CpG o/e
Cleaned data

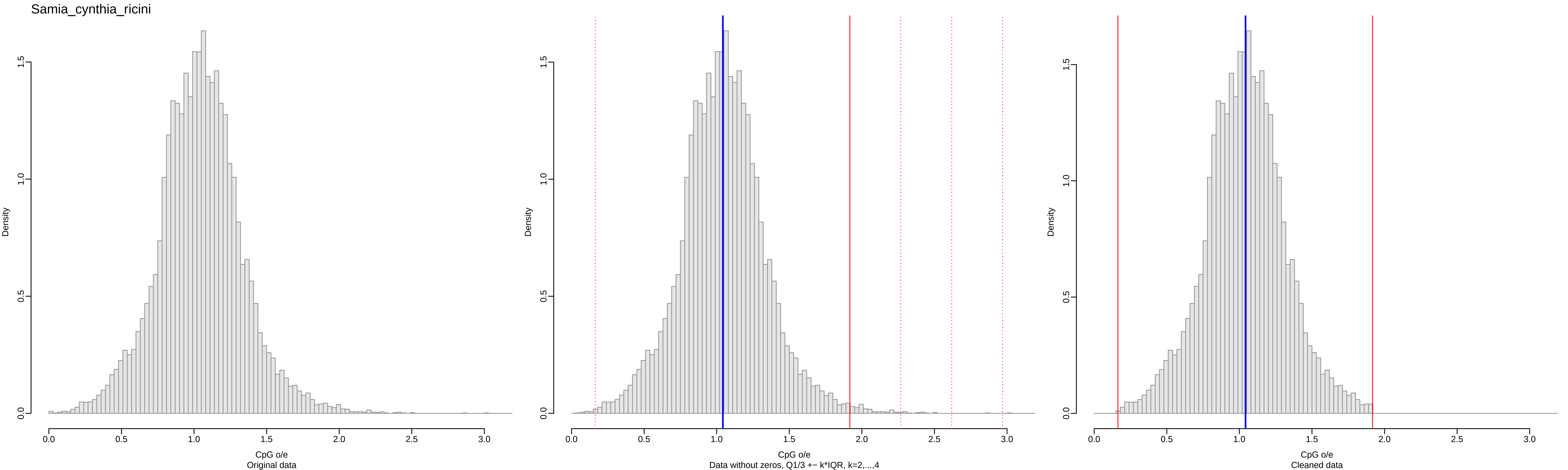

Samia_cynthia_ricini
Density
CpG o/e
Original data
Data without zeros, Q1/3 +− k*IQR, k=2,...,4
Cleaned data
0.0
0.5
1.0
1.5
2.0
2.5
3.0

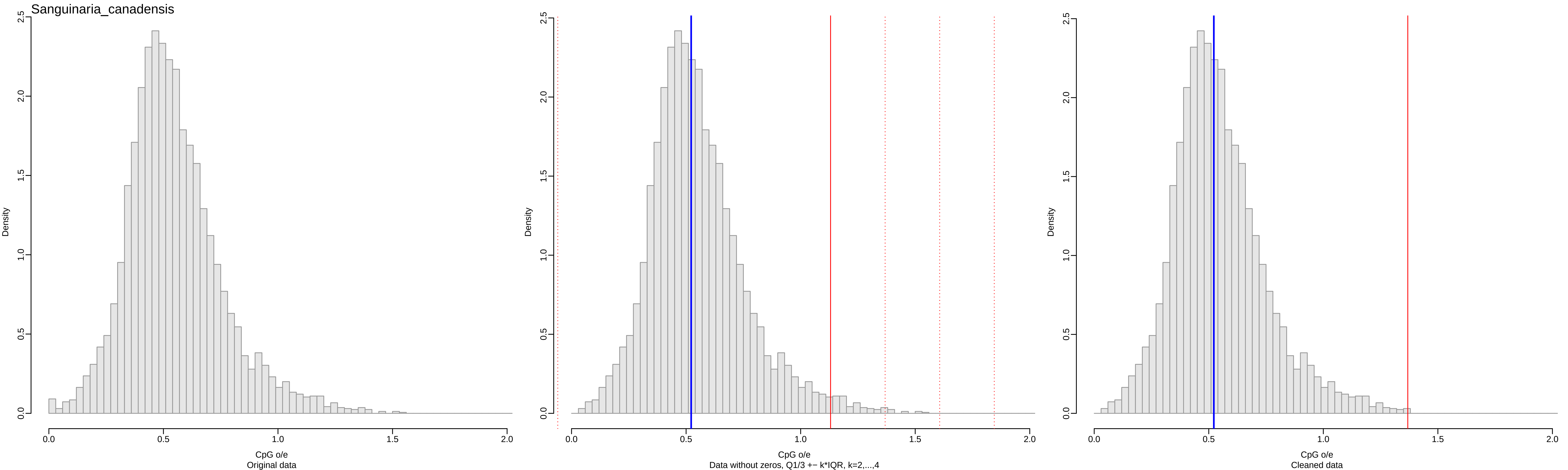

Sanguinaria_canadensis
Density
CpG o/e
Original data
Data without zeros, Q1/3 +− k*IQR, k=2,...,4
Cleaned data

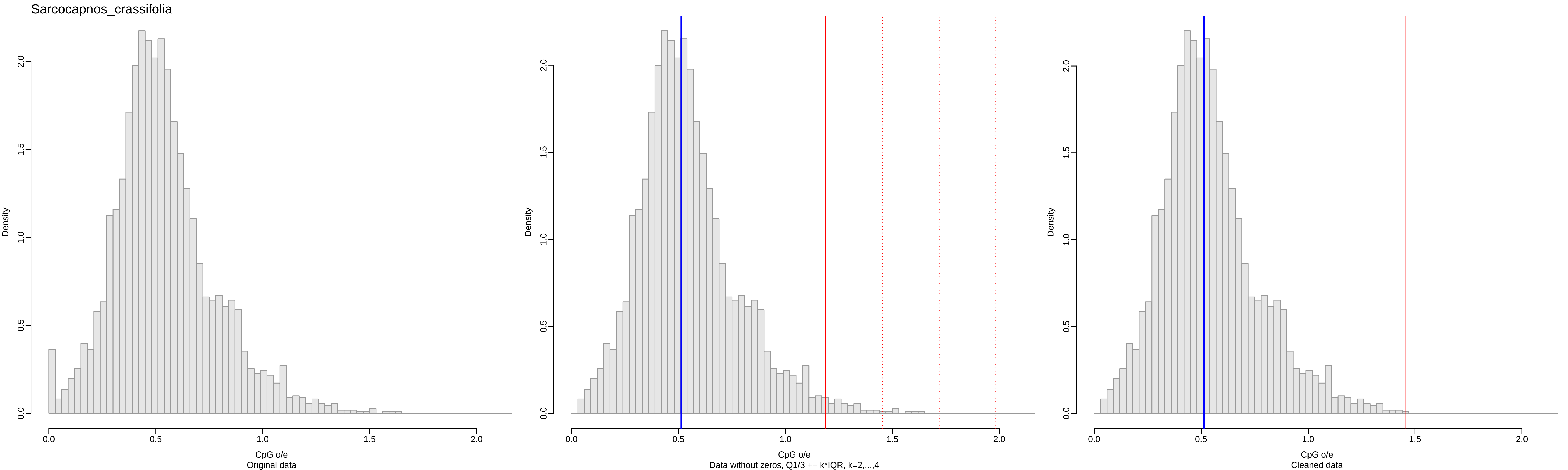
Sarcocapnos_crassifolia
Density
CpG o/e
Original data
CpG o/e
Data without zeros, Q1/3 +− k*IQR, k=2,...,4
CpG o/e
Cleaned data

Sarcocystis_neurona

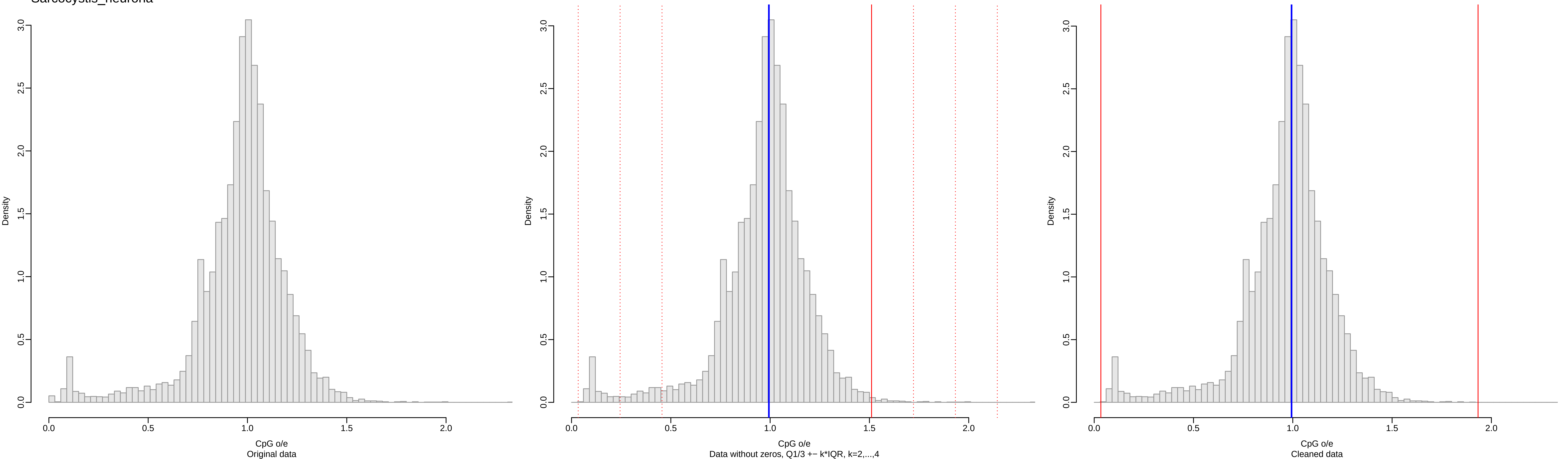

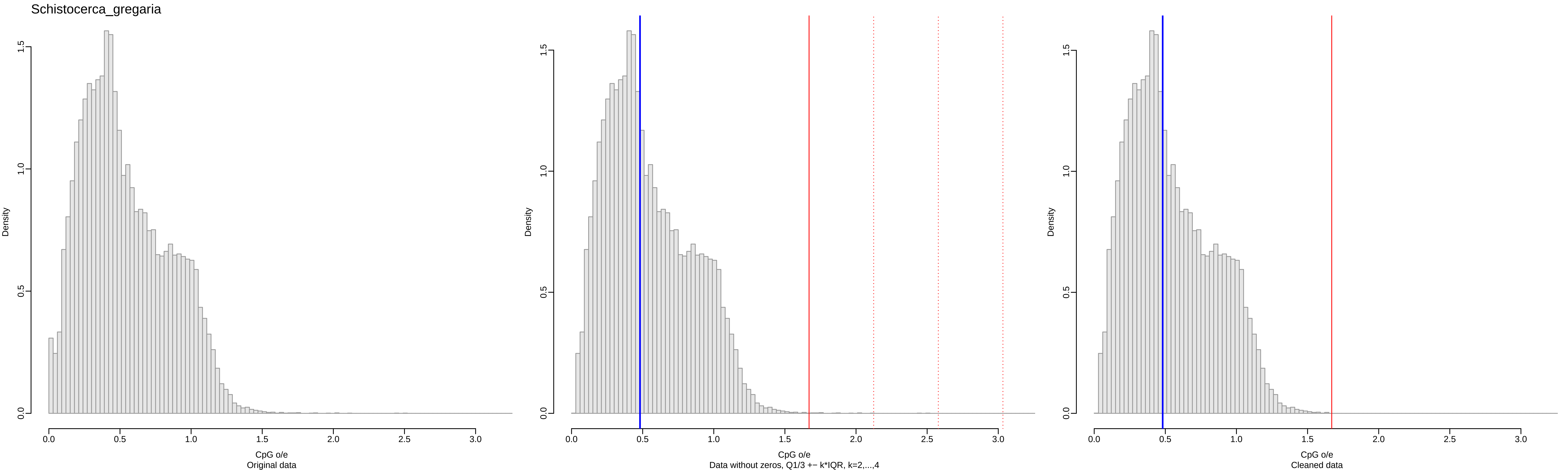

Schistocerca_gregaria
Density
CpG o/e
Original data
Data without zeros, Q1/3 +− k*IQR, k=2,...,4
Cleaned data
0.0
0.5
1.0
1.5
2.0
2.5
3.0

Schistosoma_japonicum

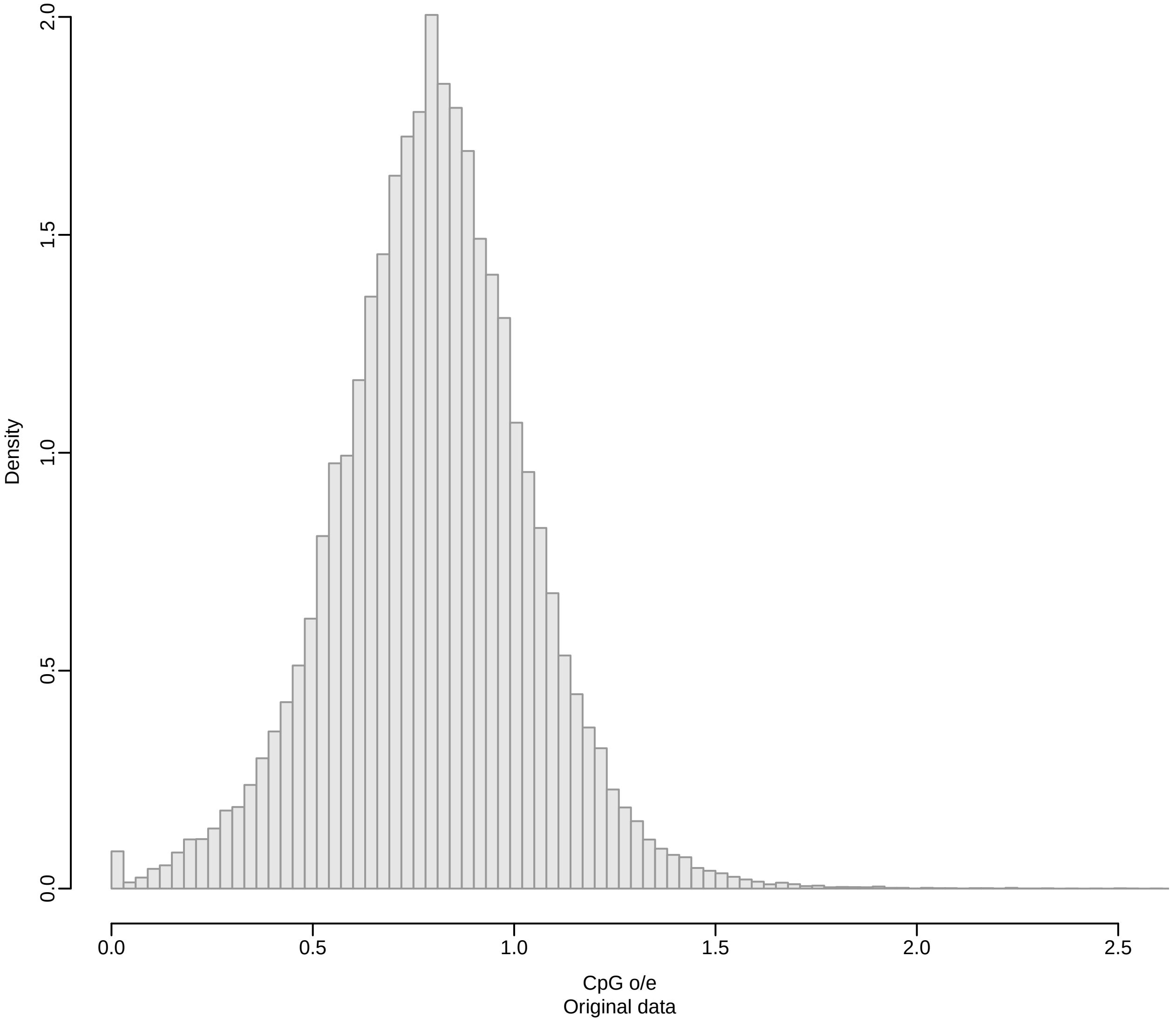

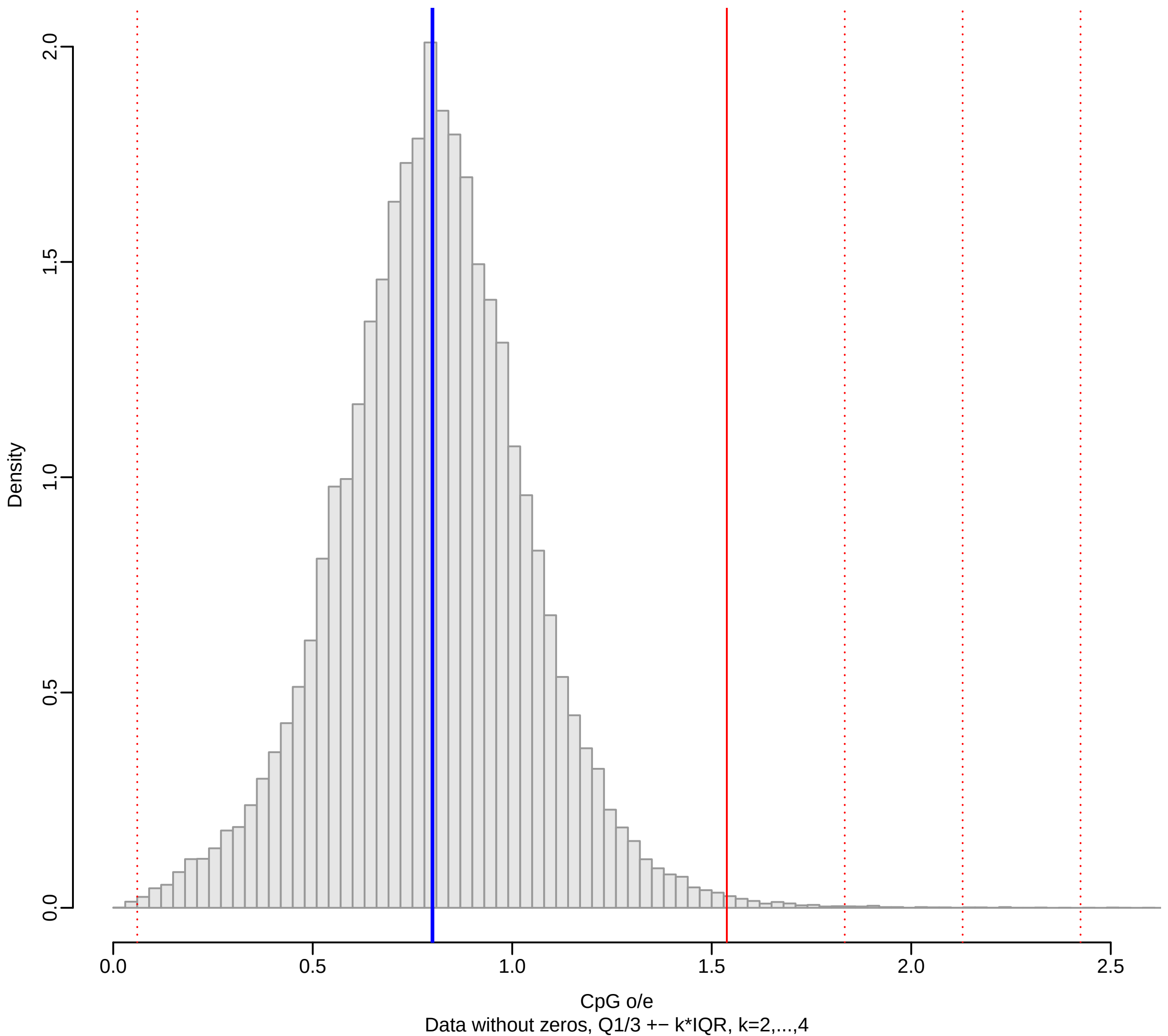

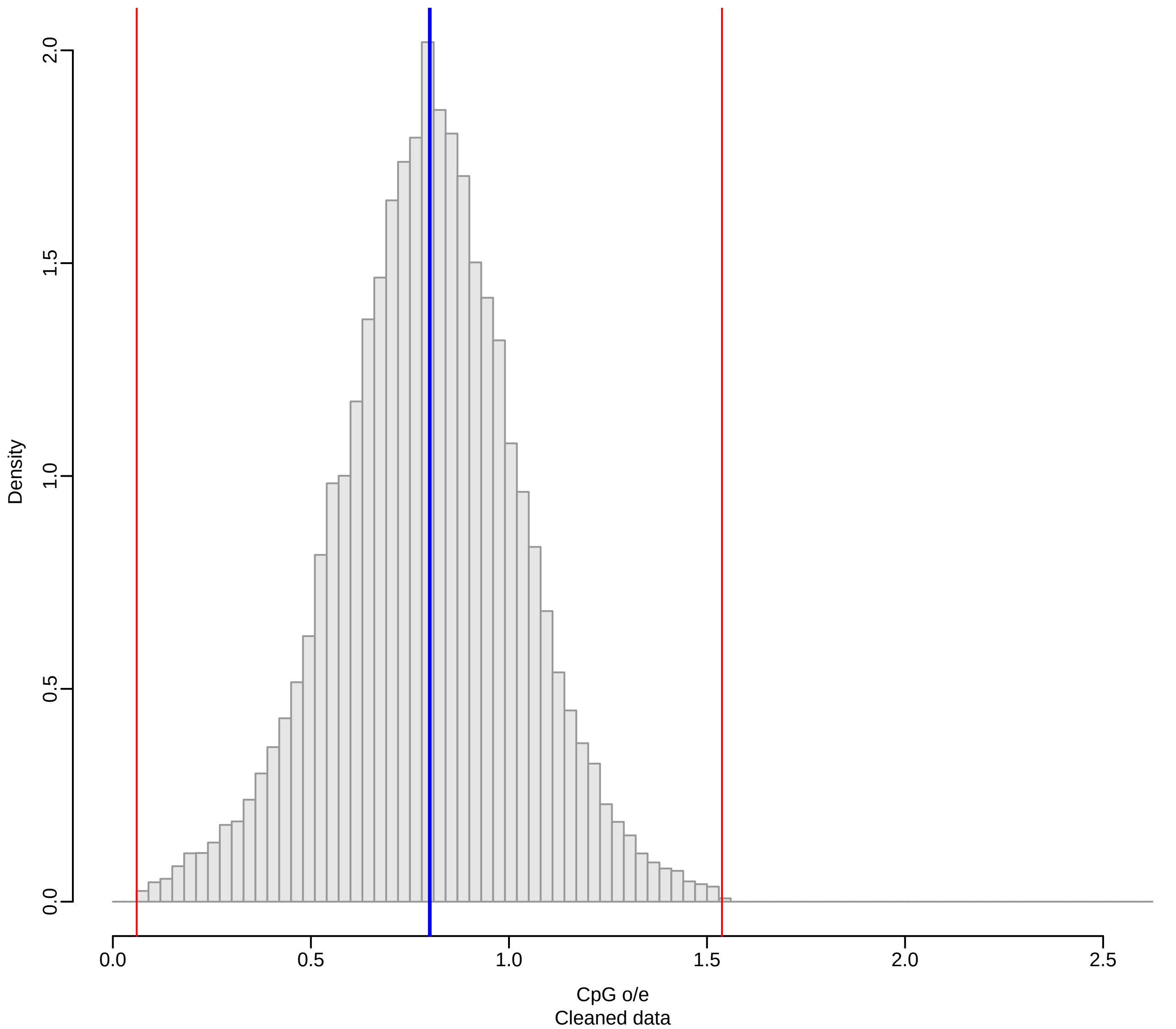

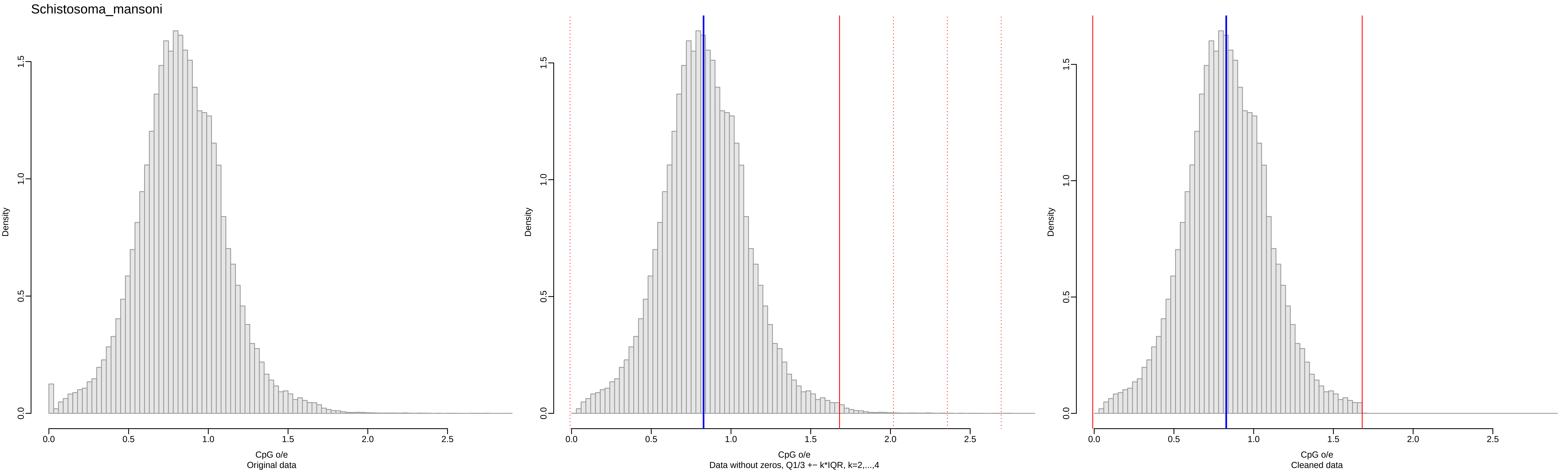

Schistosoma_mansoni
Density
0.0
0.5
1.0
1.5
2.0
2.5
CpG o/e
Original data
Data without zeros, Q1/3 +− k*IQR, k=2,...,4
Cleaned data

Schizosaccharomyces_pombe

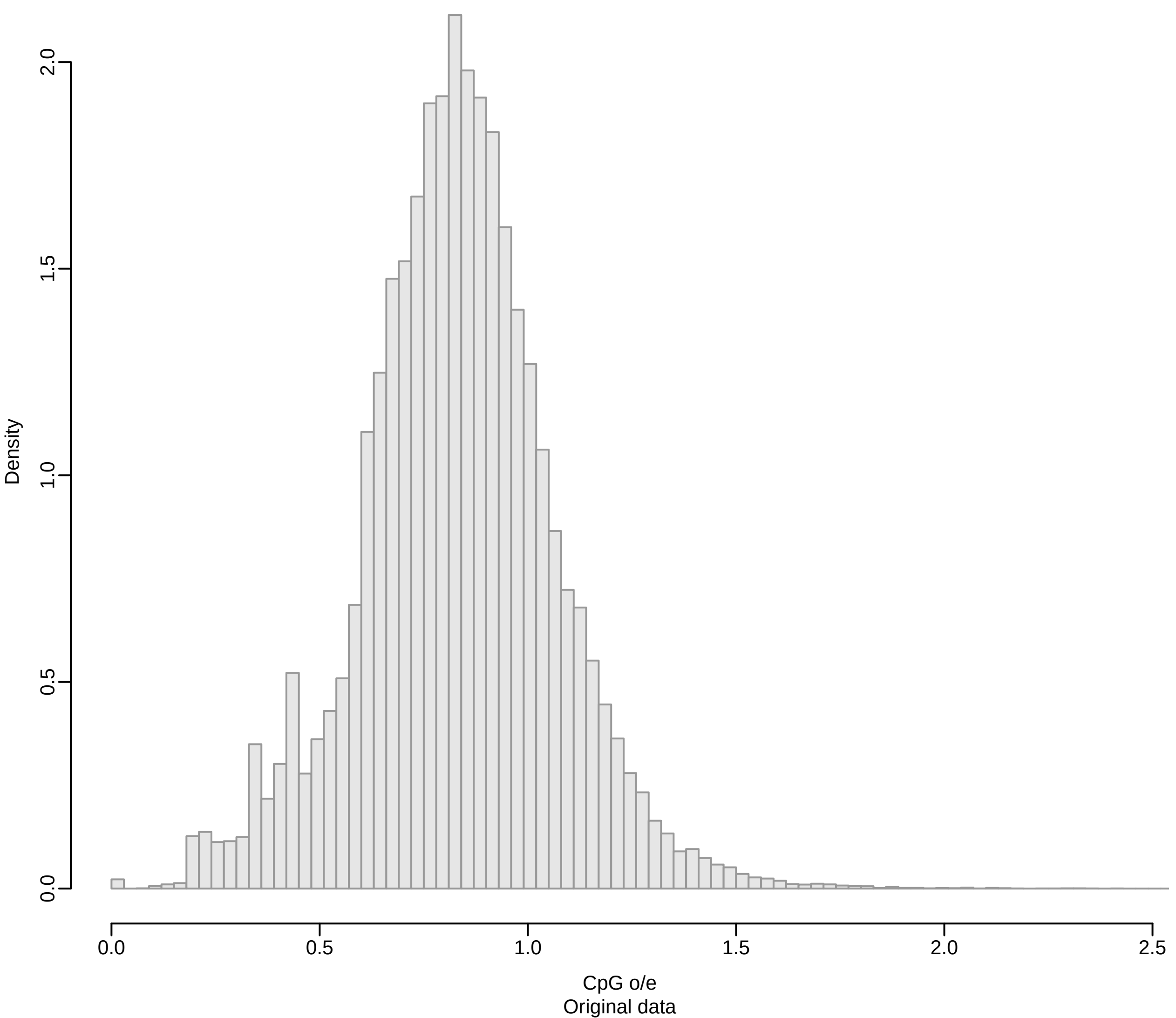

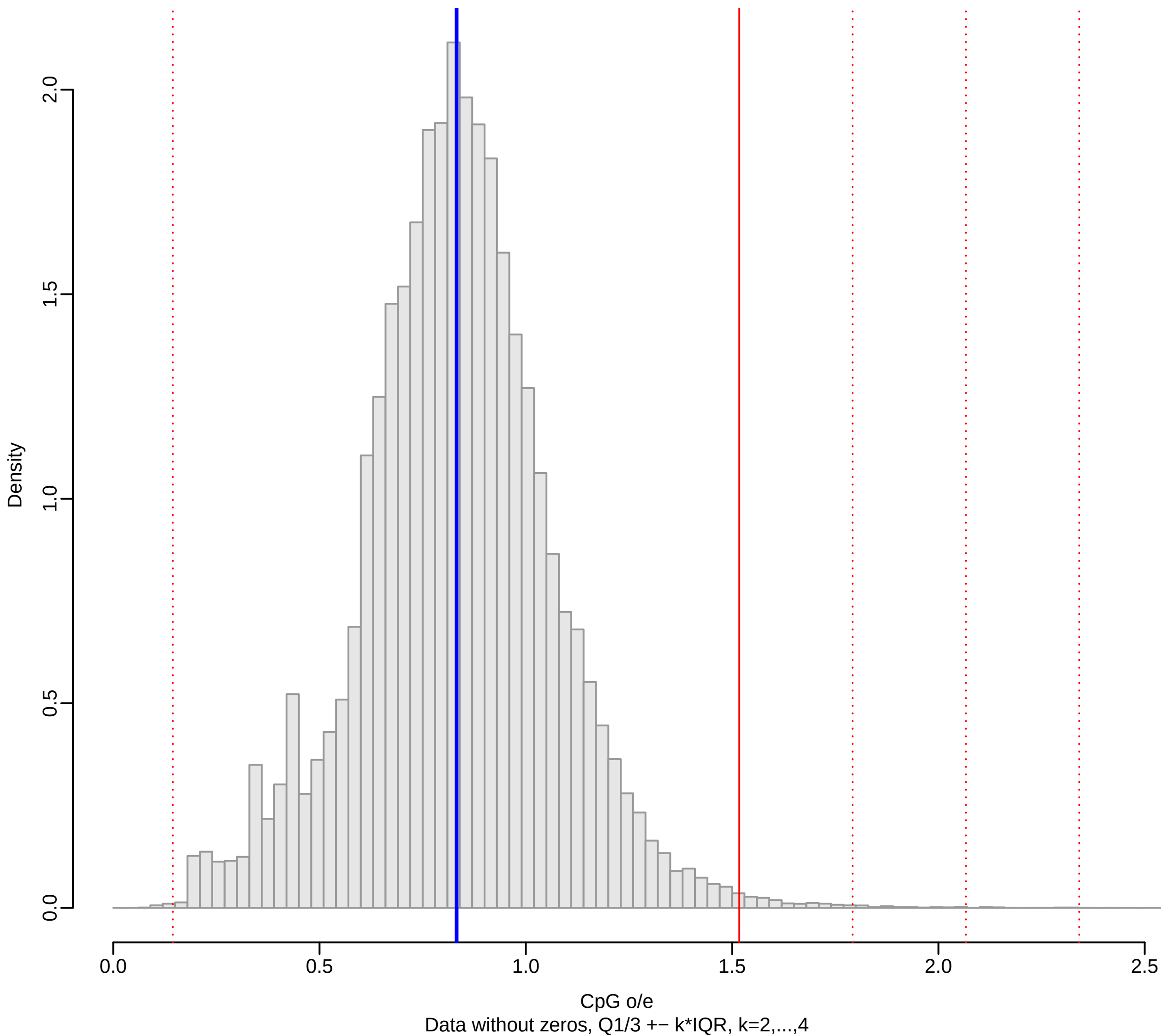

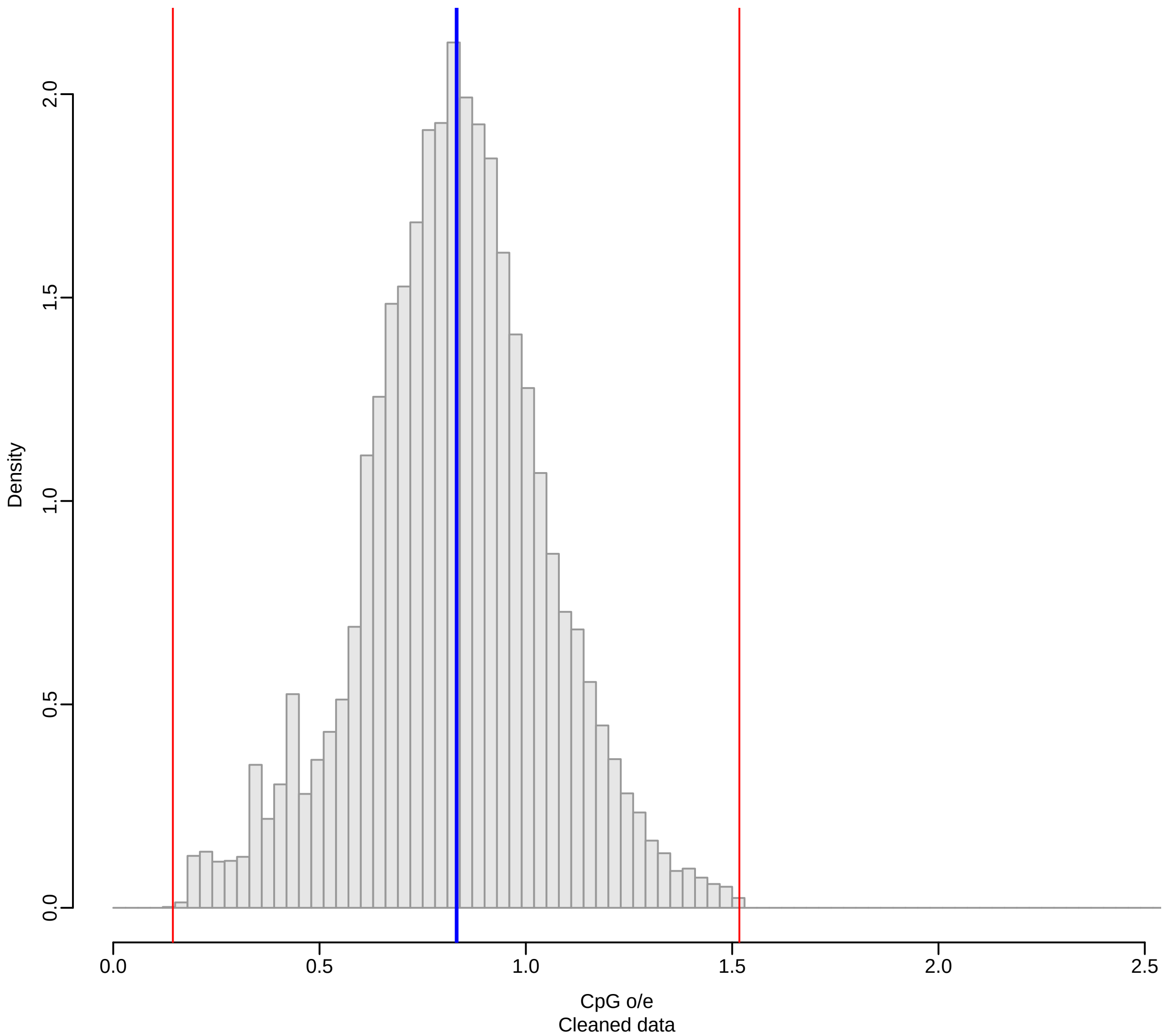

Schmidtea_mediterranea

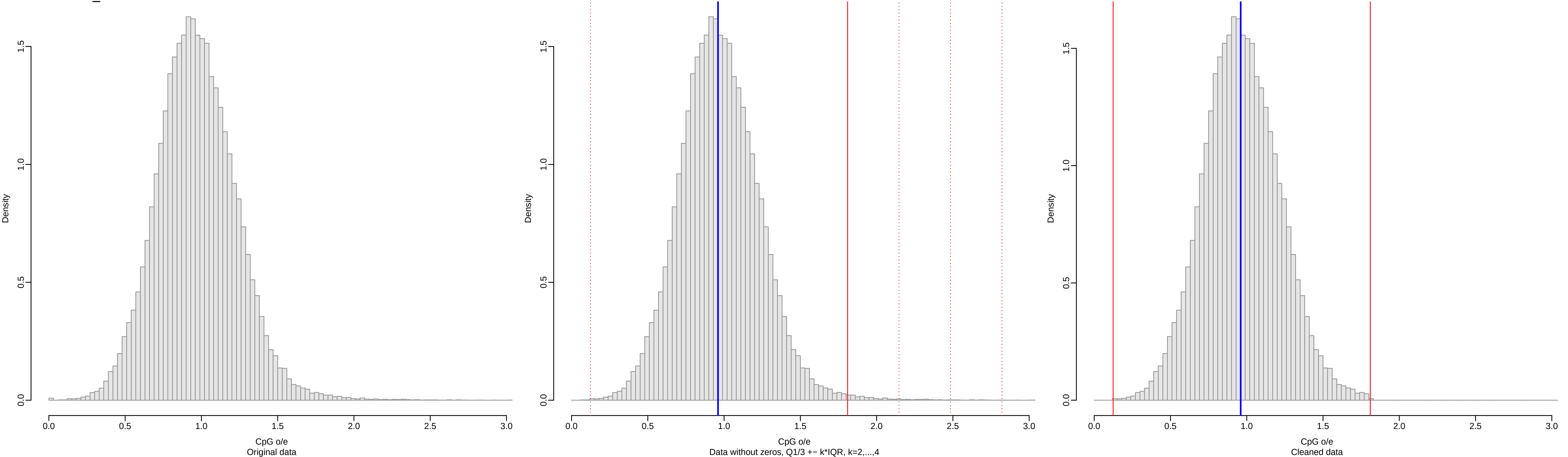

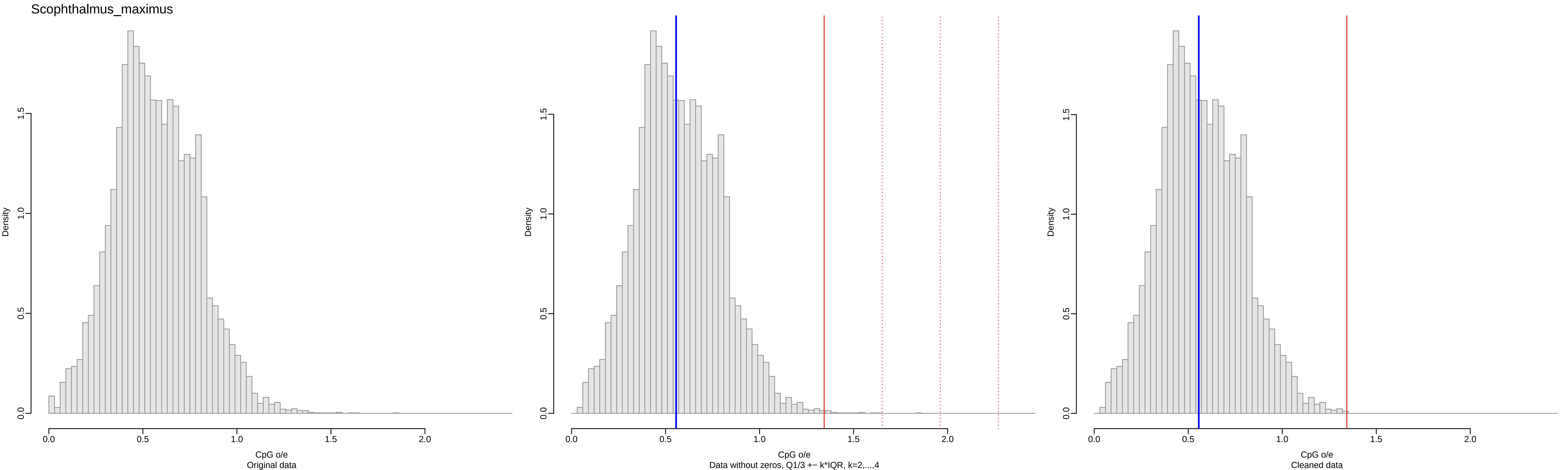

Scophthalmus_maximus
Density
0.0
0.5
1.0
1.5
2.0
CpG o/e
Original data
Data without zeros, Q1/3 +− k*IQR, k=2,...,4
Cleaned data

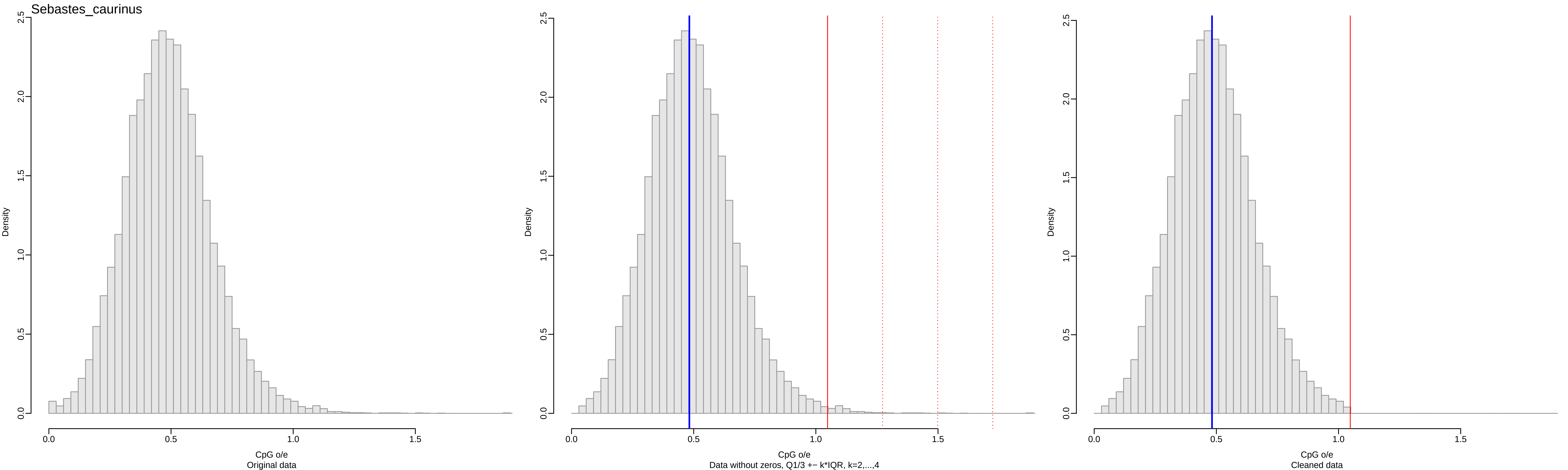
Sebastes_caurinus
Density
CpG o/e
Original data
Density
CpG o/e
Data without zeros, Q1/3 +− k*IQR, k=2,...,4
Density
CpG o/e
Cleaned data

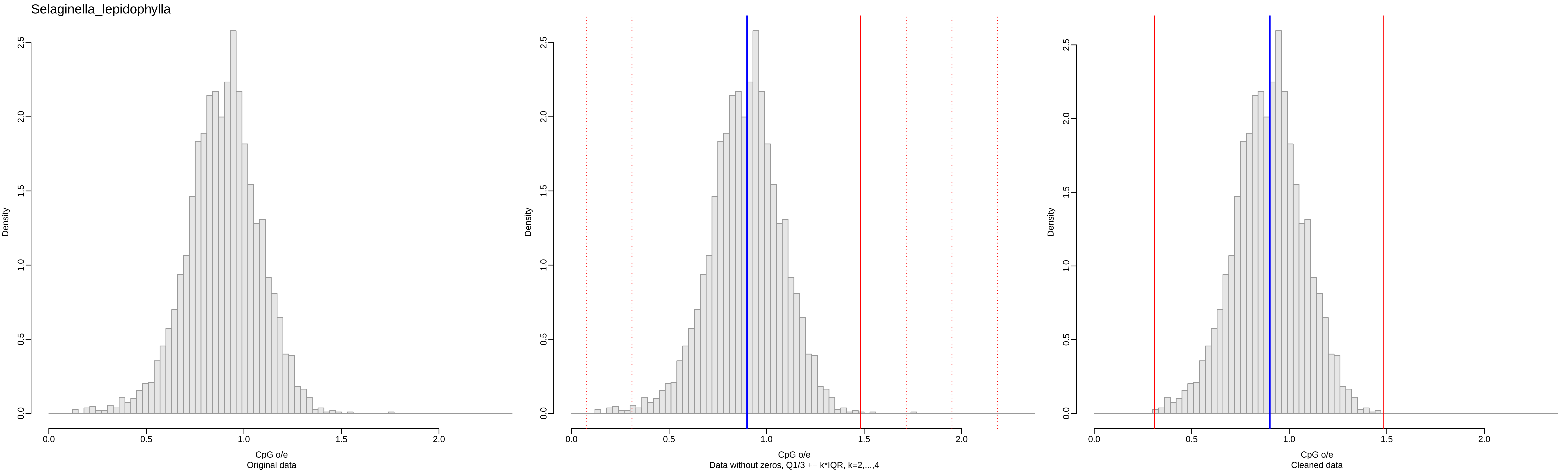

Selaginella_lepidophylla
Density
CpG o/e
Original data
Density
CpG o/e
Data without zeros, Q1/3 +− k*IQR, k=2,...,4
Density
CpG o/e
Cleaned data

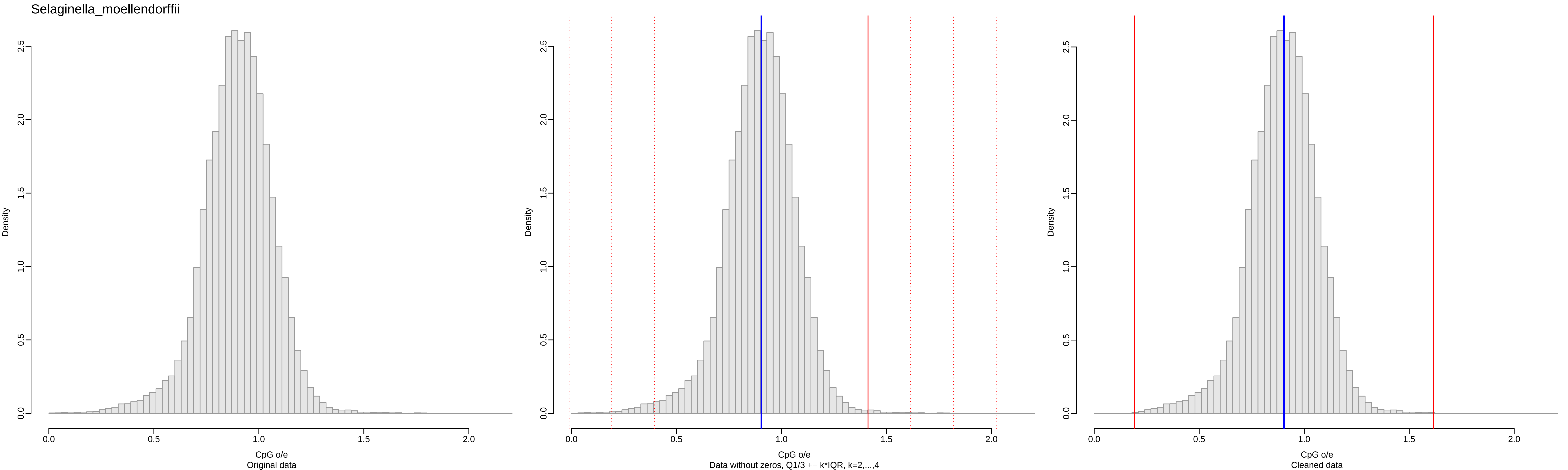

Selaginella_moellendorffii
Density
0.0
0.5
1.0
1.5
2.0
2.5
CpG o/e
Original data
Data without zeros, Q1/3 +− k*IQR, k=2,...,4
Cleaned data

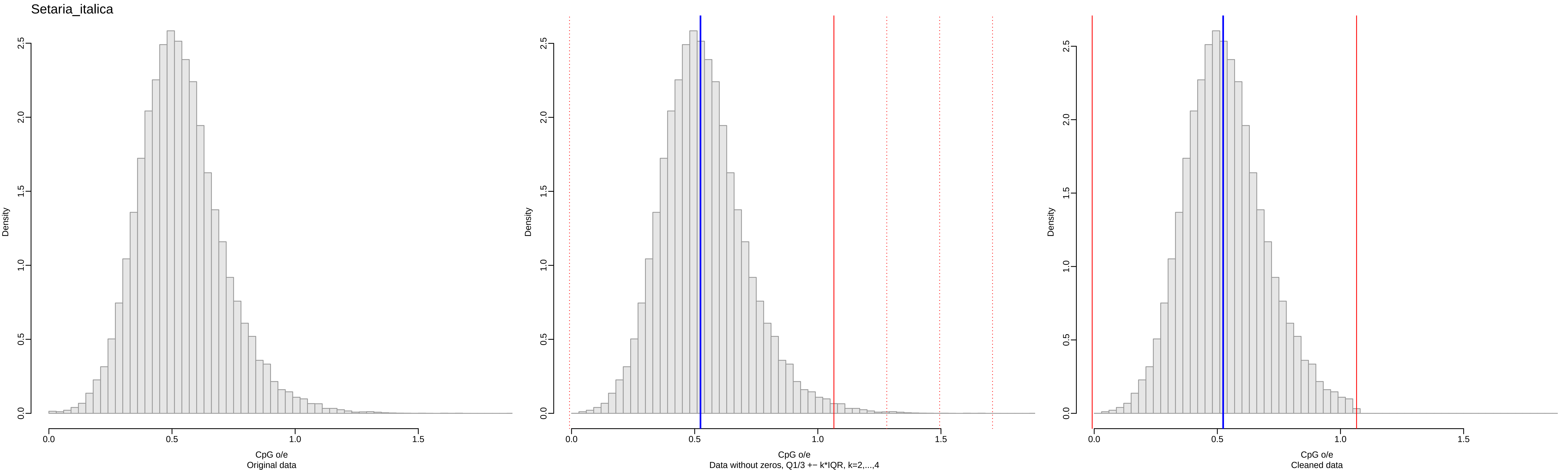

Setaria_italica
Density
CpG o/e
Original data
Density
CpG o/e
Data without zeros, Q1/3 +− k*IQR, k=2,...,4
Density
CpG o/e
Cleaned data
0.0
0.5
1.0
1.5
2.0
2.5

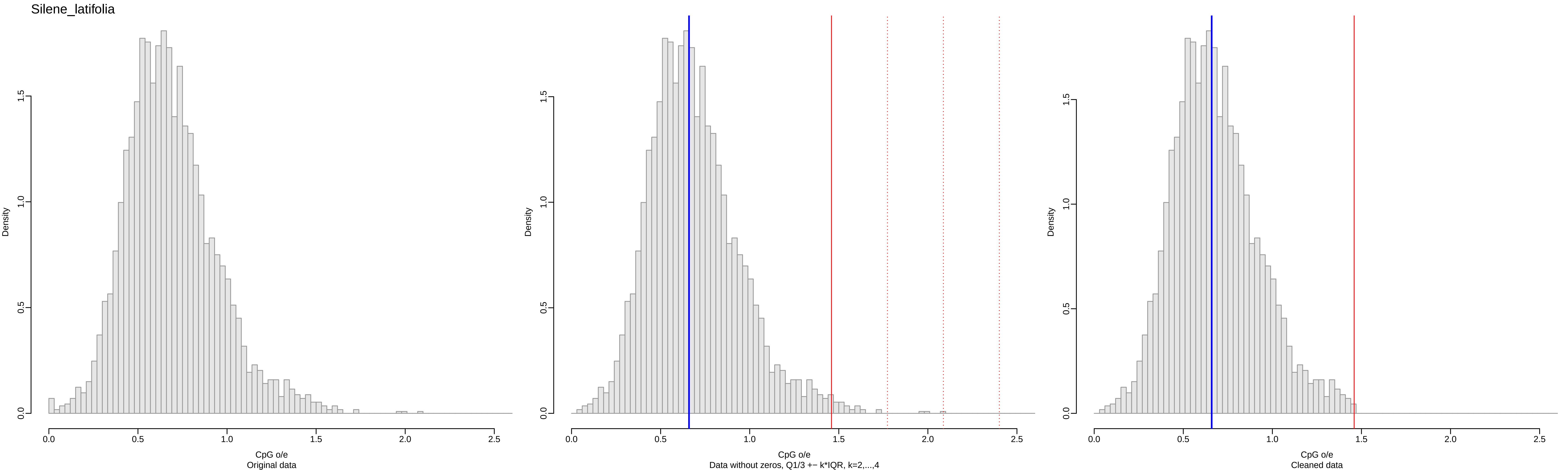

Silene_latifolia
Density
CpG o/e
Original data
Data without zeros, Q1/3 +− k*IQR, k=2,...,4
Cleaned data
0.0
0.5
1.0
1.5
2.0
2.5

Solanum_chacoense

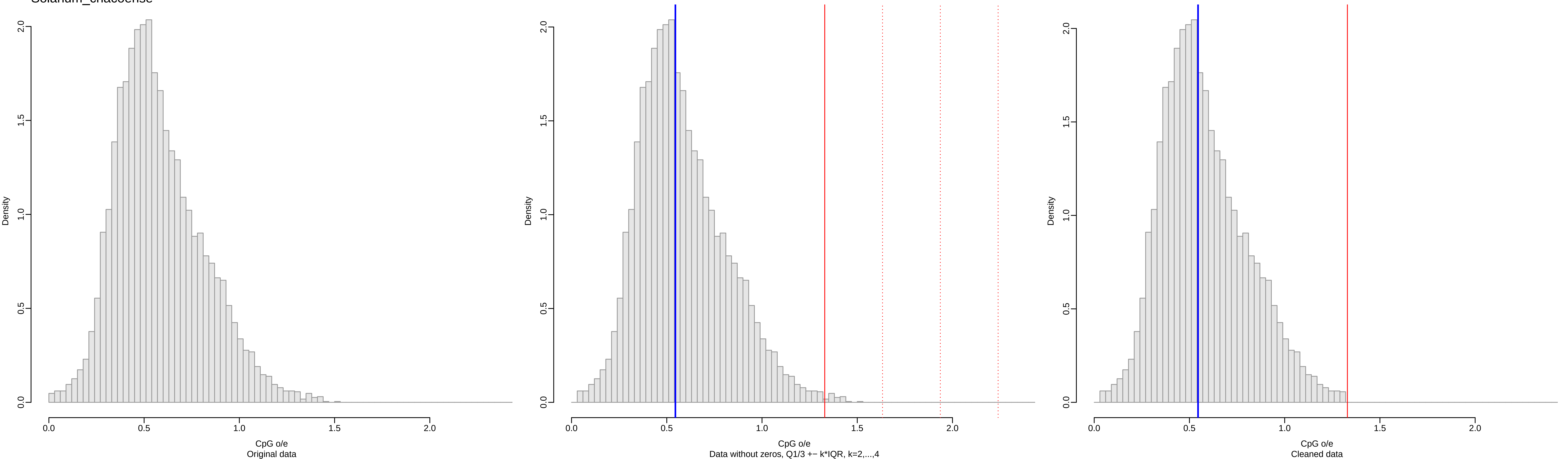

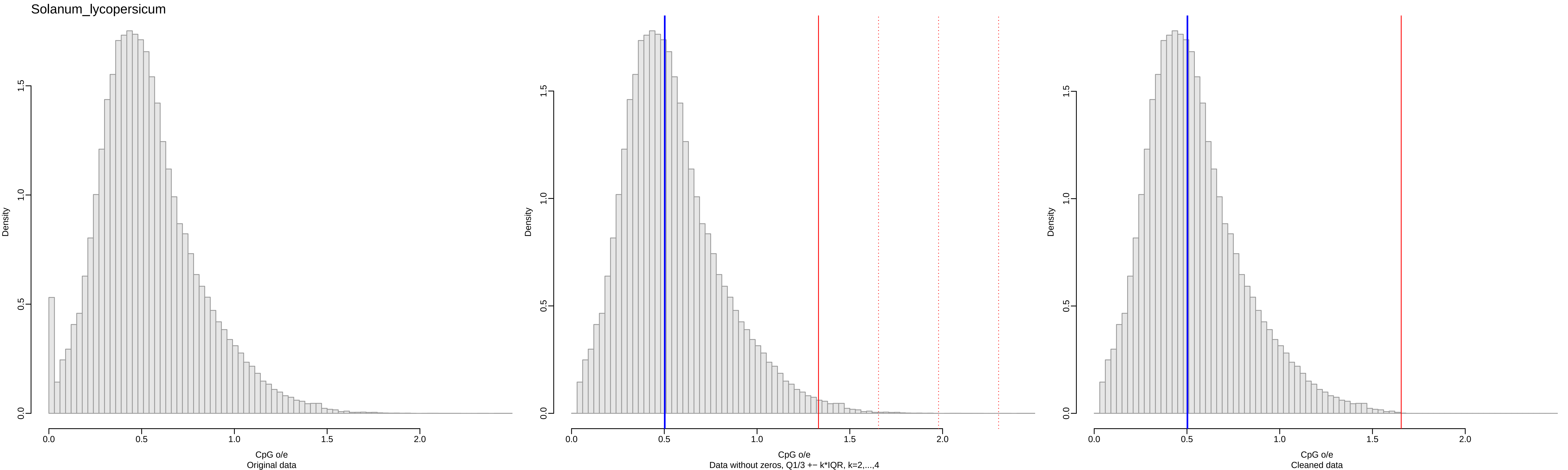
Solanum_lycopersicum
Density
0.0
0.5
1.0
1.5
2.0
CpG o/e
Original data
Data without zeros, Q1/3 +− k*IQR, k=2,...,4
Cleaned data

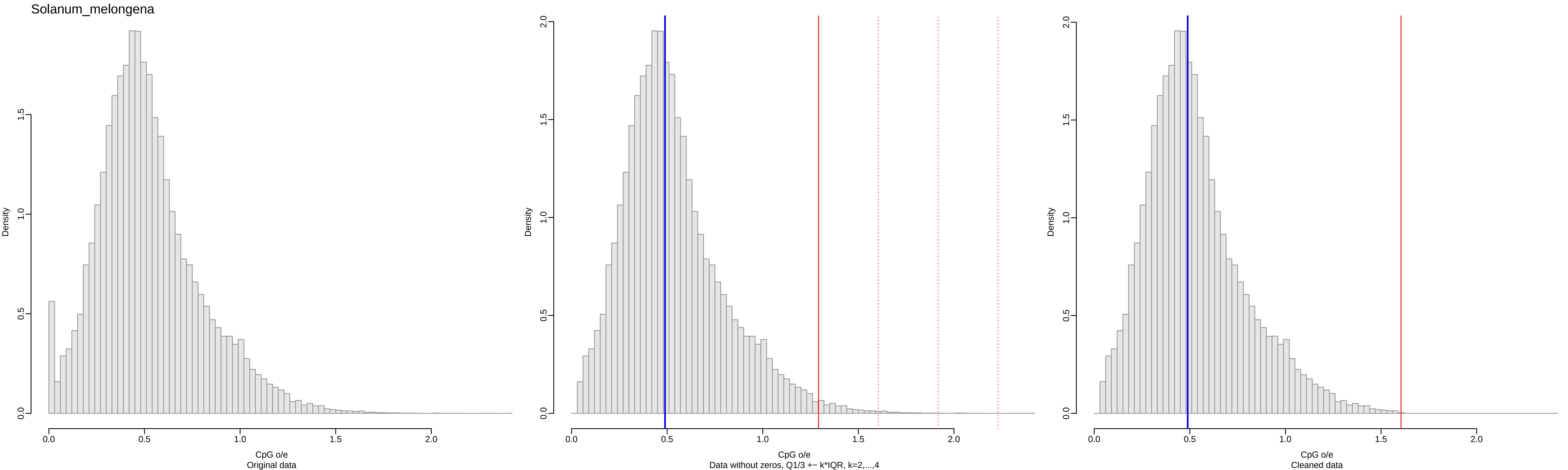
Solanum_melongena
Density
CpG o/e
Original data
Density
CpG o/e
Data without zeros, Q1/3 +− k*IQR, k=2,...,4
Density
CpG o/e
Cleaned data

Solanum_pennellii

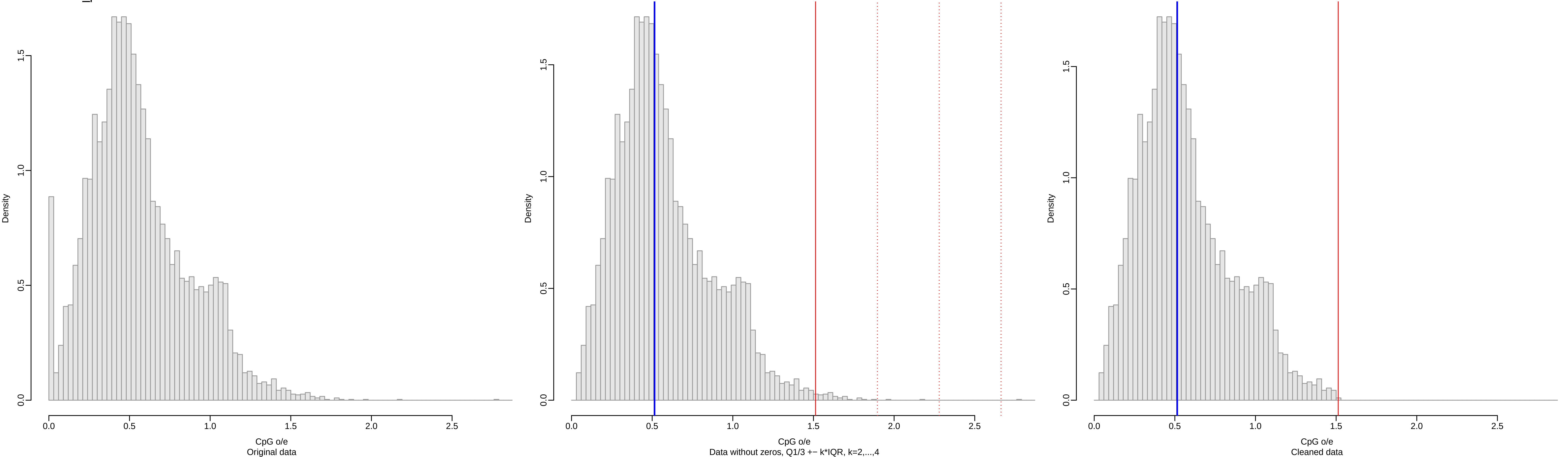

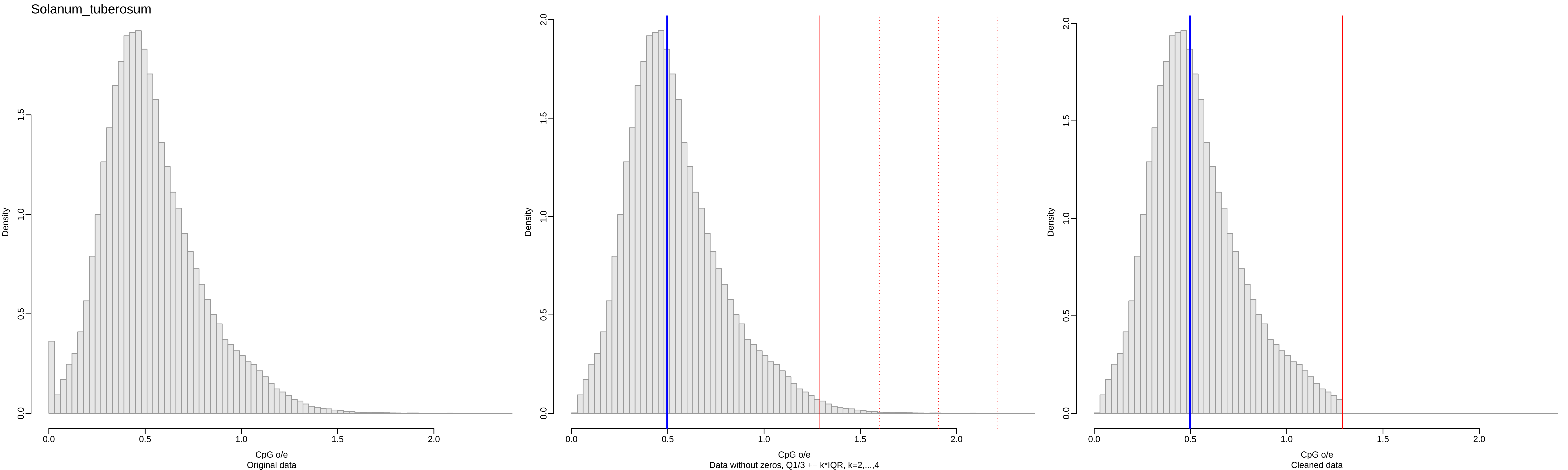

Solanum_tuberosum
Density
0.0
0.5
1.0
1.5
2.0
CpG o/e
Original data
Data without zeros, Q1/3 +− k*IQR, k=2,...,4
Cleaned data

Solea_senegalensis

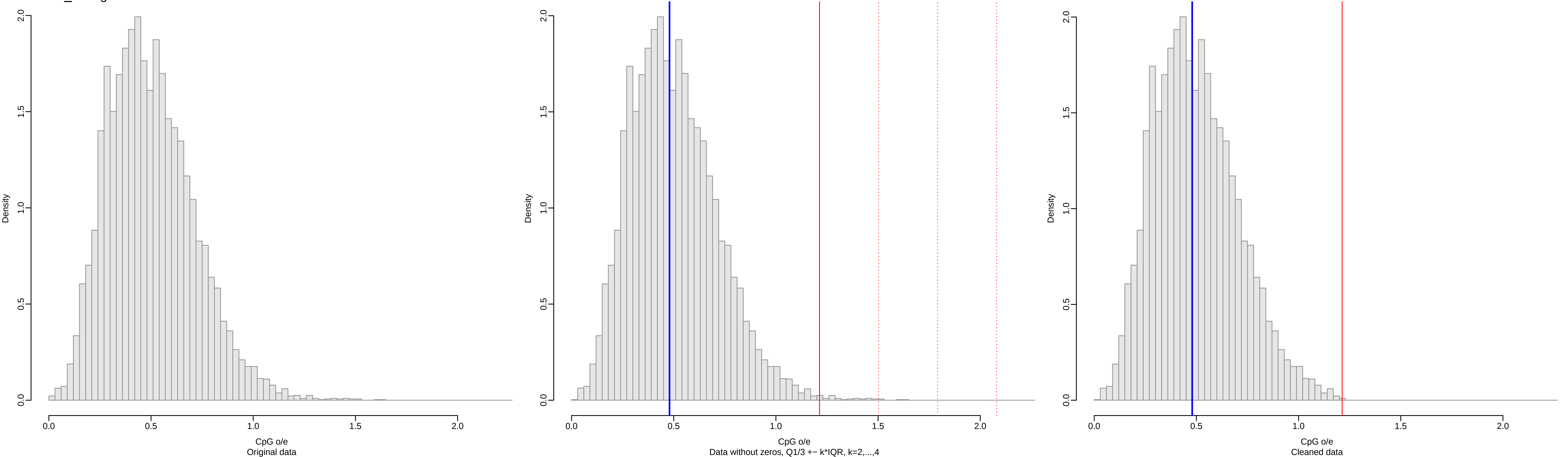

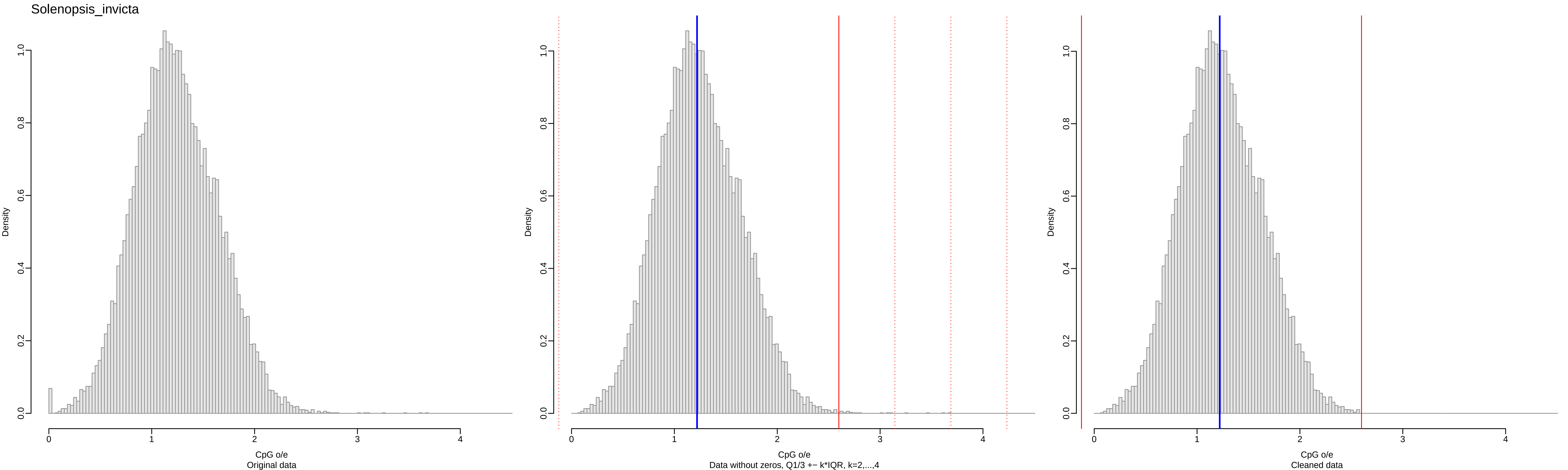
Solenopsis_invicta
Density
CpG o/e
Original data
Data without zeros, Q1/3 +− k*IQR, k=2,...,4
Cleaned data

Spalax_galili

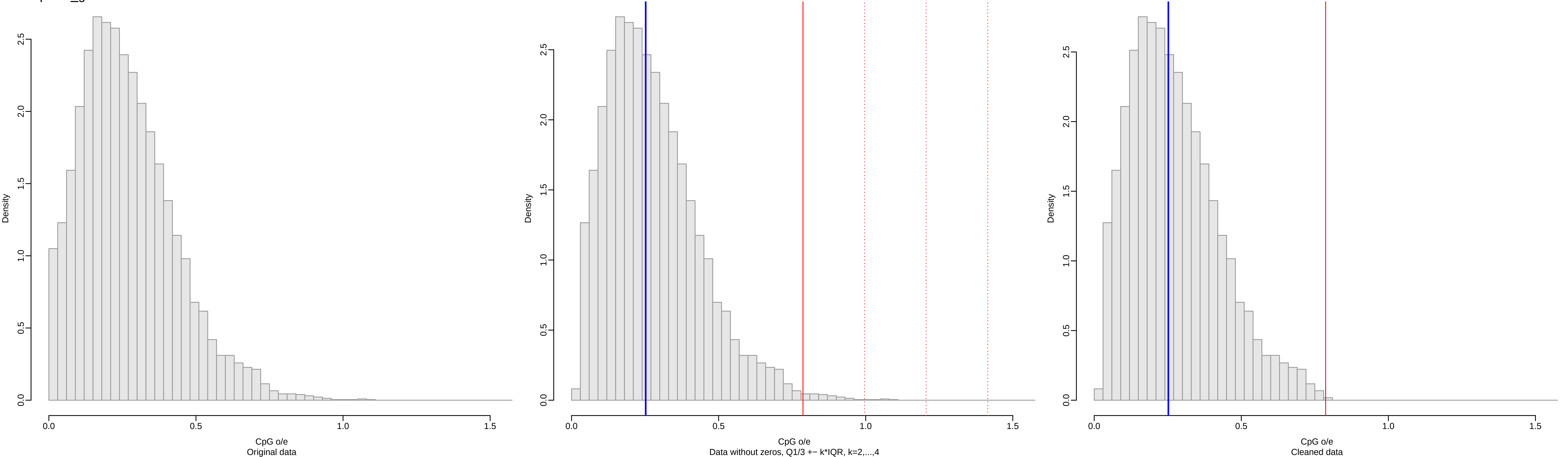

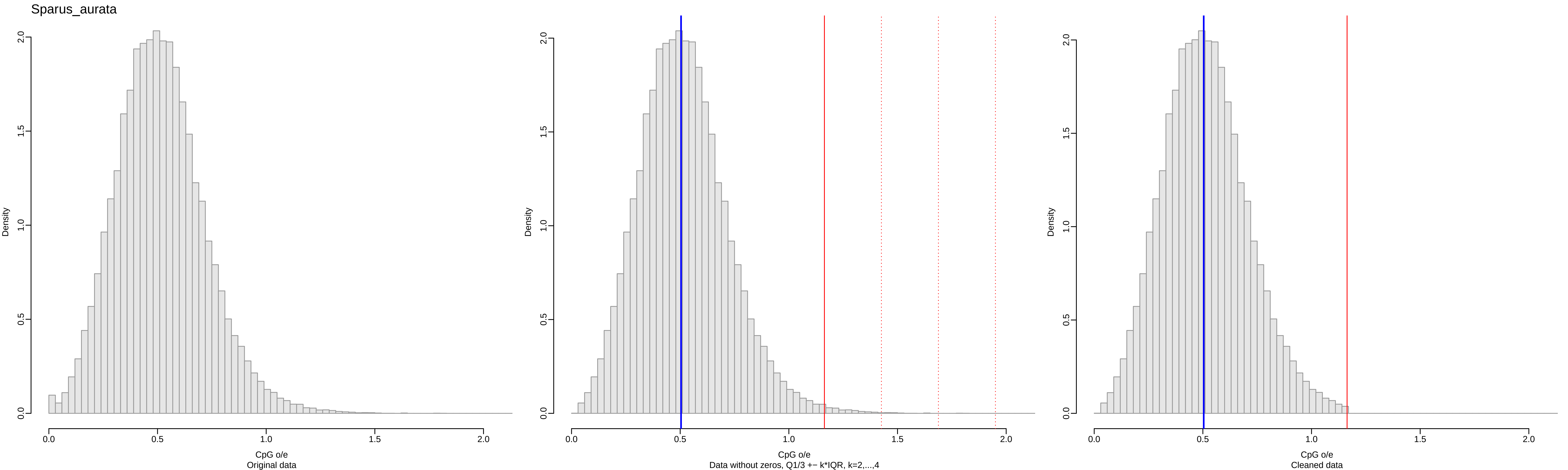
Sparus_aurata
Density
CpG o/e
Original data
Data without zeros, Q1/3 +− k*IQR, k=2,...,4
Cleaned data
0.0
0.5
1.0
1.5
2.0

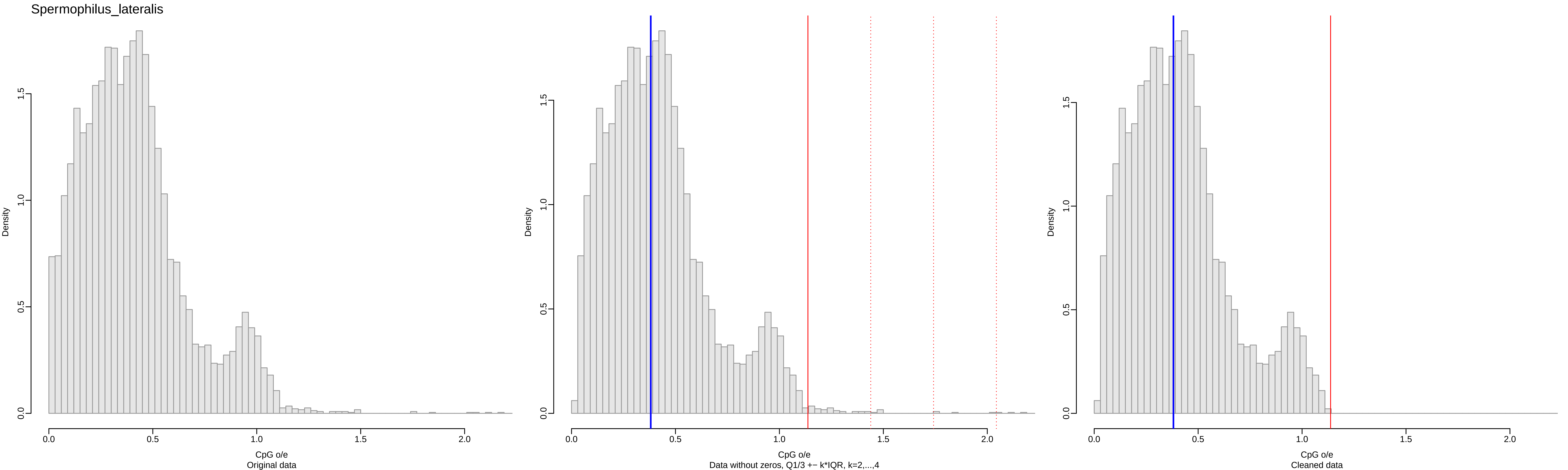
Spermophilus_lateralis
Density
CpG o/e
Original data
Data without zeros, Q1/3 +− k*IQR, k=2,...,4
Cleaned data
0.0
0.5
1.0
1.5
2.0

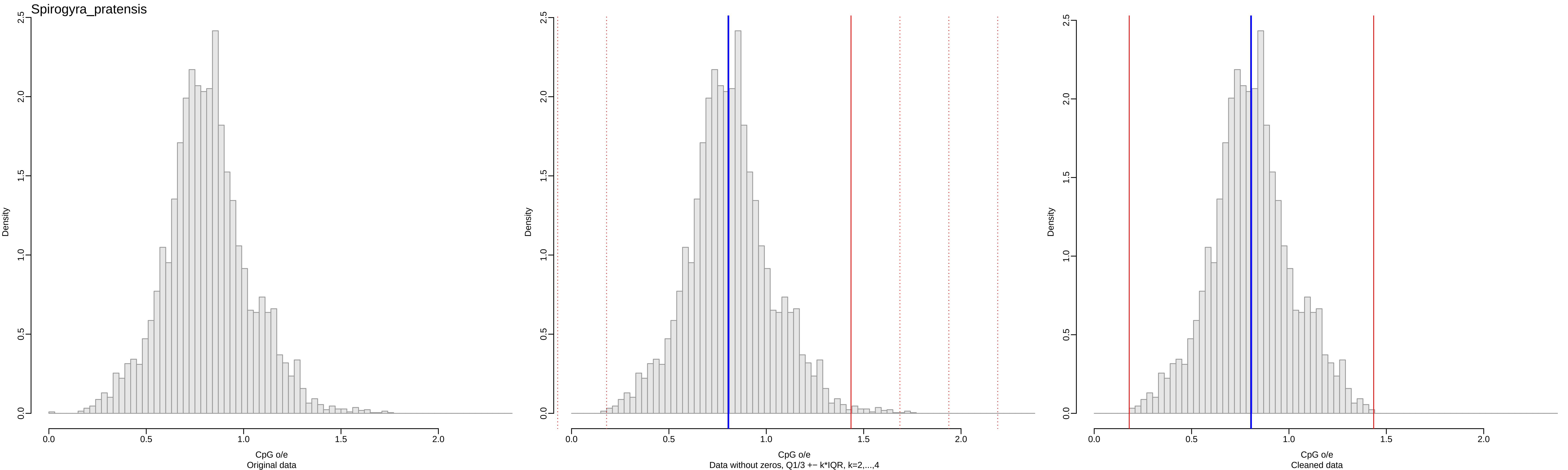

Spirogyra_pratensis
Density
CpG o/e
Original data
Data without zeros, Q1/3 +− k*IQR, k=2,...,4
Cleaned data

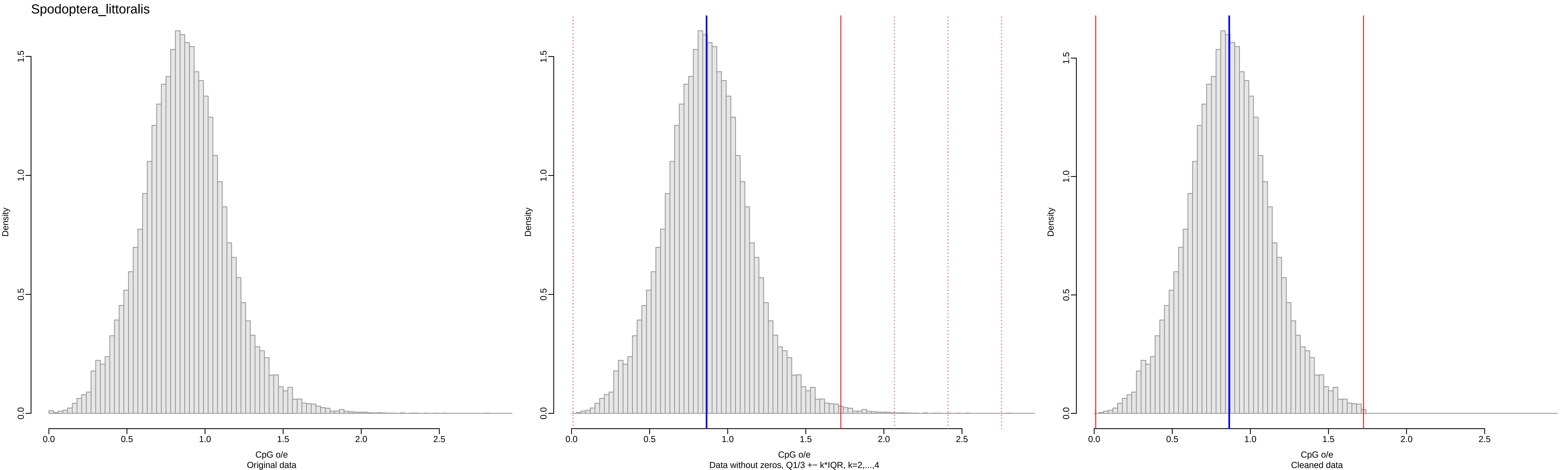

Spodoptera_littoralis
Density
0.0
0.5
1.0
1.5
0.0
0.5
1.0
1.5
2.0
2.5
CpG o/e
Original data
Data without zeros, Q1/3 +− k*IQR, k=2,...,4
Cleaned data

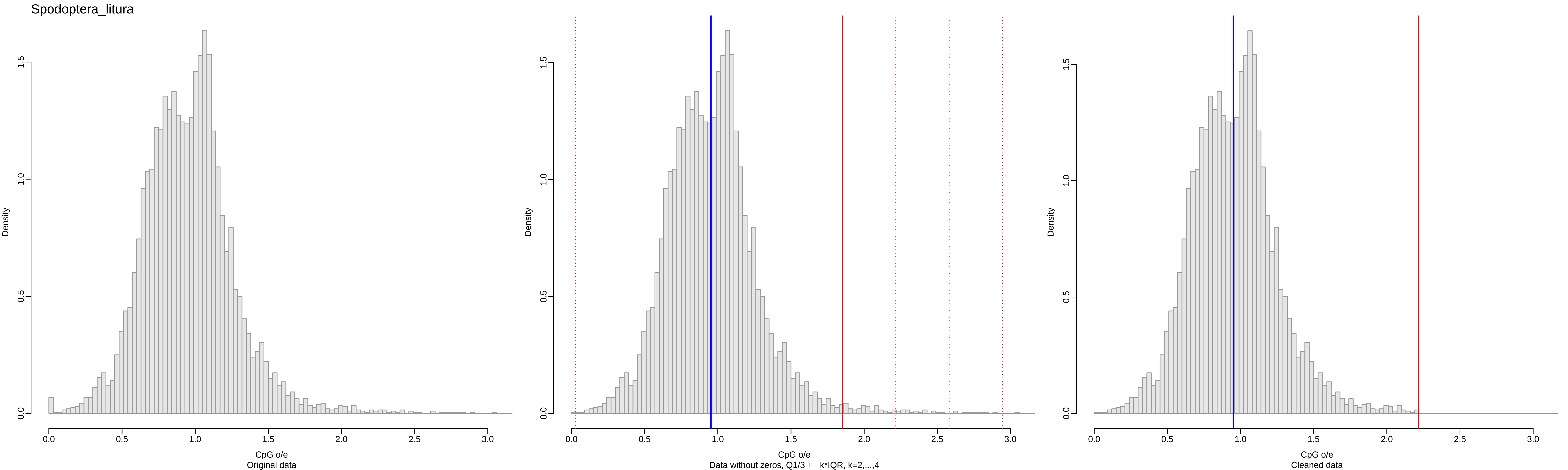
Spodoptera_litura
Density
CpG o/e
Original data
CpG o/e
Data without zeros, Q1/3 +− k*IQR, k=2,...,4
CpG o/e
Cleaned data
0.0
0.5
1.0
1.5
2.0
2.5
3.0

Steinernema_carpocapsae

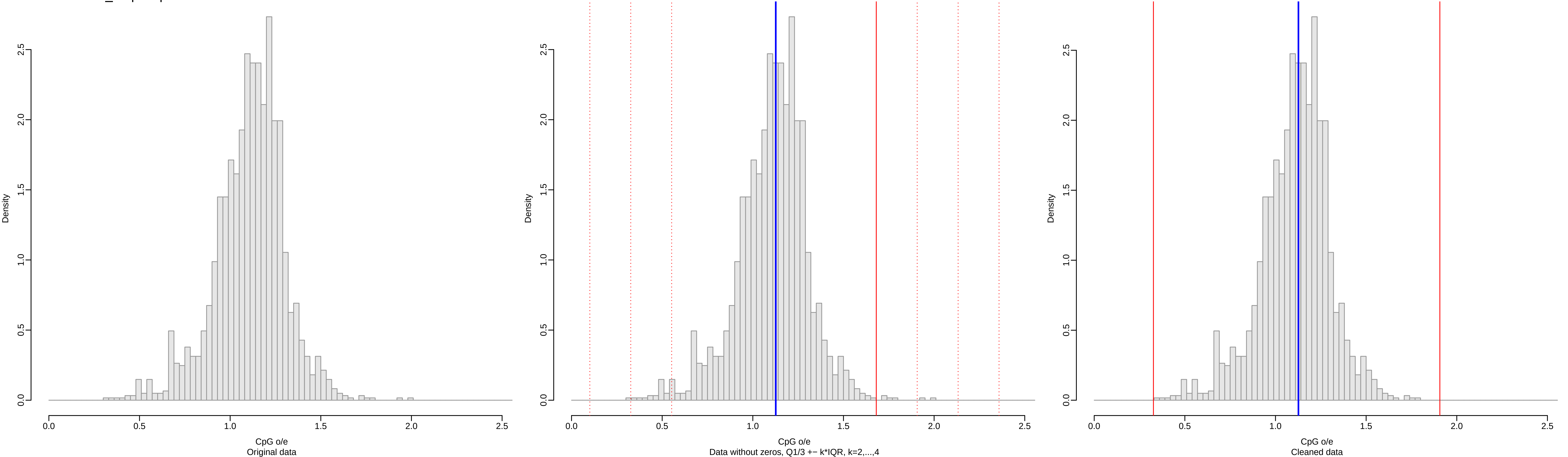

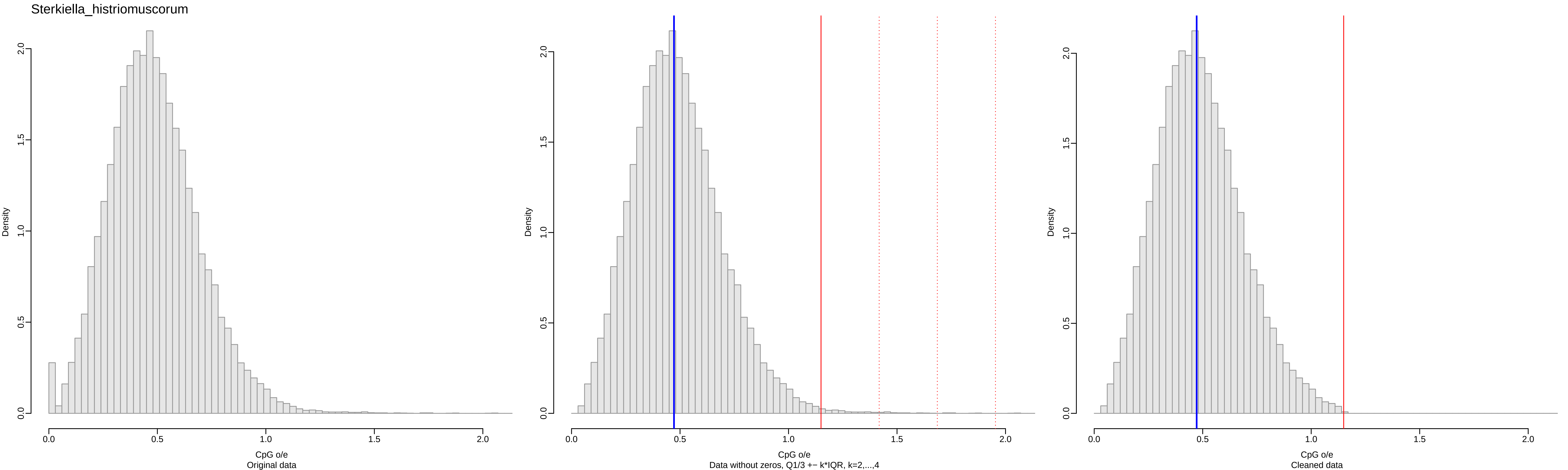
Sterkiella_histriomuscorum
Density
CpG o/e
Original data
Data without zeros, Q1/3 +− k*IQR, k=2,...,4
Cleaned data

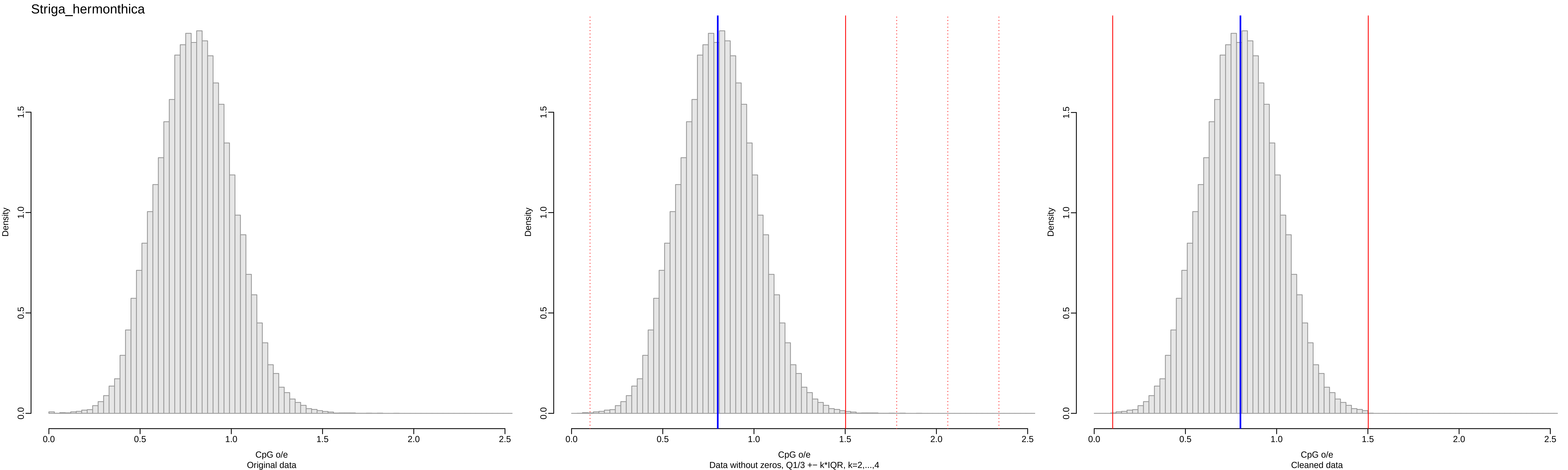

Striga_hermonthica
Density
CpG o/e
Original data
CpG o/e
Data without zeros, Q1/3 +− k*IQR, k=2,...,4
CpG o/e
Cleaned data

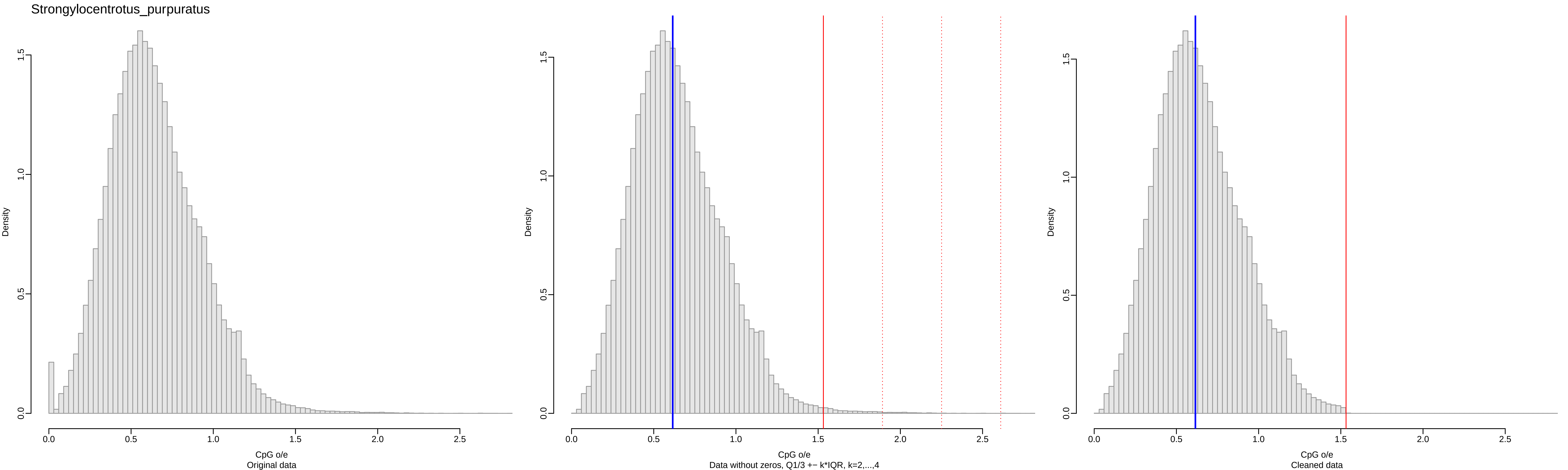

Strongylocentrotus_purpuratus
Density
CpG o/e
Original data
Data without zeros, Q1/3 +− k*IQR, k=2,...,4
Cleaned data
0.0
0.5
1.0
1.5
2.0
2.5

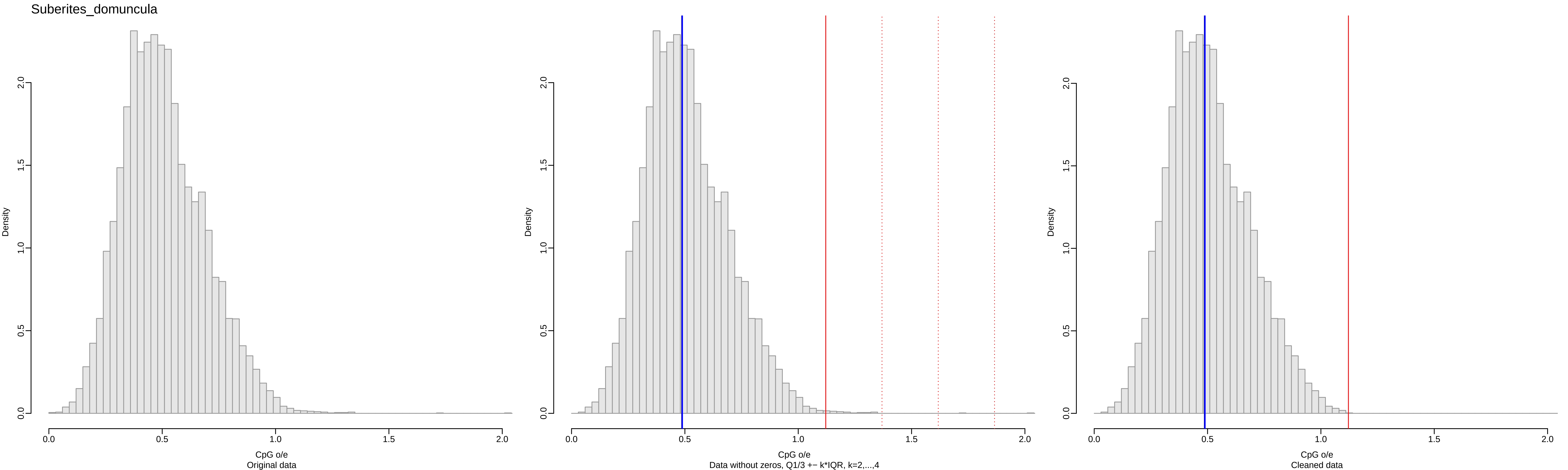
Suberites_domuncula
Density
CpG o/e
Original data
Data without zeros, Q1/3 +− k*IQR, k=2,...,4
Cleaned data
0.0
0.5
1.0
1.5
2.0

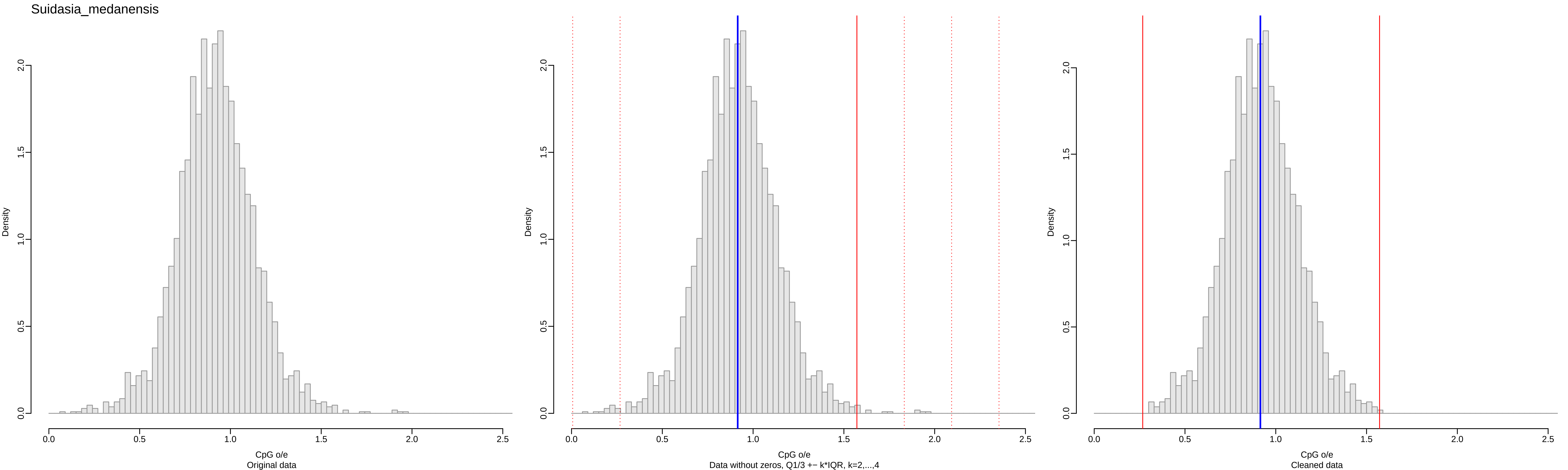
Suidasia_medanensis
Density
CpG o/e
Original data
Data without zeros, Q1/3 +− k*IQR, k=2,...,4
Cleaned data
0.0
0.5
1.0
1.5
2.0
2.5

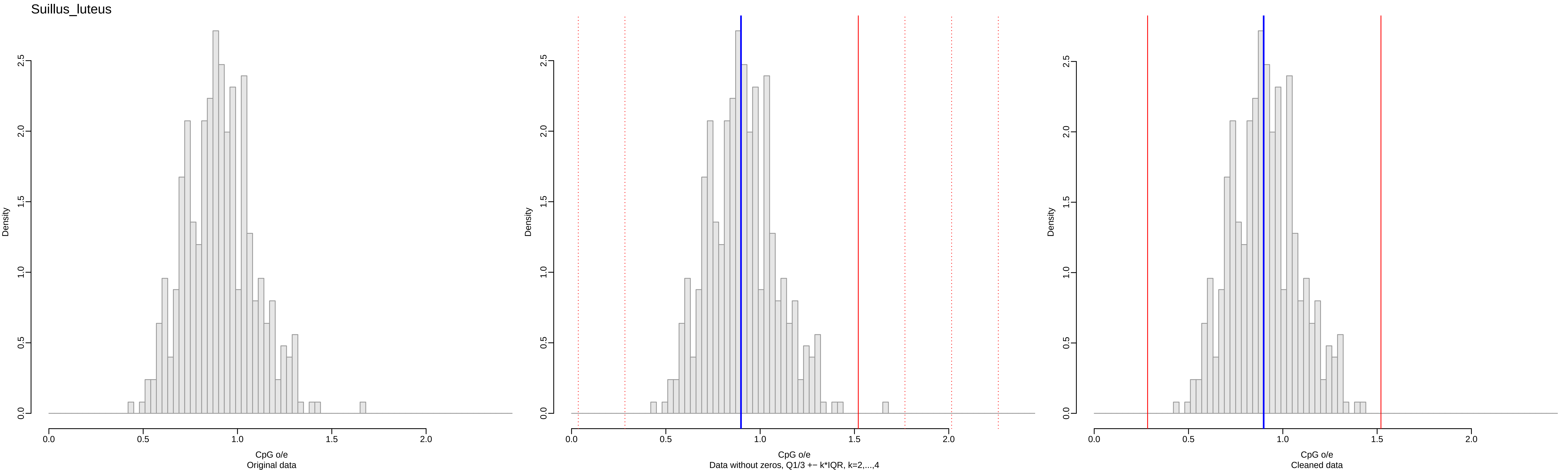

Suillus_luteus
Density
CpG o/e
Original data
Data without zeros, Q1/3 +− k*IQR, k=2,...,4
Cleaned data
0.0
0.5
1.0
1.5
2.0
2.5

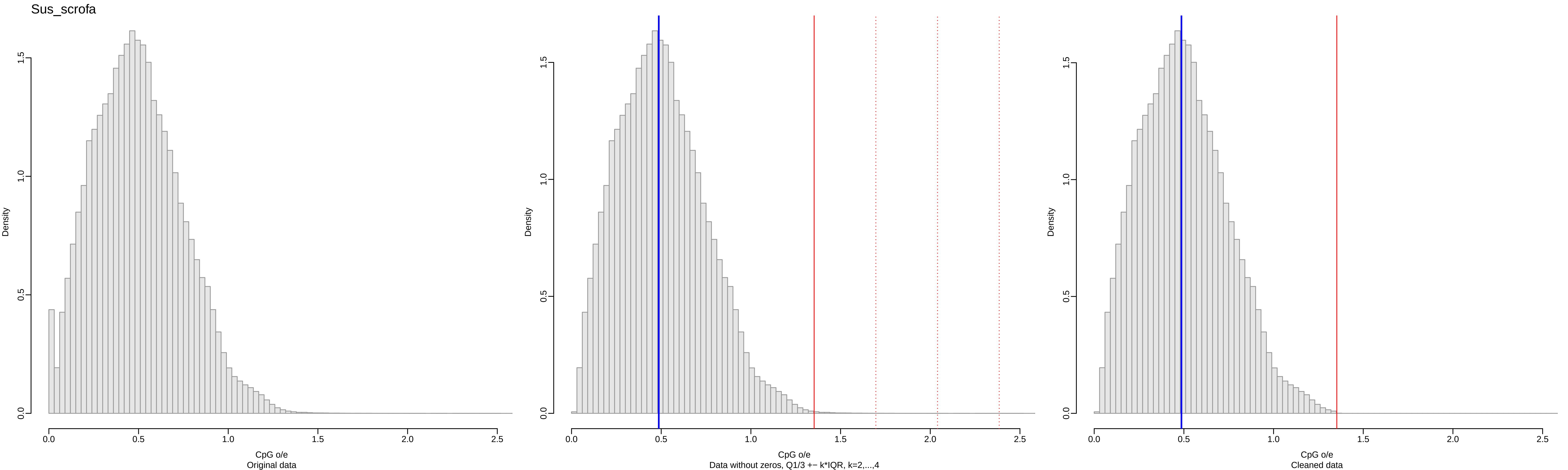

Sus_scrofa
Density
CpG o/e
Original data
Data without zeros, Q1/3 +− k*IQR, k=2,...,4
Cleaned data
0.0
0.5
1.0
1.5
2.0
2.5

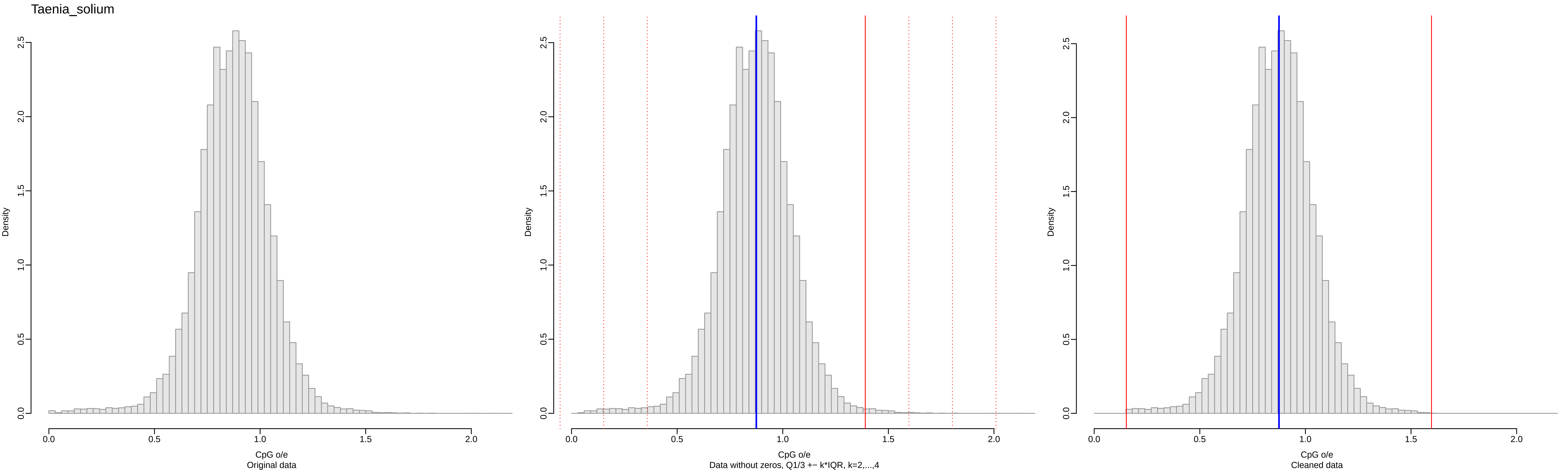

Taenia_solium
Density
CpG o/e
Original data
Data without zeros, Q1/3 +− k*IQR, k=2,...,4
Cleaned data
0.0
0.5
1.0
1.5
2.0
2.5

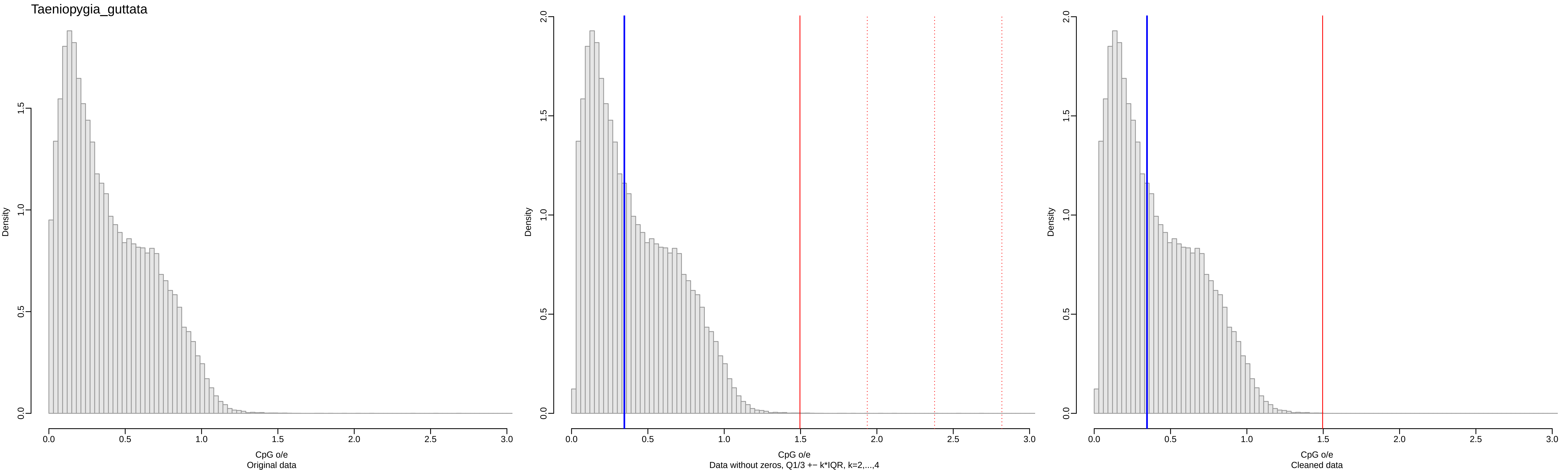
Taeniopygia_guttata
Density
CpG o/e
Original data
CpG o/e
Data without zeros, Q1/3 +− k*IQR, k=2,...,4
CpG o/e
Cleaned data

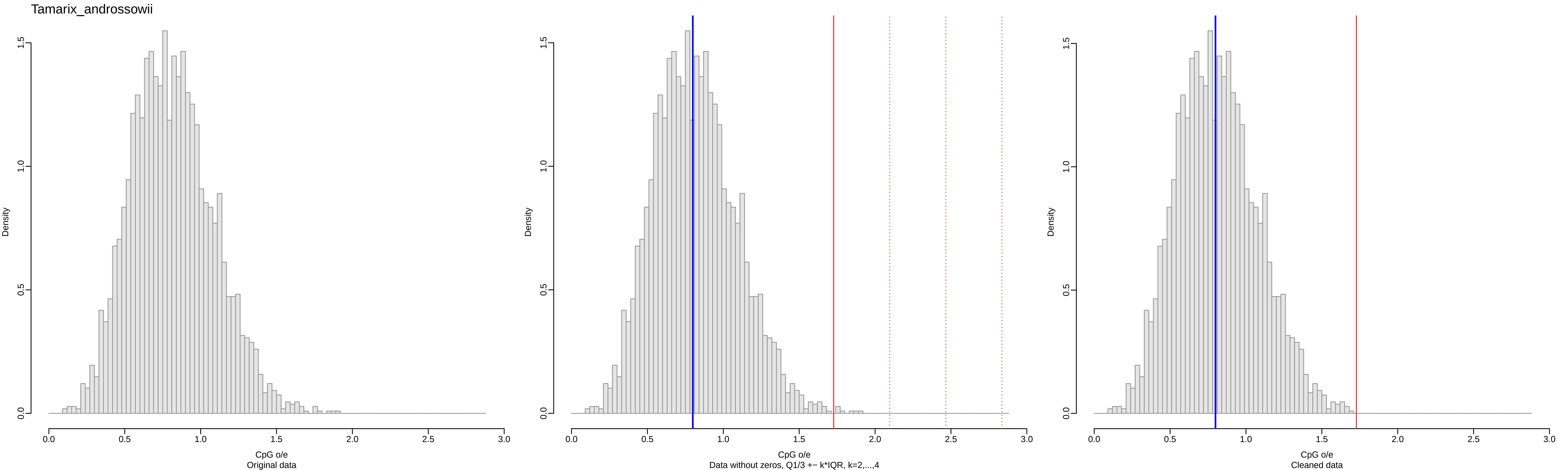

Tamarix_androssowii
Density
CpG o/e
Original data
Data without zeros, Q1/3 +− k*IQR, k=2,...,4
Cleaned data
0.0
0.5
1.0
1.5
2.0
2.5
3.0

Taphrina_deformans

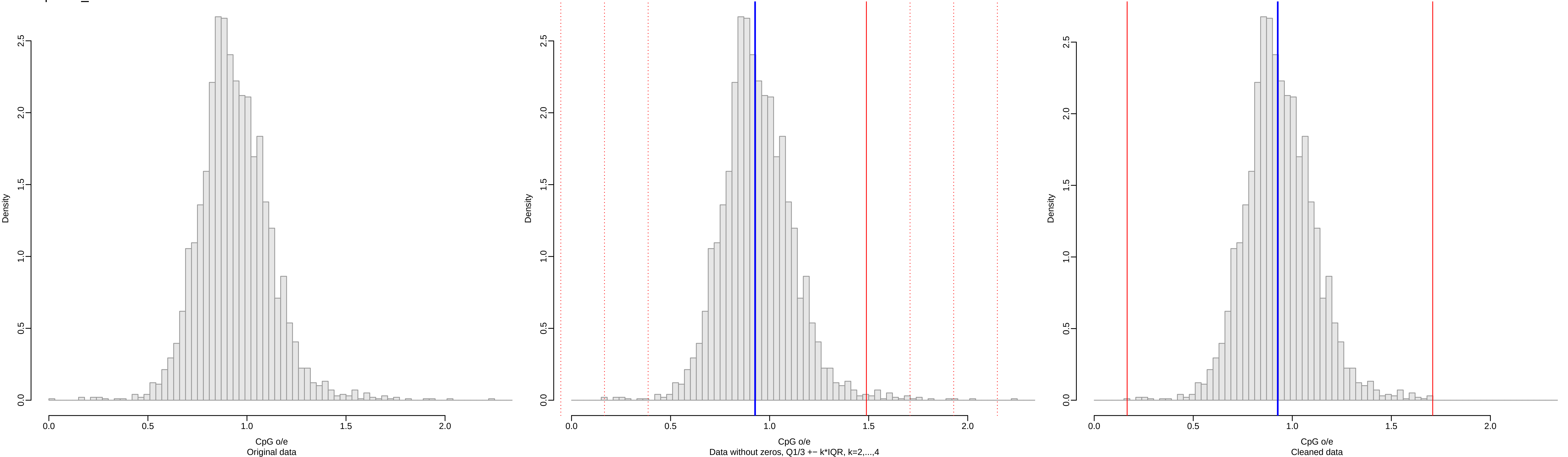

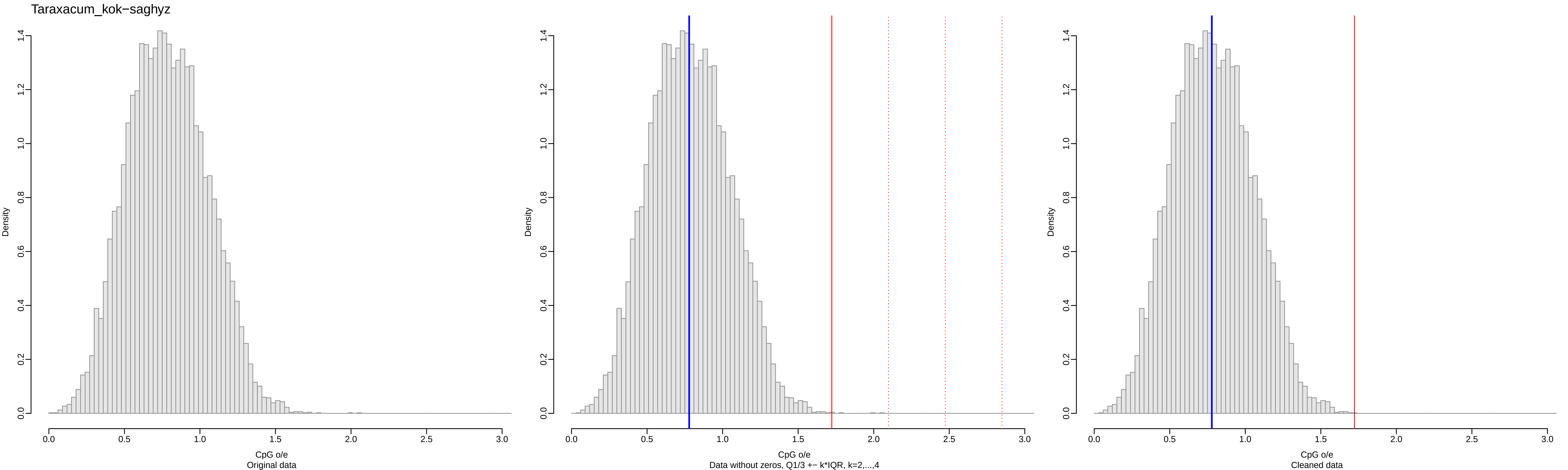
Taraxacum_kok−saghyz
Density
CpG o/e
Original data
CpG o/e
Data without zeros, Q1/3 +− k*IQR, k=2,...,4
CpG o/e
Cleaned data

Taraxacum_officinale

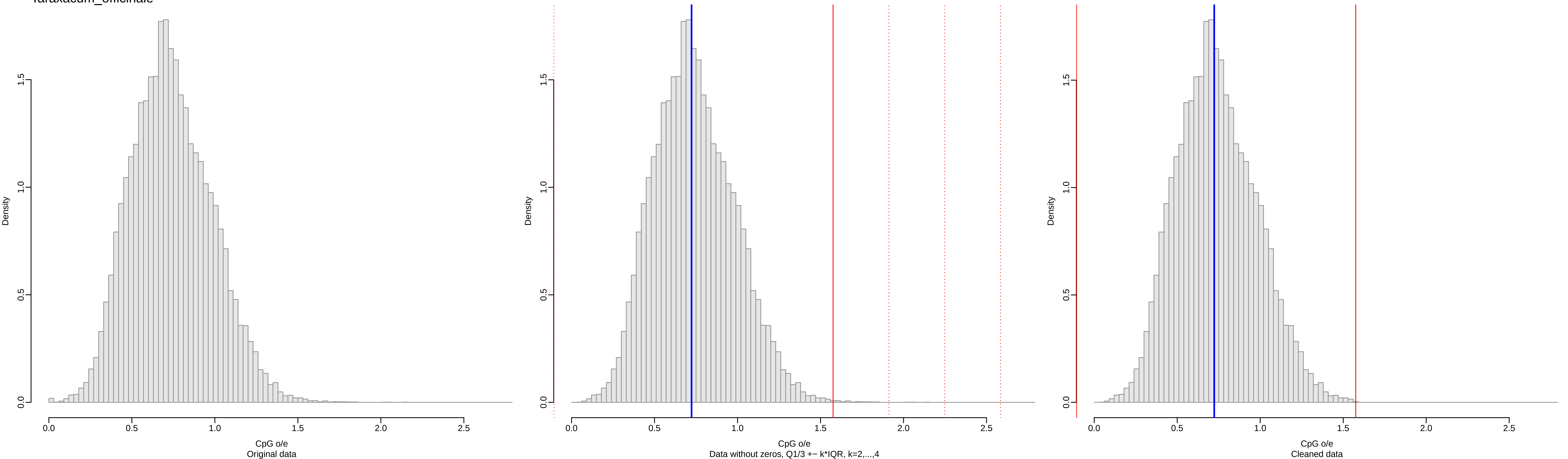

Tegillarca_granosa

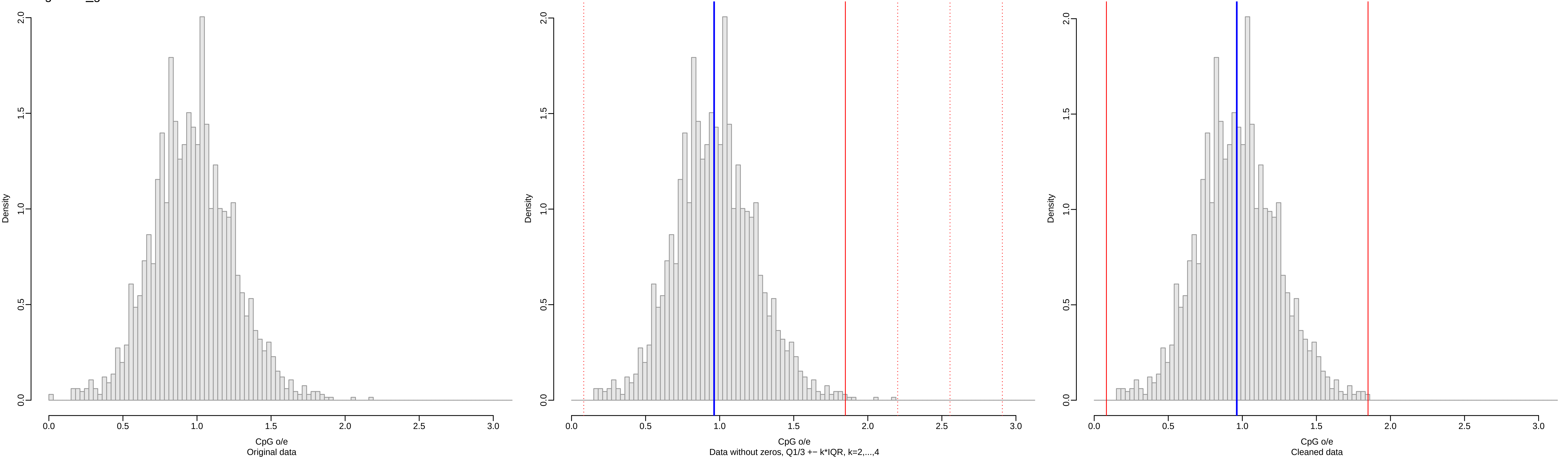

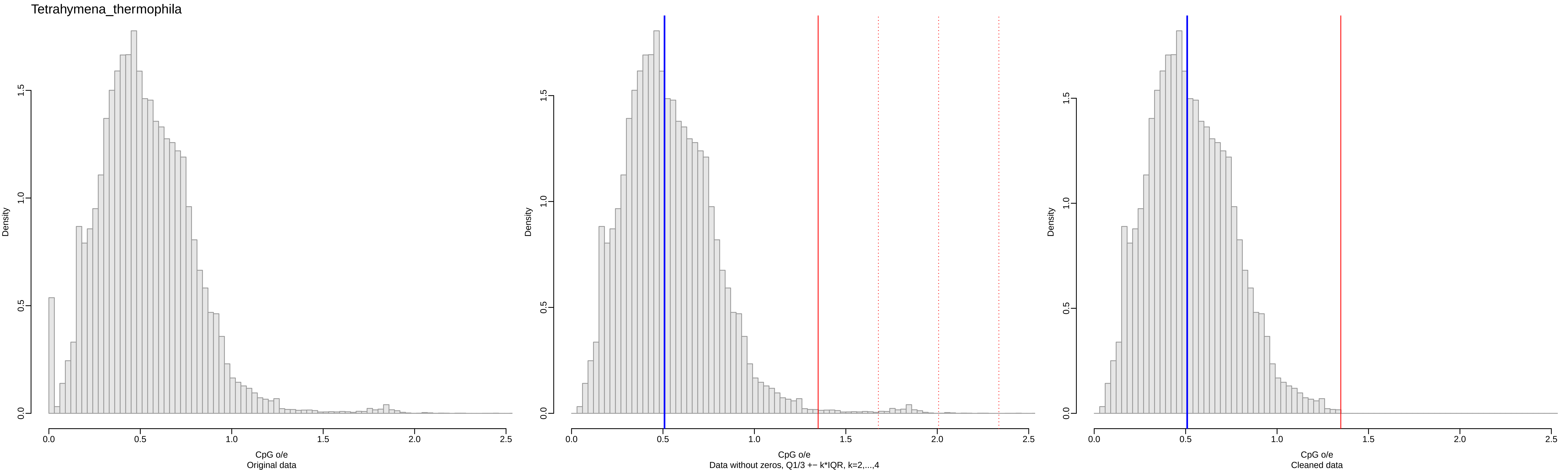

Tetrahymena_thermophila
Density
CpG o/e
Original data
Data without zeros, Q1/3 +− k*IQR, k=2,...,4
Cleaned data
0.0
0.5
1.0
1.5
2.0
2.5

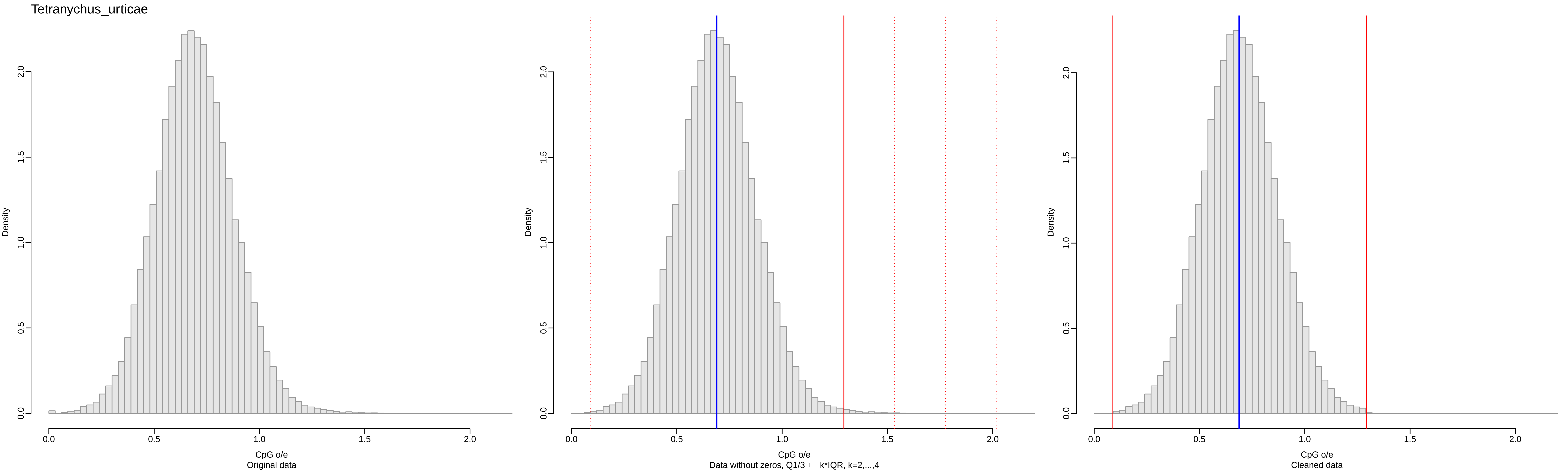
Tetranychus_urticae
Density
CpG o/e
Original data
Data without zeros, Q1/3 +− k*IQR, k=2,...,4
Cleaned data
0.0
0.5
1.0
1.5
2.0

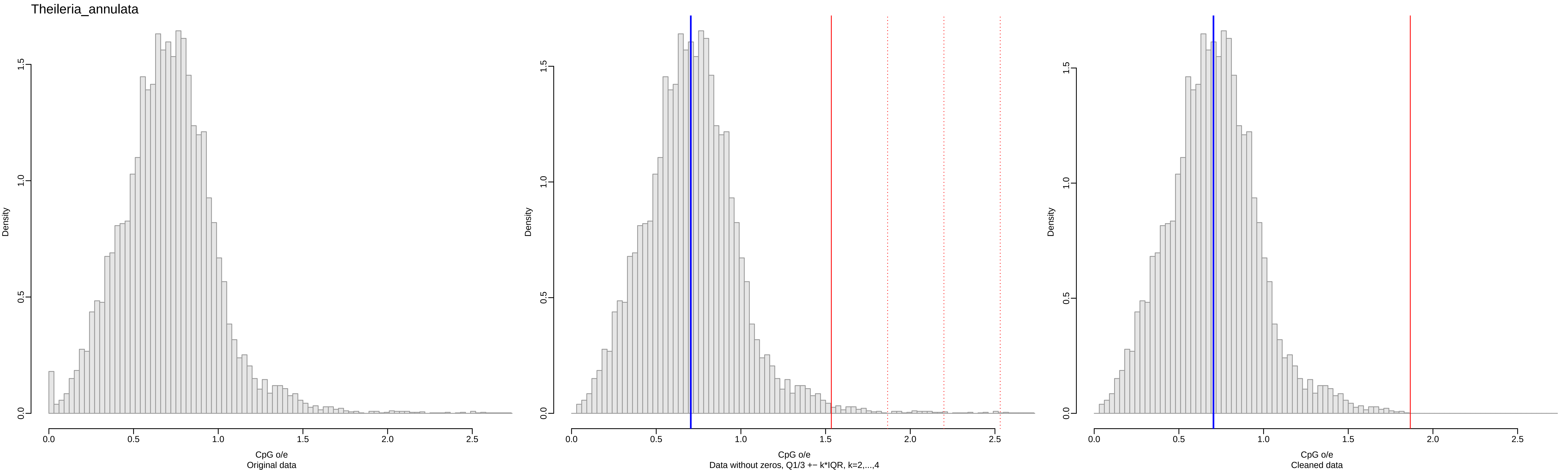

Theileria_annulata
Density
CpG o/e
Original data
Data without zeros, Q1/3 +− k*IQR, k=2,...,4
Cleaned data

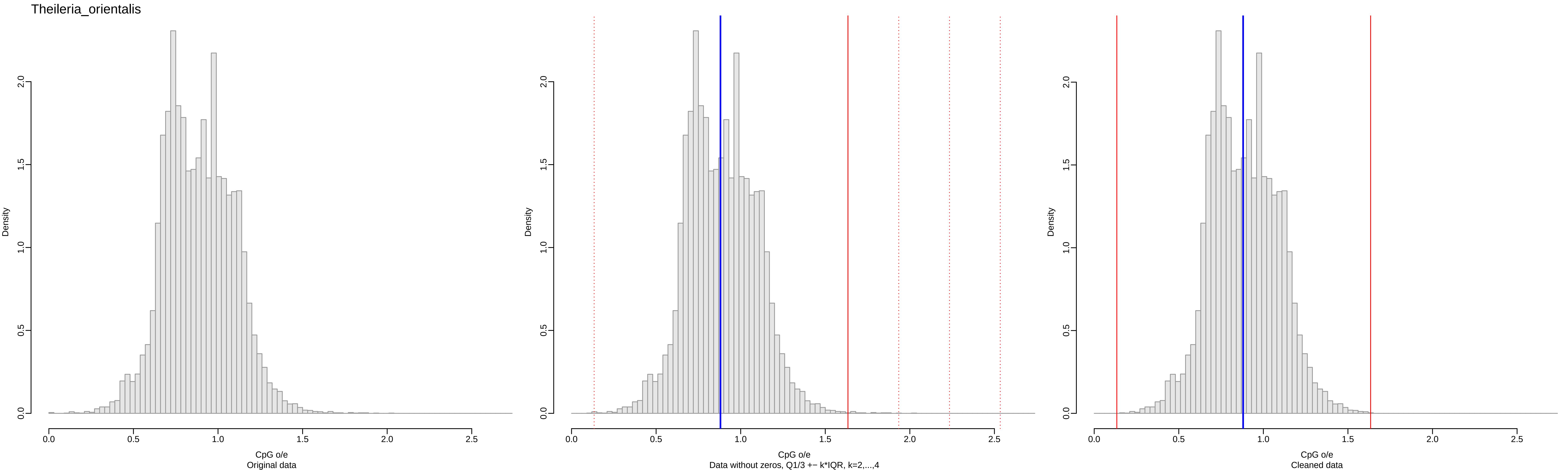
Theileria_orientalis
Density
CpG o/e
Original data
Data without zeros, Q1/3 +− k*IQR, k=2,...,4
Cleaned data
0.0
0.5
1.0
1.5
2.0
2.5

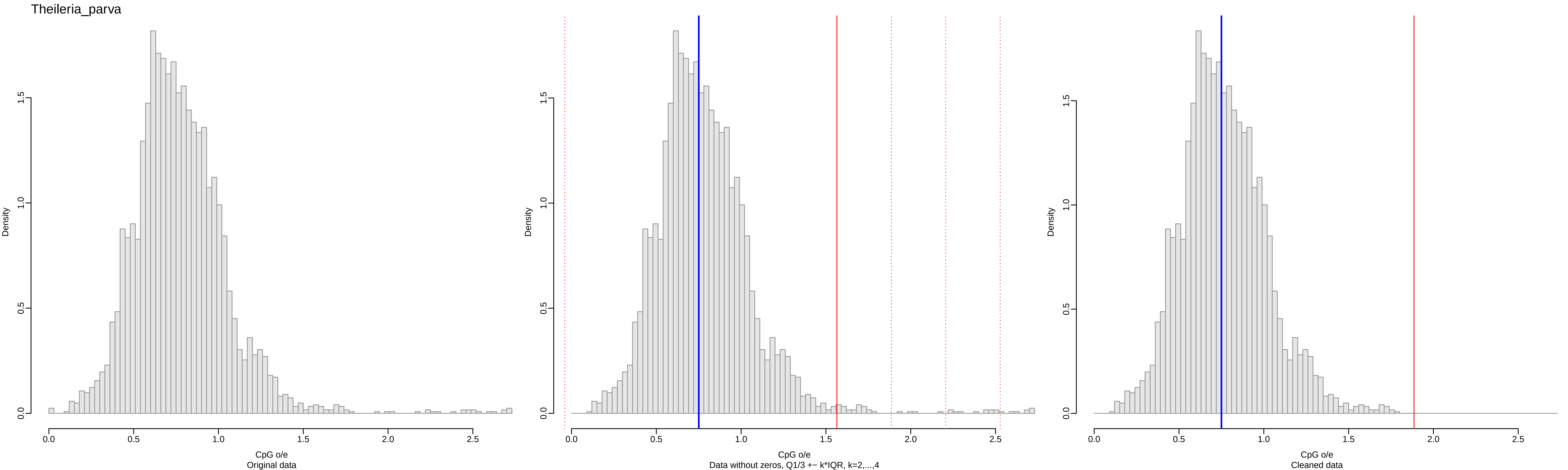
Theileria_parva
Density
CpG o/e
Original data
Data without zeros, Q1/3 +− k*IQR, k=2,...,4
Cleaned data

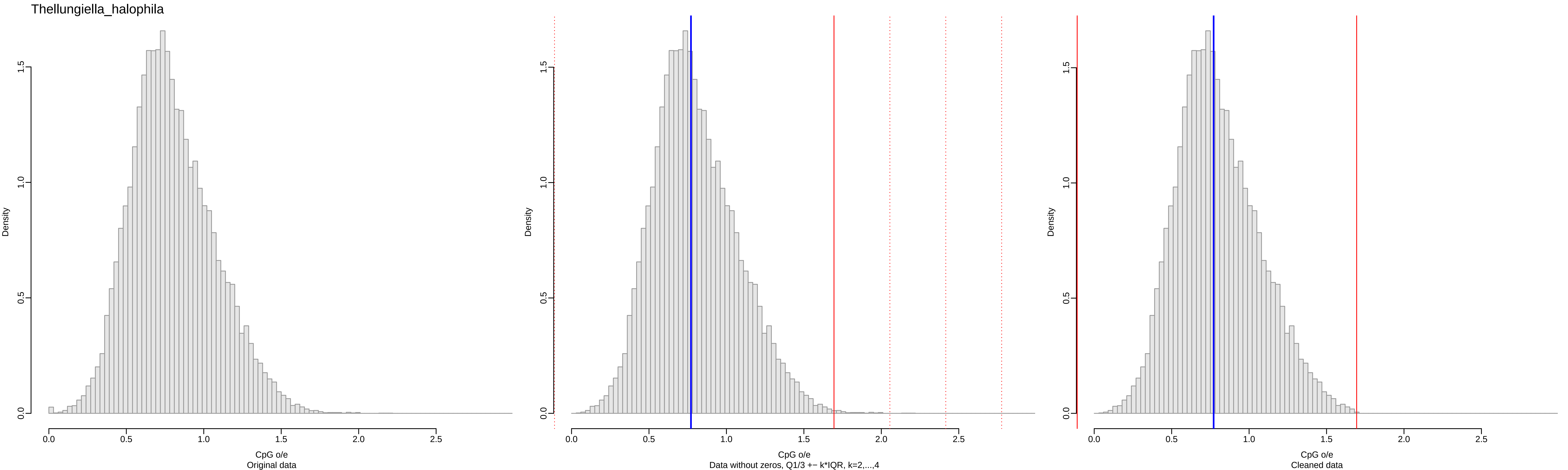
Thellungiella_halophila
Density
CpG o/e
Original data
Data without zeros, Q1/3 +− k*IQR, k=2,...,4
Cleaned data
0.0
0.5
1.0
1.5
2.0
2.5

Thlaspi_caerulescens

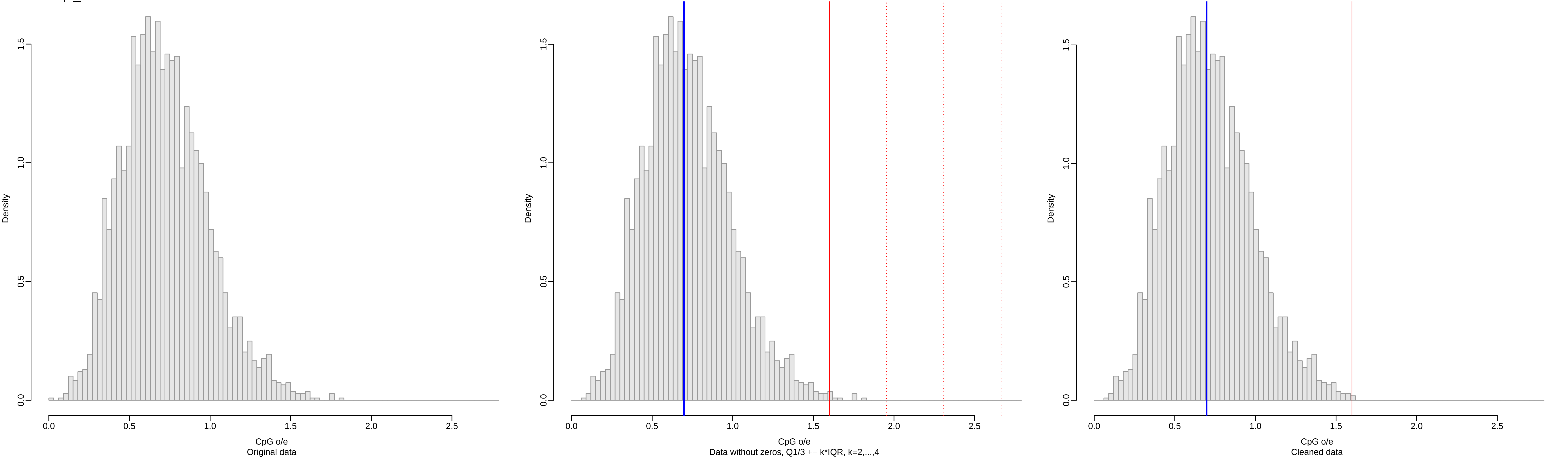

Thunnus_thynnus

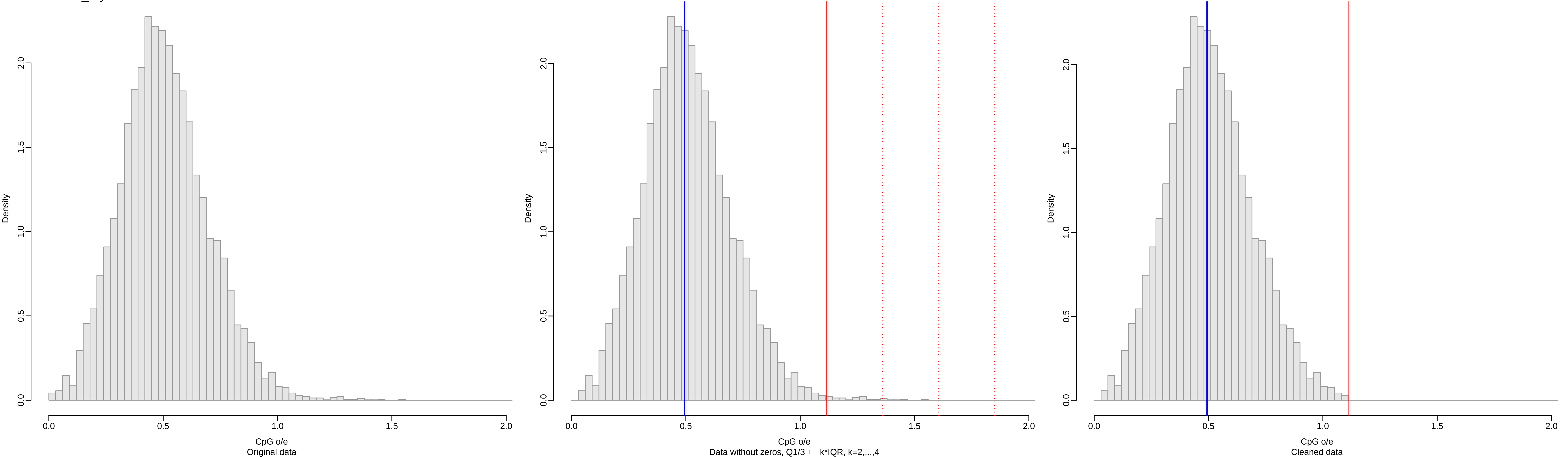

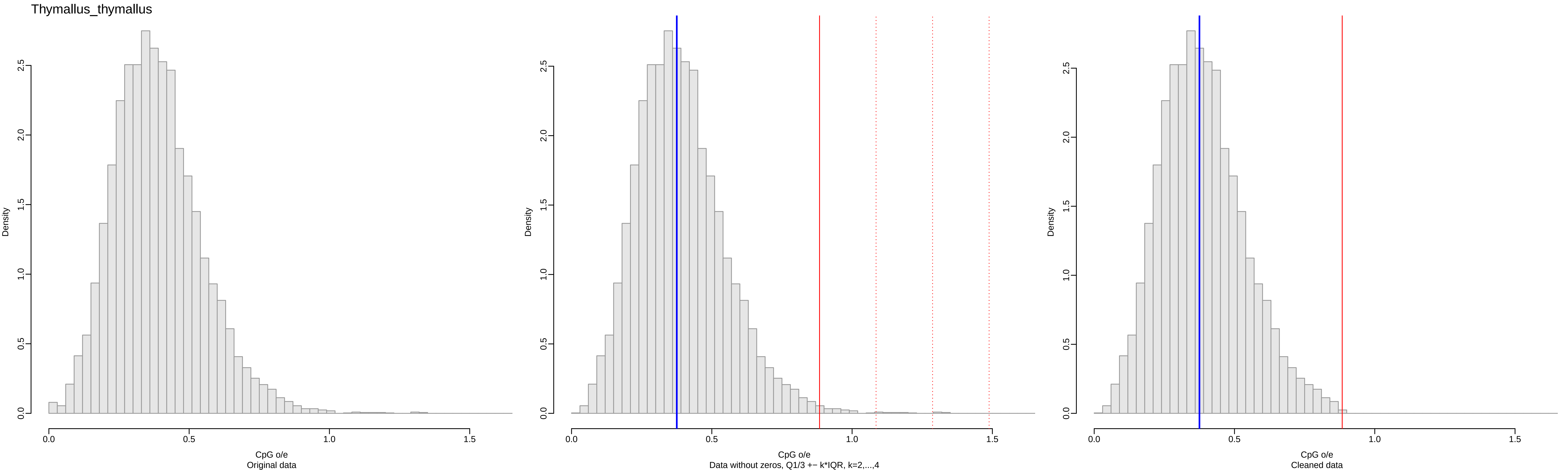
Thymallus_thymallus
Density
CpG o/e
Original data
CpG o/e
Data without zeros, Q1/3 +− k*IQR, k=2,...,4
CpG o/e
Cleaned data

Tigriopus_californicus

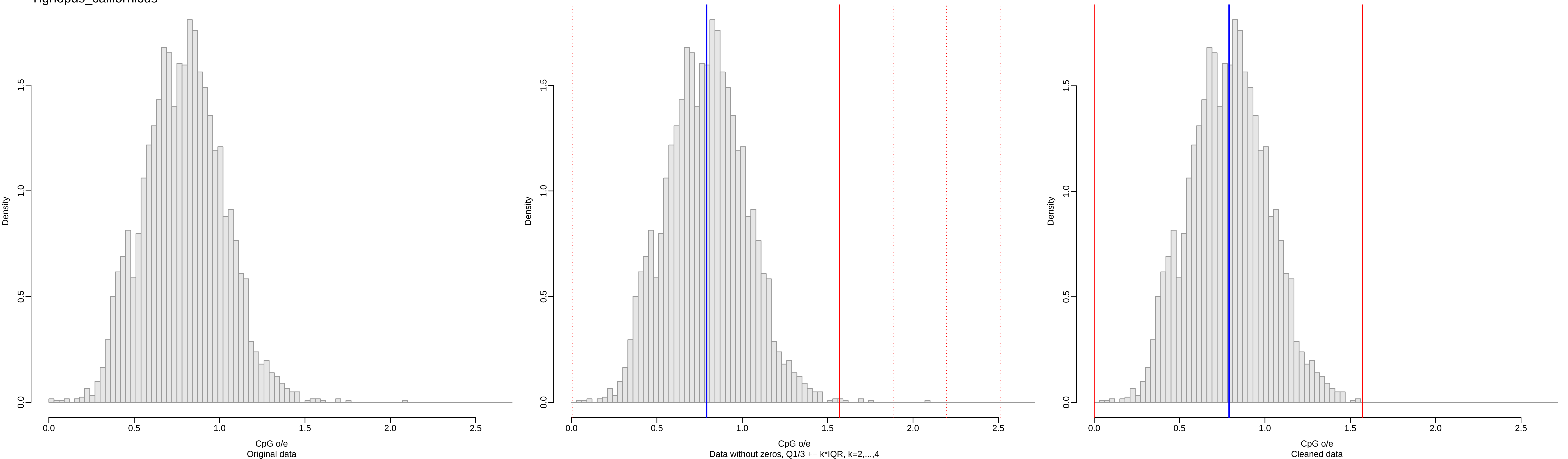

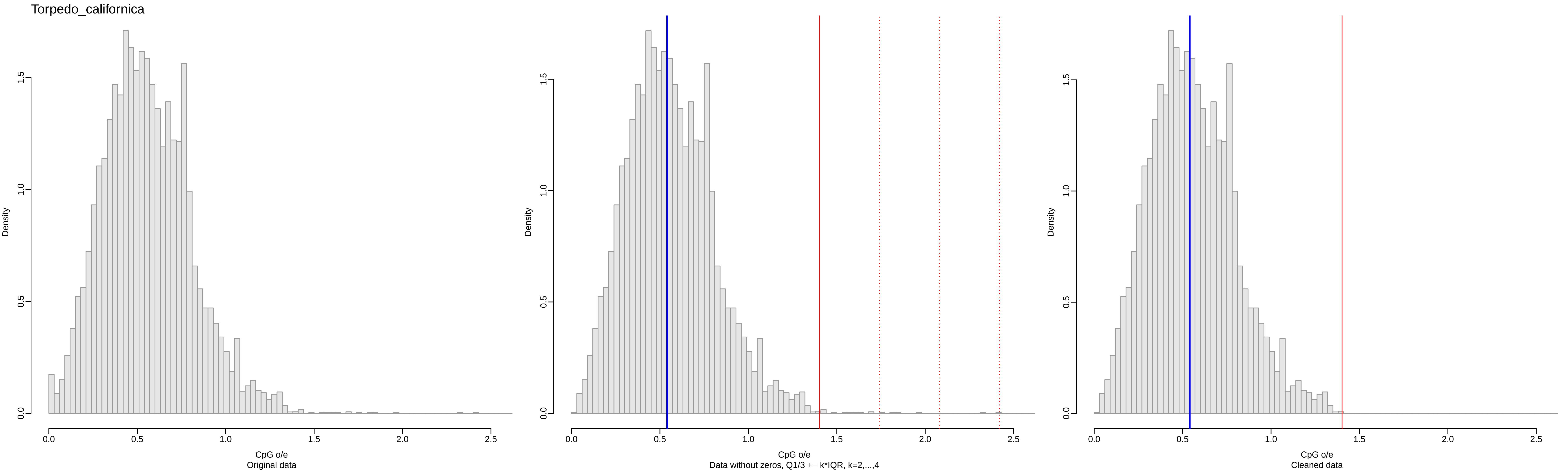

Torpedo_californica
Density
CpG o/e
Original data
Data without zeros, Q1/3 +− k*IQR, k=2,...,4
Cleaned data

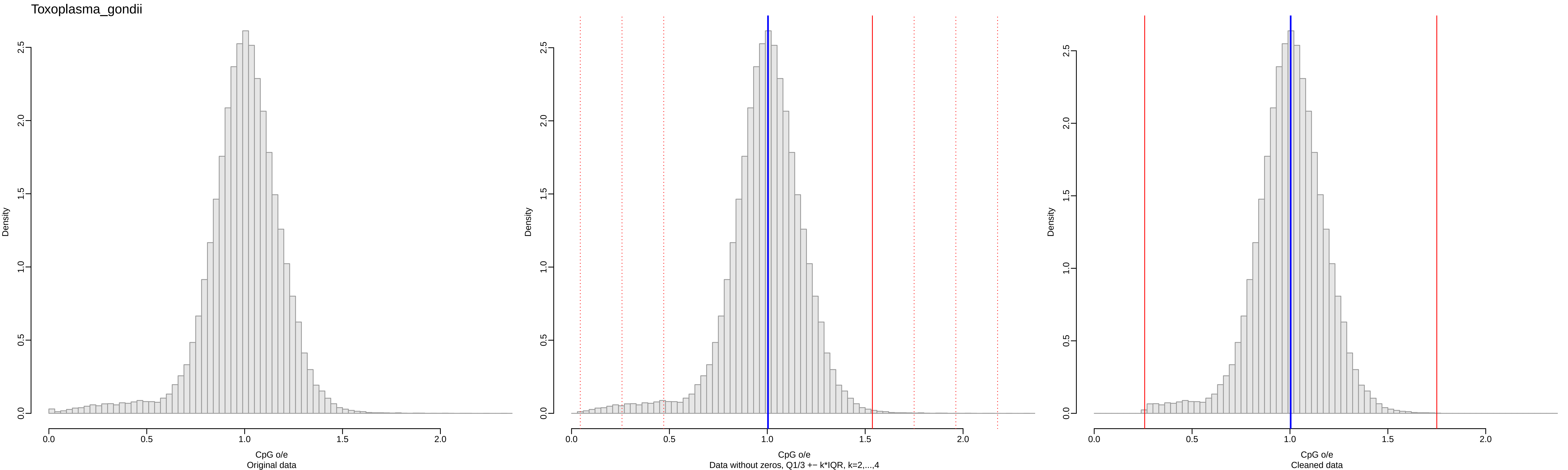

Toxoplasma_gondii
Density
CpG o/e
Original data
Density
CpG o/e
Data without zeros, Q1/3 +− k*IQR, k=2,...,4
Density
CpG o/e
Cleaned data

Toxoptera_citricida

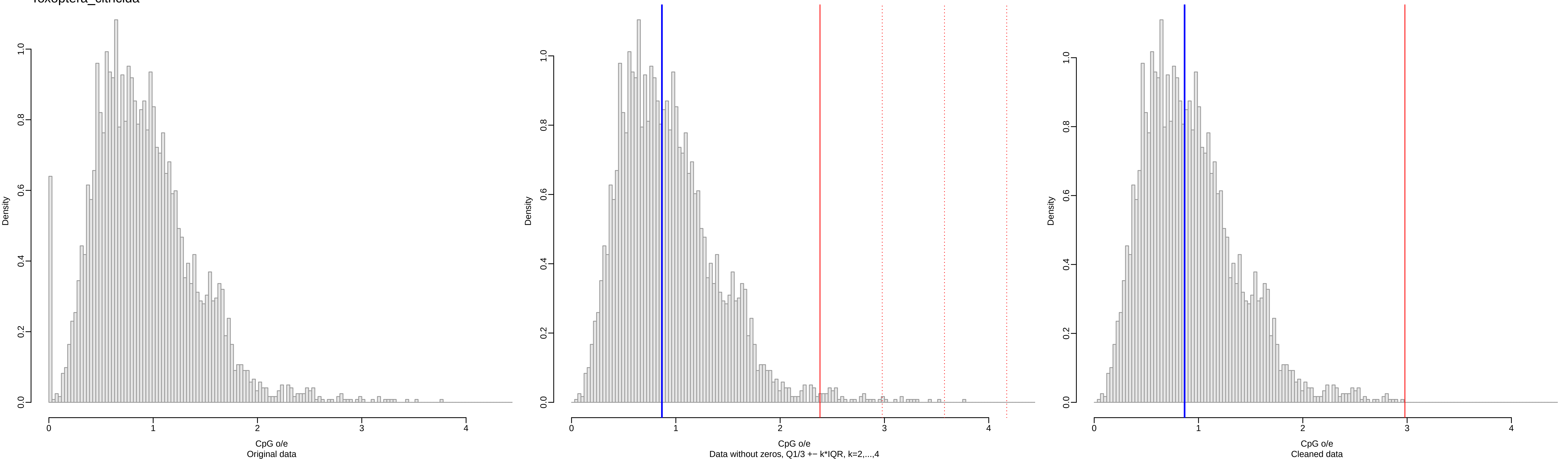

Trachemys_scripta

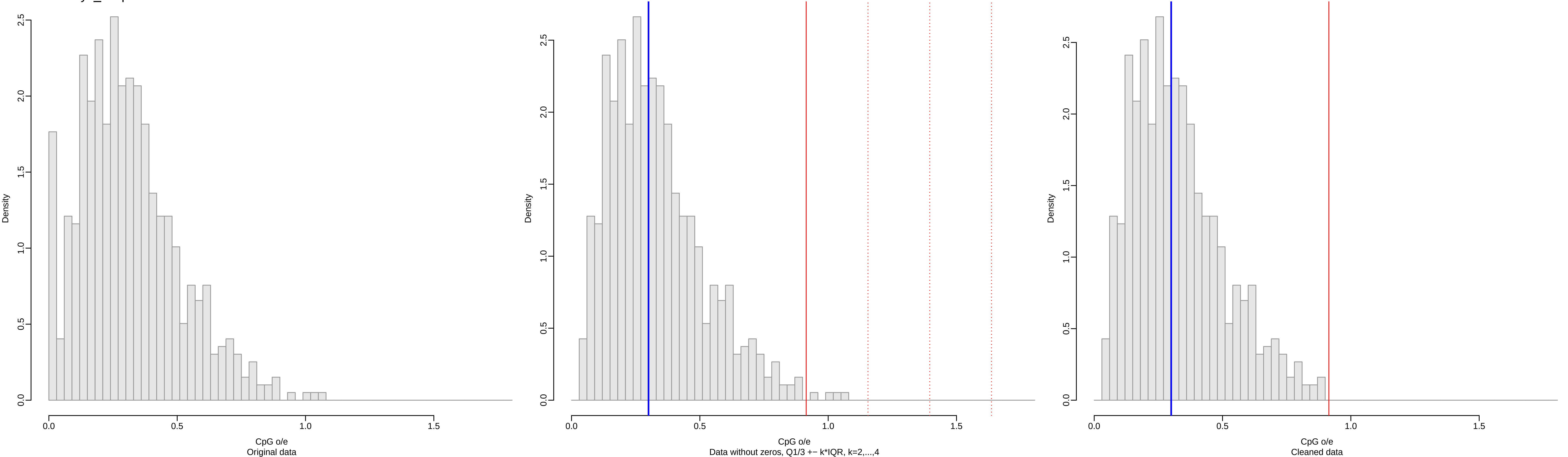

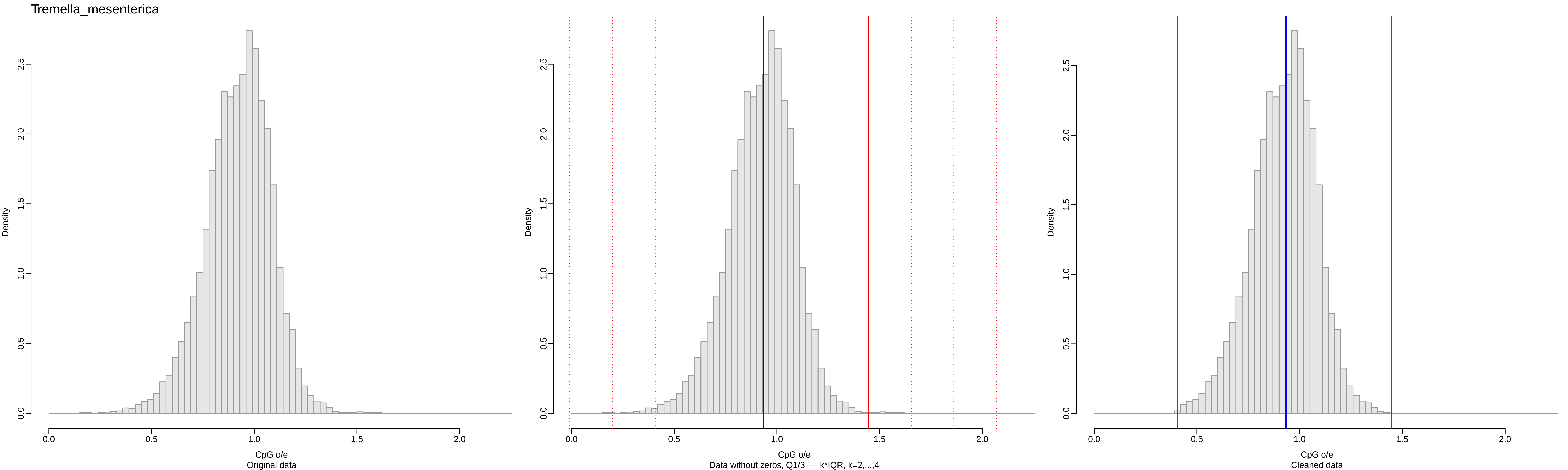

Tremella_mesenterica
Density
CpG o/e
Original data
Data without zeros, Q1/3 +− k*IQR, k=2,...,4
Cleaned data
0.0
0.5
1.0
1.5
2.0
2.5

Triatoma_brasiliensis

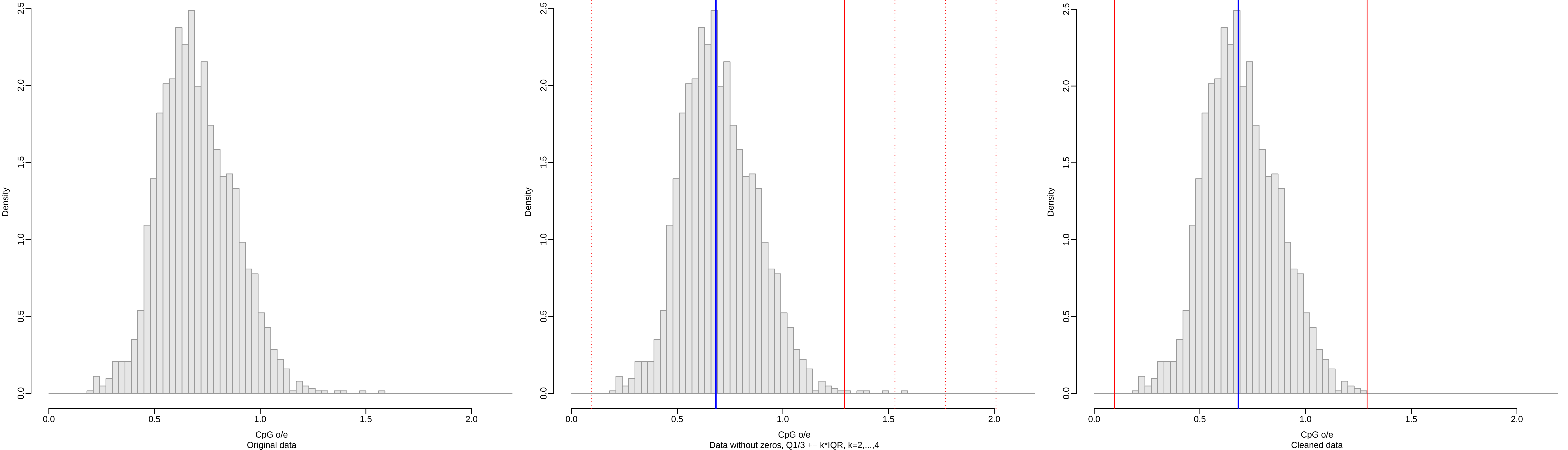

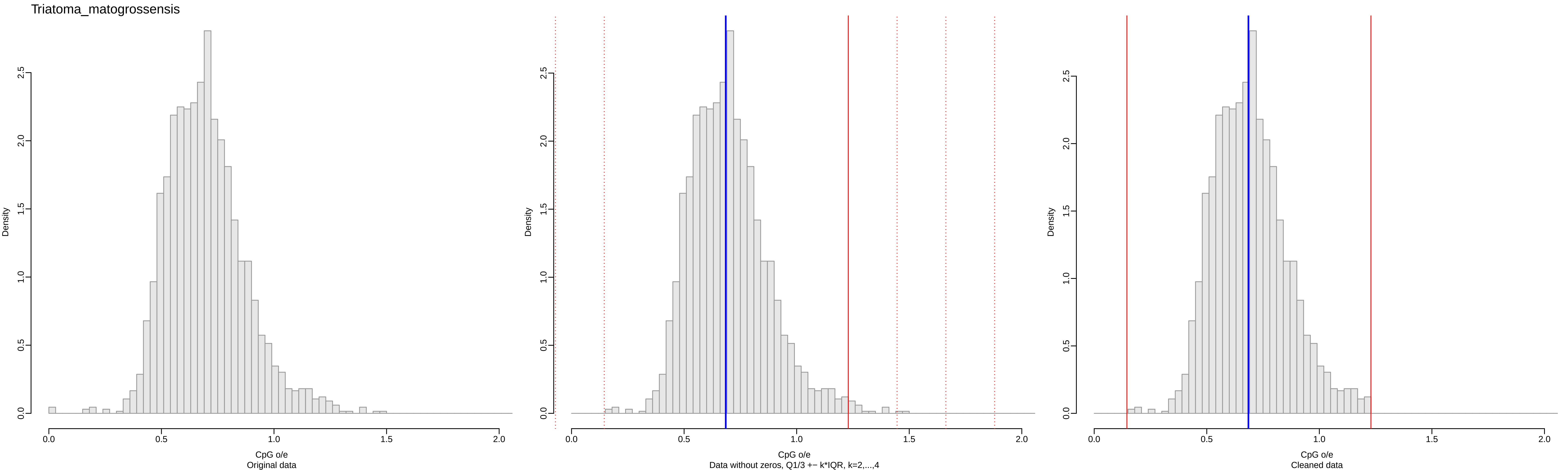

Triatoma_matogrossensis
Density
CpG o/e
Original data
Density
CpG o/e
Data without zeros, Q1/3 +− k*IQR, k=2,...,4
Density
CpG o/e
Cleaned data

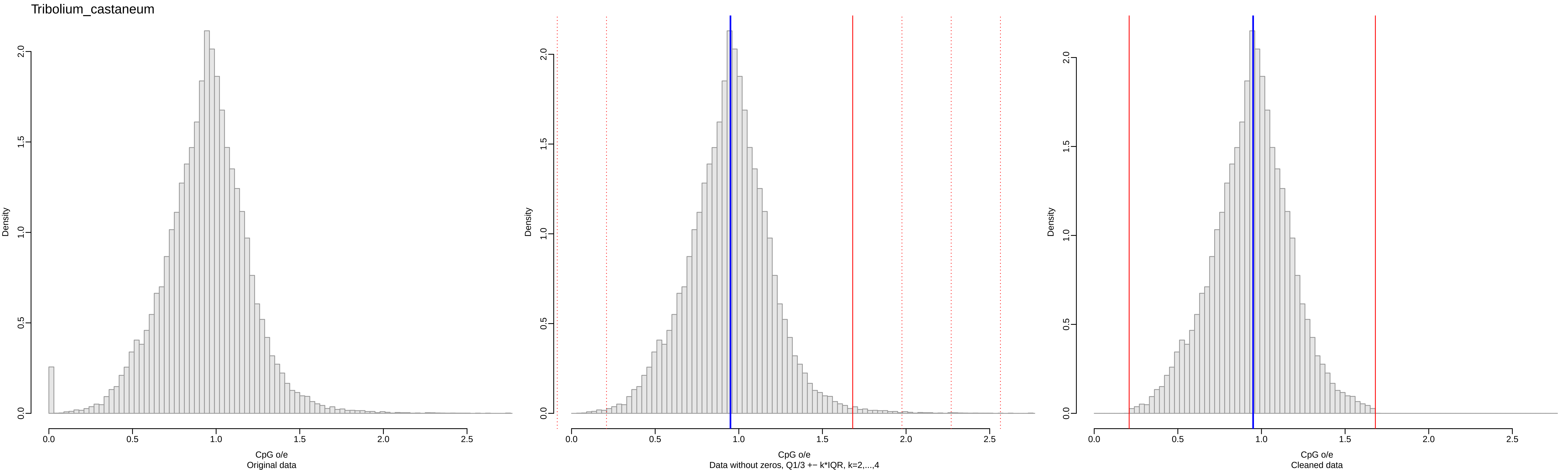

Tribolium_castaneum
Density
CpG o/e
Original data
Data without zeros, Q1/3 +− k*IQR, k=2,...,4
Cleaned data
0.0
0.5
1.0
1.5
2.0
2.5

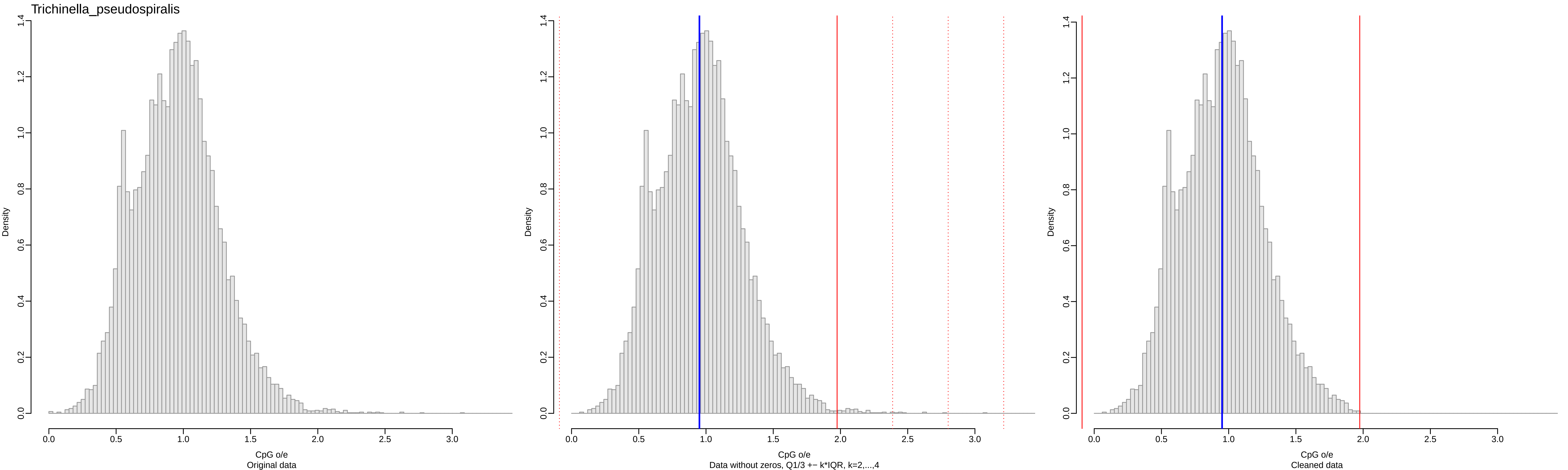

Trichinella_pseudospiralis
Density
CpG o/e
Original data
Density
CpG o/e
Data without zeros, Q1/3 +− k*IQR, k=2,...,4
Density
CpG o/e
Cleaned data

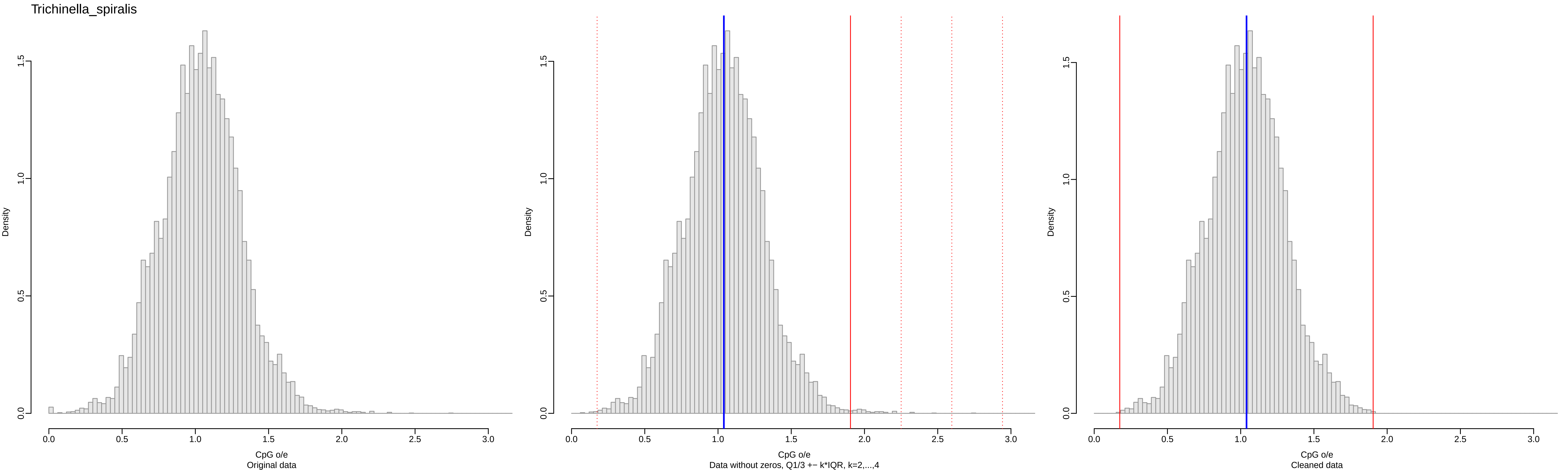

Trichinella_spiralis
Density
0.0
0.5
1.0
1.5
2.0
2.5
3.0
CpG o/e
Original data
Data without zeros, Q1/3 +− k*IQR, k=2,...,4
Cleaned data

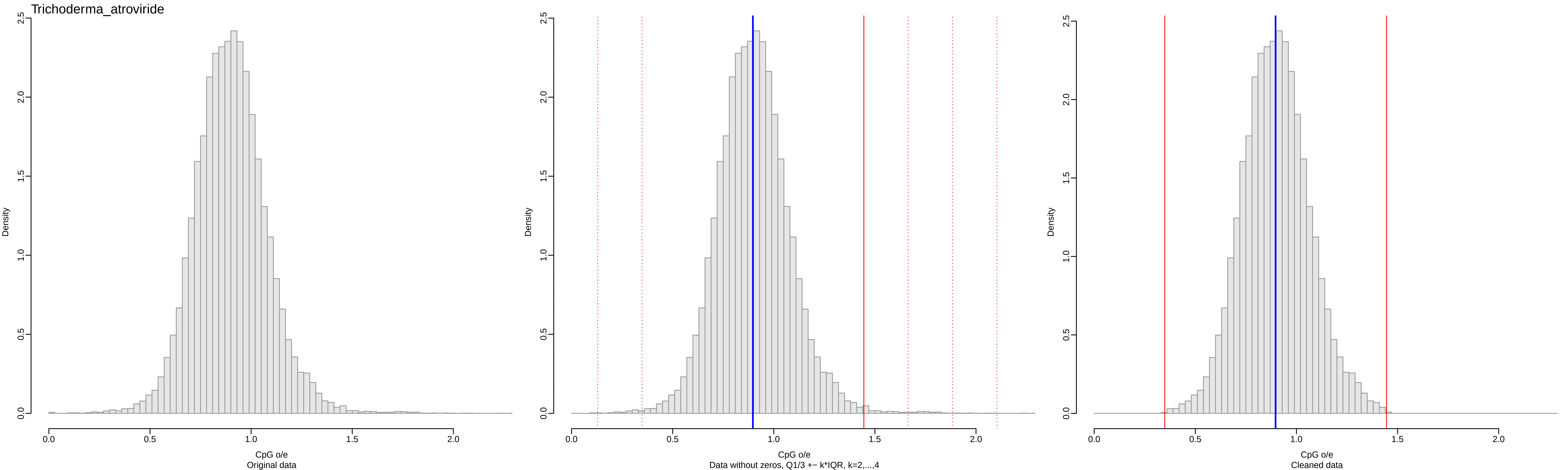
Trichoderma_atroviride
Density
CpG o/e
Original data
Data without zeros, Q1/3 +− k*IQR, k=2,...,4
Cleaned data
0.0
0.5
1.0
1.5
2.0
2.5

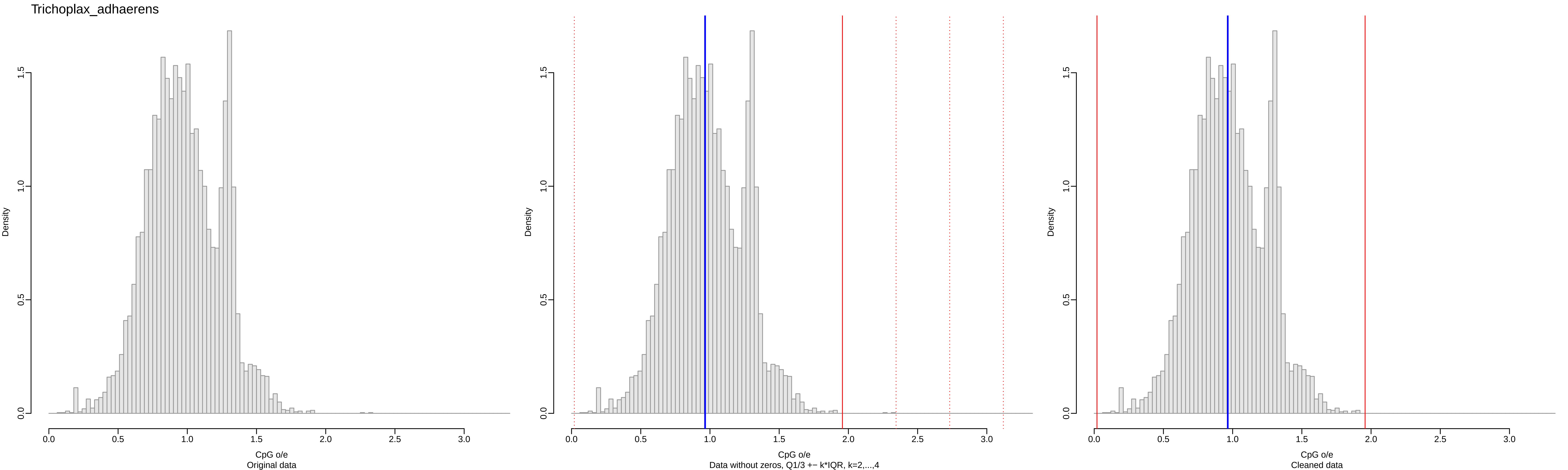
Trichoplax_adhaerens
Density
CpG o/e
Original data
Data without zeros, Q1/3 +− k*IQR, k=2,...,4
Cleaned data
0.0
0.5
1.0
1.5
2.0
2.5
3.0

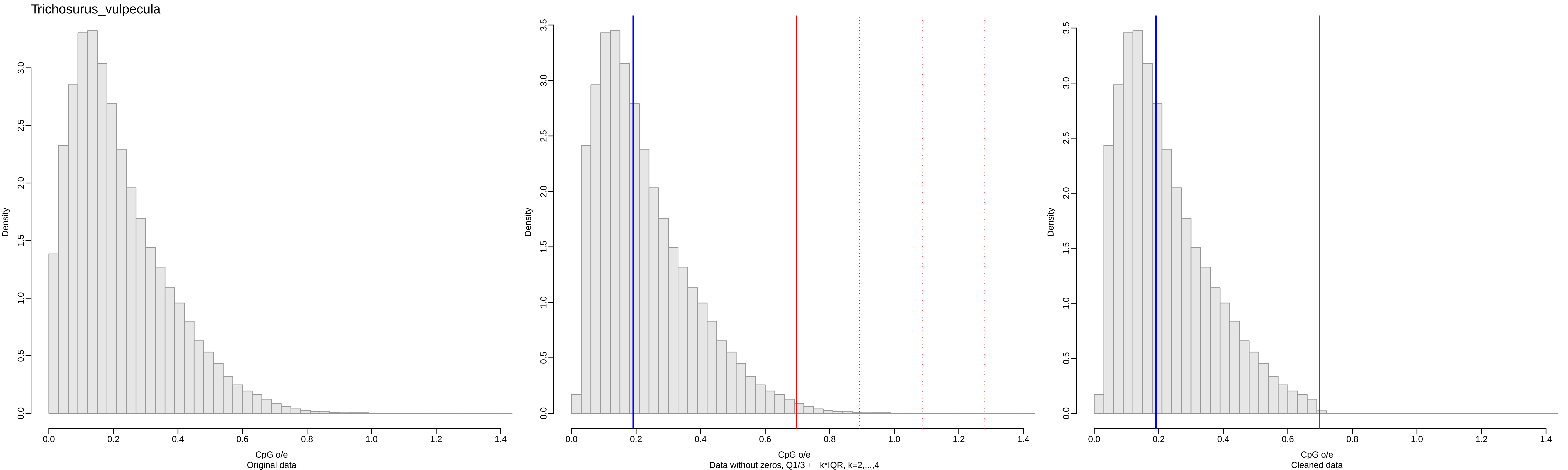

Trichosurus_vulpecula
Density
CpG o/e
Original data
Data without zeros, Q1/3 +− k*IQR, k=2,...,4
Cleaned data
0.0
0.5
1.0
1.5
2.0
2.5
3.0
3.5
0.2
0.4
0.6
0.8
1.0
1.2
1.4

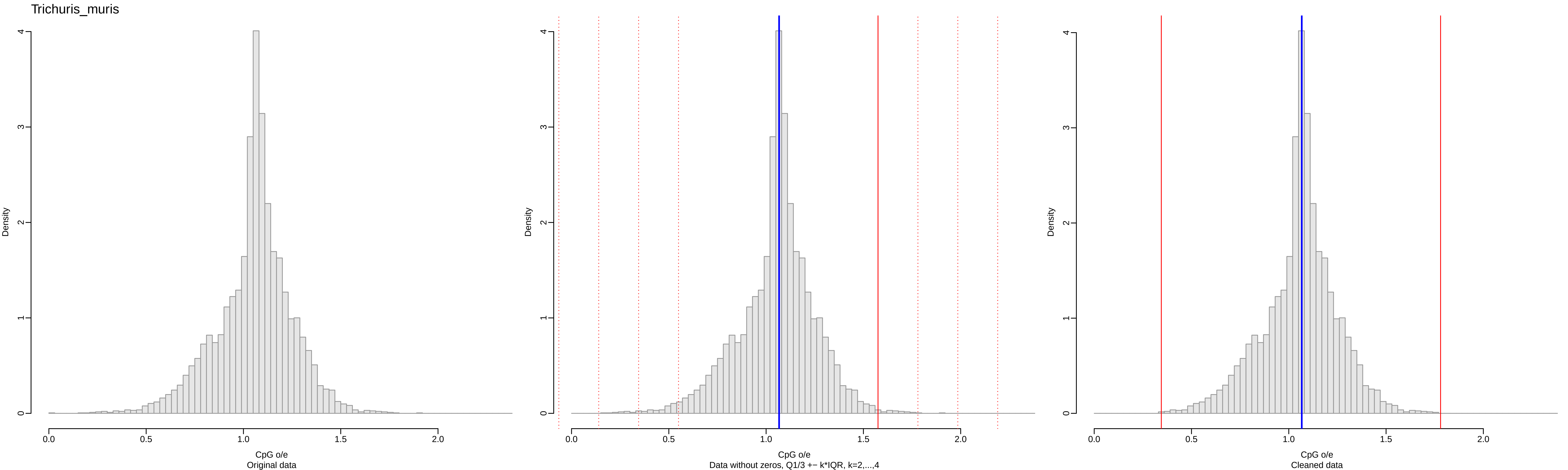
Trichuris_muris
Density
CpG o/e
Original data
Data without zeros, Q1/3 +− k*IQR, k=2,...,4
Cleaned data
0.0
0.5
1.0
1.5
2.0
0
1
2
3
4

Trichuris_vulpis

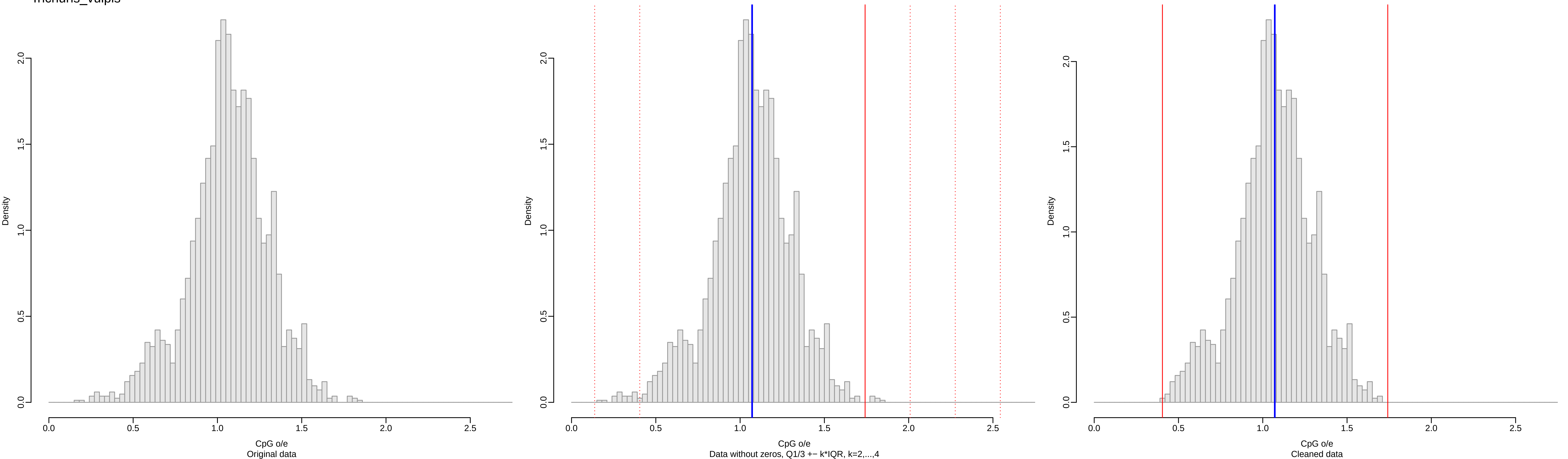

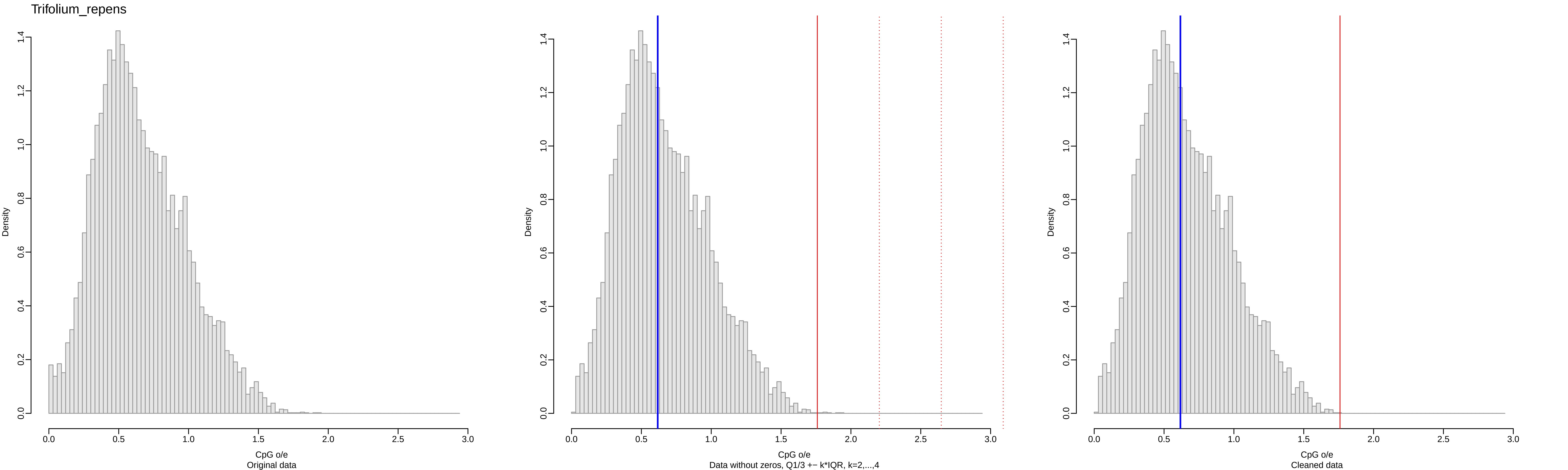
Trifolium_repens
Density
CpG o/e
Original data
Data without zeros, Q1/3 +− k*IQR, k=2,...,4
Cleaned data
0.0
0.2
0.4
0.6
0.8
1.0
1.2
1.4
0.5
1.5
2.0
2.5
3.0

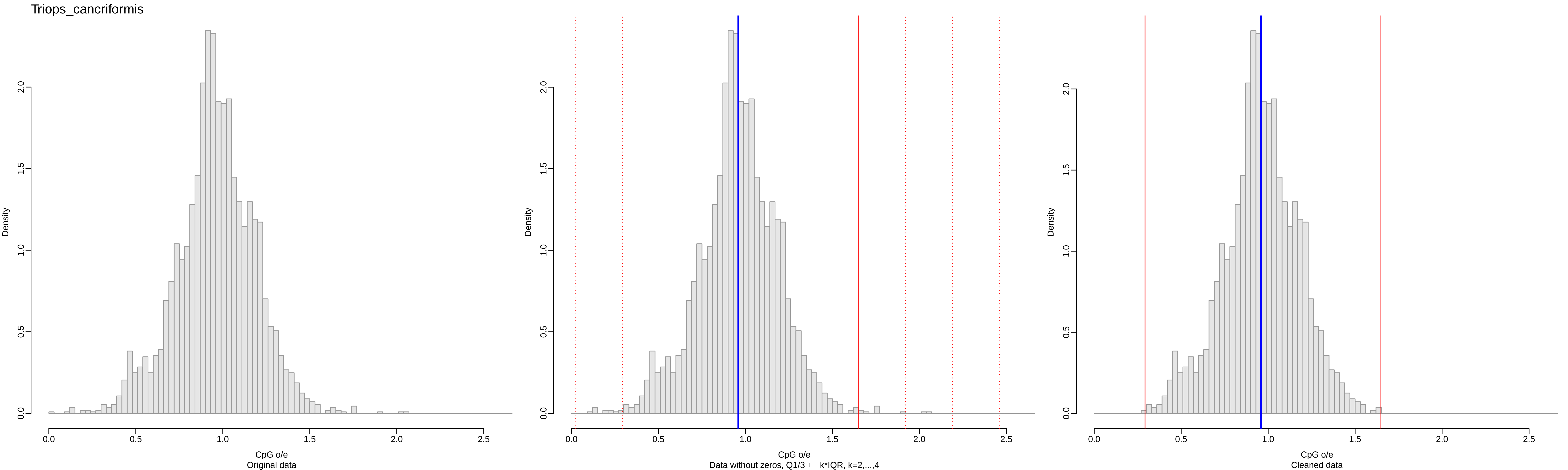

Triops_cancriformis
Density
CpG o/e
Original data
Data without zeros, Q1/3 +− k*IQR, k=2,...,4
Cleaned data

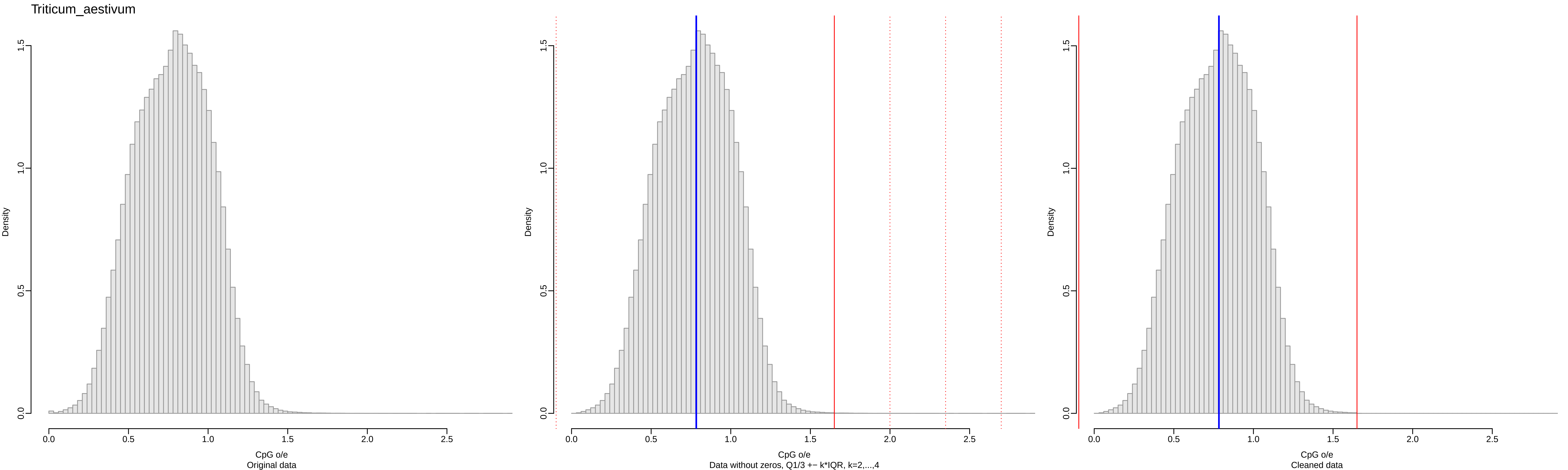
Triticum_aestivum
Density
CpG o/e
Original data
Data without zeros, Q1/3 +− k*IQR, k=2,...,4
Cleaned data
0.0
0.5
1.0
1.5
2.0
2.5

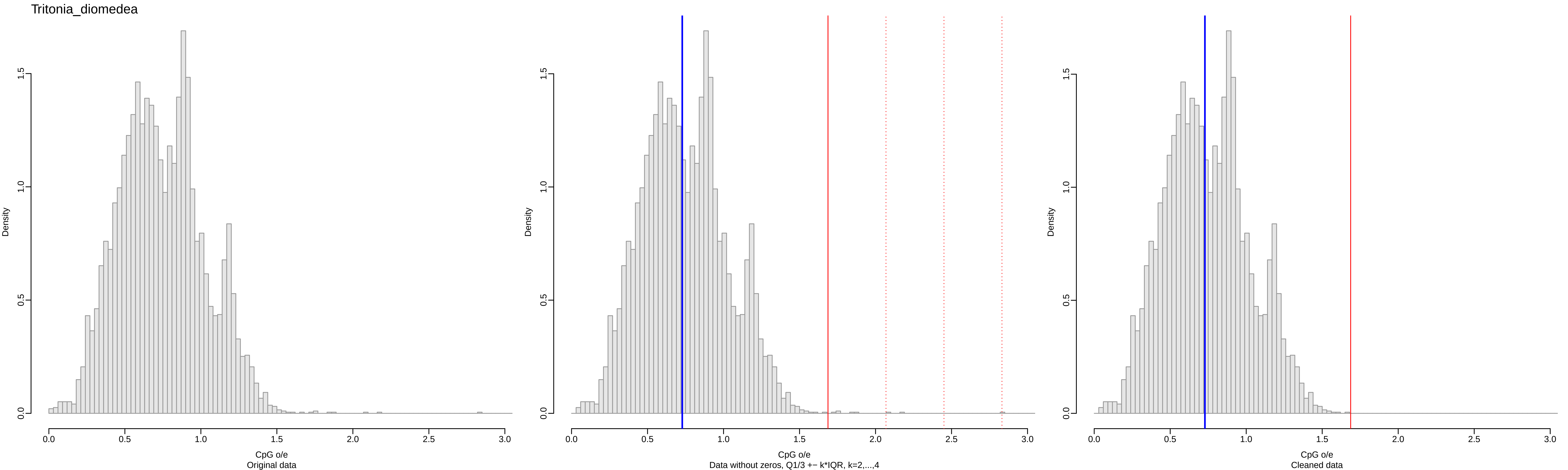

Tritonia_diomedea
Density
CpG o/e
Original data
Data without zeros, Q1/3 +− k*IQR, k=2,...,4
Cleaned data

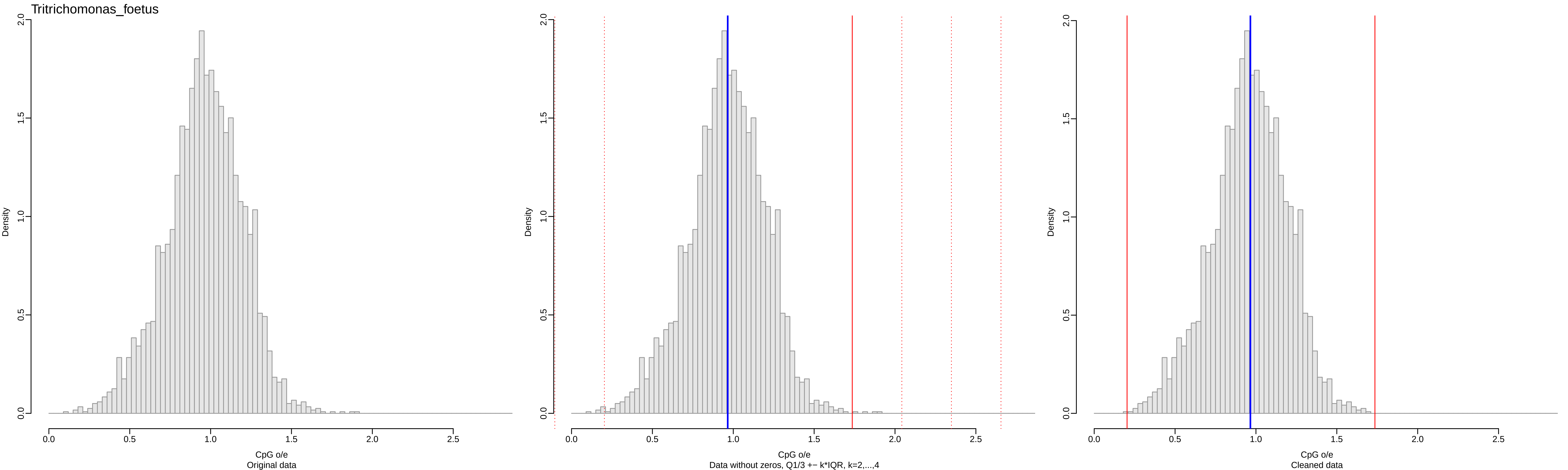

Tritrichomonas_foetus
Density
CpG o/e
Original data
Density
CpG o/e
Data without zeros, Q1/3 +− k*IQR, k=2,...,4
Density
CpG o/e
Cleaned data

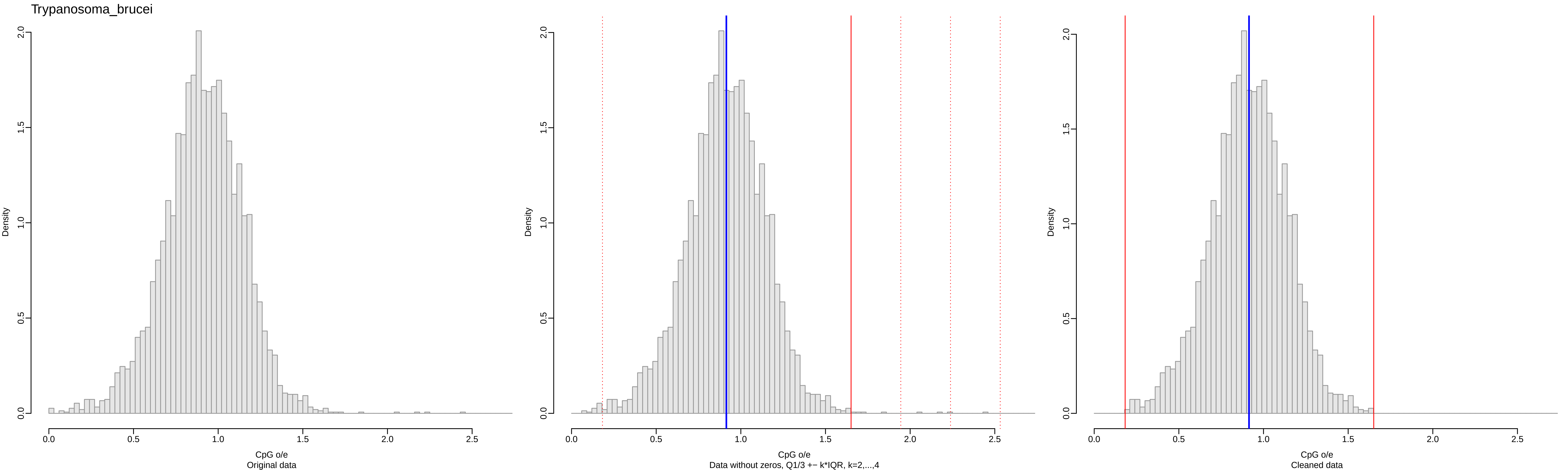

Trypanosoma_brucei
Density
CpG o/e
Original data
Data without zeros, Q1/3 +− k*IQR, k=2,...,4
Cleaned data
0.0
0.5
1.0
1.5
2.0
2.5

Trypanosoma_cruzi

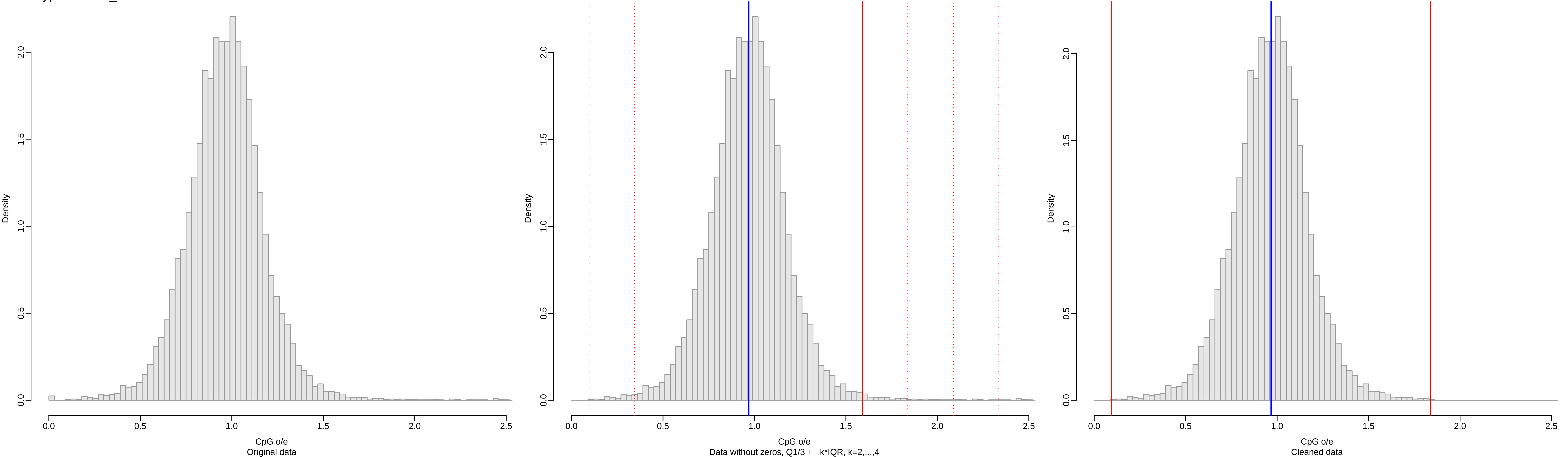

Trypanosoma_rangeli

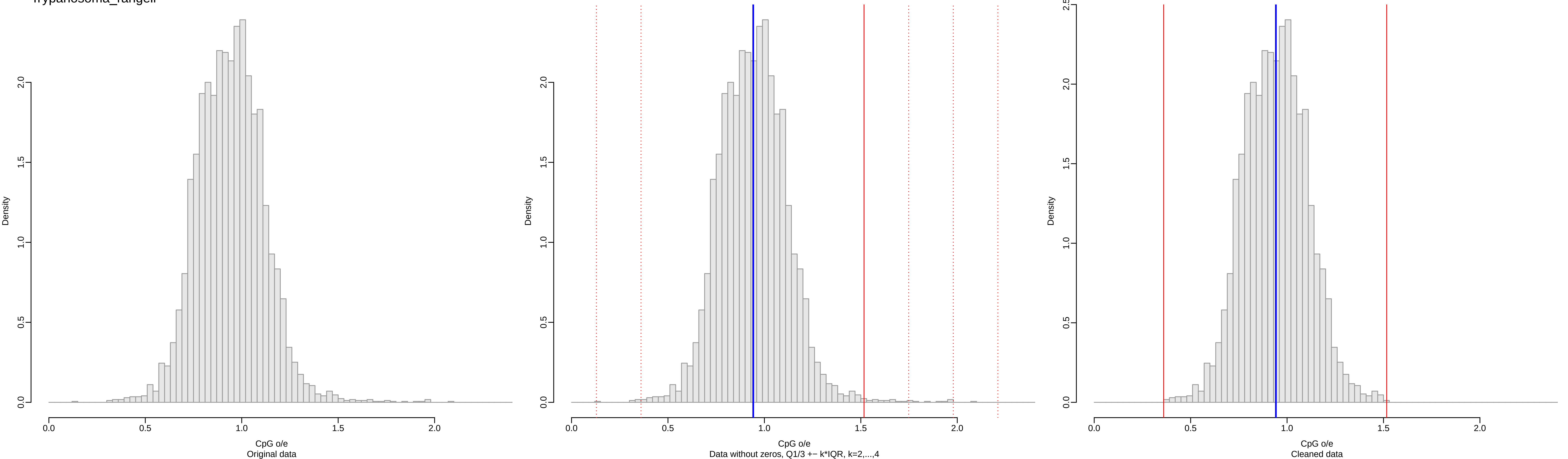

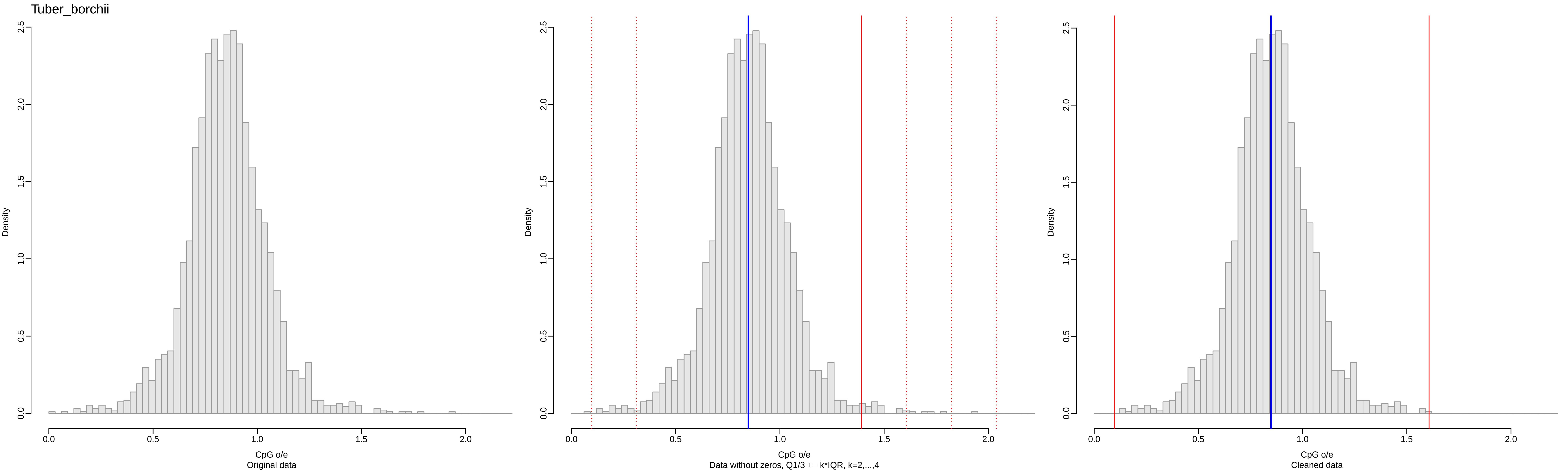

Tuber_borchii
Density
CpG o/e
Original data
Data without zeros, Q1/3 +− k*IQR, k=2,...,4
Cleaned data
0.0
0.5
1.0
1.5
2.0
2.5

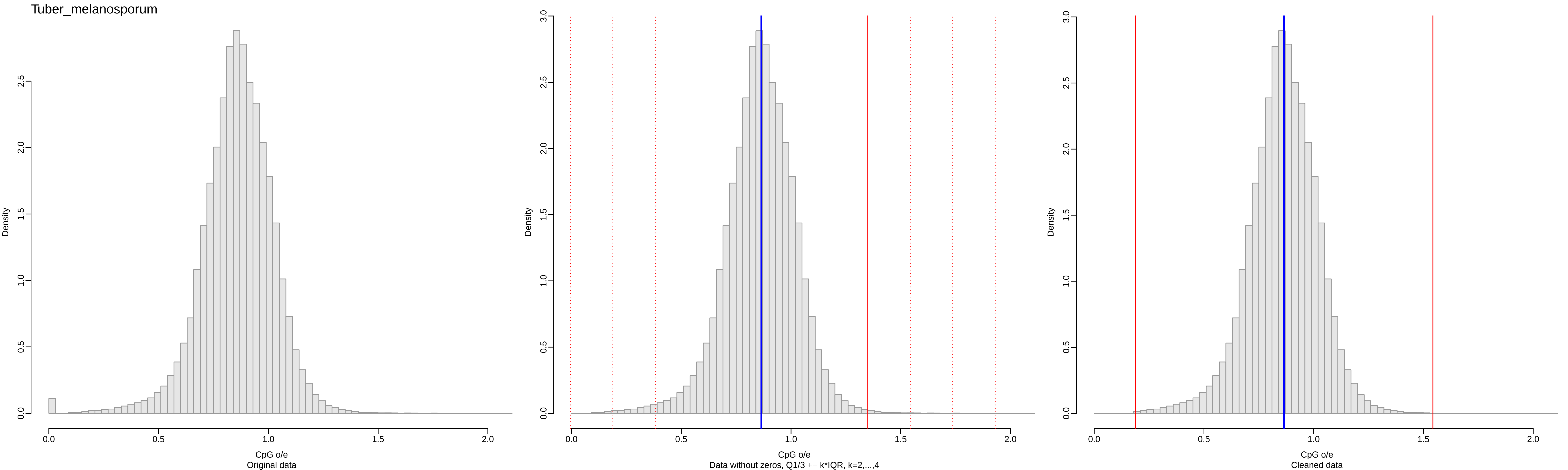

Tuber_melanosporum
Density
CpG o/e
Original data
Density
CpG o/e
Data without zeros, Q1/3 +− k*IQR, k=2,...,4
Density
CpG o/e
Cleaned data

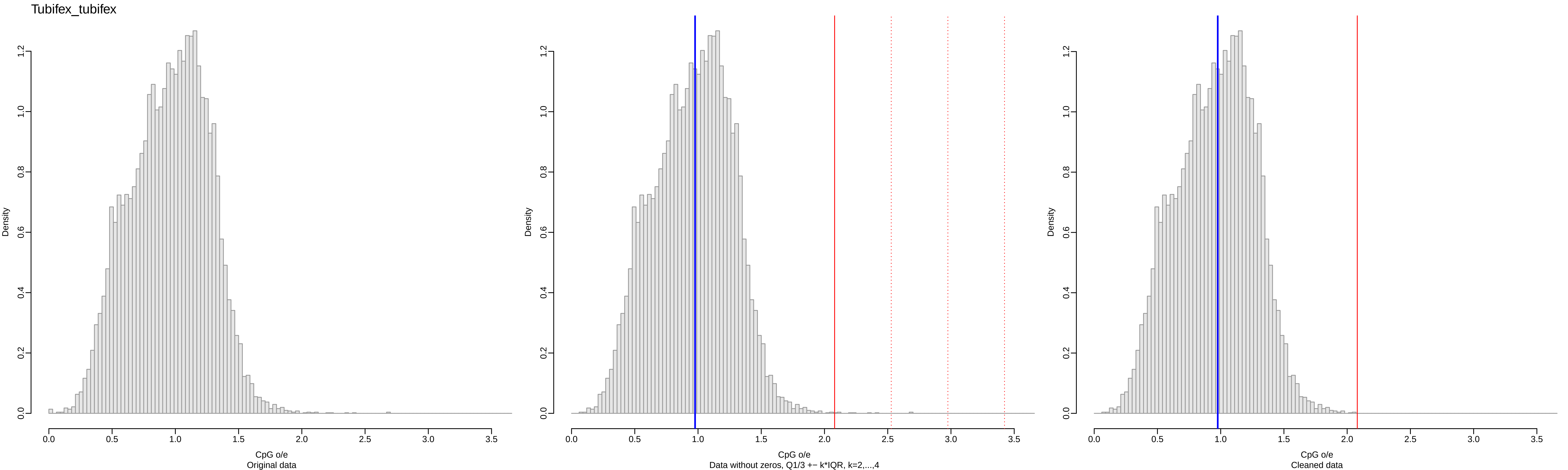

Tubifex_tubifex
Density
CpG o/e
Original data
Data without zeros, Q1/3 +− k*IQR, k=2,...,4
Cleaned data

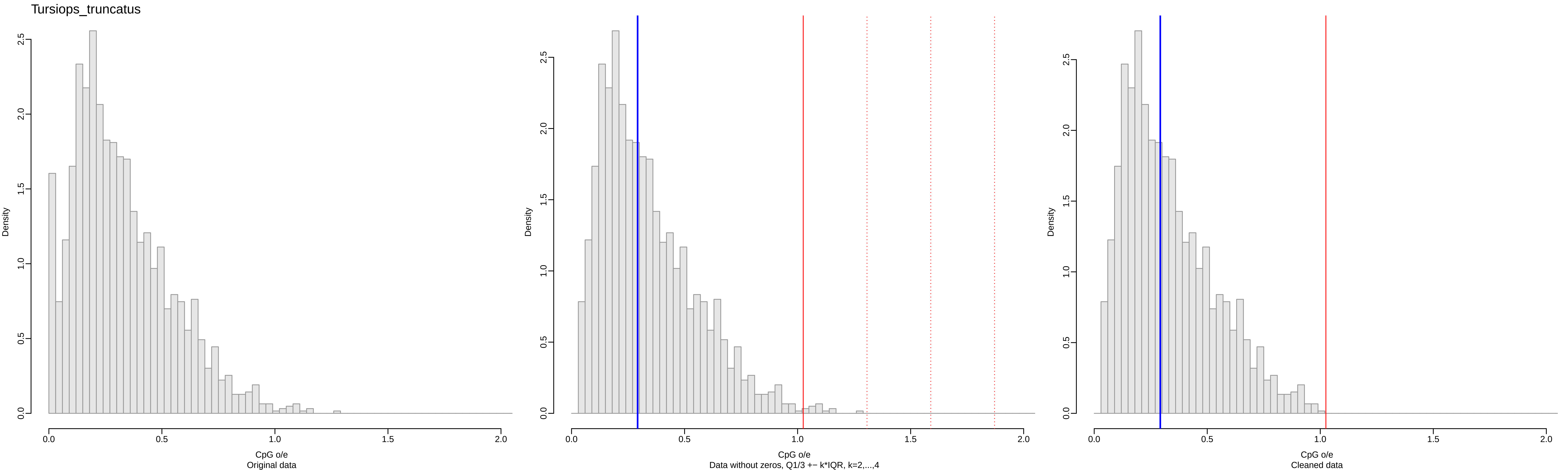
Tursiops_truncatus
Density
CpG o/e
Original data
Data without zeros, Q1/3 +− k*IQR, k=2,...,4
Cleaned data
0.0
0.5
1.0
1.5
2.0
2.5

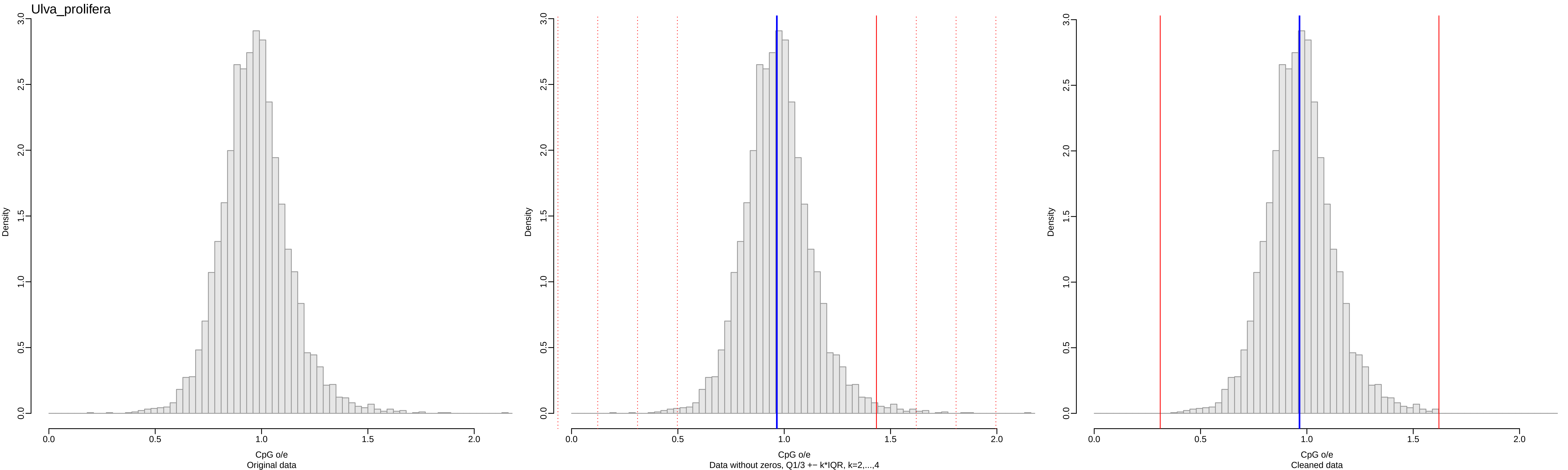

Ulva_prolifera
Density
0.0
0.5
1.0
1.5
2.0
2.5
3.0
CpG o/e
Original data
Data without zeros, Q1/3 +− k*IQR, k=2,...,4
Cleaned data

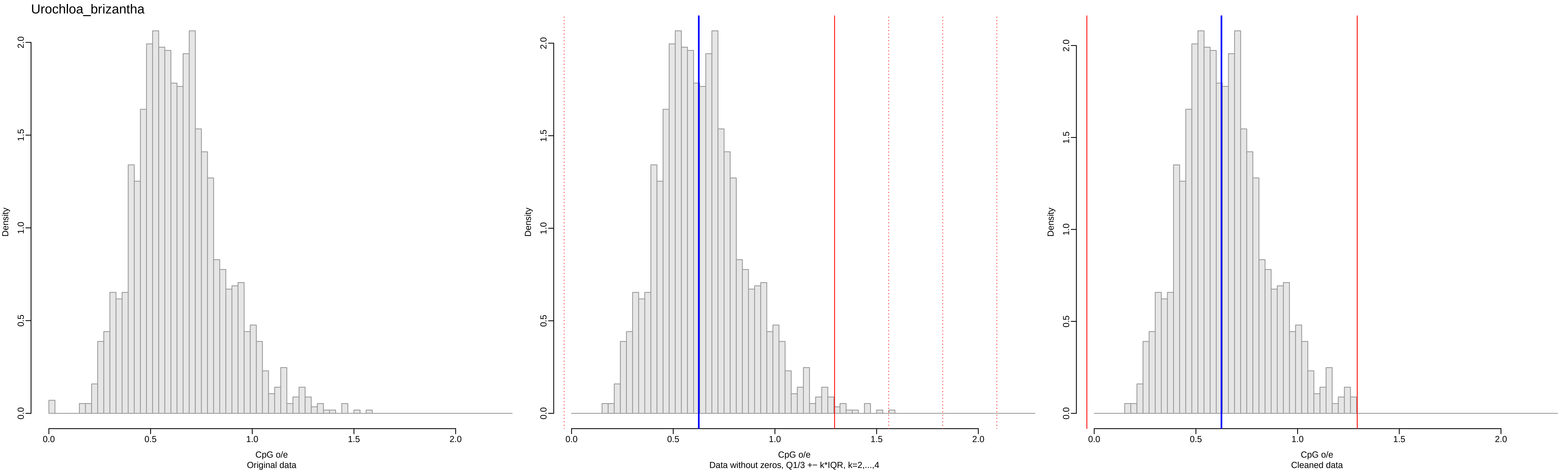

Urochloa_brizantha
Density
CpG o/e
Original data
Data without zeros, Q1/3 +− k*IQR, k=2,...,4
Cleaned data
0.0
0.5
1.0
1.5
2.0

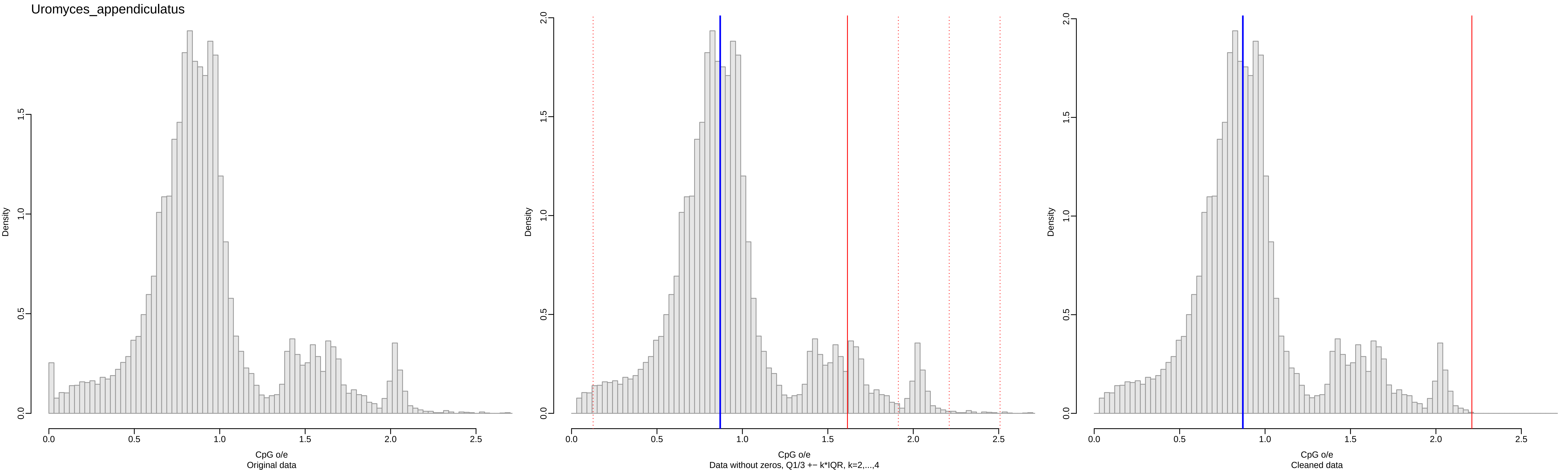

Uromyces_appendiculatus
Density
CpG o/e
Original data
Data without zeros, Q1/3 +− k*IQR, k=2,...,4
Cleaned data
0.0
0.5
1.0
1.5
2.0
2.5

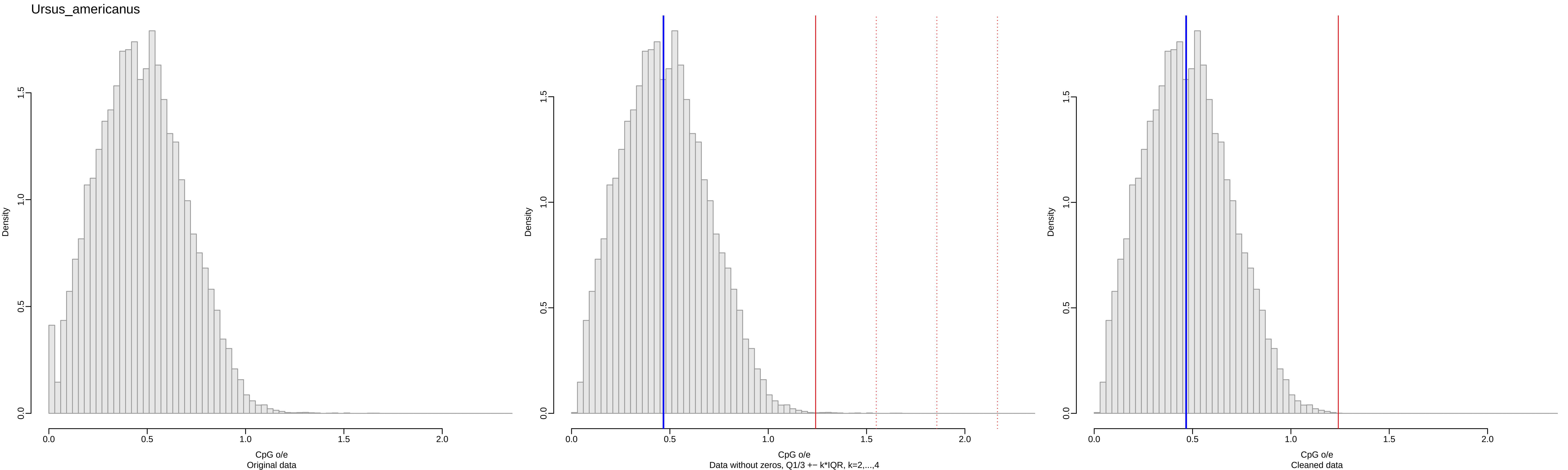
Ursus_americanus
Density
0.0
0.5
1.0
1.5
2.0
CpG o/e
Original data
Data without zeros, Q1/3 +− k*IQR, k=2,...,4
Cleaned data

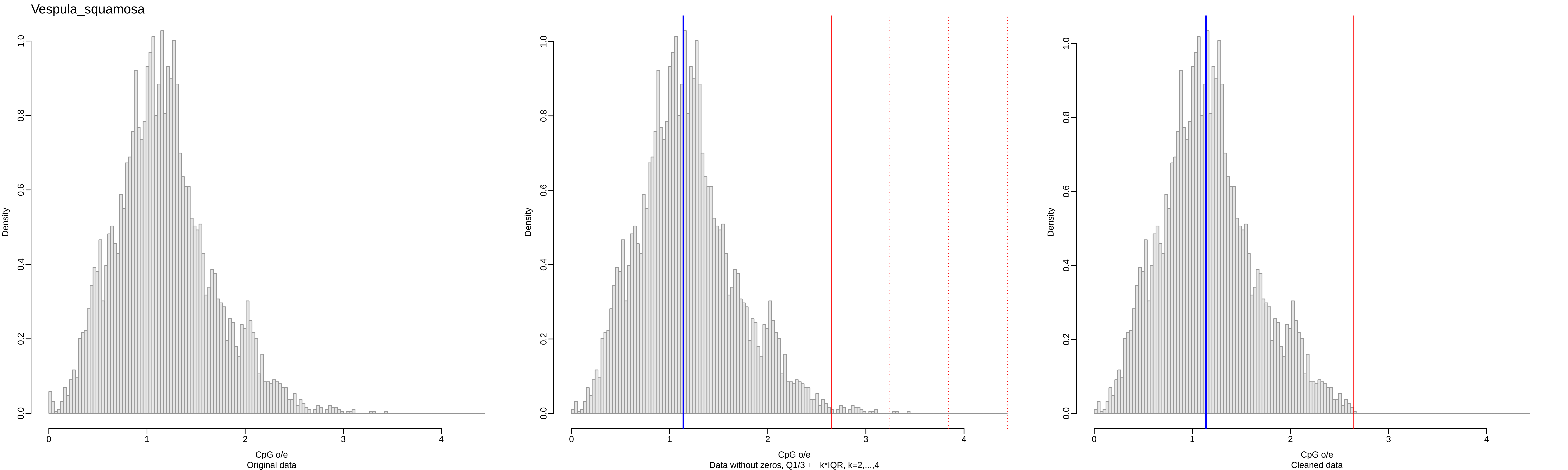
Vespula_squamosa
Density
CpG o/e
Original data
Density
CpG o/e
Data without zeros, Q1/3 +− k*IQR, k=2,...,4
Density
CpG o/e
Cleaned data

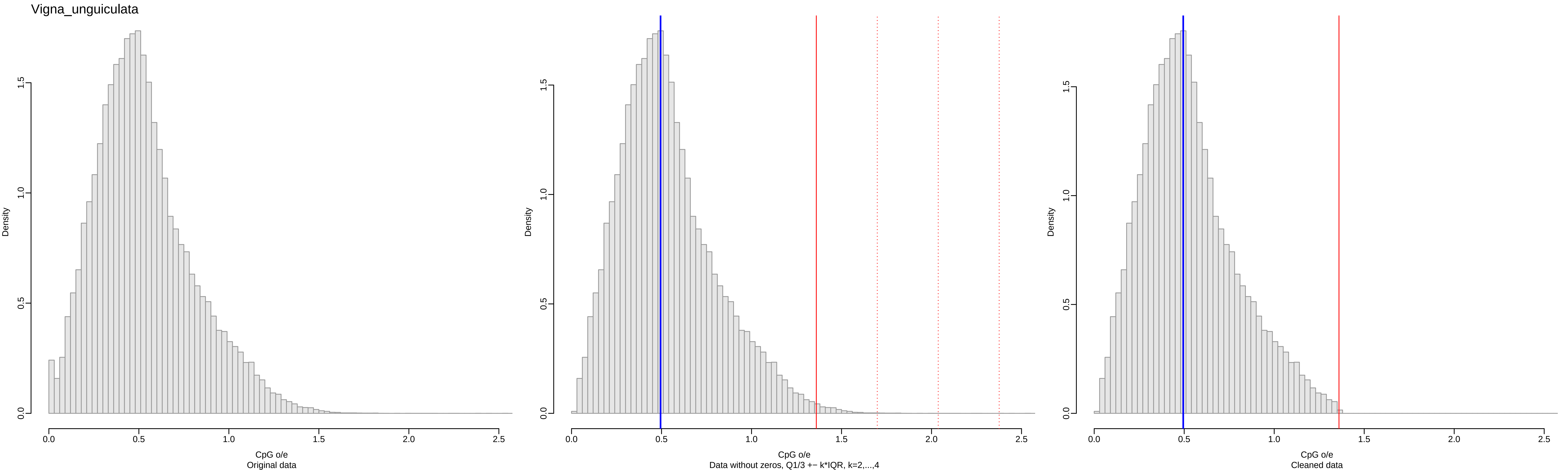
Vigna_unguiculata
Density
0.0
0.5
1.0
1.5
0.0
0.5
1.0
1.5
2.0
2.5
CpG o/e
Original data
Data without zeros, Q1/3 +− k*IQR, k=2,...,4
Cleaned data

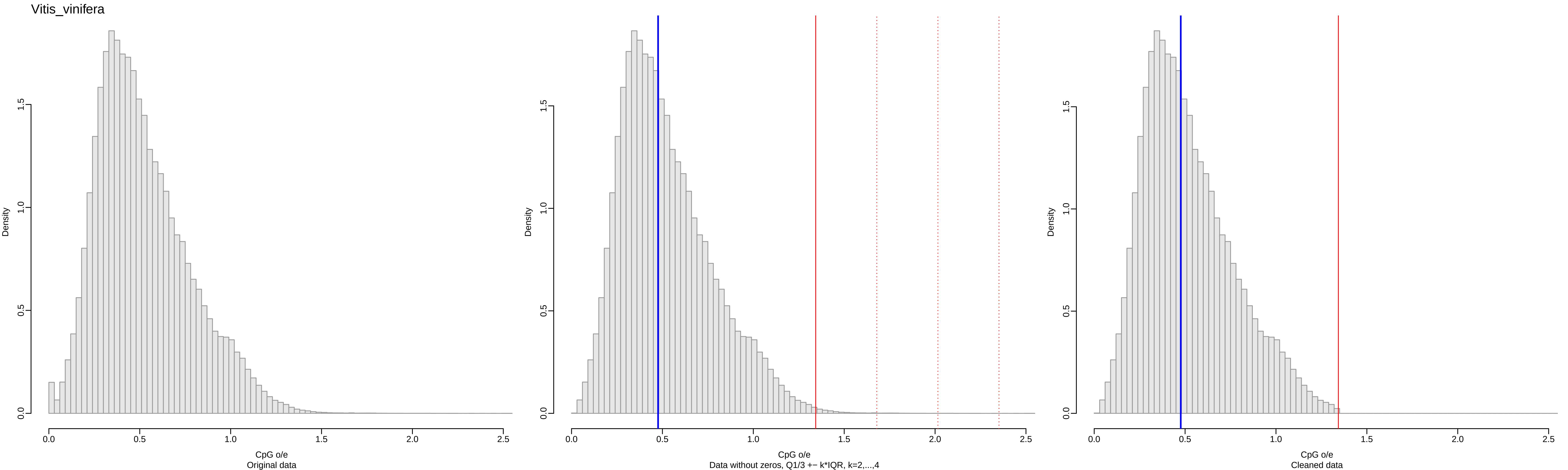

Vitis_vinifera
Density
0.0
0.5
1.0
1.5
2.0
2.5
CpG o/e
Original data
Data without zeros, Q1/3 +− k*IQR, k=2,...,4
Cleaned data

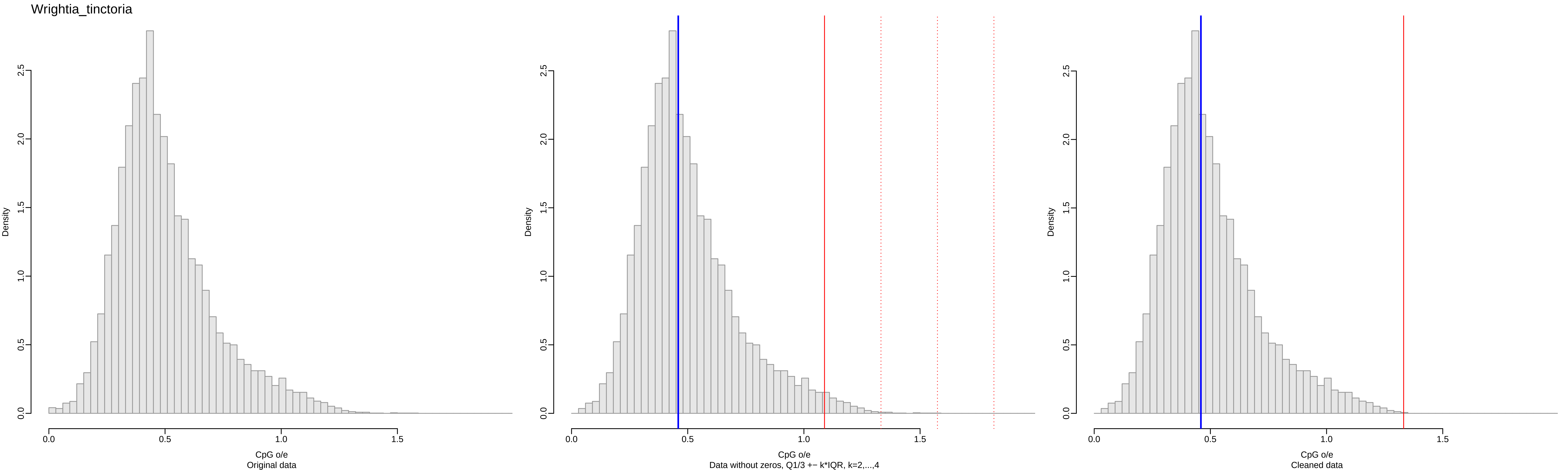

Wrightia_tinctoria
Density
CpG o/e
Original data
Data without zeros, Q1/3 +− k*IQR, k=2,...,4
Cleaned data
0.0
0.5
1.0
1.5
2.0
2.5

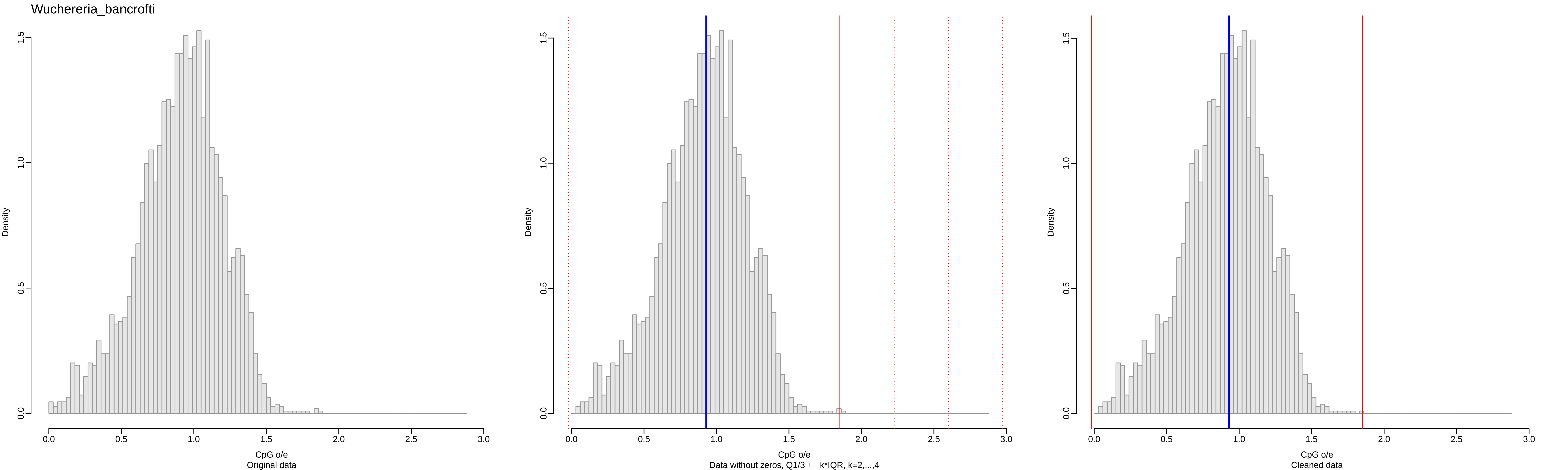

Wuchereria_bancrofti
Density
CpG o/e
Original data
Data without zeros, Q1/3 +− k*IQR, k=2,...,4
Cleaned data
0.0
0.5
1.0
1.5
2.0
2.5
3.0

Xenopus_laevis

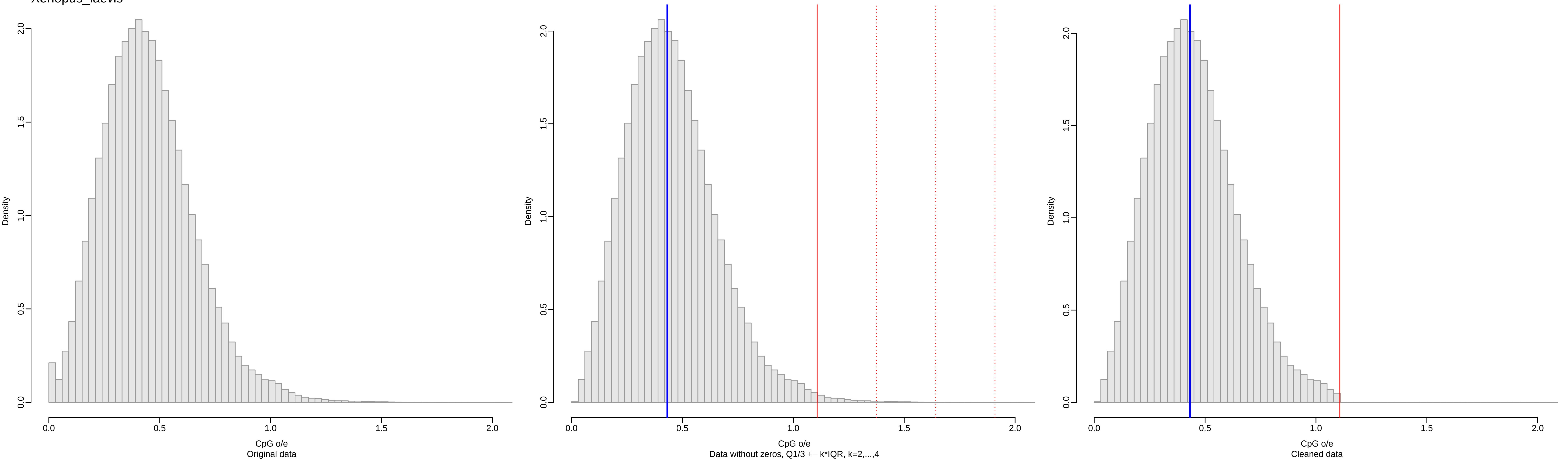

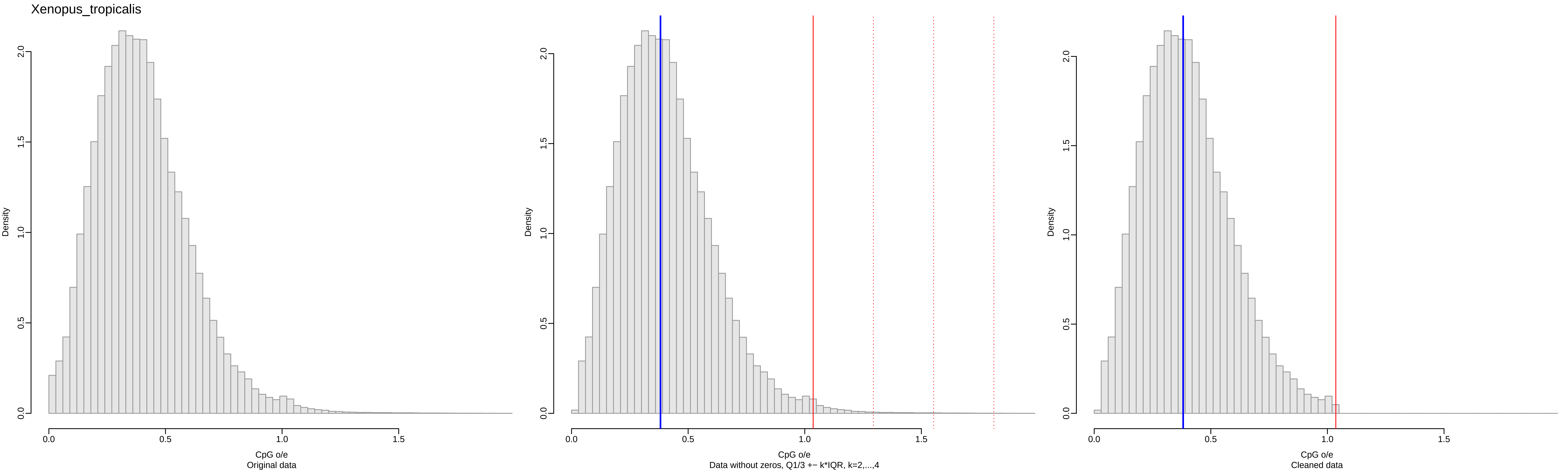

Xenopus_tropicalis
Density
CpG o/e
Original data
Data without zeros, Q1/3 +− k*IQR, k=2,...,4
Cleaned data
0.0
0.5
1.0
1.5
2.0

Yarrowia_lipolytica

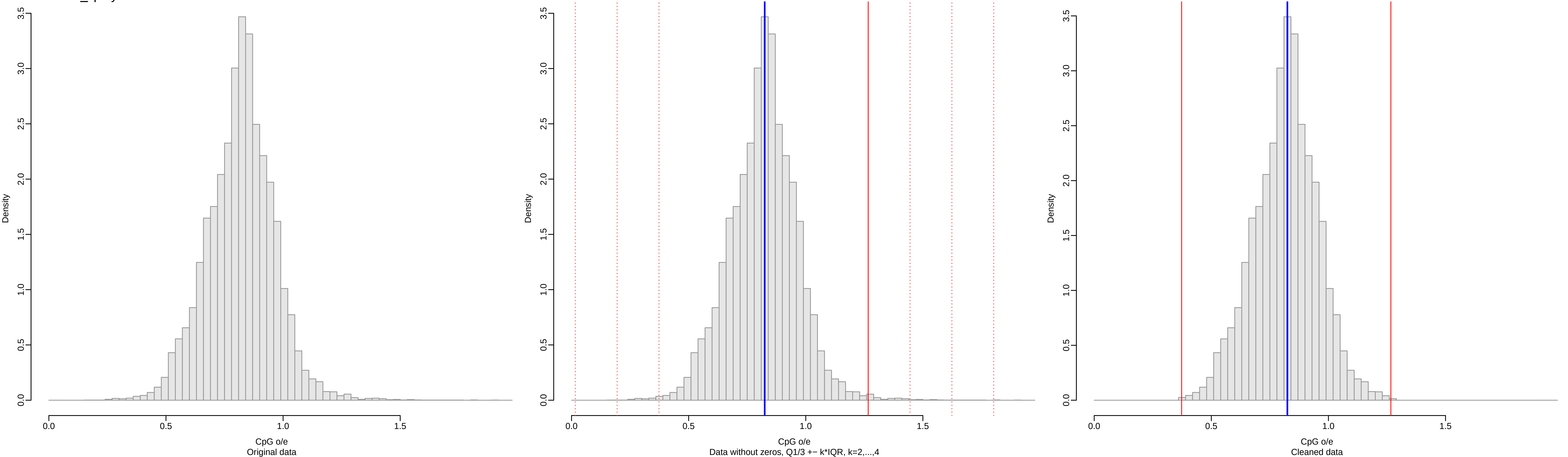

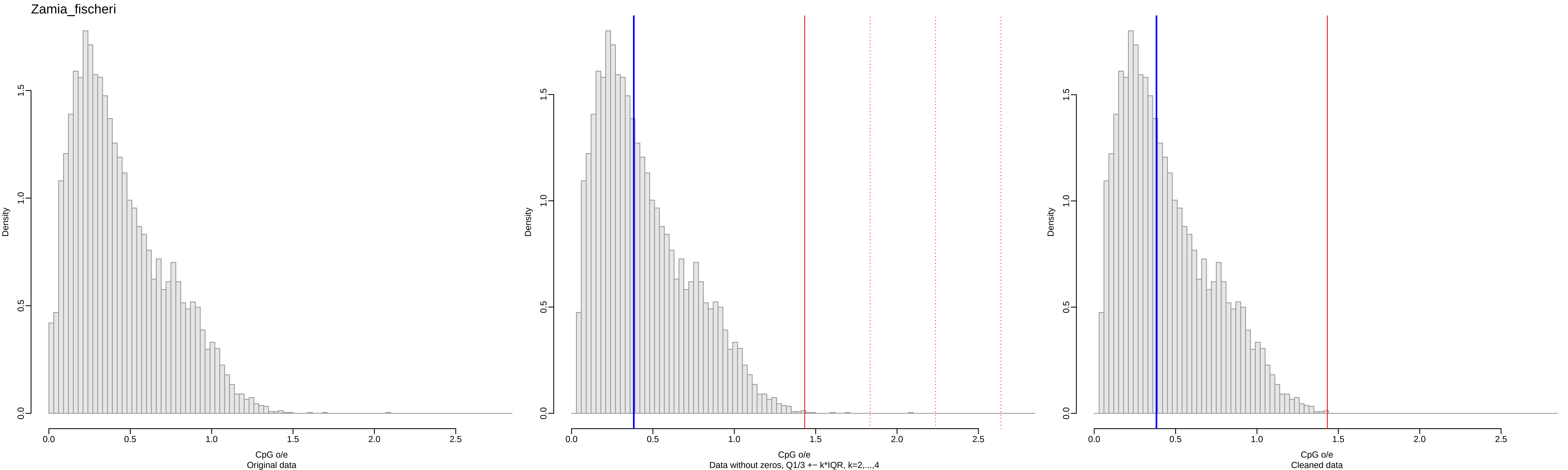

Zamia_fischeri
Density
CpG o/e
Original data
Density
CpG o/e
Data without zeros, Q1/3 +− k*IQR, k=2,...,4
Density
CpG o/e
Cleaned data

Zantedeschia_aethiopica

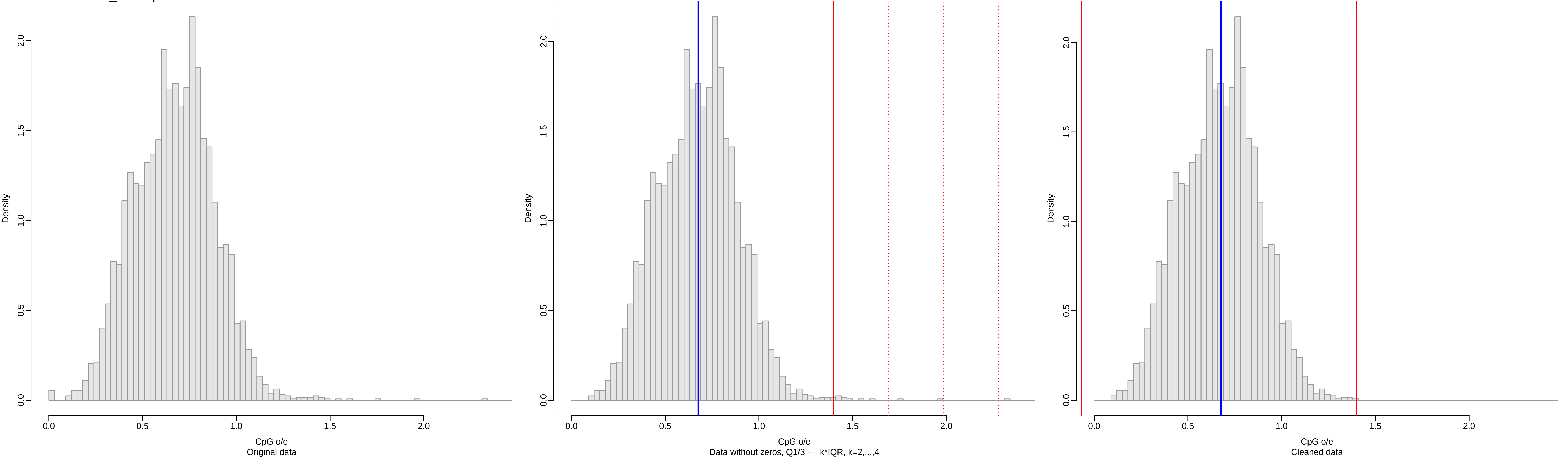

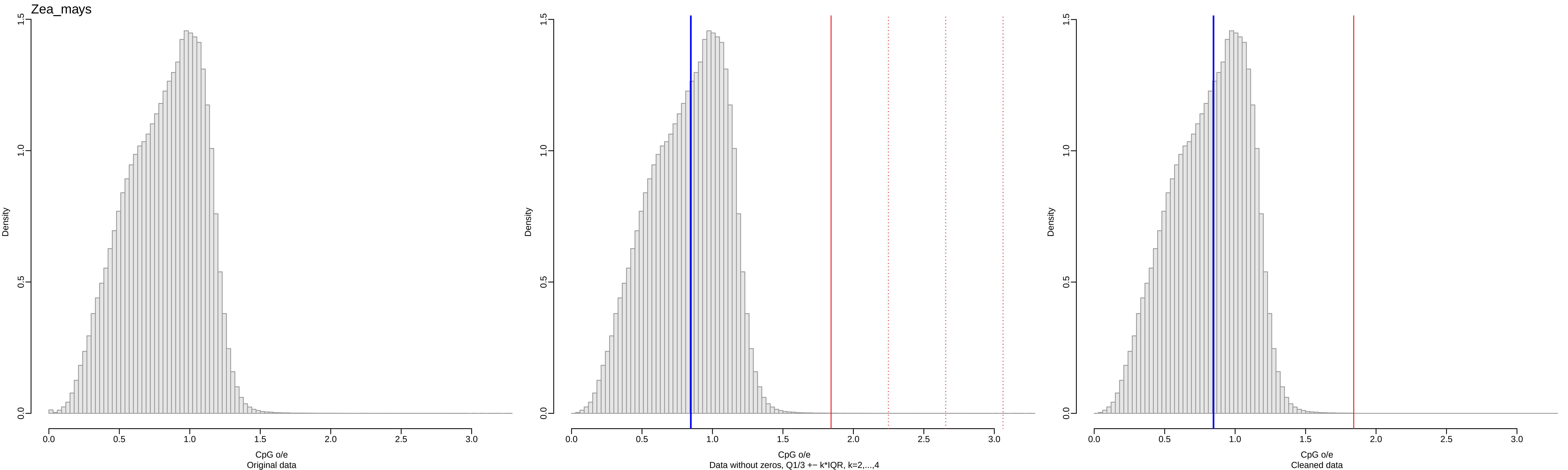
Zea_mays
Density
CpG o/e
Original data
Data without zeros, Q1/3 +− k*IQR, k=2,...,4
Cleaned data
0.0
0.5
1.0
1.5
2.0
2.5
3.0

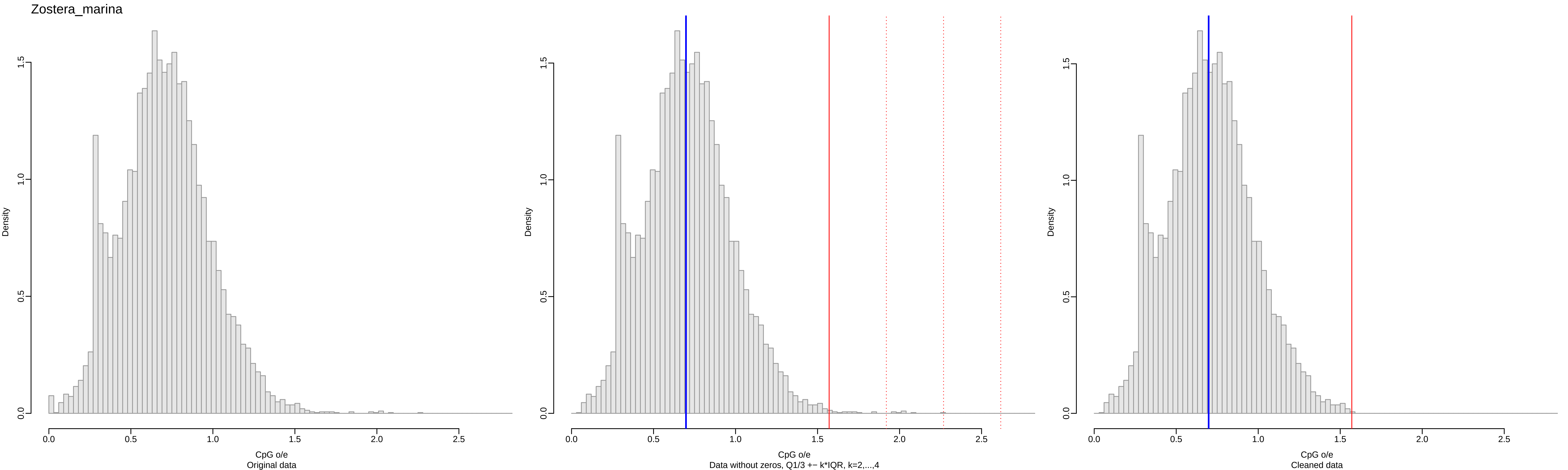
Zostera_marina
Density
CpG o/e
Original data
Data without zeros, Q1/3 +− k*IQR, k=2,...,4
Cleaned data
0.0
0.5
1.0
1.5
2.0
2.5

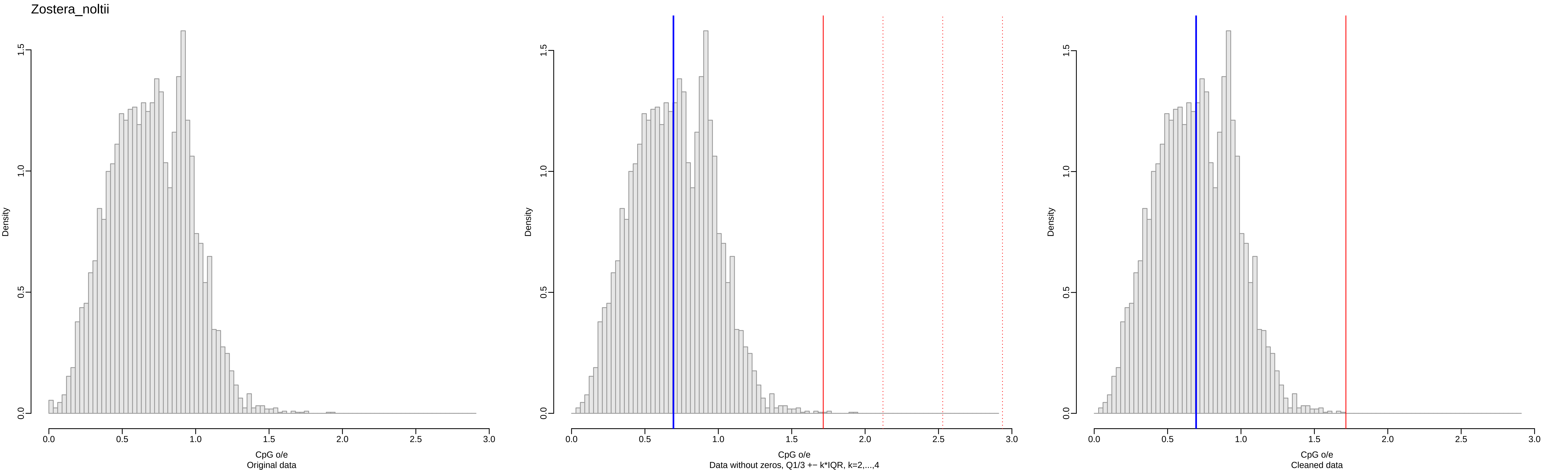
Zostera_noltii
Density
CpG o/e
Original data
CpG o/e
Data without zeros, Q1/3 +− k*IQR, k=2,...,4
CpG o/e
Cleaned data
0.0
0.5
1.0
1.5
2.0
2.5
3.0
