# Supplementary material for: Notos - a galaxy tool to analyze CpN observed expected ratios for inferring DNA methylation types
Source: BMC Bioinformatics. 2018 Mar 27;19:105. doi: 10.1186/s12859-018-2115-4 (PMC5870242; doi:10.1186/s12859-018-2115-4)
Supplement: Supplementary file 4 — CpG o/e ratios from dbEST analyzed by Notos: mode detection output - graphics. This file shows the graphical output from the density estimation step with activated option for the bootstrap procedure. (PDF 29500 kb) [file 12859_2018_2115_MOESM4_ESM.pdf]

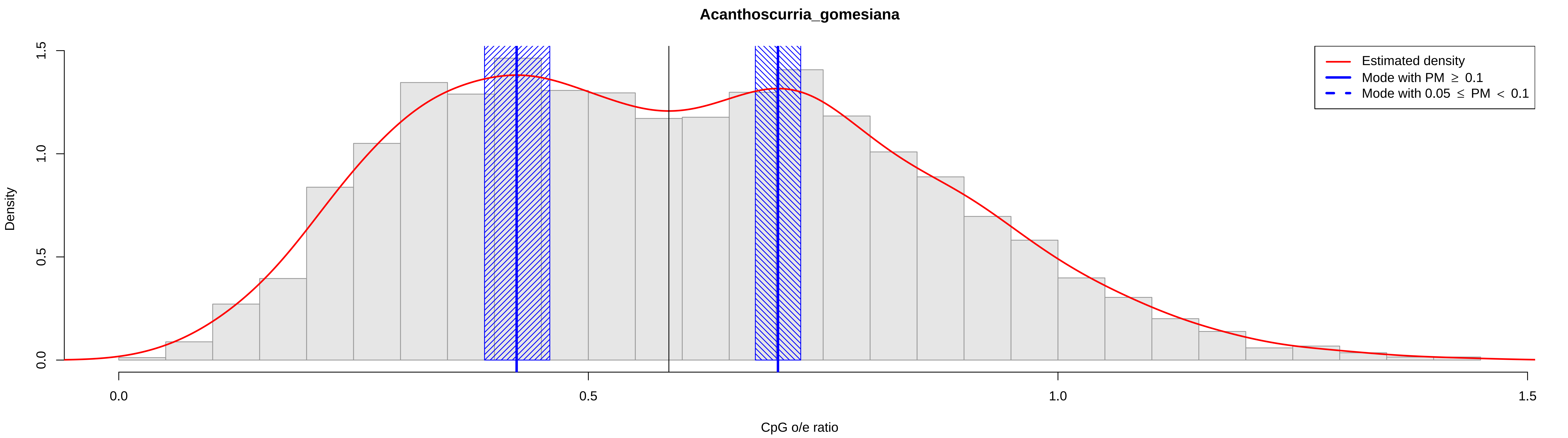

Acanthoscurria_gomesiana
Estimated density
Mode with PM ≥ 0.1
Mode with 0.05 ≤ PM < 0.1
Density
0.0
0.5
1.0
1.5
0.0
0.5
1.0
1.5
CpG o/e ratio

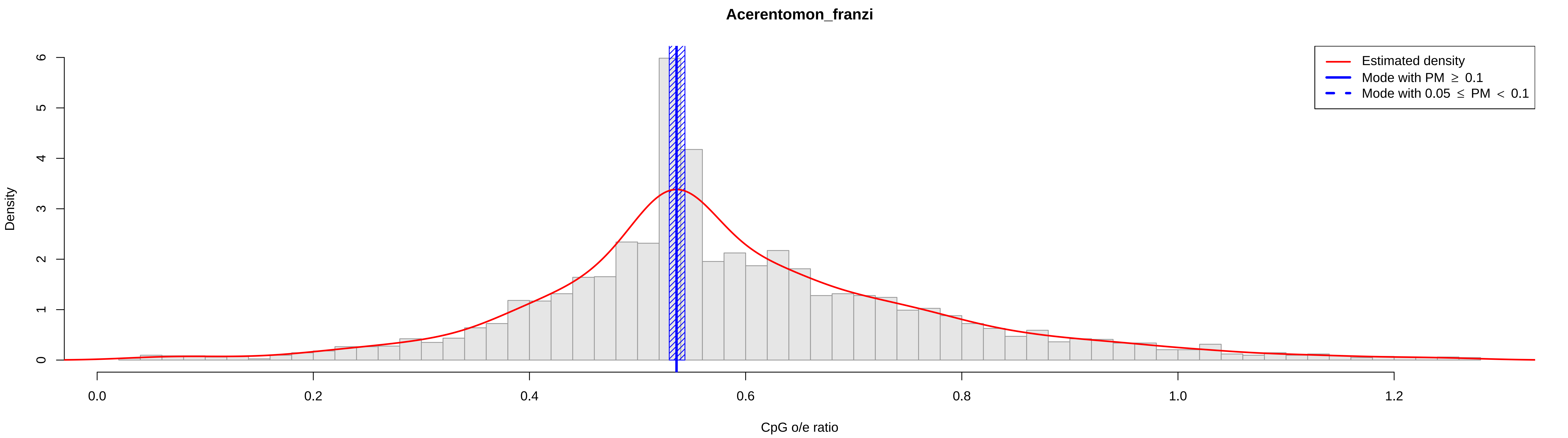

Acerentomon_franzi
Density
CpG o/e ratio
0.0
0.2
0.4
0.6
0.8
1.0
1.2
0
1
2
3
4
5
6
Estimated density
Mode with PM ≥ 0.1
Mode with 0.05 ≤ PM < 0.1

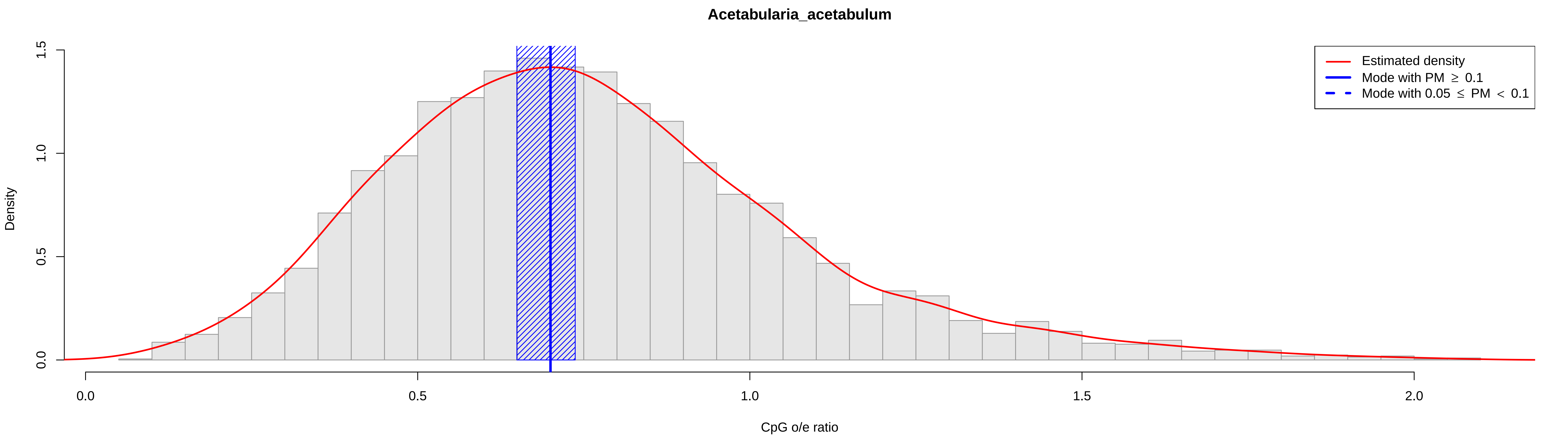
Acetabularia_acetabulum
Estimated density
Mode with PM ≥ 0.1
Mode with 0.05 ≤ PM < 0.1
Density
0.0
0.5
1.0
1.5
0.0
0.5
1.0
1.5
2.0
CpG o/e ratio

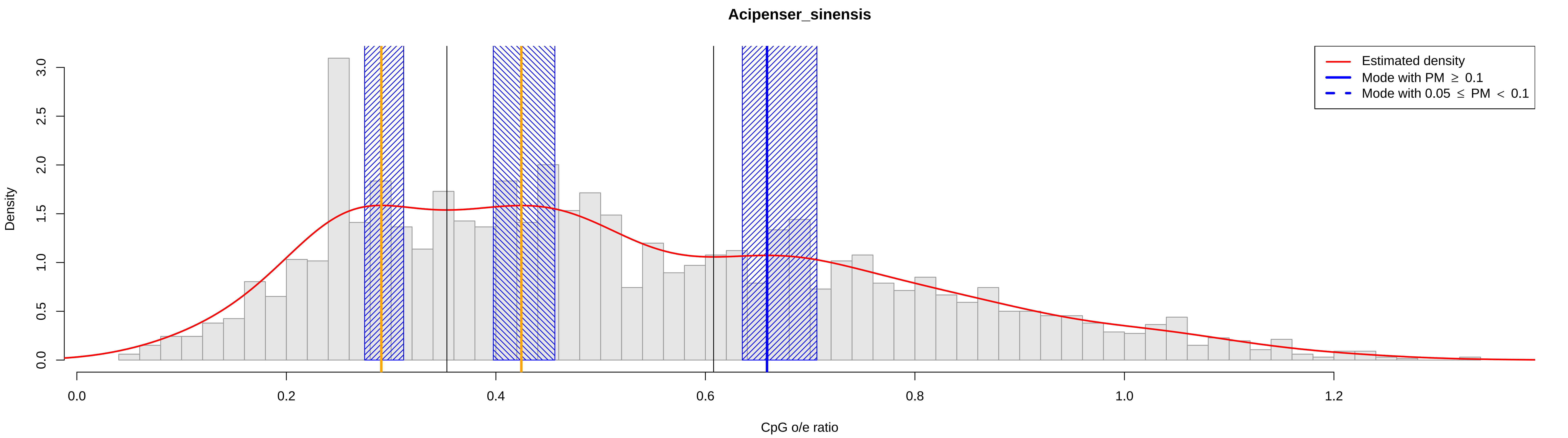

Acipenser_sinensis
Estimated density
Mode with PM ≥ 0.1
Mode with 0.05 ≤ PM < 0.1
Density
0.0
0.5
1.0
1.5
2.0
2.5
3.0
0.0
0.2
0.4
0.6
0.8
1.0
1.2
CpG o/e ratio

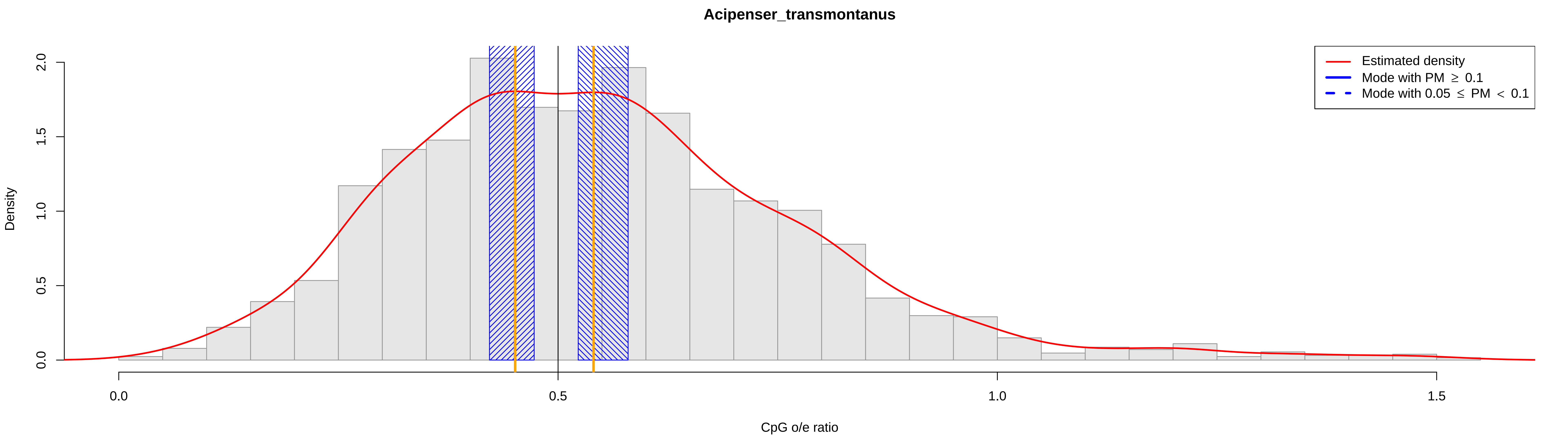
Acipenser_transmontanus
Estimated density
Mode with PM ≥ 0.1
Mode with 0.05 ≤ PM < 0.1
Density
0.0
0.5
1.0
1.5
2.0
0.0
0.5
1.0
1.5
CpG o/e ratio

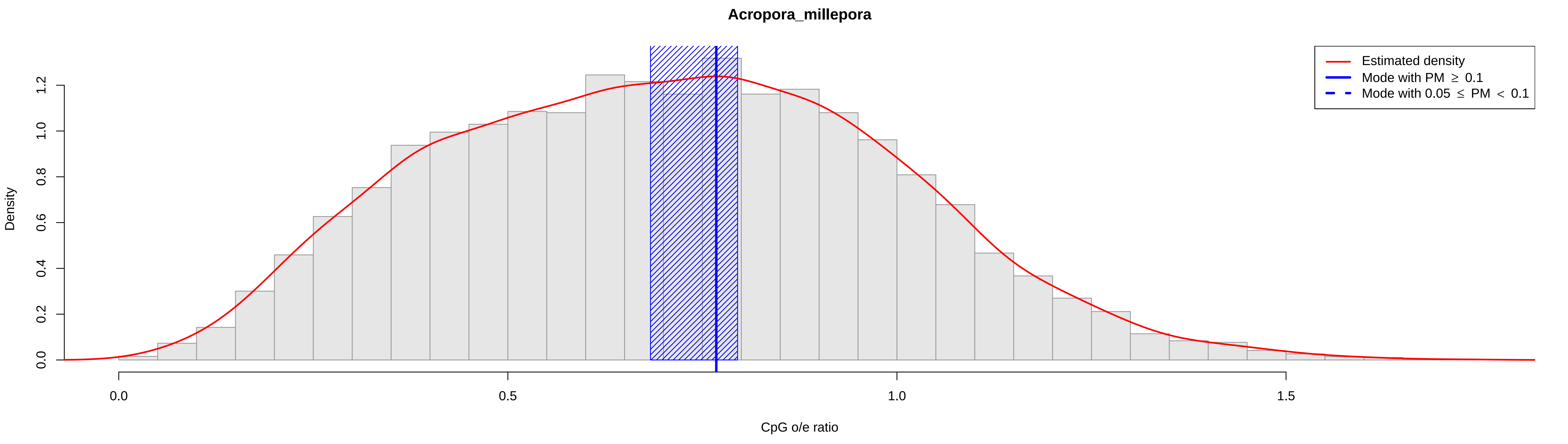

Acropora_millepora
Estimated density
Mode with PM ≥ 0.1
Mode with 0.05 ≤ PM < 0.1
Density
0.0
0.2
0.4
0.6
0.8
1.0
1.2
0.0
0.5
1.0
1.5
CpG o/e ratio

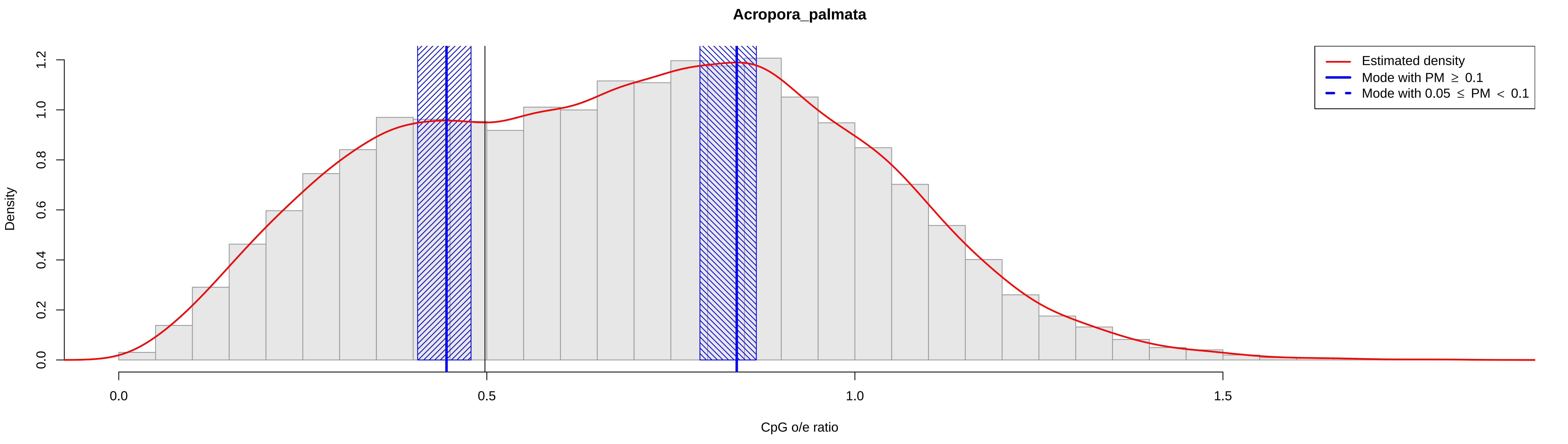

Acropora_palmata
Estimated density
Mode with PM ≥ 0.1
Mode with 0.05 ≤ PM < 0.1
Density
0.0
0.2
0.4
0.6
0.8
1.0
1.2
0.0
0.5
1.0
1.5
CpG o/e ratio

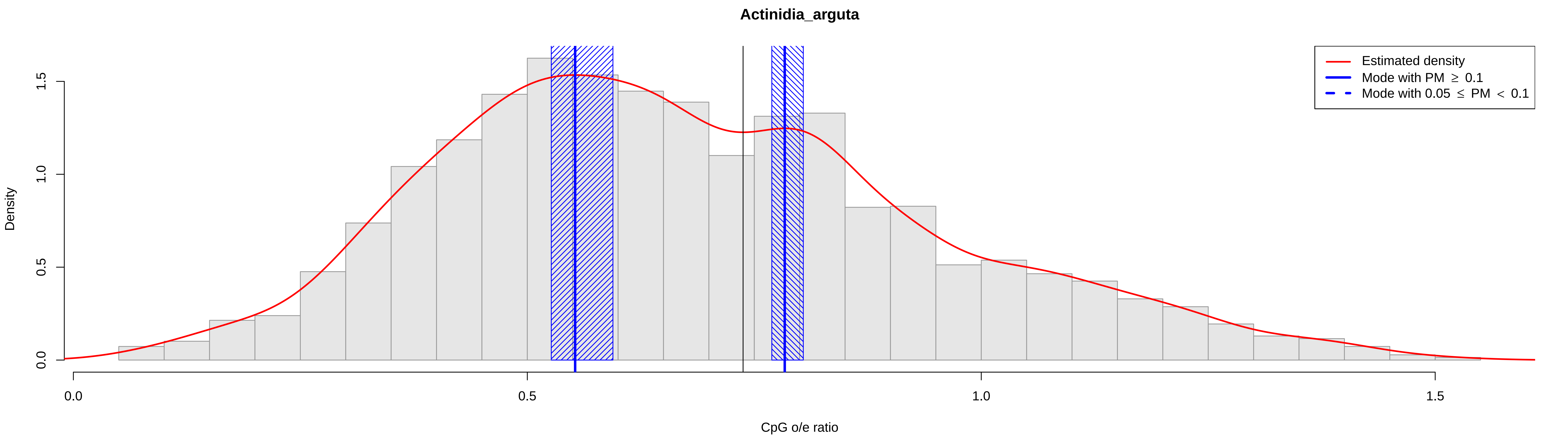

Actinidia_arguta
Estimated density
Mode with PM ≥ 0.1
Mode with 0.05 ≤ PM < 0.1
Density
0.0
0.5
1.0
1.5
0.0
0.5
1.0
1.5
CpG o/e ratio

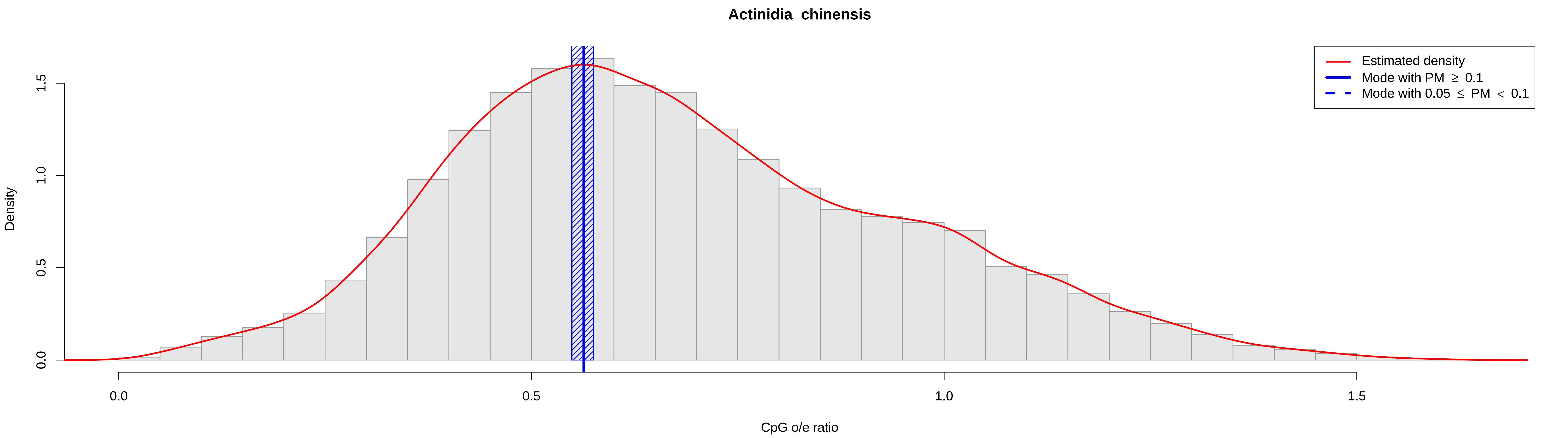

Actinidia_chinensis
Estimated density
Mode with PM ≥ 0.1
Mode with 0.05 ≤ PM < 0.1
Density
0.0
0.5
1.0
1.5
CpG o/e ratio

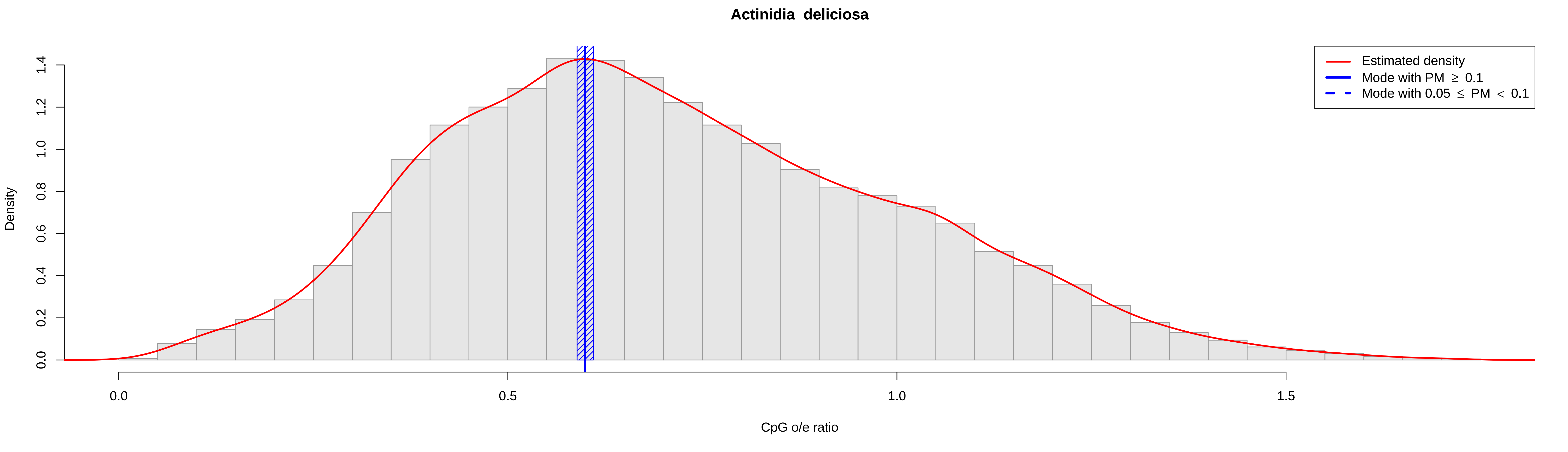
Actinidia_deliciosa
Estimated density
Mode with PM ≥ 0.1
Mode with 0.05 ≤ PM < 0.1
Density
0.0
0.2
0.4
0.6
0.8
1.0
1.2
1.4
0.0
0.5
1.0
1.5
CpG o/e ratio

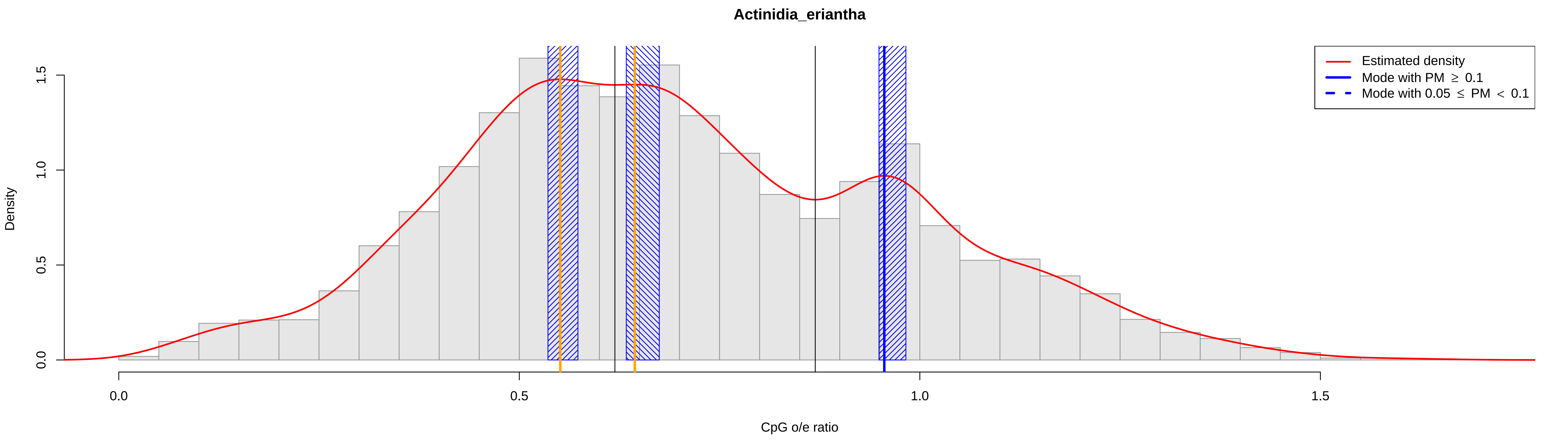

Actinidia_eriantha
Estimated density
Mode with PM ≥ 0.1
Mode with 0.05 ≤ PM < 0.1
Density
0.0
0.5
1.0
1.5
CpG o/e ratio

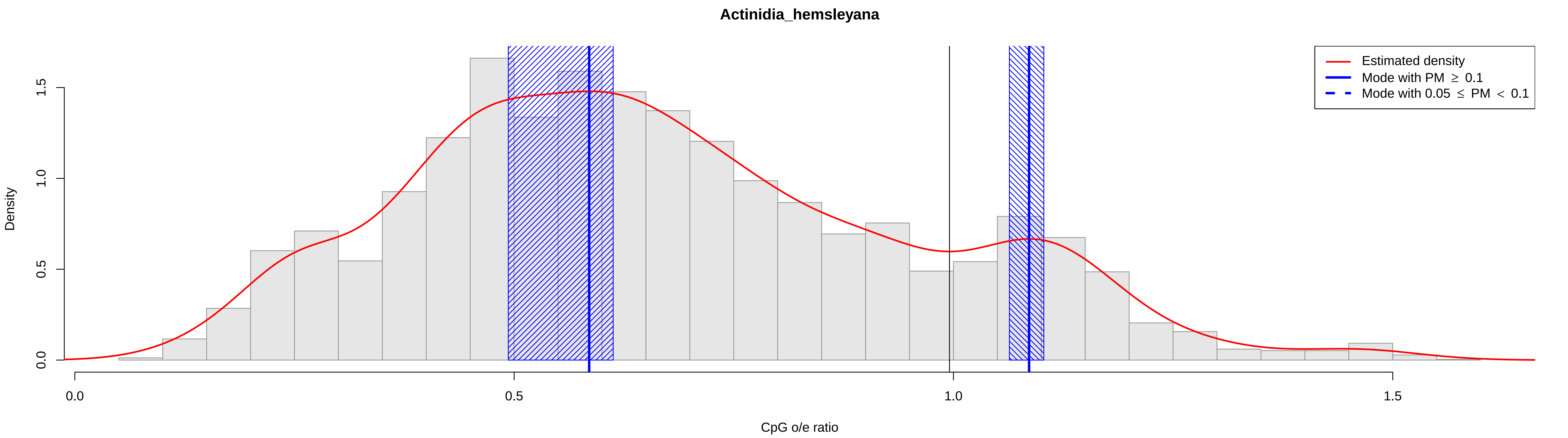

Actinidia_hemsleyana
Estimated density
Mode with PM ≥ 0.1
Mode with 0.05 ≤ PM < 0.1
Density
0.0
0.5
1.0
1.5
CpG o/e ratio

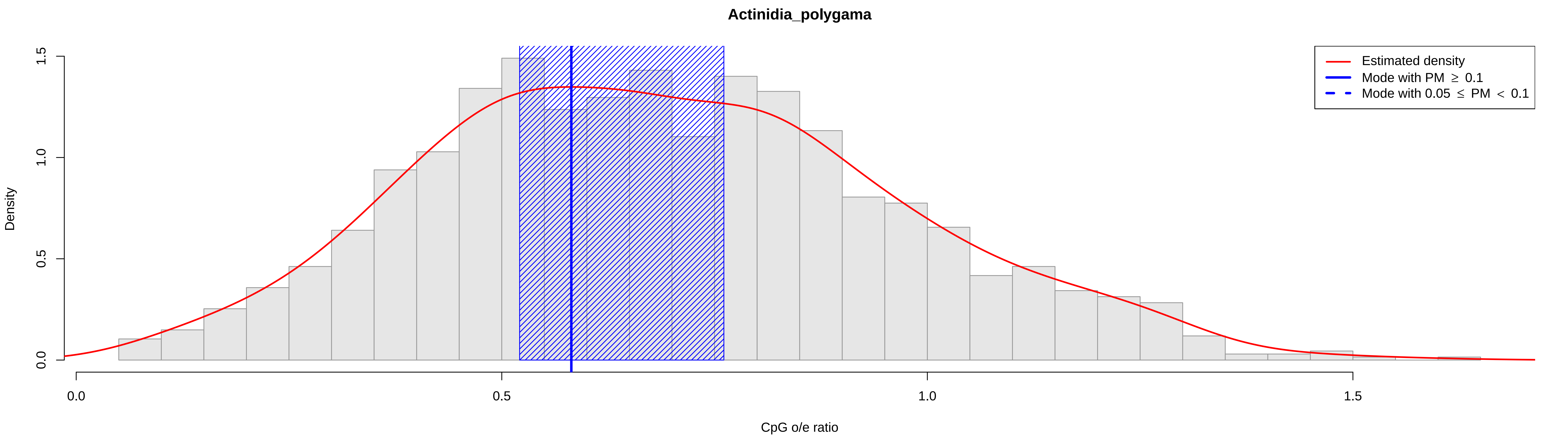

Actinidia_polygama
Estimated density
Mode with PM ≥ 0.1
Mode with 0.05 ≤ PM < 0.1
Density
0.0
0.5
1.0
1.5
0.0
0.5
1.0
1.5
CpG o/e ratio

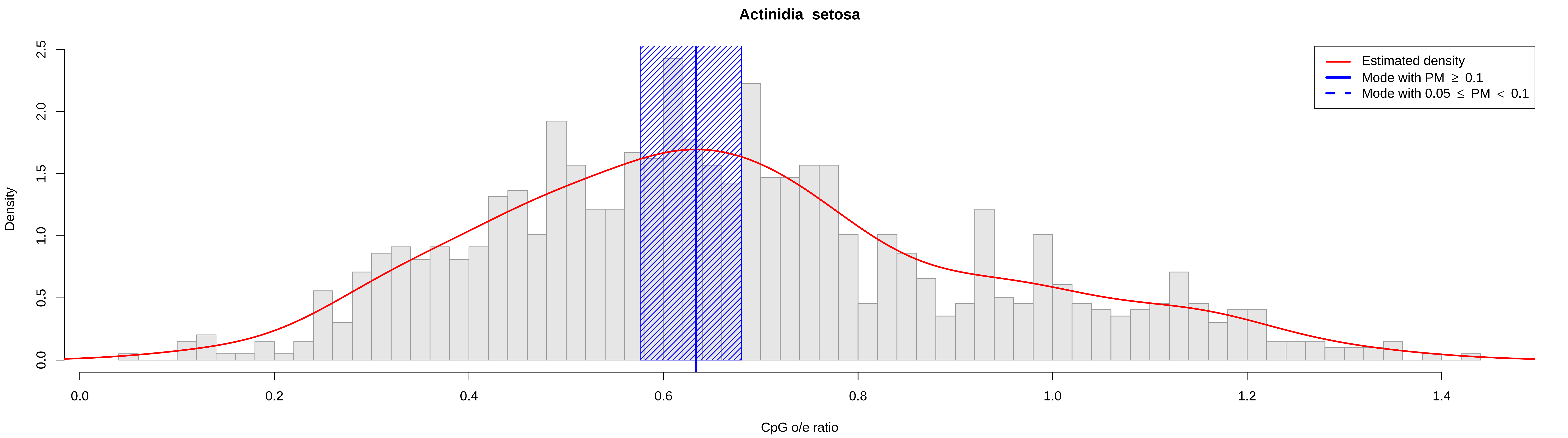
Actinidia_setosa
Estimated density
Mode with PM ≥ 0.1
Mode with 0.05 ≤ PM < 0.1
Density
CpG o/e ratio
0.0
0.5
1.0
1.5
2.0
2.5
0.0
0.2
0.4
0.6
0.8
1.0
1.2
1.4

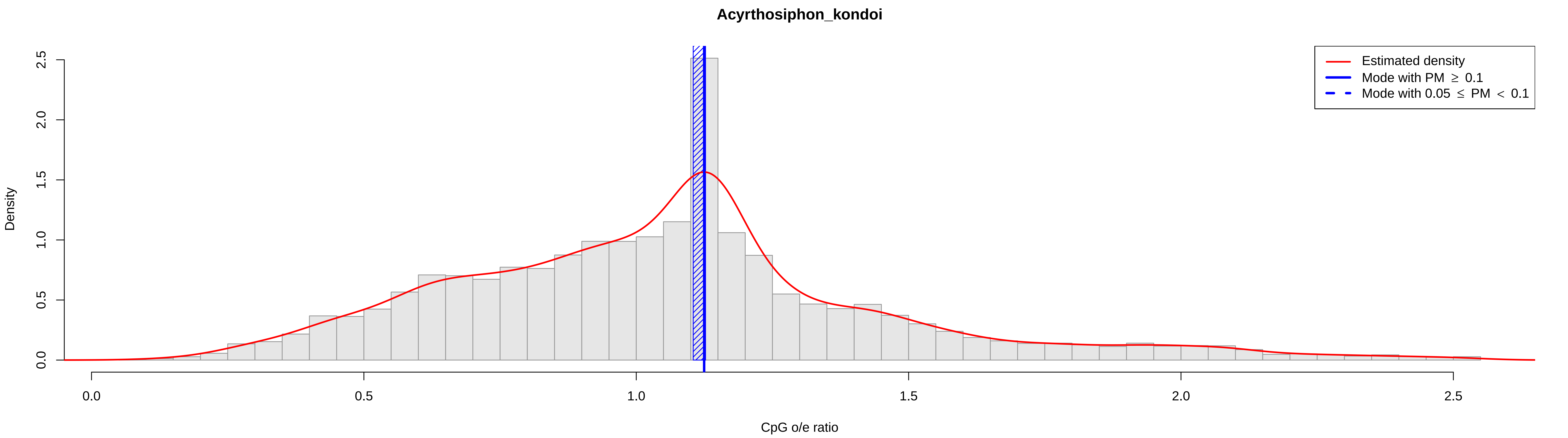

Acyrthosiphon_kondoi
Estimated density
Mode with PM ≥ 0.1
Mode with 0.05 ≤ PM < 0.1
Density
0.0
0.5
1.0
1.5
2.0
2.5
CpG o/e ratio

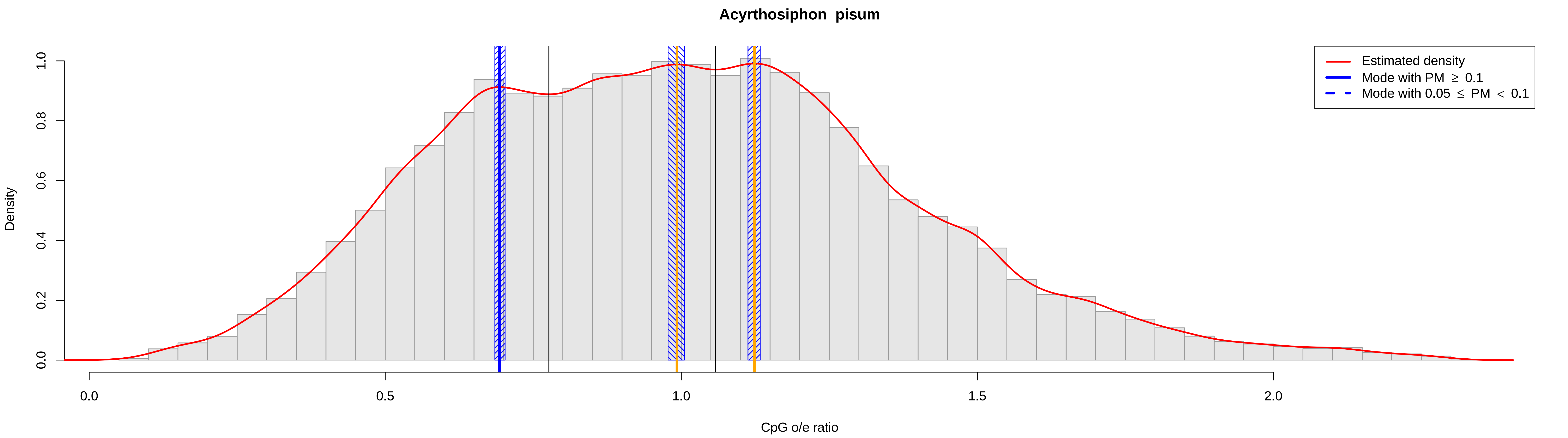
Acyrthosiphon_pisum
Estimated density
Mode with PM ≥ 0.1
Mode with 0.05 ≤ PM < 0.1
Density
CpG o/e ratio
0.0
0.2
0.4
0.6
0.8
1.0
0.0
0.5
1.0
1.5
2.0

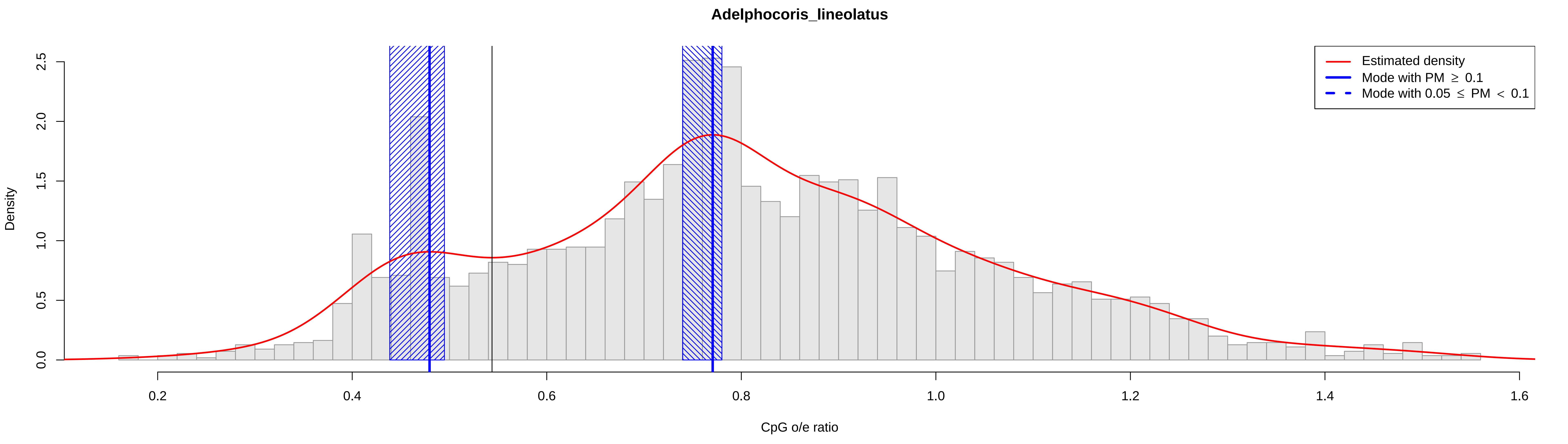

Adelphocoris_lineolatus
Estimated density
Mode with PM ≥ 0.1
Mode with 0.05 ≤ PM < 0.1
Density
0.0
0.5
1.0
1.5
2.0
2.5
0.2
0.4
0.6
0.8
1.0
1.2
1.4
1.6
CpG o/e ratio

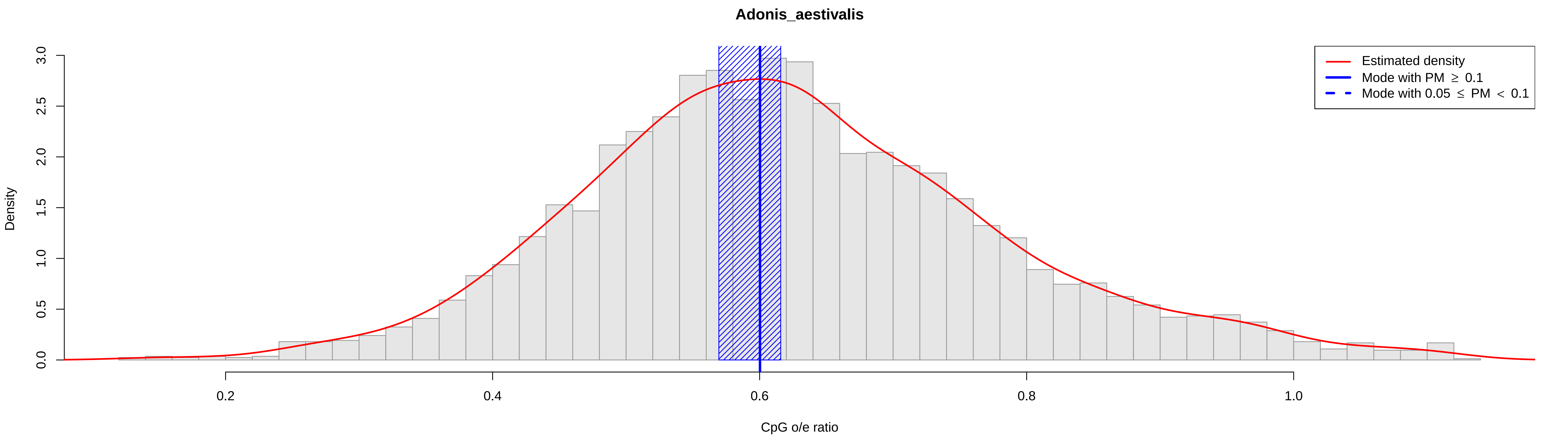

Adonis_aestivalis
Estimated density
Mode with PM ≥ 0.1
Mode with 0.05 ≤ PM < 0.1
Density
0.0
0.5
1.0
1.5
2.0
2.5
3.0
0.2
0.4
0.6
0.8
1.0
CpG o/e ratio

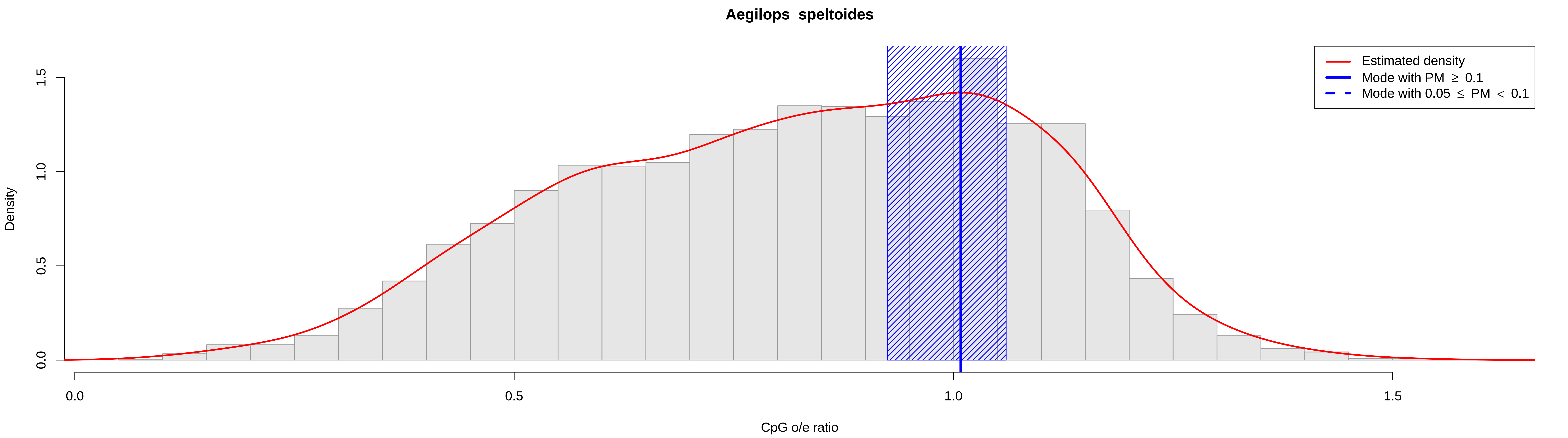
Aegilops_speltoides
Estimated density
Mode with PM ≥ 0.1
Mode with 0.05 ≤ PM < 0.1
Density
CpG o/e ratio
0.0
0.5
1.0
1.5

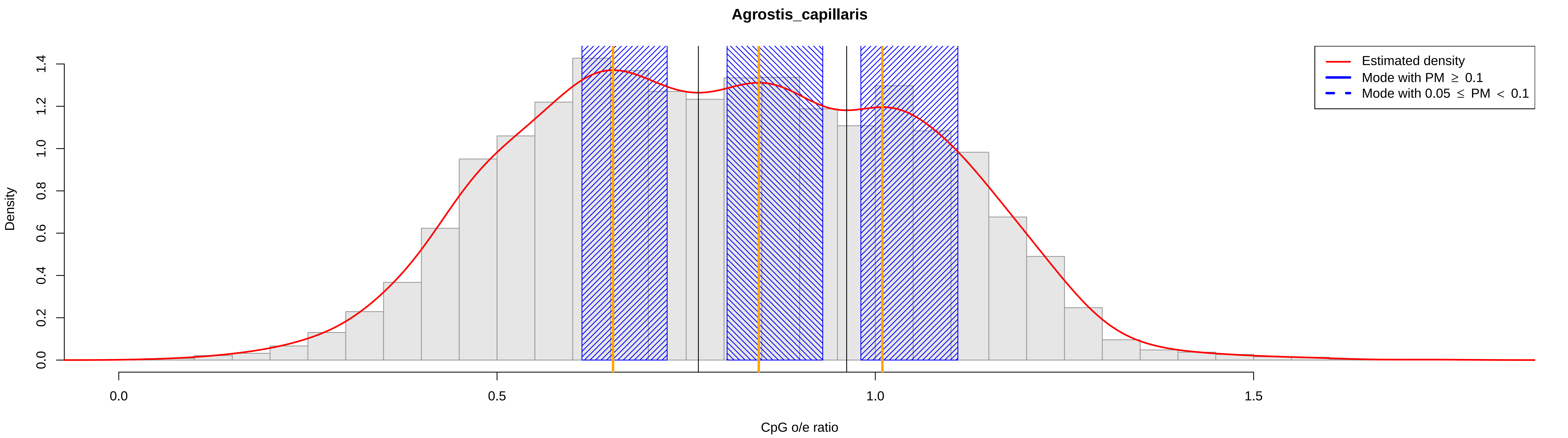
Agrostis_capillaris
Estimated density
Mode with PM ≥ 0.1
Mode with 0.05 ≤ PM < 0.1
Density
0.0
0.2
0.4
0.6
0.8
1.0
1.2
1.4
0.0
0.5
1.0
1.5
CpG o/e ratio

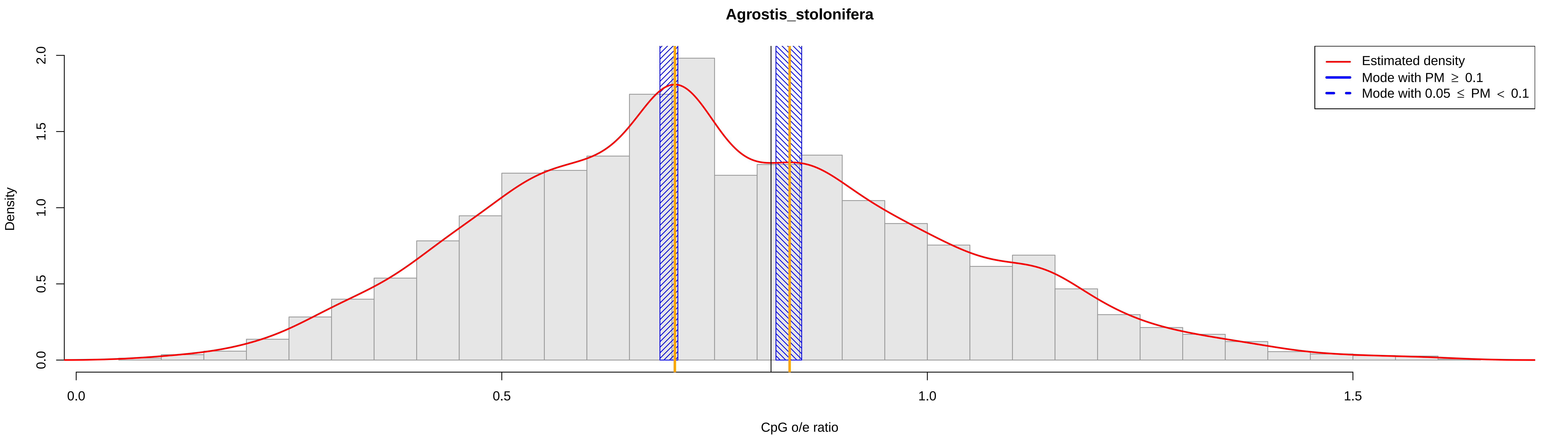

Agrostis_stolonifera
Estimated density
Mode with PM ≥ 0.1
Mode with 0.05 ≤ PM < 0.1
Density
0.0
0.5
1.0
1.5
2.0
0.0
0.5
1.0
1.5
CpG o/e ratio

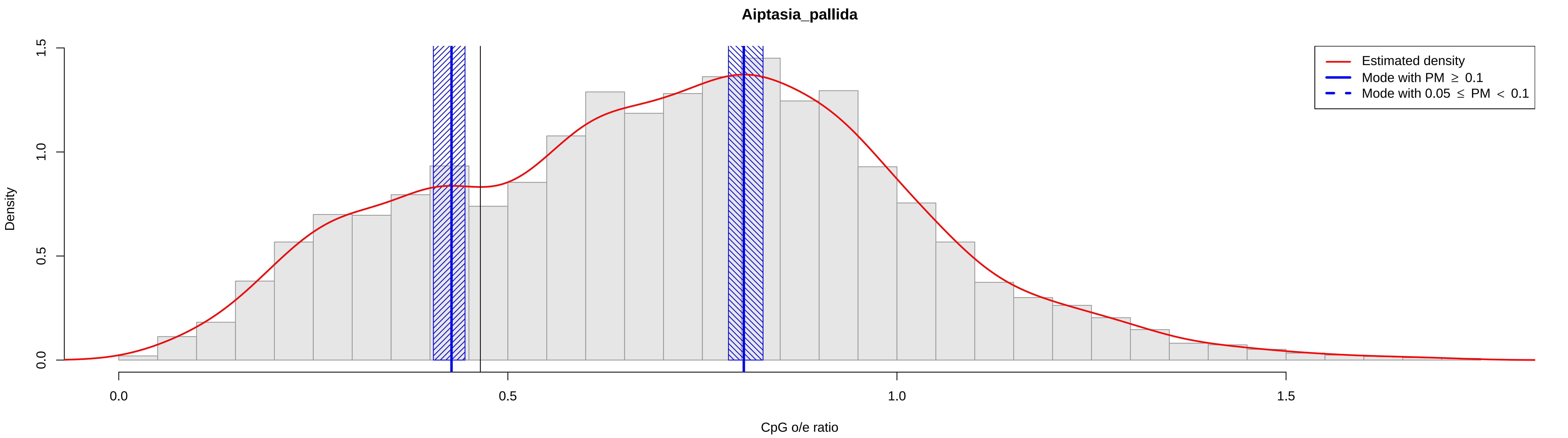
Aiptasia_pallida
Estimated density
Mode with PM ≥ 0.1
Mode with 0.05 ≤ PM < 0.1
Density
0.0
0.5
1.0
1.5
CpG o/e ratio
0.0
0.5
1.0
1.5

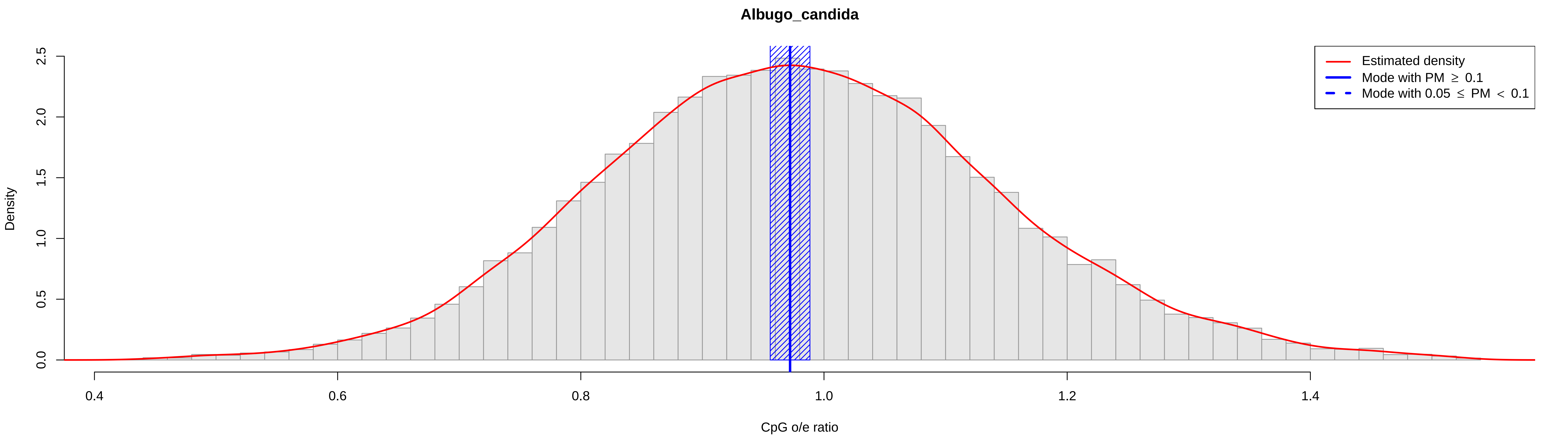
Albugo_candida
Density
0.0
0.5
1.0
1.5
2.0
2.5
0.4
0.6
0.8
1.0
1.2
1.4
CpG o/e ratio
Estimated density
Mode with PM ≥ 0.1
Mode with 0.05 ≤ PM < 0.1

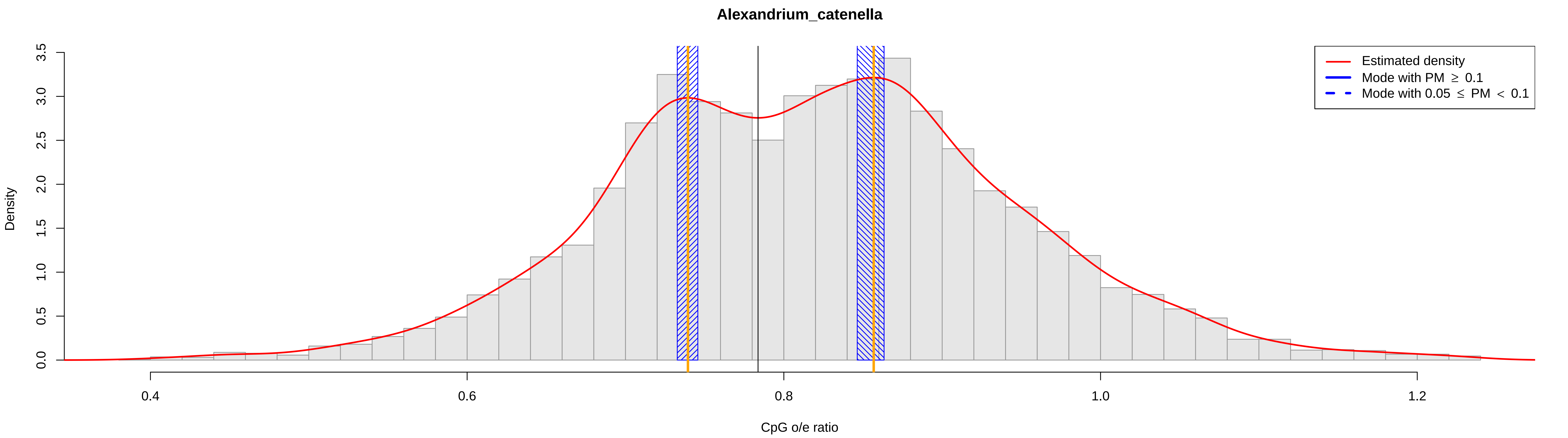
Alexandrium_catenella
Estimated density
Mode with PM ≥ 0.1
Mode with 0.05 ≤ PM < 0.1
Density
0.0
0.5
1.0
1.5
2.0
2.5
3.0
3.5
0.4
0.6
0.8
1.0
1.2
CpG o/e ratio

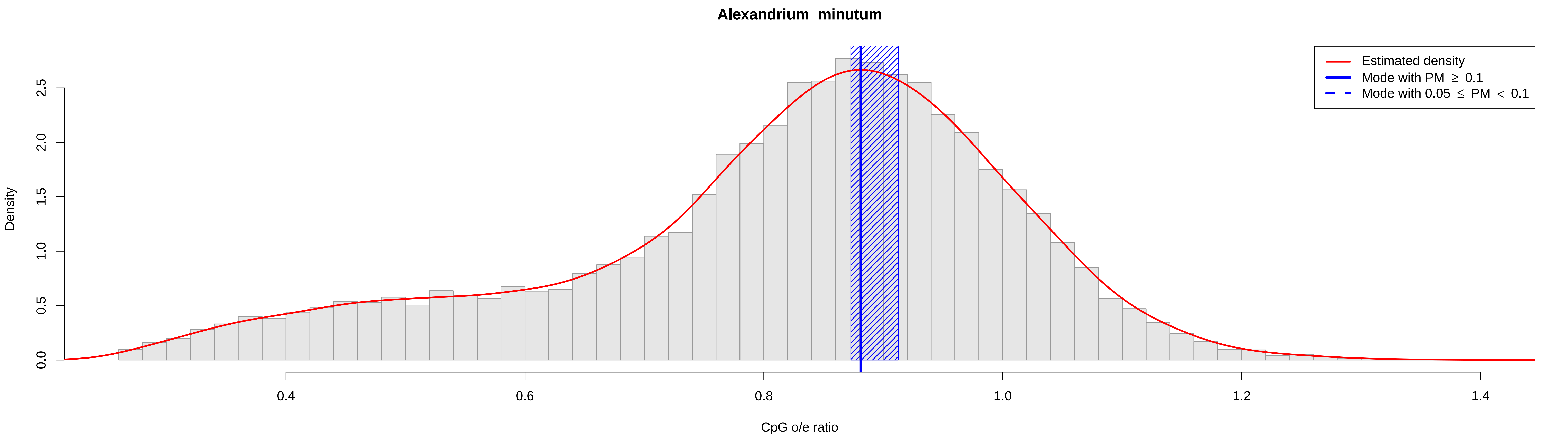

Alexandrium_minutum
Estimated density
Mode with PM ≥ 0.1
Mode with 0.05 ≤ PM < 0.1
Density
0.0
0.5
1.0
1.5
2.0
2.5
0.4
0.6
0.8
1.0
1.2
1.4
CpG o/e ratio

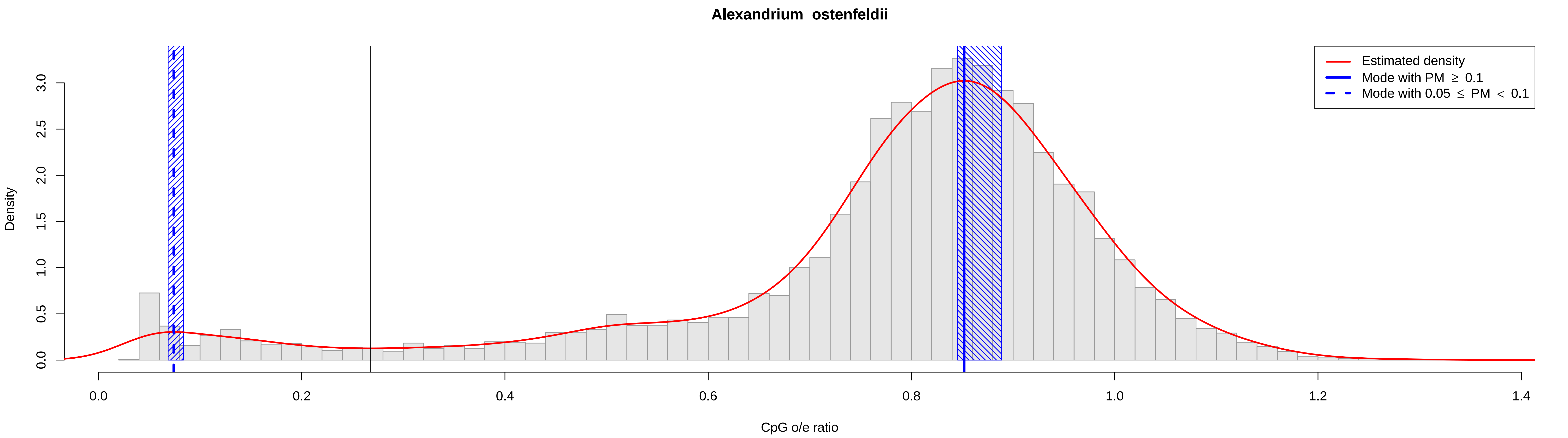

Alexandrium_ostenfeldii
Estimated density
Mode with PM ≥ 0.1
Mode with 0.05 ≤ PM < 0.1
Density
0.0
0.5
1.0
1.5
2.0
2.5
3.0
0.0
0.2
0.4
0.6
0.8
1.0
1.2
1.4
CpG o/e ratio

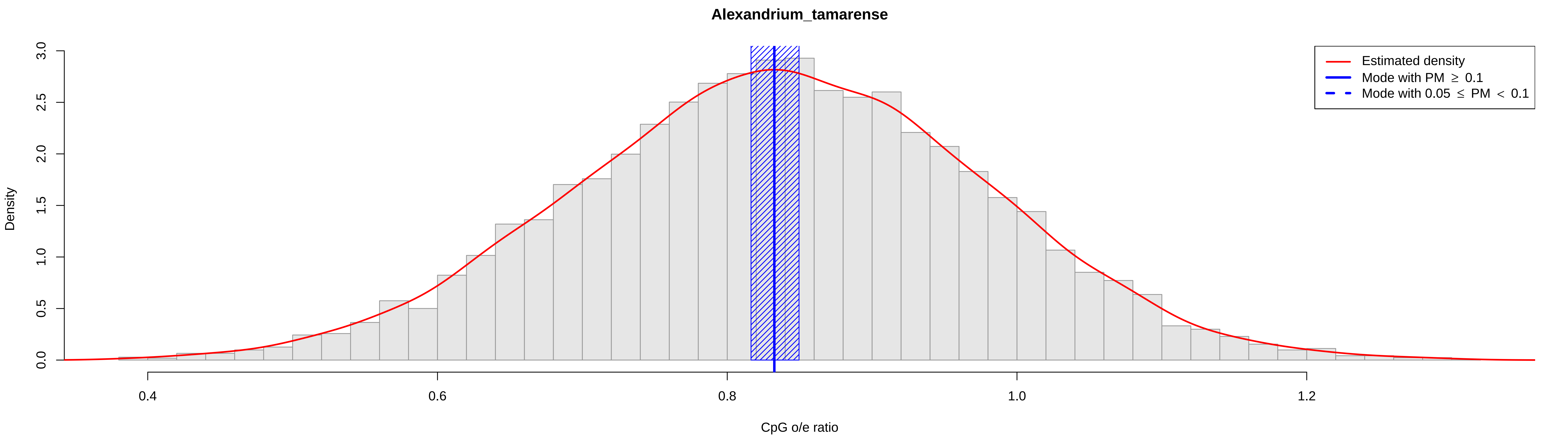
Alexandrium_tamarense
Estimated density
Mode with PM ≥ 0.1
Mode with 0.05 ≤ PM < 0.1
Density
0.0
0.5
1.0
1.5
2.0
2.5
3.0
0.4
0.6
0.8
1.0
1.2
CpG o/e ratio

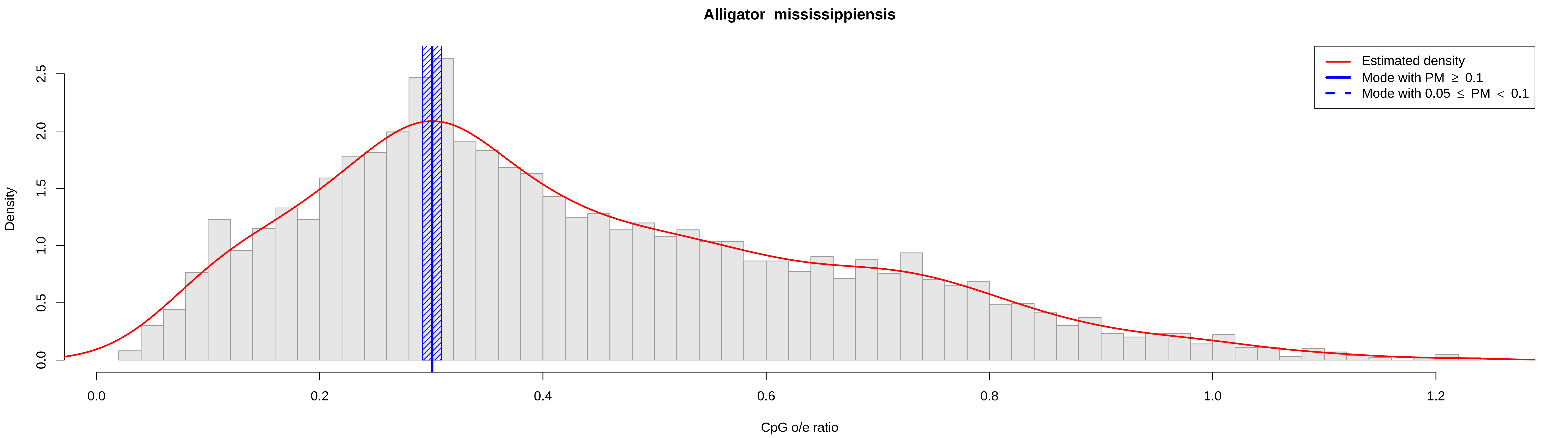

Alligator_mississippiensis
Estimated density
Mode with PM ≥ 0.1
Mode with 0.05 ≤ PM < 0.1
Density
0.0
0.5
1.0
1.5
2.0
2.5
0.0
0.2
0.4
0.6
0.8
1.0
1.2
CpG o/e ratio

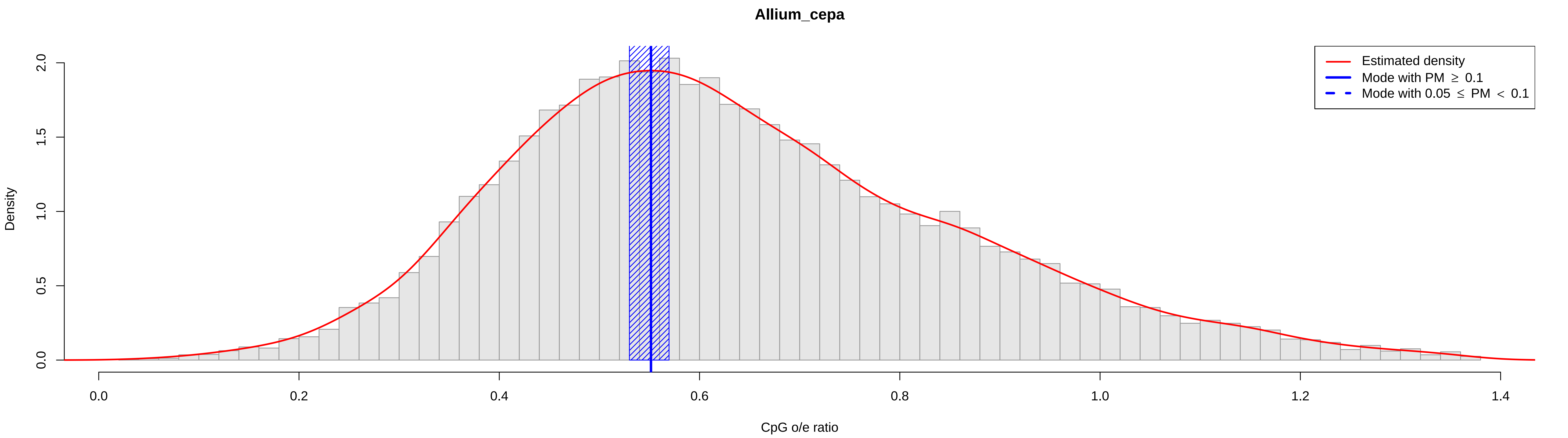

Allium_cepa
Estimated density
Mode with PM ≥ 0.1
Mode with 0.05 ≤ PM < 0.1
Density
0.0
0.5
1.0
1.5
2.0
0.0
0.2
0.4
0.6
0.8
1.0
1.2
1.4
CpG o/e ratio

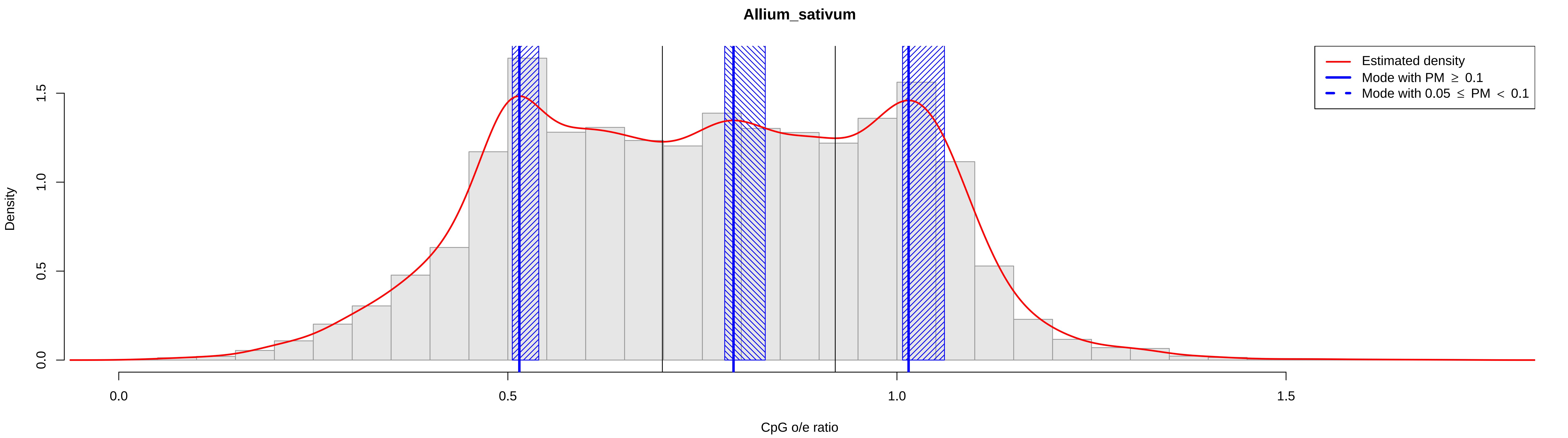
Allium_sativum
Estimated density
Mode with PM ≥ 0.1
Mode with 0.05 ≤ PM < 0.1
Density
0.0
0.5
1.0
1.5
CpG o/e ratio

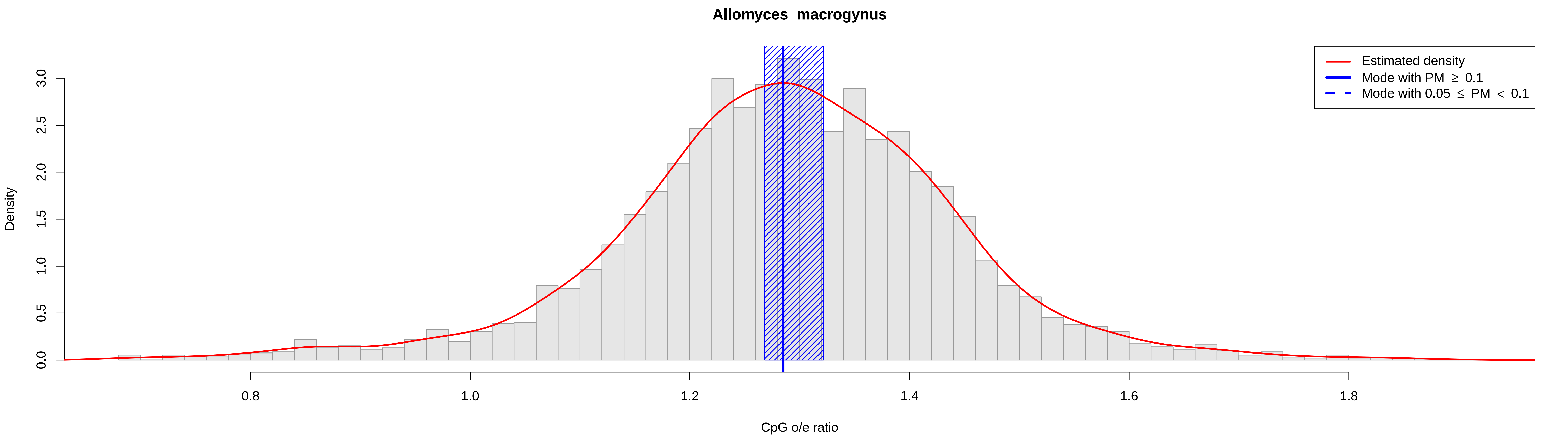

Allomyces_macrogynus
Estimated density
Mode with PM ≥ 0.1
Mode with 0.05 ≤ PM < 0.1
Density
0.0
0.5
1.0
1.5
2.0
2.5
3.0
0.8
1.0
1.2
1.4
1.6
1.8
CpG o/e ratio

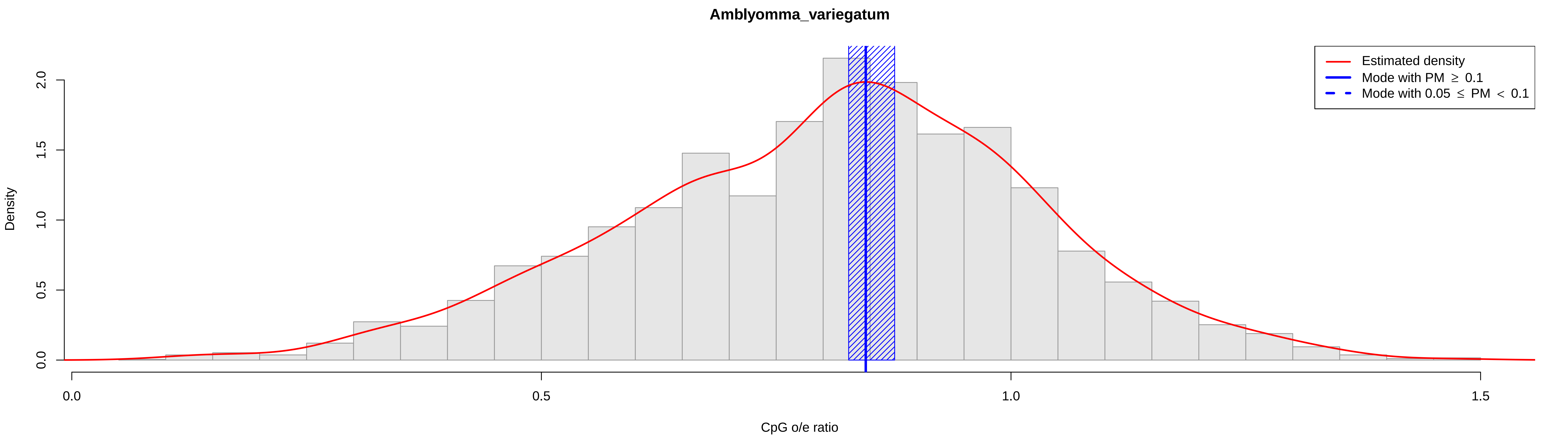

Amblyomma_variegatum
Estimated density
Mode with PM ≥ 0.1
Mode with 0.05 ≤ PM < 0.1
Density
0.0
0.5
1.0
1.5
2.0
0.0
0.5
1.0
1.5
CpG o/e ratio

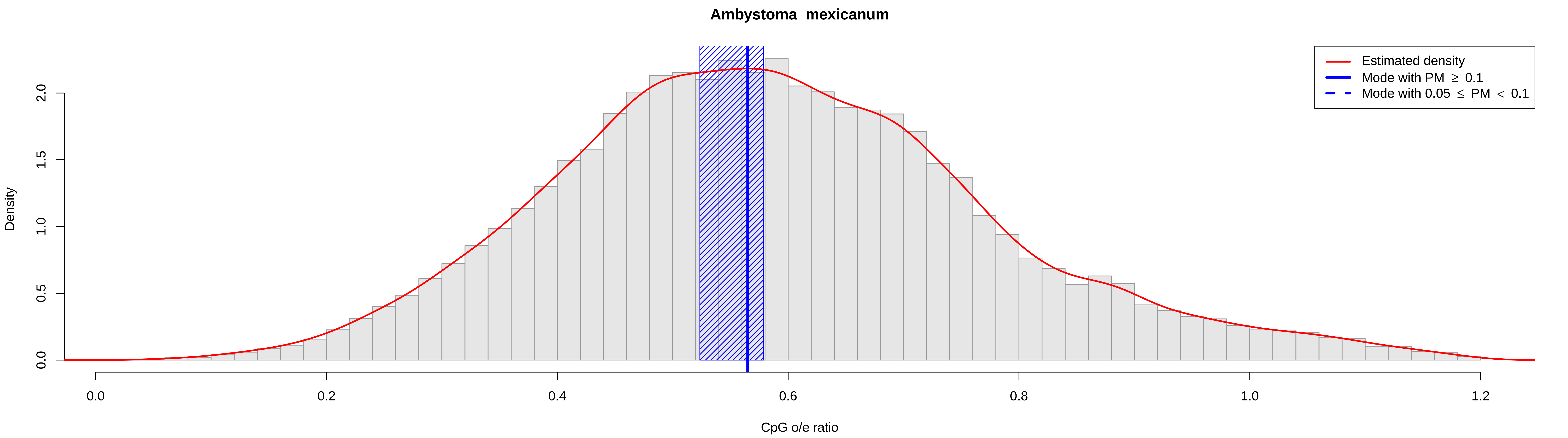

Ambystoma_mexicanum
Estimated density
Mode with PM ≥ 0.1
Mode with 0.05 ≤ PM < 0.1
Density
0.0
0.5
1.0
1.5
2.0
0.0
0.2
0.4
0.6
0.8
1.0
1.2
CpG o/e ratio

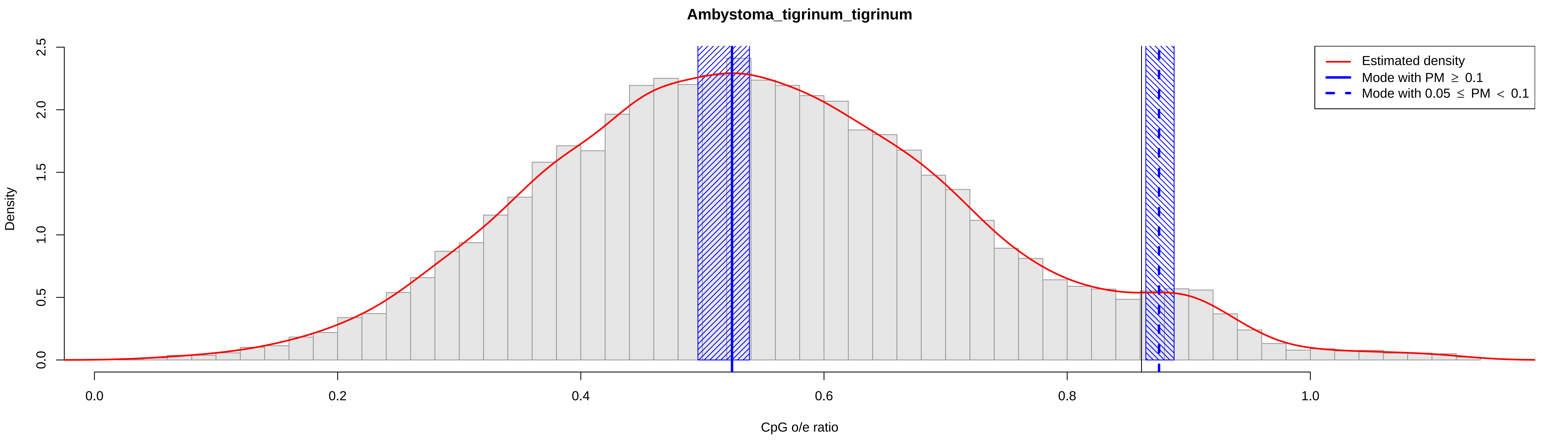

Ambystoma_tigrinum_tigrinum
Estimated density
Mode with PM ≥ 0.1
Mode with 0.05 ≤ PM < 0.1
Density
0.0
0.5
1.0
1.5
2.0
2.5
0.0
0.2
0.4
0.6
0.8
1.0
CpG o/e ratio

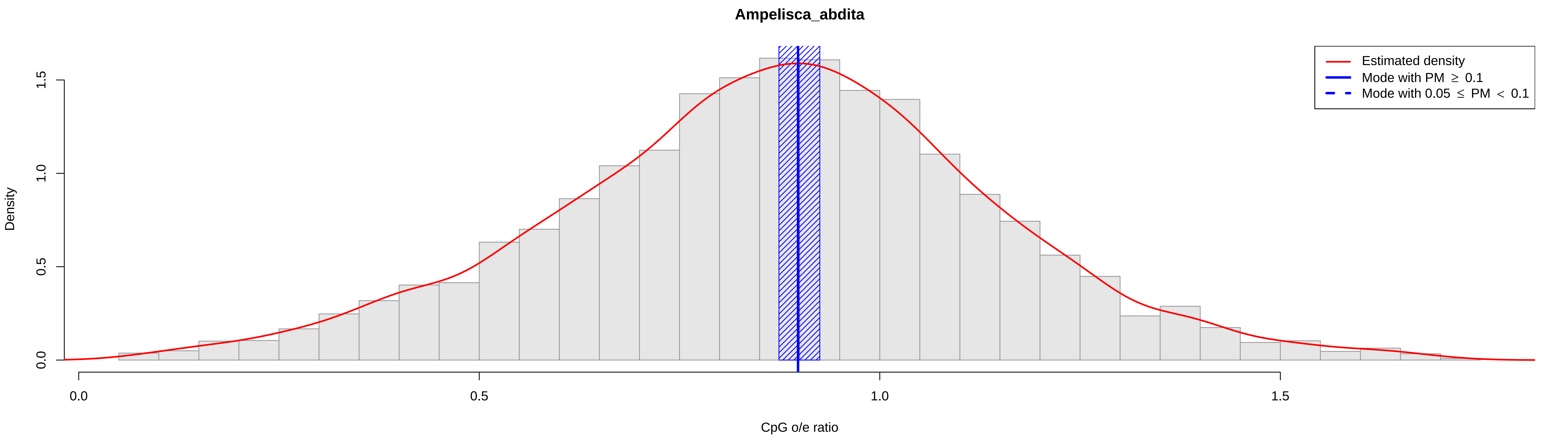
Ampelisca_abdita
Estimated density
Mode with PM ≥ 0.1
Mode with 0.05 ≤ PM < 0.1
Density
0.0
0.5
1.0
1.5
CpG o/e ratio

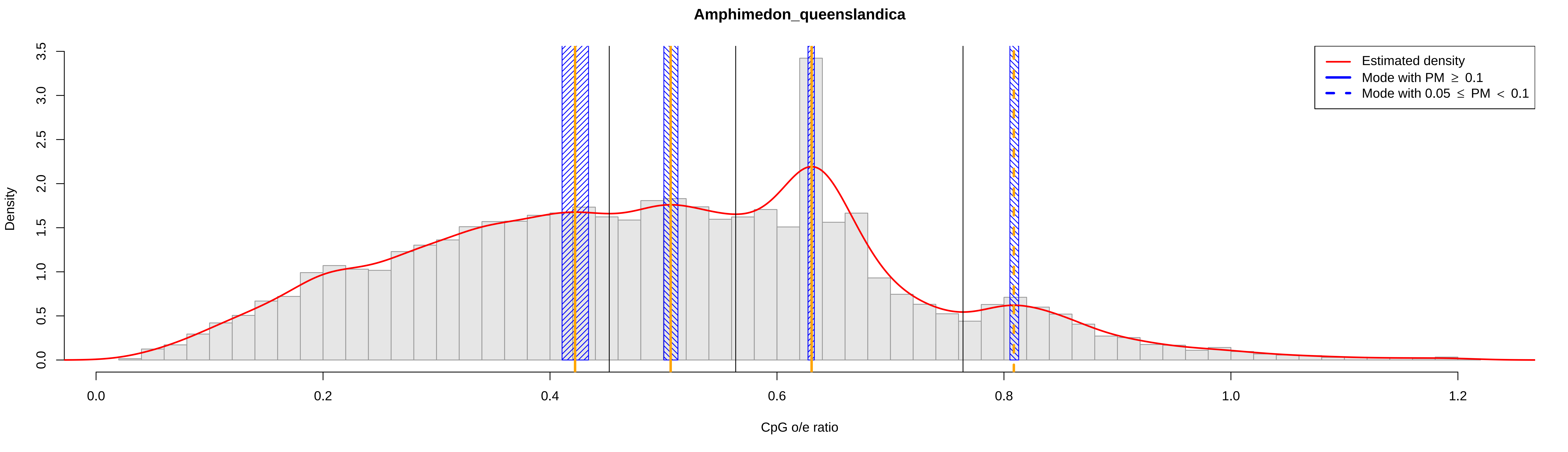
Amphimedon_queenslandica
Estimated density
Mode with PM ≥ 0.1
Mode with 0.05 ≤ PM < 0.1
Density
CpG o/e ratio
0.0
0.5
1.0
1.5
2.0
2.5
3.0
3.5
0.0
0.2
0.4
0.6
0.8
1.0
1.2

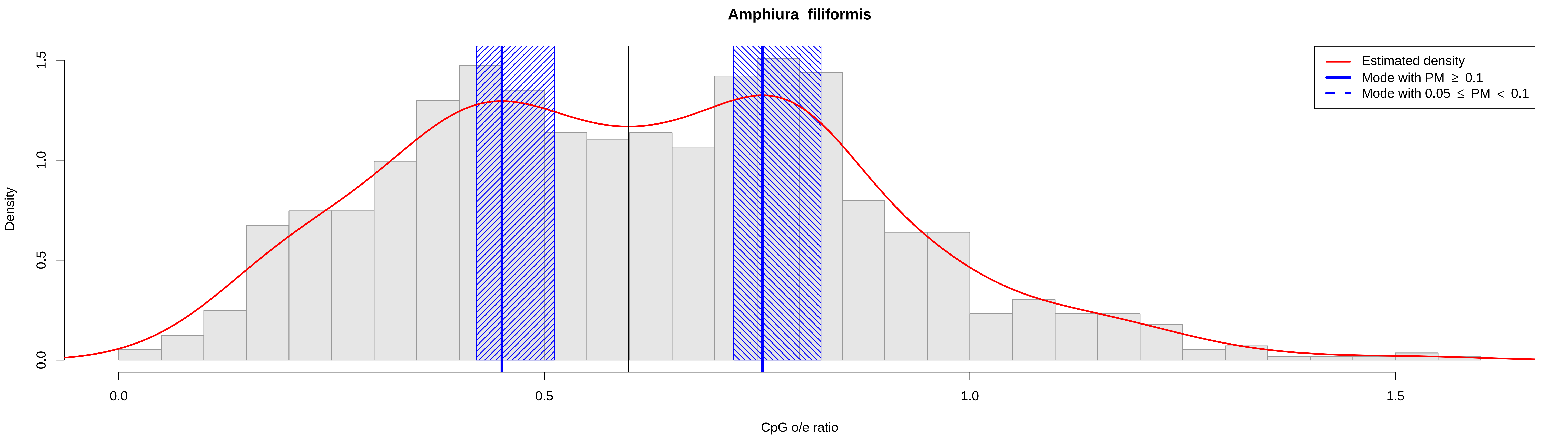
Amphiura_filiformis
Estimated density
Mode with PM ≥ 0.1
Mode with 0.05 ≤ PM < 0.1
Density
0.0
0.5
1.0
1.5
CpG o/e ratio

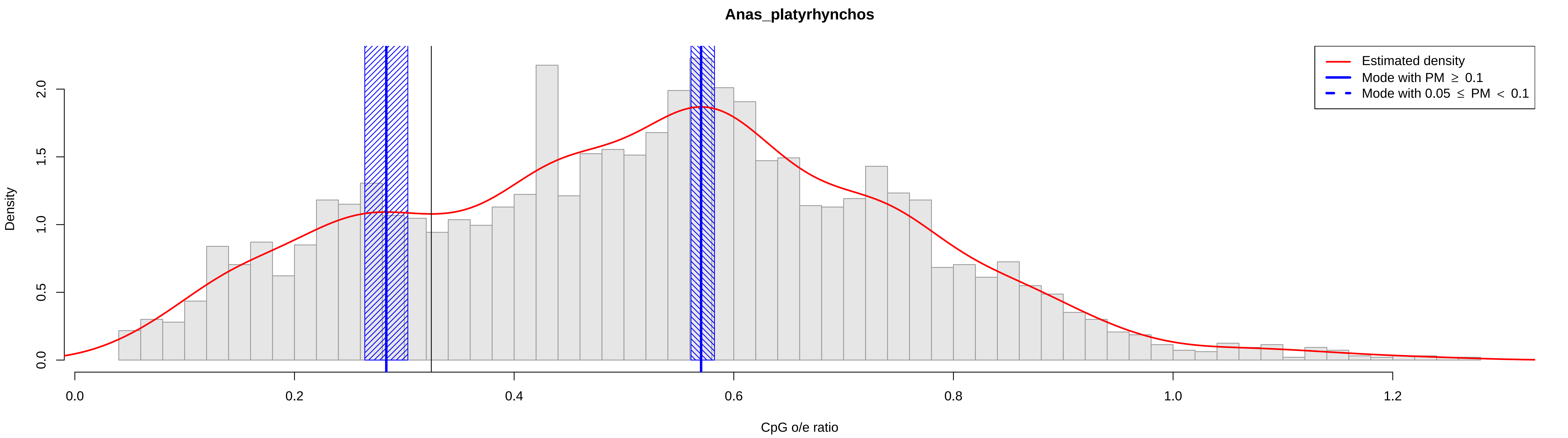

Anas_platyrhynchos
Estimated density
Mode with PM ≥ 0.1
Mode with 0.05 ≤ PM < 0.1
Density
0.0
0.5
1.0
1.5
2.0
0.0
0.2
0.4
0.6
0.8
1.0
1.2
CpG o/e ratio

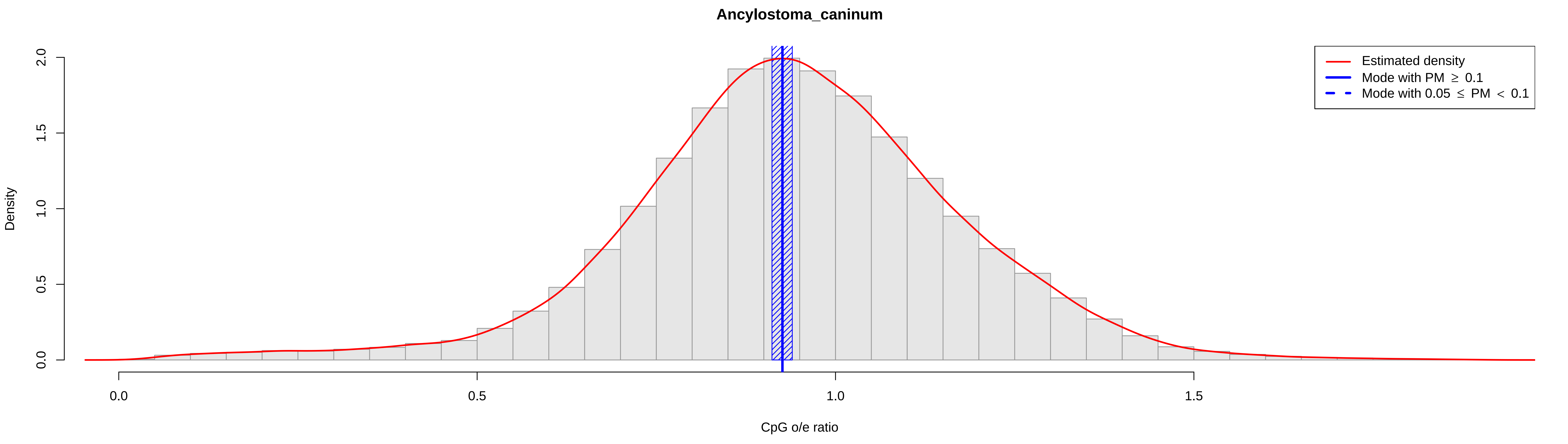

Ancylostoma_caninum
Density
CpG o/e ratio
0.0
0.5
1.0
1.5
2.0
Estimated density
Mode with PM ≥ 0.1
Mode with 0.05 ≤ PM < 0.1

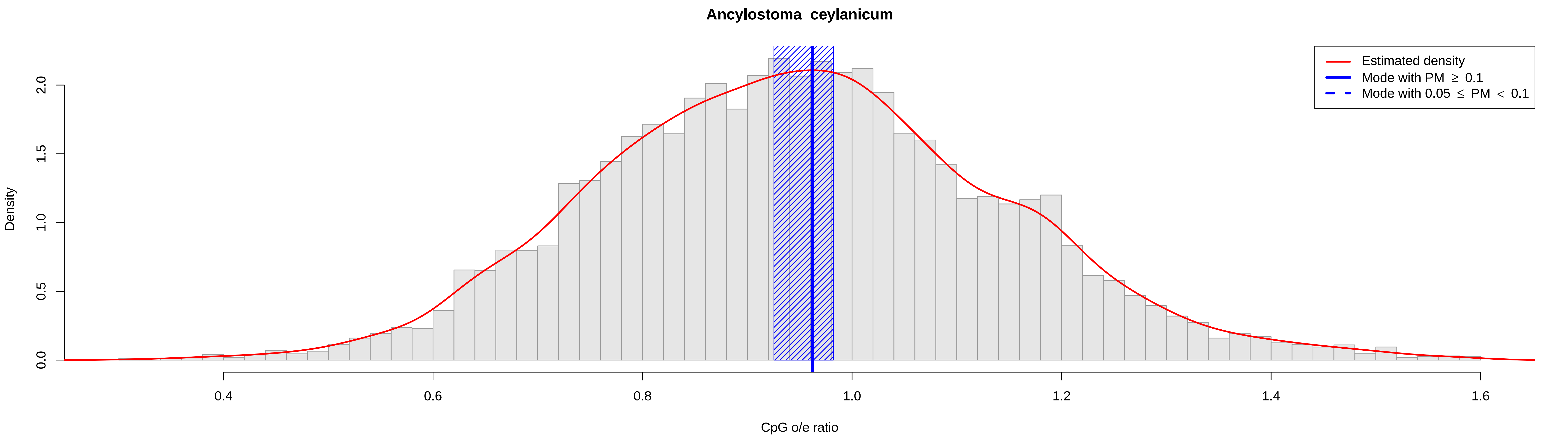
Ancylostoma_ceylanicum
Estimated density
Mode with PM ≥ 0.1
Mode with 0.05 ≤ PM < 0.1
Density
0.0
0.5
1.0
1.5
2.0
0.4
0.6
0.8
1.0
1.2
1.4
1.6
CpG o/e ratio

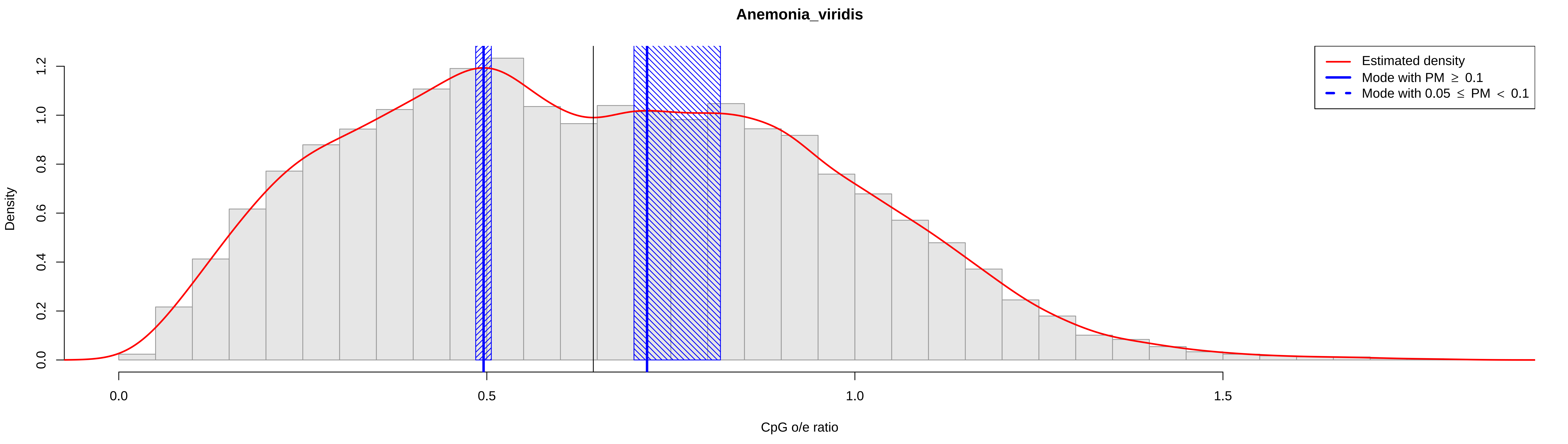

Anemonia_viridis
Estimated density
Mode with PM ≥ 0.1
Mode with 0.05 ≤ PM < 0.1
Density
0.0
0.2
0.4
0.6
0.8
1.0
1.2
0.0
0.5
1.0
1.5
CpG o/e ratio

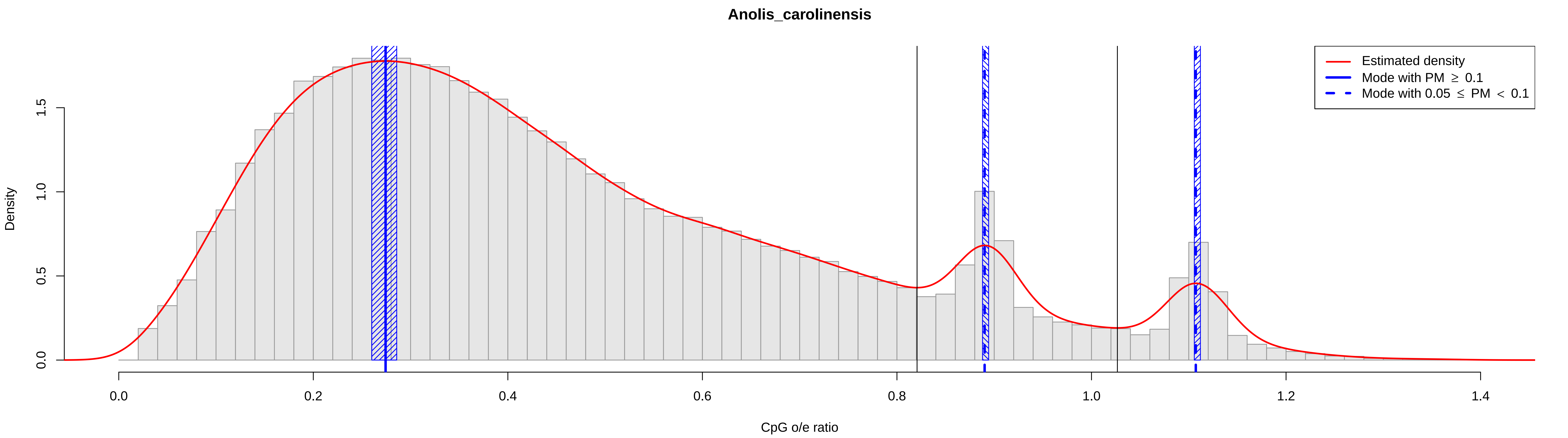

Anolis_carolinensis
Estimated density
Mode with PM ≥ 0.1
Mode with 0.05 ≤ PM < 0.1
Density
0.0
0.5
1.0
1.5
0.0
0.2
0.4
0.6
0.8
1.0
1.2
1.4
CpG o/e ratio

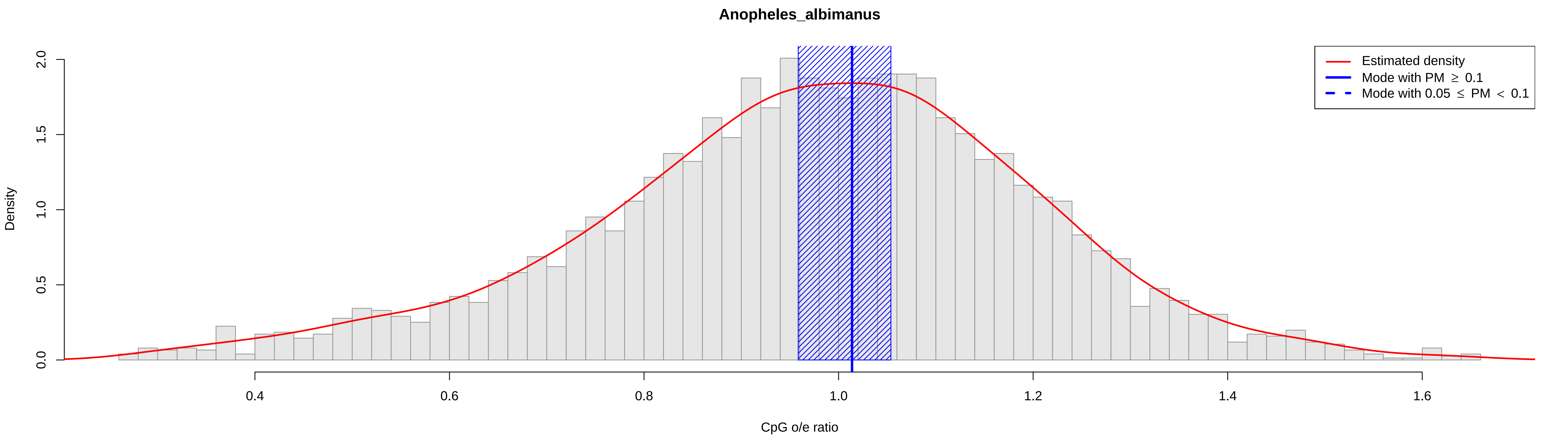

Anopheles_albimanus
Estimated density
Mode with PM ≥ 0.1
Mode with 0.05 ≤ PM < 0.1
Density
0.0
0.5
1.0
1.5
2.0
0.4
0.6
0.8
1.0
1.2
1.4
1.6
CpG o/e ratio

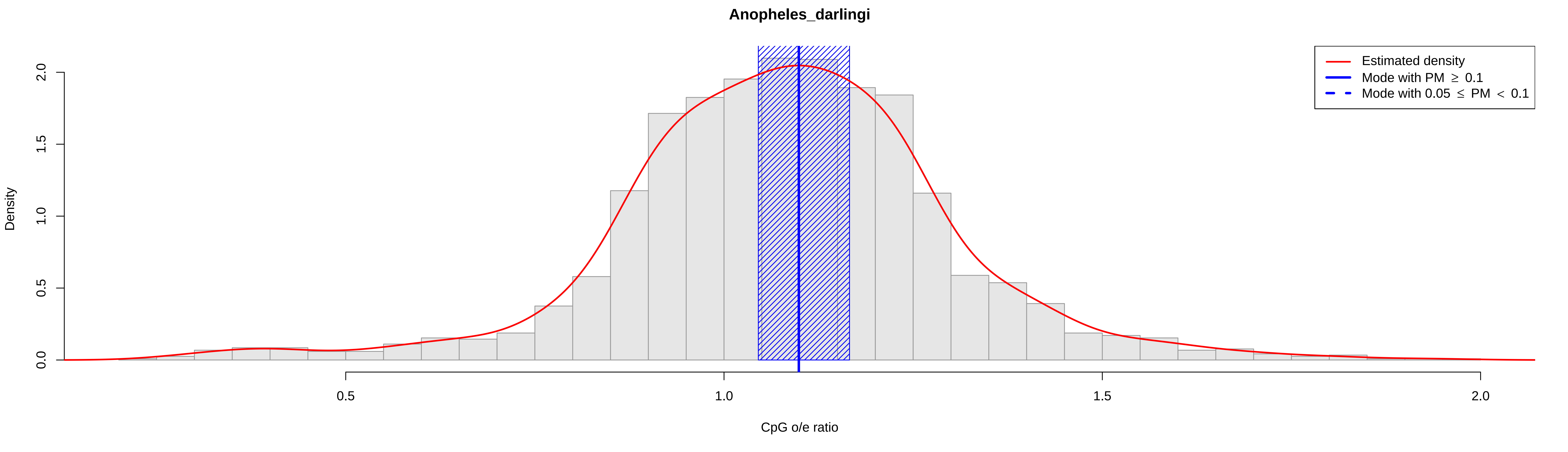

Anopheles_darlingi
Estimated density
Mode with PM ≥ 0.1
Mode with 0.05 ≤ PM < 0.1
Density
0.0
0.5
1.0
1.5
2.0
0.5
1.0
1.5
2.0
CpG o/e ratio

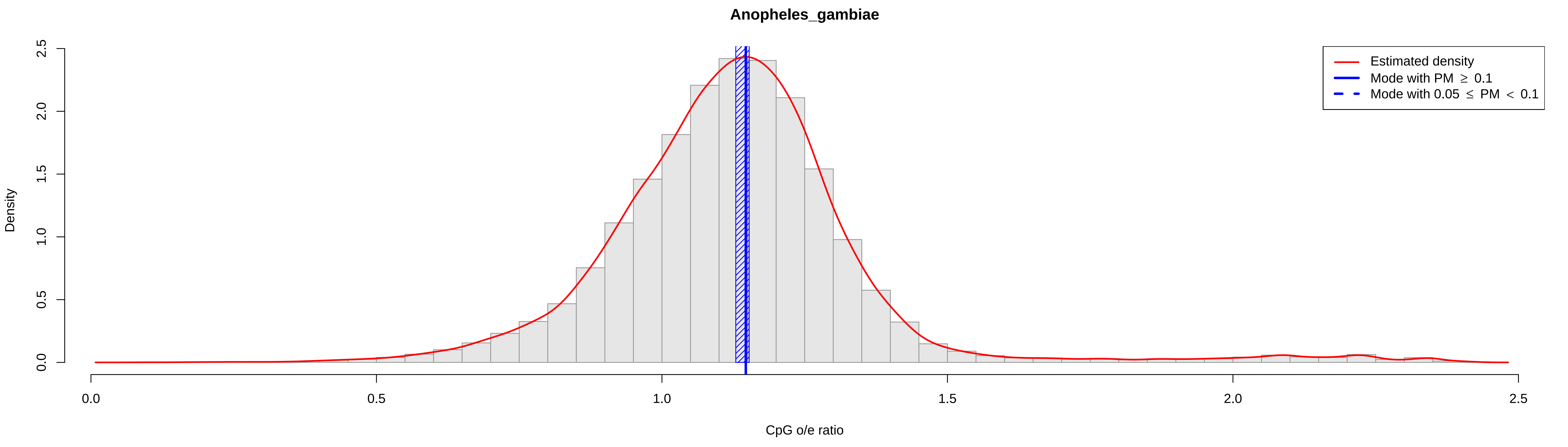

Anopheles_gambiae
Density
CpG o/e ratio
0.0
0.5
1.0
1.5
2.0
2.5
Estimated density
Mode with PM ≥ 0.1
Mode with 0.05 ≤ PM < 0.1

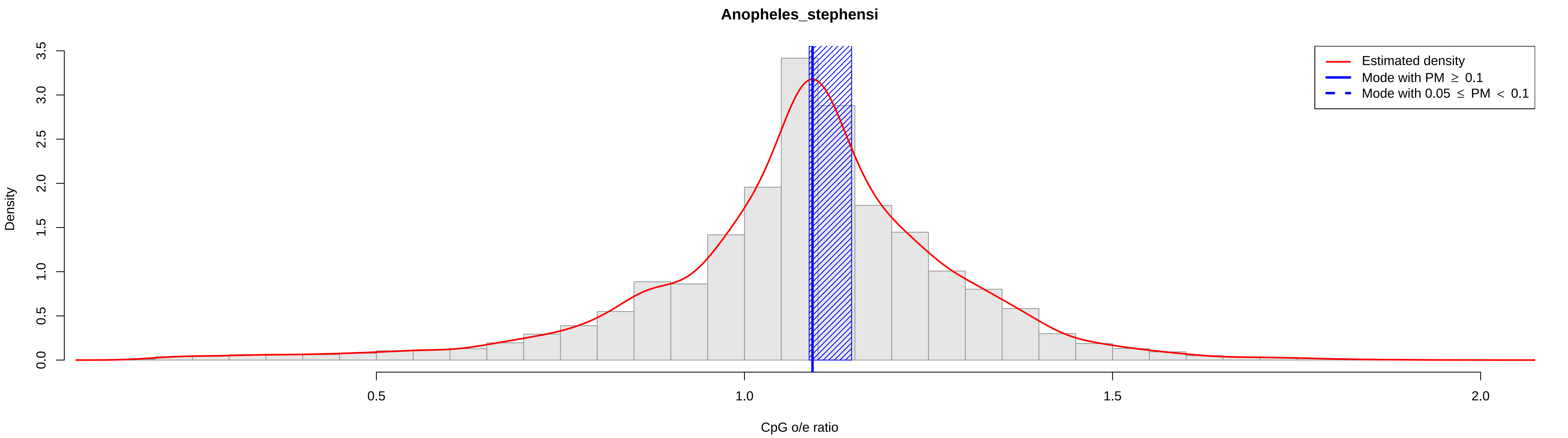
Anopheles_stephensi
Density
0.0
0.5
1.0
1.5
2.0
2.5
3.0
3.5
0.5
1.0
1.5
2.0
CpG o/e ratio
Estimated density
Mode with PM ≥ 0.1
Mode with 0.05 ≤ PM < 0.1

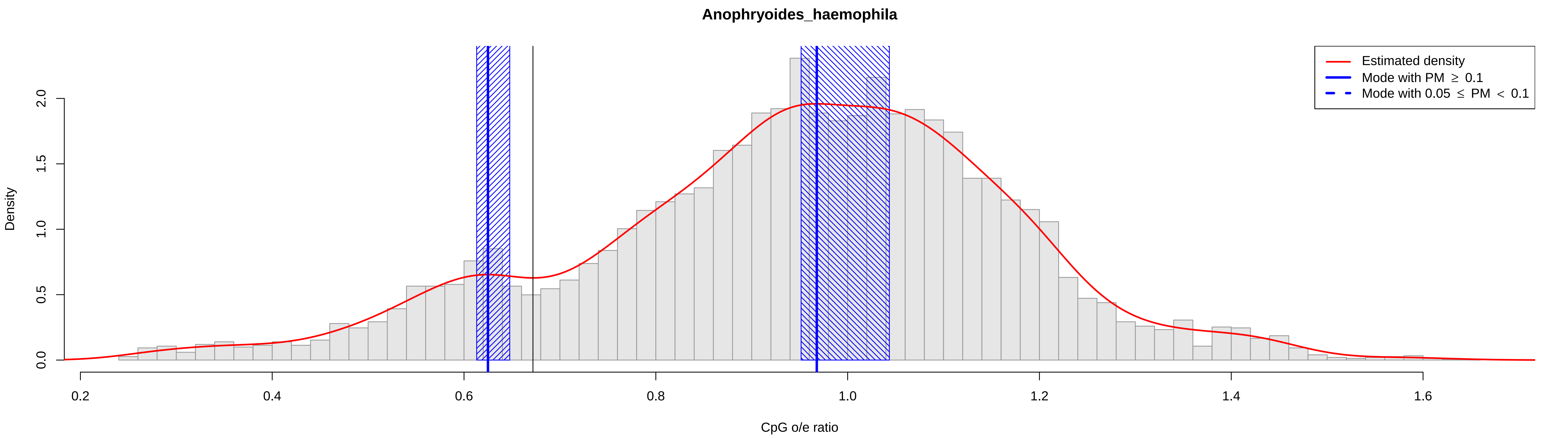
Anophryoides_haemophila
Estimated density
Mode with PM ≥ 0.1
Mode with 0.05 ≤ PM < 0.1
Density
0.0
0.5
1.0
1.5
2.0
0.2
0.4
0.6
0.8
1.0
1.2
1.4
1.6
CpG o/e ratio

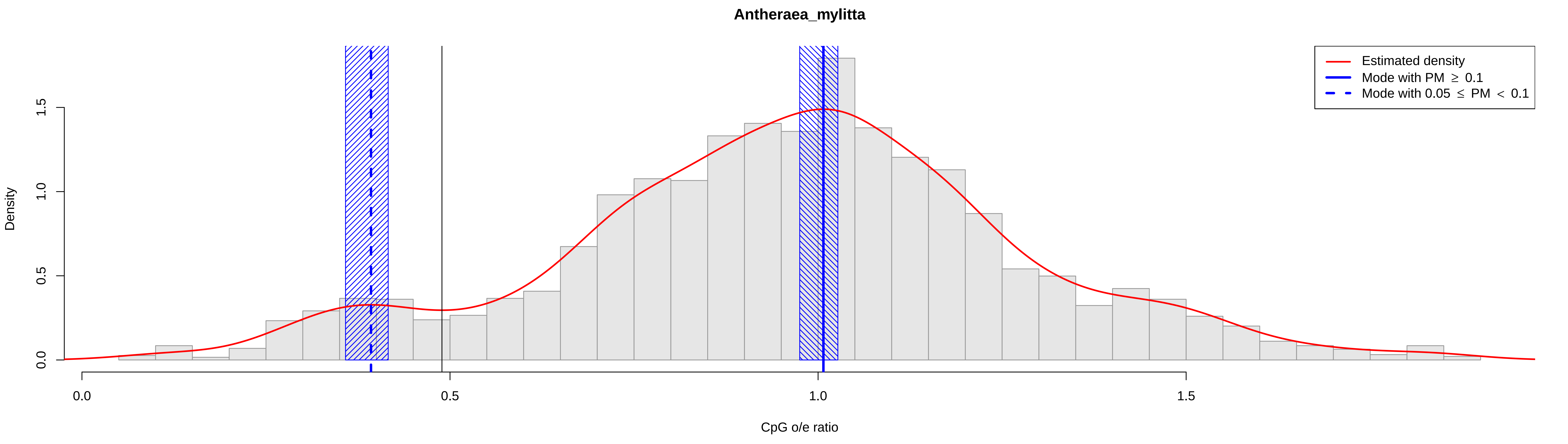

Antheraea_mylitta
Estimated density
Mode with PM ≥ 0.1
Mode with 0.05 ≤ PM < 0.1
Density
0.0
0.5
1.0
1.5
0.0
0.5
1.0
1.5
CpG o/e ratio

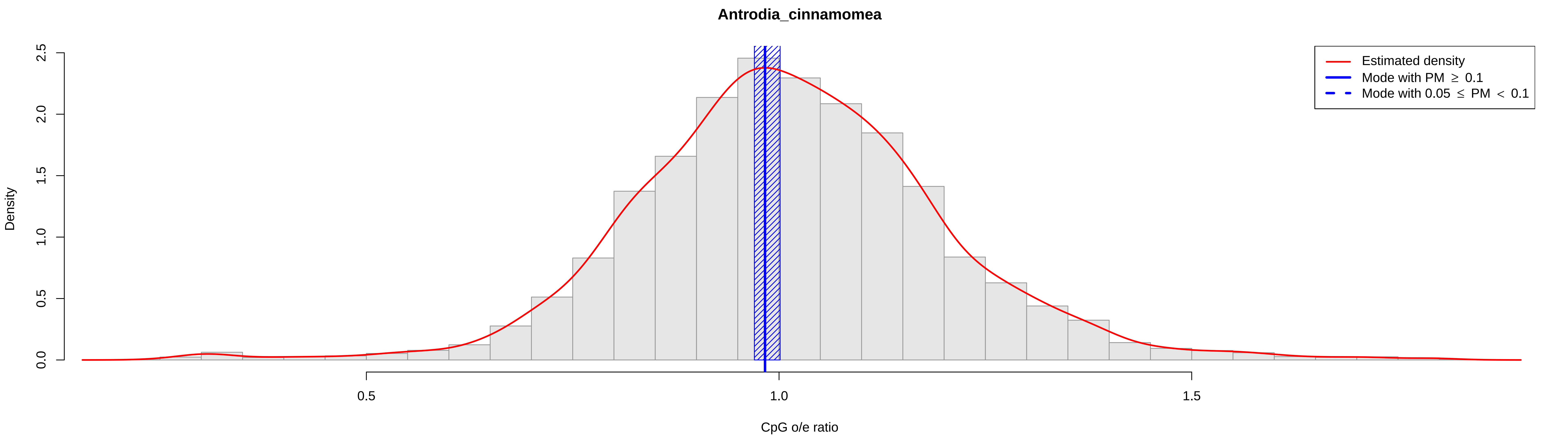
Antrodia_cinnamomea
Density
CpG o/e ratio
0.0
0.5
1.0
1.5
2.0
2.5
0.5
1.0
1.5
Estimated density
Mode with PM ≥ 0.1
Mode with 0.05 ≤ PM < 0.1

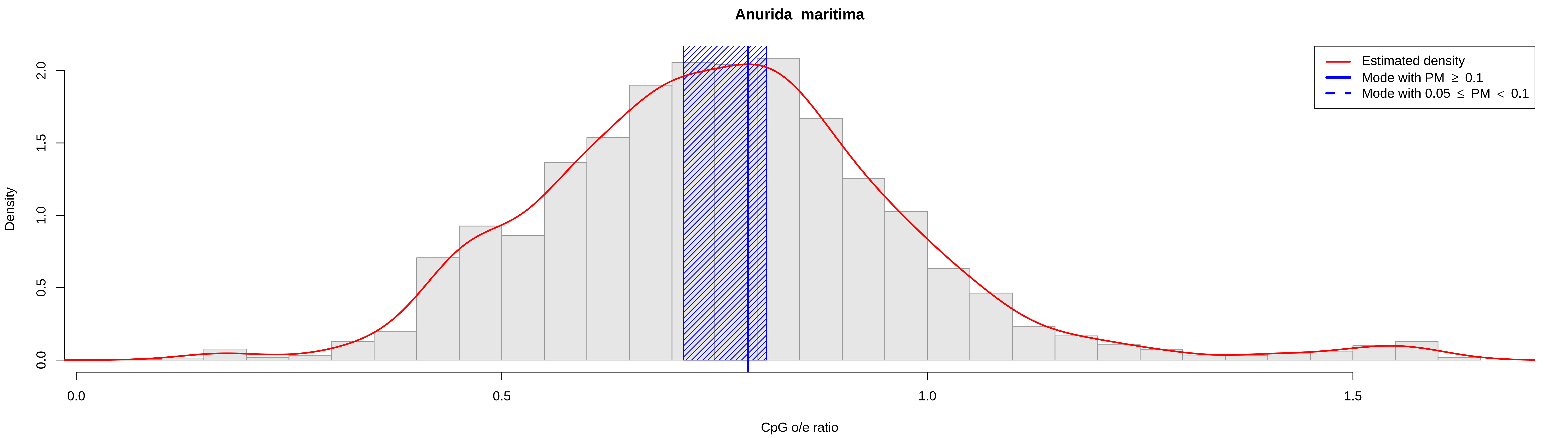

Anurida_maritima
Estimated density
Mode with PM ≥ 0.1
Mode with 0.05 ≤ PM < 0.1
Density
0.0
0.5
1.0
1.5
2.0
0.0
0.5
1.0
1.5
CpG o/e ratio

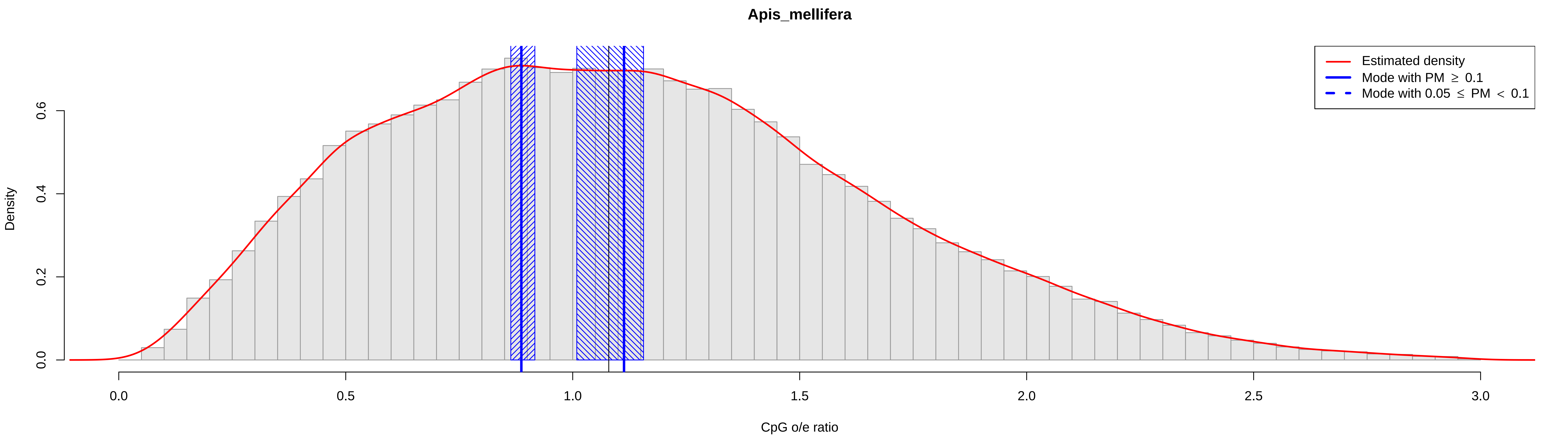

Apis_mellifera
Estimated density
Mode with PM ≥ 0.1
Mode with 0.05 ≤ PM < 0.1
Density
0.0
0.2
0.4
0.6
0.0
0.5
1.0
1.5
2.0
2.5
3.0
CpG o/e ratio

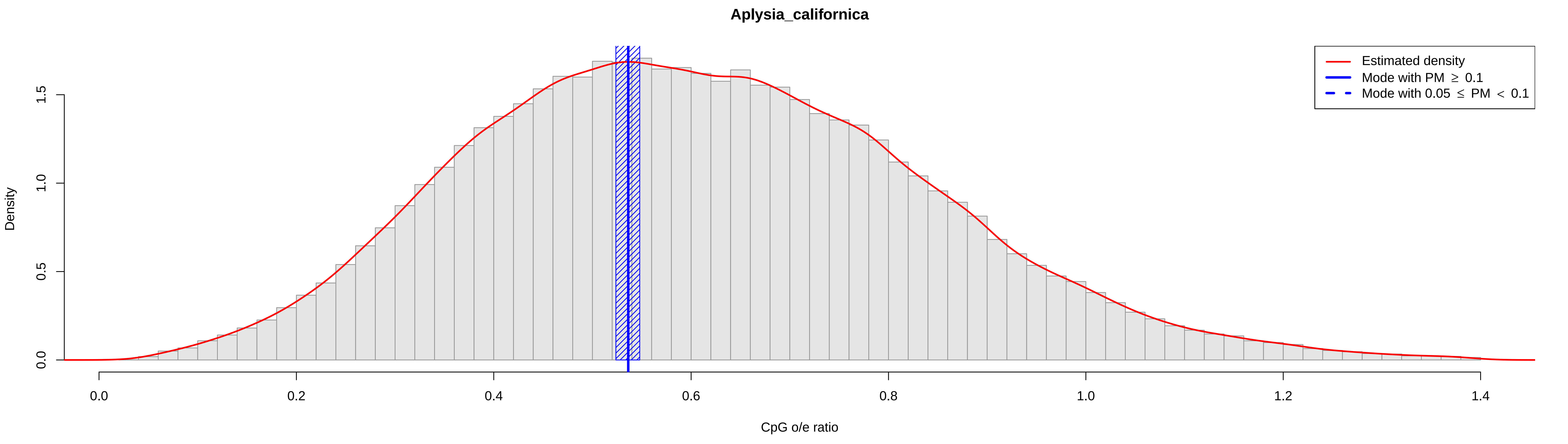

Aplysia_californica
Estimated density
Mode with PM ≥ 0.1
Mode with 0.05 ≤ PM < 0.1
Density
0.0
0.5
1.0
1.5
0.0
0.2
0.4
0.6
0.8
1.0
1.2
1.4
CpG o/e ratio

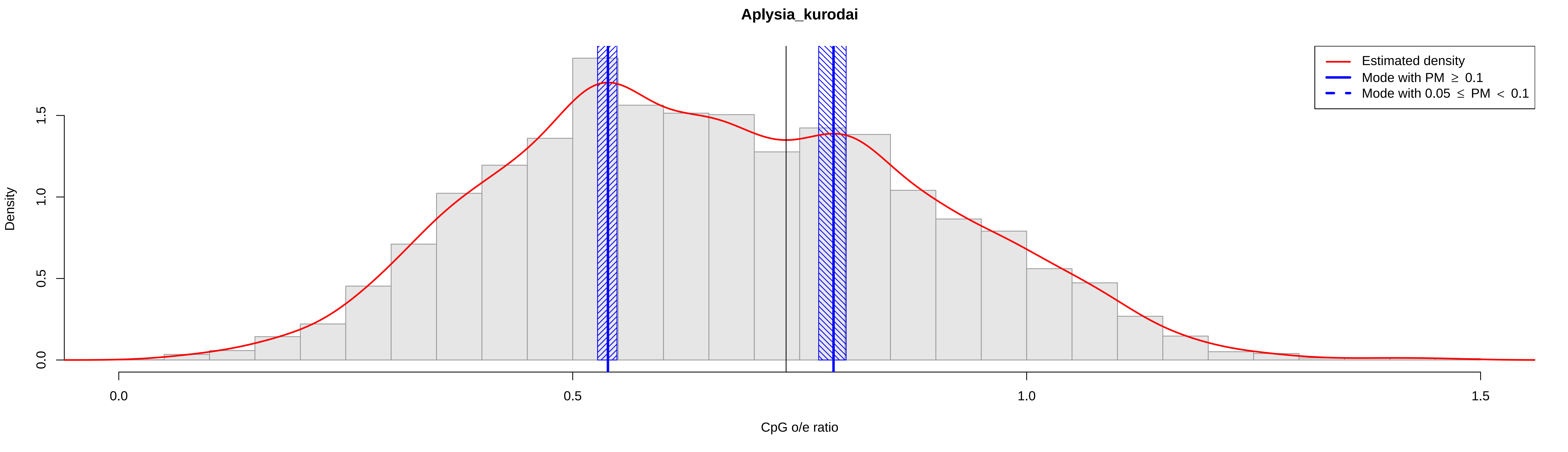

Aplysia_kurodai
Estimated density
Mode with PM ≥ 0.1
Mode with 0.05 ≤ PM < 0.1
Density
0.0
0.5
1.0
1.5
0.0
0.5
1.0
1.5
CpG o/e ratio

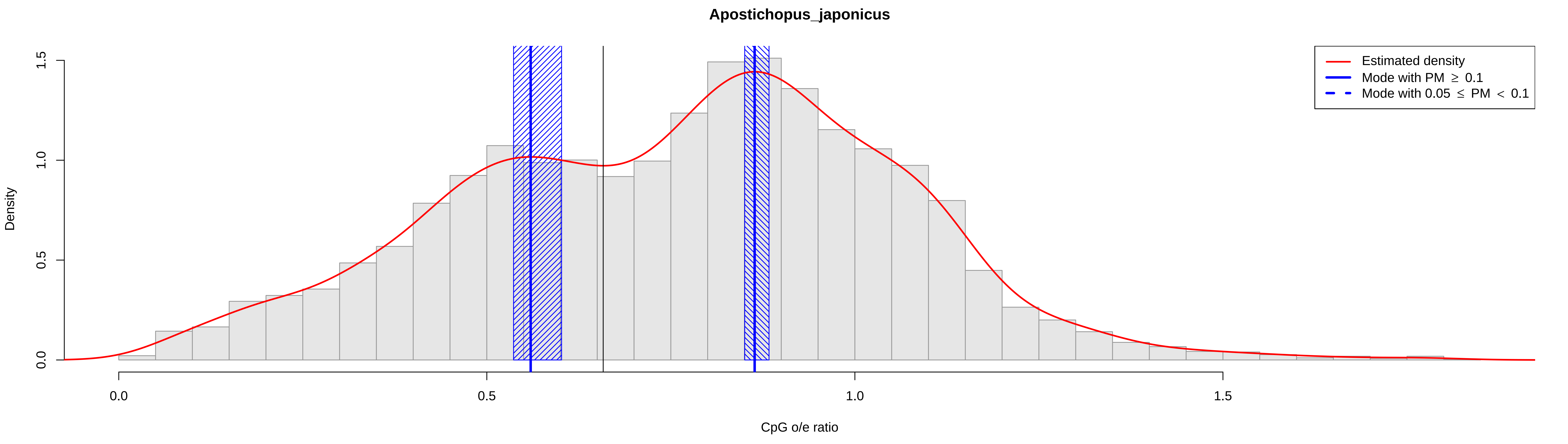
Apostichopus_japonicus
Estimated density
Mode with PM ≥ 0.1
Mode with 0.05 ≤ PM < 0.1
Density
0.0
0.5
1.0
1.5
0.0
0.5
1.0
1.5
CpG o/e ratio

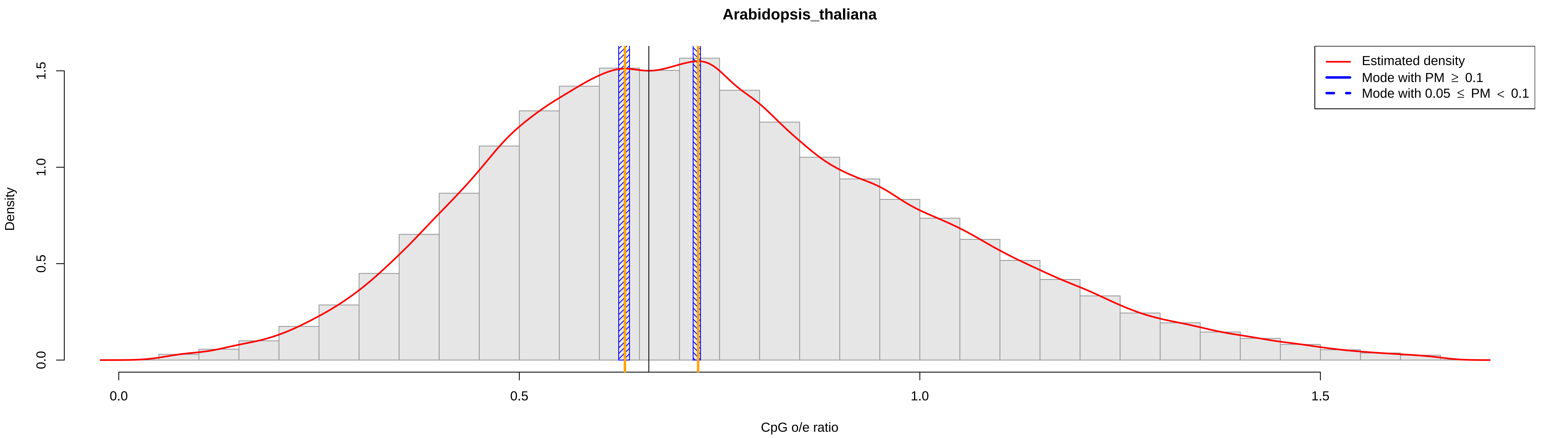
Arabidopsis_thaliana
Estimated density
Mode with PM ≥ 0.1
Mode with 0.05 ≤ PM < 0.1
Density
0.0
0.5
1.0
1.5
CpG o/e ratio
0.0
0.5
1.0
1.5

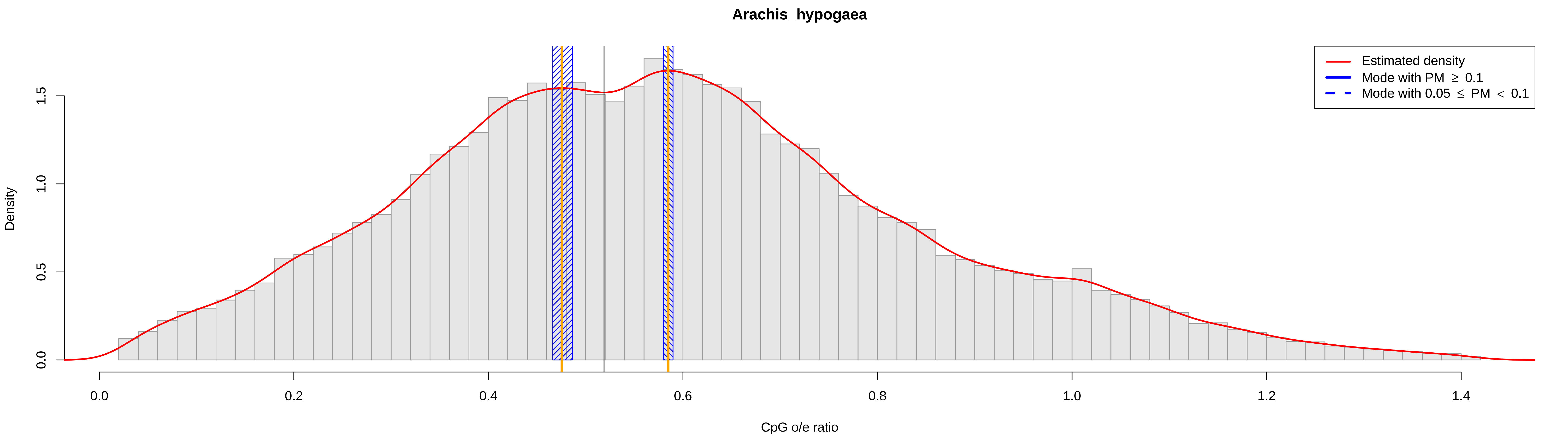

Arachis_hypogaea
Estimated density
Mode with PM ≥ 0.1
Mode with 0.05 ≤ PM < 0.1
Density
0.0
0.5
1.0
1.5
0.0
0.2
0.4
0.6
0.8
1.0
1.2
1.4
CpG o/e ratio

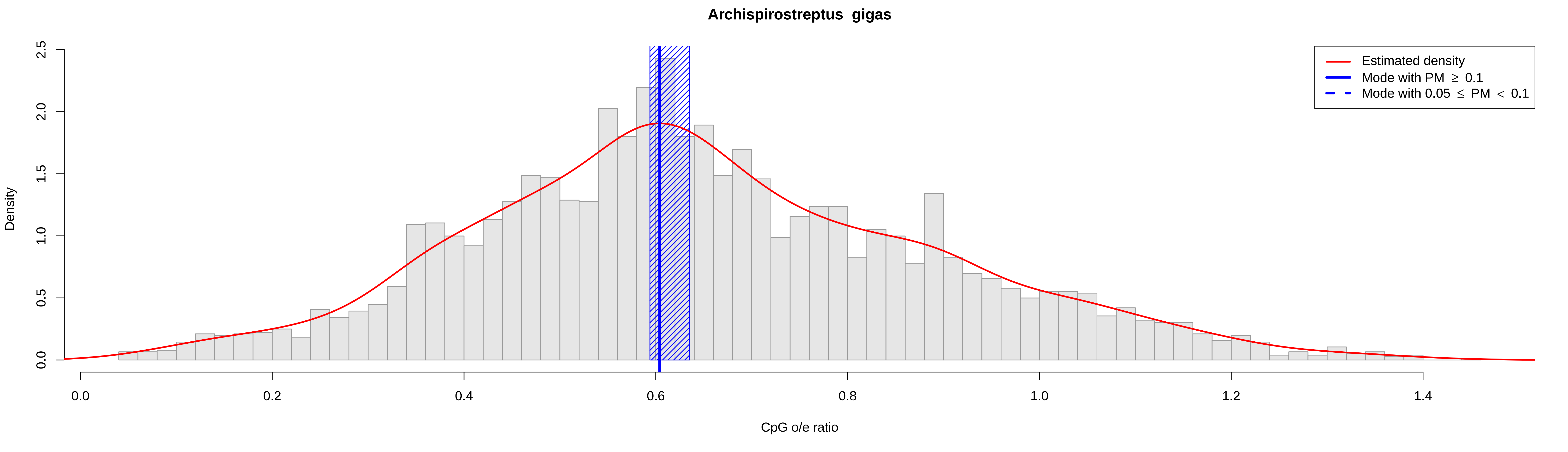

Archispirostreptus_gigas
Estimated density
Mode with PM ≥ 0.1
Mode with 0.05 ≤ PM < 0.1
Density
CpG o/e ratio
0.0
0.5
1.0
1.5
2.0
2.5
0.0
0.2
0.4
0.6
0.8
1.0
1.2
1.4

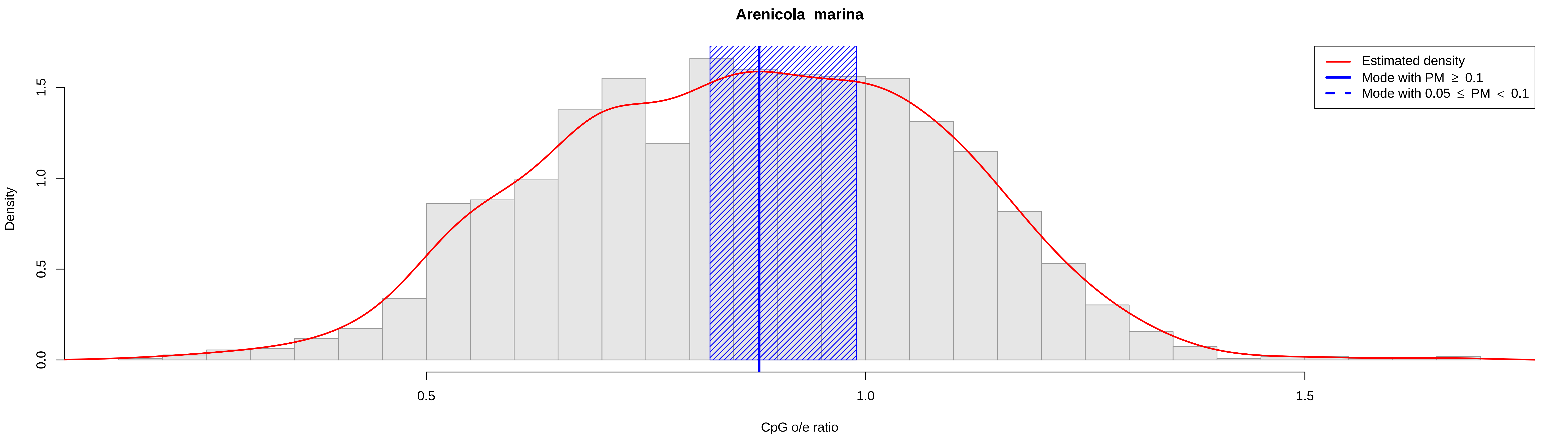

Arenicola_marina
Estimated density
Mode with PM ≥ 0.1
Mode with 0.05 ≤ PM < 0.1
Density
0.0
0.5
1.0
1.5
0.5
1.0
1.5
CpG o/e ratio

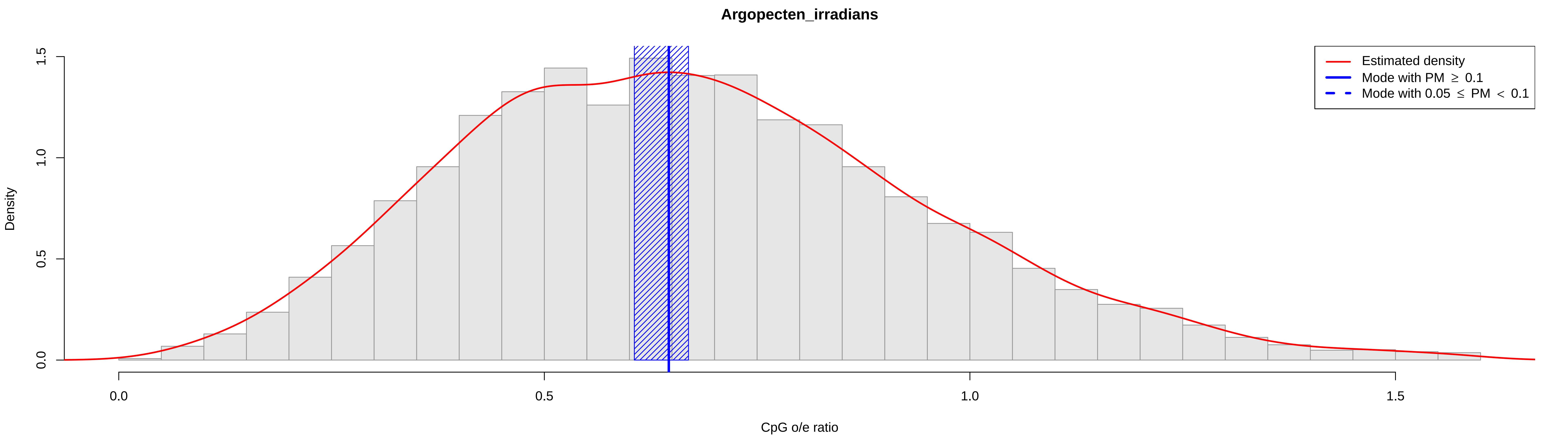
Argopecten_irradians
Estimated density
Mode with PM ≥ 0.1
Mode with 0.05 ≤ PM < 0.1
Density
0.0
0.5
1.0
1.5
CpG o/e ratio

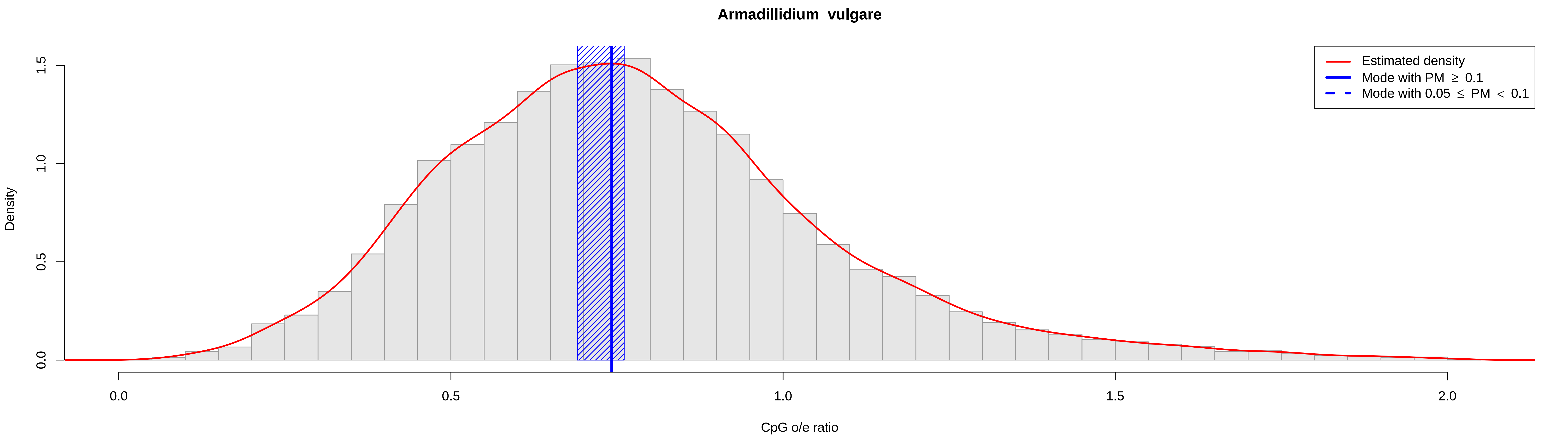
Armadillidium_vulgare
Estimated density
Mode with PM ≥ 0.1
Mode with 0.05 ≤ PM < 0.1
Density
0.0
0.5
1.0
1.5
0.0
0.5
1.0
1.5
2.0
CpG o/e ratio

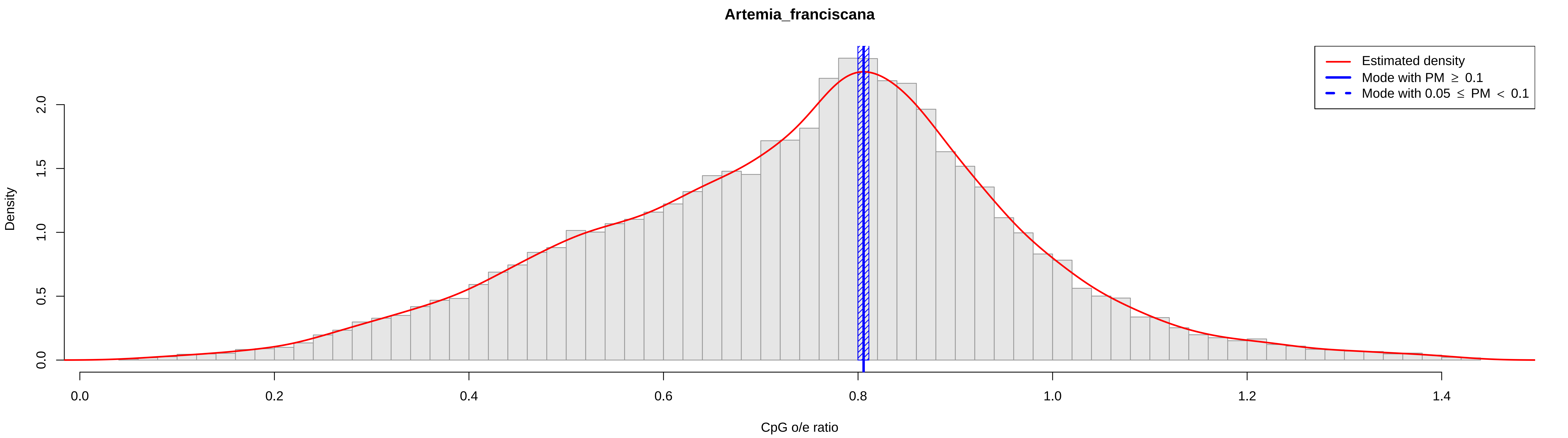

Artemia_franciscana
Estimated density
Mode with PM ≥ 0.1
Mode with 0.05 ≤ PM < 0.1
Density
0.0
0.5
1.0
1.5
2.0
0.0
0.2
0.4
0.6
0.8
1.0
1.2
1.4
CpG o/e ratio

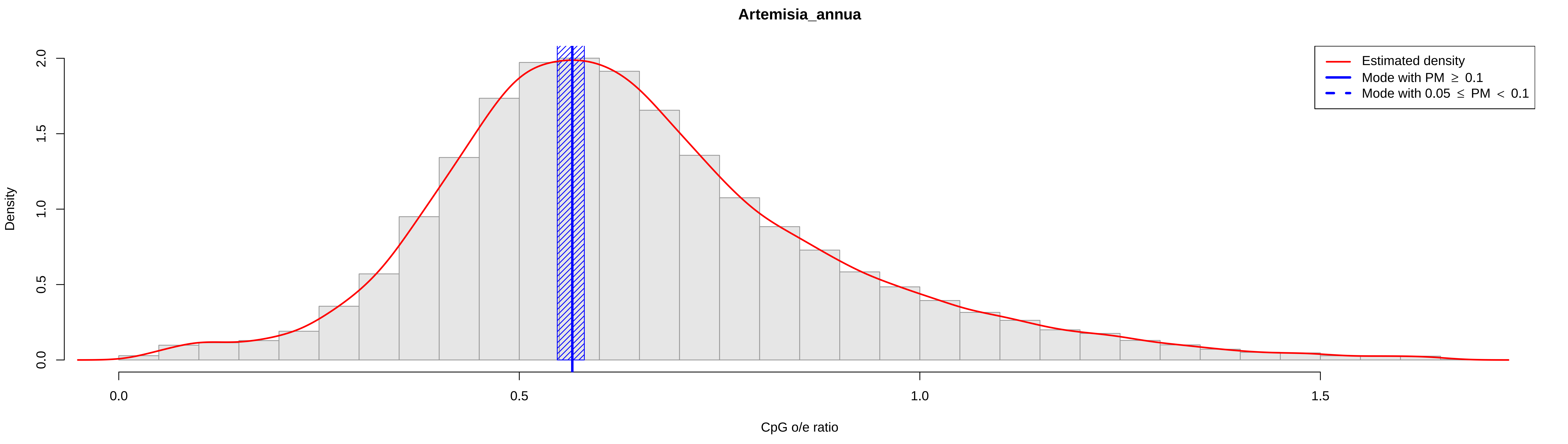

Artemisia_annua
Density
0.0
0.5
1.0
1.5
2.0
CpG o/e ratio
Estimated density
Mode with PM ≥ 0.1
Mode with 0.05 ≤ PM < 0.1

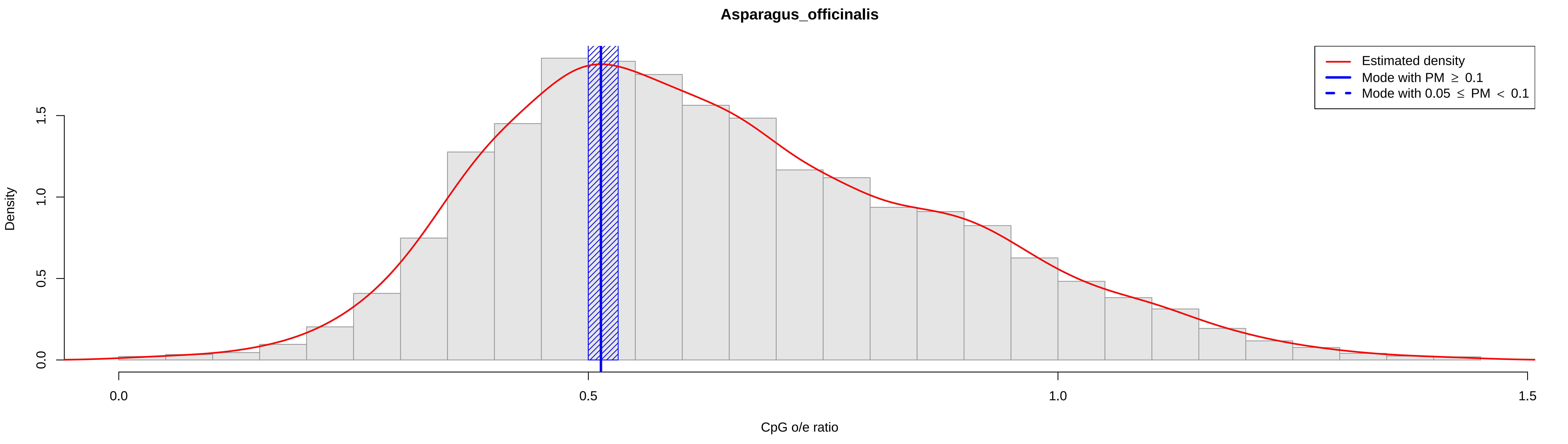

Asparagus_officinalis
Estimated density
Mode with PM ≥ 0.1
Mode with 0.05 ≤ PM < 0.1
Density
0.0
0.5
1.0
1.5
0.0
0.5
1.0
1.5
CpG o/e ratio

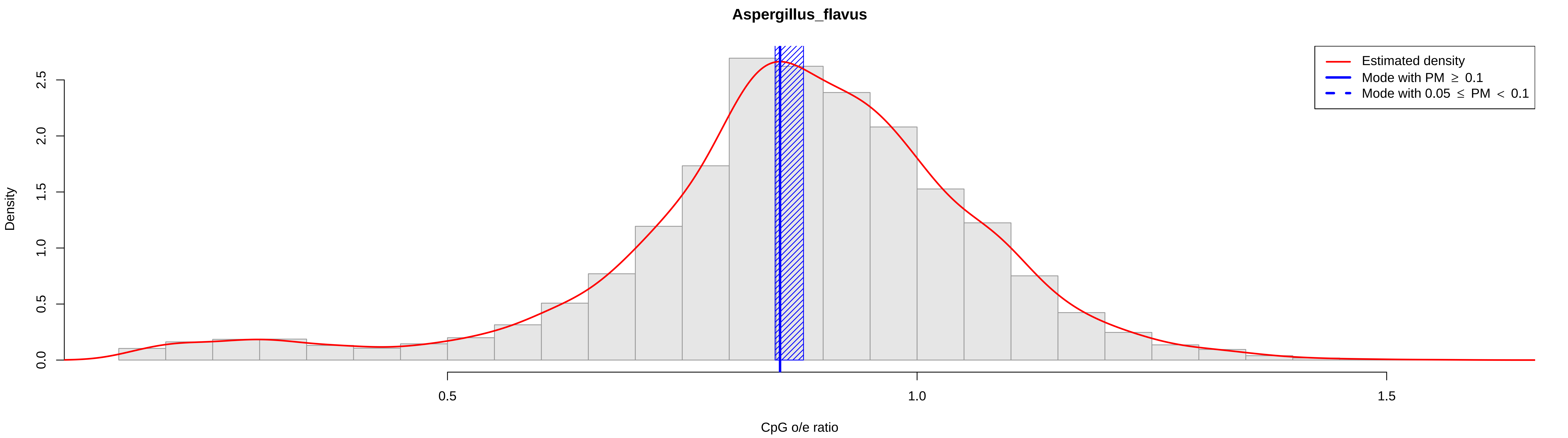

Aspergillus_flavus
Estimated density
Mode with PM ≥ 0.1
Mode with 0.05 ≤ PM < 0.1
Density
0.0
0.5
1.0
1.5
2.0
2.5
0.5
1.0
1.5
CpG o/e ratio

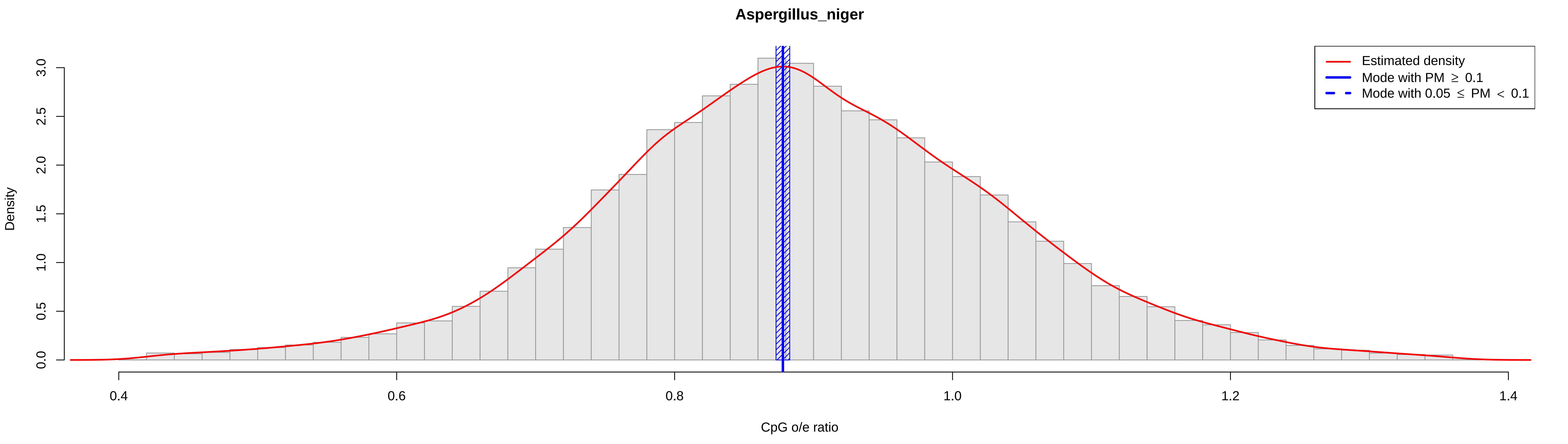
Aspergillus_niger
Estimated density
Mode with PM ≥ 0.1
Mode with 0.05 ≤ PM < 0.1
Density
0.0
0.5
1.0
1.5
2.0
2.5
3.0
0.4
0.6
0.8
1.0
1.2
1.4
CpG o/e ratio

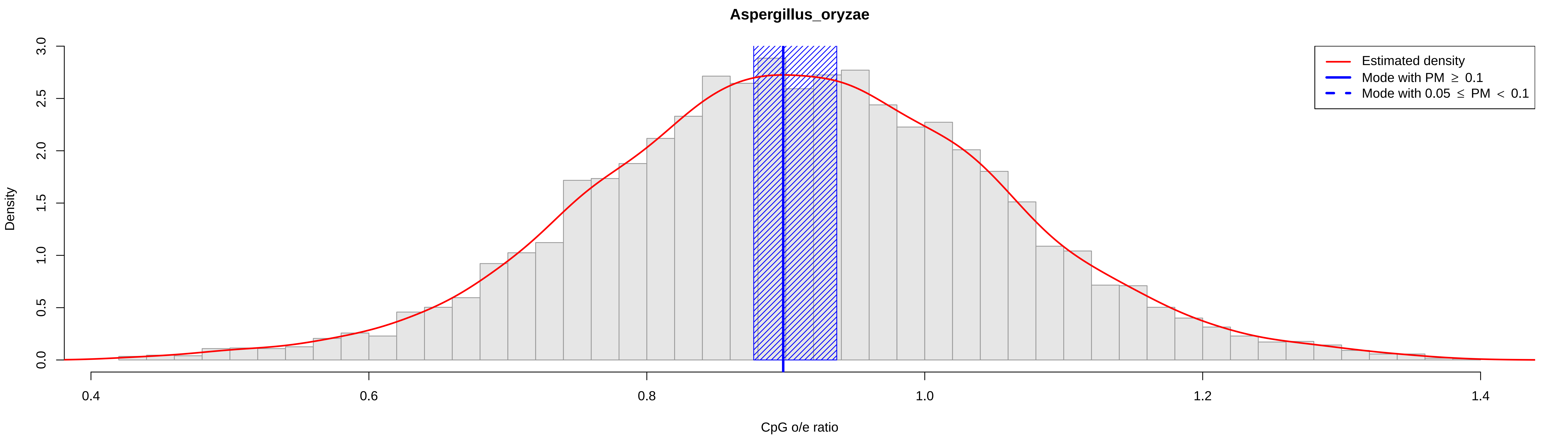

Aspergillus_oryzae
Density
0.0
0.5
1.0
1.5
2.0
2.5
3.0
0.4
0.6
0.8
1.0
1.2
1.4
CpG o/e ratio
Estimated density
Mode with PM ≥ 0.1
Mode with 0.05 ≤ PM < 0.1

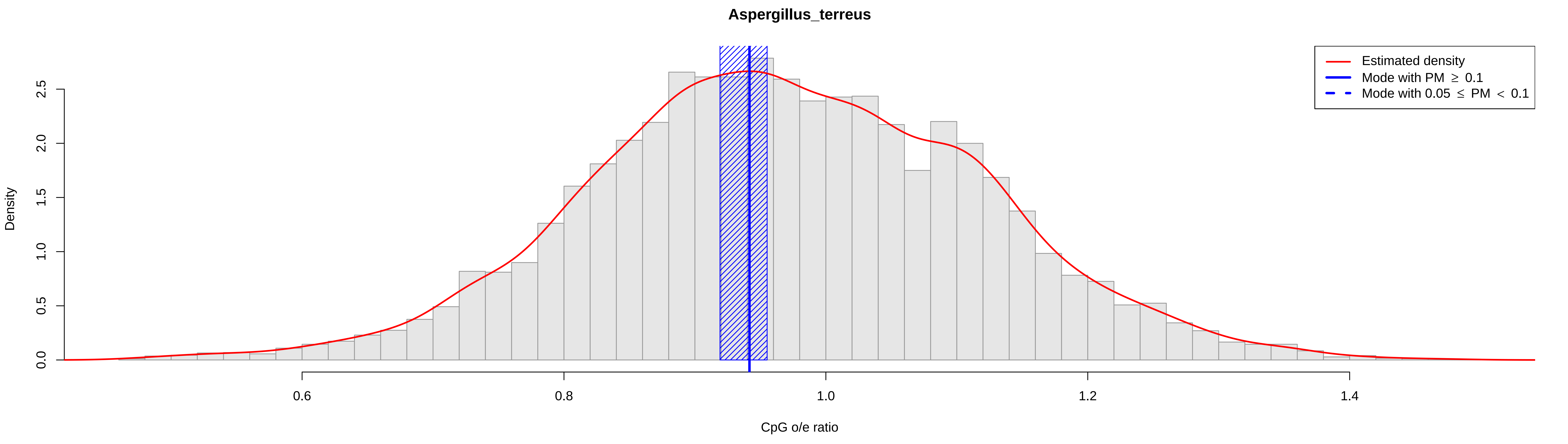
Aspergillus_terreus
Estimated density
Mode with PM ≥ 0.1
Mode with 0.05 ≤ PM < 0.1
Density
0.0
0.5
1.0
1.5
2.0
2.5
0.6
0.8
1.0
1.2
1.4
CpG o/e ratio

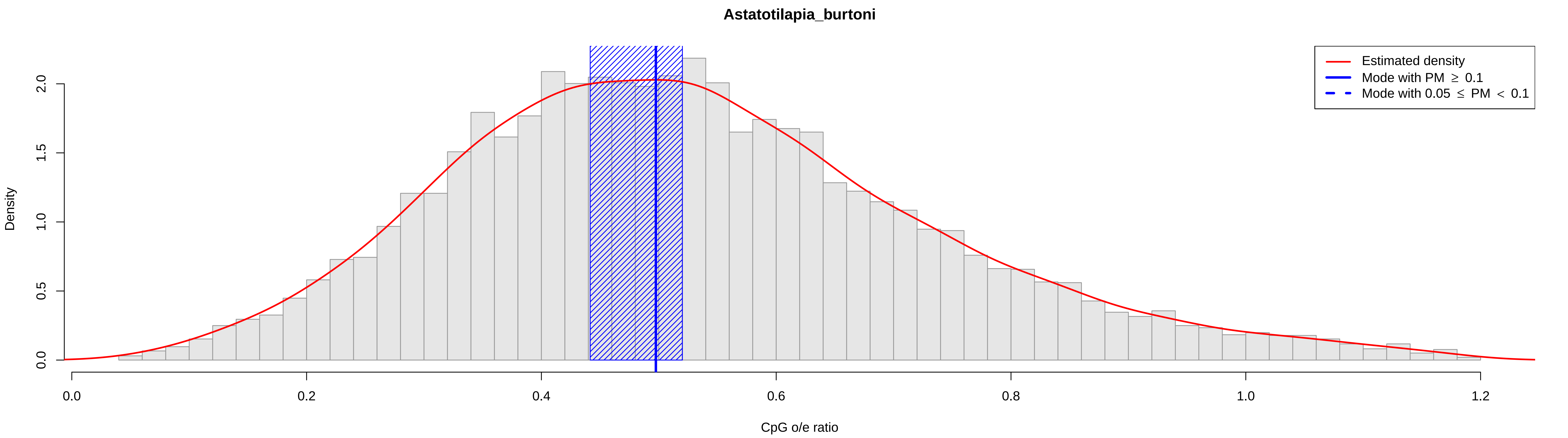

Astatotilapia_burtoni
Estimated density
Mode with PM ≥ 0.1
Mode with 0.05 ≤ PM < 0.1
Density
0.0
0.5
1.0
1.5
2.0
0.0
0.2
0.4
0.6
0.8
1.0
1.2
CpG o/e ratio

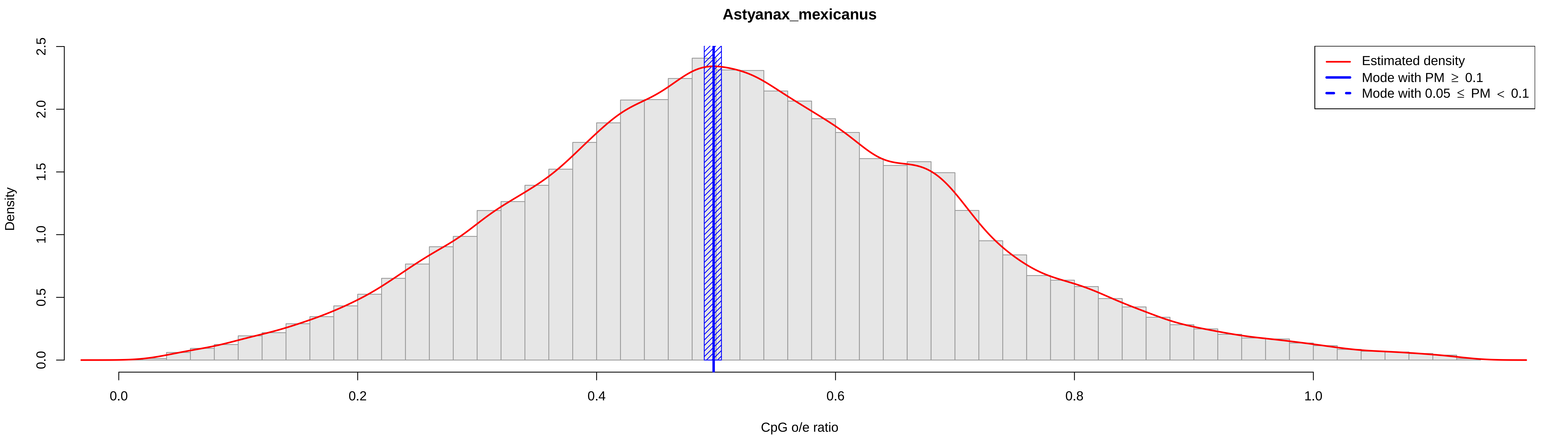

Astyanax_mexicanus
Estimated density
Mode with PM ≥ 0.1
Mode with 0.05 ≤ PM < 0.1
Density
0.0
0.5
1.0
1.5
2.0
2.5
0.0
0.2
0.4
0.6
0.8
1.0
CpG o/e ratio

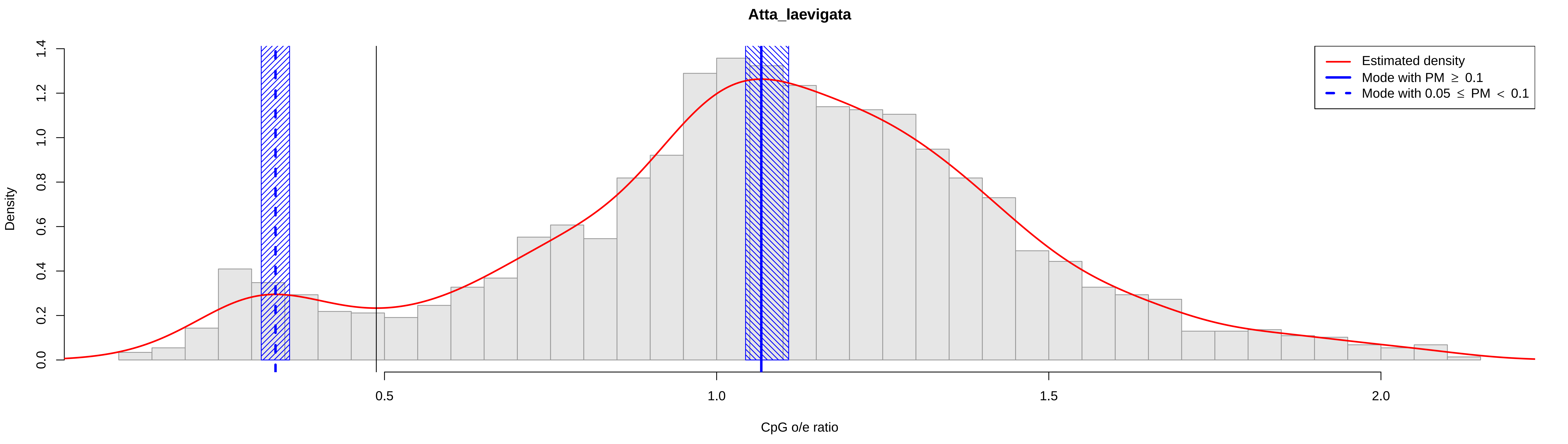
Atta_laevigata
Density
CpG o/e ratio
0.0
0.2
0.4
0.6
0.8
1.0
1.2
1.4
0.5
1.0
1.5
2.0
Estimated density
Mode with PM ≥ 0.1
Mode with 0.05 ≤ PM < 0.1

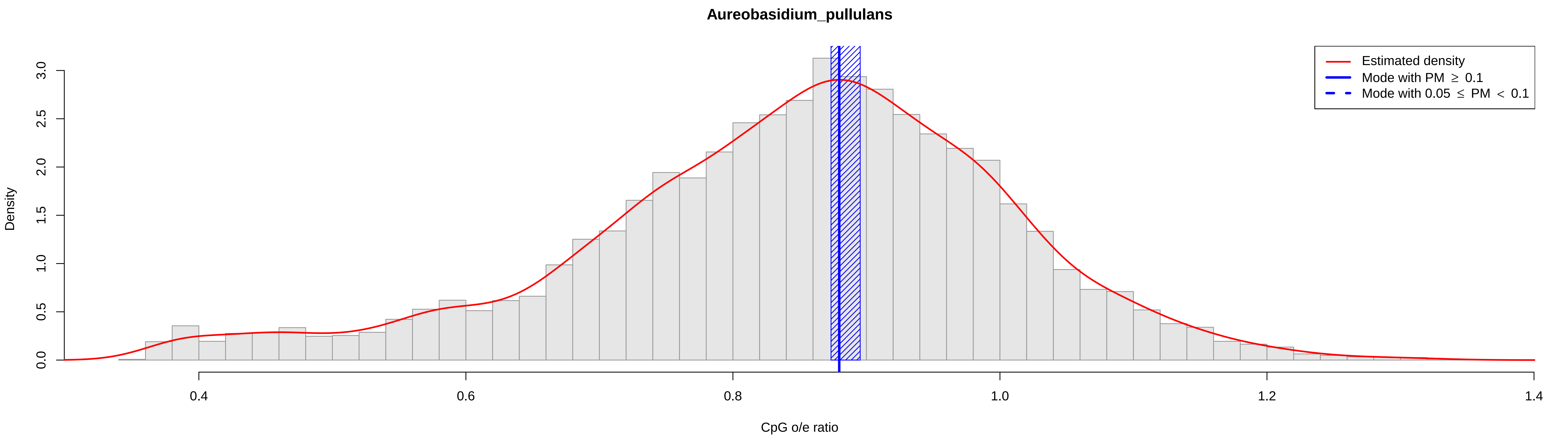

Aureobasidium_pullulans
Estimated density
Mode with PM ≥ 0.1
Mode with 0.05 ≤ PM < 0.1
Density
0.0
0.5
1.0
1.5
2.0
2.5
3.0
0.4
0.6
0.8
1.0
1.2
1.4
CpG o/e ratio

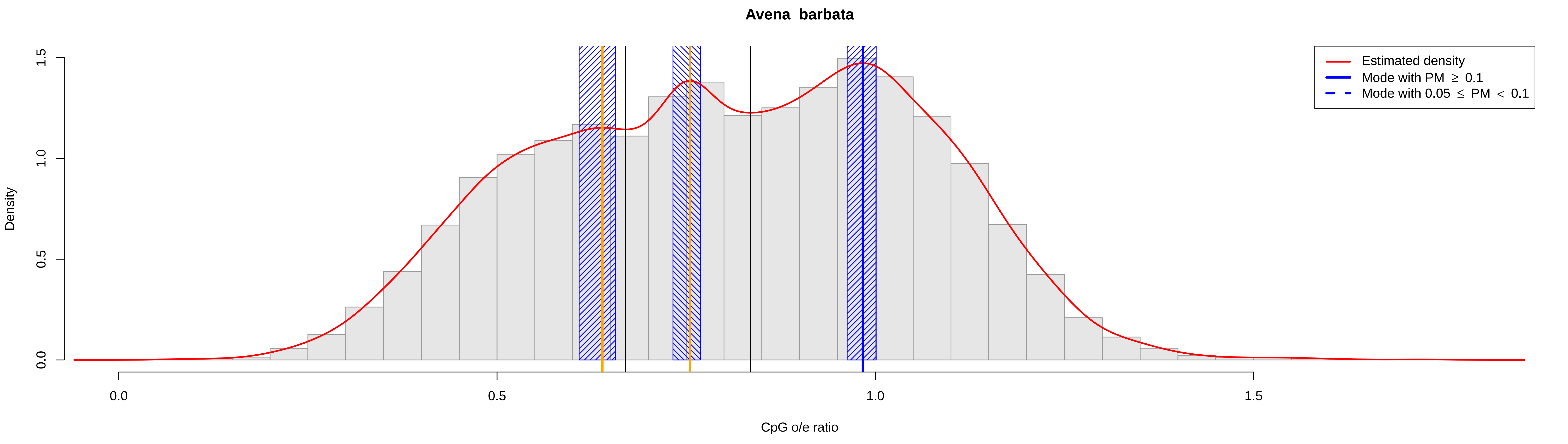
Avena_barbata
Estimated density
Mode with PM ≥ 0.1
Mode with 0.05 ≤ PM < 0.1
Density
0.0
0.5
1.0
1.5
CpG o/e ratio

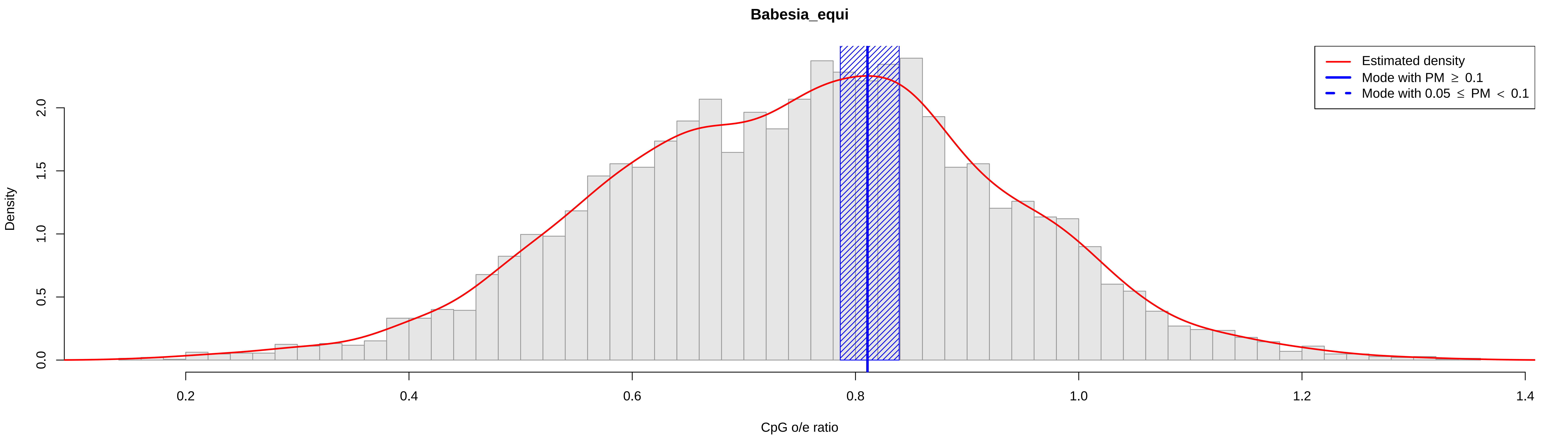

Babesia_equi
Estimated density
Mode with PM ≥ 0.1
Mode with 0.05 ≤ PM < 0.1
Density
0.0
0.5
1.0
1.5
2.0
0.2
0.4
0.6
0.8
1.0
1.2
1.4
CpG o/e ratio

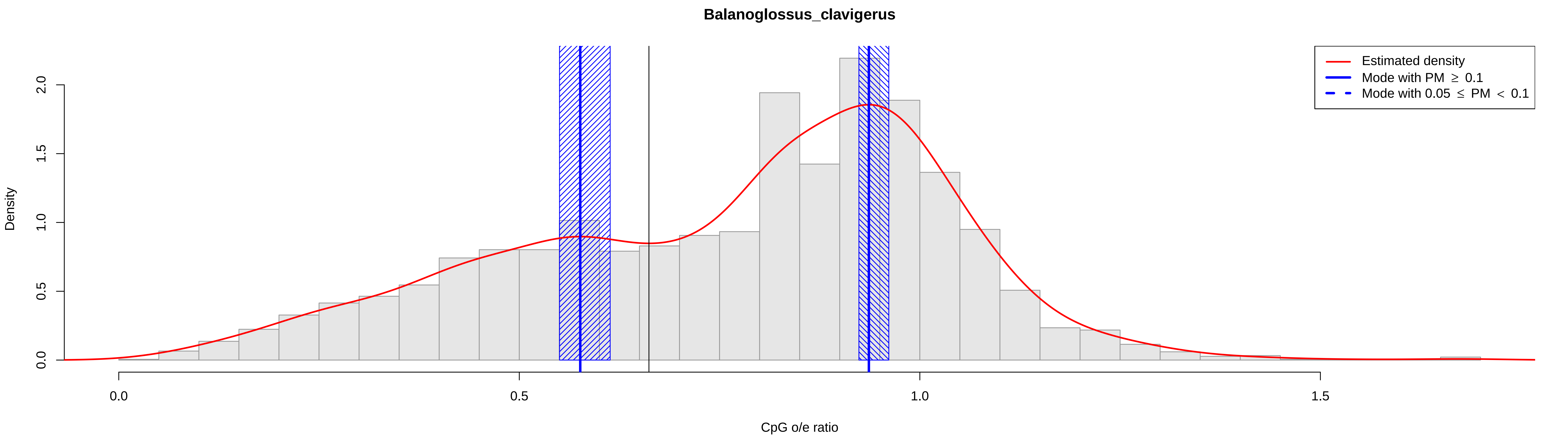
Balanoglossus_clavigerus
Estimated density
Mode with PM ≥ 0.1
Mode with 0.05 ≤ PM < 0.1
Density
0.0
0.5
1.0
1.5
2.0
0.0
0.5
1.0
1.5
CpG o/e ratio

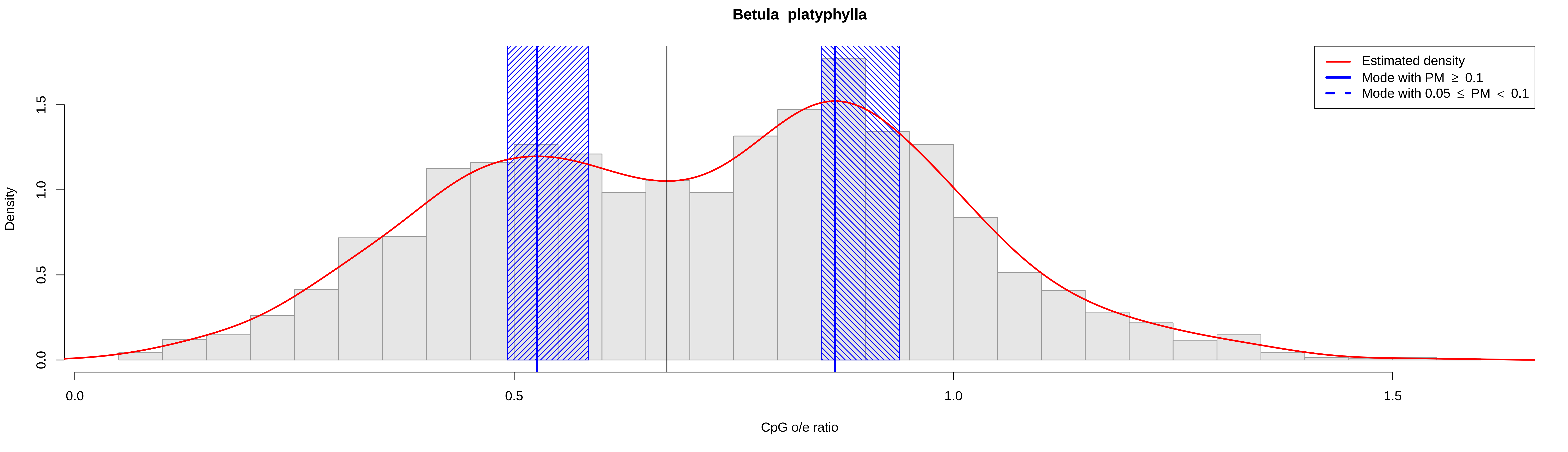
Betula_platyphylla
Estimated density
Mode with PM ≥ 0.1
Mode with 0.05 ≤ PM < 0.1
Density
0.0
0.5
1.0
1.5
CpG o/e ratio

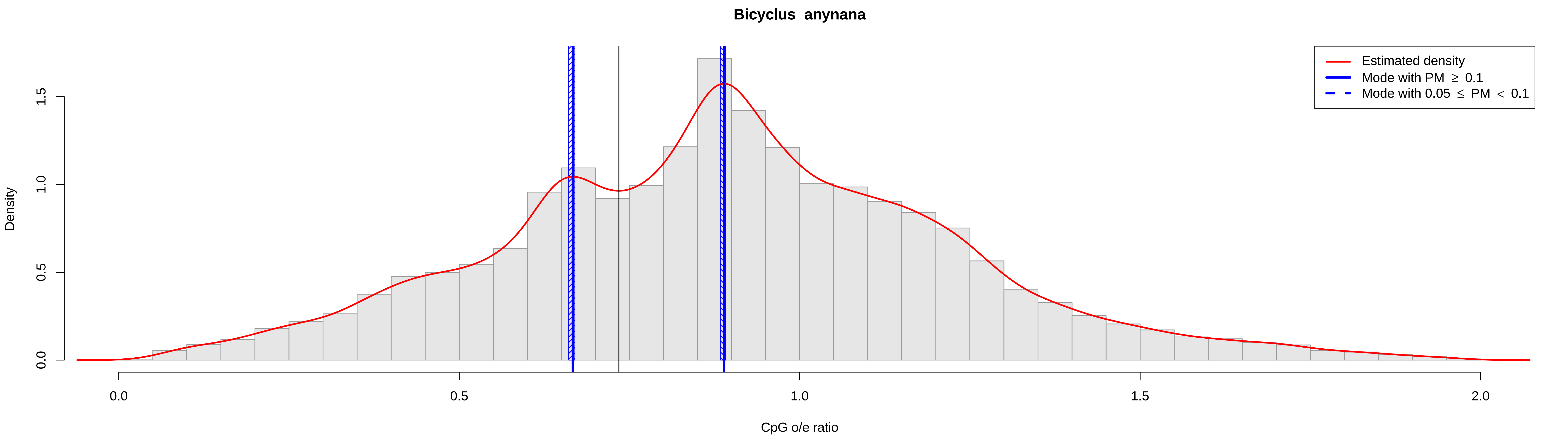
Bicyclus_anynana
Estimated density
Mode with PM ≥ 0.1
Mode with 0.05 ≤ PM < 0.1
Density
0.0
0.5
1.0
1.5
0.0
0.5
1.0
1.5
2.0
CpG o/e ratio

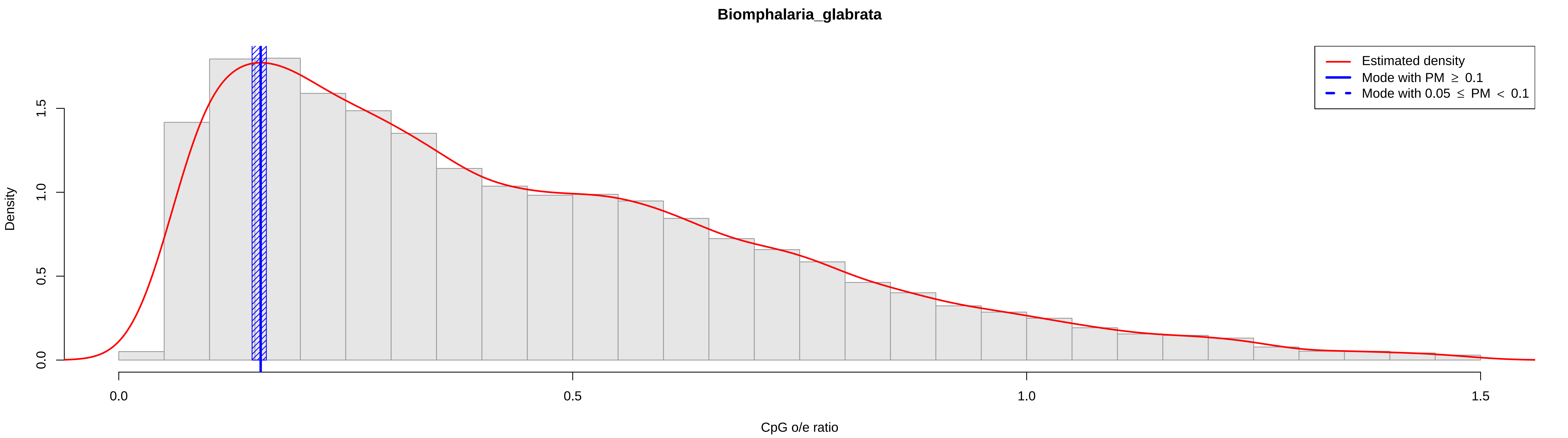

Biomphalaria_glabrata
Estimated density
Mode with PM ≥ 0.1
Mode with 0.05 ≤ PM < 0.1
Density
0.0
0.5
1.0
1.5
CpG o/e ratio

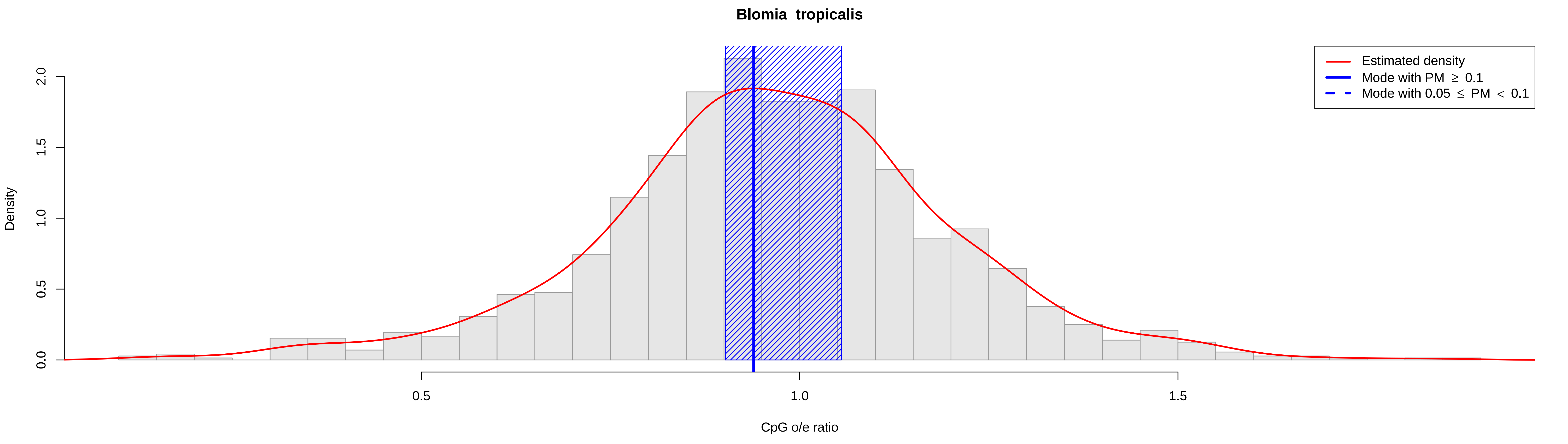
Blomia_tropicalis
Estimated density
Mode with PM ≥ 0.1
Mode with 0.05 ≤ PM < 0.1
Density
0.0
0.5
1.0
1.5
2.0
0.5
1.0
1.5
CpG o/e ratio

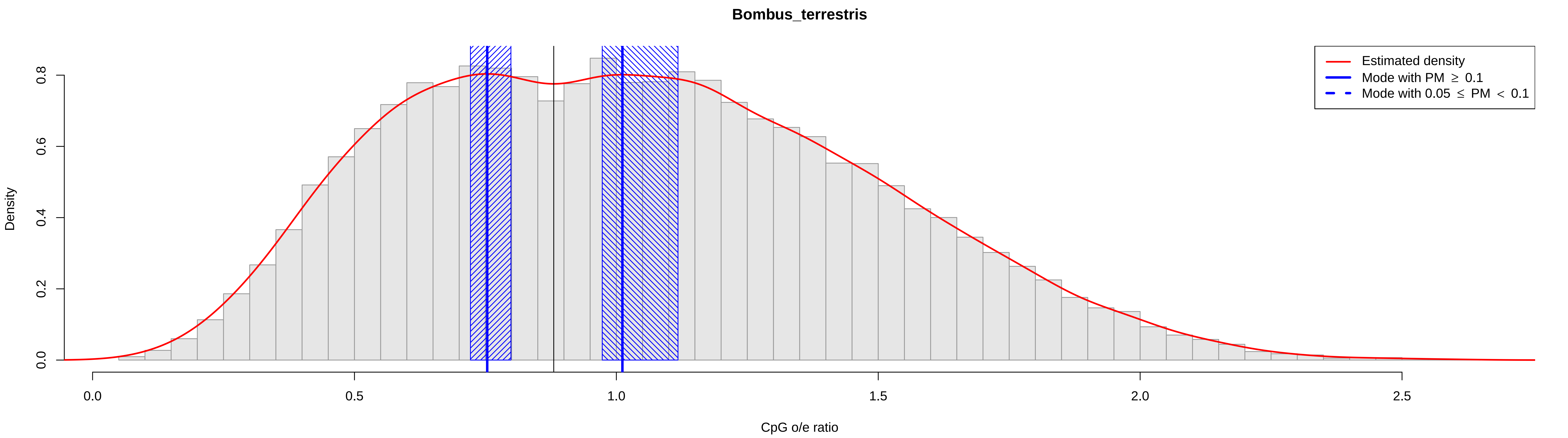

Bombus_terrestris
Estimated density
Mode with PM ≥ 0.1
Mode with 0.05 ≤ PM < 0.1
Density
0.0
0.2
0.4
0.6
0.8
0.0
0.5
1.0
1.5
2.0
2.5
CpG o/e ratio

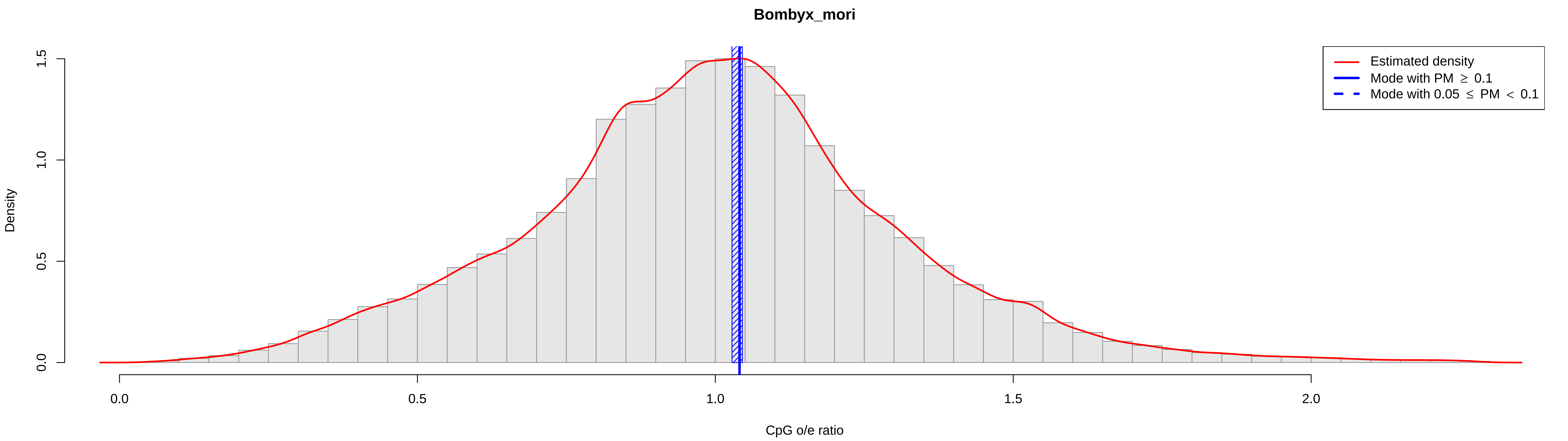

Bombyx_mori
Estimated density
Mode with PM ≥ 0.1
Mode with 0.05 ≤ PM < 0.1
Density
0.0
0.5
1.0
1.5
0.0
0.5
1.0
1.5
2.0
CpG o/e ratio

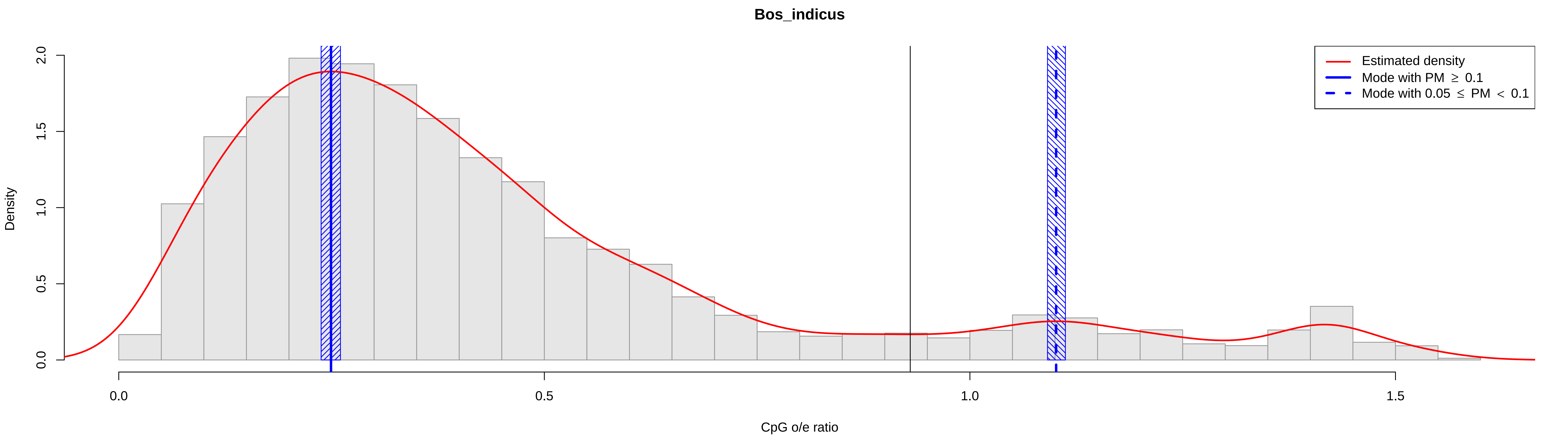
Bos_indicus
Estimated density
Mode with PM ≥ 0.1
Mode with 0.05 ≤ PM < 0.1
Density
0.0
0.5
1.0
1.5
2.0
0.0
0.5
1.0
1.5
CpG o/e ratio

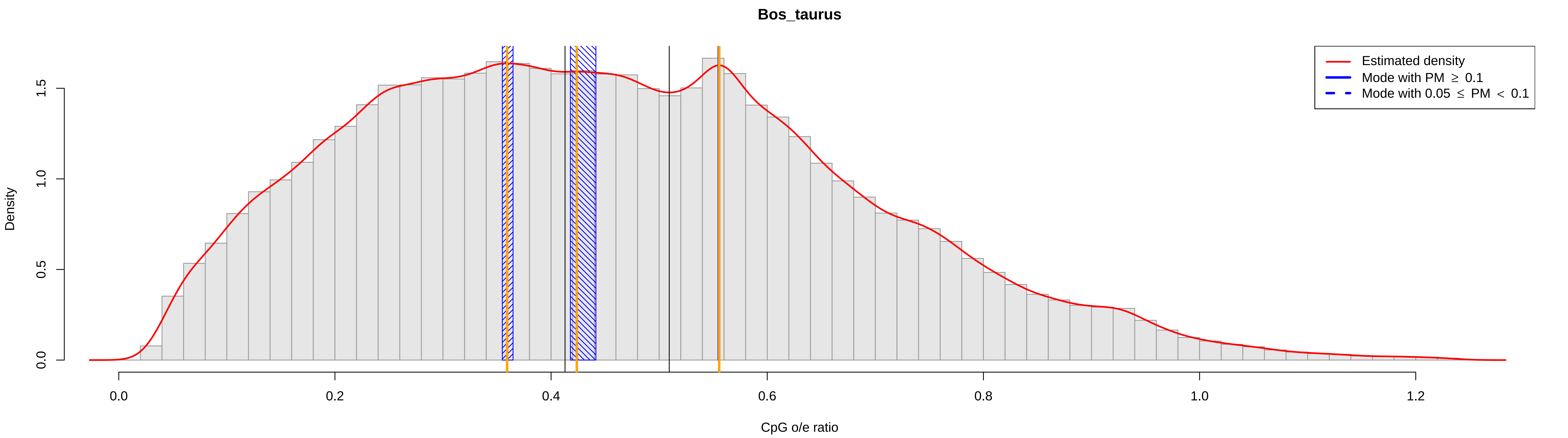

Bos_taurus
Estimated density
Mode with PM ≥ 0.1
Mode with 0.05 ≤ PM < 0.1
Density
0.0
0.5
1.0
1.5
0.0
0.2
0.4
0.6
0.8
1.0
1.2
CpG o/e ratio

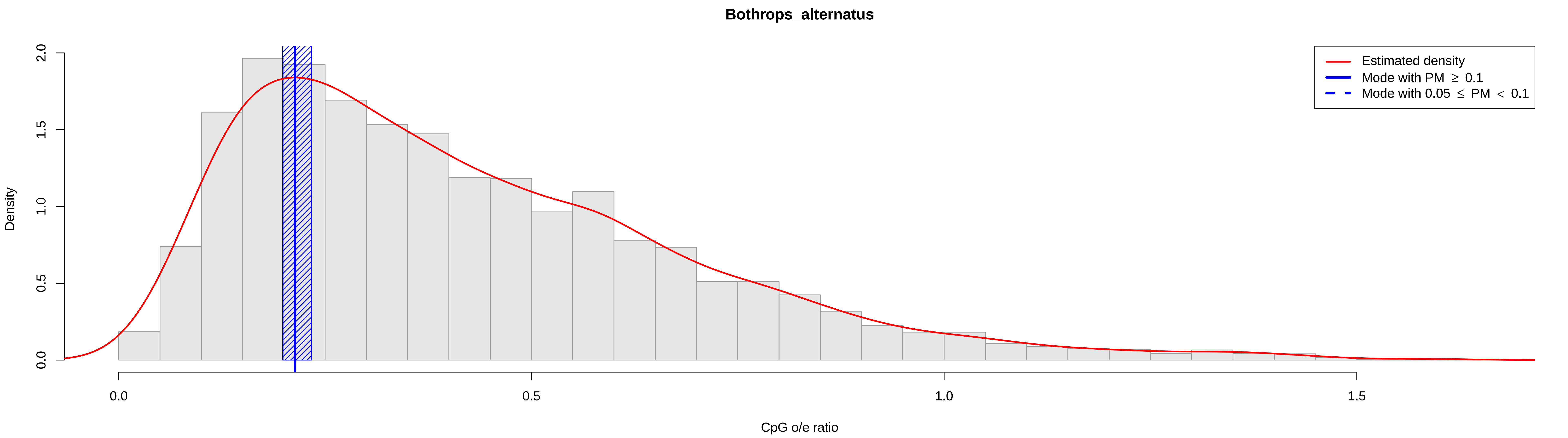

Bothrops_alternatus
Estimated density
Mode with PM ≥ 0.1
Mode with 0.05 ≤ PM < 0.1
Density
0.0
0.5
1.0
1.5
2.0
0.0
0.5
1.0
1.5
CpG o/e ratio

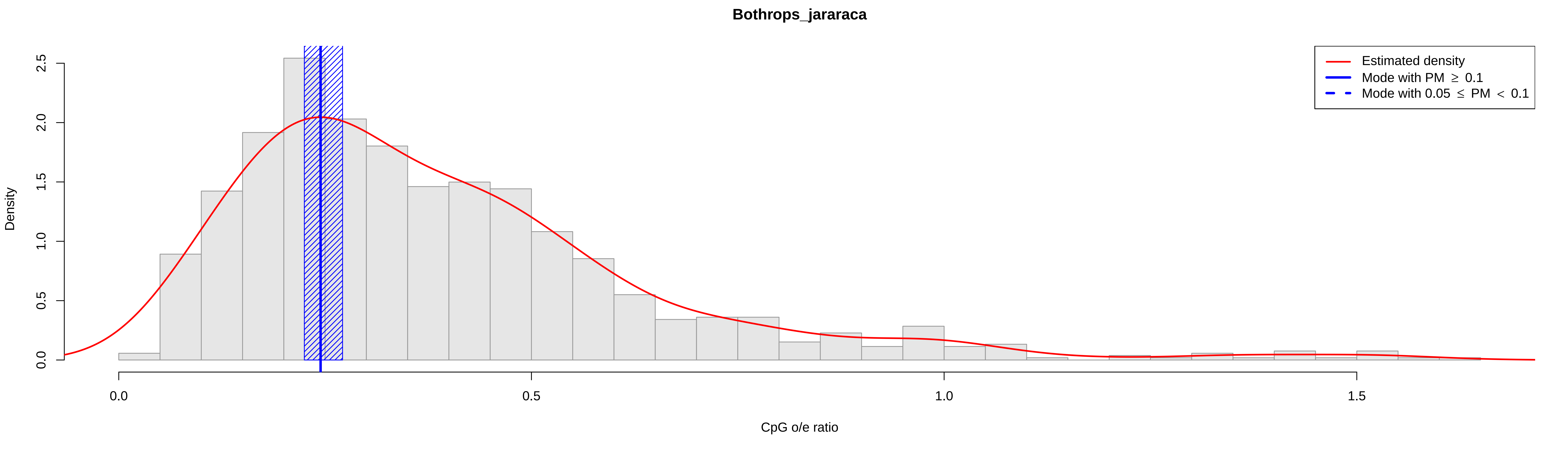
Bothrops_jararaca
Estimated density
Mode with PM ≥ 0.1
Mode with 0.05 ≤ PM < 0.1
Density
0.0
0.5
1.0
1.5
2.0
2.5
0.0
0.5
1.0
1.5
CpG o/e ratio

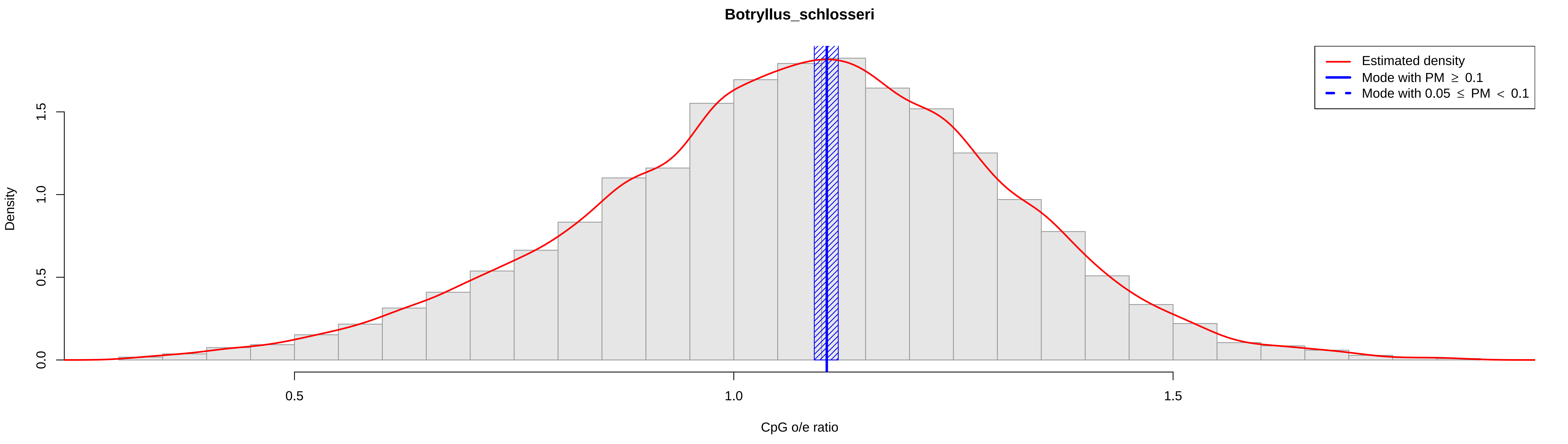

Botryllus_schlosseri
Estimated density
Mode with PM ≥ 0.1
Mode with 0.05 ≤ PM < 0.1
Density
0.0
0.5
1.0
1.5
0.5
1.0
1.5
CpG o/e ratio

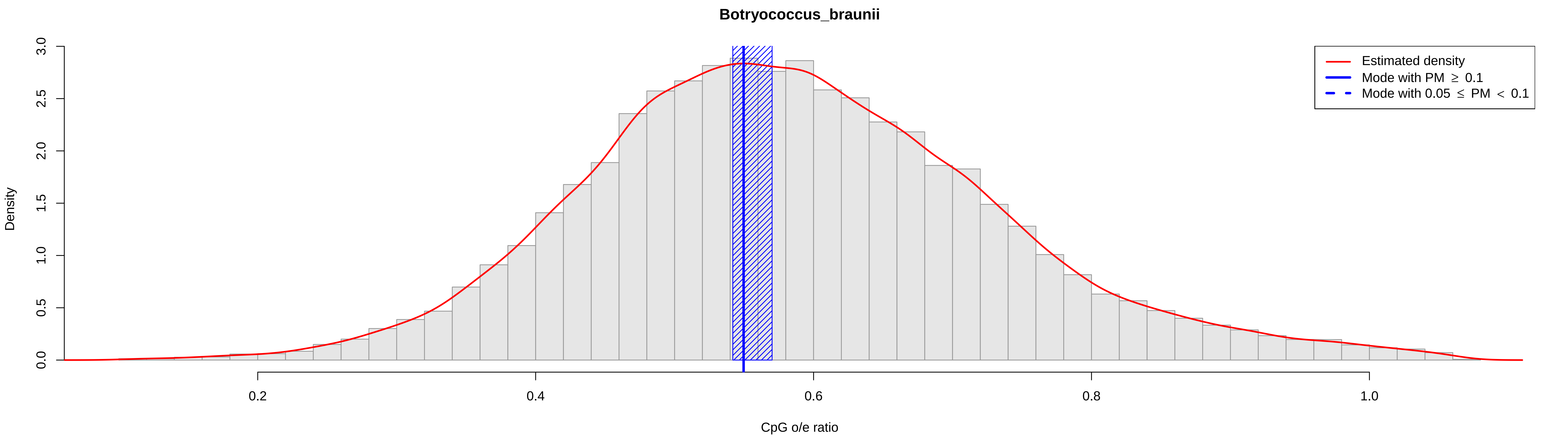

Botryococcus_braunii
Density
0.0
0.5
1.0
1.5
2.0
2.5
3.0
0.2
0.4
0.6
0.8
1.0
CpG o/e ratio
Estimated density
Mode with PM ≥ 0.1
Mode with 0.05 ≤ PM < 0.1

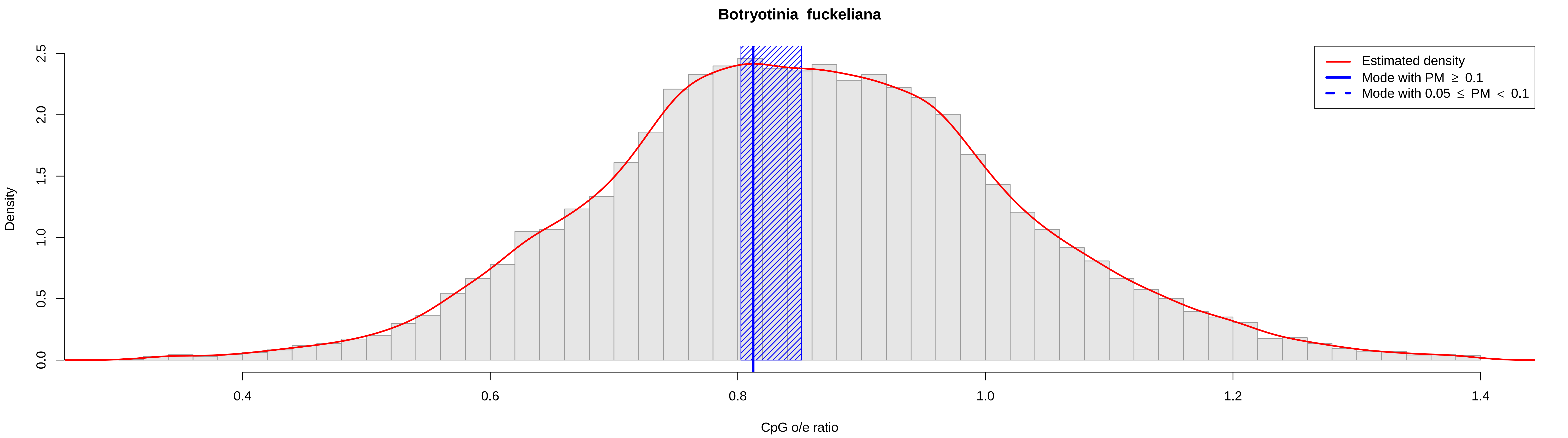

Botryotinia_fuckeliana
Density
CpG o/e ratio
0.0
0.5
1.0
1.5
2.0
2.5
0.4
0.6
0.8
1.0
1.2
1.4
Estimated density
Mode with PM ≥ 0.1
Mode with 0.05 ≤ PM < 0.1

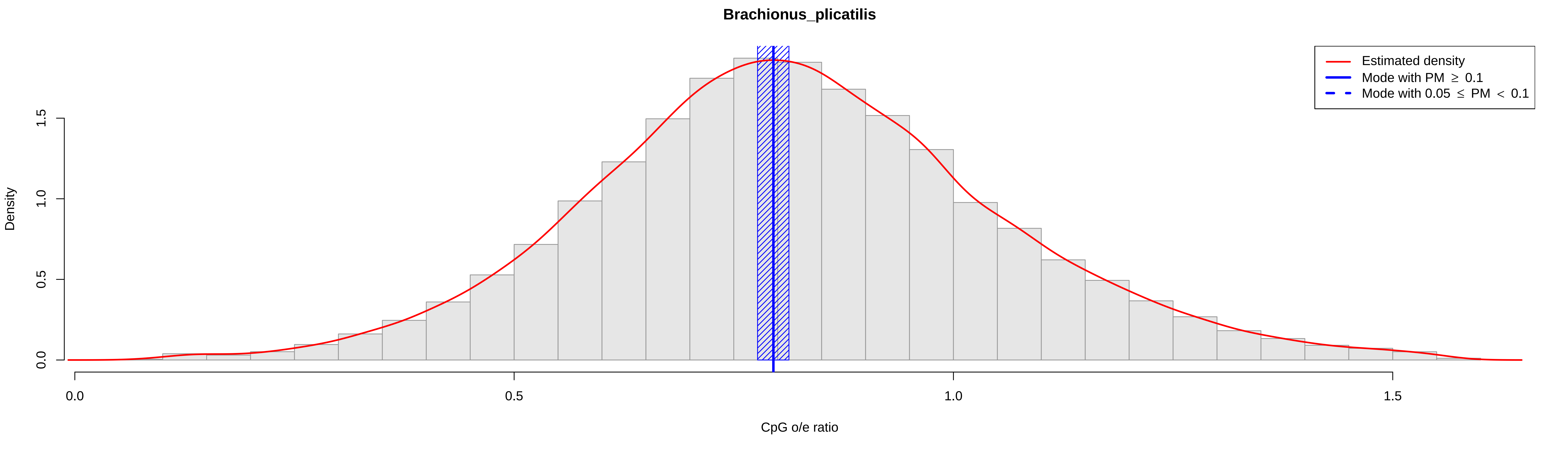
Brachionus_plicatilis
Estimated density
Mode with PM ≥ 0.1
Mode with 0.05 ≤ PM < 0.1
Density
0.0
0.5
1.0
1.5
0.0
0.5
1.0
1.5
CpG o/e ratio

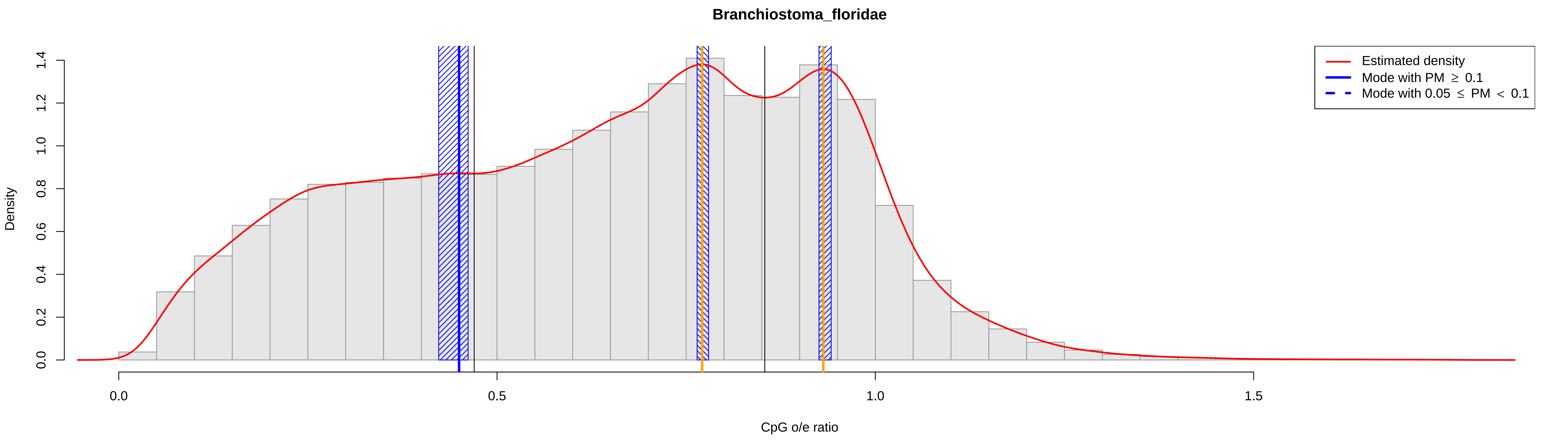

Branchiostoma_floridae
Estimated density
Mode with PM ≥ 0.1
Mode with 0.05 ≤ PM < 0.1
Density
0.0
0.2
0.4
0.6
0.8
1.0
1.2
1.4
0.0
0.5
1.0
1.5
CpG o/e ratio

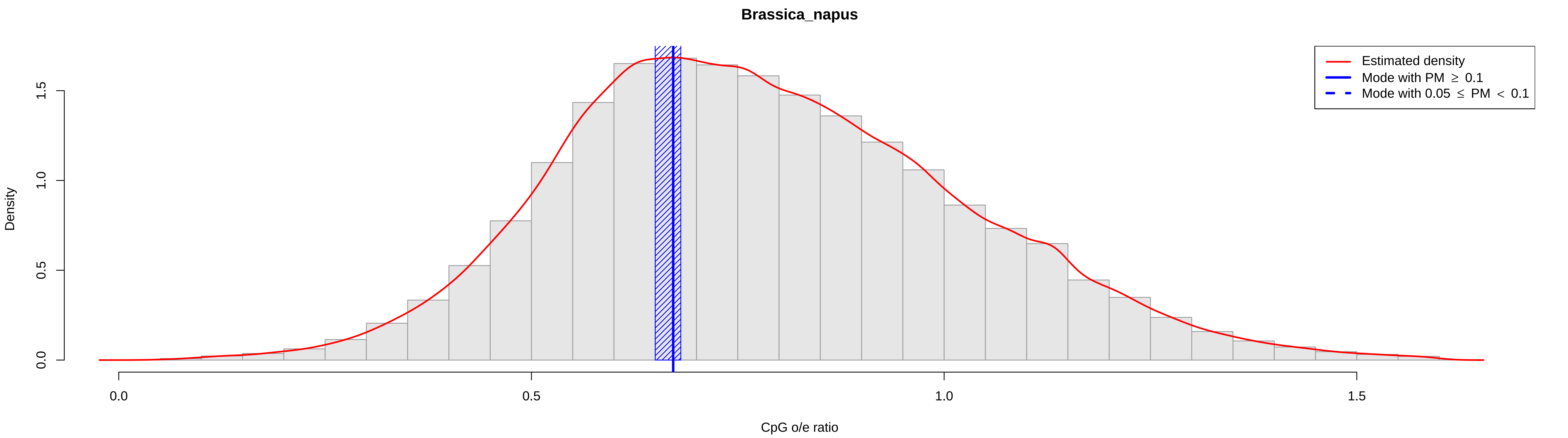
Brassica_napus
Estimated density
Mode with PM ≥ 0.1
Mode with 0.05 ≤ PM < 0.1
Density
0.0
0.5
1.0
1.5
CpG o/e ratio

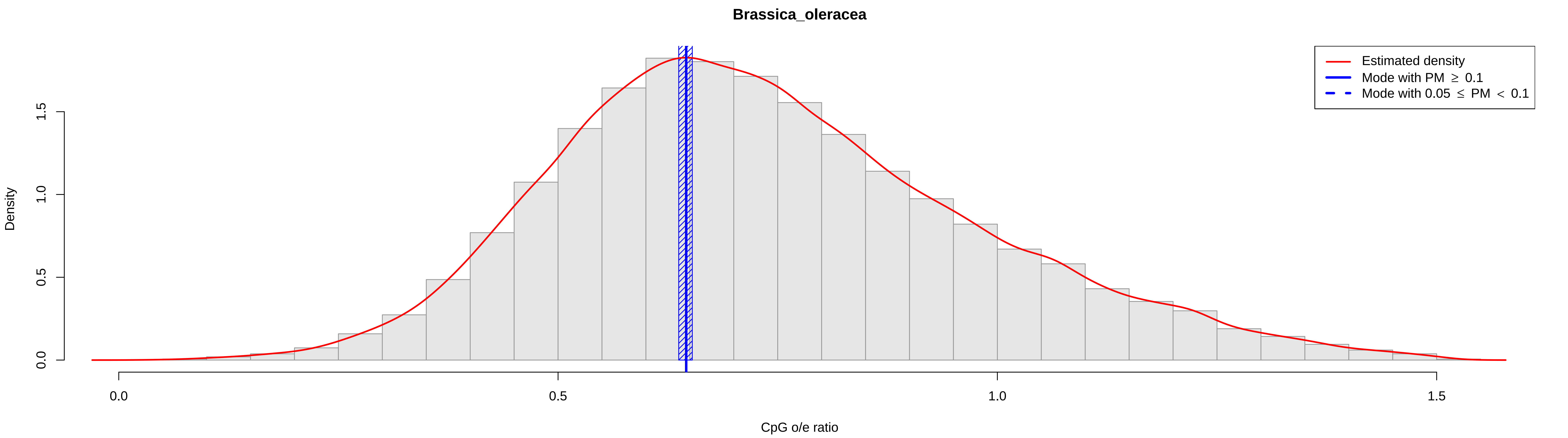

Brassica_oleracea
Estimated density
Mode with PM ≥ 0.1
Mode with 0.05 ≤ PM < 0.1
Density
0.0
0.5
1.0
1.5
CpG o/e ratio

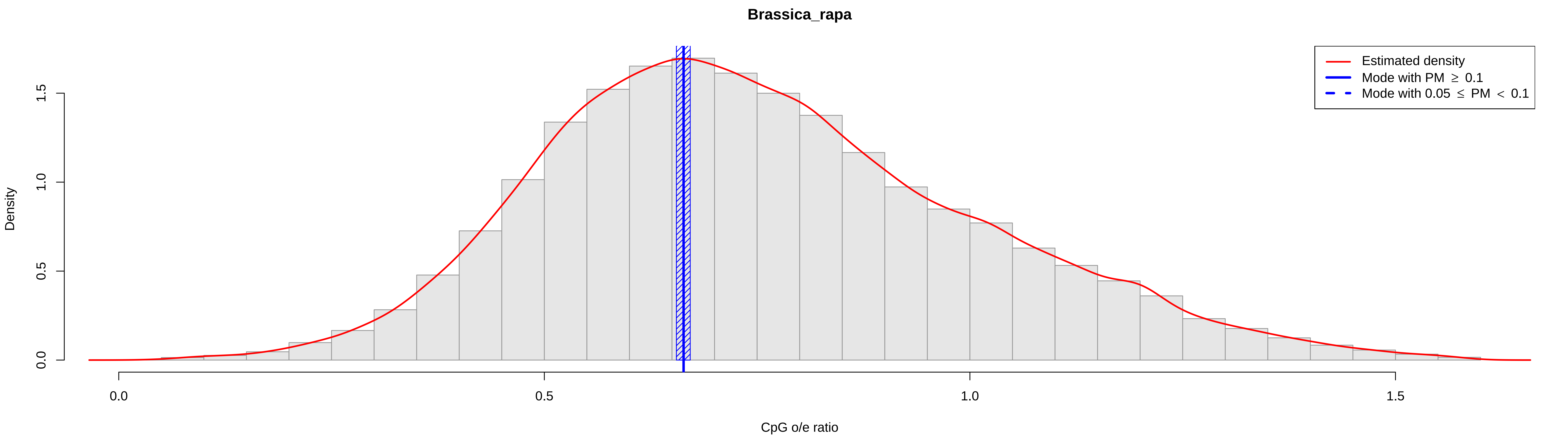

Brassica_rapa
Estimated density
Mode with PM ≥ 0.1
Mode with 0.05 ≤ PM < 0.1
Density
0.0
0.5
1.0
1.5
CpG o/e ratio

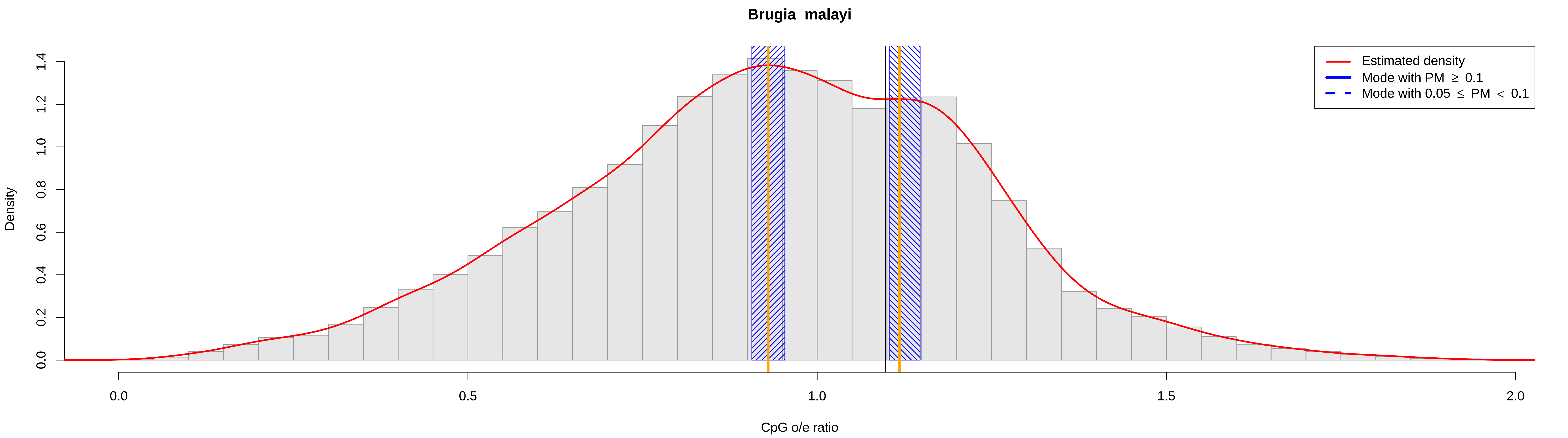

Brugia_malayi
Estimated density
Mode with PM ≥ 0.1
Mode with 0.05 ≤ PM < 0.1
Density
0.0
0.2
0.4
0.6
0.8
1.0
1.2
1.4
0.0
0.5
1.0
1.5
2.0
CpG o/e ratio

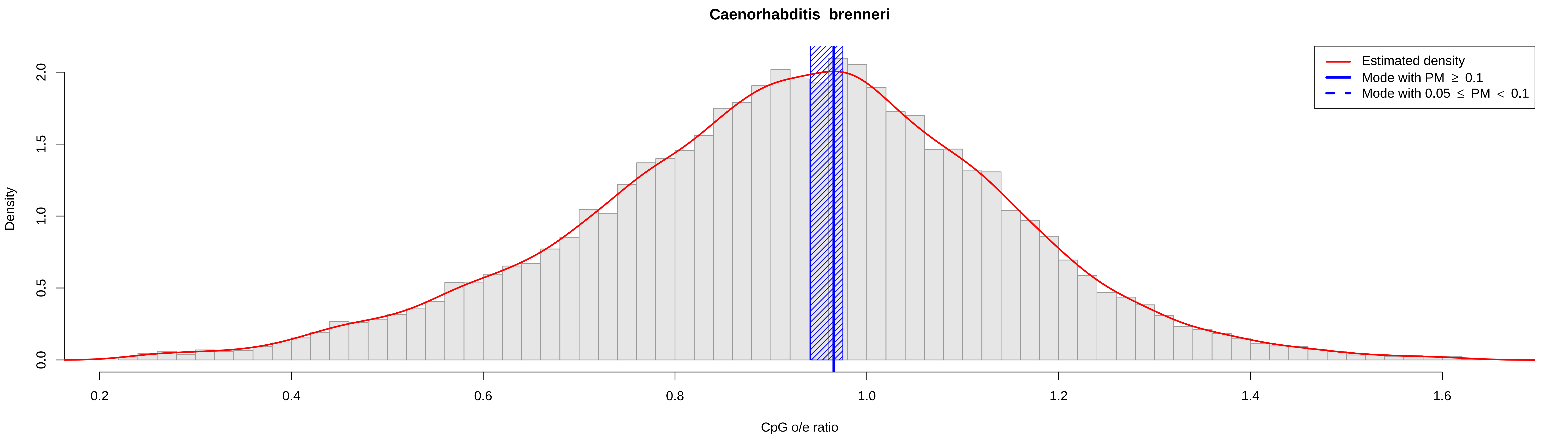

Caenorhabditis_brenneri
Estimated density
Mode with PM ≥ 0.1
Mode with 0.05 ≤ PM < 0.1
Density
0.0
0.5
1.0
1.5
2.0
0.2
0.4
0.6
0.8
1.0
1.2
1.4
1.6
CpG o/e ratio

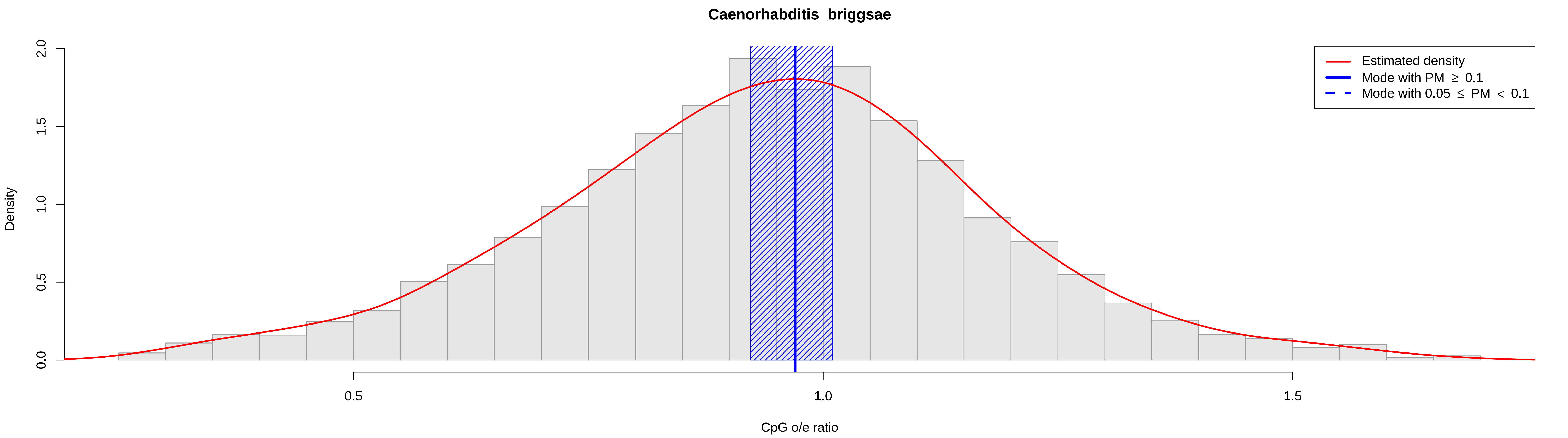

Caenorhabditis_briggsae
Estimated density
Mode with PM ≥ 0.1
Mode with 0.05 ≤ PM < 0.1
Density
0.0
0.5
1.0
1.5
2.0
0.5
1.0
1.5
CpG o/e ratio

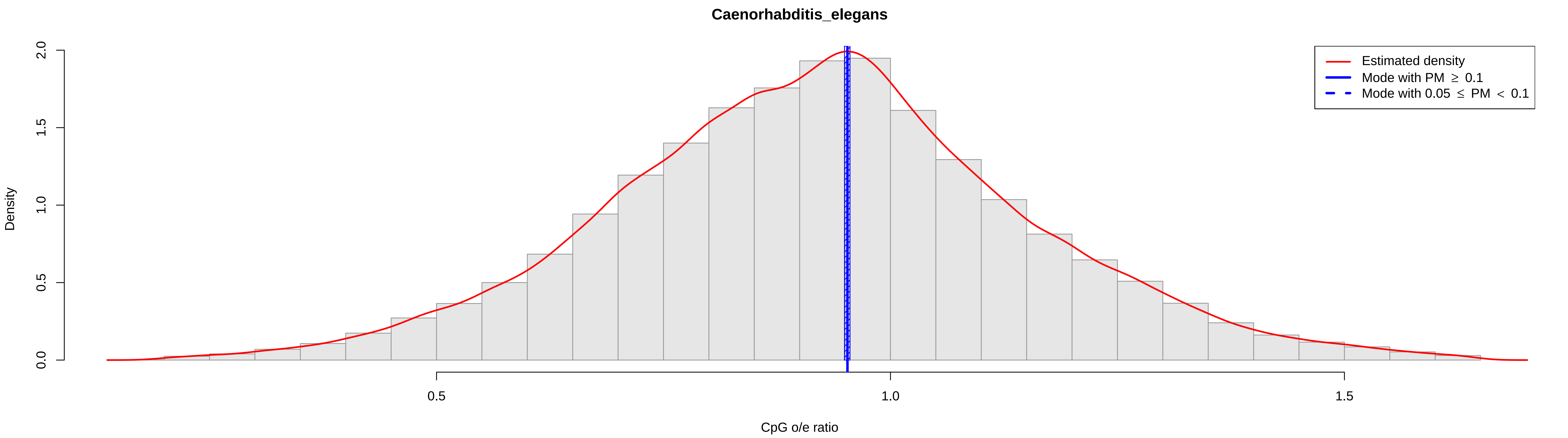

Caenorhabditis_elegans
Estimated density
Mode with PM ≥ 0.1
Mode with 0.05 ≤ PM < 0.1
Density
0.0
0.5
1.0
1.5
2.0
0.5
1.0
1.5
CpG o/e ratio

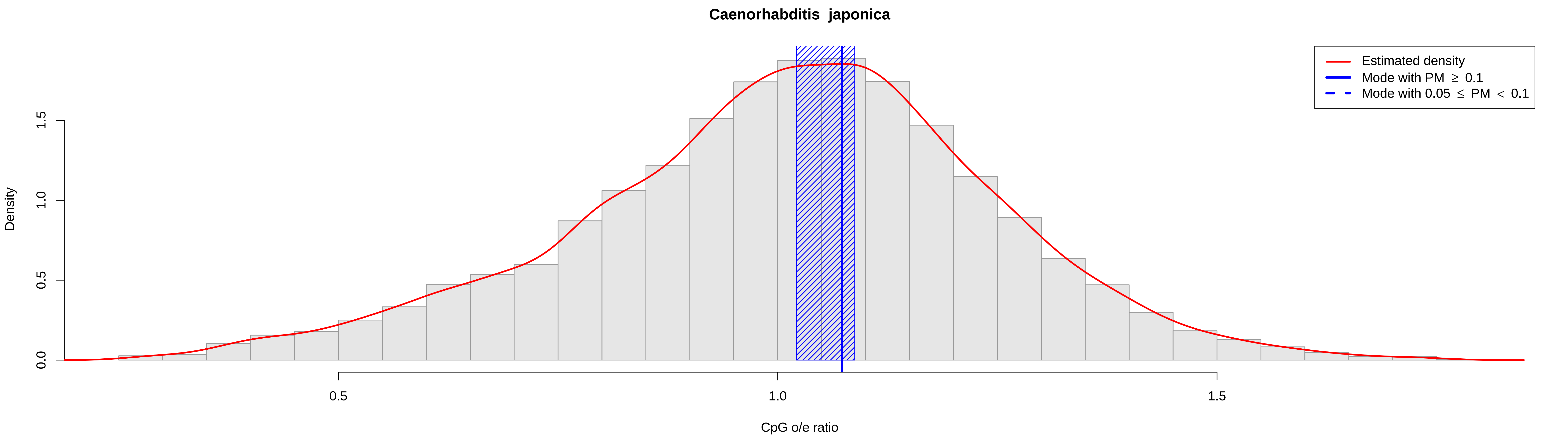

Caenorhabditis_japonica
Estimated density
Mode with PM ≥ 0.1
Mode with 0.05 ≤ PM < 0.1
Density
0.0
0.5
1.0
1.5
0.5
1.0
1.5
CpG o/e ratio

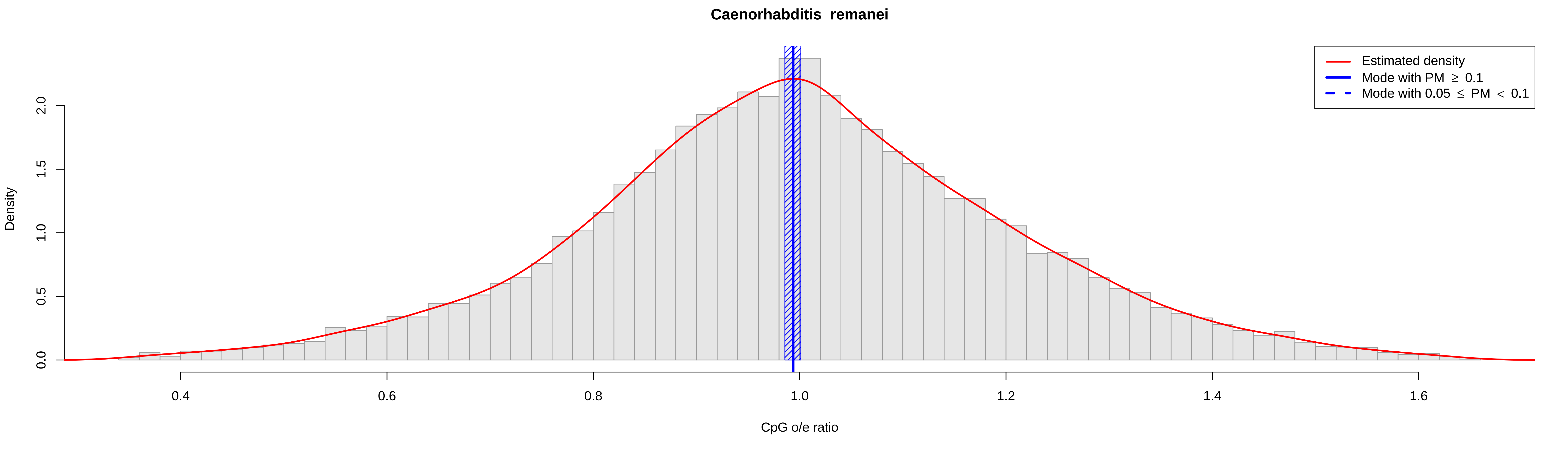

Caenorhabditis_remanei
Estimated density
Mode with PM ≥ 0.1
Mode with 0.05 ≤ PM < 0.1
Density
0.0
0.5
1.0
1.5
2.0
0.4
0.6
0.8
1.0
1.2
1.4
1.6
CpG o/e ratio

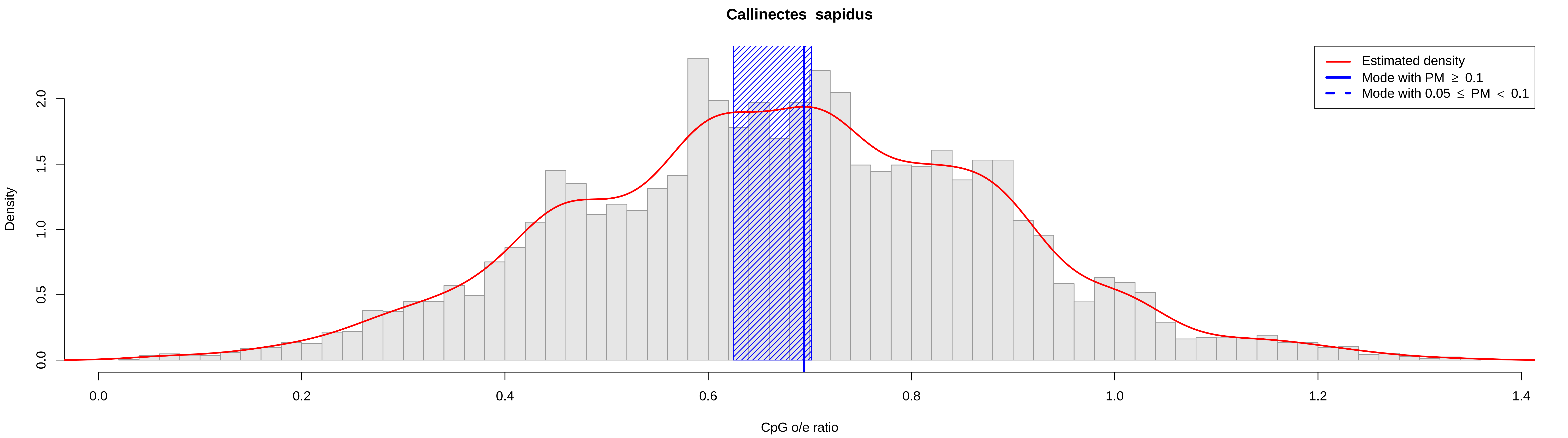

Callinectes_sapidus
Estimated density
Mode with PM ≥ 0.1
Mode with 0.05 ≤ PM < 0.1
Density
0.0
0.5
1.0
1.5
2.0
0.0
0.2
0.4
0.6
0.8
1.0
1.2
1.4
CpG o/e ratio

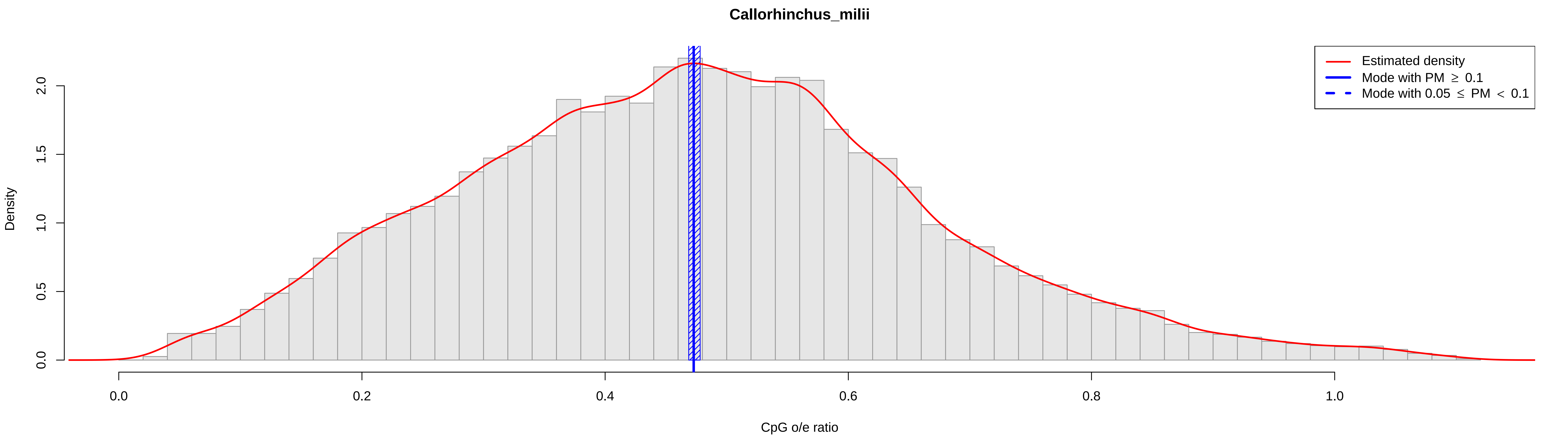

Callorhinchus_milii
Estimated density
Mode with PM ≥ 0.1
Mode with 0.05 ≤ PM < 0.1
Density
0.0
0.5
1.0
1.5
2.0
0.0
0.2
0.4
0.6
0.8
1.0
CpG o/e ratio

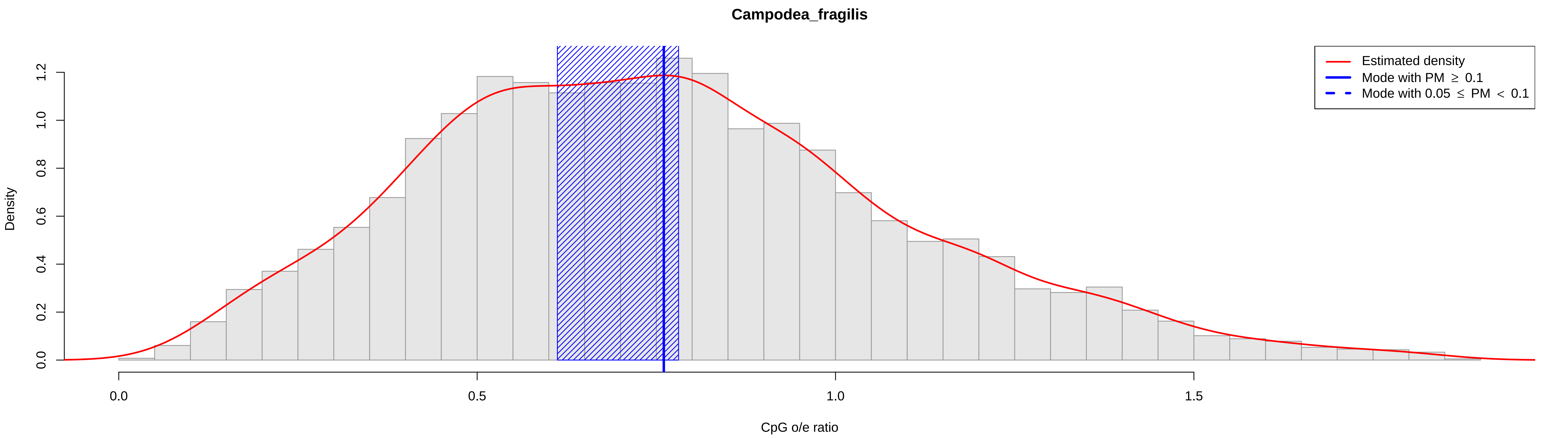

Campodea_fragilis
Estimated density
Mode with PM ≥ 0.1
Mode with 0.05 ≤ PM < 0.1
Density
0.0
0.2
0.4
0.6
0.8
1.0
1.2
0.0
0.5
1.0
1.5
CpG o/e ratio

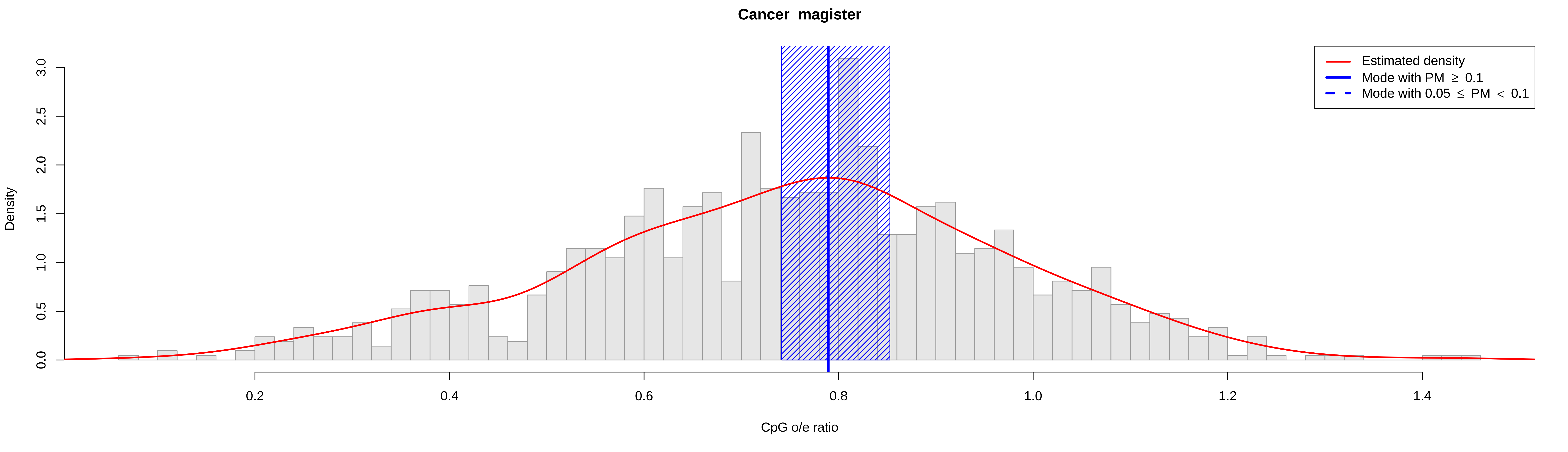

Cancer_magister
Estimated density
Mode with PM ≥ 0.1
Mode with 0.05 ≤ PM < 0.1
Density
0.0
0.5
1.0
1.5
2.0
2.5
3.0
0.2
0.4
0.6
0.8
1.0
1.2
1.4
CpG o/e ratio

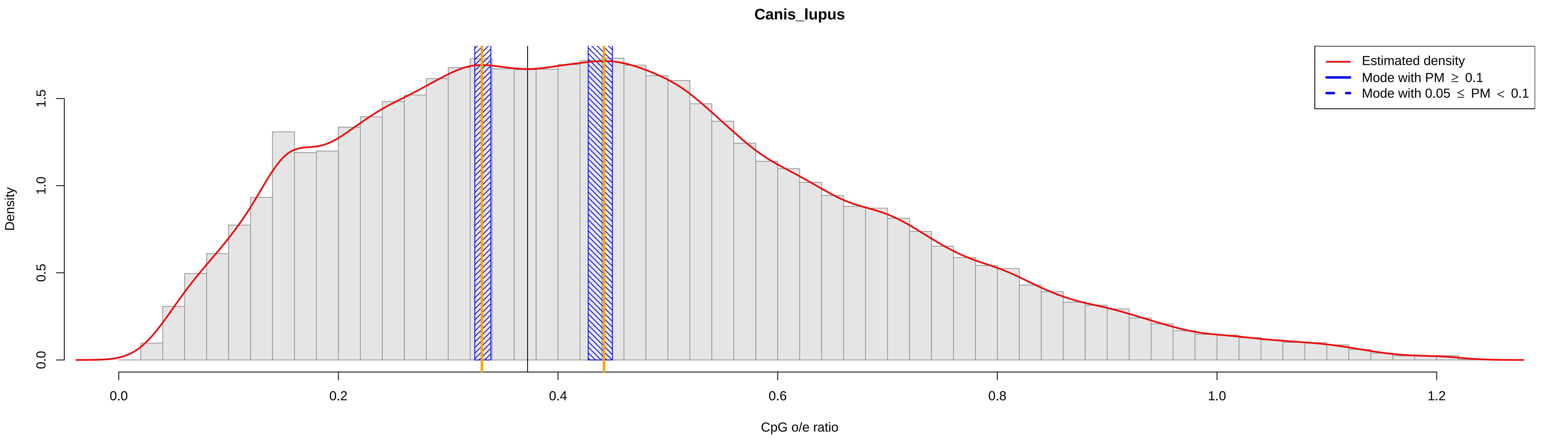
Canis_lupus
Estimated density
Mode with PM ≥ 0.1
Mode with 0.05 ≤ PM < 0.1
Density
0.0
0.5
1.0
1.5
0.0
0.2
0.4
0.6
0.8
1.0
1.2
CpG o/e ratio

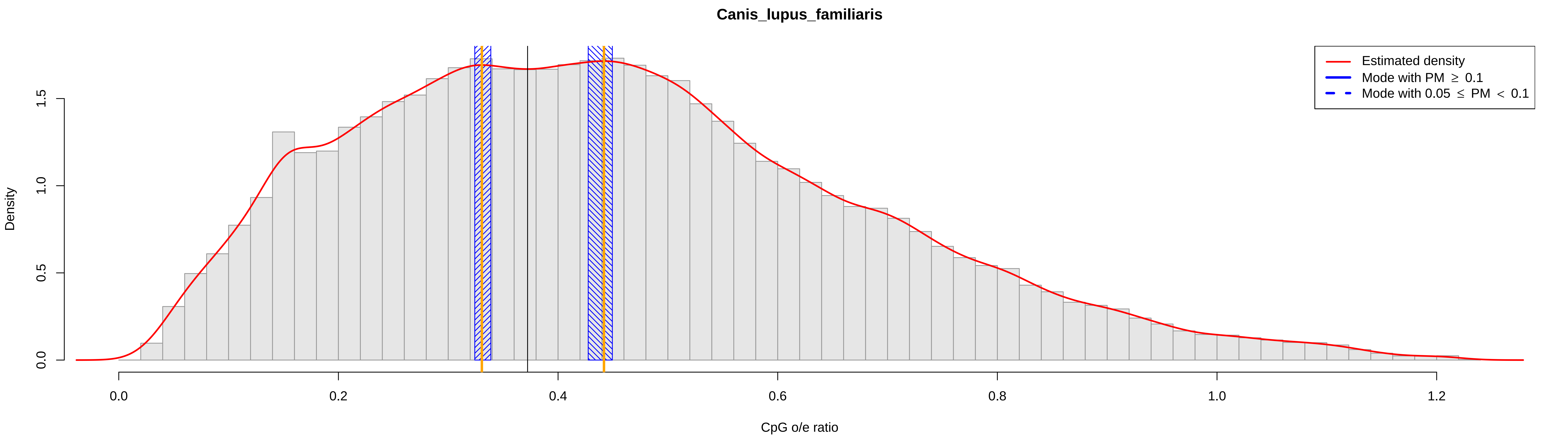

Canis_lupus_familiaris
Estimated density
Mode with PM ≥ 0.1
Mode with 0.05 ≤ PM < 0.1
Density
0.0
0.5
1.0
1.5
0.0
0.2
0.4
0.6
0.8
1.0
1.2
CpG o/e ratio

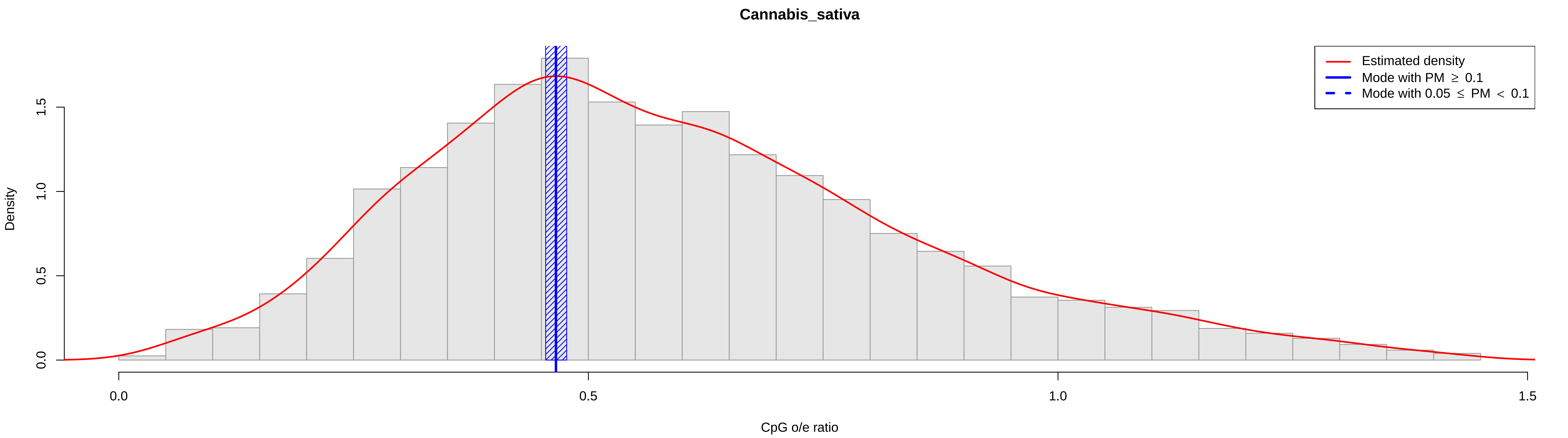

Cannabis_sativa
Estimated density
Mode with PM ≥ 0.1
Mode with 0.05 ≤ PM < 0.1
Density
0.0
0.5
1.0
1.5
0.0
0.5
1.0
1.5
CpG o/e ratio

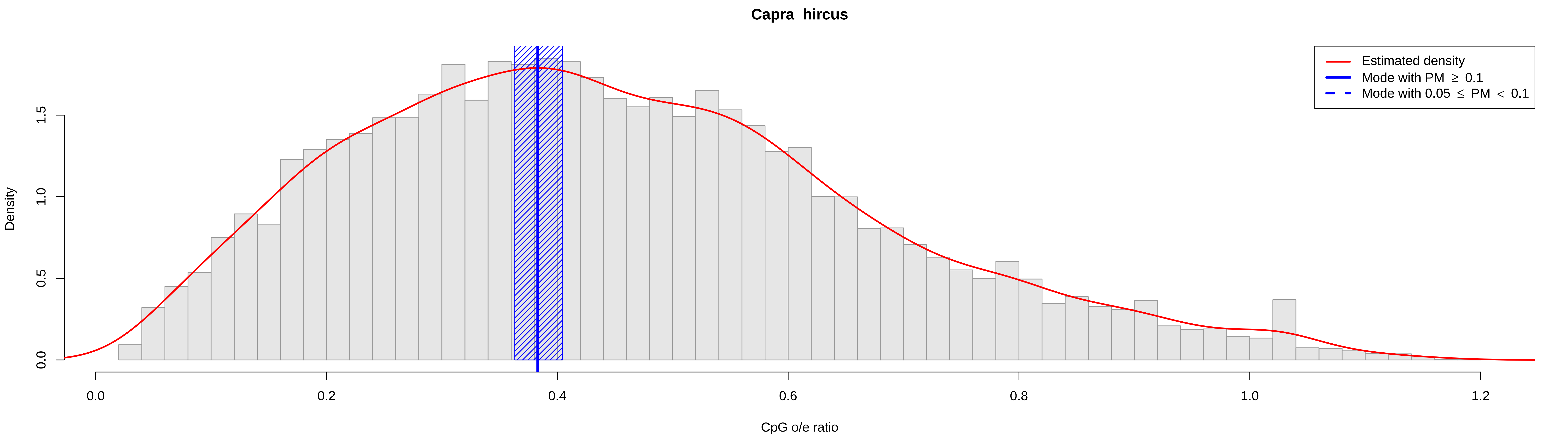

Capra_hircus
Estimated density
Mode with PM ≥ 0.1
Mode with 0.05 ≤ PM < 0.1
Density
0.0
0.5
1.0
1.5
0.0
0.2
0.4
0.6
0.8
1.0
1.2
CpG o/e ratio

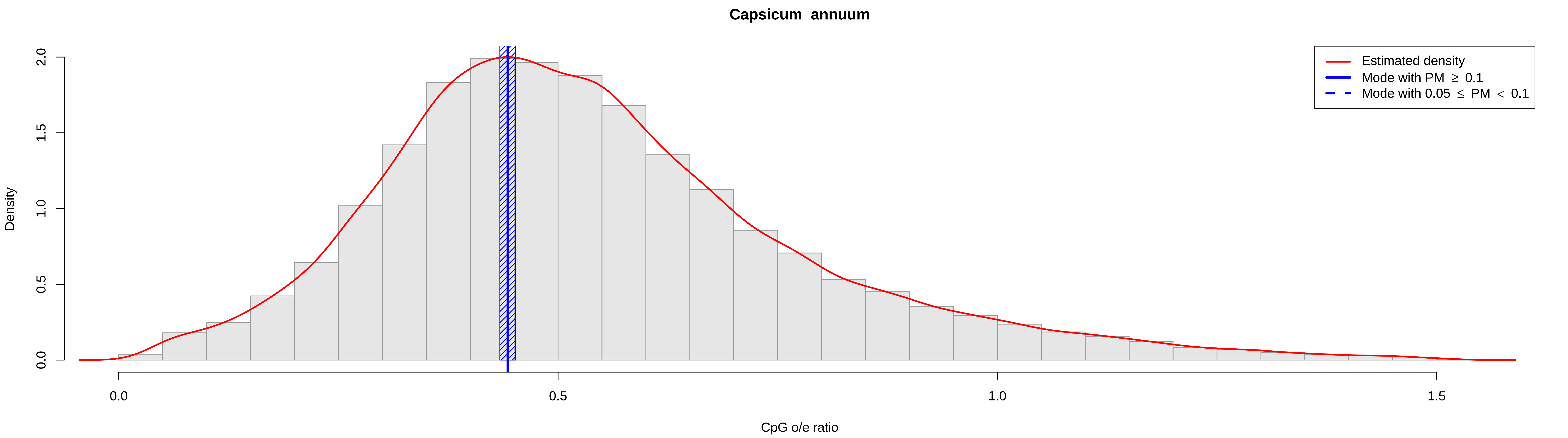
Capsicum_annuum
Estimated density
Mode with PM ≥ 0.1
Mode with 0.05 ≤ PM < 0.1
Density
0.0
0.5
1.0
1.5
2.0
0.0
0.5
1.0
1.5
CpG o/e ratio

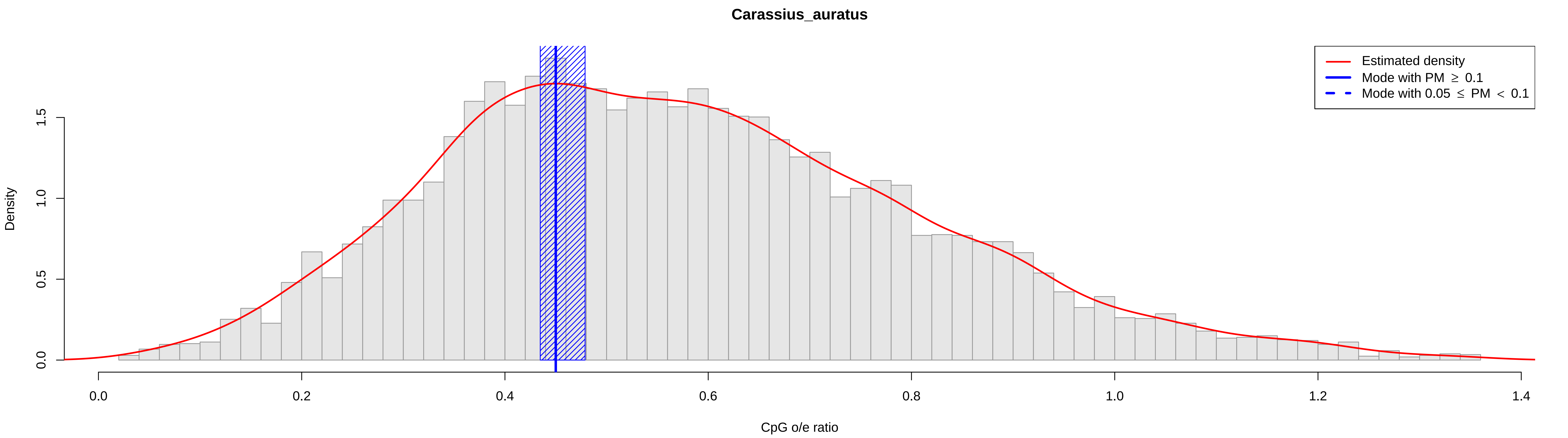

Carassius_auratus
Estimated density
Mode with PM ≥ 0.1
Mode with 0.05 ≤ PM < 0.1
Density
0.0
0.5
1.0
1.5
0.0
0.2
0.4
0.6
0.8
1.0
1.2
1.4
CpG o/e ratio

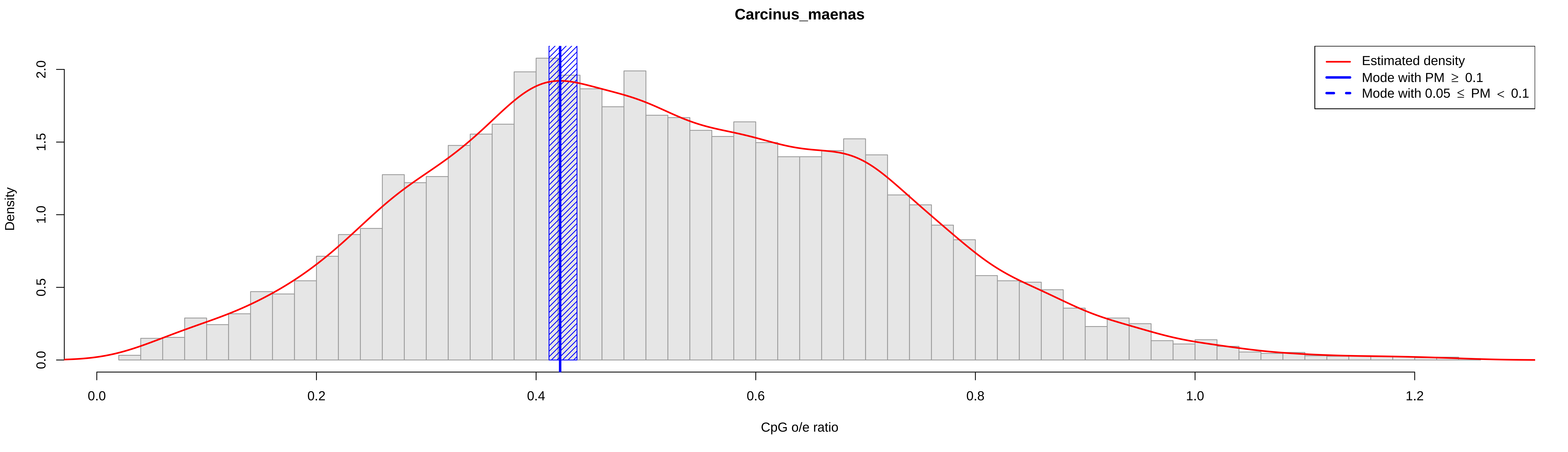

Carcinus_maenas
Estimated density
Mode with PM ≥ 0.1
Mode with 0.05 ≤ PM < 0.1
Density
0.0
0.5
1.0
1.5
2.0
0.0
0.2
0.4
0.6
0.8
1.0
1.2
CpG o/e ratio

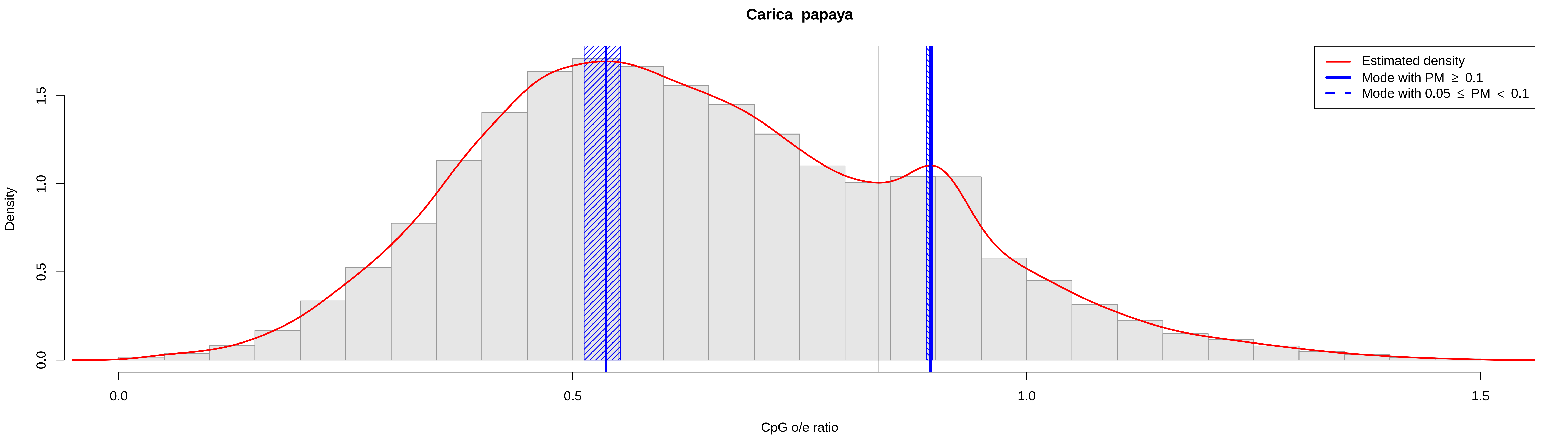

Carica_papaya
Estimated density
Mode with PM ≥ 0.1
Mode with 0.05 ≤ PM < 0.1
Density
0.0
0.5
1.0
1.5
0.0
0.5
1.0
1.5
CpG o/e ratio

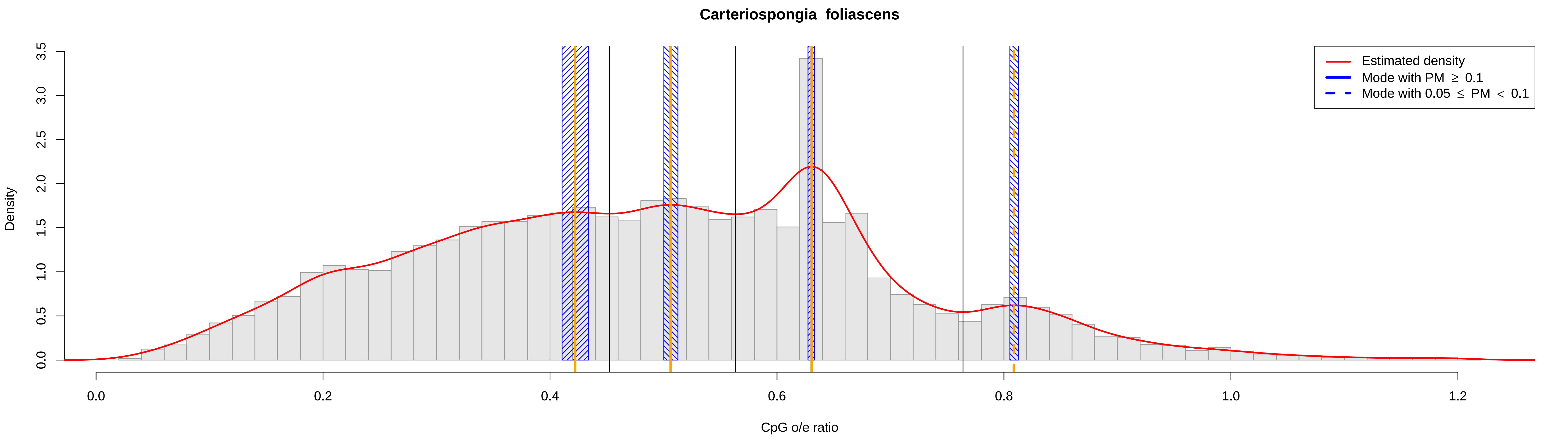

Carteriospongia_foliascens
Estimated density
Mode with PM ≥ 0.1
Mode with 0.05 ≤ PM < 0.1
Density
0.0
0.5
1.0
1.5
2.0
2.5
3.0
3.5
0.0
0.2
0.4
0.6
0.8
1.0
1.2
CpG o/e ratio

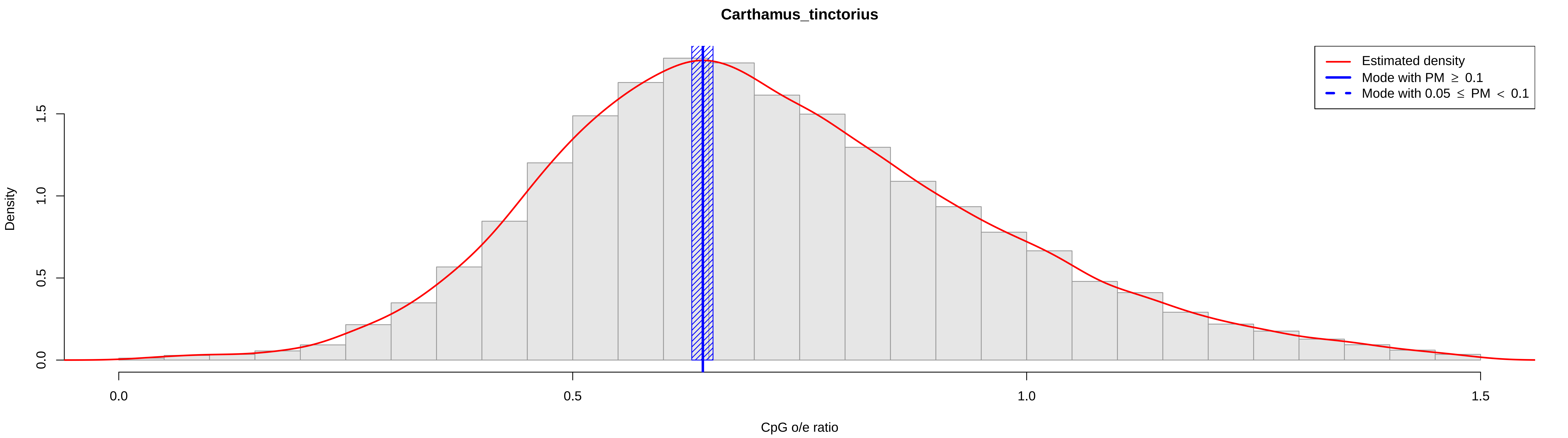

Carthamus_tinctorius
Estimated density
Mode with PM ≥ 0.1
Mode with 0.05 ≤ PM < 0.1
Density
0.0
0.5
1.0
1.5
CpG o/e ratio
0.0
0.5
1.0
1.5

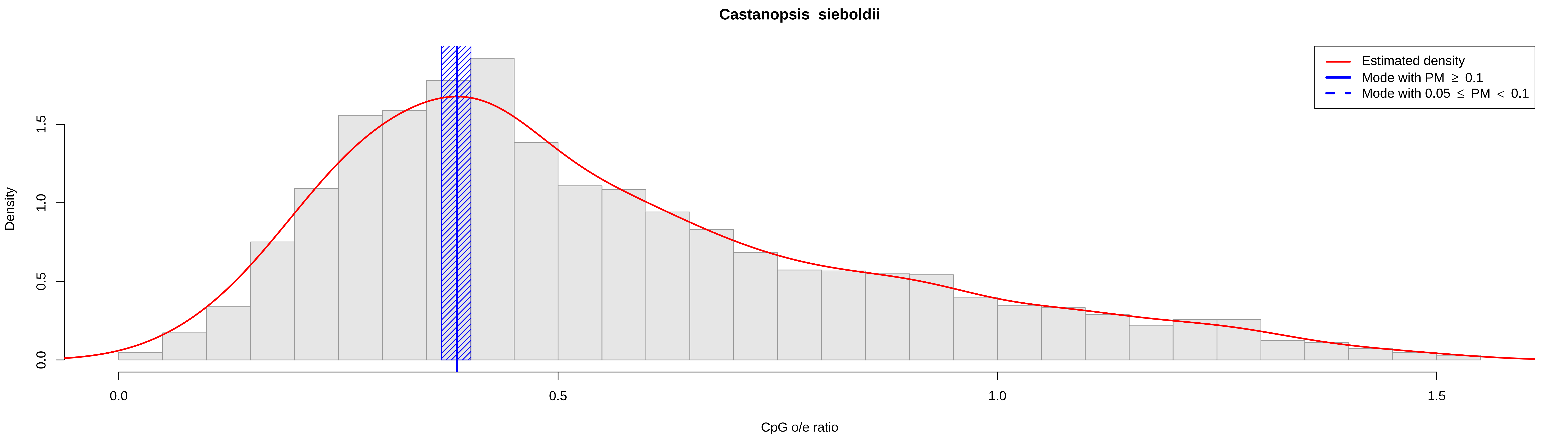

Castanopsis_sieboldii
Estimated density
Mode with PM ≥ 0.1
Mode with 0.05 ≤ PM < 0.1
Density
0.0
0.5
1.0
1.5
0.0
0.5
1.0
1.5
CpG o/e ratio

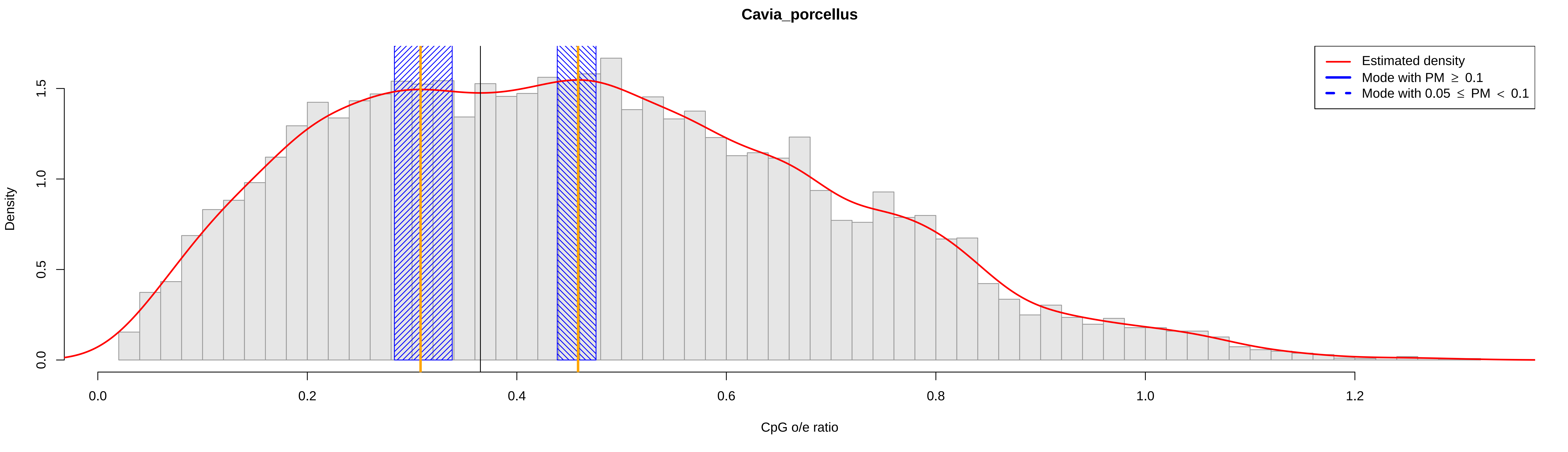

Cavia_porcellus
Estimated density
Mode with PM ≥ 0.1
Mode with 0.05 ≤ PM < 0.1
Density
0.0
0.5
1.0
1.5
0.0
0.2
0.4
0.6
0.8
1.0
1.2
CpG o/e ratio

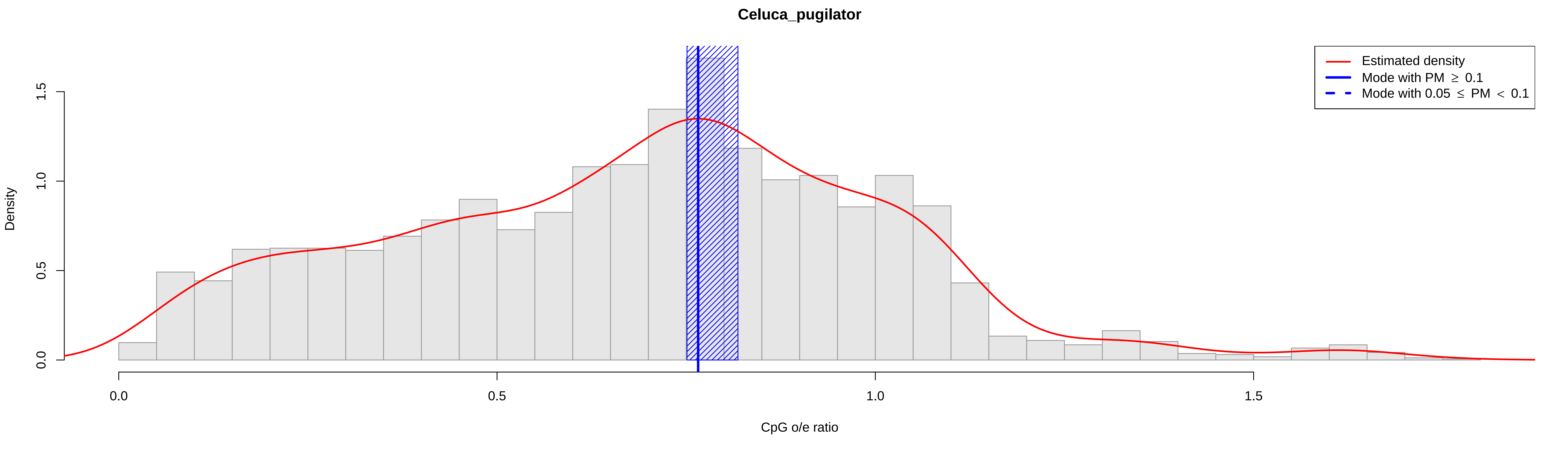

Celuca_pugilator
Estimated density
Mode with PM ≥ 0.1
Mode with 0.05 ≤ PM < 0.1
Density
0.0
0.5
1.0
1.5
0.0
0.5
1.0
1.5
CpG o/e ratio

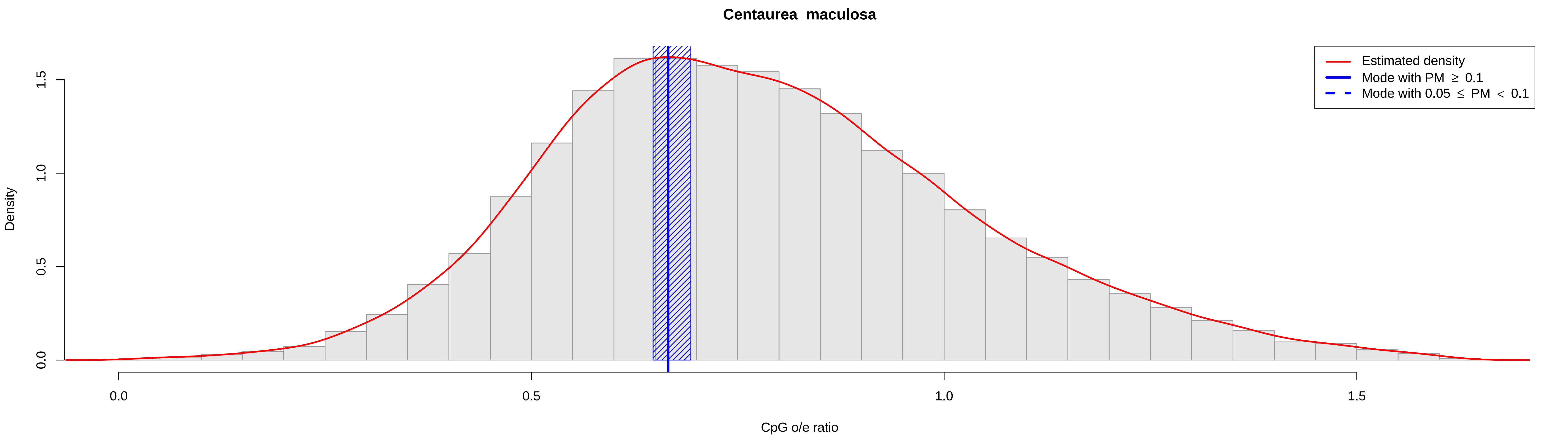
Centaurea_maculosa
Estimated density
Mode with PM ≥ 0.1
Mode with 0.05 ≤ PM < 0.1
Density
0.0
0.5
1.0
1.5
CpG o/e ratio

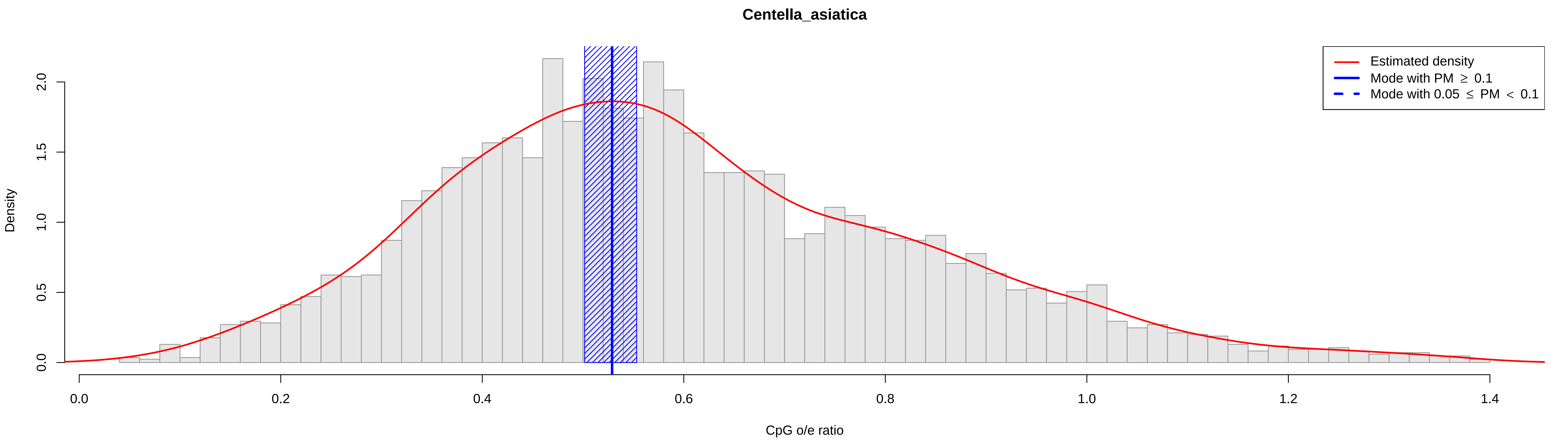

Centella_asiatica
Estimated density
Mode with PM ≥ 0.1
Mode with 0.05 ≤ PM < 0.1
Density
0.0
0.5
1.0
1.5
2.0
0.0
0.2
0.4
0.6
0.8
1.0
1.2
1.4
CpG o/e ratio

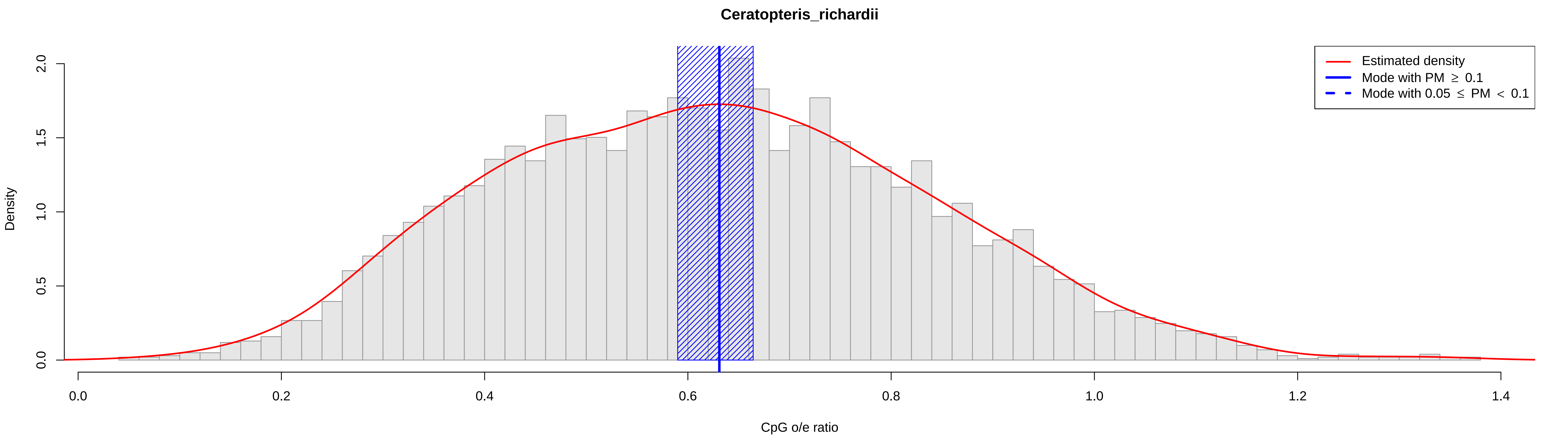
Ceratopteris_richardii
Estimated density
Mode with PM ≥ 0.1
Mode with 0.05 ≤ PM < 0.1
Density
CpG o/e ratio
0.0
0.5
1.0
1.5
2.0
0.0
0.2
0.4
0.6
0.8
1.0
1.2
1.4

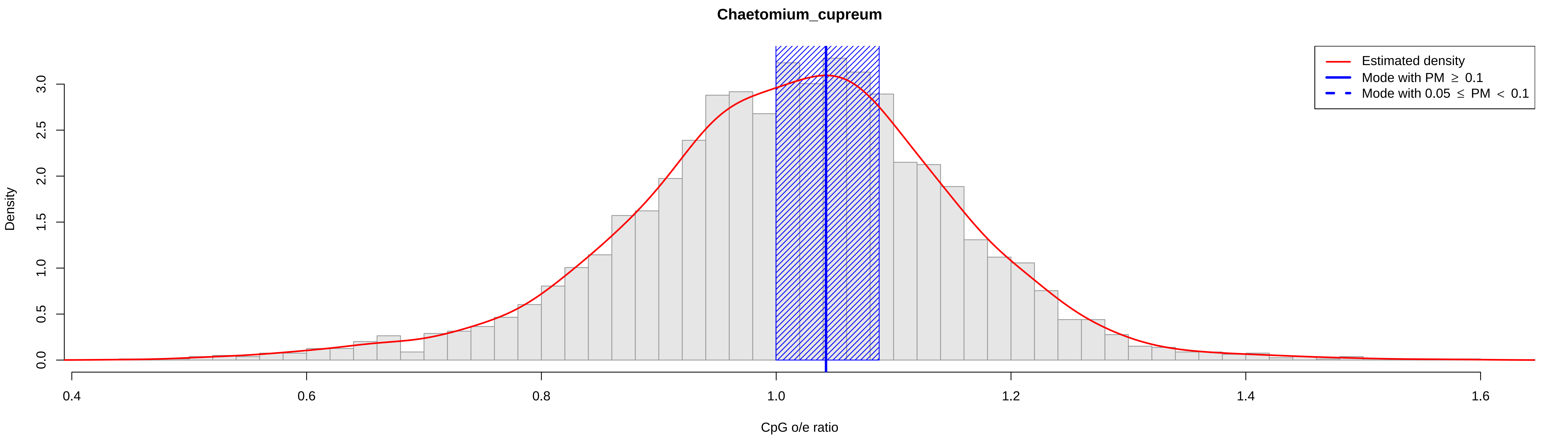

Chaetomium_cupreum
Estimated density
Mode with PM ≥ 0.1
Mode with 0.05 ≤ PM < 0.1
Density
0.0
0.5
1.0
1.5
2.0
2.5
3.0
0.4
0.6
0.8
1.0
1.2
1.4
1.6
CpG o/e ratio

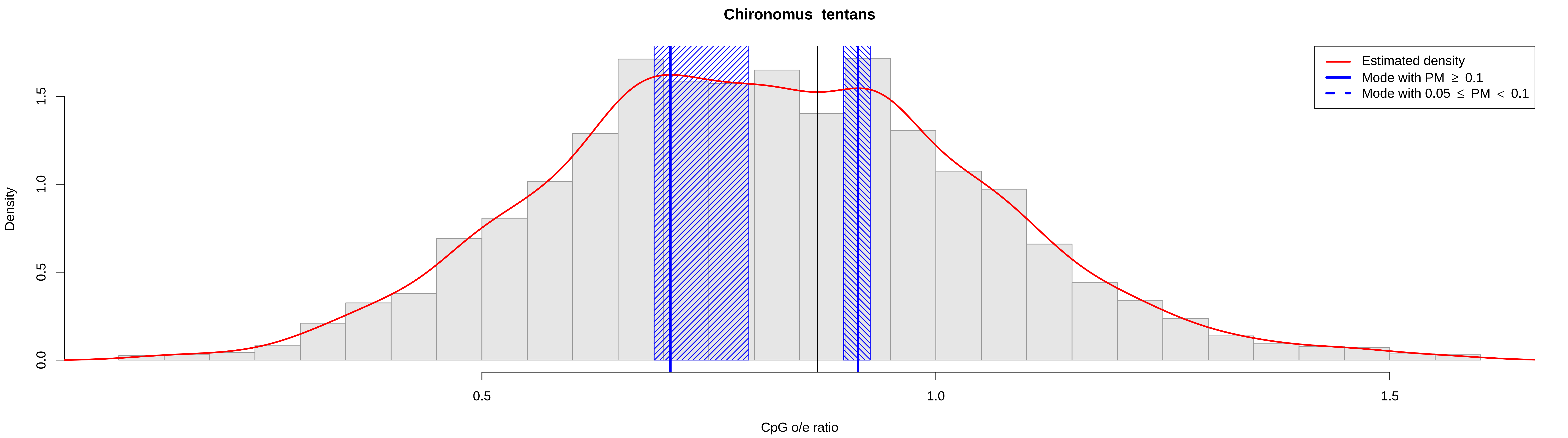

Chironomus_tentans
Estimated density
Mode with PM ≥ 0.1
Mode with 0.05 ≤ PM < 0.1
Density
0.0
0.5
1.0
1.5
0.5
1.0
1.5
CpG o/e ratio

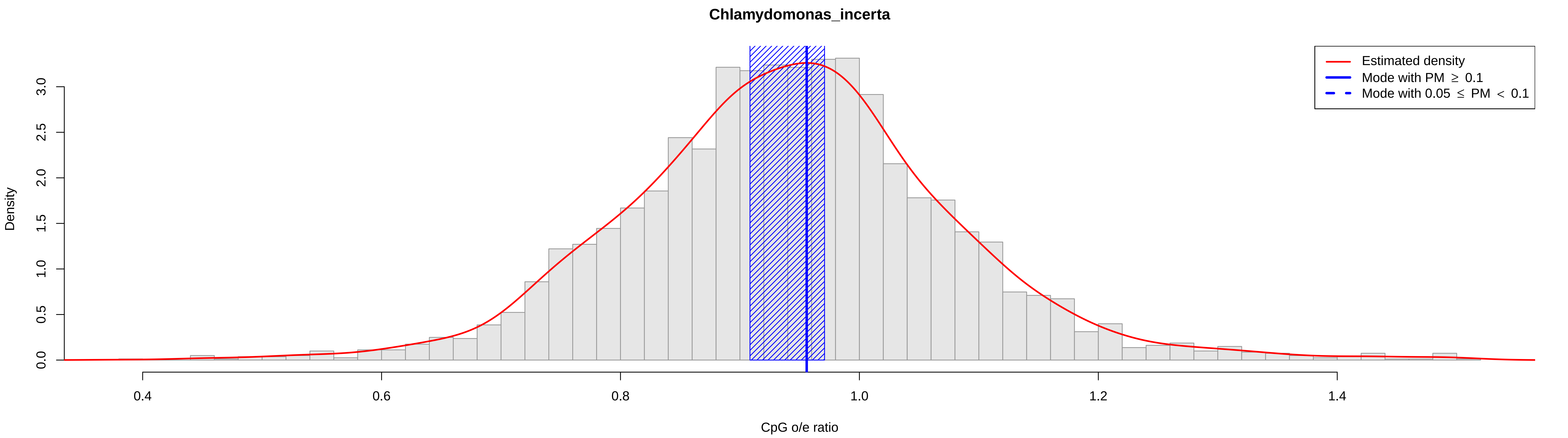

Chlamydomonas_incerta
Estimated density
Mode with PM ≥ 0.1
Mode with 0.05 ≤ PM < 0.1
Density
0.0
0.5
1.0
1.5
2.0
2.5
3.0
0.4
0.6
0.8
1.0
1.2
1.4
CpG o/e ratio

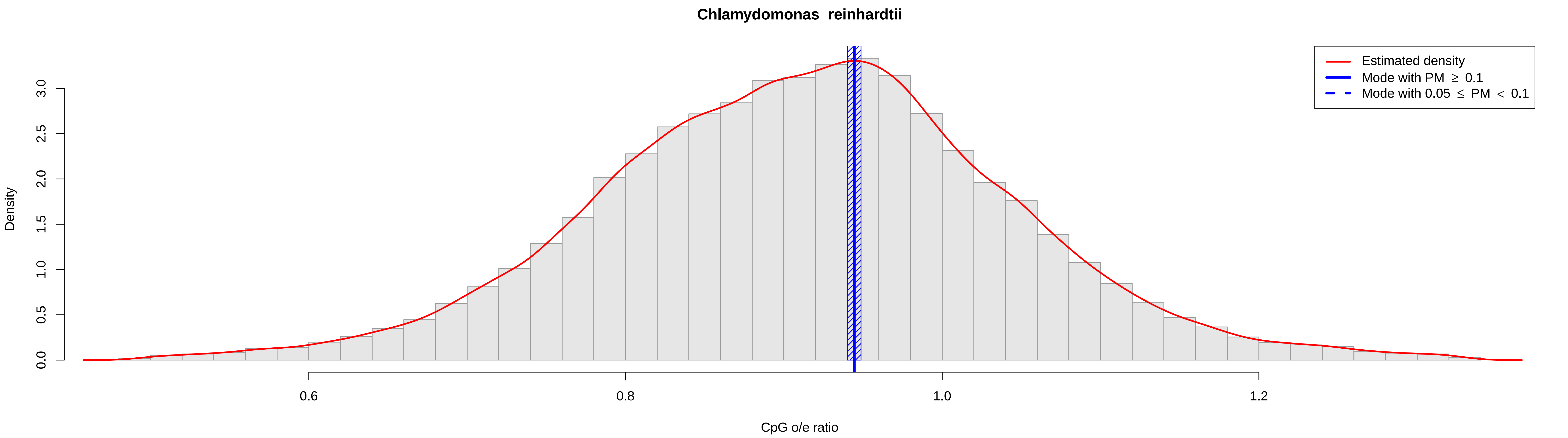
Chlamydomonas_reinhardtii
Estimated density
Mode with PM ≥ 0.1
Mode with 0.05 ≤ PM < 0.1
Density
0.0
0.5
1.0
1.5
2.0
2.5
3.0
0.6
0.8
1.0
1.2
CpG o/e ratio

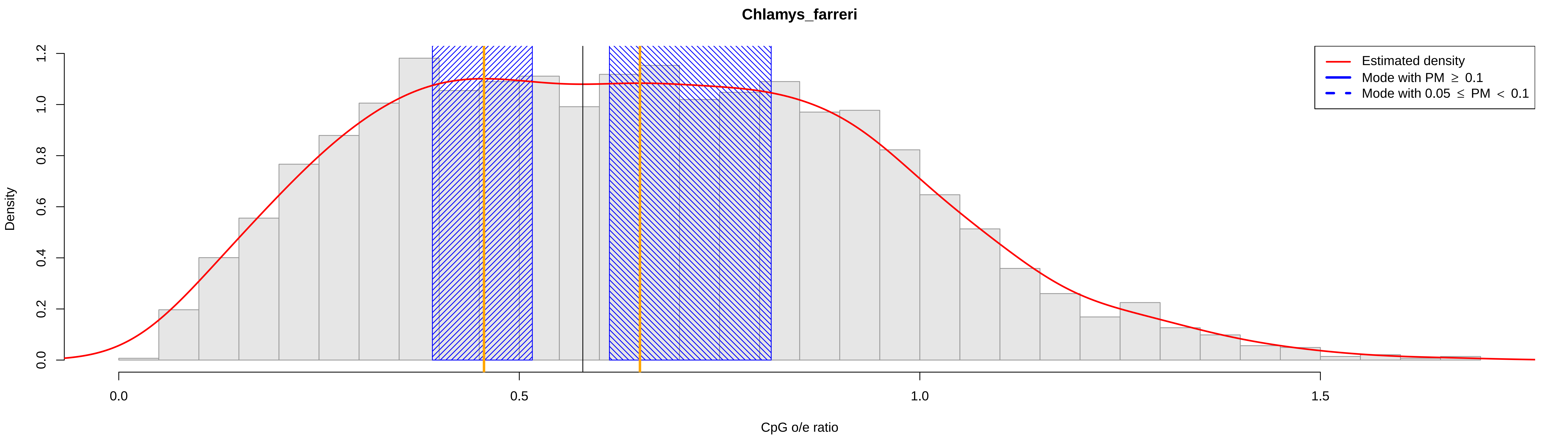

Chlamys_farreri
Estimated density
Mode with PM ≥ 0.1
Mode with 0.05 ≤ PM < 0.1
Density
0.0
0.2
0.4
0.6
0.8
1.0
1.2
0.0
0.5
1.0
1.5
CpG o/e ratio

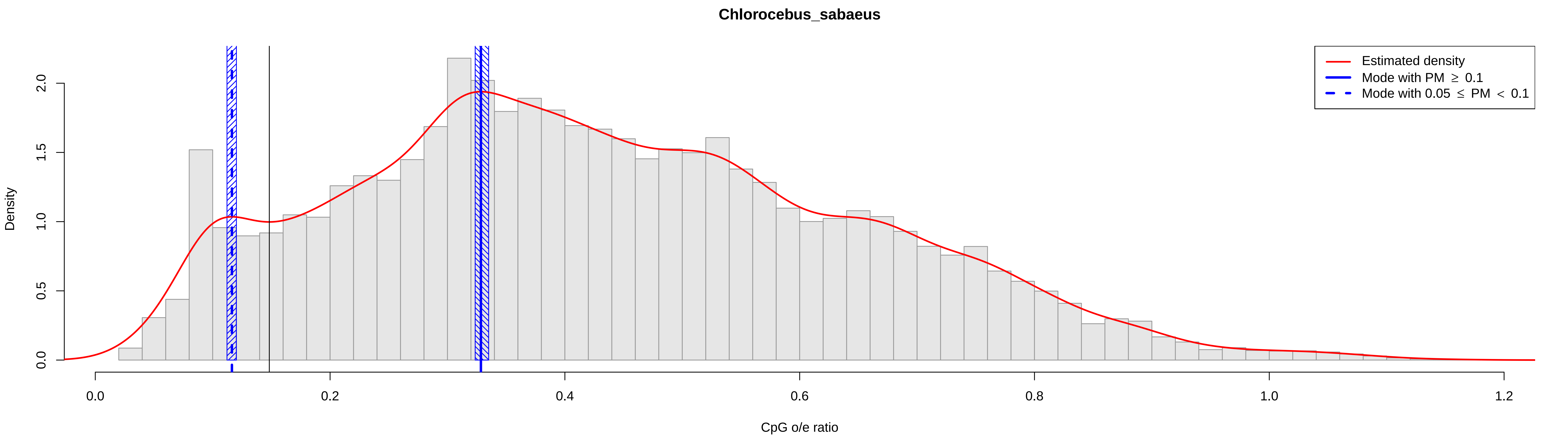

Chlorocebus_sabaeus
Estimated density
Mode with PM ≥ 0.1
Mode with 0.05 ≤ PM < 0.1
Density
0.0
0.5
1.0
1.5
2.0
0.0
0.2
0.4
0.6
0.8
1.0
1.2
CpG o/e ratio

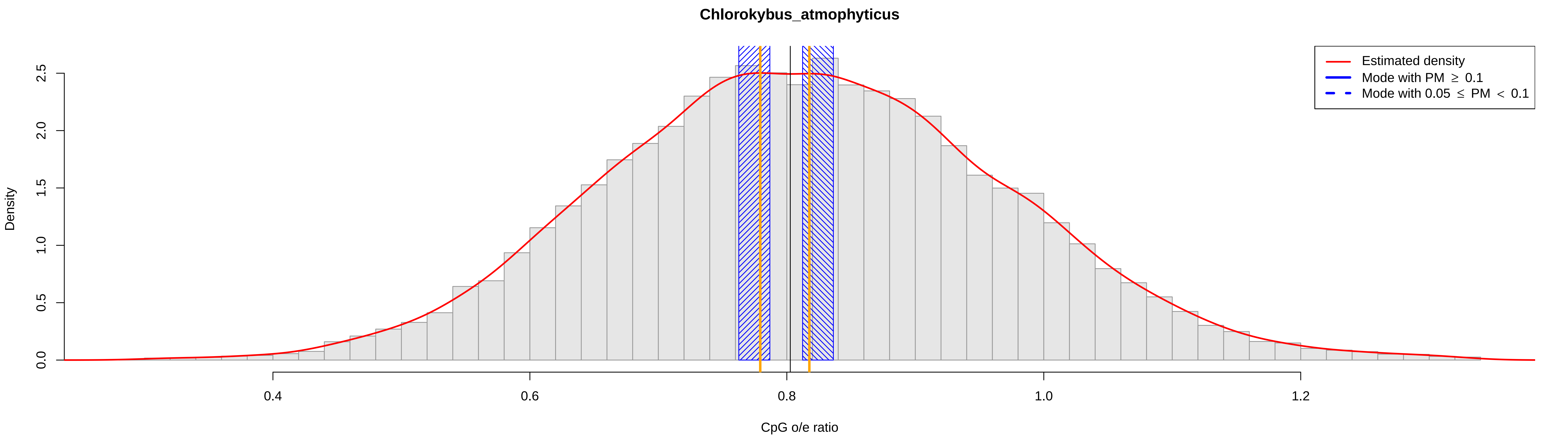

Chlorokybus_atmophyticus
Estimated density
Mode with PM ≥ 0.1
Mode with 0.05 ≤ PM < 0.1
Density
0.0
0.5
1.0
1.5
2.0
2.5
0.4
0.6
0.8
1.0
1.2
CpG o/e ratio

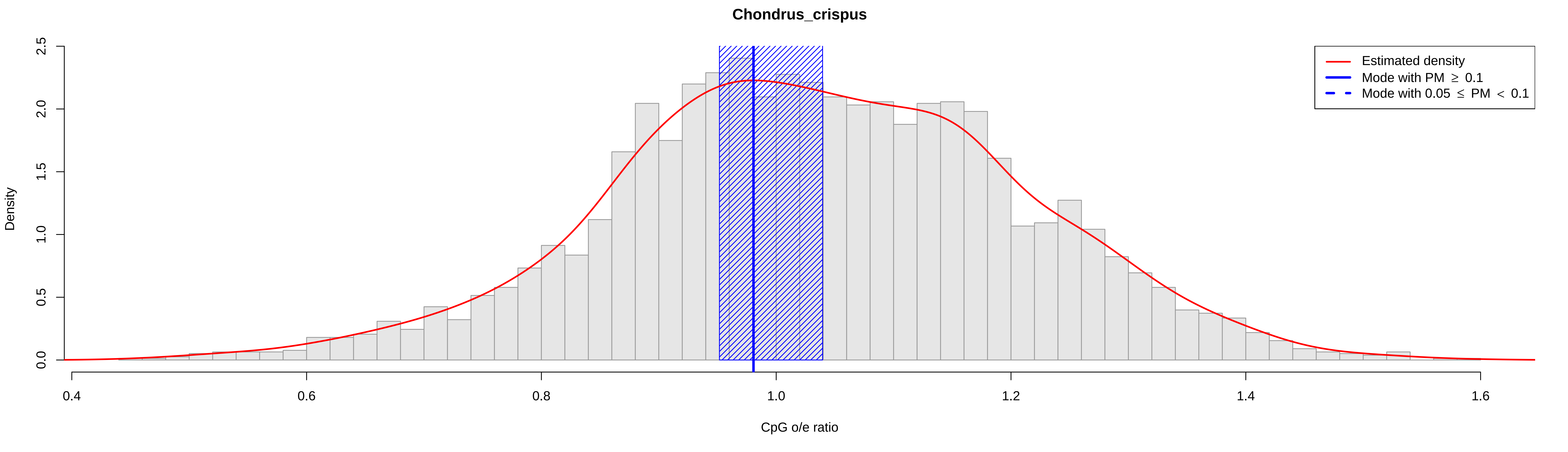

Chondrus_crispus
Density
CpG o/e ratio
0.0
0.5
1.0
1.5
2.0
2.5
0.4
0.6
0.8
1.0
1.2
1.4
1.6
Estimated density
Mode with PM ≥ 0.1
Mode with 0.05 ≤ PM < 0.1

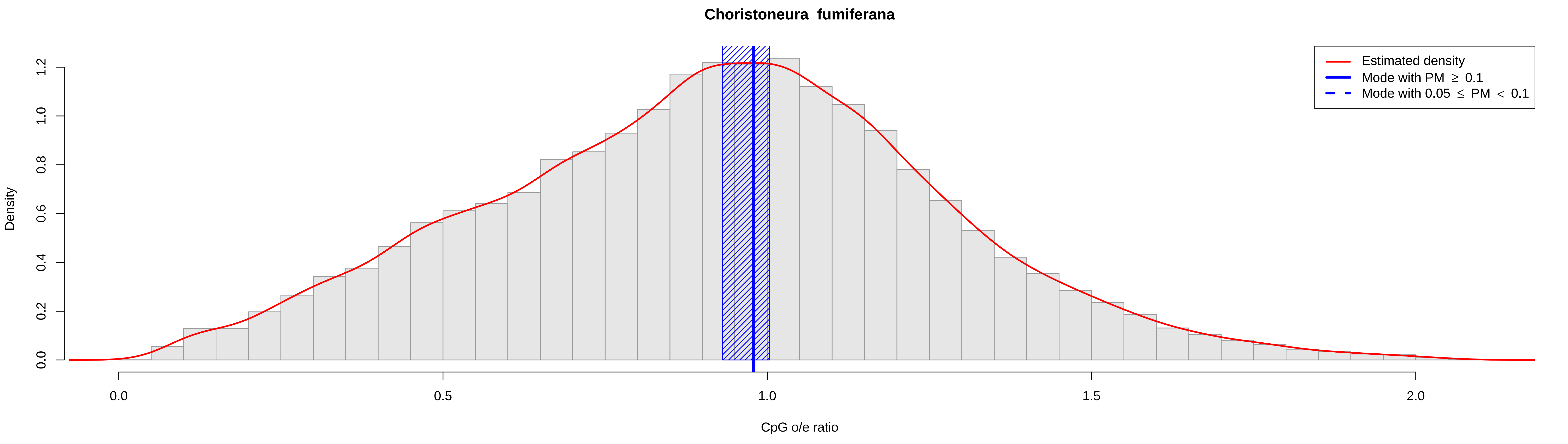

Choristoneura_fumiferana
Estimated density
Mode with PM ≥ 0.1
Mode with 0.05 ≤ PM < 0.1
Density
0.0
0.2
0.4
0.6
0.8
1.0
1.2
0.0
0.5
1.0
1.5
2.0
CpG o/e ratio

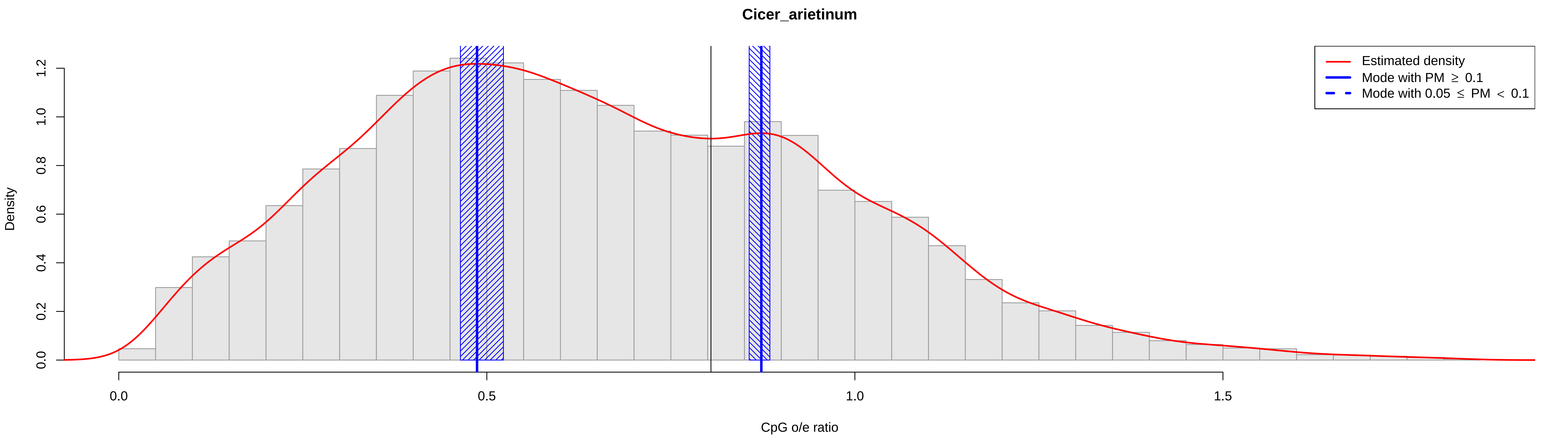
Cicer_arietinum
Estimated density
Mode with PM ≥ 0.1
Mode with 0.05 ≤ PM < 0.1
Density
0.0
0.2
0.4
0.6
0.8
1.0
1.2
0.0
0.5
1.0
1.5
CpG o/e ratio

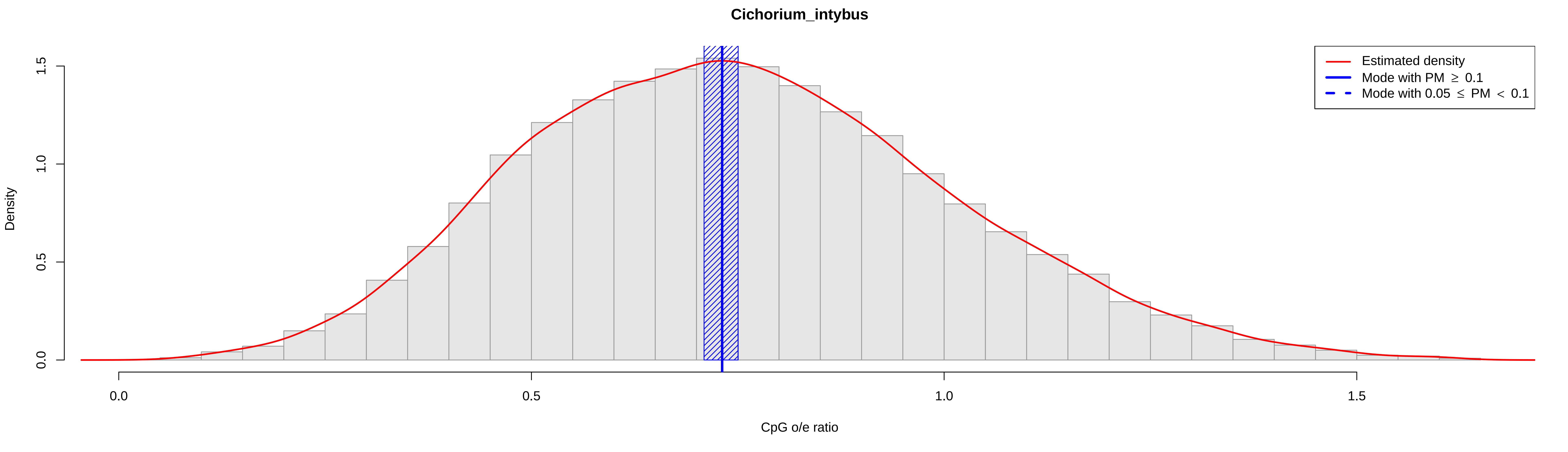

Cichorium_intybus
Estimated density
Mode with PM ≥ 0.1
Mode with 0.05 ≤ PM < 0.1
Density
0.0
0.5
1.0
1.5
CpG o/e ratio

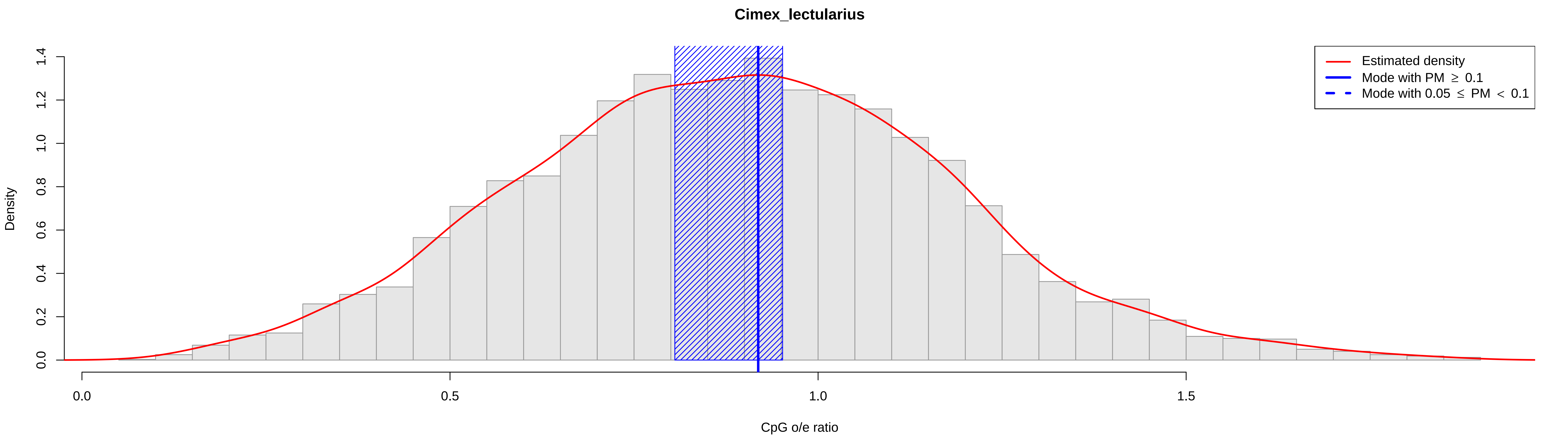

Cimex_lectularius
Estimated density
Mode with PM ≥ 0.1
Mode with 0.05 ≤ PM < 0.1
Density
0.0
0.2
0.4
0.6
0.8
1.0
1.2
1.4
0.0
0.5
1.0
1.5
CpG o/e ratio

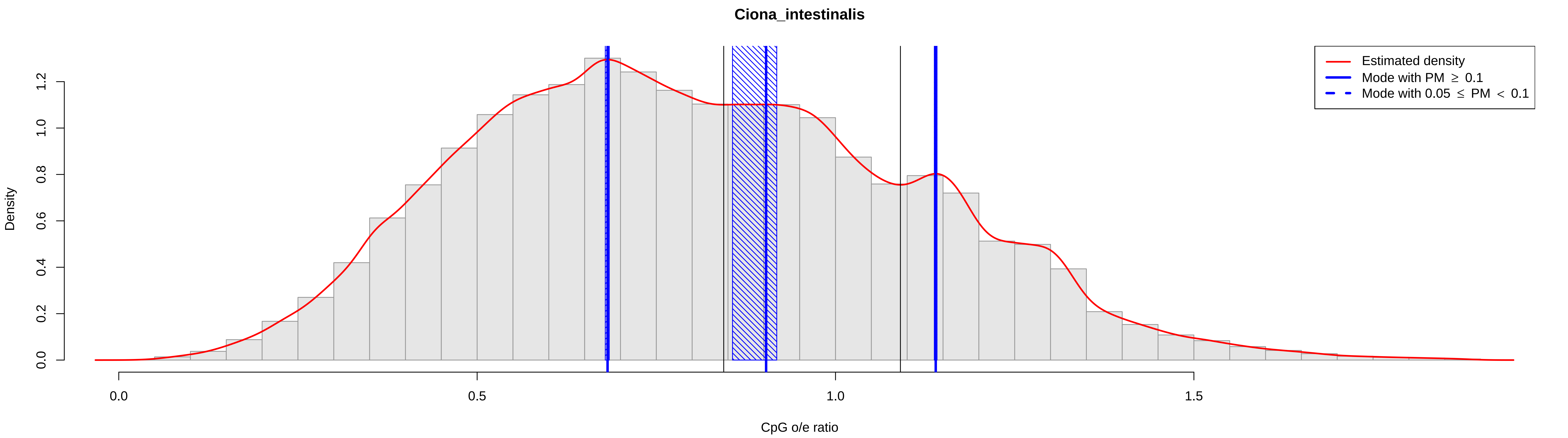

Ciona_intestinalis
Estimated density
Mode with PM ≥ 0.1
Mode with 0.05 ≤ PM < 0.1
Density
0.0
0.2
0.4
0.6
0.8
1.0
1.2
0.0
0.5
1.0
1.5
CpG o/e ratio

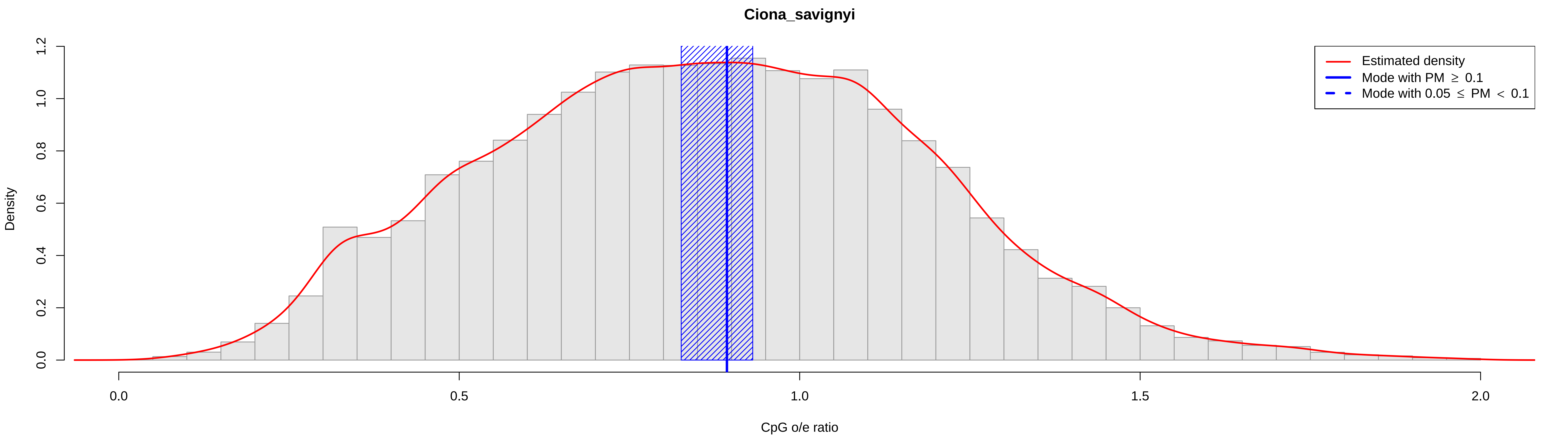
Ciona_savignyi
Estimated density
Mode with PM ≥ 0.1
Mode with 0.05 ≤ PM < 0.1
Density
0.0
0.2
0.4
0.6
0.8
1.0
1.2
0.0
0.5
1.0
1.5
2.0
CpG o/e ratio

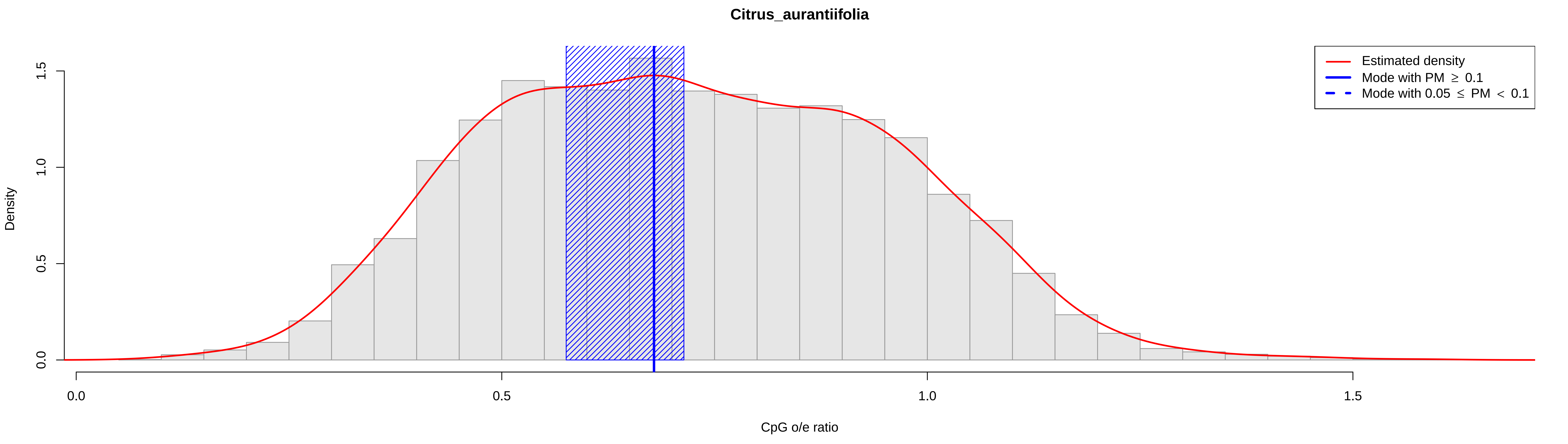

Citrus_aurantiifolia
Estimated density
Mode with PM ≥ 0.1
Mode with 0.05 ≤ PM < 0.1
Density
0.0
0.5
1.0
1.5
0.0
0.5
1.0
1.5
CpG o/e ratio

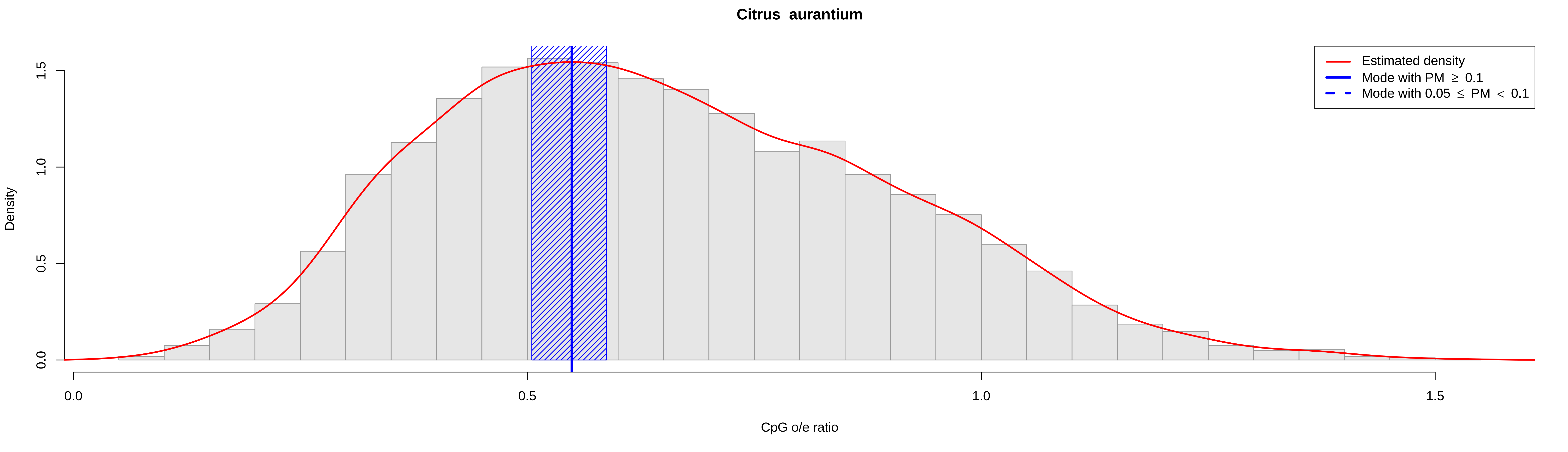

Citrus_aurantium
Estimated density
Mode with PM ≥ 0.1
Mode with 0.05 ≤ PM < 0.1
Density
0.0
0.5
1.0
1.5
0.0
0.5
1.0
1.5
CpG o/e ratio

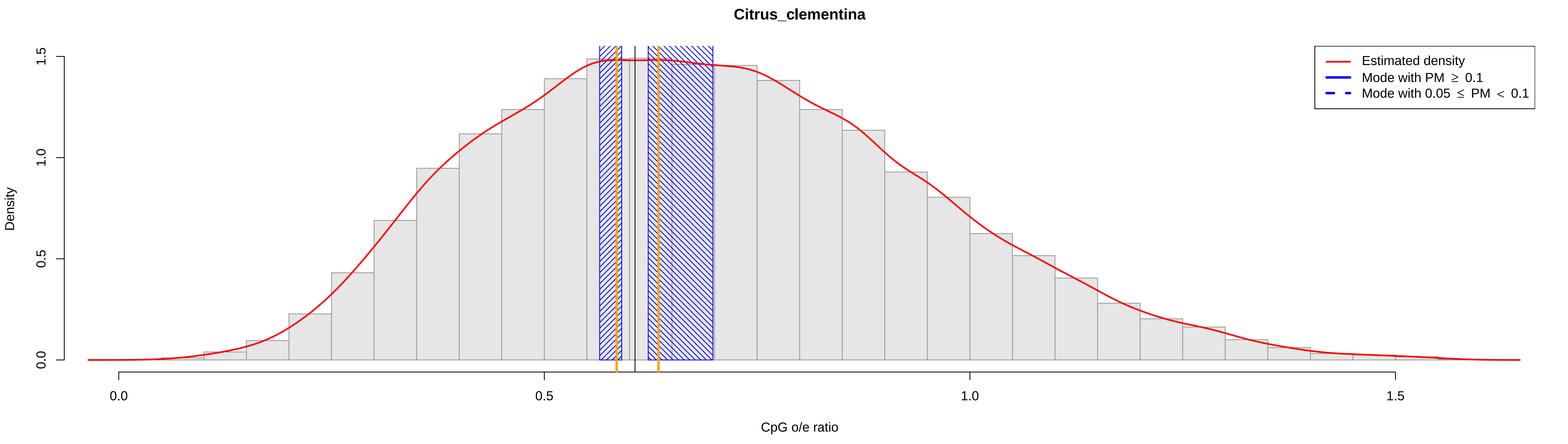
Citrus_clementina
Estimated density
Mode with PM ≥ 0.1
Mode with 0.05 ≤ PM < 0.1
Density
0.0
0.5
1.0
1.5
0.0
0.5
1.0
1.5
CpG o/e ratio

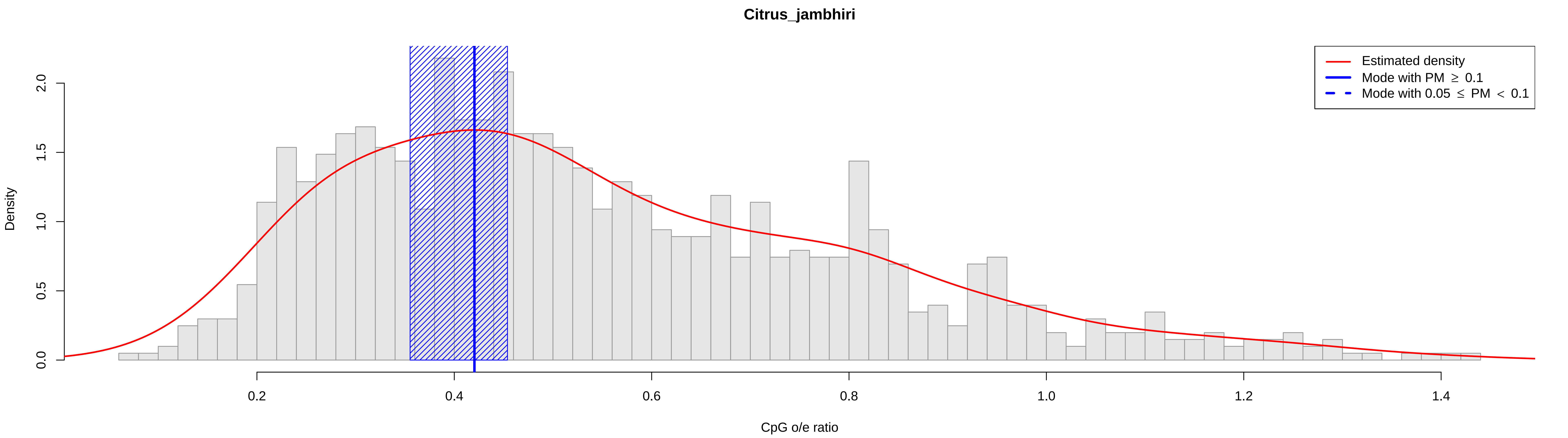

Citrus_jambhiri
Estimated density
Mode with PM ≥ 0.1
Mode with 0.05 ≤ PM < 0.1
Density
0.0
0.5
1.0
1.5
2.0
0.2
0.4
0.6
0.8
1.0
1.2
1.4
CpG o/e ratio

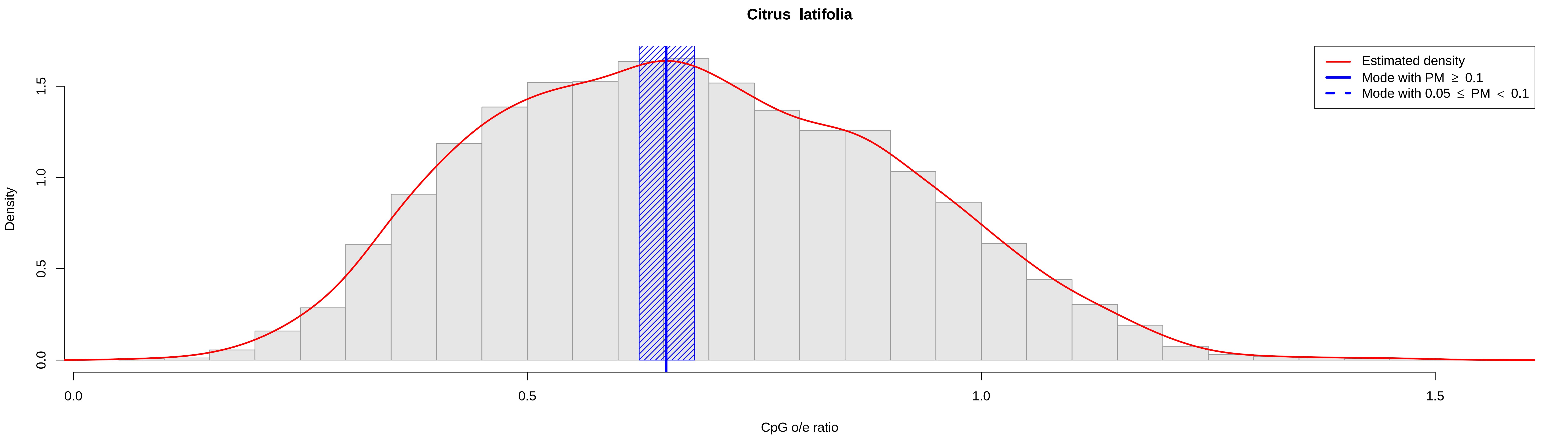

Citrus_latifolia
Estimated density
Mode with PM ≥ 0.1
Mode with 0.05 ≤ PM < 0.1
Density
0.0
0.5
1.0
1.5
CpG o/e ratio

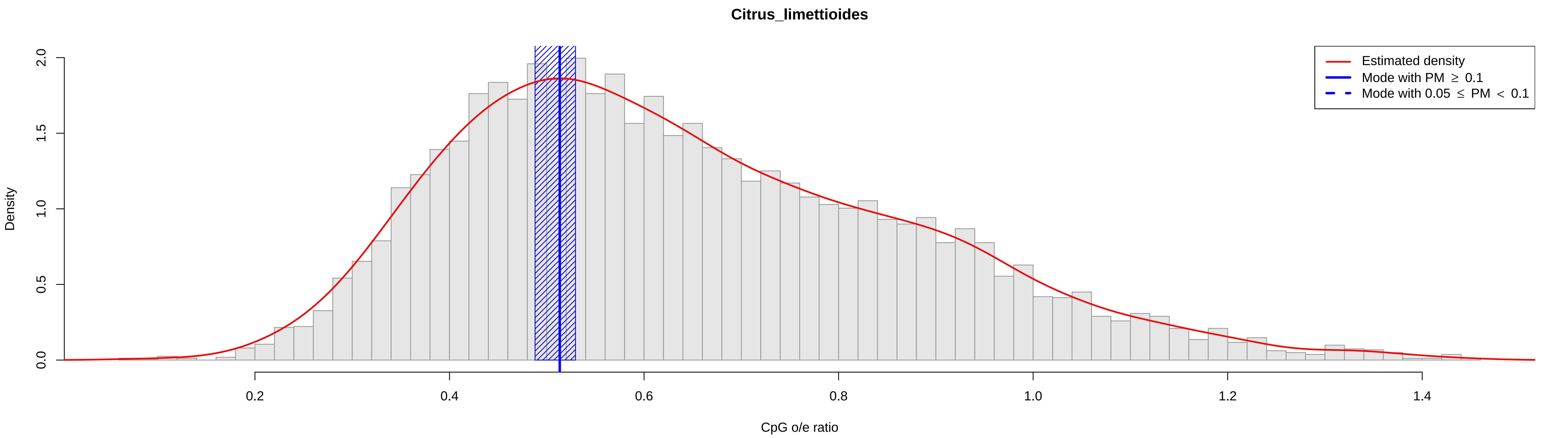

Citrus_limettioides
Estimated density
Mode with PM ≥ 0.1
Mode with 0.05 ≤ PM < 0.1
Density
0.0
0.5
1.0
1.5
2.0
0.2
0.4
0.6
0.8
1.0
1.2
1.4
CpG o/e ratio

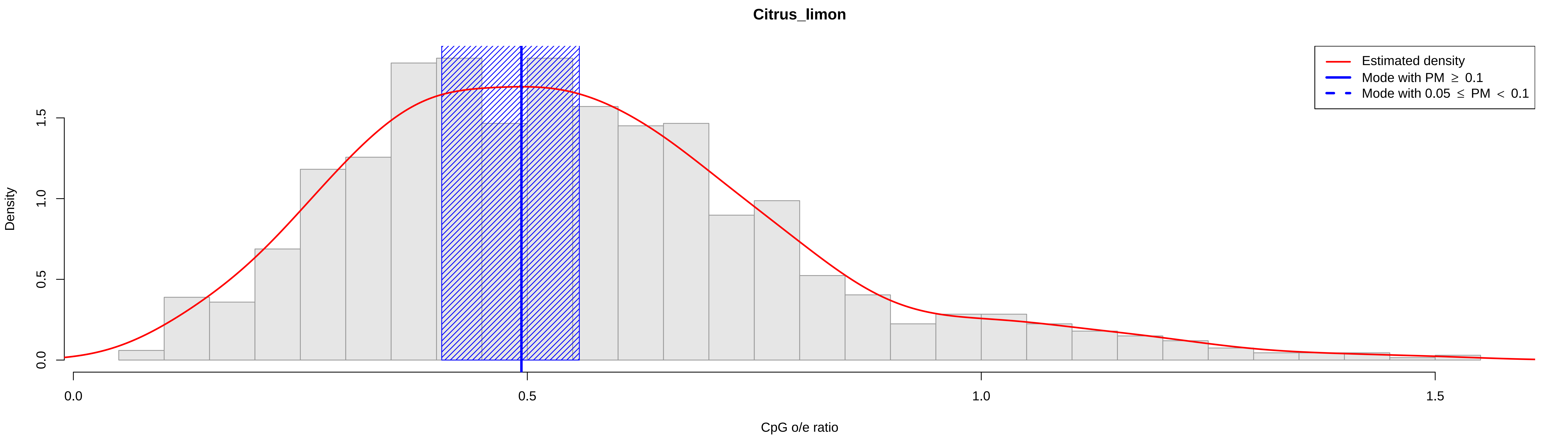

Citrus_limon
Estimated density
Mode with PM ≥ 0.1
Mode with 0.05 ≤ PM < 0.1
Density
0.0
0.5
1.0
1.5
CpG o/e ratio

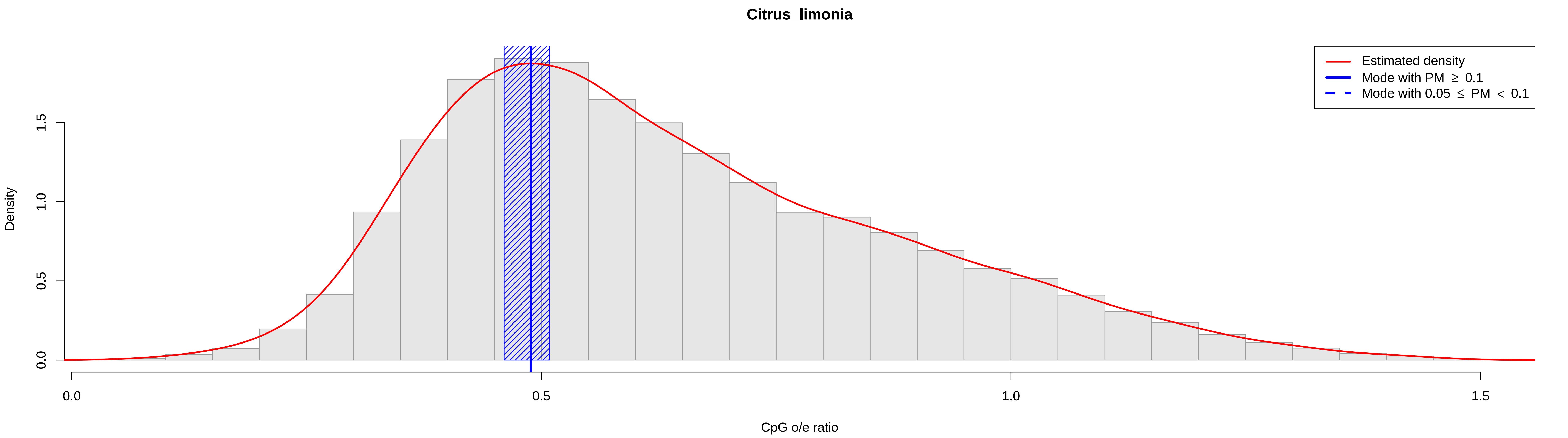

Citrus_limonia
Estimated density
Mode with PM ≥ 0.1
Mode with 0.05 ≤ PM < 0.1
Density
0.0
0.5
1.0
1.5
CpG o/e ratio

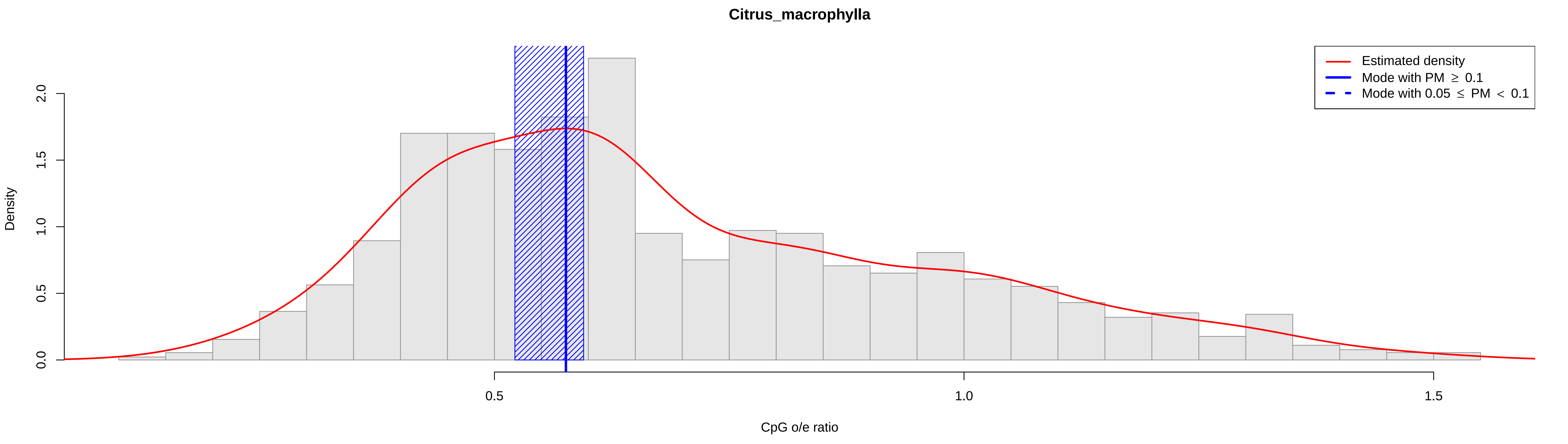
Citrus_macrophylla
Estimated density
Mode with PM ≥ 0.1
Mode with 0.05 ≤ PM < 0.1
Density
0.0
0.5
1.0
1.5
2.0
0.5
1.0
1.5
CpG o/e ratio

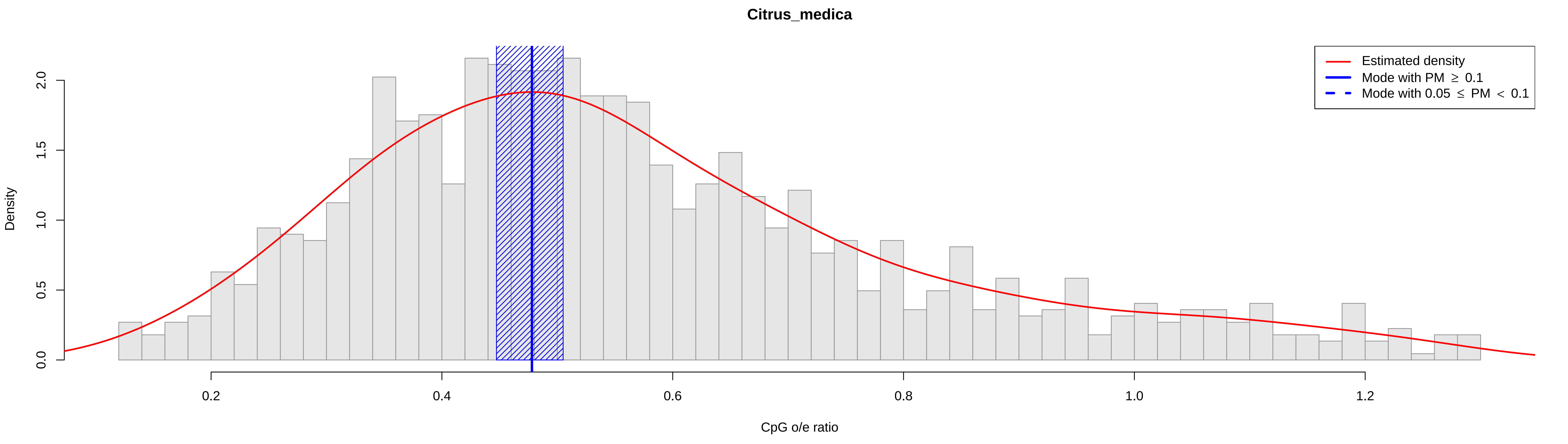
Citrus_medica
Estimated density
Mode with PM ≥ 0.1
Mode with 0.05 ≤ PM < 0.1
Density
0.0
0.5
1.0
1.5
2.0
0.2
0.4
0.6
0.8
1.0
1.2
CpG o/e ratio

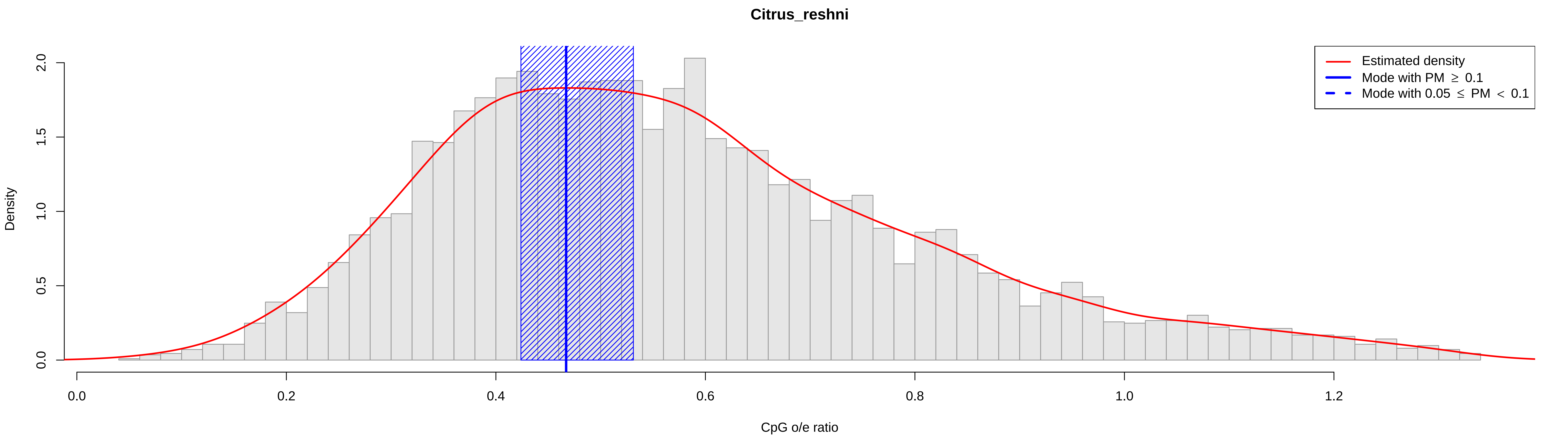

Citrus_reshni
Estimated density
Mode with PM ≥ 0.1
Mode with 0.05 ≤ PM < 0.1
Density
0.0
0.5
1.0
1.5
2.0
0.0
0.2
0.4
0.6
0.8
1.0
1.2
CpG o/e ratio

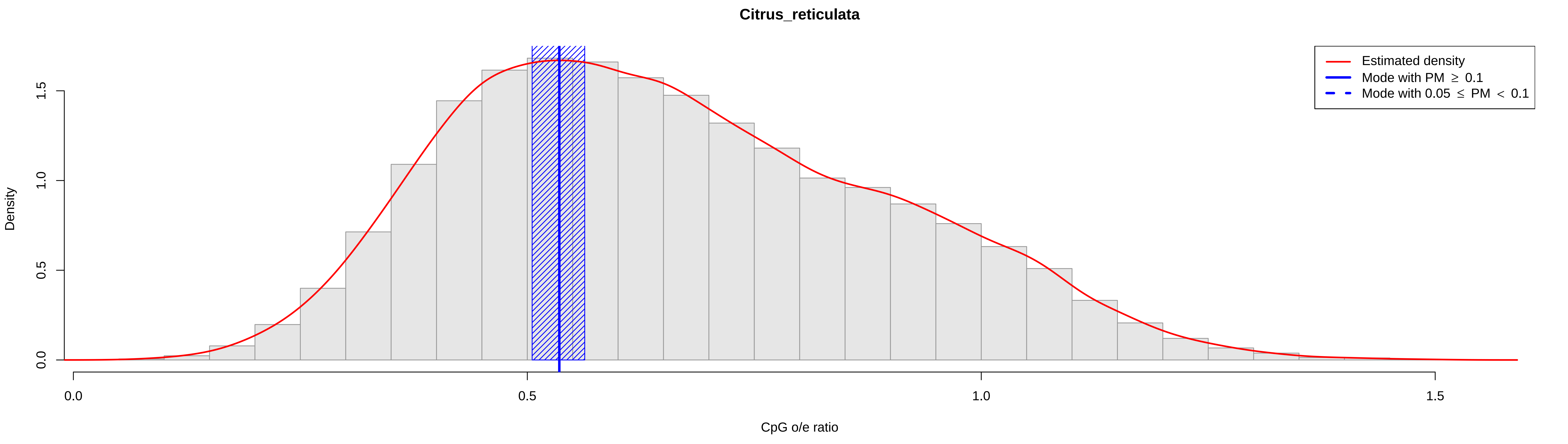

Citrus_reticulata
Estimated density
Mode with PM ≥ 0.1
Mode with 0.05 ≤ PM < 0.1
Density
0.0
0.5
1.0
1.5
0.0
0.5
1.0
1.5
CpG o/e ratio

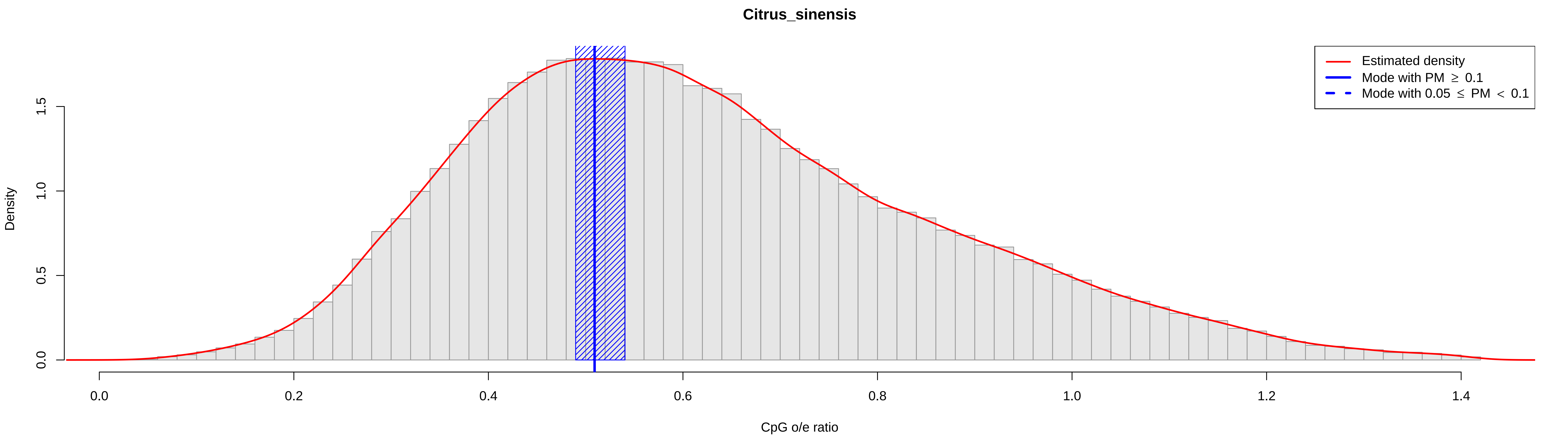
Citrus_sinensis
Estimated density
Mode with PM ≥ 0.1
Mode with 0.05 ≤ PM < 0.1
Density
0.0
0.5
1.0
1.5
0.0
0.2
0.4
0.6
0.8
1.0
1.2
1.4
CpG o/e ratio

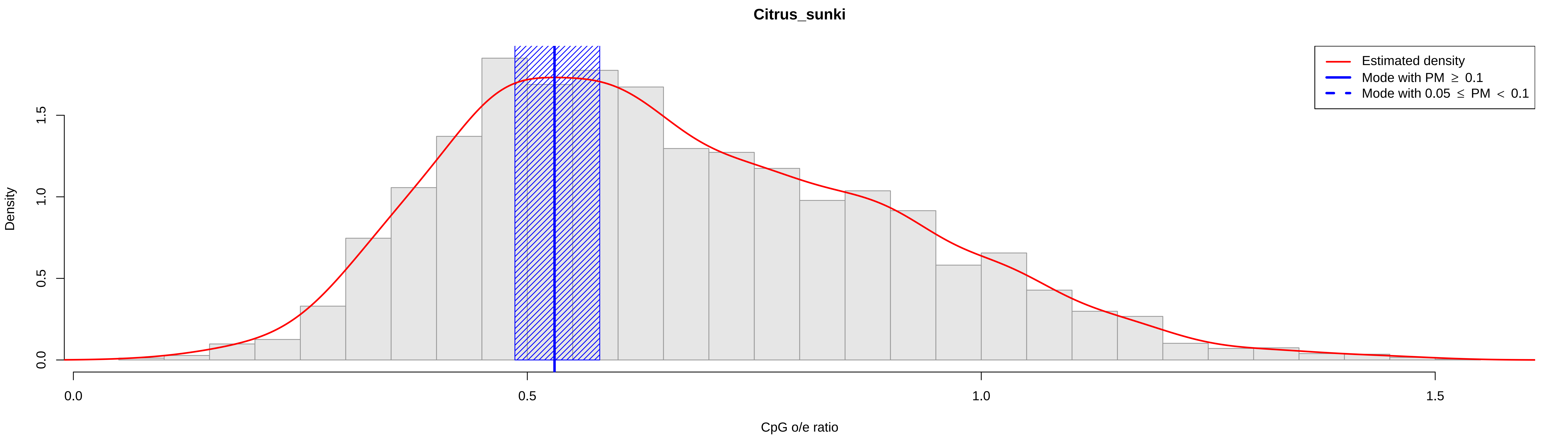

Citrus_sunki
Estimated density
Mode with PM ≥ 0.1
Mode with 0.05 ≤ PM < 0.1
Density
0.0
0.5
1.0
1.5
CpG o/e ratio
0.0
0.5
1.0
1.5

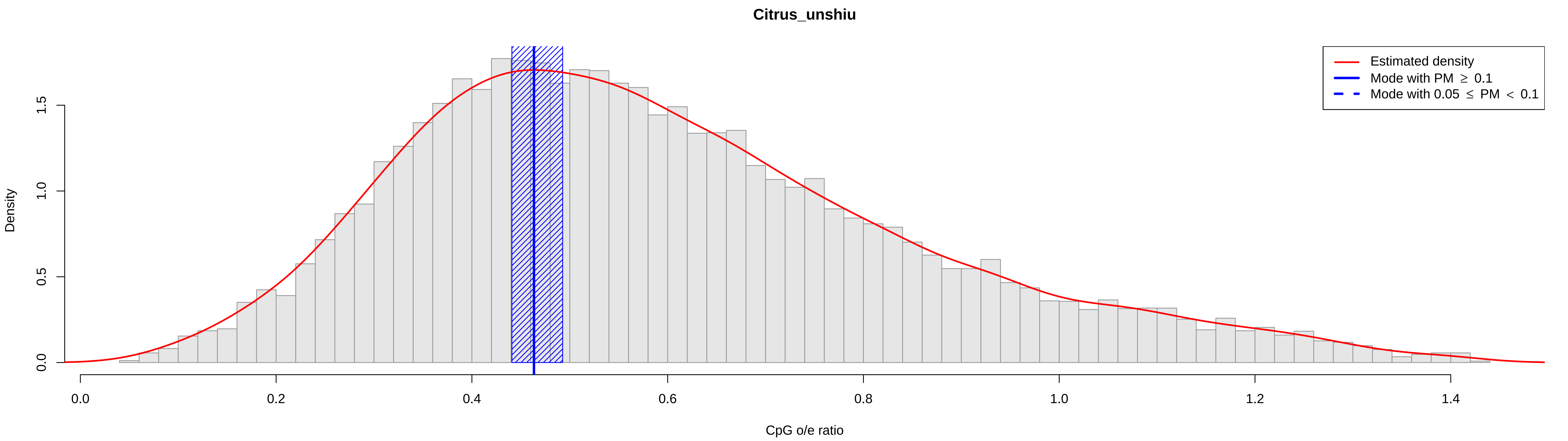

Citrus_unshiu
Estimated density
Mode with PM ≥ 0.1
Mode with 0.05 ≤ PM < 0.1
Density
0.0
0.5
1.0
1.5
0.0
0.2
0.4
0.6
0.8
1.0
1.2
1.4
CpG o/e ratio

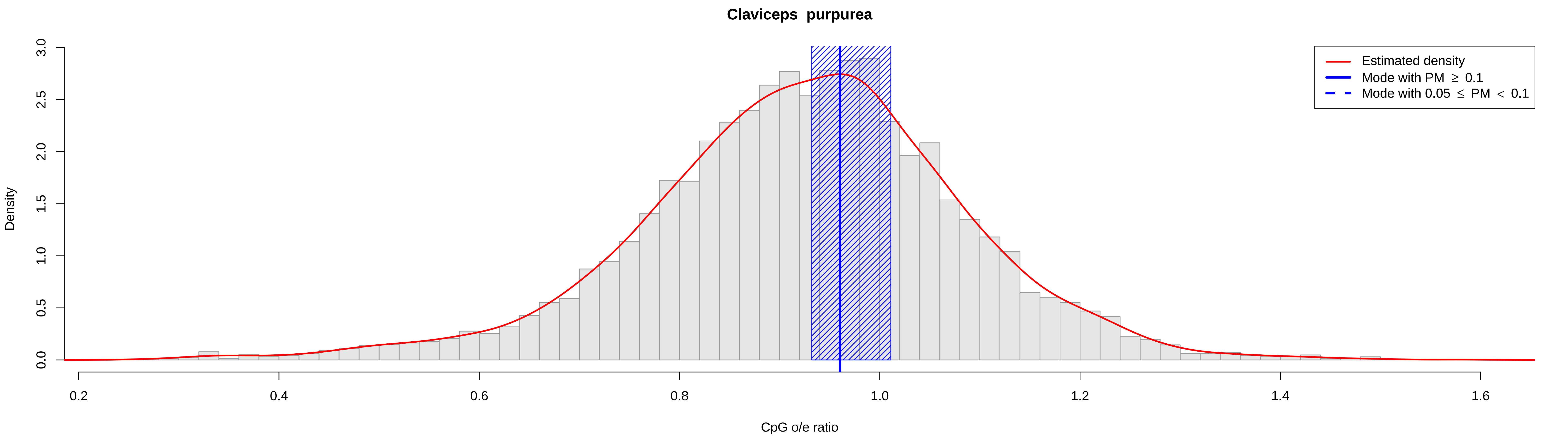

Claviceps_purpurea
Density
CpG o/e ratio
0.0
0.5
1.0
1.5
2.0
2.5
3.0
0.2
0.4
0.6
0.8
1.0
1.2
1.4
1.6
Estimated density
Mode with PM ≥ 0.1
Mode with 0.05 ≤ PM < 0.1

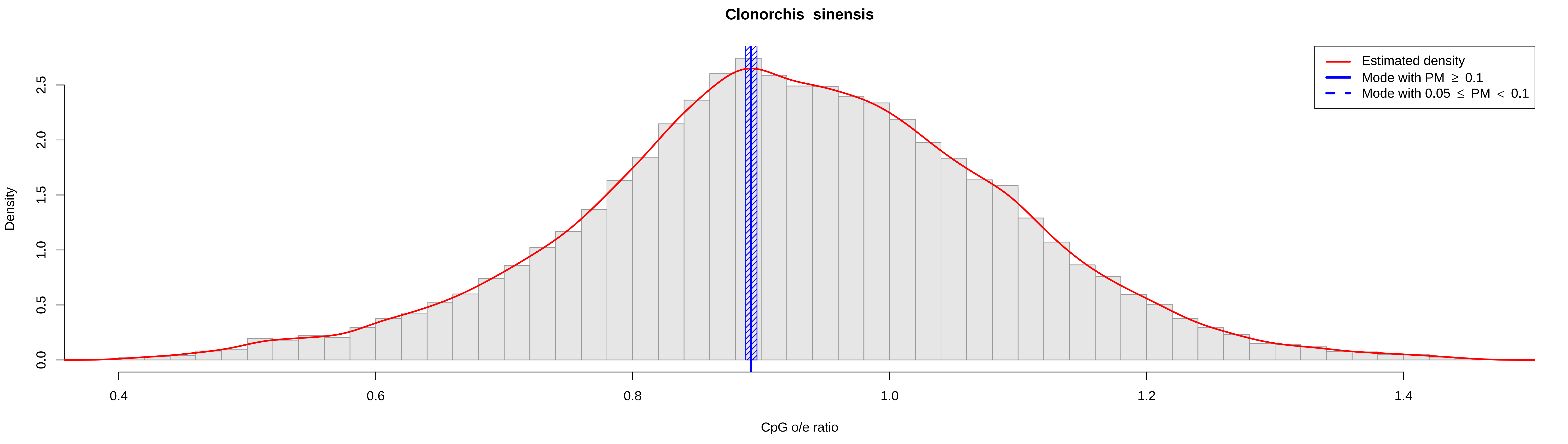

Clonorchis_sinensis
Estimated density
Mode with PM ≥ 0.1
Mode with 0.05 ≤ PM < 0.1
Density
0.0
0.5
1.0
1.5
2.0
2.5
0.4
0.6
0.8
1.0
1.2
1.4
CpG o/e ratio

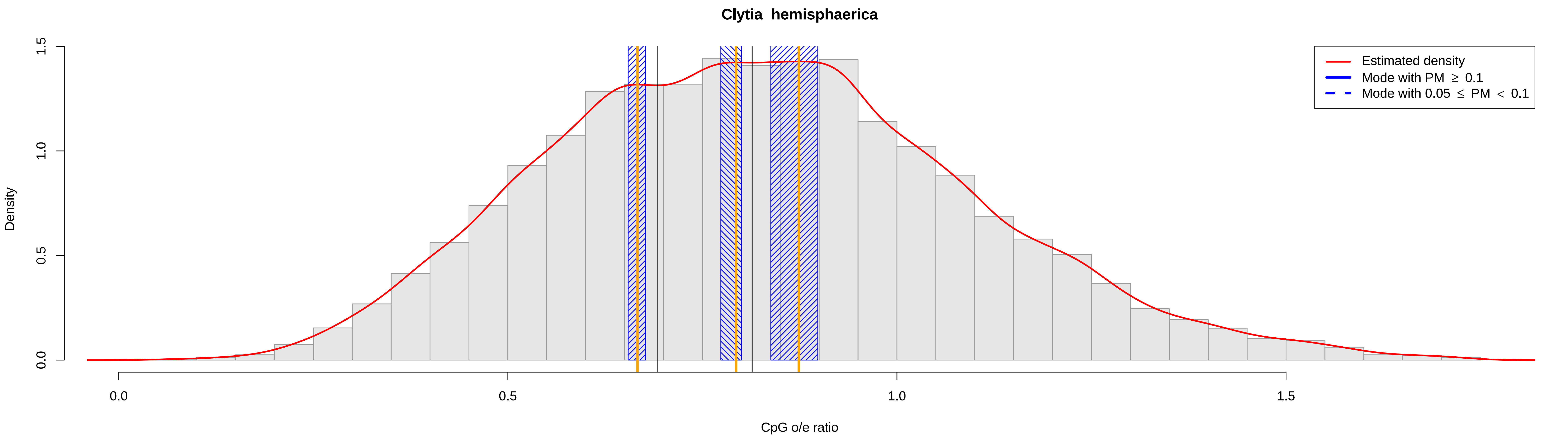

Clytia_hemisphaerica
Density
0.0
0.5
1.0
1.5
CpG o/e ratio
Estimated density
Mode with PM ≥ 0.1
Mode with 0.05 ≤ PM < 0.1

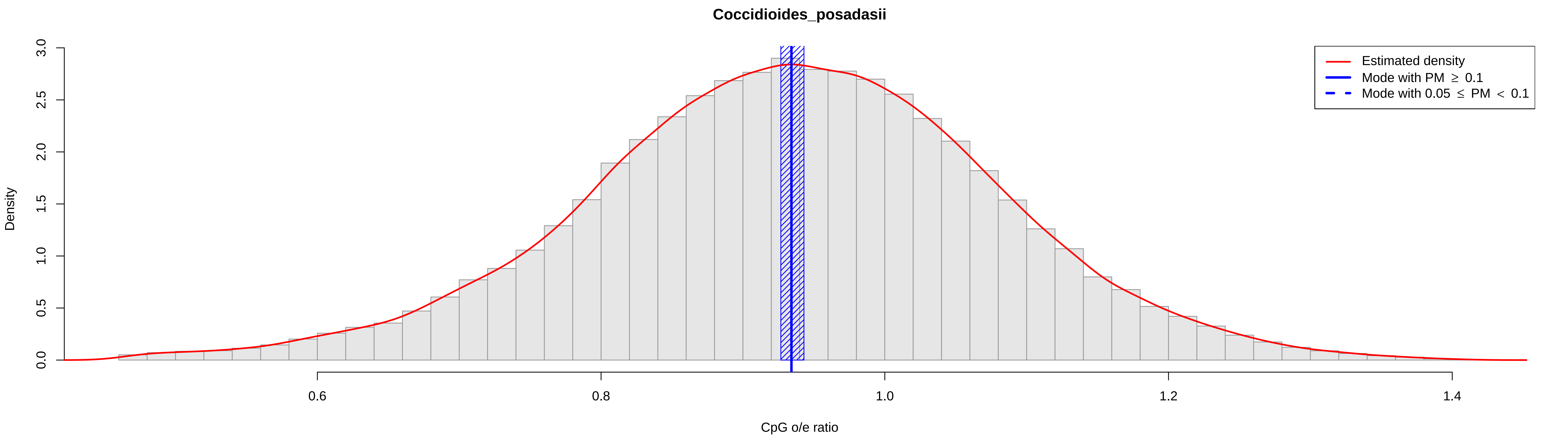
Coccidioides_posadasii
Density
CpG o/e ratio
0.0
0.5
1.0
1.5
2.0
2.5
3.0
0.6
0.8
1.0
1.2
1.4
Estimated density
Mode with PM ≥ 0.1
Mode with 0.05 ≤ PM < 0.1

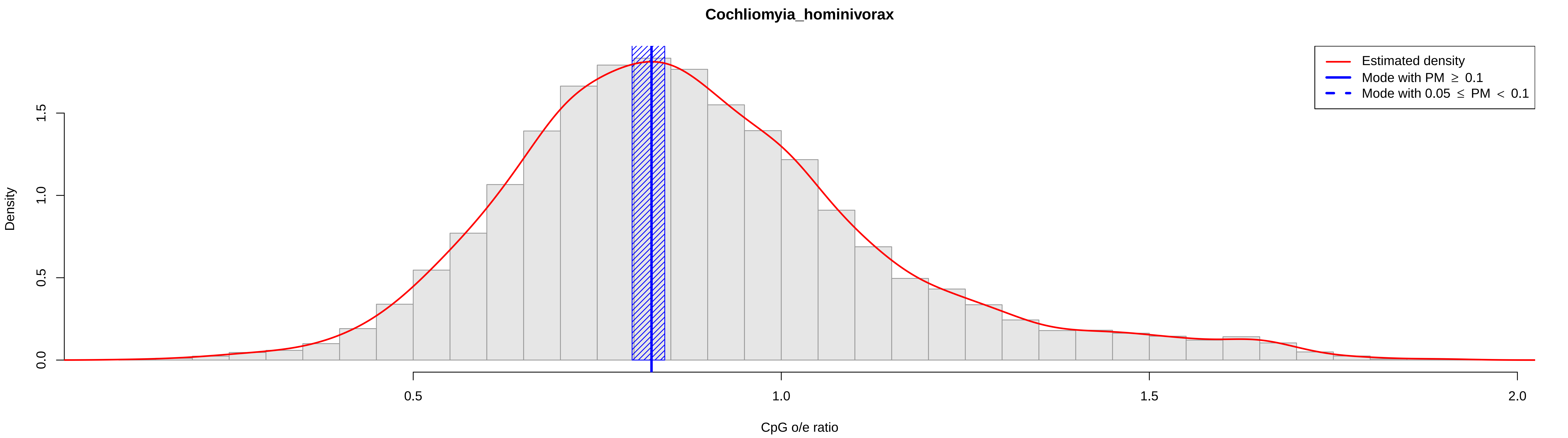

Cochliomyia_hominivorax
Estimated density
Mode with PM ≥ 0.1
Mode with 0.05 ≤ PM < 0.1
Density
0.0
0.5
1.0
1.5
0.5
1.0
1.5
2.0
CpG o/e ratio

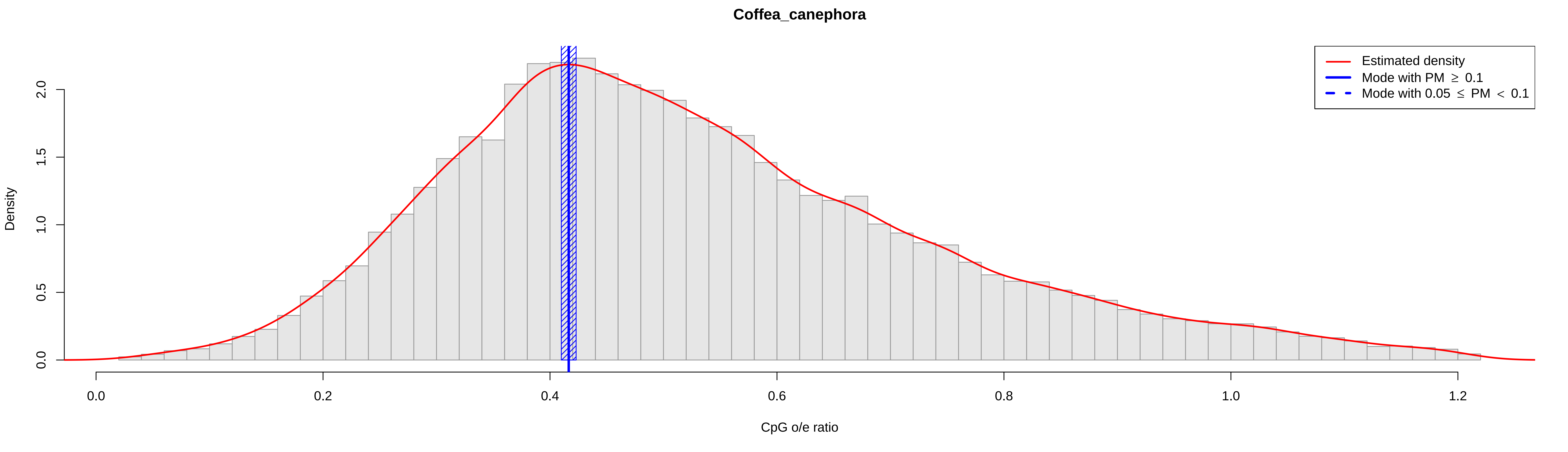
Coffea_canephora
Estimated density
Mode with PM ≥ 0.1
Mode with 0.05 ≤ PM < 0.1
Density
0.0
0.5
1.0
1.5
2.0
0.0
0.2
0.4
0.6
0.8
1.0
1.2
CpG o/e ratio

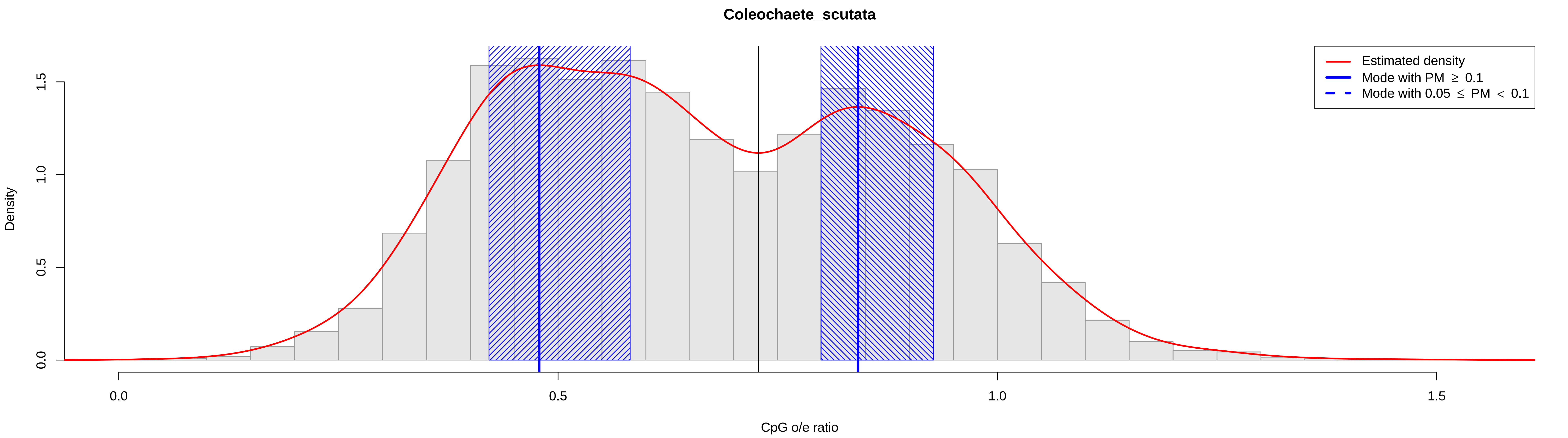
Coleochaete_scutata
Estimated density
Mode with PM ≥ 0.1
Mode with 0.05 ≤ PM < 0.1
Density
0.0
0.5
1.0
1.5
0.0
0.5
1.0
1.5
CpG o/e ratio

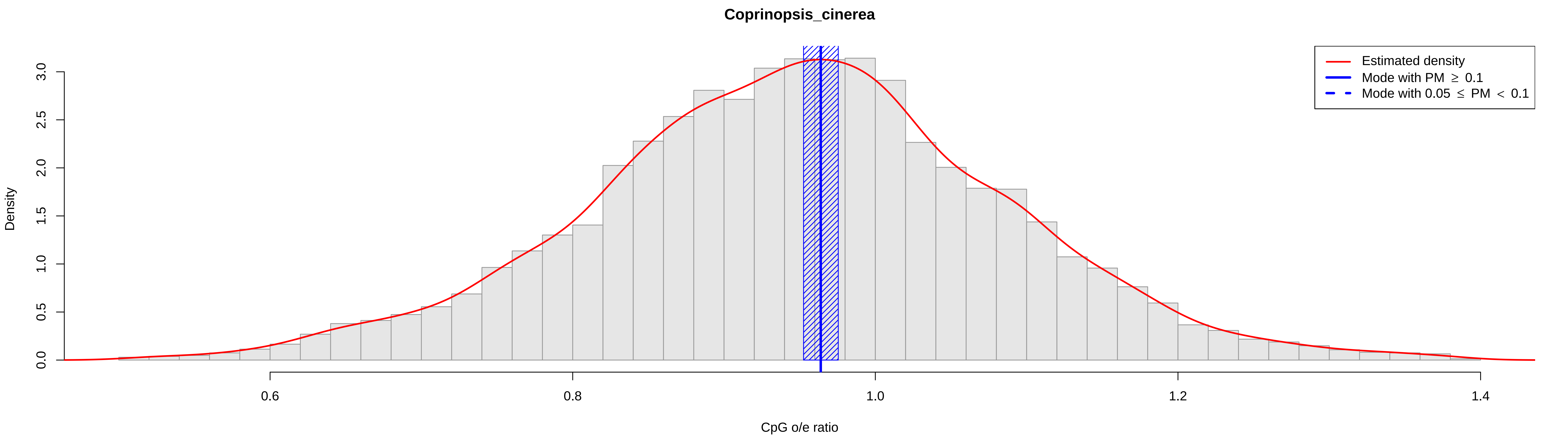

Coprinopsis_cinerea
Estimated density
Mode with PM ≥ 0.1
Mode with 0.05 ≤ PM < 0.1
Density
0.0
0.5
1.0
1.5
2.0
2.5
3.0
0.6
0.8
1.0
1.2
1.4
CpG o/e ratio

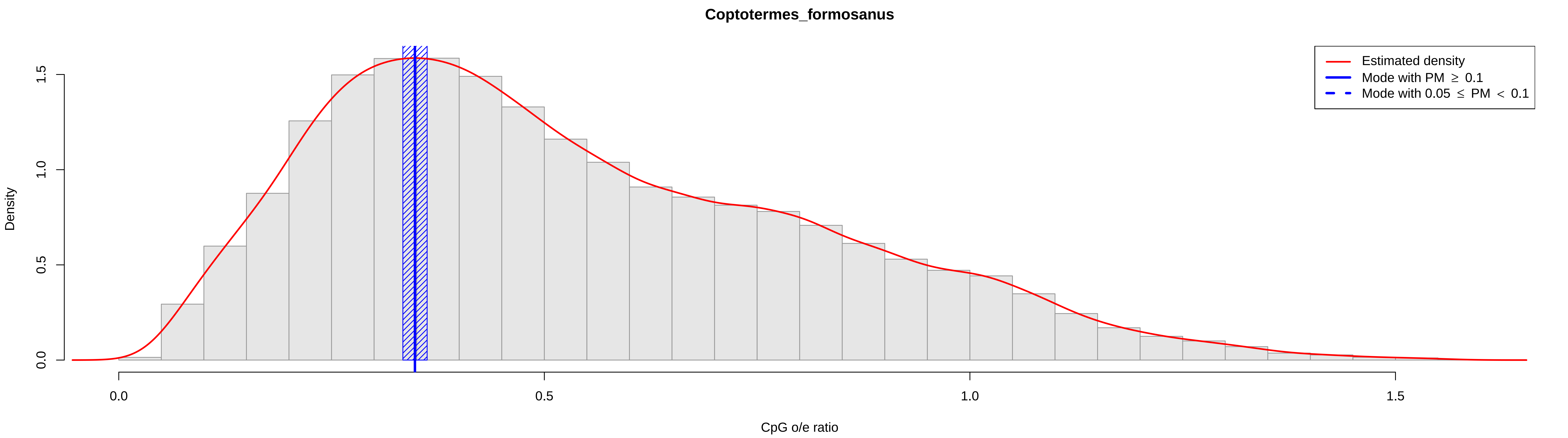
Coptotermes_formosanus
Estimated density
Mode with PM ≥ 0.1
Mode with 0.05 ≤ PM < 0.1
Density
0.0
0.5
1.0
1.5
0.0
0.5
1.0
1.5
CpG o/e ratio

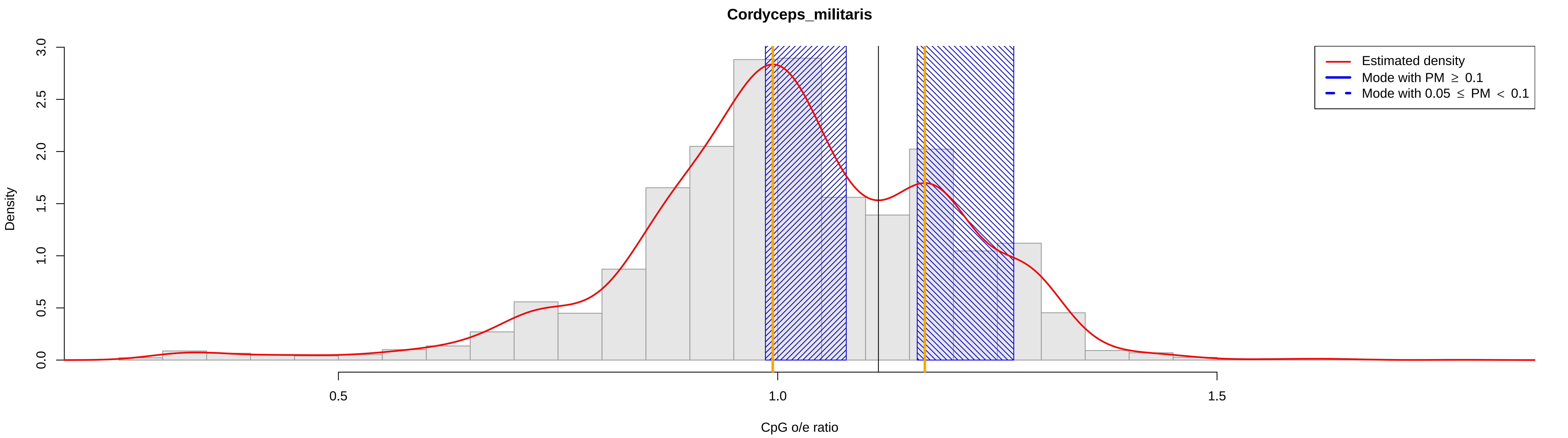
Cordyceps_militaris
Density
0.0
0.5
1.0
1.5
2.0
2.5
3.0
0.5
1.0
1.5
CpG o/e ratio
Estimated density
Mode with PM ≥ 0.1
Mode with 0.05 ≤ PM < 0.1

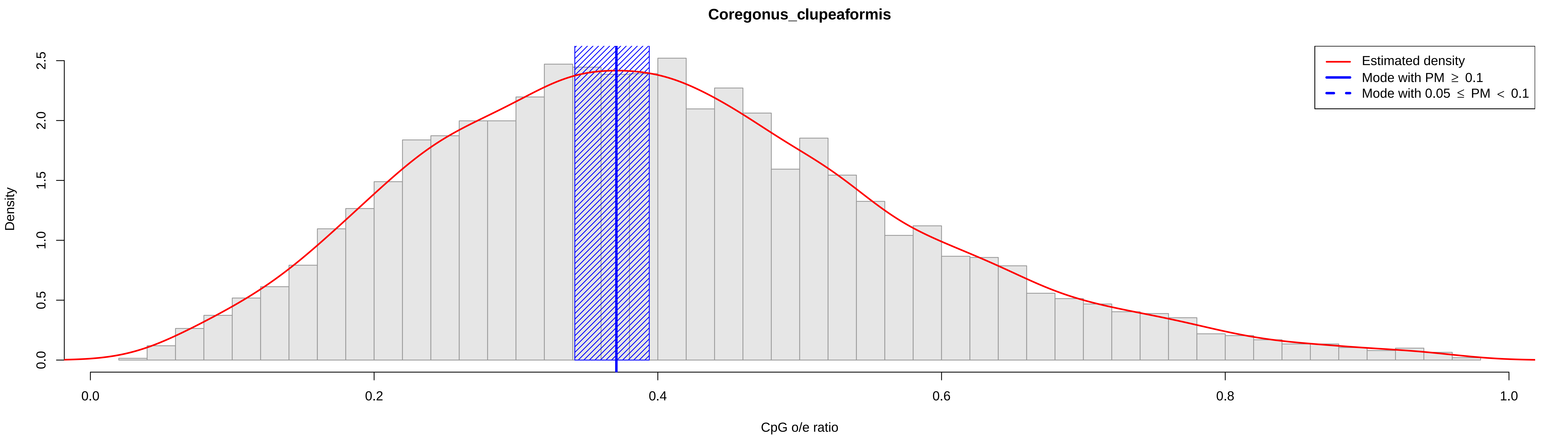

Coregonus_clupeaformis
Estimated density
Mode with PM ≥ 0.1
Mode with 0.05 ≤ PM < 0.1
Density
0.0
0.5
1.0
1.5
2.0
2.5
0.0
0.2
0.4
0.6
0.8
1.0
CpG o/e ratio

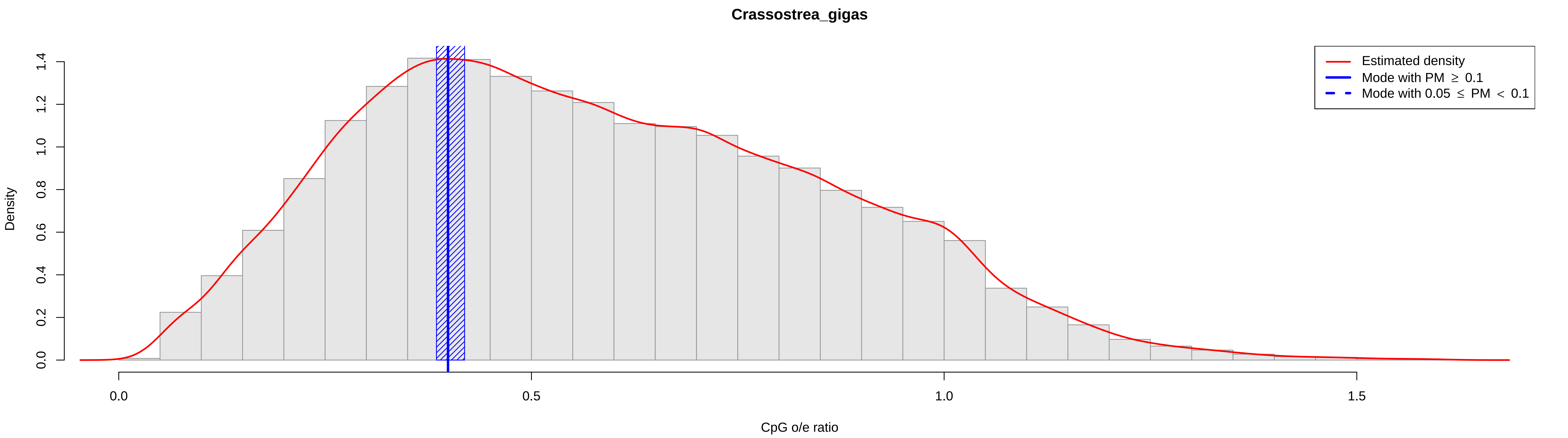
Crassostrea_gigas
Estimated density
Mode with PM ≥ 0.1
Mode with 0.05 ≤ PM < 0.1
Density
0.0
0.2
0.4
0.6
0.8
1.0
1.2
1.4
0.0
0.5
1.0
1.5
CpG o/e ratio

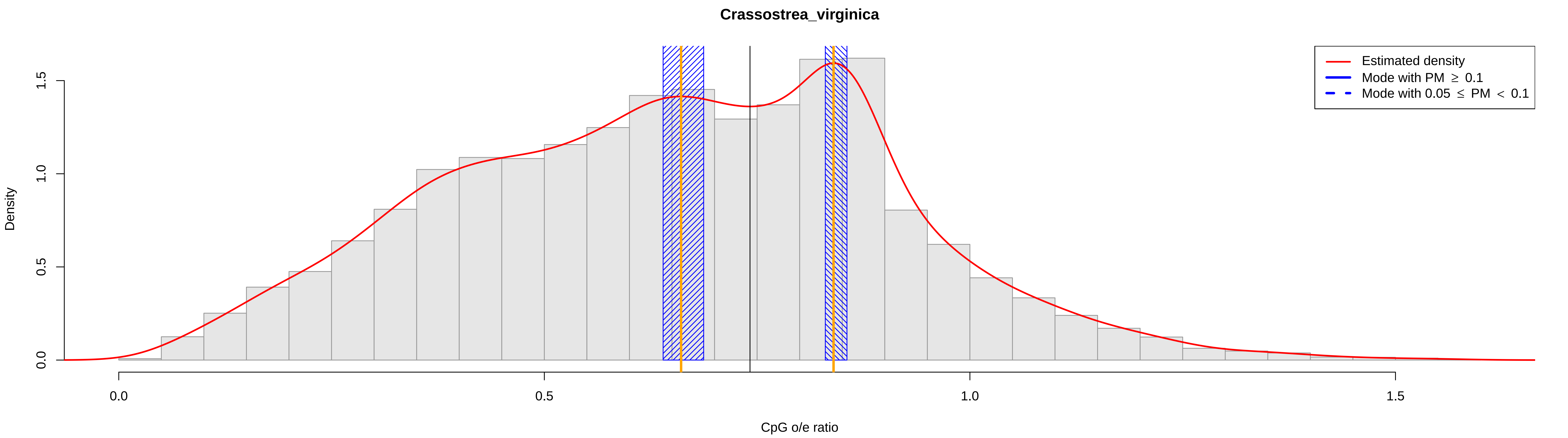

Crassostrea_virginica
Estimated density
Mode with PM ≥ 0.1
Mode with 0.05 ≤ PM < 0.1
Density
0.0
0.5
1.0
1.5
0.0
0.5
1.0
1.5
CpG o/e ratio

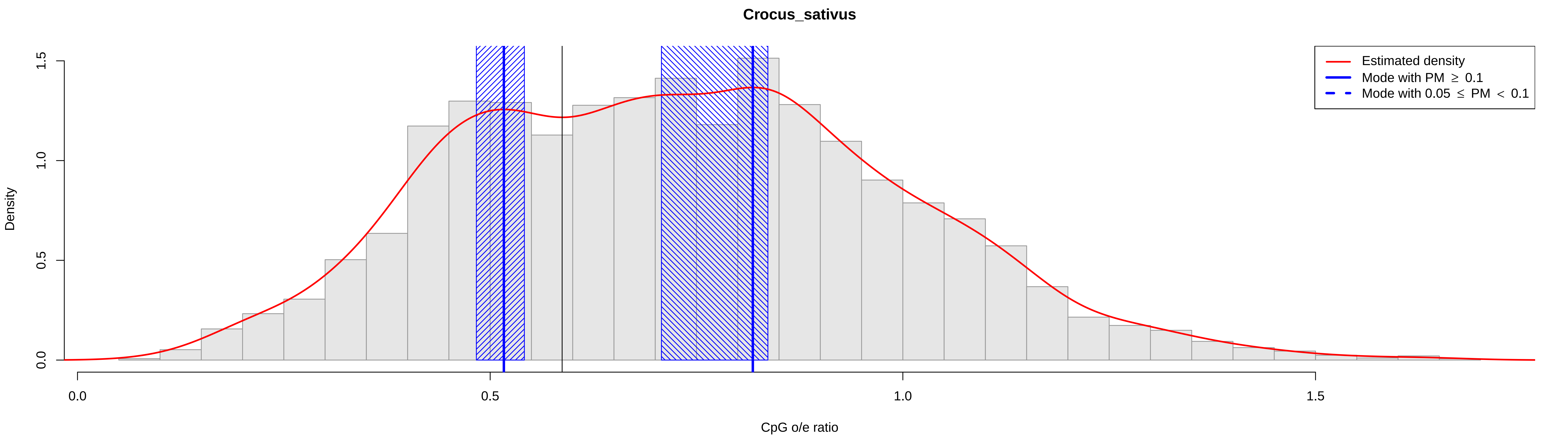

Crocus_sativus
Estimated density
Mode with PM ≥ 0.1
Mode with 0.05 ≤ PM < 0.1
Density
0.0
0.5
1.0
1.5
0.0
0.5
1.0
1.5
CpG o/e ratio

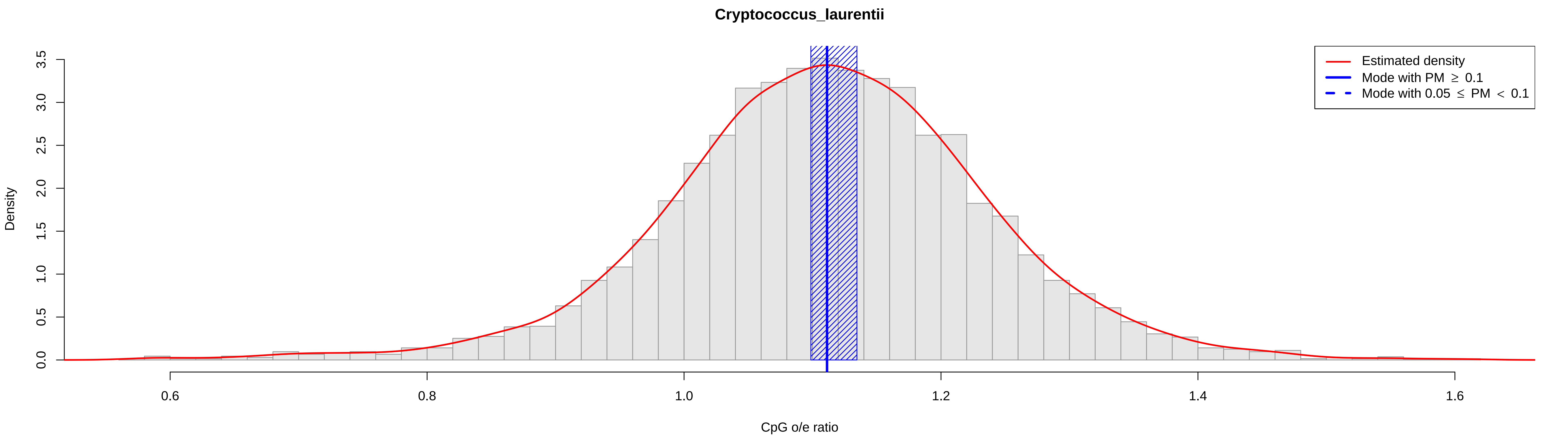
Cryptococcus_laurentii
Estimated density
Mode with PM ≥ 0.1
Mode with 0.05 ≤ PM < 0.1
Density
0.0
0.5
1.0
1.5
2.0
2.5
3.0
3.5
0.6
0.8
1.0
1.2
1.4
1.6
CpG o/e ratio

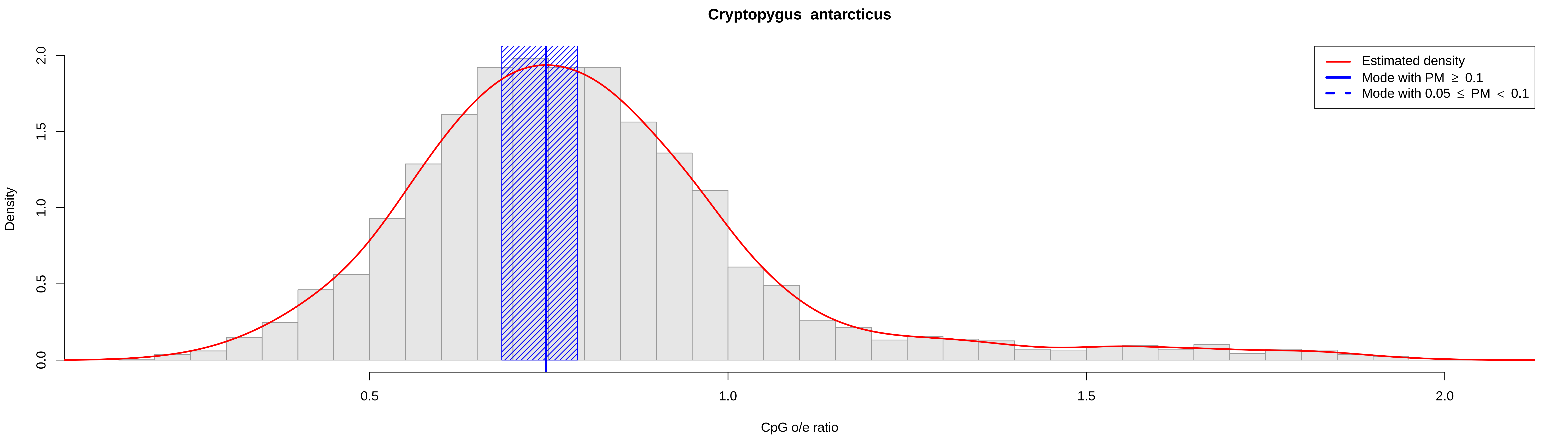

Cryptopygus_antarcticus
Estimated density
Mode with PM ≥ 0.1
Mode with 0.05 ≤ PM < 0.1
Density
0.0
0.5
1.0
1.5
2.0
0.5
1.0
1.5
2.0
CpG o/e ratio

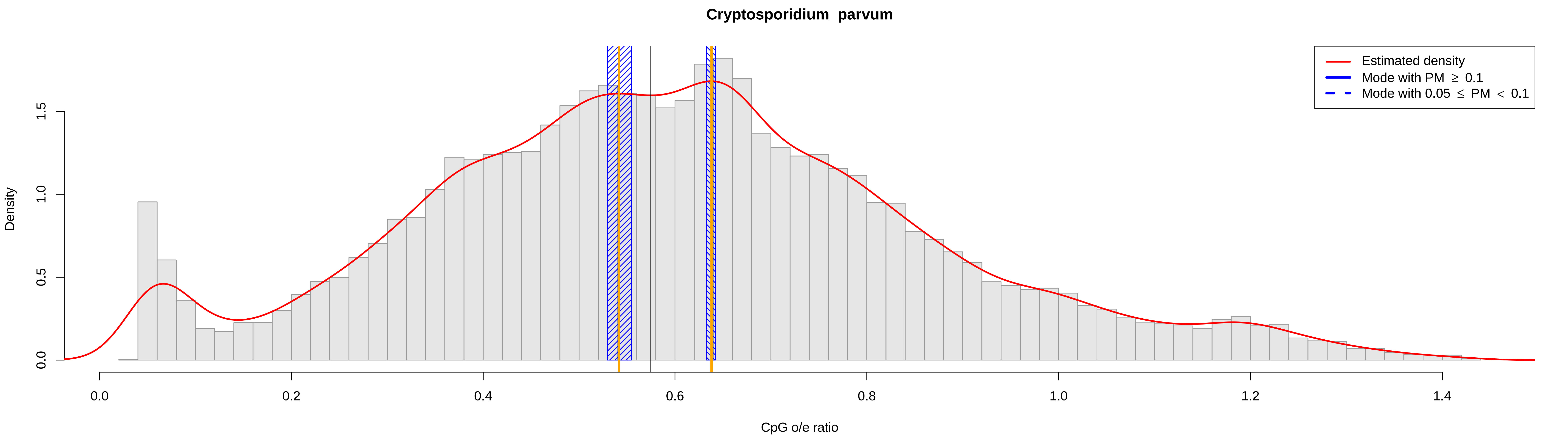

Cryptosporidium_parvum
Estimated density
Mode with PM ≥ 0.1
Mode with 0.05 ≤ PM < 0.1
Density
0.0
0.5
1.0
1.5
0.0
0.2
0.4
0.6
0.8
1.0
1.2
1.4
CpG o/e ratio

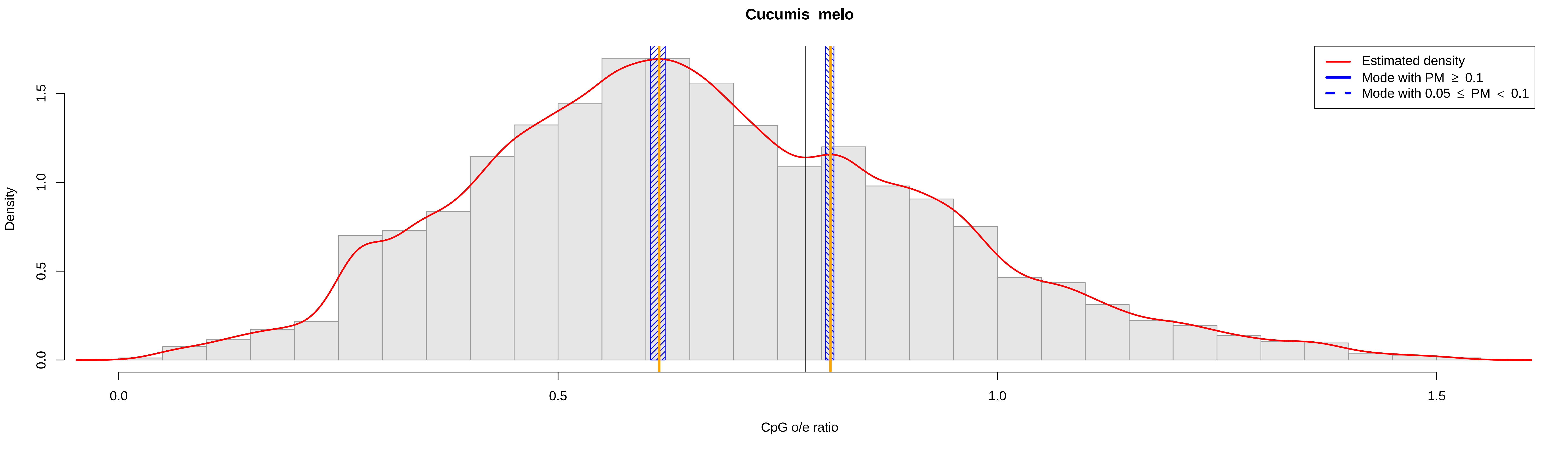
Cucumis_melo
Estimated density
Mode with PM ≥ 0.1
Mode with 0.05 ≤ PM < 0.1
Density
0.0
0.5
1.0
1.5
CpG o/e ratio
0.0
0.5
1.0
1.5

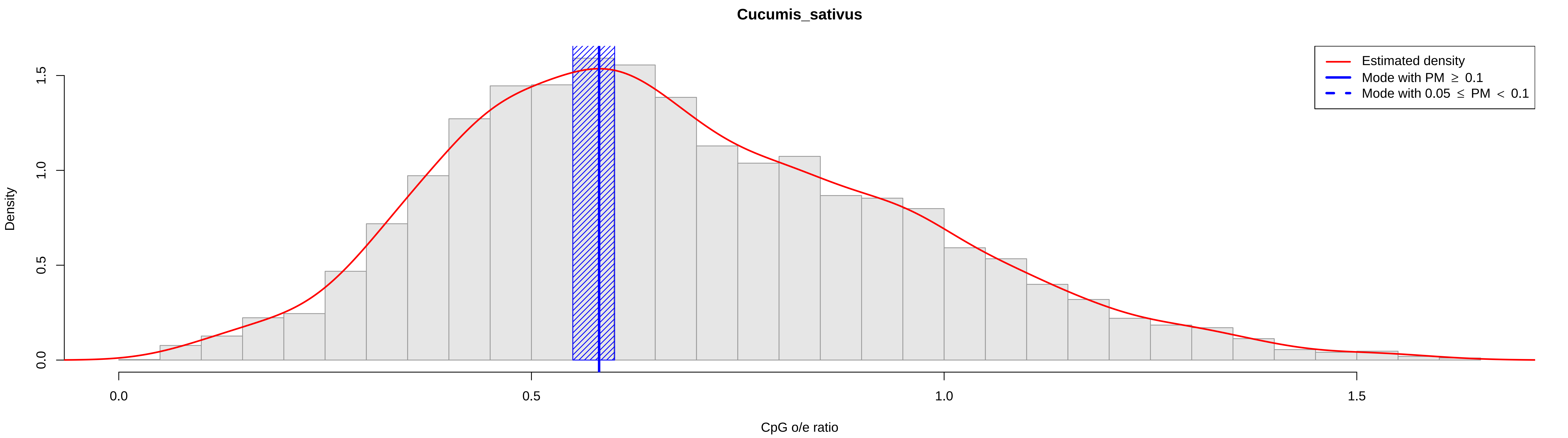
Cucumis_sativus
Estimated density
Mode with PM ≥ 0.1
Mode with 0.05 ≤ PM < 0.1
Density
0.0
0.5
1.0
1.5
CpG o/e ratio

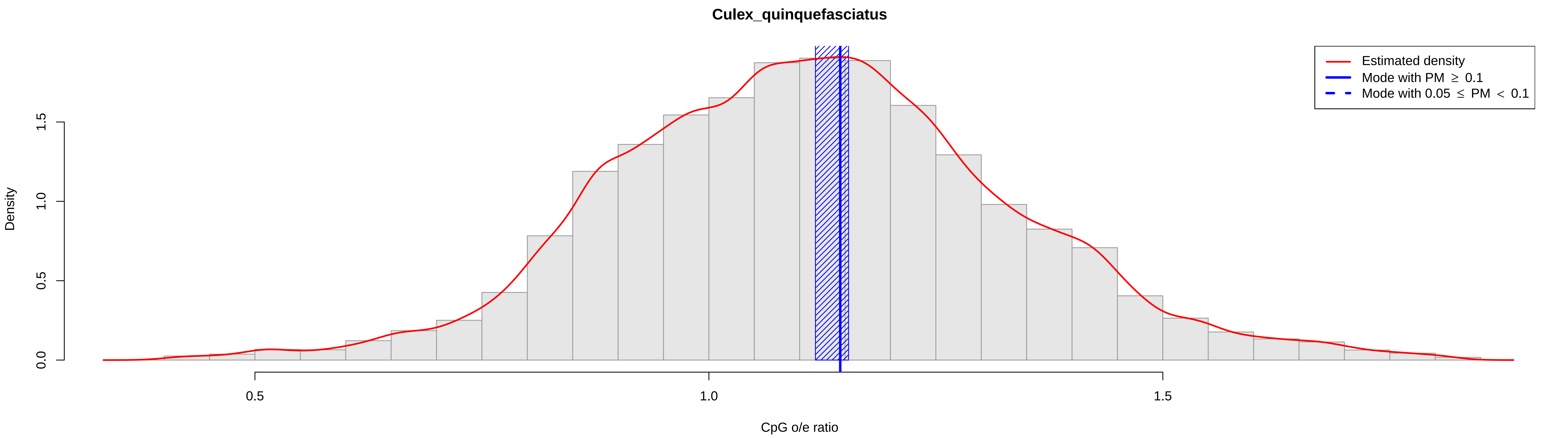

Culex_quinquefasciatus
Estimated density
Mode with PM ≥ 0.1
Mode with 0.05 ≤ PM < 0.1
Density
0.0
0.5
1.0
1.5
0.5
1.0
1.5
CpG o/e ratio

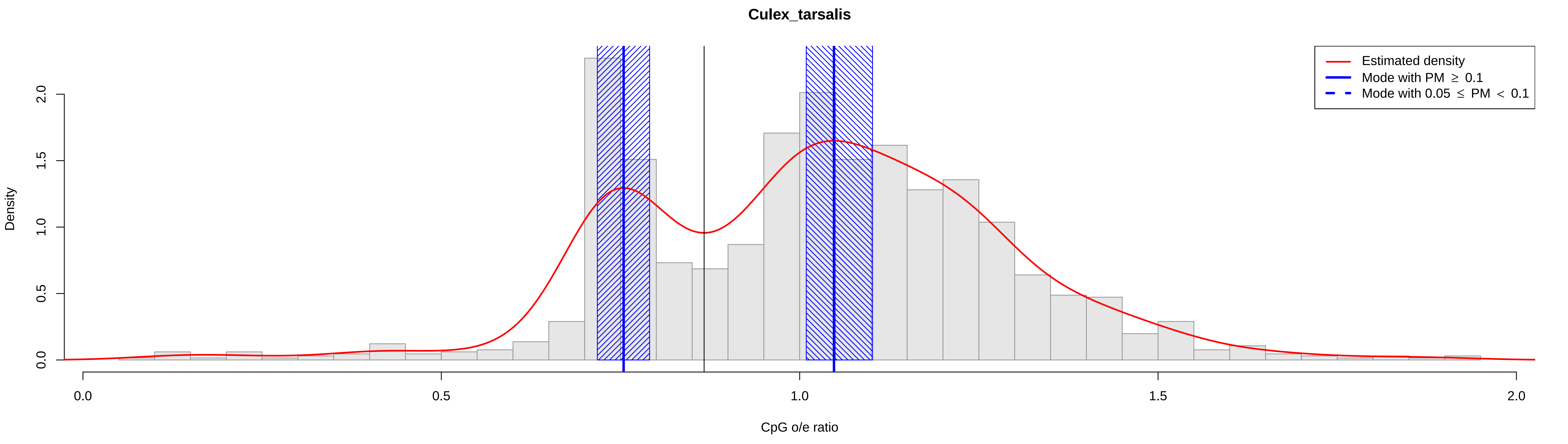

Culex_tarsalis
Estimated density
Mode with PM ≥ 0.1
Mode with 0.05 ≤ PM < 0.1
Density
0.0
0.5
1.0
1.5
2.0
0.0
0.5
1.0
1.5
2.0
CpG o/e ratio

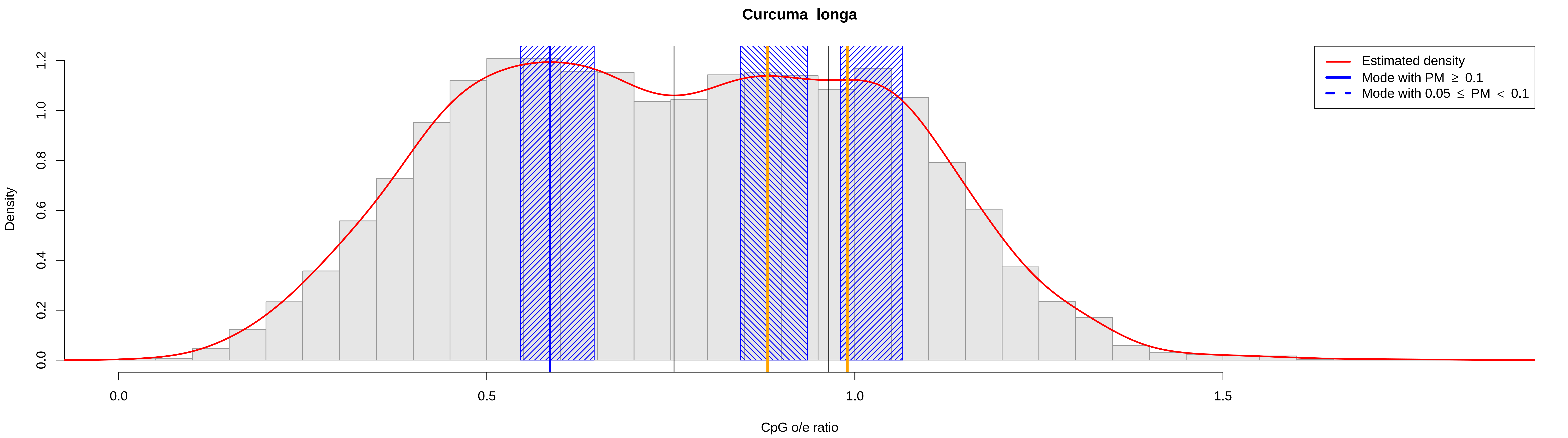
Curcuma_longa
Estimated density
Mode with PM ≥ 0.1
Mode with 0.05 ≤ PM < 0.1
Density
0.0
0.2
0.4
0.6
0.8
1.0
1.2
0.0
0.5
1.0
1.5
CpG o/e ratio

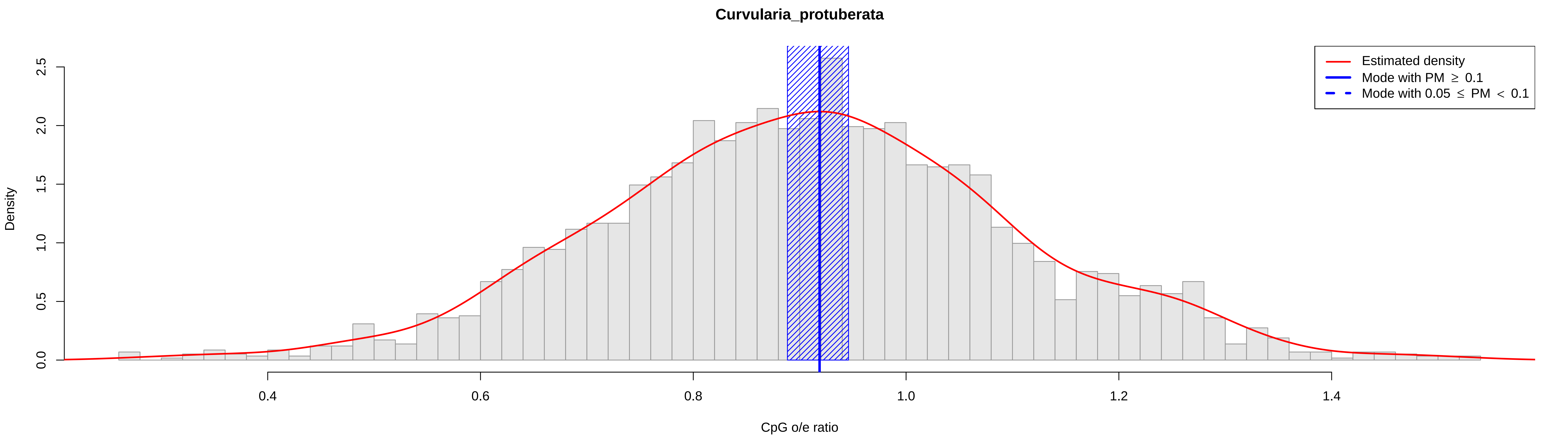

Curvularia_protuberata
Estimated density
Mode with PM ≥ 0.1
Mode with 0.05 ≤ PM < 0.1
Density
0.0
0.5
1.0
1.5
2.0
2.5
0.4
0.6
0.8
1.0
1.2
1.4
CpG o/e ratio

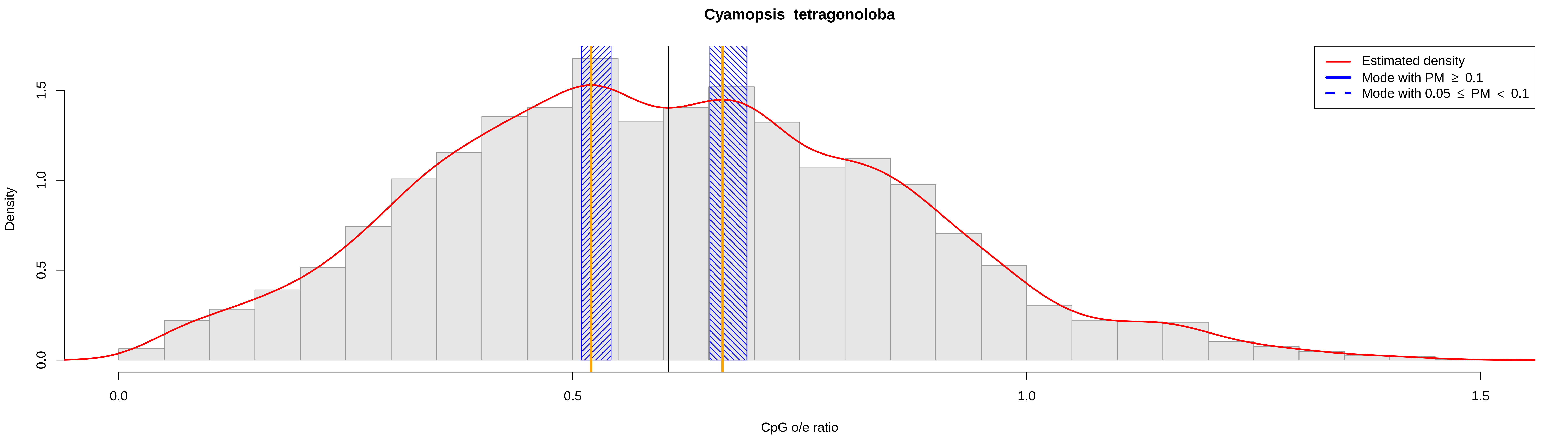

Cyamopsis_tetragonoloba
Estimated density
Mode with PM ≥ 0.1
Mode with 0.05 ≤ PM < 0.1
Density
0.0
0.5
1.0
1.5
0.0
0.5
1.0
1.5
CpG o/e ratio

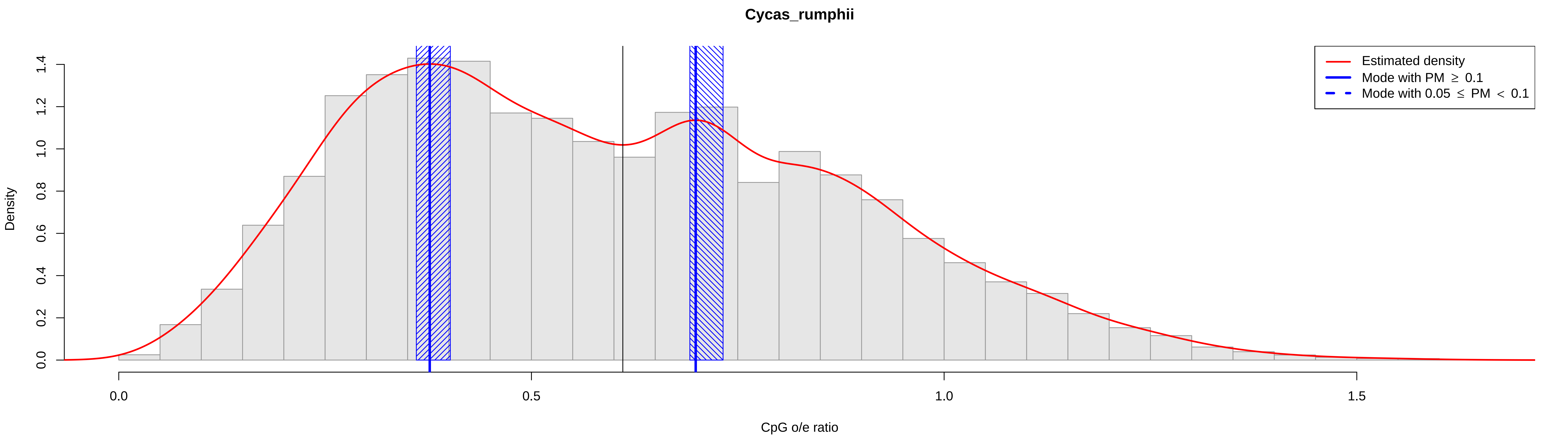

Cycas_rumphii
Estimated density
Mode with PM ≥ 0.1
Mode with 0.05 ≤ PM < 0.1
Density
0.0
0.2
0.4
0.6
0.8
1.0
1.2
1.4
0.0
0.5
1.0
1.5
CpG o/e ratio

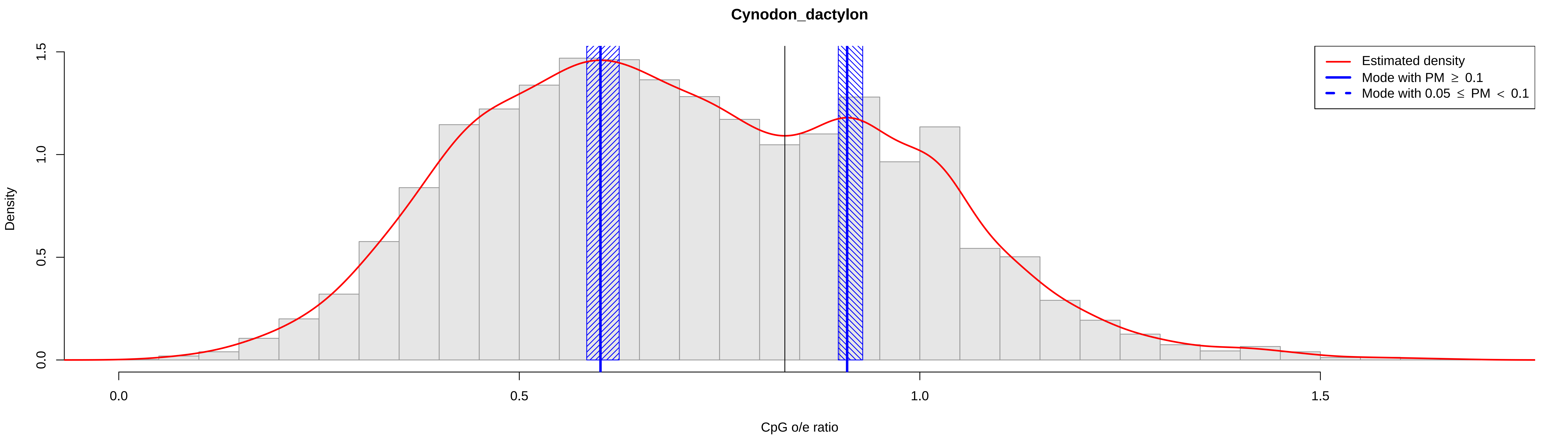
Cynodon_dactylon
Estimated density
Mode with PM ≥ 0.1
Mode with 0.05 ≤ PM < 0.1
Density
0.0
0.5
1.0
1.5
CpG o/e ratio
0.0
0.5
1.0
1.5

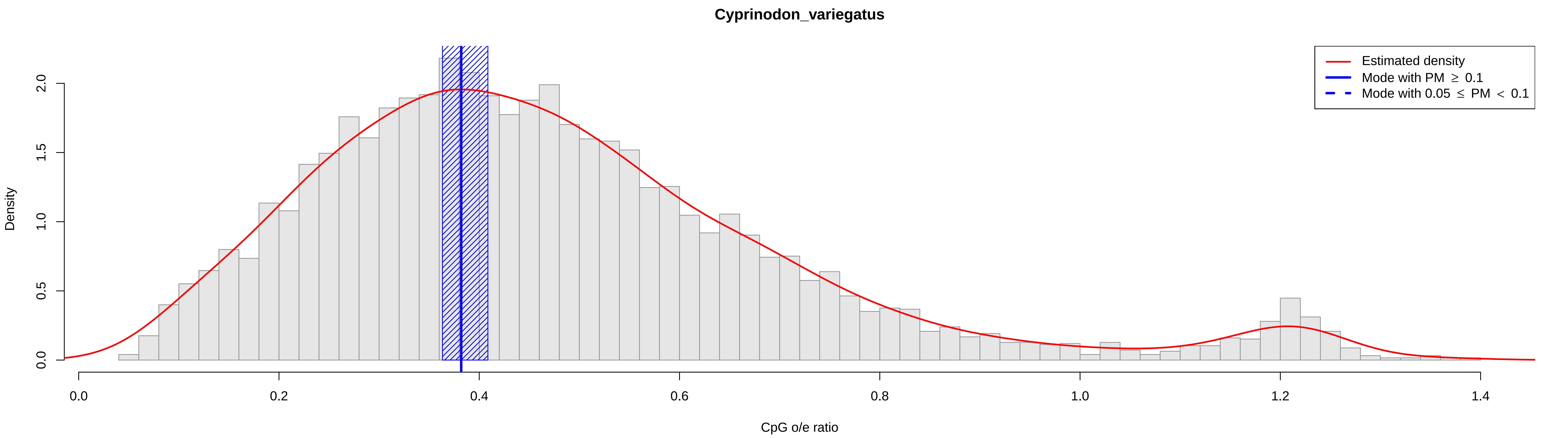
Cyprinodon_variegatus
Density
CpG o/e ratio
0.0
0.5
1.0
1.5
2.0
0.0
0.2
0.4
0.6
0.8
1.0
1.2
1.4
Estimated density
Mode with PM ≥ 0.1
Mode with 0.05 ≤ PM < 0.1

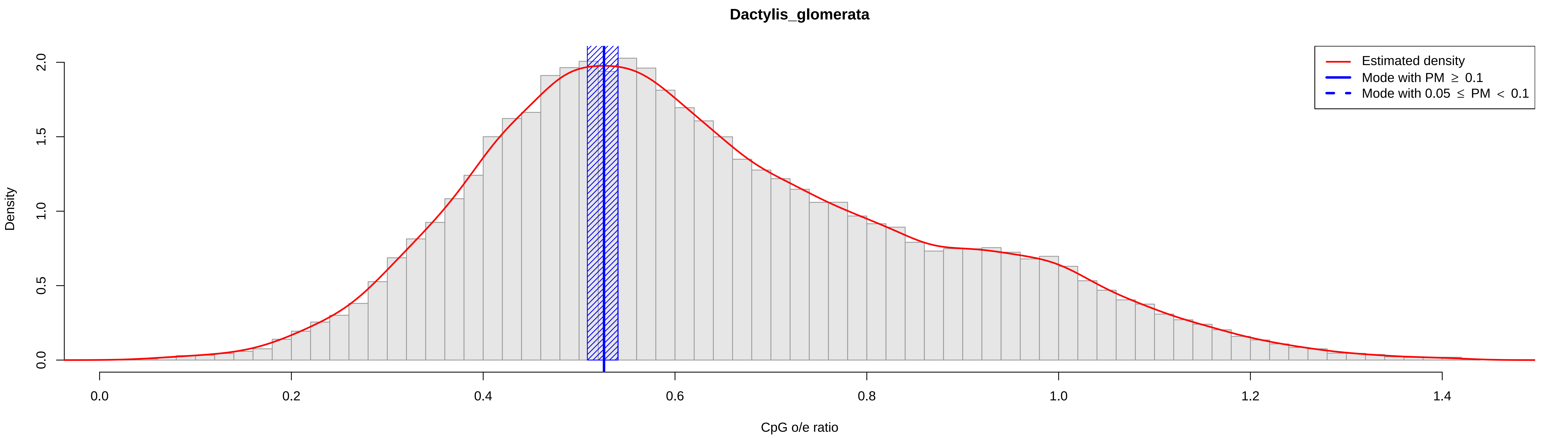

Dactylis_glomerata
Estimated density
Mode with PM ≥ 0.1
Mode with 0.05 ≤ PM < 0.1
Density
0.0
0.5
1.0
1.5
2.0
0.0
0.2
0.4
0.6
0.8
1.0
1.2
1.4
CpG o/e ratio

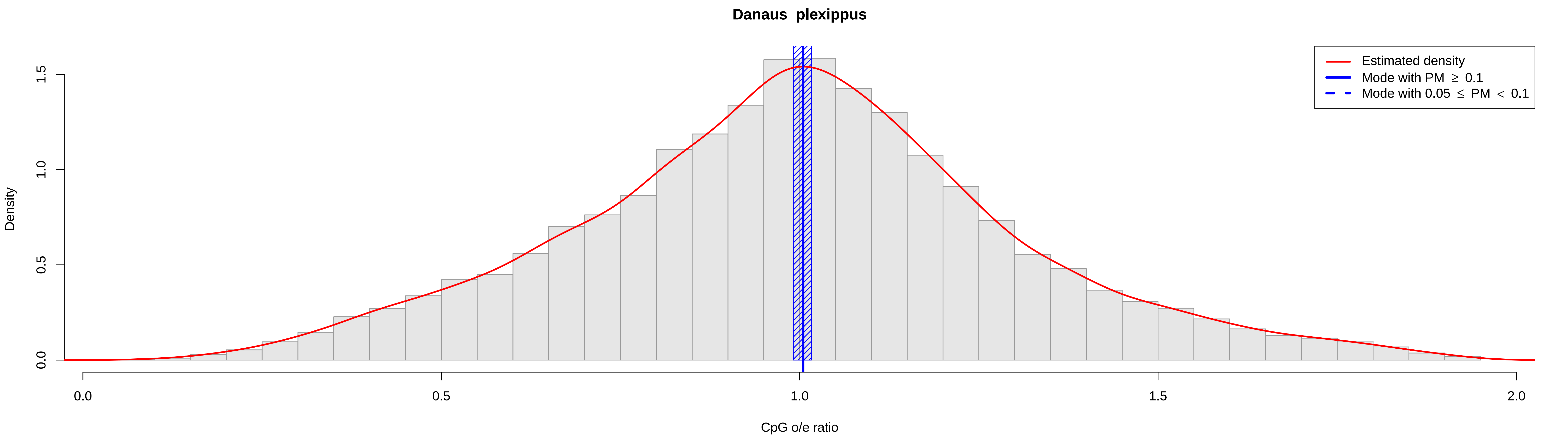
Danaus_plexippus
Estimated density
Mode with PM ≥ 0.1
Mode with 0.05 ≤ PM < 0.1
Density
0.0
0.5
1.0
1.5
0.0
0.5
1.0
1.5
2.0
CpG o/e ratio

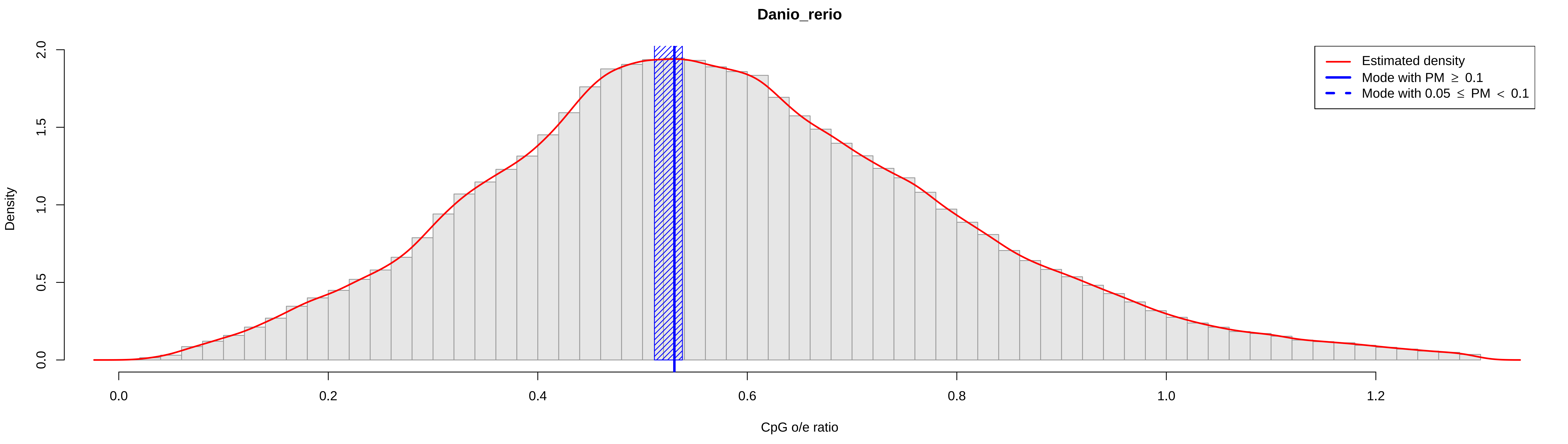
Danio_rerio
Estimated density
Mode with PM ≥ 0.1
Mode with 0.05 ≤ PM < 0.1
Density
0.0
0.5
1.0
1.5
2.0
0.0
0.2
0.4
0.6
0.8
1.0
1.2
CpG o/e ratio

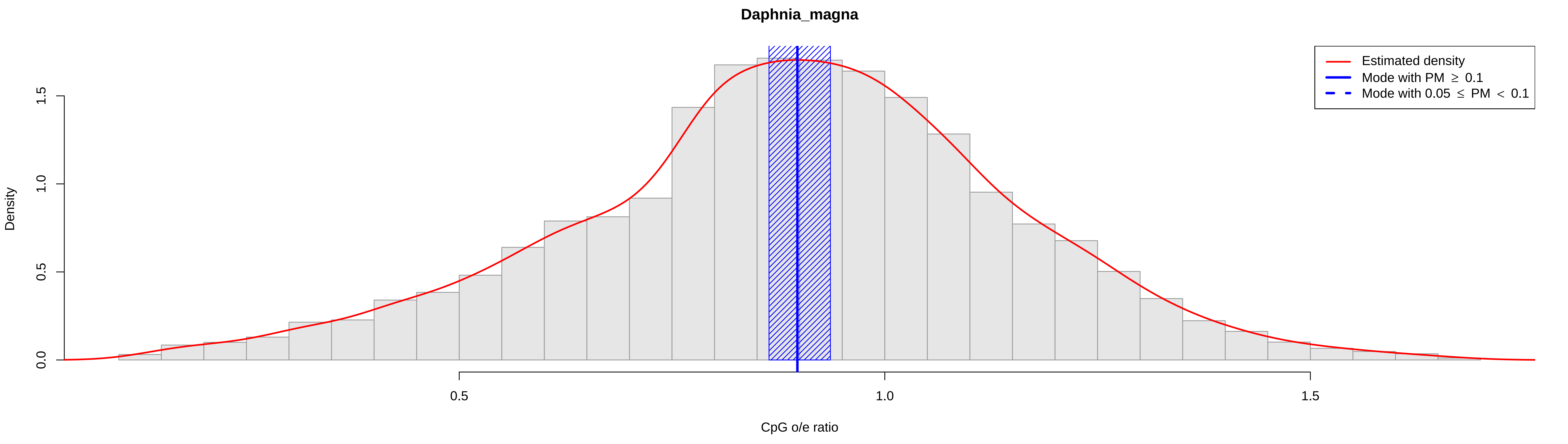
Daphnia_magna
Estimated density
Mode with PM ≥ 0.1
Mode with 0.05 ≤ PM < 0.1
Density
0.0
0.5
1.0
1.5
0.5
1.0
1.5
CpG o/e ratio

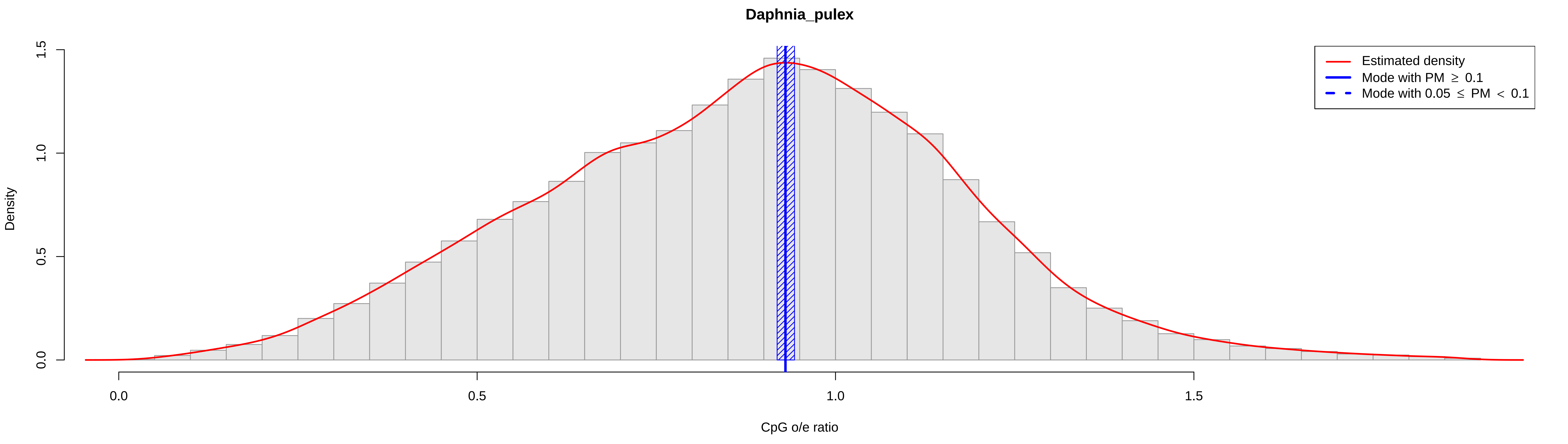
Daphnia_pulex
Estimated density
Mode with PM ≥ 0.1
Mode with 0.05 ≤ PM < 0.1
Density
0.0
0.5
1.0
1.5
CpG o/e ratio

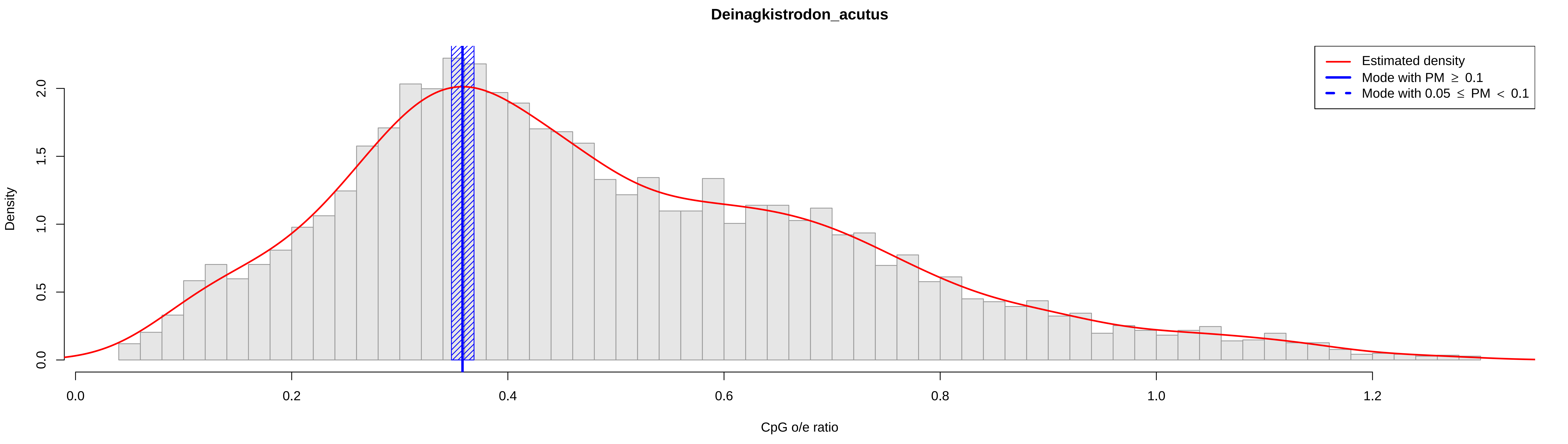
Deinagkistrodon_acutus
Estimated density
Mode with PM ≥ 0.1
Mode with 0.05 ≤ PM < 0.1
Density
0.0
0.5
1.0
1.5
2.0
0.0
0.2
0.4
0.6
0.8
1.0
1.2
CpG o/e ratio

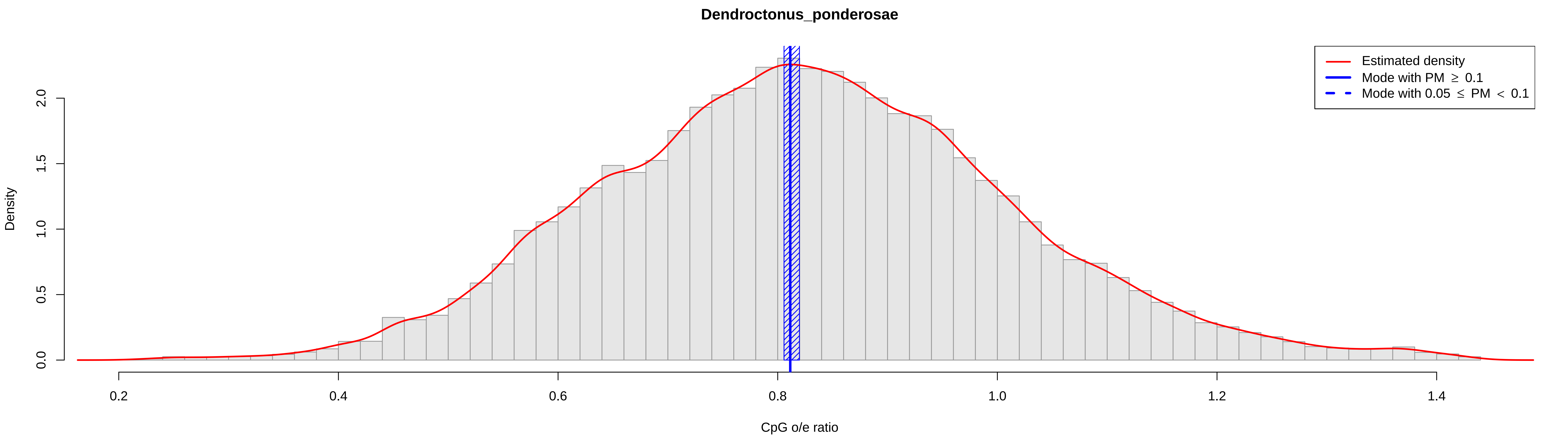
Dendroctonus_ponderosae
Estimated density
Mode with PM ≥ 0.1
Mode with 0.05 ≤ PM < 0.1
Density
0.0
0.5
1.0
1.5
2.0
0.2
0.4
0.6
0.8
1.0
1.2
1.4
CpG o/e ratio

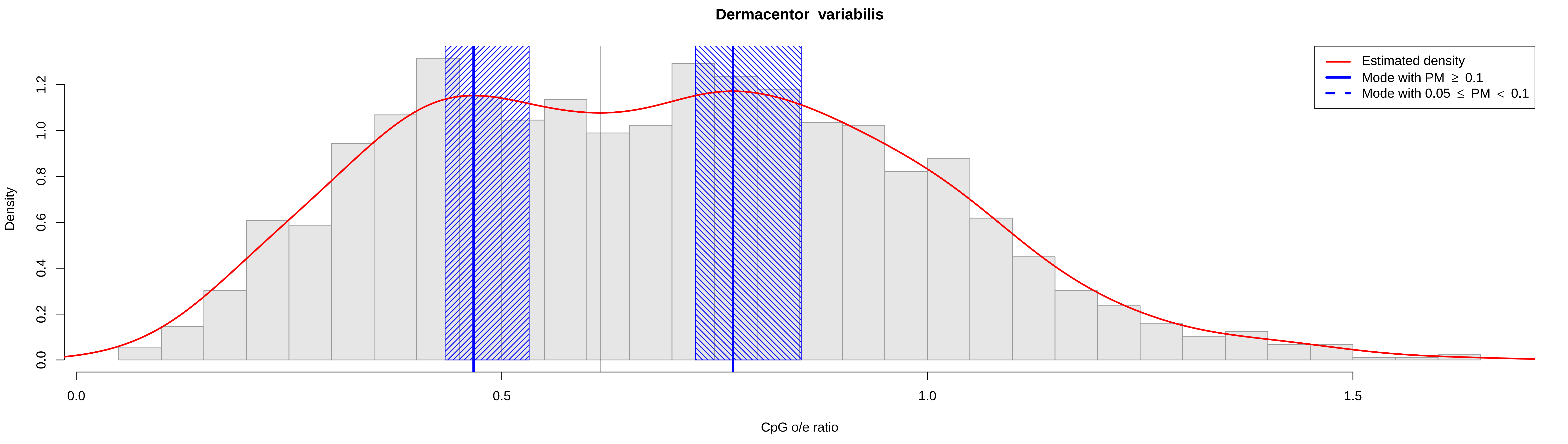
Dermacentor_variabilis
Estimated density
Mode with PM ≥ 0.1
Mode with 0.05 ≤ PM < 0.1
Density
0.0
0.2
0.4
0.6
0.8
1.0
1.2
0.0
0.5
1.0
1.5
CpG o/e ratio

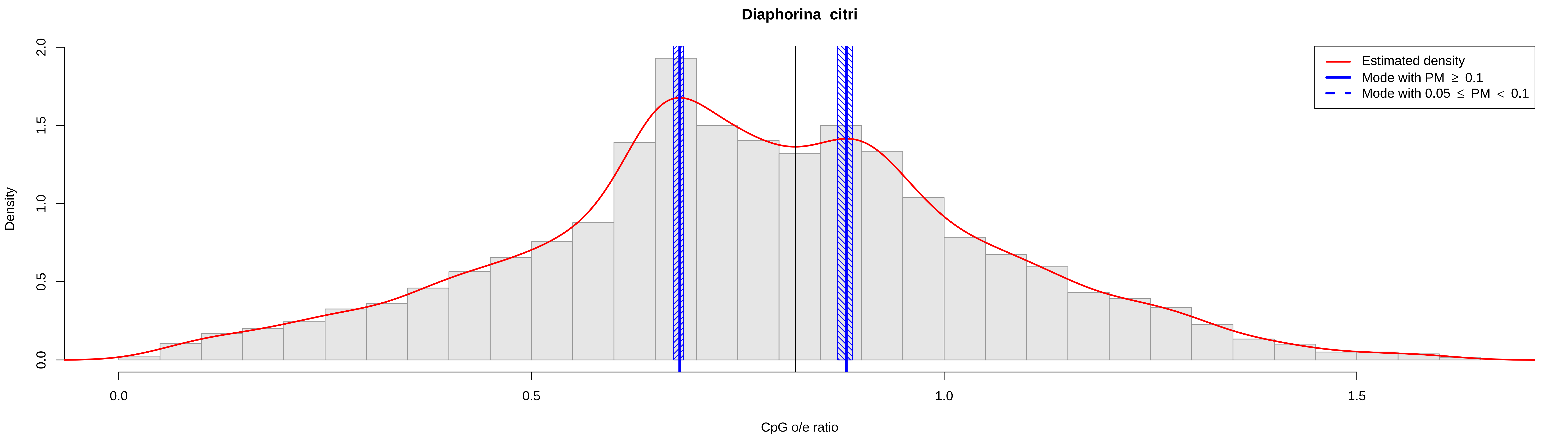

Diaphorina_citri
Estimated density
Mode with PM ≥ 0.1
Mode with 0.05 ≤ PM < 0.1
Density
0.0
0.5
1.0
1.5
2.0
0.0
0.5
1.0
1.5
CpG o/e ratio

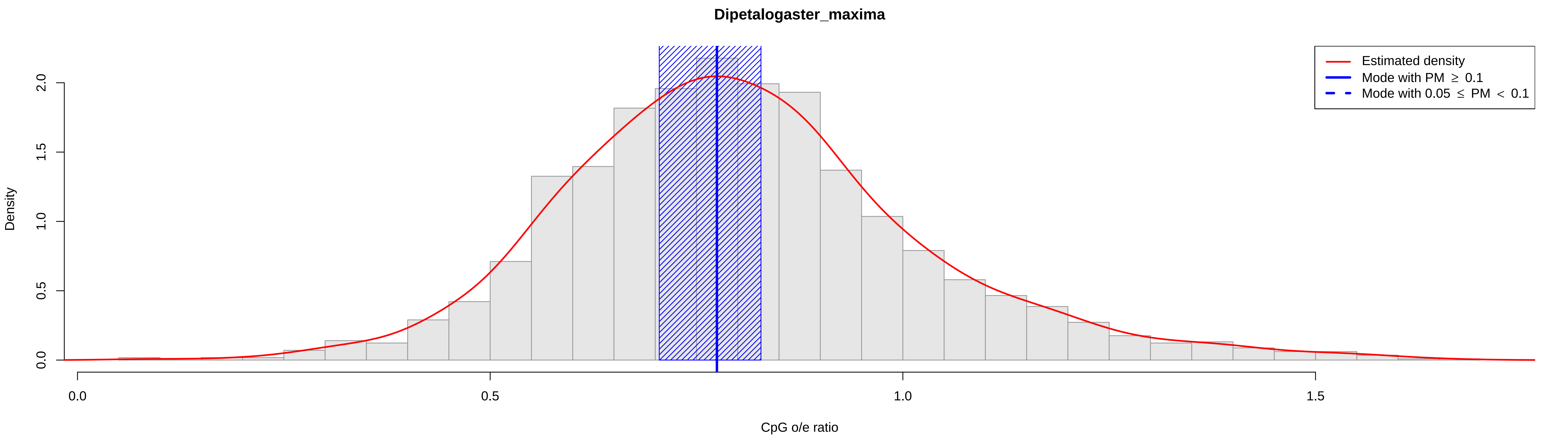
Dipetalogaster_maxima
Estimated density
Mode with PM ≥ 0.1
Mode with 0.05 ≤ PM < 0.1
Density
0.0
0.5
1.0
1.5
2.0
0.0
0.5
1.0
1.5
CpG o/e ratio

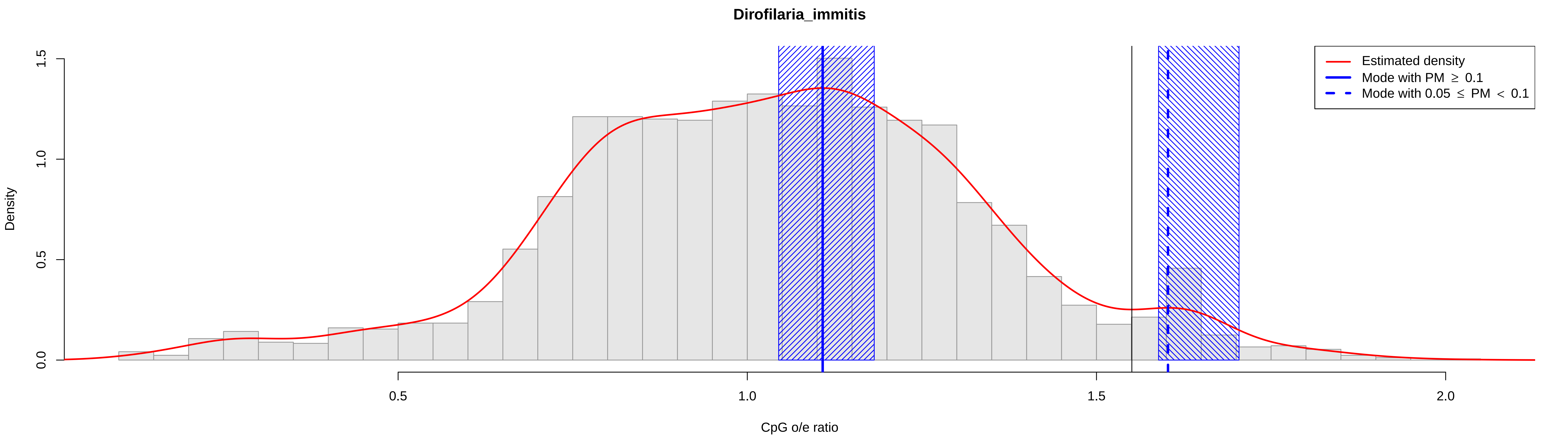
Dirofilaria_immitis
Estimated density
Mode with PM ≥ 0.1
Mode with 0.05 ≤ PM < 0.1
Density
0.0
0.5
1.0
1.5
0.5
1.0
1.5
2.0
CpG o/e ratio

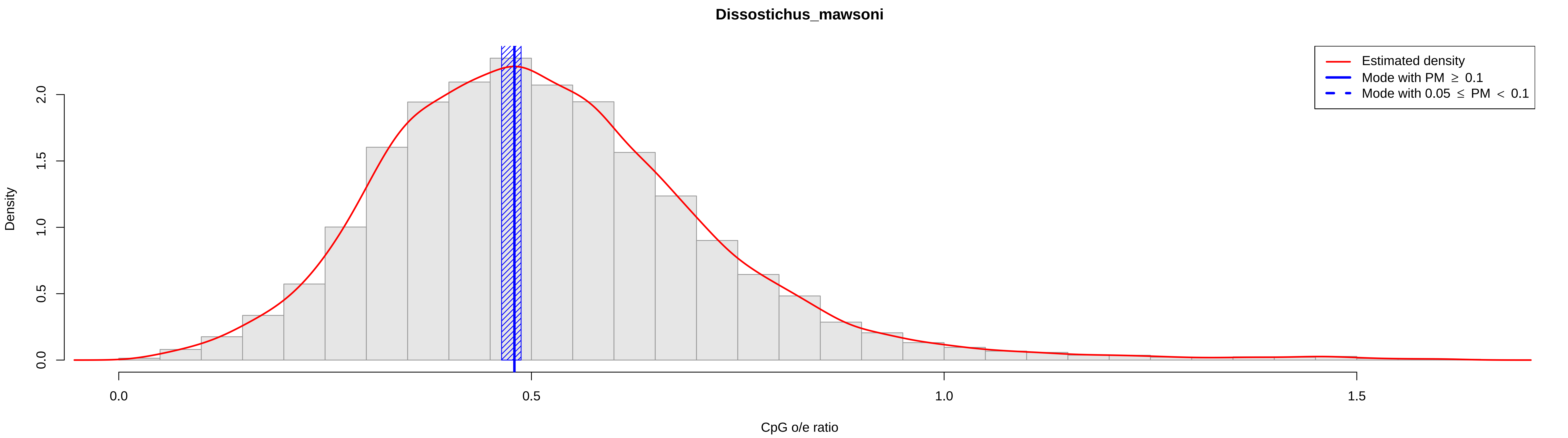

Dissostichus_mawsoni
Estimated density
Mode with PM ≥ 0.1
Mode with 0.05 ≤ PM < 0.1
Density
0.0
0.5
1.0
1.5
2.0
0.0
0.5
1.0
1.5
CpG o/e ratio

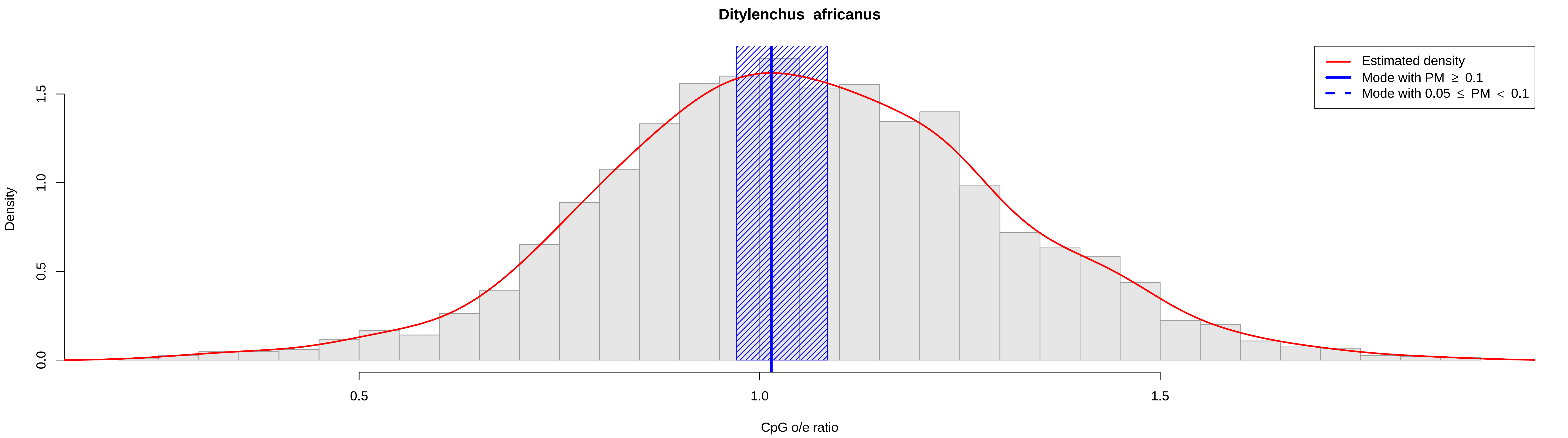

Ditylenchus_africanus
Estimated density
Mode with PM ≥ 0.1
Mode with 0.05 ≤ PM < 0.1
Density
0.0
0.5
1.0
1.5
0.5
1.0
1.5
CpG o/e ratio

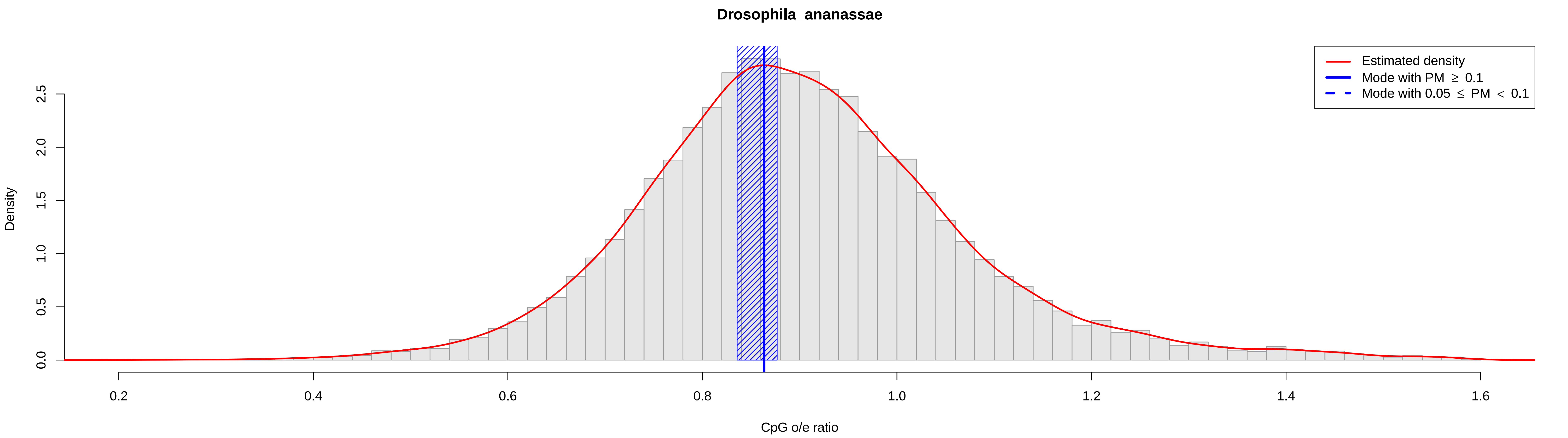

Drosophila_ananassae
Estimated density
Mode with PM ≥ 0.1
Mode with 0.05 ≤ PM < 0.1
Density
0.0
0.5
1.0
1.5
2.0
2.5
0.2
0.4
0.6
0.8
1.0
1.2
1.4
1.6
CpG o/e ratio

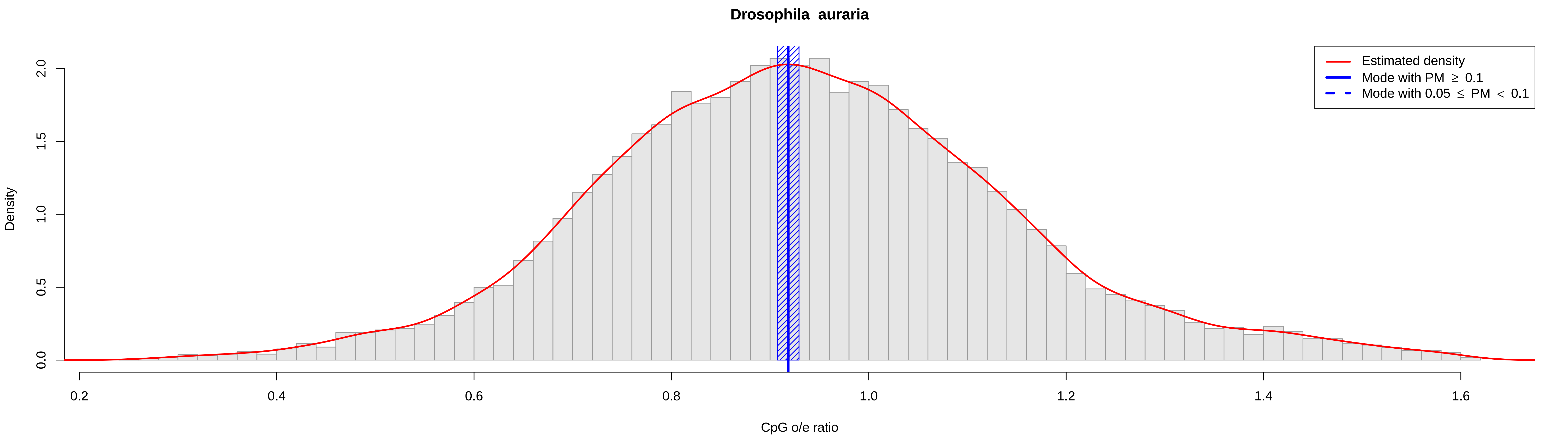

Drosophila_auraria
Estimated density
Mode with PM ≥ 0.1
Mode with 0.05 ≤ PM < 0.1
Density
0.0
0.5
1.0
1.5
2.0
0.2
0.4
0.6
0.8
1.0
1.2
1.4
1.6
CpG o/e ratio

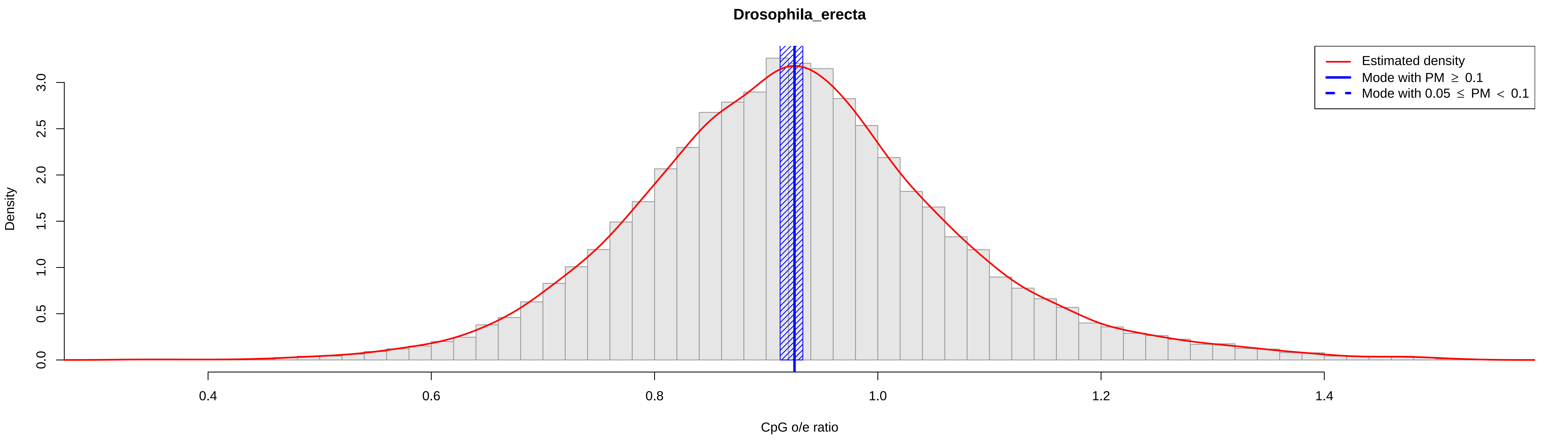

Drosophila_erecta
Estimated density
Mode with PM ≥ 0.1
Mode with 0.05 ≤ PM < 0.1
Density
0.0
0.5
1.0
1.5
2.0
2.5
3.0
0.4
0.6
0.8
1.0
1.2
1.4
CpG o/e ratio

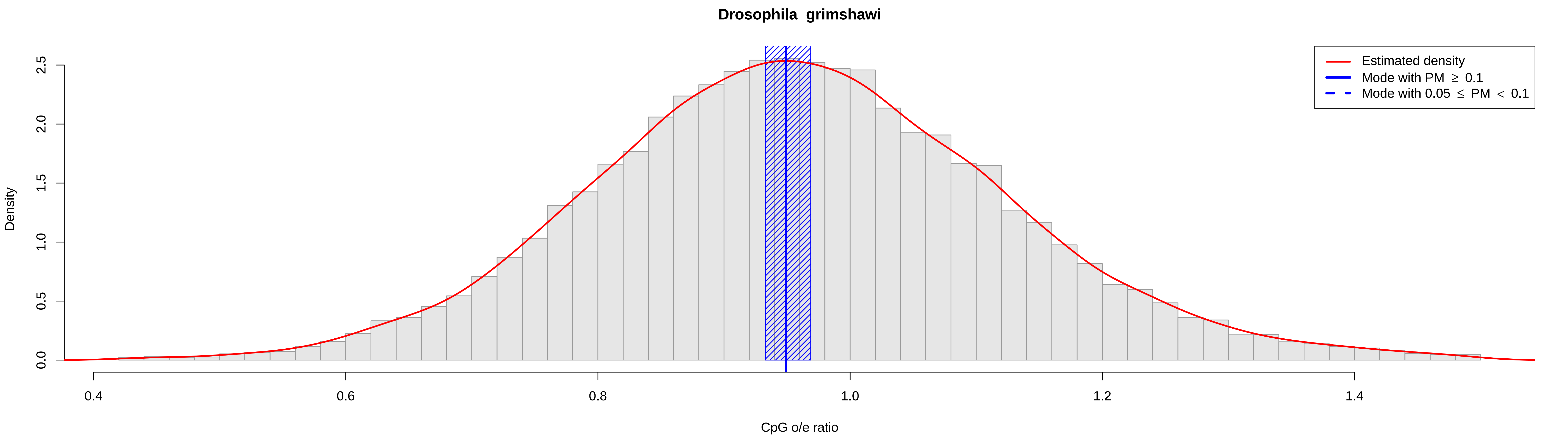

Drosophila_grimshawi
Estimated density
Mode with PM ≥ 0.1
Mode with 0.05 ≤ PM < 0.1
Density
0.0
0.5
1.0
1.5
2.0
2.5
0.4
0.6
0.8
1.0
1.2
1.4
CpG o/e ratio

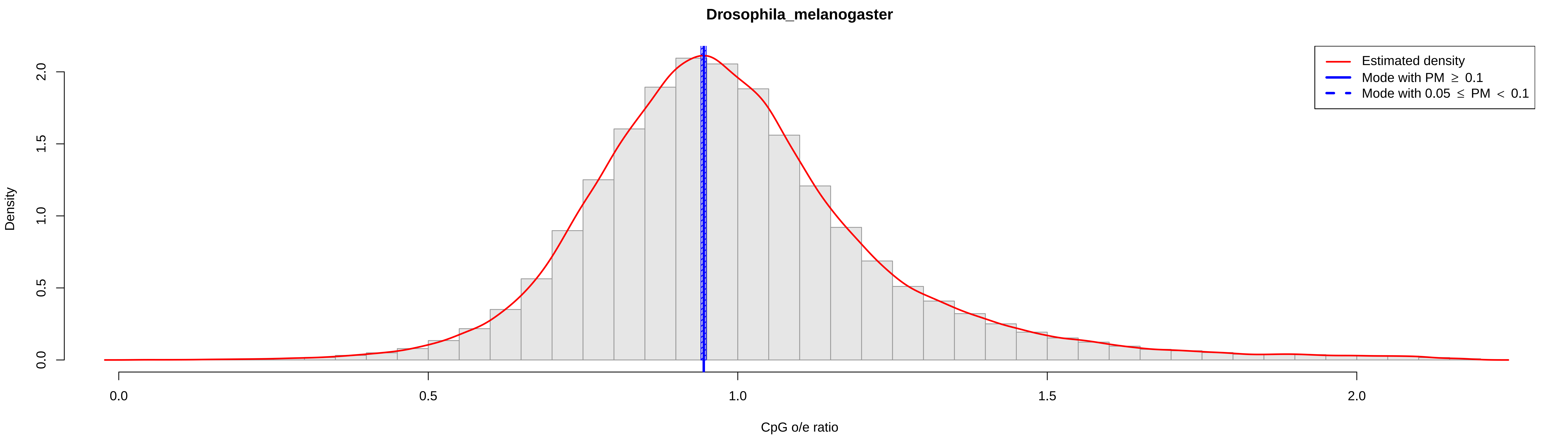
Drosophila_melanogaster
Estimated density
Mode with PM ≥ 0.1
Mode with 0.05 ≤ PM < 0.1
Density
0.0
0.5
1.0
1.5
2.0
0.0
0.5
1.0
1.5
2.0
CpG o/e ratio

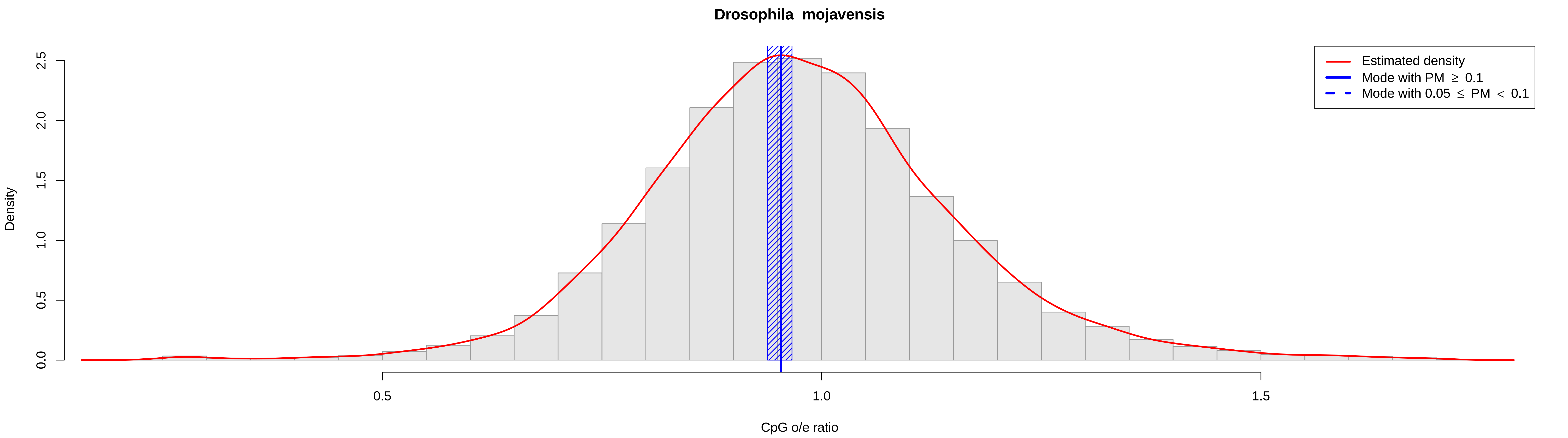
Drosophila_mojavensis
Density
0.0
0.5
1.0
1.5
2.0
2.5
0.5
1.0
1.5
CpG o/e ratio
Estimated density
Mode with PM ≥ 0.1
Mode with 0.05 ≤ PM < 0.1

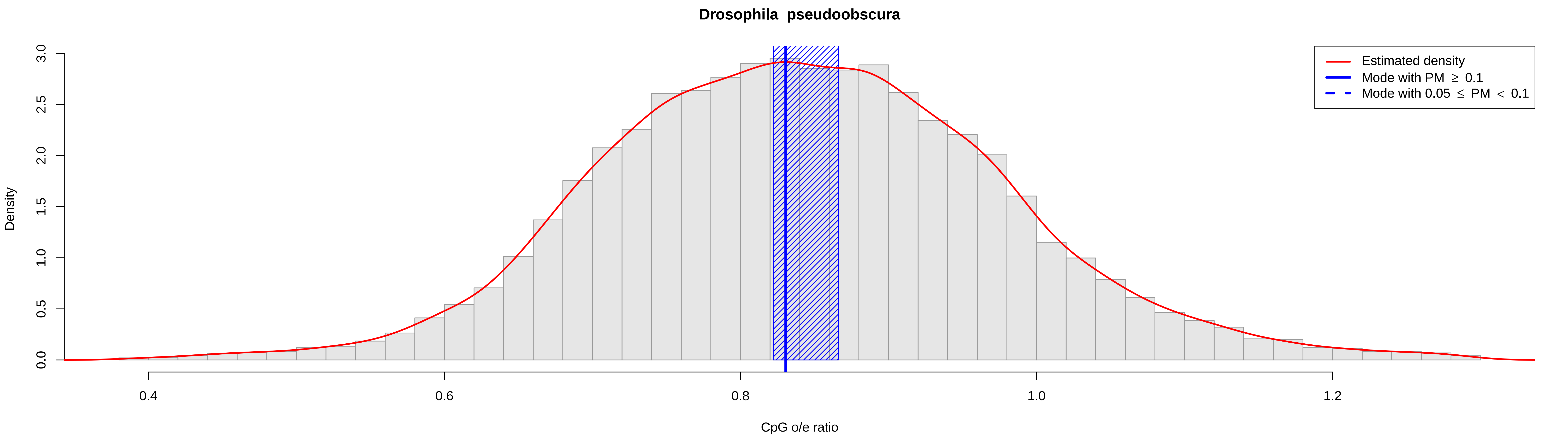

Drosophila_pseudoobscura
Estimated density
Mode with PM ≥ 0.1
Mode with 0.05 ≤ PM < 0.1
Density
0.0
0.5
1.0
1.5
2.0
2.5
3.0
0.4
0.6
0.8
1.0
1.2
CpG o/e ratio

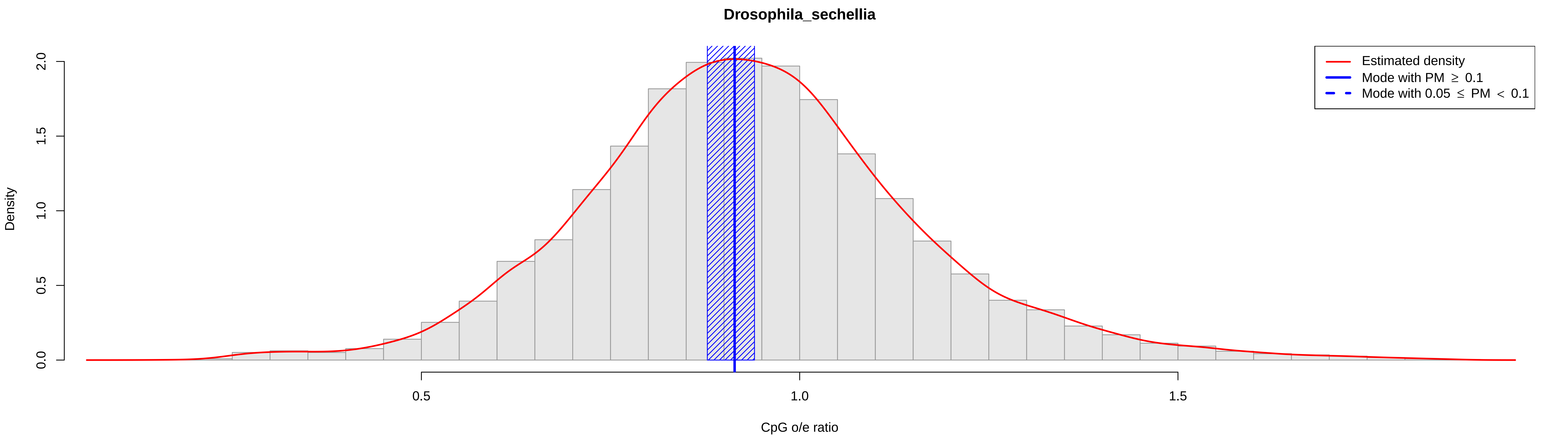

Drosophila_sechellia
Estimated density
Mode with PM ≥ 0.1
Mode with 0.05 ≤ PM < 0.1
Density
0.0
0.5
1.0
1.5
2.0
0.5
1.0
1.5
CpG o/e ratio

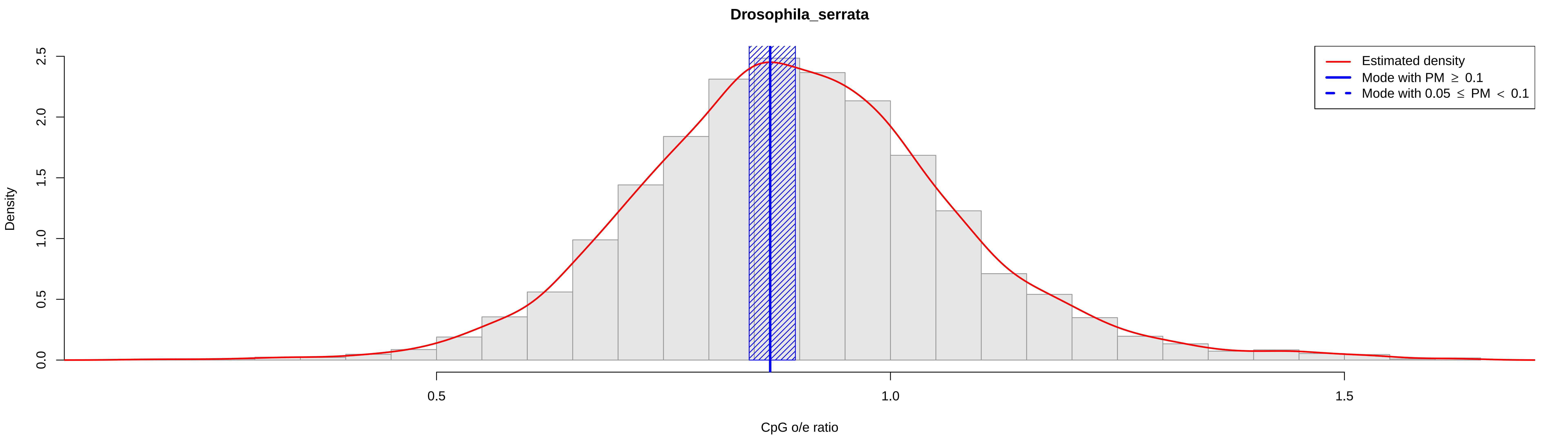

Drosophila_serrata
Density
CpG o/e ratio
0.0
0.5
1.0
1.5
2.0
2.5
0.5
1.0
1.5
Estimated density
Mode with PM ≥ 0.1
Mode with 0.05 ≤ PM < 0.1

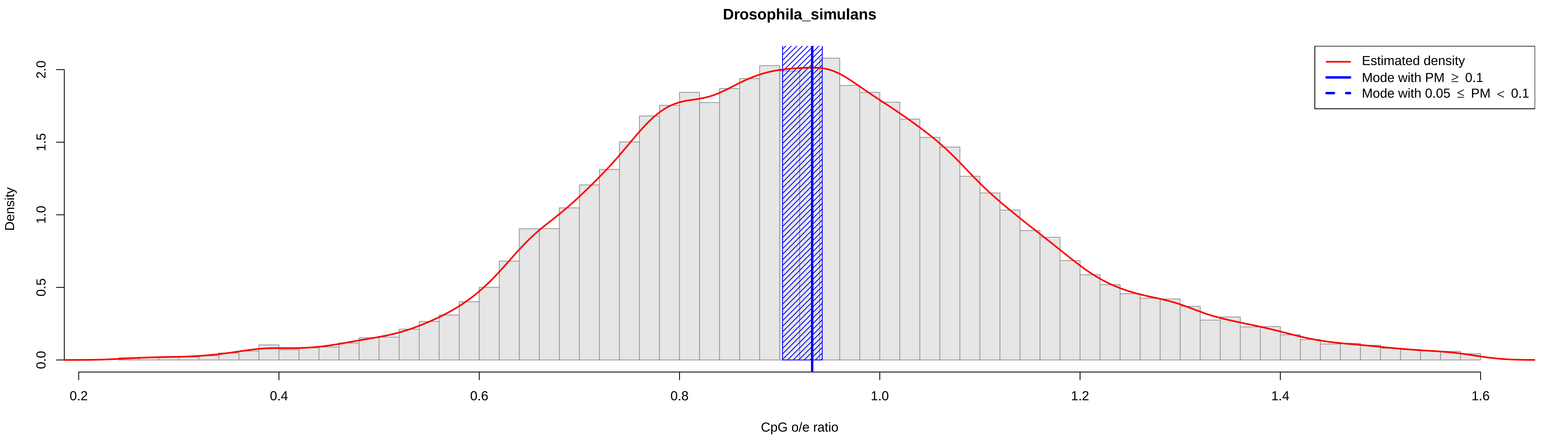

Drosophila_simulans
Estimated density
Mode with PM ≥ 0.1
Mode with 0.05 ≤ PM < 0.1
Density
0.0
0.5
1.0
1.5
2.0
0.2
0.4
0.6
0.8
1.0
1.2
1.4
1.6
CpG o/e ratio

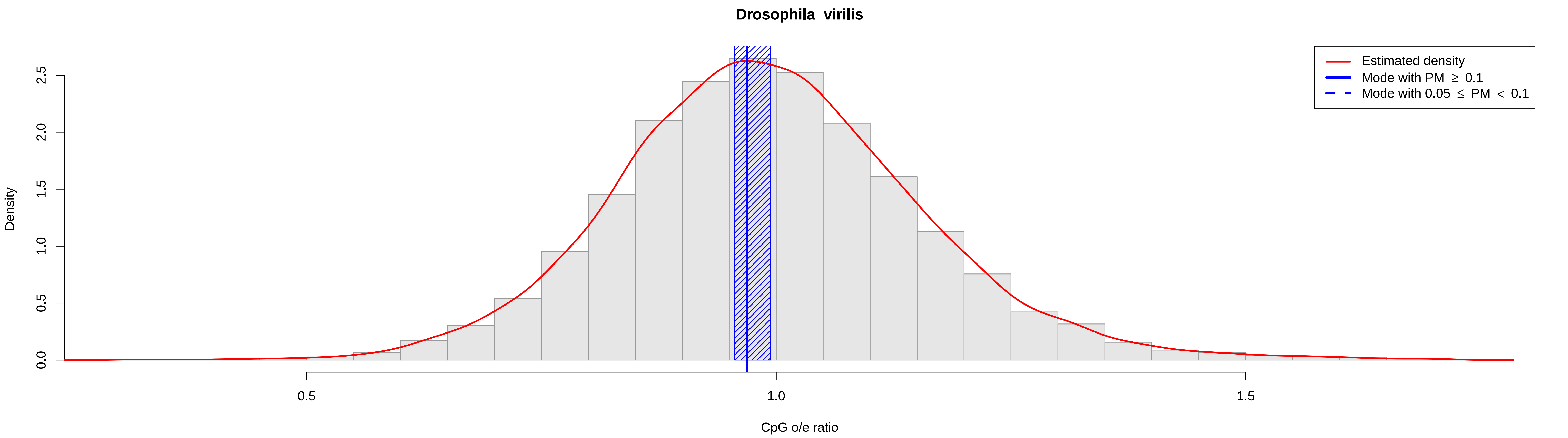

Drosophila_virilis
Estimated density
Mode with PM ≥ 0.1
Mode with 0.05 ≤ PM < 0.1
Density
0.0
0.5
1.0
1.5
2.0
2.5
0.5
1.0
1.5
CpG o/e ratio

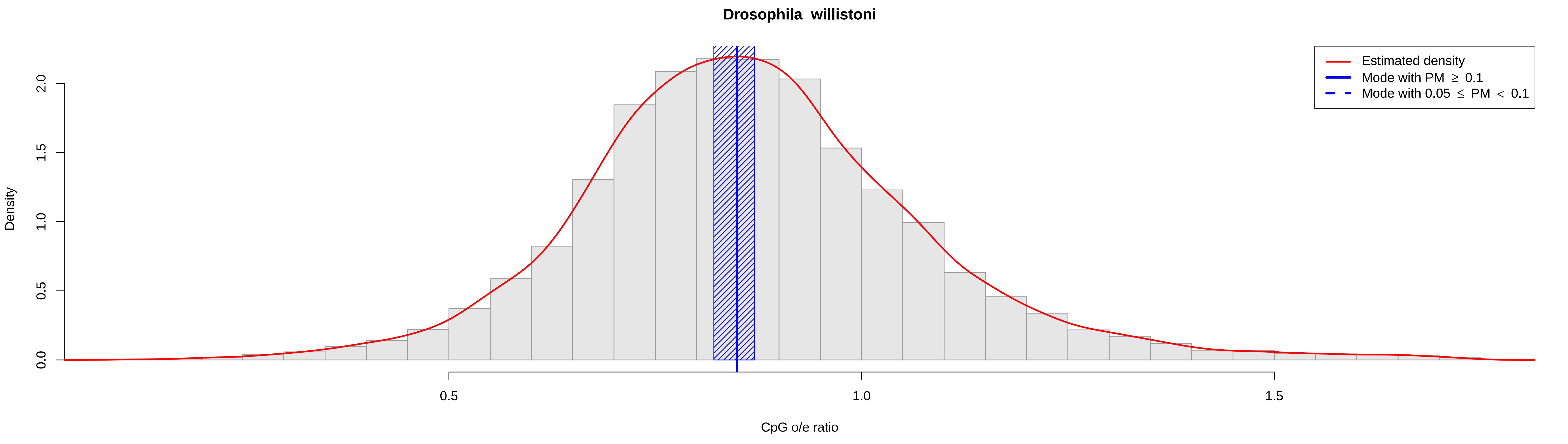

Drosophila_willistoni
Estimated density
Mode with PM ≥ 0.1
Mode with 0.05 ≤ PM < 0.1
Density
0.0
0.5
1.0
1.5
2.0
0.5
1.0
1.5
CpG o/e ratio

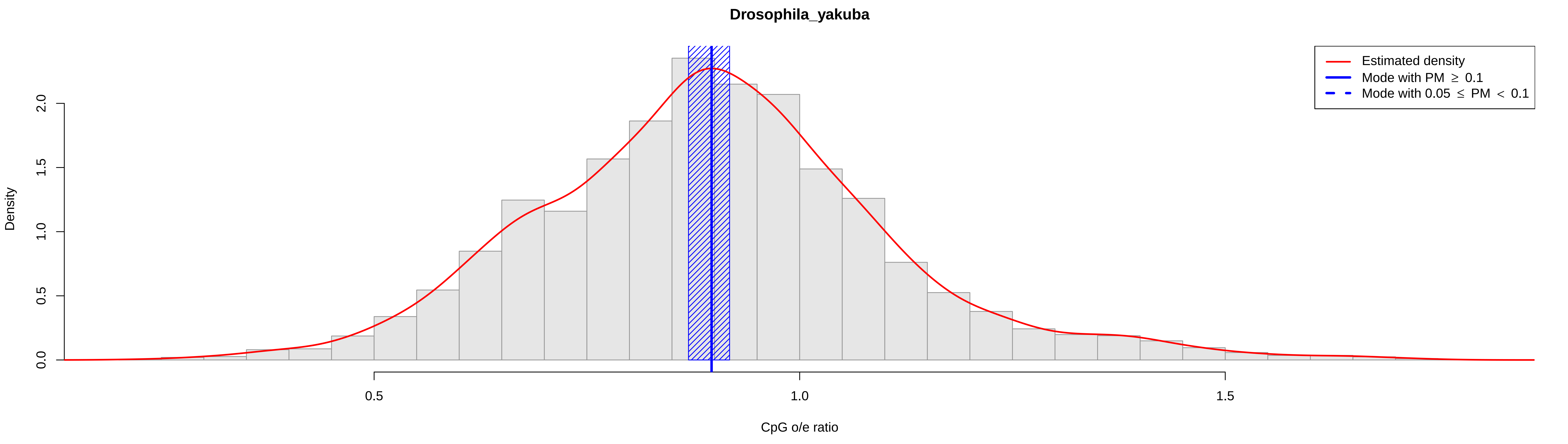

Drosophila_yakuba
Estimated density
Mode with PM ≥ 0.1
Mode with 0.05 ≤ PM < 0.1
Density
0.0
0.5
1.0
1.5
2.0
0.5
1.0
1.5
CpG o/e ratio

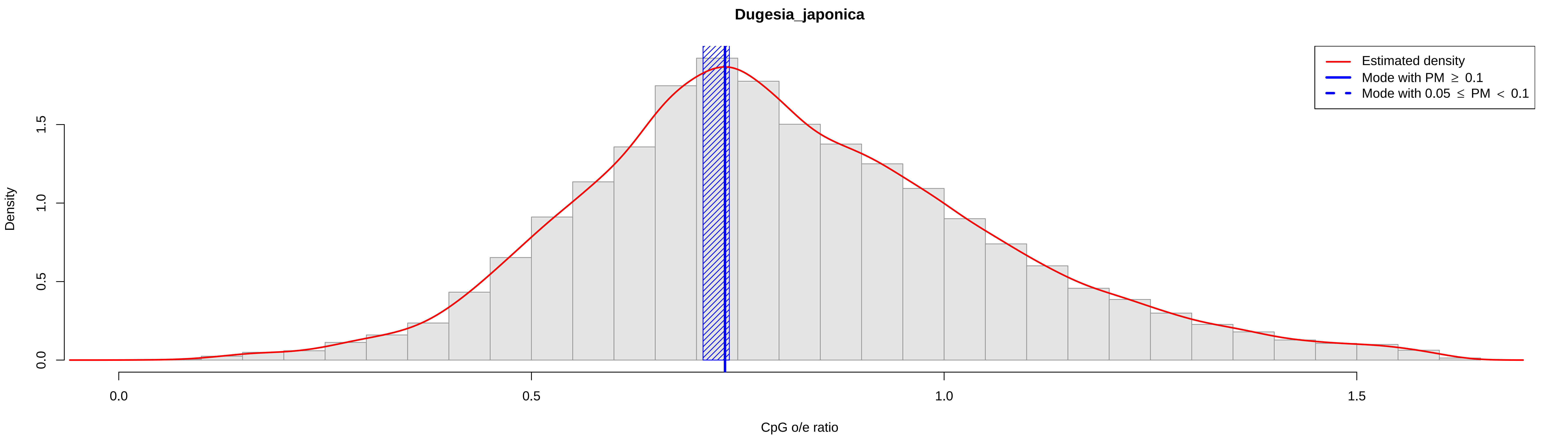

Dugesia_japonica
Estimated density
Mode with PM ≥ 0.1
Mode with 0.05 ≤ PM < 0.1
Density
0.0
0.5
1.0
1.5
0.0
0.5
1.0
1.5
CpG o/e ratio

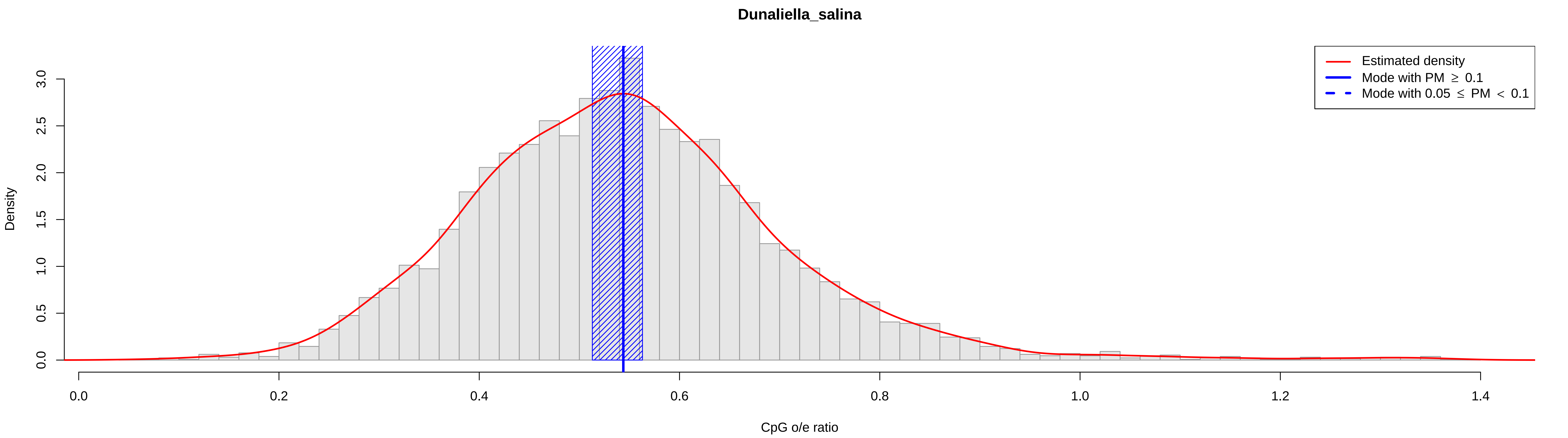

Dunaliella_salina
Estimated density
Mode with PM ≥ 0.1
Mode with 0.05 ≤ PM < 0.1
Density
0.0
0.5
1.0
1.5
2.0
2.5
3.0
0.0
0.2
0.4
0.6
0.8
1.0
1.2
1.4
CpG o/e ratio

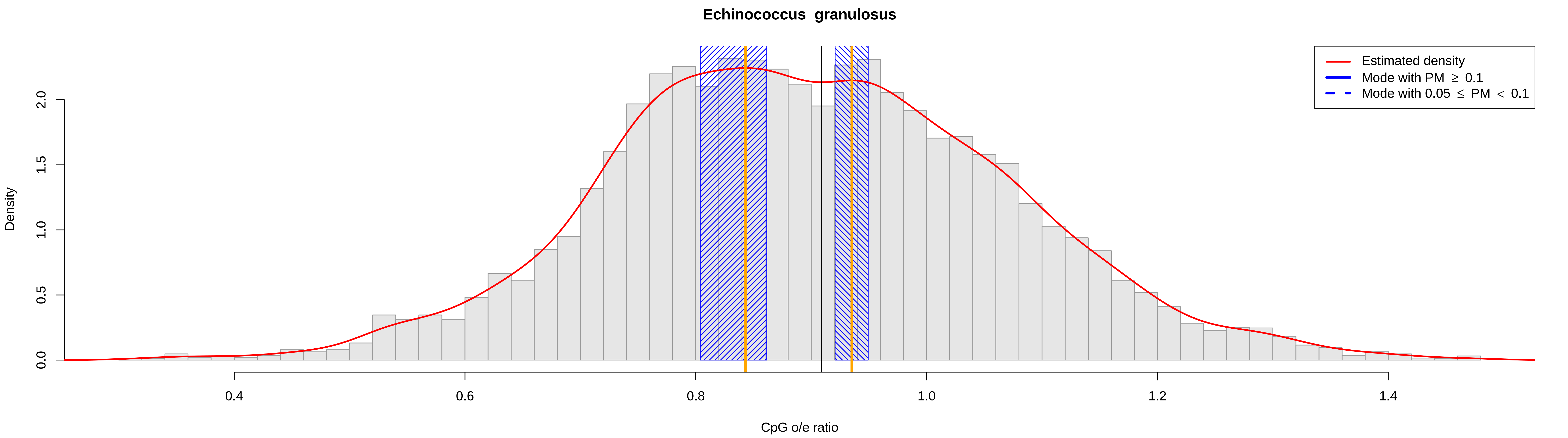
Echinococcus_granulosus
Estimated density
Mode with PM ≥ 0.1
Mode with 0.05 ≤ PM < 0.1
Density
0.0
0.5
1.0
1.5
2.0
0.4
0.6
0.8
1.0
1.2
1.4
CpG o/e ratio

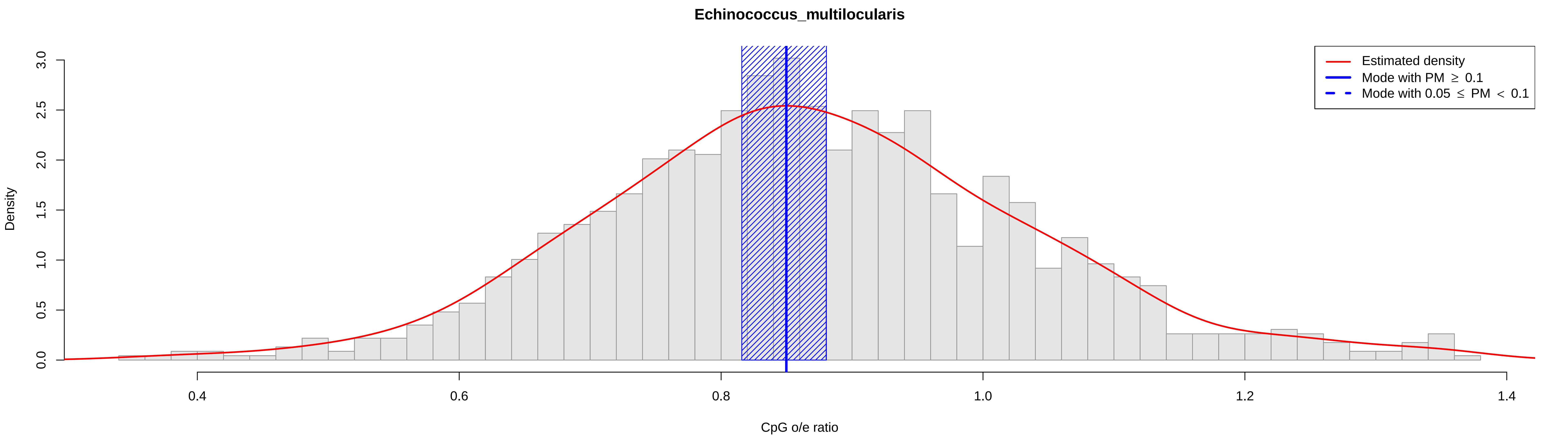

Echinococcus_multilocularis
Estimated density
Mode with PM ≥ 0.1
Mode with 0.05 ≤ PM < 0.1
Density
0.0
0.5
1.0
1.5
2.0
2.5
3.0
0.4
0.6
0.8
1.0
1.2
1.4
CpG o/e ratio

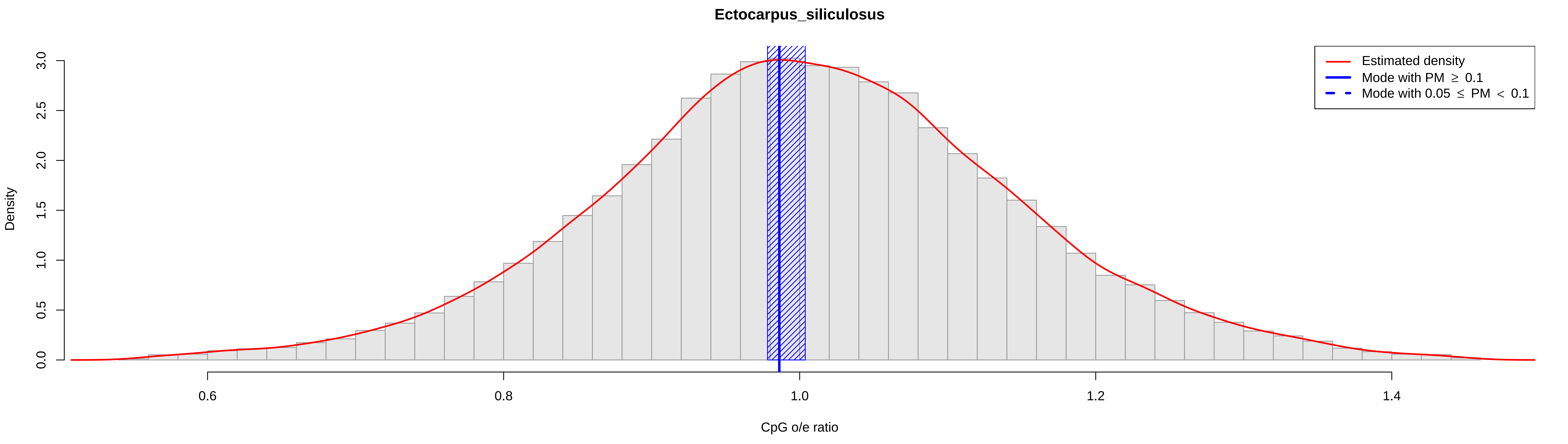
Ectocarpus_siliculosus
Estimated density
Mode with PM ≥ 0.1
Mode with 0.05 ≤ PM < 0.1
Density
0.0
0.5
1.0
1.5
2.0
2.5
3.0
0.6
0.8
1.0
1.2
1.4
CpG o/e ratio

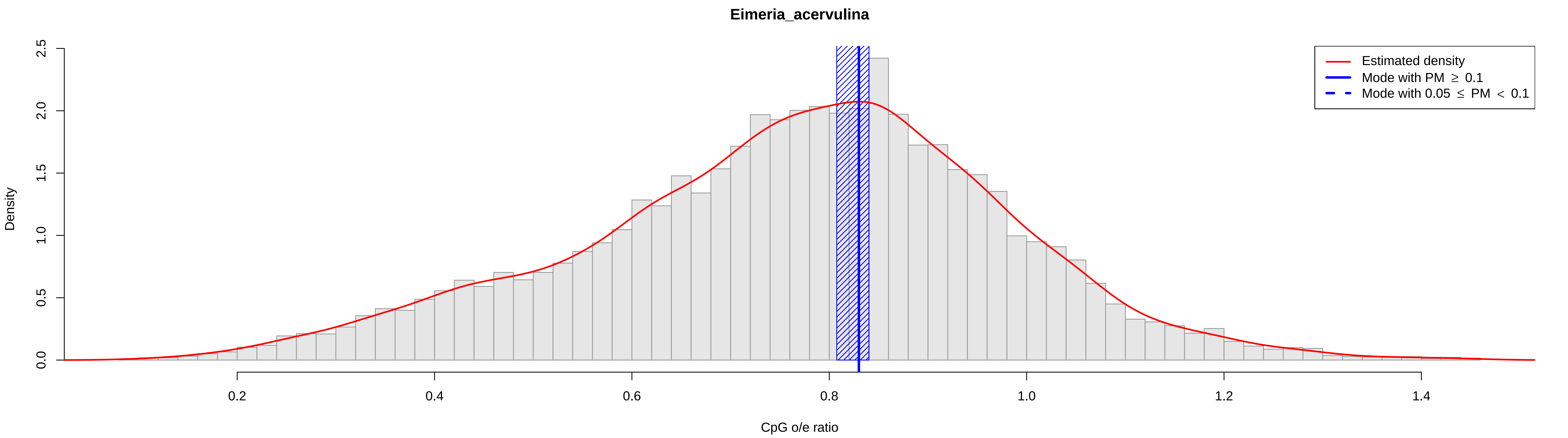

Eimeria_acervulina
Estimated density
Mode with PM ≥ 0.1
Mode with 0.05 ≤ PM < 0.1
Density
0.0
0.5
1.0
1.5
2.0
2.5
0.2
0.4
0.6
0.8
1.0
1.2
1.4
CpG o/e ratio

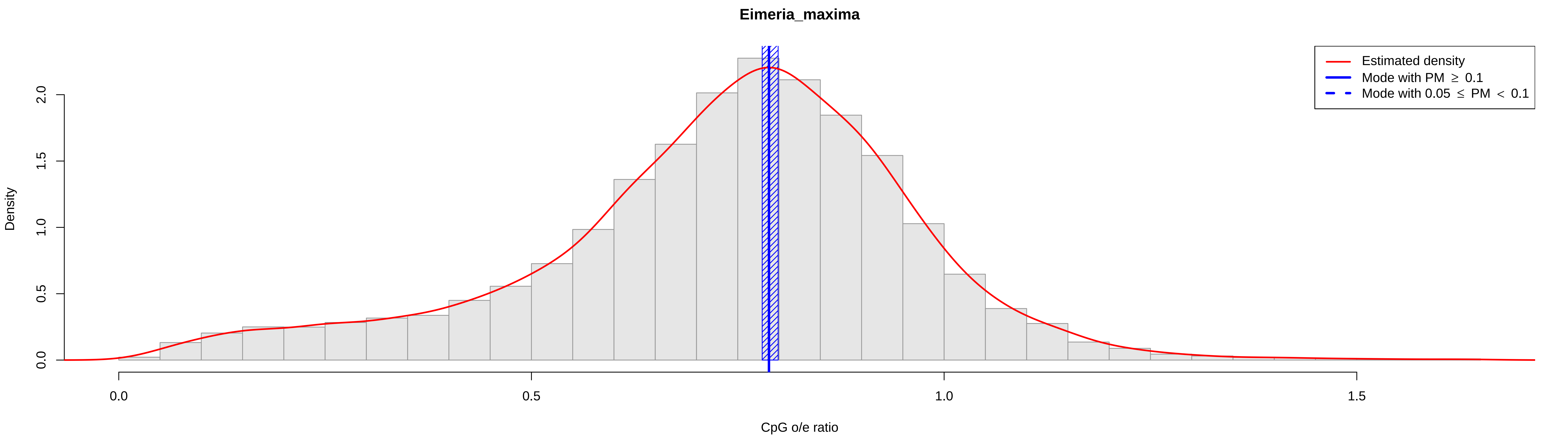

Eimeria_maxima
Estimated density
Mode with PM ≥ 0.1
Mode with 0.05 ≤ PM < 0.1
Density
0.0
0.5
1.0
1.5
2.0
0.0
0.5
1.0
1.5
CpG o/e ratio

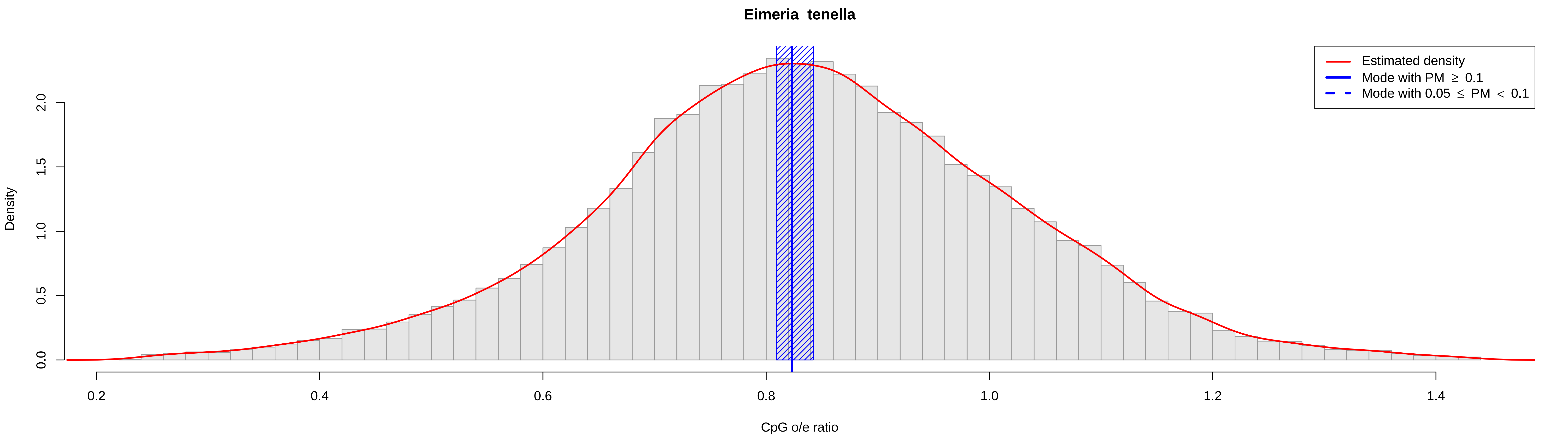

Eimeria_tenella
Estimated density
Mode with PM ≥ 0.1
Mode with 0.05 ≤ PM < 0.1
Density
0.0
0.5
1.0
1.5
2.0
0.2
0.4
0.6
0.8
1.0
1.2
1.4
CpG o/e ratio

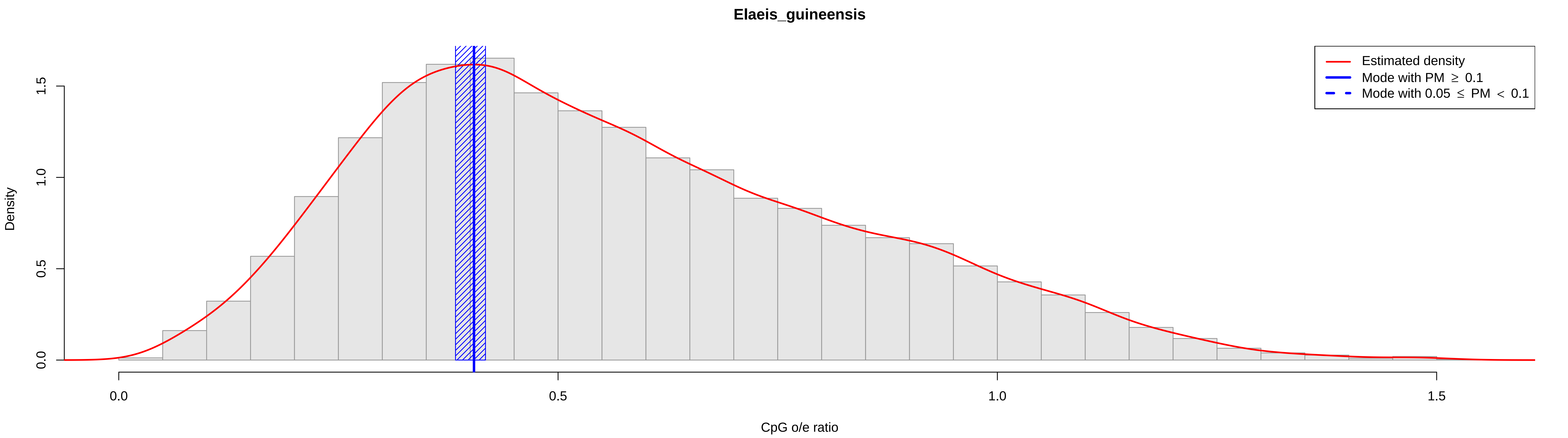
Elaeis_guineensis
Estimated density
Mode with PM ≥ 0.1
Mode with 0.05 ≤ PM < 0.1
Density
0.0
0.5
1.0
1.5
CpG o/e ratio

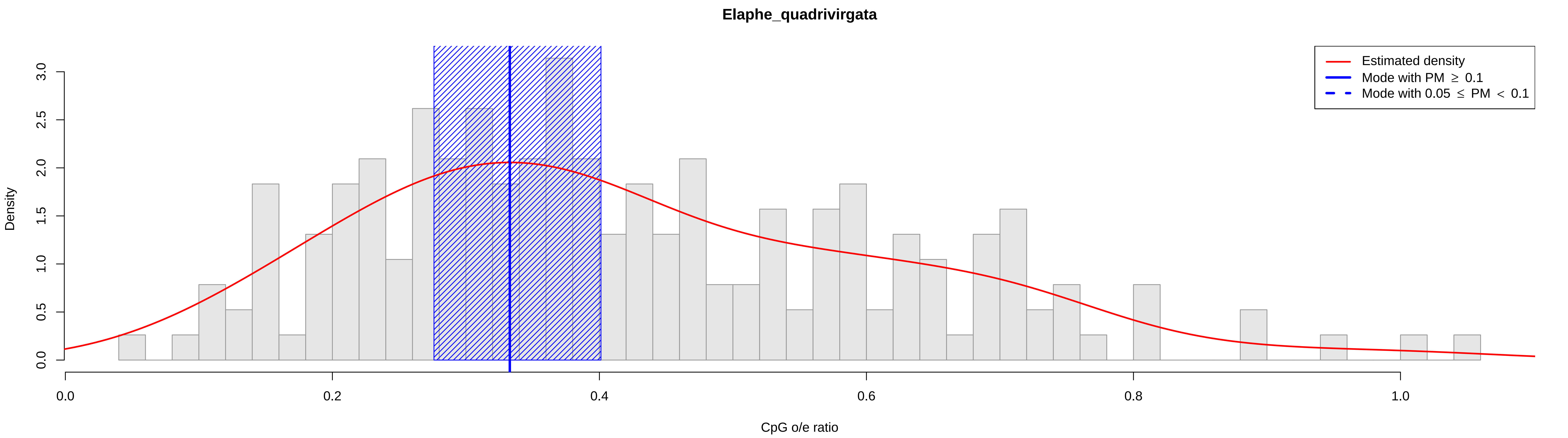
Elaphe_quadrivirgata
Estimated density
Mode with PM ≥ 0.1
Mode with 0.05 ≤ PM < 0.1
Density
CpG o/e ratio
0.0
0.5
1.0
1.5
2.0
2.5
3.0
0.0
0.2
0.4
0.6
0.8
1.0

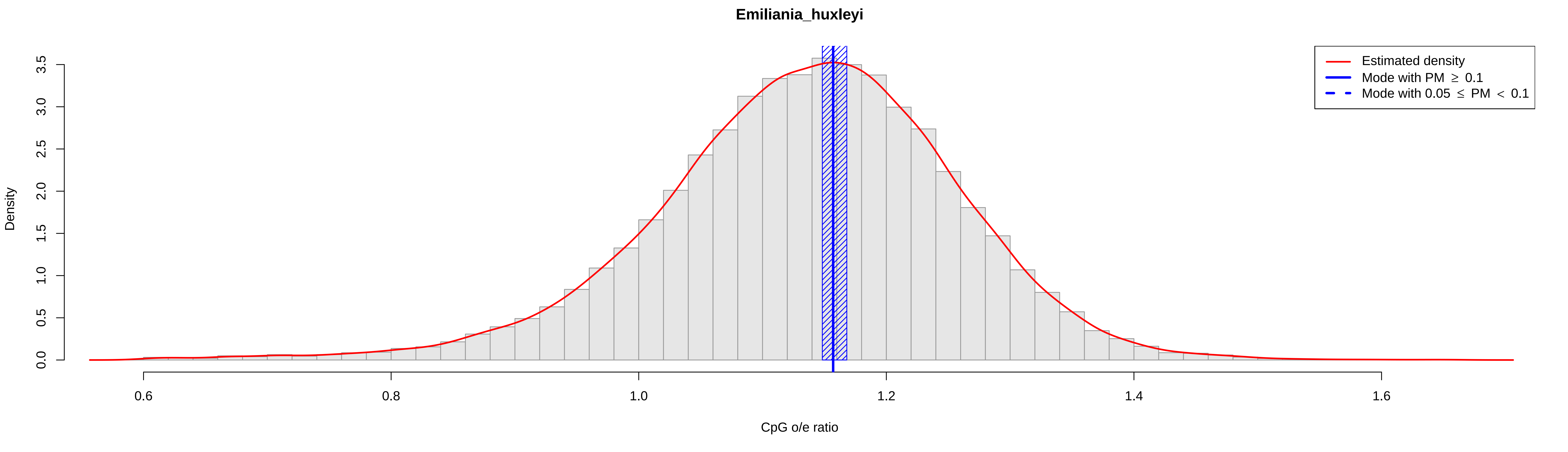

Emiliania_huxleyi
Estimated density
Mode with PM ≥ 0.1
Mode with 0.05 ≤ PM < 0.1
Density
0.0
0.5
1.0
1.5
2.0
2.5
3.0
3.5
0.6
0.8
1.0
1.2
1.4
1.6
CpG o/e ratio

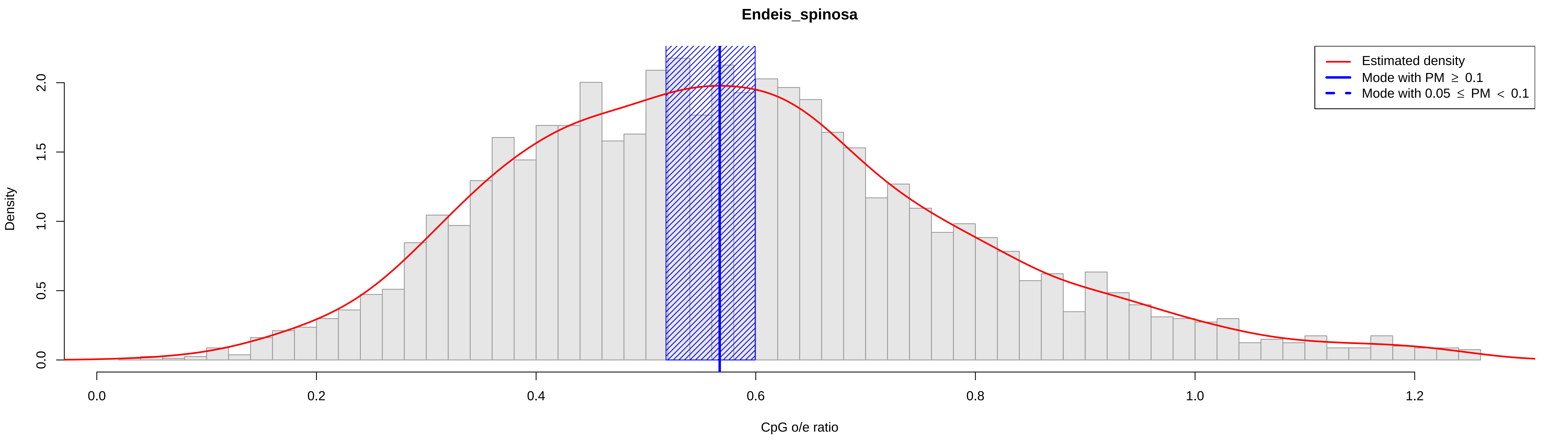

Endeis_spinosa
Estimated density
Mode with PM ≥ 0.1
Mode with 0.05 ≤ PM < 0.1
Density
0.0
0.5
1.0
1.5
2.0
0.0
0.2
0.4
0.6
0.8
1.0
1.2
CpG o/e ratio

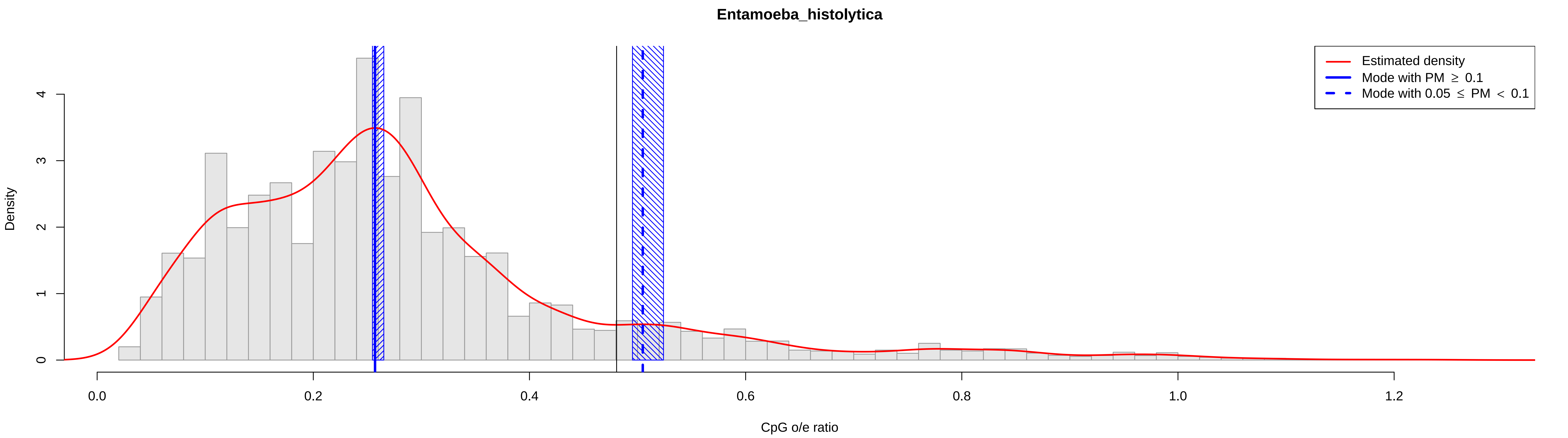

Entamoeba_histolytica
Estimated density
Mode with PM ≥ 0.1
Mode with 0.05 ≤ PM < 0.1
Density
0
1
2
3
4
0.0
0.2
0.4
0.6
0.8
1.0
1.2
CpG o/e ratio

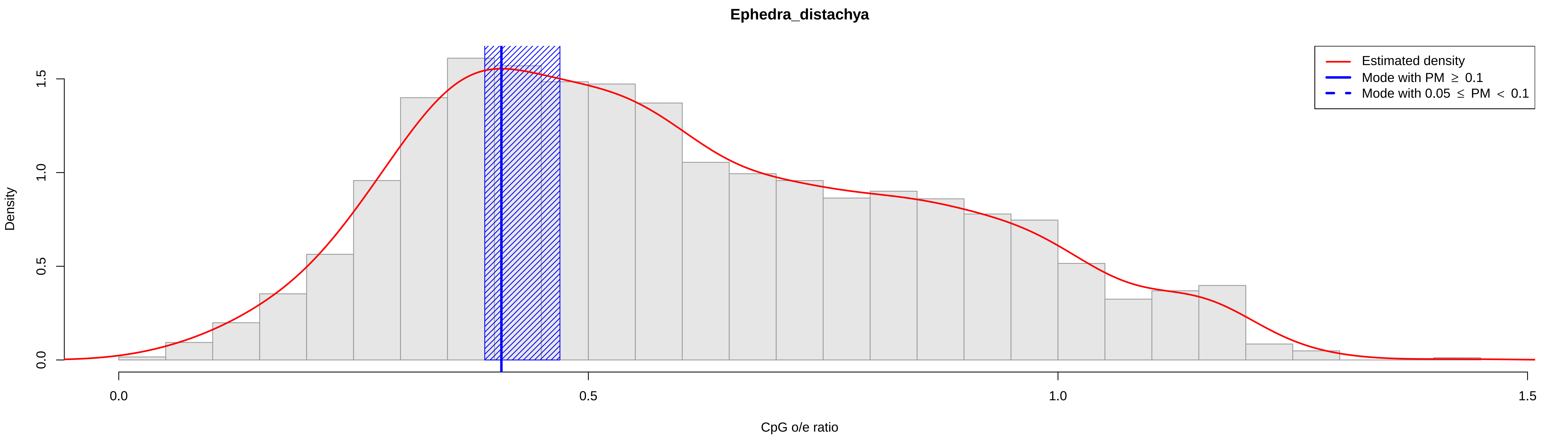

Ephedra_distachya
Estimated density
Mode with PM ≥ 0.1
Mode with 0.05 ≤ PM < 0.1
Density
0.0
0.5
1.0
1.5
0.0
0.5
1.0
1.5
CpG o/e ratio

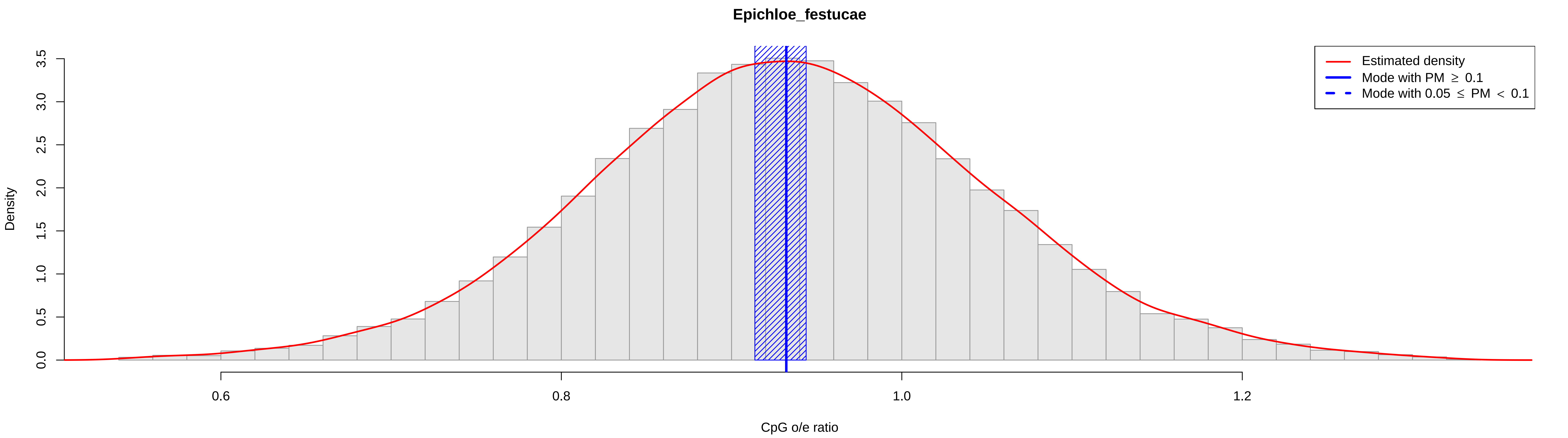

Epichloe_festucae
Estimated density
Mode with PM ≥ 0.1
Mode with 0.05 ≤ PM < 0.1
Density
0.0
0.5
1.0
1.5
2.0
2.5
3.0
3.5
0.6
0.8
1.0
1.2
CpG o/e ratio

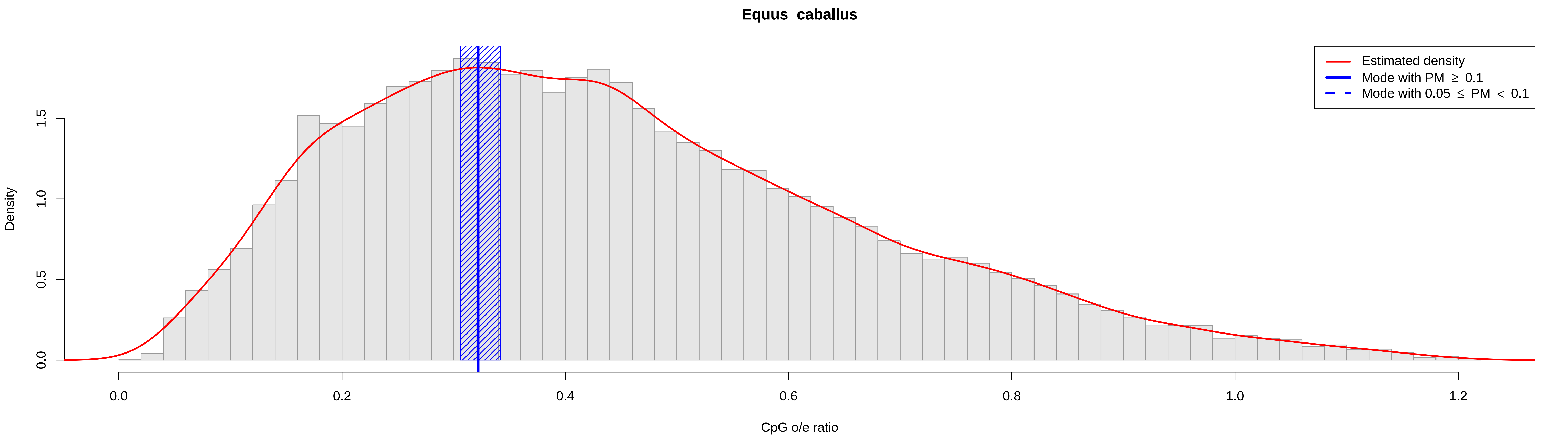

Equus_caballus
Estimated density
Mode with PM ≥ 0.1
Mode with 0.05 ≤ PM < 0.1
Density
0.0
0.5
1.0
1.5
0.0
0.2
0.4
0.6
0.8
1.0
1.2
CpG o/e ratio

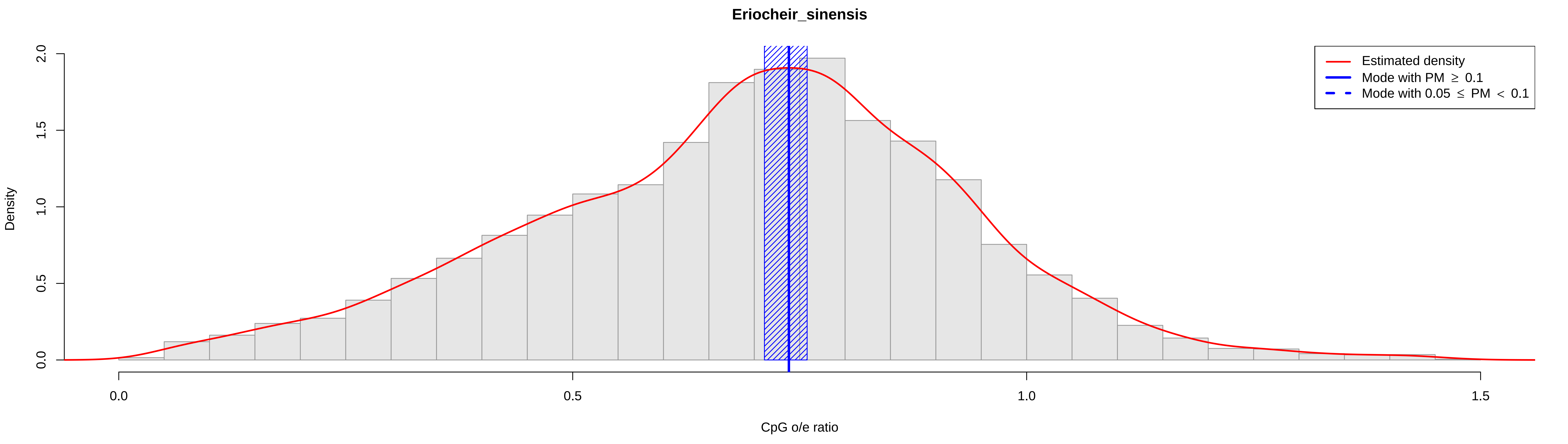

Eriocheir_sinensis
Estimated density
Mode with PM ≥ 0.1
Mode with 0.05 ≤ PM < 0.1
Density
0.0
0.5
1.0
1.5
2.0
0.0
0.5
1.0
1.5
CpG o/e ratio

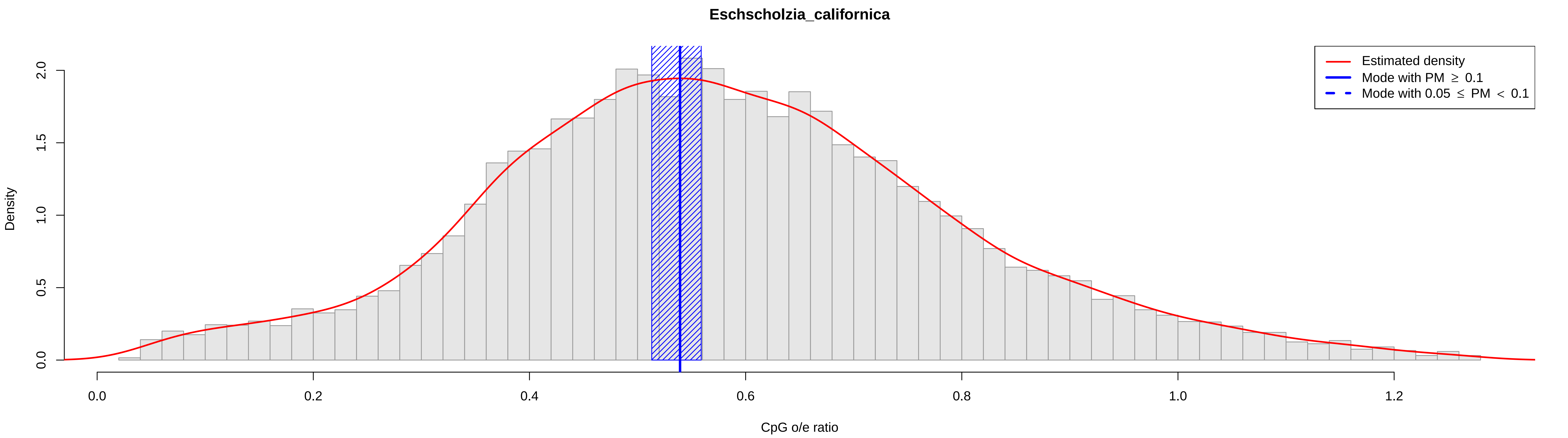

Eschscholzia_californica
Estimated density
Mode with PM ≥ 0.1
Mode with 0.05 ≤ PM < 0.1
Density
0.0
0.5
1.0
1.5
2.0
0.0
0.2
0.4
0.6
0.8
1.0
1.2
CpG o/e ratio

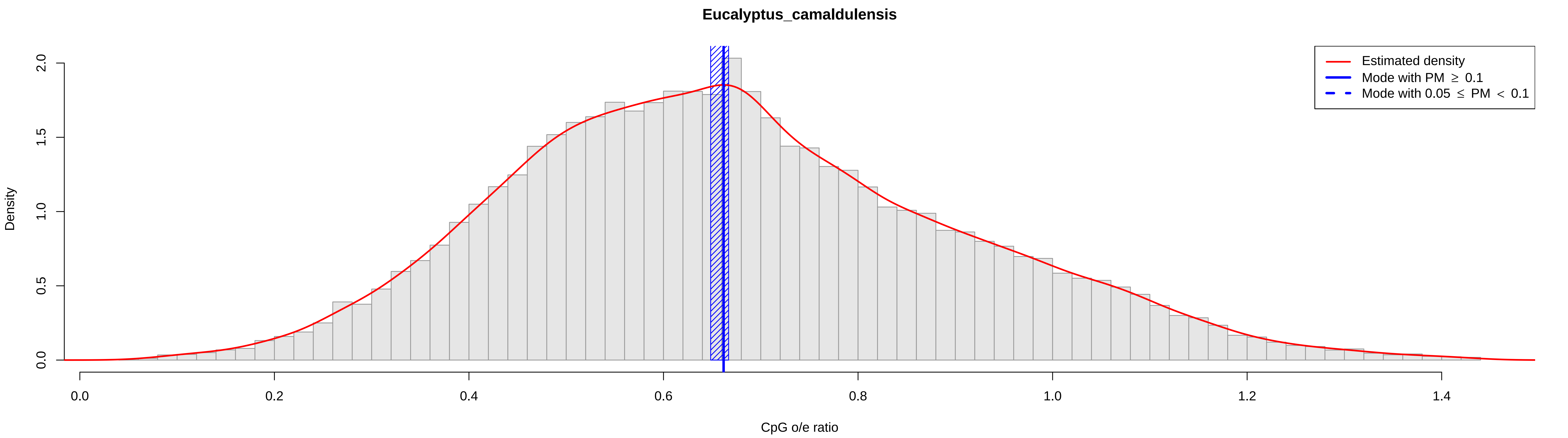
Eucalyptus_camaldulensis
Estimated density
Mode with PM ≥ 0.1
Mode with 0.05 ≤ PM < 0.1
Density
0.0
0.5
1.0
1.5
2.0
0.0
0.2
0.4
0.6
0.8
1.0
1.2
1.4
CpG o/e ratio

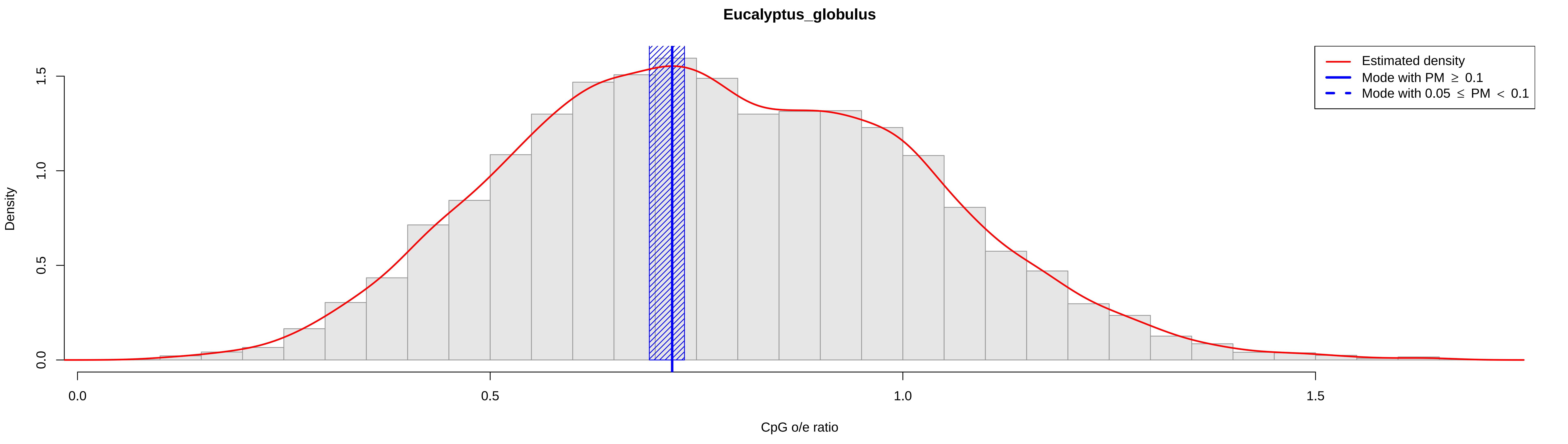

Eucalyptus_globulus
Estimated density
Mode with PM ≥ 0.1
Mode with 0.05 ≤ PM < 0.1
Density
CpG o/e ratio
0.0
0.5
1.0
1.5

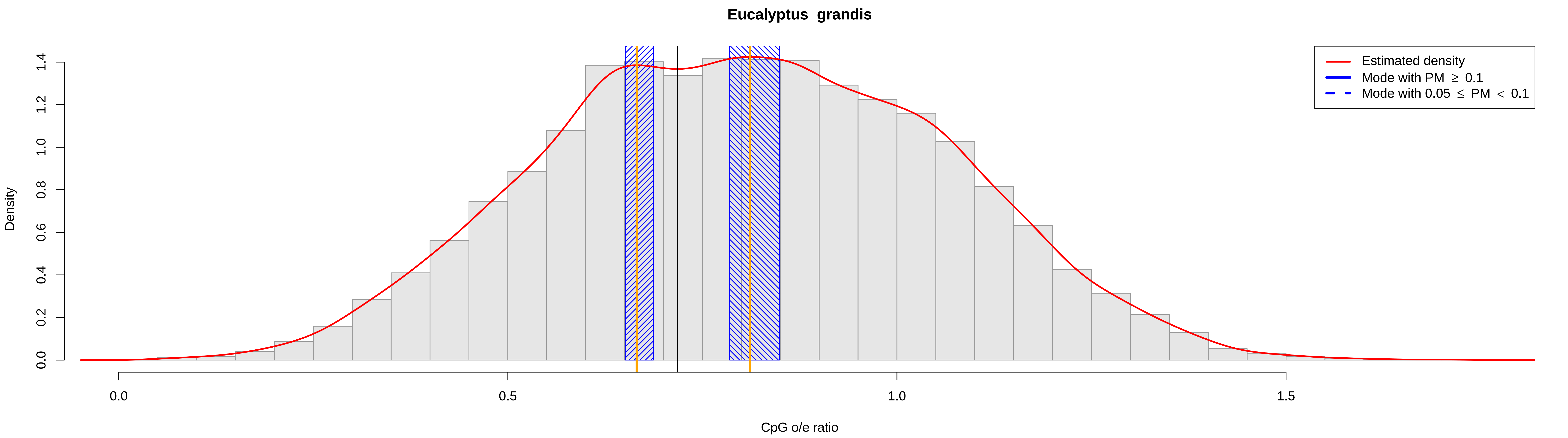

Eucalyptus_grandis
Estimated density
Mode with PM ≥ 0.1
Mode with 0.05 ≤ PM < 0.1
Density
0.0
0.2
0.4
0.6
0.8
1.0
1.2
1.4
0.0
0.5
1.0
1.5
CpG o/e ratio

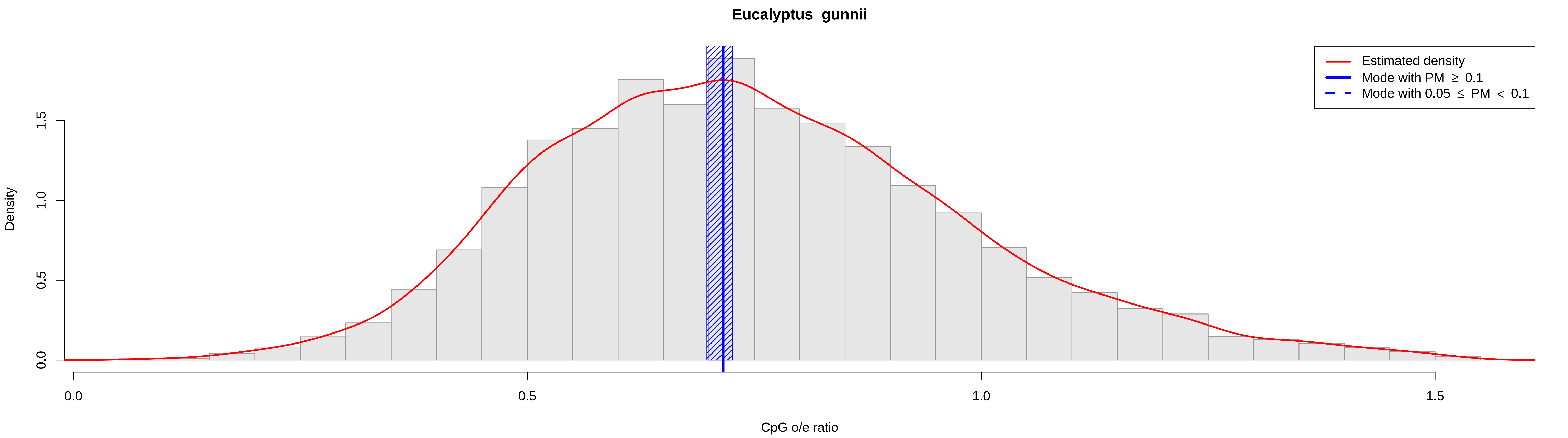

Eucalyptus_gunnii
Estimated density
Mode with PM ≥ 0.1
Mode with 0.05 ≤ PM < 0.1
Density
0.0
0.5
1.0
1.5
0.0
0.5
1.0
1.5
CpG o/e ratio

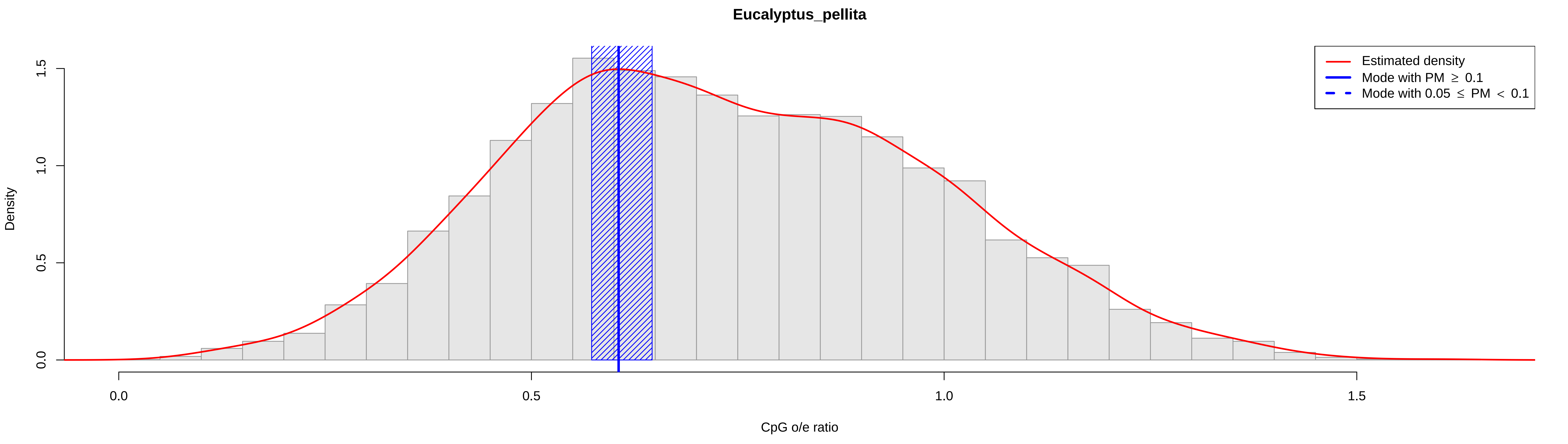

Eucalyptus_pellita
Estimated density
Mode with PM ≥ 0.1
Mode with 0.05 ≤ PM < 0.1
Density
0.0
0.5
1.0
1.5
0.0
0.5
1.0
1.5
CpG o/e ratio

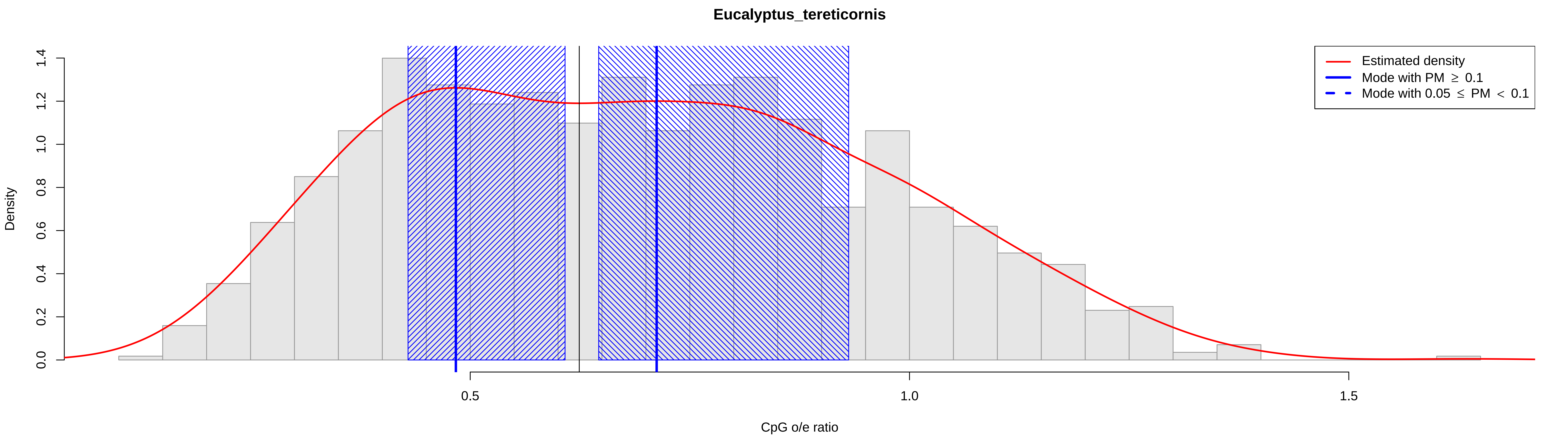

Eucalyptus_tereticornis
Density
0.0
0.2
0.4
0.6
0.8
1.0
1.2
1.4
0.5
1.0
1.5
CpG o/e ratio
Estimated density
Mode with PM ≥ 0.1
Mode with 0.05 ≤ PM < 0.1

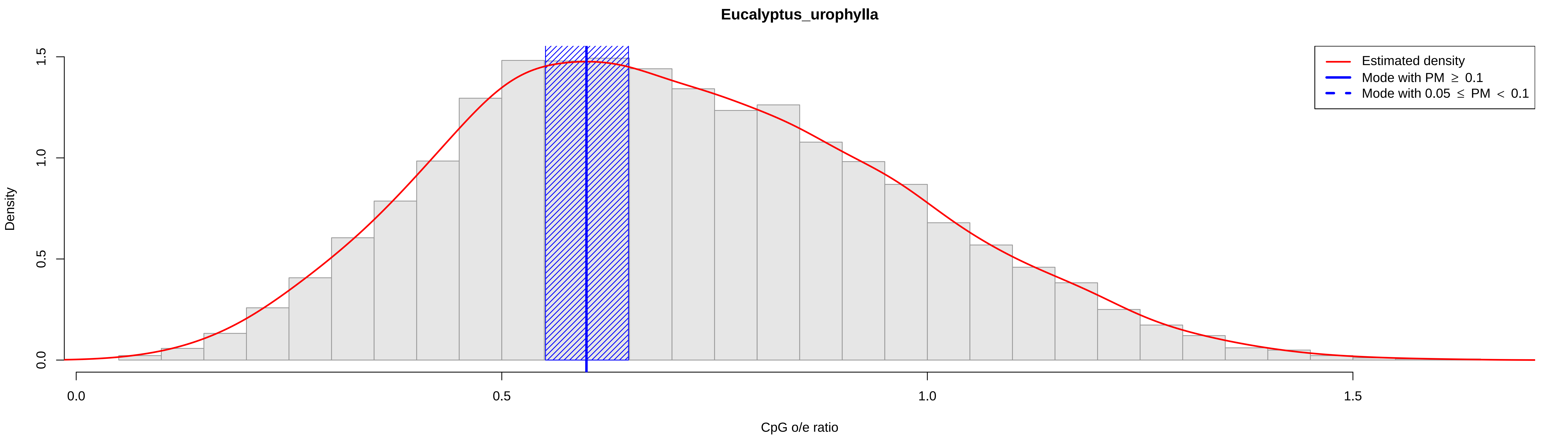

Eucalyptus_urophylla
Estimated density
Mode with PM ≥ 0.1
Mode with 0.05 ≤ PM < 0.1
Density
0.0
0.5
1.0
1.5
0.0
0.5
1.0
1.5
CpG o/e ratio

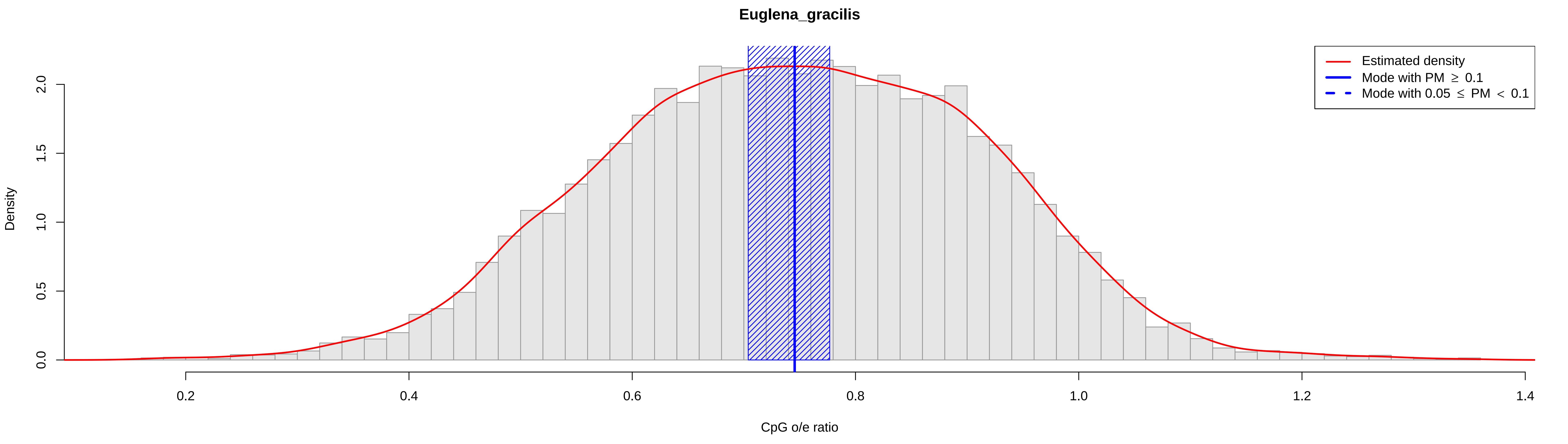

Euglena_gracilis
Estimated density
Mode with PM ≥ 0.1
Mode with 0.05 ≤ PM < 0.1
Density
0.0
0.5
1.0
1.5
2.0
0.2
0.4
0.6
0.8
1.0
1.2
1.4
CpG o/e ratio

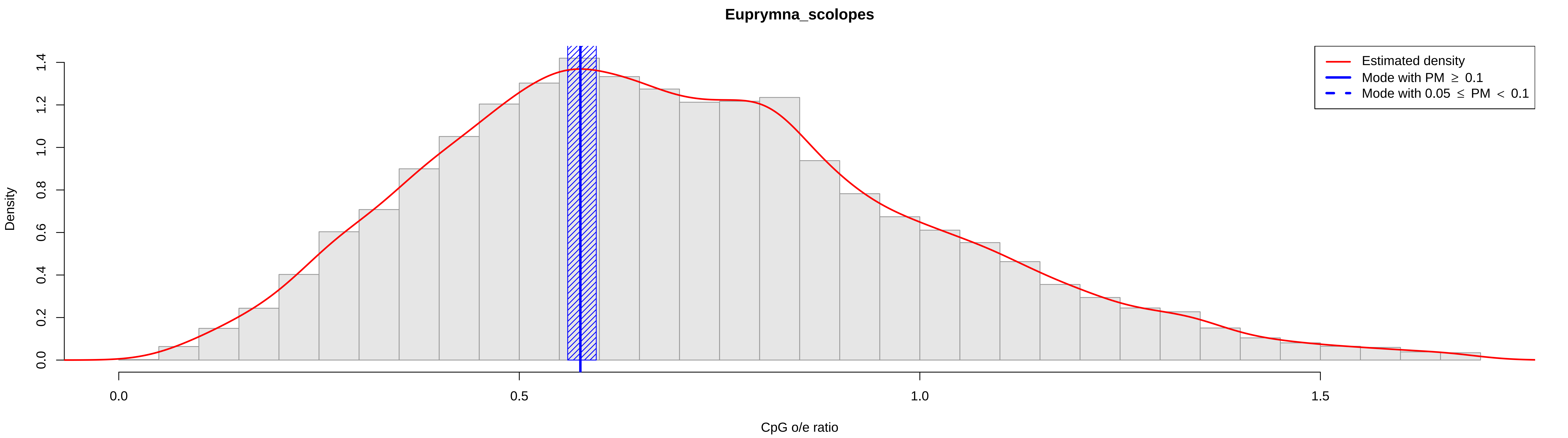
Euprymna_scolopes
Estimated density
Mode with PM ≥ 0.1
Mode with 0.05 ≤ PM < 0.1
Density
0.0
0.2
0.4
0.6
0.8
1.0
1.2
1.4
0.0
0.5
1.0
1.5
CpG o/e ratio

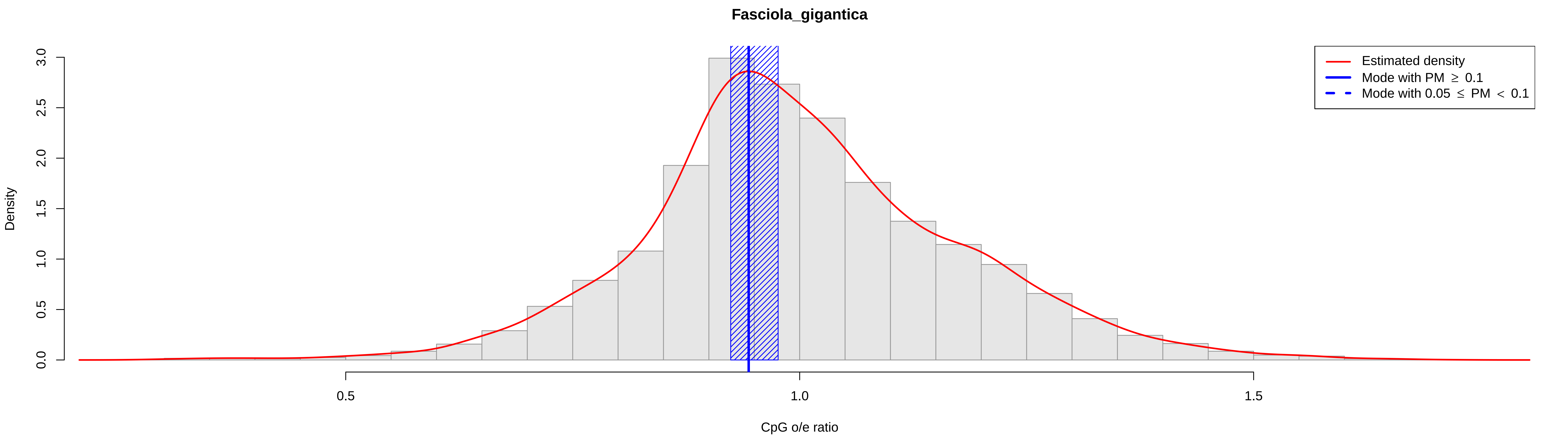
Fasciola_gigantica
Density
0.0
0.5
1.0
1.5
2.0
2.5
3.0
0.5
1.0
1.5
CpG o/e ratio
Estimated density
Mode with PM ≥ 0.1
Mode with 0.05 ≤ PM < 0.1

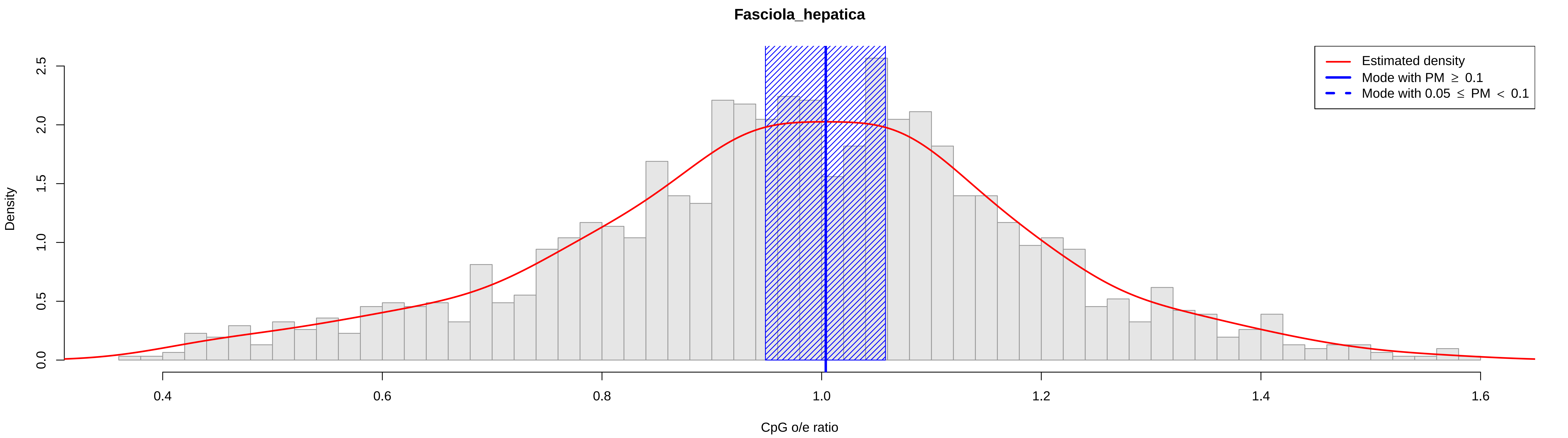
Fasciola_hepatica
Estimated density
Mode with PM ≥ 0.1
Mode with 0.05 ≤ PM < 0.1
Density
0.0
0.5
1.0
1.5
2.0
2.5
0.4
0.6
0.8
1.0
1.2
1.4
1.6
CpG o/e ratio

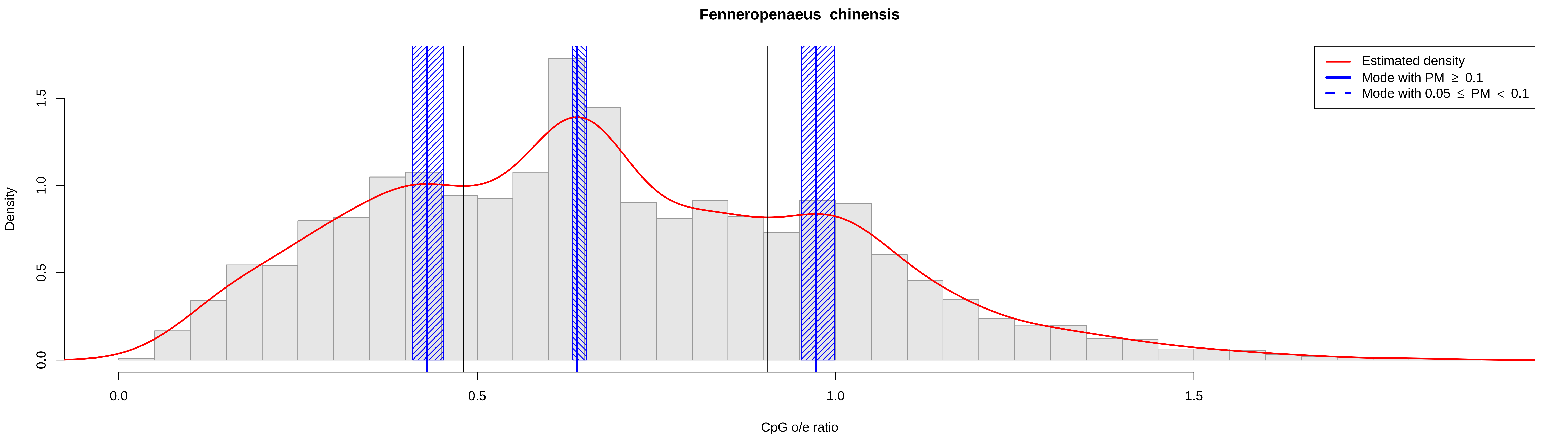

Fenneropenaeus_chinensis
Estimated density
Mode with PM ≥ 0.1
Mode with 0.05 ≤ PM < 0.1
Density
0.0
0.5
1.0
1.5
0.0
0.5
1.0
1.5
CpG o/e ratio

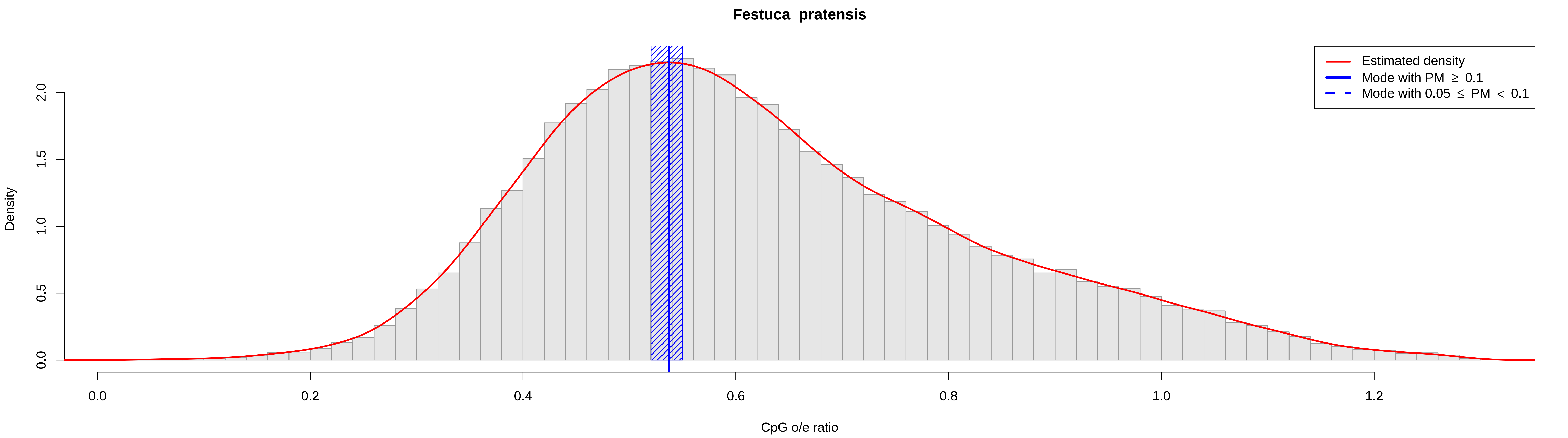

Festuca_pratensis
Estimated density
Mode with PM ≥ 0.1
Mode with 0.05 ≤ PM < 0.1
Density
0.0
0.5
1.0
1.5
2.0
0.0
0.2
0.4
0.6
0.8
1.0
1.2
CpG o/e ratio

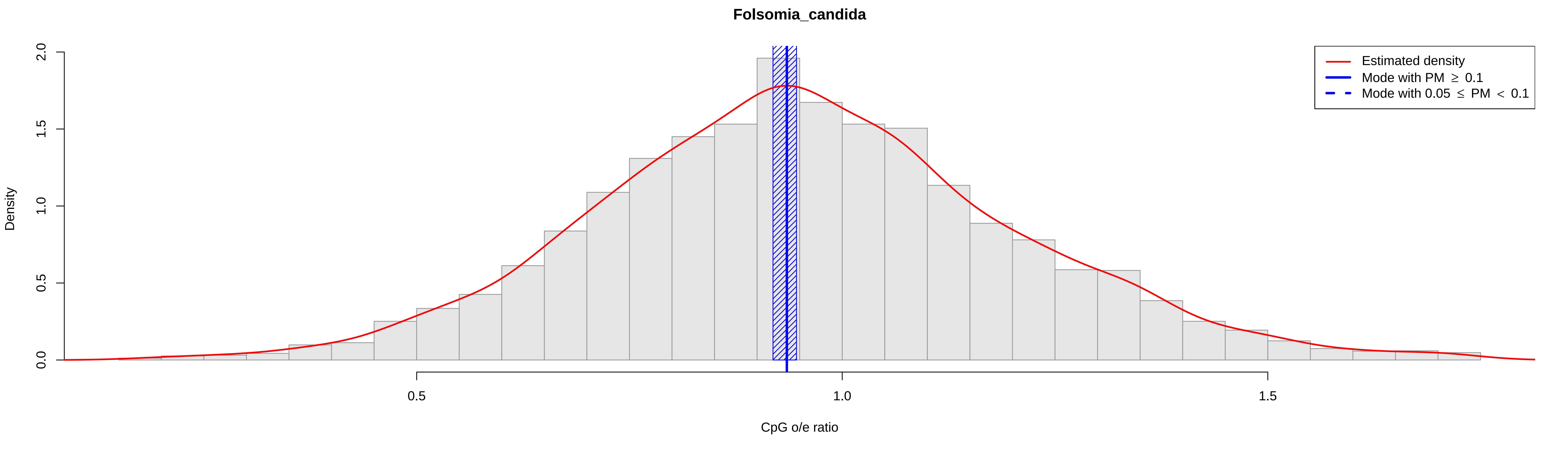

Folsomia_candida
Estimated density
Mode with PM ≥ 0.1
Mode with 0.05 ≤ PM < 0.1
Density
0.0
0.5
1.0
1.5
2.0
0.5
1.0
1.5
CpG o/e ratio

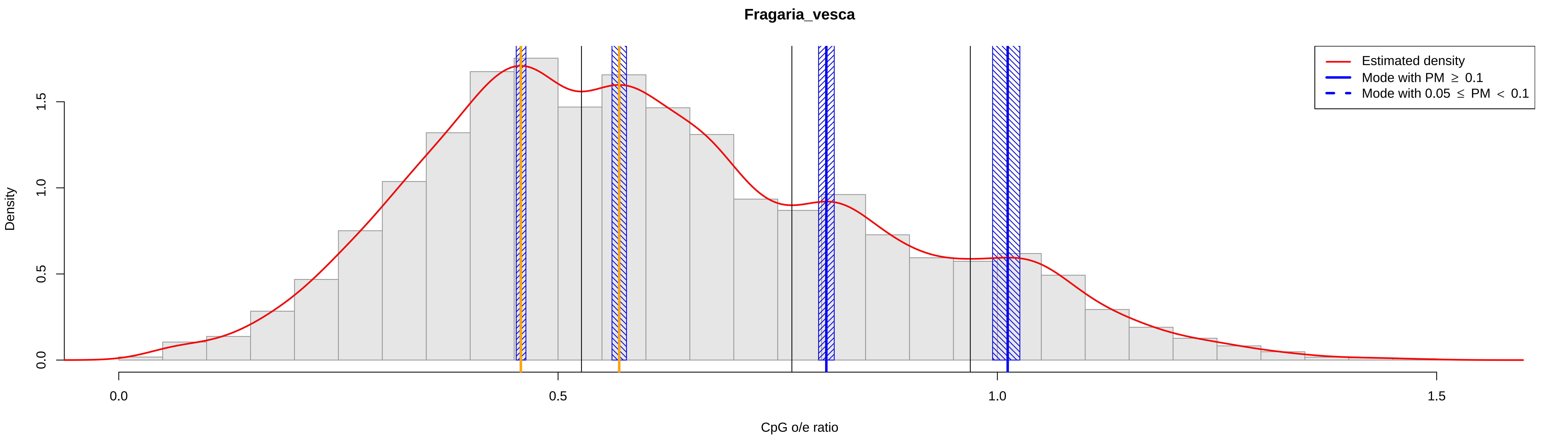
Fragaria_vesca
Estimated density
Mode with PM ≥ 0.1
Mode with 0.05 ≤ PM < 0.1
Density
0.0
0.5
1.0
1.5
0.0
0.5
1.0
1.5
CpG o/e ratio

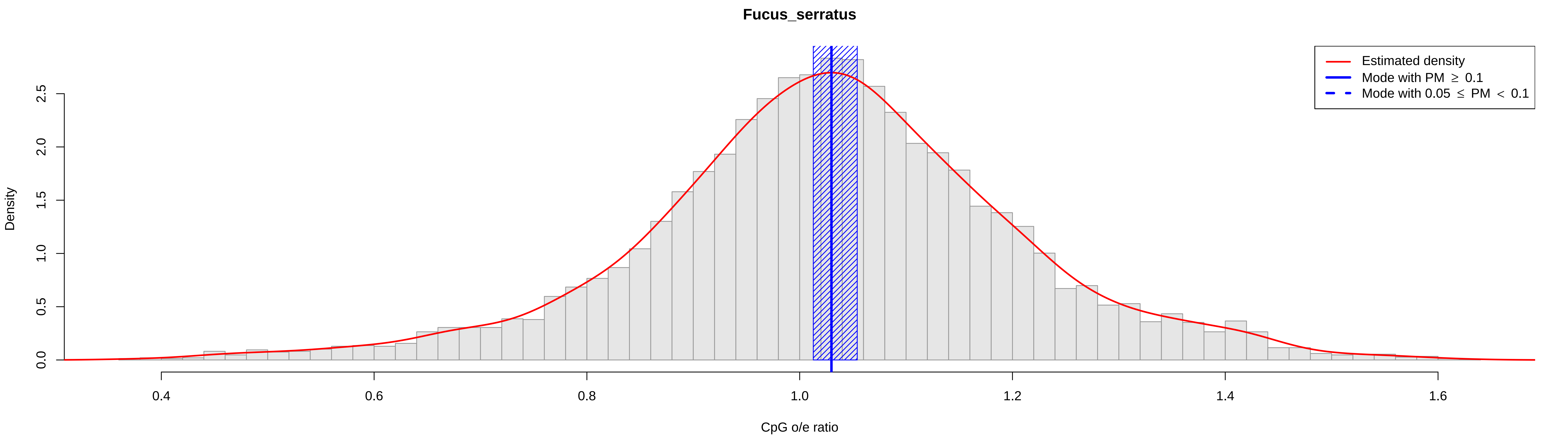
Fucus_serratus
Estimated density
Mode with PM ≥ 0.1
Mode with 0.05 ≤ PM < 0.1
Density
0.0
0.5
1.0
1.5
2.0
2.5
0.4
0.6
0.8
1.0
1.2
1.4
1.6
CpG o/e ratio

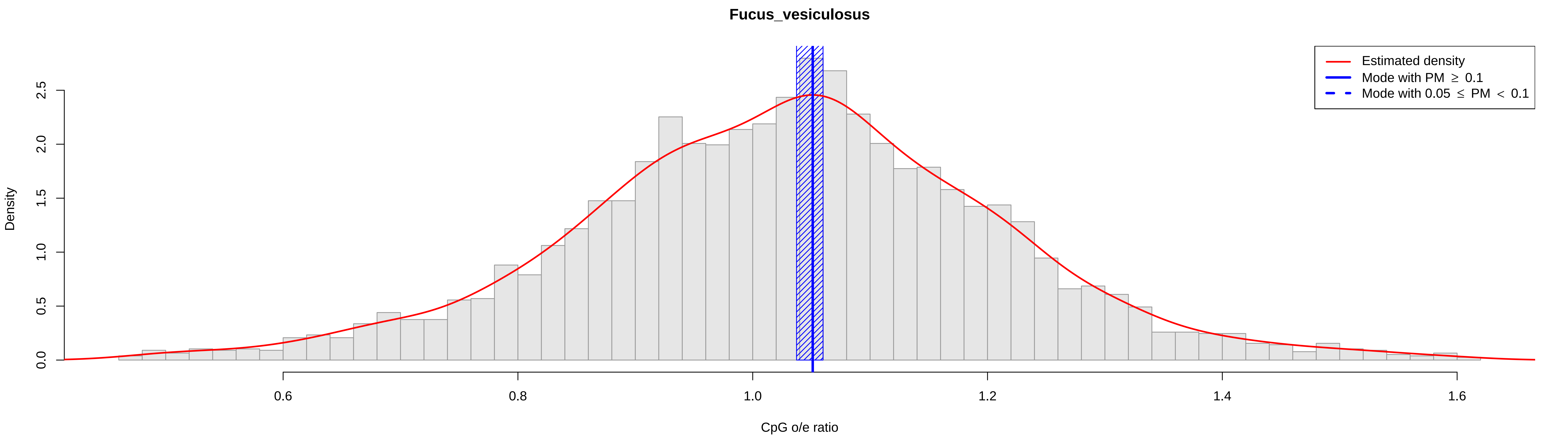
Fucus_vesiculosus
Estimated density
Mode with PM ≥ 0.1
Mode with 0.05 ≤ PM < 0.1
Density
0.0
0.5
1.0
1.5
2.0
2.5
0.6
0.8
1.0
1.2
1.4
1.6
CpG o/e ratio

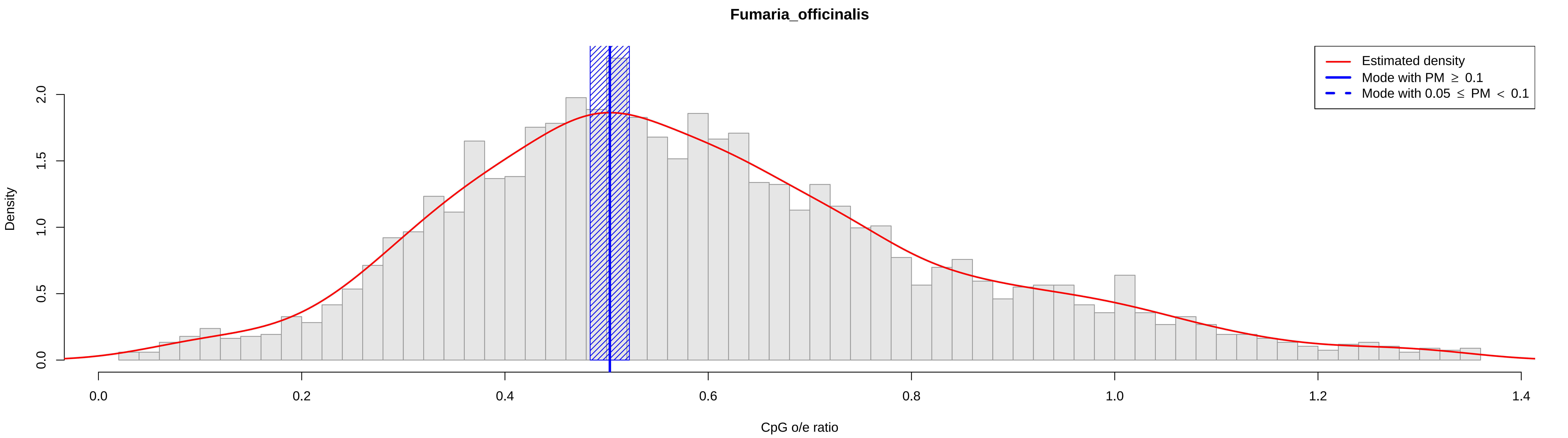
Fumaria_officinalis
Estimated density
Mode with PM ≥ 0.1
Mode with 0.05 ≤ PM < 0.1
Density
0.0
0.5
1.0
1.5
2.0
0.0
0.2
0.4
0.6
0.8
1.0
1.2
1.4
CpG o/e ratio

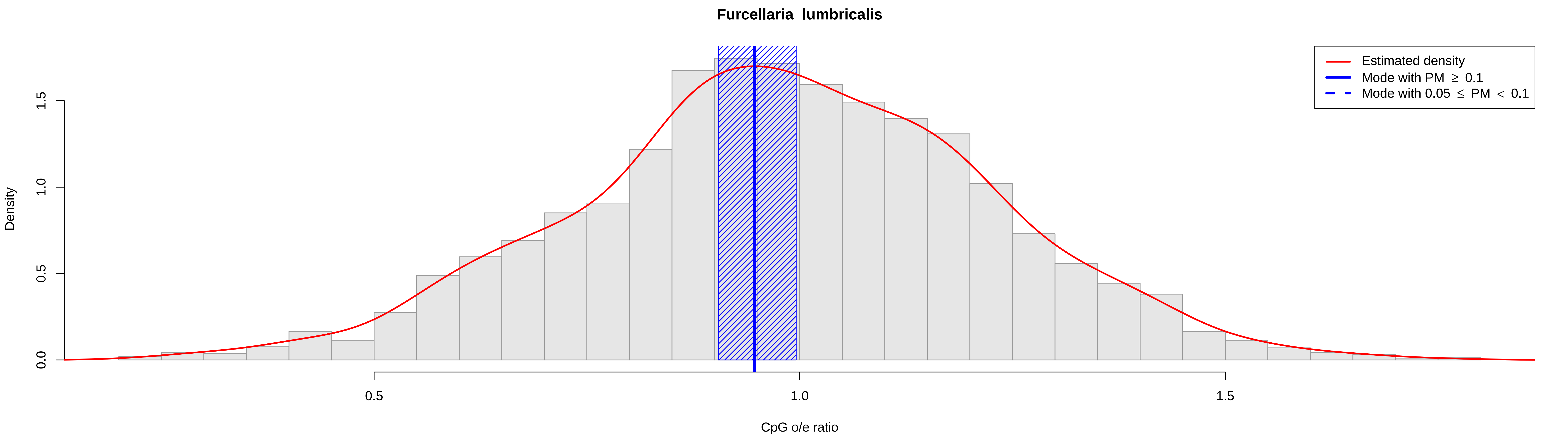

Furcellaria_lumbricalis
Estimated density
Mode with PM ≥ 0.1
Mode with 0.05 ≤ PM < 0.1
Density
0.0
0.5
1.0
1.5
0.5
1.0
1.5
CpG o/e ratio

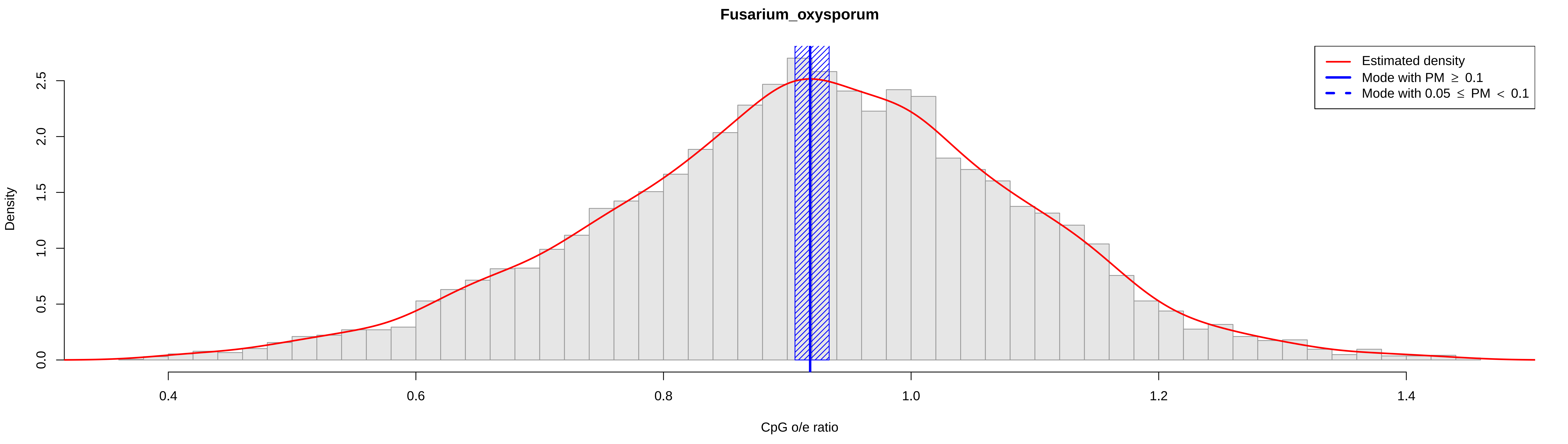

Fusarium_oxysporum
Estimated density
Mode with PM ≥ 0.1
Mode with 0.05 ≤ PM < 0.1
Density
0.0
0.5
1.0
1.5
2.0
2.5
0.4
0.6
0.8
1.0
1.2
1.4
CpG o/e ratio

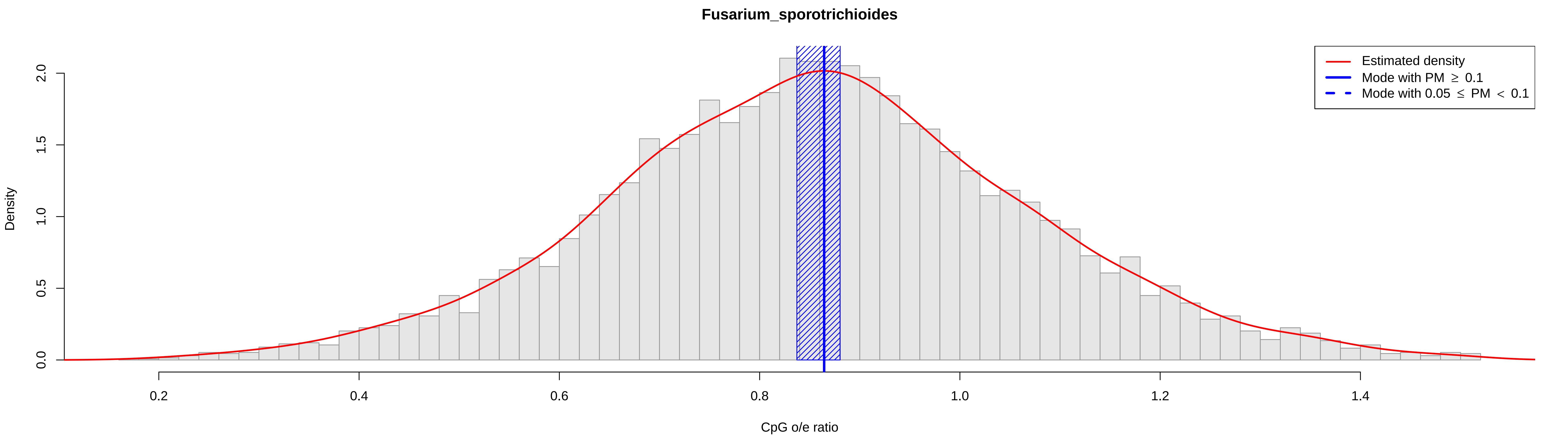

Fusarium_sporotrichioides
Estimated density
Mode with PM ≥ 0.1
Mode with 0.05 ≤ PM < 0.1
Density
0.0
0.5
1.0
1.5
2.0
0.2
0.4
0.6
0.8
1.0
1.2
1.4
CpG o/e ratio

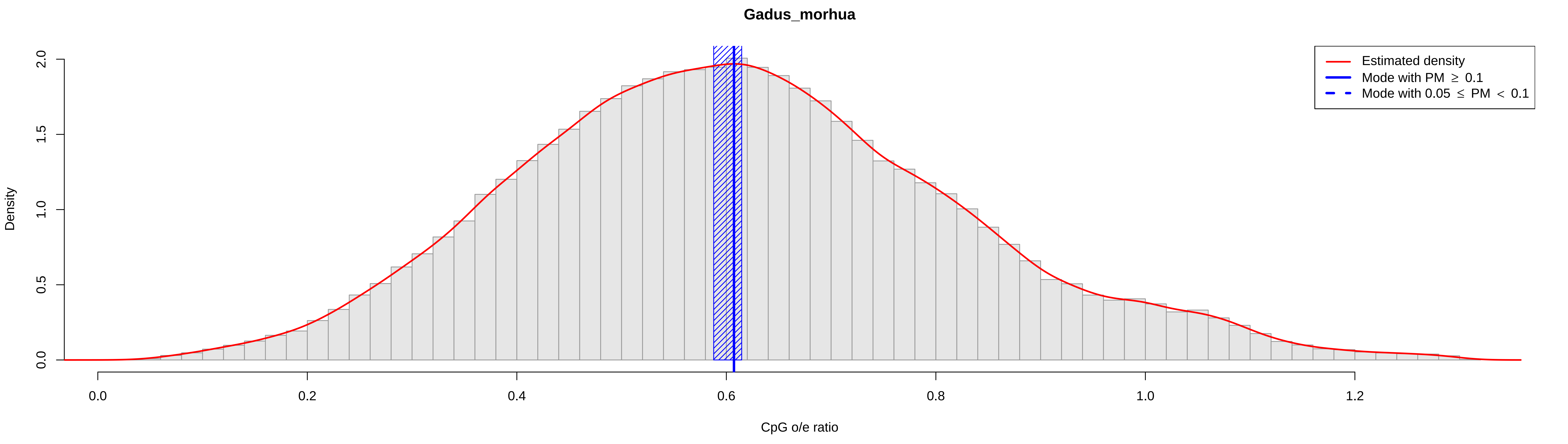

Gadus_morhua
Estimated density
Mode with PM ≥ 0.1
Mode with 0.05 ≤ PM < 0.1
Density
CpG o/e ratio
0.0
0.5
1.0
1.5
2.0
0.0
0.2
0.4
0.6
0.8
1.0
1.2

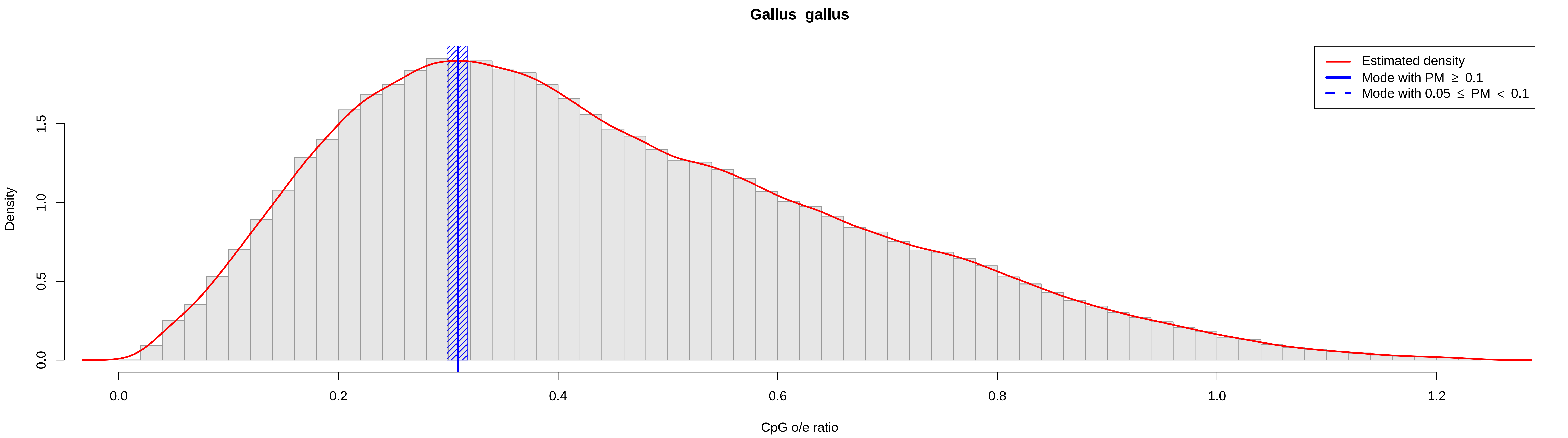

Gallus_gallus
Estimated density
Mode with PM ≥ 0.1
Mode with 0.05 ≤ PM < 0.1
Density
0.0
0.5
1.0
1.5
0.0
0.2
0.4
0.6
0.8
1.0
1.2
CpG o/e ratio

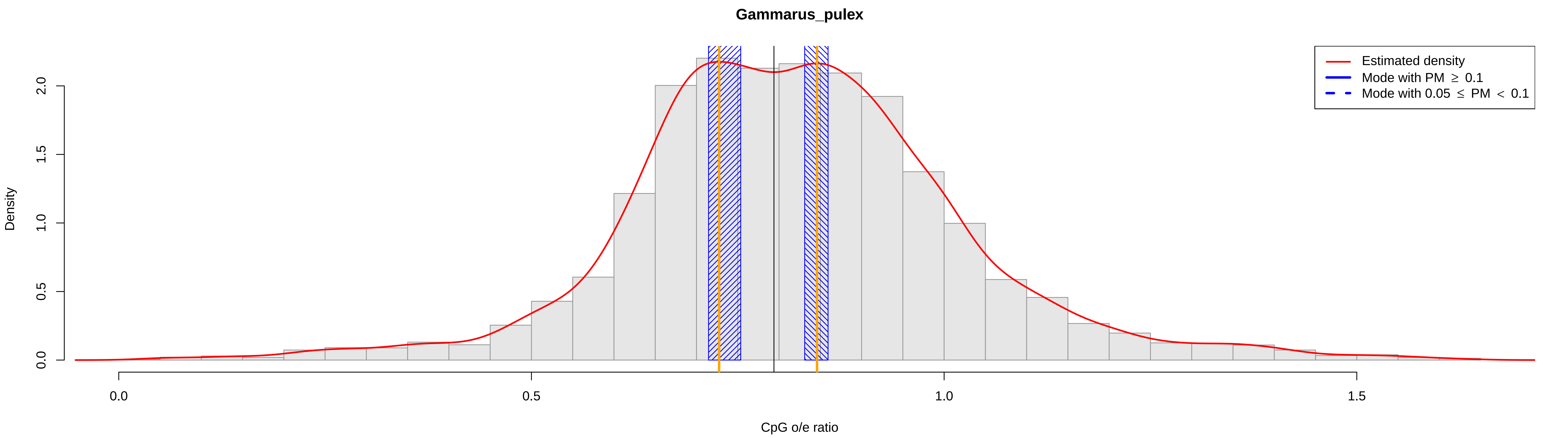

Gammarus_pulex
Estimated density
Mode with PM ≥ 0.1
Mode with 0.05 ≤ PM < 0.1
Density
0.0
0.5
1.0
1.5
2.0
0.0
0.5
1.0
1.5
CpG o/e ratio

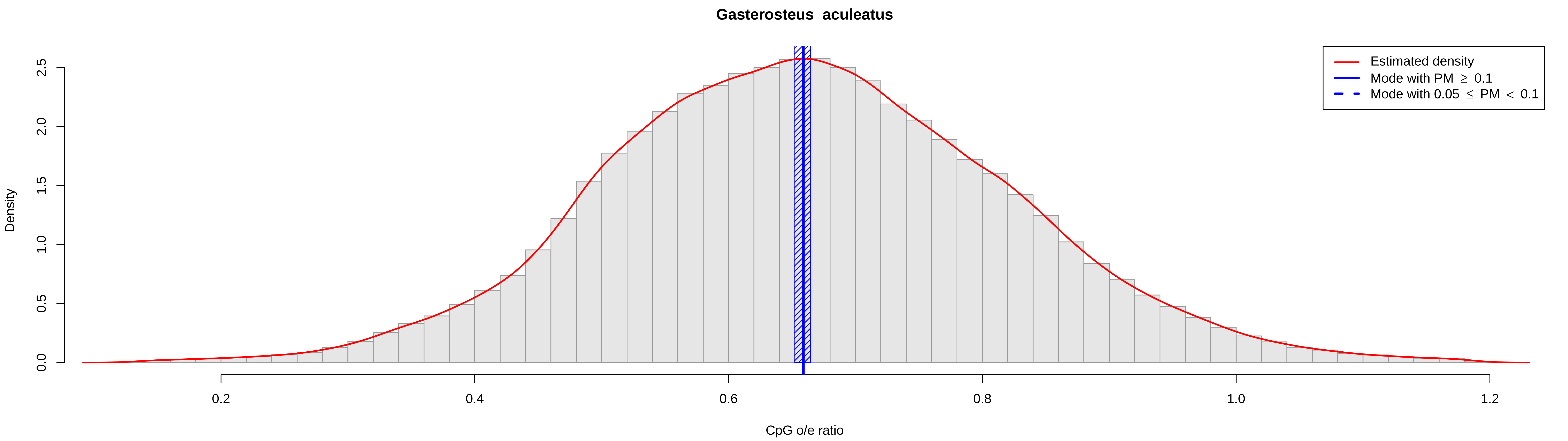

Gasterosteus_aculeatus
Estimated density
Mode with PM ≥ 0.1
Mode with 0.05 ≤ PM < 0.1
Density
0.0
0.5
1.0
1.5
2.0
2.5
0.2
0.4
0.6
0.8
1.0
1.2
CpG o/e ratio

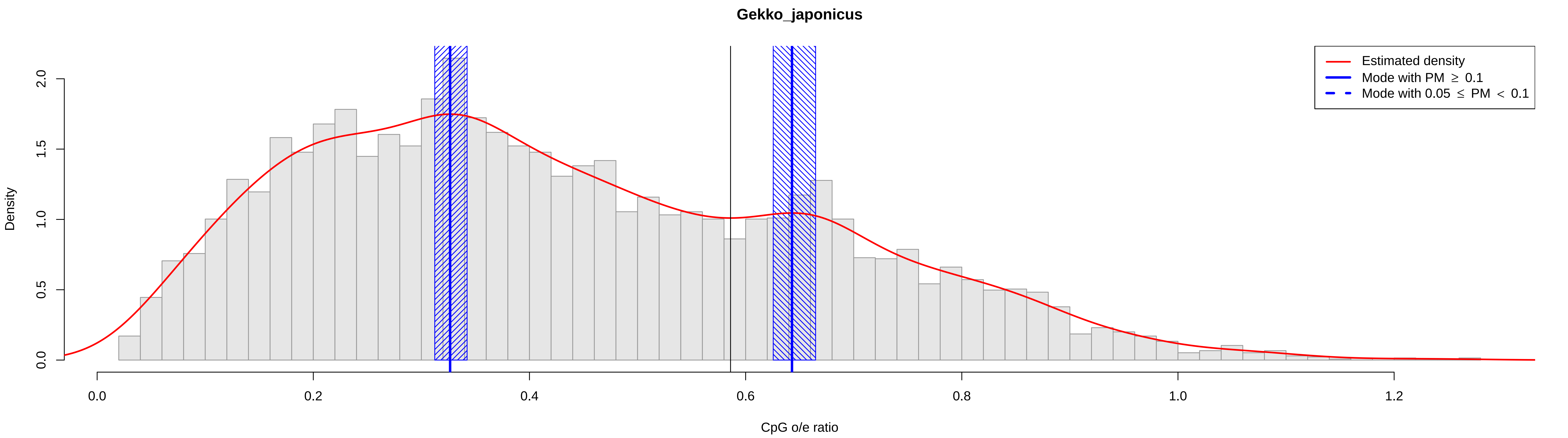
Gekko_japonicus
Estimated density
Mode with PM ≥ 0.1
Mode with 0.05 ≤ PM < 0.1
Density
0.0
0.5
1.0
1.5
2.0
0.0
0.2
0.4
0.6
0.8
1.0
1.2
CpG o/e ratio

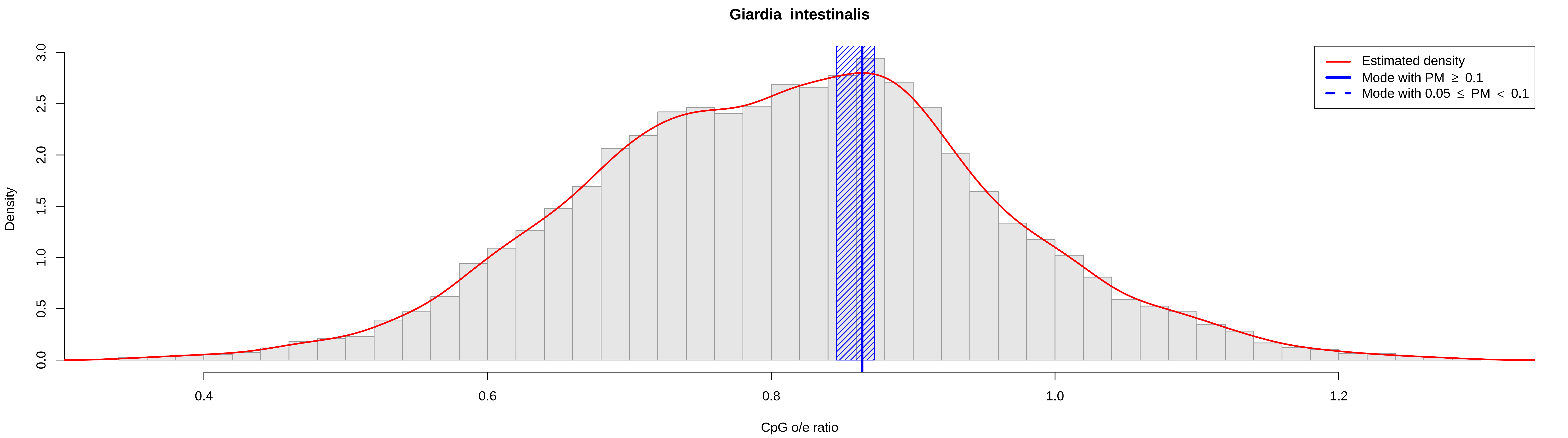

Giardia_intestinalis
Estimated density
Mode with PM ≥ 0.1
Mode with 0.05 ≤ PM < 0.1
Density
0.0
0.5
1.0
1.5
2.0
2.5
3.0
0.4
0.6
0.8
1.0
1.2
CpG o/e ratio

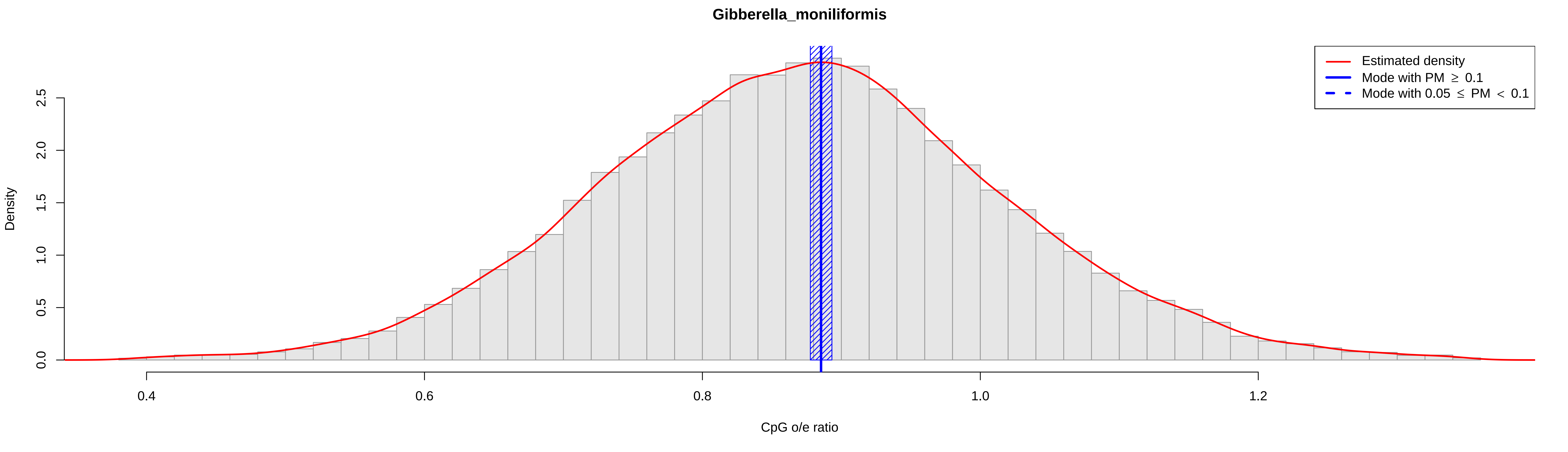

Gibberella_moniliformis
Estimated density
Mode with PM ≥ 0.1
Mode with 0.05 ≤ PM < 0.1
Density
0.0
0.5
1.0
1.5
2.0
2.5
0.4
0.6
0.8
1.0
1.2
CpG o/e ratio

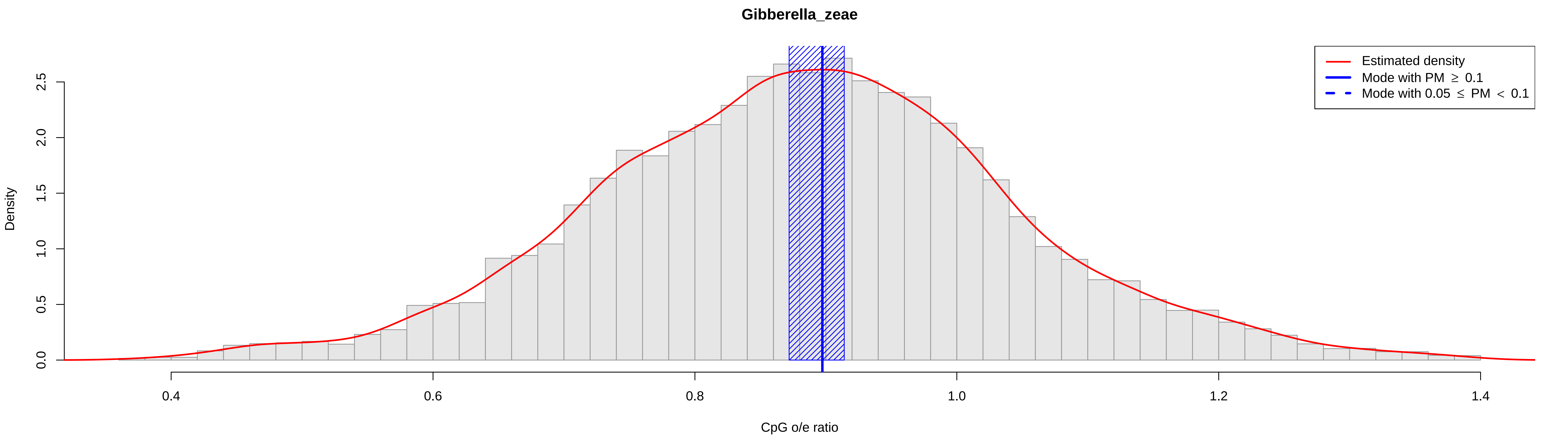
Gibberella_zeae
Estimated density
Mode with PM ≥ 0.1
Mode with 0.05 ≤ PM < 0.1
Density
0.0
0.5
1.0
1.5
2.0
2.5
0.4
0.6
0.8
1.0
1.2
1.4
CpG o/e ratio

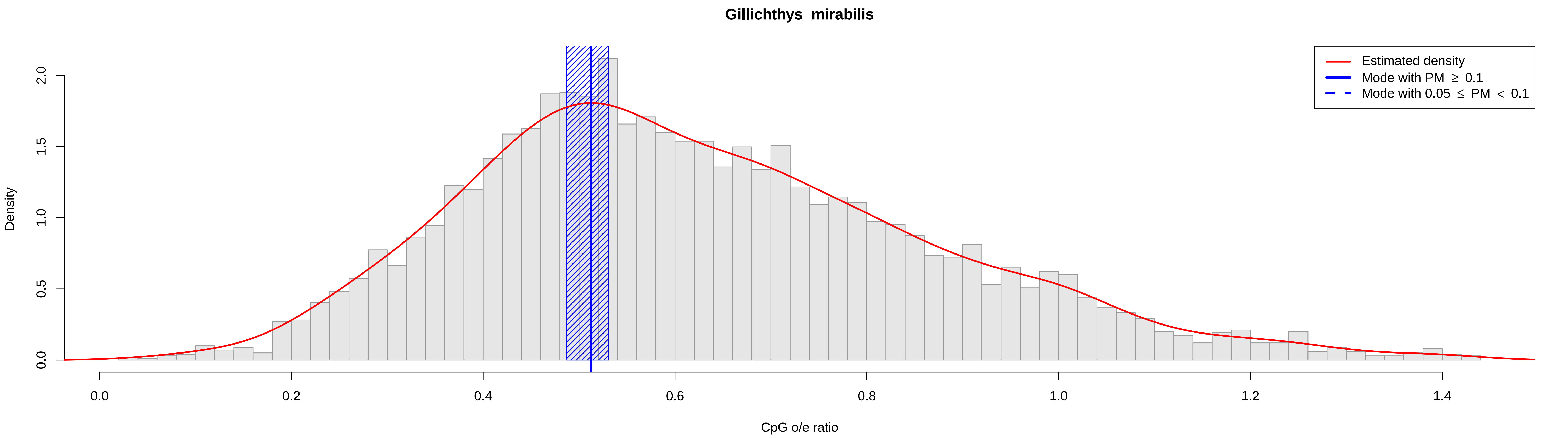
Gillichthys_mirabilis
Estimated density
Mode with PM ≥ 0.1
Mode with 0.05 ≤ PM < 0.1
Density
0.0
0.5
1.0
1.5
2.0
0.0
0.2
0.4
0.6
0.8
1.0
1.2
1.4
CpG o/e ratio

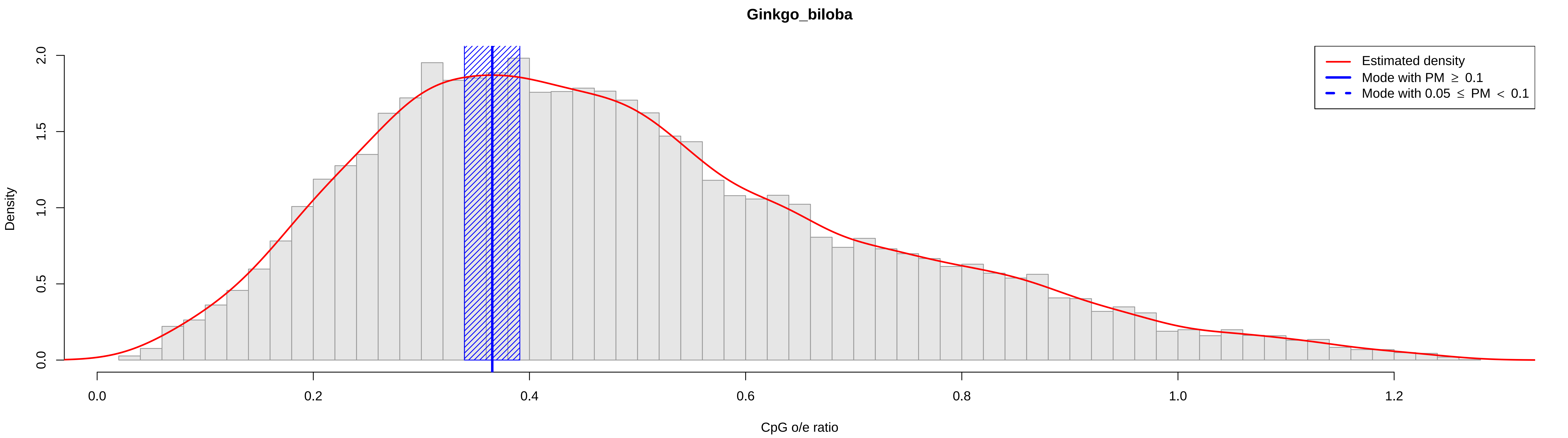

Ginkgo_biloba
Estimated density
Mode with PM ≥ 0.1
Mode with 0.05 ≤ PM < 0.1
Density
0.0
0.5
1.0
1.5
2.0
0.0
0.2
0.4
0.6
0.8
1.0
1.2
CpG o/e ratio

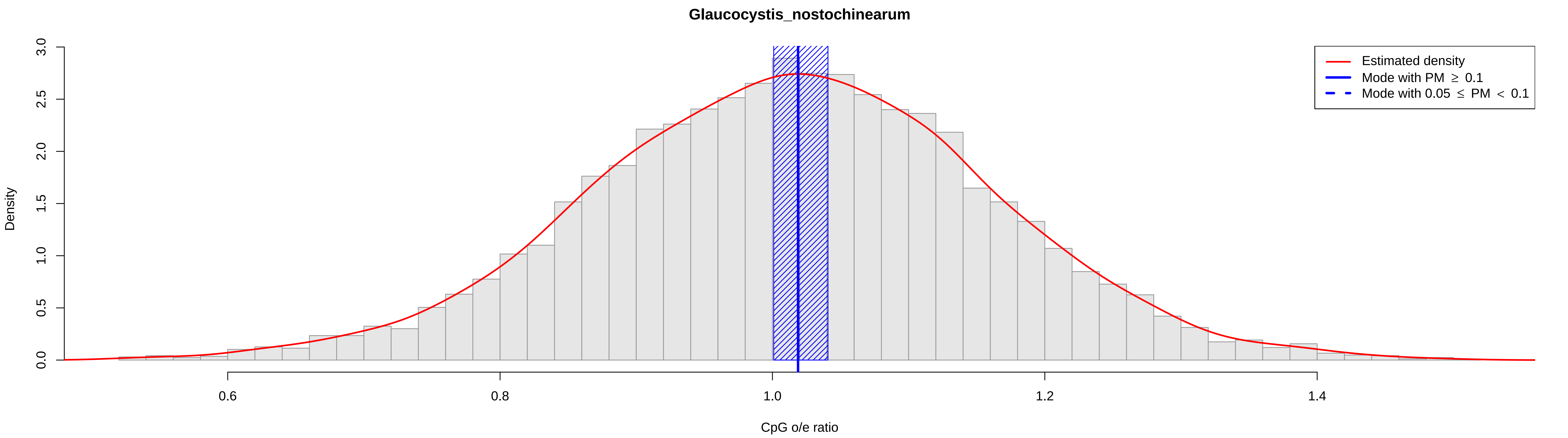
Glaucocystis_nostochinearum
Density
CpG o/e ratio
0.0
0.5
1.0
1.5
2.0
2.5
3.0
0.6
0.8
1.0
1.2
1.4
Estimated density
Mode with PM ≥ 0.1
Mode with 0.05 ≤ PM < 0.1

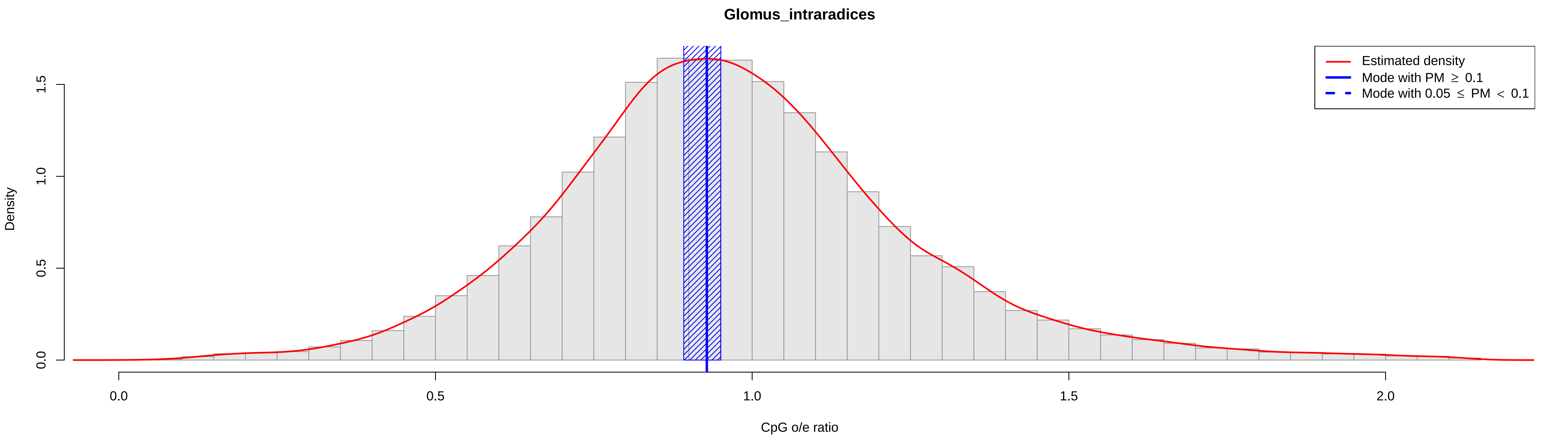

Glomus_intraradices
Estimated density
Mode with PM ≥ 0.1
Mode with 0.05 ≤ PM < 0.1
Density
0.0
0.5
1.0
1.5
0.0
0.5
1.0
1.5
2.0
CpG o/e ratio

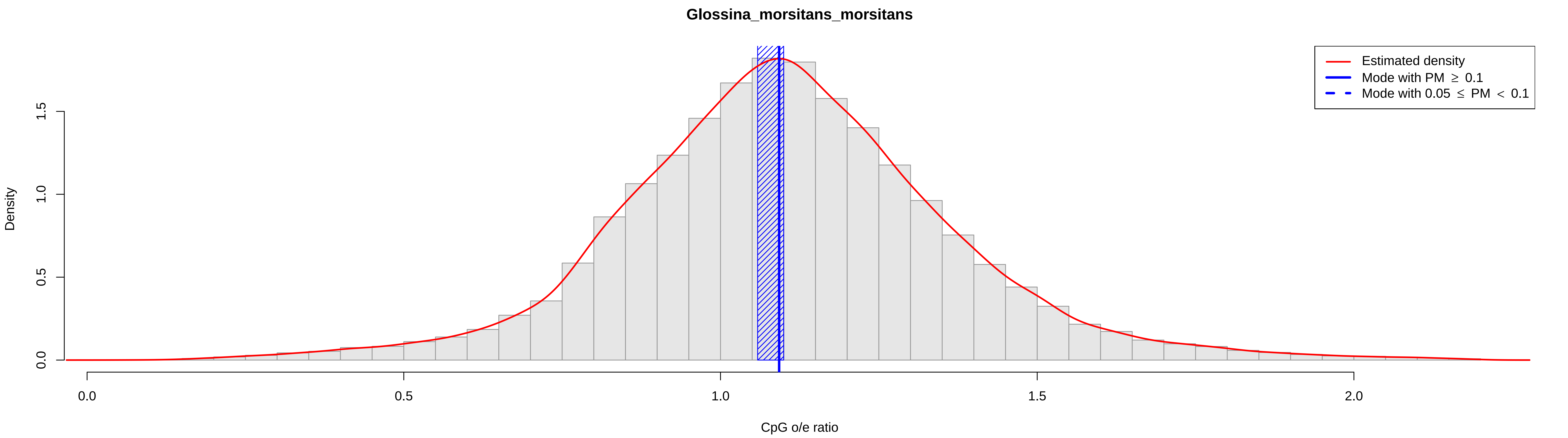
Glossina_morsitans_morsitans
Estimated density
Mode with PM ≥ 0.1
Mode with 0.05 ≤ PM < 0.1
Density
0.0
0.5
1.0
1.5
0.0
0.5
1.0
1.5
2.0
CpG o/e ratio

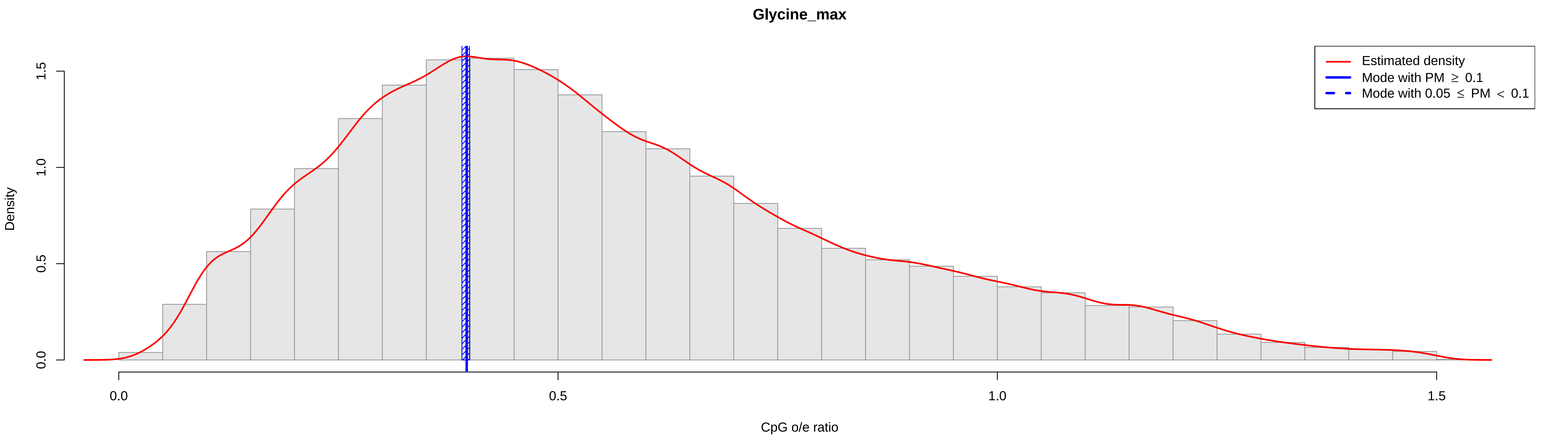

Glycine_max
Estimated density
Mode with PM ≥ 0.1
Mode with 0.05 ≤ PM < 0.1
Density
0.0
0.5
1.0
1.5
CpG o/e ratio

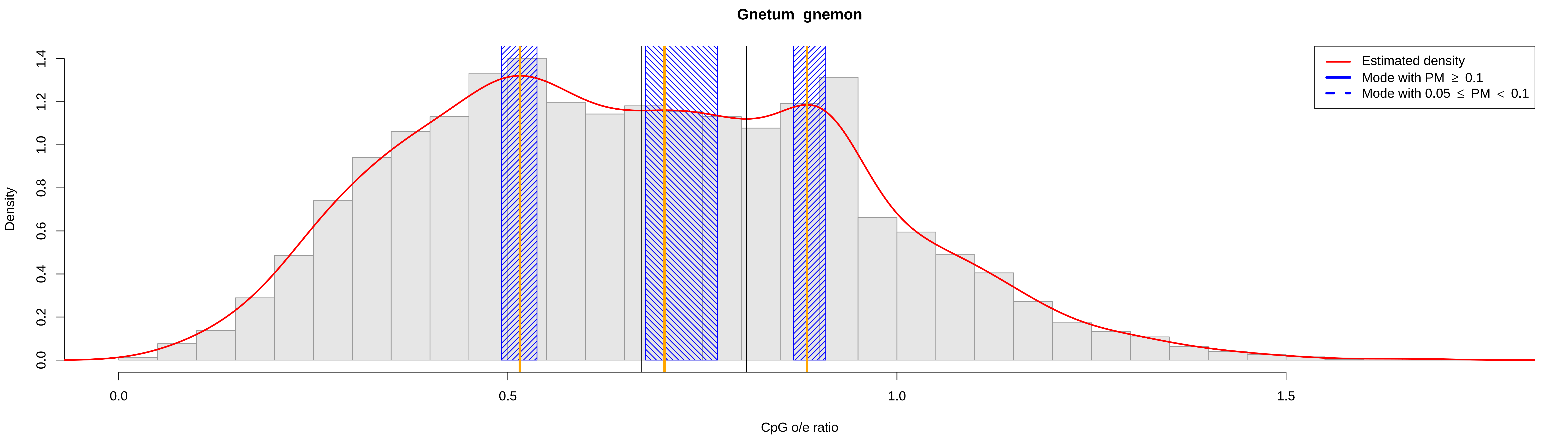

Gnetum_gnemon
Estimated density
Mode with PM ≥ 0.1
Mode with 0.05 ≤ PM < 0.1
Density
CpG o/e ratio
0.0
0.2
0.4
0.6
0.8
1.0
1.2
1.4
0.0
0.5
1.0
1.5

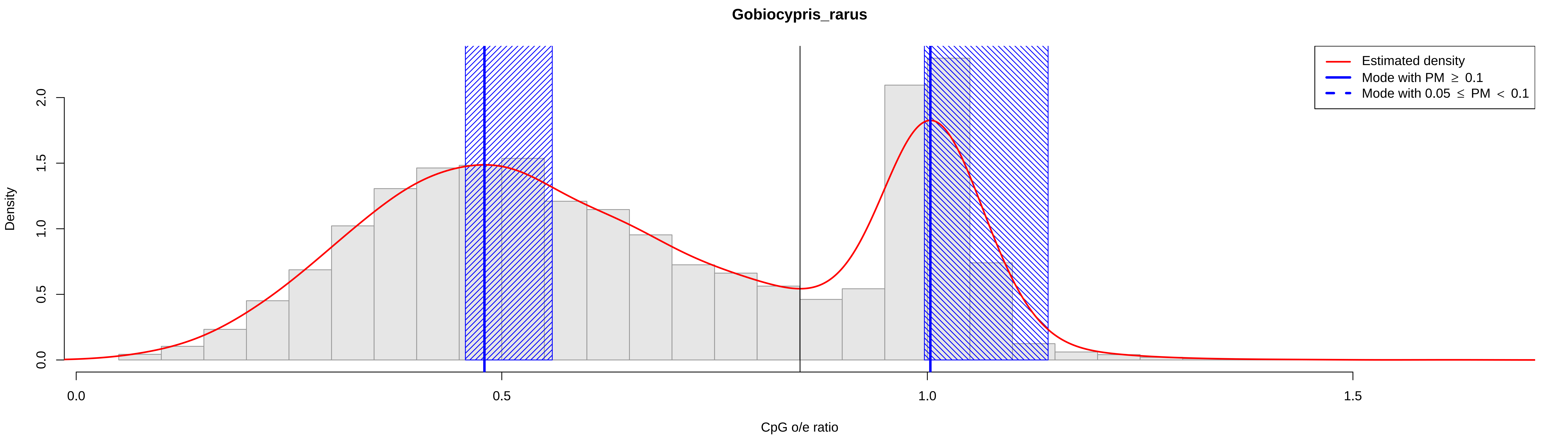
Gobiocypris_rarus
Estimated density
Mode with PM ≥ 0.1
Mode with 0.05 ≤ PM < 0.1
Density
0.0
0.5
1.0
1.5
2.0
0.0
0.5
1.0
1.5
CpG o/e ratio

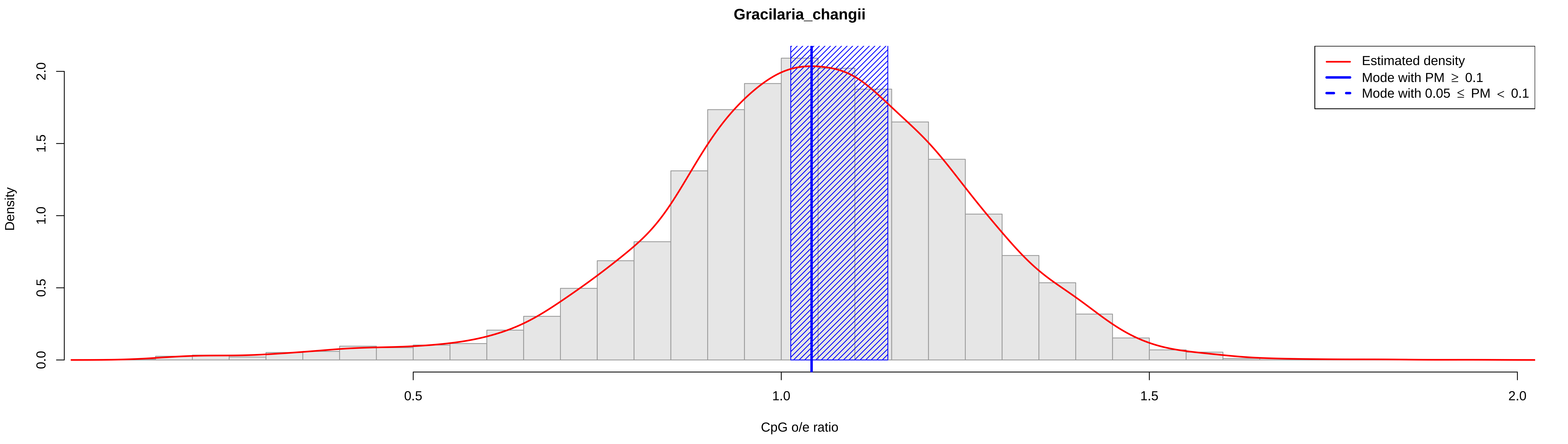

Gracilaria_changii
Estimated density
Mode with PM ≥ 0.1
Mode with 0.05 ≤ PM < 0.1
Density
0.0
0.5
1.0
1.5
2.0
0.5
1.0
1.5
2.0
CpG o/e ratio

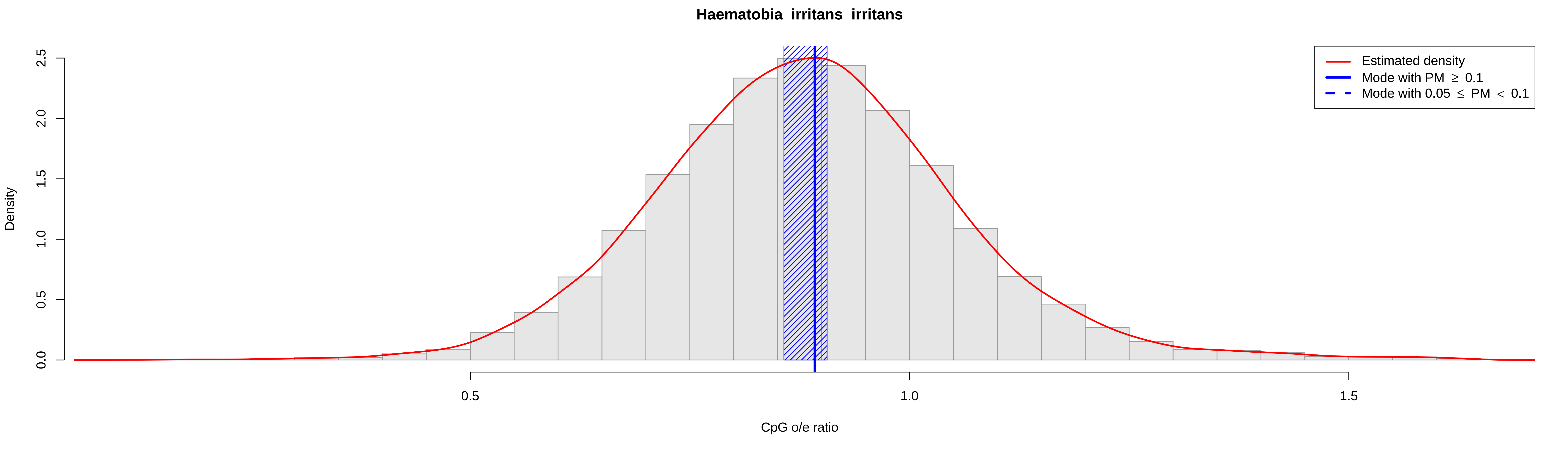

Haematobia_irritans_irritans
Estimated density
Mode with PM ≥ 0.1
Mode with 0.05 ≤ PM < 0.1
Density
0.0
0.5
1.0
1.5
2.0
2.5
0.5
1.0
1.5
CpG o/e ratio

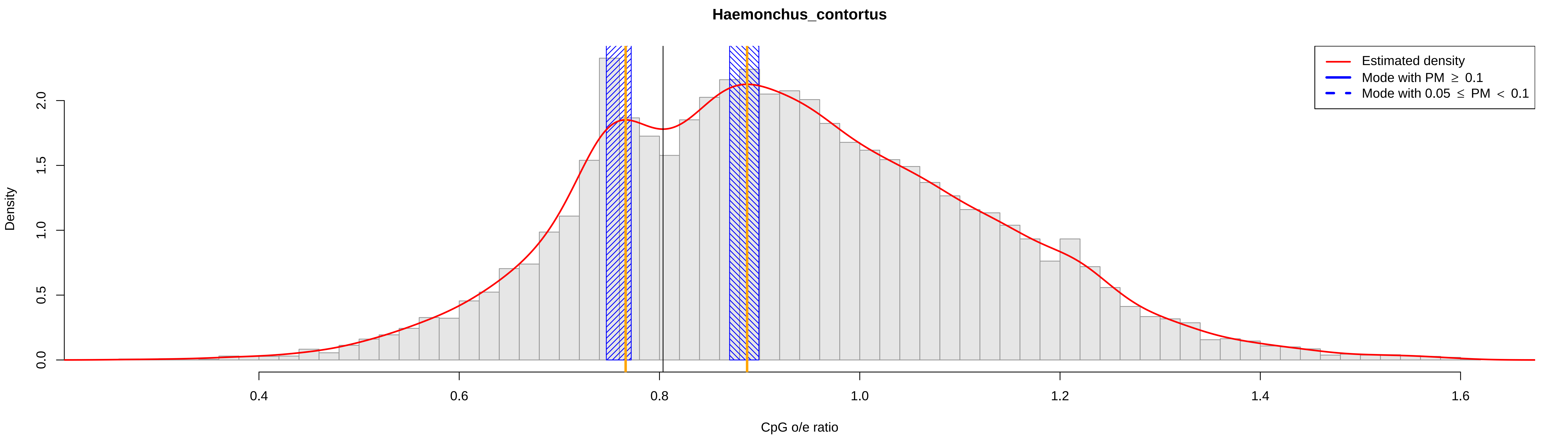
Haemonchus_contortus
Estimated density
Mode with PM ≥ 0.1
Mode with 0.05 ≤ PM < 0.1
Density
0.0
0.5
1.0
1.5
2.0
0.4
0.6
0.8
1.0
1.2
1.4
1.6
CpG o/e ratio

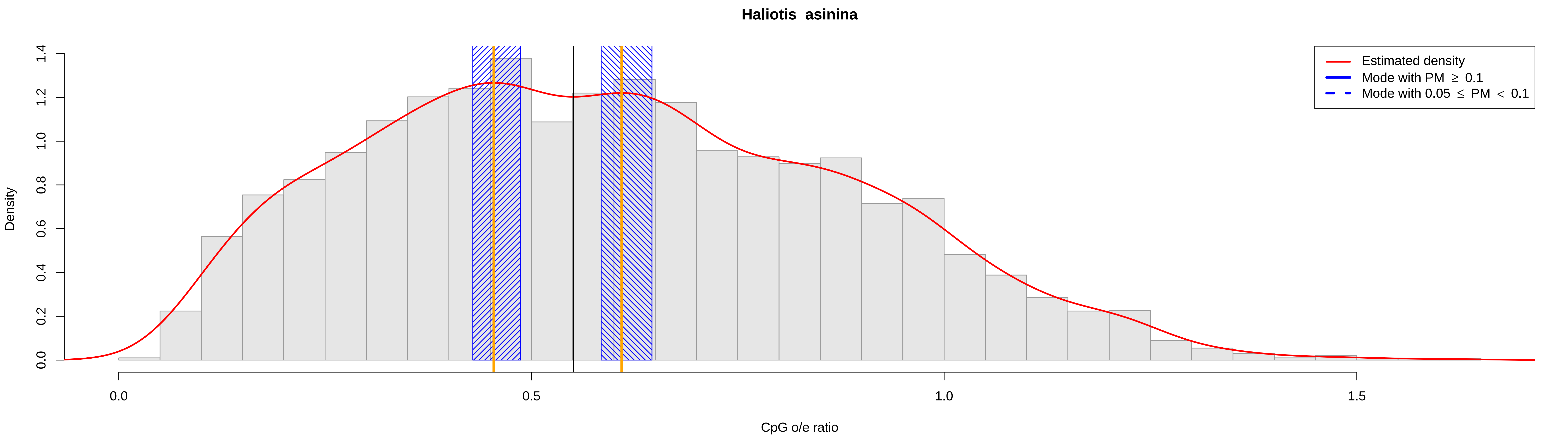

Haliotis_asinina
Estimated density
Mode with PM ≥ 0.1
Mode with 0.05 ≤ PM < 0.1
Density
0.0
0.2
0.4
0.6
0.8
1.0
1.2
1.4
0.0
0.5
1.0
1.5
CpG o/e ratio

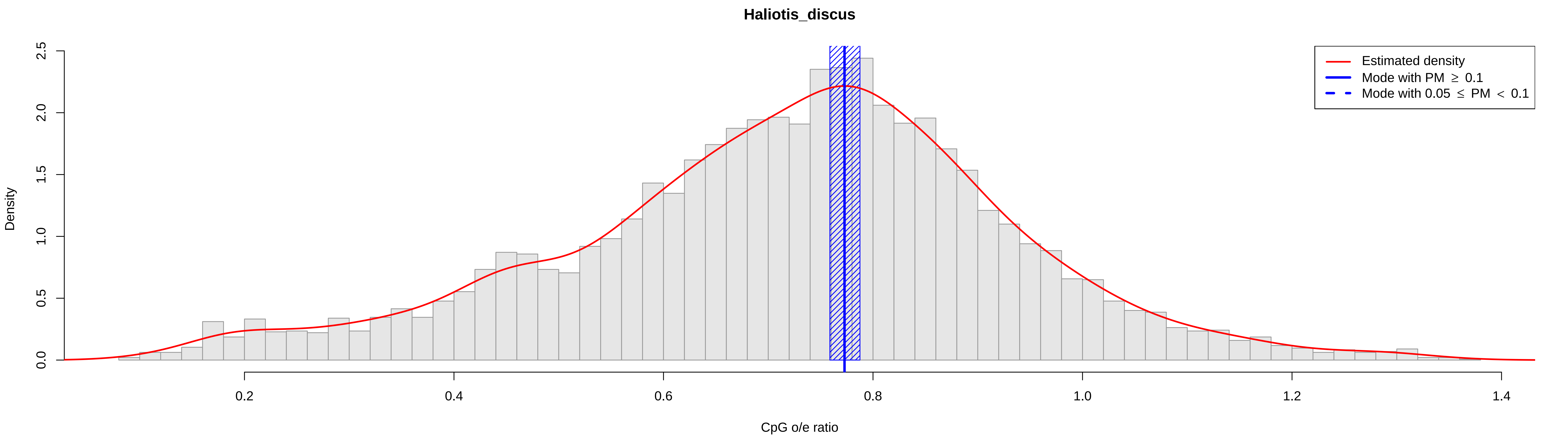
Haliotis_discus
Estimated density
Mode with PM ≥ 0.1
Mode with 0.05 ≤ PM < 0.1
Density
0.0
0.5
1.0
1.5
2.0
2.5
0.2
0.4
0.6
0.8
1.0
1.2
1.4
CpG o/e ratio

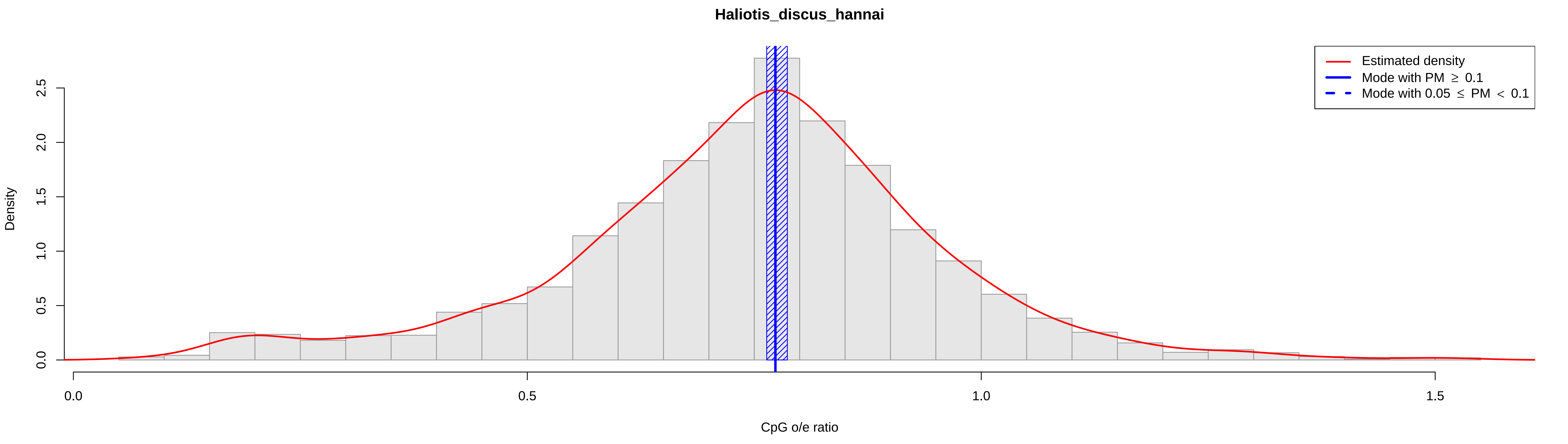

Haliotis_discus_hannai
Estimated density
Mode with PM ≥ 0.1
Mode with 0.05 ≤ PM < 0.1
Density
0.0
0.5
1.0
1.5
2.0
2.5
0.0
0.5
1.0
1.5
CpG o/e ratio

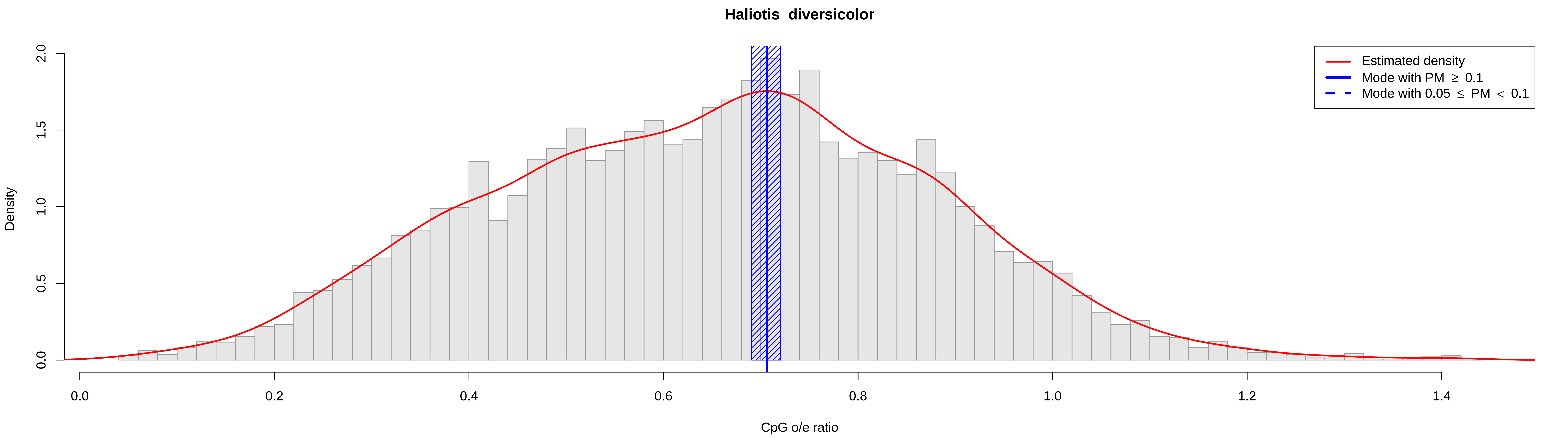

Haliotis_diversicolor
Estimated density
Mode with PM ≥ 0.1
Mode with 0.05 ≤ PM < 0.1
Density
0.0
0.5
1.0
1.5
2.0
0.0
0.2
0.4
0.6
0.8
1.0
1.2
1.4
CpG o/e ratio

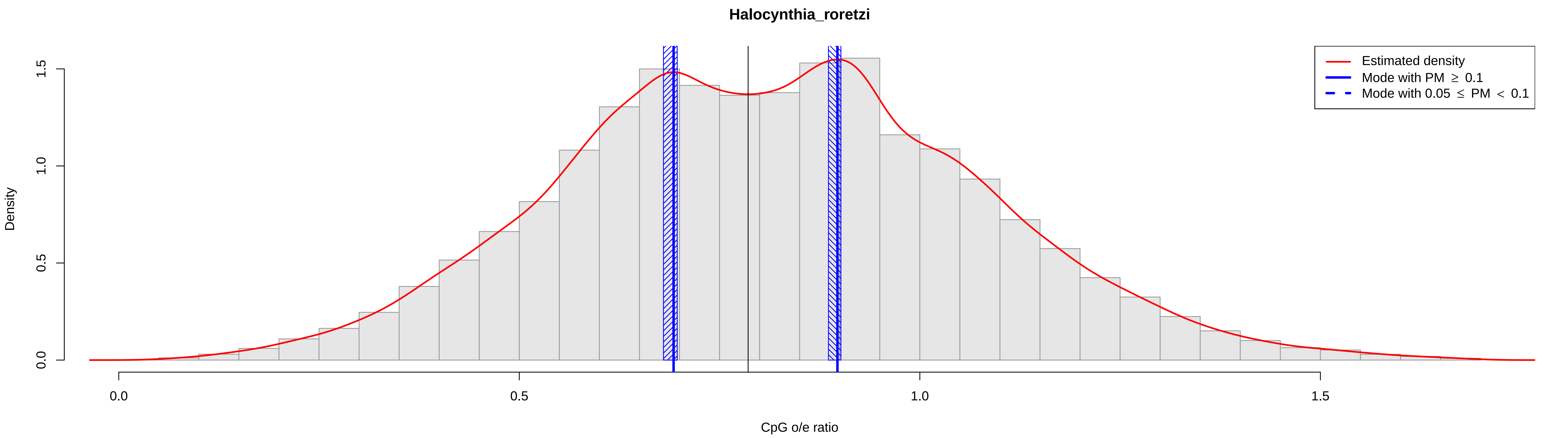
Halocynthia_roretzi
Estimated density
Mode with PM ≥ 0.1
Mode with 0.05 ≤ PM < 0.1
Density
0.0
0.5
1.0
1.5
CpG o/e ratio
0.0
0.5
1.0
1.5

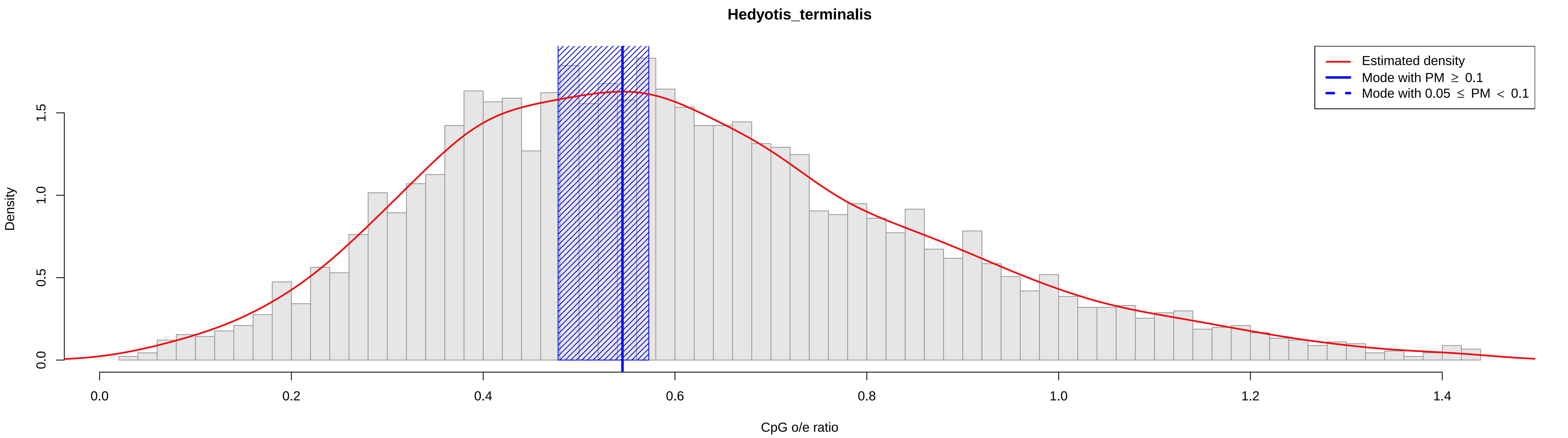

Hedyotis_terminalis
Estimated density
Mode with PM ≥ 0.1
Mode with 0.05 ≤ PM < 0.1
Density
0.0
0.5
1.0
1.5
0.0
0.2
0.4
0.6
0.8
1.0
1.2
1.4
CpG o/e ratio

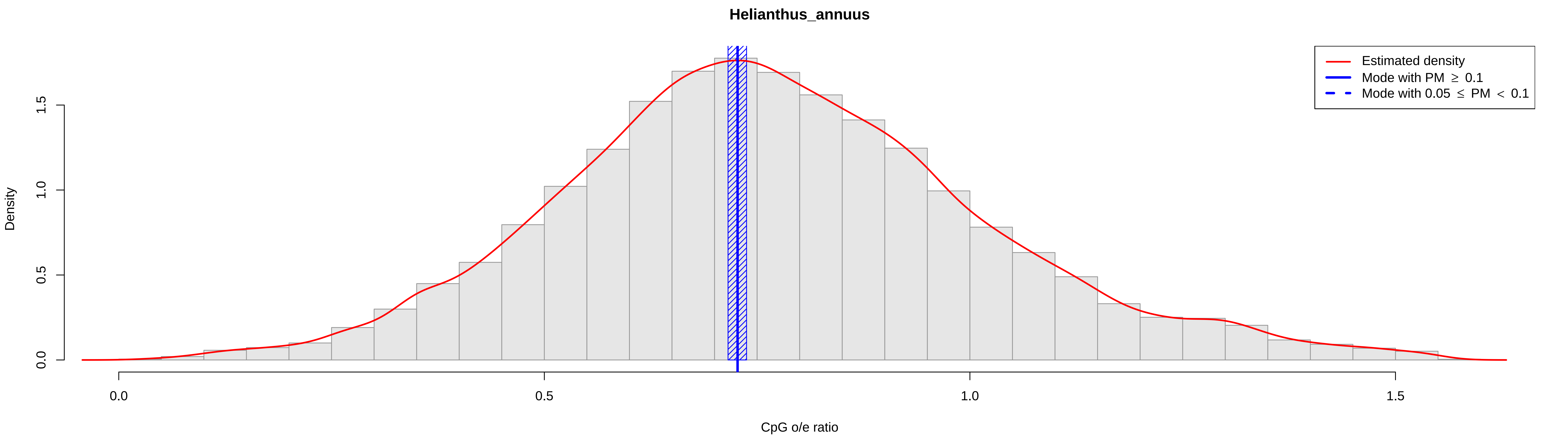

Helianthus_annuus
Estimated density
Mode with PM ≥ 0.1
Mode with 0.05 ≤ PM < 0.1
Density
0.0
0.5
1.0
1.5
0.0
0.5
1.0
1.5
CpG o/e ratio

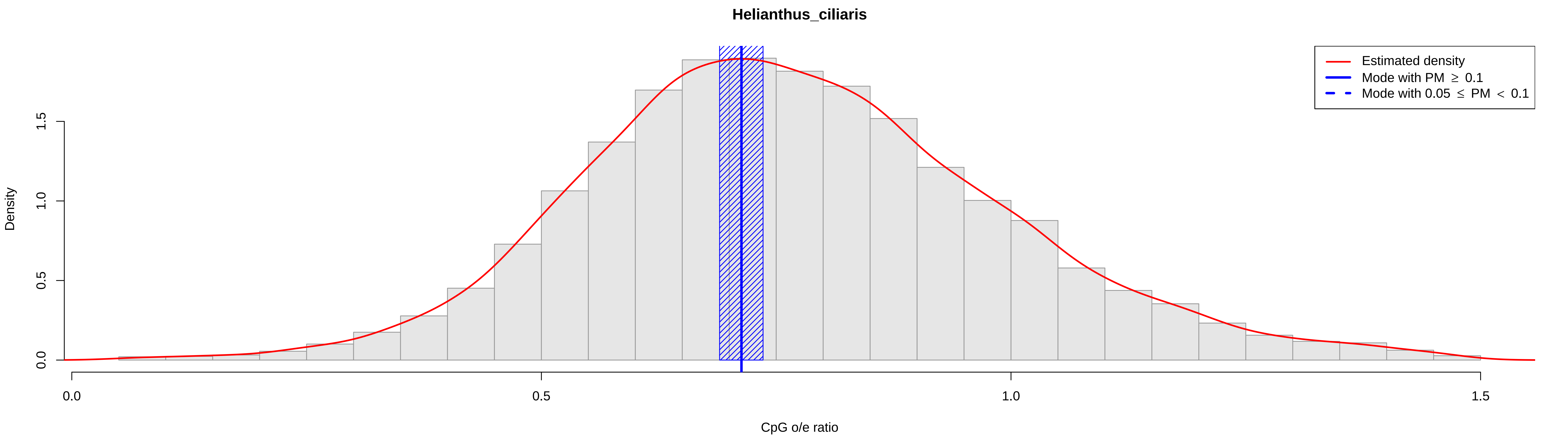

Helianthus_ciliaris
Estimated density
Mode with PM ≥ 0.1
Mode with 0.05 ≤ PM < 0.1
Density
0.0
0.5
1.0
1.5
0.0
0.5
1.0
1.5
CpG o/e ratio

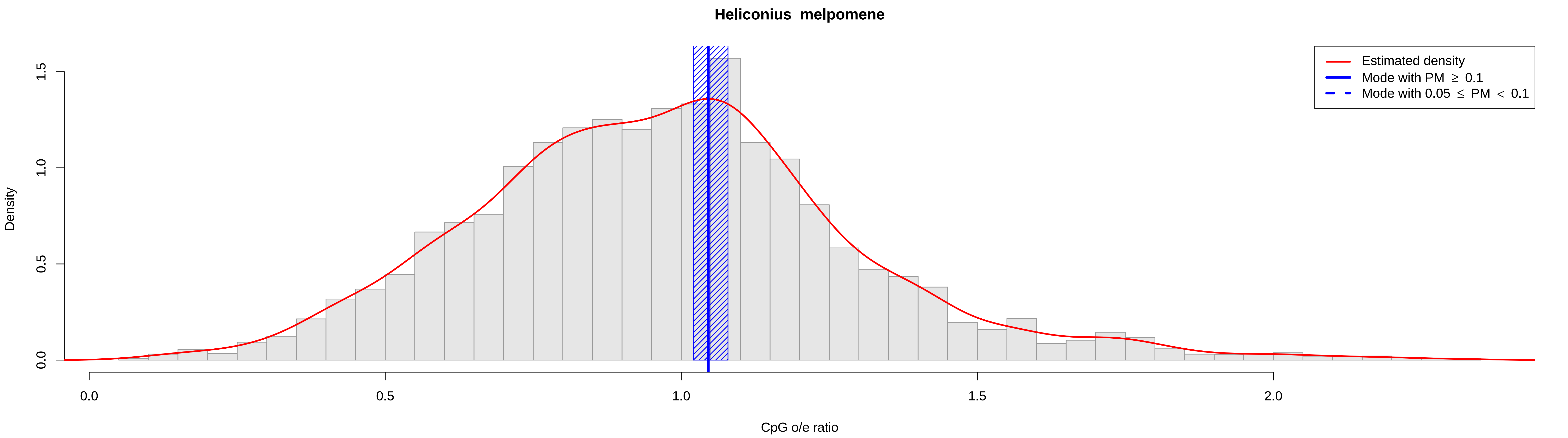

Heliconius_melpomene
Estimated density
Mode with PM ≥ 0.1
Mode with 0.05 ≤ PM < 0.1
Density
0.0
0.5
1.0
1.5
0.0
0.5
1.0
1.5
2.0
CpG o/e ratio

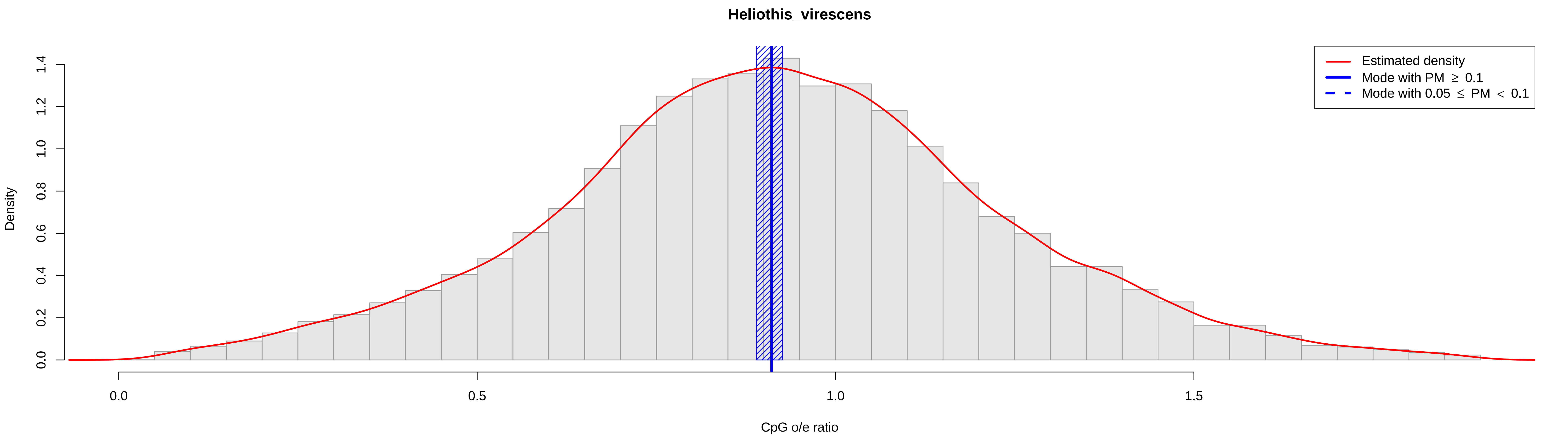
Heliothis_virescens
Estimated density
Mode with PM ≥ 0.1
Mode with 0.05 ≤ PM < 0.1
Density
0.0
0.2
0.4
0.6
0.8
1.0
1.2
1.4
0.0
0.5
1.0
1.5
CpG o/e ratio

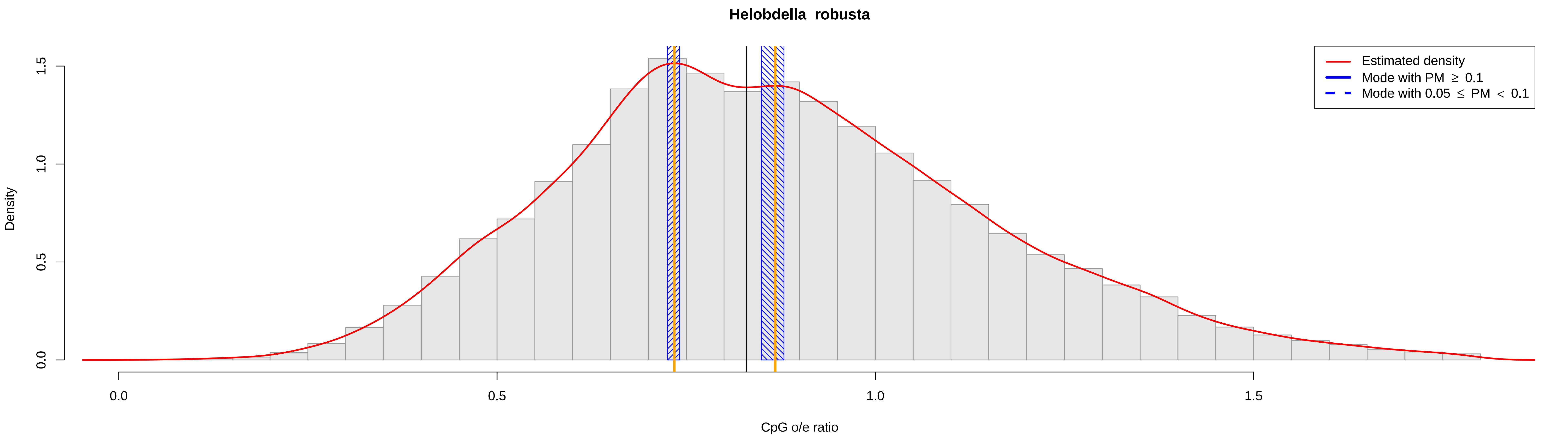

Helobdella_robusta
Estimated density
Mode with PM ≥ 0.1
Mode with 0.05 ≤ PM < 0.1
Density
0.0
0.5
1.0
1.5
CpG o/e ratio

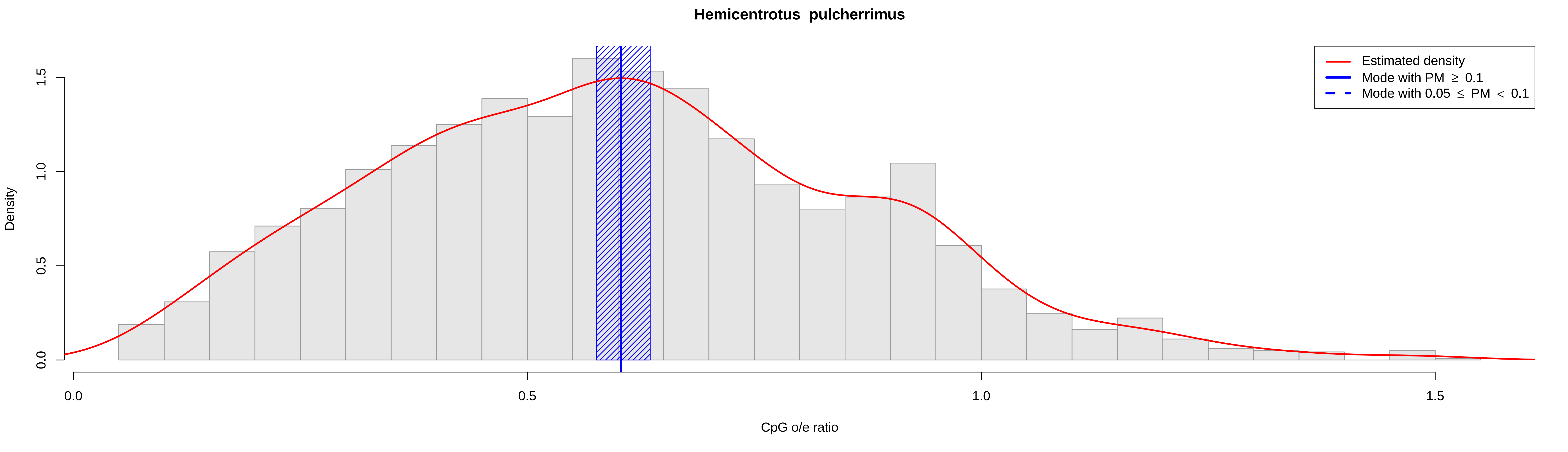
Hemicentrotus_pulcherrimus
Estimated density
Mode with PM ≥ 0.1
Mode with 0.05 ≤ PM < 0.1
Density
0.0
0.5
1.0
1.5
0.0
0.5
1.0
1.5
CpG o/e ratio

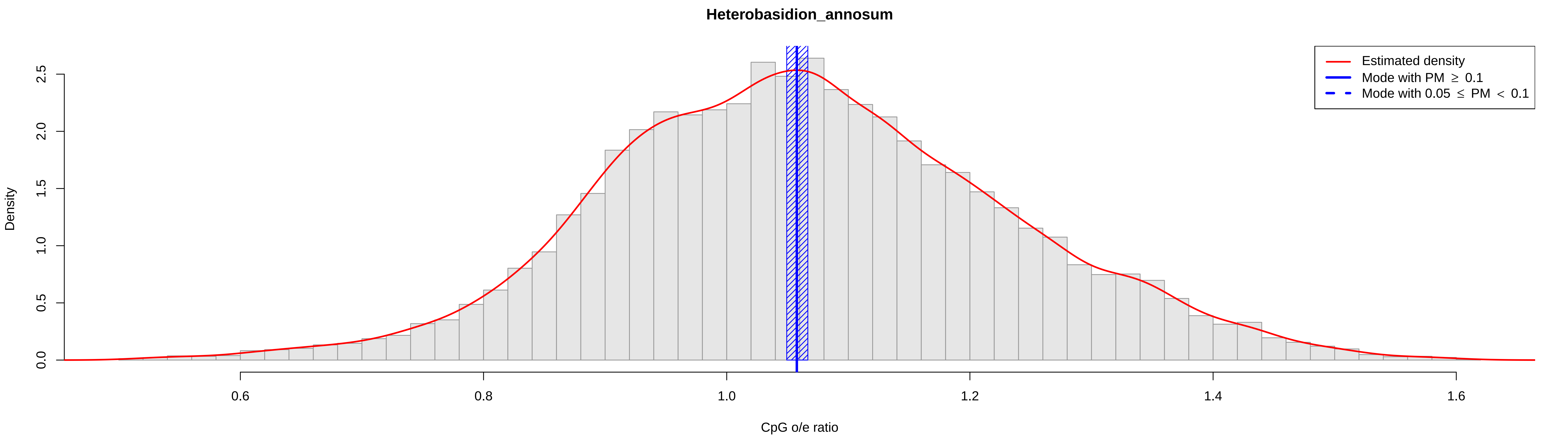

Heterobasidion_annosum
Estimated density
Mode with PM ≥ 0.1
Mode with 0.05 ≤ PM < 0.1
Density
0.0
0.5
1.0
1.5
2.0
2.5
0.6
0.8
1.0
1.2
1.4
1.6
CpG o/e ratio

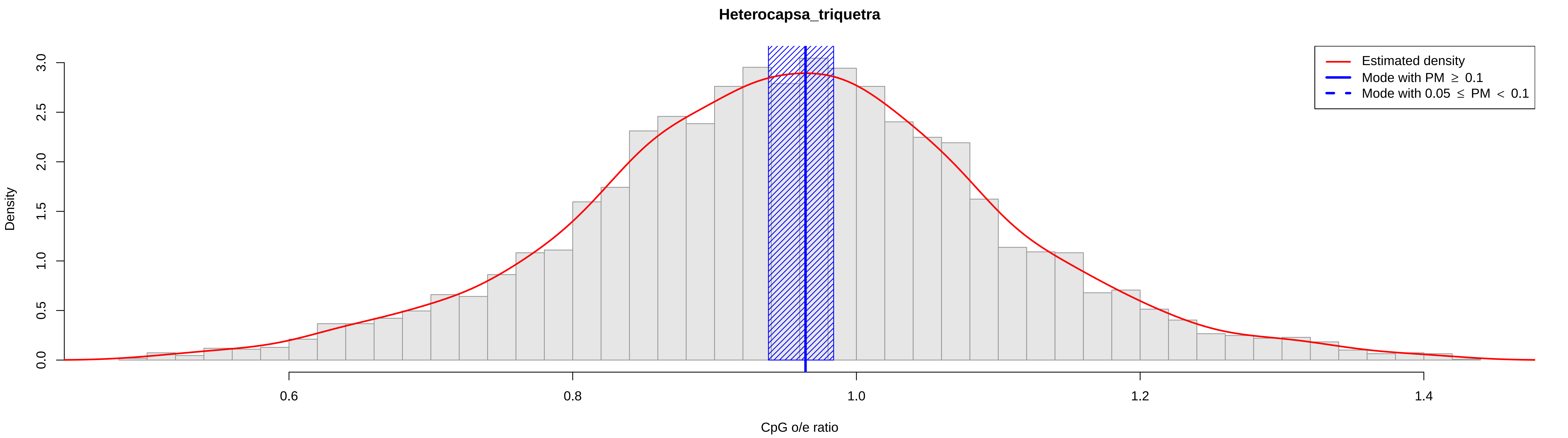

Heterocapsa_triquetra
Estimated density
Mode with PM ≥ 0.1
Mode with 0.05 ≤ PM < 0.1
Density
0.0
0.5
1.0
1.5
2.0
2.5
3.0
0.6
0.8
1.0
1.2
1.4
CpG o/e ratio

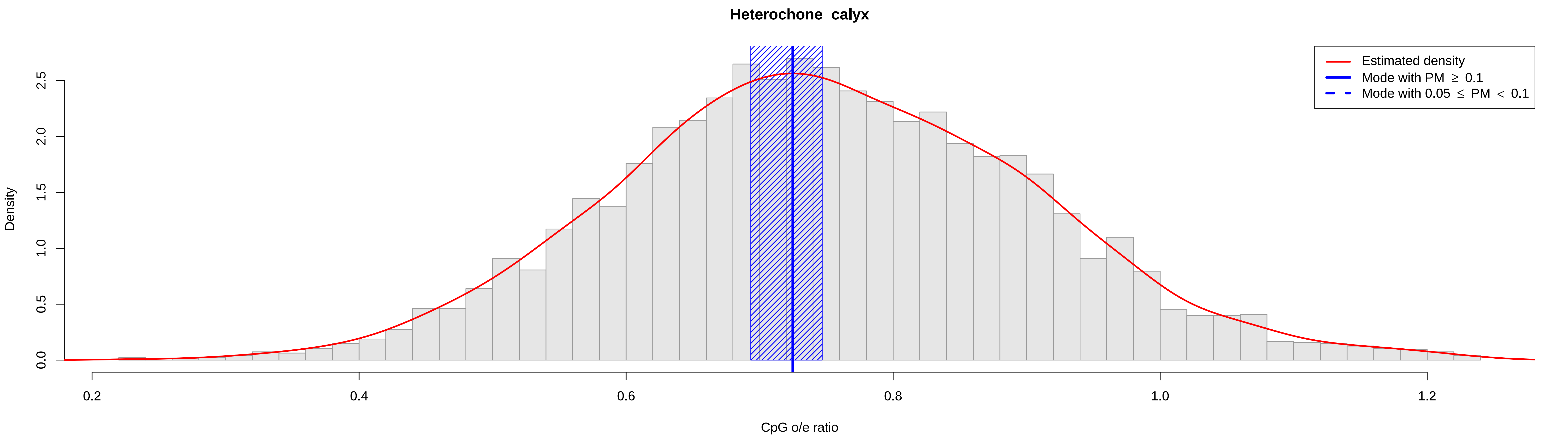

Heterochone_calyx
Estimated density
Mode with PM ≥ 0.1
Mode with 0.05 ≤ PM < 0.1
Density
0.0
0.5
1.0
1.5
2.0
2.5
0.2
0.4
0.6
0.8
1.0
1.2
CpG o/e ratio

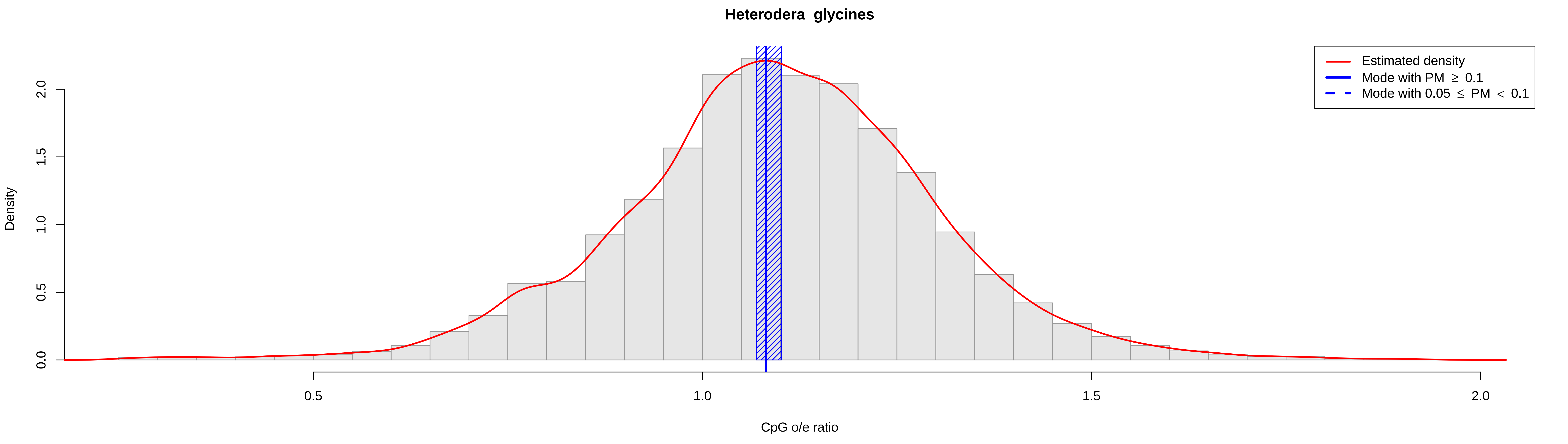

Heterodera_glycines
Estimated density
Mode with PM ≥ 0.1
Mode with 0.05 ≤ PM < 0.1
Density
0.0
0.5
1.0
1.5
2.0
0.5
1.0
1.5
2.0
CpG o/e ratio

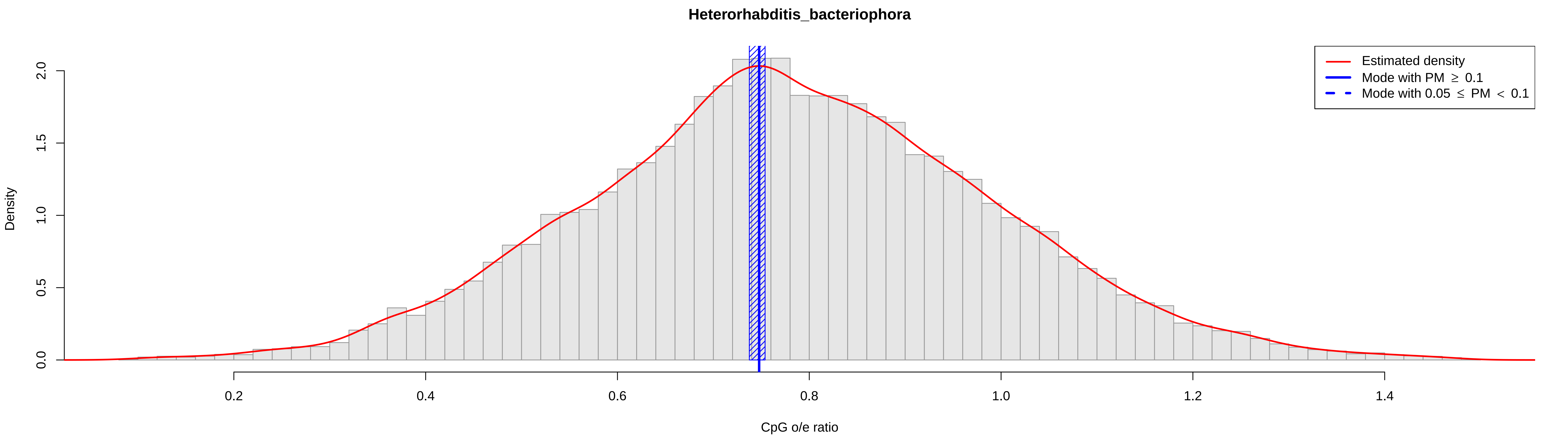
Heterorhabditis_bacteriophora
Estimated density
Mode with PM ≥ 0.1
Mode with 0.05 ≤ PM < 0.1
Density
0.0
0.5
1.0
1.5
2.0
0.2
0.4
0.6
0.8
1.0
1.2
1.4
CpG o/e ratio

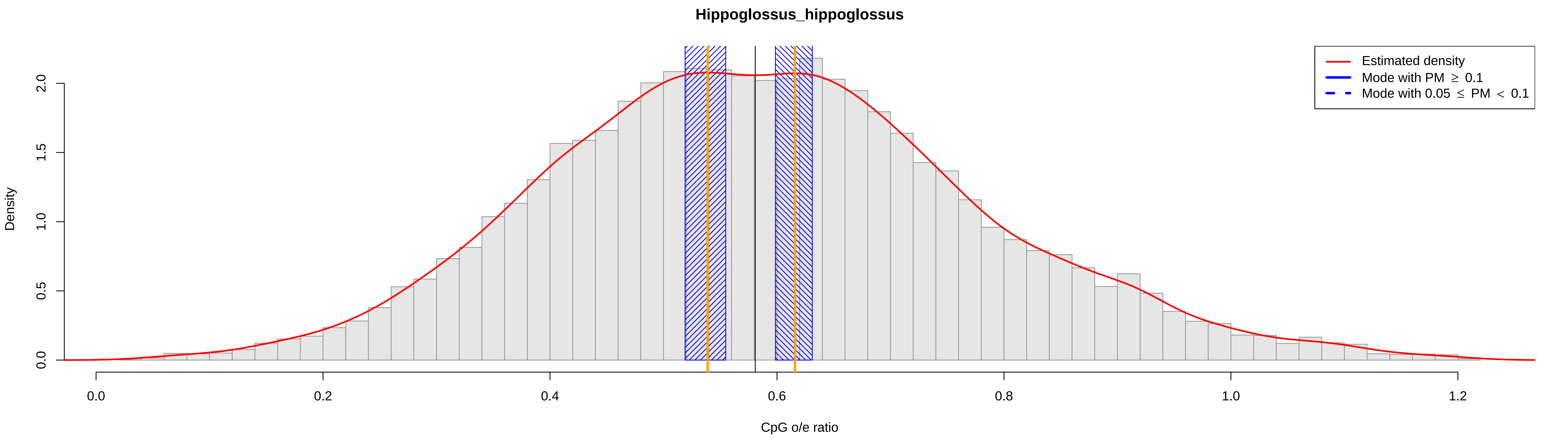

Hippoglossus_hippoglossus
Estimated density
Mode with PM ≥ 0.1
Mode with 0.05 ≤ PM < 0.1
Density
0.0
0.5
1.0
1.5
2.0
0.0
0.2
0.4
0.6
0.8
1.0
1.2
CpG o/e ratio

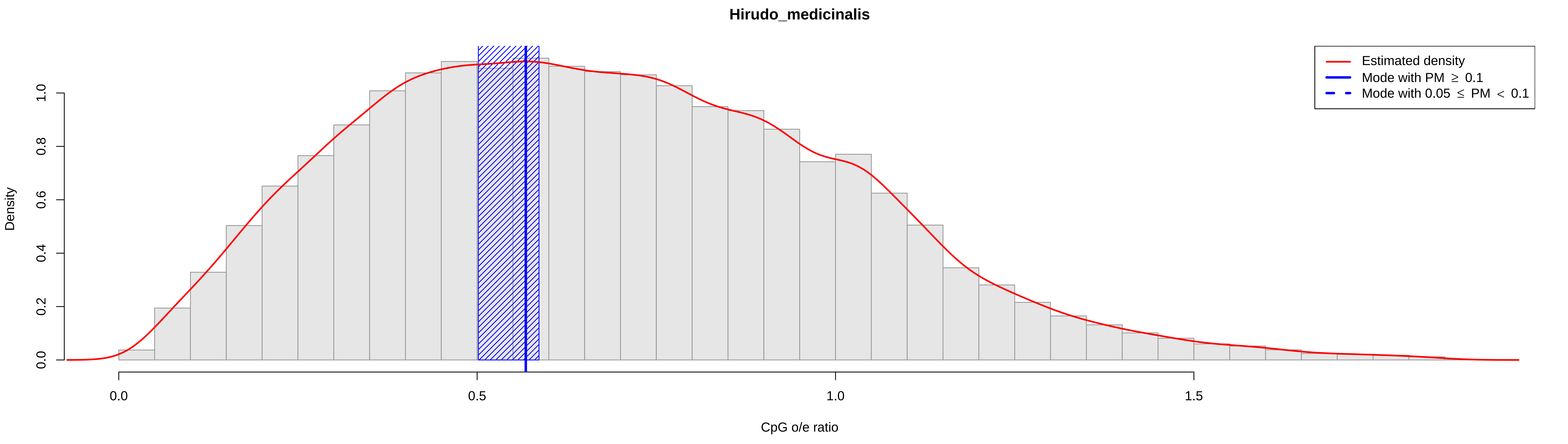

Hirudo_medicinalis
Estimated density
Mode with PM ≥ 0.1
Mode with 0.05 ≤ PM < 0.1
Density
0.0
0.2
0.4
0.6
0.8
1.0
0.0
0.5
1.0
1.5
CpG o/e ratio

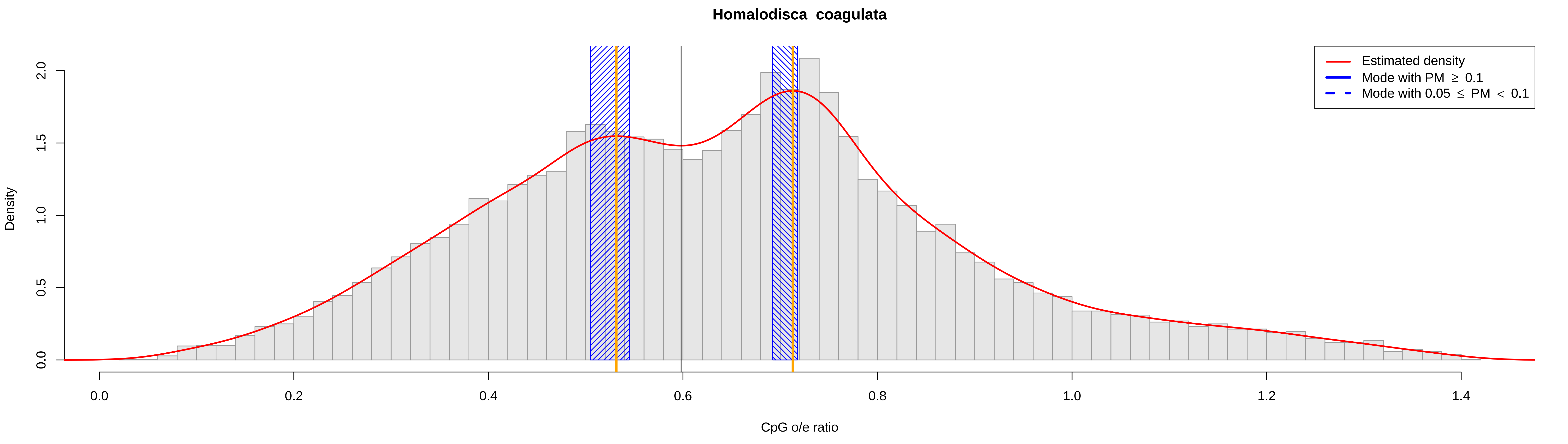
Homalodisca_coagulata
Estimated density
Mode with PM ≥ 0.1
Mode with 0.05 ≤ PM < 0.1
Density
2.0
1.5
1.0
0.5
0.0
0.0
0.2
0.4
0.6
0.8
1.0
1.2
1.4
CpG o/e ratio

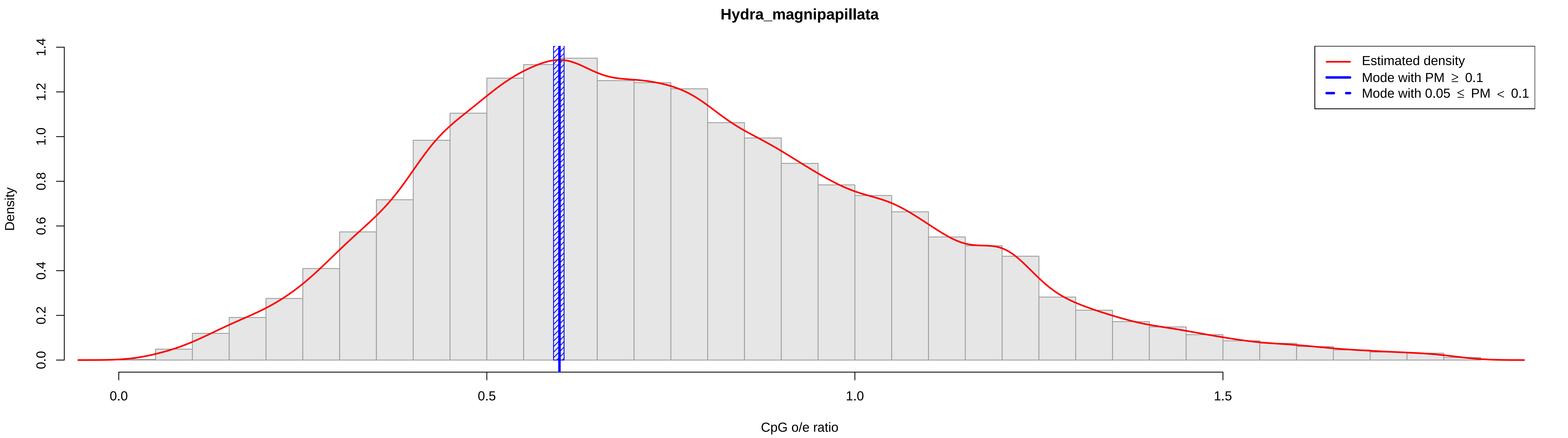
Hydra_magnipapillata
Estimated density
Mode with PM ≥ 0.1
Mode with 0.05 ≤ PM < 0.1
Density
0.0
0.2
0.4
0.6
0.8
1.0
1.2
1.4
0.0
0.5
1.0
1.5
CpG o/e ratio

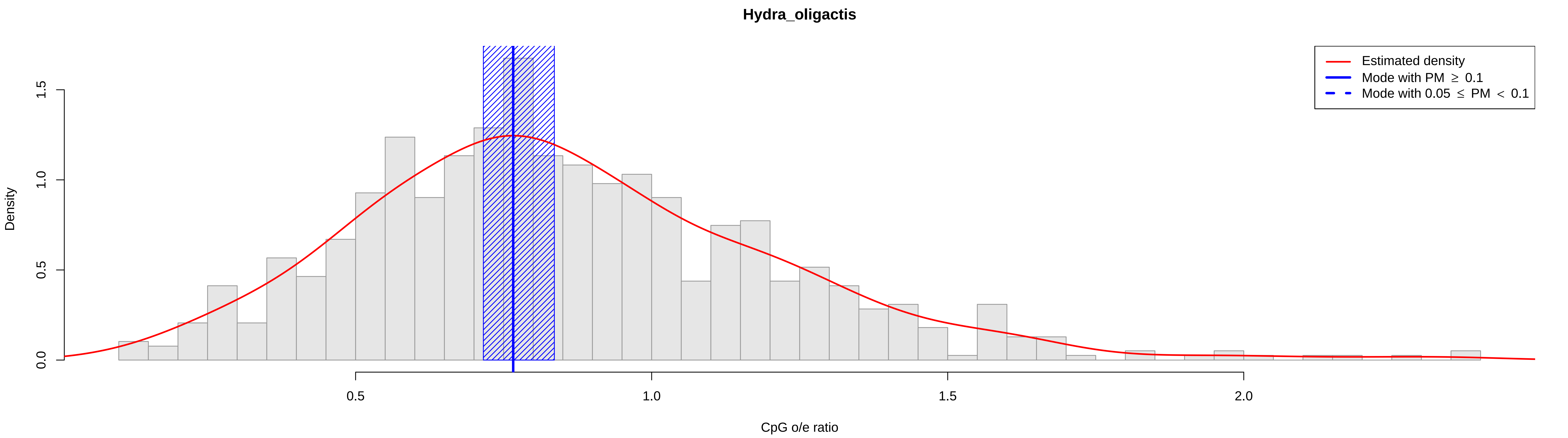

Hydra_oligactis
Estimated density
Mode with PM ≥ 0.1
Mode with 0.05 ≤ PM < 0.1
Density
0.0
0.5
1.0
1.5
0.5
1.0
1.5
2.0
CpG o/e ratio

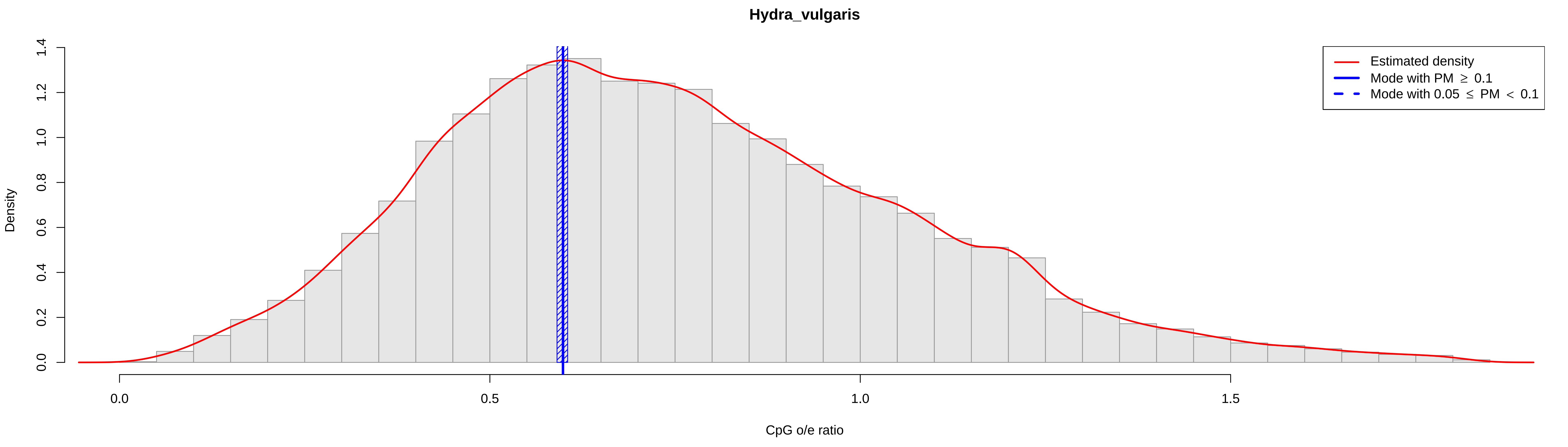
Hydra_vulgaris
Density
0.0
0.2
0.4
0.6
0.8
1.0
1.2
1.4
0.0
0.5
1.0
1.5
CpG o/e ratio
Estimated density
Mode with PM ≥ 0.1
Mode with 0.05 ≤ PM < 0.1

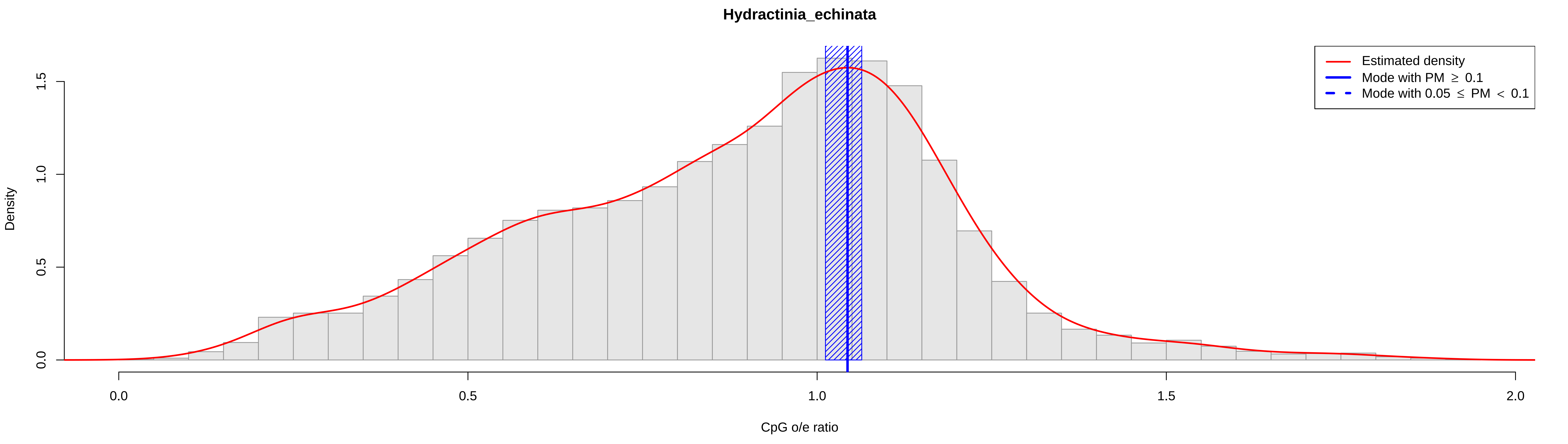

Hydractinia_echinata
Estimated density
Mode with PM ≥ 0.1
Mode with 0.05 ≤ PM < 0.1
Density
0.0
0.5
1.0
1.5
0.0
0.5
1.0
1.5
2.0
CpG o/e ratio

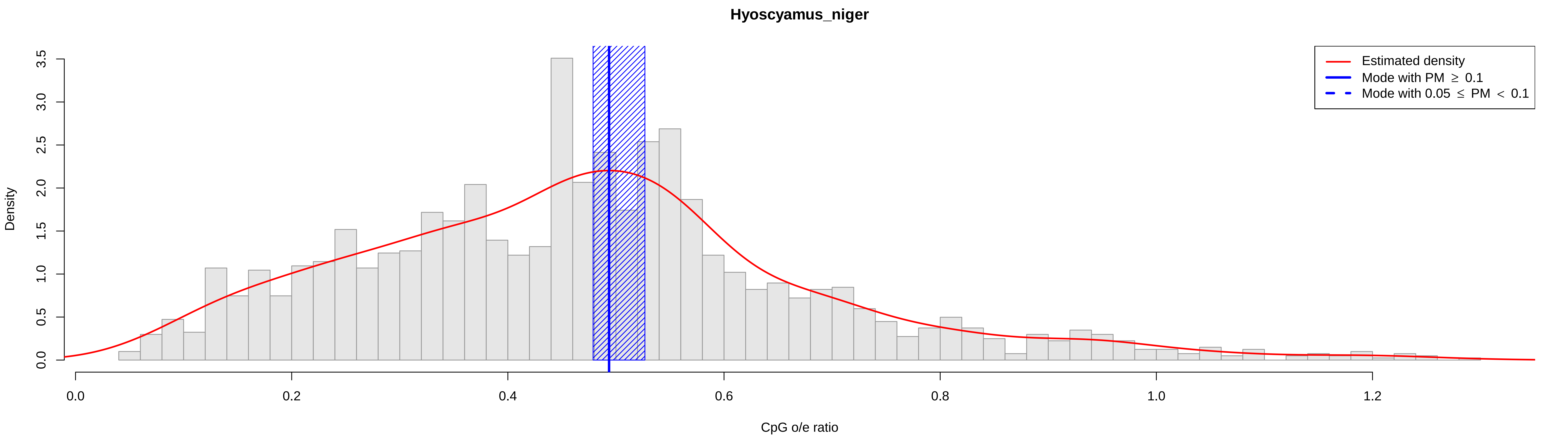

Hyoscyamus_niger
Estimated density
Mode with PM ≥ 0.1
Mode with 0.05 ≤ PM < 0.1
Density
CpG o/e ratio
0.0
0.5
1.0
1.5
2.0
2.5
3.0
3.5
0.0
0.2
0.4
0.6
0.8
1.0
1.2

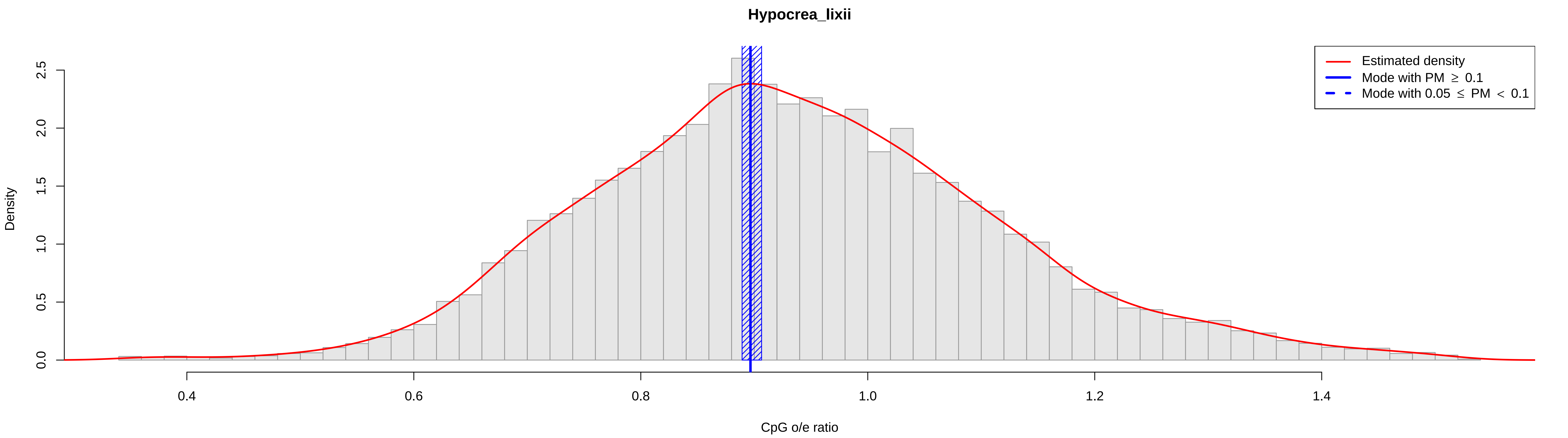

Hypocrea_lixii
Estimated density
Mode with PM ≥ 0.1
Mode with 0.05 ≤ PM < 0.1
Density
0.0
0.5
1.0
1.5
2.0
2.5
0.4
0.6
0.8
1.0
1.2
1.4
CpG o/e ratio

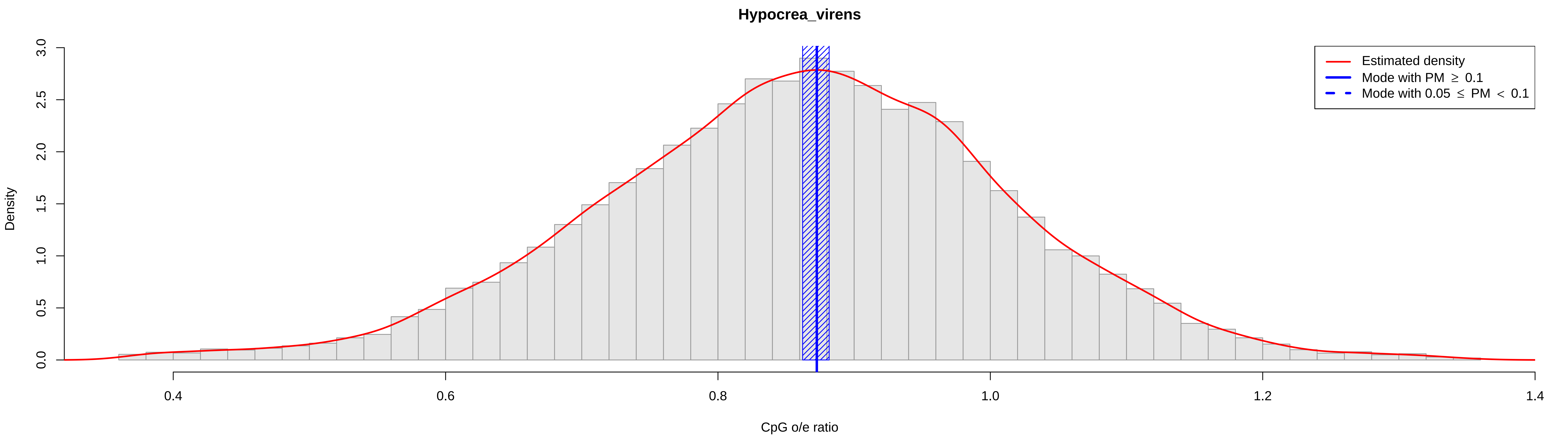

Hypocrea_virens
Estimated density
Mode with PM ≥ 0.1
Mode with 0.05 ≤ PM < 0.1
Density
0.0
0.5
1.0
1.5
2.0
2.5
3.0
0.4
0.6
0.8
1.0
1.2
1.4
CpG o/e ratio

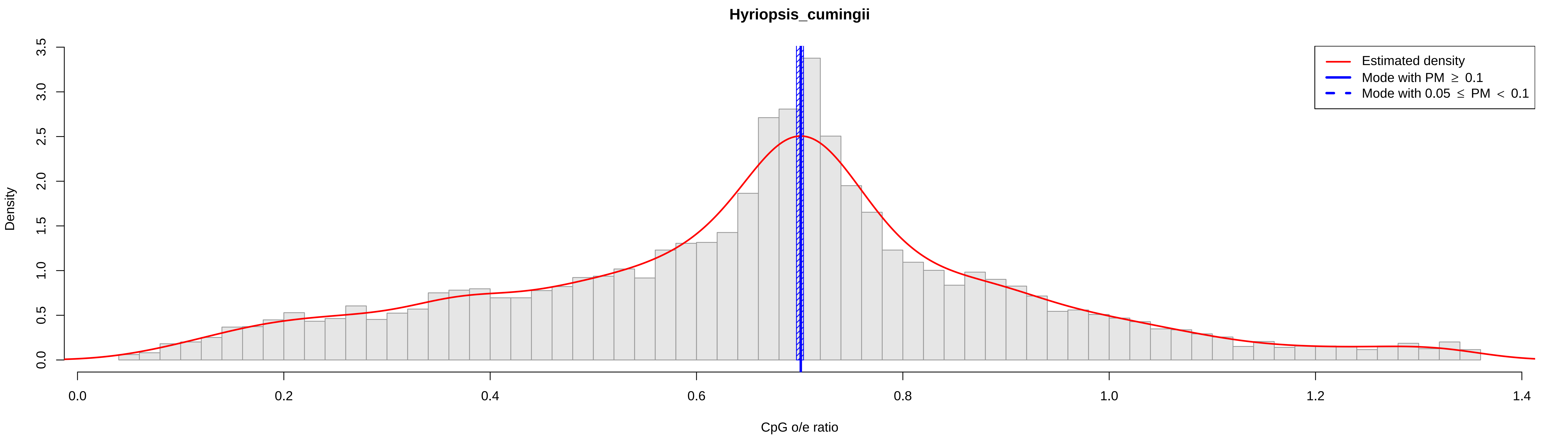
Hyriopsis_cumingii
Estimated density
Mode with PM ≥ 0.1
Mode with 0.05 ≤ PM < 0.1
Density
0.0
0.5
1.0
1.5
2.0
2.5
3.0
3.5
0.0
0.2
0.4
0.6
0.8
1.0
1.2
1.4
CpG o/e ratio

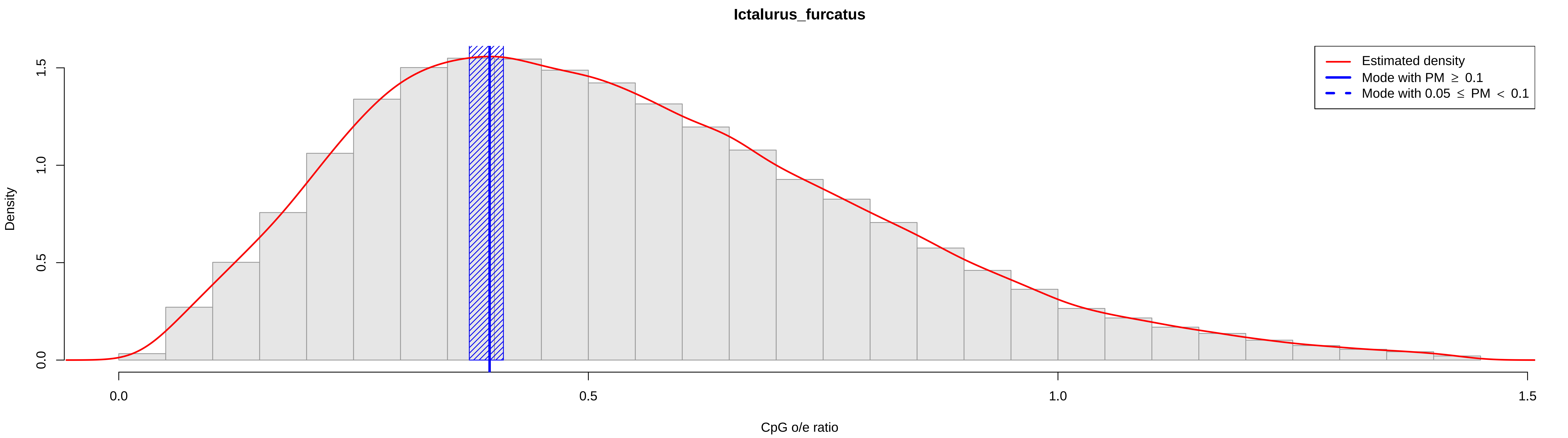
Ictalurus_furcatus
Estimated density
Mode with PM ≥ 0.1
Mode with 0.05 ≤ PM < 0.1
Density
0.0
0.5
1.0
1.5
0.0
0.5
1.0
1.5
CpG o/e ratio

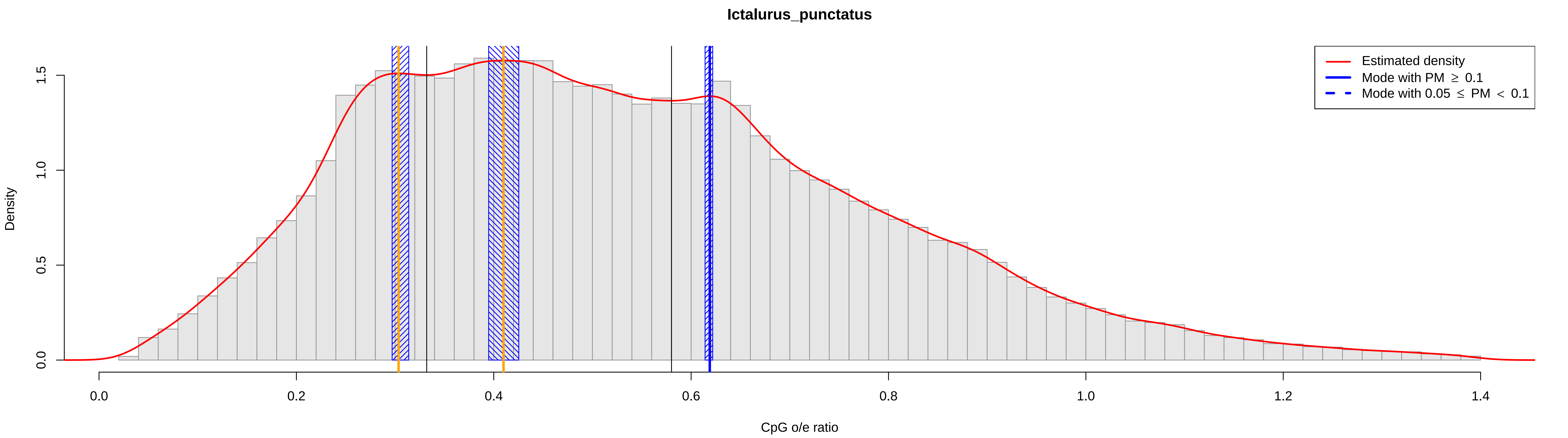
Ictalurus_punctatus
Estimated density
Mode with PM ≥ 0.1
Mode with 0.05 ≤ PM < 0.1
Density
0.0
0.5
1.0
1.5
0.0
0.2
0.4
0.6
0.8
1.0
1.2
1.4
CpG o/e ratio

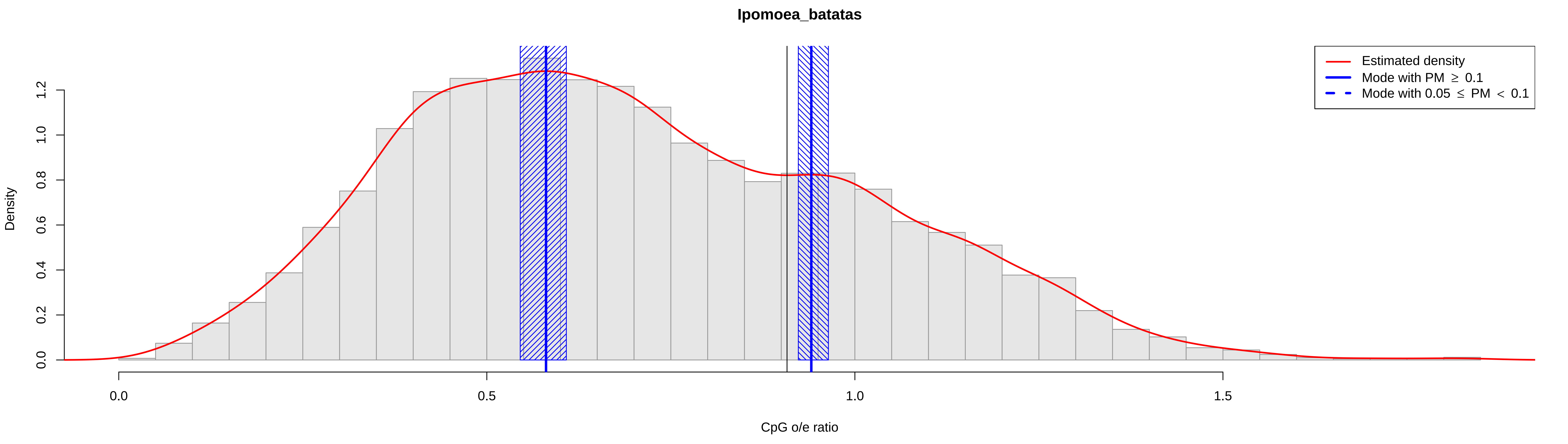

Ipomoea_batatas
Estimated density
Mode with PM ≥ 0.1
Mode with 0.05 ≤ PM < 0.1
Density
0.0
0.2
0.4
0.6
0.8
1.0
1.2
0.0
0.5
1.0
1.5
CpG o/e ratio

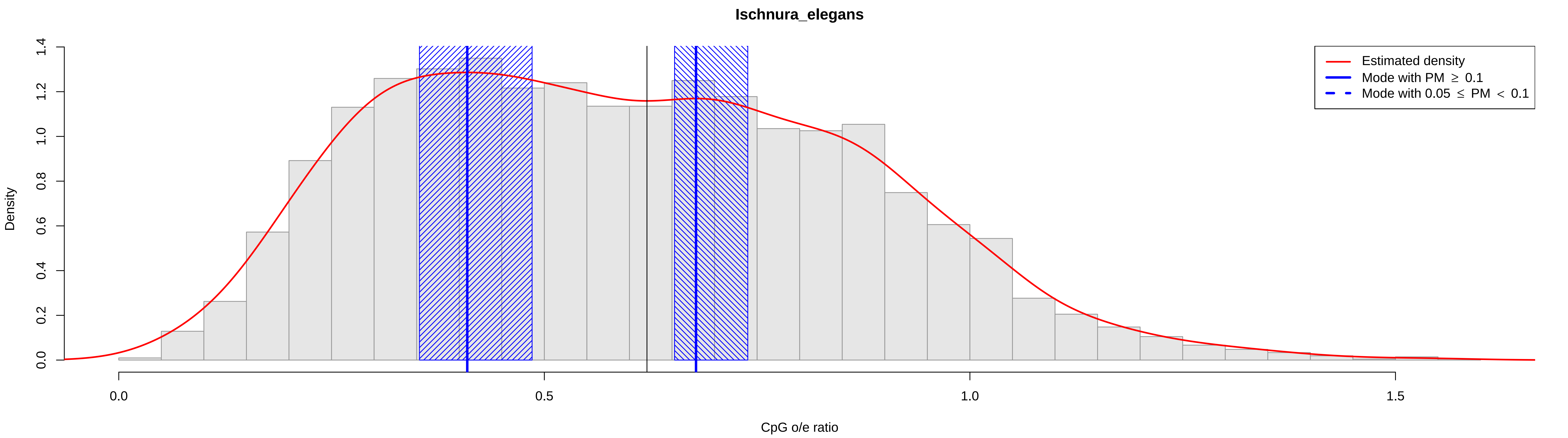

Ischnura_elegans
Estimated density
Mode with PM ≥ 0.1
Mode with 0.05 ≤ PM < 0.1
Density
CpG o/e ratio
0.0
0.2
0.4
0.6
0.8
1.0
1.2
1.4
0.0
0.5
1.0
1.5

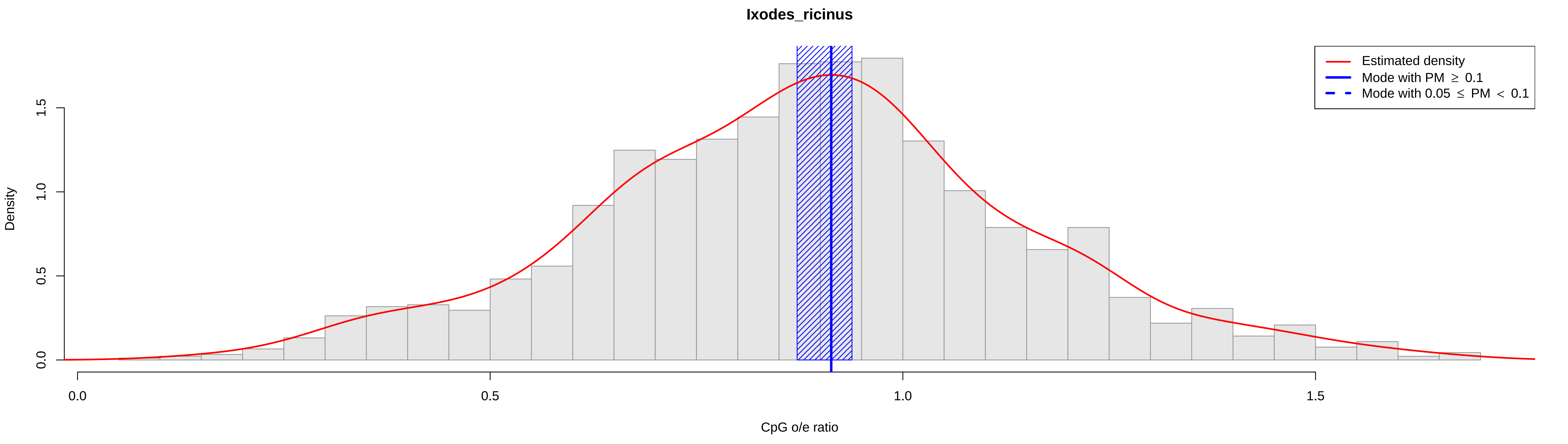

Ixodes_ricinus
Estimated density
Mode with PM ≥ 0.1
Mode with 0.05 ≤ PM < 0.1
Density
0.0
0.5
1.0
1.5
0.0
0.5
1.0
1.5
CpG o/e ratio

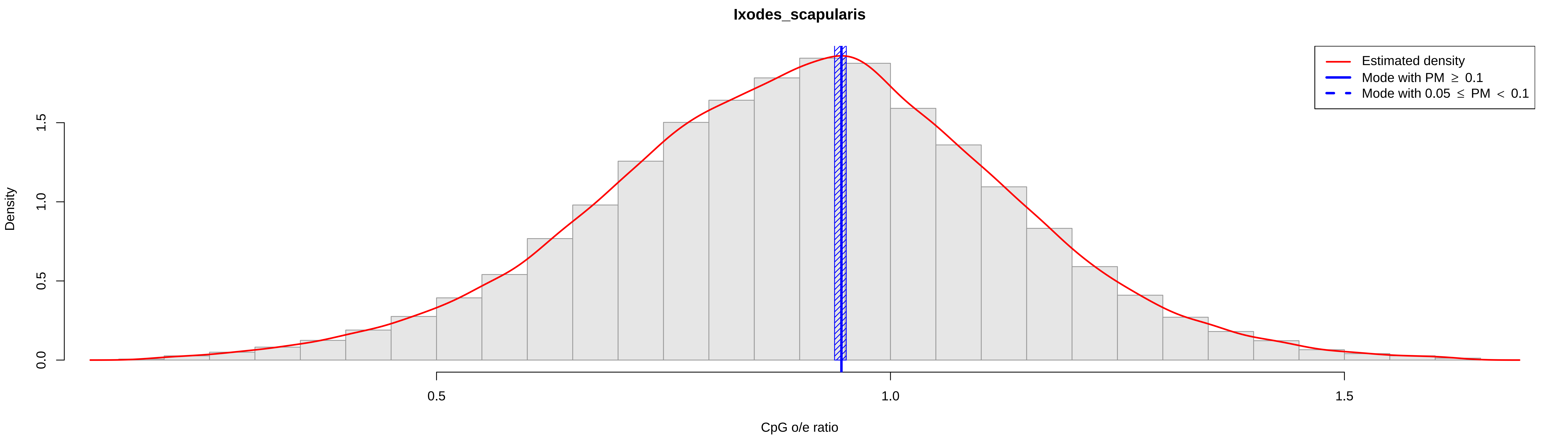
Ixodes_scapularis
Estimated density
Mode with PM ≥ 0.1
Mode with 0.05 ≤ PM < 0.1
Density
0.0
0.5
1.0
1.5
0.5
1.0
1.5
CpG o/e ratio

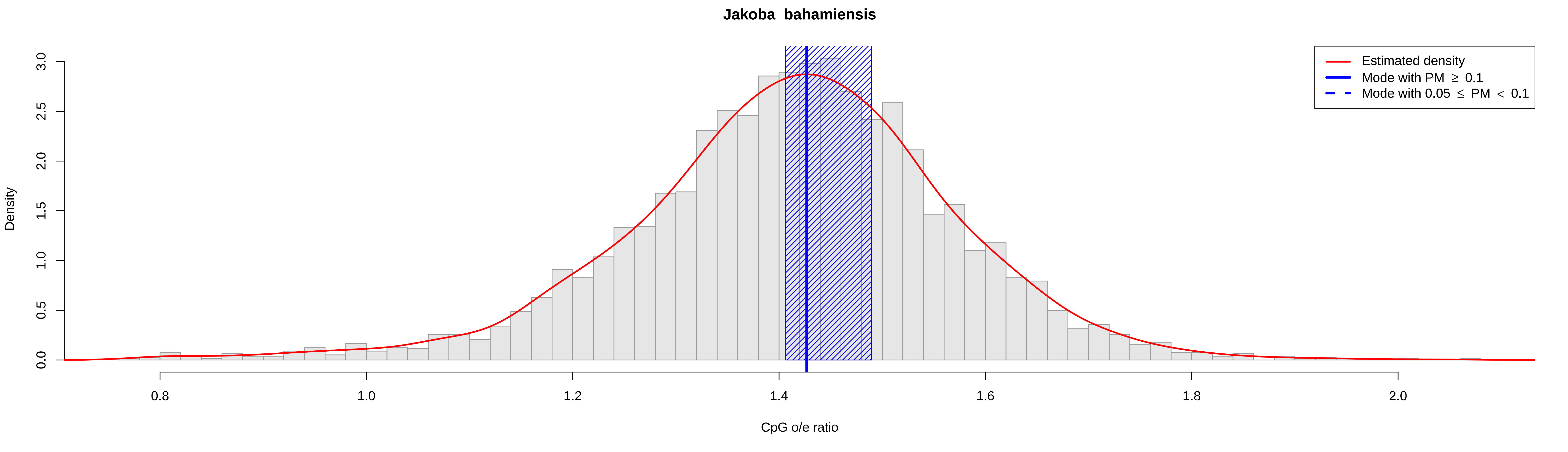

Jakoba_bahamiensis
Density
0.0
0.5
1.0
1.5
2.0
2.5
3.0
0.8
1.0
1.2
1.4
1.6
1.8
2.0
CpG o/e ratio
Estimated density
Mode with PM ≥ 0.1
Mode with 0.05 ≤ PM < 0.1

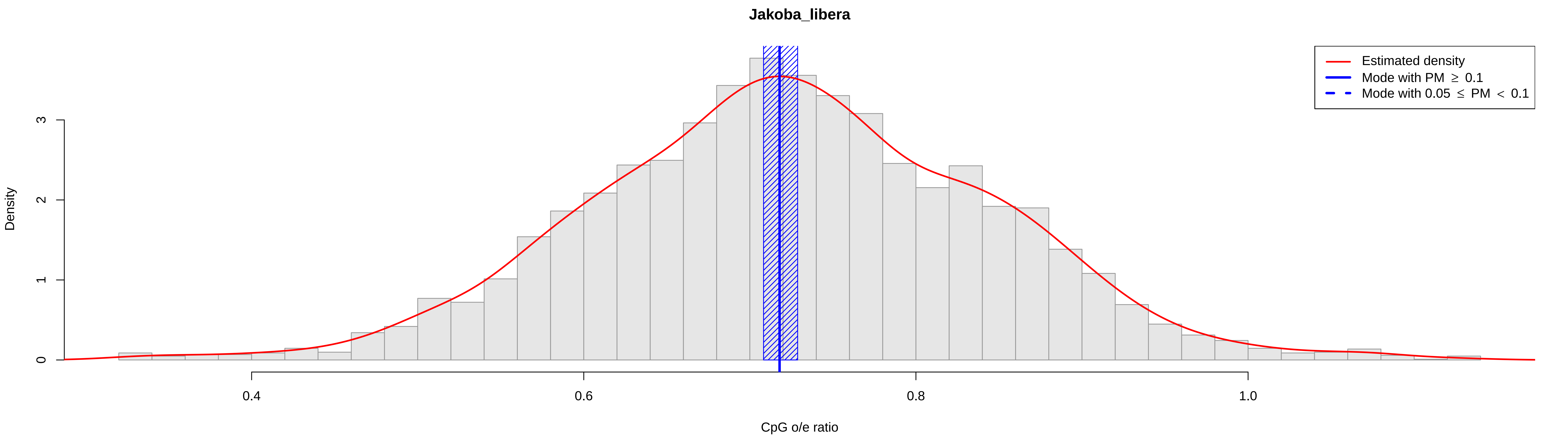

Jakoba_libera
Estimated density
Mode with PM ≥ 0.1
Mode with 0.05 ≤ PM < 0.1
Density
0
1
2
3
0.4
0.6
0.8
1.0
CpG o/e ratio

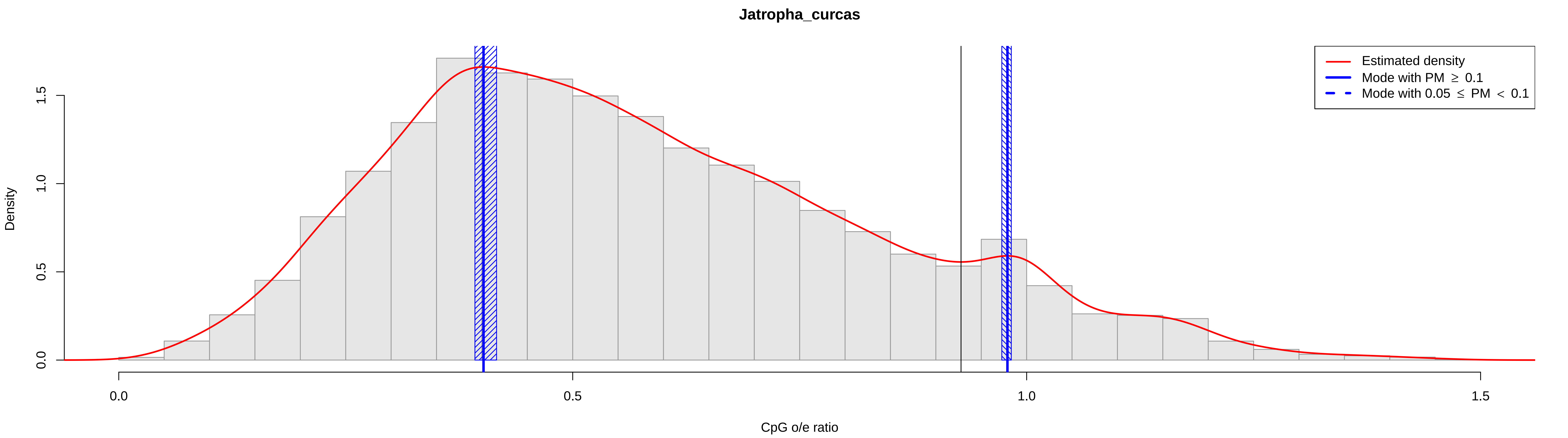

Jatropha_curcas
Estimated density
Mode with PM ≥ 0.1
Mode with 0.05 ≤ PM < 0.1
Density
0.0
0.5
1.0
1.5
0.0
0.5
1.0
1.5
CpG o/e ratio

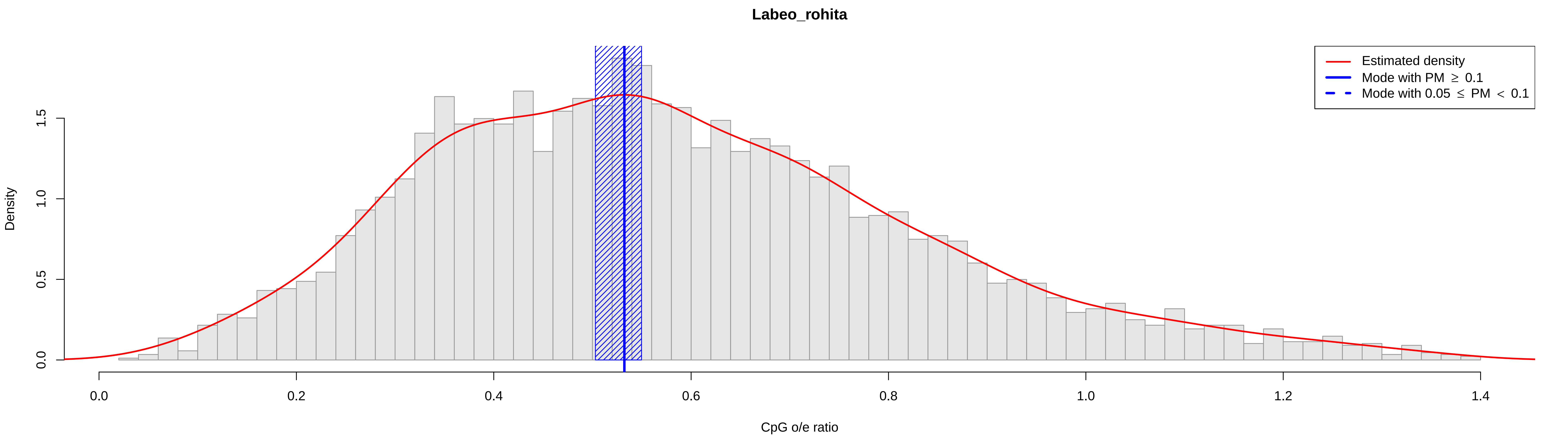
Labeo_rohita
Estimated density
Mode with PM ≥ 0.1
Mode with 0.05 ≤ PM < 0.1
Density
0.0
0.5
1.0
1.5
0.0
0.2
0.4
0.6
0.8
1.0
1.2
1.4
CpG o/e ratio

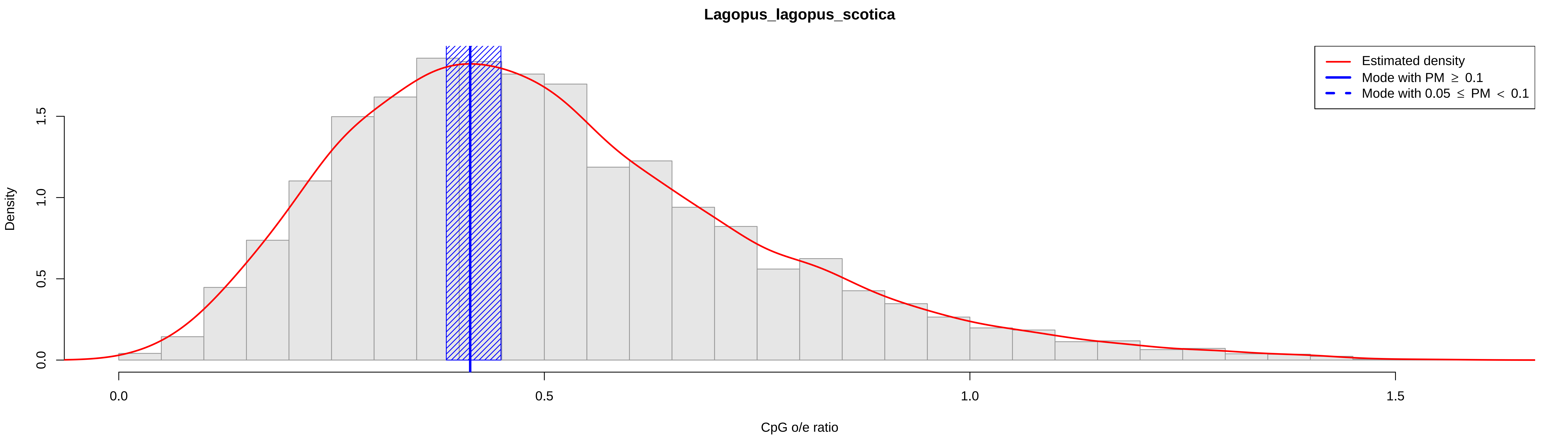
Lagopus_lagopus_scotica
Estimated density
Mode with PM ≥ 0.1
Mode with 0.05 ≤ PM < 0.1
Density
0.0
0.5
1.0
1.5
CpG o/e ratio

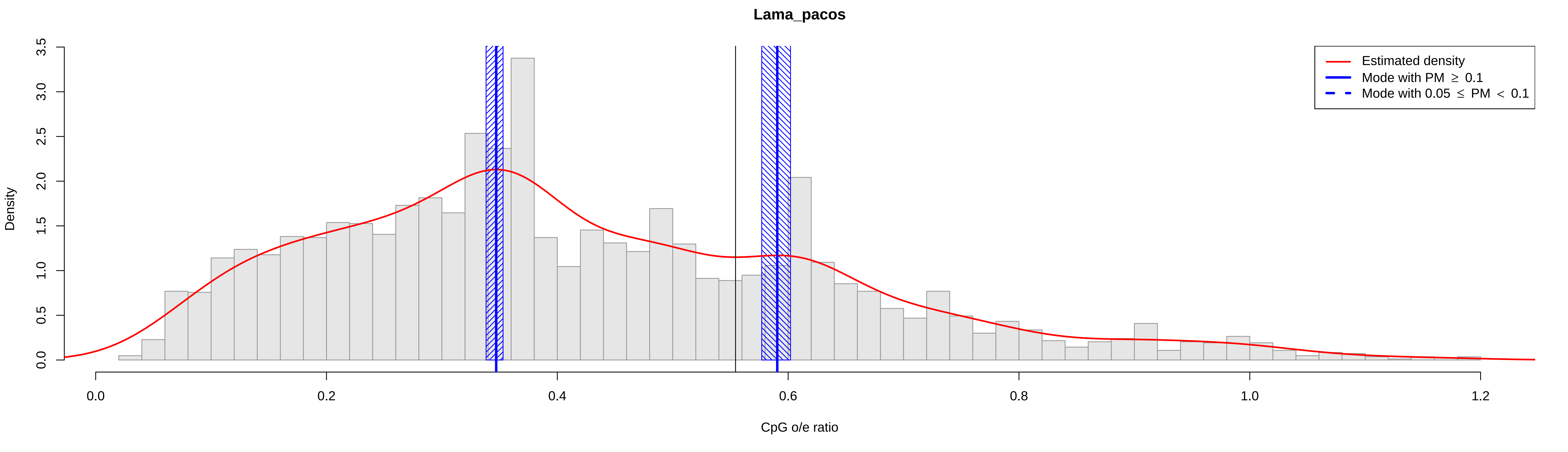

Lama_pacos
Estimated density
Mode with PM ≥ 0.1
Mode with 0.05 ≤ PM < 0.1
Density
CpG o/e ratio
0.0
0.5
1.0
1.5
2.0
2.5
3.0
3.5
0.0
0.2
0.4
0.6
0.8
1.0
1.2

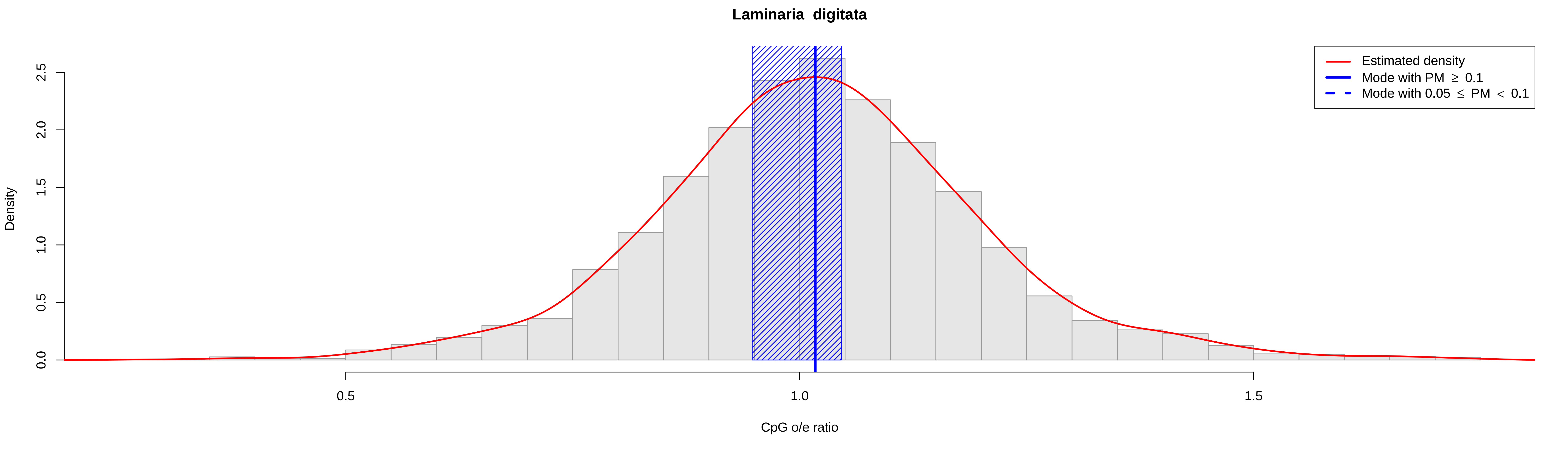
Laminaria_digitata
Estimated density
Mode with PM ≥ 0.1
Mode with 0.05 ≤ PM < 0.1
Density
0.0
0.5
1.0
1.5
2.0
2.5
0.5
1.0
1.5
CpG o/e ratio

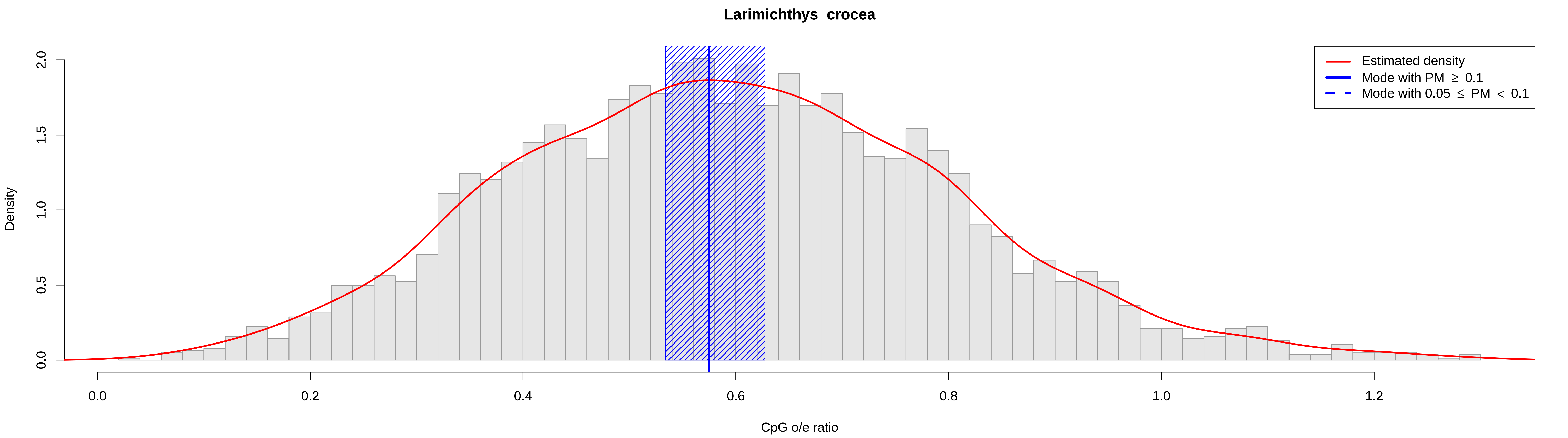
Larimichthys_crocea
Estimated density
Mode with PM ≥ 0.1
Mode with 0.05 ≤ PM < 0.1
Density
CpG o/e ratio
0.0
0.5
1.0
1.5
2.0
0.0
0.2
0.4
0.6
0.8
1.0
1.2

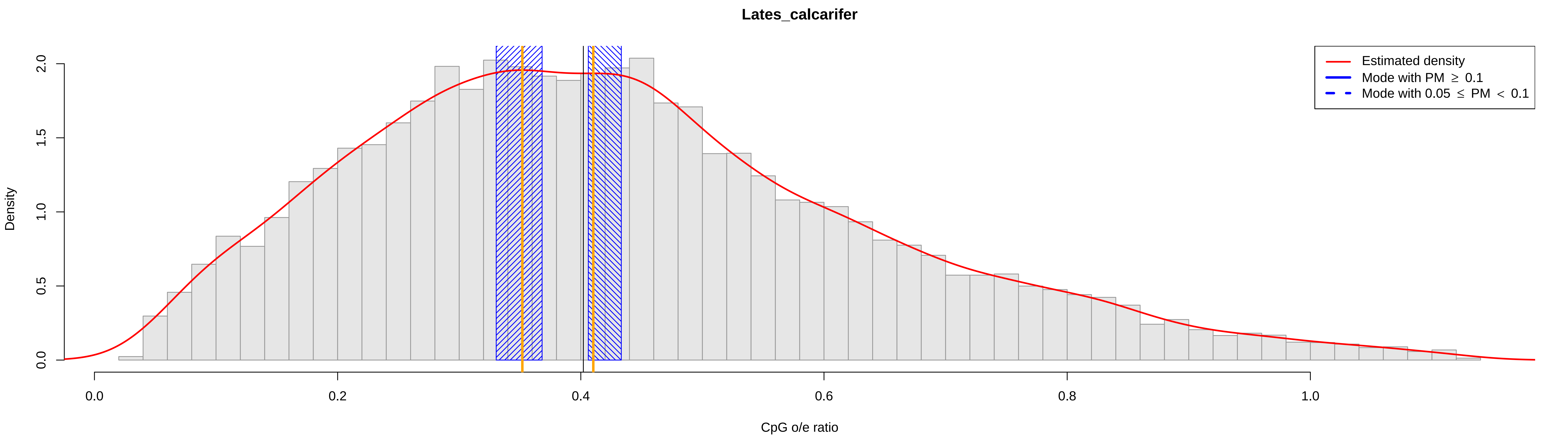

Lates_calcarifer
Estimated density
Mode with PM ≥ 0.1
Mode with 0.05 ≤ PM < 0.1
Density
0.0
0.5
1.0
1.5
2.0
0.0
0.2
0.4
0.6
0.8
1.0
CpG o/e ratio

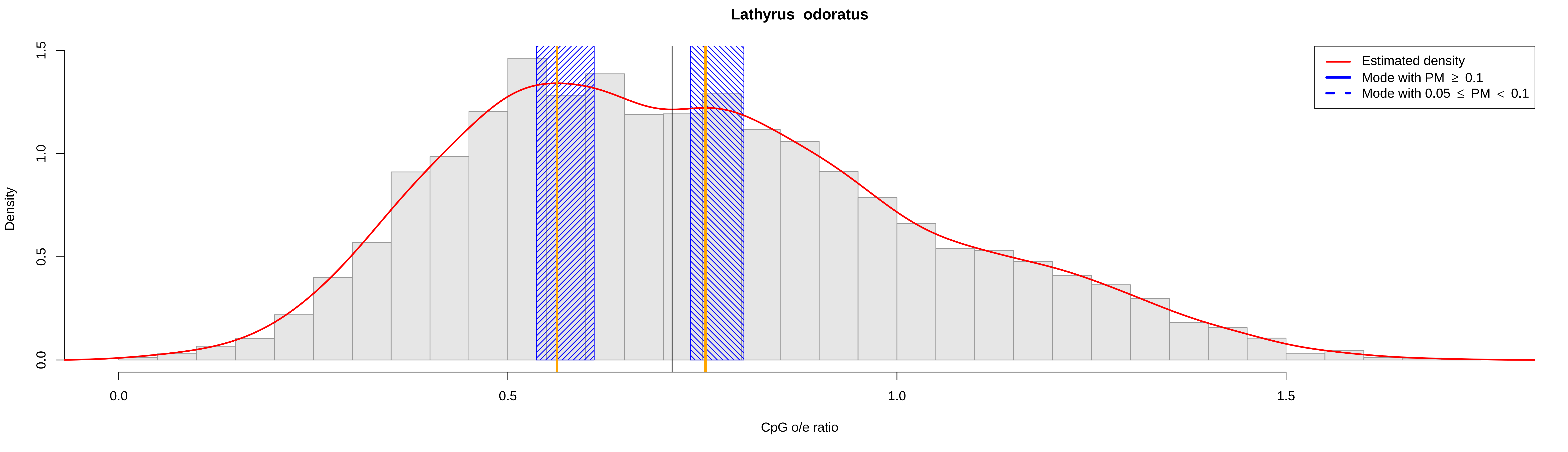

Lathyrus_odoratus
Estimated density
Mode with PM ≥ 0.1
Mode with 0.05 ≤ PM < 0.1
Density
0.0
0.5
1.0
1.5
0.0
0.5
1.0
1.5
CpG o/e ratio

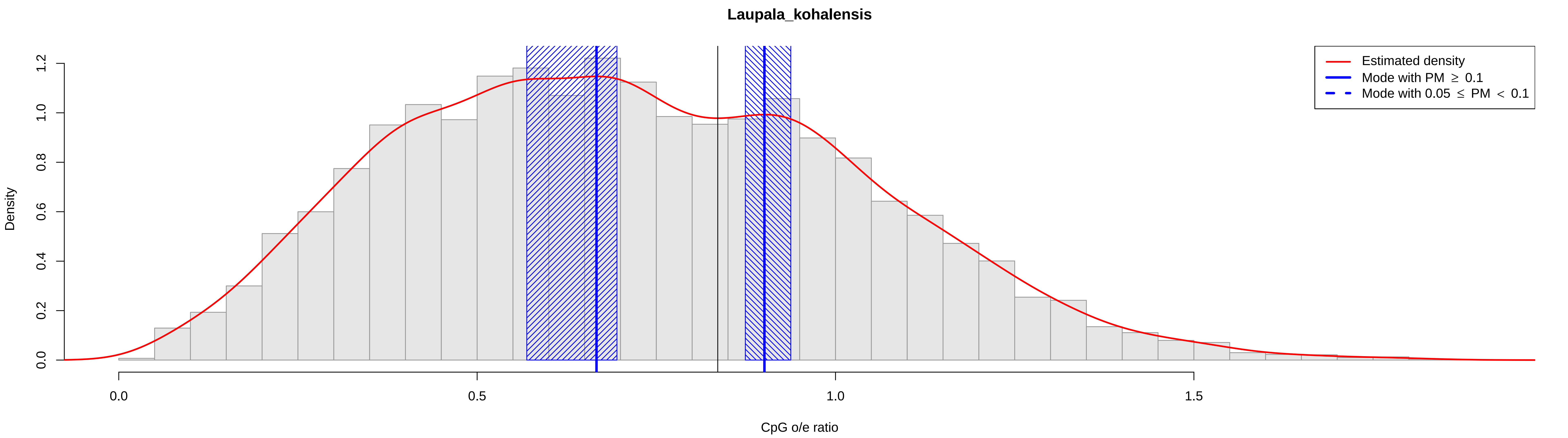

Laupala_kohalensis
Estimated density
Mode with PM ≥ 0.1
Mode with 0.05 ≤ PM < 0.1
Density
0.0
0.2
0.4
0.6
0.8
1.0
1.2
0.0
0.5
1.0
1.5
CpG o/e ratio

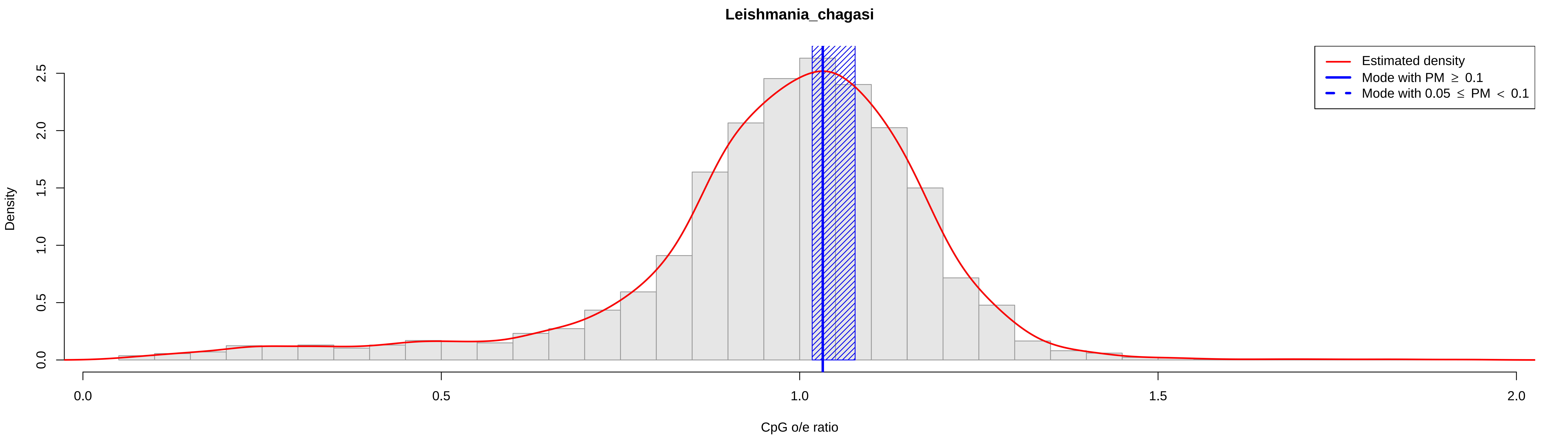

Leishmania_chagasi
Estimated density
Mode with PM ≥ 0.1
Mode with 0.05 ≤ PM < 0.1
Density
0.0
0.5
1.0
1.5
2.0
2.5
0.0
0.5
1.0
1.5
2.0
CpG o/e ratio

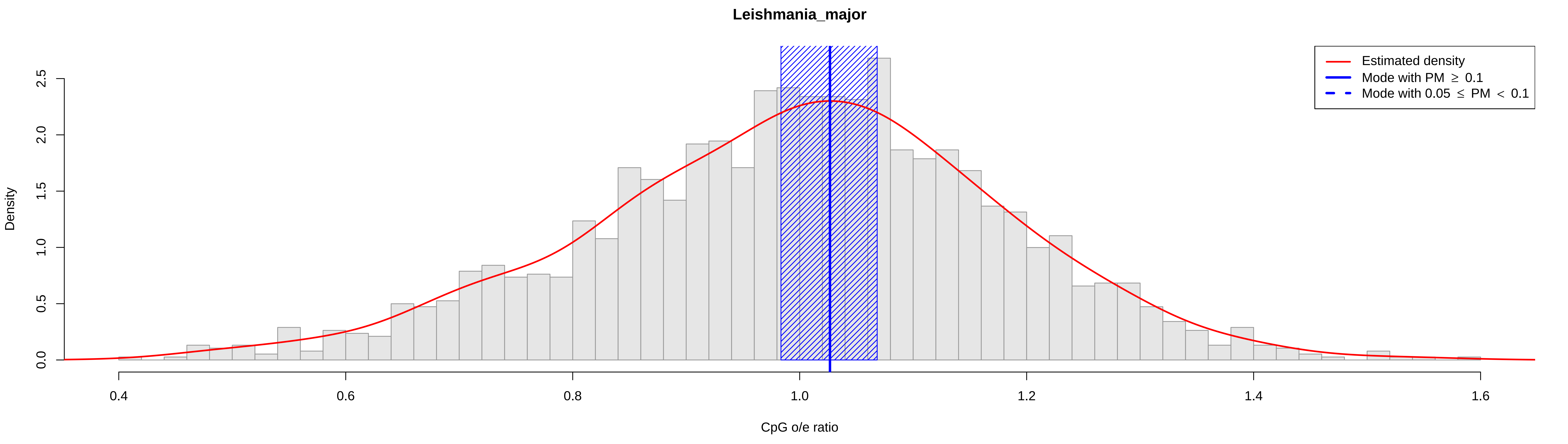
Leishmania_major
Estimated density
Mode with PM ≥ 0.1
Mode with 0.05 ≤ PM < 0.1
Density
0.0
0.5
1.0
1.5
2.0
2.5
0.4
0.6
0.8
1.0
1.2
1.4
1.6
CpG o/e ratio

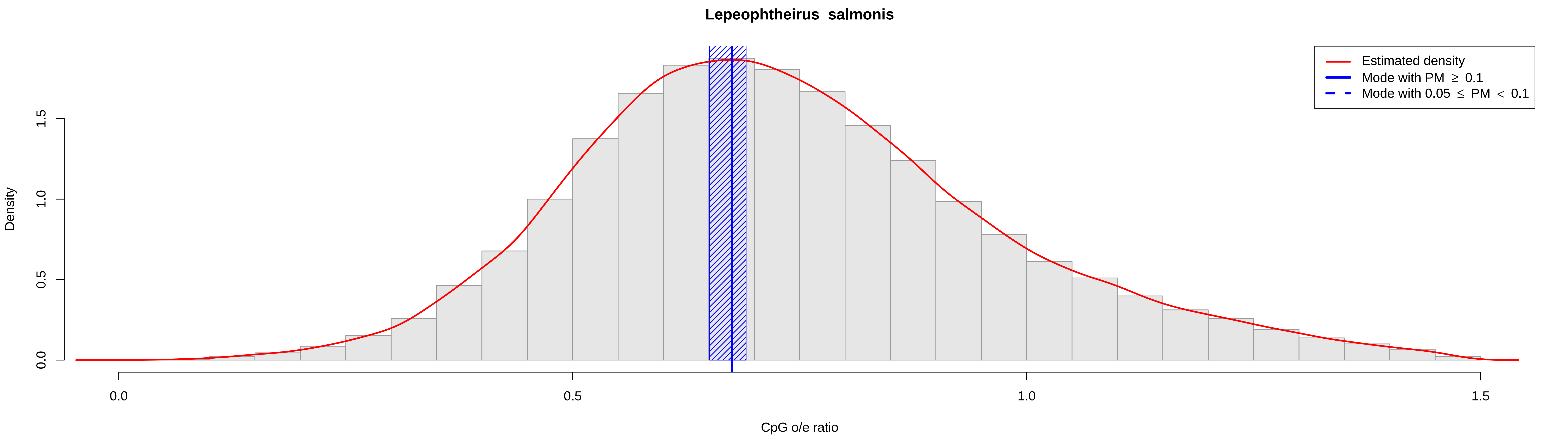
Lepeophtheirus_salmonis
Estimated density
Mode with PM ≥ 0.1
Mode with 0.05 ≤ PM < 0.1
Density
0.0
0.5
1.0
1.5
CpG o/e ratio

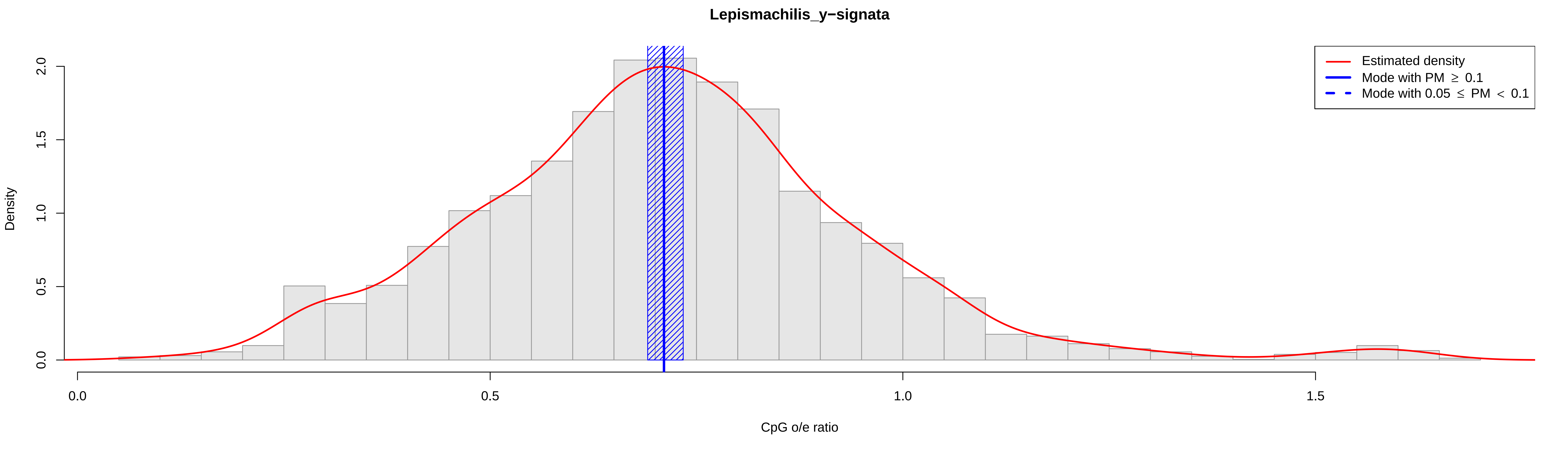

Lepismachilis_y−signata
Estimated density
Mode with PM ≥ 0.1
Mode with 0.05 ≤ PM < 0.1
Density
0.0
0.5
1.0
1.5
2.0
0.0
0.5
1.0
1.5
CpG o/e ratio

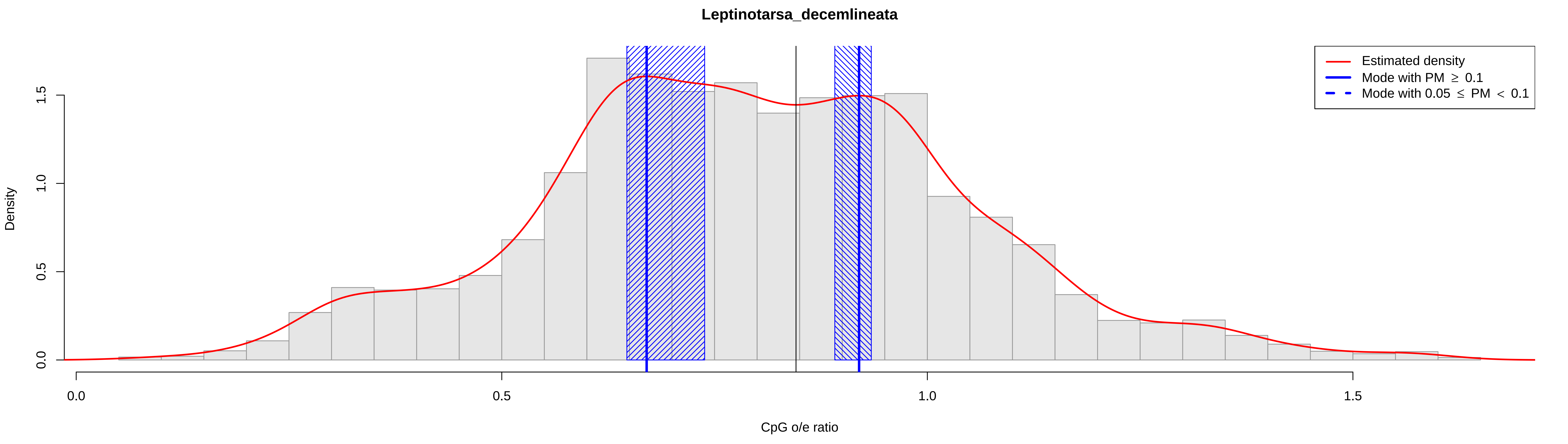

Leptinotarsa_decemlineata
Estimated density
Mode with PM ≥ 0.1
Mode with 0.05 ≤ PM < 0.1
Density
0.0
0.5
1.0
1.5
0.0
0.5
1.0
1.5
CpG o/e ratio

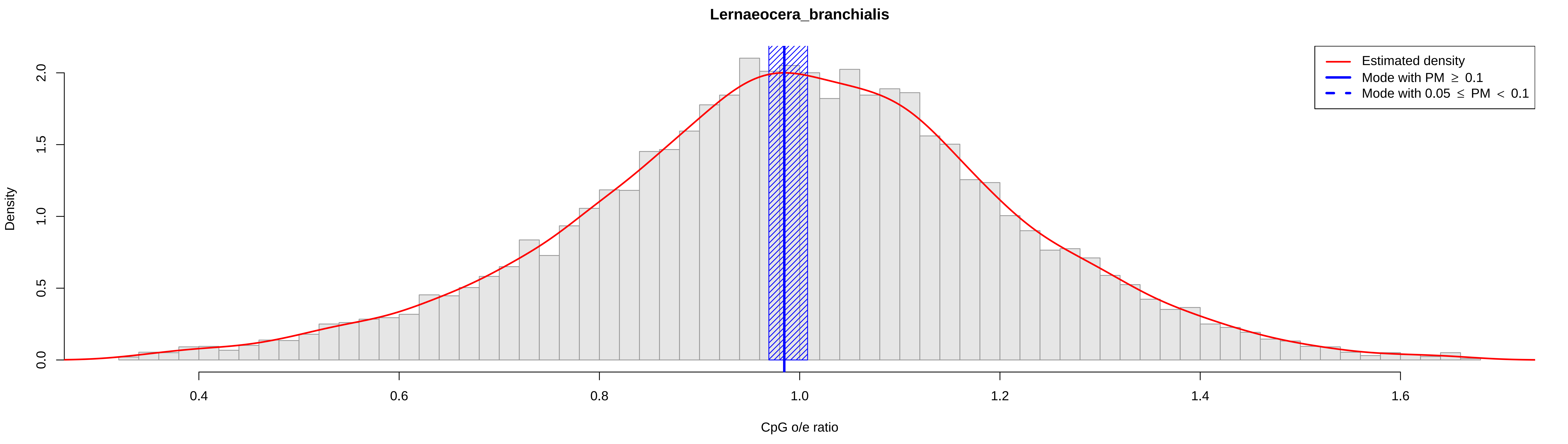
Lernaeocera_branchialis
Estimated density
Mode with PM ≥ 0.1
Mode with 0.05 ≤ PM < 0.1
Density
0.0
0.5
1.0
1.5
2.0
0.4
0.6
0.8
1.0
1.2
1.4
1.6
CpG o/e ratio

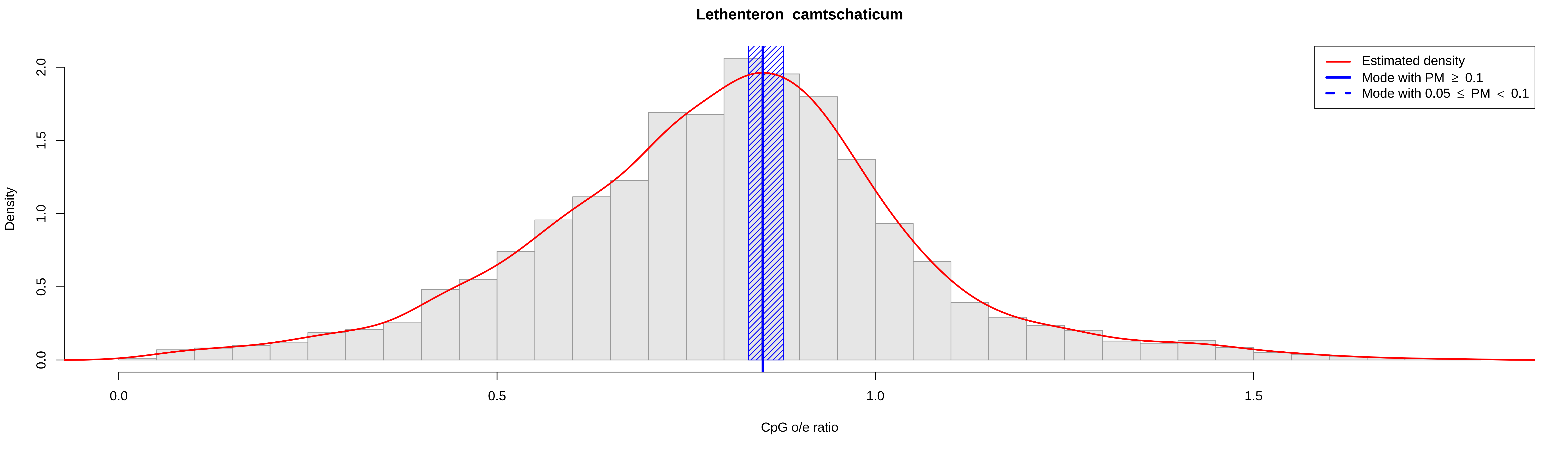

Lethenteron_camtschaticum
Density
CpG o/e ratio
0.0
0.5
1.0
1.5
2.0
Estimated density
Mode with PM ≥ 0.1
Mode with 0.05 ≤ PM < 0.1

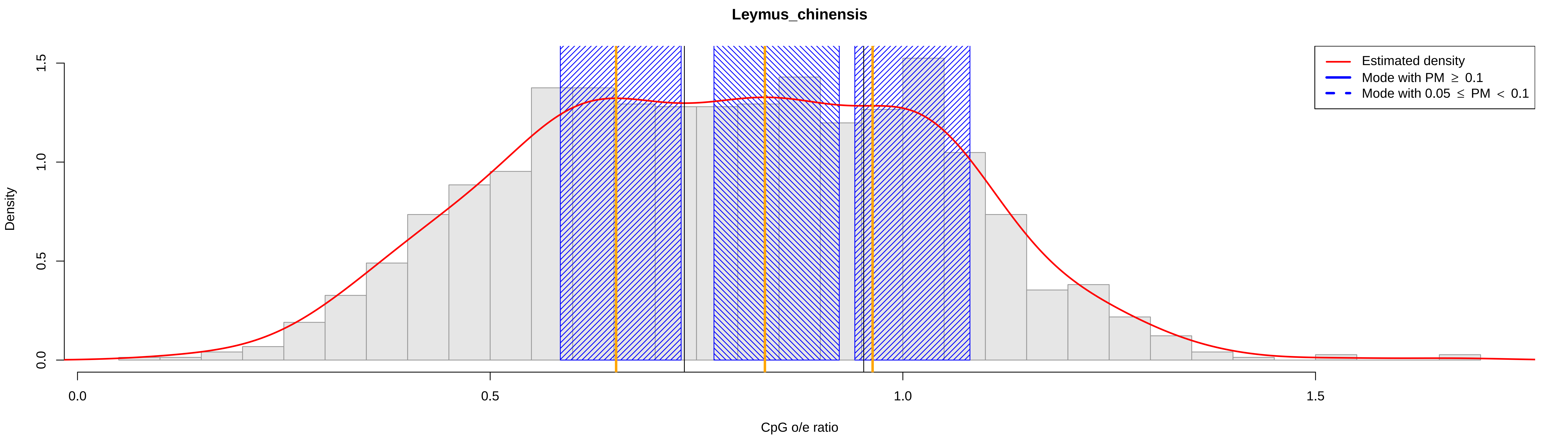
Leymus_chinensis
Estimated density
Mode with PM ≥ 0.1
Mode with 0.05 ≤ PM < 0.1
Density
0.0
0.5
1.0
1.5
0.0
0.5
1.0
1.5
CpG o/e ratio

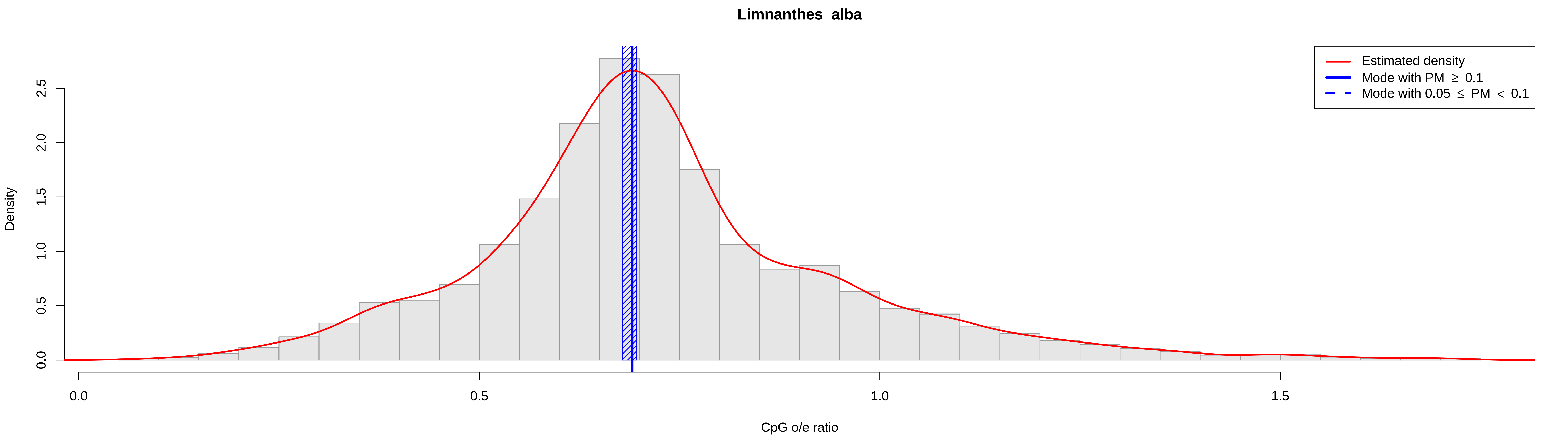

Limnanthes_alba
Estimated density
Mode with PM ≥ 0.1
Mode with 0.05 ≤ PM < 0.1
Density
0.0
0.5
1.0
1.5
2.0
2.5
0.0
0.5
1.0
1.5
CpG o/e ratio

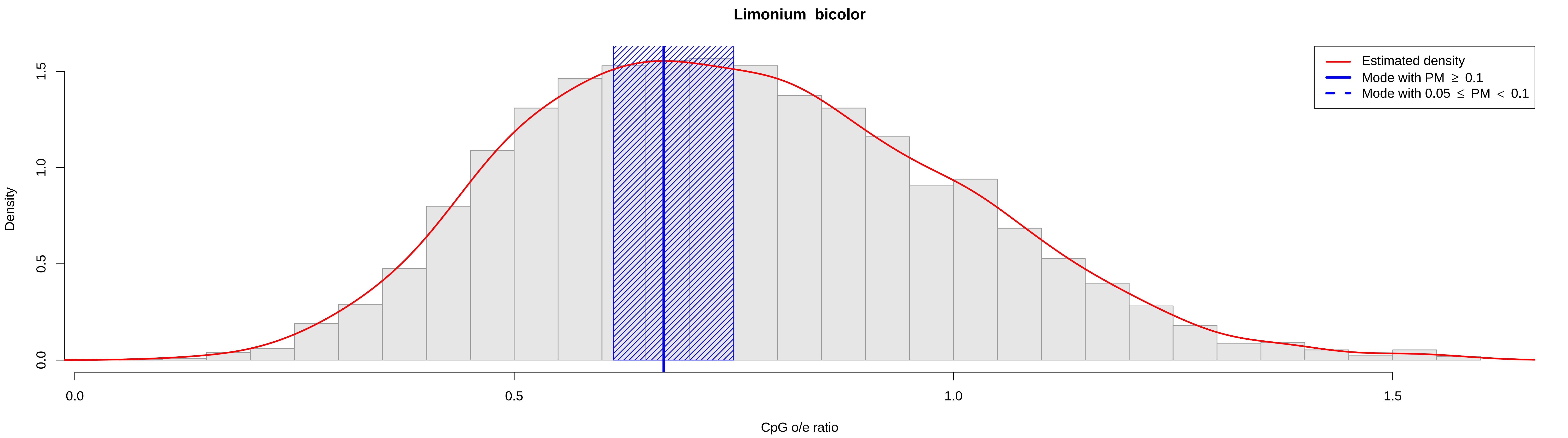
Limonium_bicolor
Estimated density
Mode with PM ≥ 0.1
Mode with 0.05 ≤ PM < 0.1
Density
CpG o/e ratio
0.0
0.5
1.0
1.5

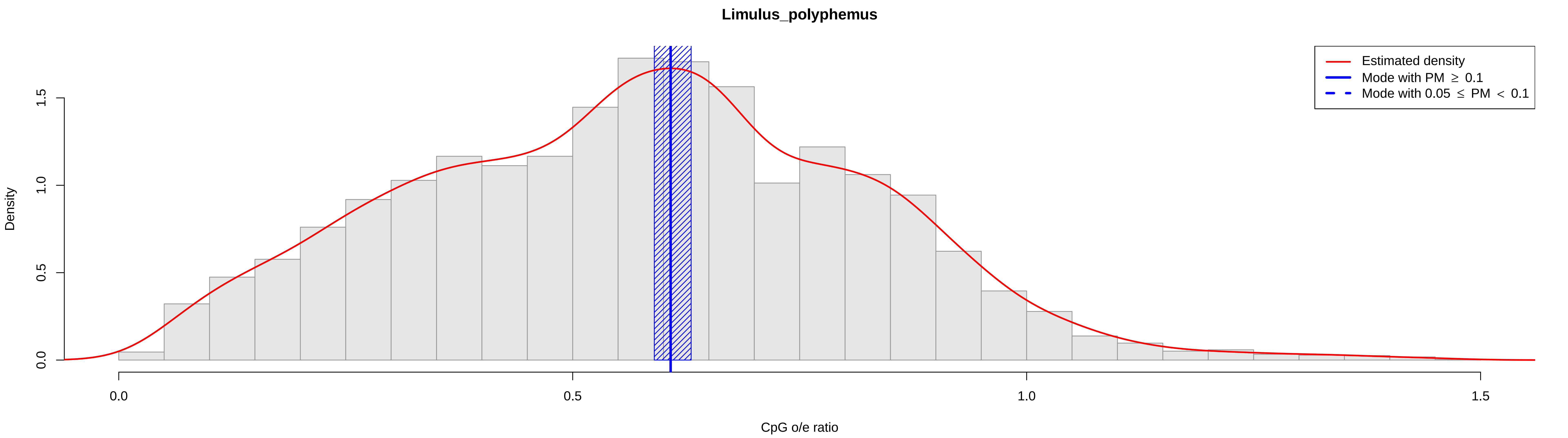
Limulus_polyphemus
Estimated density
Mode with PM ≥ 0.1
Mode with 0.05 ≤ PM < 0.1
Density
0.0
0.5
1.0
1.5
CpG o/e ratio

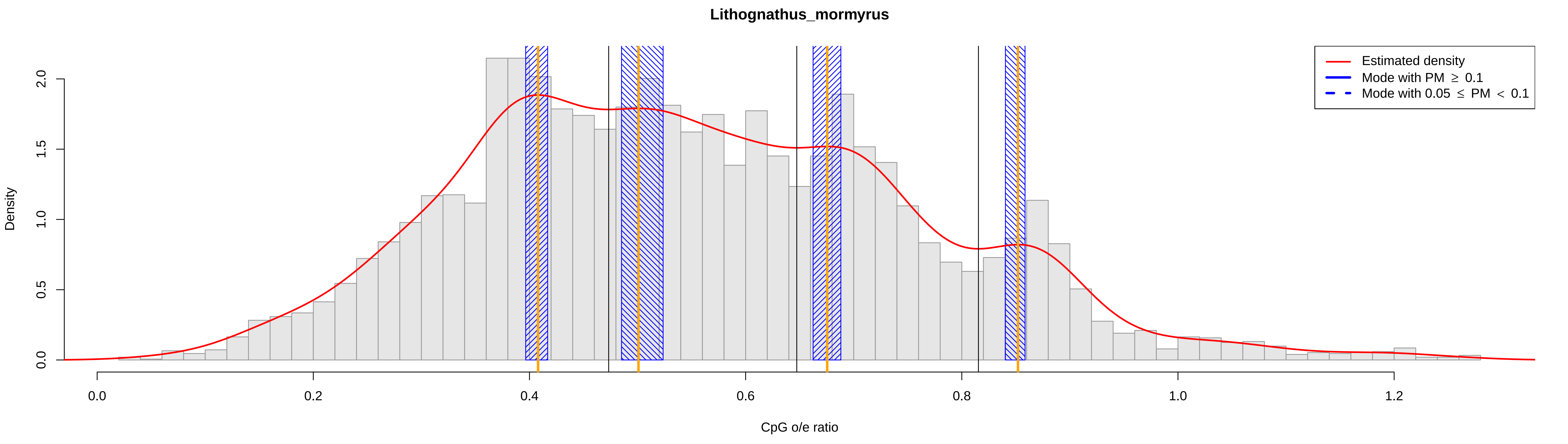
Lithognathus_mormyrus
Estimated density
Mode with PM ≥ 0.1
Mode with 0.05 ≤ PM < 0.1
Density
0.0
0.5
1.0
1.5
2.0
0.0
0.2
0.4
0.6
0.8
1.0
1.2
CpG o/e ratio

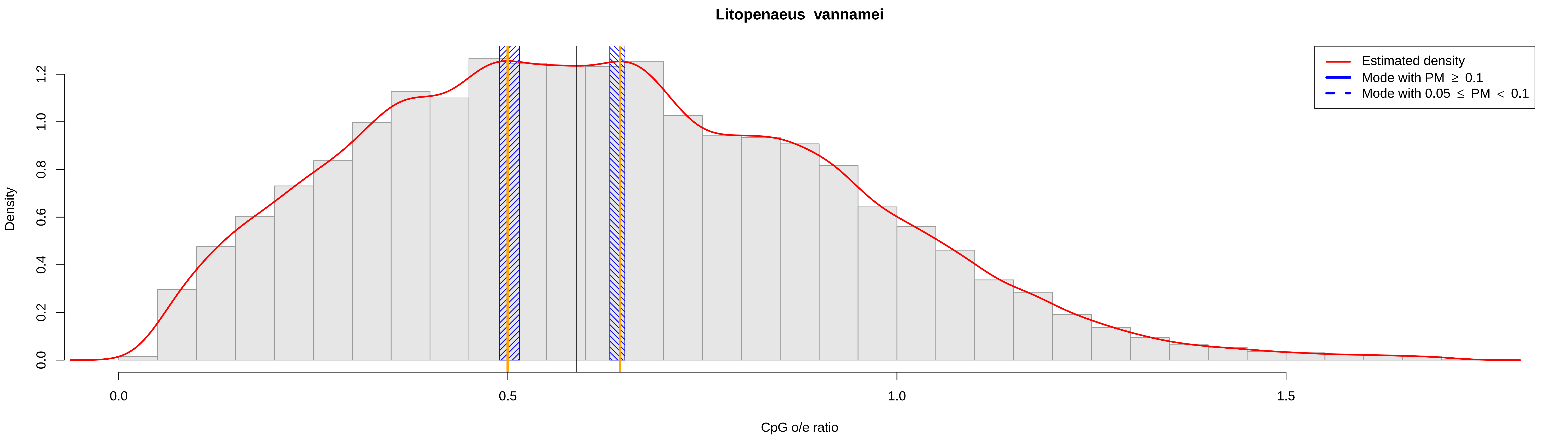

Litopenaeus_vannamei
Estimated density
Mode with PM ≥ 0.1
Mode with 0.05 ≤ PM < 0.1
Density
0.0
0.2
0.4
0.6
0.8
1.0
1.2
0.0
0.5
1.0
1.5
CpG o/e ratio

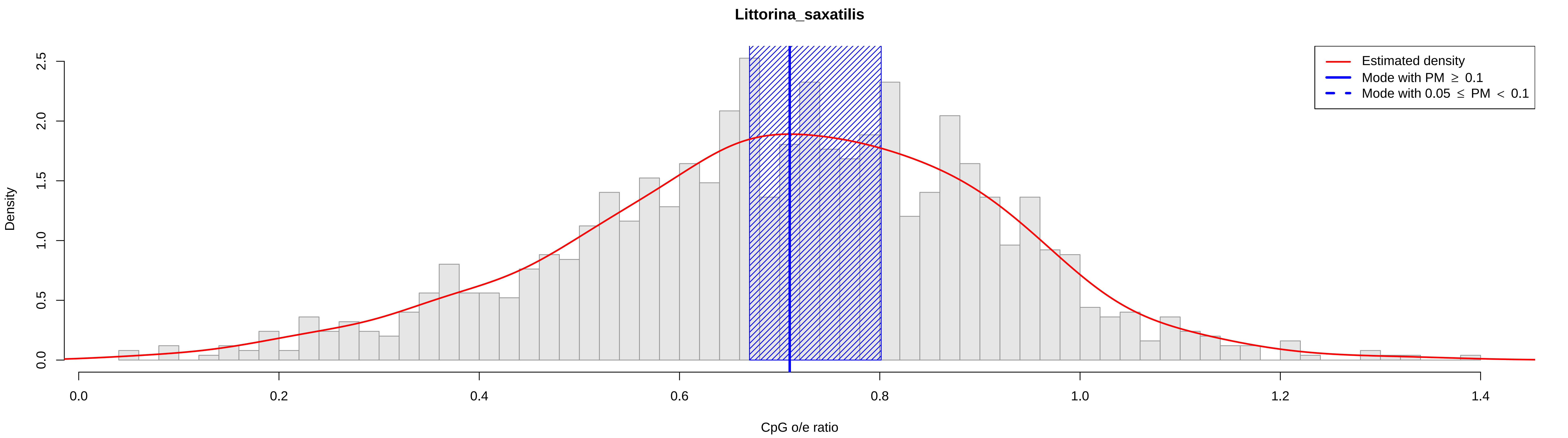
Littorina_saxatilis
Estimated density
Mode with PM ≥ 0.1
Mode with 0.05 ≤ PM < 0.1
Density
0.0
0.5
1.0
1.5
2.0
2.5
0.0
0.2
0.4
0.6
0.8
1.0
1.2
1.4
CpG o/e ratio

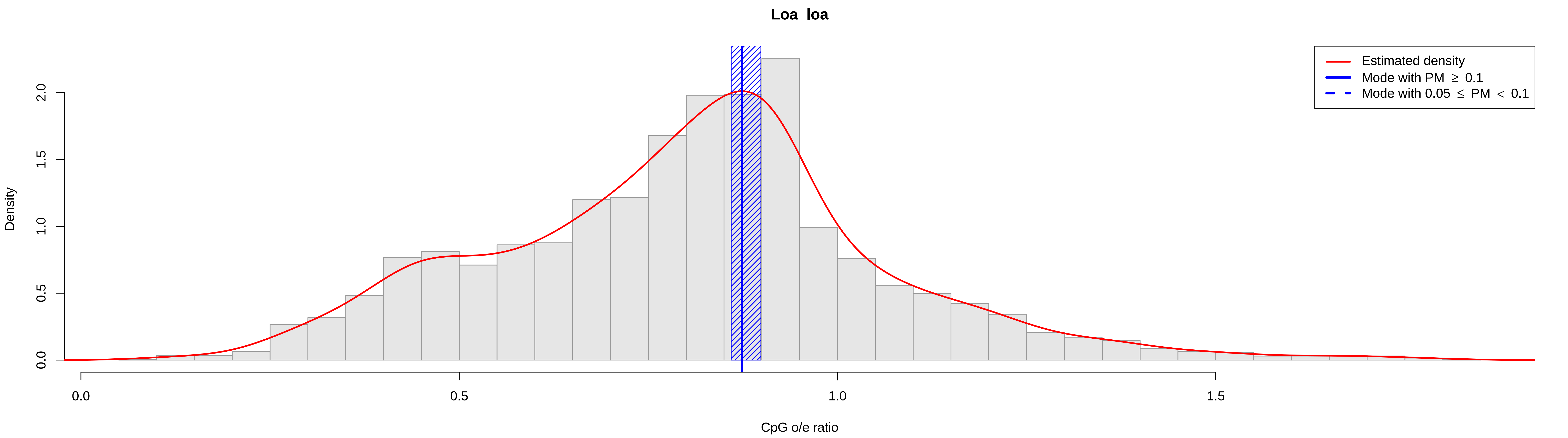
Loa_loa
Estimated density
Mode with PM ≥ 0.1
Mode with 0.05 ≤ PM < 0.1
Density
0.0
0.5
1.0
1.5
2.0
0.0
0.5
1.0
1.5
CpG o/e ratio

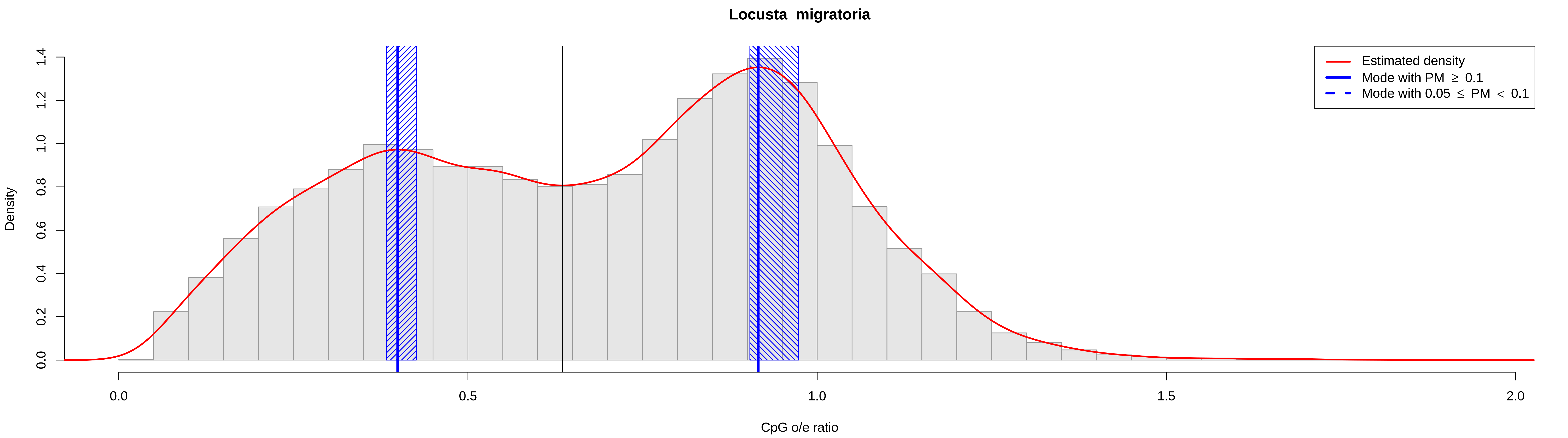
Locusta_migratoria
Estimated density
Mode with PM ≥ 0.1
Mode with 0.05 ≤ PM < 0.1
Density
0.0
0.2
0.4
0.6
0.8
1.0
1.2
1.4
0.0
0.5
1.0
1.5
2.0
CpG o/e ratio

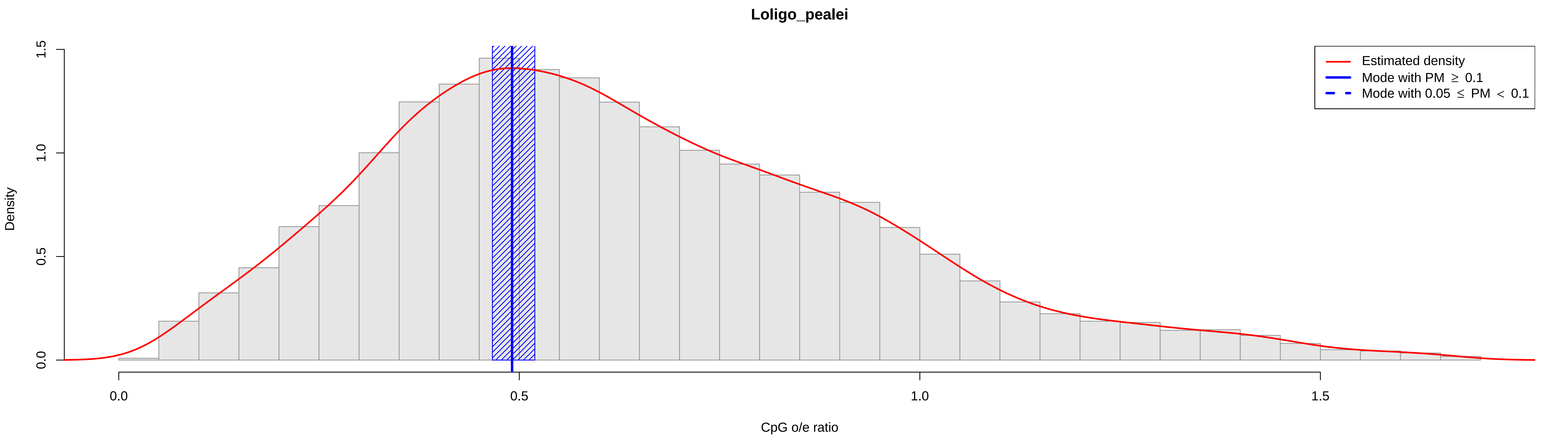

Loligo_pealei
Estimated density
Mode with PM ≥ 0.1
Mode with 0.05 ≤ PM < 0.1
Density
0.0
0.5
1.0
1.5
0.0
0.5
1.0
1.5
CpG o/e ratio

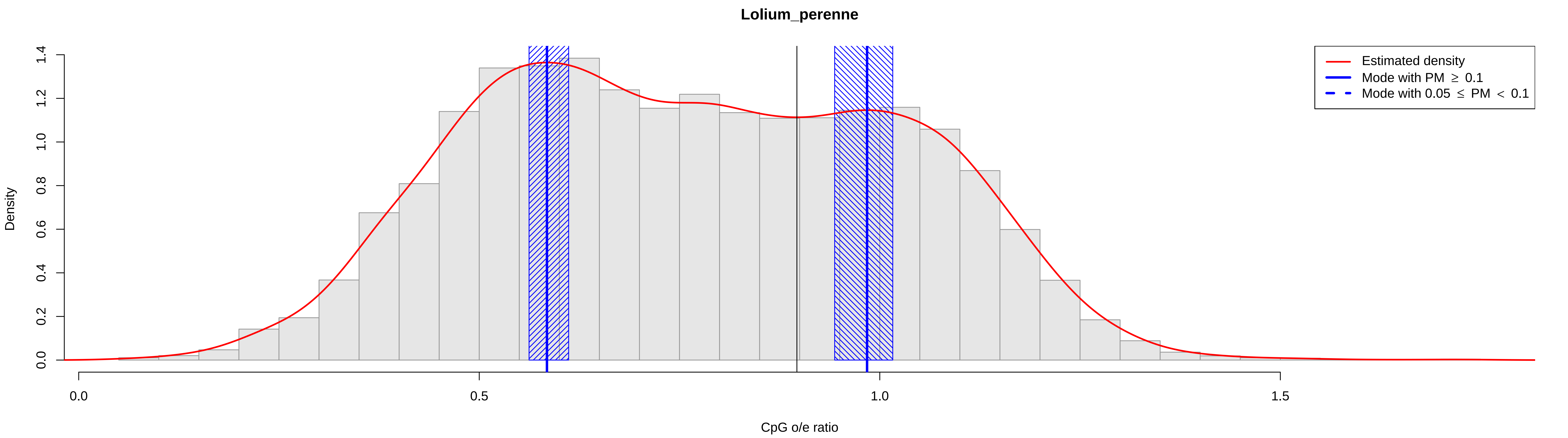

Lolium_perenne
Density
0.0
0.2
0.4
0.6
0.8
1.0
1.2
1.4
0.0
0.5
1.0
1.5
CpG o/e ratio
Estimated density
Mode with PM ≥ 0.1
Mode with 0.05 ≤ PM < 0.1

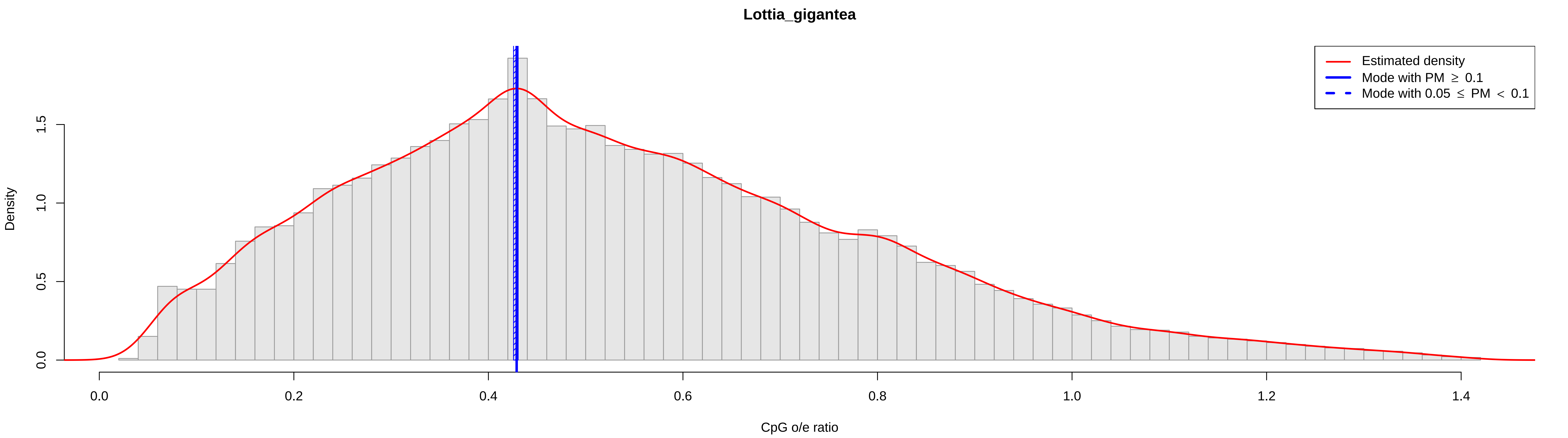

Lottia_gigantea
Estimated density
Mode with PM ≥ 0.1
Mode with 0.05 ≤ PM < 0.1
Density
0.0
0.5
1.0
1.5
0.0
0.2
0.4
0.6
0.8
1.0
1.2
1.4
CpG o/e ratio

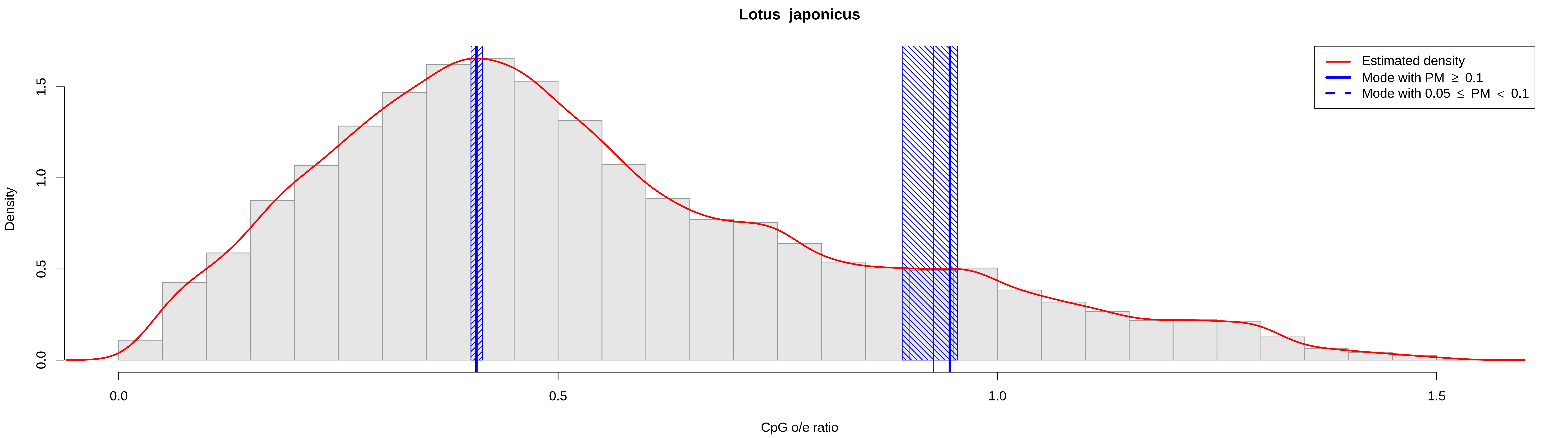
Lotus_japonicus
Estimated density
Mode with PM ≥ 0.1
Mode with 0.05 ≤ PM < 0.1
Density
0.0
0.5
1.0
1.5
0.0
0.5
1.0
1.5
CpG o/e ratio

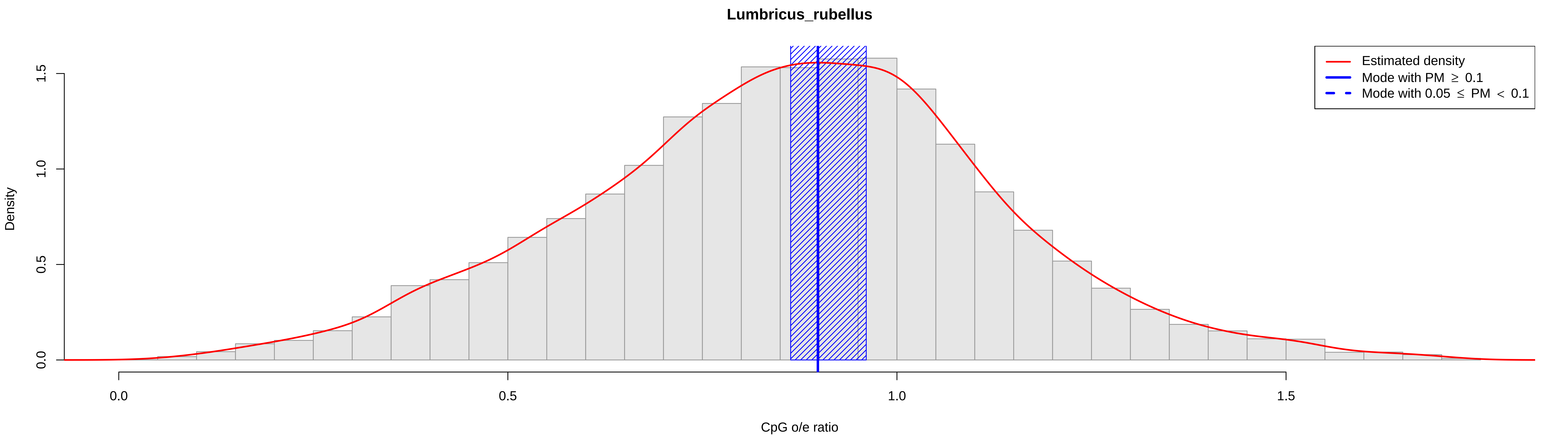

Lumbricus_rubellus
Estimated density
Mode with PM ≥ 0.1
Mode with 0.05 ≤ PM < 0.1
Density
0.0
0.5
1.0
1.5
0.0
0.5
1.0
1.5
CpG o/e ratio

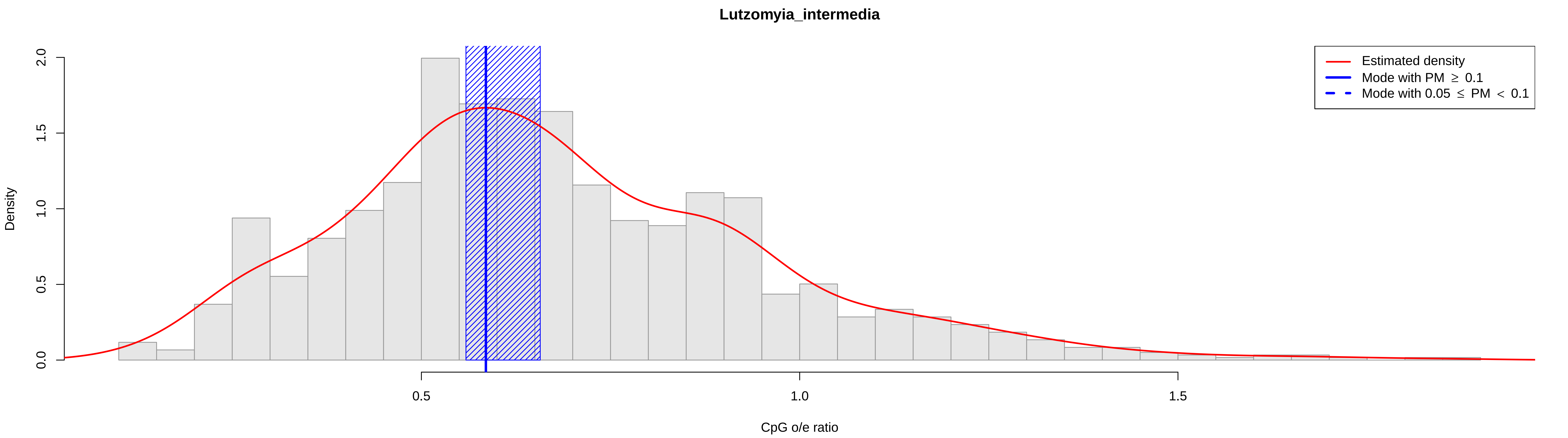
Lutzomyia_intermedia
Estimated density
Mode with PM ≥ 0.1
Mode with 0.05 ≤ PM < 0.1
Density
0.0
0.5
1.0
1.5
2.0
0.5
1.0
1.5
CpG o/e ratio

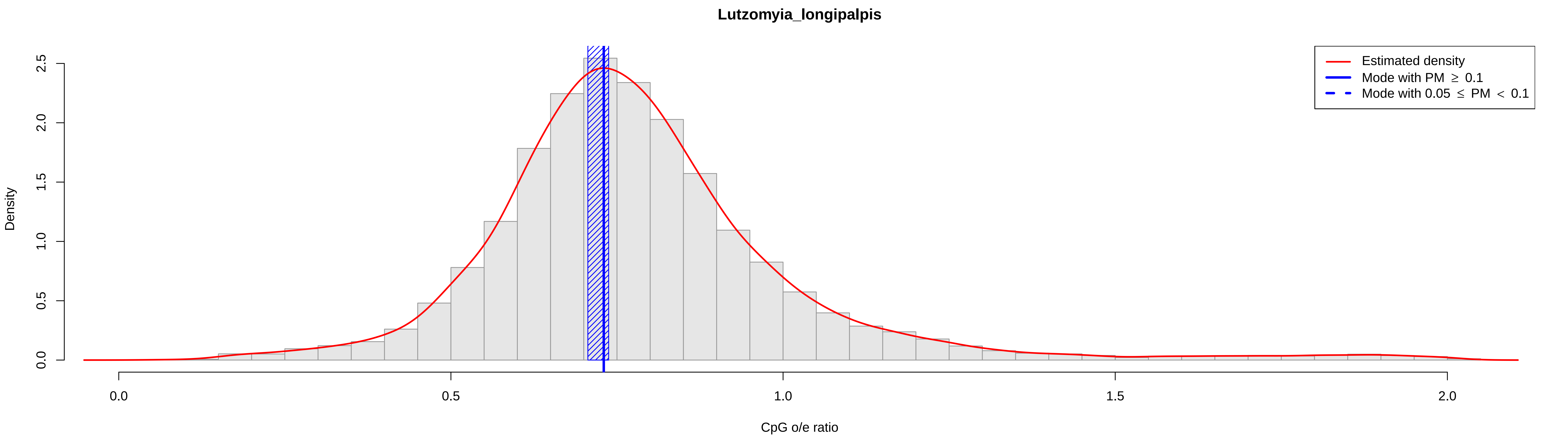

Lutzomyia_longipalpis
Estimated density
Mode with PM ≥ 0.1
Mode with 0.05 ≤ PM < 0.1
Density
0.0
0.5
1.0
1.5
2.0
2.5
0.0
0.5
1.0
1.5
2.0
CpG o/e ratio

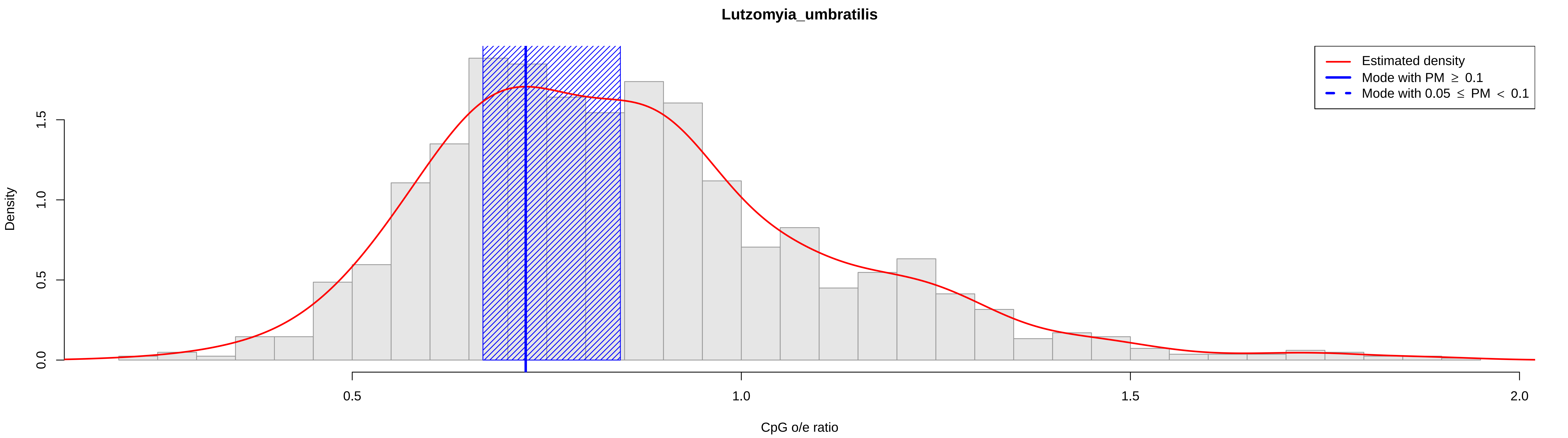

Lutzomyia_umbratilis
Estimated density
Mode with PM ≥ 0.1
Mode with 0.05 ≤ PM < 0.1
Density
0.0
0.5
1.0
1.5
0.5
1.0
1.5
2.0
CpG o/e ratio

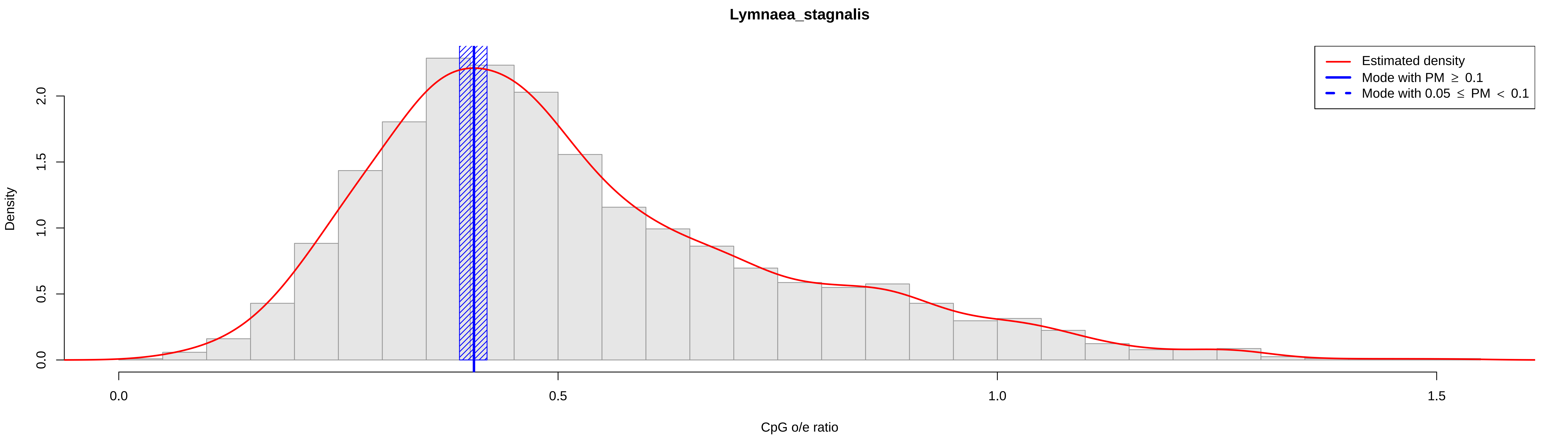

Lymnaea_stagnalis
Estimated density
Mode with PM ≥ 0.1
Mode with 0.05 ≤ PM < 0.1
Density
0.0
0.5
1.0
1.5
2.0
CpG o/e ratio
0.0
0.5
1.0
1.5

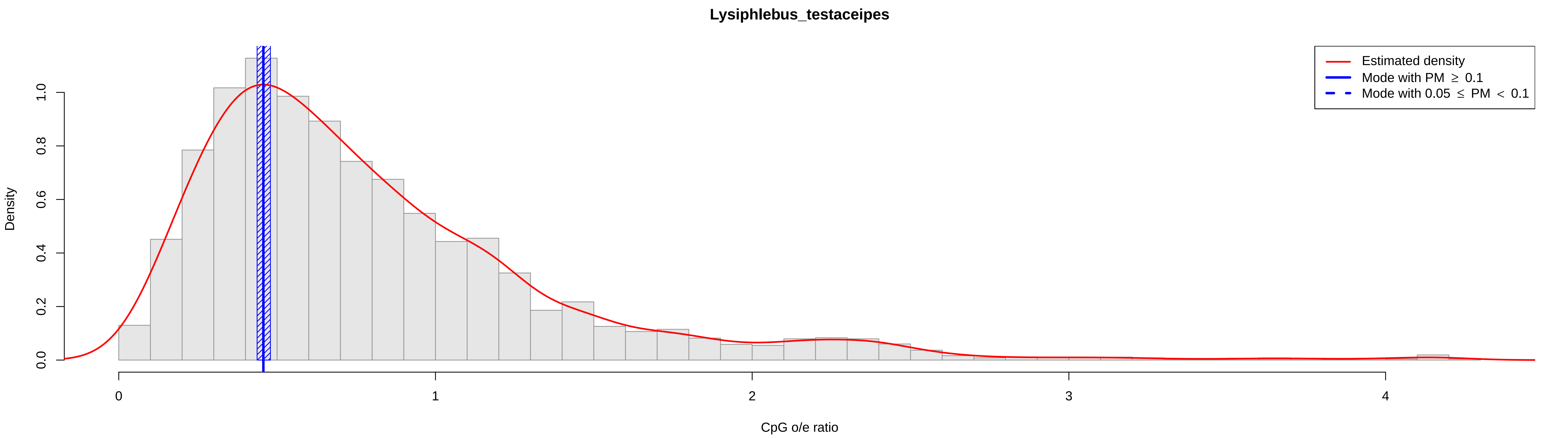

Lysiphlebus_testaceipes
Estimated density
Mode with PM ≥ 0.1
Mode with 0.05 ≤ PM < 0.1
Density
0.0
0.2
0.4
0.6
0.8
1.0
0
1
2
3
4
CpG o/e ratio

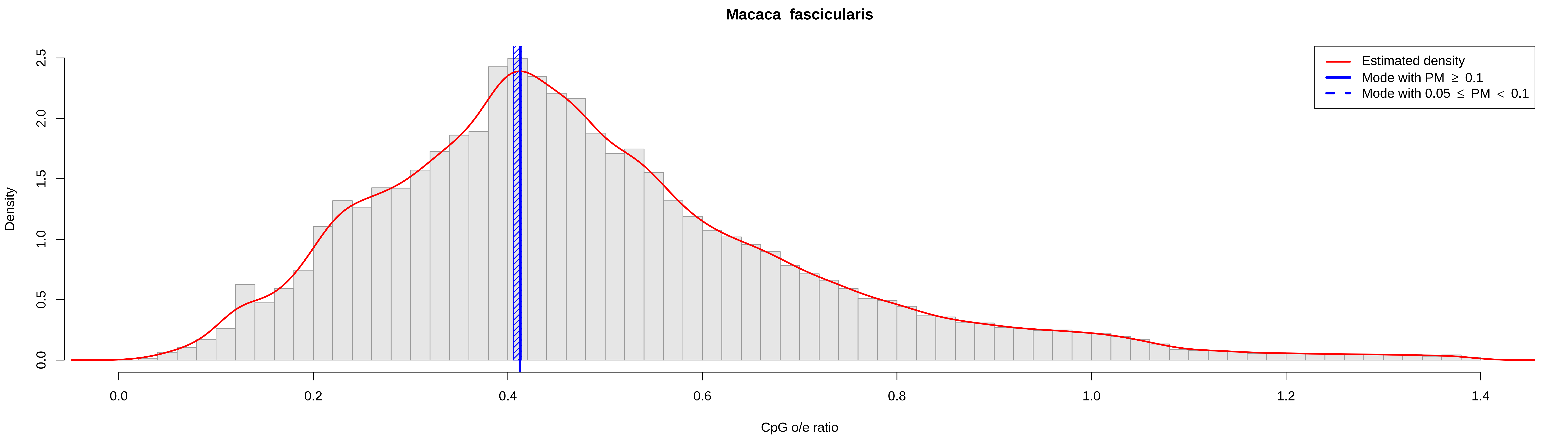
Macaca_fascicularis
Estimated density
Mode with PM ≥ 0.1
Mode with 0.05 ≤ PM < 0.1
Density
0.0
0.5
1.0
1.5
2.0
2.5
0.0
0.2
0.4
0.6
0.8
1.0
1.2
1.4
CpG o/e ratio

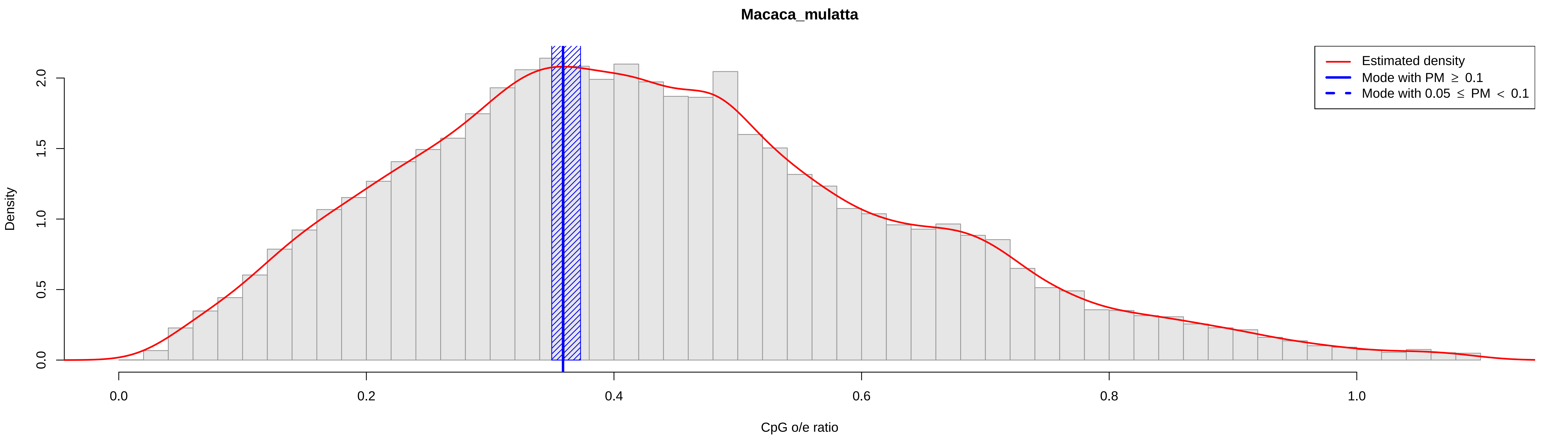

Macaca_mulatta
Estimated density
Mode with PM ≥ 0.1
Mode with 0.05 ≤ PM < 0.1
Density
0.0
0.5
1.0
1.5
2.0
0.0
0.2
0.4
0.6
0.8
1.0
CpG o/e ratio

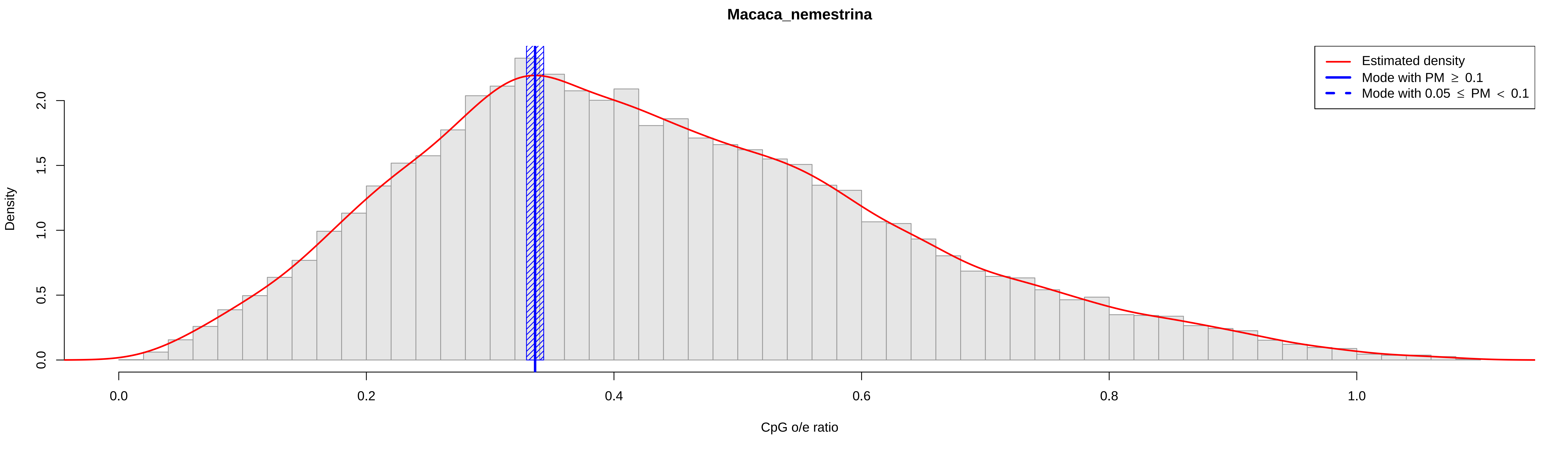

Macaca_nemestrina
Estimated density
Mode with PM ≥ 0.1
Mode with 0.05 ≤ PM < 0.1
Density
0.0
0.5
1.0
1.5
2.0
0.0
0.2
0.4
0.6
0.8
1.0
CpG o/e ratio

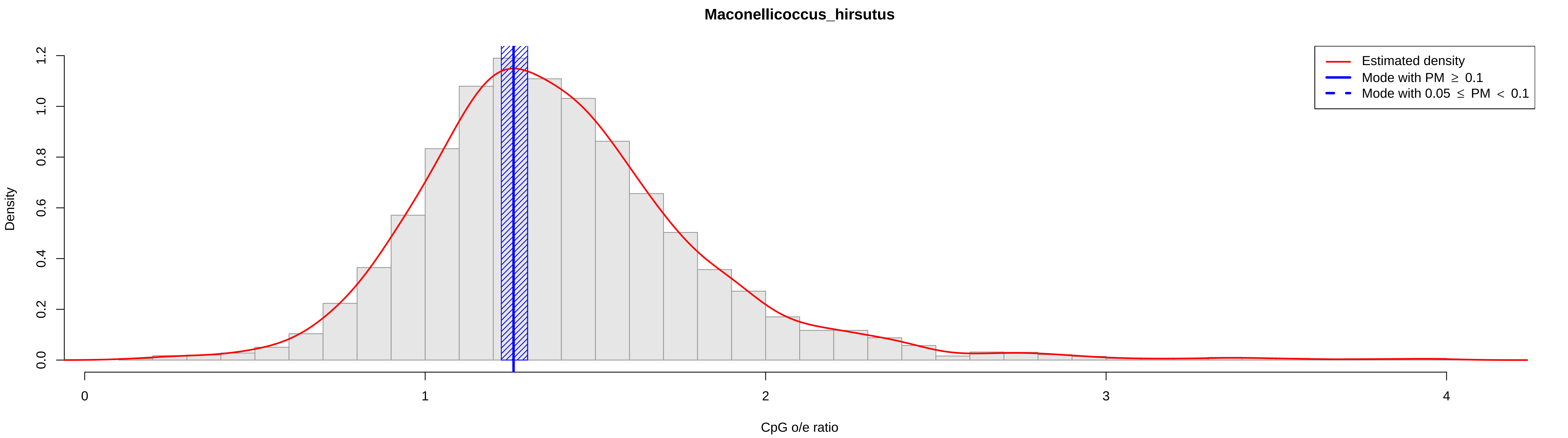

Maconellicoccus_hirsutus
Estimated density
Mode with PM ≥ 0.1
Mode with 0.05 ≤ PM < 0.1
Density
0.0
0.2
0.4
0.6
0.8
1.0
1.2
0
1
2
3
4
CpG o/e ratio

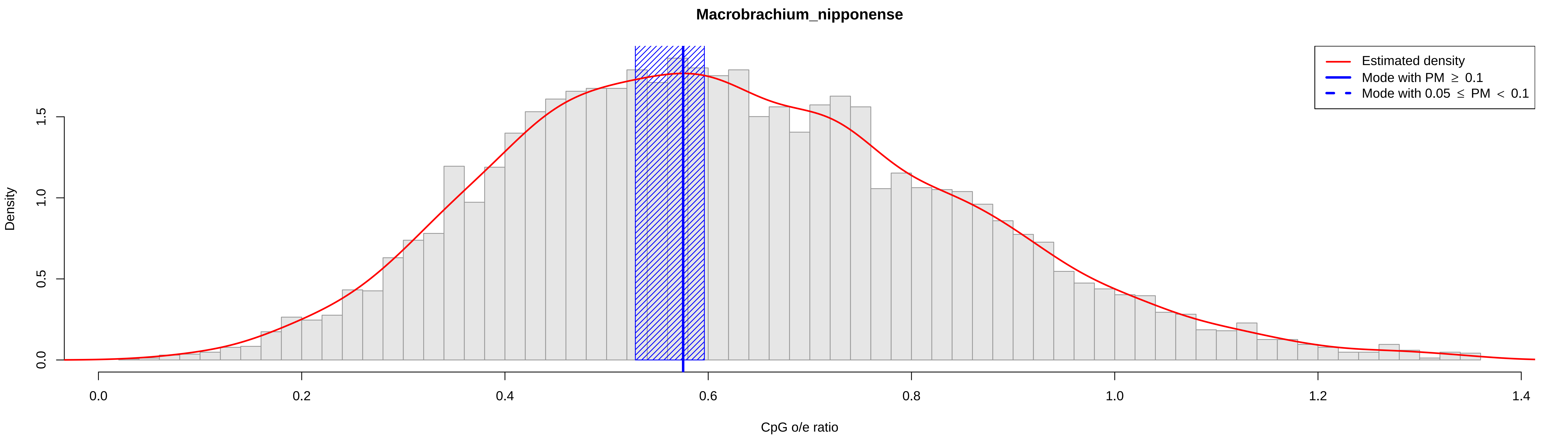
Macrobrachium_nipponense
Estimated density
Mode with PM ≥ 0.1
Mode with 0.05 ≤ PM < 0.1
Density
0.0
0.5
1.0
1.5
0.0
0.2
0.4
0.6
0.8
1.0
1.2
1.4
CpG o/e ratio

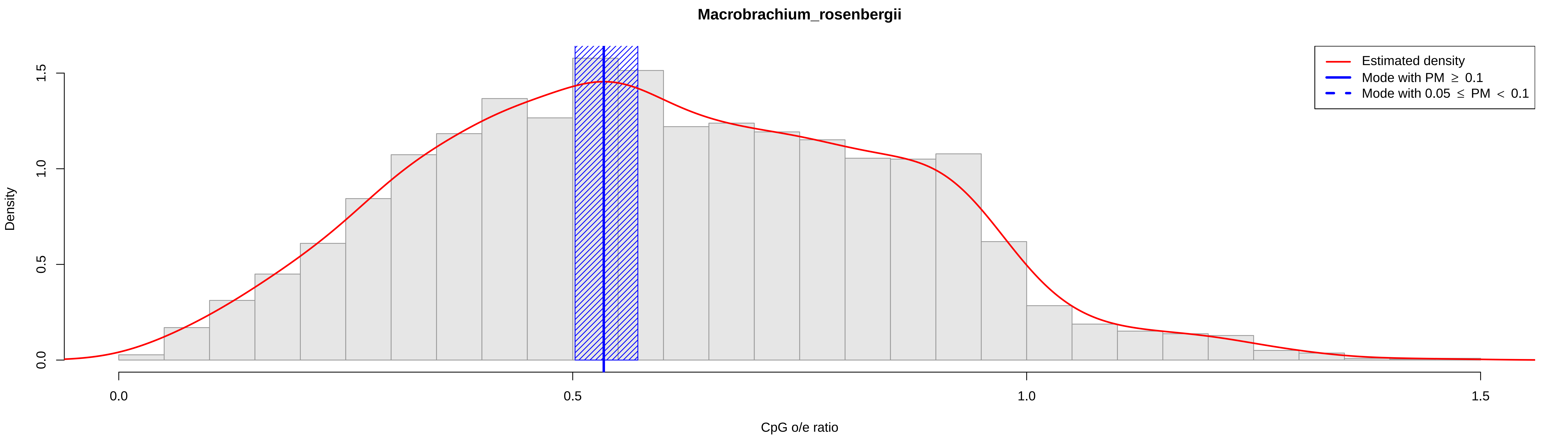

Macrobrachium_rosenbergii
Estimated density
Mode with PM ≥ 0.1
Mode with 0.05 ≤ PM < 0.1
Density
0.0
0.5
1.0
1.5
0.0
0.5
1.0
1.5
CpG o/e ratio

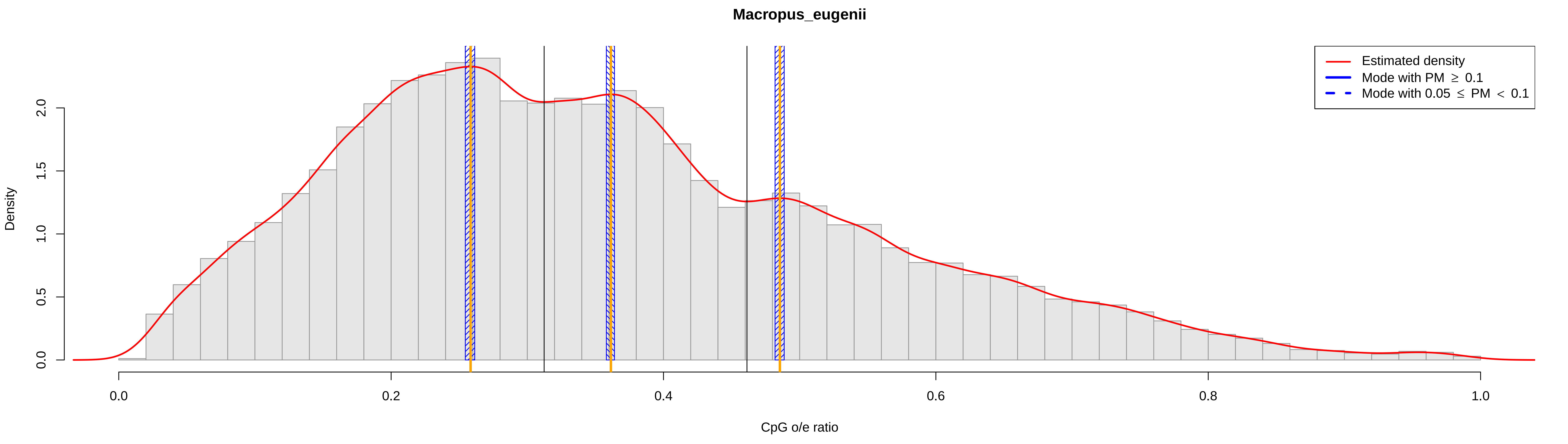
Macropus_eugenii
Estimated density
Mode with PM ≥ 0.1
Mode with 0.05 ≤ PM < 0.1
Density
0.0
0.5
1.0
1.5
2.0
0.0
0.2
0.4
0.6
0.8
1.0
CpG o/e ratio

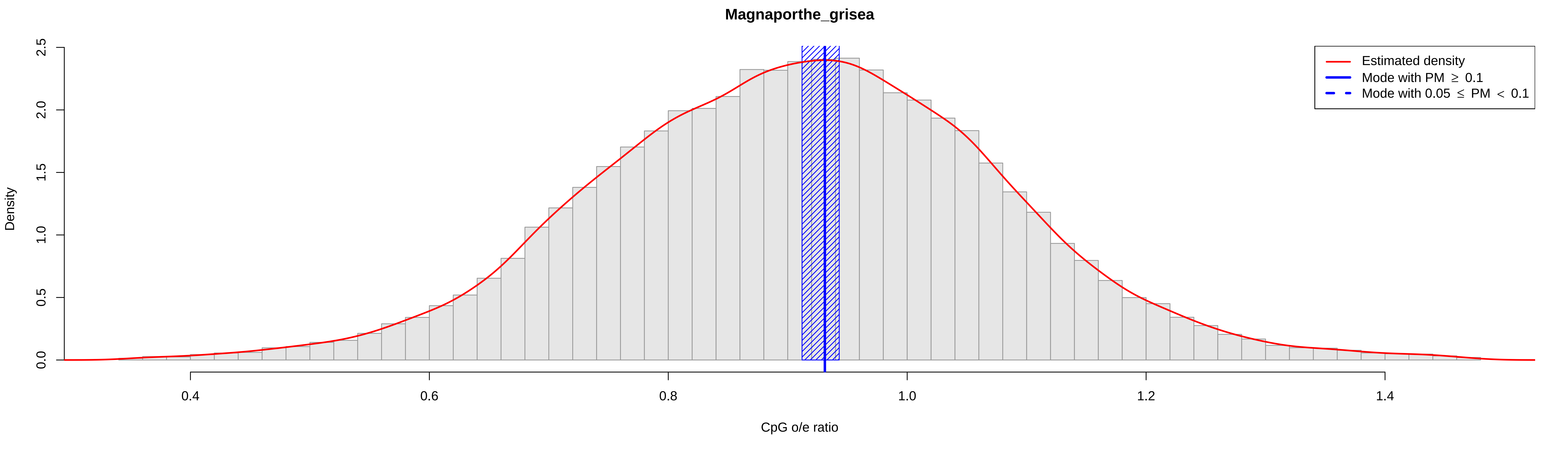
Magnaporthe_grisea
Density
0.0
0.5
1.0
1.5
2.0
2.5
0.4
0.6
0.8
1.0
1.2
1.4
CpG o/e ratio
Estimated density
Mode with PM ≥ 0.1
Mode with 0.05 ≤ PM < 0.1

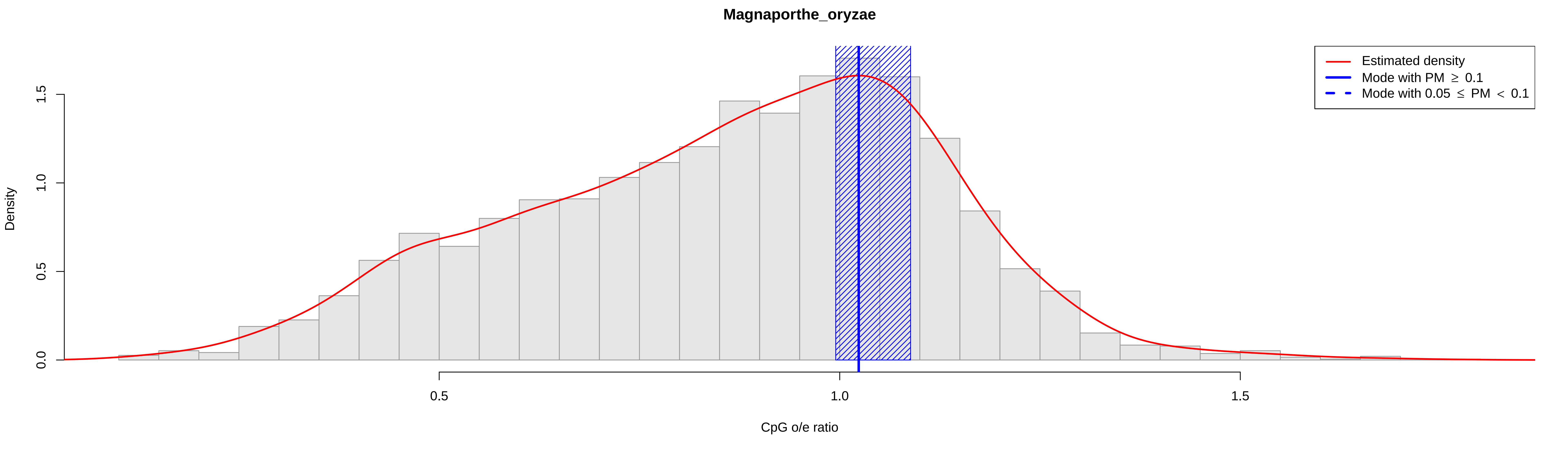
Magnaporthe_oryzae
Estimated density
Mode with PM ≥ 0.1
Mode with 0.05 ≤ PM < 0.1
Density
0.0
0.5
1.0
1.5
0.5
1.0
1.5
CpG o/e ratio

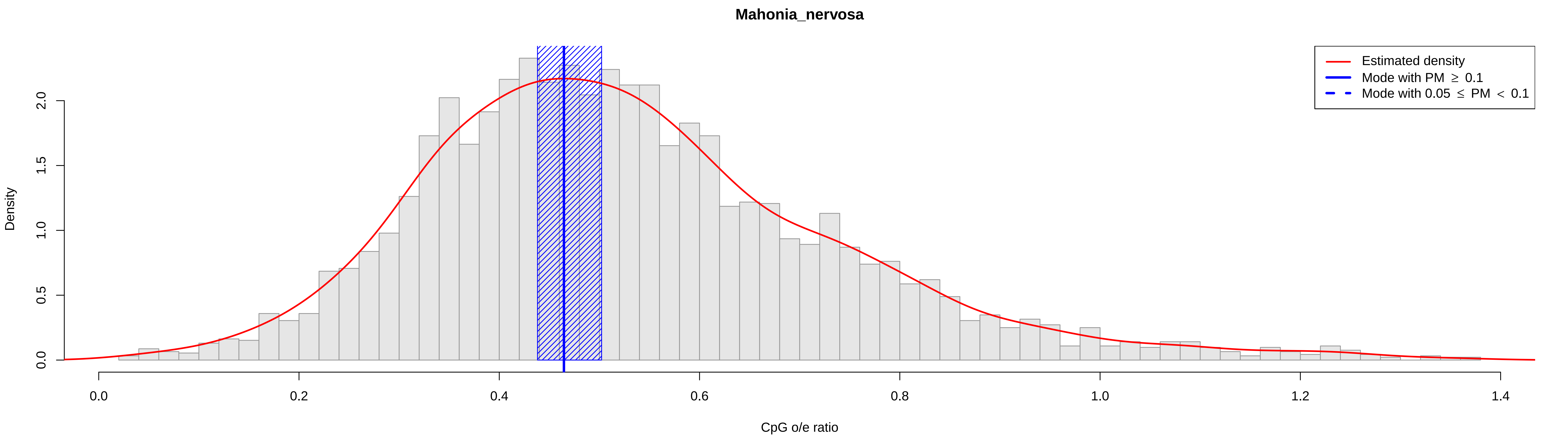

Mahonia_nervosa
Estimated density
Mode with PM ≥ 0.1
Mode with 0.05 ≤ PM < 0.1
Density
0.0
0.5
1.0
1.5
2.0
0.0
0.2
0.4
0.6
0.8
1.0
1.2
1.4
CpG o/e ratio

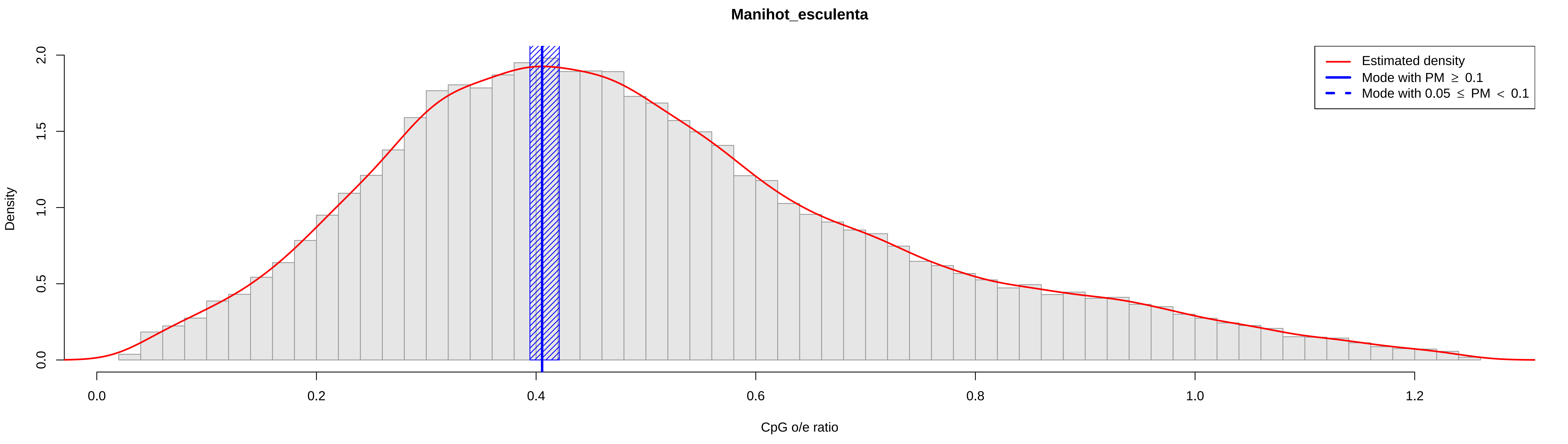
Manihot_esculenta
Estimated density
Mode with PM ≥ 0.1
Mode with 0.05 ≤ PM < 0.1
Density
CpG o/e ratio
0.0
0.5
1.0
1.5
2.0
0.0
0.2
0.4
0.6
0.8
1.0
1.2

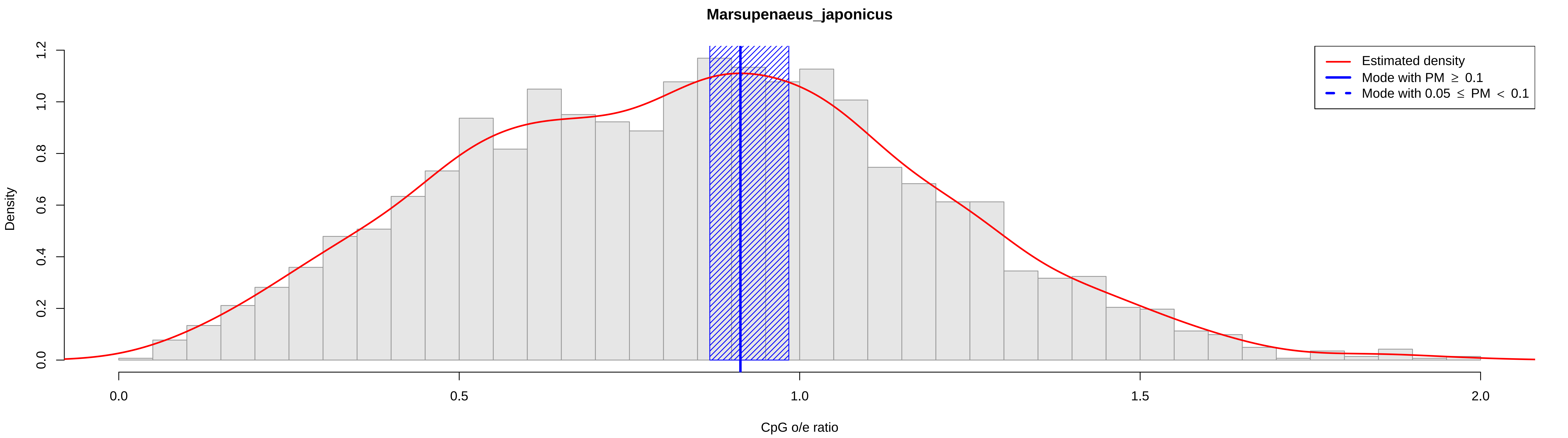

Marsupenaeus_japonicus
Estimated density
Mode with PM ≥ 0.1
Mode with 0.05 ≤ PM < 0.1
Density
0.0
0.2
0.4
0.6
0.8
1.0
1.2
0.0
0.5
1.0
1.5
2.0
CpG o/e ratio

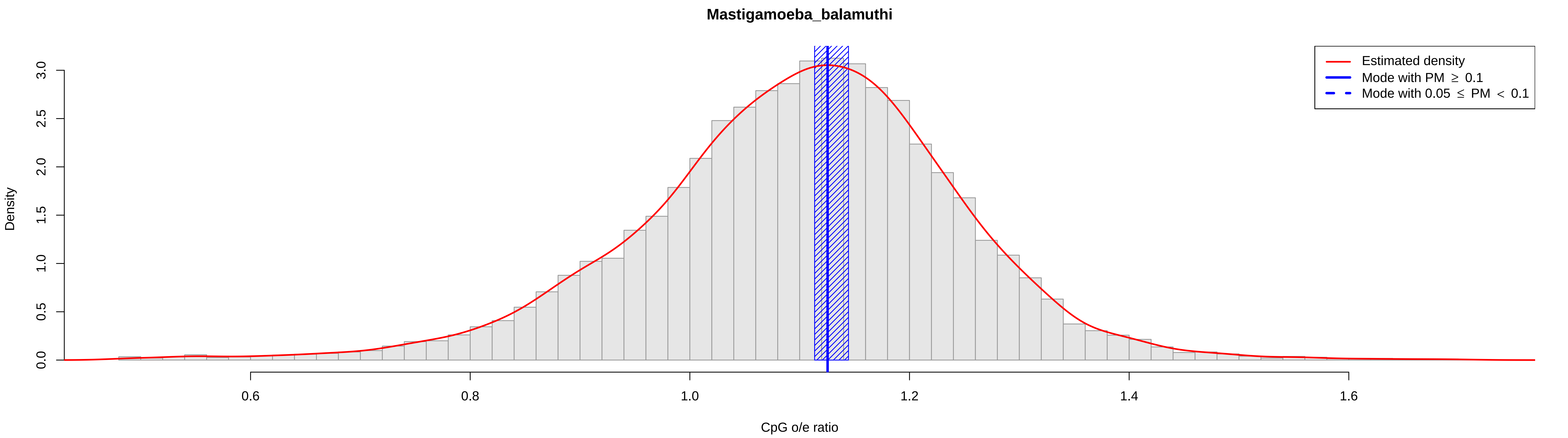

Mastigamoeba_balamuthi
Estimated density
Mode with PM ≥ 0.1
Mode with 0.05 ≤ PM < 0.1
Density
0.0
0.5
1.0
1.5
2.0
2.5
3.0
0.6
0.8
1.0
1.2
1.4
1.6
CpG o/e ratio

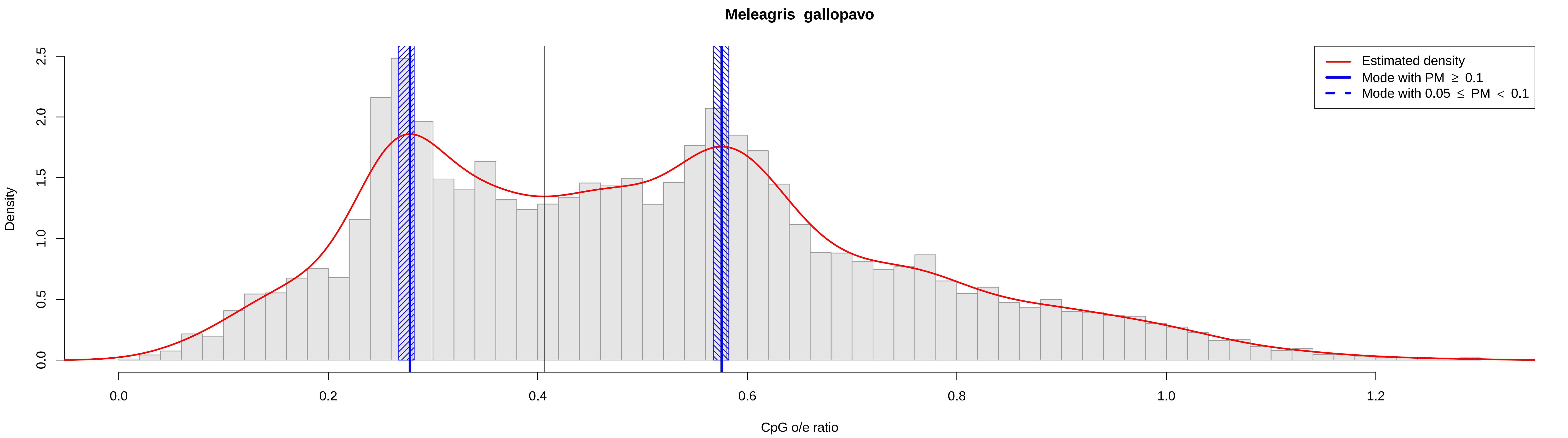

Meleagris_gallopavo
Density
CpG o/e ratio
0.0
0.5
1.0
1.5
2.0
2.5
0.0
0.2
0.4
0.6
0.8
1.0
1.2
Estimated density
Mode with PM ≥ 0.1
Mode with 0.05 ≤ PM < 0.1

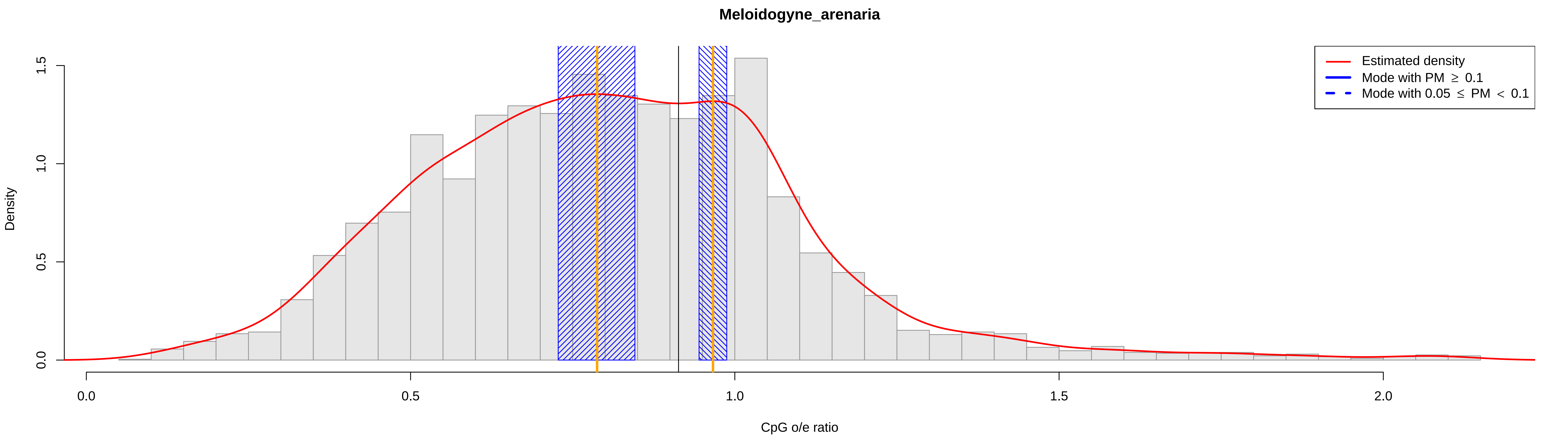

Meloidogyne_arenaria
Density
CpG o/e ratio
0.0
0.5
1.0
1.5
2.0
Estimated density
Mode with PM ≥ 0.1
Mode with 0.05 ≤ PM < 0.1

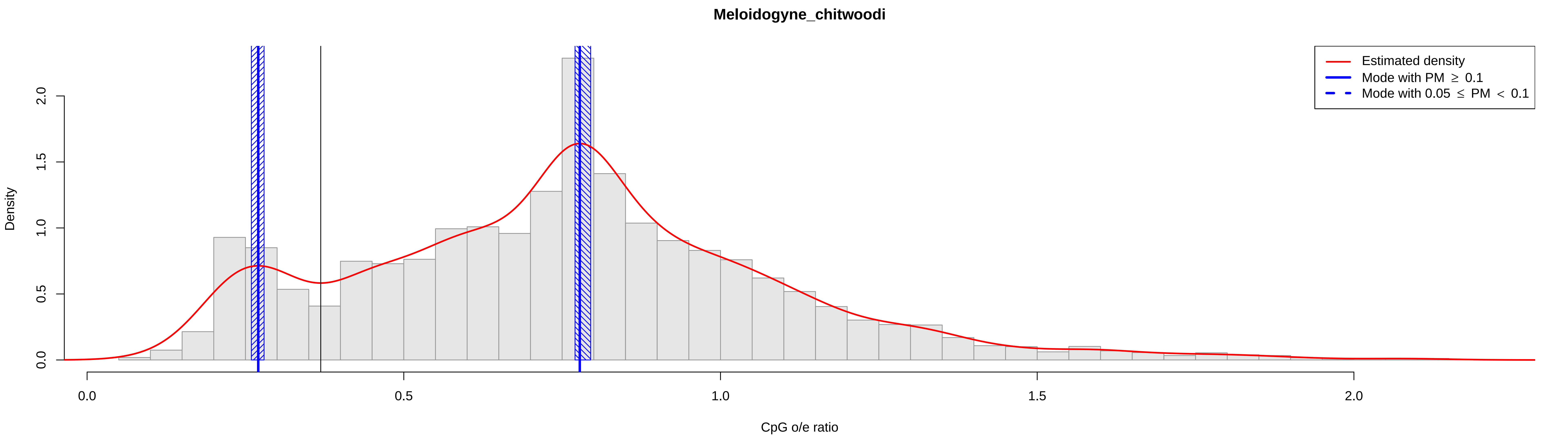

Meloidogyne_chitwoodi
Estimated density
Mode with PM ≥ 0.1
Mode with 0.05 ≤ PM < 0.1
Density
0.0
0.5
1.0
1.5
2.0
CpG o/e ratio

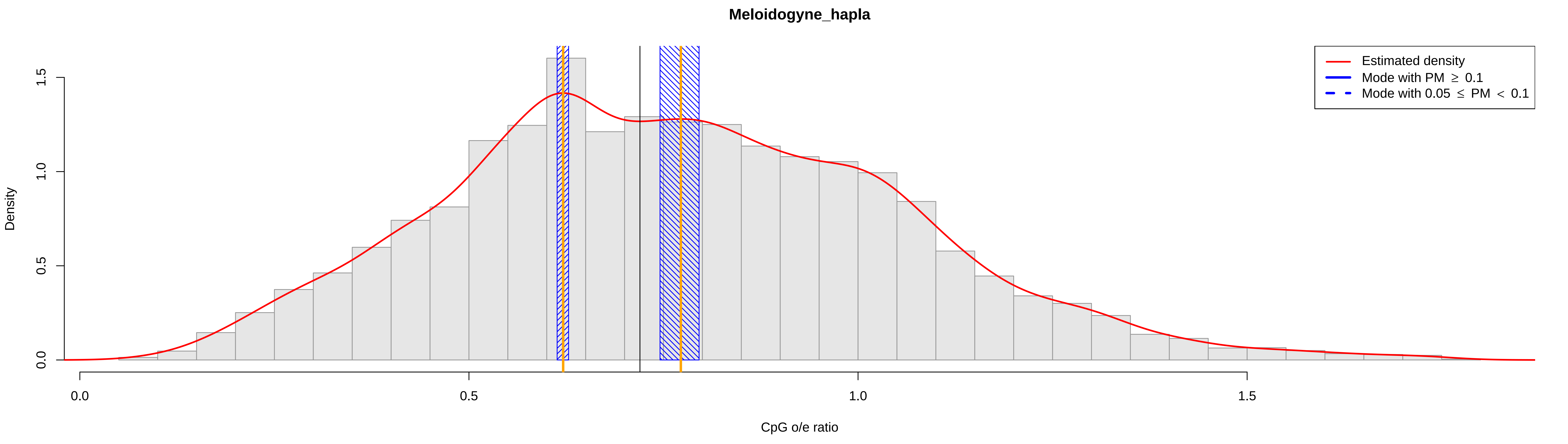

Meloidogyne_hapla
Estimated density
Mode with PM ≥ 0.1
Mode with 0.05 ≤ PM < 0.1
Density
0.0
0.5
1.0
1.5
0.0
0.5
1.0
1.5
CpG o/e ratio

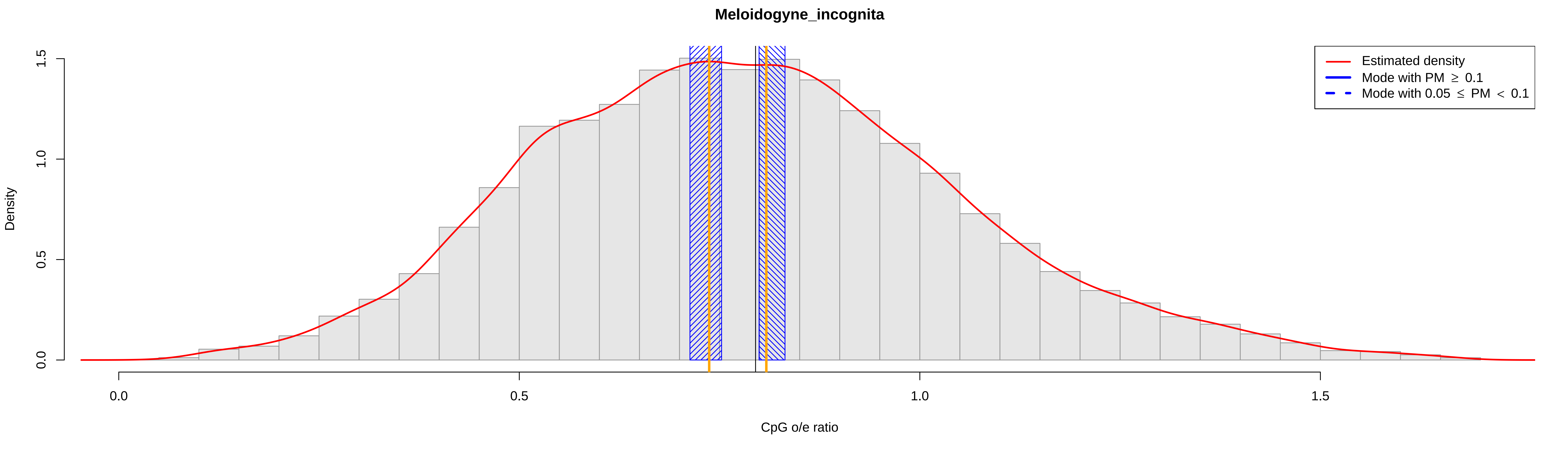

Meloidogyne_incognita
Estimated density
Mode with PM ≥ 0.1
Mode with 0.05 ≤ PM < 0.1
Density
0.0
0.5
1.0
1.5
CpG o/e ratio

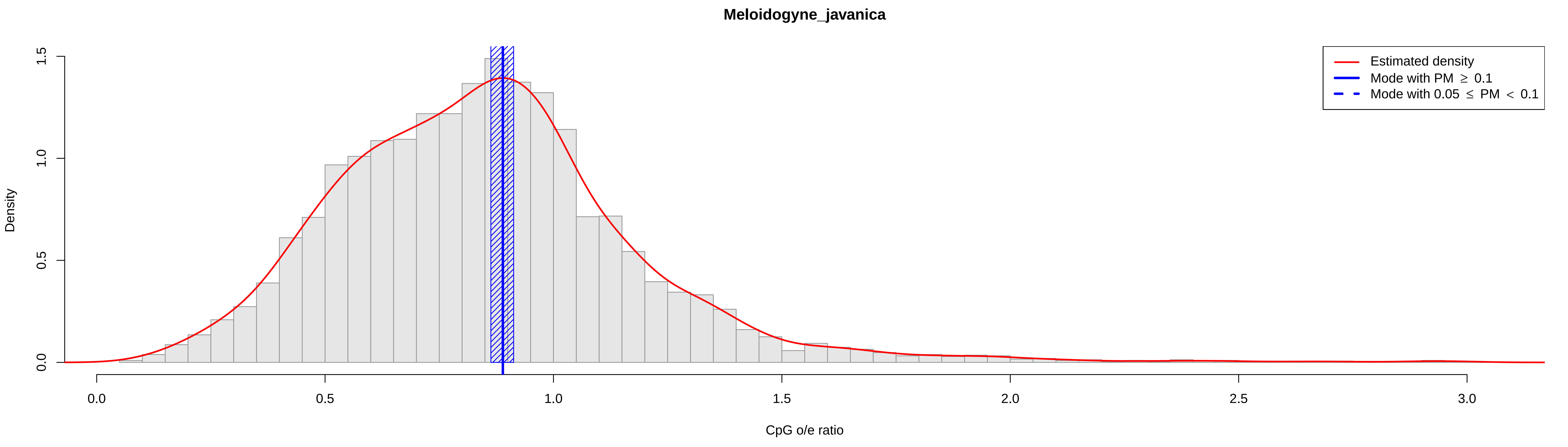

Meloidogyne_javanica
Estimated density
Mode with PM ≥ 0.1
Mode with 0.05 ≤ PM < 0.1
Density
0.0
0.5
1.0
1.5
0.0
0.5
1.0
1.5
2.0
2.5
3.0
CpG o/e ratio

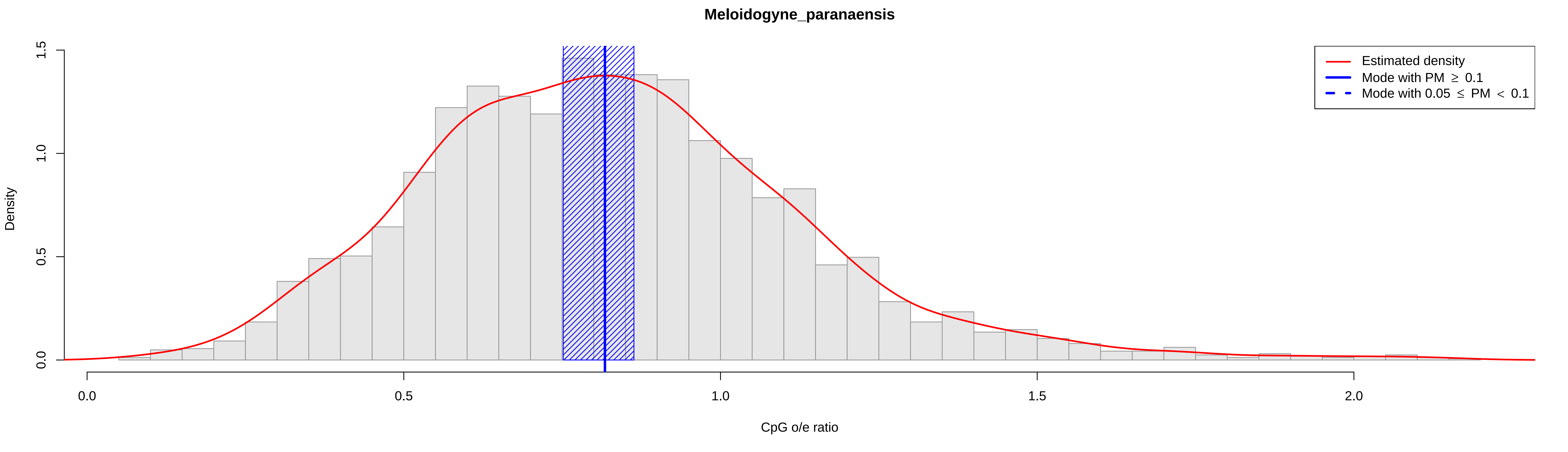

Meloidogyne_paranaensis
Estimated density
Mode with PM ≥ 0.1
Mode with 0.05 ≤ PM < 0.1
Density
0.0
0.5
1.0
1.5
0.0
0.5
1.0
1.5
2.0
CpG o/e ratio

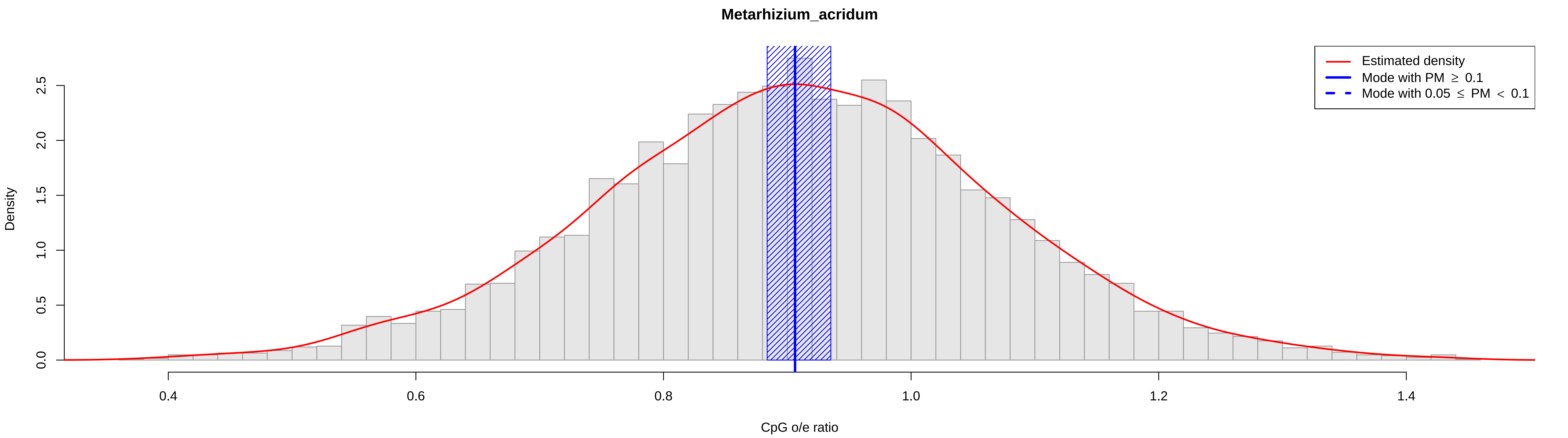

Metarhizium_acridum
Estimated density
Mode with PM ≥ 0.1
Mode with 0.05 ≤ PM < 0.1
Density
0.0
0.5
1.0
1.5
2.0
2.5
0.4
0.6
0.8
1.0
1.2
1.4
CpG o/e ratio

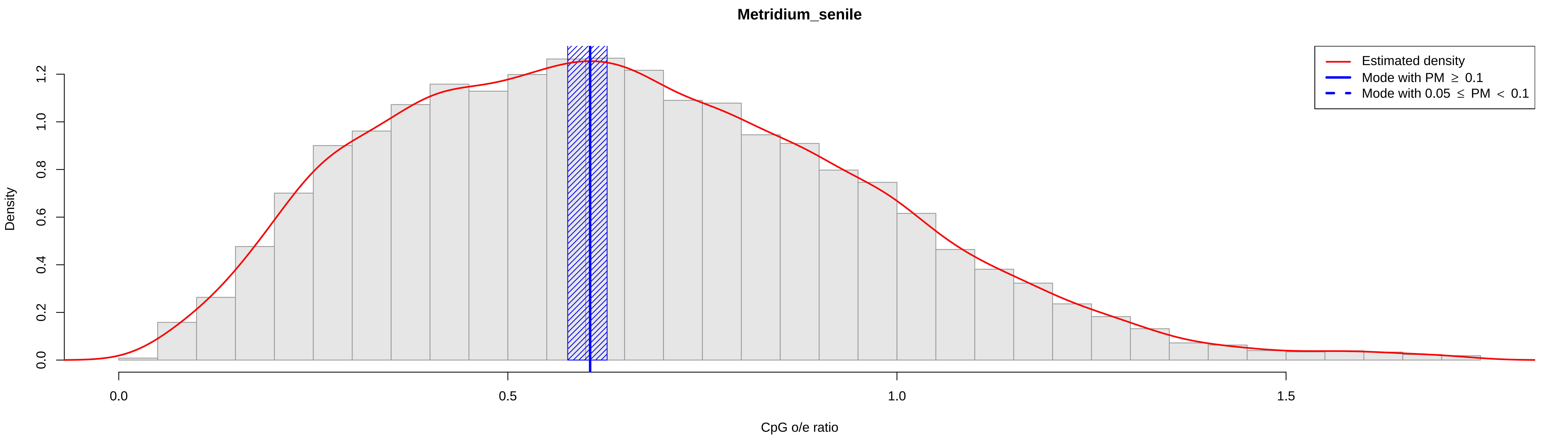

Metridium_senile
Estimated density
Mode with PM ≥ 0.1
Mode with 0.05 ≤ PM < 0.1
Density
0.0
0.2
0.4
0.6
0.8
1.0
1.2
0.0
0.5
1.0
1.5
CpG o/e ratio

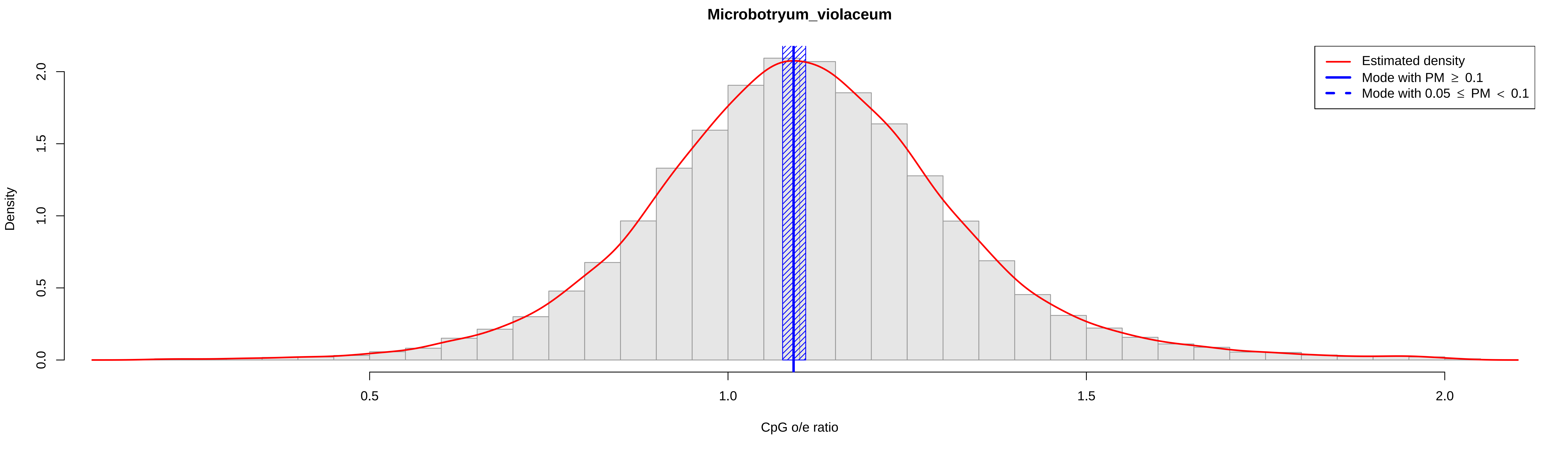
Microbotryum_violaceum
Estimated density
Mode with PM ≥ 0.1
Mode with 0.05 ≤ PM < 0.1
Density
0.0
0.5
1.0
1.5
2.0
0.5
1.0
1.5
2.0
CpG o/e ratio

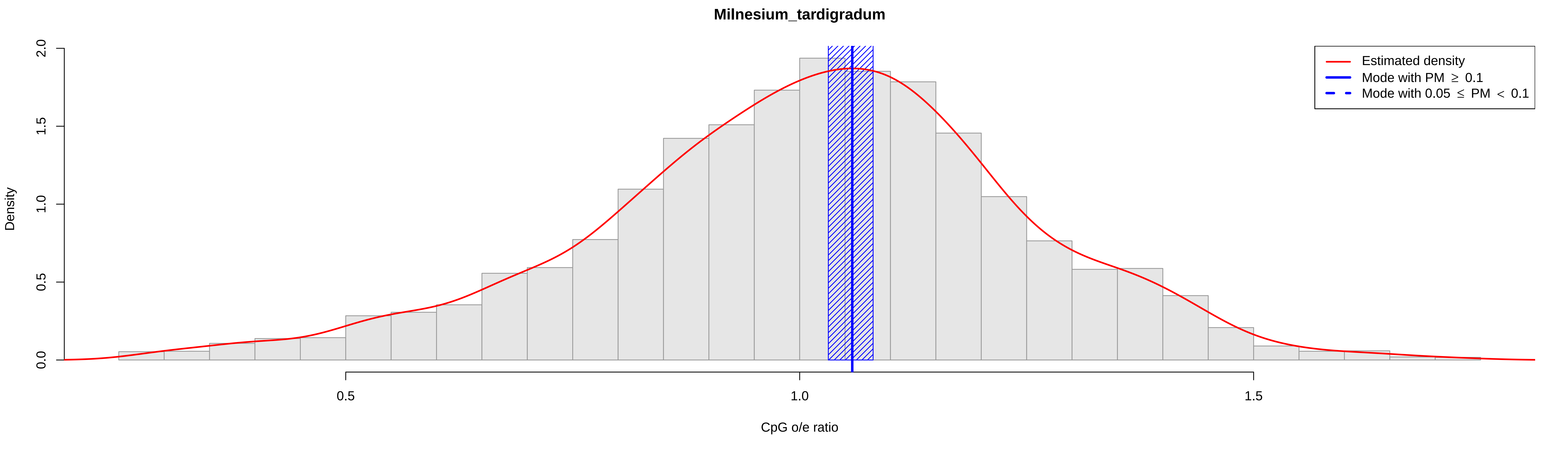
Milnesium_tardigradum
Estimated density
Mode with PM ≥ 0.1
Mode with 0.05 ≤ PM < 0.1
Density
0.0
0.5
1.0
1.5
2.0
0.5
1.0
1.5
CpG o/e ratio

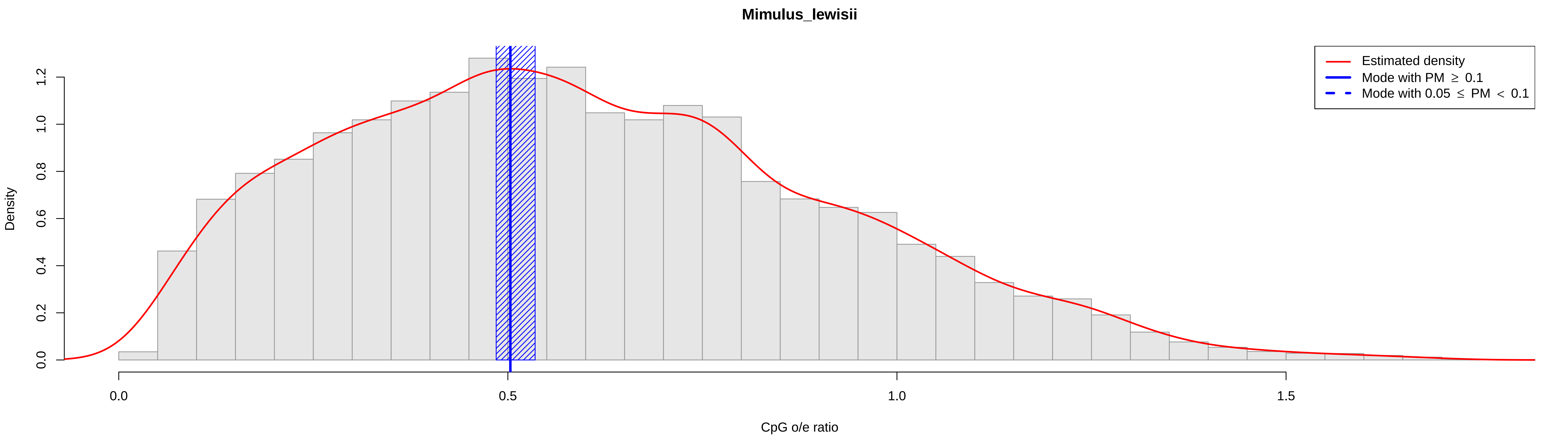
Mimulus_lewisii
Estimated density
Mode with PM ≥ 0.1
Mode with 0.05 ≤ PM < 0.1
Density
0.0
0.2
0.4
0.6
0.8
1.0
1.2
0.0
0.5
1.0
1.5
CpG o/e ratio

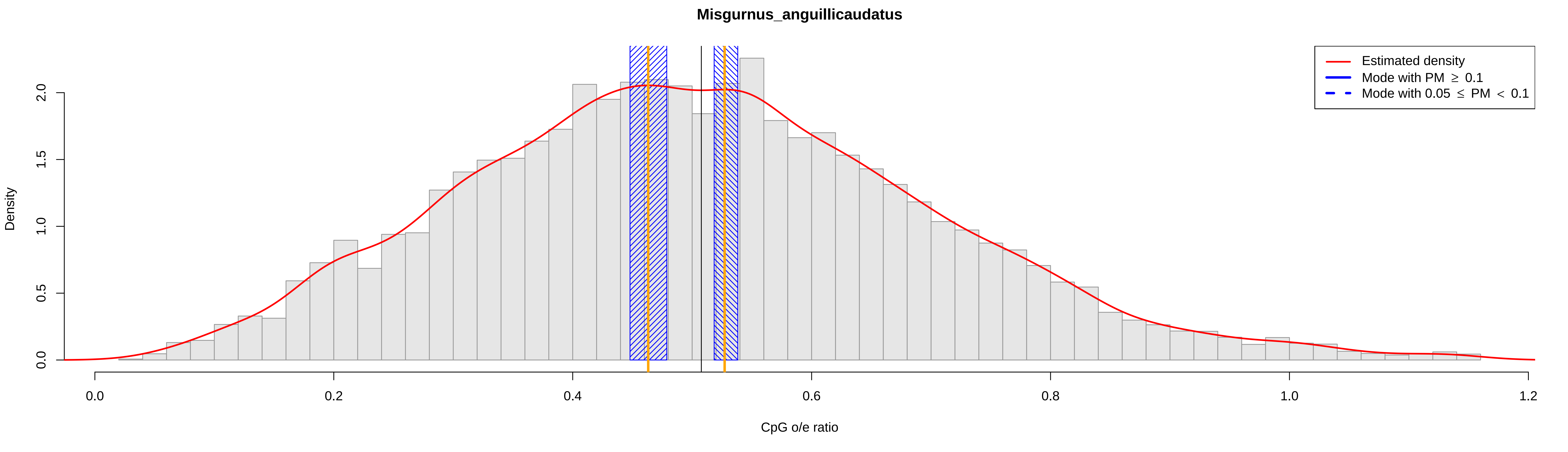
Misgurnus_anguillicaudatus
Estimated density
Mode with PM ≥ 0.1
Mode with 0.05 ≤ PM < 0.1
Density
0.0
0.5
1.0
1.5
2.0
0.0
0.2
0.4
0.6
0.8
1.0
1.2
CpG o/e ratio

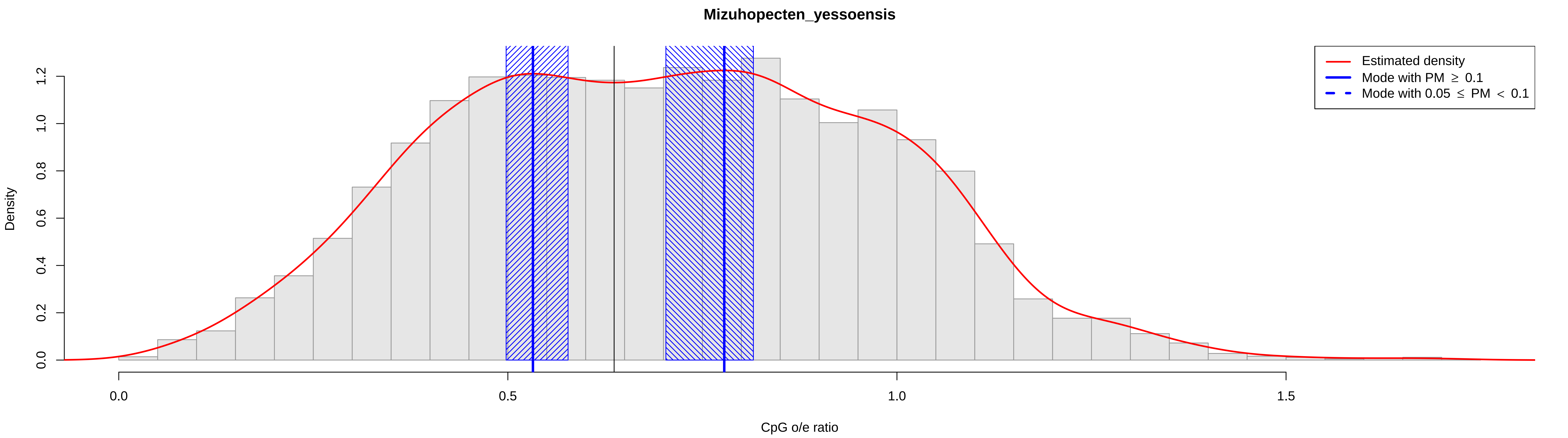
Mizuhopecten_yessoensis
Estimated density
Mode with PM ≥ 0.1
Mode with 0.05 ≤ PM < 0.1
Density
0.0
0.2
0.4
0.6
0.8
1.0
1.2
0.0
0.5
1.0
1.5
CpG o/e ratio

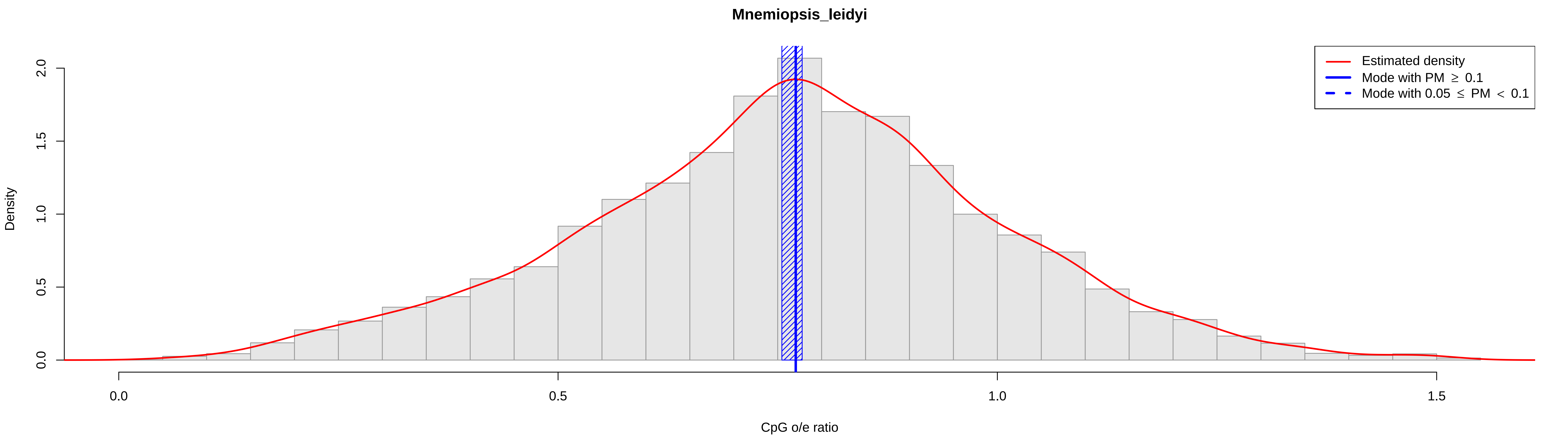

Mnemiopsis_leidyi
Estimated density
Mode with PM ≥ 0.1
Mode with 0.05 ≤ PM < 0.1
Density
0.0
0.5
1.0
1.5
2.0
0.0
0.5
1.0
1.5
CpG o/e ratio

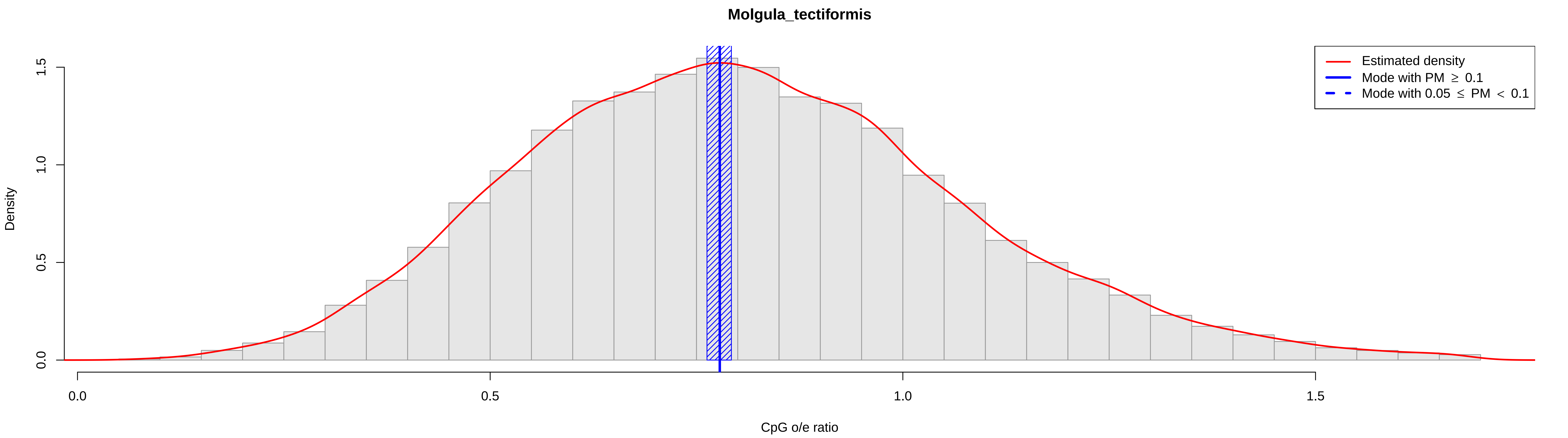
Molgula_tectiformis
Estimated density
Mode with PM ≥ 0.1
Mode with 0.05 ≤ PM < 0.1
Density
0.0
0.5
1.0
1.5
CpG o/e ratio

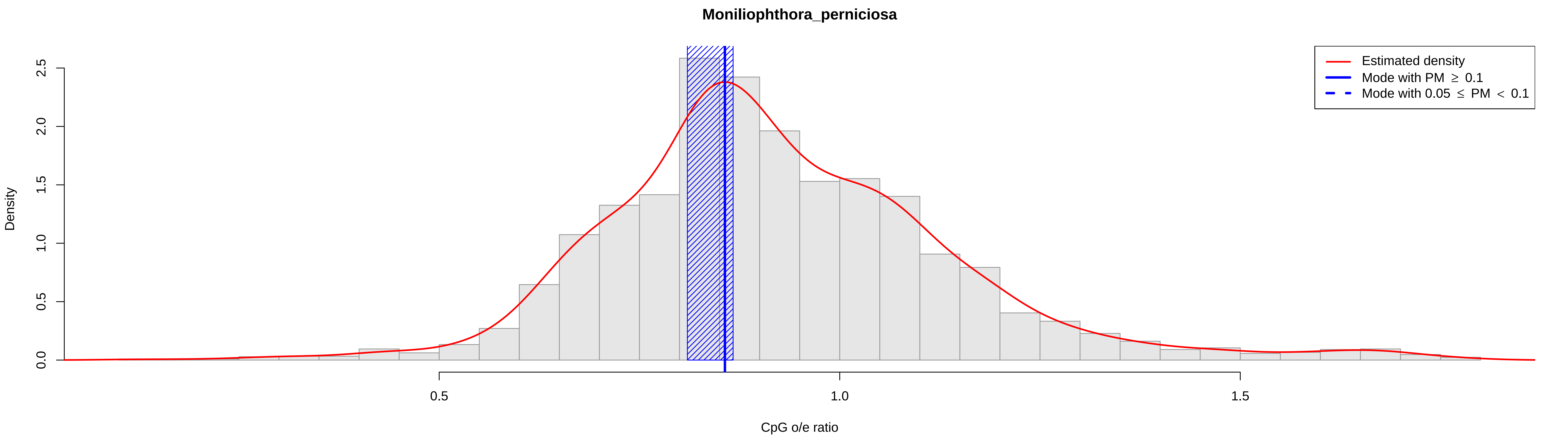
Moniliophthora_perniciosa
Estimated density
Mode with PM ≥ 0.1
Mode with 0.05 ≤ PM < 0.1
Density
0.0
0.5
1.0
1.5
2.0
2.5
0.5
1.0
1.5
CpG o/e ratio

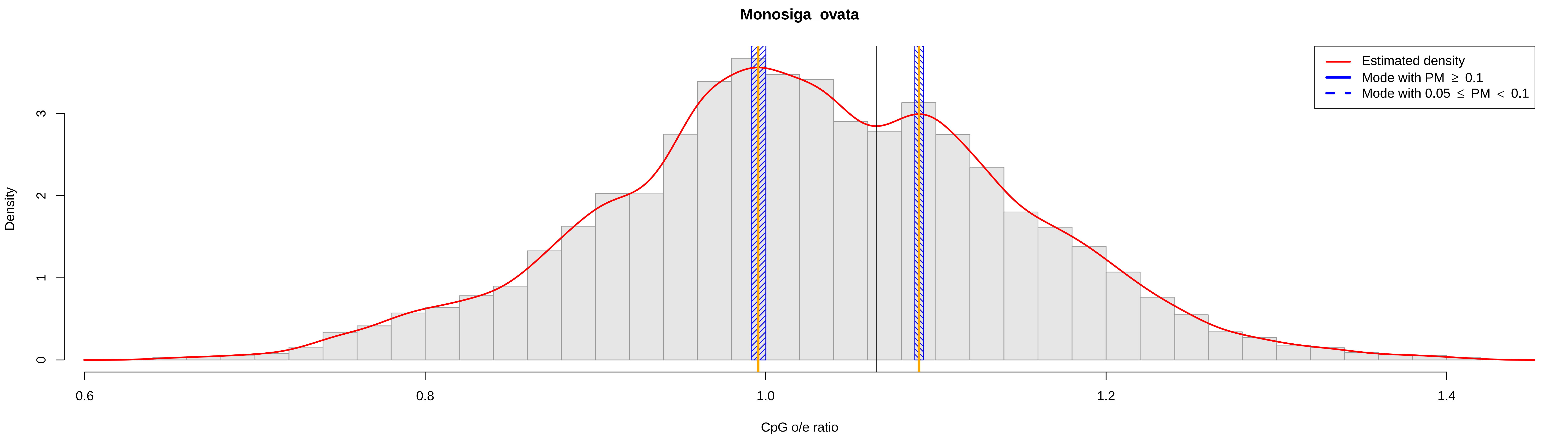
Monosiga_ovata
Estimated density
Mode with PM ≥ 0.1
Mode with 0.05 ≤ PM < 0.1
Density
CpG o/e ratio
0
1
2
3
0.6
0.8
1.0
1.2
1.4

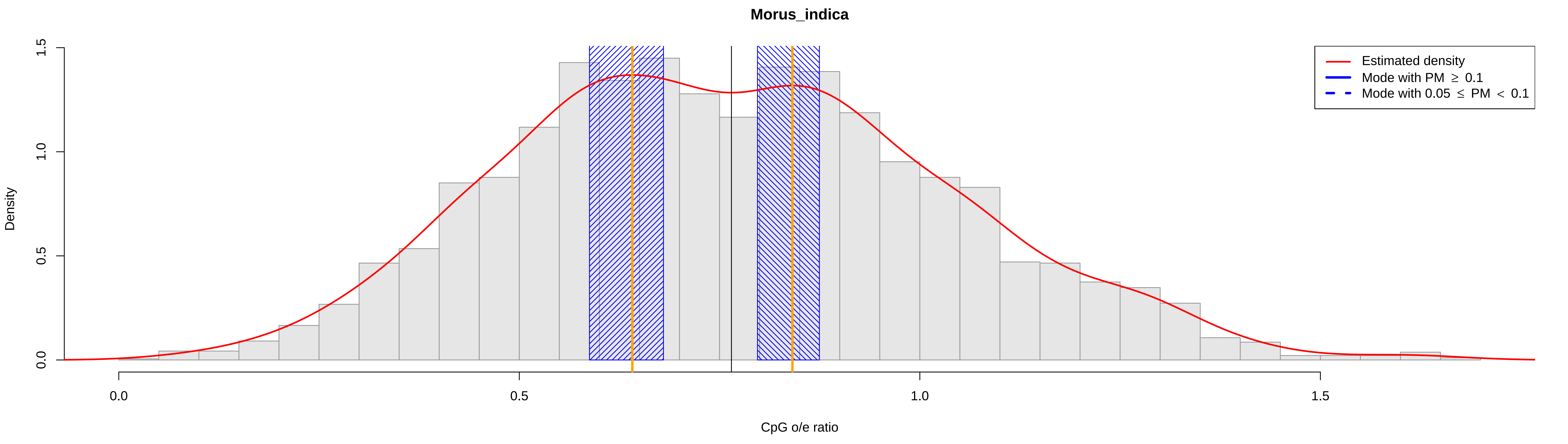

Morus_indica
Estimated density
Mode with PM ≥ 0.1
Mode with 0.05 ≤ PM < 0.1
Density
0.0
0.5
1.0
1.5
0.0
0.5
1.0
1.5
CpG o/e ratio

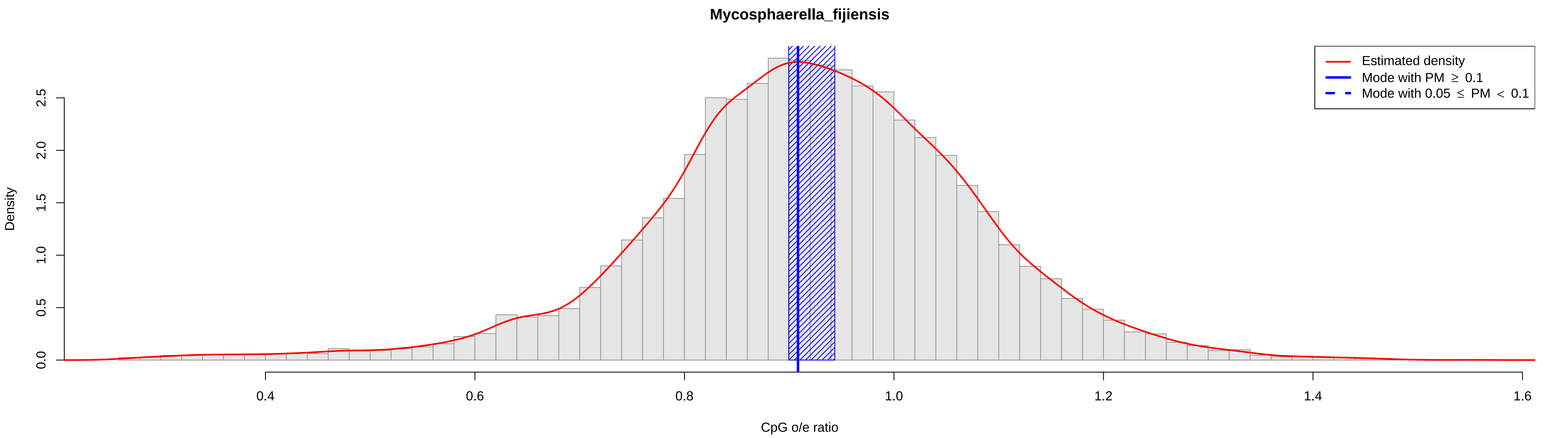

Mycosphaerella_fijiensis
Estimated density
Mode with PM ≥ 0.1
Mode with 0.05 ≤ PM < 0.1
Density
0.0
0.5
1.0
1.5
2.0
2.5
0.4
0.6
0.8
1.0
1.2
1.4
1.6
CpG o/e ratio

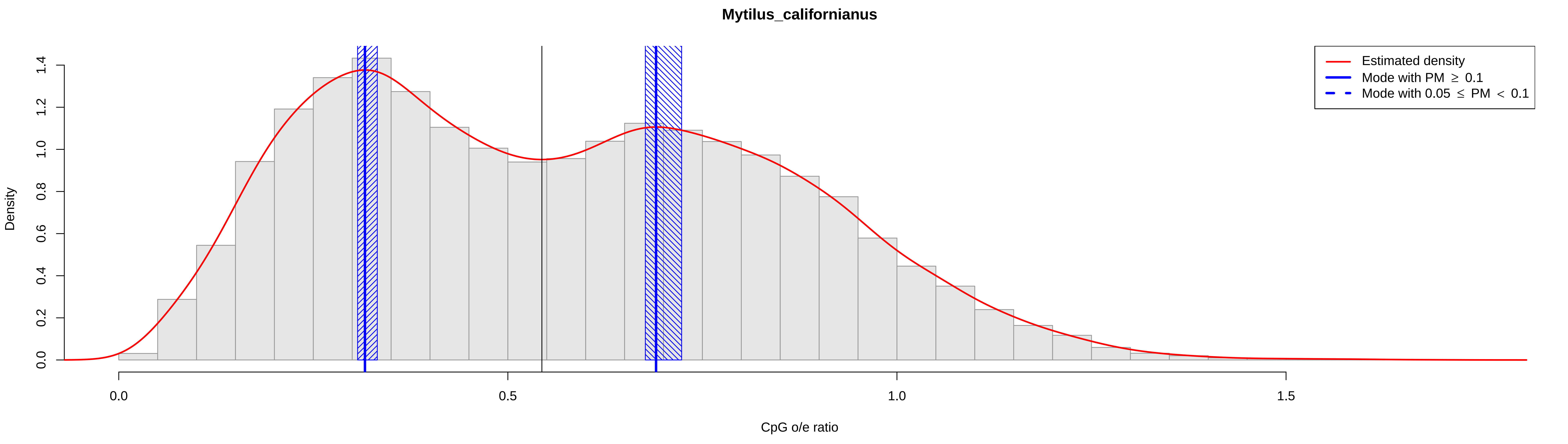
Mytilus_californianus
Estimated density
Mode with PM ≥ 0.1
Mode with 0.05 ≤ PM < 0.1
Density
0.0
0.2
0.4
0.6
0.8
1.0
1.2
1.4
0.0
0.5
1.0
1.5
CpG o/e ratio

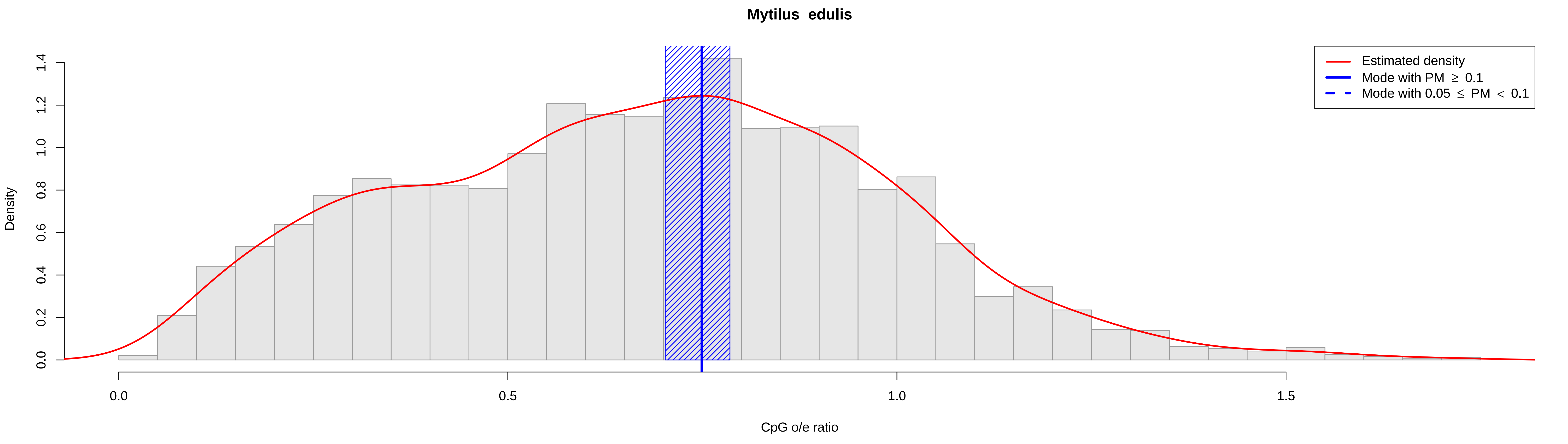
Mytilus_edulis
Estimated density
Mode with PM ≥ 0.1
Mode with 0.05 ≤ PM < 0.1
Density
0.0
0.2
0.4
0.6
0.8
1.0
1.2
1.4
0.0
0.5
1.0
1.5
CpG o/e ratio

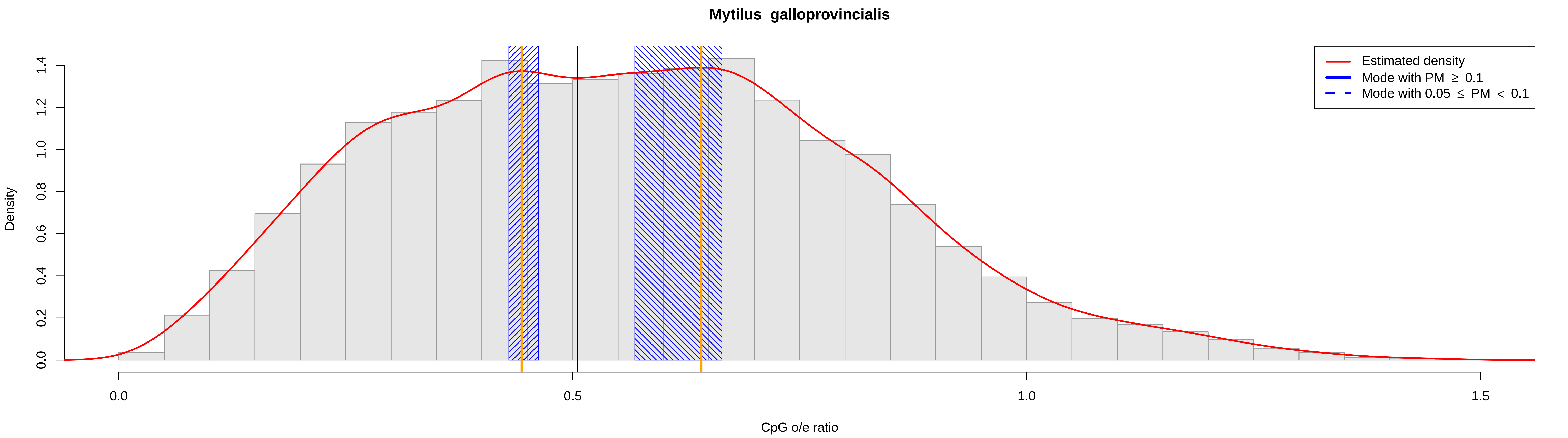

Mytilus_galloprovincialis
Estimated density
Mode with PM ≥ 0.1
Mode with 0.05 ≤ PM < 0.1
Density
0.0
0.2
0.4
0.6
0.8
1.0
1.2
1.4
0.0
0.5
1.0
1.5
CpG o/e ratio

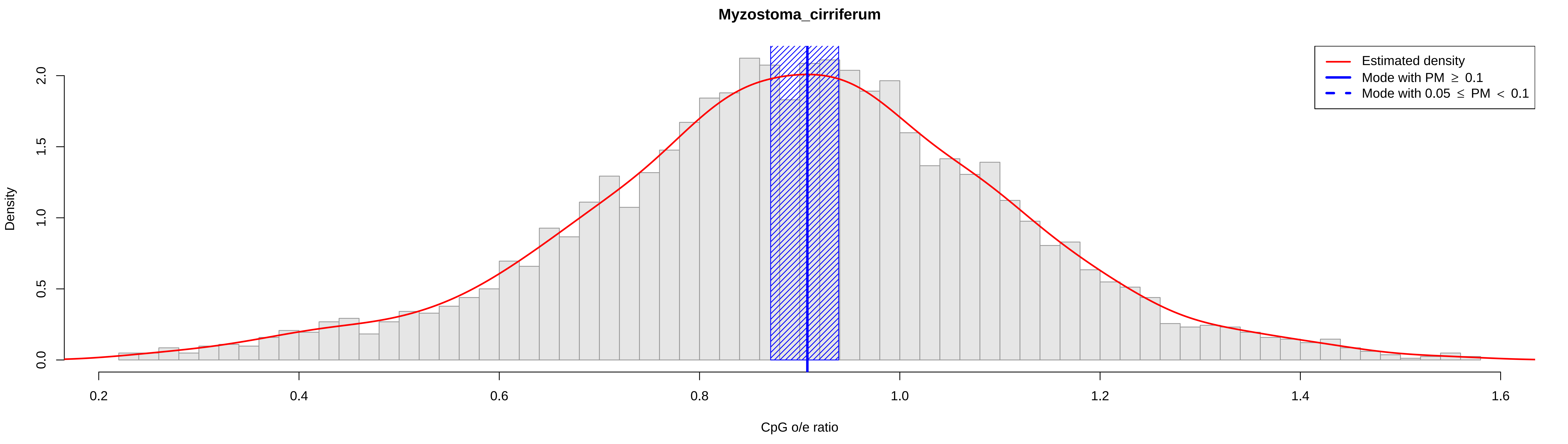

Myzostoma_cirriferum
Estimated density
Mode with PM ≥ 0.1
Mode with 0.05 ≤ PM < 0.1
Density
0.0
0.5
1.0
1.5
2.0
0.2
0.4
0.6
0.8
1.0
1.2
1.4
1.6
CpG o/e ratio

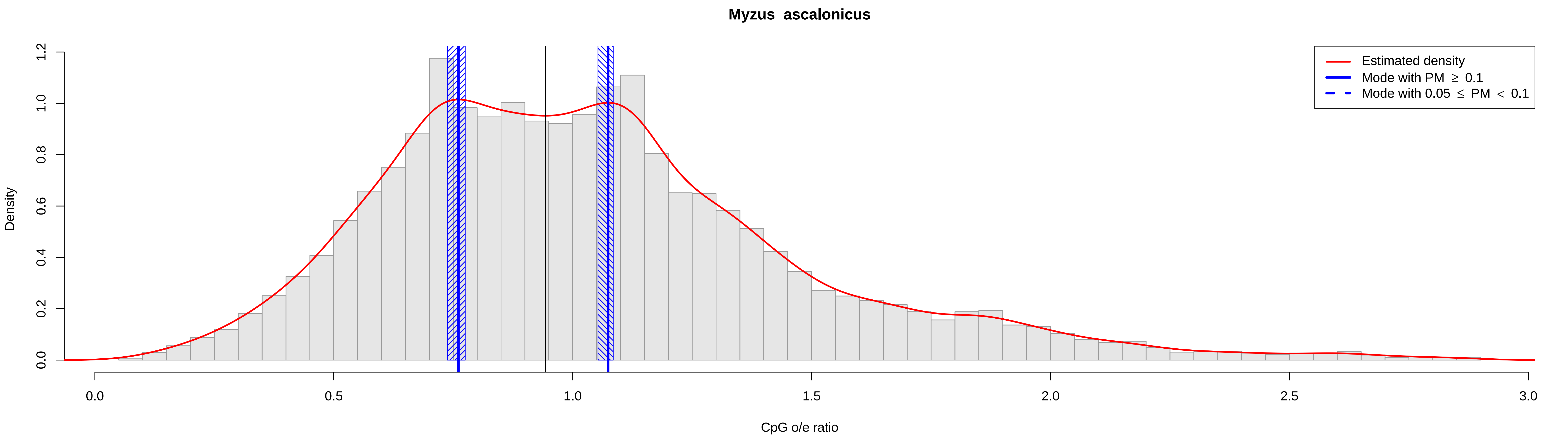

Myzus_ascalonicus
Estimated density
Mode with PM ≥ 0.1
Mode with 0.05 ≤ PM < 0.1
Density
0.0
0.2
0.4
0.6
0.8
1.0
1.2
0.0
0.5
1.0
1.5
2.0
2.5
3.0
CpG o/e ratio

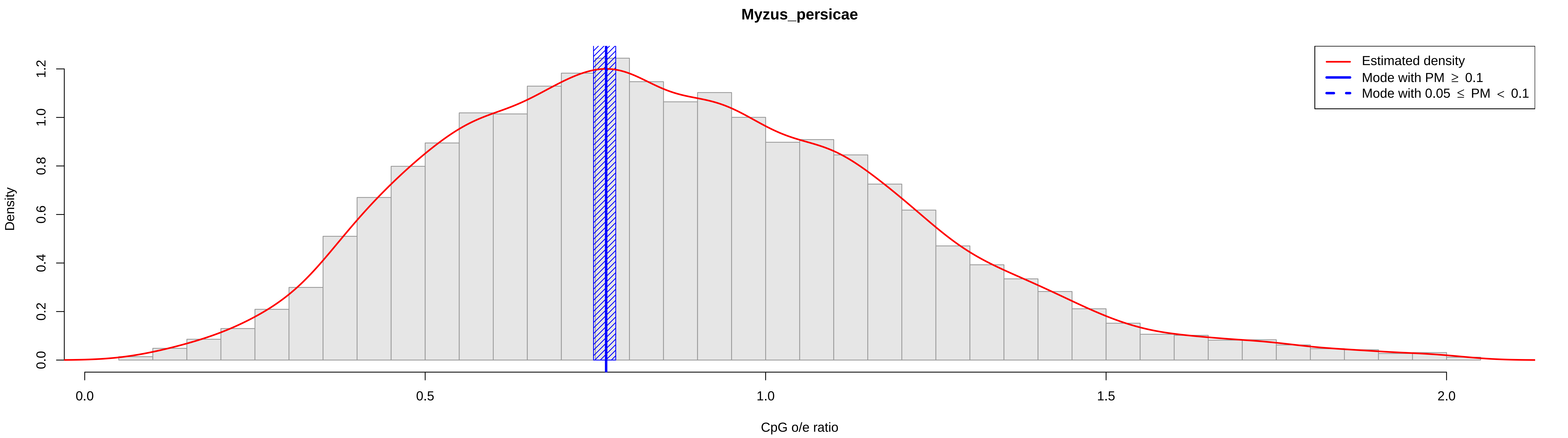
Myzus_persicae
Estimated density
Mode with PM ≥ 0.1
Mode with 0.05 ≤ PM < 0.1
Density
0.0
0.2
0.4
0.6
0.8
1.0
1.2
0.0
0.5
1.0
1.5
2.0
CpG o/e ratio

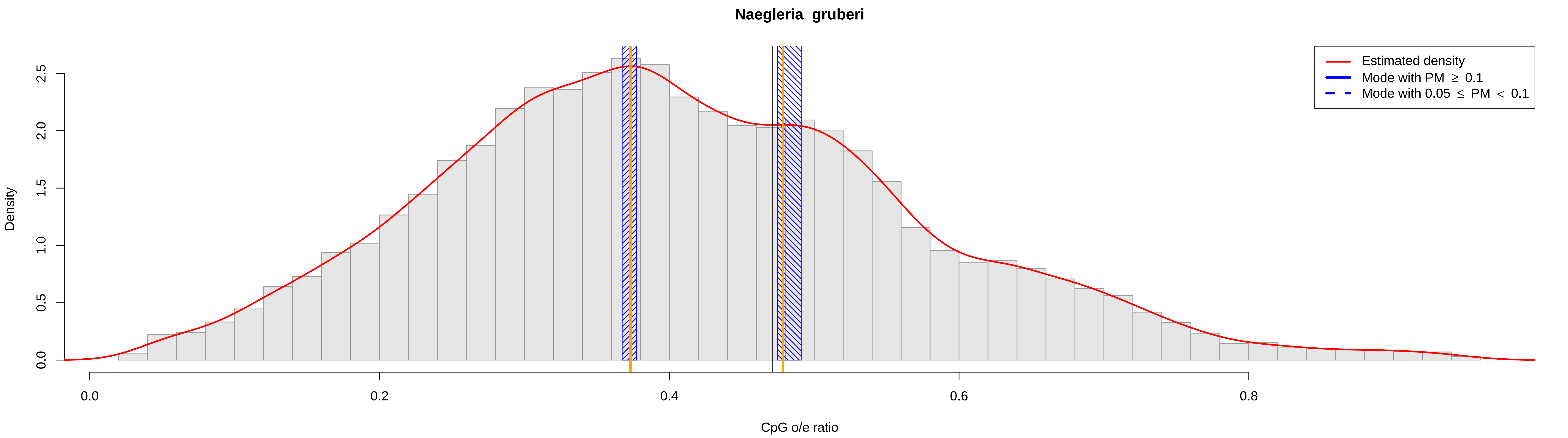
Naegleria_gruberi
Estimated density
Mode with PM ≥ 0.1
Mode with 0.05 ≤ PM < 0.1
Density
0.0
0.5
1.0
1.5
2.0
2.5
0.0
0.2
0.4
0.6
0.8
CpG o/e ratio

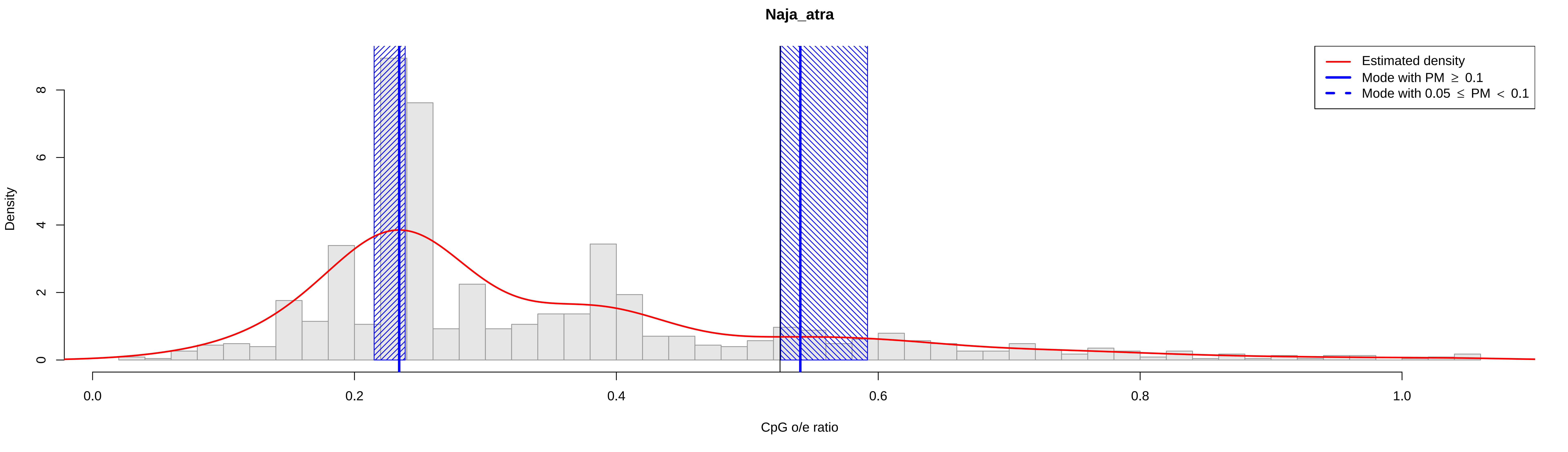

Naja_atra
Estimated density
Mode with PM ≥ 0.1
Mode with 0.05 ≤ PM < 0.1
Density
0
2
4
6
8
0.0
0.2
0.4
0.6
0.8
1.0
CpG o/e ratio

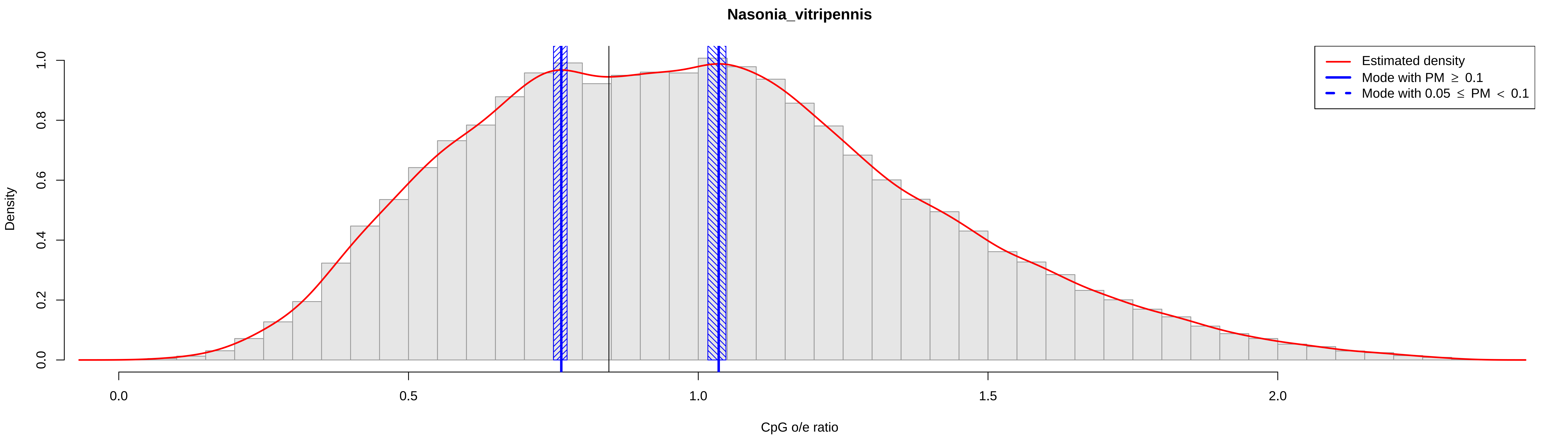
Nasonia_vitripennis
Estimated density
Mode with PM ≥ 0.1
Mode with 0.05 ≤ PM < 0.1
Density
0.0
0.2
0.4
0.6
0.8
1.0
0.0
0.5
1.0
1.5
2.0
CpG o/e ratio

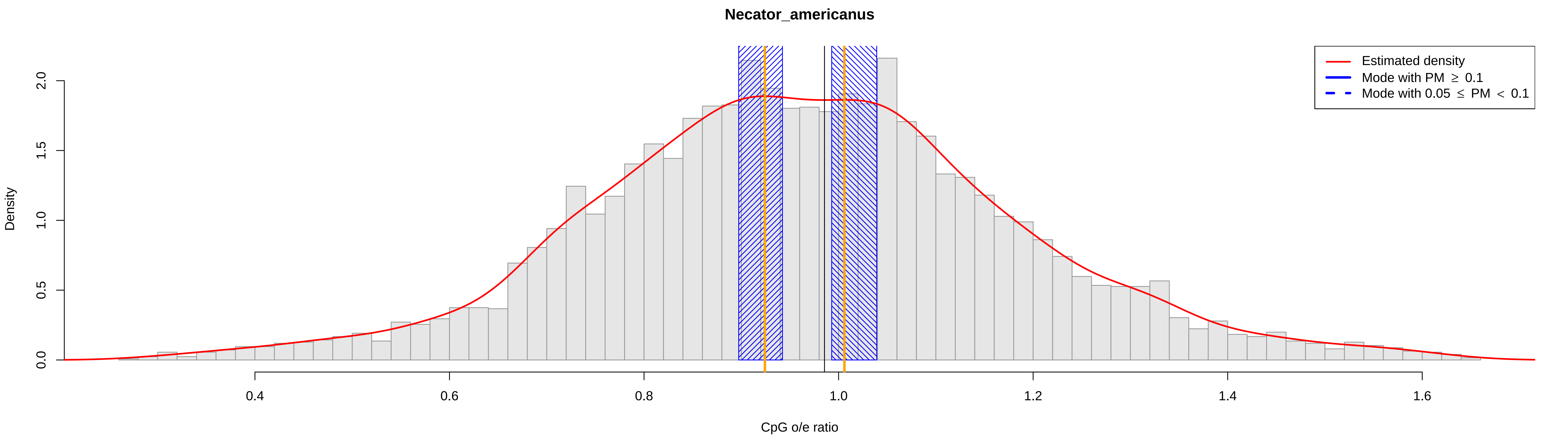

Necator_americanus
Estimated density
Mode with PM ≥ 0.1
Mode with 0.05 ≤ PM < 0.1
Density
0.0
0.5
1.0
1.5
2.0
0.4
0.6
0.8
1.0
1.2
1.4
1.6
CpG o/e ratio

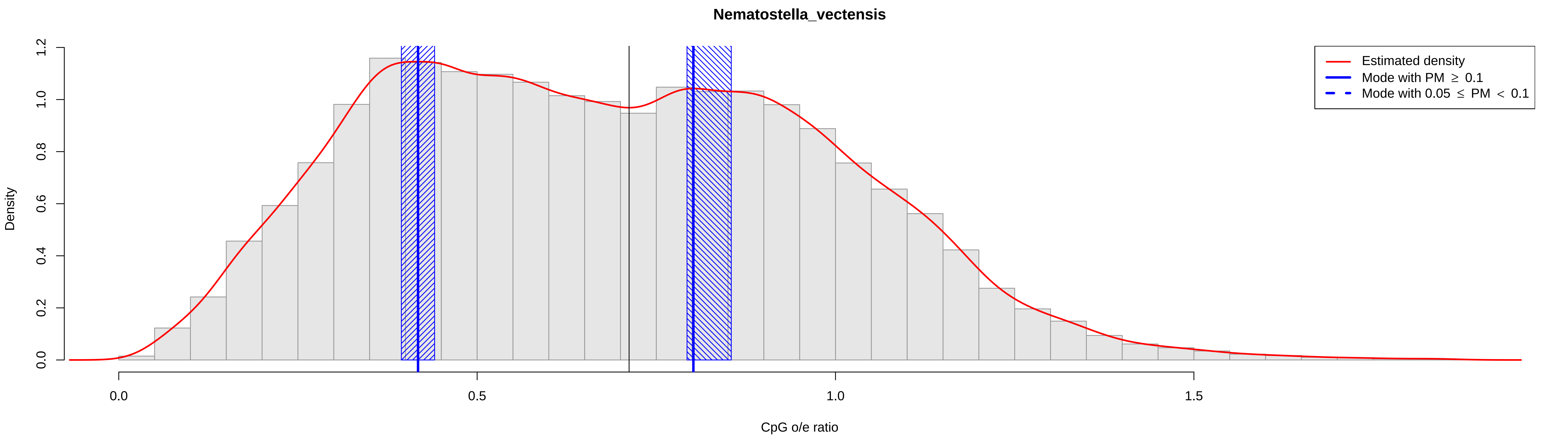
Nematostella_vectensis
Density
CpG o/e ratio
0.0
0.2
0.4
0.6
0.8
1.0
1.2
0.0
0.5
1.0
1.5
Estimated density
Mode with PM ≥ 0.1
Mode with 0.05 ≤ PM < 0.1

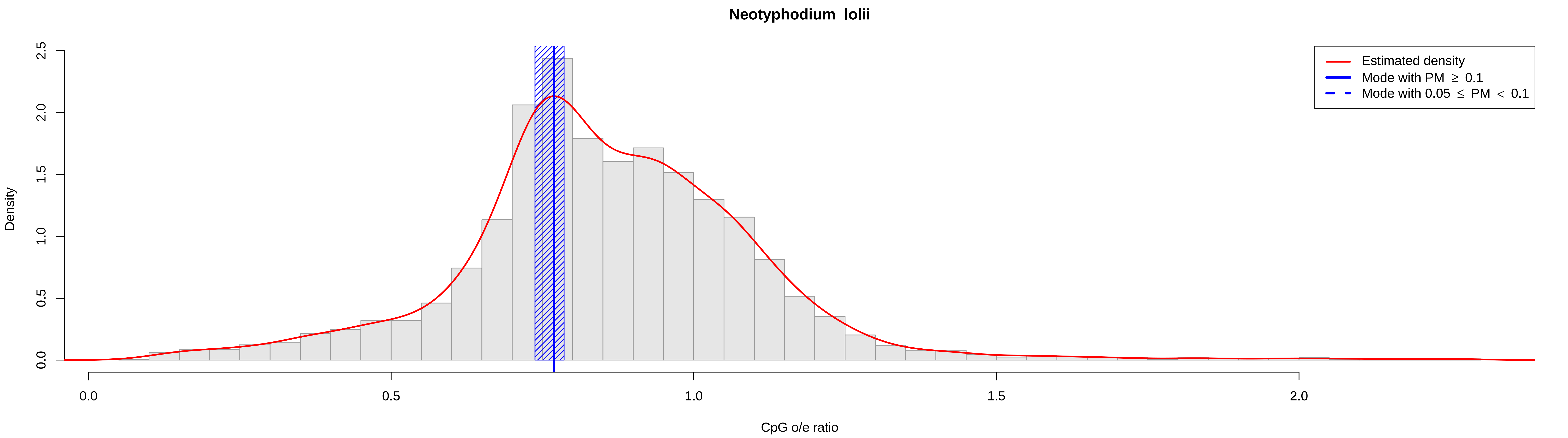
Neotyphodium_lolii
Estimated density
Mode with PM ≥ 0.1
Mode with 0.05 ≤ PM < 0.1
Density
0.0
0.5
1.0
1.5
2.0
2.5
0.0
0.5
1.0
1.5
2.0
CpG o/e ratio

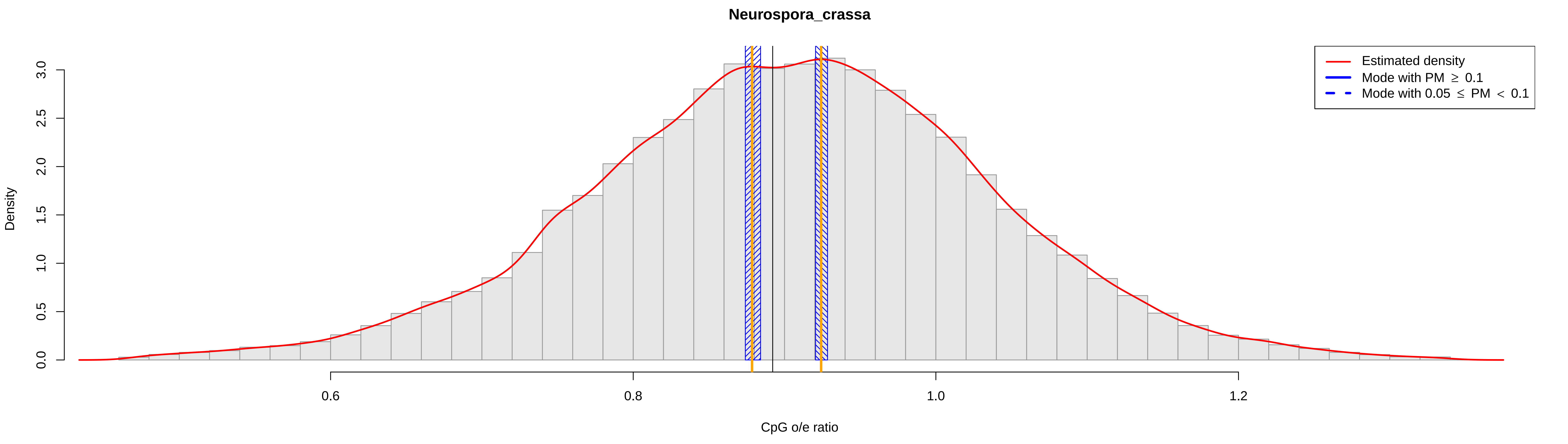

Neurospora_crassa
Estimated density
Mode with PM ≥ 0.1
Mode with 0.05 ≤ PM < 0.1
Density
0.0
0.5
1.0
1.5
2.0
2.5
3.0
0.6
0.8
1.0
1.2
CpG o/e ratio

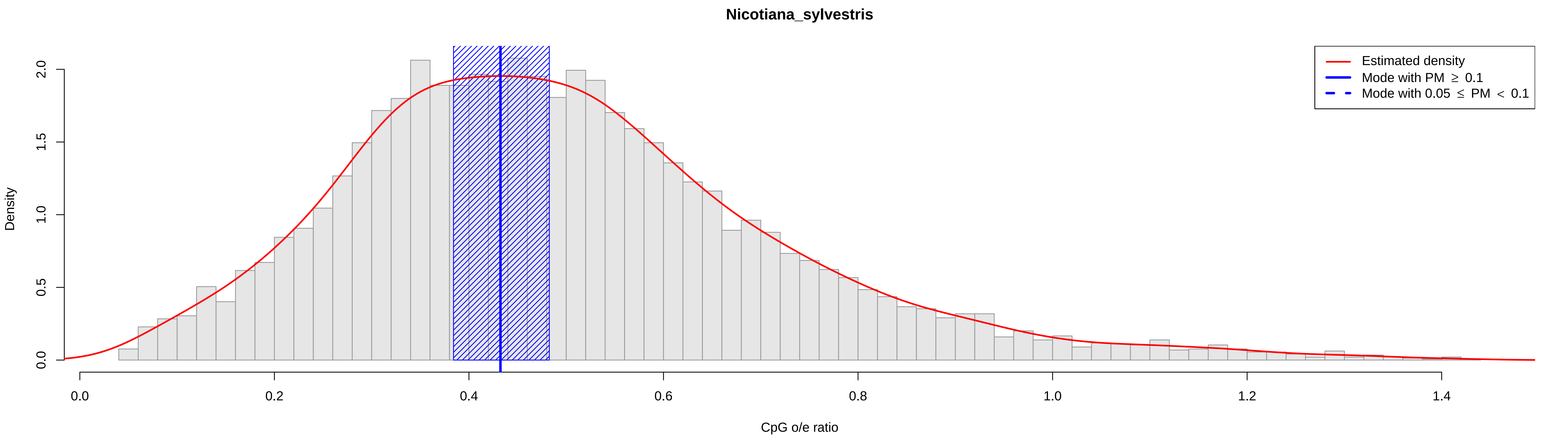

Nicotiana_sylvestris
Estimated density
Mode with PM ≥ 0.1
Mode with 0.05 ≤ PM < 0.1
Density
0.0
0.5
1.0
1.5
2.0
0.0
0.2
0.4
0.6
0.8
1.0
1.2
1.4
CpG o/e ratio

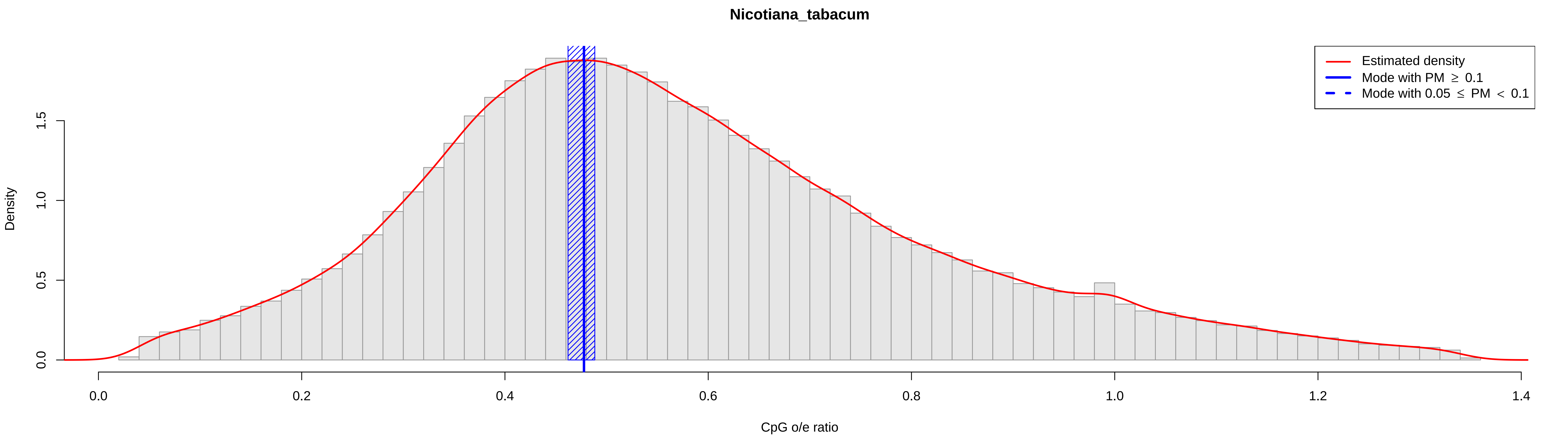

Nicotiana_tabacum
Estimated density
Mode with PM ≥ 0.1
Mode with 0.05 ≤ PM < 0.1
Density
0.0
0.5
1.0
1.5
0.0
0.2
0.4
0.6
0.8
1.0
1.2
1.4
CpG o/e ratio

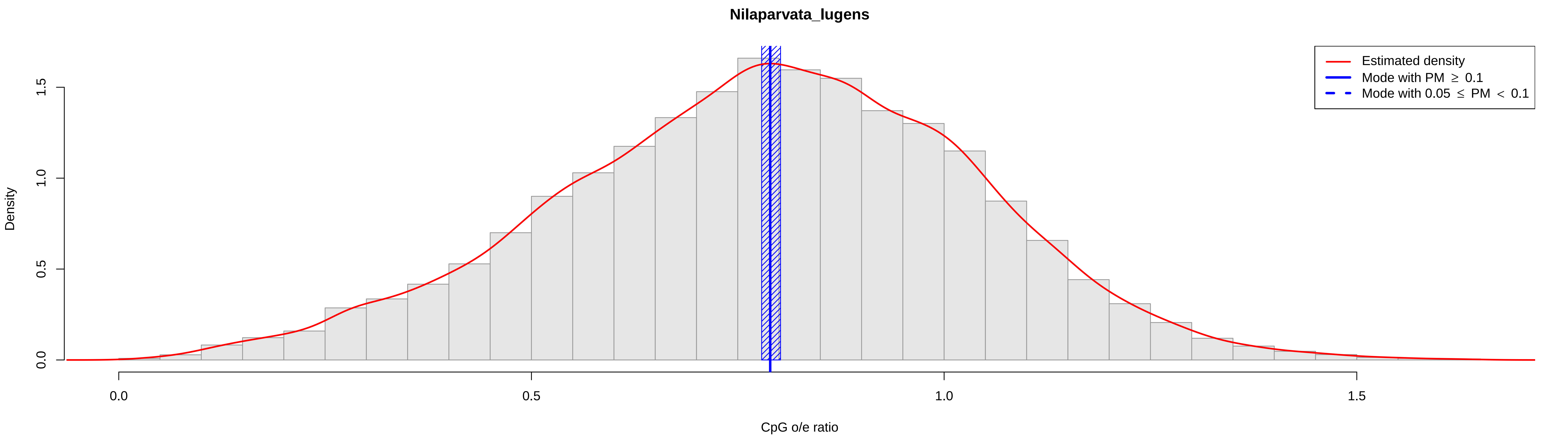

Nilaparvata_lugens
Estimated density
Mode with PM ≥ 0.1
Mode with 0.05 ≤ PM < 0.1
Density
0.0
0.5
1.0
1.5
CpG o/e ratio

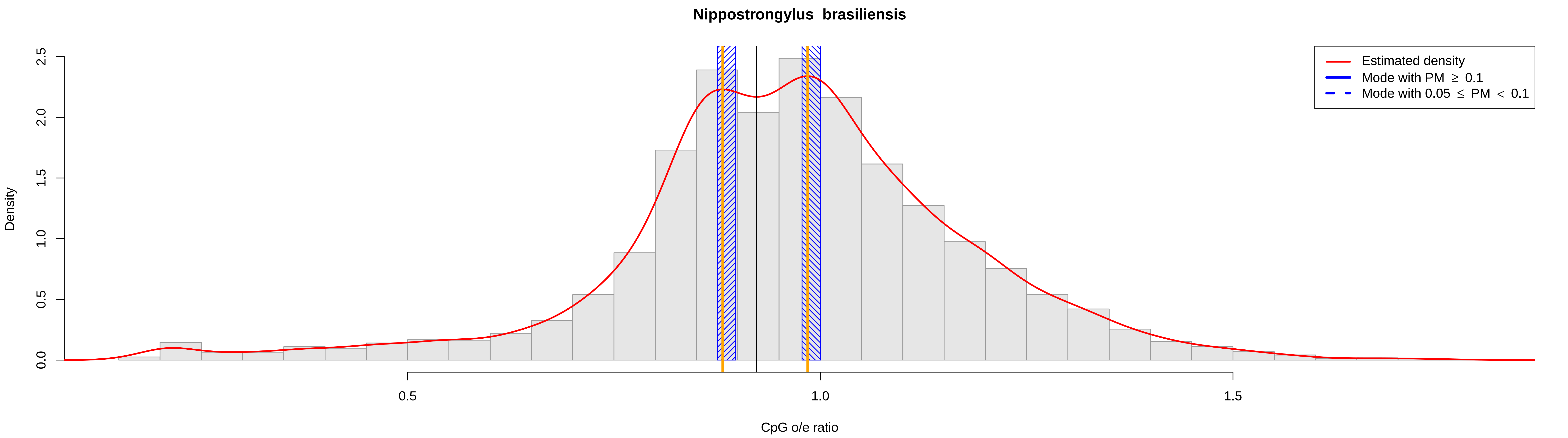

Nippostrongylus_brasiliensis
Estimated density
Mode with PM ≥ 0.1
Mode with 0.05 ≤ PM < 0.1
Density
0.0
0.5
1.0
1.5
2.0
2.5
0.5
1.0
1.5
CpG o/e ratio

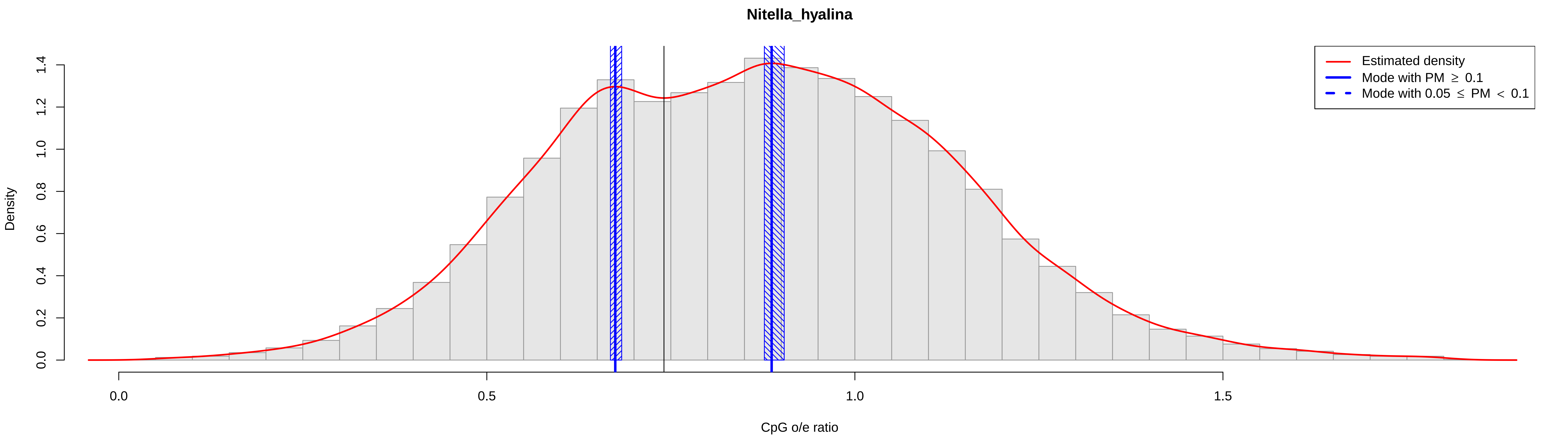

Nitella_hyalina
Estimated density
Mode with PM ≥ 0.1
Mode with 0.05 ≤ PM < 0.1
Density
0.0
0.2
0.4
0.6
0.8
1.0
1.2
1.4
0.0
0.5
1.0
1.5
CpG o/e ratio

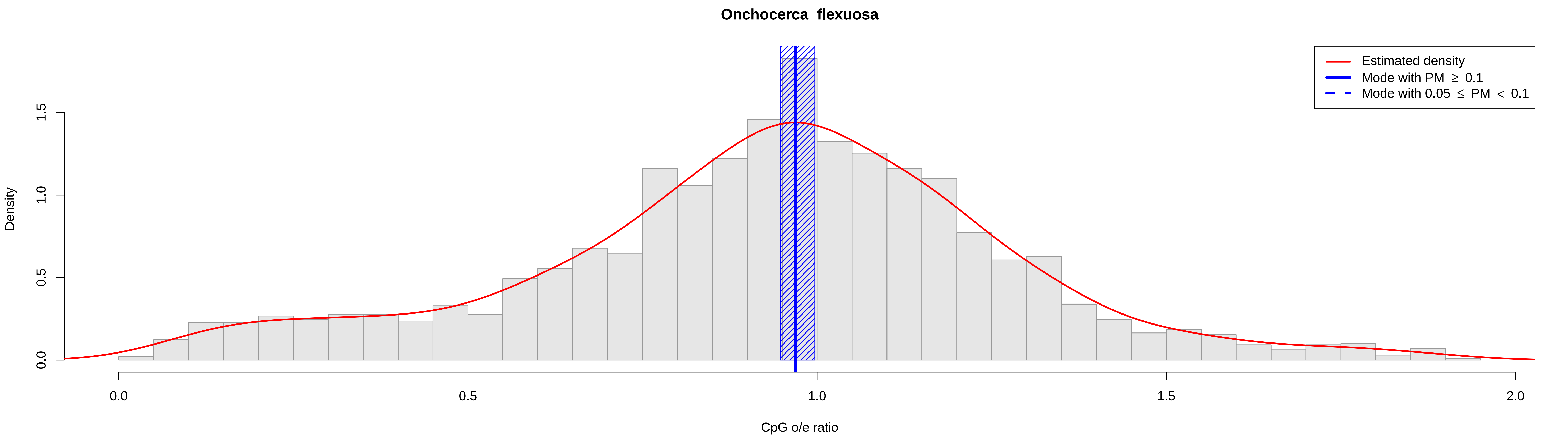

Onchocerca_flexuosa
Estimated density
Mode with PM ≥ 0.1
Mode with 0.05 ≤ PM < 0.1
Density
0.0
0.5
1.0
1.5
2.0
CpG o/e ratio

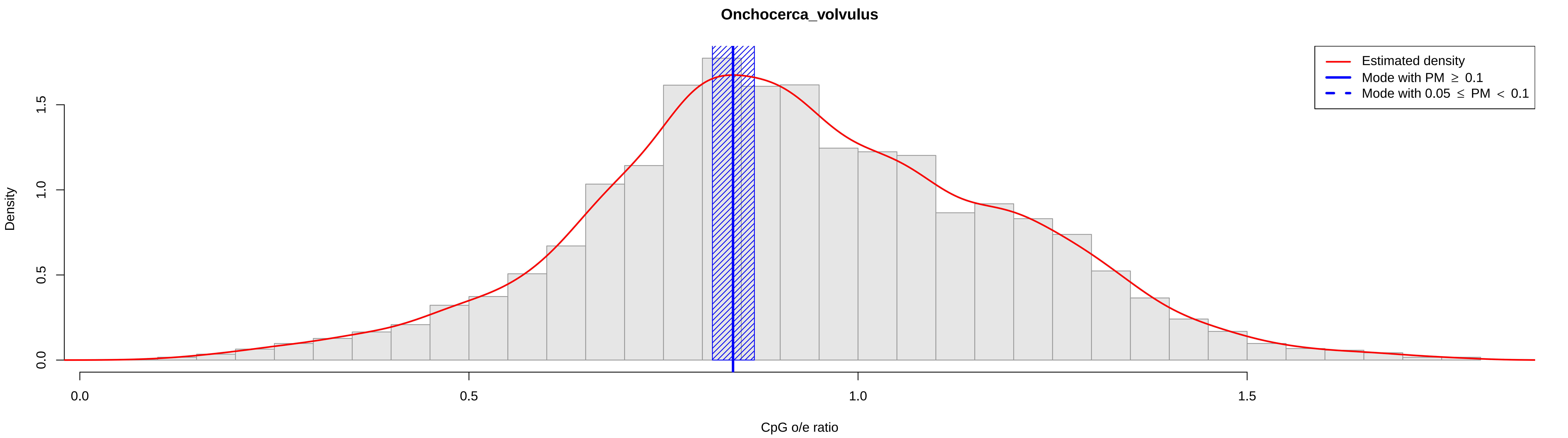

Onchocerca_volvulus
Estimated density
Mode with PM ≥ 0.1
Mode with 0.05 ≤ PM < 0.1
Density
0.0
0.5
1.0
1.5
0.0
0.5
1.0
1.5
CpG o/e ratio

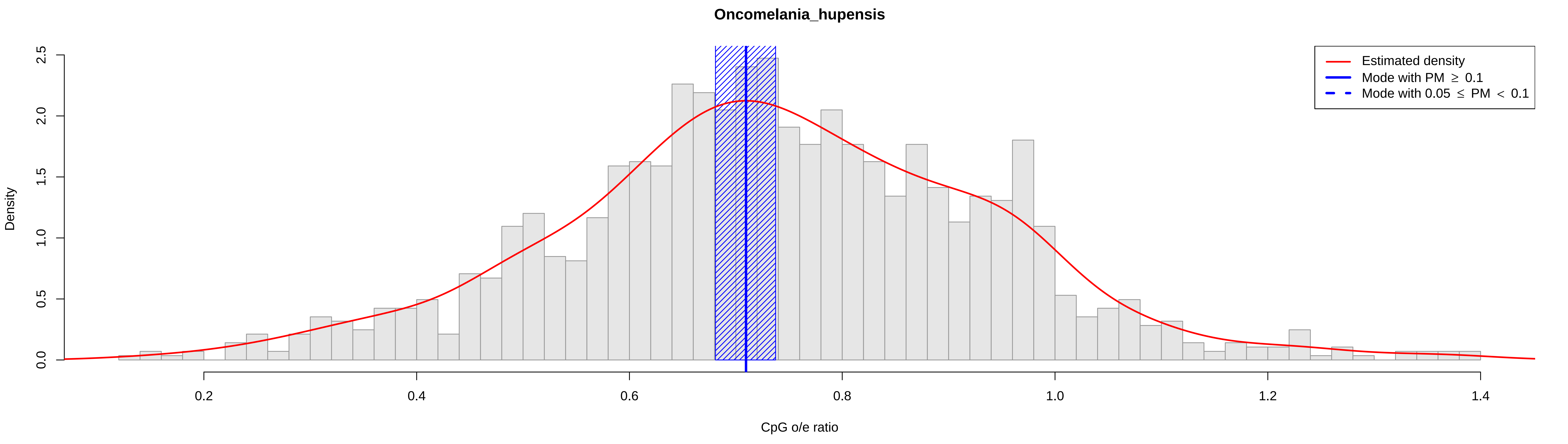
Oncomelania_hupensis
Estimated density
Mode with PM ≥ 0.1
Mode with 0.05 ≤ PM < 0.1
Density
CpG o/e ratio
0.0
0.5
1.0
1.5
2.0
2.5
0.2
0.4
0.6
0.8
1.0
1.2
1.4

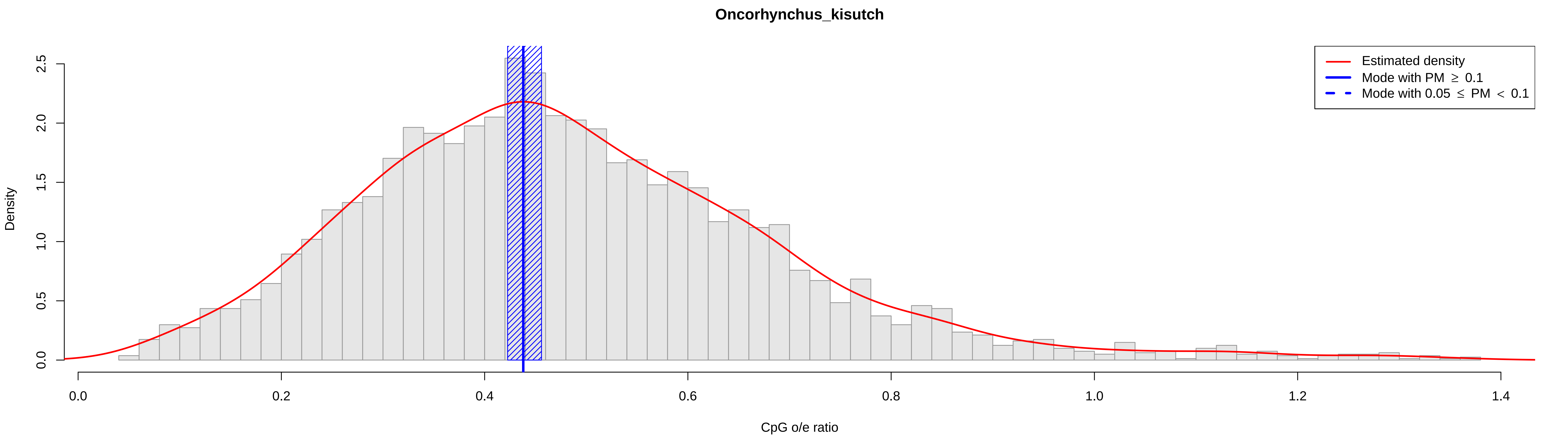

Oncorhynchus_kisutch
Estimated density
Mode with PM ≥ 0.1
Mode with 0.05 ≤ PM < 0.1
Density
0.0
0.5
1.0
1.5
2.0
2.5
0.0
0.2
0.4
0.6
0.8
1.0
1.2
1.4
CpG o/e ratio

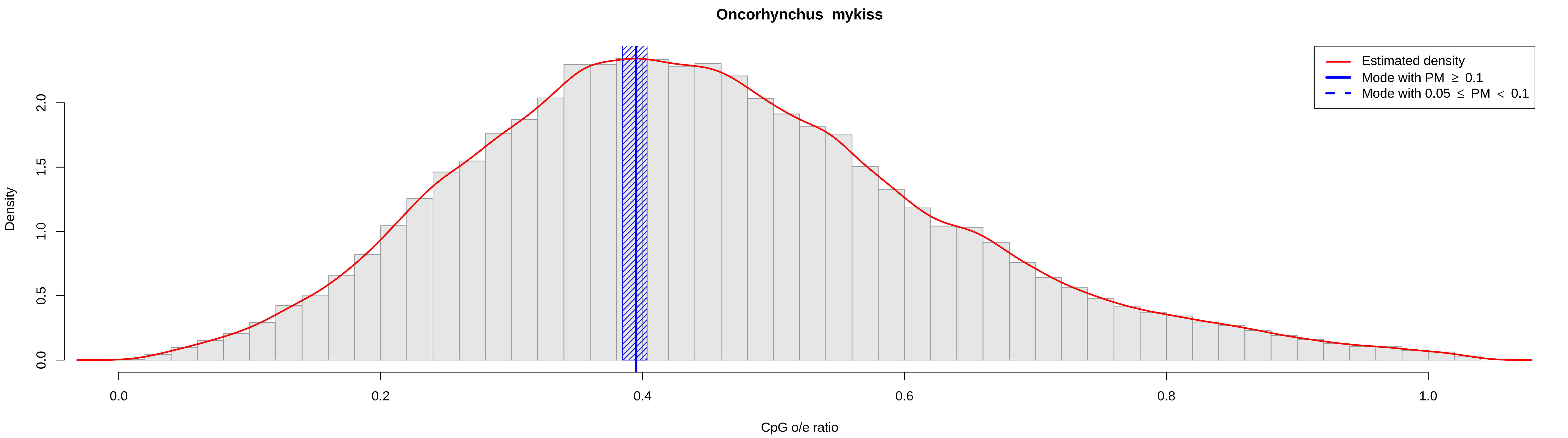

Oncorhynchus_mykiss
Estimated density
Mode with PM ≥ 0.1
Mode with 0.05 ≤ PM < 0.1
Density
0.0
0.5
1.0
1.5
2.0
0.0
0.2
0.4
0.6
0.8
1.0
CpG o/e ratio

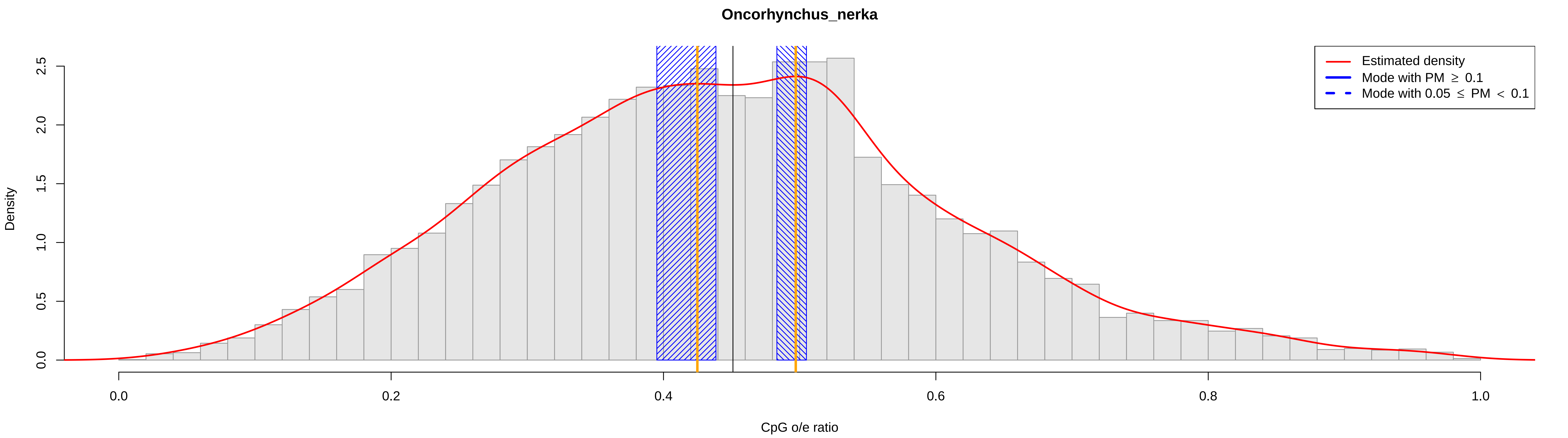
Oncorhynchus_nerka
Estimated density
Mode with PM ≥ 0.1
Mode with 0.05 ≤ PM < 0.1
Density
0.0
0.5
1.0
1.5
2.0
2.5
0.0
0.2
0.4
0.6
0.8
1.0
CpG o/e ratio

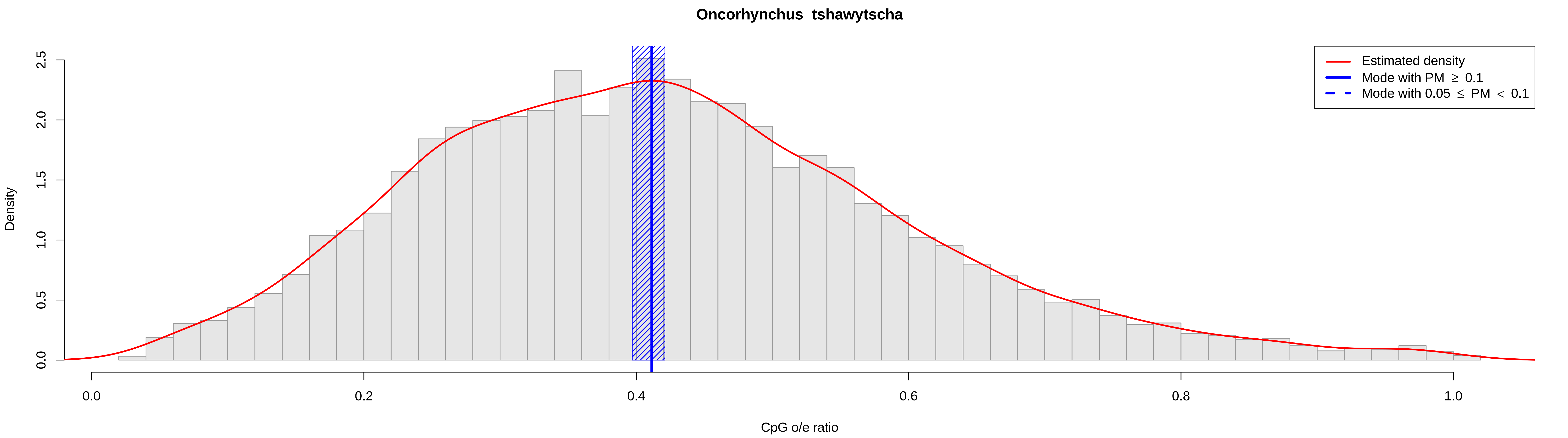
Oncorhynchus_tshawytscha
Estimated density
Mode with PM ≥ 0.1
Mode with 0.05 ≤ PM < 0.1
Density
0.0
0.5
1.0
1.5
2.0
2.5
0.0
0.2
0.4
0.6
0.8
1.0
CpG o/e ratio

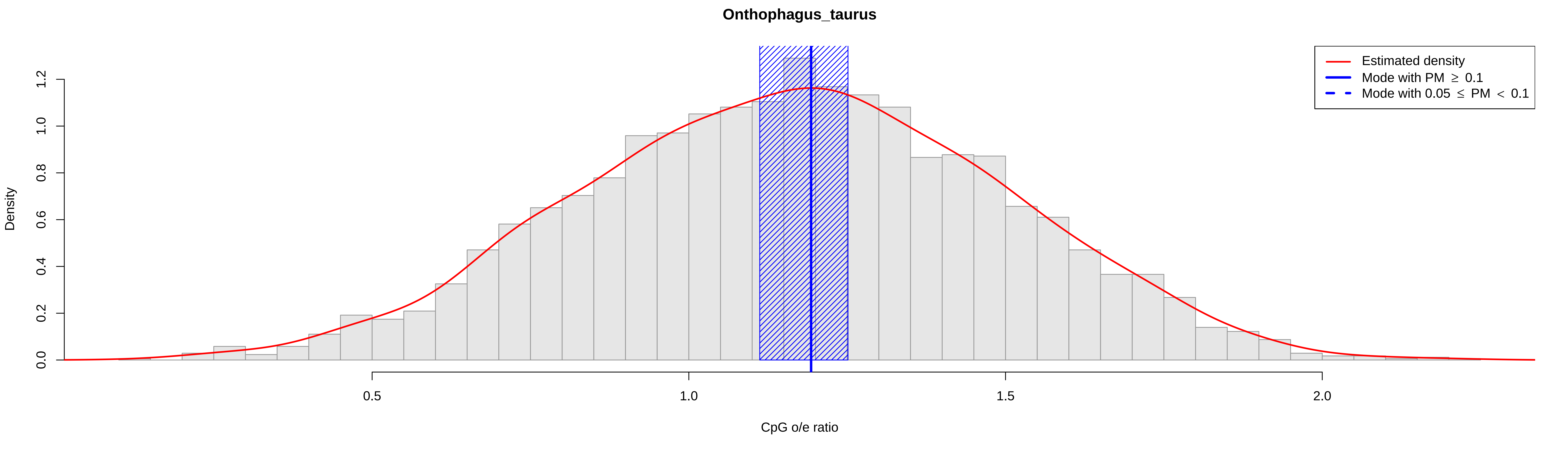

Onthophagus_taurus
Estimated density
Mode with PM ≥ 0.1
Mode with 0.05 ≤ PM < 0.1
Density
0.0
0.2
0.4
0.6
0.8
1.0
1.2
0.5
1.0
1.5
2.0
CpG o/e ratio

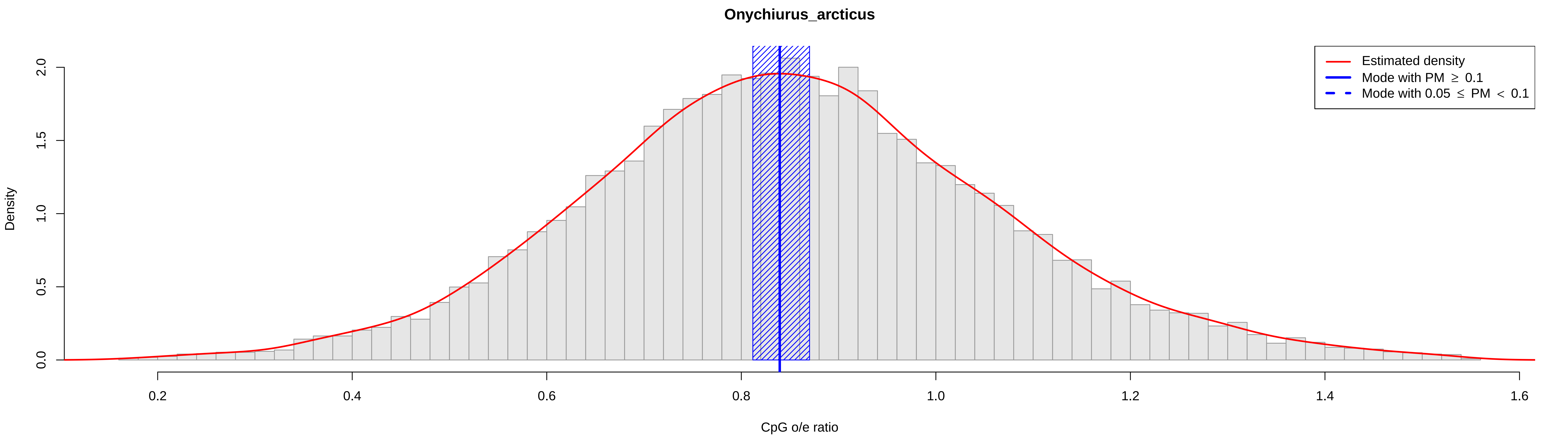

Onychiurus_arcticus
Estimated density
Mode with PM ≥ 0.1
Mode with 0.05 ≤ PM < 0.1
Density
0.0
0.5
1.0
1.5
2.0
0.2
0.4
0.6
0.8
1.0
1.2
1.4
1.6
CpG o/e ratio

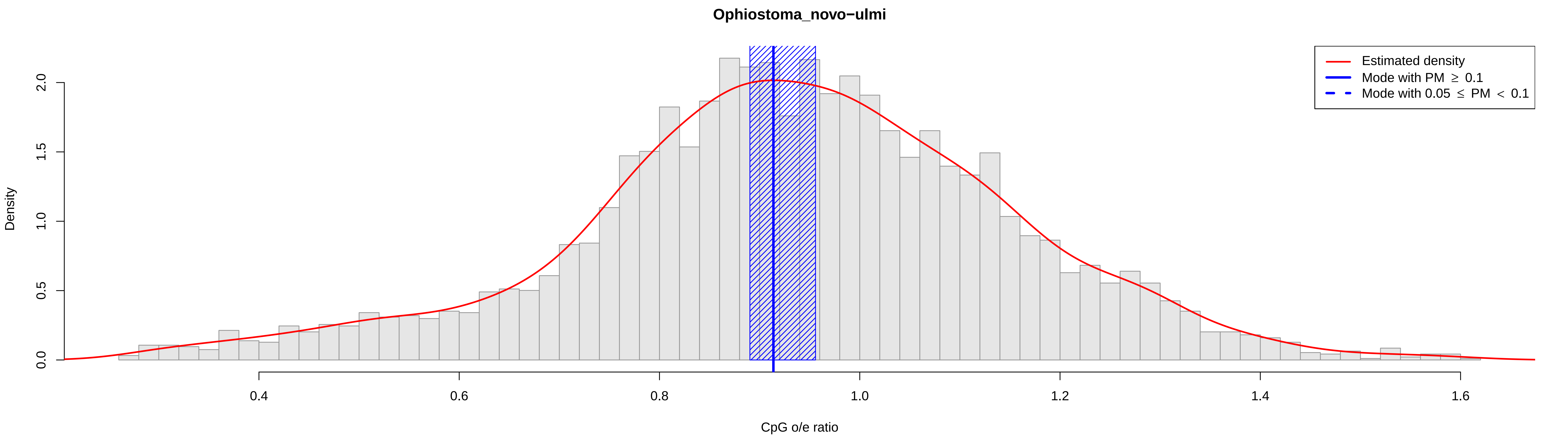
Ophiostoma_novo−ulmi
Estimated density
Mode with PM ≥ 0.1
Mode with 0.05 ≤ PM < 0.1
Density
0.0
0.5
1.0
1.5
2.0
0.4
0.6
0.8
1.0
1.2
1.4
1.6
CpG o/e ratio

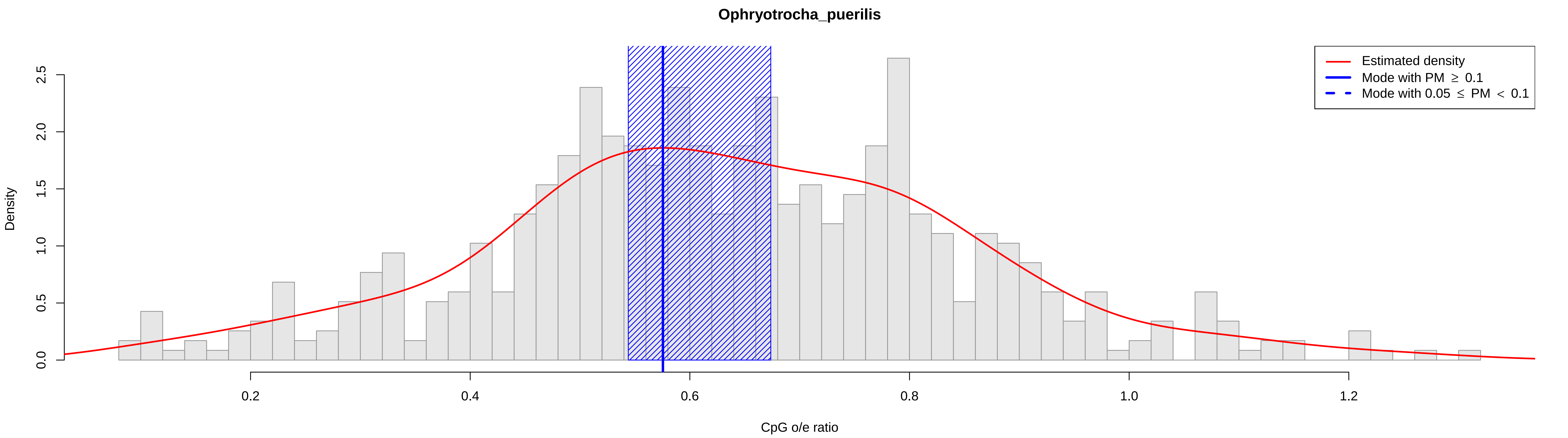

Ophryotrocha_puerilis
Estimated density
Mode with PM ≥ 0.1
Mode with 0.05 ≤ PM < 0.1
Density
0.0
0.5
1.0
1.5
2.0
2.5
0.2
0.4
0.6
0.8
1.0
1.2
CpG o/e ratio

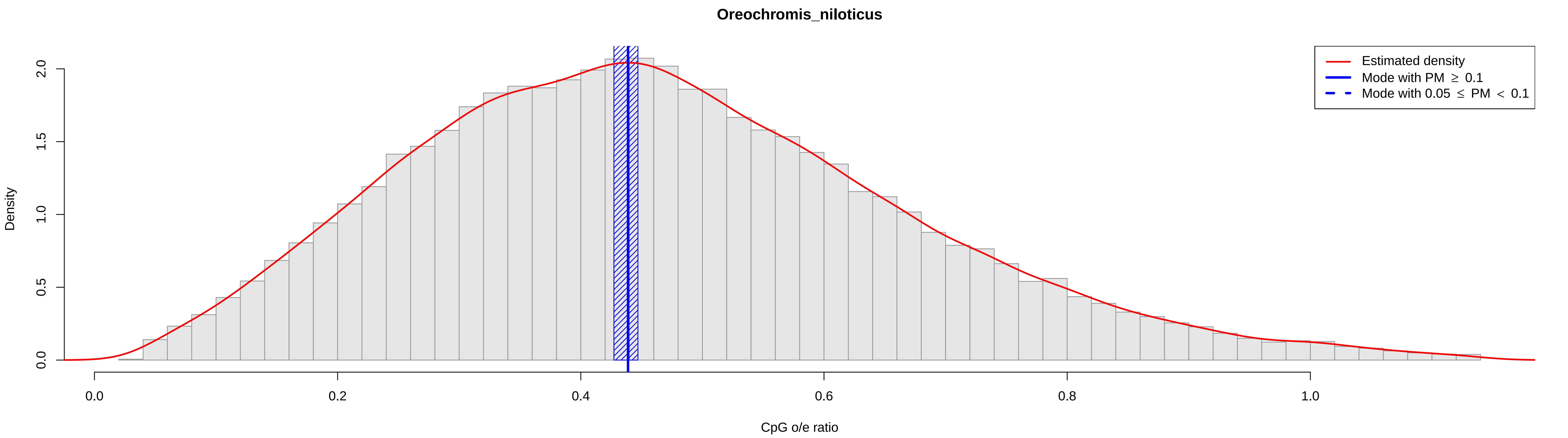

Oreochromis_niloticus
Estimated density
Mode with PM ≥ 0.1
Mode with 0.05 ≤ PM < 0.1
Density
0.0
0.5
1.0
1.5
2.0
0.0
0.2
0.4
0.6
0.8
1.0
CpG o/e ratio

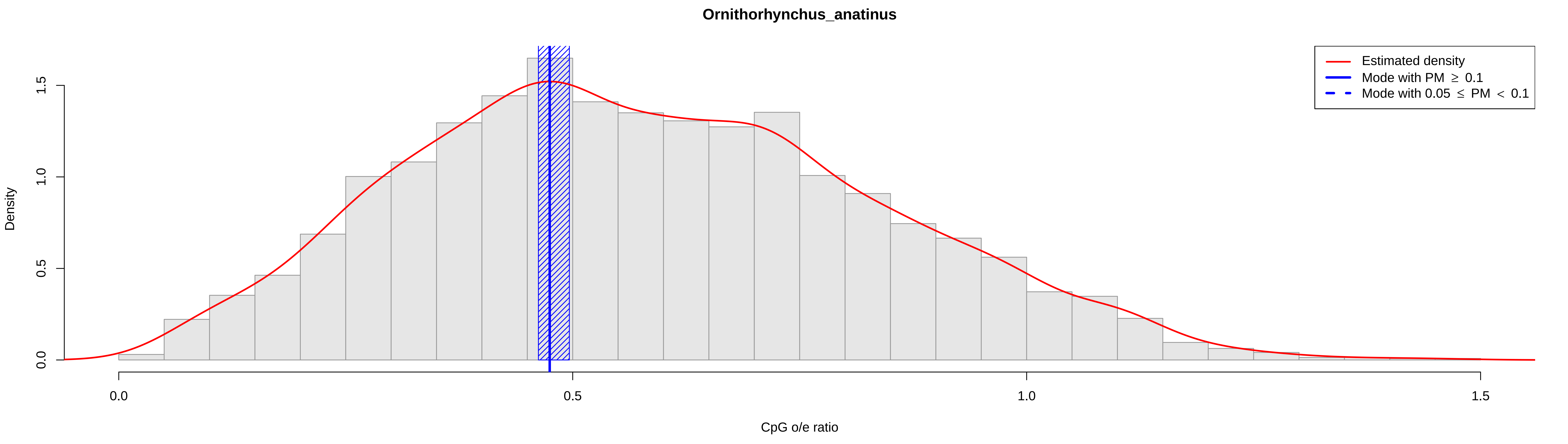

Ornithorhynchus_anatinus
Estimated density
Mode with PM ≥ 0.1
Mode with 0.05 ≤ PM < 0.1
Density
0.0
0.5
1.0
1.5
0.0
0.5
1.0
1.5
CpG o/e ratio

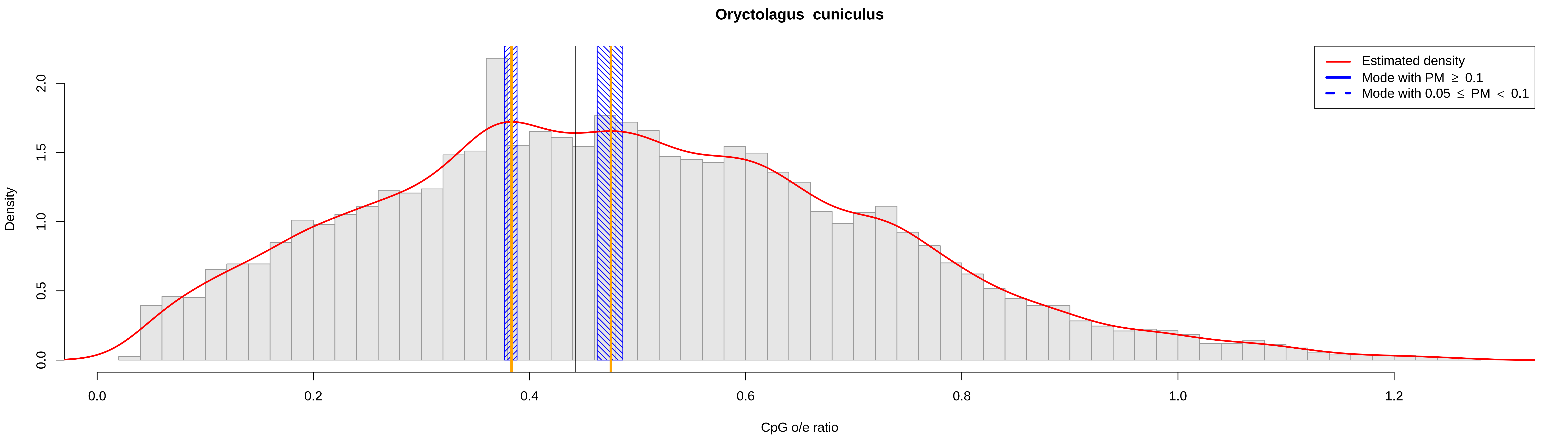
Oryctolagus_cuniculus
Estimated density
Mode with PM ≥ 0.1
Mode with 0.05 ≤ PM < 0.1
Density
0.0
0.5
1.0
1.5
2.0
0.0
0.2
0.4
0.6
0.8
1.0
1.2
CpG o/e ratio

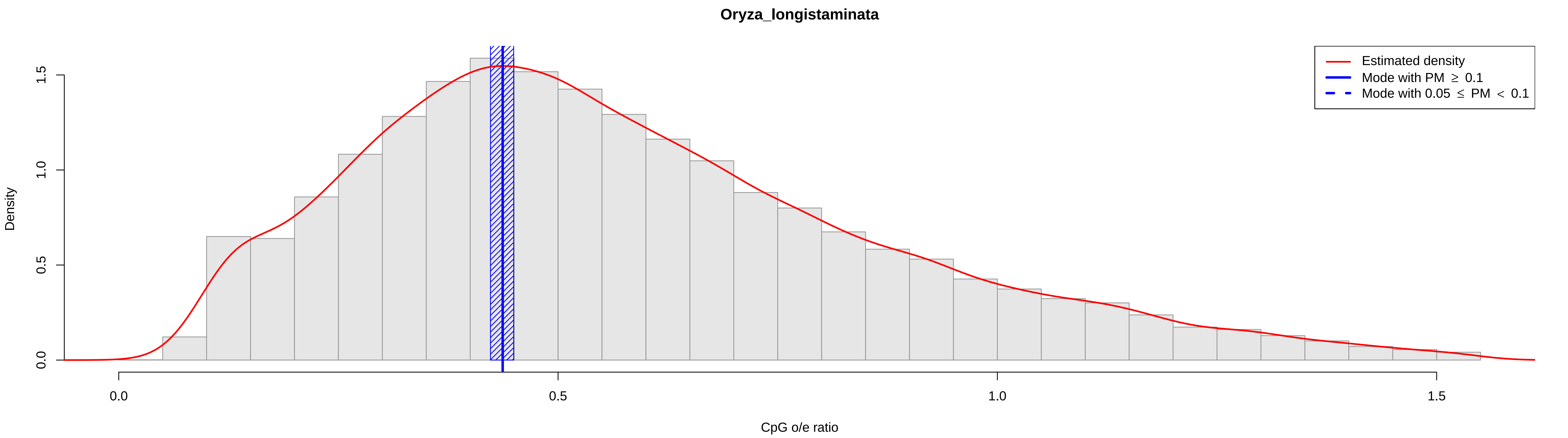

Oryza_longistaminata
Density
0.0
0.5
1.0
1.5
CpG o/e ratio
Estimated density
Mode with PM ≥ 0.1
Mode with 0.05 ≤ PM < 0.1

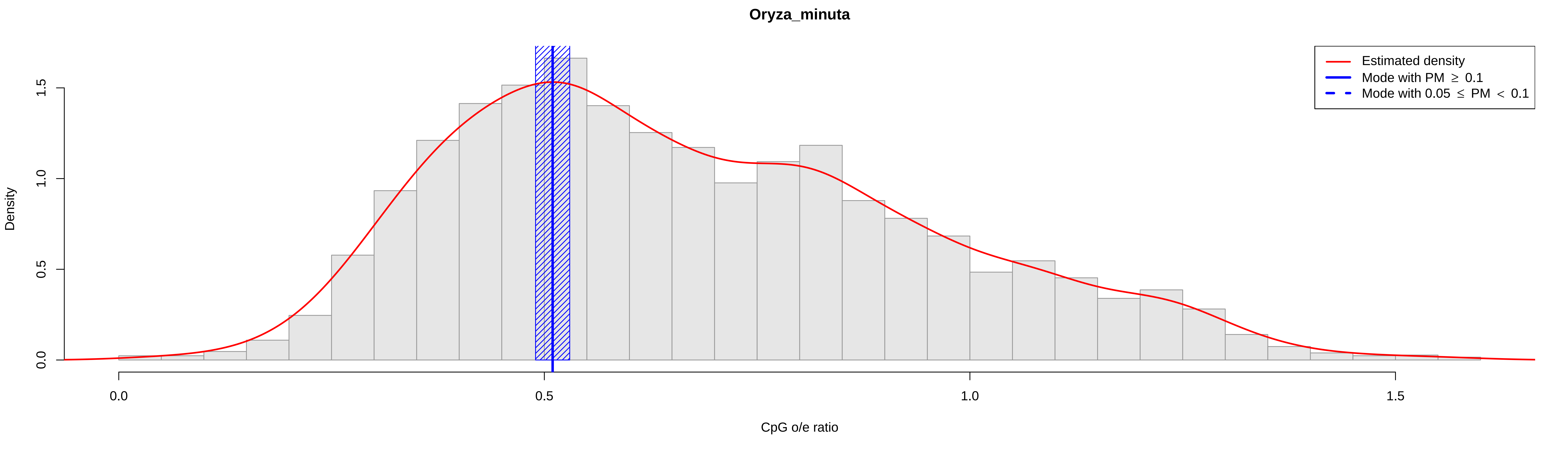

Oryza_minuta
Estimated density
Mode with PM ≥ 0.1
Mode with 0.05 ≤ PM < 0.1
Density
0.0
0.5
1.0
1.5
CpG o/e ratio
0.0
0.5
1.0
1.5

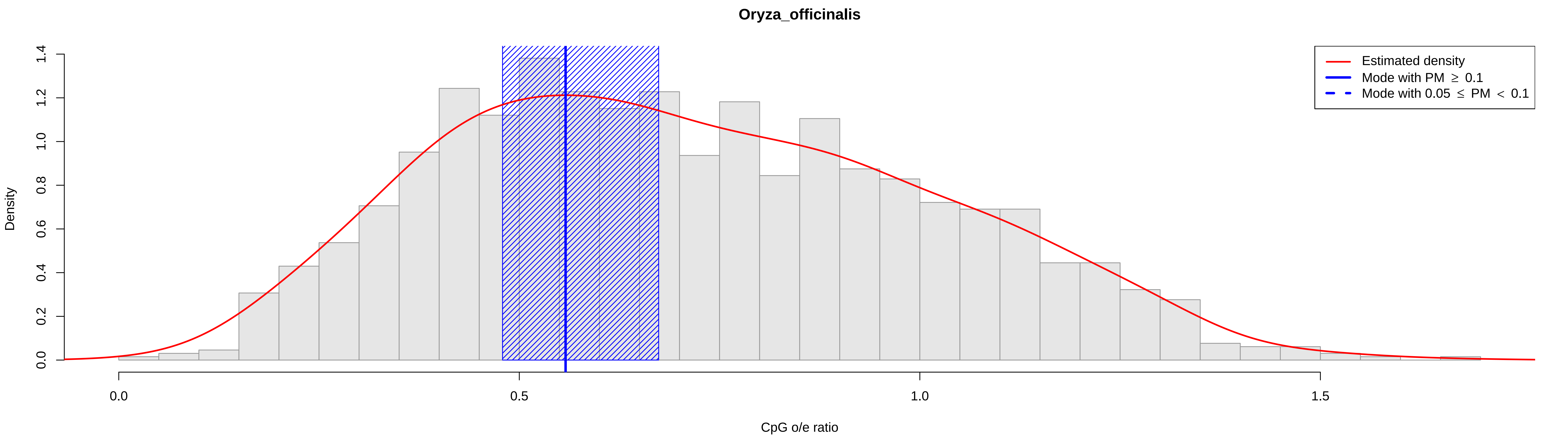

Oryza_officinalis
Estimated density
Mode with PM ≥ 0.1
Mode with 0.05 ≤ PM < 0.1
Density
CpG o/e ratio
0.0
0.2
0.4
0.6
0.8
1.0
1.2
1.4
0.0
0.5
1.0
1.5

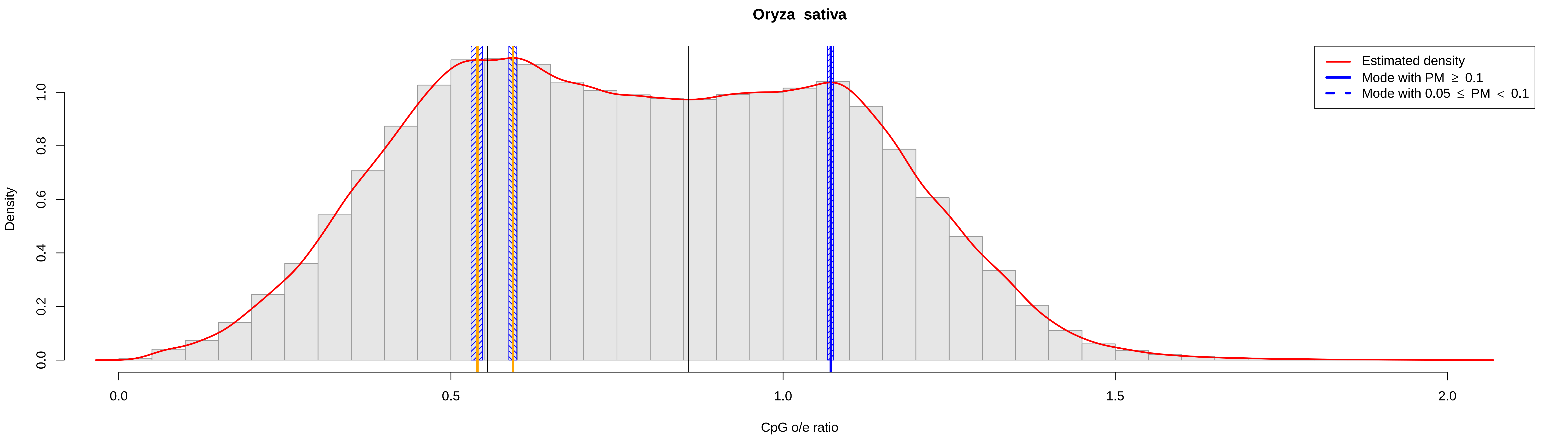
Oryza_sativa
Estimated density
Mode with PM ≥ 0.1
Mode with 0.05 ≤ PM < 0.1
Density
0.0
0.2
0.4
0.6
0.8
1.0
0.0
0.5
1.0
1.5
2.0
CpG o/e ratio

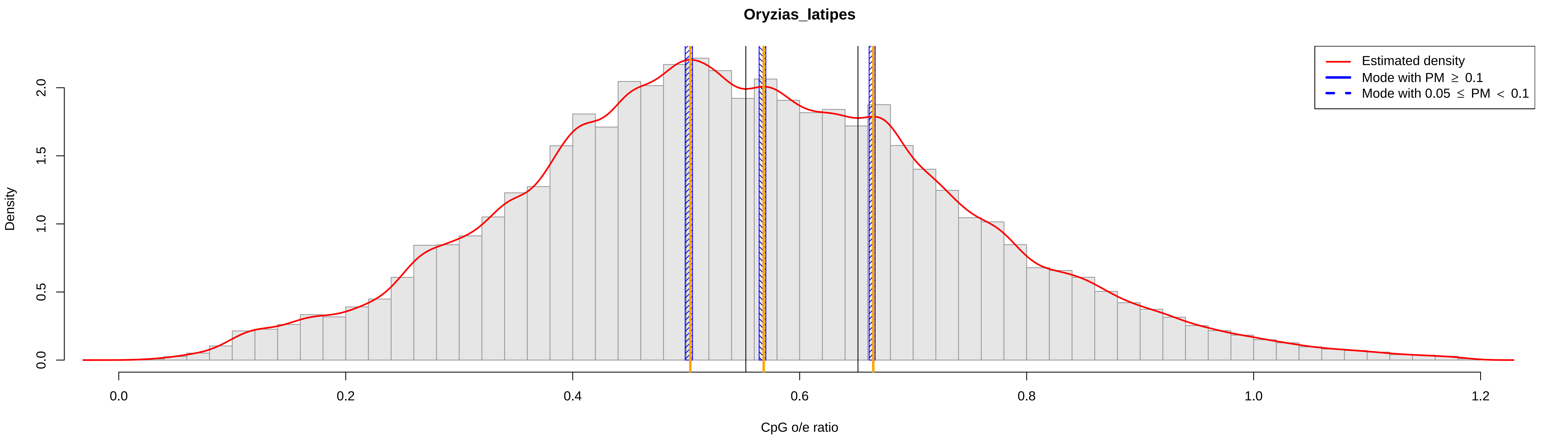
Oryzias_latipes
Estimated density
Mode with PM ≥ 0.1
Mode with 0.05 ≤ PM < 0.1
Density
0.0
0.5
1.0
1.5
2.0
0.0
0.2
0.4
0.6
0.8
1.0
1.2
CpG o/e ratio

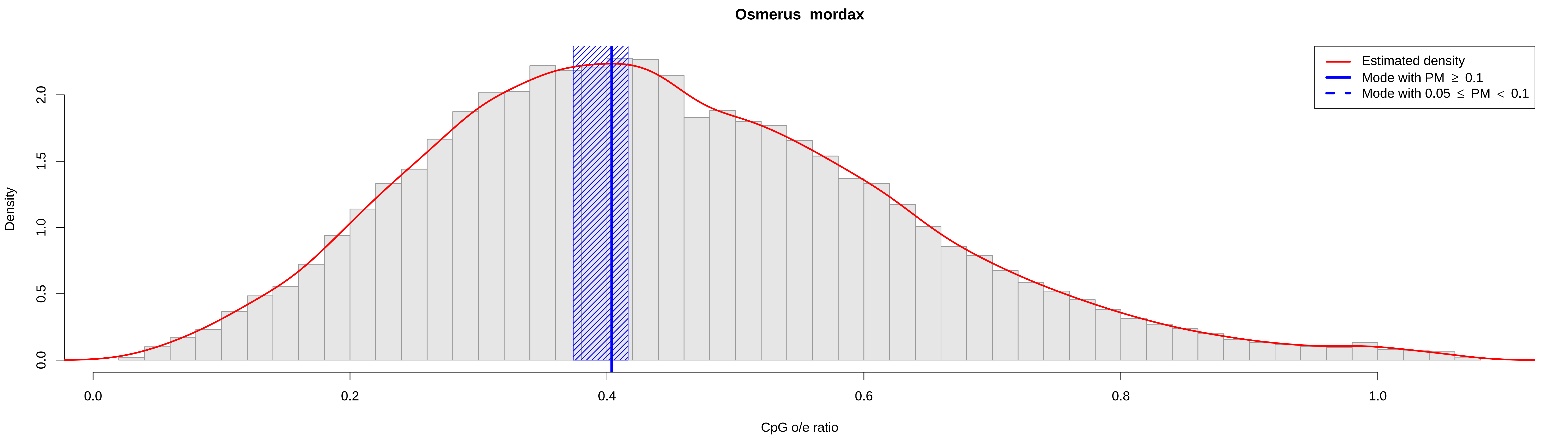
Osmerus_mordax
Density
0.0
0.5
1.0
1.5
2.0
0.0
0.2
0.4
0.6
0.8
1.0
CpG o/e ratio
Estimated density
Mode with PM ≥ 0.1
Mode with 0.05 ≤ PM < 0.1

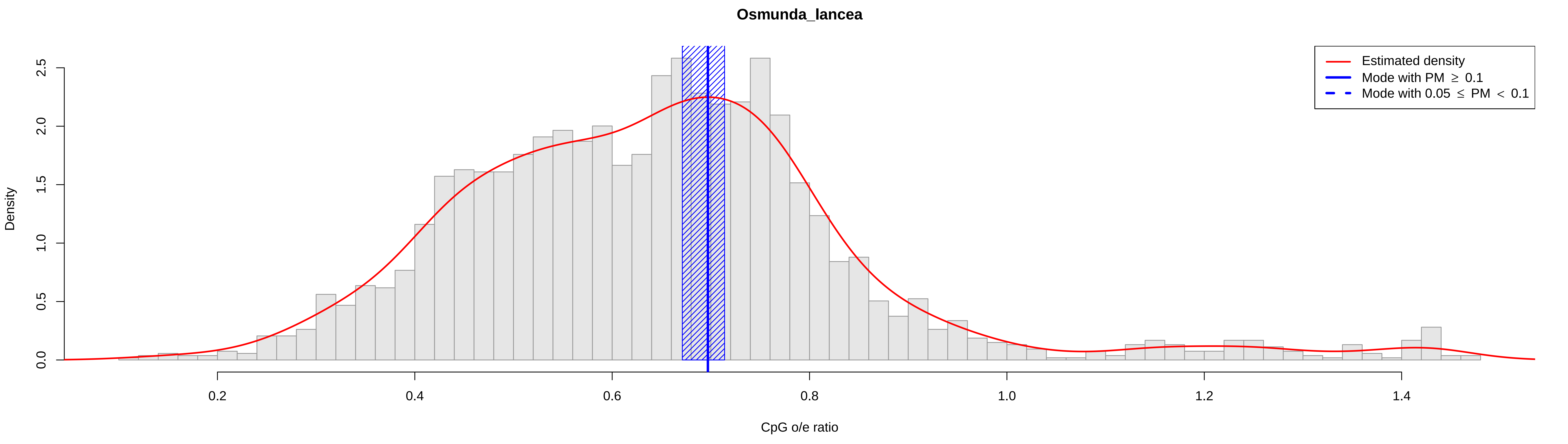
Osmunda_lancea
Estimated density
Mode with PM ≥ 0.1
Mode with 0.05 ≤ PM < 0.1
Density
0.0
0.5
1.0
1.5
2.0
2.5
0.2
0.4
0.6
0.8
1.0
1.2
1.4
CpG o/e ratio

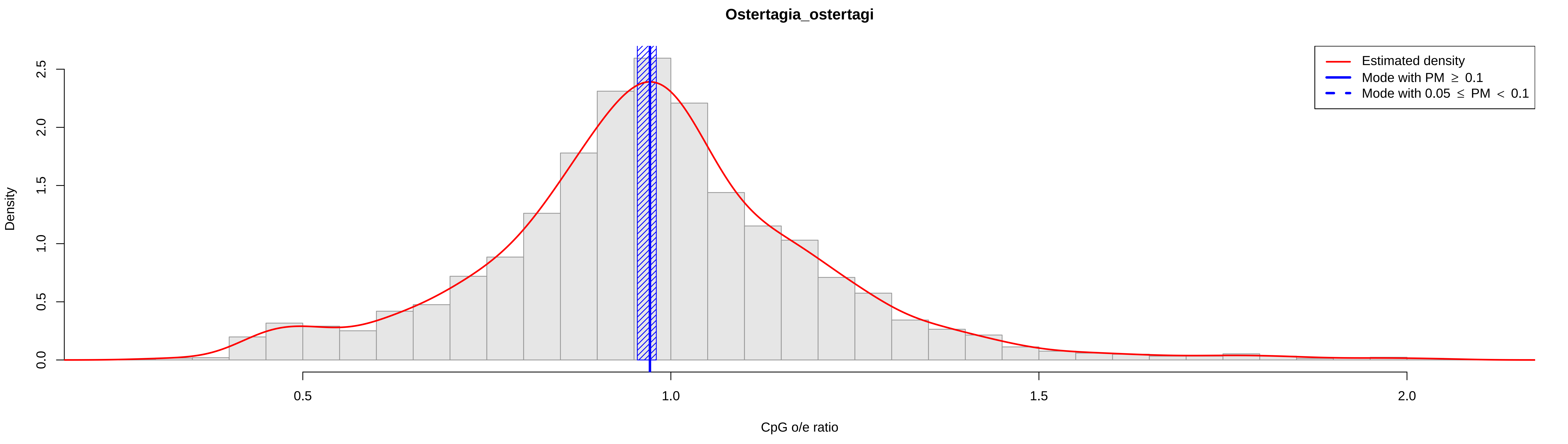
Ostertagia_ostertagi
Estimated density
Mode with PM ≥ 0.1
Mode with 0.05 ≤ PM < 0.1
Density
0.0
0.5
1.0
1.5
2.0
2.5
0.5
1.0
1.5
2.0
CpG o/e ratio

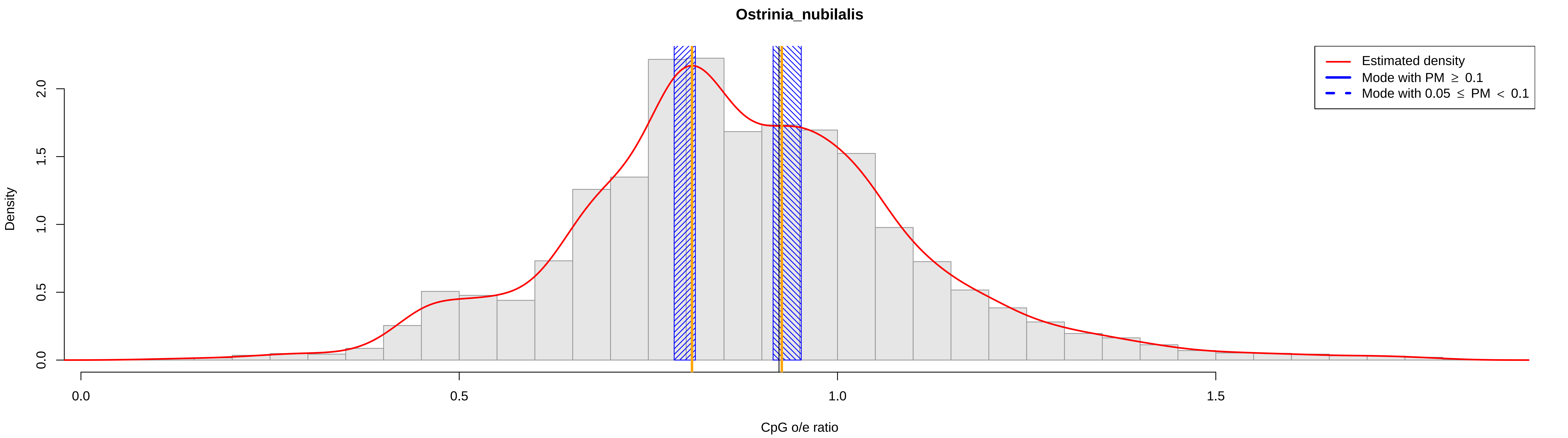

Ostrinia_nubilalis
Estimated density
Mode with PM ≥ 0.1
Mode with 0.05 ≤ PM < 0.1
Density
0.0
0.5
1.0
1.5
2.0
0.0
0.5
1.0
1.5
CpG o/e ratio

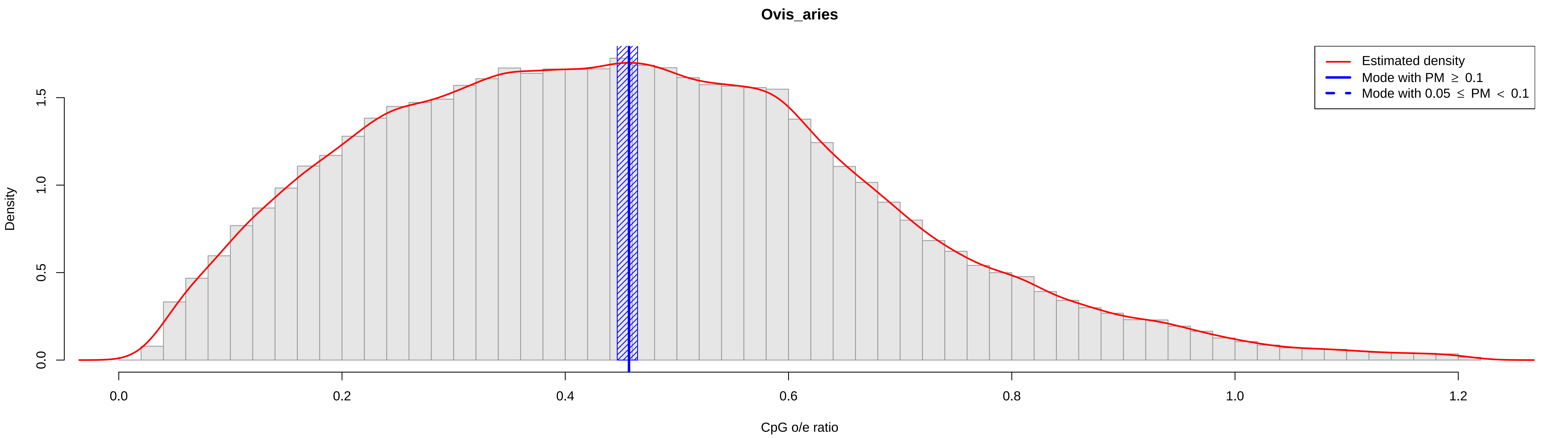
Ovis_aries
Estimated density
Mode with PM ≥ 0.1
Mode with 0.05 ≤ PM < 0.1
Density
0.0
0.5
1.0
1.5
0.0
0.2
0.4
0.6
0.8
1.0
1.2
CpG o/e ratio

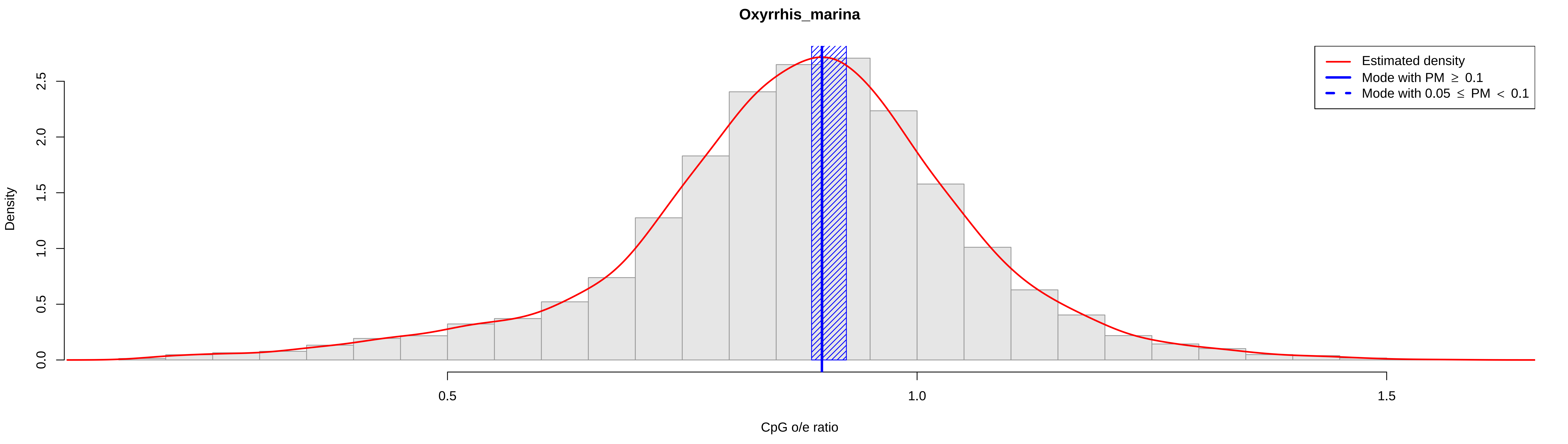

Oxyrrhis_marina
Estimated density
Mode with PM ≥ 0.1
Mode with 0.05 ≤ PM < 0.1
Density
0.0
0.5
1.0
1.5
2.0
2.5
0.5
1.0
1.5
CpG o/e ratio

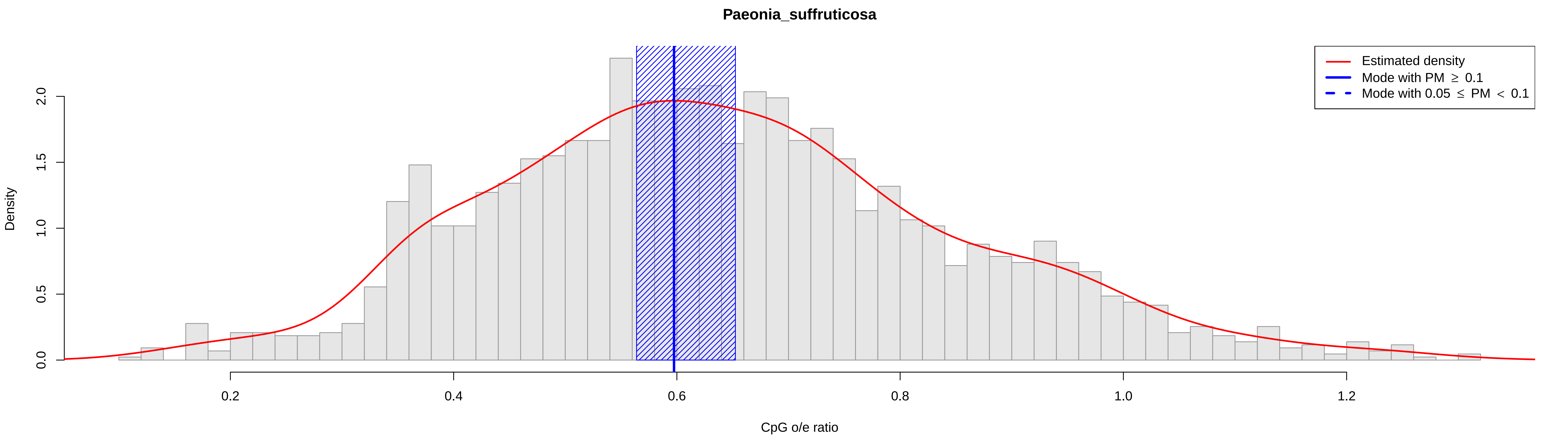

Paeonia_suffruticosa
Estimated density
Mode with PM ≥ 0.1
Mode with 0.05 ≤ PM < 0.1
Density
0.0
0.5
1.0
1.5
2.0
0.2
0.4
0.6
0.8
1.0
1.2
CpG o/e ratio

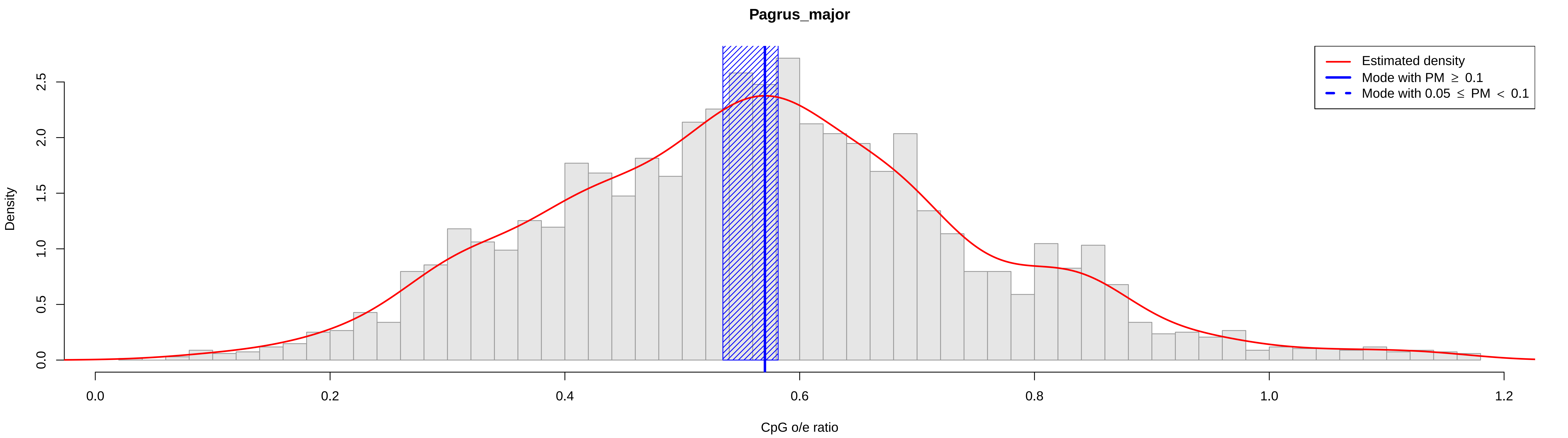

Pagrus_major
Estimated density
Mode with PM ≥ 0.1
Mode with 0.05 ≤ PM < 0.1
Density
0.0
0.5
1.0
1.5
2.0
2.5
0.0
0.2
0.4
0.6
0.8
1.0
1.2
CpG o/e ratio

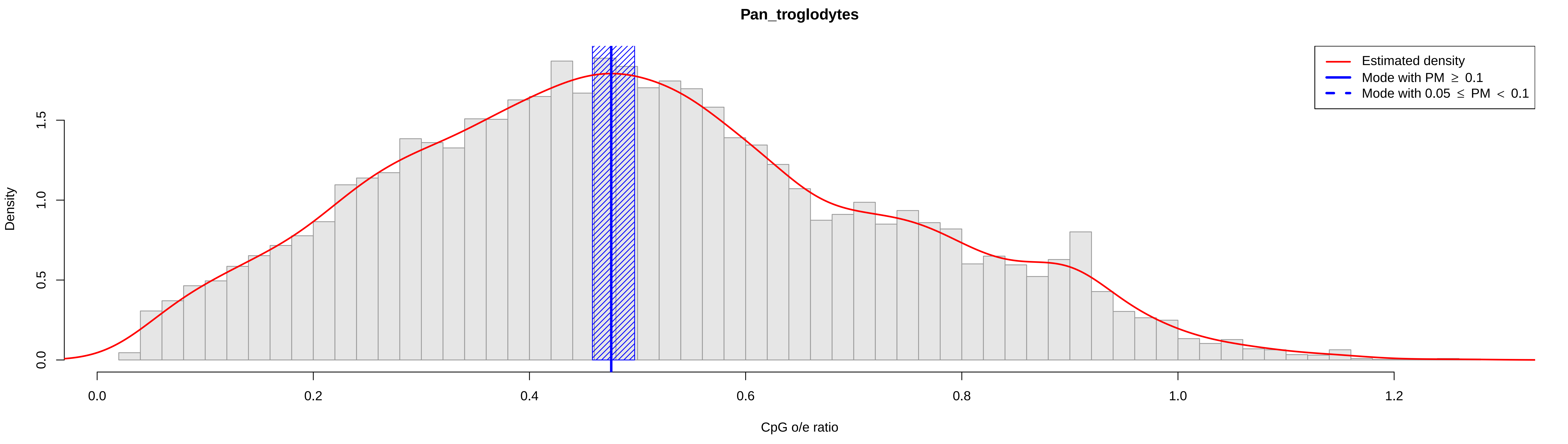

Pan_troglodytes
Estimated density
Mode with PM ≥ 0.1
Mode with 0.05 ≤ PM < 0.1
Density
0.0
0.5
1.0
1.5
0.0
0.2
0.4
0.6
0.8
1.0
1.2
CpG o/e ratio

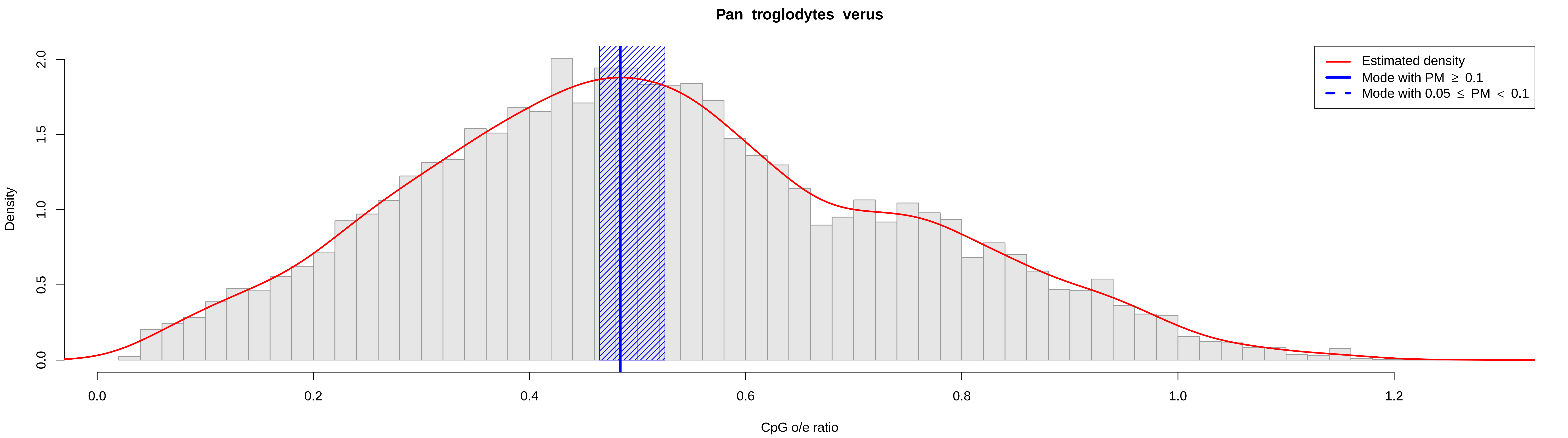

Pan_troglodytes_verus
Estimated density
Mode with PM ≥ 0.1
Mode with 0.05 ≤ PM < 0.1
Density
0.0
0.5
1.0
1.5
2.0
0.0
0.2
0.4
0.6
0.8
1.0
1.2
CpG o/e ratio

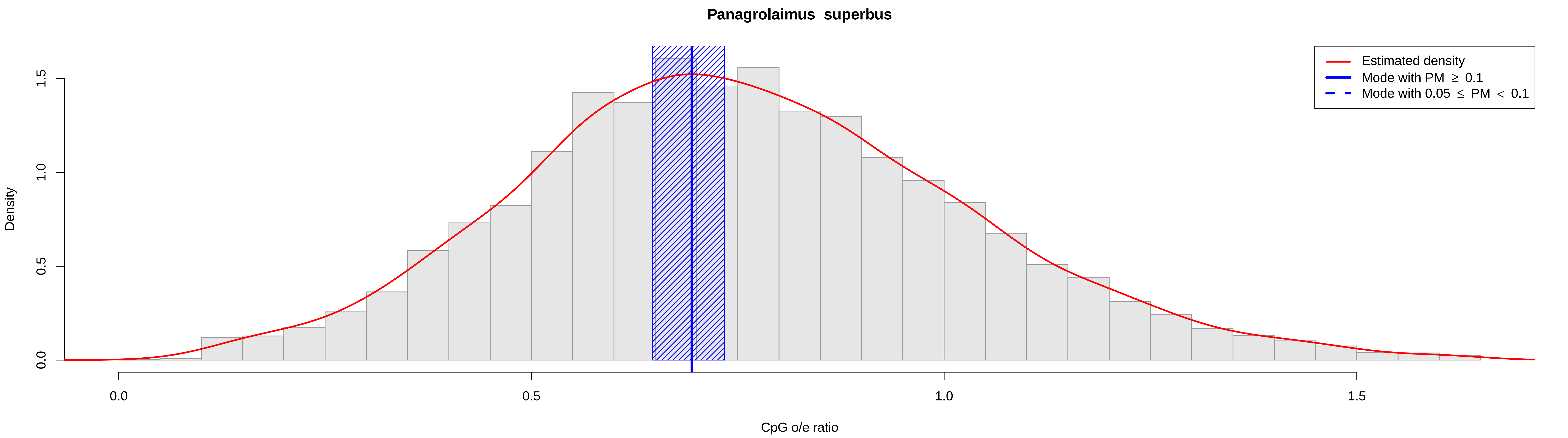

Panagrolaimus_superbus
Estimated density
Mode with PM ≥ 0.1
Mode with 0.05 ≤ PM < 0.1
Density
0.0
0.5
1.0
1.5
CpG o/e ratio

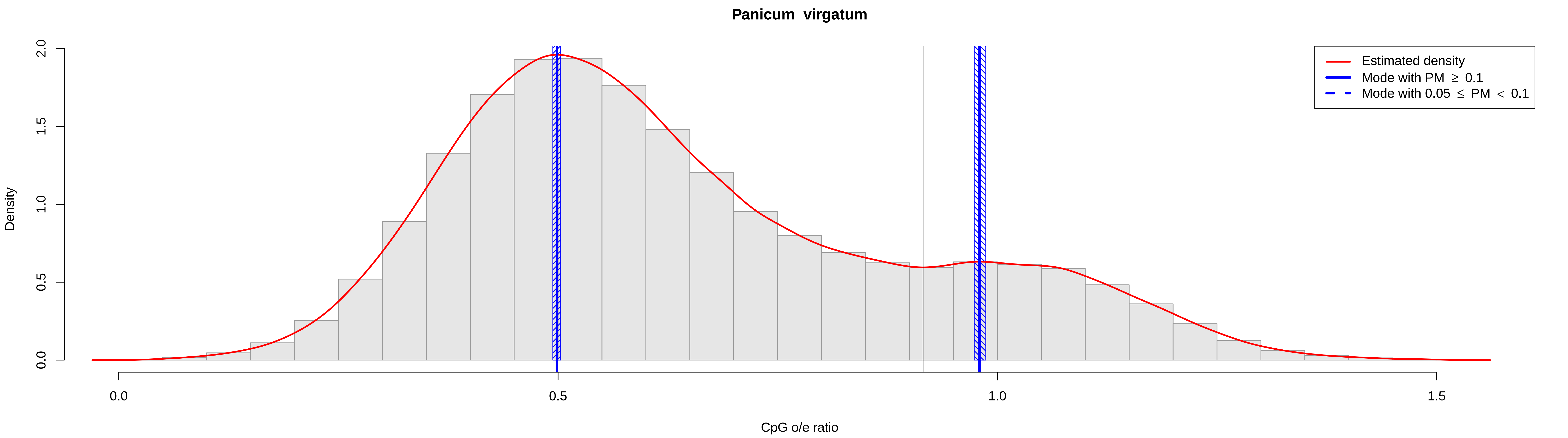

Panicum_virgatum
Estimated density
Mode with PM ≥ 0.1
Mode with 0.05 ≤ PM < 0.1
Density
0.0
0.5
1.0
1.5
2.0
CpG o/e ratio

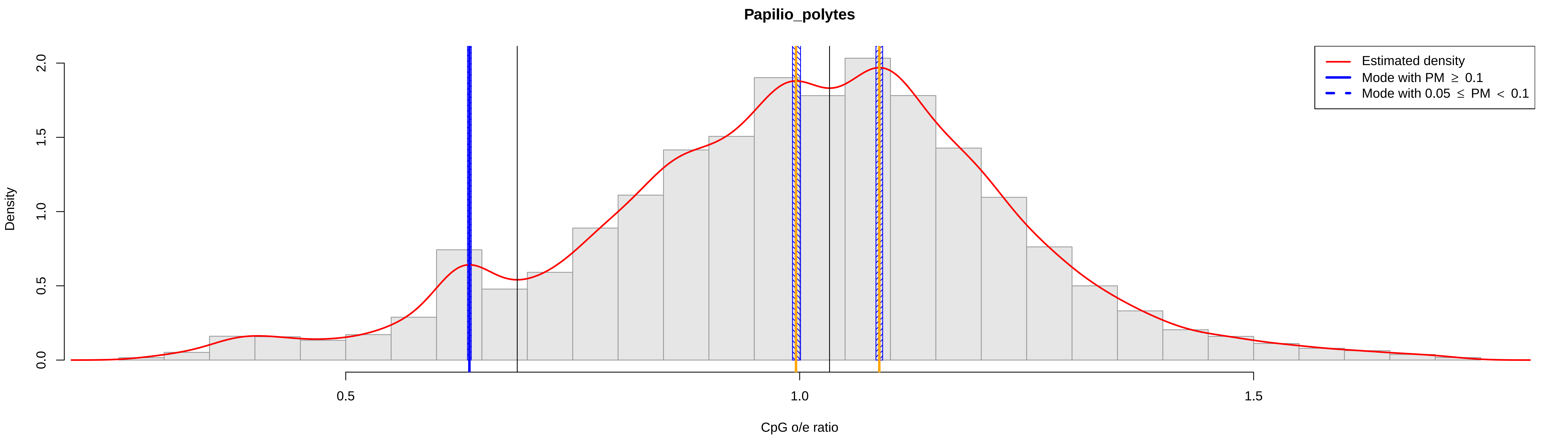

Papilio_polytes
Estimated density
Mode with PM ≥ 0.1
Mode with 0.05 ≤ PM < 0.1
Density
0.0
0.5
1.0
1.5
2.0
0.5
1.0
1.5
CpG o/e ratio

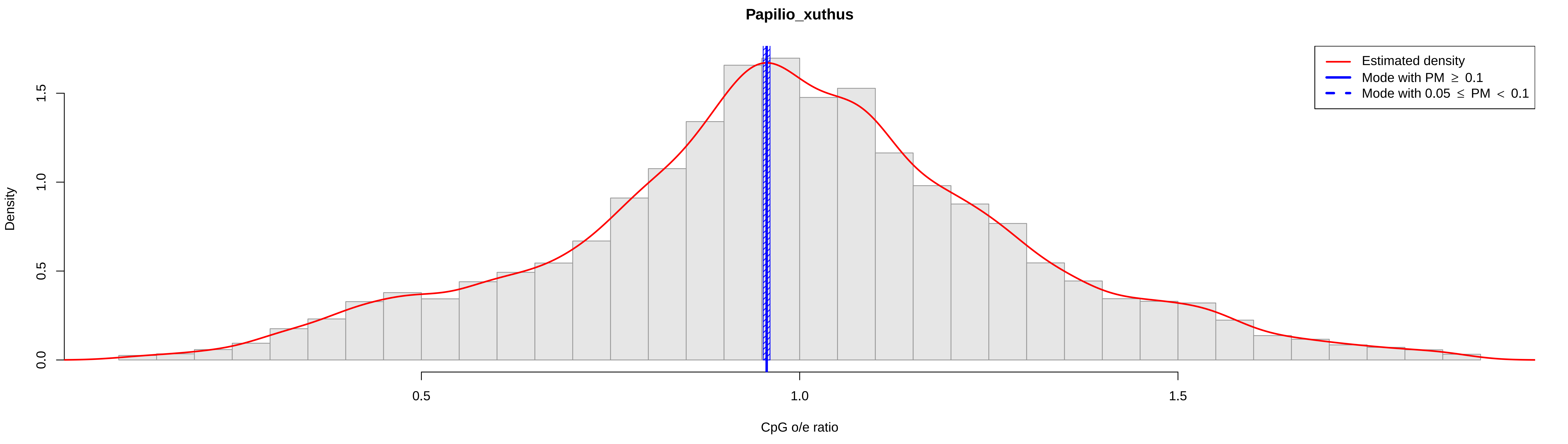

Papilio_xuthus
Estimated density
Mode with PM ≥ 0.1
Mode with 0.05 ≤ PM < 0.1
Density
0.0
0.5
1.0
1.5
0.5
1.0
1.5
CpG o/e ratio

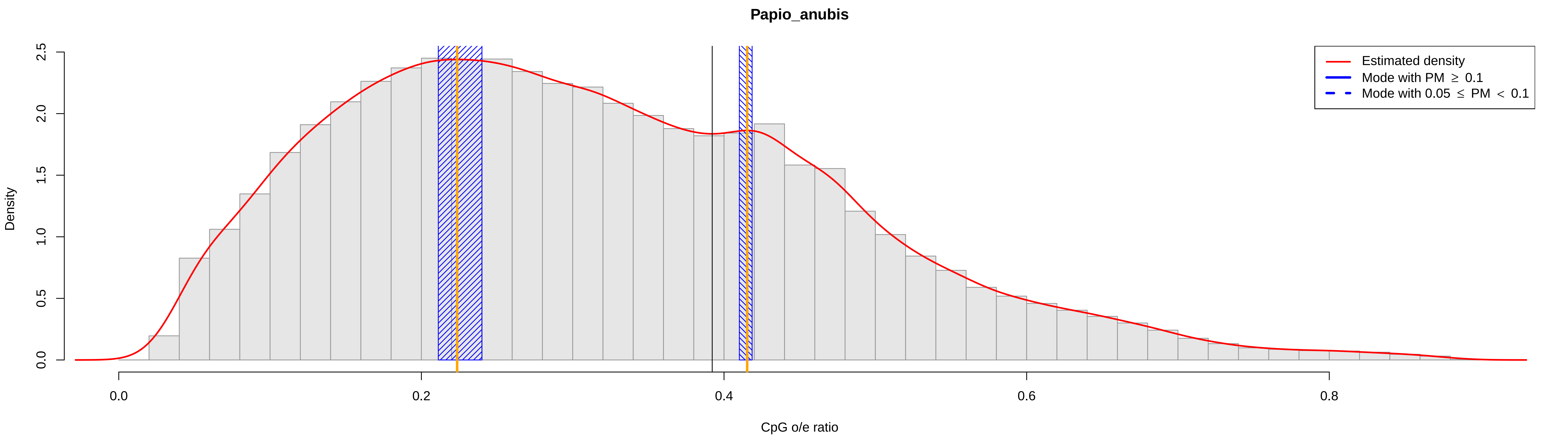
Papio_anubis
Estimated density
Mode with PM ≥ 0.1
Mode with 0.05 ≤ PM < 0.1
Density
0.0
0.5
1.0
1.5
2.0
2.5
0.0
0.2
0.4
0.6
0.8
CpG o/e ratio

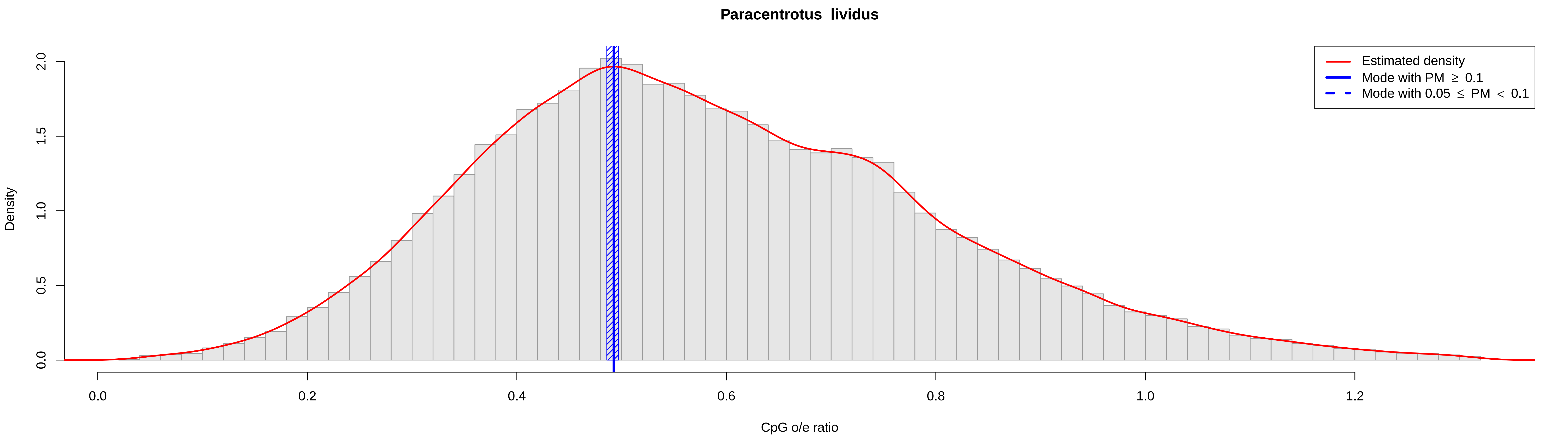

Paracentrotus_lividus
Estimated density
Mode with PM ≥ 0.1
Mode with 0.05 ≤ PM < 0.1
Density
0.0
0.5
1.0
1.5
2.0
0.0
0.2
0.4
0.6
0.8
1.0
1.2
CpG o/e ratio

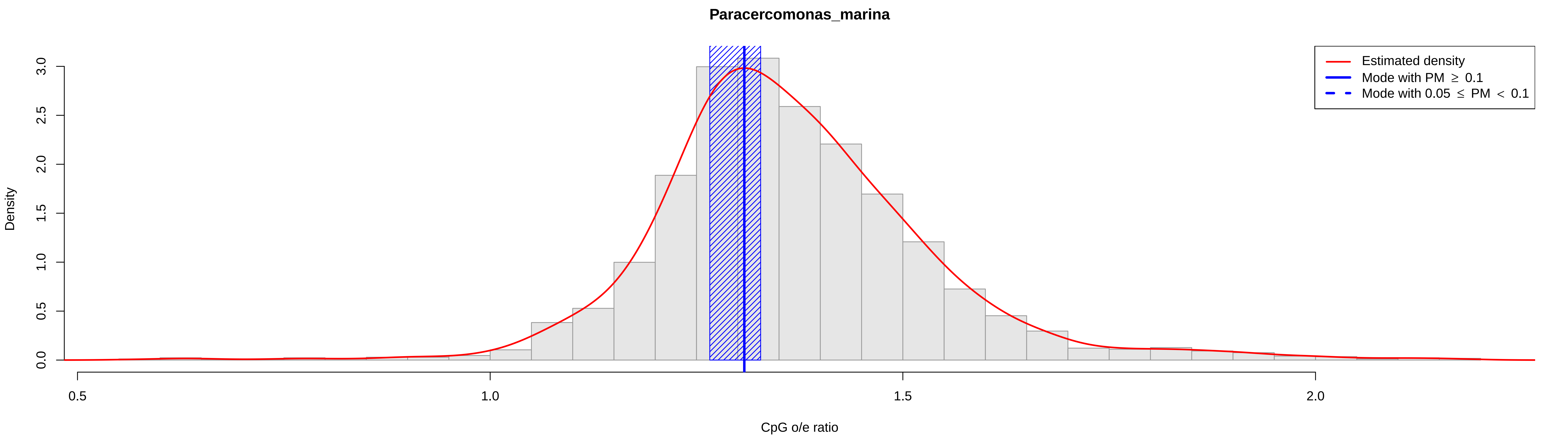
Paracercomonas_marina
Estimated density
Mode with PM ≥ 0.1
Mode with 0.05 ≤ PM < 0.1
Density
0.0
0.5
1.0
1.5
2.0
2.5
3.0
0.5
1.0
1.5
2.0
CpG o/e ratio

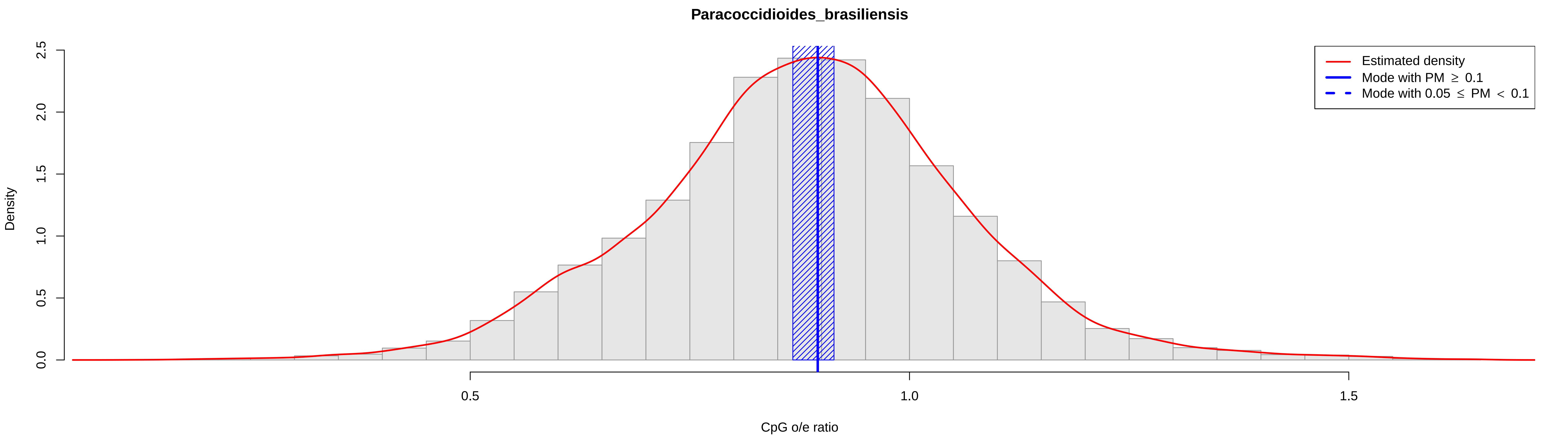

Paracoccidioides_brasiliensis
Density
0.0
0.5
1.0
1.5
2.0
2.5
0.5
1.0
1.5
CpG o/e ratio
Estimated density
Mode with PM ≥ 0.1
Mode with 0.05 ≤ PM < 0.1

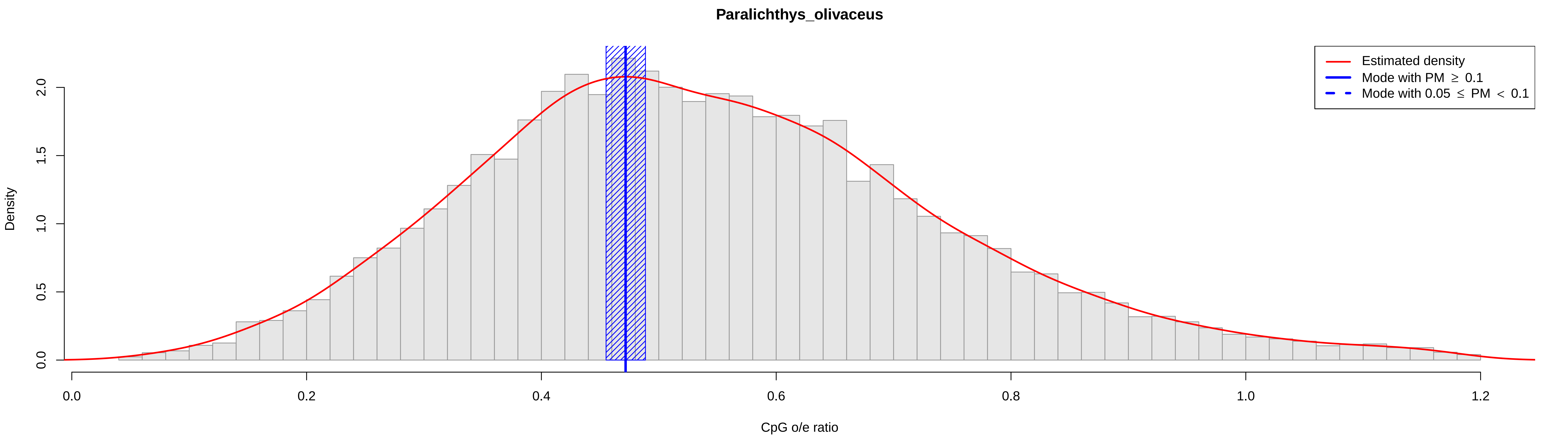
Paralichthys_olivaceus
Estimated density
Mode with PM ≥ 0.1
Mode with 0.05 ≤ PM < 0.1
Density
0.0
0.5
1.0
1.5
2.0
0.0
0.2
0.4
0.6
0.8
1.0
1.2
CpG o/e ratio

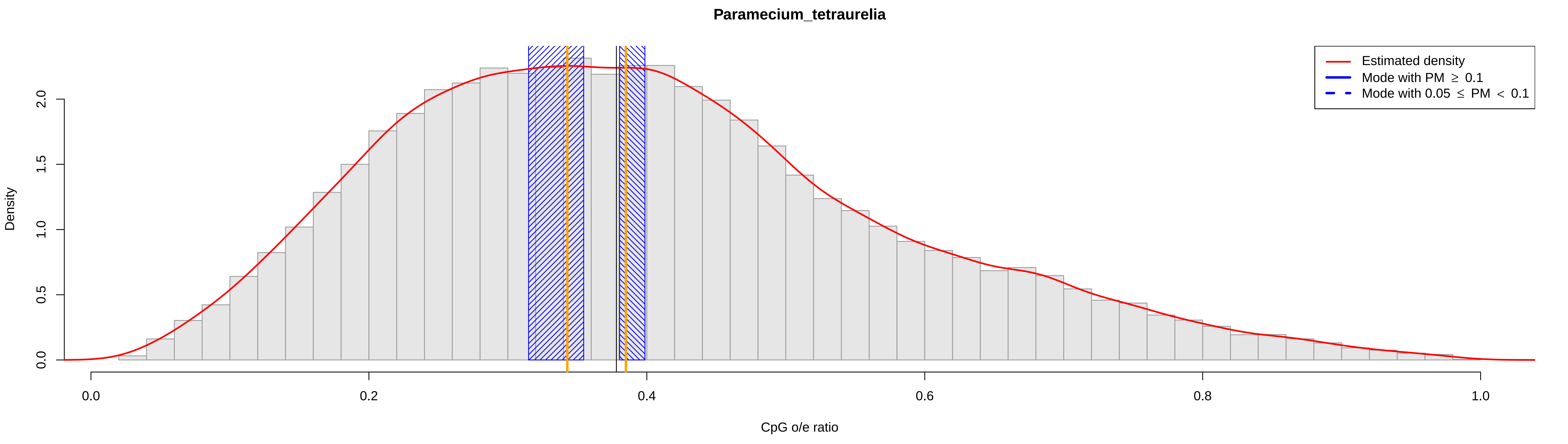

Paramecium_tetraurelia
Estimated density
Mode with PM ≥ 0.1
Mode with 0.05 ≤ PM < 0.1
Density
0.0
0.5
1.0
1.5
2.0
0.0
0.2
0.4
0.6
0.8
1.0
CpG o/e ratio

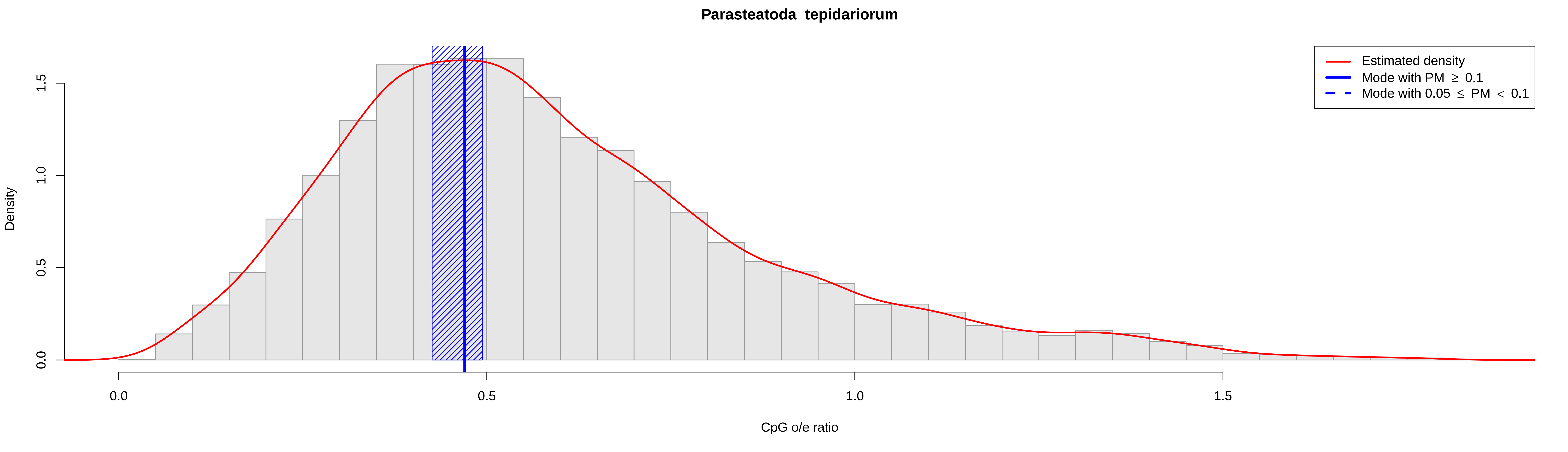

Parasteatoda_tepidariorum
Estimated density
Mode with PM ≥ 0.1
Mode with 0.05 ≤ PM < 0.1
Density
0.0
0.5
1.0
1.5
CpG o/e ratio

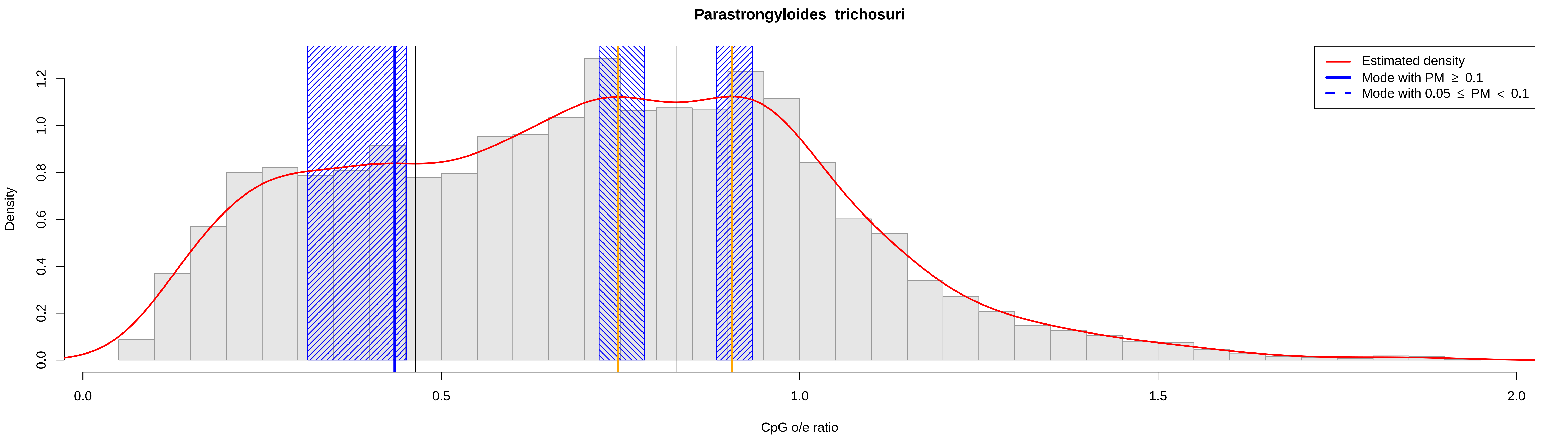

Parastrongyloides_trichosuri
Estimated density
Mode with PM ≥ 0.1
Mode with 0.05 ≤ PM < 0.1
Density
0.0
0.2
0.4
0.6
0.8
1.0
1.2
0.0
0.5
1.0
1.5
2.0
CpG o/e ratio

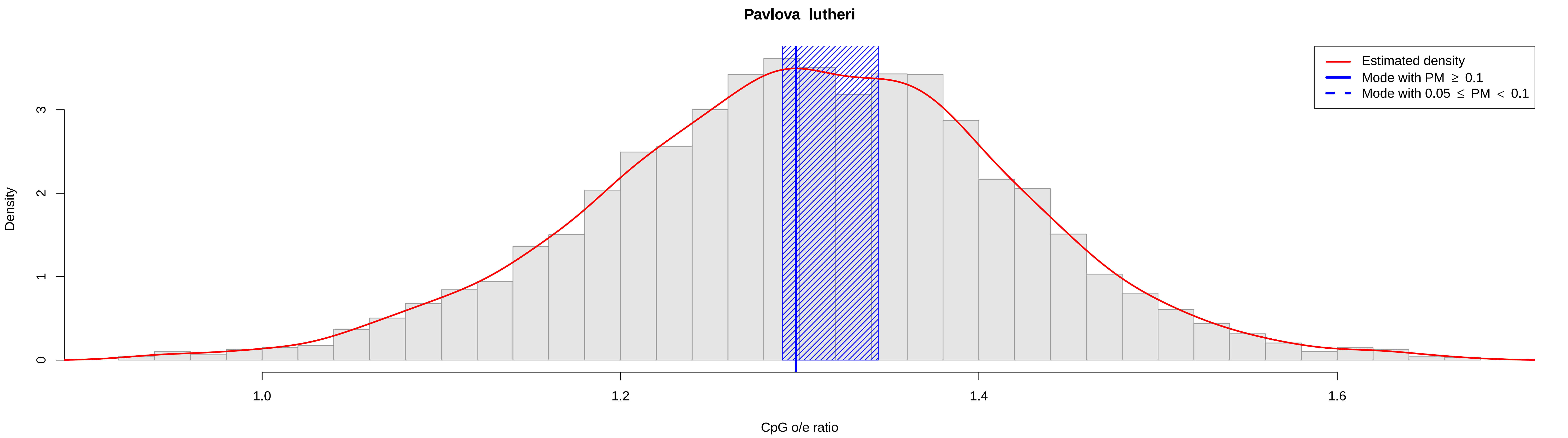
Pavlova_lutheri
Estimated density
Mode with PM ≥ 0.1
Mode with 0.05 ≤ PM < 0.1
Density
0
1
2
3
1.0
1.2
1.4
1.6
CpG o/e ratio

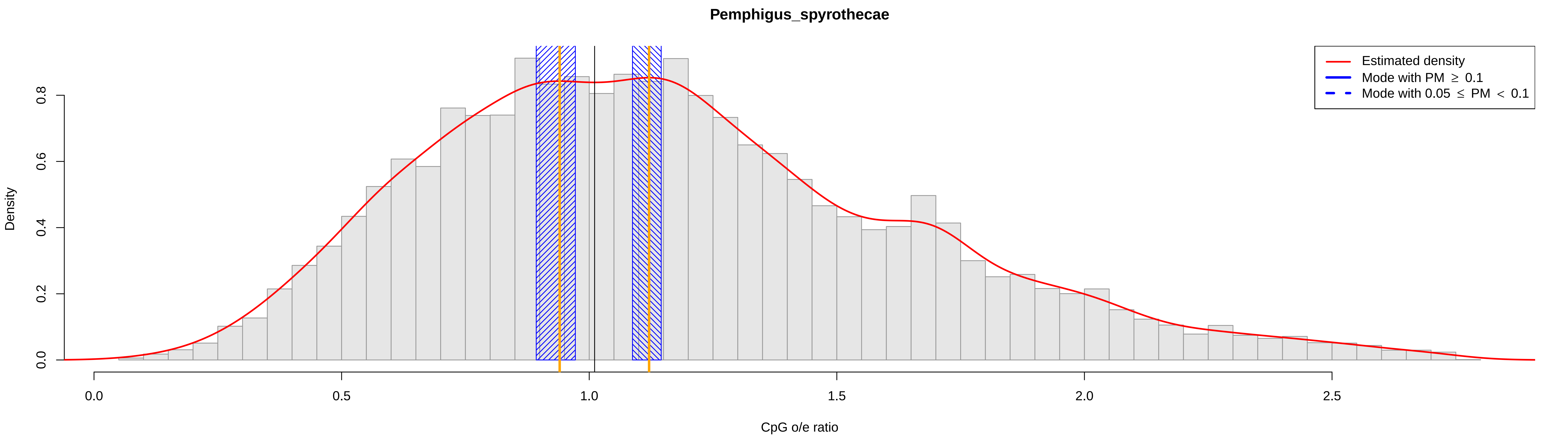

Pemphigus_spyrothecae
Estimated density
Mode with PM ≥ 0.1
Mode with 0.05 ≤ PM < 0.1
Density
0.0
0.2
0.4
0.6
0.8
0.0
0.5
1.0
1.5
2.0
2.5
CpG o/e ratio

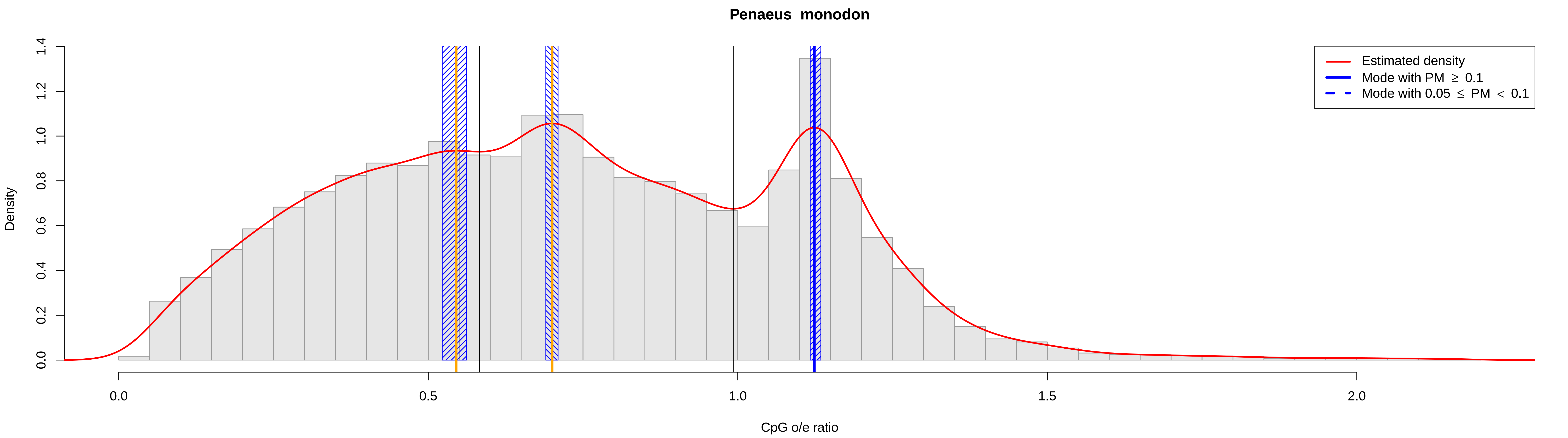
Penaeus_monodon
Estimated density
Mode with PM ≥ 0.1
Mode with 0.05 ≤ PM < 0.1
Density
0.0
0.2
0.4
0.6
0.8
1.0
1.2
1.4
0.0
0.5
1.0
1.5
2.0
CpG o/e ratio

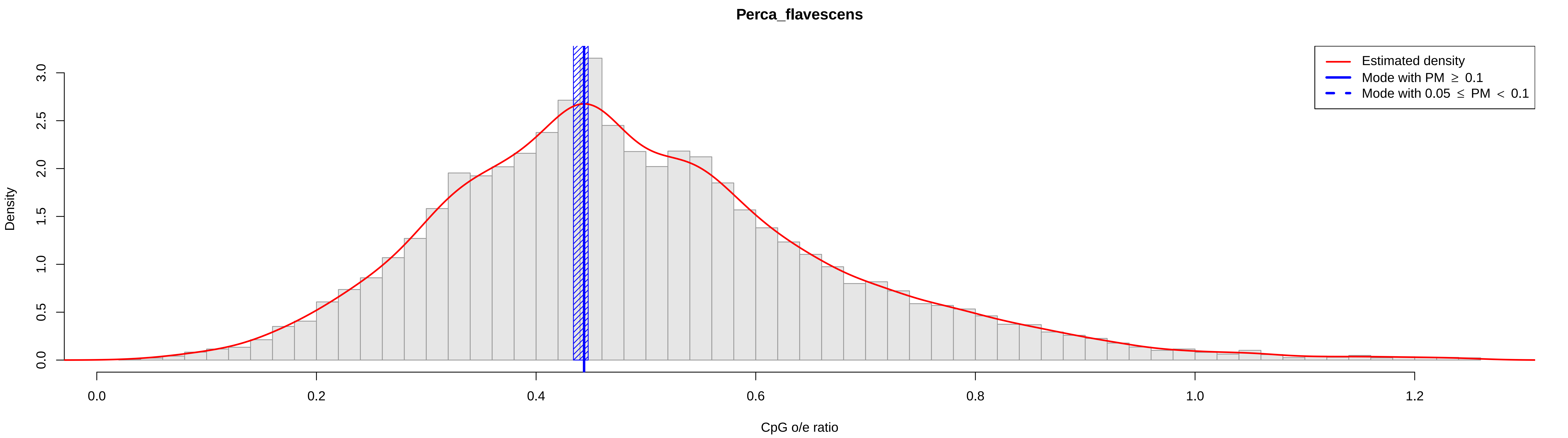

Perca_flavescens
Estimated density
Mode with PM ≥ 0.1
Mode with 0.05 ≤ PM < 0.1
Density
0.0
0.5
1.0
1.5
2.0
2.5
3.0
0.0
0.2
0.4
0.6
0.8
1.0
1.2
CpG o/e ratio

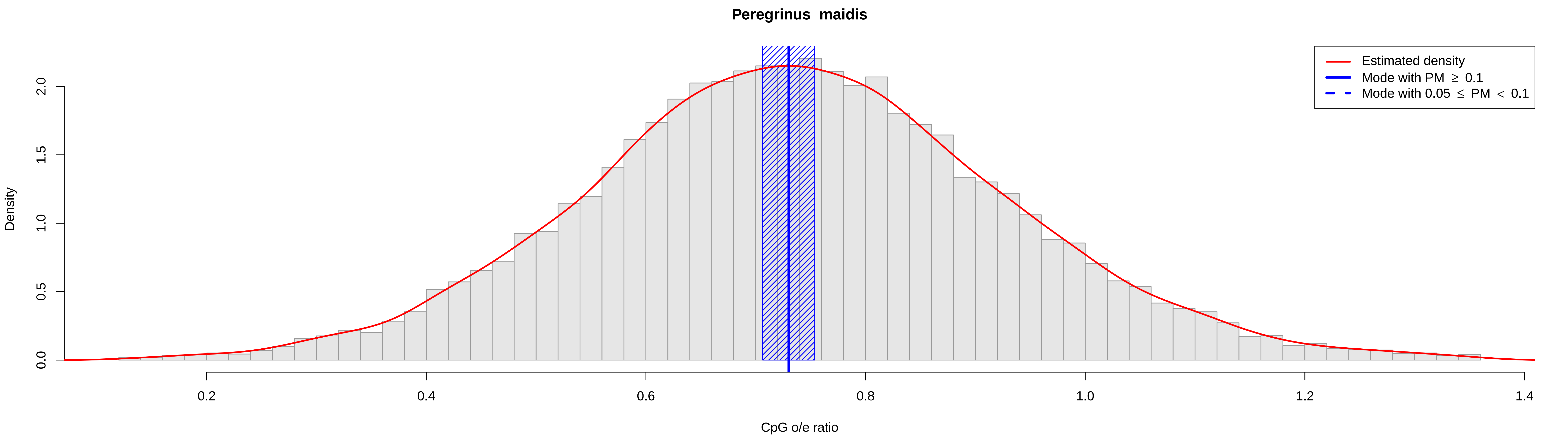

Peregrinus_maidis
Estimated density
Mode with PM ≥ 0.1
Mode with 0.05 ≤ PM < 0.1
Density
0.0
0.5
1.0
1.5
2.0
0.2
0.4
0.6
0.8
1.0
1.2
1.4
CpG o/e ratio

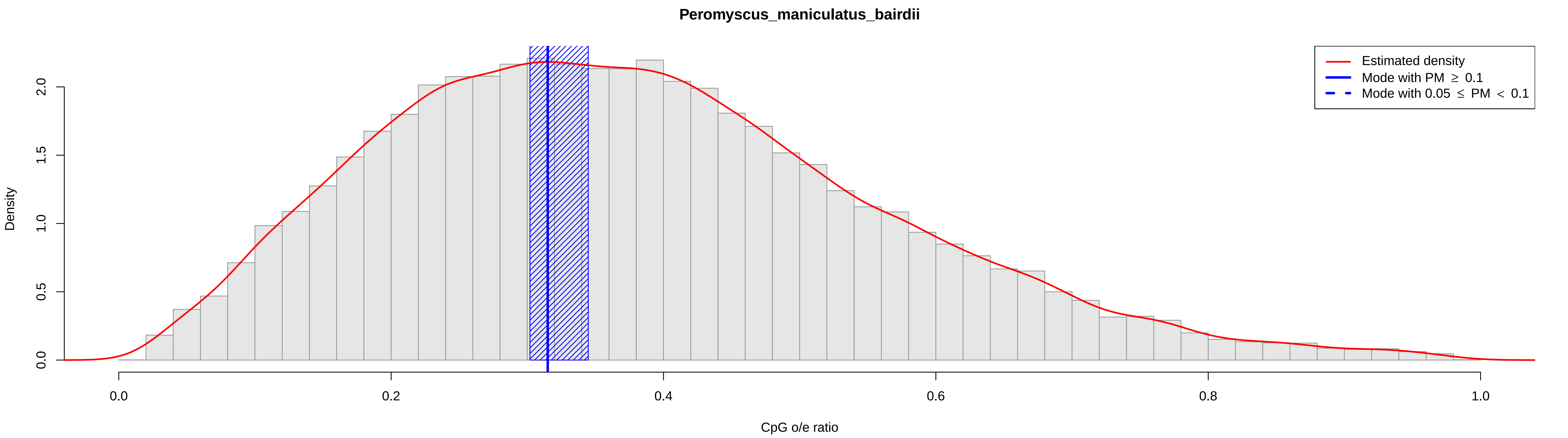
Peromyscus_maniculatus_bairdii
Estimated density
Mode with PM ≥ 0.1
Mode with 0.05 ≤ PM < 0.1
Density
CpG o/e ratio
0.0
0.5
1.0
1.5
2.0
0.0
0.2
0.4
0.6
0.8
1.0

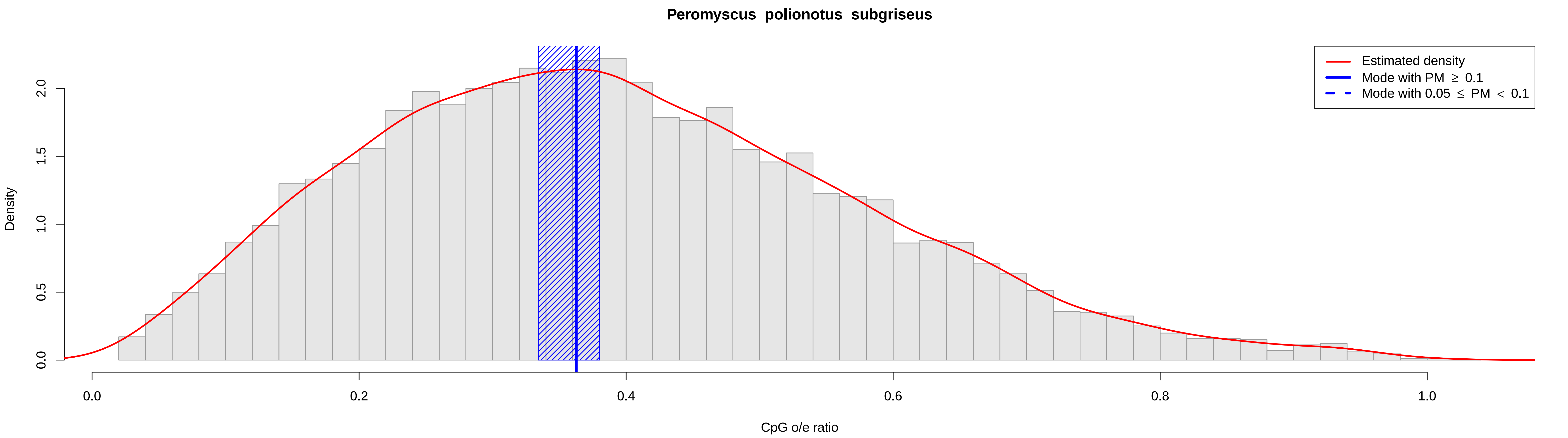

Peromyscus_polionotus_subgriseus
Estimated density
Mode with PM ≥ 0.1
Mode with 0.05 ≤ PM < 0.1
Density
0.0
0.5
1.0
1.5
2.0
0.0
0.2
0.4
0.6
0.8
1.0
CpG o/e ratio

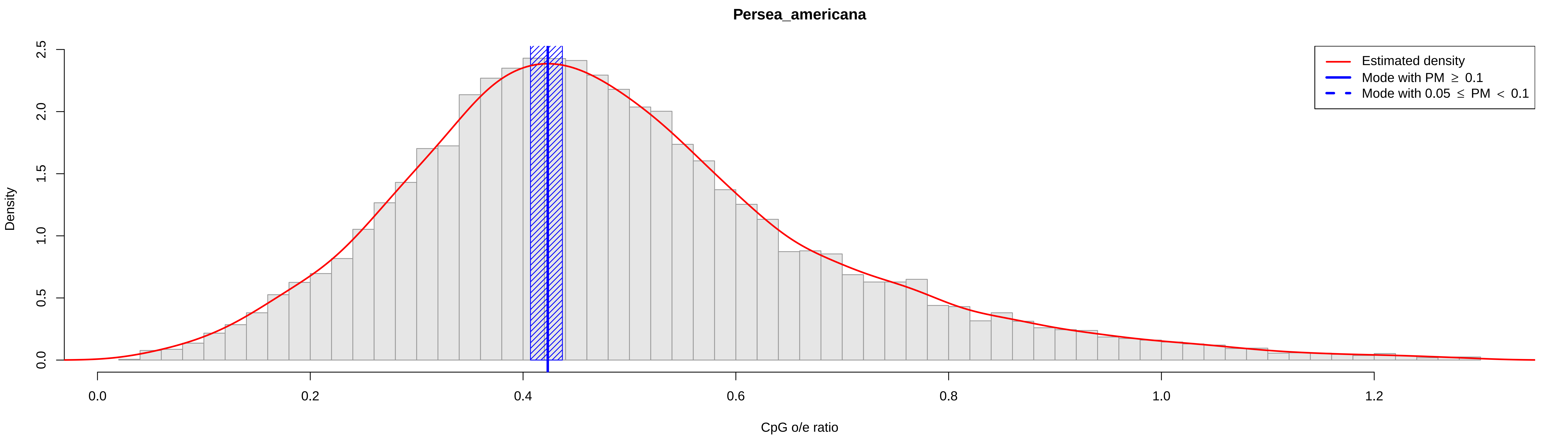

Persea_americana
Estimated density
Mode with PM ≥ 0.1
Mode with 0.05 ≤ PM < 0.1
Density
0.0
0.5
1.0
1.5
2.0
2.5
0.0
0.2
0.4
0.6
0.8
1.0
1.2
CpG o/e ratio

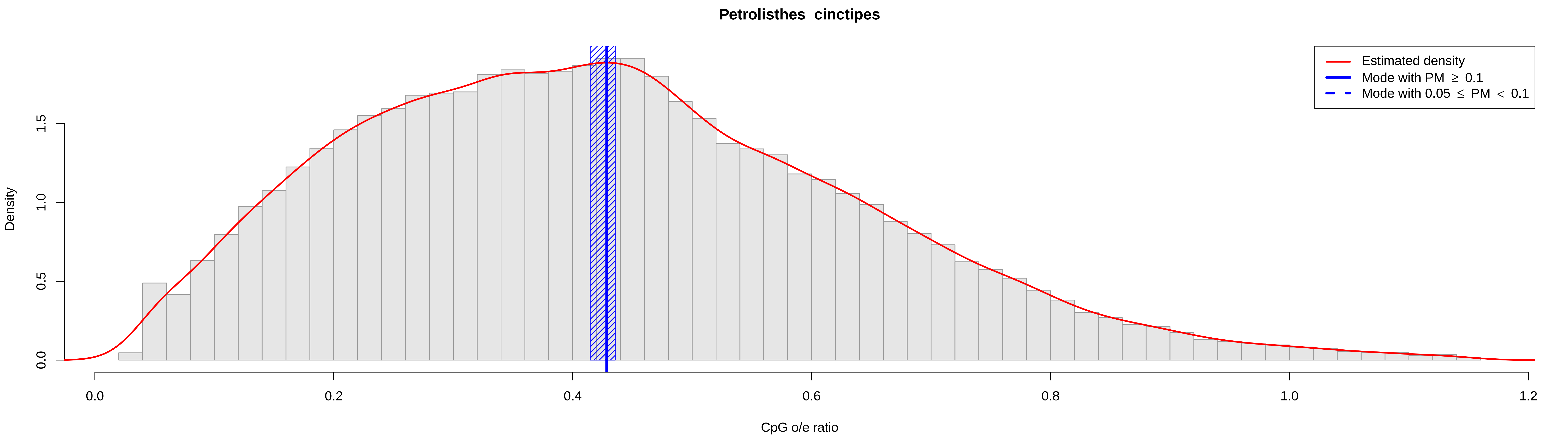

Petrolisthes_cinctipes
Estimated density
Mode with PM ≥ 0.1
Mode with 0.05 ≤ PM < 0.1
Density
0.0
0.5
1.0
1.5
0.0
0.2
0.4
0.6
0.8
1.0
1.2
CpG o/e ratio

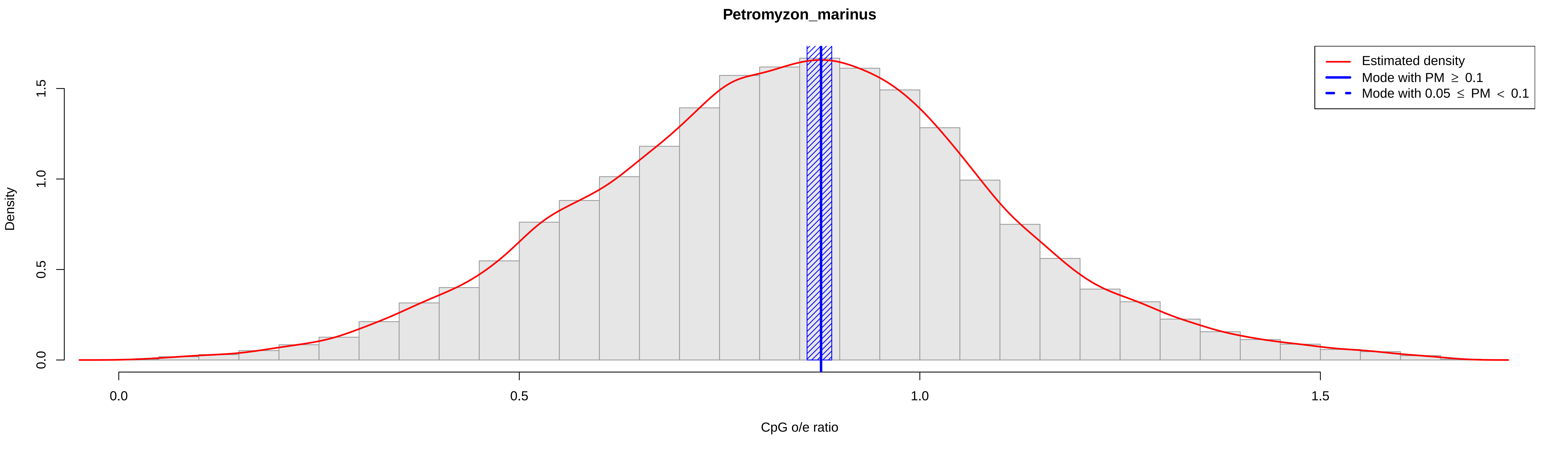
Petromyzon_marinus
Estimated density
Mode with PM ≥ 0.1
Mode with 0.05 ≤ PM < 0.1
Density
0.0
0.5
1.0
1.5
CpG o/e ratio

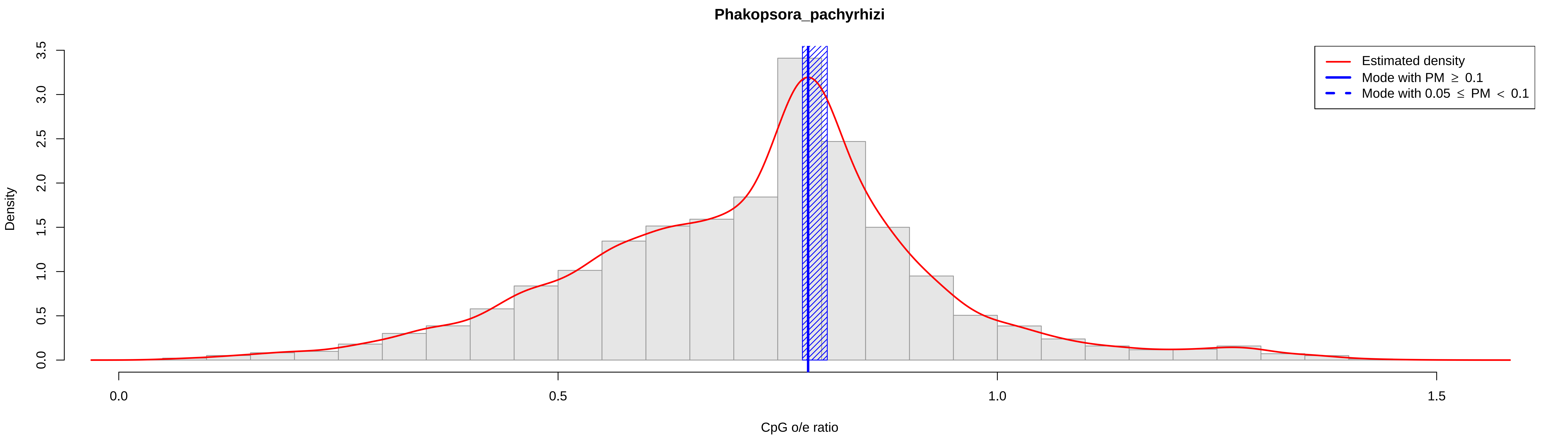

Phakopsora_pachyrhizi
Estimated density
Mode with PM ≥ 0.1
Mode with 0.05 ≤ PM < 0.1
Density
0.0
0.5
1.0
1.5
2.0
2.5
3.0
3.5
0.0
0.5
1.0
1.5
CpG o/e ratio

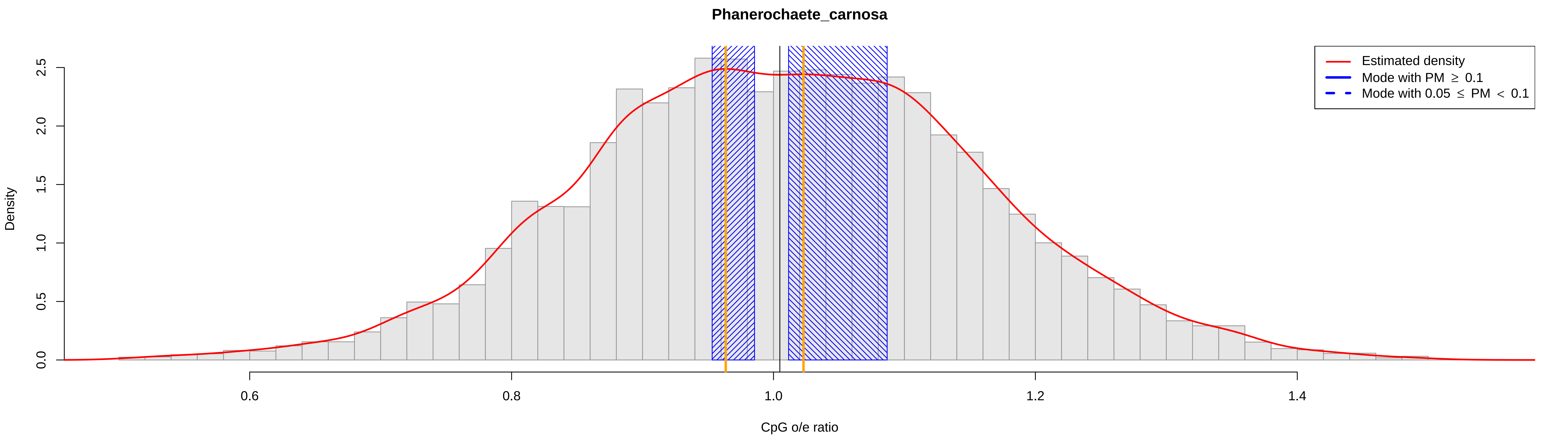

Phanerochaete_carnosa
Density
CpG o/e ratio
0.0
0.5
1.0
1.5
2.0
2.5
0.6
0.8
1.0
1.2
1.4
Estimated density
Mode with PM ≥ 0.1
Mode with 0.05 ≤ PM < 0.1

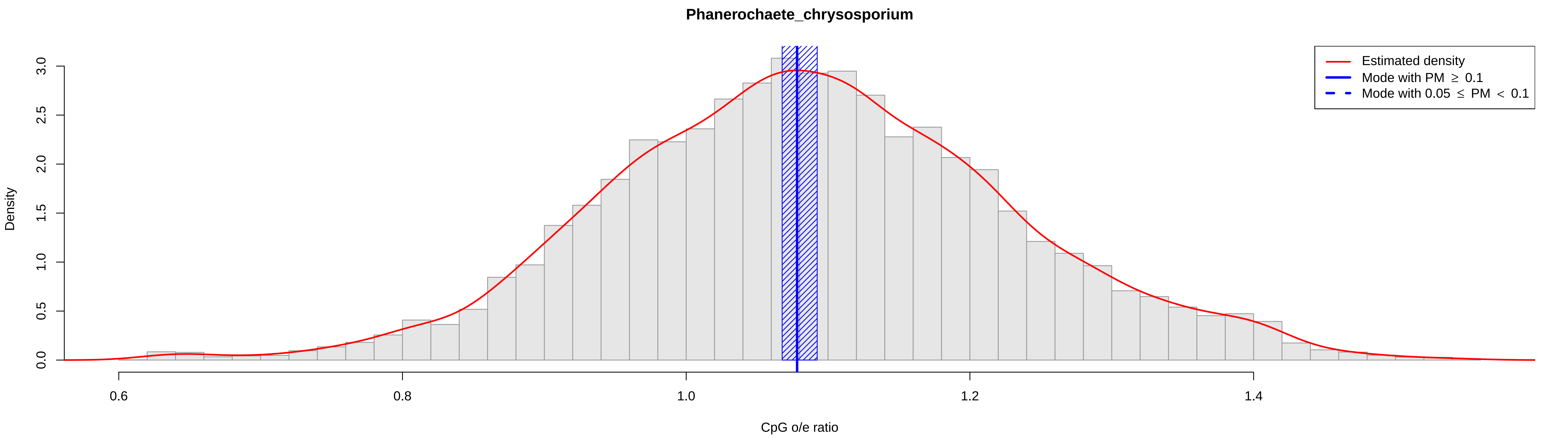
Phanerochaete_chrysosporium
Estimated density
Mode with PM ≥ 0.1
Mode with 0.05 ≤ PM < 0.1
Density
0.0
0.5
1.0
1.5
2.0
2.5
3.0
0.6
0.8
1.0
1.2
1.4
CpG o/e ratio

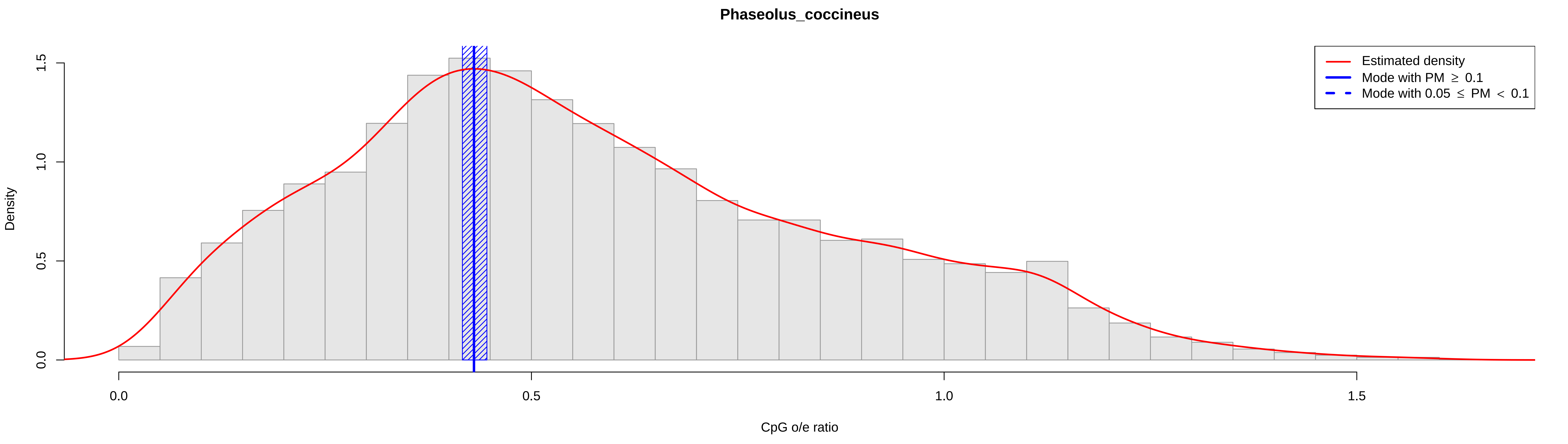

Phaseolus_coccineus
Estimated density
Mode with PM ≥ 0.1
Mode with 0.05 ≤ PM < 0.1
Density
0.0
0.5
1.0
1.5
0.0
0.5
1.0
1.5
CpG o/e ratio

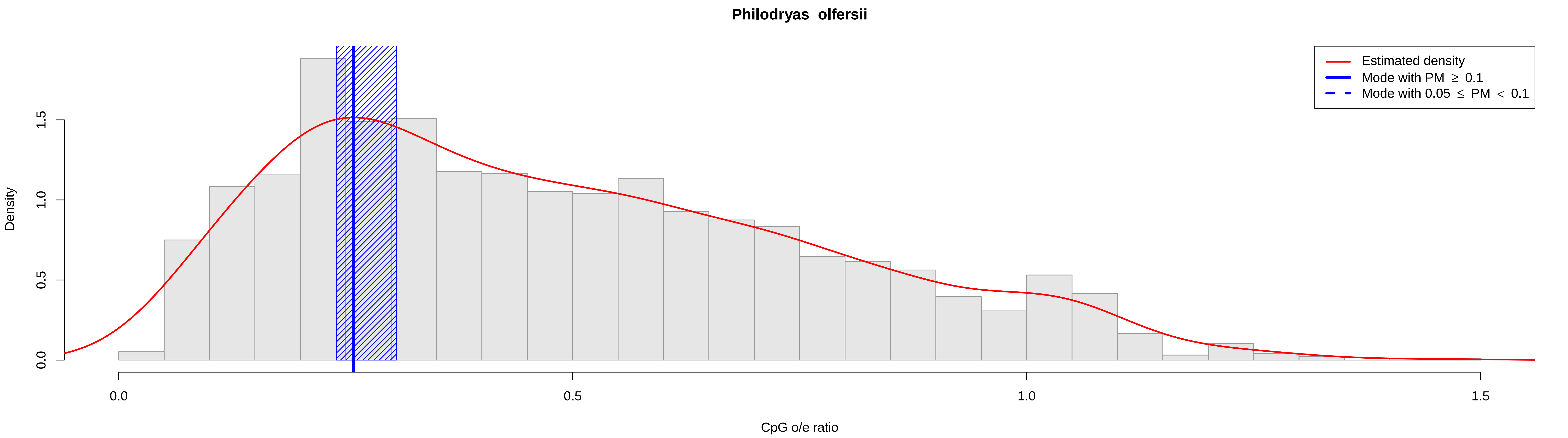
Philodryas_olfersii
Estimated density
Mode with PM ≥ 0.1
Mode with 0.05 ≤ PM < 0.1
Density
0.0
0.5
1.0
1.5
0.0
0.5
1.0
1.5
CpG o/e ratio

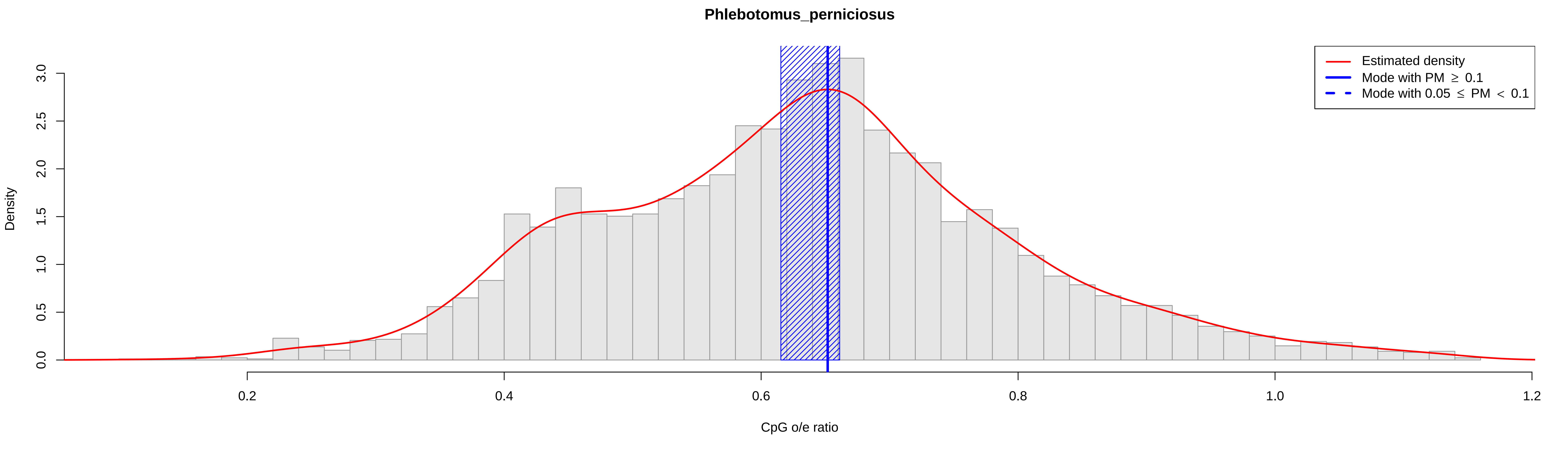
Phlebotomus_perniciosus
Estimated density
Mode with PM ≥ 0.1
Mode with 0.05 ≤ PM < 0.1
Density
0.0
0.5
1.0
1.5
2.0
2.5
3.0
0.2
0.4
0.6
0.8
1.0
1.2
CpG o/e ratio

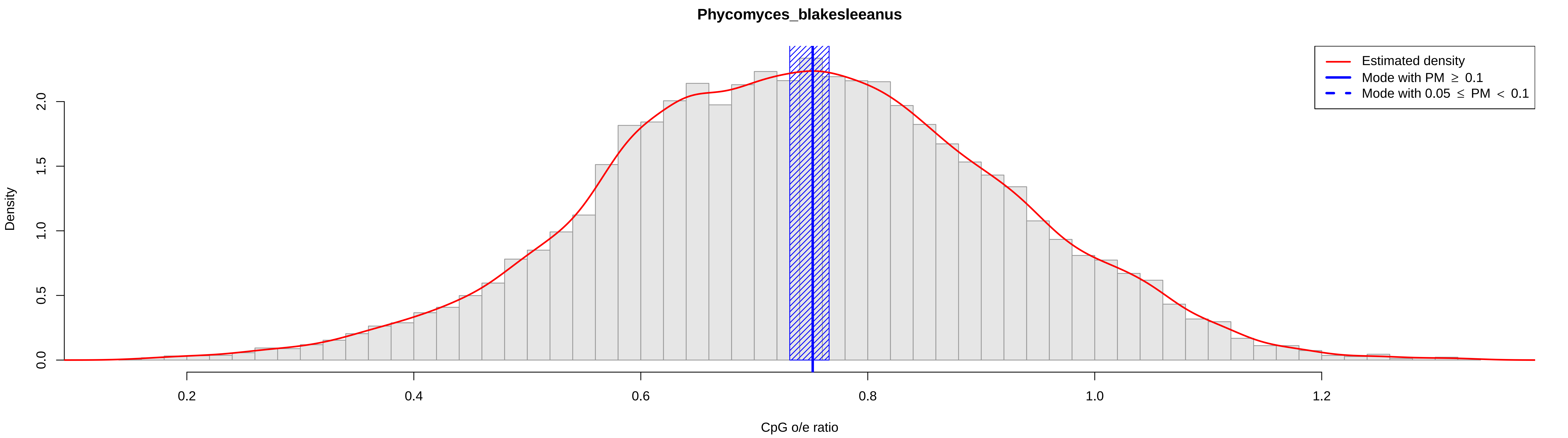

Phycomyces_blakesleeanus
Estimated density
Mode with PM ≥ 0.1
Mode with 0.05 ≤ PM < 0.1
Density
0.0
0.5
1.0
1.5
2.0
0.2
0.4
0.6
0.8
1.0
1.2
CpG o/e ratio

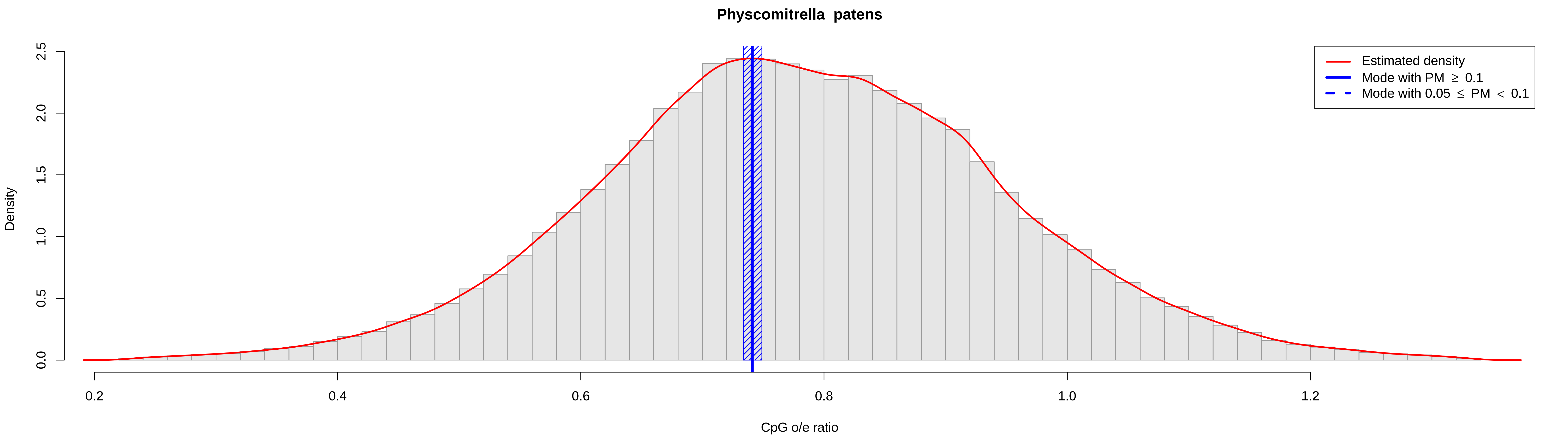

Physcomitrella_patens
Estimated density
Mode with PM ≥ 0.1
Mode with 0.05 ≤ PM < 0.1
Density
0.0
0.5
1.0
1.5
2.0
2.5
0.2
0.4
0.6
0.8
1.0
1.2
CpG o/e ratio

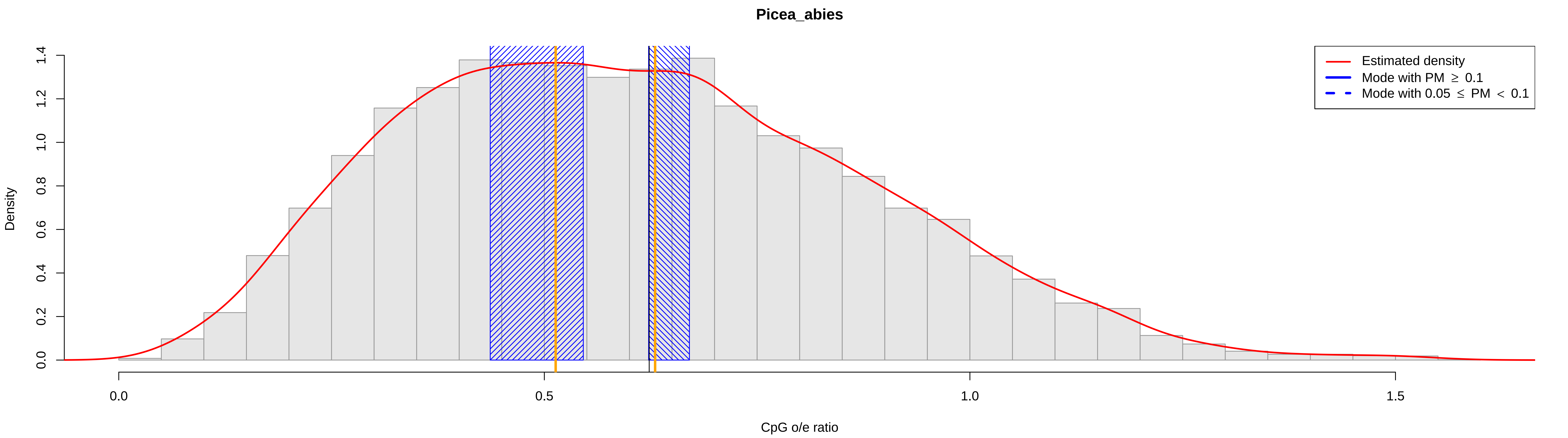

Picea_abies
Estimated density
Mode with PM ≥ 0.1
Mode with 0.05 ≤ PM < 0.1
Density
0.0
0.2
0.4
0.6
0.8
1.0
1.2
1.4
0.0
0.5
1.0
1.5
CpG o/e ratio

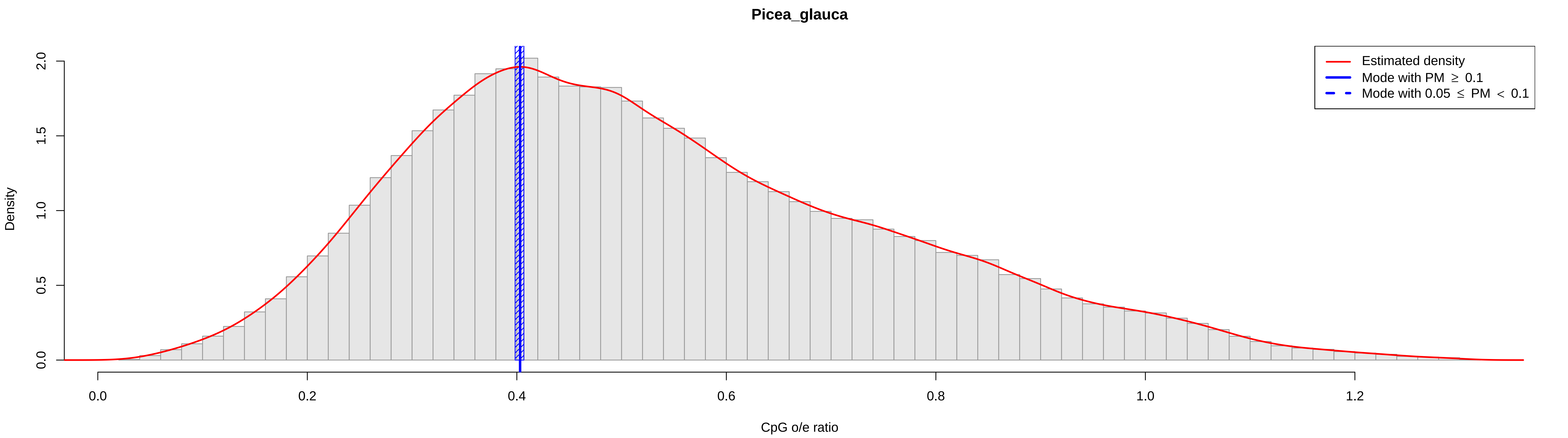

Picea_glauca
Estimated density
Mode with PM ≥ 0.1
Mode with 0.05 ≤ PM < 0.1
Density
0.0
0.5
1.0
1.5
2.0
0.0
0.2
0.4
0.6
0.8
1.0
1.2
CpG o/e ratio

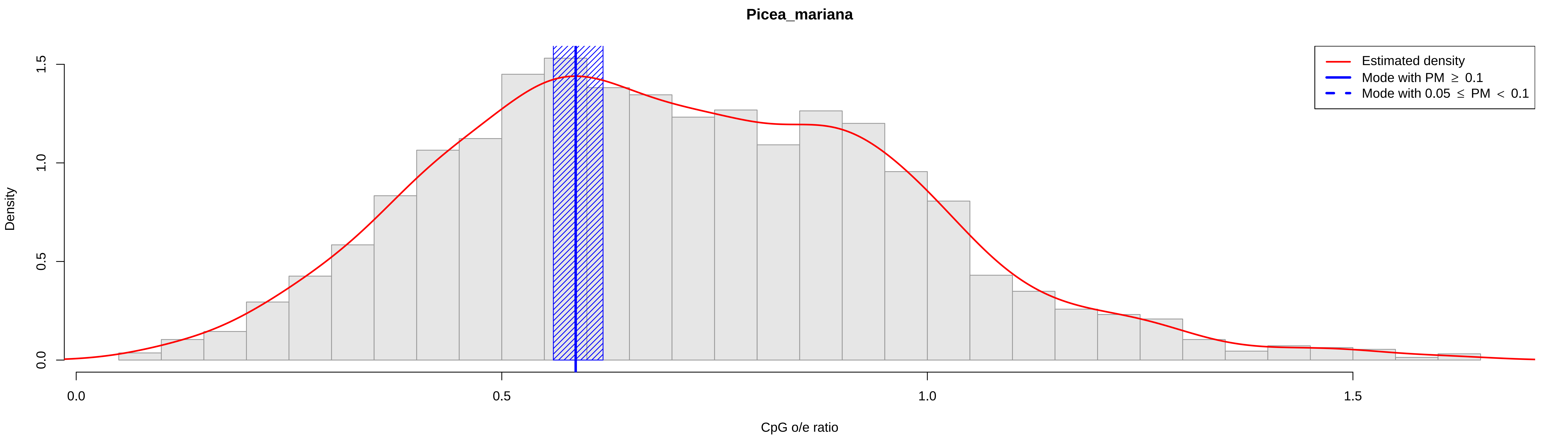

Picea_mariana
Density
CpG o/e ratio
0.0
0.5
1.0
1.5
Estimated density
Mode with PM ≥ 0.1
Mode with 0.05 ≤ PM < 0.1

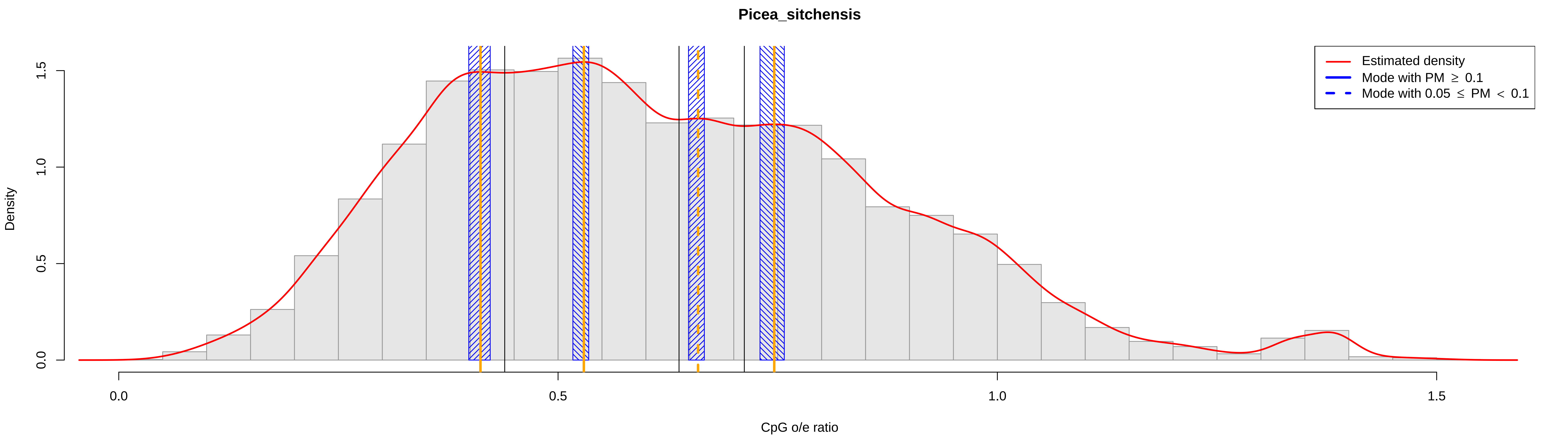

Picea_sitchensis
Estimated density
Mode with PM ≥ 0.1
Mode with 0.05 ≤ PM < 0.1
Density
0.0
0.5
1.0
1.5
0.0
0.5
1.0
1.5
CpG o/e ratio

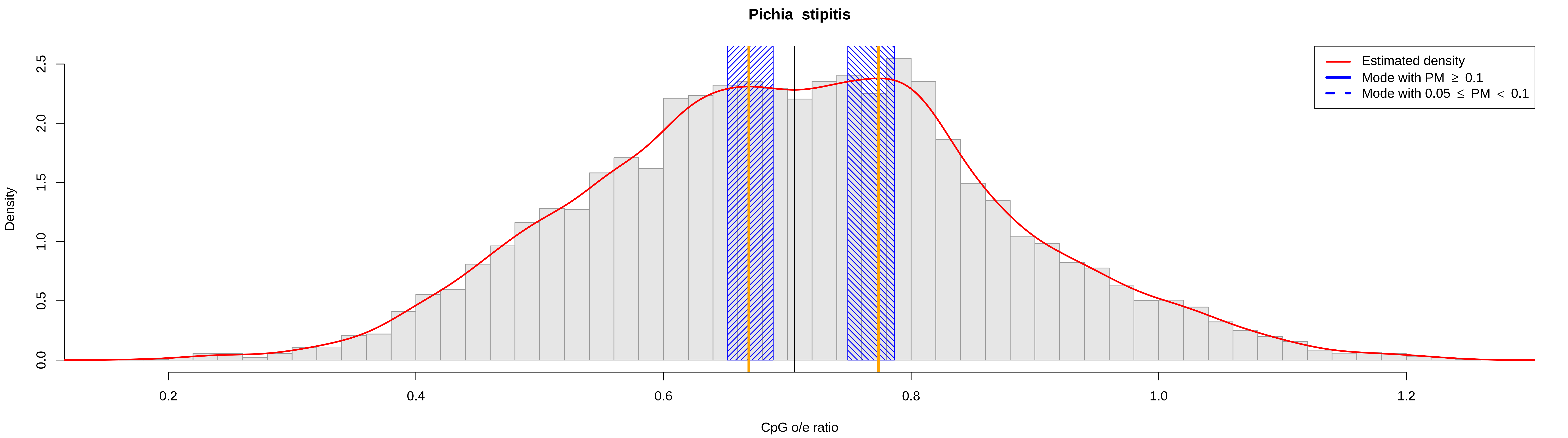

Pichia_stipitis
Estimated density
Mode with PM ≥ 0.1
Mode with 0.05 ≤ PM < 0.1
Density
0.0
0.5
1.0
1.5
2.0
2.5
0.2
0.4
0.6
0.8
1.0
1.2
CpG o/e ratio

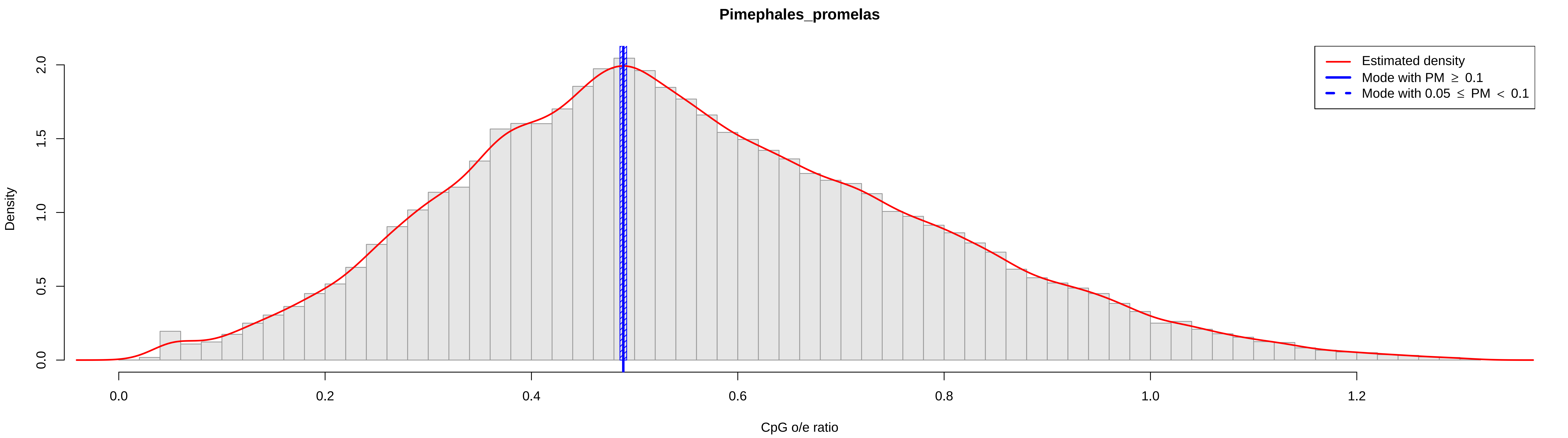

Pimephales_promelas
Estimated density
Mode with PM ≥ 0.1
Mode with 0.05 ≤ PM < 0.1
Density
0.0
0.5
1.0
1.5
2.0
0.0
0.2
0.4
0.6
0.8
1.0
1.2
CpG o/e ratio

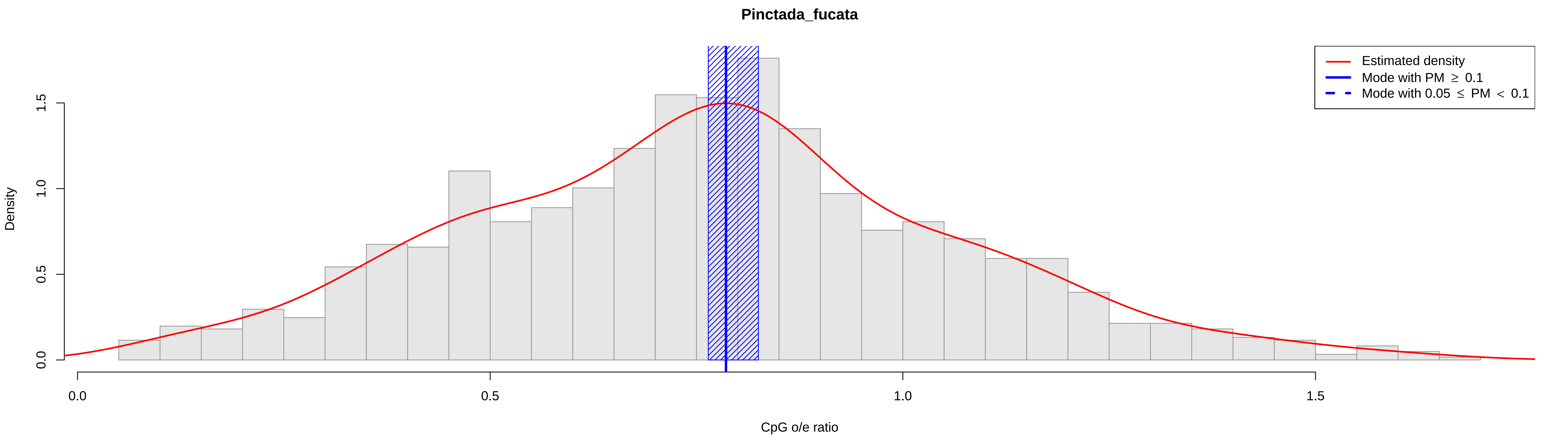

Pinctada_fucata
Estimated density
Mode with PM ≥ 0.1
Mode with 0.05 ≤ PM < 0.1
Density
0.0
0.5
1.0
1.5
CpG o/e ratio

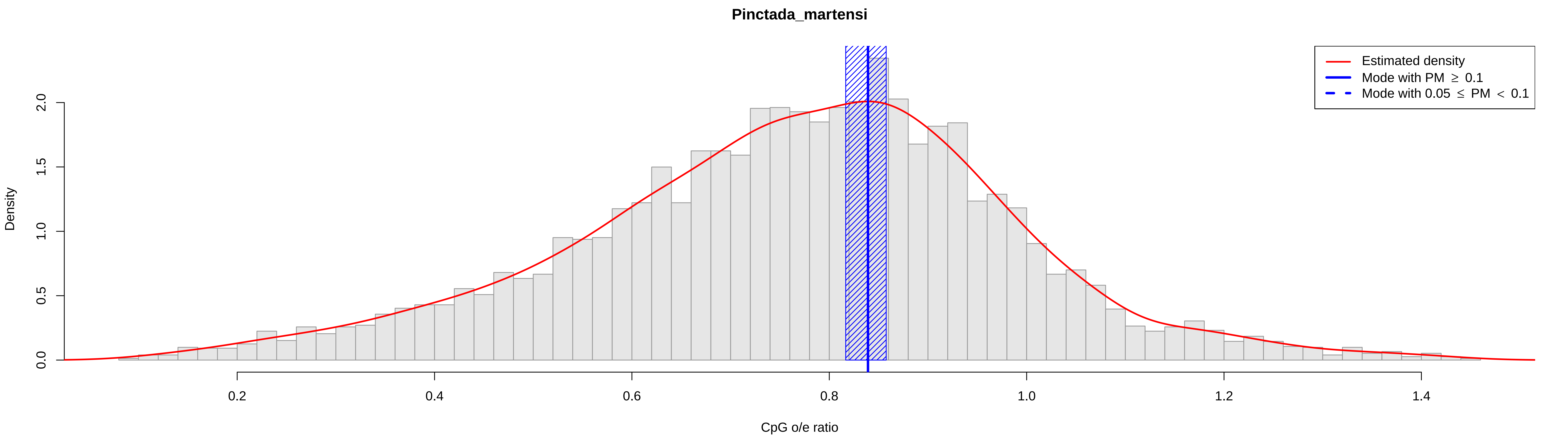

Pinctada_martensi
Estimated density
Mode with PM ≥ 0.1
Mode with 0.05 ≤ PM < 0.1
Density
0.0
0.5
1.0
1.5
2.0
0.2
0.4
0.6
0.8
1.0
1.2
1.4
CpG o/e ratio

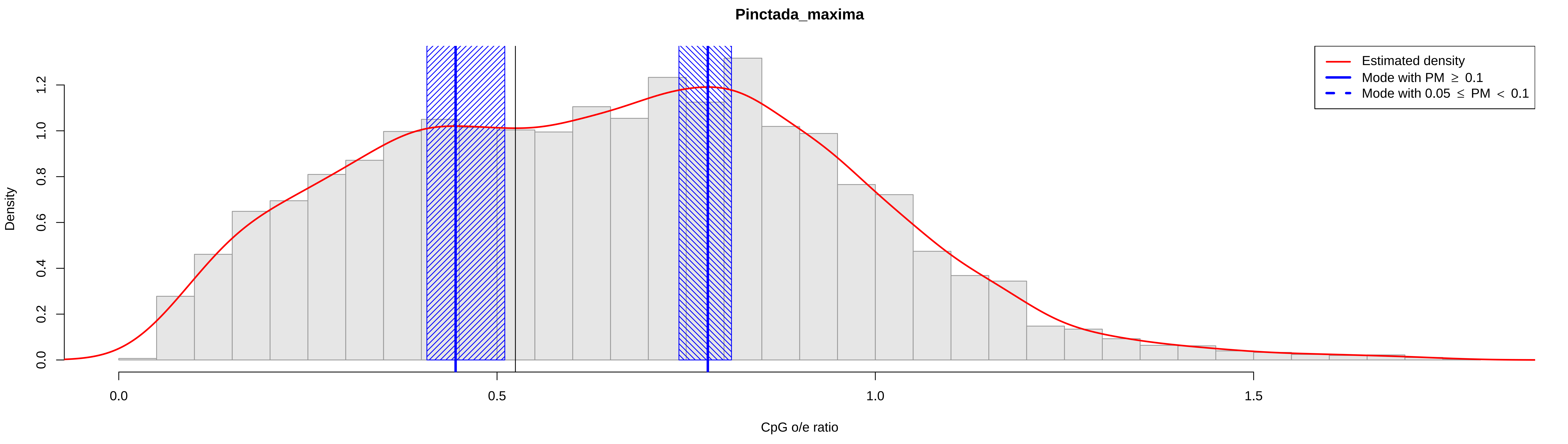

Pinctada_maxima
Estimated density
Mode with PM ≥ 0.1
Mode with 0.05 ≤ PM < 0.1
Density
0.0
0.2
0.4
0.6
0.8
1.0
1.2
0.0
0.5
1.0
1.5
CpG o/e ratio

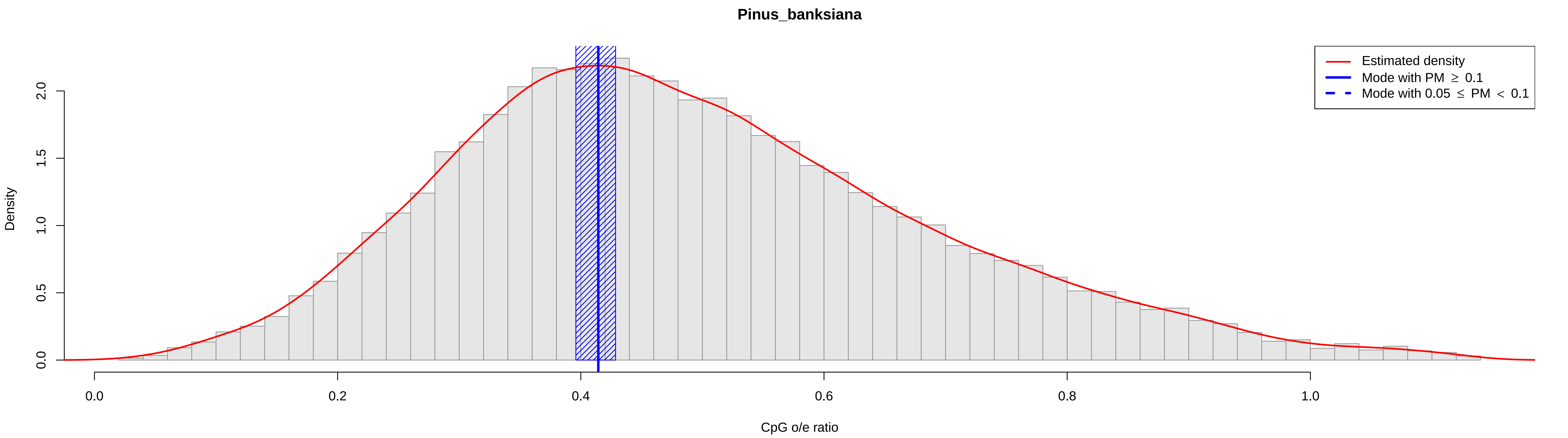

Pinus_banksiana
Estimated density
Mode with PM ≥ 0.1
Mode with 0.05 ≤ PM < 0.1
Density
0.0
0.5
1.0
1.5
2.0
0.0
0.2
0.4
0.6
0.8
1.0
CpG o/e ratio

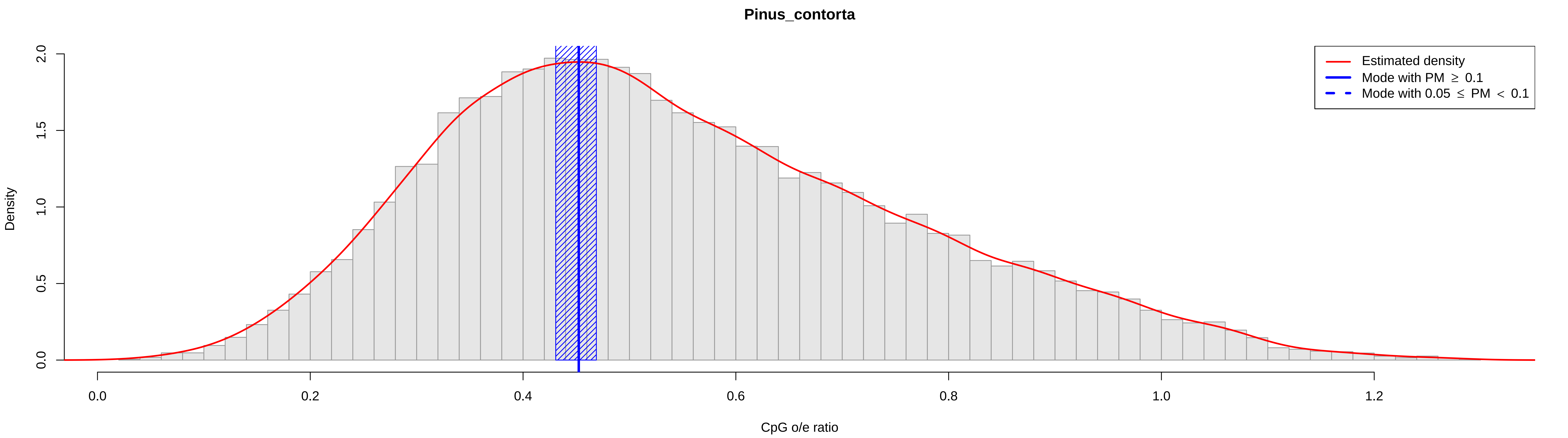
Pinus_contorta
Estimated density
Mode with PM ≥ 0.1
Mode with 0.05 ≤ PM < 0.1
Density
0.0
0.5
1.0
1.5
2.0
0.0
0.2
0.4
0.6
0.8
1.0
1.2
CpG o/e ratio

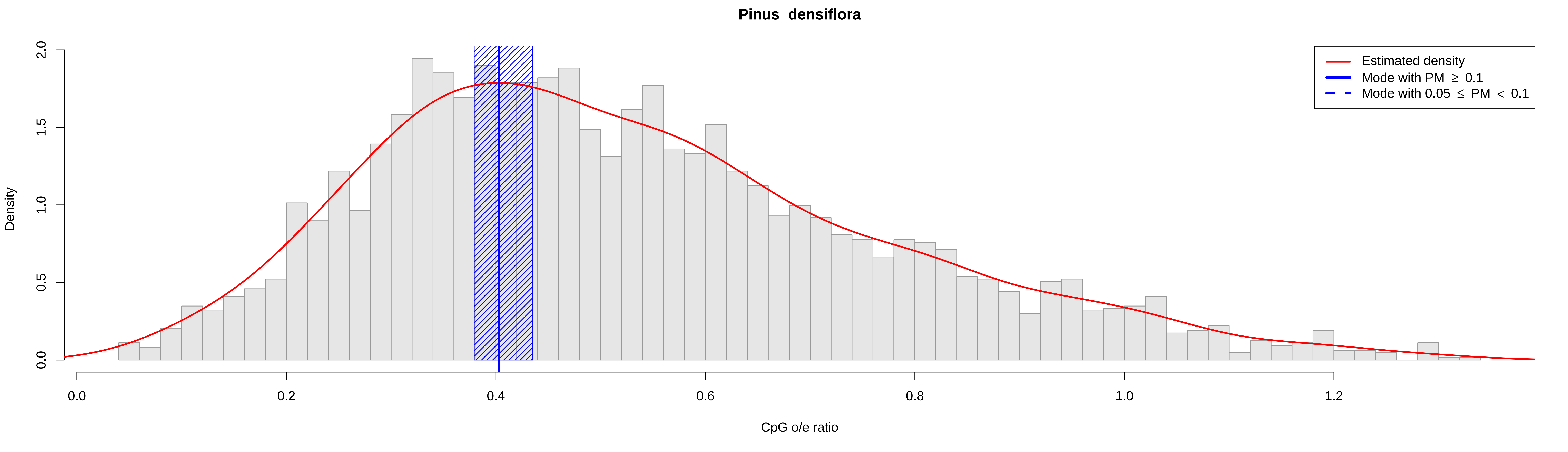

Pinus_densiflora
Estimated density
Mode with PM ≥ 0.1
Mode with 0.05 ≤ PM < 0.1
Density
0.0
0.5
1.0
1.5
2.0
0.0
0.2
0.4
0.6
0.8
1.0
1.2
CpG o/e ratio

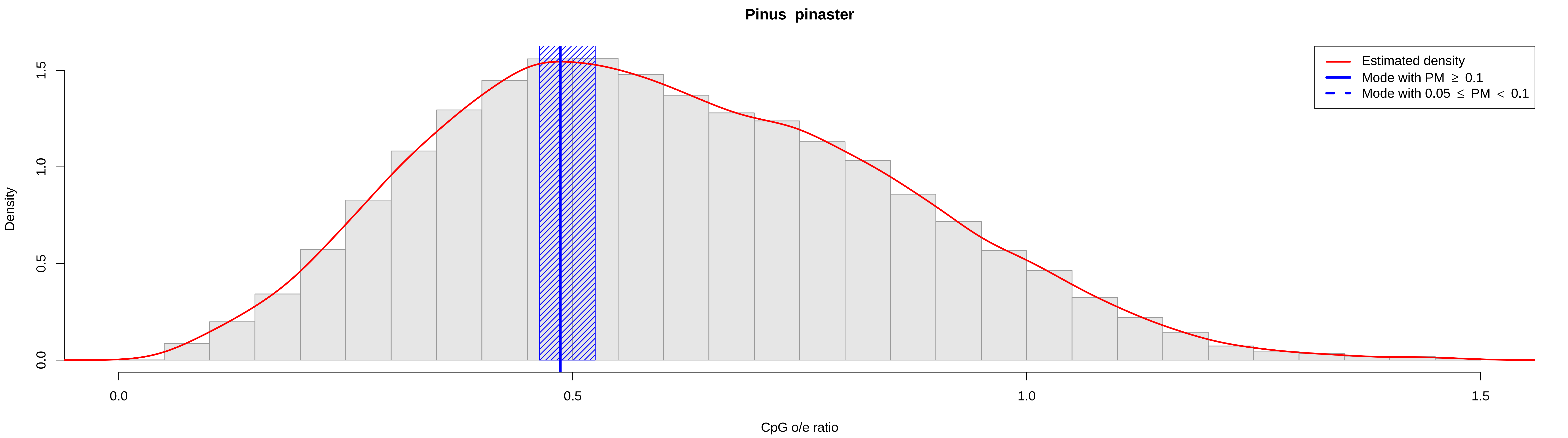

Pinus_pinaster
Estimated density
Mode with PM ≥ 0.1
Mode with 0.05 ≤ PM < 0.1
Density
0.0
0.5
1.0
1.5
CpG o/e ratio
0.0
0.5
1.0
1.5

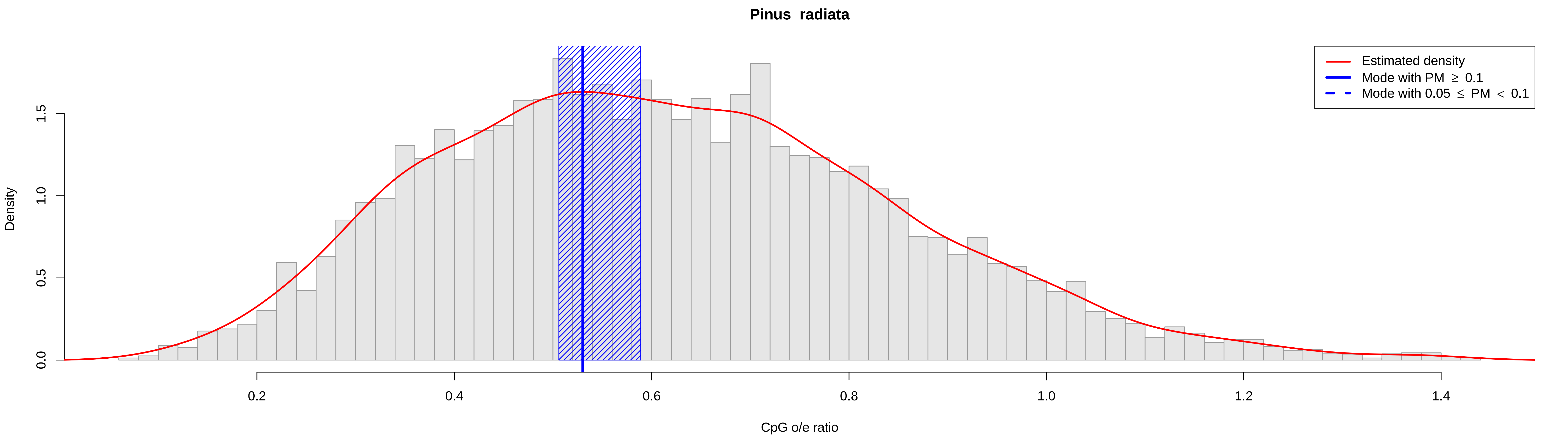
Pinus_radiata
Estimated density
Mode with PM ≥ 0.1
Mode with 0.05 ≤ PM < 0.1
Density
0.0
0.5
1.0
1.5
0.2
0.4
0.6
0.8
1.0
1.2
1.4
CpG o/e ratio

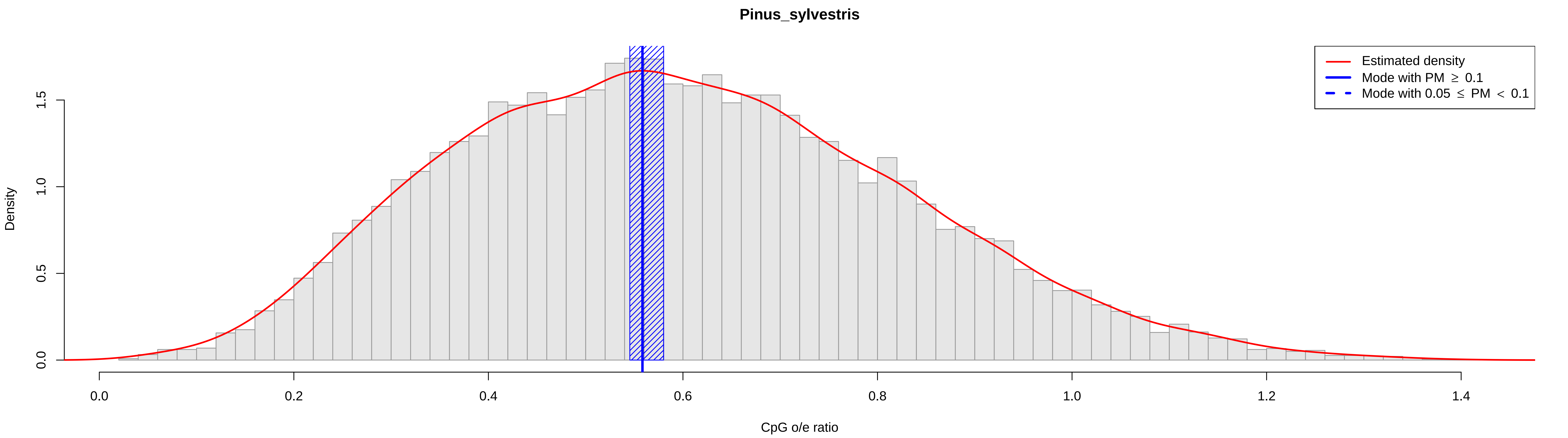

Pinus_sylvestris
Density
CpG o/e ratio
0.0
0.5
1.0
1.5
0.0
0.2
0.4
0.6
0.8
1.0
1.2
1.4
Estimated density
Mode with PM ≥ 0.1
Mode with 0.05 ≤ PM < 0.1

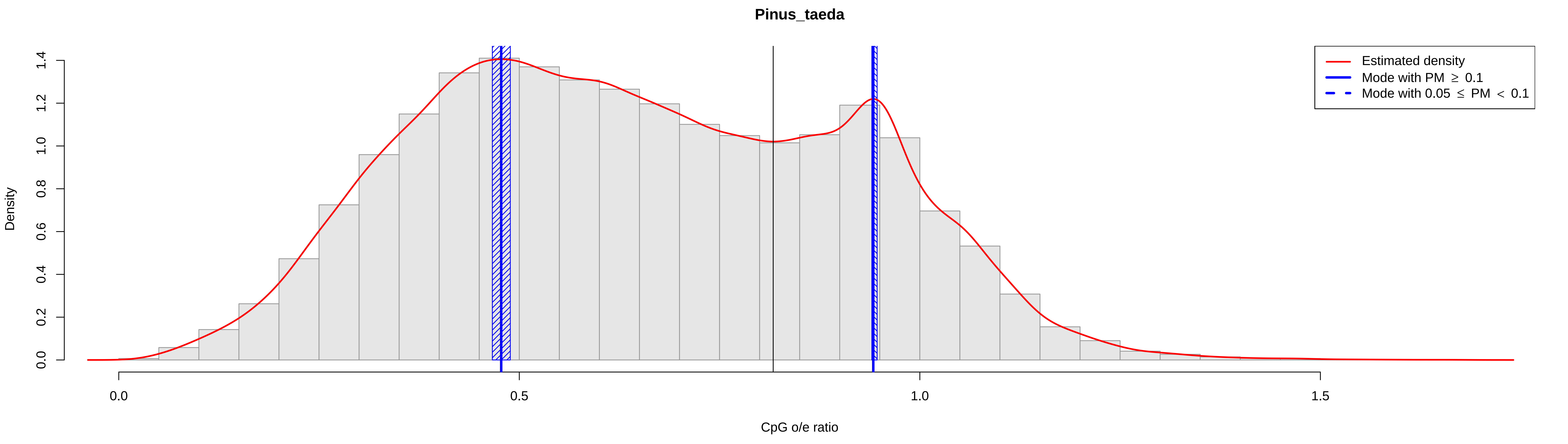

Pinus_taeda
Estimated density
Mode with PM ≥ 0.1
Mode with 0.05 ≤ PM < 0.1
Density
0.0
0.2
0.4
0.6
0.8
1.0
1.2
1.4
0.0
0.5
1.0
1.5
CpG o/e ratio

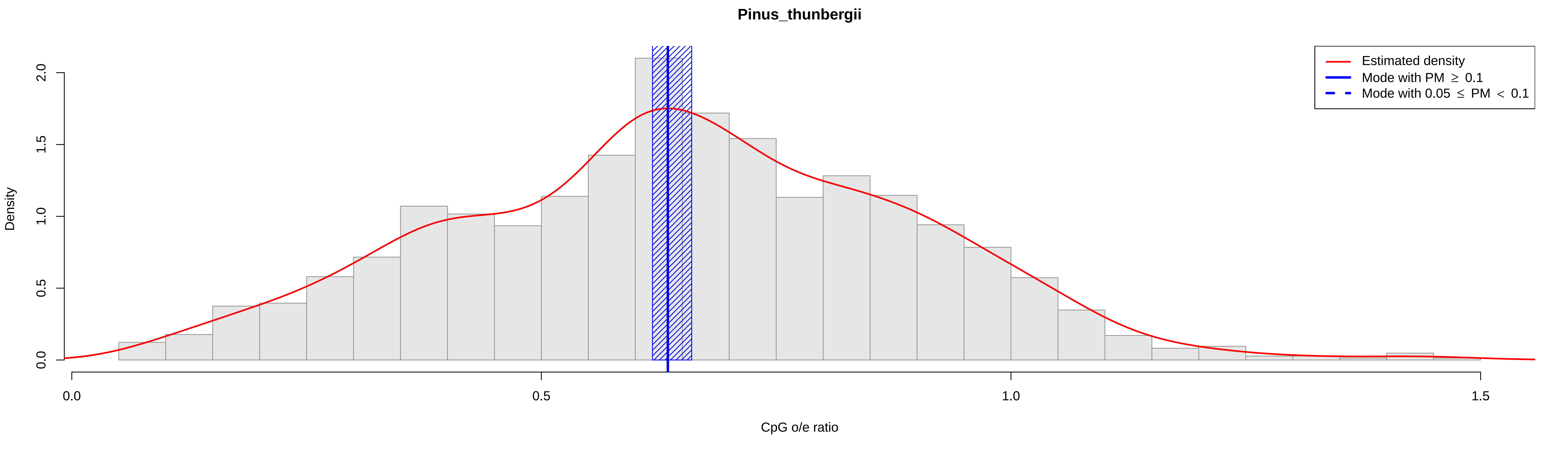
Pinus_thunbergii
Estimated density
Mode with PM ≥ 0.1
Mode with 0.05 ≤ PM < 0.1
Density
0.0
0.5
1.0
1.5
2.0
0.0
0.5
1.0
1.5
CpG o/e ratio

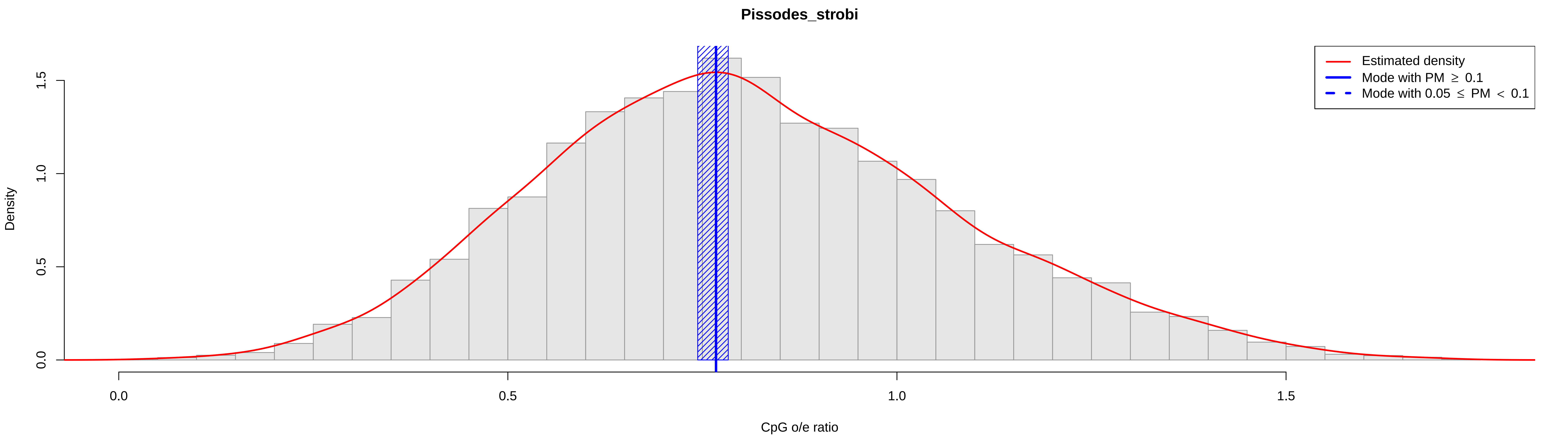

Pissodes_strobi
Estimated density
Mode with PM ≥ 0.1
Mode with 0.05 ≤ PM < 0.1
Density
0.0
0.5
1.0
1.5
0.0
0.5
1.0
1.5
CpG o/e ratio

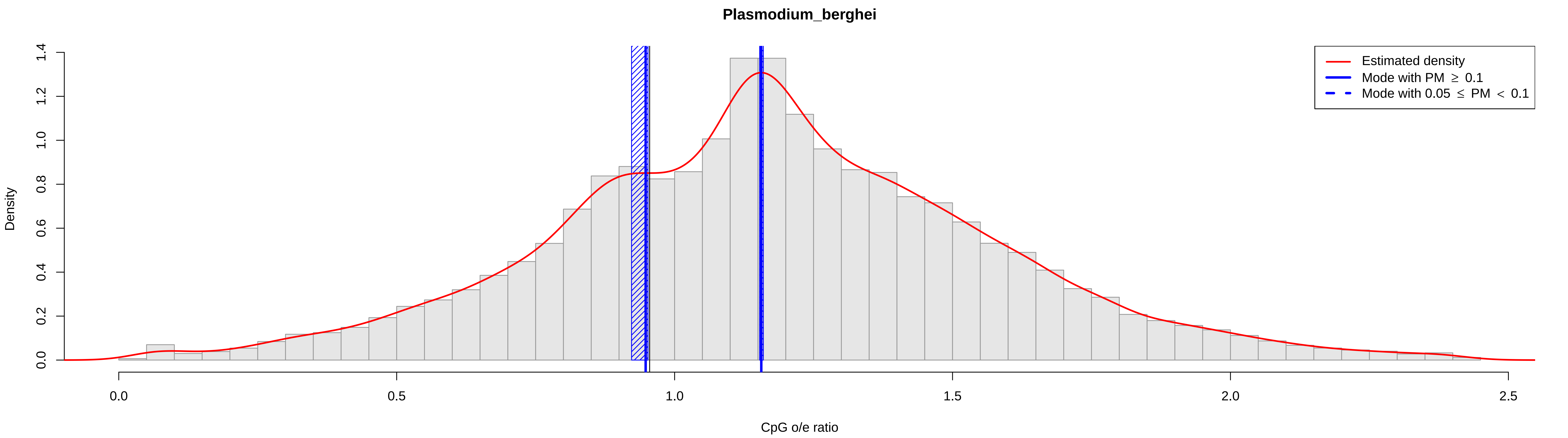
Plasmodium_berghei
Density
CpG o/e ratio
0.0
0.2
0.4
0.6
0.8
1.0
1.2
1.4
0.0
0.5
1.0
1.5
2.0
2.5
Estimated density
Mode with PM ≥ 0.1
Mode with 0.05 ≤ PM < 0.1

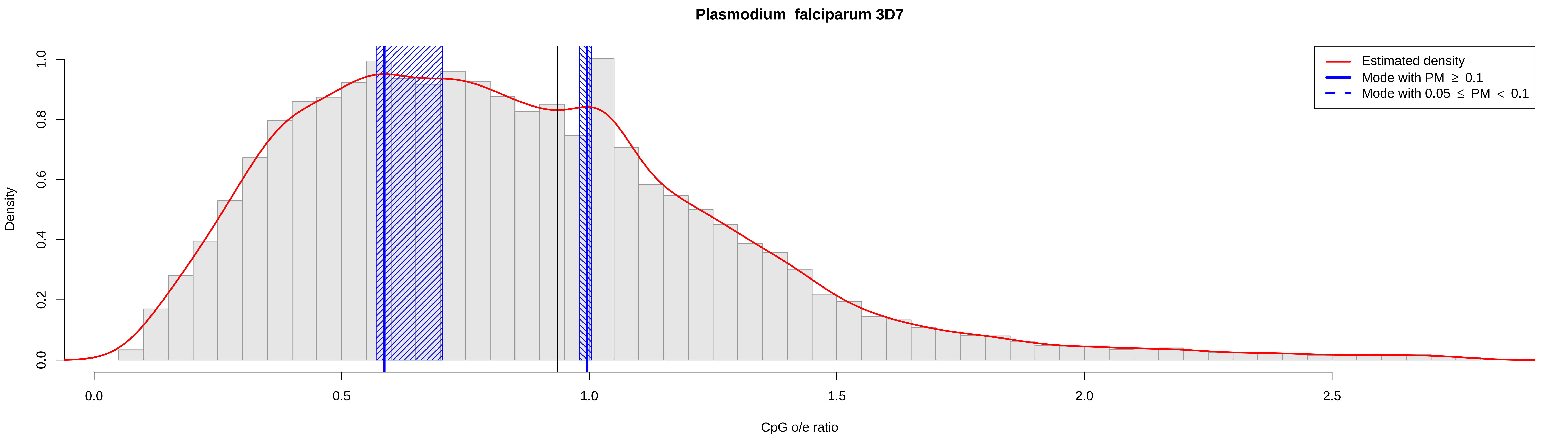

Plasmodium_falciparum 3D7
Density
CpG o/e ratio
0.0
0.2
0.4
0.6
0.8
1.0
0.0
0.5
1.0
1.5
2.0
2.5
Estimated density
Mode with PM ≥ 0.1
Mode with 0.05 ≤ PM < 0.1

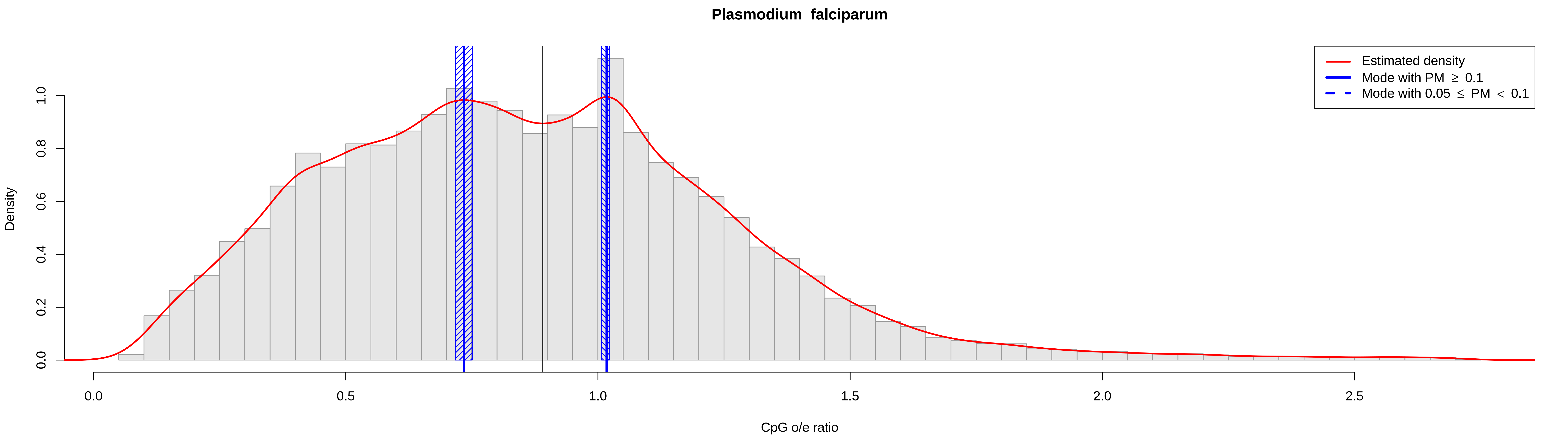
Plasmodium_falciparum
Estimated density
Mode with PM ≥ 0.1
Mode with 0.05 ≤ PM < 0.1
Density
0.0
0.2
0.4
0.6
0.8
1.0
0.0
0.5
1.0
1.5
2.0
2.5
CpG o/e ratio

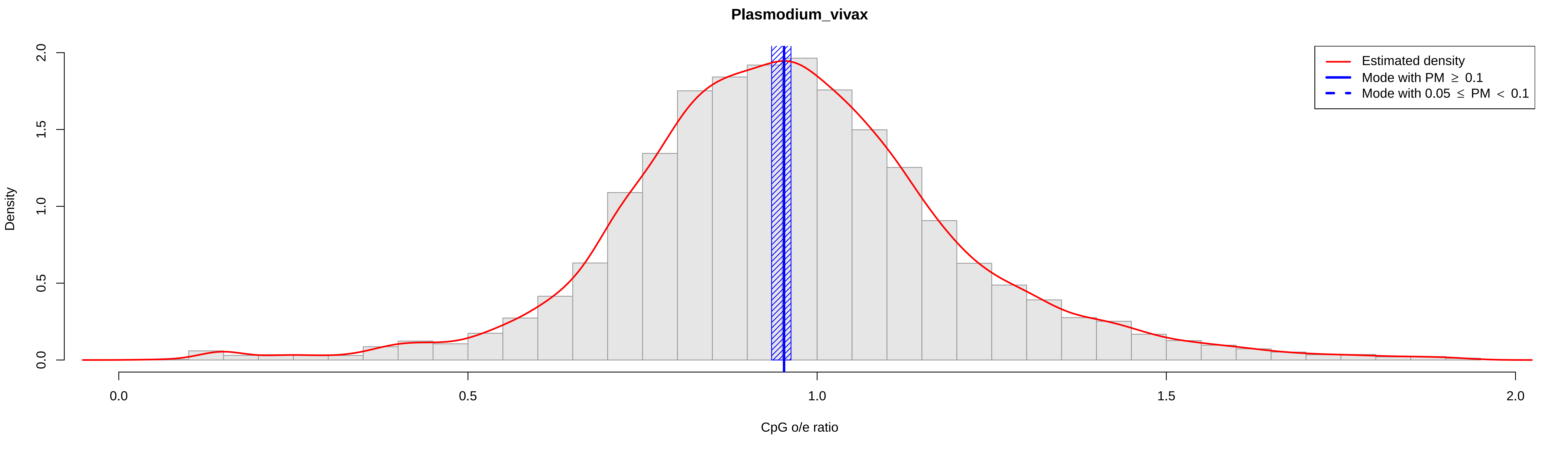
Plasmodium_vivax
Estimated density
Mode with PM ≥ 0.1
Mode with 0.05 ≤ PM < 0.1
Density
0.0
0.5
1.0
1.5
2.0
CpG o/e ratio

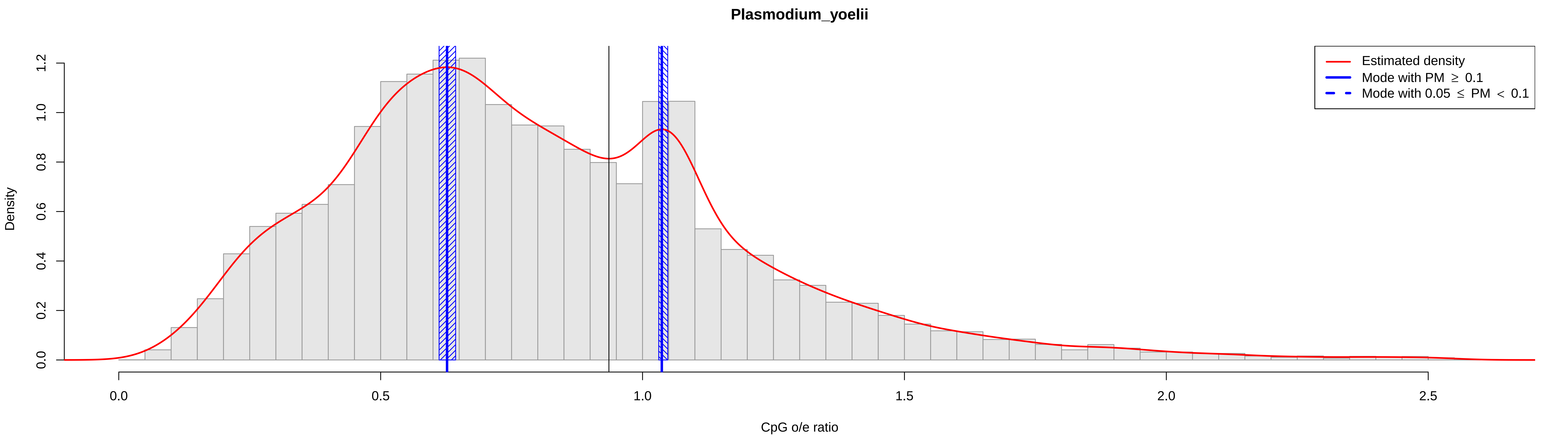
Plasmodium_yoelii
Density
CpG o/e ratio
0.0
0.2
0.4
0.6
0.8
1.0
1.2
0.0
0.5
1.0
1.5
2.0
2.5
Estimated density
Mode with PM ≥ 0.1
Mode with 0.05 ≤ PM < 0.1

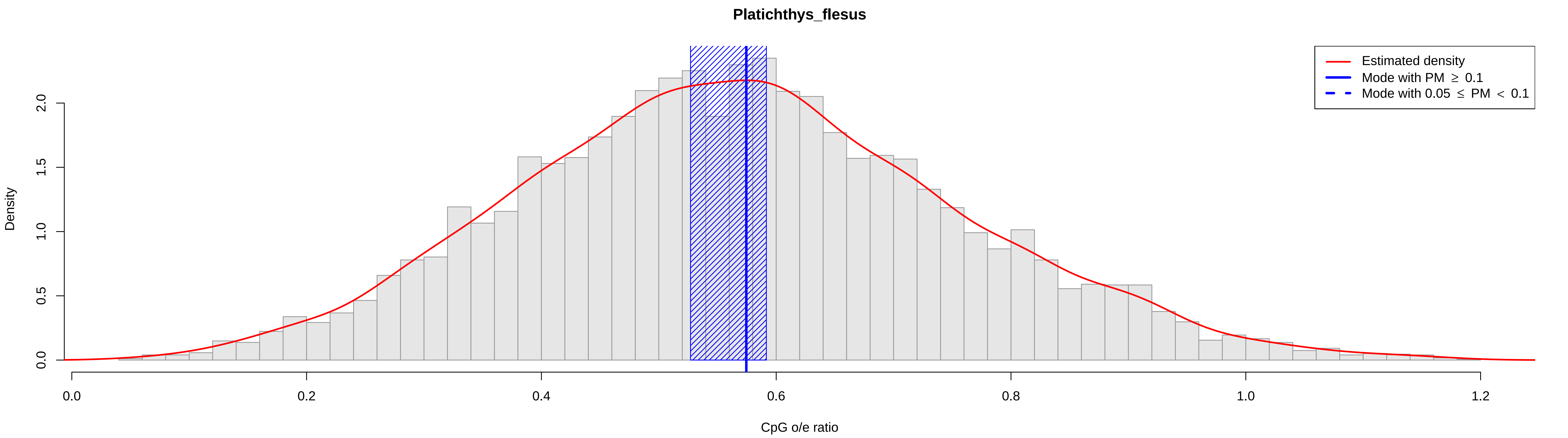

Platichthys_flesus
Estimated density
Mode with PM ≥ 0.1
Mode with 0.05 ≤ PM < 0.1
Density
0.0
0.5
1.0
1.5
2.0
0.0
0.2
0.4
0.6
0.8
1.0
1.2
CpG o/e ratio

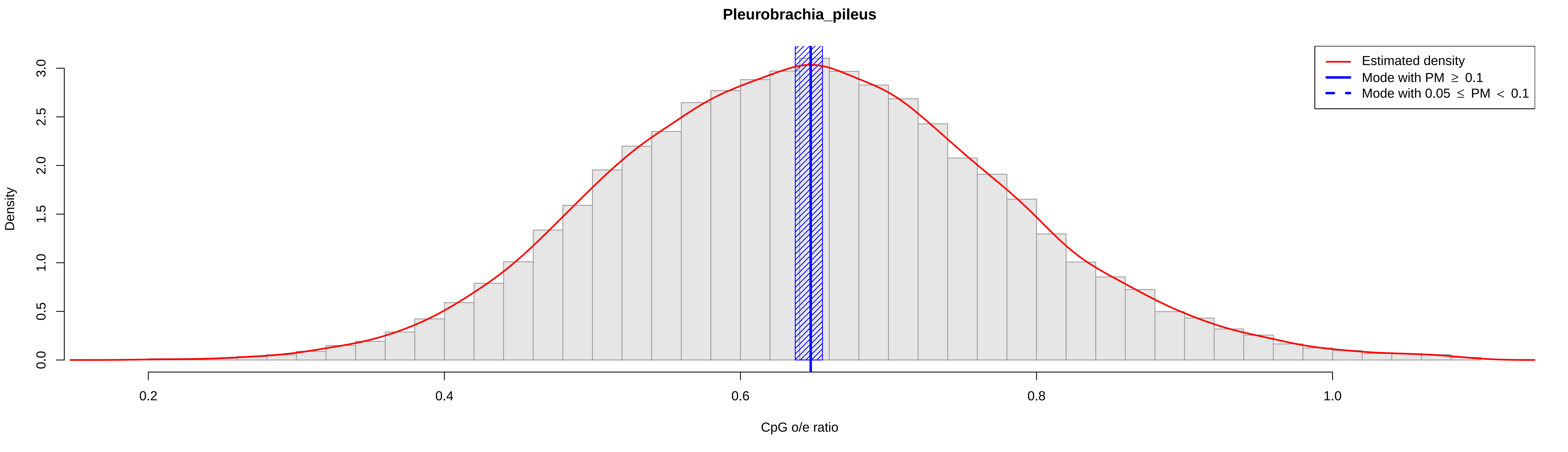

Pleurobrachia_pileus
Estimated density
Mode with PM ≥ 0.1
Mode with 0.05 ≤ PM < 0.1
Density
0.0
0.5
1.0
1.5
2.0
2.5
3.0
0.2
0.4
0.6
0.8
1.0
CpG o/e ratio

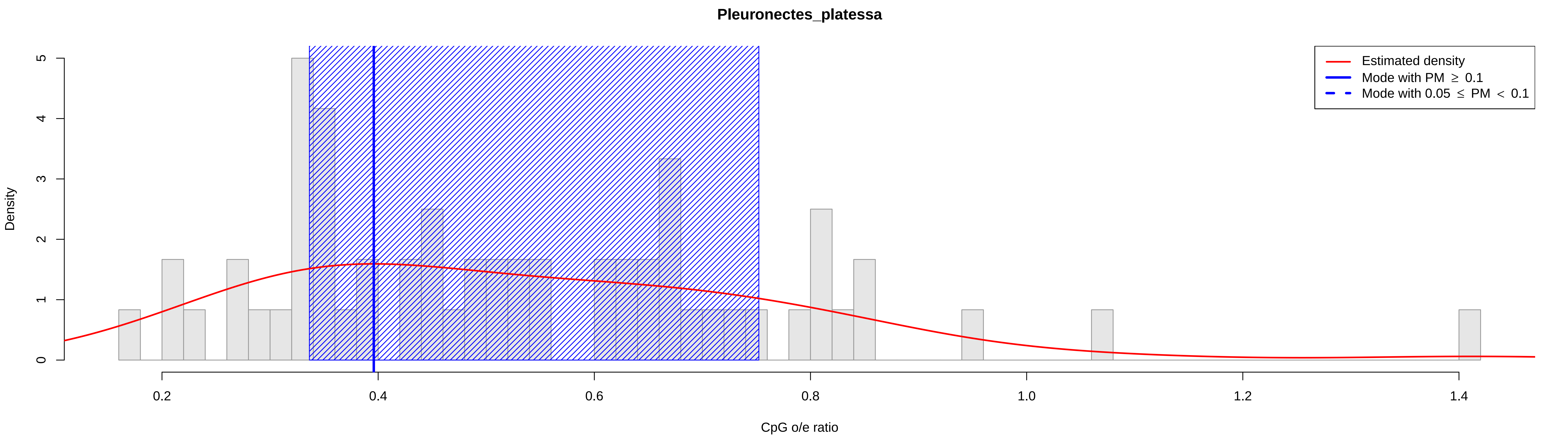

Pleuronectes_platessa
Estimated density
Mode with PM ≥ 0.1
Mode with 0.05 ≤ PM < 0.1
Density
0
1
2
3
4
5
0.2
0.4
0.6
0.8
1.0
1.2
1.4
CpG o/e ratio

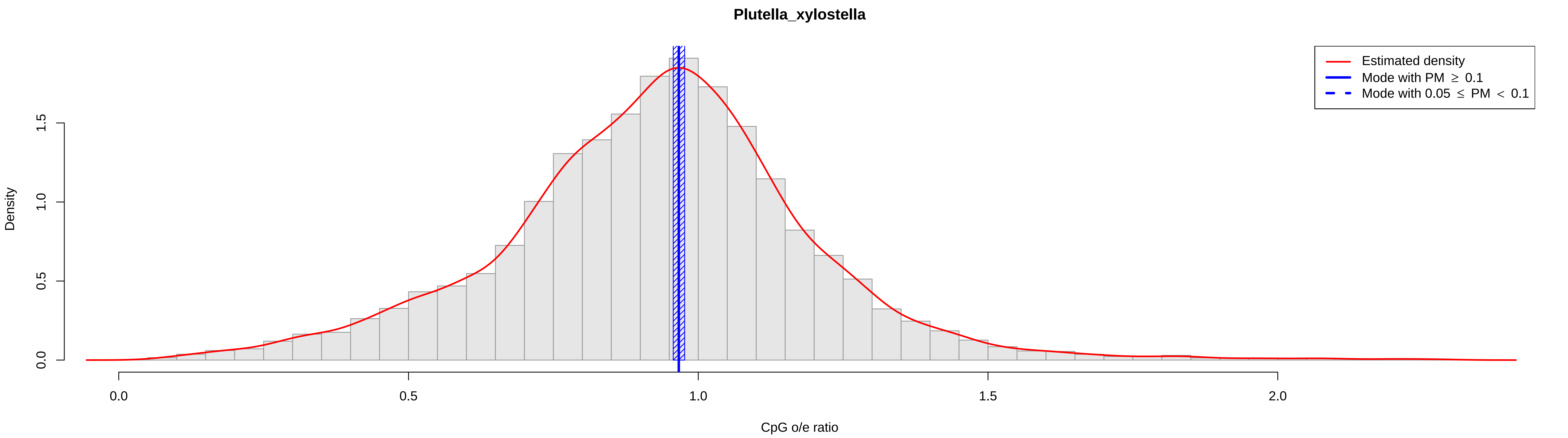

Plutella_xylostella
Estimated density
Mode with PM ≥ 0.1
Mode with 0.05 ≤ PM < 0.1
Density
0.0
0.5
1.0
1.5
0.0
0.5
1.0
1.5
2.0
CpG o/e ratio

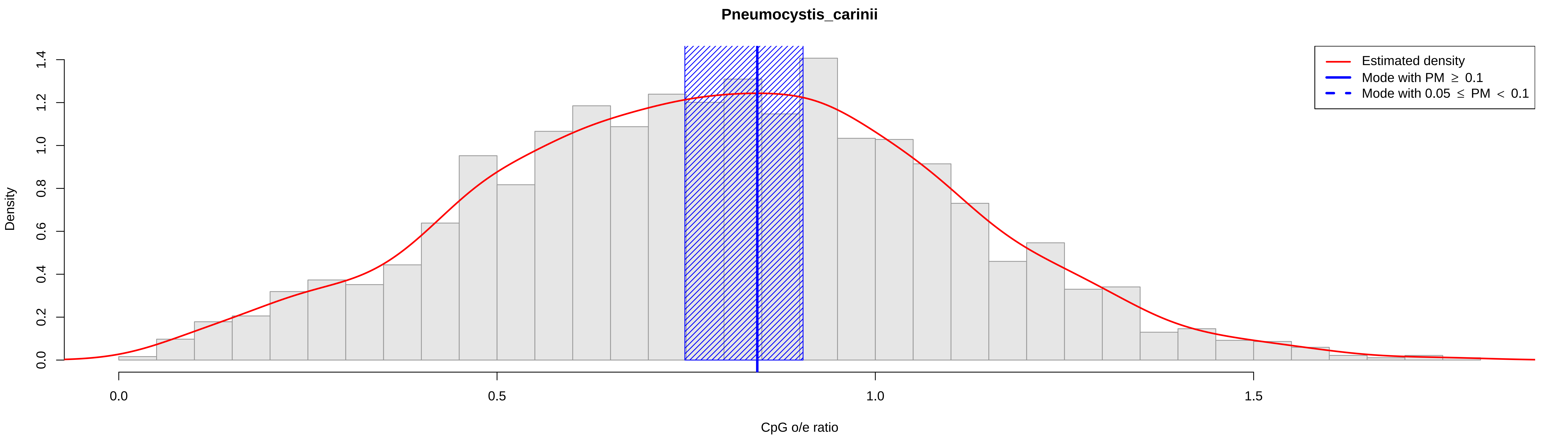
Pneumocystis_carinii
Estimated density
Mode with PM ≥ 0.1
Mode with 0.05 ≤ PM < 0.1
Density
0.0
0.2
0.4
0.6
0.8
1.0
1.2
1.4
0.0
0.5
1.0
1.5
CpG o/e ratio

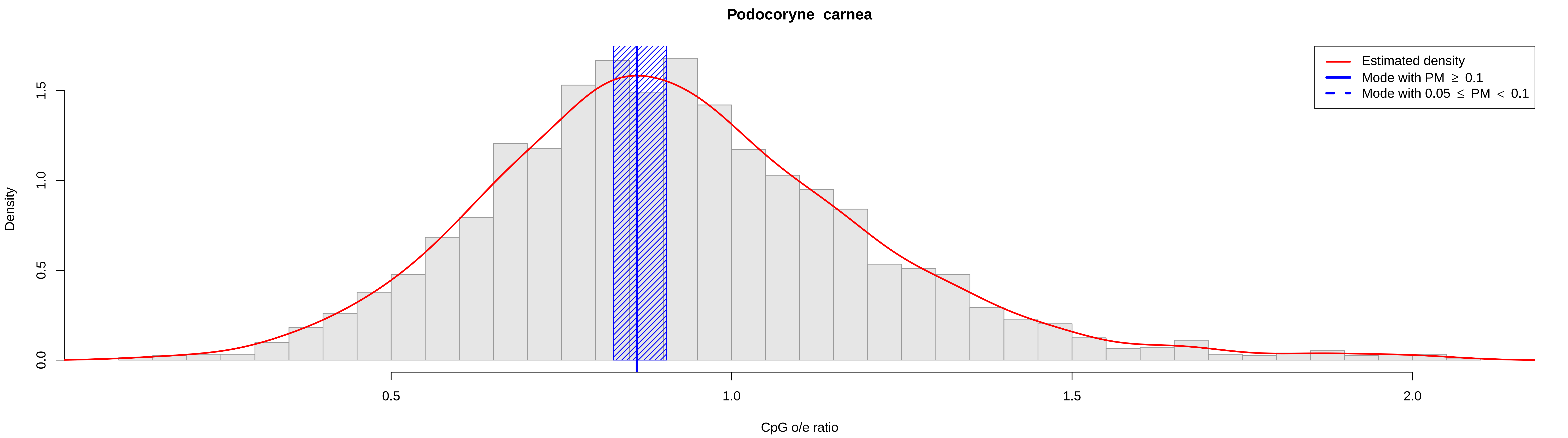

Podocoryne_carnea
Estimated density
Mode with PM ≥ 0.1
Mode with 0.05 ≤ PM < 0.1
Density
0.0
0.5
1.0
1.5
0.5
1.0
1.5
2.0
CpG o/e ratio

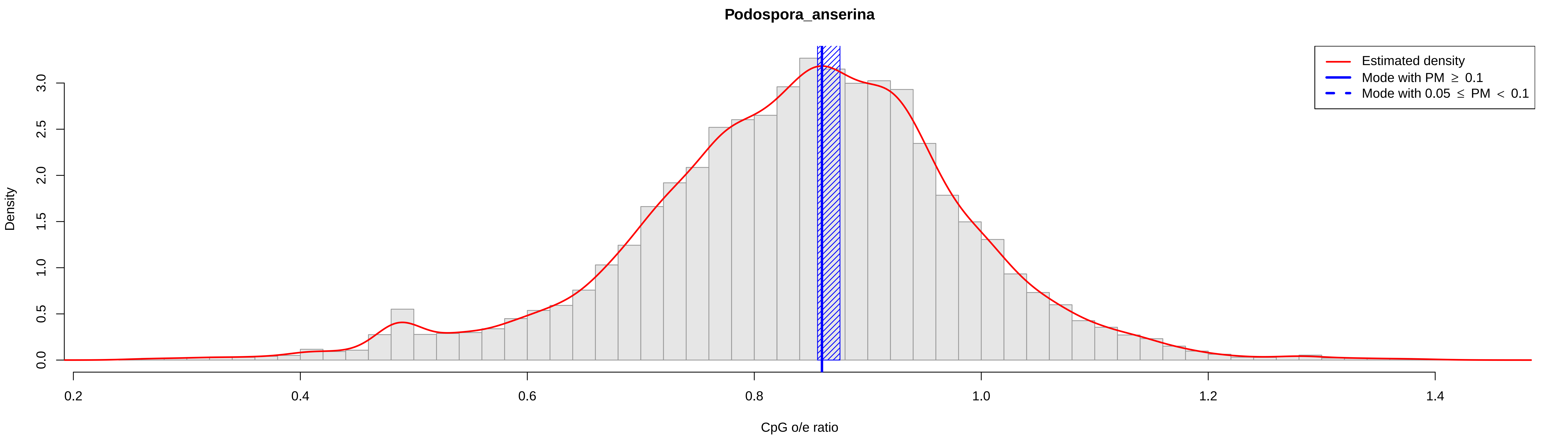

Podospora_anserina
Estimated density
Mode with PM ≥ 0.1
Mode with 0.05 ≤ PM < 0.1
Density
0.0
0.5
1.0
1.5
2.0
2.5
3.0
0.2
0.4
0.6
0.8
1.0
1.2
1.4
CpG o/e ratio

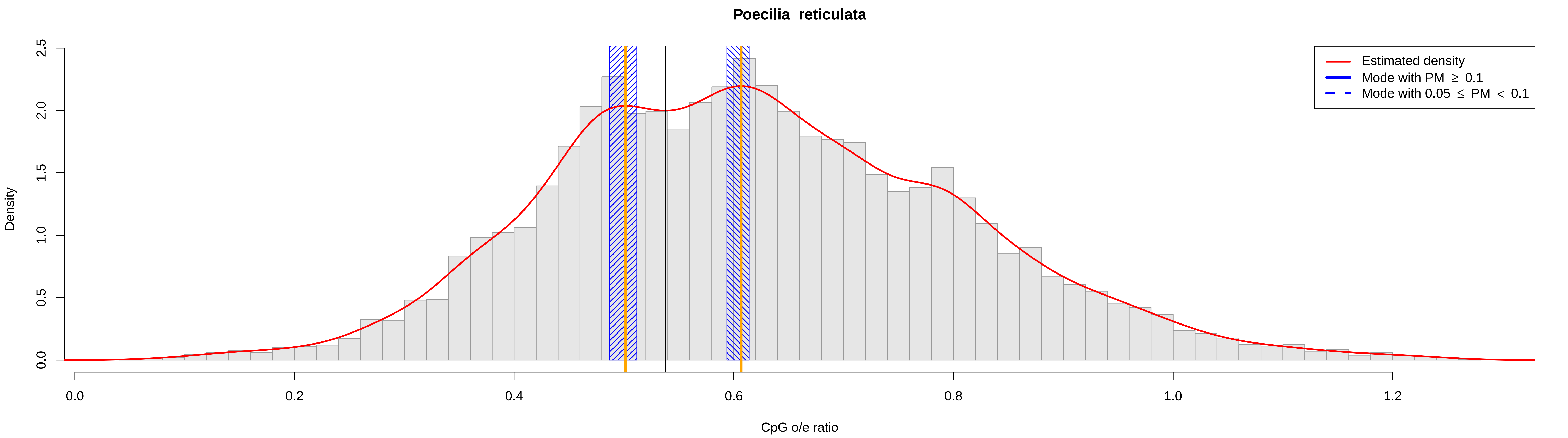

Poecilia_reticulata
Estimated density
Mode with PM ≥ 0.1
Mode with 0.05 ≤ PM < 0.1
Density
0.0
0.5
1.0
1.5
2.0
2.5
0.0
0.2
0.4
0.6
0.8
1.0
1.2
CpG o/e ratio

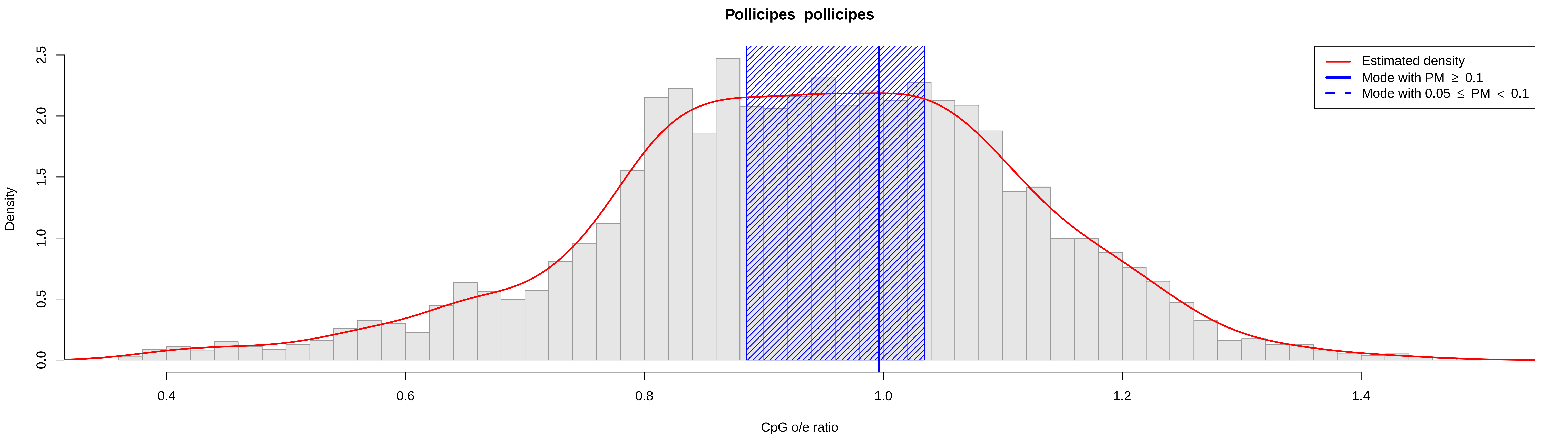

Pollicipes_pollicipes
Density
0.0
0.5
1.0
1.5
2.0
2.5
0.4
0.6
0.8
1.0
1.2
1.4
CpG o/e ratio
Estimated density
Mode with PM ≥ 0.1
Mode with 0.05 ≤ PM < 0.1

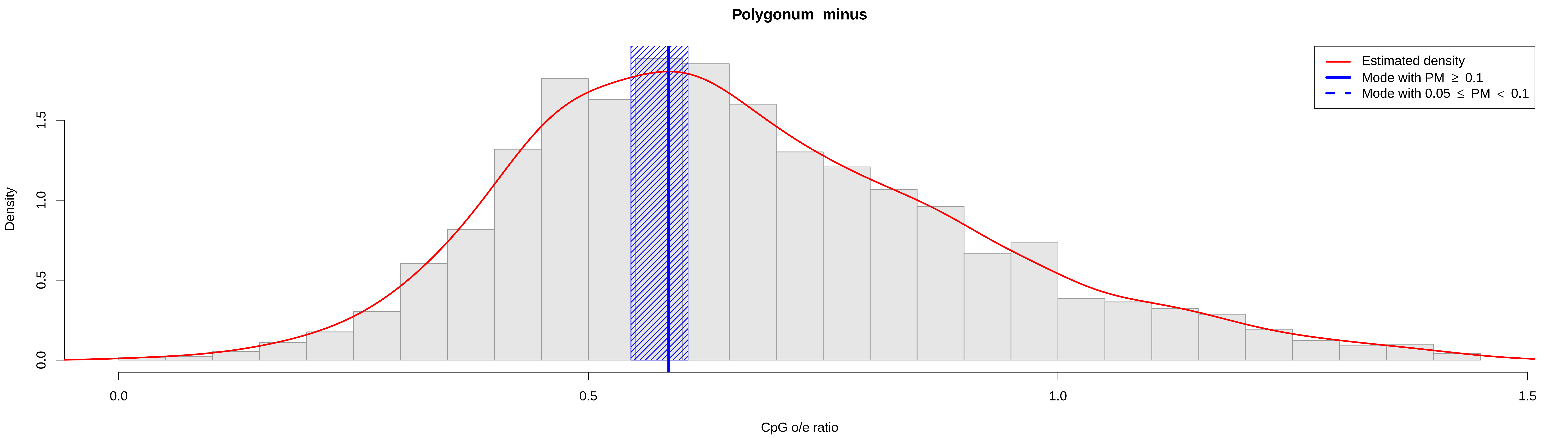
Polygonum_minus
Estimated density
Mode with PM ≥ 0.1
Mode with 0.05 ≤ PM < 0.1
Density
0.0
0.5
1.0
1.5
0.0
0.5
1.0
1.5
CpG o/e ratio

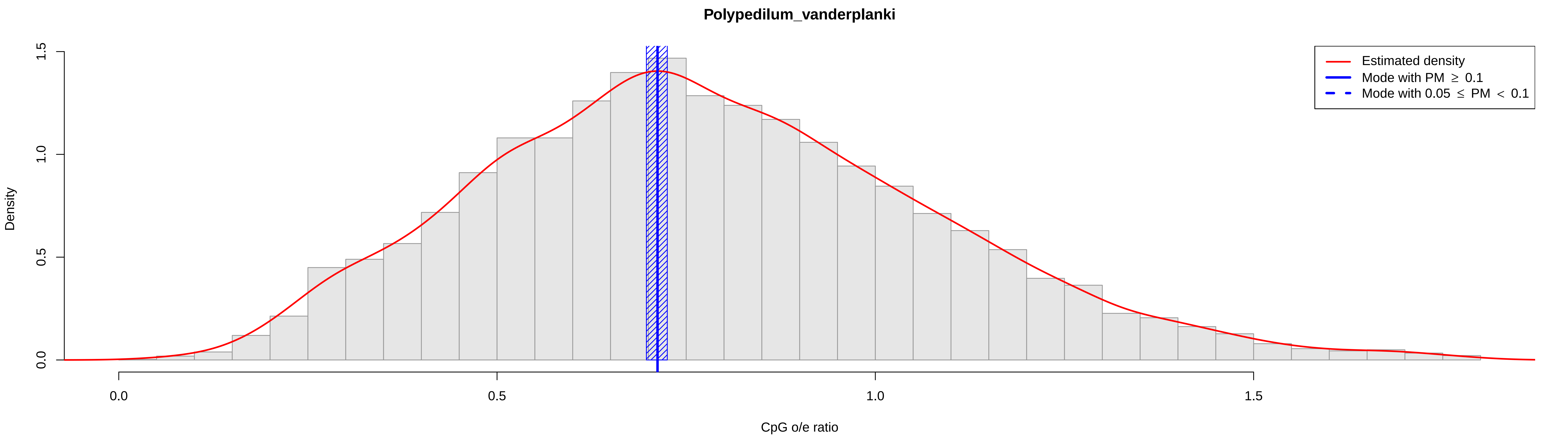
Polypedilum_vanderplanki
Estimated density
Mode with PM ≥ 0.1
Mode with 0.05 ≤ PM < 0.1
Density
CpG o/e ratio
0.0
0.5
1.0
1.5

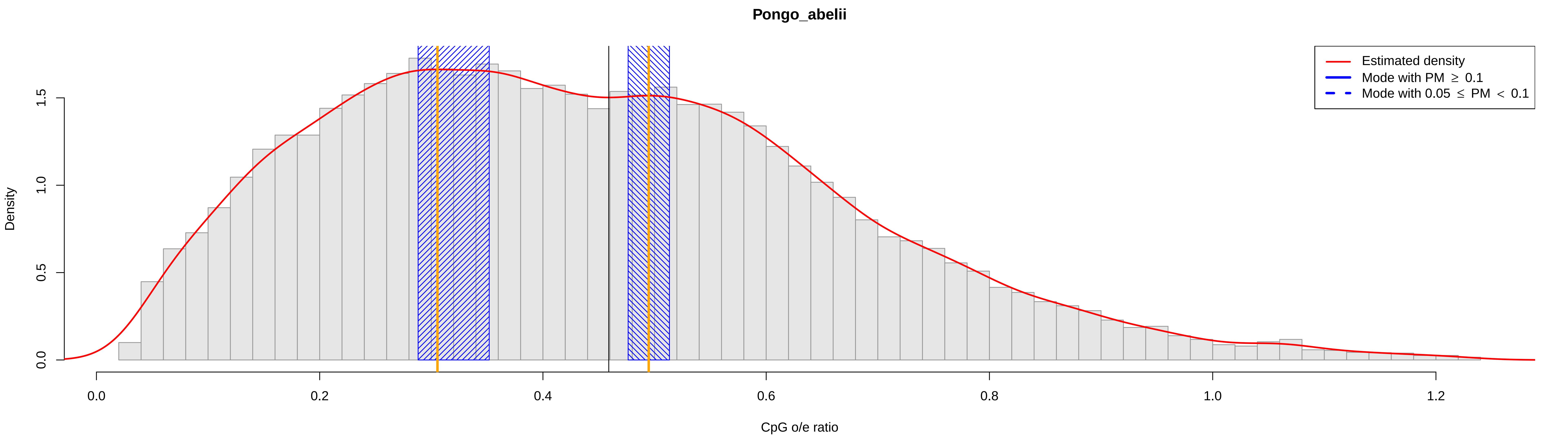

Pongo_abelii
Estimated density
Mode with PM ≥ 0.1
Mode with 0.05 ≤ PM < 0.1
Density
0.0
0.5
1.0
1.5
0.0
0.2
0.4
0.6
0.8
1.0
1.2
CpG o/e ratio

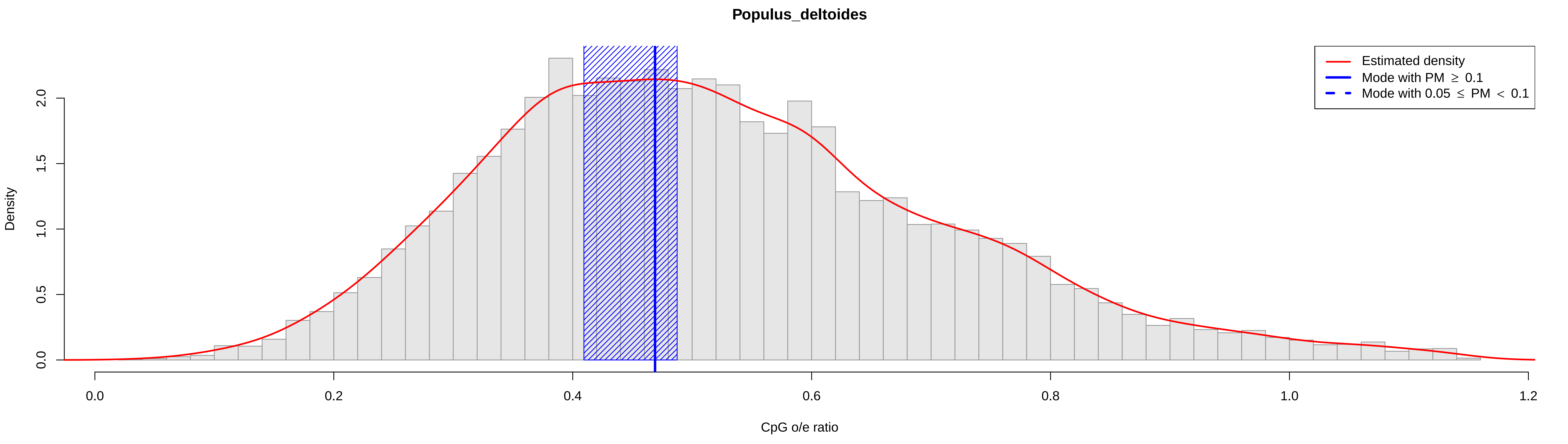

Populus_deltoides
Estimated density
Mode with PM ≥ 0.1
Mode with 0.05 ≤ PM < 0.1
Density
0.0
0.5
1.0
1.5
2.0
0.0
0.2
0.4
0.6
0.8
1.0
1.2
CpG o/e ratio

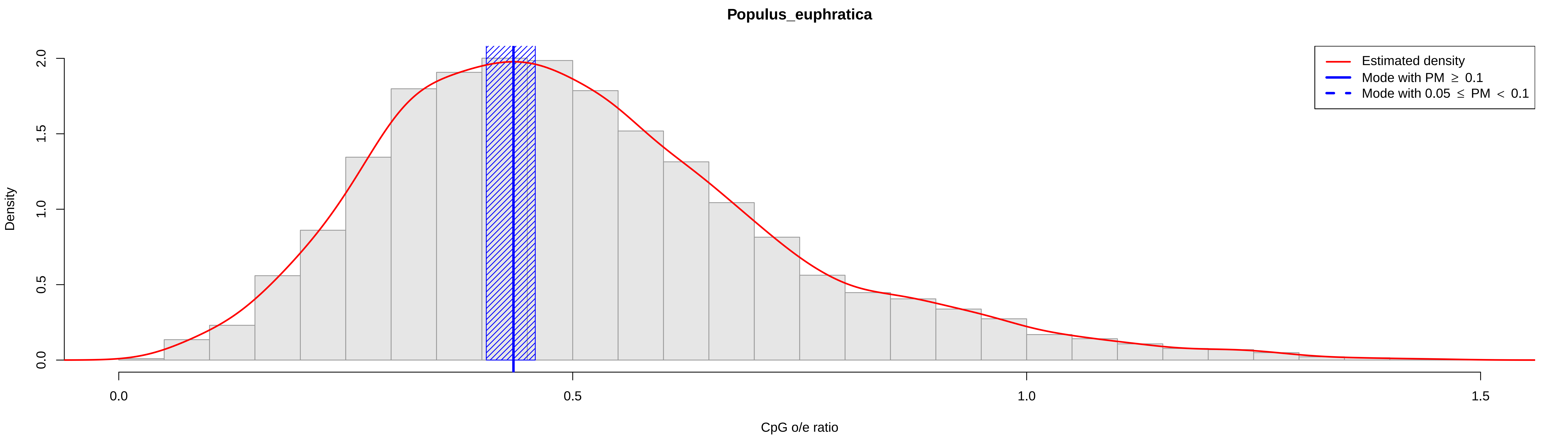

Populus_euphratica
Estimated density
Mode with PM ≥ 0.1
Mode with 0.05 ≤ PM < 0.1
Density
0.0
0.5
1.0
1.5
2.0
0.0
0.5
1.0
1.5
CpG o/e ratio

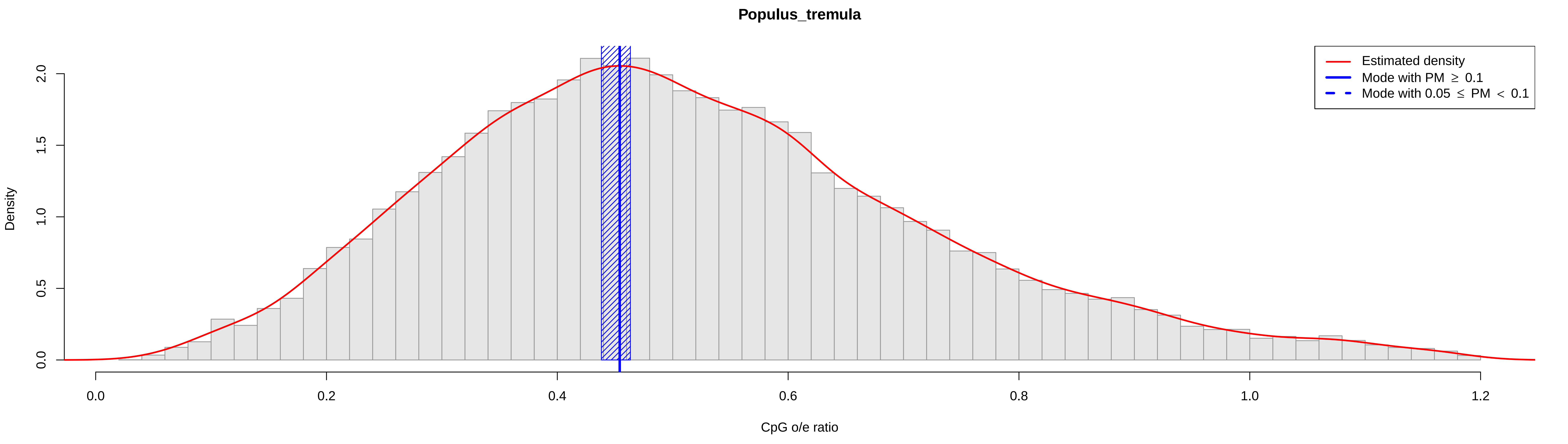
Populus_tremula
Estimated density
Mode with PM ≥ 0.1
Mode with 0.05 ≤ PM < 0.1
Density
0.0
0.5
1.0
1.5
2.0
0.0
0.2
0.4
0.6
0.8
1.0
1.2
CpG o/e ratio

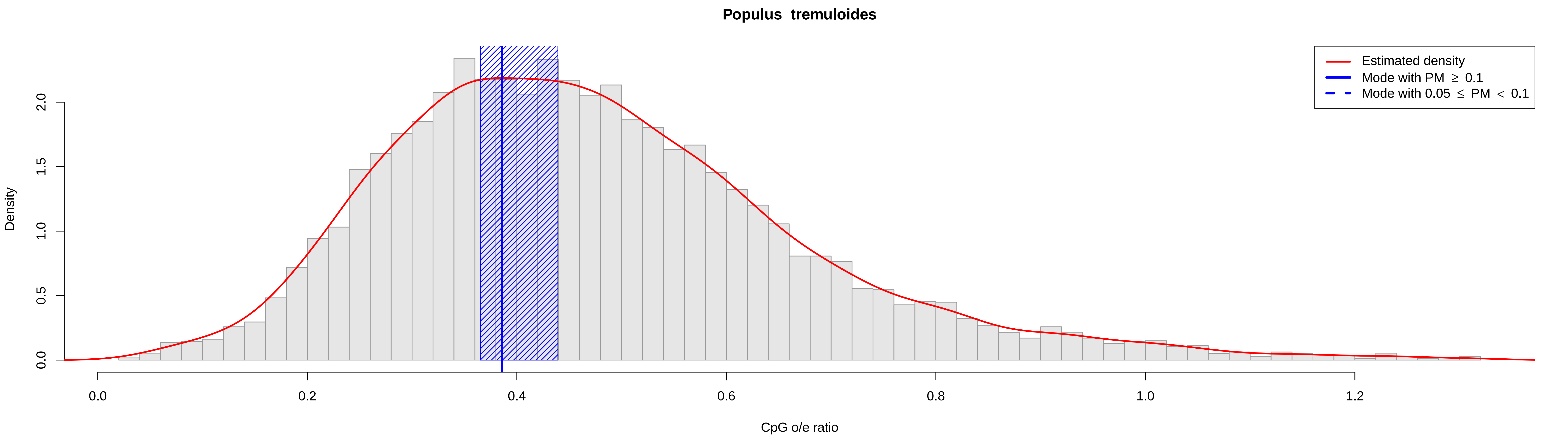
Populus_tremuloides
Estimated density
Mode with PM ≥ 0.1
Mode with 0.05 ≤ PM < 0.1
Density
0.0
0.5
1.0
1.5
2.0
0.0
0.2
0.4
0.6
0.8
1.0
1.2
CpG o/e ratio

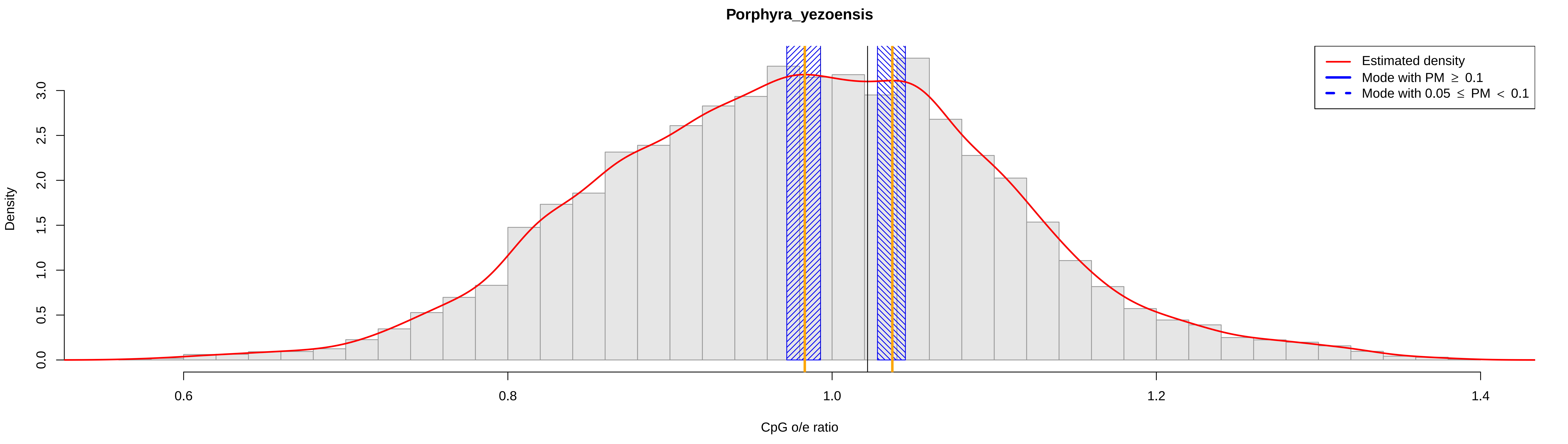

Porphyra_yezoensis
Estimated density
Mode with PM ≥ 0.1
Mode with 0.05 ≤ PM < 0.1
Density
0.0
0.5
1.0
1.5
2.0
2.5
3.0
0.6
0.8
1.0
1.2
1.4
CpG o/e ratio

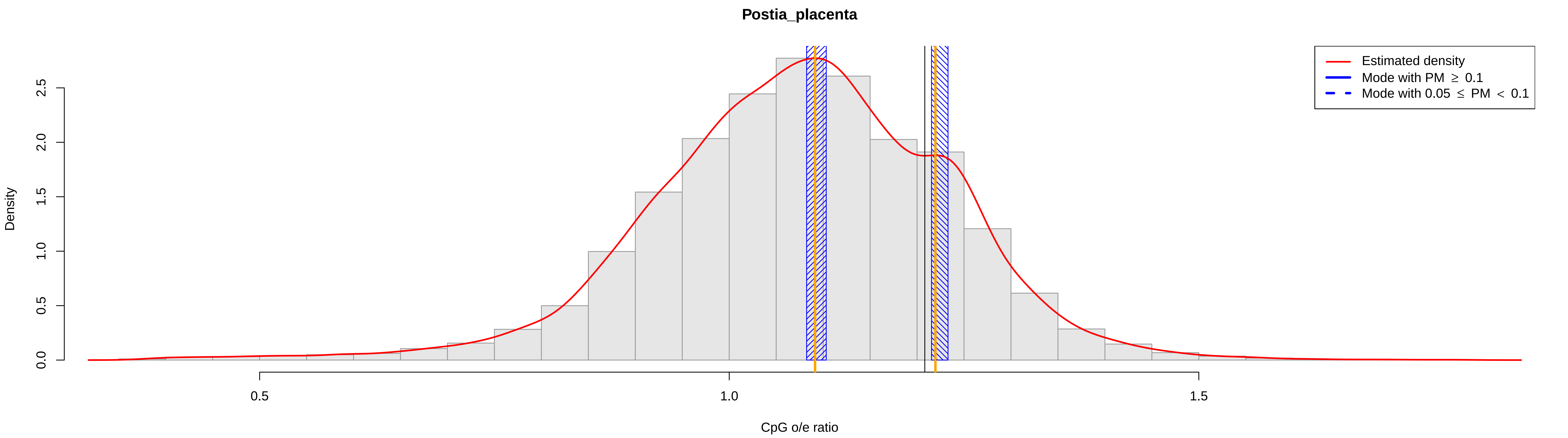

Postia_placenta
Estimated density
Mode with PM ≥ 0.1
Mode with 0.05 ≤ PM < 0.1
Density
0.0
0.5
1.0
1.5
2.0
2.5
0.5
1.0
1.5
CpG o/e ratio

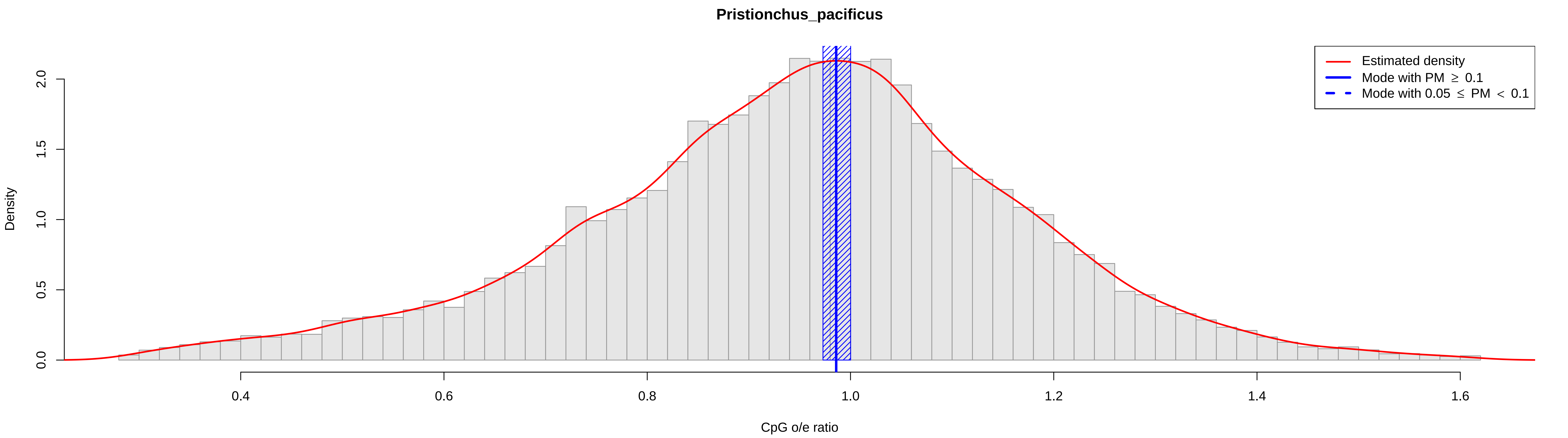

Pristionchus_pacificus
Estimated density
Mode with PM ≥ 0.1
Mode with 0.05 ≤ PM < 0.1
Density
0.0
0.5
1.0
1.5
2.0
0.4
0.6
0.8
1.0
1.2
1.4
1.6
CpG o/e ratio

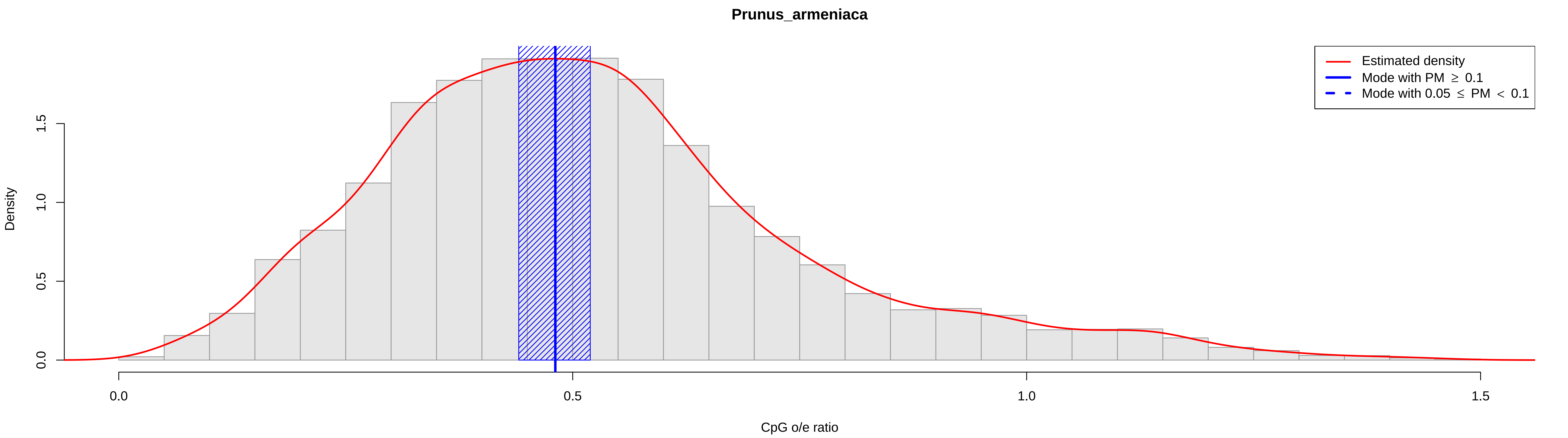

Prunus_armeniaca
Estimated density
Mode with PM ≥ 0.1
Mode with 0.05 ≤ PM < 0.1
Density
0.0
0.5
1.0
1.5
CpG o/e ratio

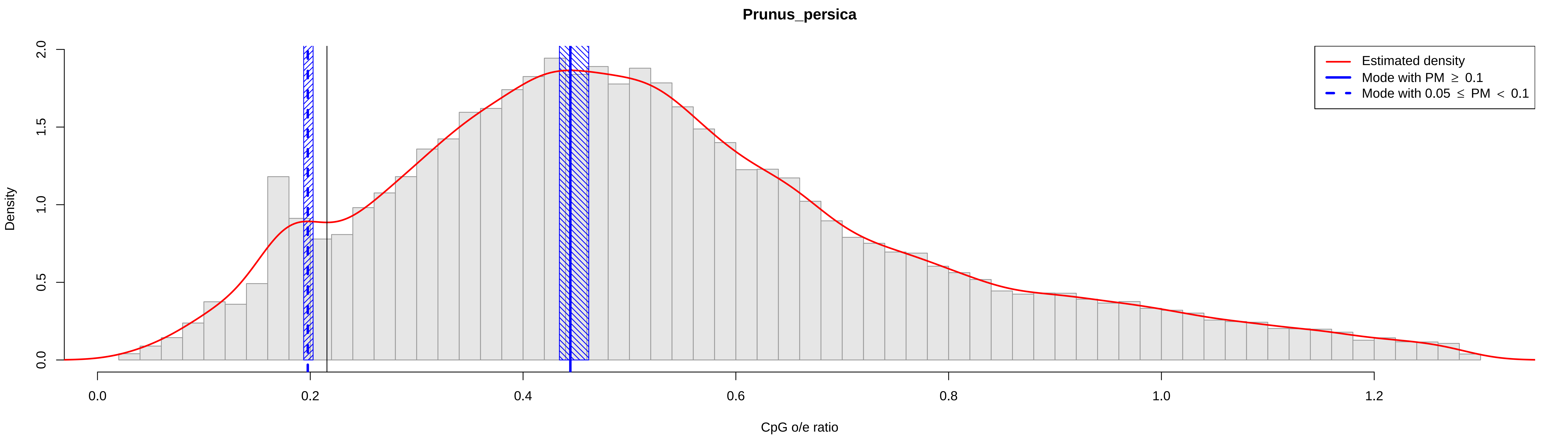
Prunus_persica
Estimated density
Mode with PM ≥ 0.1
Mode with 0.05 ≤ PM < 0.1
Density
CpG o/e ratio
0.0
0.5
1.0
1.5
2.0
0.0
0.2
0.4
0.6
0.8
1.0
1.2

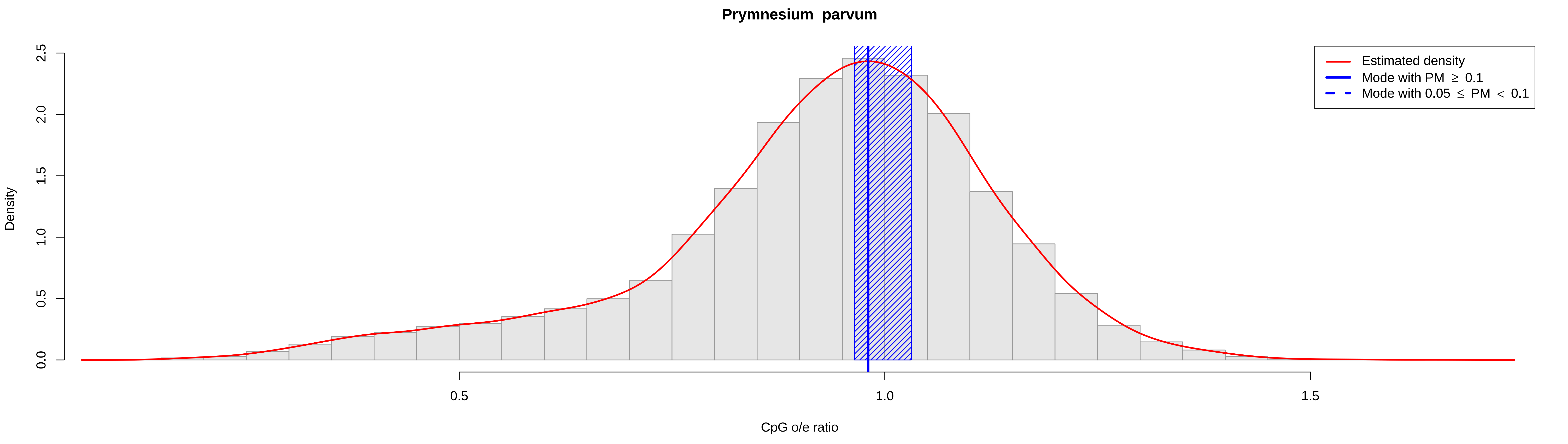
Prymnesium_parvum
Estimated density
Mode with PM ≥ 0.1
Mode with 0.05 ≤ PM < 0.1
Density
0.0
0.5
1.0
1.5
2.0
2.5
0.5
1.0
1.5
CpG o/e ratio

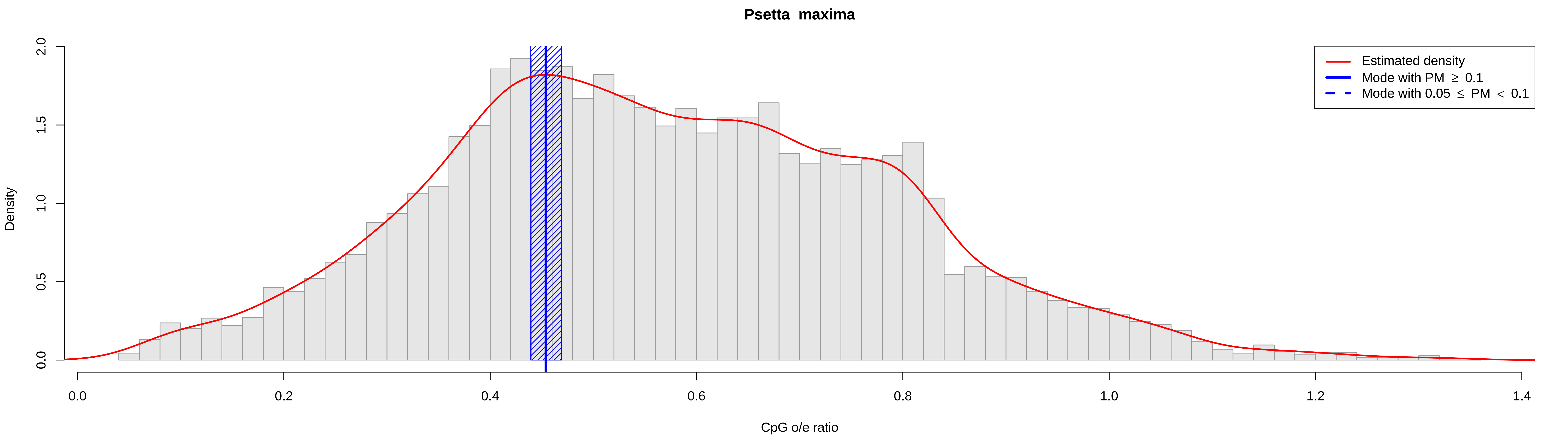

Psetta_maxima
Estimated density
Mode with PM ≥ 0.1
Mode with 0.05 ≤ PM < 0.1
Density
0.0
0.5
1.0
1.5
2.0
0.0
0.2
0.4
0.6
0.8
1.0
1.2
1.4
CpG o/e ratio

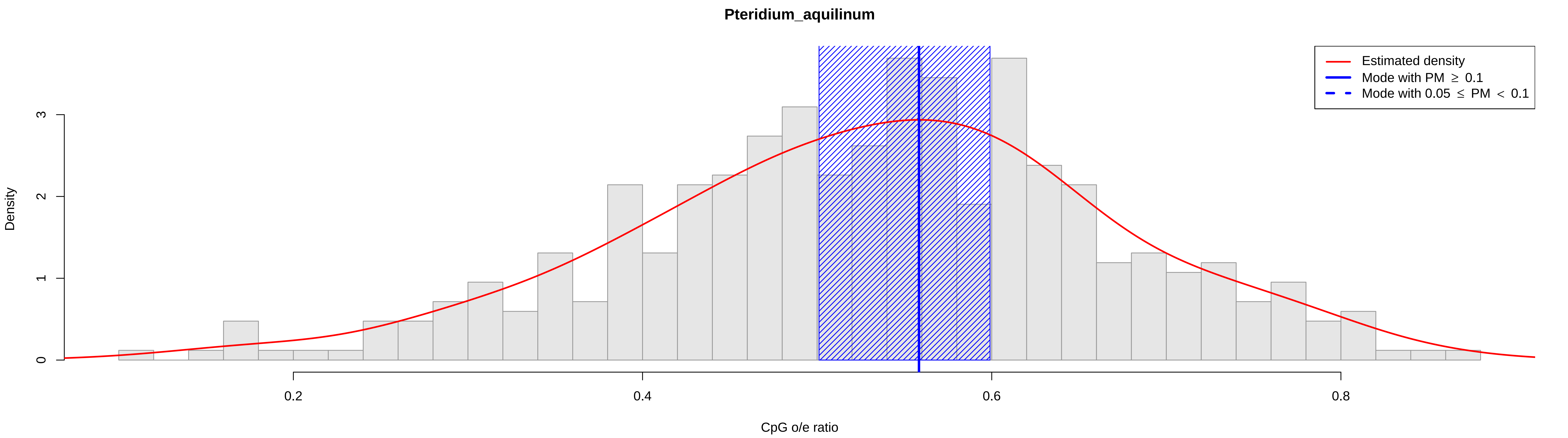
Pteridium_aquilinum
Estimated density
Mode with PM ≥ 0.1
Mode with 0.05 ≤ PM < 0.1
Density
0
1
2
3
0.2
0.4
0.6
0.8
CpG o/e ratio

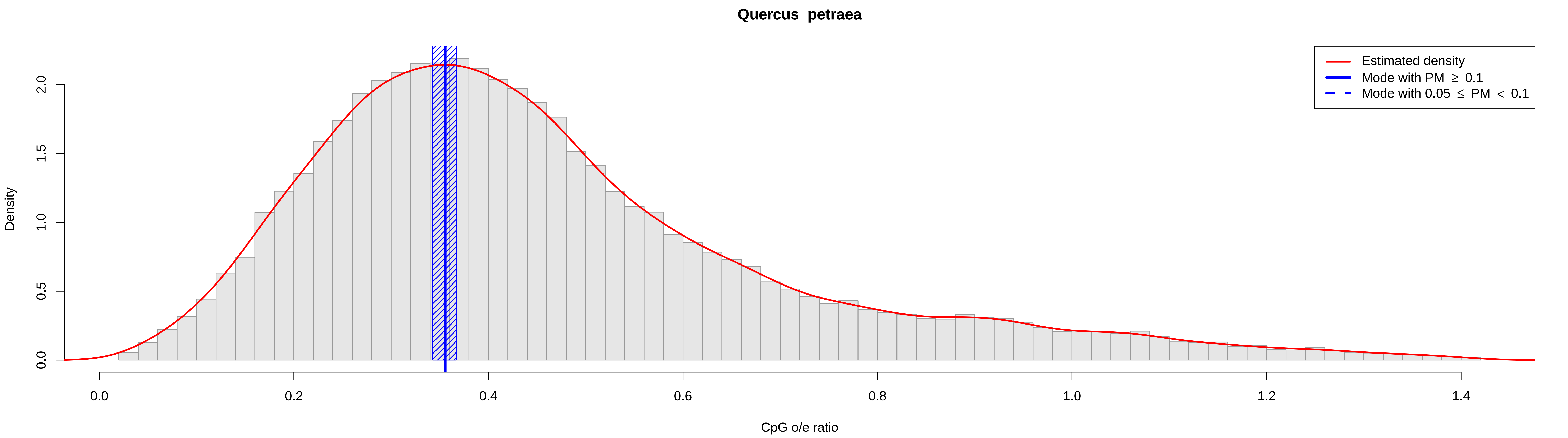

Quercus_petraea
Estimated density
Mode with PM ≥ 0.1
Mode with 0.05 ≤ PM < 0.1
Density
0.0
0.5
1.0
1.5
2.0
0.0
0.2
0.4
0.6
0.8
1.0
1.2
1.4
CpG o/e ratio

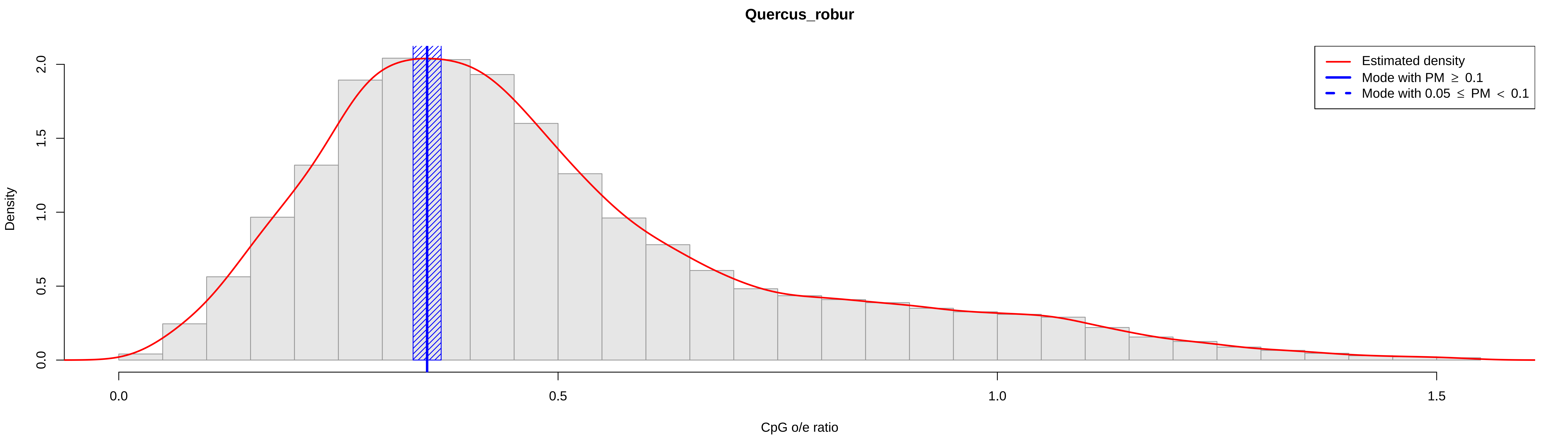
Quercus_robur
Estimated density
Mode with PM ≥ 0.1
Mode with 0.05 ≤ PM < 0.1
Density
0.0
0.5
1.0
1.5
2.0
0.0
0.5
1.0
1.5
CpG o/e ratio

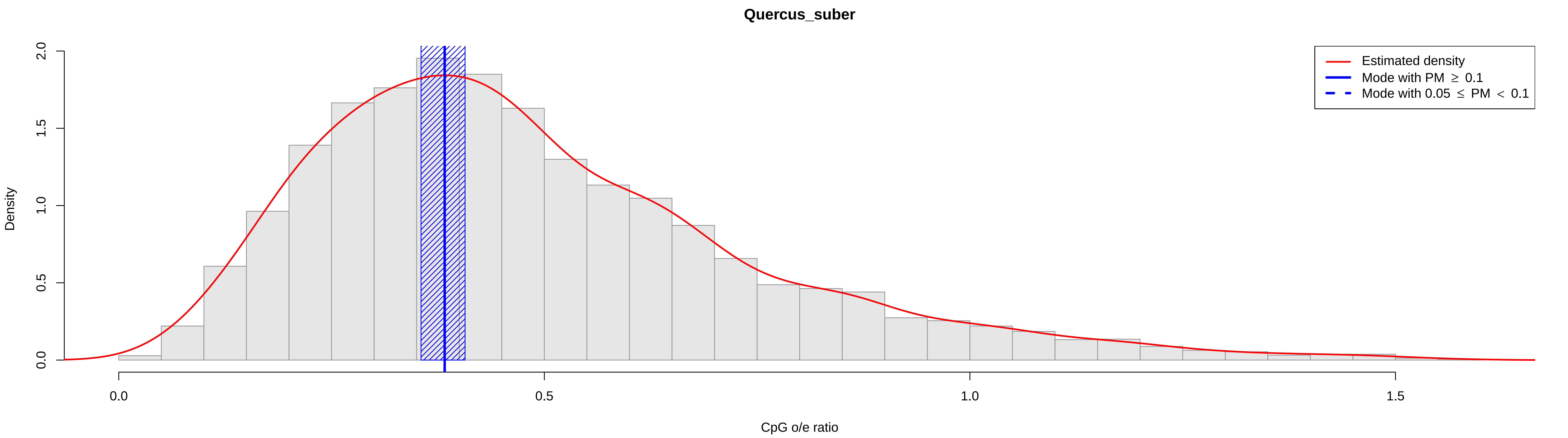
Quercus_suber
Density
CpG o/e ratio
0.0
0.5
1.0
1.5
2.0
0.0
0.5
1.0
1.5
Estimated density
Mode with PM ≥ 0.1
Mode with 0.05 ≤ PM < 0.1

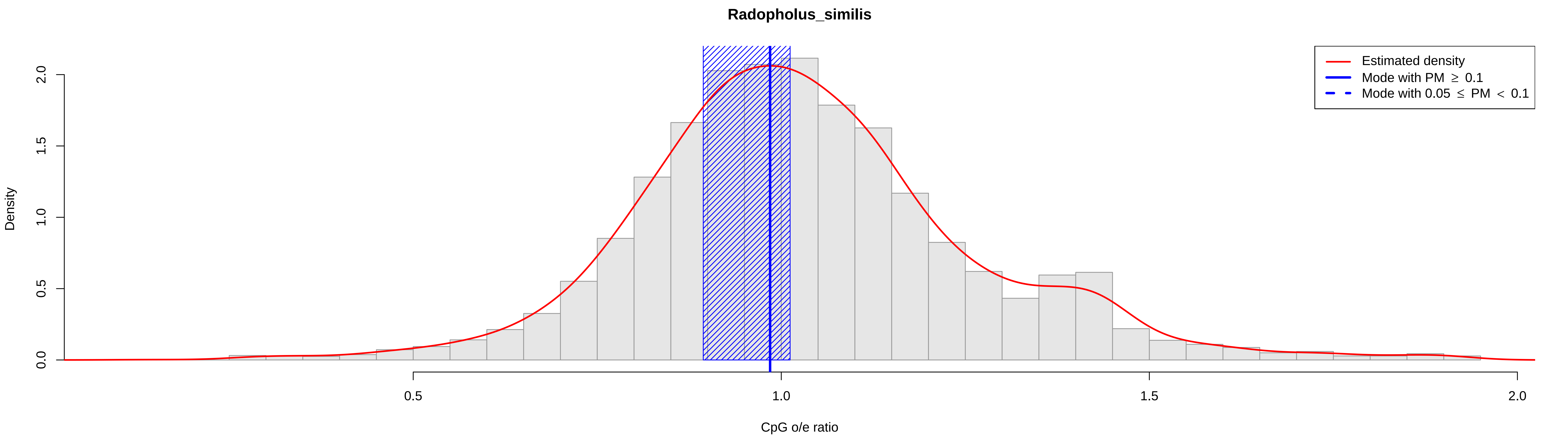

Radopholus_similis
Estimated density
Mode with PM ≥ 0.1
Mode with 0.05 ≤ PM < 0.1
Density
0.0
0.5
1.0
1.5
2.0
0.5
1.0
1.5
2.0
CpG o/e ratio

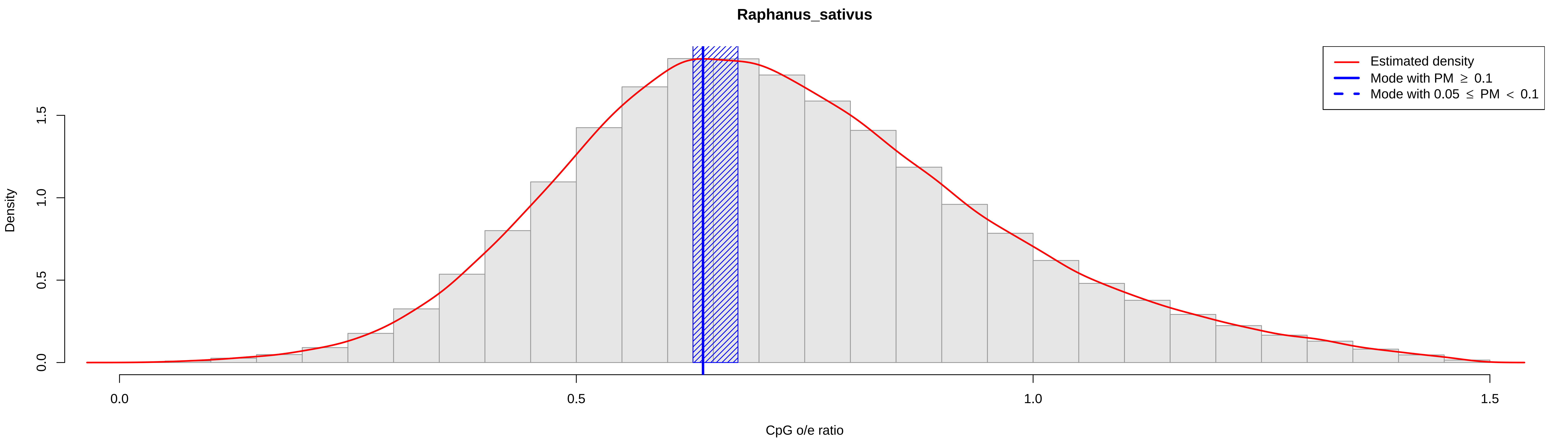

Raphanus_sativus
Estimated density
Mode with PM ≥ 0.1
Mode with 0.05 ≤ PM < 0.1
Density
0.0
0.5
1.0
1.5
CpG o/e ratio

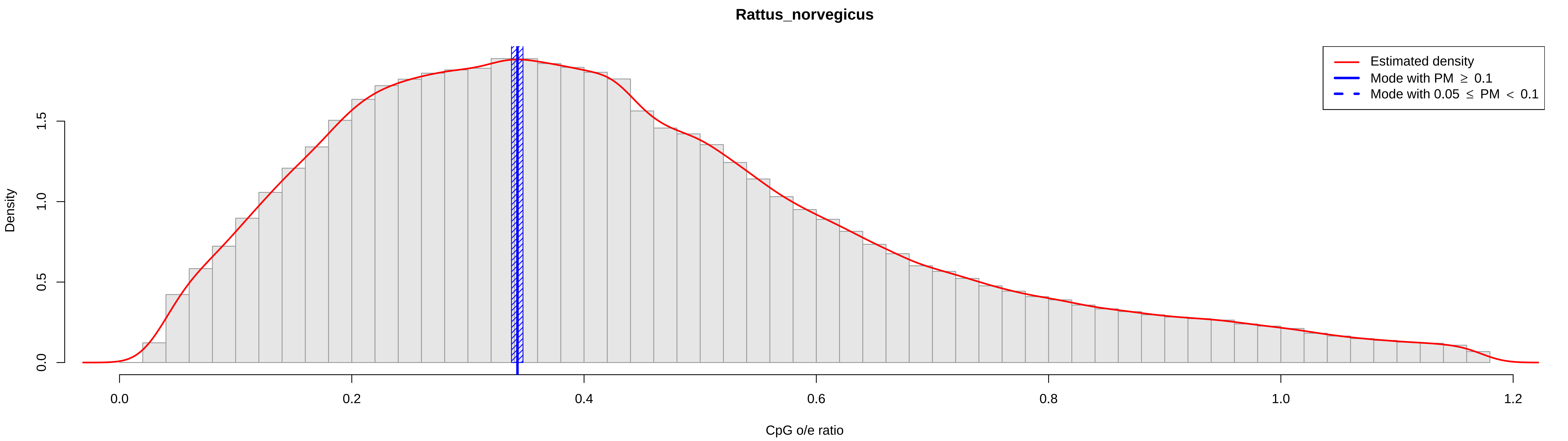

Rattus_norvegicus
Estimated density
Mode with PM ≥ 0.1
Mode with 0.05 ≤ PM < 0.1
Density
0.0
0.5
1.0
1.5
0.0
0.2
0.4
0.6
0.8
1.0
1.2
CpG o/e ratio

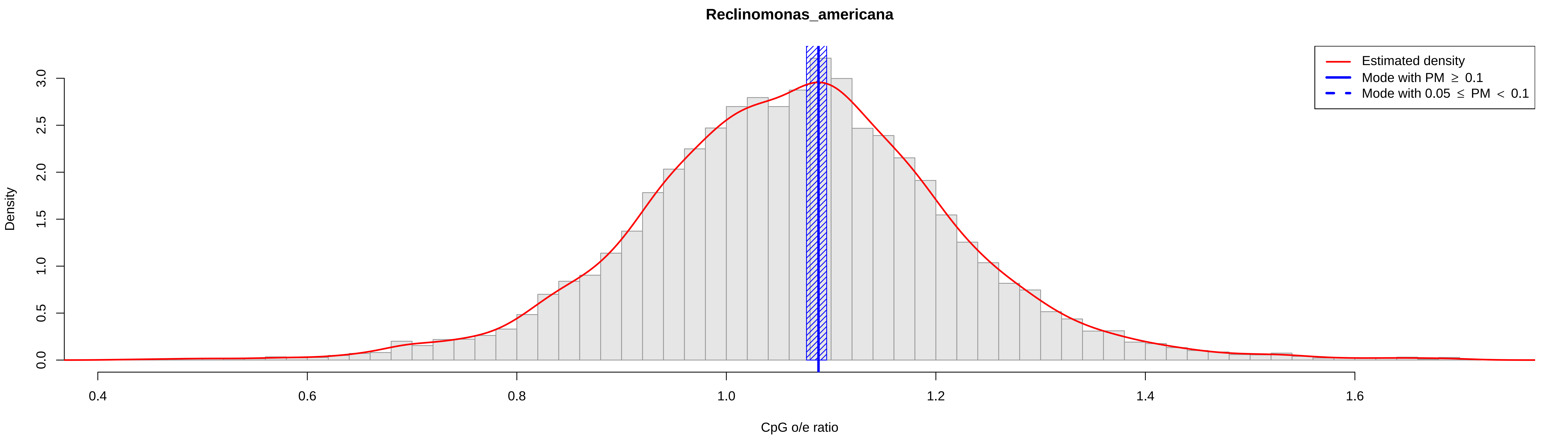

Reclinomonas_americana
Estimated density
Mode with PM ≥ 0.1
Mode with 0.05 ≤ PM < 0.1
Density
0.0
0.5
1.0
1.5
2.0
2.5
3.0
0.4
0.6
0.8
1.0
1.2
1.4
1.6
CpG o/e ratio

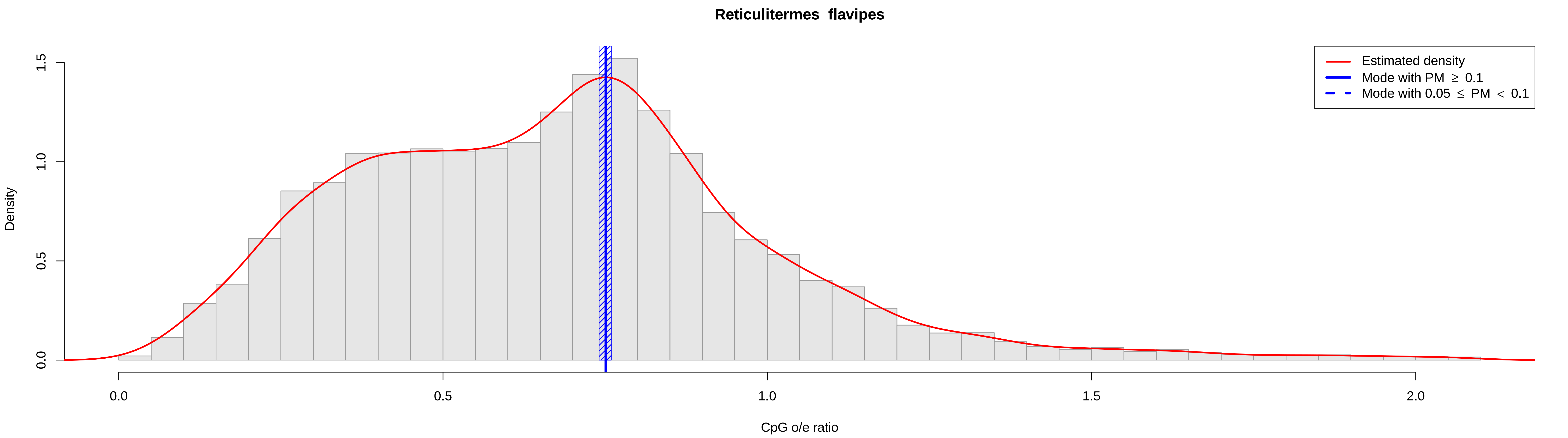
Reticulitermes_flavipes
Estimated density
Mode with PM ≥ 0.1
Mode with 0.05 ≤ PM < 0.1
Density
0.0
0.5
1.0
1.5
0.0
0.5
1.0
1.5
2.0
CpG o/e ratio

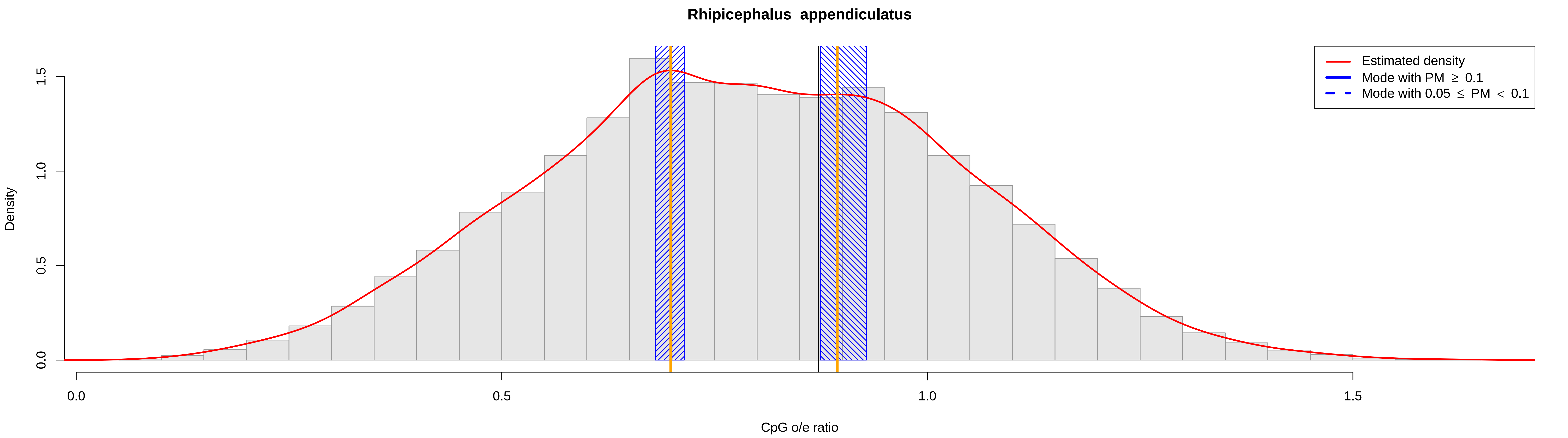
Rhipicephalus_appendiculatus
Estimated density
Mode with PM ≥ 0.1
Mode with 0.05 ≤ PM < 0.1
Density
0.0
0.5
1.0
1.5
CpG o/e ratio

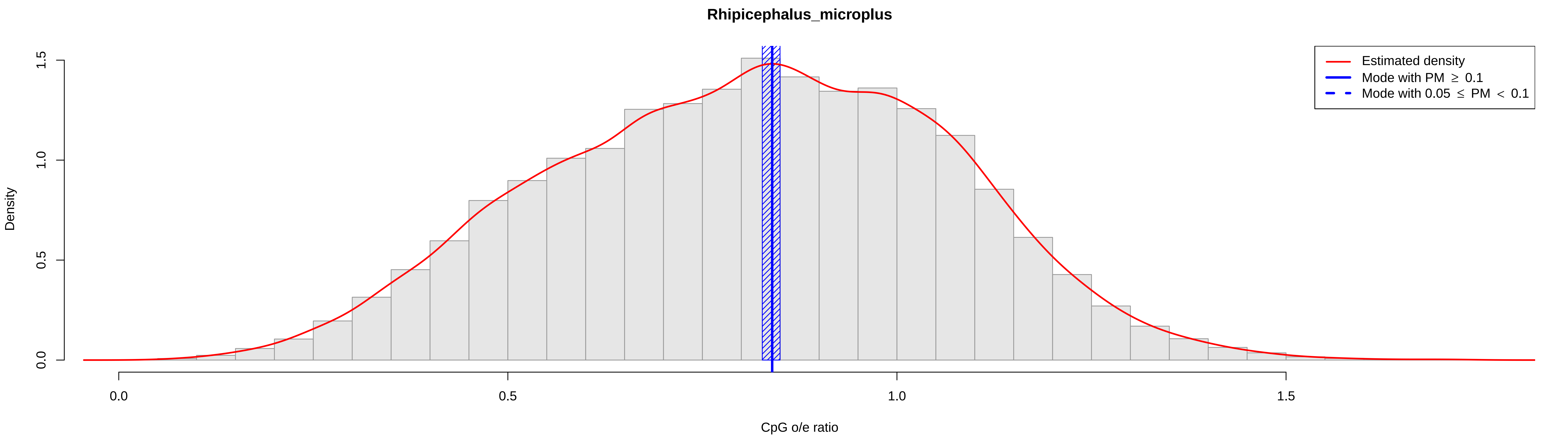

Rhipicephalus_microplus
Estimated density
Mode with PM ≥ 0.1
Mode with 0.05 ≤ PM < 0.1
Density
0.0
0.5
1.0
1.5
CpG o/e ratio

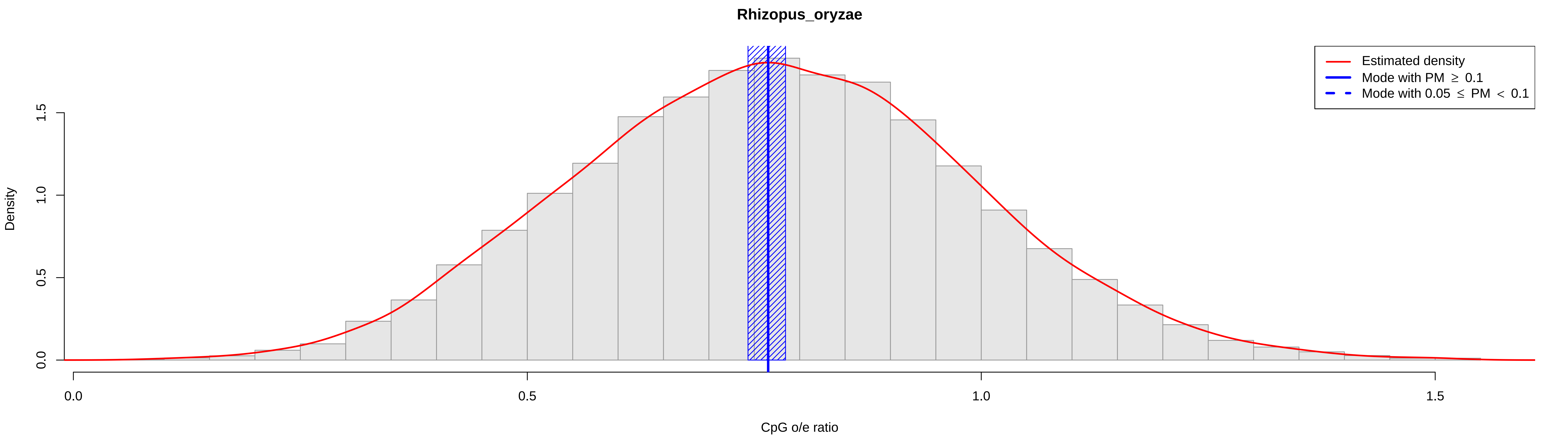

Rhizopus_oryzae
Estimated density
Mode with PM ≥ 0.1
Mode with 0.05 ≤ PM < 0.1
Density
0.0
0.5
1.0
1.5
0.0
0.5
1.0
1.5
CpG o/e ratio

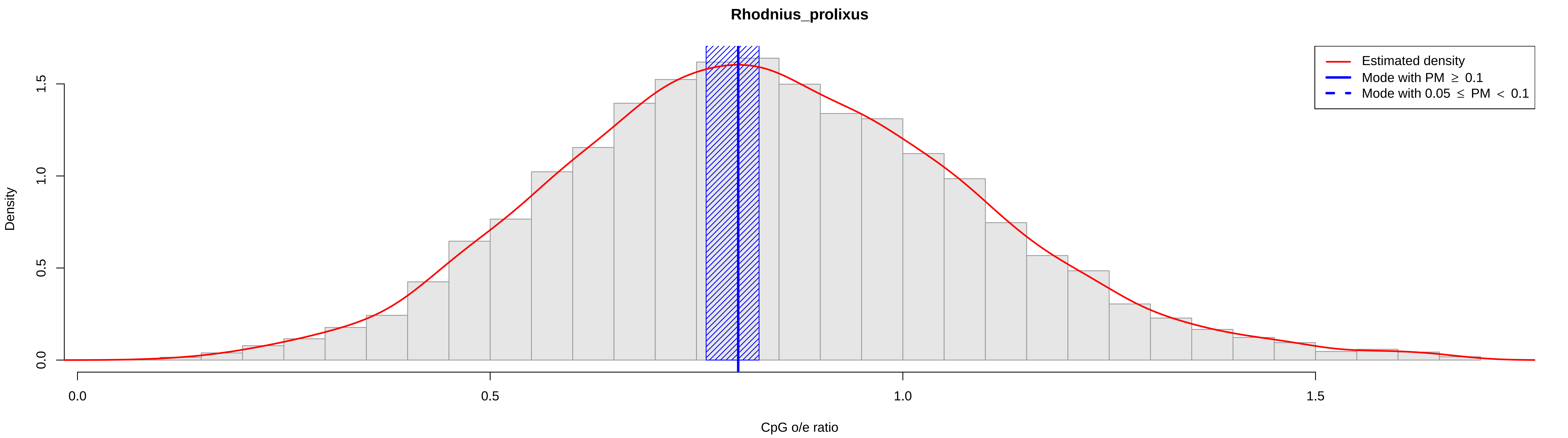
Rhodnius_prolixus
Estimated density
Mode with PM ≥ 0.1
Mode with 0.05 ≤ PM < 0.1
Density
0.0
0.5
1.0
1.5
CpG o/e ratio

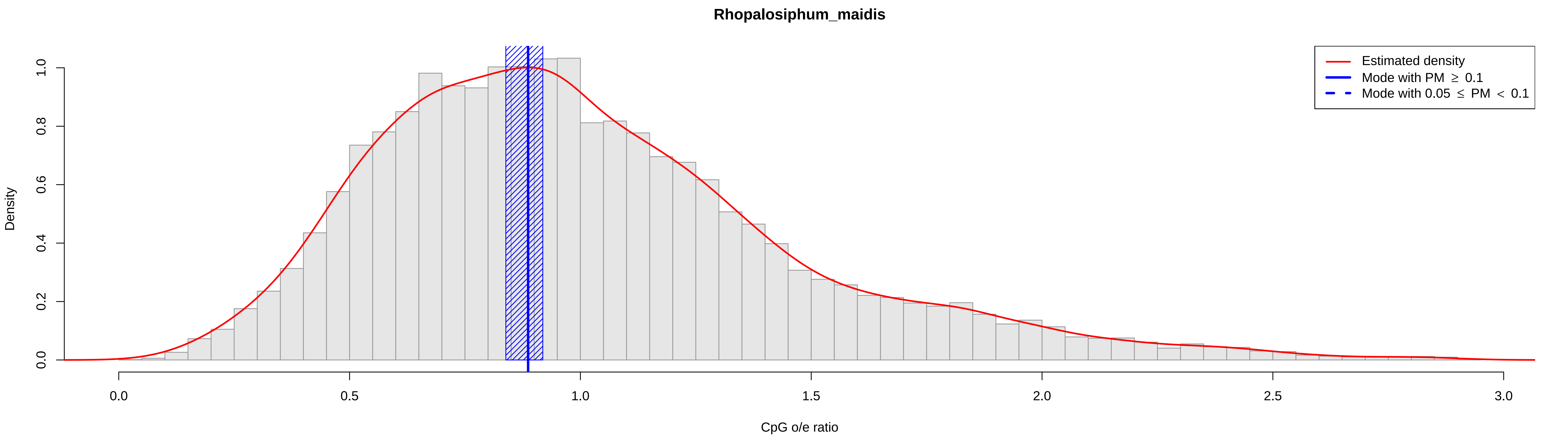

Rhopalosiphum_maidis
Estimated density
Mode with PM ≥ 0.1
Mode with 0.05 ≤ PM < 0.1
Density
0.0
0.2
0.4
0.6
0.8
1.0
0.0
0.5
1.0
1.5
2.0
2.5
3.0
CpG o/e ratio

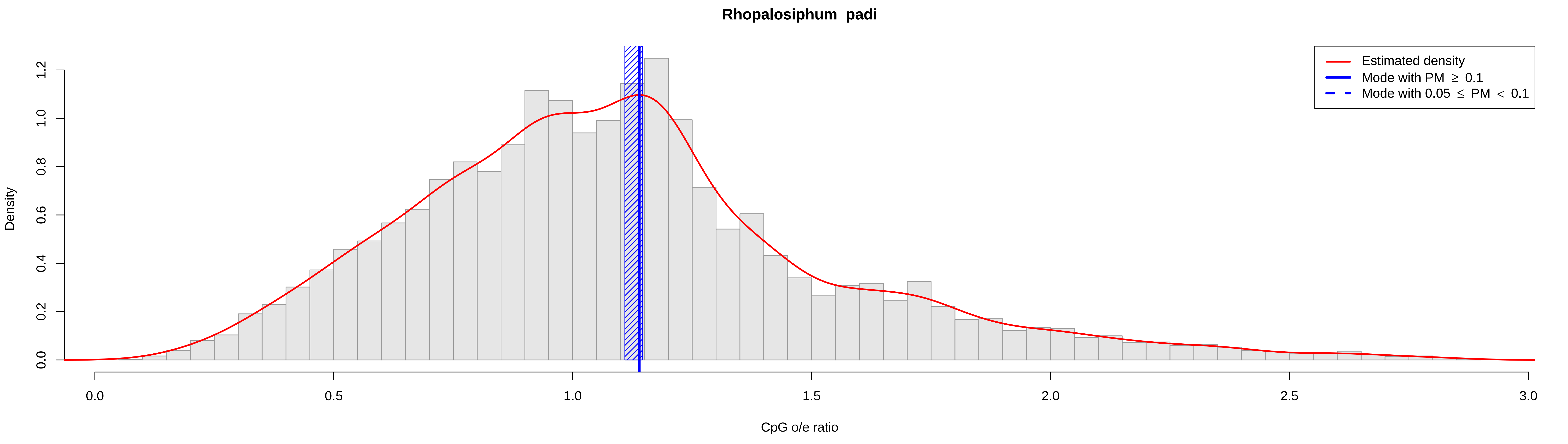

Rhopalosiphum_padi
Estimated density
Mode with PM ≥ 0.1
Mode with 0.05 ≤ PM < 0.1
Density
0.0
0.2
0.4
0.6
0.8
1.0
1.2
0.0
0.5
1.0
1.5
2.0
2.5
3.0
CpG o/e ratio

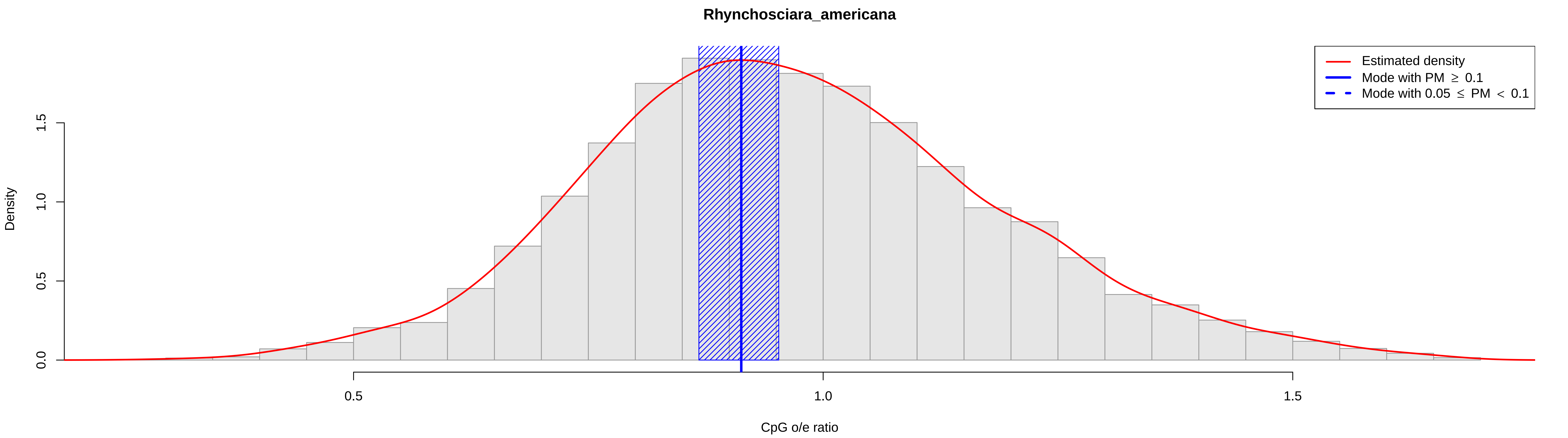

Rhynchosciara_americana
Estimated density
Mode with PM ≥ 0.1
Mode with 0.05 ≤ PM < 0.1
Density
0.0
0.5
1.0
1.5
0.5
1.0
1.5
CpG o/e ratio

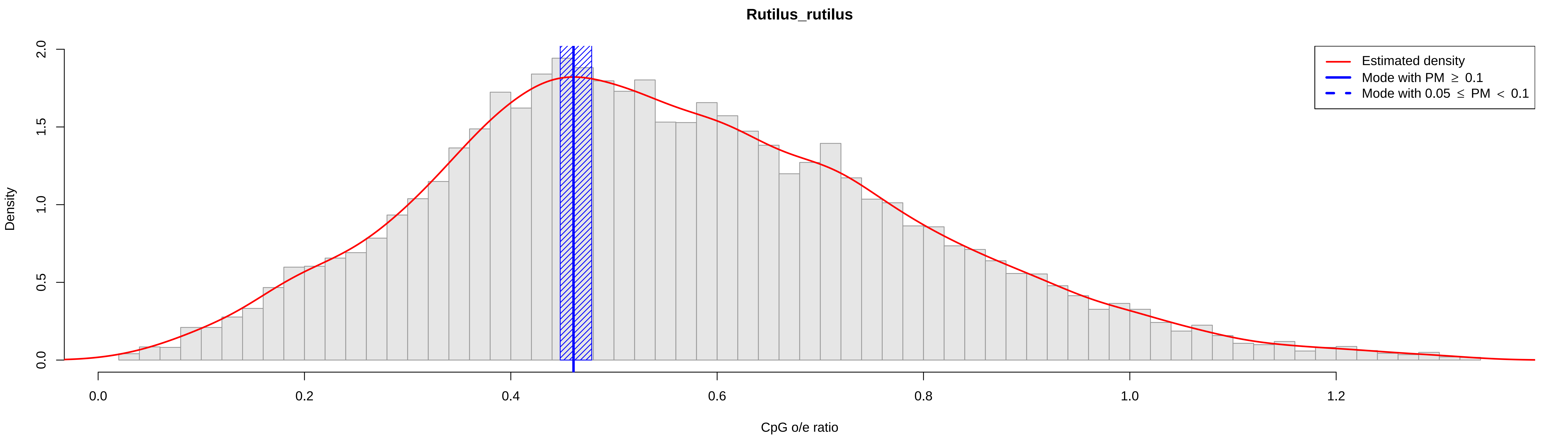

Rutilus_rutilus
Estimated density
Mode with PM ≥ 0.1
Mode with 0.05 ≤ PM < 0.1
Density
0.0
0.5
1.0
1.5
2.0
0.0
0.2
0.4
0.6
0.8
1.0
1.2
CpG o/e ratio

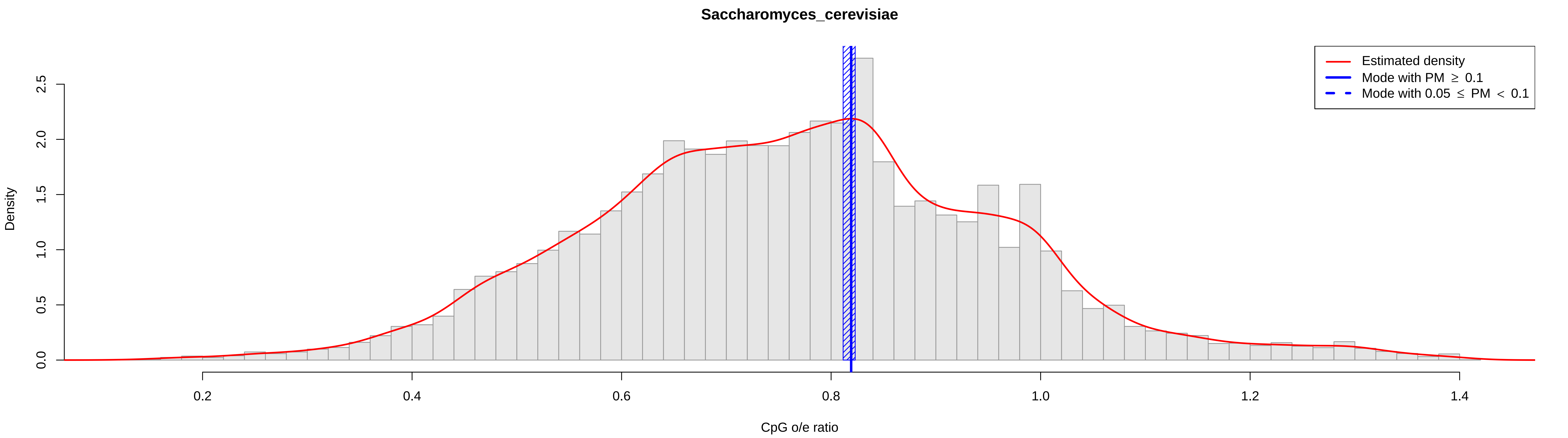
Saccharomyces_cerevisiae
Estimated density
Mode with PM ≥ 0.1
Mode with 0.05 ≤ PM < 0.1
Density
0.0
0.5
1.0
1.5
2.0
2.5
0.2
0.4
0.6
0.8
1.0
1.2
1.4
CpG o/e ratio

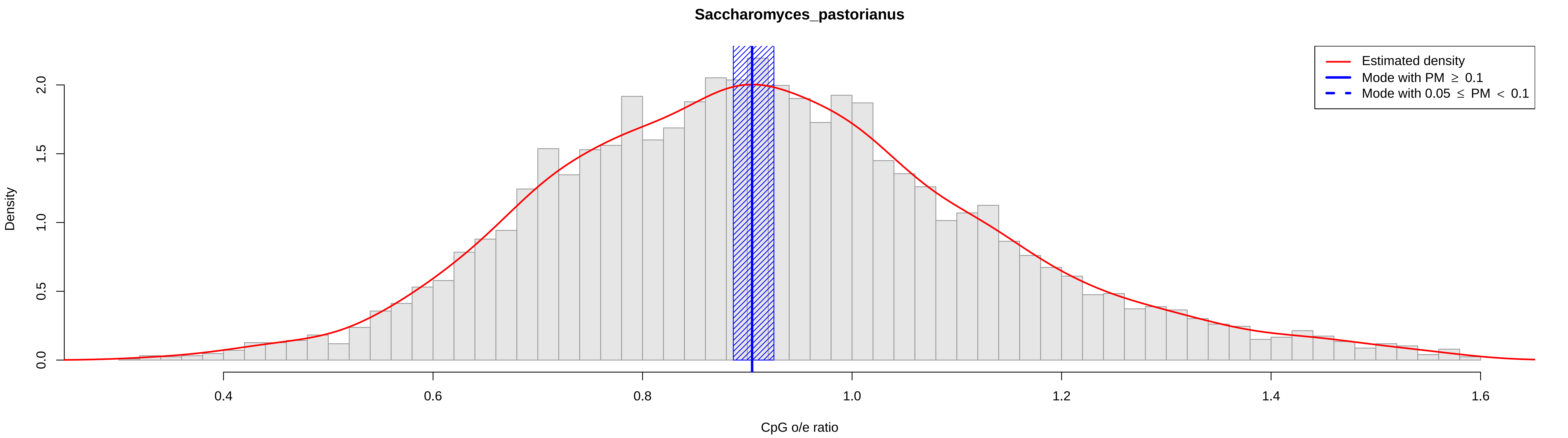

Saccharomyces_pastorianus
Estimated density
Mode with PM ≥ 0.1
Mode with 0.05 ≤ PM < 0.1
Density
0.0
0.5
1.0
1.5
2.0
0.4
0.6
0.8
1.0
1.2
1.4
1.6
CpG o/e ratio

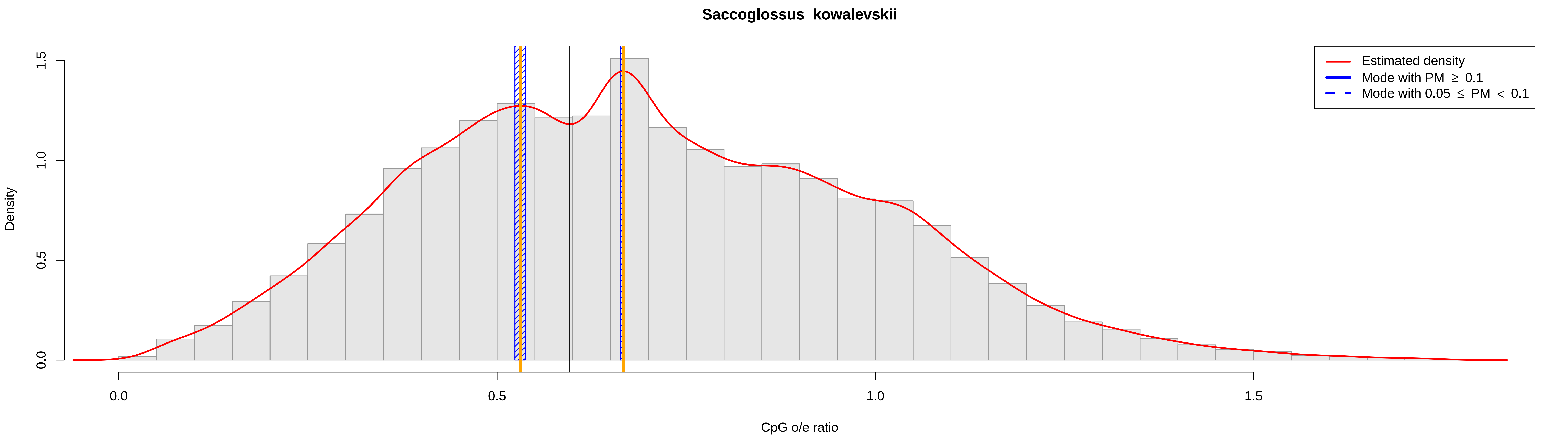
Saccoglossus_kowalevskii
Density
CpG o/e ratio
0.0
0.5
1.0
1.5
Estimated density
Mode with PM ≥ 0.1
Mode with 0.05 ≤ PM < 0.1

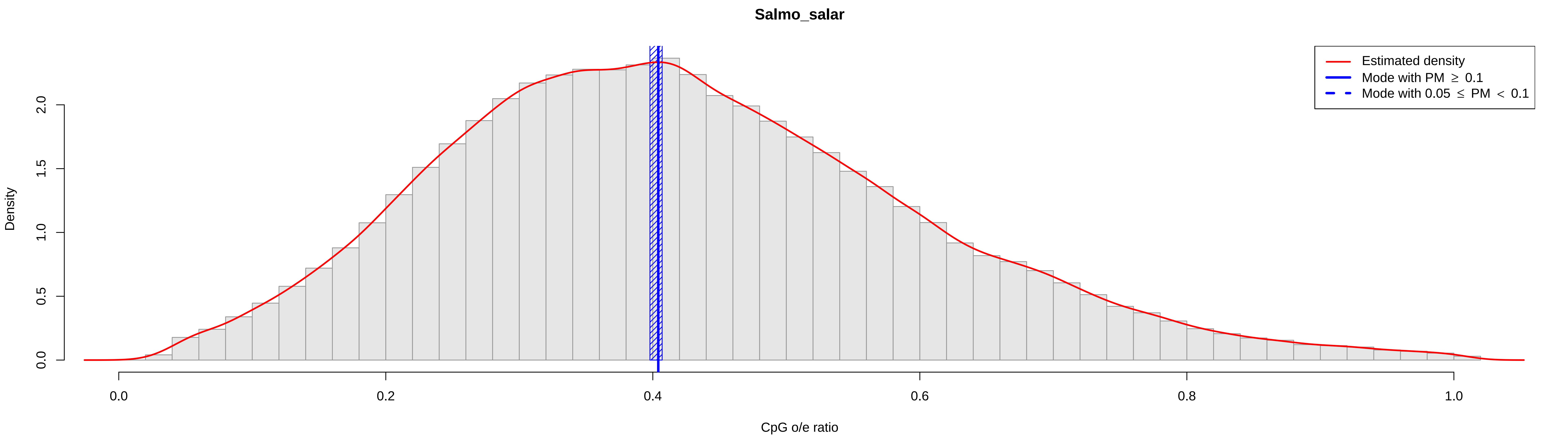
Salmo_salar
Estimated density
Mode with PM ≥ 0.1
Mode with 0.05 ≤ PM < 0.1
Density
0.0
0.5
1.0
1.5
2.0
0.0
0.2
0.4
0.6
0.8
1.0
CpG o/e ratio

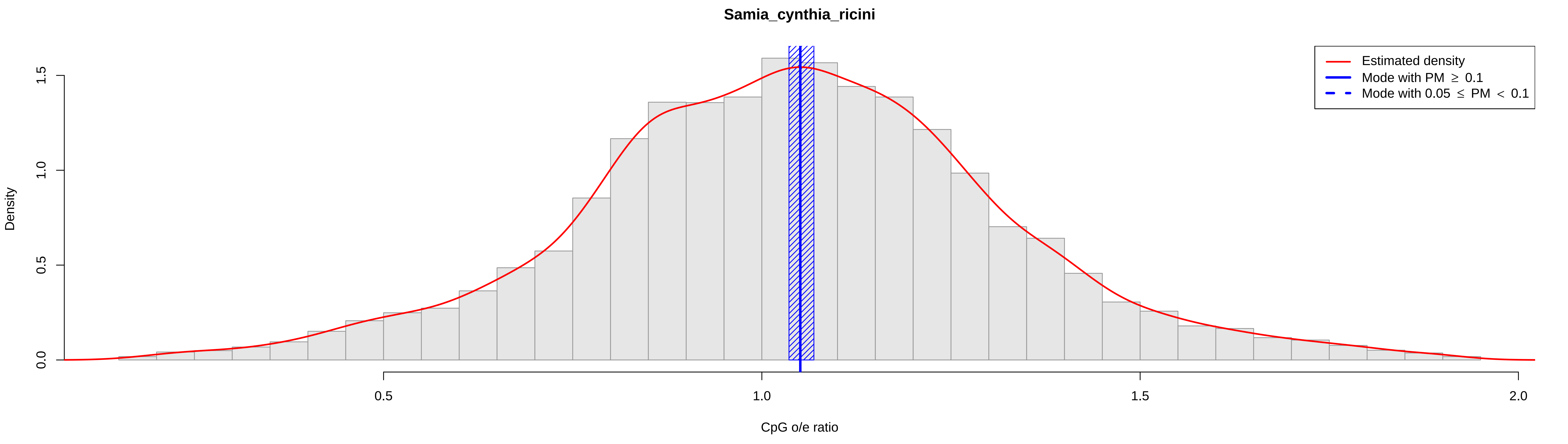

Samia_cynthia_ricini
Estimated density
Mode with PM ≥ 0.1
Mode with 0.05 ≤ PM < 0.1
Density
0.0
0.5
1.0
1.5
0.5
1.0
1.5
2.0
CpG o/e ratio

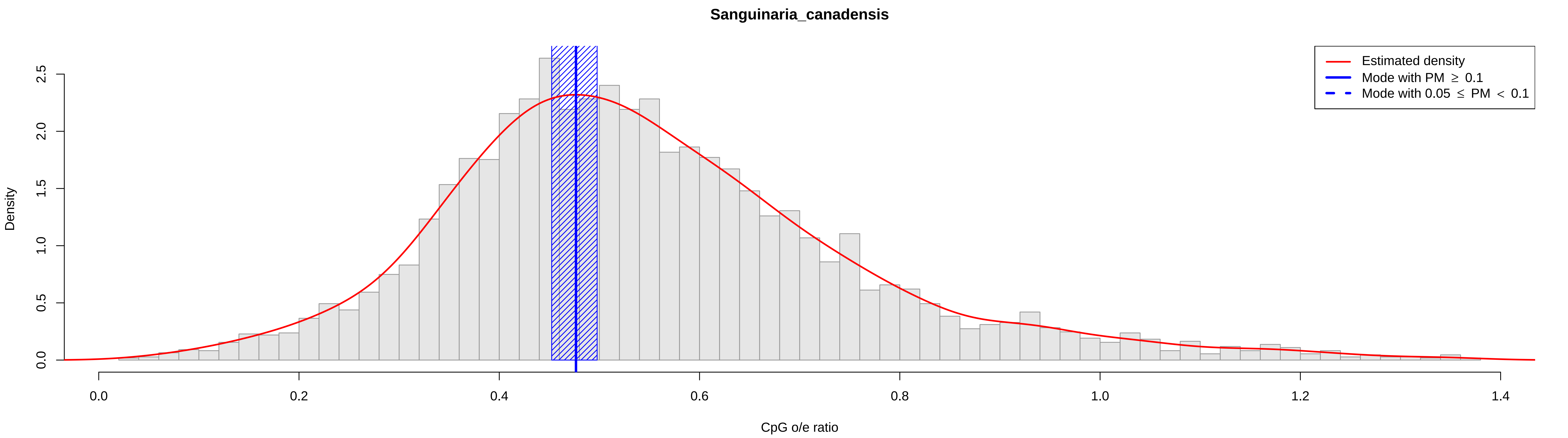

Sanguinaria_canadensis
Estimated density
Mode with PM ≥ 0.1
Mode with 0.05 ≤ PM < 0.1
Density
0.0
0.5
1.0
1.5
2.0
2.5
0.0
0.2
0.4
0.6
0.8
1.0
1.2
1.4
CpG o/e ratio

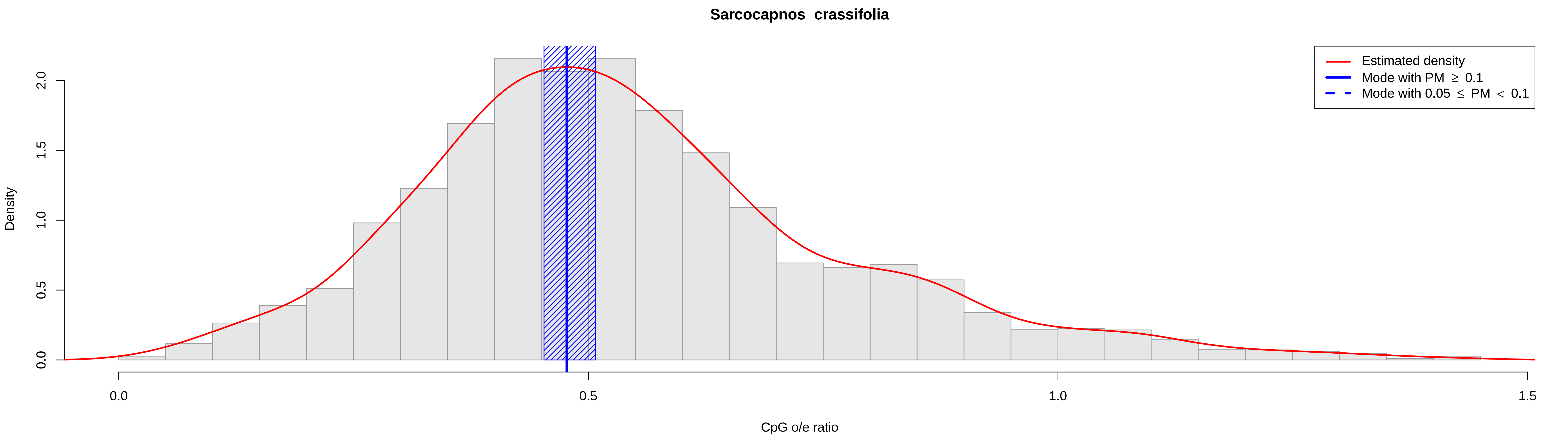

Sarcocapnos_crassifolia
Estimated density
Mode with PM ≥ 0.1
Mode with 0.05 ≤ PM < 0.1
Density
0.0
0.5
1.0
1.5
2.0
0.0
0.5
1.0
1.5
CpG o/e ratio

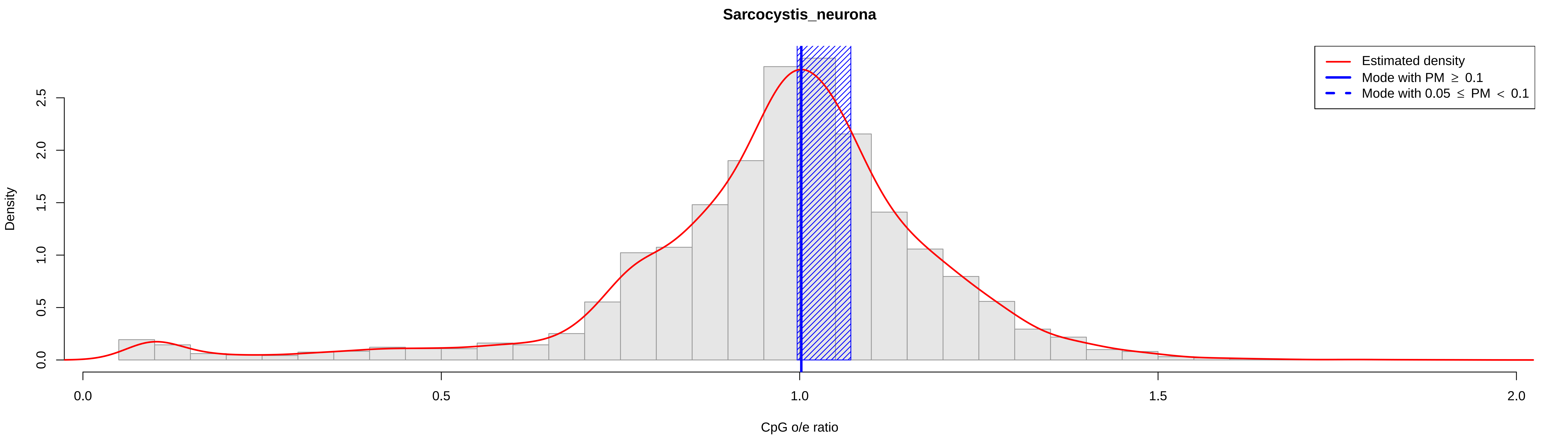
Sarcocystis_neurona
Estimated density
Mode with PM ≥ 0.1
Mode with 0.05 ≤ PM < 0.1
Density
0.0
0.5
1.0
1.5
2.0
2.5
0.0
0.5
1.0
1.5
2.0
CpG o/e ratio

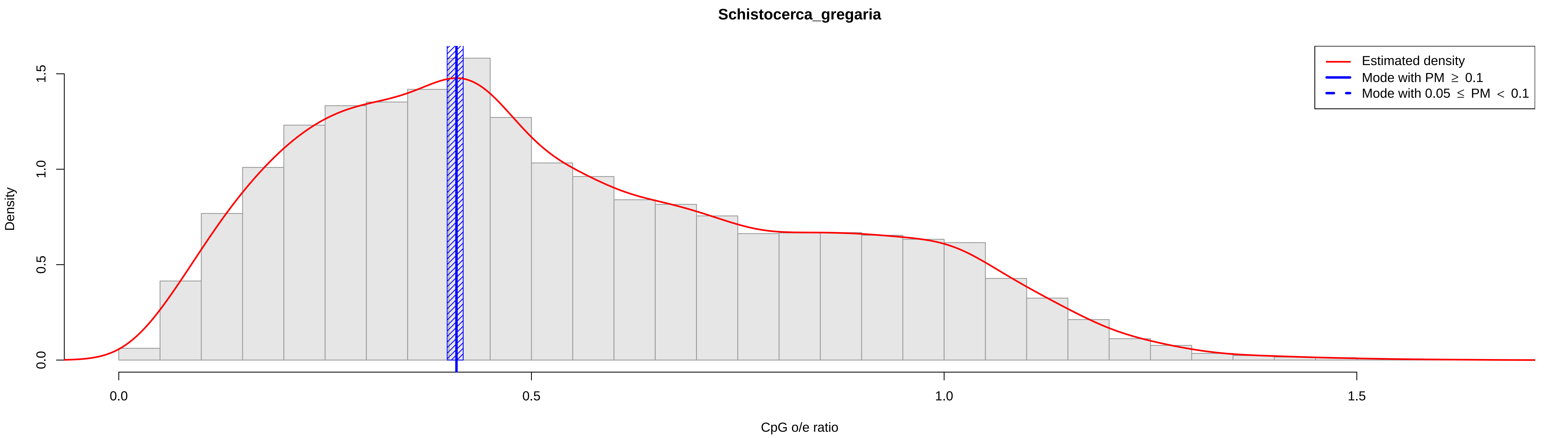

Schistocerca_gregaria
Estimated density
Mode with PM ≥ 0.1
Mode with 0.05 ≤ PM < 0.1
Density
0.0
0.5
1.0
1.5
CpG o/e ratio

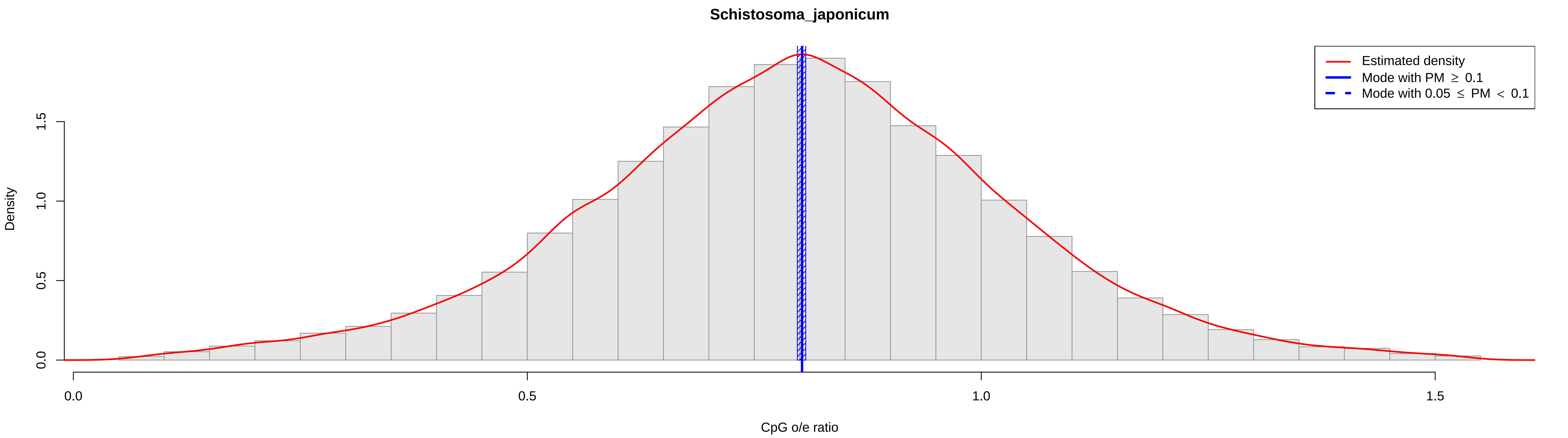

Schistosoma_japonicum
Estimated density
Mode with PM ≥ 0.1
Mode with 0.05 ≤ PM < 0.1
Density
0.0
0.5
1.0
1.5
CpG o/e ratio

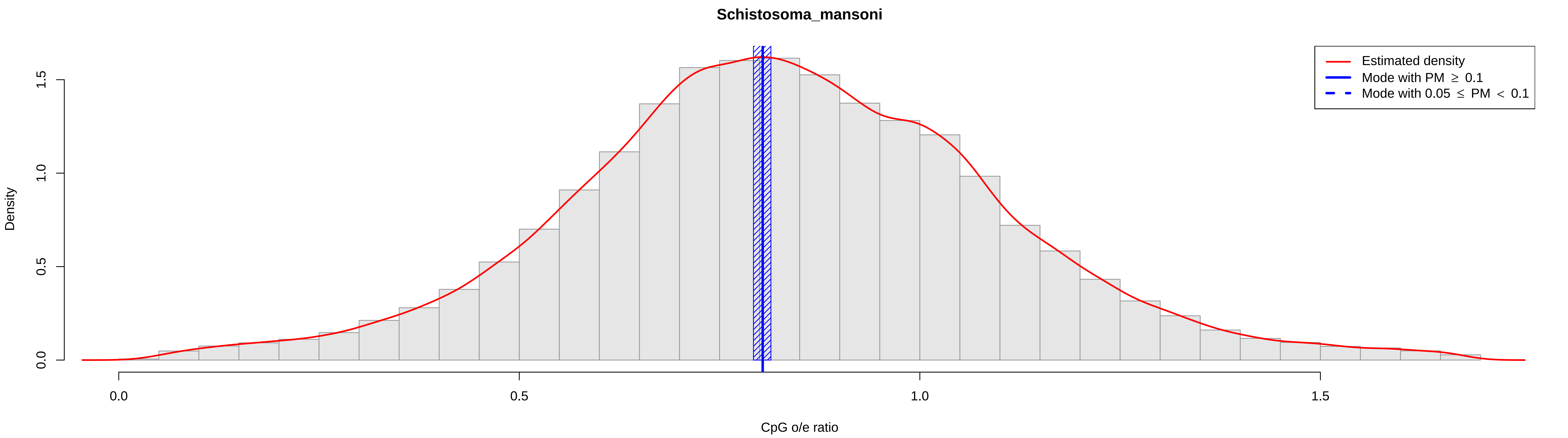

Schistosoma_mansoni
Estimated density
Mode with PM ≥ 0.1
Mode with 0.05 ≤ PM < 0.1
Density
0.0
0.5
1.0
1.5
0.0
0.5
1.0
1.5
CpG o/e ratio

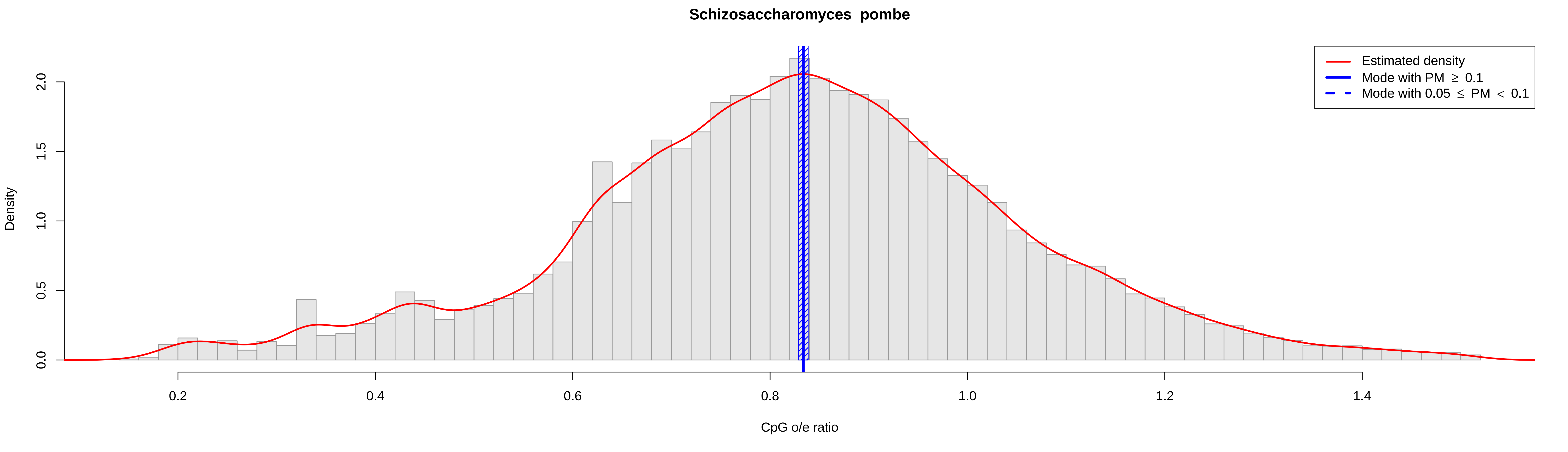

Schizosaccharomyces_pombe
Estimated density
Mode with PM ≥ 0.1
Mode with 0.05 ≤ PM < 0.1
Density
0.0
0.5
1.0
1.5
2.0
0.2
0.4
0.6
0.8
1.0
1.2
1.4
CpG o/e ratio

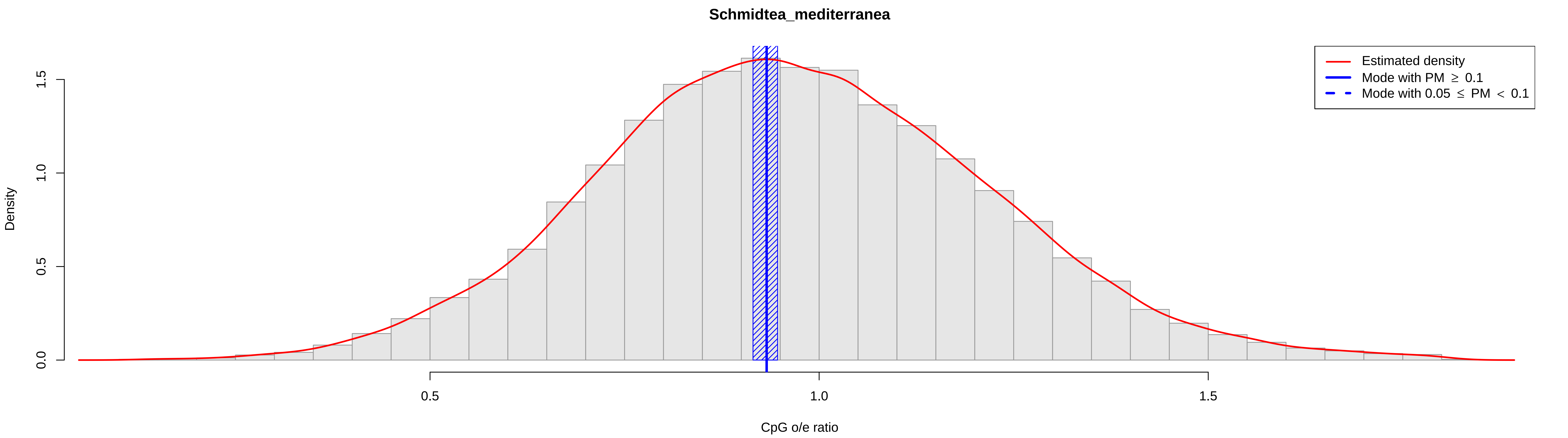

Schmidtea_mediterranea
Estimated density
Mode with PM ≥ 0.1
Mode with 0.05 ≤ PM < 0.1
Density
0.0
0.5
1.0
1.5
0.5
1.0
1.5
CpG o/e ratio

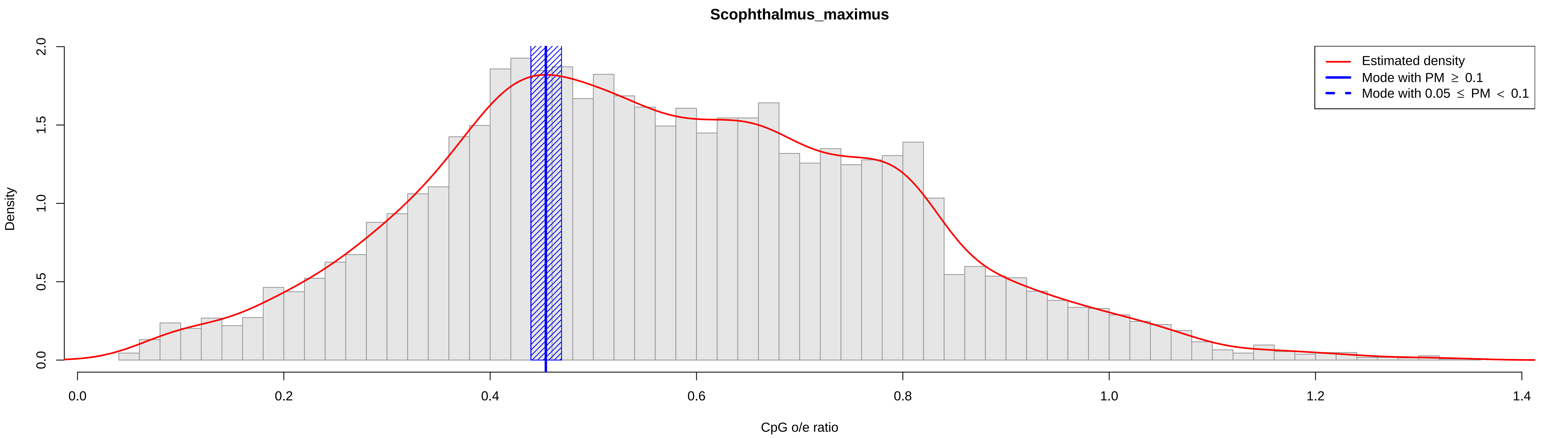

Scophthalmus_maximus
Estimated density
Mode with PM ≥ 0.1
Mode with 0.05 ≤ PM < 0.1
Density
CpG o/e ratio
0.0
0.5
1.0
1.5
2.0
0.0
0.2
0.4
0.6
0.8
1.0
1.2
1.4

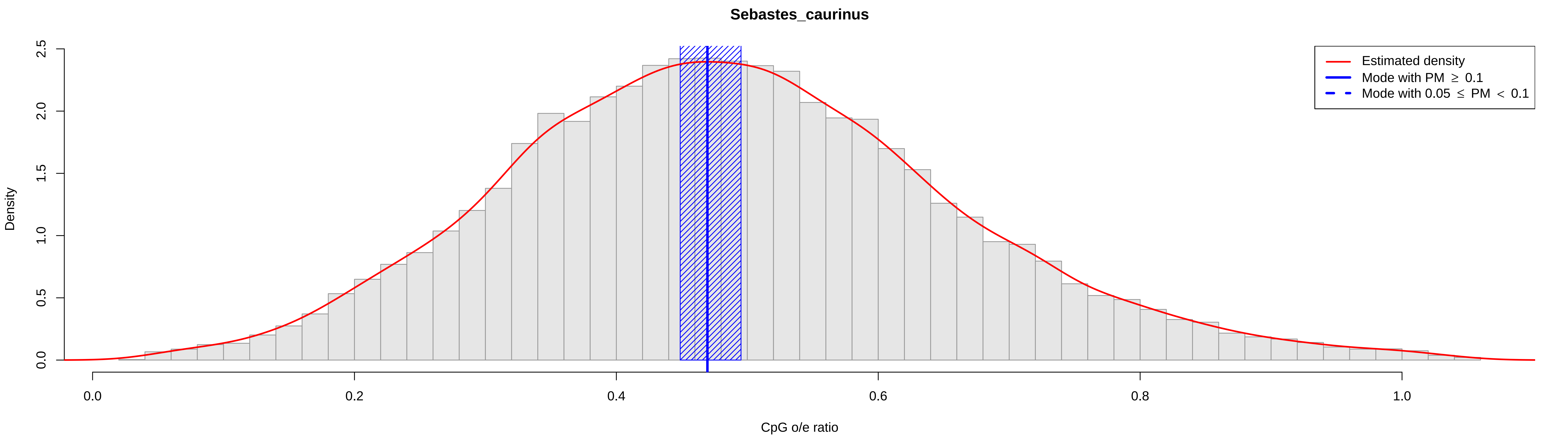
Sebastes_caurinus
Density
CpG o/e ratio
0.0
0.5
1.0
1.5
2.0
2.5
0.0
0.2
0.4
0.6
0.8
1.0
Estimated density
Mode with PM ≥ 0.1
Mode with 0.05 ≤ PM < 0.1

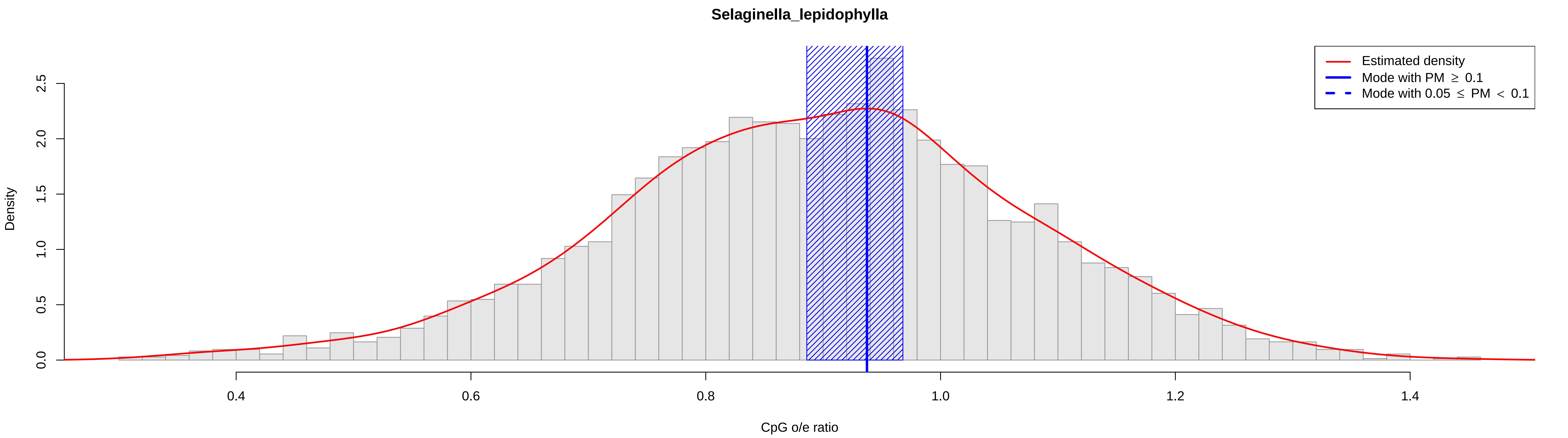

Selaginella_lepidophylla
Estimated density
Mode with PM ≥ 0.1
Mode with 0.05 ≤ PM < 0.1
Density
0.0
0.5
1.0
1.5
2.0
2.5
0.4
0.6
0.8
1.0
1.2
1.4
CpG o/e ratio

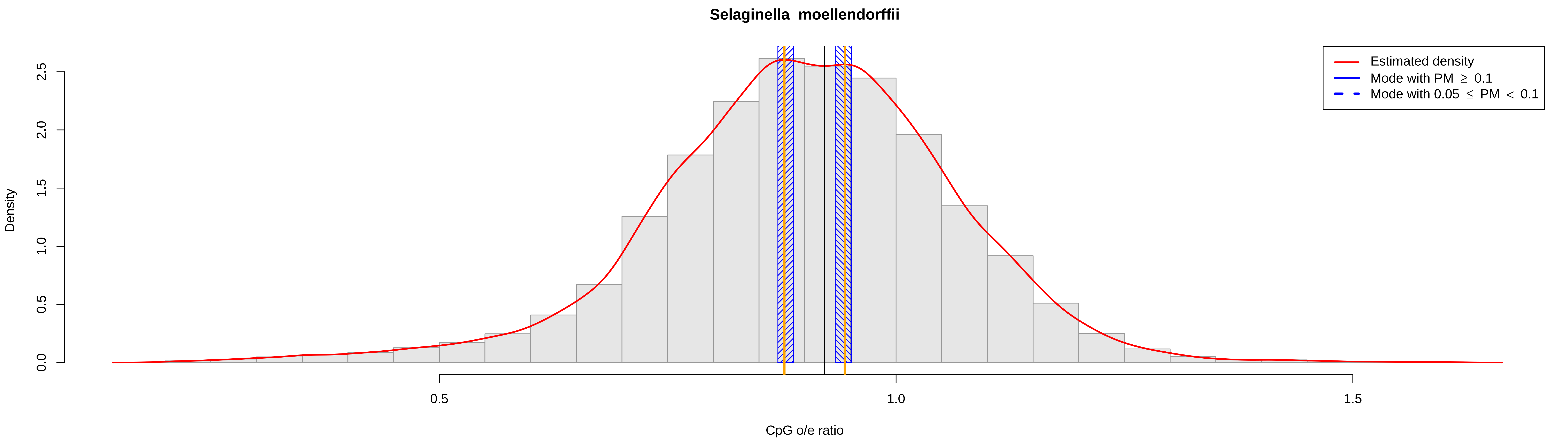

Selaginella_moellendorffii
Estimated density
Mode with PM ≥ 0.1
Mode with 0.05 ≤ PM < 0.1
Density
0.0
0.5
1.0
1.5
2.0
2.5
0.5
1.0
1.5
CpG o/e ratio

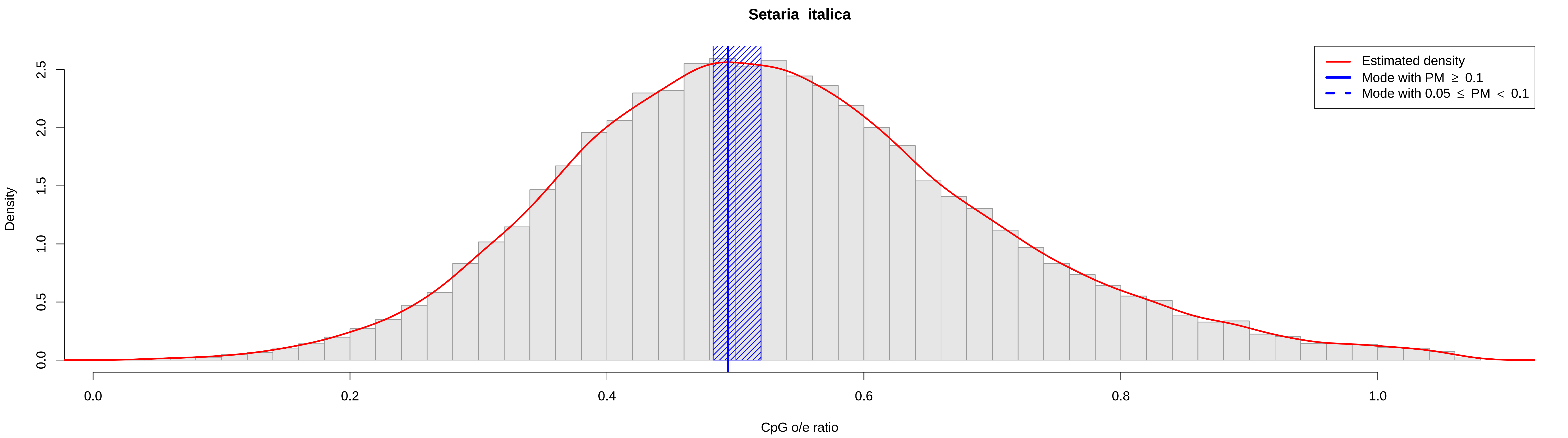

Setaria_italica
Estimated density
Mode with PM ≥ 0.1
Mode with 0.05 ≤ PM < 0.1
Density
0.0
0.5
1.0
1.5
2.0
2.5
0.0
0.2
0.4
0.6
0.8
1.0
CpG o/e ratio

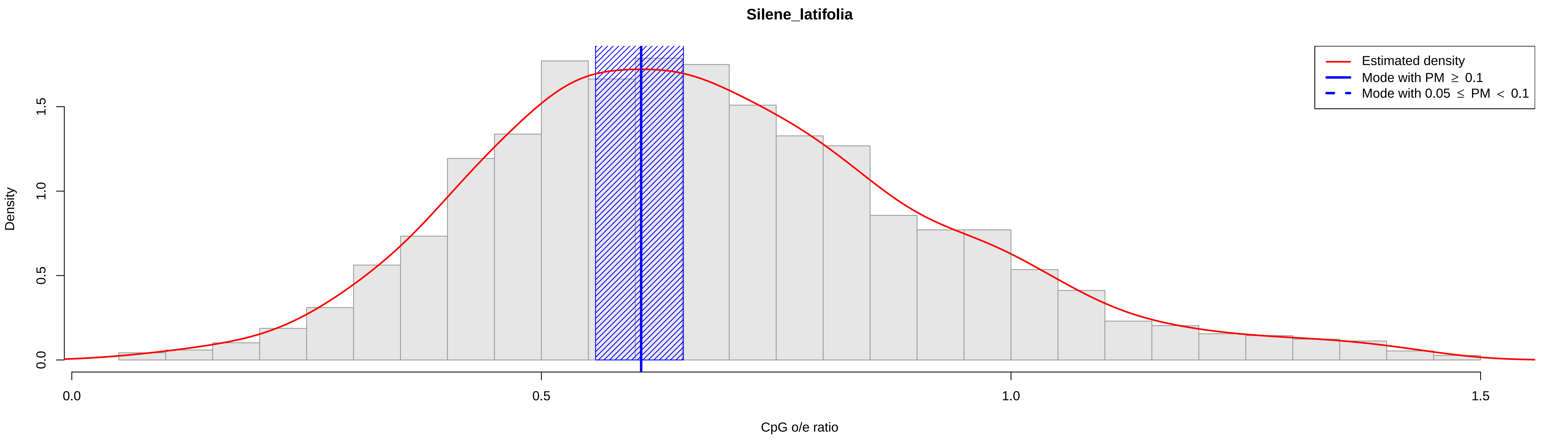

Silene_latifolia
Estimated density
Mode with PM ≥ 0.1
Mode with 0.05 ≤ PM < 0.1
Density
0.0
0.5
1.0
1.5
CpG o/e ratio

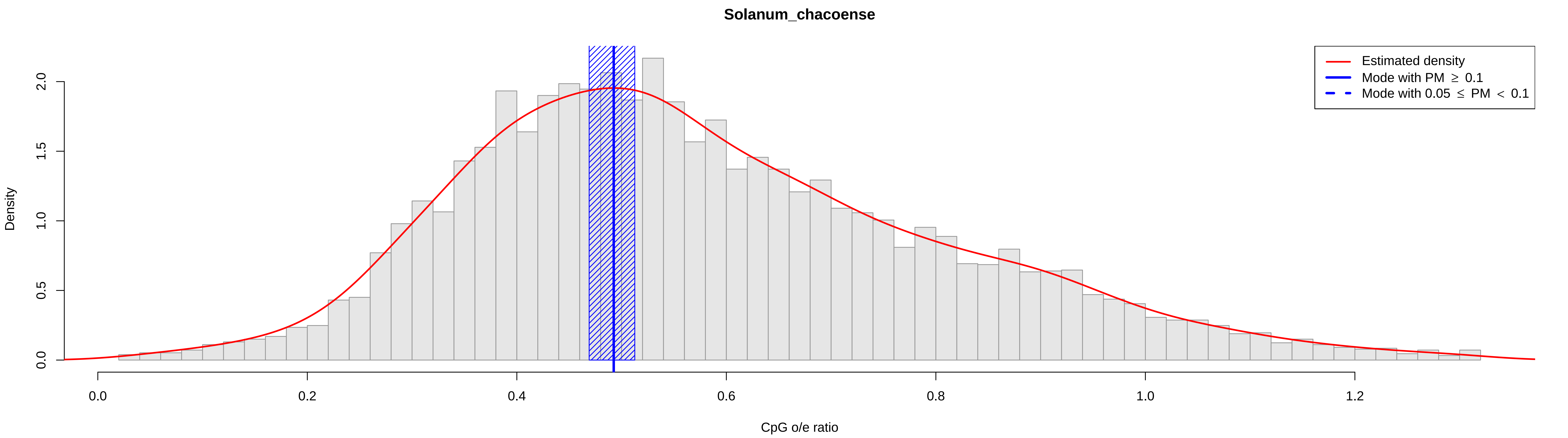
Solanum_chacoense
Estimated density
Mode with PM ≥ 0.1
Mode with 0.05 ≤ PM < 0.1
Density
0.0
0.5
1.0
1.5
2.0
0.0
0.2
0.4
0.6
0.8
1.0
1.2
CpG o/e ratio

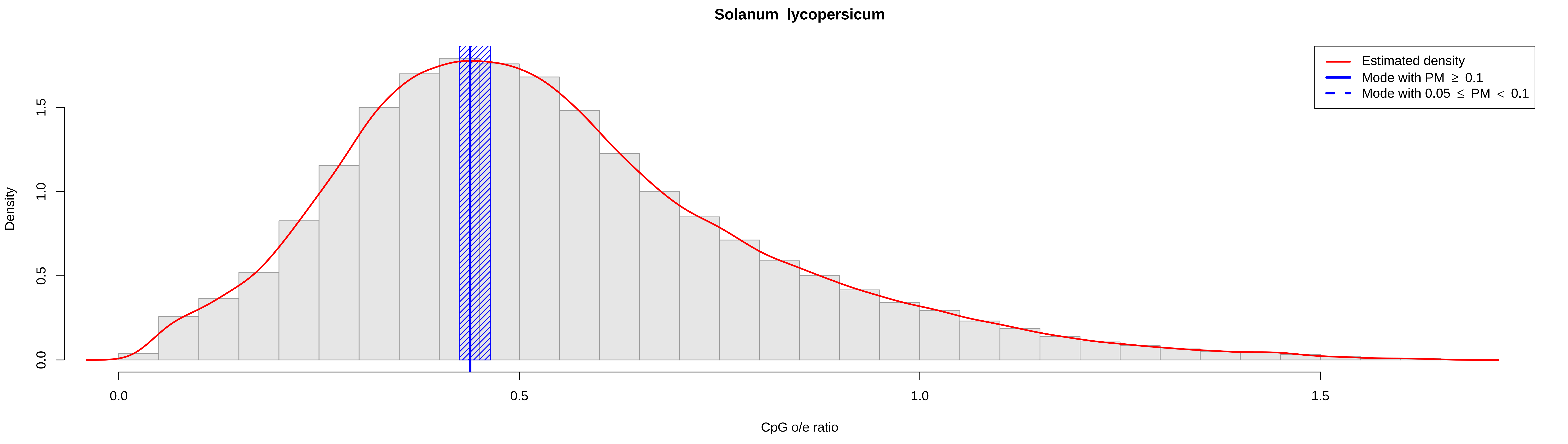

Solanum_lycopersicum
Estimated density
Mode with PM ≥ 0.1
Mode with 0.05 ≤ PM < 0.1
Density
0.0
0.5
1.0
1.5
0.0
0.5
1.0
1.5
CpG o/e ratio

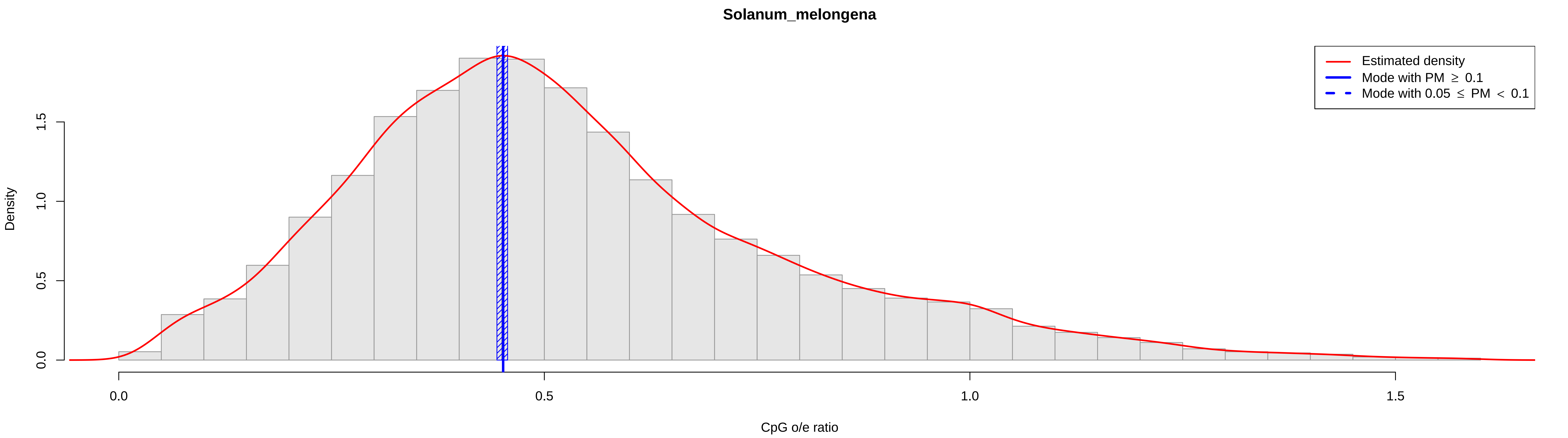
Solanum_melongena
Estimated density
Mode with PM ≥ 0.1
Mode with 0.05 ≤ PM < 0.1
Density
0.0
0.5
1.0
1.5
0.0
0.5
1.0
1.5
CpG o/e ratio

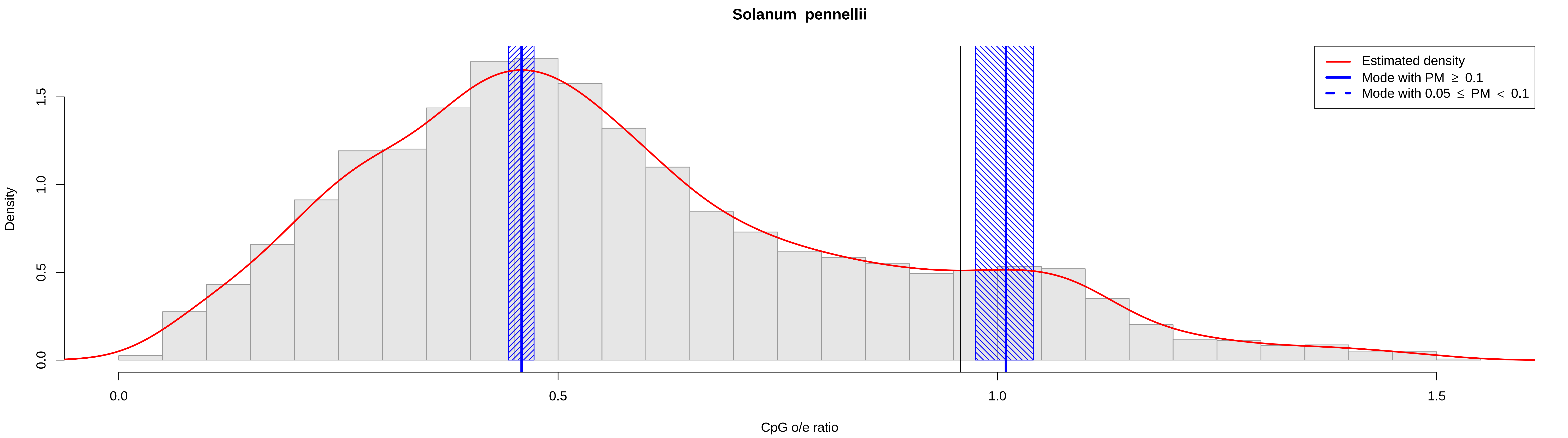

Solanum_pennellii
Estimated density
Mode with PM ≥ 0.1
Mode with 0.05 ≤ PM < 0.1
Density
0.0
0.5
1.0
1.5
0.0
0.5
1.0
1.5
CpG o/e ratio

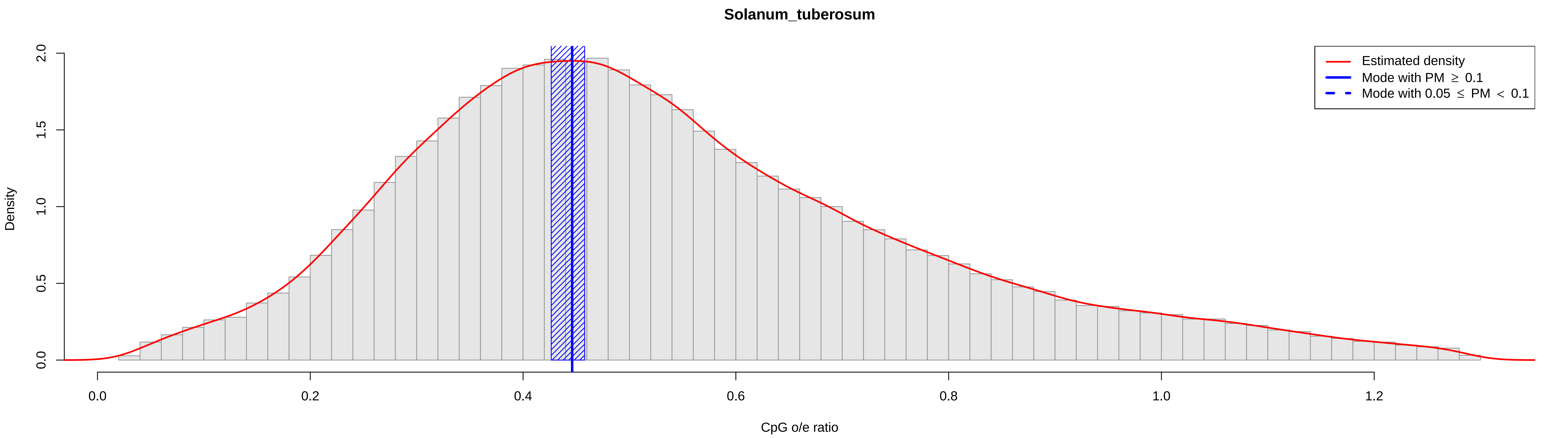

Solanum_tuberosum
Estimated density
Mode with PM ≥ 0.1
Mode with 0.05 ≤ PM < 0.1
Density
0.0
0.5
1.0
1.5
2.0
0.0
0.2
0.4
0.6
0.8
1.0
1.2
CpG o/e ratio

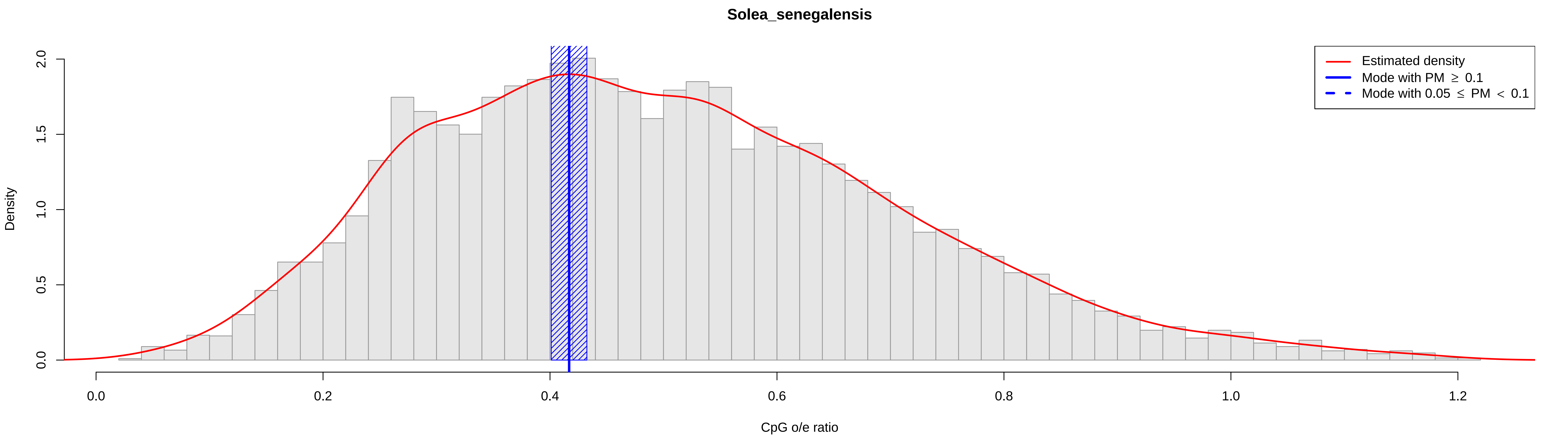
Solea_senegalensis
Estimated density
Mode with PM ≥ 0.1
Mode with 0.05 ≤ PM < 0.1
Density
0.0
0.5
1.0
1.5
2.0
0.0
0.2
0.4
0.6
0.8
1.0
1.2
CpG o/e ratio

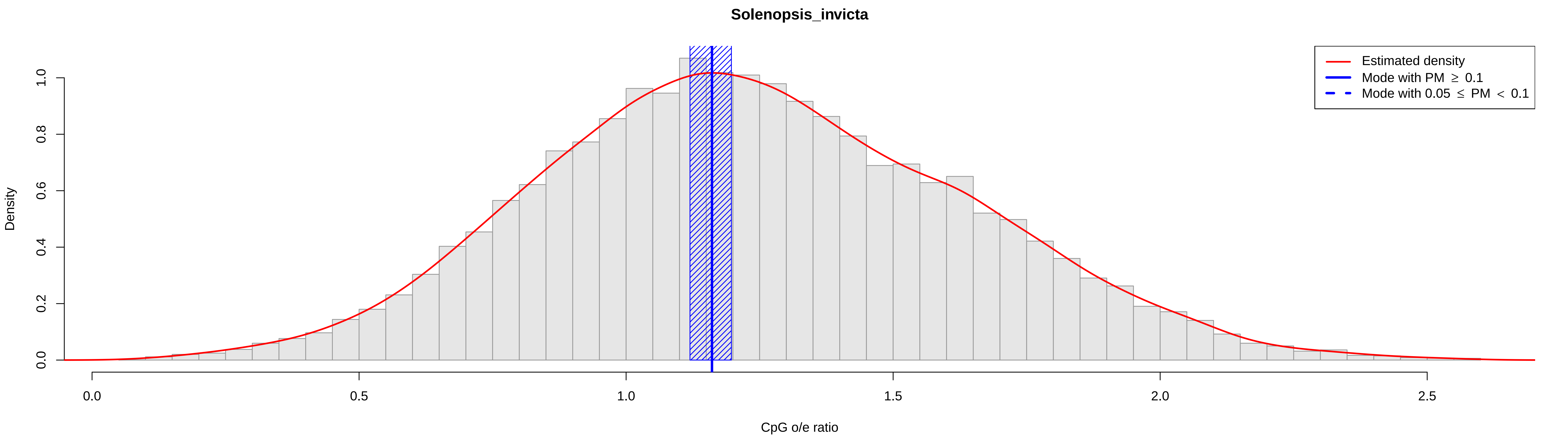

Solenopsis_invicta
Estimated density
Mode with PM ≥ 0.1
Mode with 0.05 ≤ PM < 0.1
Density
0.0
0.2
0.4
0.6
0.8
1.0
0.0
0.5
1.0
1.5
2.0
2.5
CpG o/e ratio

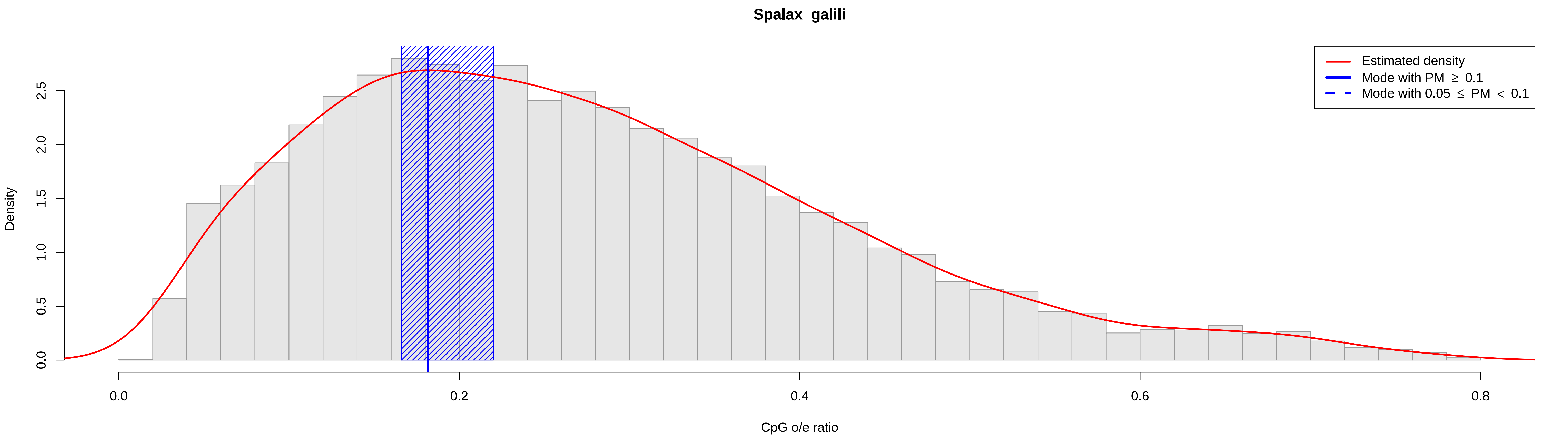
Spalax_galili
Estimated density
Mode with PM ≥ 0.1
Mode with 0.05 ≤ PM < 0.1
Density
0.0
0.5
1.0
1.5
2.0
2.5
0.0
0.2
0.4
0.6
0.8
CpG o/e ratio

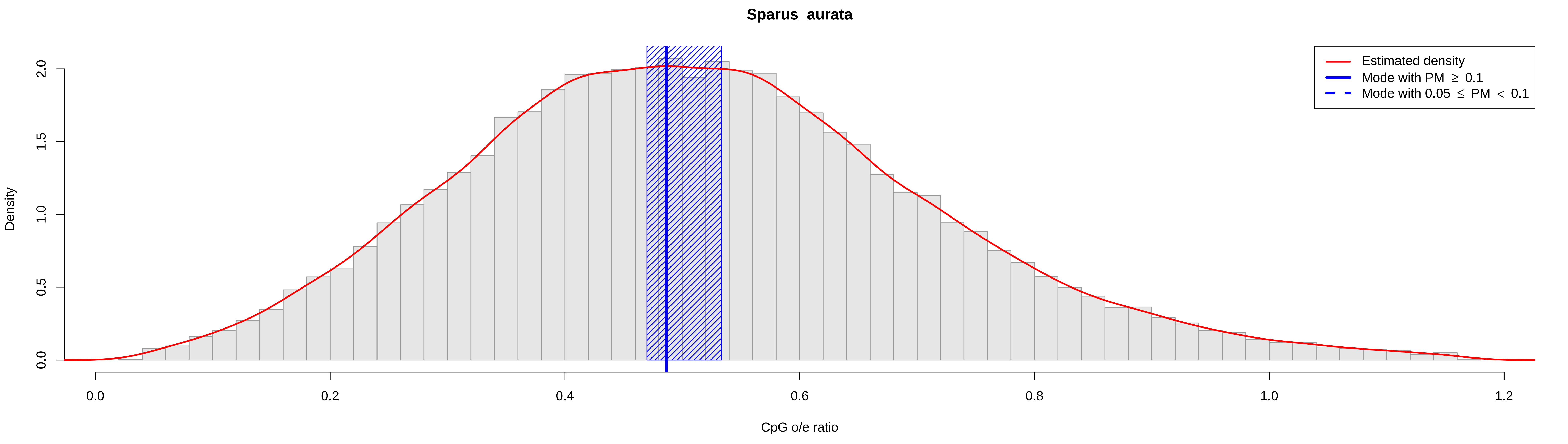
Sparus_aurata
Estimated density
Mode with PM ≥ 0.1
Mode with 0.05 ≤ PM < 0.1
Density
0.0
0.5
1.0
1.5
2.0
0.0
0.2
0.4
0.6
0.8
1.0
1.2
CpG o/e ratio

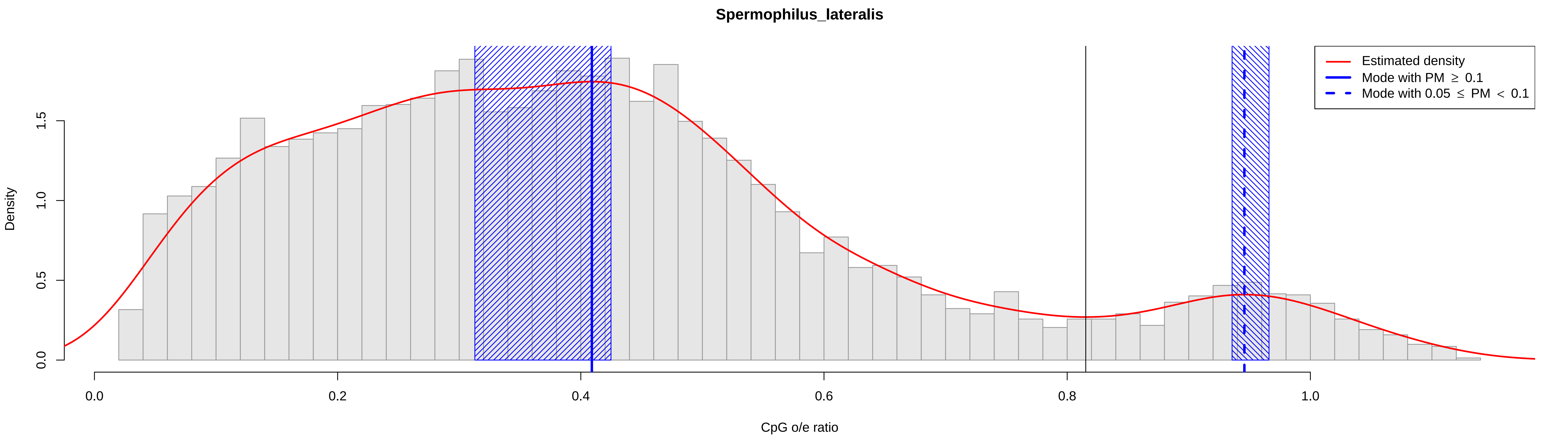
Spermophilus_lateralis
Estimated density
Mode with PM ≥ 0.1
Mode with 0.05 ≤ PM < 0.1
Density
0.0
0.5
1.0
1.5
0.0
0.2
0.4
0.6
0.8
1.0
CpG o/e ratio

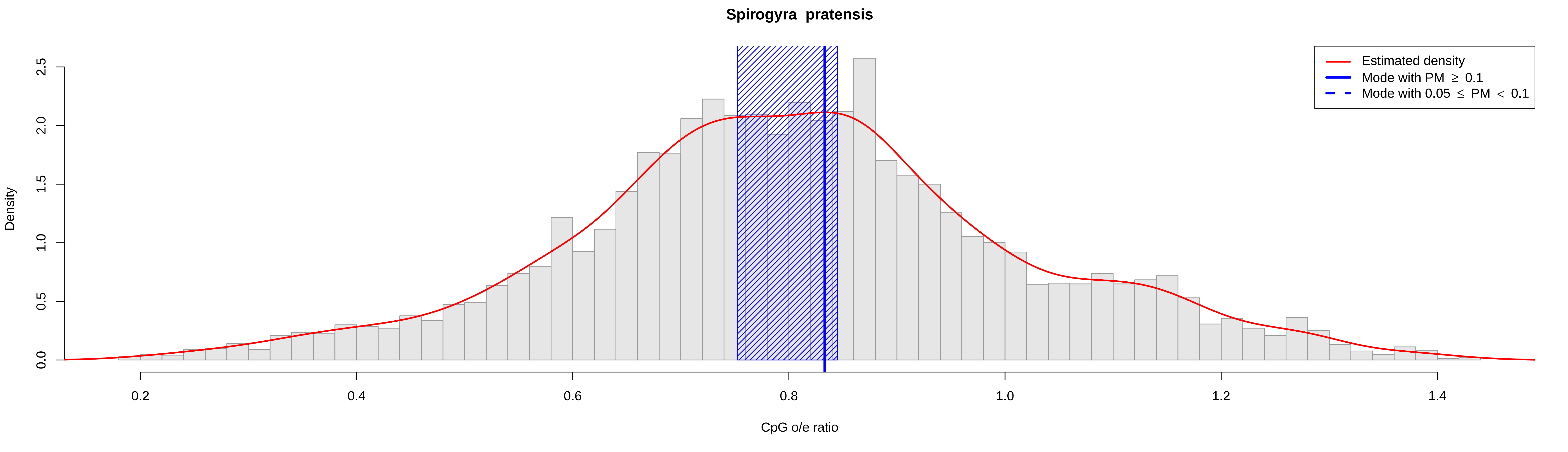

Spirogyra_pratensis
Estimated density
Mode with PM ≥ 0.1
Mode with 0.05 ≤ PM < 0.1
Density
0.0
0.5
1.0
1.5
2.0
2.5
0.2
0.4
0.6
0.8
1.0
1.2
1.4
CpG o/e ratio

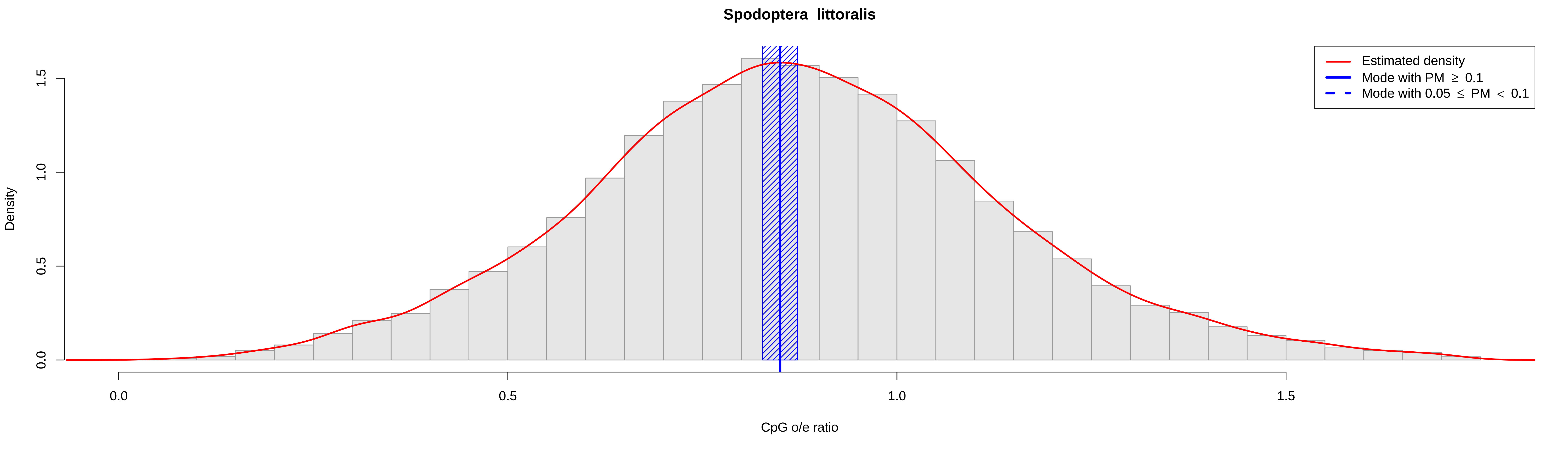
Spodoptera_littoralis
Estimated density
Mode with PM ≥ 0.1
Mode with 0.05 ≤ PM < 0.1
Density
0.0
0.5
1.0
1.5
0.0
0.5
1.0
1.5
CpG o/e ratio

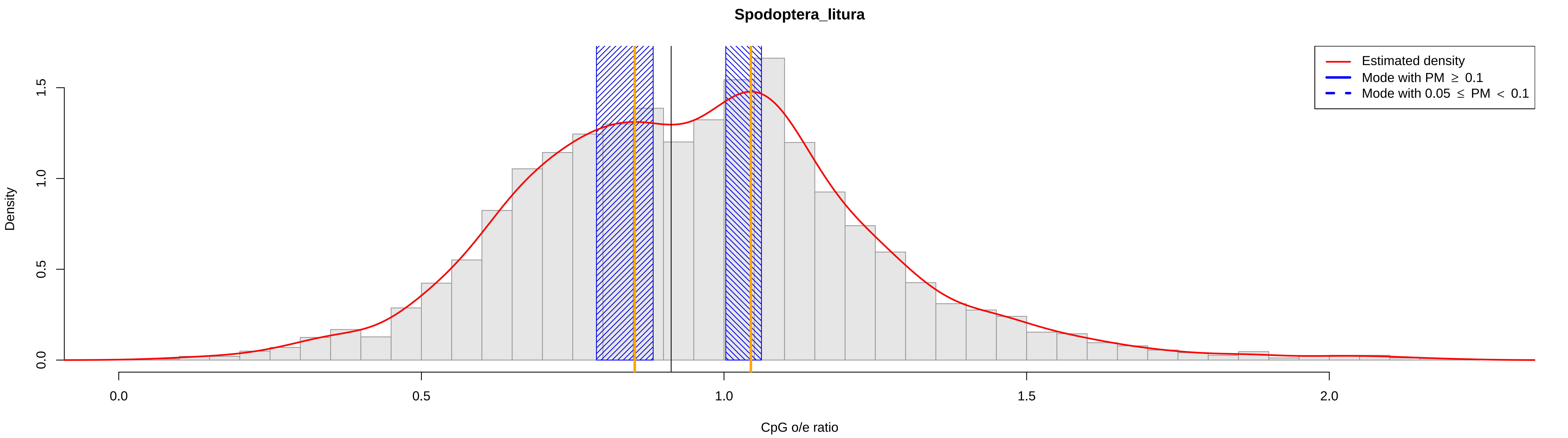
Spodoptera_litura
Estimated density
Mode with PM ≥ 0.1
Mode with 0.05 ≤ PM < 0.1
Density
0.0
0.5
1.0
1.5
0.0
0.5
1.0
1.5
2.0
CpG o/e ratio

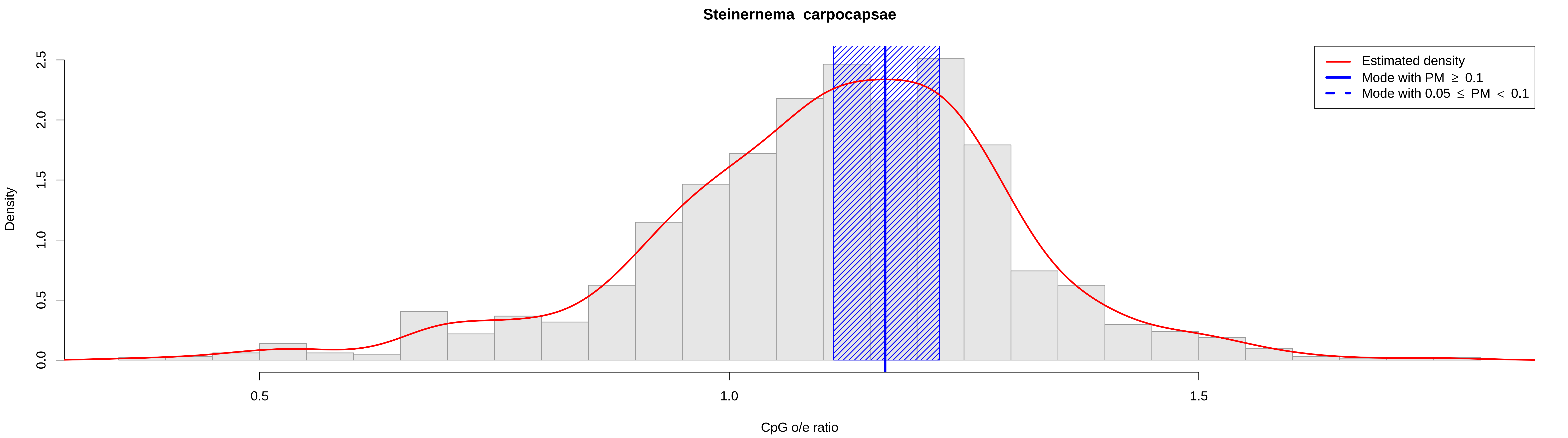

Steinernema_carpocapsae
Estimated density
Mode with PM ≥ 0.1
Mode with 0.05 ≤ PM < 0.1
Density
0.0
0.5
1.0
1.5
2.0
2.5
0.5
1.0
1.5
CpG o/e ratio

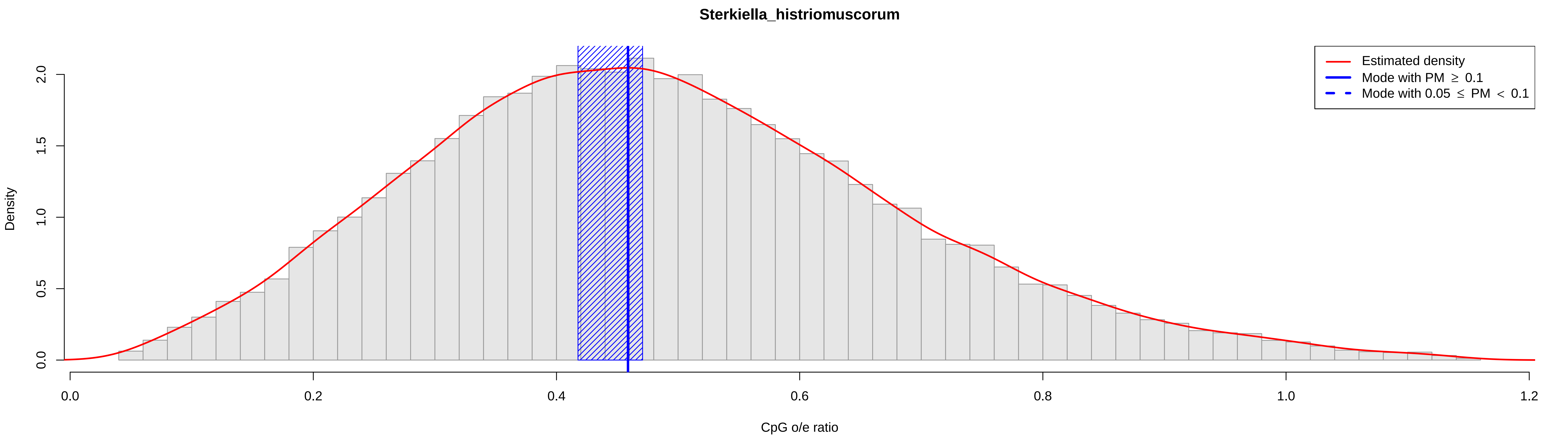

Sterkiella_histriomuscorum
Estimated density
Mode with PM ≥ 0.1
Mode with 0.05 ≤ PM < 0.1
Density
0.0
0.5
1.0
1.5
2.0
0.0
0.2
0.4
0.6
0.8
1.0
1.2
CpG o/e ratio

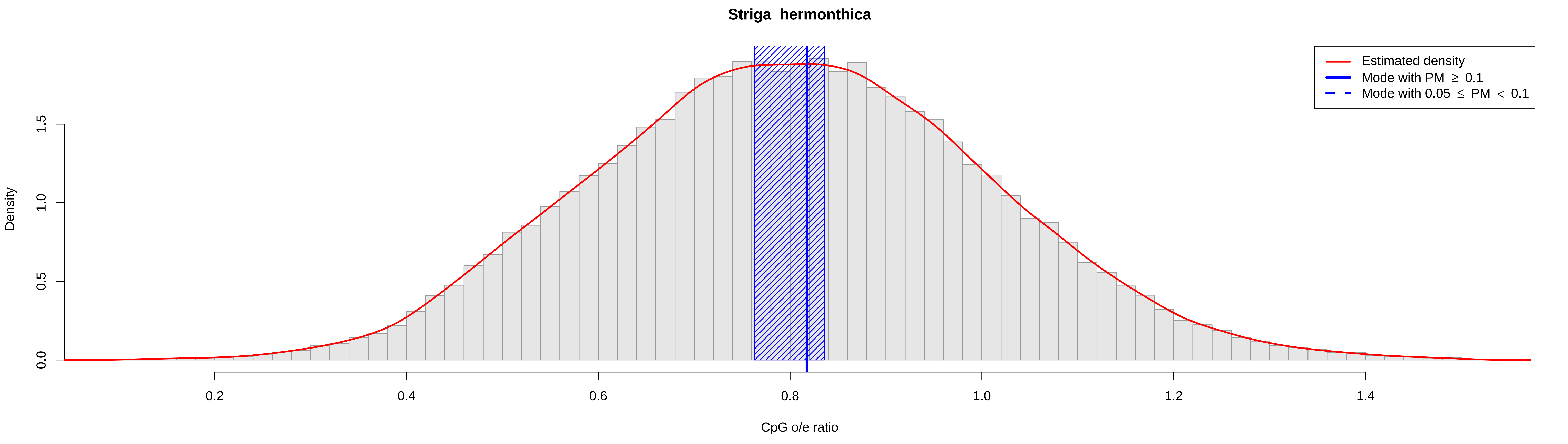
Striga_hermonthica
Estimated density
Mode with PM ≥ 0.1
Mode with 0.05 ≤ PM < 0.1
Density
0.0
0.5
1.0
1.5
0.2
0.4
0.6
0.8
1.0
1.2
1.4
CpG o/e ratio

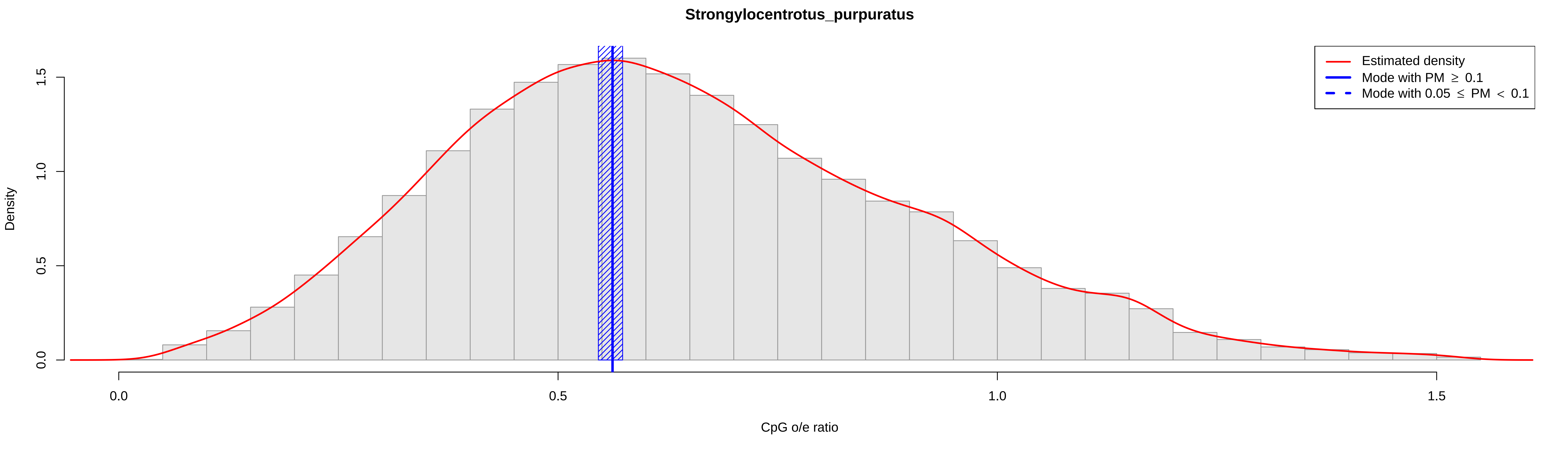

Strongylocentrotus_purpuratus
Estimated density
Mode with PM ≥ 0.1
Mode with 0.05 ≤ PM < 0.1
Density
0.0
0.5
1.0
1.5
0.0
0.5
1.0
1.5
CpG o/e ratio

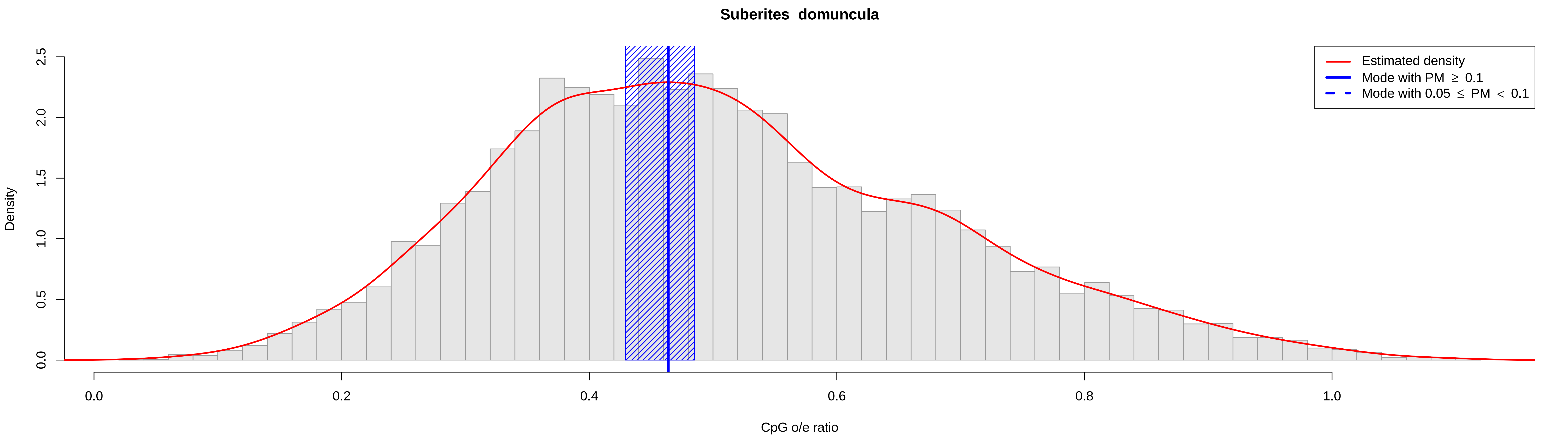
Suberites_domuncula
Estimated density
Mode with PM ≥ 0.1
Mode with 0.05 ≤ PM < 0.1
Density
0.0
0.5
1.0
1.5
2.0
2.5
0.0
0.2
0.4
0.6
0.8
1.0
CpG o/e ratio

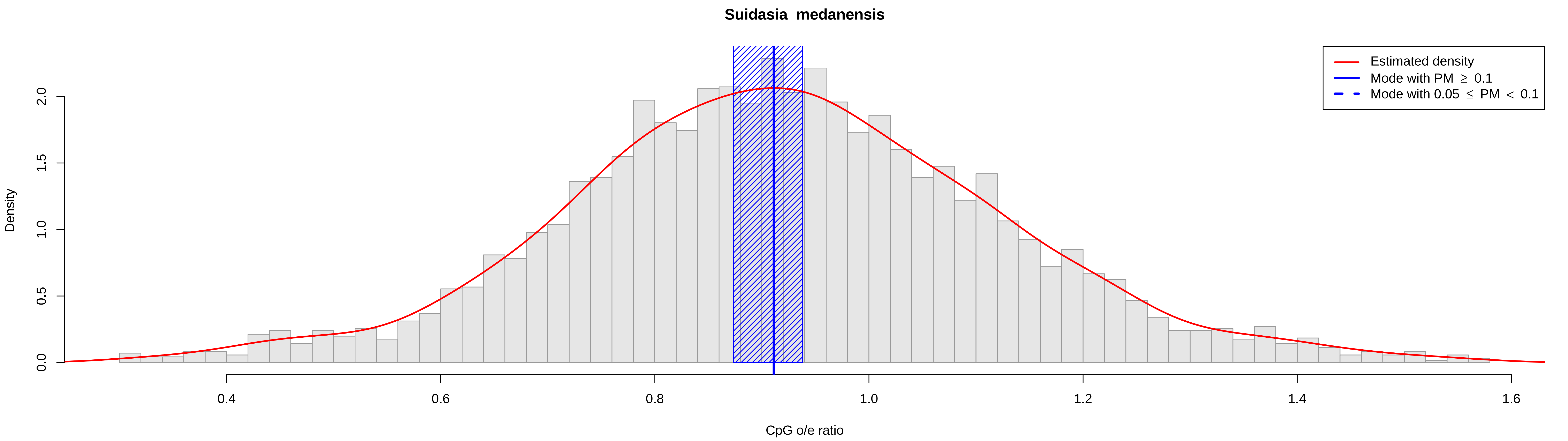

Suidasia_medanensis
Estimated density
Mode with PM ≥ 0.1
Mode with 0.05 ≤ PM < 0.1
Density
0.0
0.5
1.0
1.5
2.0
0.4
0.6
0.8
1.0
1.2
1.4
1.6
CpG o/e ratio

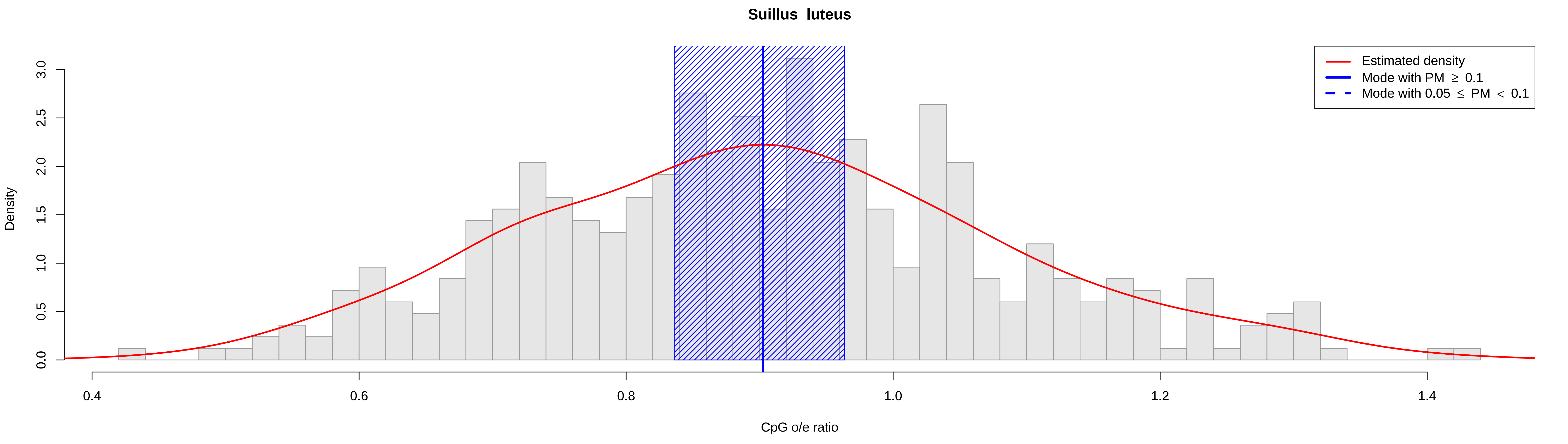

Suillus_luteus
Estimated density
Mode with PM ≥ 0.1
Mode with 0.05 ≤ PM < 0.1
Density
0.0
0.5
1.0
1.5
2.0
2.5
3.0
0.4
0.6
0.8
1.0
1.2
1.4
CpG o/e ratio

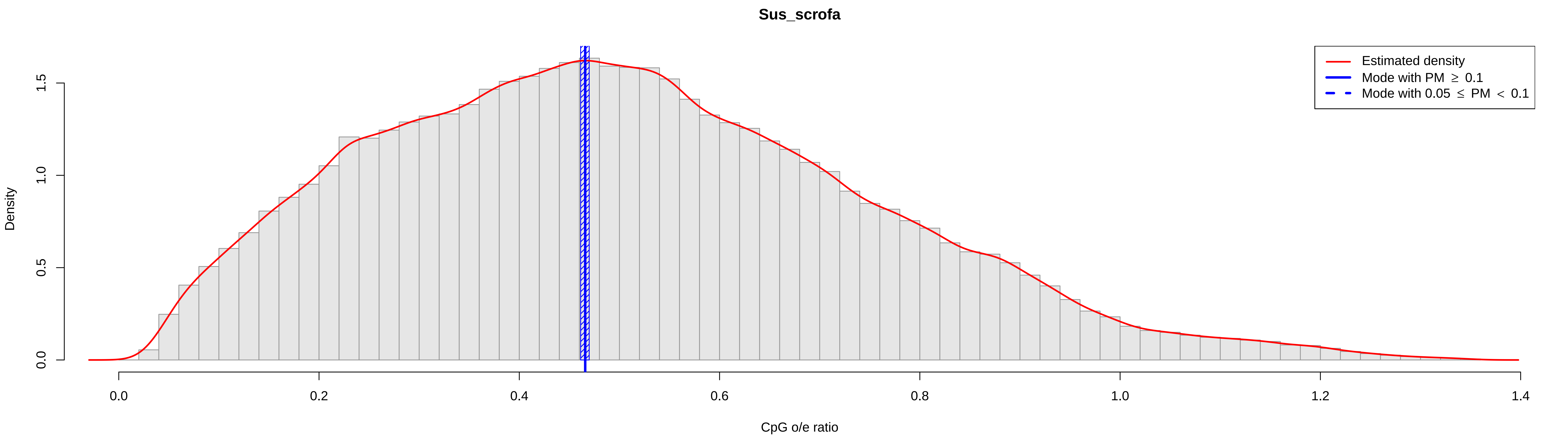

Sus_scrofa
Estimated density
Mode with PM ≥ 0.1
Mode with 0.05 ≤ PM < 0.1
Density
0.0
0.5
1.0
1.5
0.0
0.2
0.4
0.6
0.8
1.0
1.2
1.4
CpG o/e ratio

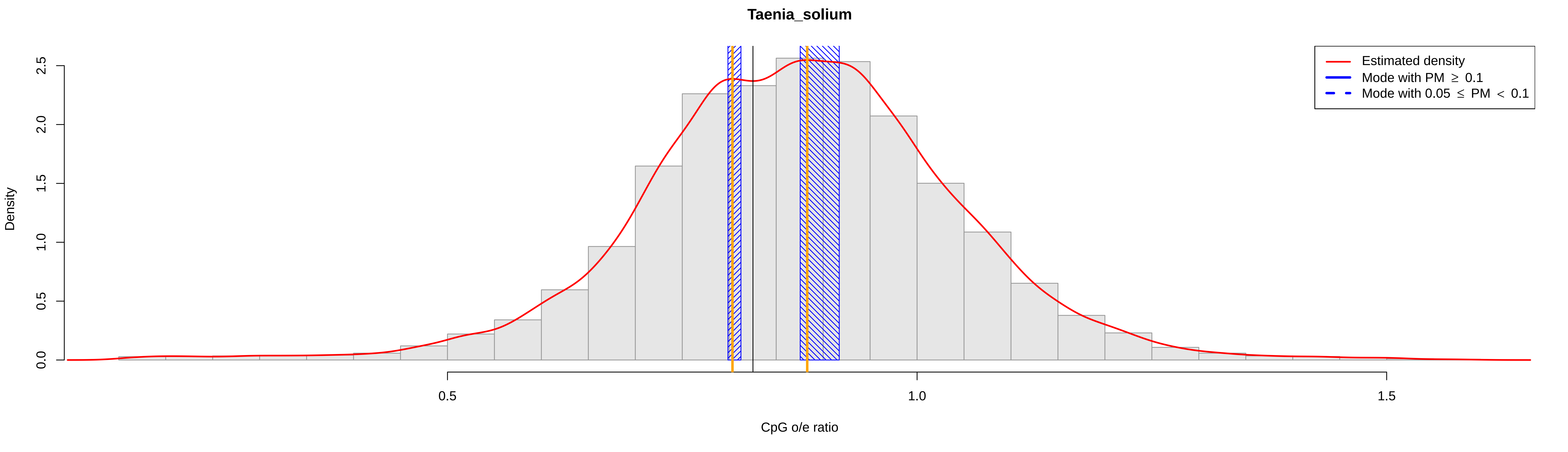
Taenia_solium
Estimated density
Mode with PM ≥ 0.1
Mode with 0.05 ≤ PM < 0.1
Density
0.0
0.5
1.0
1.5
2.0
2.5
0.5
1.0
1.5
CpG o/e ratio

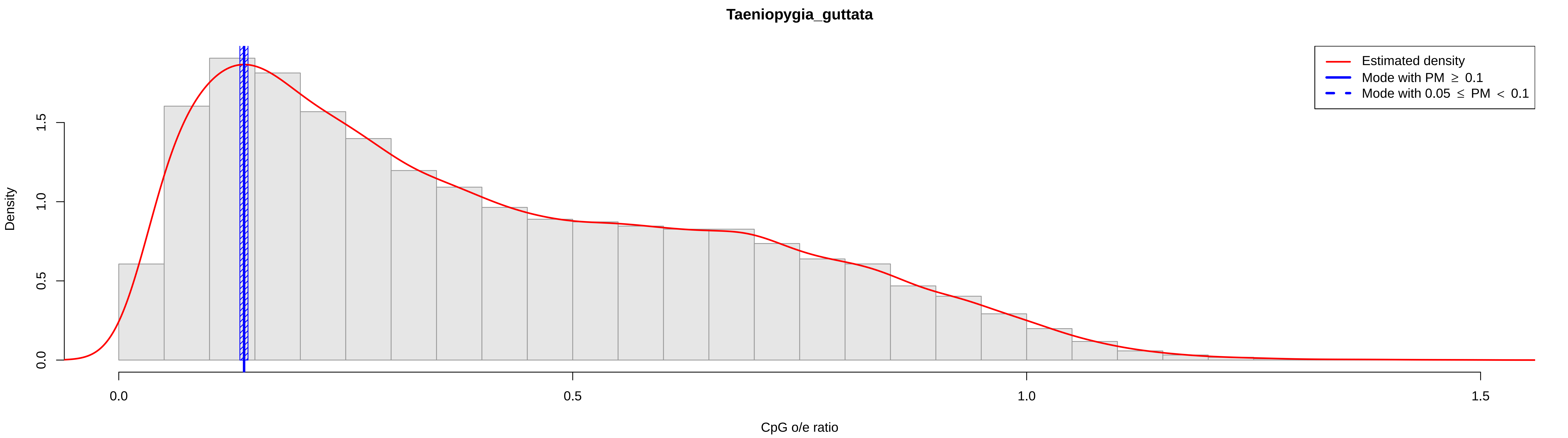
Taeniopygia_guttata
Estimated density
Mode with PM ≥ 0.1
Mode with 0.05 ≤ PM < 0.1
Density
0.0
0.5
1.0
1.5
CpG o/e ratio

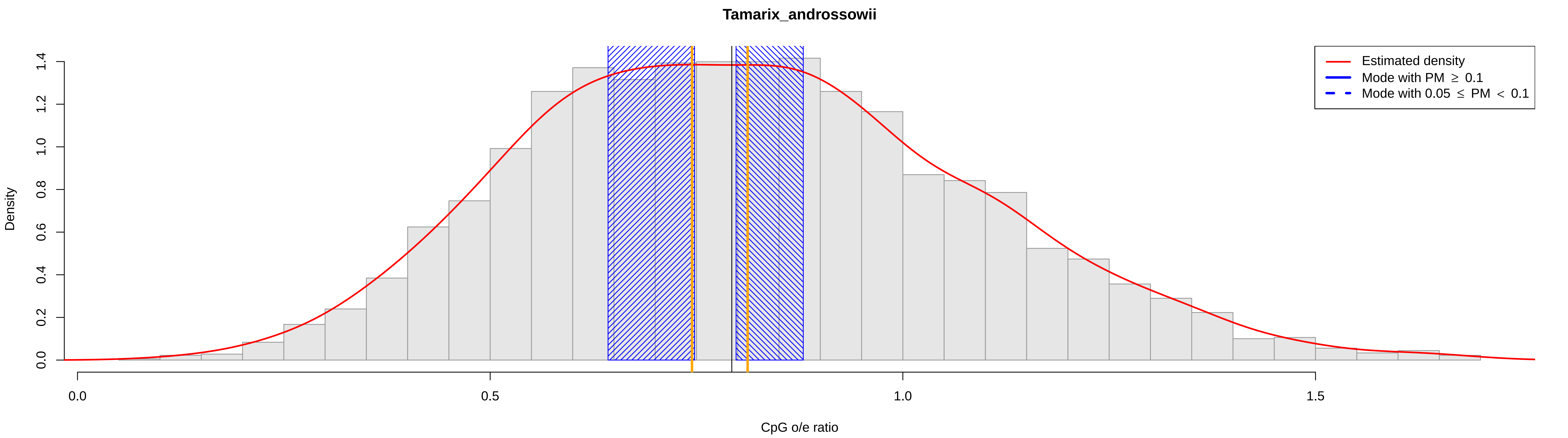

Tamarix_androssowii
Estimated density
Mode with PM ≥ 0.1
Mode with 0.05 ≤ PM < 0.1
Density
0.0
0.2
0.4
0.6
0.8
1.0
1.2
1.4
0.0
0.5
1.0
1.5
CpG o/e ratio

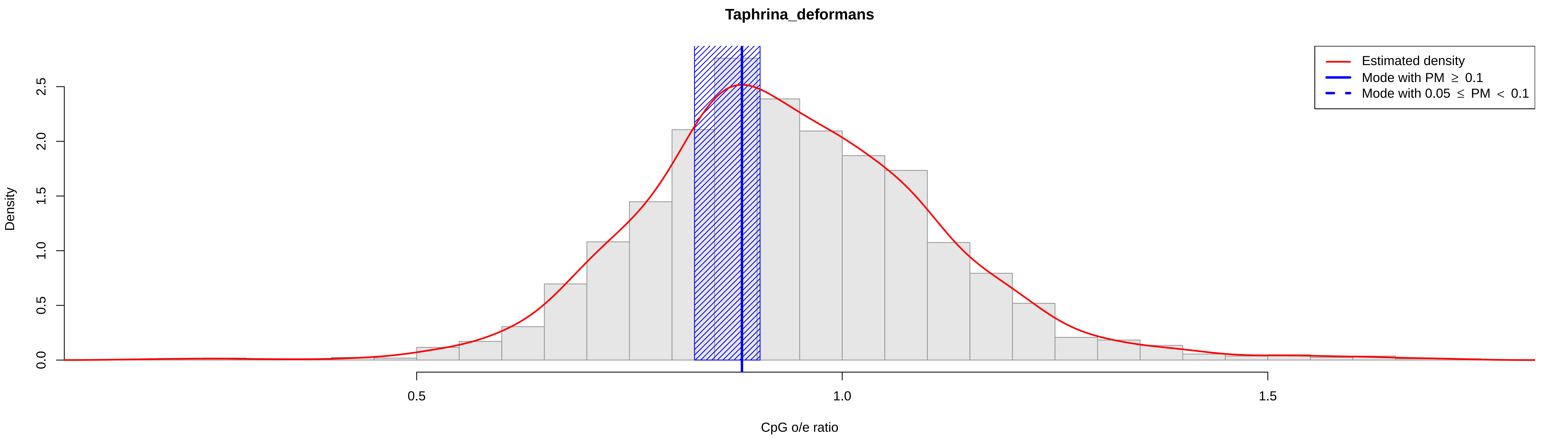

Taphrina_deformans
Estimated density
Mode with PM ≥ 0.1
Mode with 0.05 ≤ PM < 0.1
Density
0.0
0.5
1.0
1.5
2.0
2.5
0.5
1.0
1.5
CpG o/e ratio

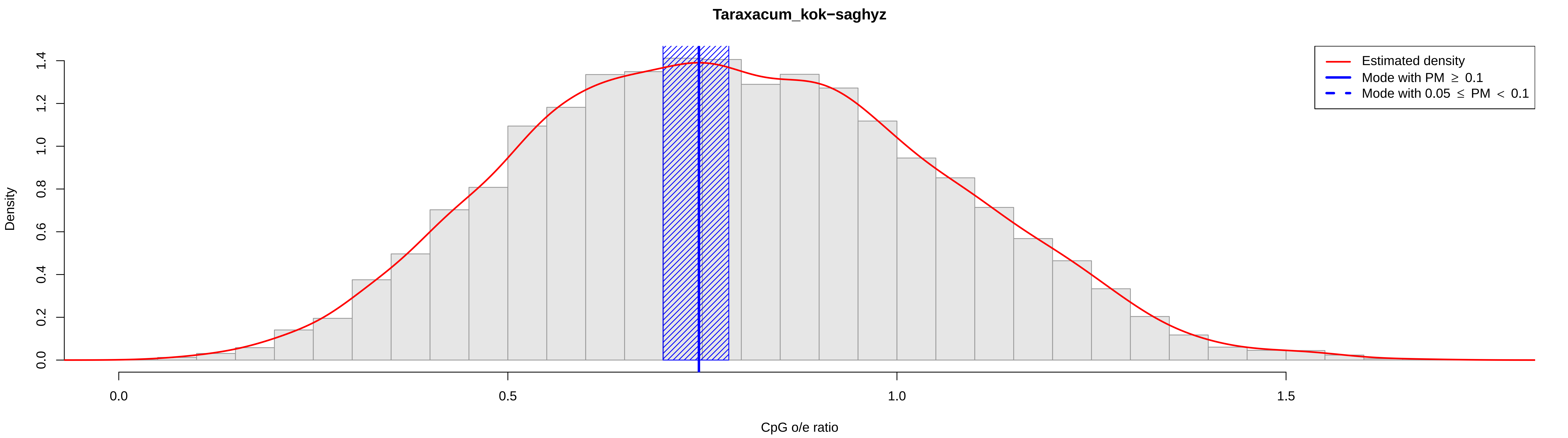
Taraxacum_kok−saghyz
Estimated density
Mode with PM ≥ 0.1
Mode with 0.05 ≤ PM < 0.1
Density
0.0
0.2
0.4
0.6
0.8
1.0
1.2
1.4
0.0
0.5
1.0
1.5
CpG o/e ratio

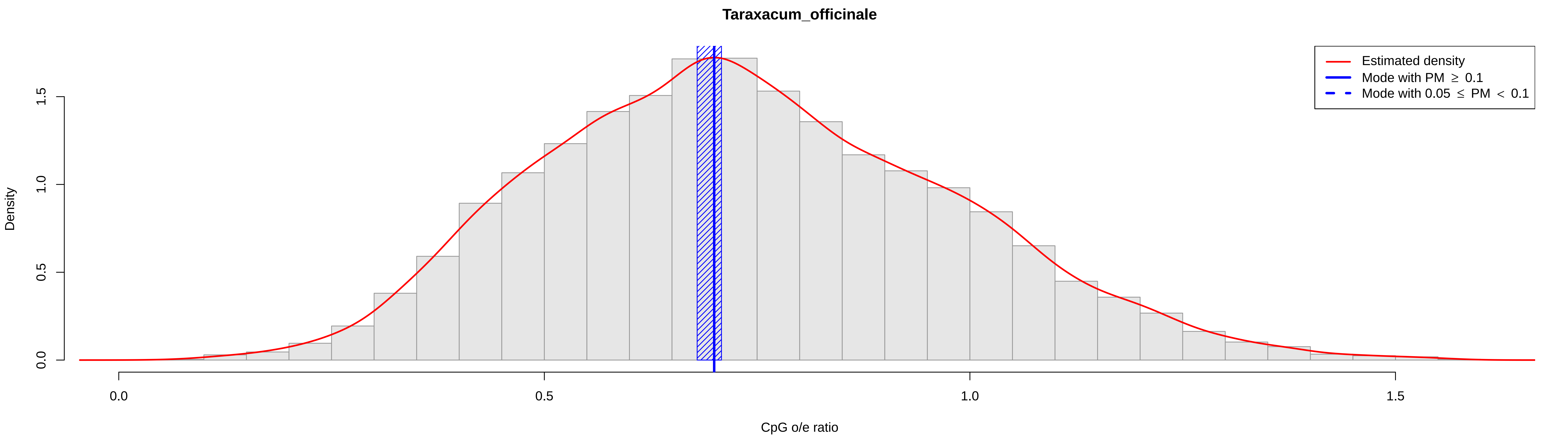
Taraxacum_officinale
Estimated density
Mode with PM ≥ 0.1
Mode with 0.05 ≤ PM < 0.1
Density
0.0
0.5
1.0
1.5
0.0
0.5
1.0
1.5
CpG o/e ratio

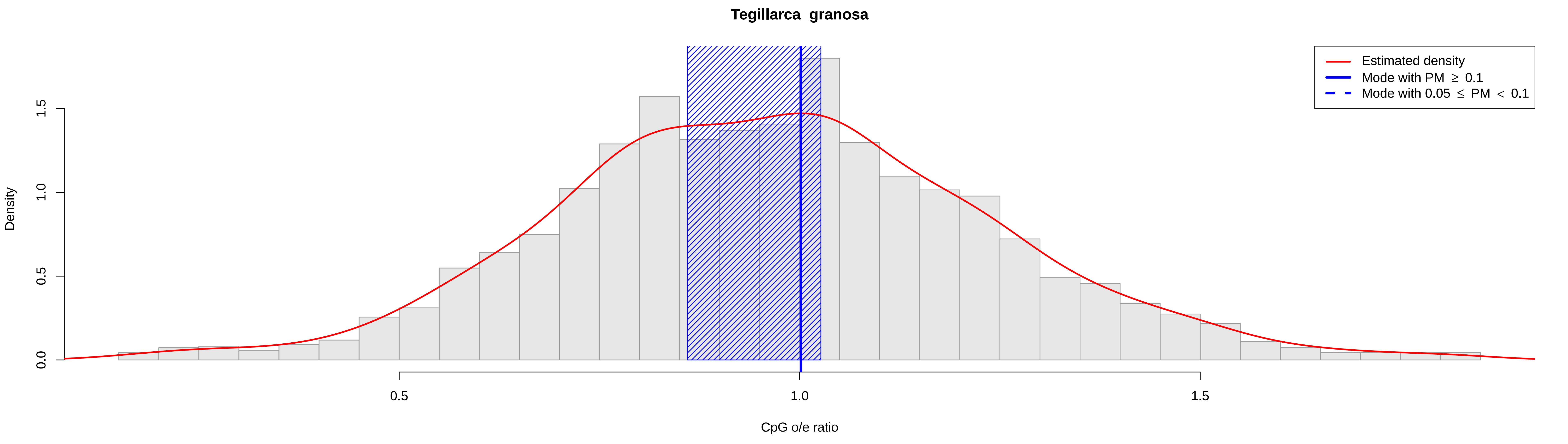

Tegillarca_granosa
Estimated density
Mode with PM ≥ 0.1
Mode with 0.05 ≤ PM < 0.1
Density
0.0
0.5
1.0
1.5
0.5
1.0
1.5
CpG o/e ratio

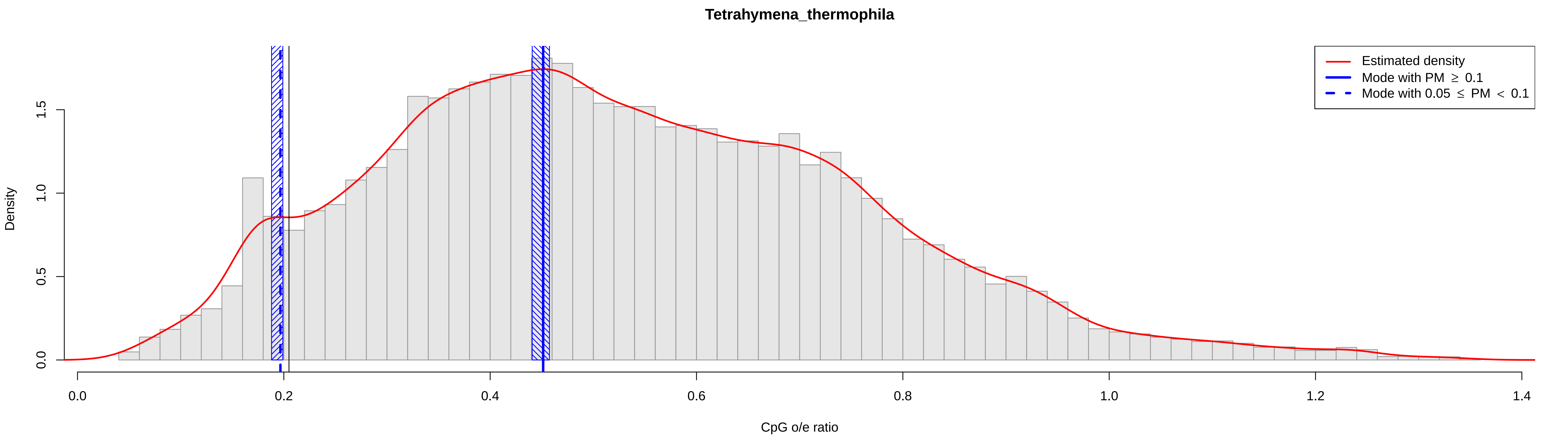

Tetrahymena_thermophila
Estimated density
Mode with PM ≥ 0.1
Mode with 0.05 ≤ PM < 0.1
Density
0.0
0.5
1.0
1.5
0.0
0.2
0.4
0.6
0.8
1.0
1.2
1.4
CpG o/e ratio

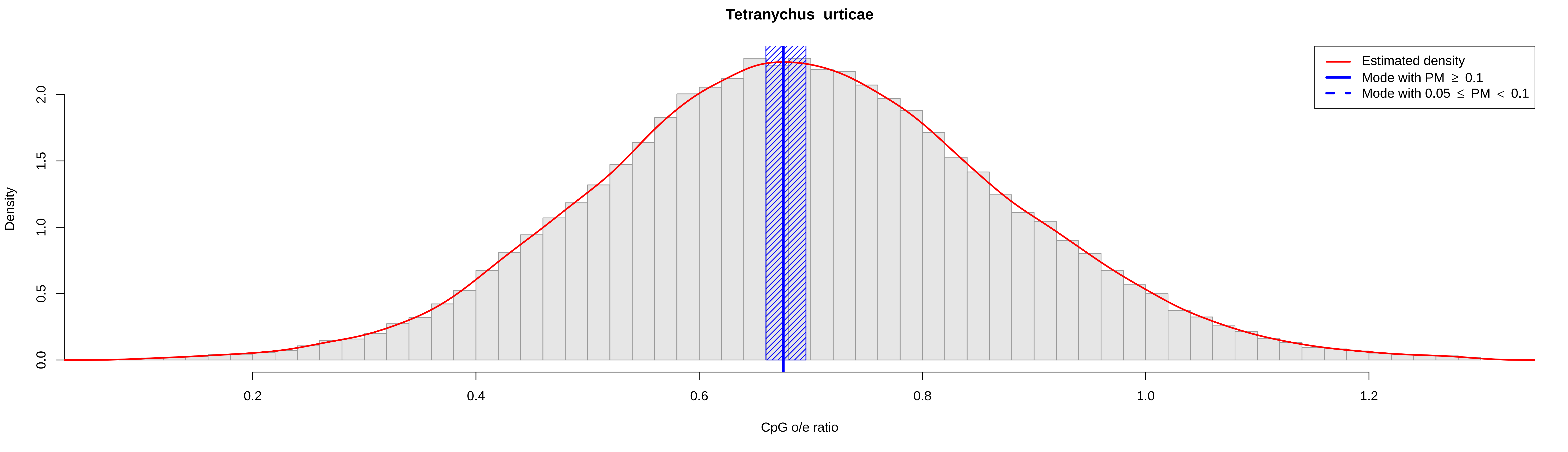
Tetranychus_urticae
Estimated density
Mode with PM ≥ 0.1
Mode with 0.05 ≤ PM < 0.1
Density
0.0
0.5
1.0
1.5
2.0
0.2
0.4
0.6
0.8
1.0
1.2
CpG o/e ratio

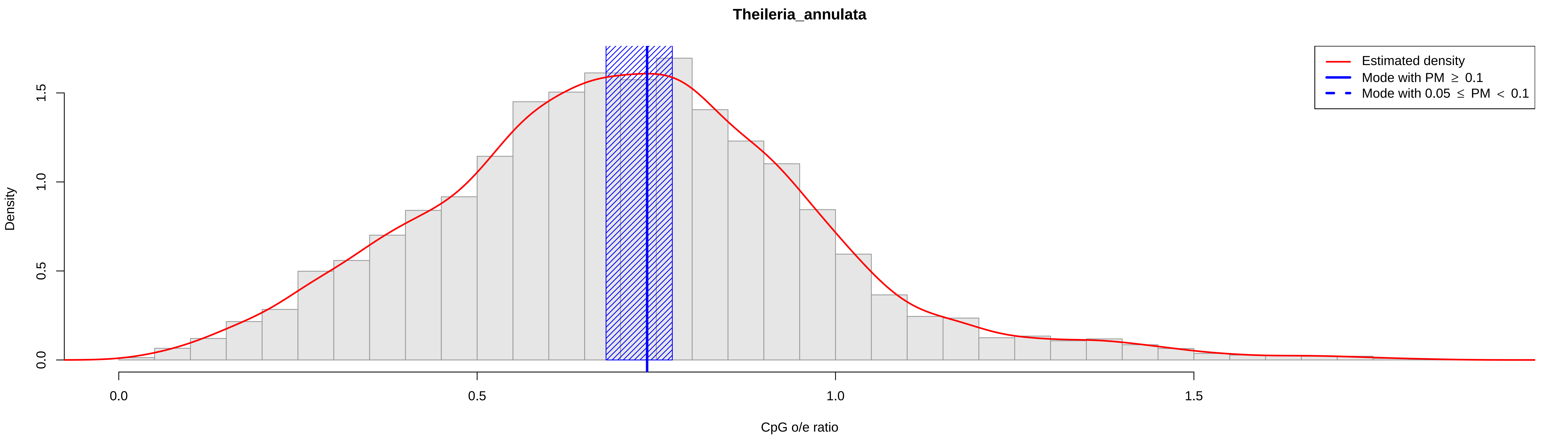

Theileria_annulata
Estimated density
Mode with PM ≥ 0.1
Mode with 0.05 ≤ PM < 0.1
Density
0.0
0.5
1.0
1.5
CpG o/e ratio

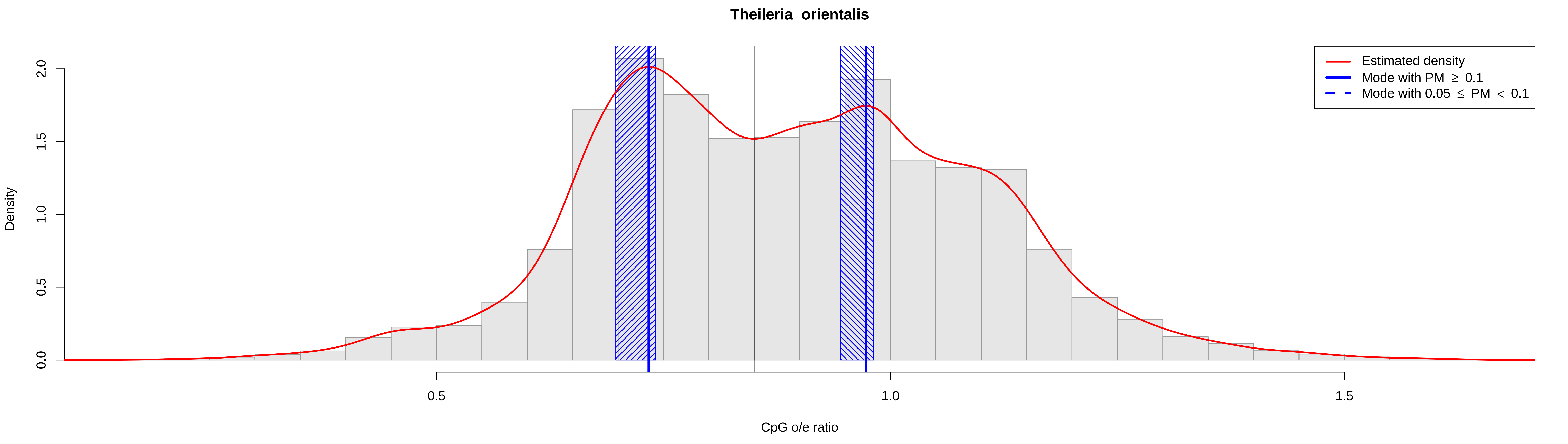

Theileria_orientalis
Estimated density
Mode with PM ≥ 0.1
Mode with 0.05 ≤ PM < 0.1
Density
0.0
0.5
1.0
1.5
2.0
0.5
1.0
1.5
CpG o/e ratio

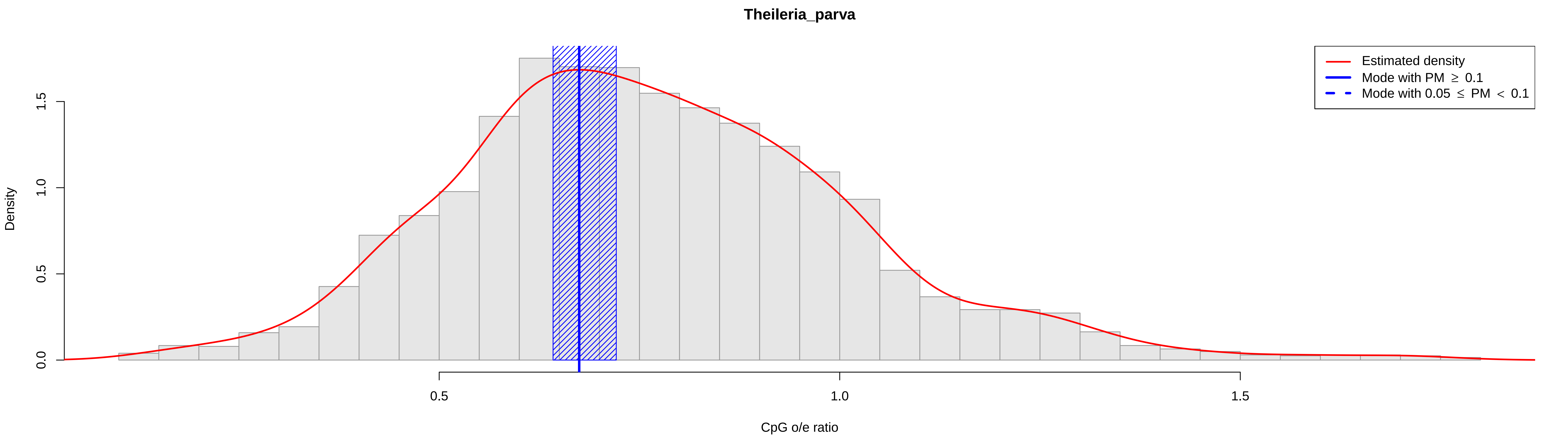

Theileria_parva
Estimated density
Mode with PM ≥ 0.1
Mode with 0.05 ≤ PM < 0.1
Density
0.0
0.5
1.0
1.5
0.5
1.0
1.5
CpG o/e ratio

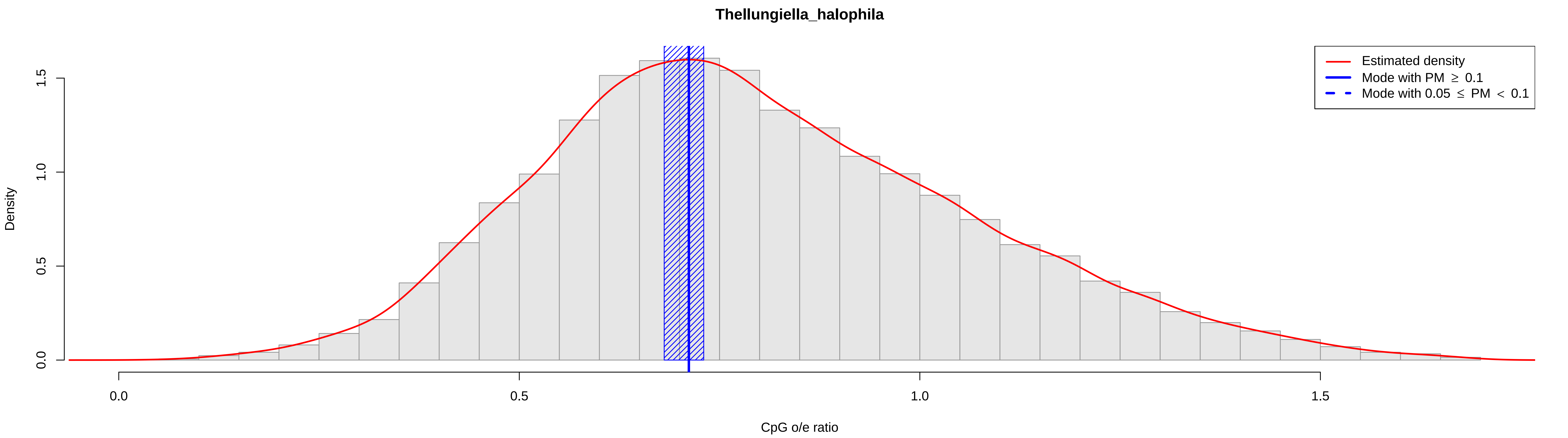

Thellungiella_halophila
Estimated density
Mode with PM ≥ 0.1
Mode with 0.05 ≤ PM < 0.1
Density
0.0
0.5
1.0
1.5
0.0
0.5
1.0
1.5
CpG o/e ratio

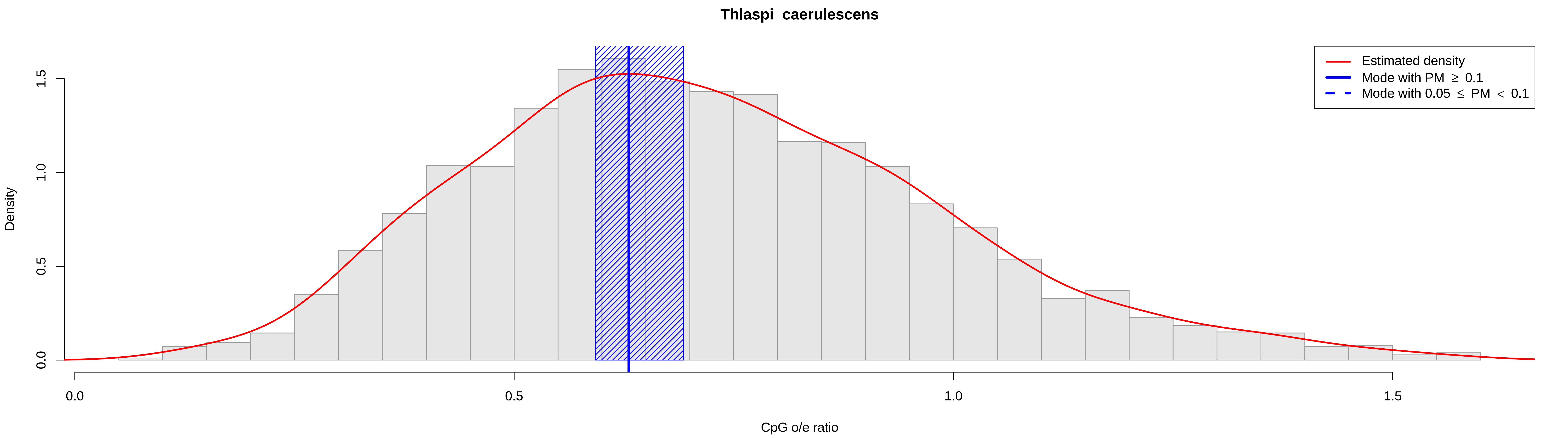

Thlaspi_caerulescens
Estimated density
Mode with PM ≥ 0.1
Mode with 0.05 ≤ PM < 0.1
Density
0.0
0.5
1.0
1.5
CpG o/e ratio

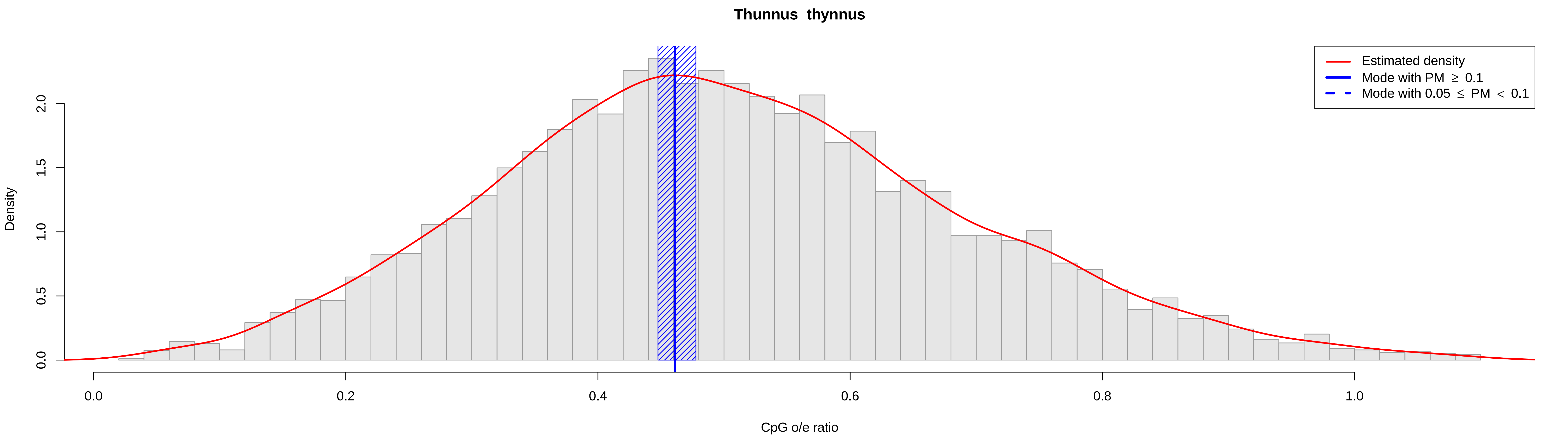

Thunnus_thynnus
Estimated density
Mode with PM ≥ 0.1
Mode with 0.05 ≤ PM < 0.1
Density
0.0
0.5
1.0
1.5
2.0
0.0
0.2
0.4
0.6
0.8
1.0
CpG o/e ratio

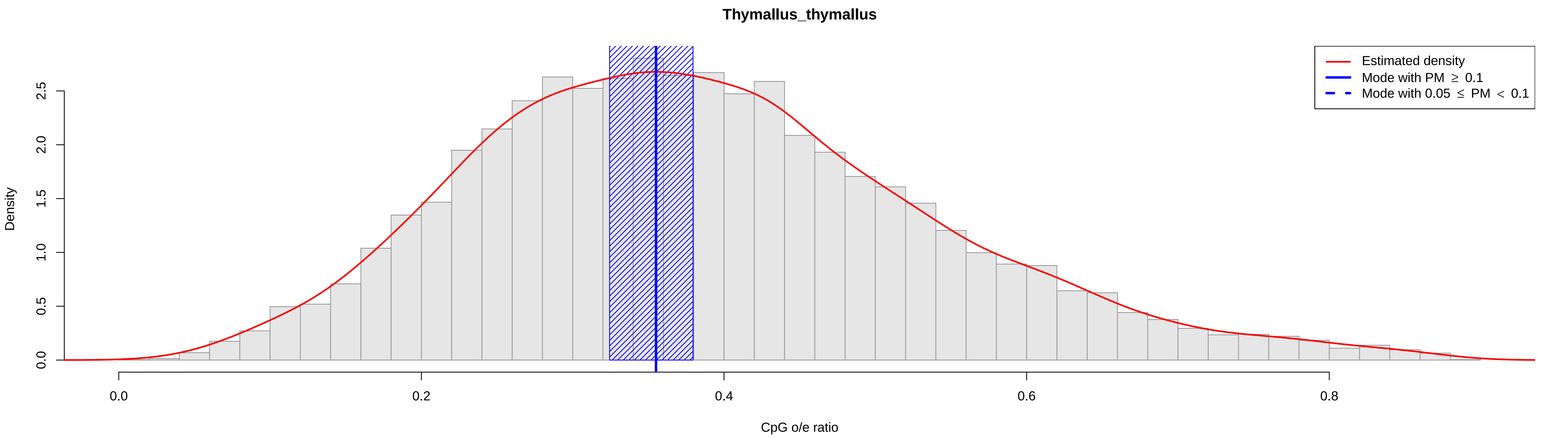

Thymallus_thymallus
Estimated density
Mode with PM ≥ 0.1
Mode with 0.05 ≤ PM < 0.1
Density
0.0
0.5
1.0
1.5
2.0
2.5
0.0
0.2
0.4
0.6
0.8
CpG o/e ratio

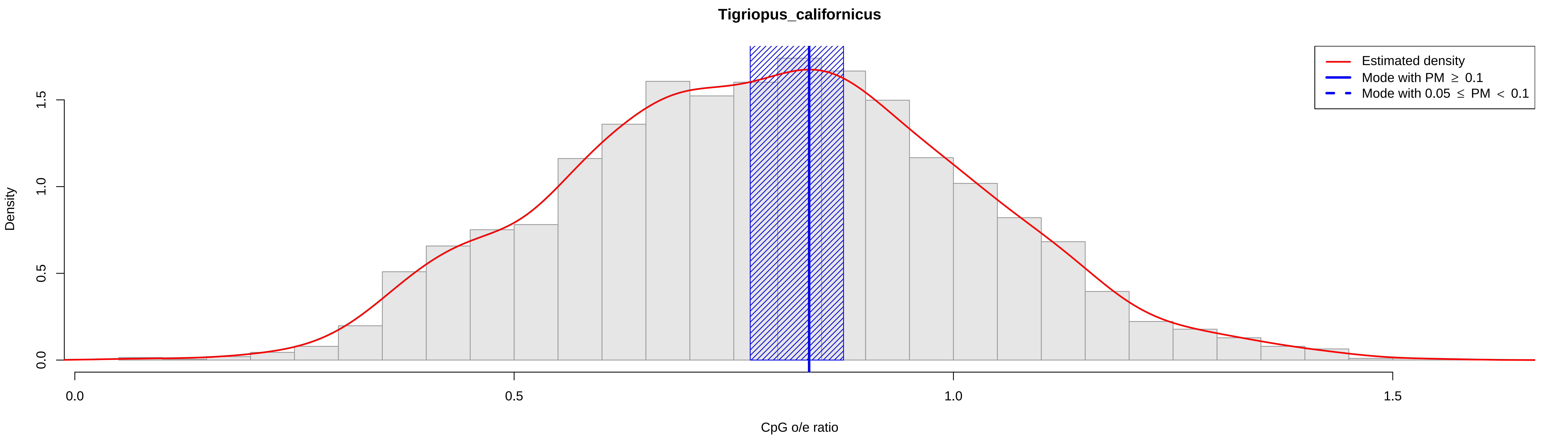

Tigriopus_californicus
Estimated density
Mode with PM ≥ 0.1
Mode with 0.05 ≤ PM < 0.1
Density
0.0
0.5
1.0
1.5
0.0
0.5
1.0
1.5
CpG o/e ratio

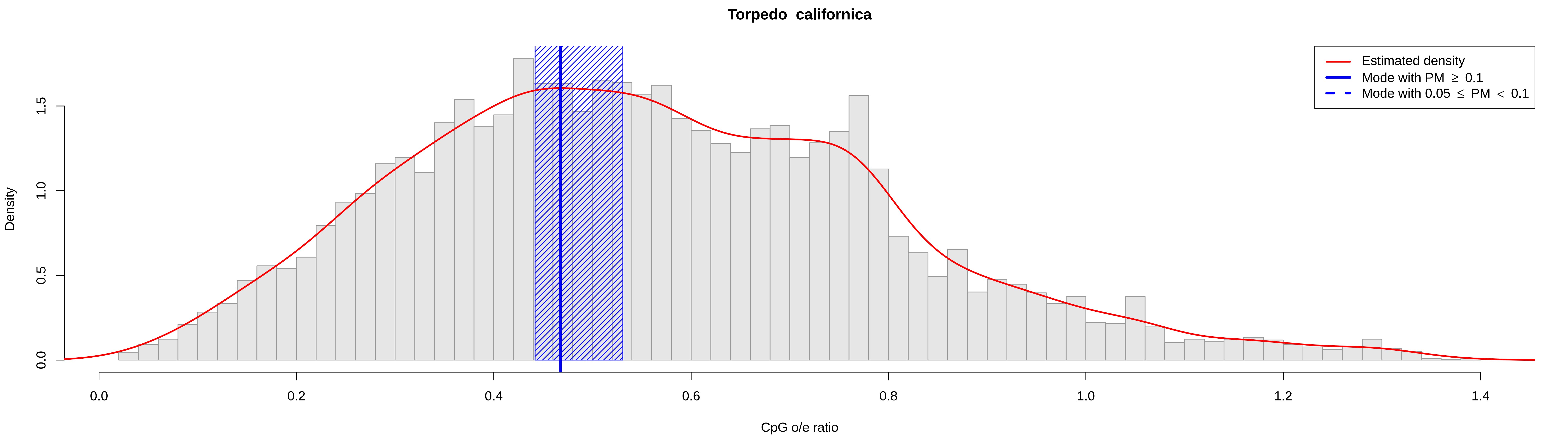
Torpedo_californica
Estimated density
Mode with PM ≥ 0.1
Mode with 0.05 ≤ PM < 0.1
Density
0.0
0.5
1.0
1.5
0.0
0.2
0.4
0.6
0.8
1.0
1.2
1.4
CpG o/e ratio

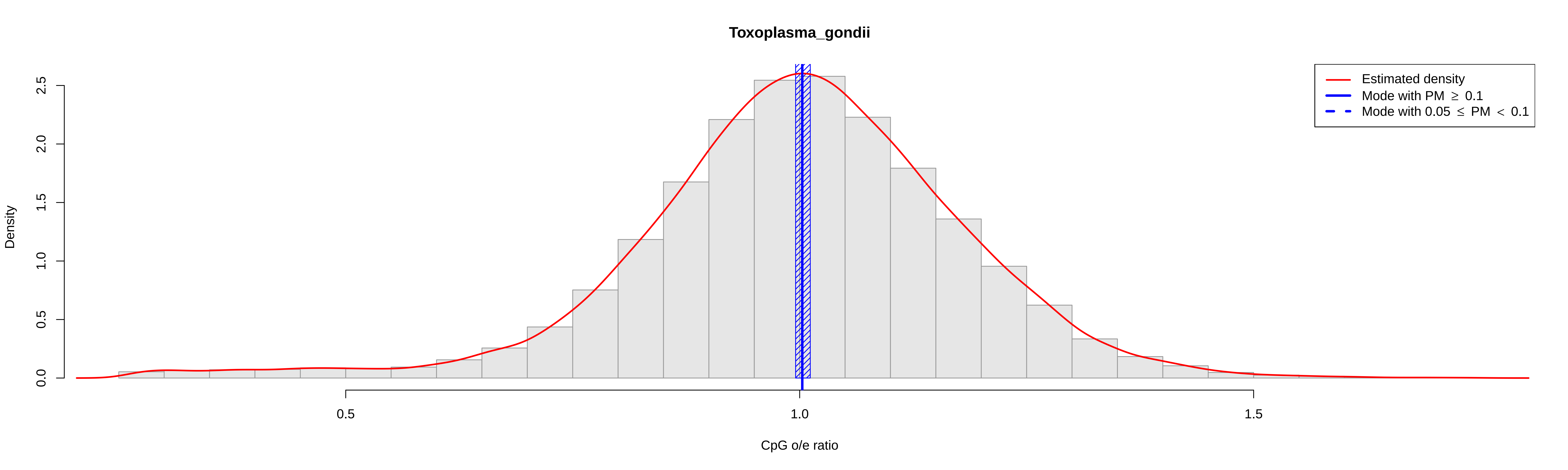

Toxoplasma_gondii
Density
0.0
0.5
1.0
1.5
2.0
2.5
0.5
1.0
1.5
CpG o/e ratio
Estimated density
Mode with PM ≥ 0.1
Mode with 0.05 ≤ PM < 0.1

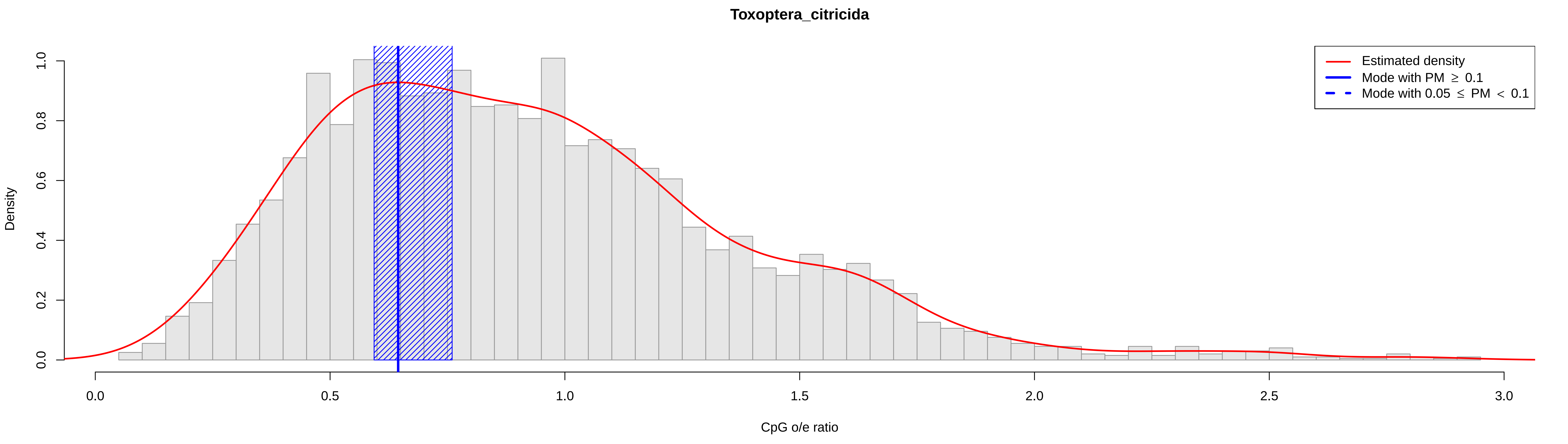

Toxoptera_citricida
Estimated density
Mode with PM ≥ 0.1
Mode with 0.05 ≤ PM < 0.1
Density
CpG o/e ratio
0.0
0.2
0.4
0.6
0.8
1.0
0.0
0.5
1.0
1.5
2.0
2.5
3.0

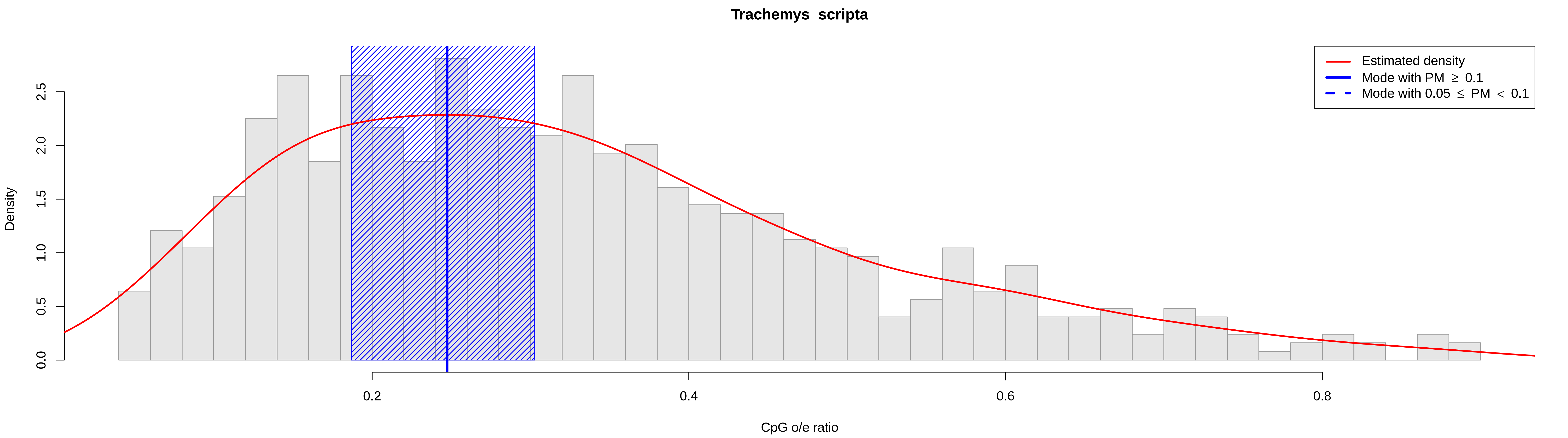

Trachemys_scripta
Estimated density
Mode with PM ≥ 0.1
Mode with 0.05 ≤ PM < 0.1
Density
0.0
0.5
1.0
1.5
2.0
2.5
0.2
0.4
0.6
0.8
CpG o/e ratio

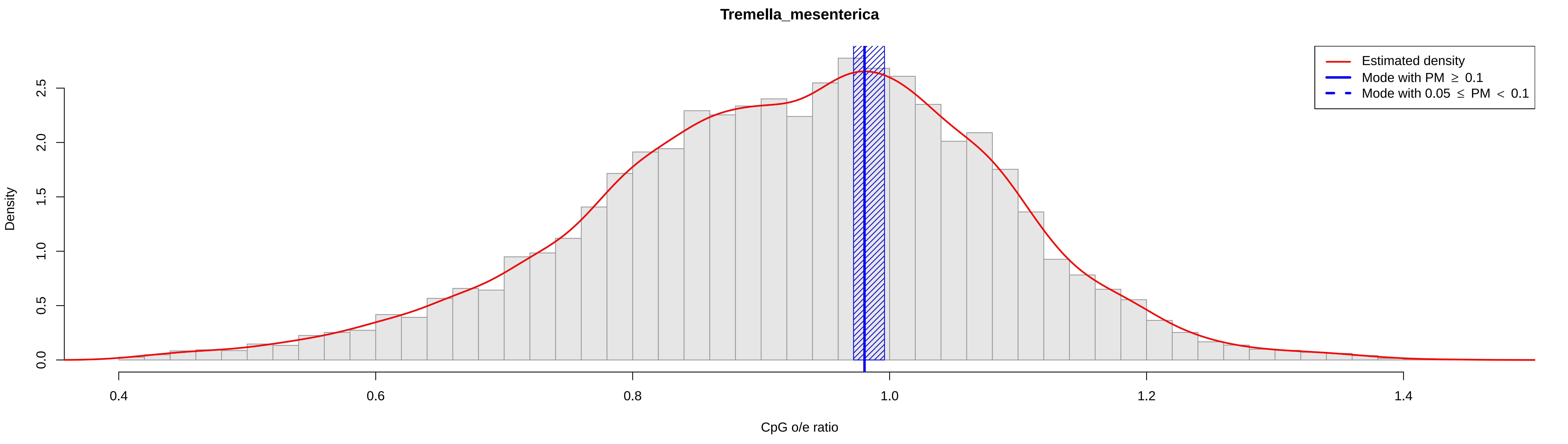
Tremella_mesenterica
Estimated density
Mode with PM ≥ 0.1
Mode with 0.05 ≤ PM < 0.1
Density
0.0
0.5
1.0
1.5
2.0
2.5
0.4
0.6
0.8
1.0
1.2
1.4
CpG o/e ratio

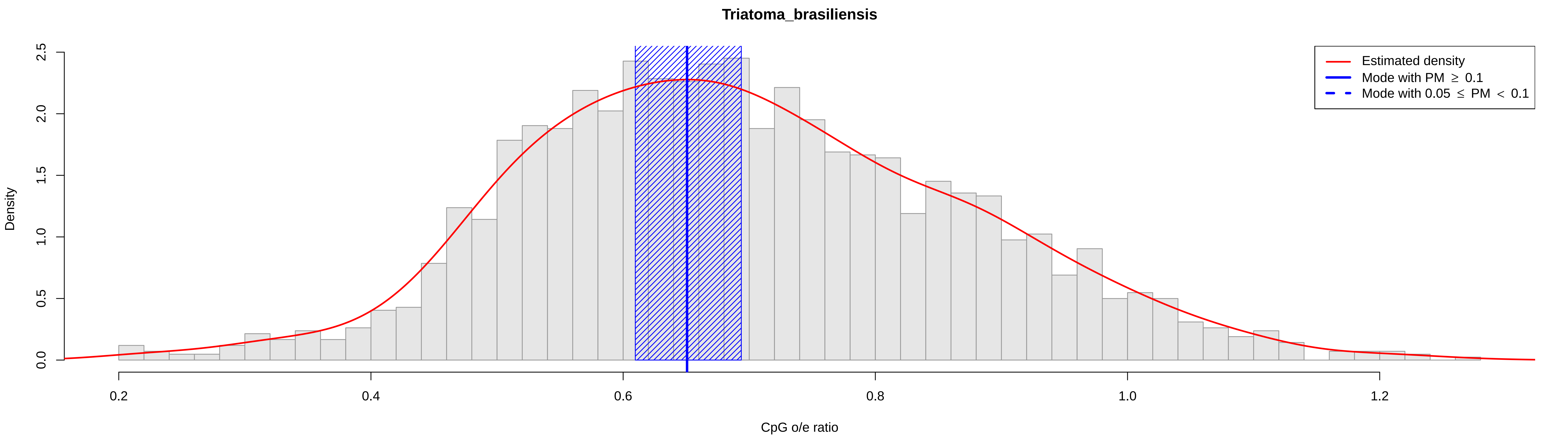

Triatoma_brasiliensis
Estimated density
Mode with PM ≥ 0.1
Mode with 0.05 ≤ PM < 0.1
Density
0.0
0.5
1.0
1.5
2.0
2.5
0.2
0.4
0.6
0.8
1.0
1.2
CpG o/e ratio

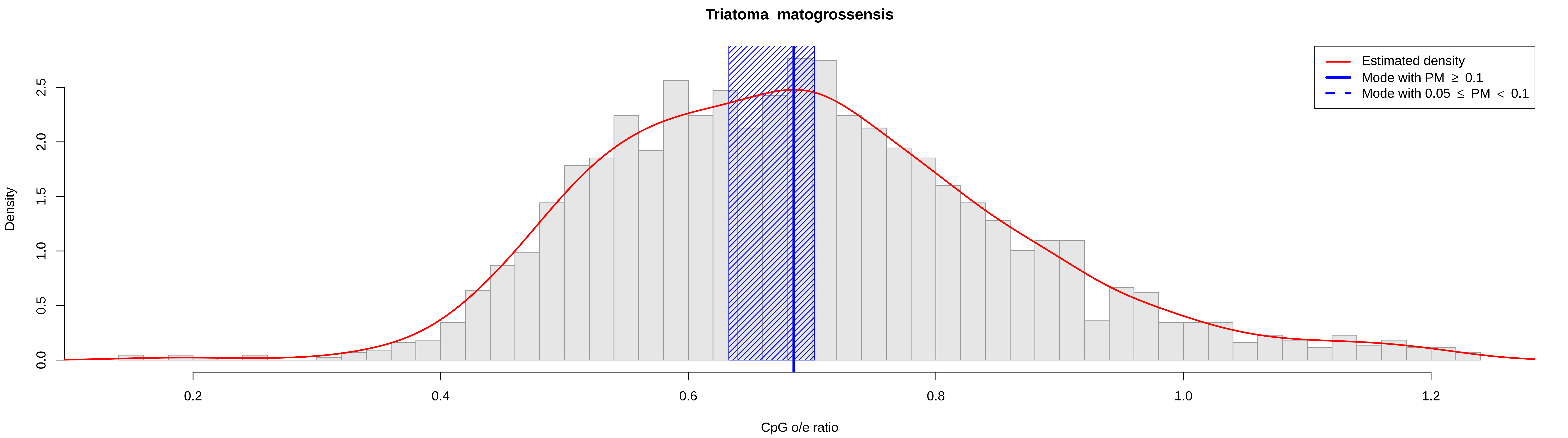
Triatoma_matogrossensis
Estimated density
Mode with PM ≥ 0.1
Mode with 0.05 ≤ PM < 0.1
Density
0.0
0.5
1.0
1.5
2.0
2.5
0.2
0.4
0.6
0.8
1.0
1.2
CpG o/e ratio

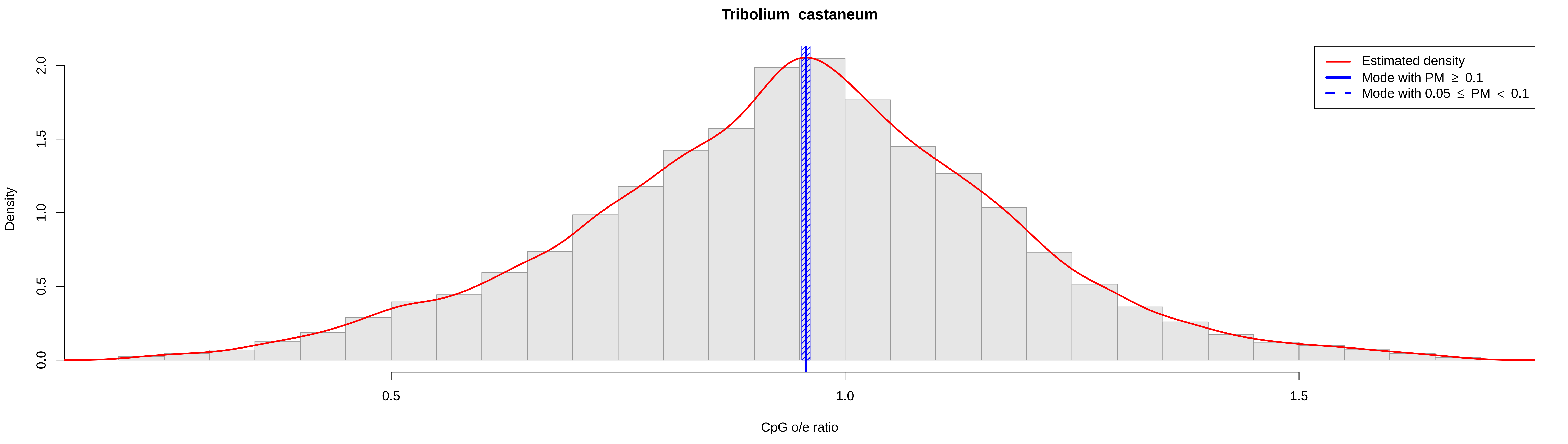
Tribolium_castaneum
Estimated density
Mode with PM ≥ 0.1
Mode with 0.05 ≤ PM < 0.1
Density
0.0
0.5
1.0
1.5
2.0
0.5
1.0
1.5
CpG o/e ratio

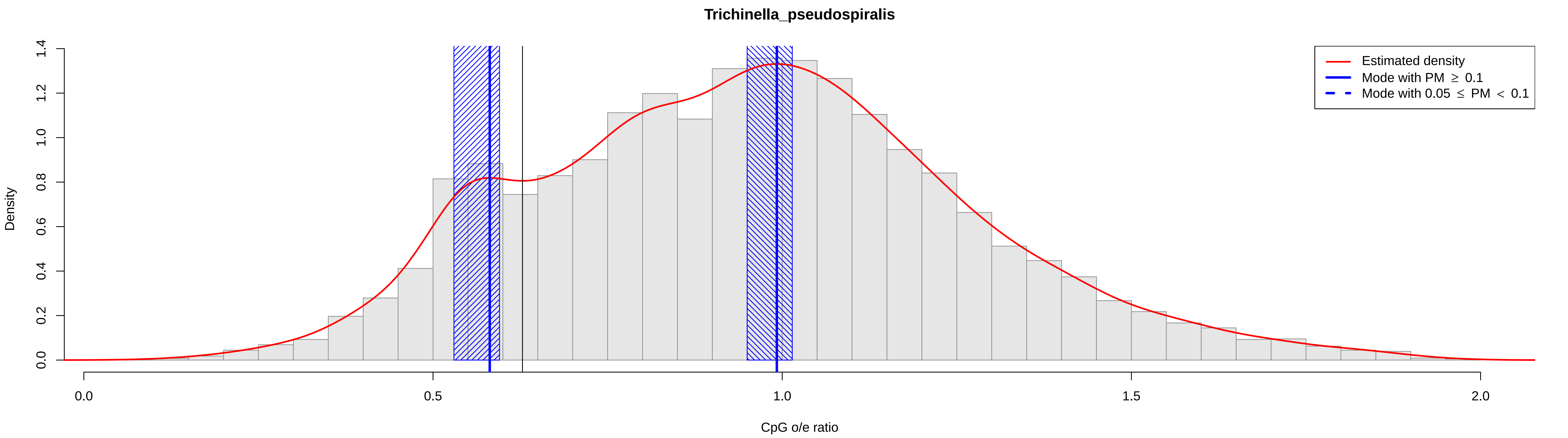

Trichinella_pseudospiralis
Estimated density
Mode with PM ≥ 0.1
Mode with 0.05 ≤ PM < 0.1
Density
0.0
0.2
0.4
0.6
0.8
1.0
1.2
1.4
0.0
0.5
1.0
1.5
2.0
CpG o/e ratio

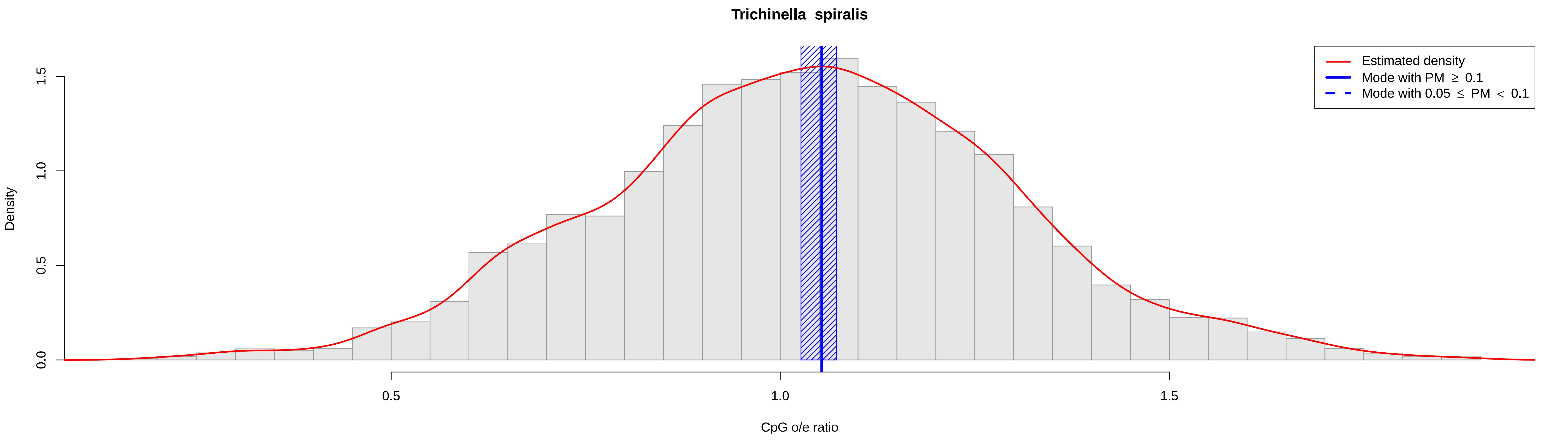

Trichinella_spiralis
Estimated density
Mode with PM ≥ 0.1
Mode with 0.05 ≤ PM < 0.1
Density
0.0
0.5
1.0
1.5
0.5
1.0
1.5
CpG o/e ratio

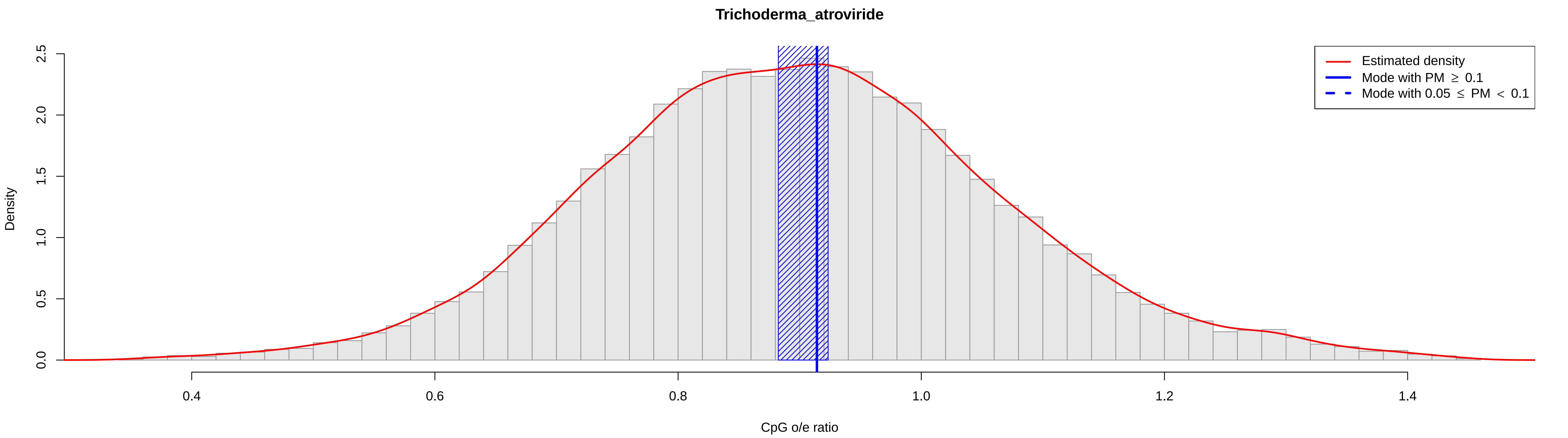
Trichoderma_atroviride
Estimated density
Mode with PM ≥ 0.1
Mode with 0.05 ≤ PM < 0.1
Density
0.0
0.5
1.0
1.5
2.0
2.5
0.4
0.6
0.8
1.0
1.2
1.4
CpG o/e ratio

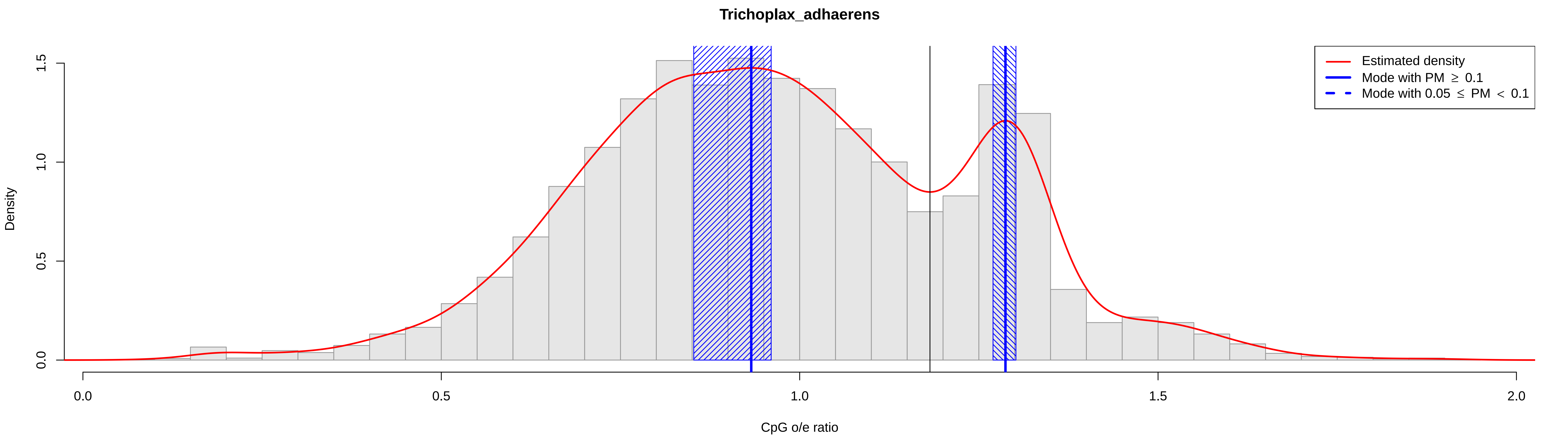

Trichoplax_adhaerens
Estimated density
Mode with PM ≥ 0.1
Mode with 0.05 ≤ PM < 0.1
Density
0.0
0.5
1.0
1.5
0.0
0.5
1.0
1.5
2.0
CpG o/e ratio

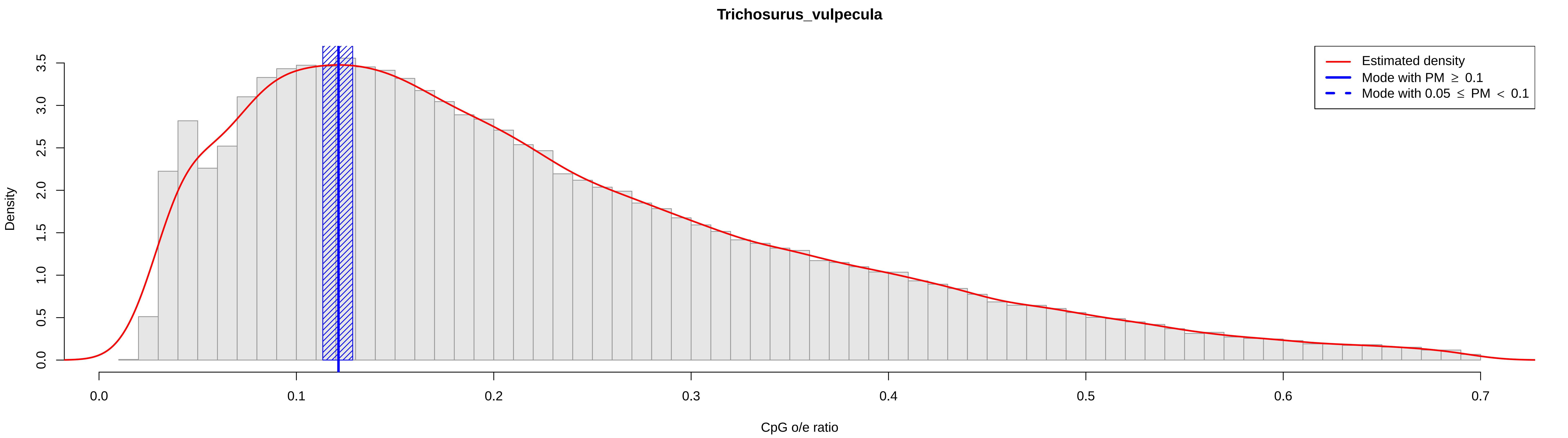
Trichosurus_vulpecula
Estimated density
Mode with PM ≥ 0.1
Mode with 0.05 ≤ PM < 0.1
Density
0.0
0.5
1.0
1.5
2.0
2.5
3.0
3.5
0.0
0.1
0.2
0.3
0.4
0.5
0.6
0.7
CpG o/e ratio

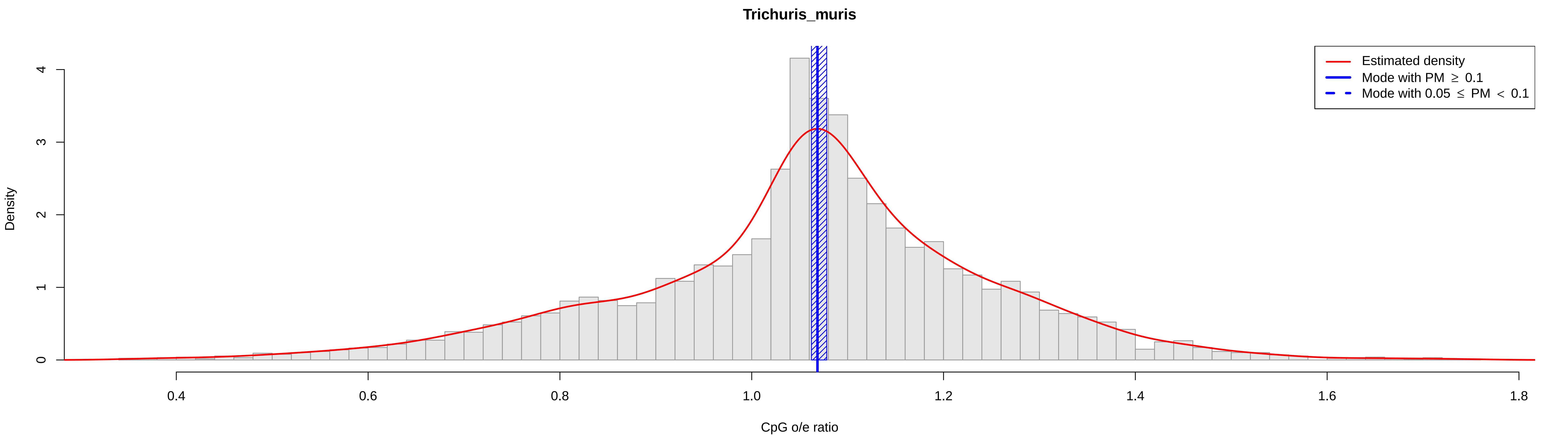
Trichuris_muris
Estimated density
Mode with PM ≥ 0.1
Mode with 0.05 ≤ PM < 0.1
Density
0
1
2
3
4
0.4
0.6
0.8
1.0
1.2
1.4
1.6
1.8
CpG o/e ratio

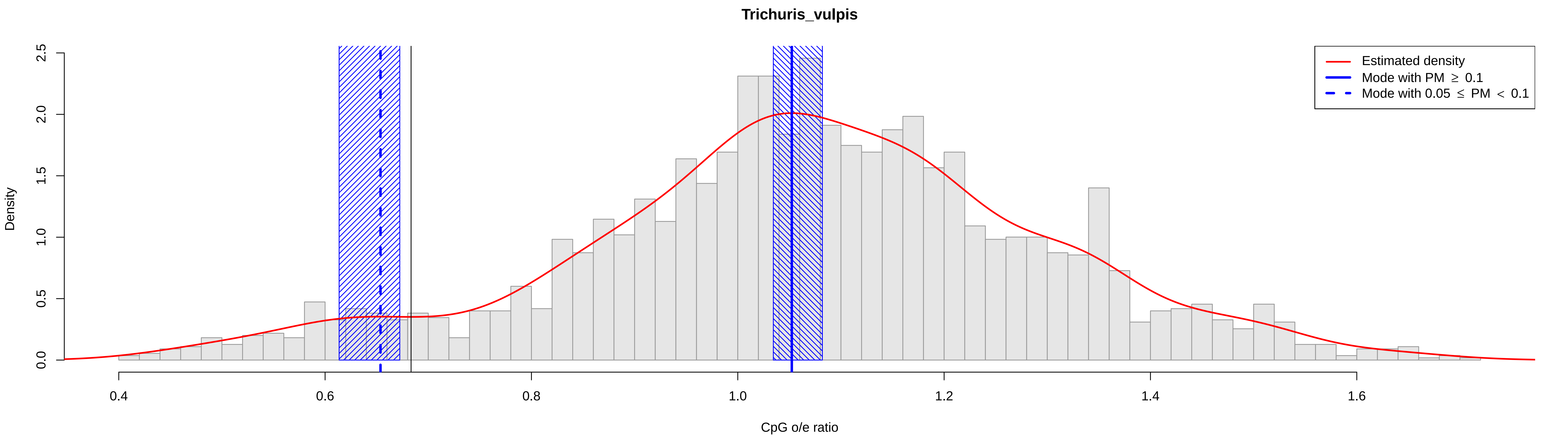

Trichuris_vulpis
Density
CpG o/e ratio
0.0
0.5
1.0
1.5
2.0
2.5
0.4
0.6
0.8
1.0
1.2
1.4
1.6
Estimated density
Mode with PM ≥ 0.1
Mode with 0.05 ≤ PM < 0.1

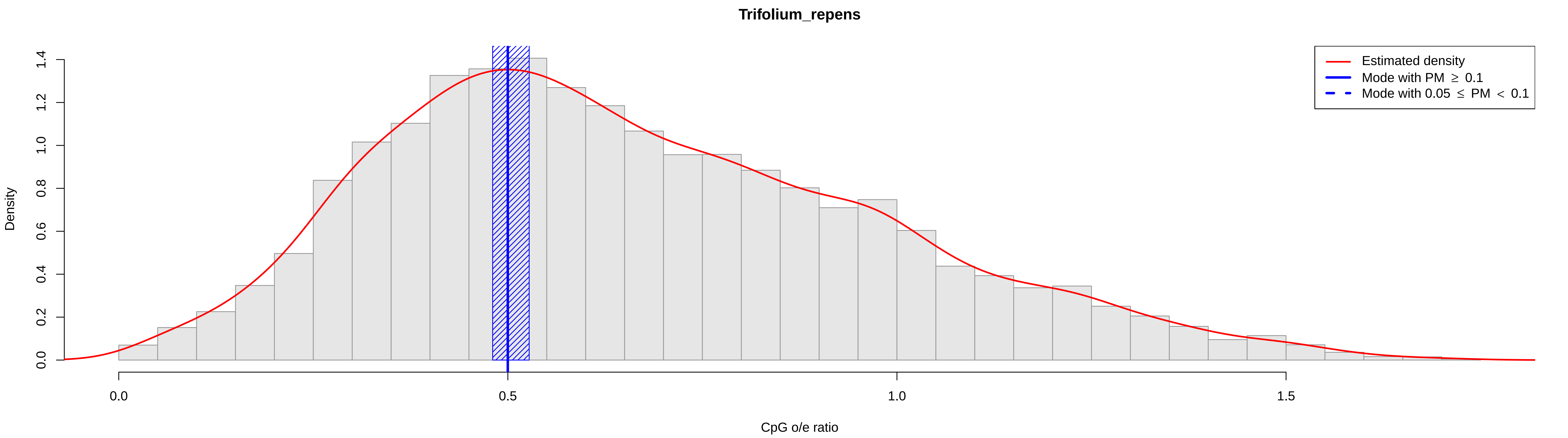
Trifolium_repens
Estimated density
Mode with PM ≥ 0.1
Mode with 0.05 ≤ PM < 0.1
Density
0.0
0.2
0.4
0.6
0.8
1.0
1.2
1.4
0.0
0.5
1.0
1.5
CpG o/e ratio

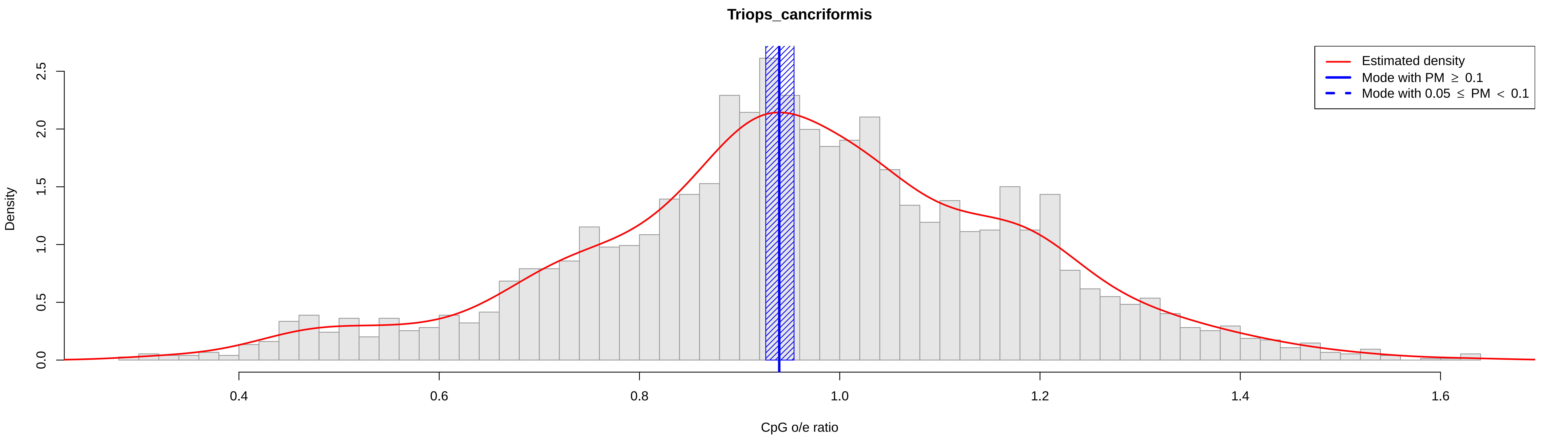
Triops_cancriformis
Estimated density
Mode with PM ≥ 0.1
Mode with 0.05 ≤ PM < 0.1
Density
0.0
0.5
1.0
1.5
2.0
2.5
0.4
0.6
0.8
1.0
1.2
1.4
1.6
CpG o/e ratio

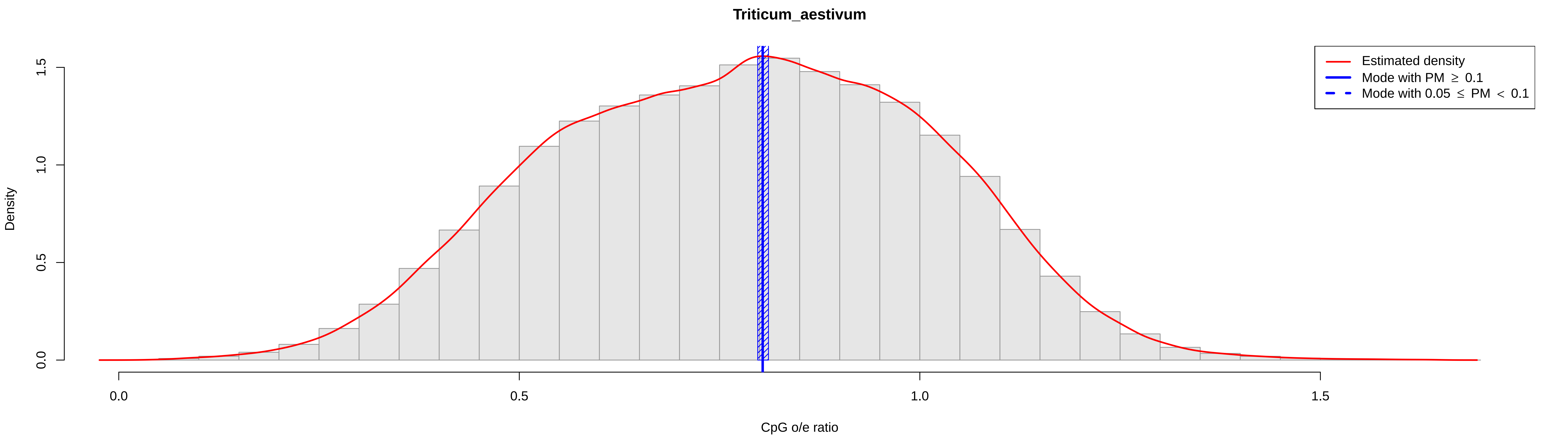
Triticum_aestivum
Estimated density
Mode with PM ≥ 0.1
Mode with 0.05 ≤ PM < 0.1
Density
CpG o/e ratio
0.0
0.5
1.0
1.5

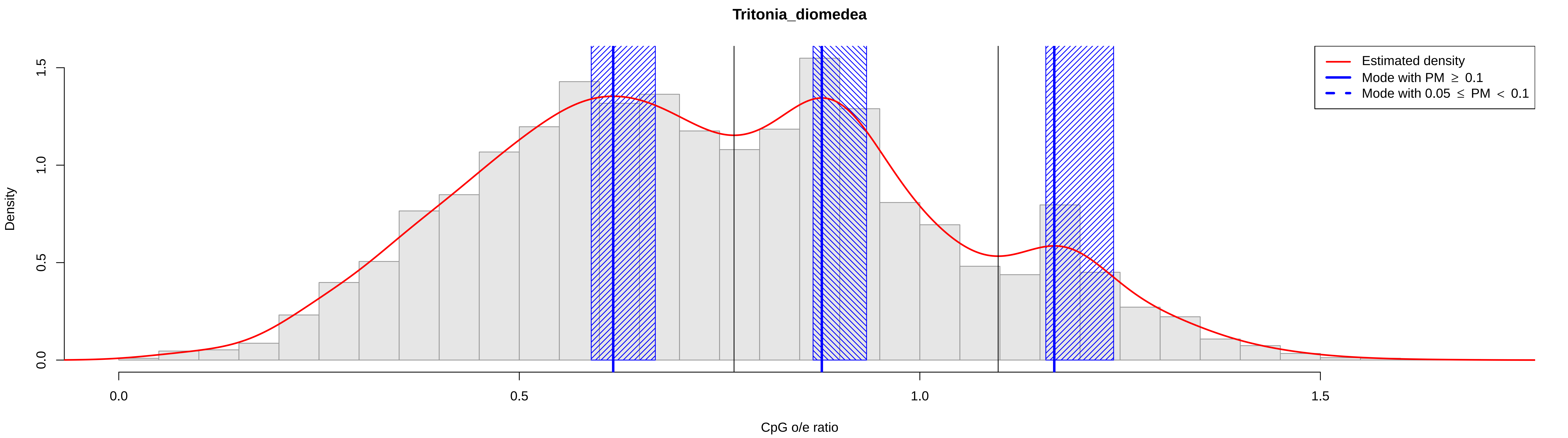
Tritonia_diomedea
Density
1.5
1.0
0.5
0.0
0.0
0.5
1.0
1.5
CpG o/e ratio
Estimated density
Mode with PM ≥ 0.1
Mode with 0.05 ≤ PM < 0.1

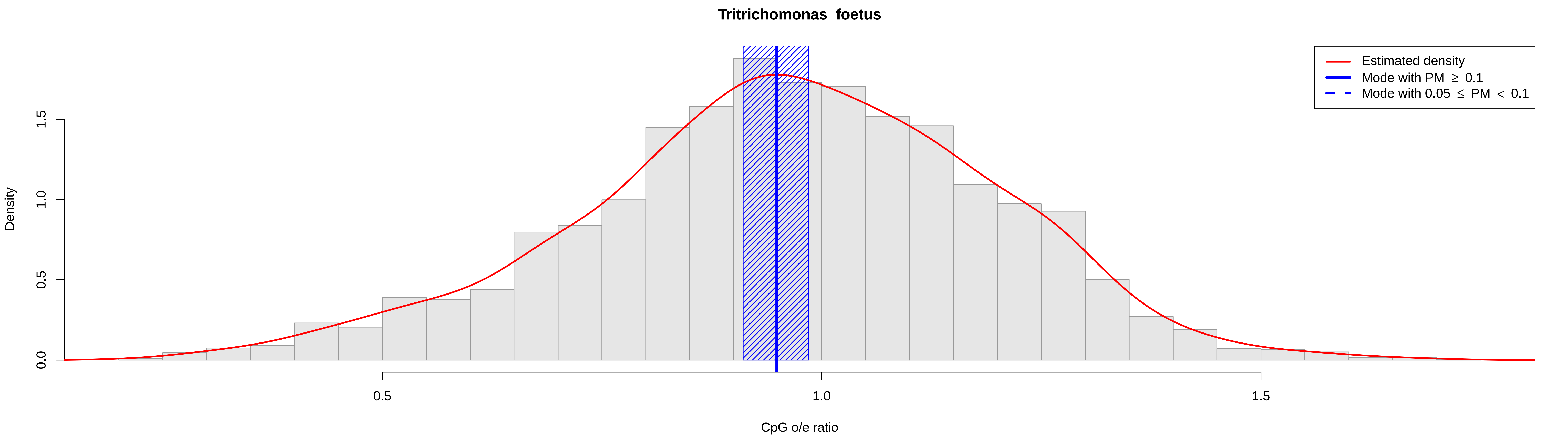

Tritrichomonas_foetus
Estimated density
Mode with PM ≥ 0.1
Mode with 0.05 ≤ PM < 0.1
Density
0.0
0.5
1.0
1.5
0.5
1.0
1.5
CpG o/e ratio

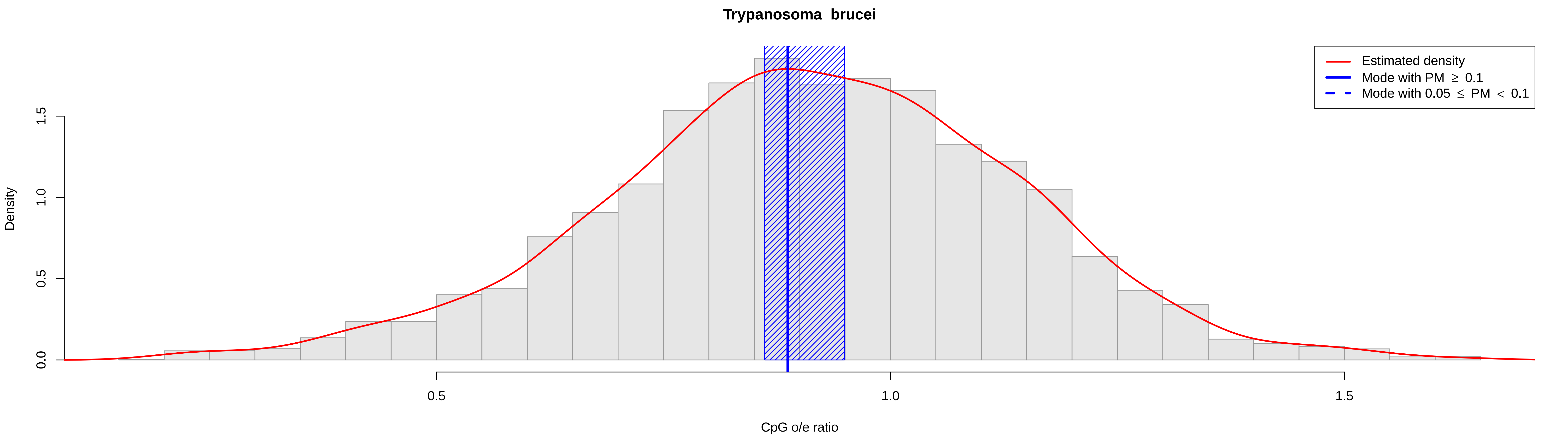
Trypanosoma_brucei
Estimated density
Mode with PM ≥ 0.1
Mode with 0.05 ≤ PM < 0.1
Density
0.0
0.5
1.0
1.5
0.5
1.0
1.5
CpG o/e ratio

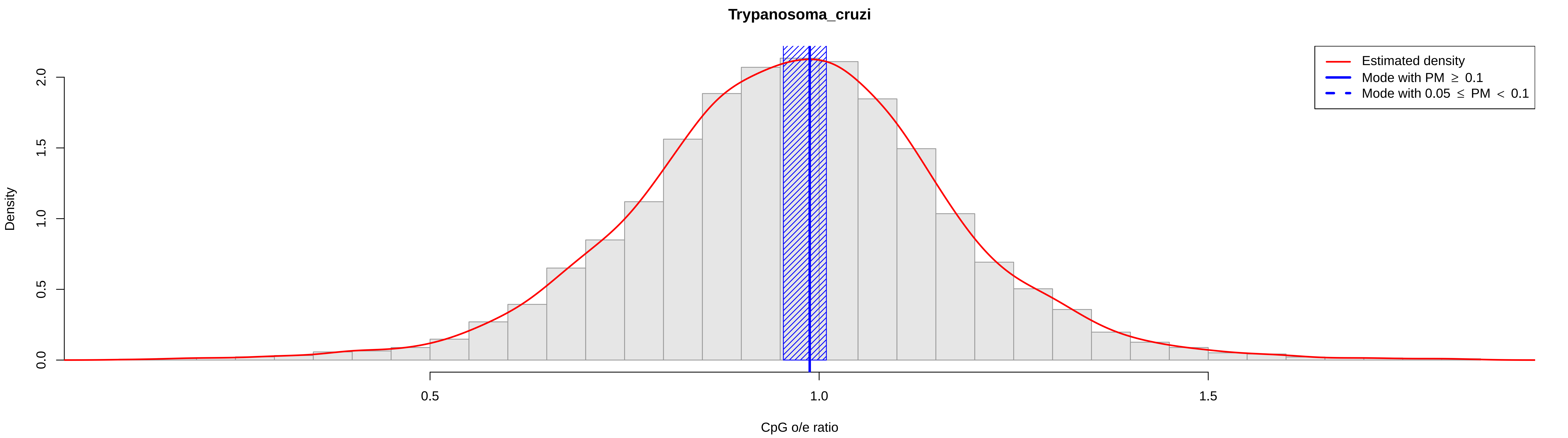
Trypanosoma_cruzi
Estimated density
Mode with PM ≥ 0.1
Mode with 0.05 ≤ PM < 0.1
Density
0.0
0.5
1.0
1.5
2.0
0.5
1.0
1.5
CpG o/e ratio

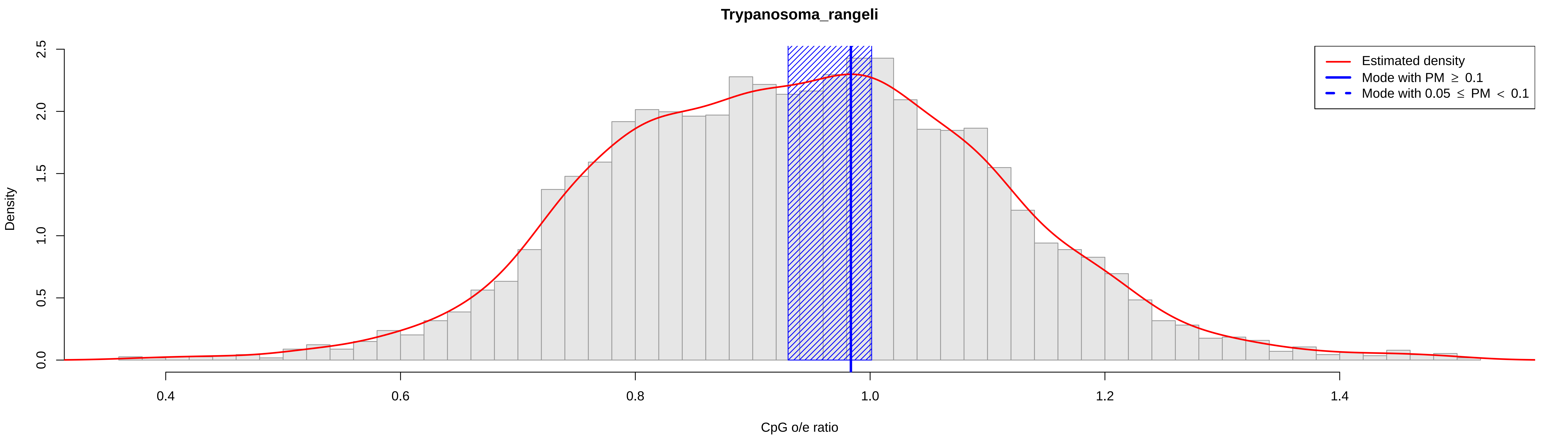
Trypanosoma_rangeli
Density
0.0
0.5
1.0
1.5
2.0
2.5
0.4
0.6
0.8
1.0
1.2
1.4
CpG o/e ratio
Estimated density
Mode with PM ≥ 0.1
Mode with 0.05 ≤ PM < 0.1

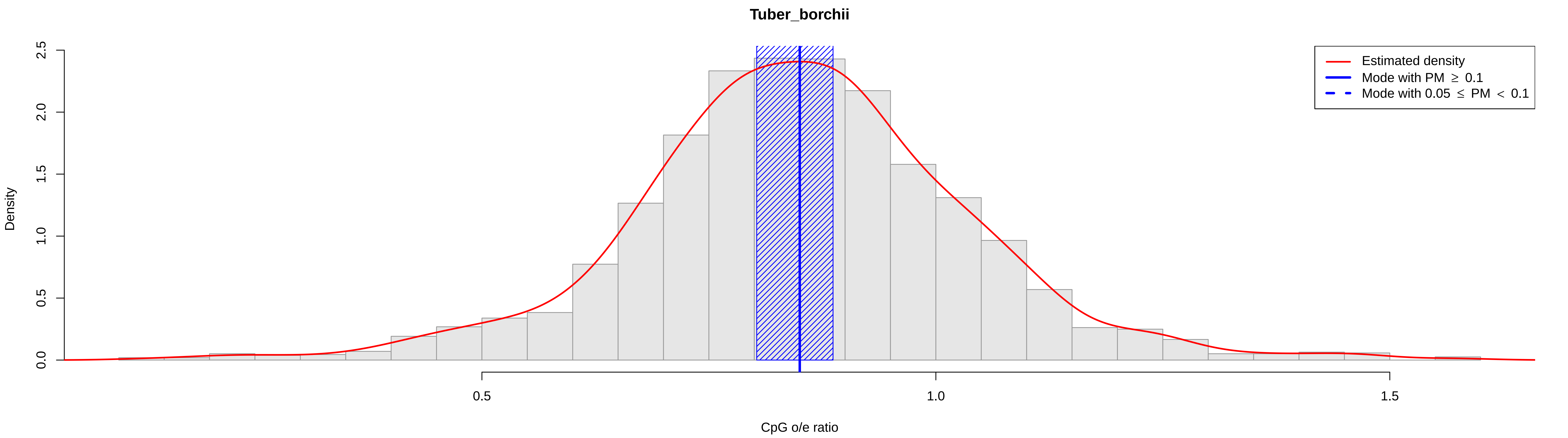

Tuber_borchii
Estimated density
Mode with PM ≥ 0.1
Mode with 0.05 ≤ PM < 0.1
Density
0.0
0.5
1.0
1.5
2.0
2.5
0.5
1.0
1.5
CpG o/e ratio

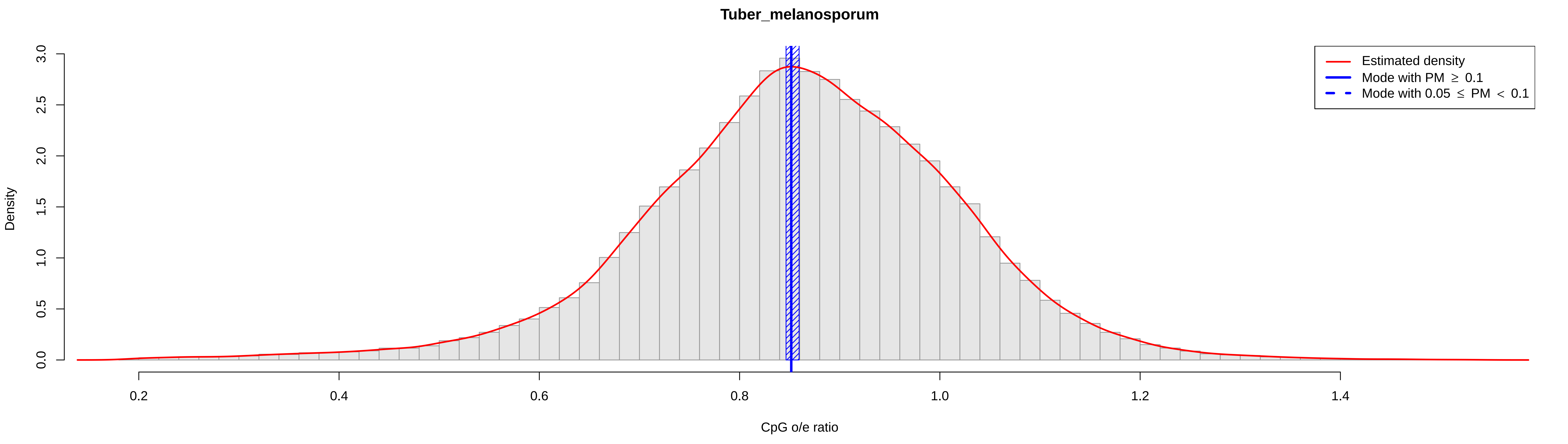
Tuber_melanosporum
Estimated density
Mode with PM ≥ 0.1
Mode with 0.05 ≤ PM < 0.1
Density
0.0
0.5
1.0
1.5
2.0
2.5
3.0
0.2
0.4
0.6
0.8
1.0
1.2
1.4
CpG o/e ratio

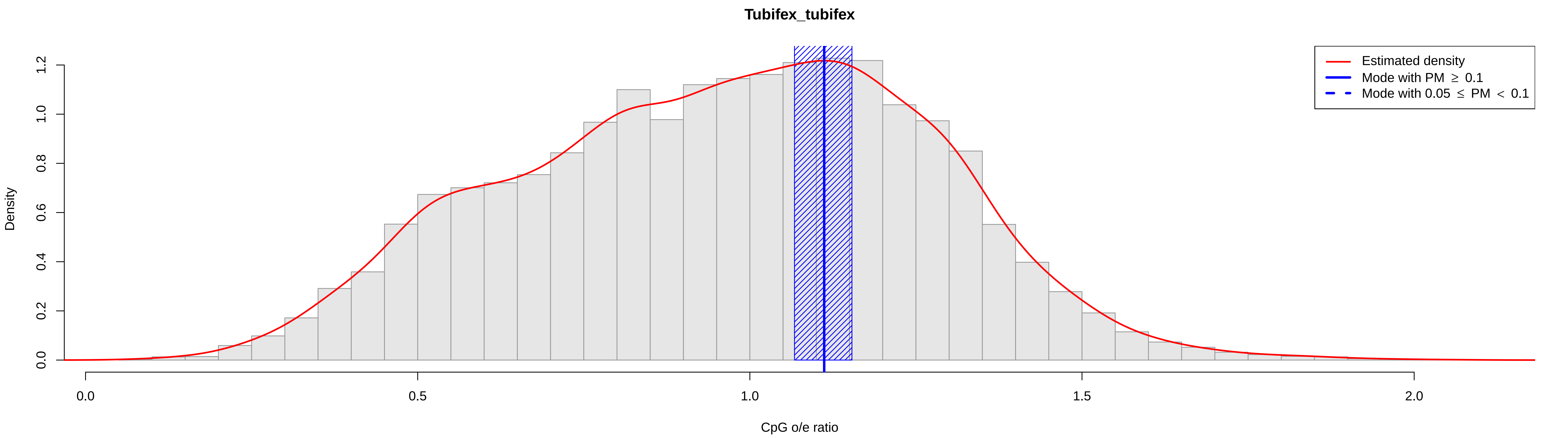
Tubifex_tubifex
Estimated density
Mode with PM ≥ 0.1
Mode with 0.05 ≤ PM < 0.1
Density
0.0
0.2
0.4
0.6
0.8
1.0
1.2
0.0
0.5
1.0
1.5
2.0
CpG o/e ratio

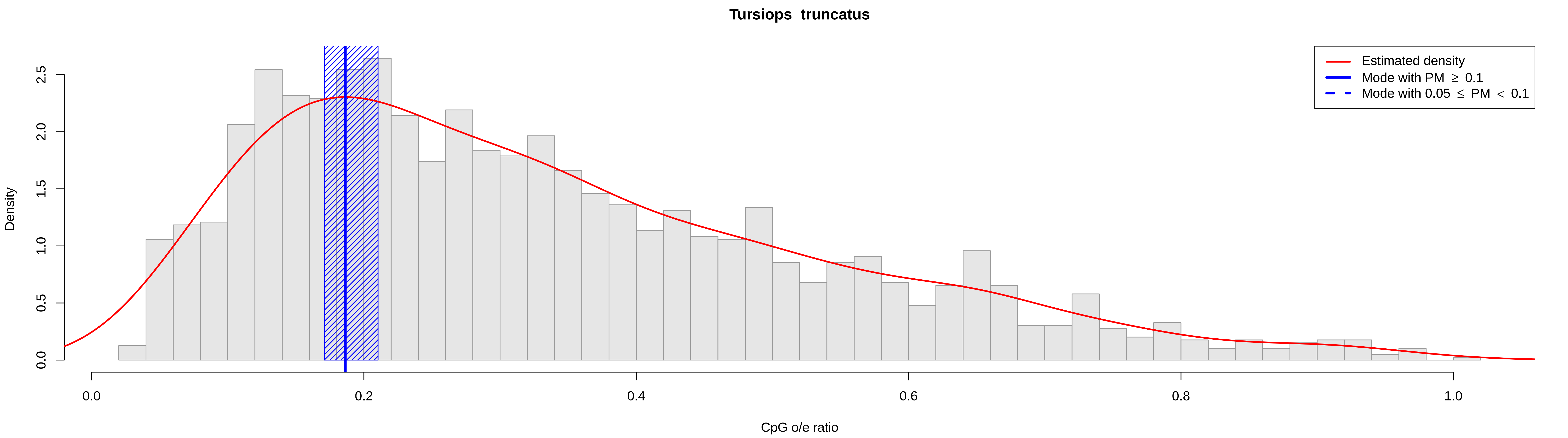

Tursiops_truncatus
Estimated density
Mode with PM ≥ 0.1
Mode with 0.05 ≤ PM < 0.1
Density
0.0
0.5
1.0
1.5
2.0
2.5
0.0
0.2
0.4
0.6
0.8
1.0
CpG o/e ratio

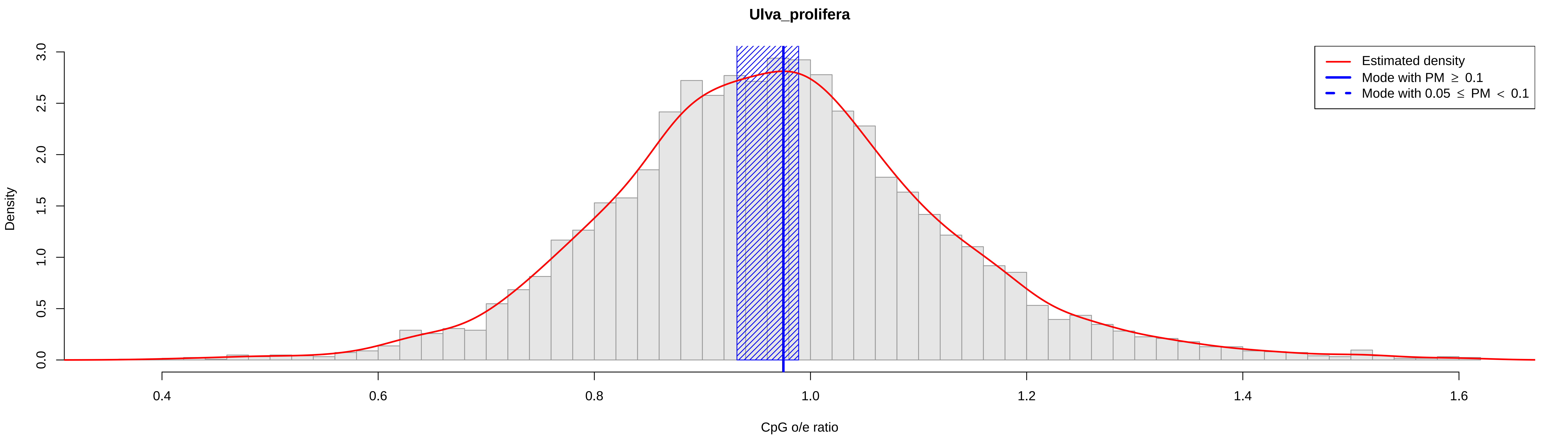

Ulva_prolifera
Estimated density
Mode with PM ≥ 0.1
Mode with 0.05 ≤ PM < 0.1
Density
0.0
0.5
1.0
1.5
2.0
2.5
3.0
0.4
0.6
0.8
1.0
1.2
1.4
1.6
CpG o/e ratio

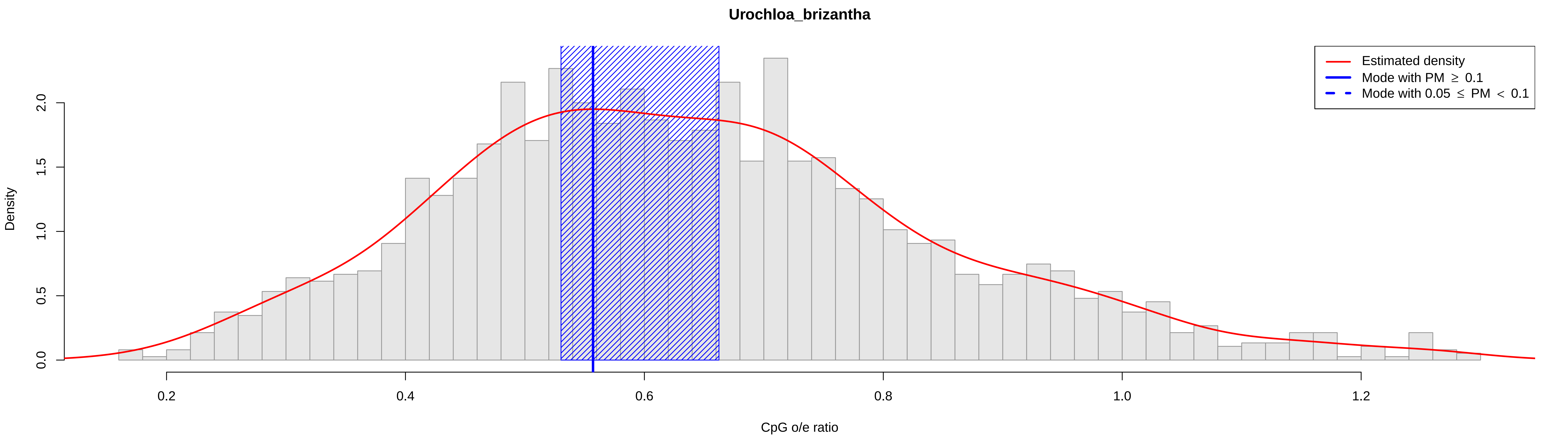

Urochloa_brizantha
Estimated density
Mode with PM ≥ 0.1
Mode with 0.05 ≤ PM < 0.1
Density
0.0
0.5
1.0
1.5
2.0
0.2
0.4
0.6
0.8
1.0
1.2
CpG o/e ratio

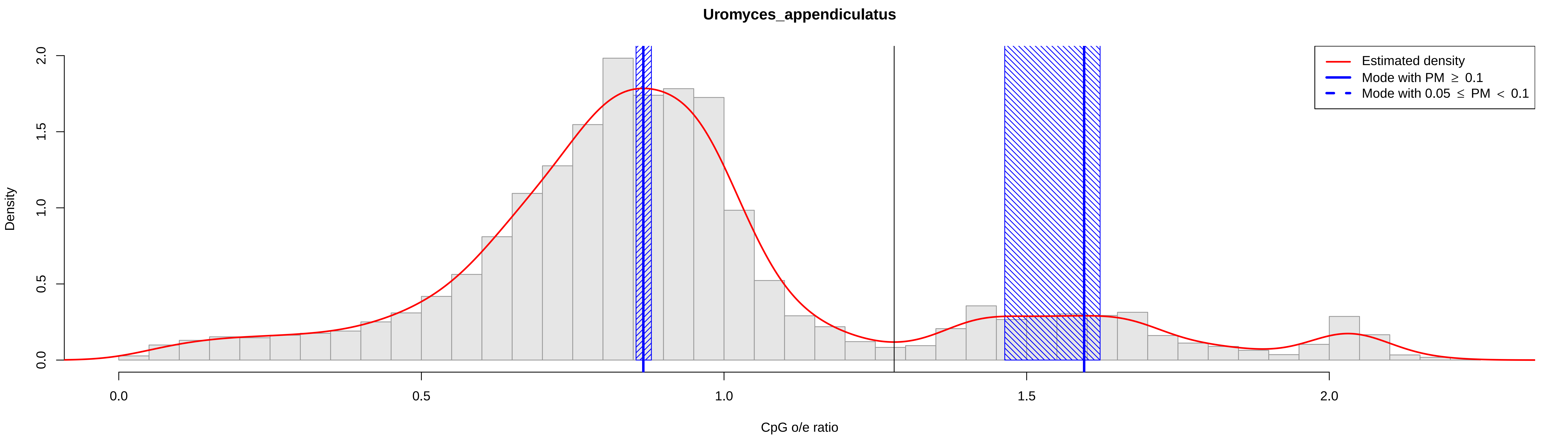

Uromyces_appendiculatus
Estimated density
Mode with PM ≥ 0.1
Mode with 0.05 ≤ PM < 0.1
Density
0.0
0.5
1.0
1.5
2.0
0.0
0.5
1.0
1.5
2.0
CpG o/e ratio

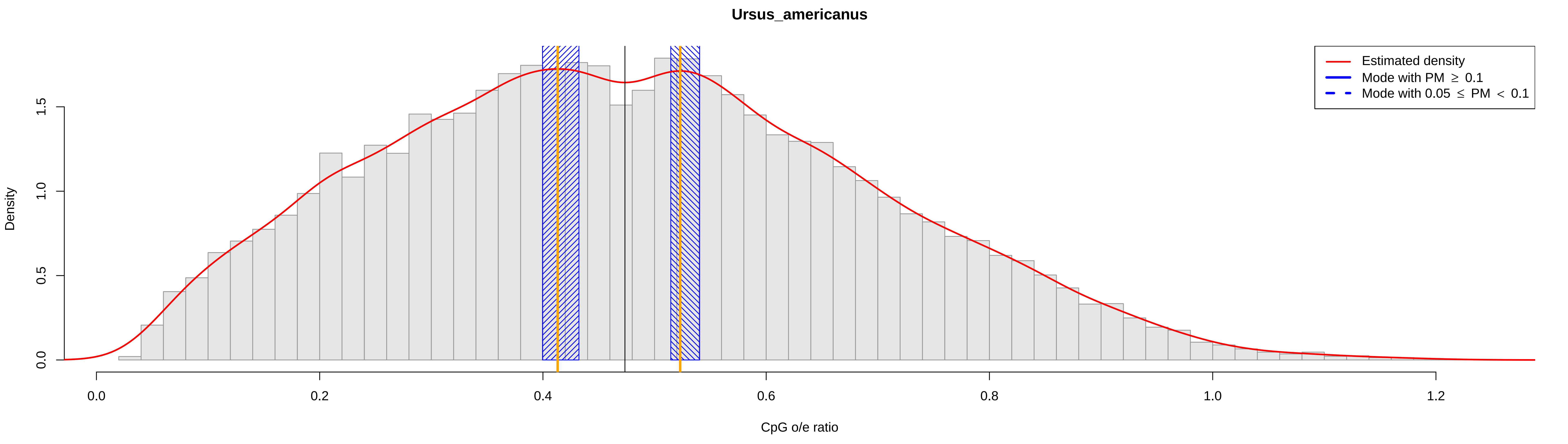
Ursus_americanus
Estimated density
Mode with PM ≥ 0.1
Mode with 0.05 ≤ PM < 0.1
Density
0.0
0.5
1.0
1.5
0.0
0.2
0.4
0.6
0.8
1.0
1.2
CpG o/e ratio

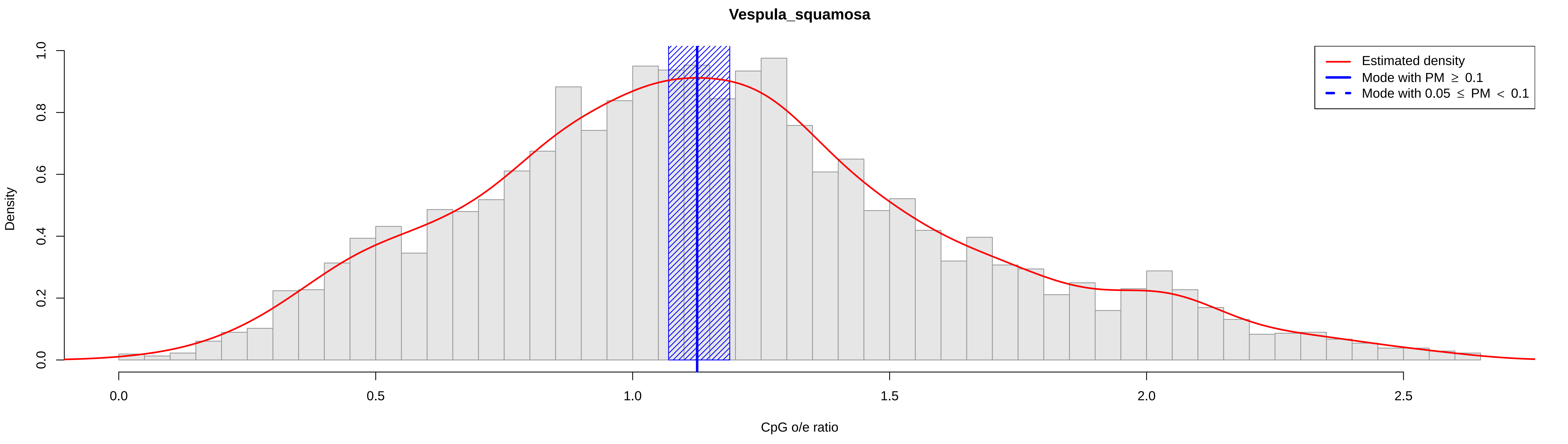

Vespula_squamosa
Estimated density
Mode with PM ≥ 0.1
Mode with 0.05 ≤ PM < 0.1
Density
0.0
0.2
0.4
0.6
0.8
1.0
0.0
0.5
1.0
1.5
2.0
2.5
CpG o/e ratio

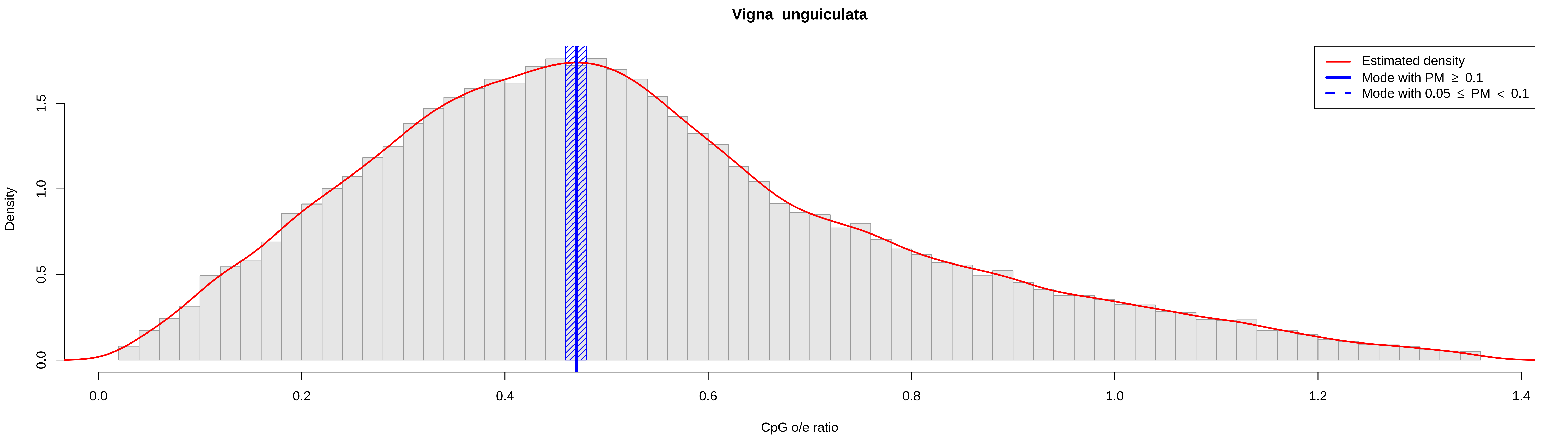
Vigna_unguiculata
Estimated density
Mode with PM ≥ 0.1
Mode with 0.05 ≤ PM < 0.1
Density
CpG o/e ratio
0.0
0.5
1.0
1.5
0.0
0.2
0.4
0.6
0.8
1.0
1.2
1.4

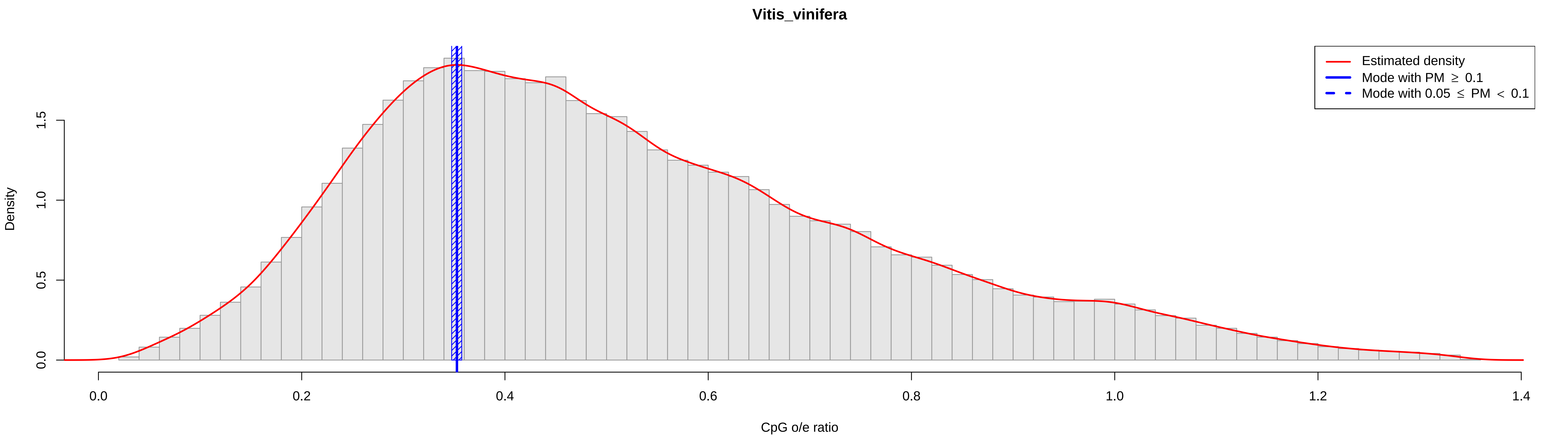

Vitis_vinifera
Estimated density
Mode with PM ≥ 0.1
Mode with 0.05 ≤ PM < 0.1
Density
0.0
0.5
1.0
1.5
0.0
0.2
0.4
0.6
0.8
1.0
1.2
1.4
CpG o/e ratio

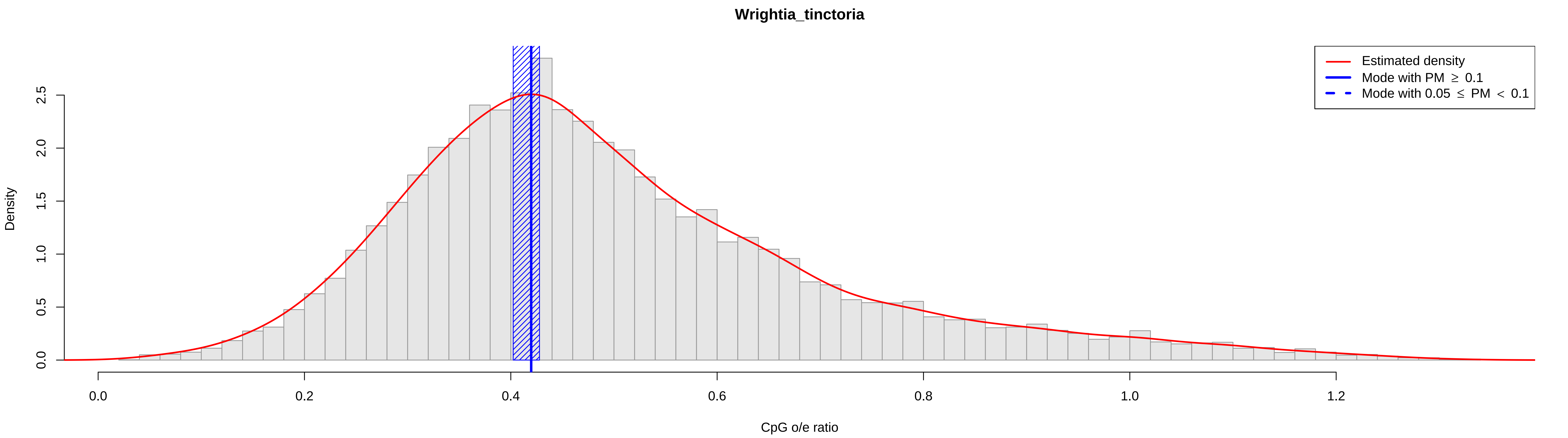

Wrightia_tinctoria
Estimated density
Mode with PM ≥ 0.1
Mode with 0.05 ≤ PM < 0.1
Density
0.0
0.5
1.0
1.5
2.0
2.5
0.0
0.2
0.4
0.6
0.8
1.0
1.2
CpG o/e ratio

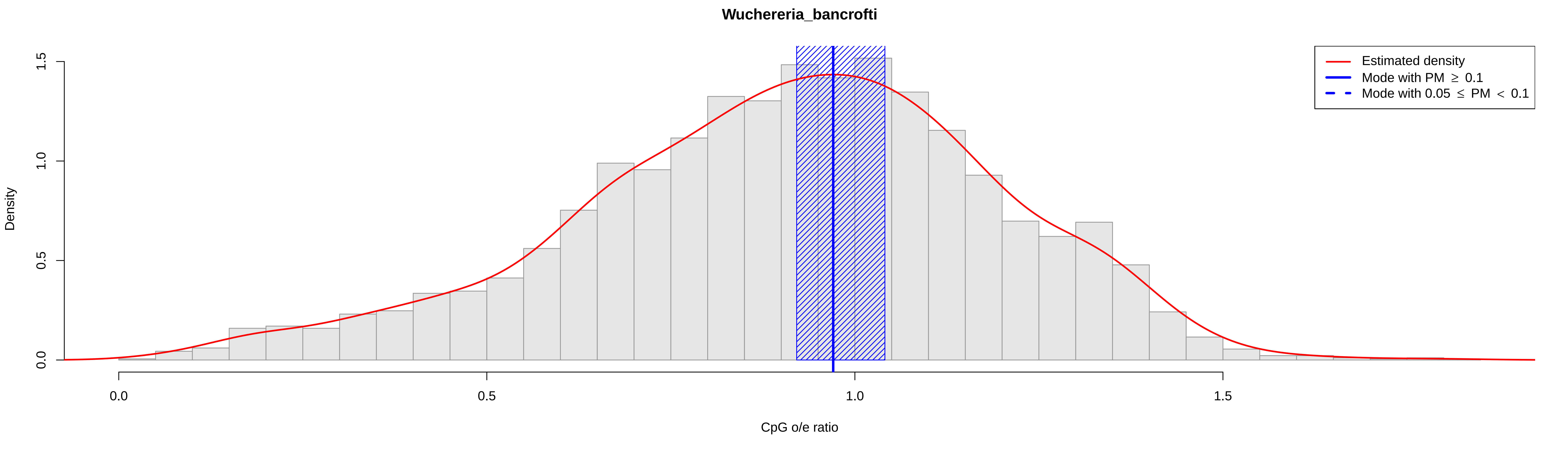

Wuchereria_bancrofti
Estimated density
Mode with PM ≥ 0.1
Mode with 0.05 ≤ PM < 0.1
Density
0.0
0.5
1.0
1.5
0.0
0.5
1.0
1.5
CpG o/e ratio

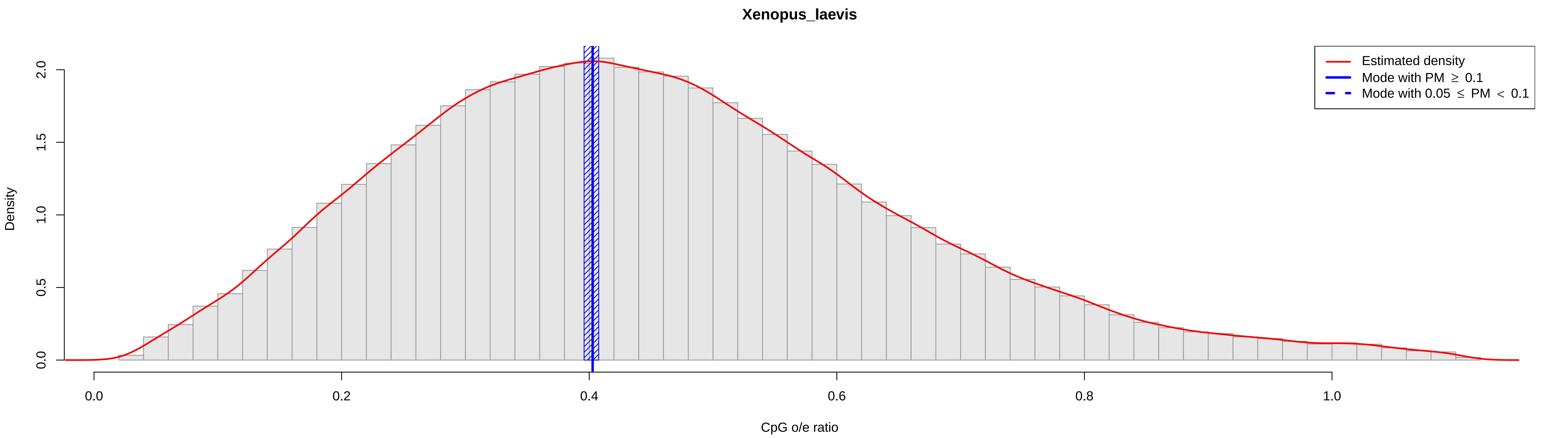
Xenopus_laevis
Estimated density
Mode with PM ≥ 0.1
Mode with 0.05 ≤ PM < 0.1
Density
0.0
0.5
1.0
1.5
2.0
0.0
0.2
0.4
0.6
0.8
1.0
CpG o/e ratio

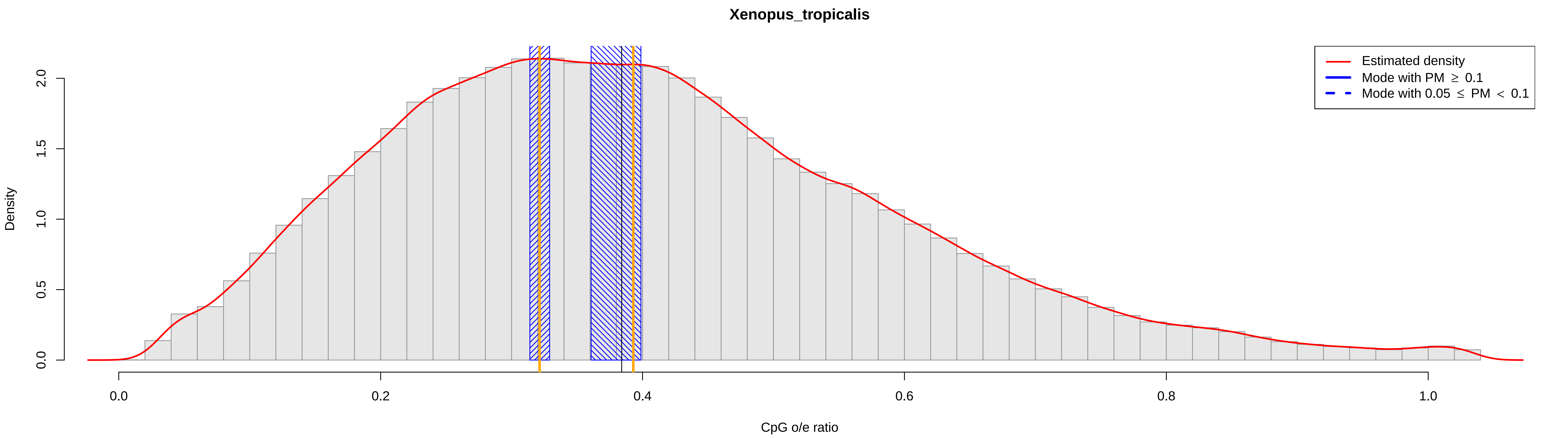

Xenopus_tropicalis
Estimated density
Mode with PM ≥ 0.1
Mode with 0.05 ≤ PM < 0.1
Density
0.0
0.5
1.0
1.5
2.0
0.0
0.2
0.4
0.6
0.8
1.0
CpG o/e ratio

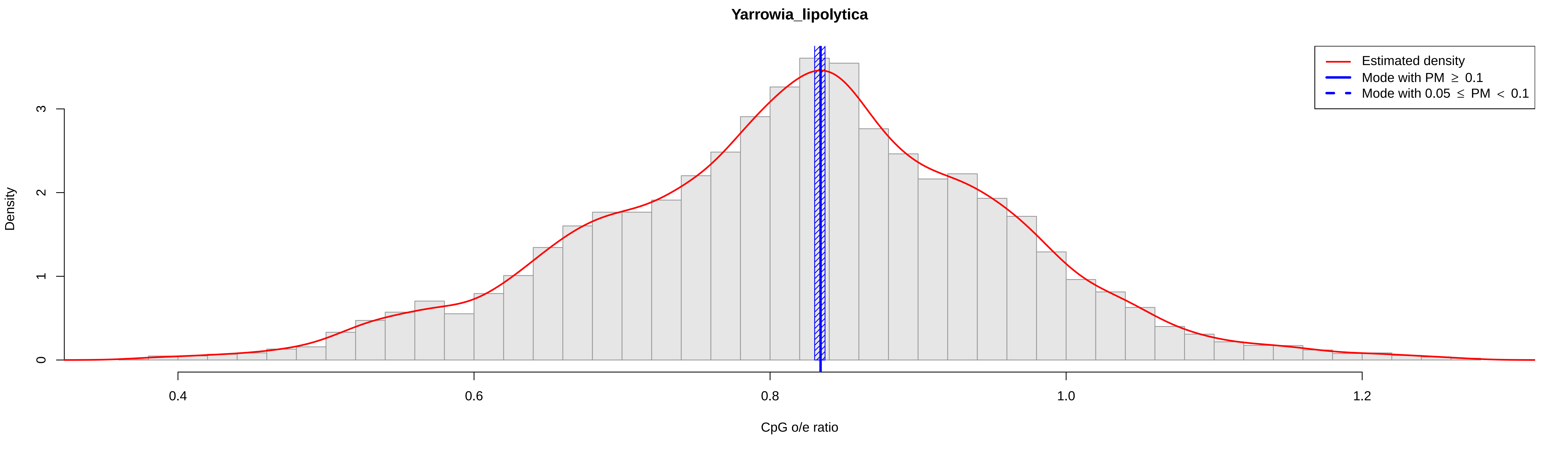

Yarrowia_lipolytica
Estimated density
Mode with PM ≥ 0.1
Mode with 0.05 ≤ PM < 0.1
Density
0
1
2
3
0.4
0.6
0.8
1.0
1.2
CpG o/e ratio

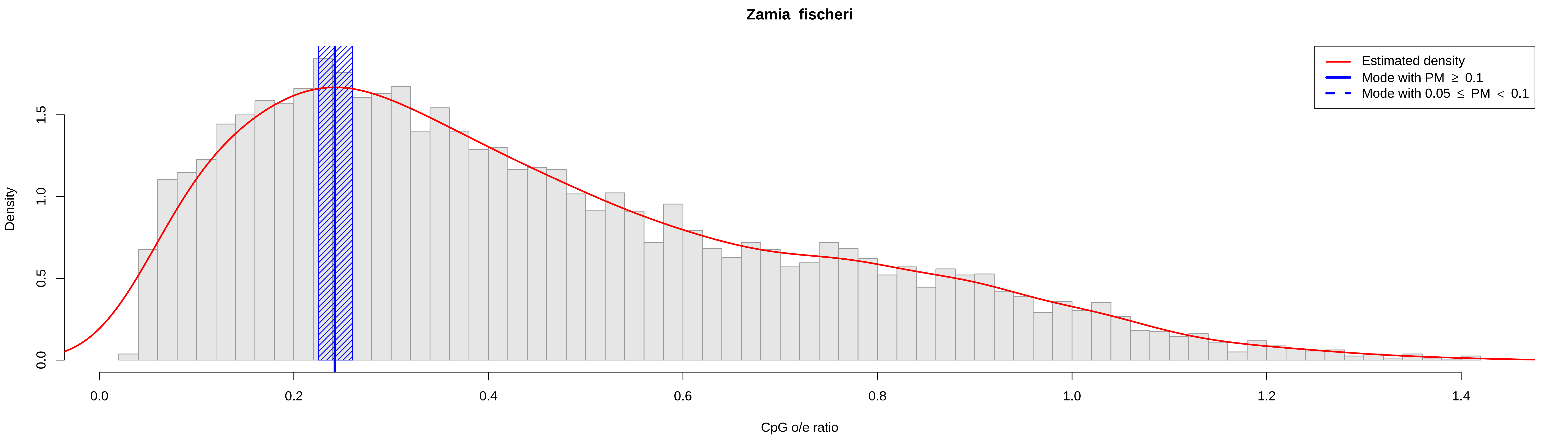

Zamia_fischeri
Estimated density
Mode with PM ≥ 0.1
Mode with 0.05 ≤ PM < 0.1
Density
0.0
0.5
1.0
1.5
0.0
0.2
0.4
0.6
0.8
1.0
1.2
1.4
CpG o/e ratio

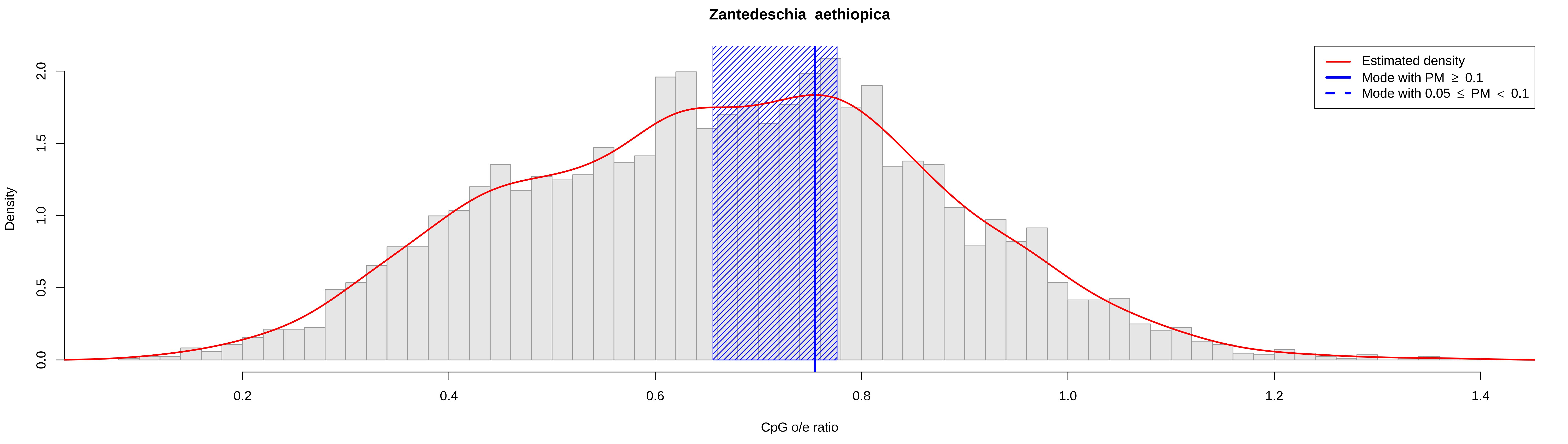
Zantedeschia_aethiopica
Estimated density
Mode with PM ≥ 0.1
Mode with 0.05 ≤ PM < 0.1
Density
0.0
0.5
1.0
1.5
2.0
0.2
0.4
0.6
0.8
1.0
1.2
1.4
CpG o/e ratio

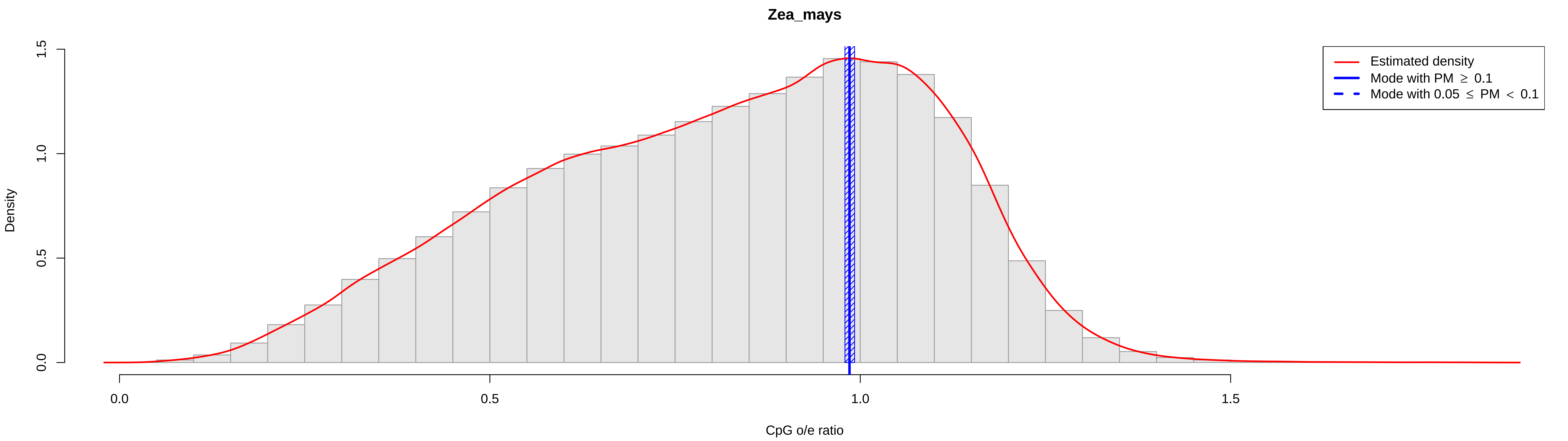

Zea_mays
Density
0.0
0.5
1.0
1.5
CpG o/e ratio
Estimated density
Mode with PM ≥ 0.1
Mode with 0.05 ≤ PM < 0.1

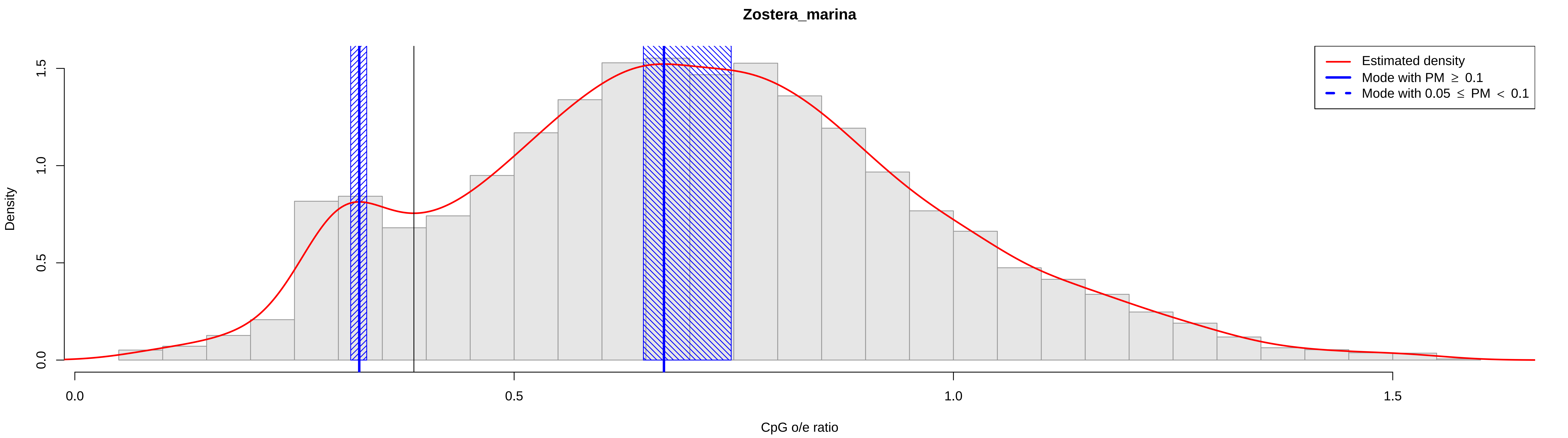

Zostera_marina
Estimated density
Mode with PM ≥ 0.1
Mode with 0.05 ≤ PM < 0.1
Density
0.0
0.5
1.0
1.5
0.0
0.5
1.0
1.5
CpG o/e ratio

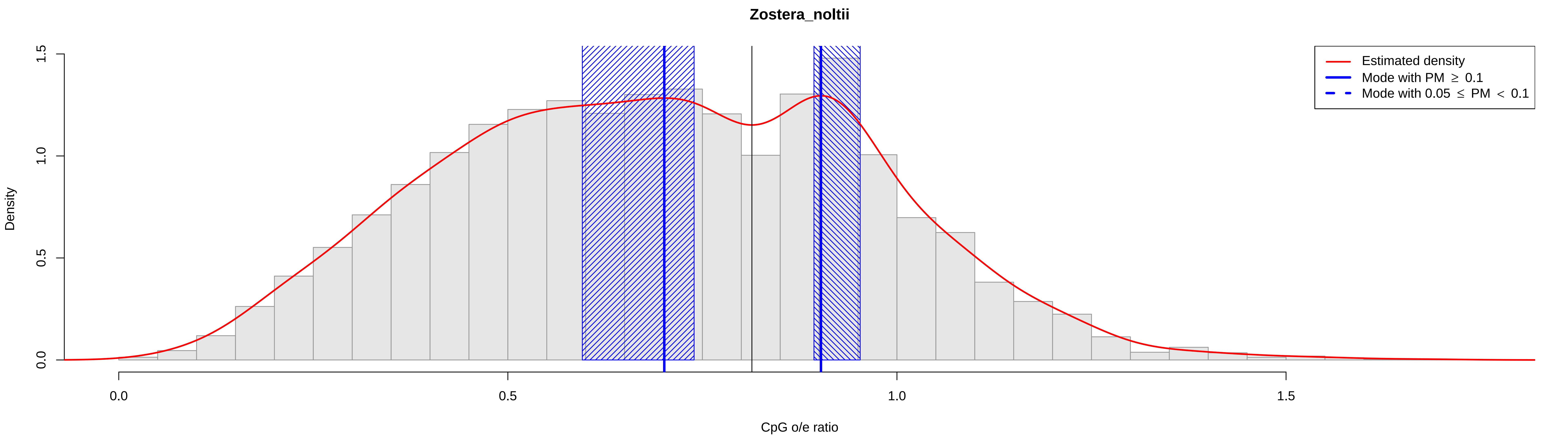

Zostera_noltii
Estimated density
Mode with PM ≥ 0.1
Mode with 0.05 ≤ PM < 0.1
Density
0.0
0.5
1.0
1.5
0.0
0.5
1.0
1.5
CpG o/e ratio
