# Supplementary material for: Notos - a galaxy tool to analyze CpN observed expected ratios for inferring DNA methylation types
Source: BMC Bioinformatics. 2018 Mar 27;19:105. doi: 10.1186/s12859-018-2115-4 (PMC5870242; doi:10.1186/s12859-018-2115-4)
Supplement: Supplementary file 3 — Details on kernel density estimation. This file contains additional details on the underlying theory of kernel density estimation. (PDF 273 kb) [file 12859_2018_2115_MOESM3_ESM.pdf]

Kernel density estimation

Kernel density estimation is a non-parametric method to estimate an unknown one-dimensional distribution f based on a given sample from this distribution. Kernel density estimators are closely related to histograms, but usually they are smoother. Denoting the sample by $x_1, \ldots, x_n$, the kernel density estimator of f is then defined as

$$\hat{f}_h(x) = \frac{1}{n} \sum_{i=1}^{n} K_h(x - x_i) = \frac{1}{nh} \sum_{i=1}^{n} K\left(\frac{x - x_i}{h}\right).$$

Here K is the so-called kernel, i.e., a non-negative function that integrates to one and h is the so-called bandwidth of the kernel. Furthermore, the so-called scaled kernel K_h is defined as $K_h(x) = \frac{1}{h} K(\frac{x}{h})$. That is, K_h is obtained by stretching or shrinking, respectively, K regarding its width by the factor h, rescaling its height so that K again integrates up to one. A frequent choice for the kernel is the Gaussian kernel, i.e., $K(y) = \phi(y)$, where ϕ is the standard normal density function, i.e.,

$$\phi(y) = \frac{1}{\sqrt{2\pi}} e^{-\frac{1}{2}y^2}$$

Alternative kernel definitions are, e.g., uniform, triangular, and Epanechnikov, to name only a few. For the remainder of this document, we will consider the Gaussian kernel case.

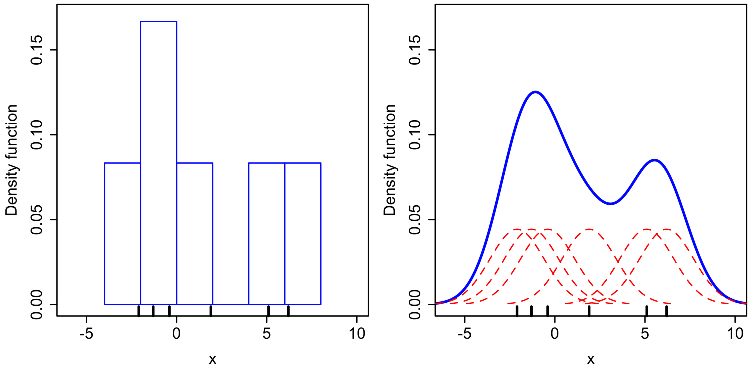

Figure 1: A histogram (left) and a kernel density estimator (right) for 6 data points $x_1 = -2.1$, $x_2 = -1.3$, $x_3 = -0.4$, $x_4 = 1.9$, $x_5 = 5.1$, $x_6 = 6.2$, using Gaussian kernels with a variance of 2.25, i.e., $h = 1.5$. The 6 individual kernels are colored in red, the resulting kernel density estimator in blue. The figure has been taken from Wikipedia (https://en.wikipedia.org/wiki/Kernel_density_estimation).

Figure 1 illustrates the principle of kernel density estimation by means of a small sample with six observations.

The bandwidth h is an important parameter for kernel density estimation. It allows to control the smoothness of the kernel density estimator. More precisely, the higher h is chosen, the smoother the resulting kernel density estimator becomes. This effect is illustrated in Figure 2. One the one hand, one can observe the kernel density estimator becomes over-smoothed for high values of h, which results in much of the underlying structure to be obscured. On the other hand, the kernel density estimator becomes under-smoothed for low values of h, resulting in too many spurious data artifacts.

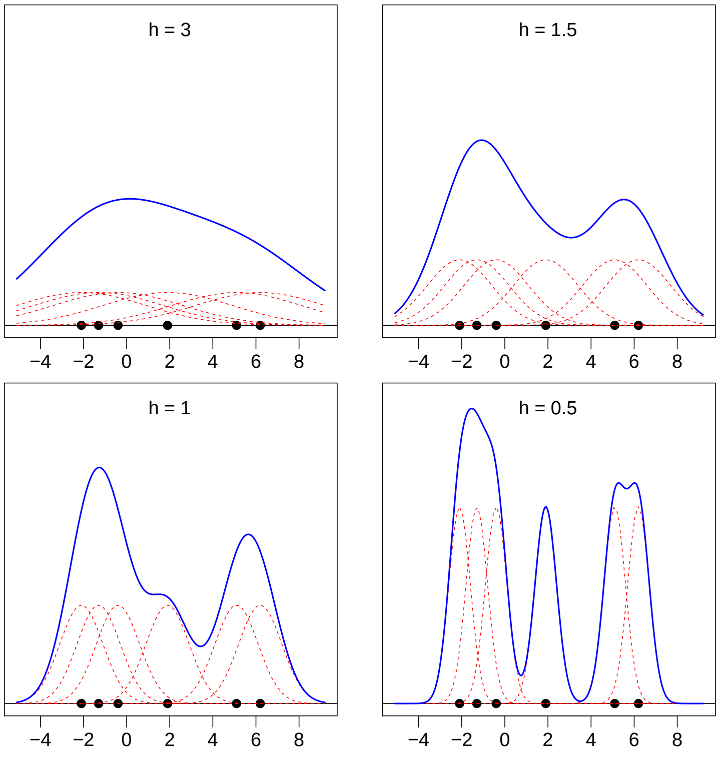

Figure 2: Kernel density estimators for $h = 3, 1.5, 1, 0.5$ using the same data as above.

Various approaches exist to determine appropriate values for h, such as such as cross-validation, Silverman's rule (Silverman, 1986), and Scott's variation of Sil-

verman's rule (Scott, 1992). Silverman's rule sets

$$h = 0.9 \min\left(\hat{\sigma}_x, \frac{\mathrm{IQR}_x}{1.34}\right) n^{-1/5},$$

where n, $\hat{\sigma}_x$ and IQR_x correspond to the sample size, the standard deviation of the sample and the sample's interquartile range. For Scott's variation of the bandwidth, the value 0.9 is replaced by 1.06.

References

Scott, D. W. (1992). *Multivariate Density Estimation: Theory, Practice, and Visualization*, Volume 156.

Silverman, B. W. (1986). Density estimation for statistics and data analysis. *Monographs on Statistics and Applied Probability 37*(1), 1–22.
